# Generation of a high resolution map of sRNAs from *Fusarium graminearum* and analysis of responses to viral infection

**DOI:** 10.1038/srep26151

**Published:** 2016-05-18

**Authors:** Shuangchao Wang, Pengfei Li, Jingze Zhang, Dewen Qiu, Lihua Guo

**Affiliations:** 1 State Key Laboratory for Biology of Plant Disease and Insect Pests, Institute of Plant Protection, Chinese Academy of Agricultural Sciences, Beijing, 100193, China

## Abstract

Previously, we characterized F. graminearum hypovirus 1 (FgHV1) and F. graminearum hypovirus 2 (FgHV2), which are the only two hypoviruses in *F. graminearum* that are closely related to Cryphonectria hypovirus 1 (CHV1) and Cryphonectria hypovirus 2 (CHV2) in the *Hypoviridae* family. In this study, we preliminarily elucidated the RNA silencing mechanism of the *F. graminearum*/hypovirus system from a small RNA (sRNA) perspective by using HiSeq deep sequencing. The length distributions of *F. graminearum* sRNA were altered by hypoviral infection. Potential microRNA-like (milRNA) candidates were differentially expressed between the hypovirus-free and hypovirus-infected library types. Extensive virus-derived small interfering RNAs (vsiRNAs) were also principally defined. The 1,831,081 and 3,254,758 total reads generated from the FgHV1 and FgHV2 genomes in *F. graminearum* yielded the first high-resolution sRNA maps of fungal viruses. In addition, extensive bioinformatics searches identified a large number of transcripts that are potentially targeted by vsiRNAs, several of which were effectively down-regulated. In particular, the RNA silencing-related genes FgDicer1 and FgRdRp5 were predicted targets of FgHV1- and FgHV2-derived siRNAs, possibly revealing a novel anti-RNA silencing strategy employed by mycoviruses.

RNA interference (RNAi) is a conserved eukaryotic gene regulatory mechanism that uses small noncoding RNAs to mediate transcriptional or post-transcriptional gene silencing^1^,^2^,^3^. Through a base-pairing mechanism, RNA silencing helps to regulate protein levels and to restrain the gene expression of parasitic and pathogenic invaders, such as viruses^4^. The RNAi pathway is thought to have evolved as a form of nucleic acid-based immunity to viruses in plants and insects^5^.

Our understanding of RNA silencing in fungi is poor relative to our understanding of RNA silencing in plants and animals. *Neurospora crassa* has been used as a model fungus to explore the mechanism of RNA silencing in fungi. Several genes and proteins in *N. crassa* (an RNA-dependent RNA polymerase called QDE1, an Argonaute protein called QDE2, Sad-1 and sms-2) are involved in post-transcriptional gene silencing^6^,^7^,^8^,^9^,^10^. However, the use of *N. crassa* as an experimental system for RNAi research has been limited by the lack of viral infection in this fungus. Insights into viral infections can help to illuminate the mechanism of RNA silencing during interactions between fungi and viruses. Evidence that RNA silencing functions as an antiviral defence mechanism in fungi has only been reported for *Cryphonectria parasitica* and *Aspergillus nidulans*. In *C. parasitica*, a single Dicer-like (DCL) gene, *dcl-2*, and a single Argonaute (AGO) gene, *agl-2*, play important roles in the host defence against CHV1 infection^11^,^12^. Furthermore, both *dcl-2* and *agl-2* are essential for mycoviral RNA recombination in *C. parasitica*^13^. Small RNAs, particularly microRNAs (miRNAs), small interfering RNAs (endo-siRNAs), and PIWI-interfering RNAs (piRNAs), play critical roles in the process of RNA silencing^14^. In *A. nidulans*, a virus 341-derived siRNA was detected at a high level in an Argonaute mutant, indicating that this virus is targeted by the RNAi machinery^15^. In CHV1-infected *C. parasitica*, 171 vsiRNAs were detected and sequenced^16^. Recently, Chen *et al.* characterized the functions of the core RNAi pathway-related genes in *F. graminearum* and demonstrated the critical roles of the Argonaute protein FgAgo1 and the dicer protein FgDicer2^17^.

*F. graminearum* (teleomorph *Gibberella zeae*) is the causal agent of *Fusarium* head blight (or scab) of wheat and barley, which can cause cereal mycotoxin contamination and severe yield loss. To date, we have identified two hypoviruses that infect *F. graminearum* strains in China. The first hypovirus, FgHV1, was closely related to CHV1 in the *Hypoviridae* family^18^. Another hypovirus, FgHV2, causes dramatic phenotypic changes, including reductions in the mycelial growth rate, in conidia production and in deoxynivalenol concentration^19^. Thus, the *F. graminearum*/hypovirus system can furnish a platform from which to investigate RNA silencing in *F. graminearum*.

The recent development of next-generation sequencing techniques has vastly facilitated the discovery of novel small regulatory RNAs. Herein, to characterize the properties of sRNAs and to gain insight into the RNA silencing process during the antiviral response in *F. graminearum*, four sRNA libraries that were prepared from virus-free wild type *F. graminearum* strains or from hypovirus-infected strains that harboured the hypoviruses FgHV1 or FgHV2 were deep sequenced to generate extensive data regarding their sRNAs. We analysed the total sRNA and miRNA profiles and the FgHV1- and FgHV2-derived siRNA profiles in an attempt to elucidate the relationship between sRNA production and the host RNA silencing system functions. To our knowledge, this report is the first to study the responses of *F. graminearum* to viral infection at the sRNA level. Our work will help to decipher the mechanisms of induction and suppression of host RNA silencing during the interaction of fungi and viruses.

## Results

### Small RNAs are differentially expressed in hypovirus-infected strains relative to isogenic virus-free strains

To confirm the respective presence and absence of FgHV1 and FgHV2 genomic RNA in the FgHV1-infected strain HN10, the FgHV1-free strain HN10-11F, the FgHV2-infected strain JS16 and the FgHV2-free strain JS16F, we extracted dsRNA and total RNA from these four strains and performed RT-PCR analysis on the total RNA. FgHV1 and FgHV2 dsRNA replication intermediates were detected in the virus-infected strains HN10 and JS16, respectively, while no dsRNA was detected in the virus-free strains HN10-11F and JS16F. PCR amplification of viral genome-specific sequences was observed in the virus-infected strains HN10 and JS16, while no bands were observed for the virus-free strains HN10-11F and JS16F (data not shown).

**Figure 1:**
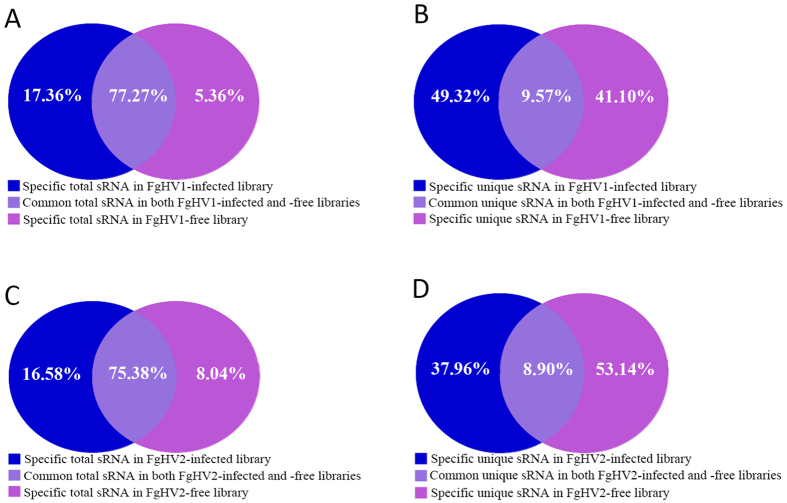
Summary of the common and specific total RNAs and the unique sRNAs among the virus-infected and virus-free libraries. Venn diagrams are shown for total sRNA comparisons between the FgHV1-free and FgHV1-infected libraries (**A**) and between the FgHV2-free and FgHV2-infected libraries (**C**). Venn diagrams are shown for unique sRNA comparisons between the FgHV1-free and FgHV1-infected libraries (**B**) and between the FgHV2-free and FgHV2-infected libraries (**D**). Blue, red and purple sections represent the percentages of the virus-infected library, the virus-free library and both libraries, respectively.

**Figure 2:**
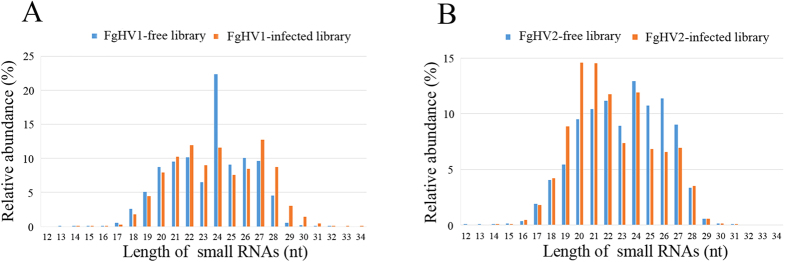
Size distribution of sRNA. The size distributions of the total sRNAs produced from the FgHV1-free and FgHV1-infected libraries are shown (**A**). The size distributions of sRNAs originating from the FgHV2-free and FgHV2-infected libraries are shown (**B**). The *x*-axis indicates the sRNA size (nt), and the *y*-axis shows the proportions of sRNAs of different sizes in the virus-free and virus-infected libraries.

Using the Illumina Solexa platform, the sRNAs produced in virus-free and virus-infected *F. graminearum* strains were identified by deep sequencing. Deep sequencing yielded 11,423,975 and 12,108,968 total reads for the FgHV1-infected and FgHV1-free libraries, respectively, and 13,586,176 and 13,392,162 reads for the FgHV2-infected and FgHV2-free libraries, respectively (Table 1). After removing the adaptors and filtering out low-quality tags and contaminants due to adaptor-adaptor ligation, the remaining clean reads were counted as sequence tags. Nearly equal numbers of perfectly matching clean reads were obtained from the FgHV1-infected and FgHV1-free libraries (11,090,406 and 11,693,092 reads, respectively). Similarly, for the FgHV2-infected and FgHV2-free libraries, 13,149,451 and 12,778,696 clean reads were obtained, respectively (Table 1). The common and unique total sRNAs in the FgHV1-infected and FgHV1-free group (group 1) and the FgHV2-infected and FgHV2-free group (group 2) were then analysed. As shown in Fig. 1, between the FgHV1-infected and FgHV1-free libraries, unique tags represented 9.57% while common tags represented 77.27%; the corresponding values for the FgHV2-infected and FgHV2-free libraries were 8.90% and 75.38%. Typically, a large difference in reads exists among different samples while the common reads are concentrated, which demonstrates good uniformity among different samples throughout the sequencing. In the hypovirus-free libraries from these two groups, the 24-nt class of sRNAs was the most dominant class, accounting for 22.40% and 12.91% of the total sRNAs of the FgHV1-free and FgHV2-free libraries, respectively (Fig. 2). This result is in accord with the rules of sRNA distribution derived from some plant species^20^. In the FgHV1-infected library, the 27-nt class of sRNAs was represented by the highest peaks, followed by the 22-nt class, while in the FgHV2-infected library, the 20-nt class of sRNAs was represented by the highest peaks, followed by the 21-nt class (Fig. 2). Notably, in the FgHV1-infected library, the sRNAs had peaks at approximately 22 and 27 nt, placing the sRNAs into a class similar to that of the PIWI-interacting RNAs (piRNAs), which are the longest of the sRNAs. The piRNAs binding to the PIWI clade of AGOs are involved in transposon regulation^21^. Notably, the percentage of 24-nt reads in the FgHV1-infected library was decreased by nearly one-half compared to the FgHV1-free library. However, the number of 24-nt sRNA reads in the FgHV2-infected library was not drastically affected relative to the FgHV2-free library (Fig. 2). In plants, repeat-associated siRNAs that are 24 nt in length are involved in the methylation and silencing of many transposons and repeats^22^. The overall size-distribution patterns of sRNAs between the hypovirus-infected and hypovirus-free libraries were quite different, suggesting that infection with the FgHV1 and FgHV2 hypoviruses can affect sRNA accumulation in their host, *F. graminearum*.

### MicroRNA-like RNAs are regulated in response to hypoviral infection in *F. graminearum*

MicroRNAs (miRNAs) are a class of endogenous short non-coding RNAs that post-transcriptionally and negatively regulate gene expression. It has been demonstrated that miRNAs are associated with cellular changes after viral infection. However, because there is little information on fungi in the miRBase, we attempted to identify milRNA homologues using the standard criteria for both plants and animals. Using the MIREAP program, 60 milRNAs were defined as milRNA candidates in FgHV1-free and FgHV1-infected *F. graminearum* (Group 1) (Table S1). Similarly, in the FgHV2-free and FgHV2-infected libraries (Group 2), 64 potential milRNA candidates were identified (Table S2). The sizes of the milRNA candidates in these two groups varied from 20 to 23 nt, with a peak at 22 nt. Moreover, the average folding free energies of the milRNA candidates in Groups 1 and 2 were −56.50 and −54.07 kcal mol^−1^, respectively, which are only slightly higher than those of the *Sclerotinia sclerotiorum* and *Arabidopsis* miRNA precursors. Among the milRNA candidates, four were found in both Group 1 and Group 2. The milRNA candidate Fg-milRNA-1, which was identified by Chen *et al.*^17^, was also identified in our work as part of Group 2.

**Figure 3:**
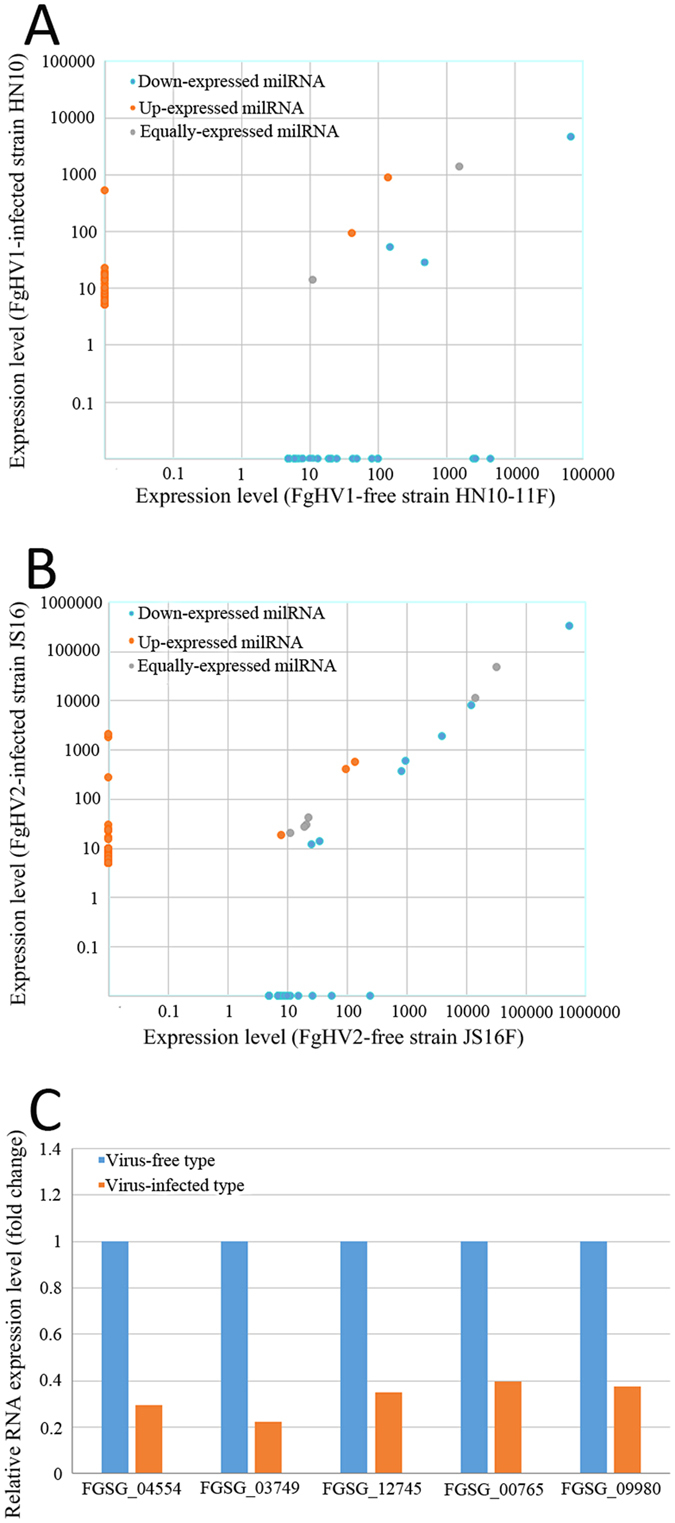
The expression patterns of milRNAs in hypovirus-free and hypovirus-infected *F. graminearum*. (**A**) Comparison of the milRNA expression patterns in FgHV1-free and FgHV1-infected *F. graminearum*. (**B**) The expression levels of 64 milRNA candidates in FgHV2-free and FgHV2-infected *F. graminearum*. (**C**) Analysis of the relative expression levels of miRNA target genes by high-throughput sequencing. FGSG_04554 and FGSG_03749 were targeted by FgHV1-derived Fg-HN-milR 36 and Fg-HN-milR 37, respectively. FGSG_12745, FGSG_00765 and FGSG_09980 were targeted by FgHV2-derived Fg-JS-milR 50, Fg-JS-milR 58 and Fg-JS-milR 62, respectively.

The expression of milRNAs in the different libraries was also compared. Twenty-four milRNA candidates were detected in FgHV1-infected libraries, indicating that these milRNA candidates might be FgHV1-independent (Fig. 3A). Thirty-four potential milRNA candidates were specifically expressed in FgHV2-infected *F. graminearum* compared to the FgHV2-free strain (Fig. 3B). There were also some milRNA candidates that were identified in the hypovirus-free libraries but not in the hypovirus-infected libraries. We employed psRobot and TargetFinder to predict the targets of *F. graminearum* milRNAs and obtained 236 and 293 putative target genes for the milRNA candidates in Group 1 and Group 2, respectively. The results showed that 18 genes were targeted by the milRNA candidates that were identified in both Group 1 and Group 2. The functions of these milRNA-targeted genes are related to the oxidative stress response, the tethering/docking stage of vacuole fusion, and RNA (cytosine-5) methyltransferases, among others. Our high-throughput mRNA sequencing results showed that the expression levels of five transcripts, which were targeted by the FgHV1-dependent Fg-HN-milR 36 and Fg-HN-milR 37 and by the FgHV2-dependent Fg-JS-milR 50, Fg-JS-milR 58, and Fg-JS-milR 62, were significantly down-regulated (Fig. 3C).

### A large number of hypovirus-derived siRNAs are detectable in hypovirus-infected *F. graminearum*

Previous reports indicated that a large number of vsiRNAs may correlate with the accumulation of viral RNA. To determine whether viral RNA triggered RNA silencing in the host, we searched for vsiRNAs in the total sRNA library. Similarly, the sRNAs in the virus-infected library were mapped to the FgHV1 and FgHV2 viral genomic and antigenomic RNA sequences. In total, there were 1,831,081 (88,911 unique) and 3,254,758 (113,257 unique) reads that perfectly aligned to the FgHV1 and FgHV2 genomes, respectively, accounting for approximately 16.51% and 24.75% of the total clean sRNA reads in the FgHV1-infected and FgHV2-infected libraries, respectively (Table 1). As expected, no FgHV1-derived sRNAs were detected in the FgHV1-free library as perfect matches. In contrast, there were few sRNA reads that matched the FgHV2 genome in the FgHV2-free library. The alignment results also showed that vsiRNAs were derived from both the positive and negative strands of the viral RNA and covered both coding and intergenic regions.

### Hypovirus-derived siRNAs are sized from 18 to 32 nt with a peak at 21 nt

**Figure 4:**
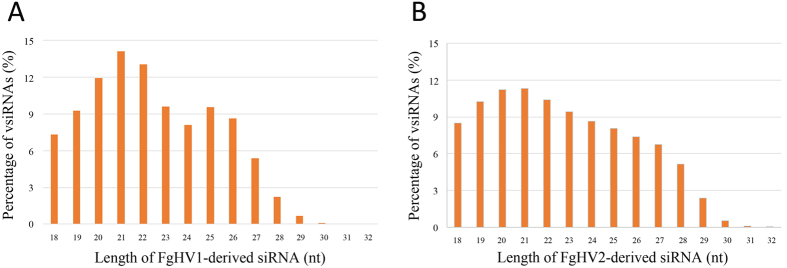
Size distribution and abundance of sRNAs matching the FgHV1 genome (A) and FgHV2 genome (B) in the virus-infected libraries. The *y*-axis indicates the proportions of vsiRNAs matching the FgHV1 and FgHV2 genomes, and the *x*-axis represents the length distribution.

In *Arabidopsis thaliana*, different Dicer proteins are responsible for the cleavage of sRNAs of different lengths. Among them, Dicer4 and Dicer2 are responsible for the cleavage of 21- and 22-nt sRNAs, respectively^23^. In *F. graminearum*, there are two Dicer homologs, which induced us to analyse the size distribution of vsiRNAs in *F. graminearum* to identify similarities and differences compared with current model systems^17^,^19^. In both the FgHV1-infected and FgHV2-infected libraries, the total vsiRNAs ranged from 18 to 32 nt, with a peak at 21 nt (Fig. 4). This result is consistent with previous reports that 21-nt vsiRNAs predominate in several virus-infected plants^20^,^24^. In contrast, 21- and 22-nt vsiRNAs did not predominate the total vsiRNAs, as they represented 27.22% and 21.73% of the total FgHV1- and FgHV2-derived siRNAs, respectively. This difference suggests that there is a cluster of DCLs that are involved in the RNA silencing system of *F. graminearum.*

### Hypovirus-derived siRNAs are distributed along the RNA genomes in a nonrandom pattern, with the majority derived from the positive strand

Alignment analysis revealed that a greater proportion of vsiRNAs from both FgHV1 and FgHV2 were derived from the sense strand than from the antisense strand of the viral genome. Specifically, the ratios of vsiRNAs originating from the positive strand to vsiRNAs originating from the negative strand were 1.38:1 and 1.96:1 for the FgHV1 and FgHV2 genomes, respectively. A more specific analysis indicated that many more of the FgHV1- and FgHV2-derived 21- to 24-nt siRNAs were derived from the positive strand than from the negative strand, as observed for the total FgHV1- and FgHV2-derived siRNAs and for CHV1-derived sRNAs.

**Figure 5:**
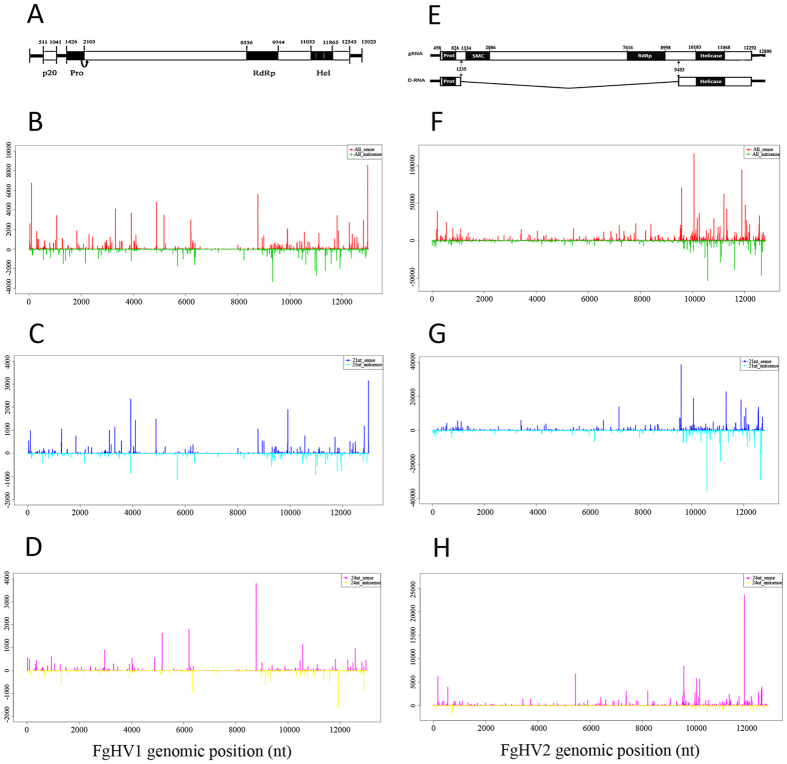
Origins and positions of vsiRNAs along the viral genome. The FgHV1 (**A**) and FgHV2 (**E**) genome organization is shown along with the defective RNA from FgHV2. The vsiRNA distribution profiles along the FgHV1 (**B**) and FgHV2 genomes (**F**) are shown. The vsiRNAs derived from the positive strand of the viral genome are indicated above the line representing the length of the viral genome, while the vsiRNAs derived from the negative strand are indicated below the line. The distributions of 21-nt vsiRNAs are shown along the FgHV1 (**C**) and FgHV2 (**G**) genomes. The 21-nt vsiRNAs that mapped to the genomic and antigenomic strands are shown in blue and green, respectively. The profiles of 24-nt vsiRNAs are shown along the FgHV1 (**D**) and FgHV2 (**H**) genomes. Red represents 24-nt vsiRNAs matching the genomic strand, while yellow indicates 24-nt vsiRNAs matching the antigenomic strand. The *x*-axis shows schematic representations of FgVH1 and FgHV2 genomic organization. The *y*-axis depicts the numbers of vsiRNAs matching the genomic and antigenomic sequences.

To determine the distribution of the vsiRNAs across the FgHV1 and FgHV2 genomes, we used Perl scripts to localize the vsiRNAs to the genomes based on their 5′-terminal nucleotide genome locations and polarities. As shown in Fig. 5, the vsiRNAs were distributed along both the positive and negative strands of both the FgHV1 and FgHV2 genomes in a non-random pattern. The FgHV1-derived siRNAs nearly saturated the coding and intergenic regions, which contain two open reading frames encoding a 20-kDa protease and multiple functional proteins totalling 421 kDa (Fig. 5B). In contrast, the FgHV2-derived siRNAs were mainly distributed along the 5′- and 3′-terminal regions of the sense and antisense strands of the FgHV2 genome (Fig. 5F). These results indicate that *F. graminearum*-encoded DCLs do not show strong strand preference but do have preferential targets across the FgHV1 and FgHV2 genomes. Further analysis revealed that vsiRNA-generating hotspots were present on both the positive and negative strands of both the FgHV1 and FgHV2 genomes. FgHV1 genome regions corresponding to the extreme 5′-end (approximately at nucleotide position 102) and to the 3′-terminal region (at an approximate nucleotide position of 12,984) tended to produce higher levels of vsiRNAs. In contrast, relatively lower levels of vsiRNAs were detected between map positions 6,394 and 8,759. Notably, when we mapped the FgHV2-derived siRNAs along the D-RNA segment of the FgHV2 genome, the regions corresponding to the D-RNA segment contained most of the hotspots (Fig. 5E,F). Specific analyses indicated that the distribution peaks of FgHV1- and FgHV2-derived 21- and 24-nt siRNAs overlapped with the peaks of total vsiRNAs and displayed similar distribution patterns to the total vsiRNAs (Fig. 5C,D,G,H). At many of the hotspots, various vsiRNA classes redundantly accumulated, suggesting that different *F. graminearum*-encoding DCL enzymes might recognize similar RNA sequences or structures and competitively cleave the region.

In addition to the dsRNA replication intermediates, highly structured positive single-strand genomic RNA can also serve as a substrate for Dicer enzymes. To determine whether the secondary structures of regions along the viral RNA contribute to vsiRNA production, we evaluated the secondary structures of the regions corresponding to the high vsiRNA abundance at the FgHV1 and FgHV2 5′- and 3′-terminal noncoding regions (for which a much higher abundance of vsiRNAs originated from the positive strand than from the negative strand) by utilising RNAfold software, as previously described^25^. As shown in Fig. S1, RNA secondary structures were predicted in the 5′-and 3′-terminal regions. Although many hotspots were located in positions that form stem-loop structures, close correlations between hotspots and predicted secondary structure could not be identified. Other factors may contribute to the higher abundance of vsiRNAs generated from the positive genomic strand than from the negative genomic strand.

### The most abundant 5′-terminal nucleotides of the FgHV1- and FgHV2-derived siRNAs are guanine (G) and uridine (U), respectively

**Figure 6:**
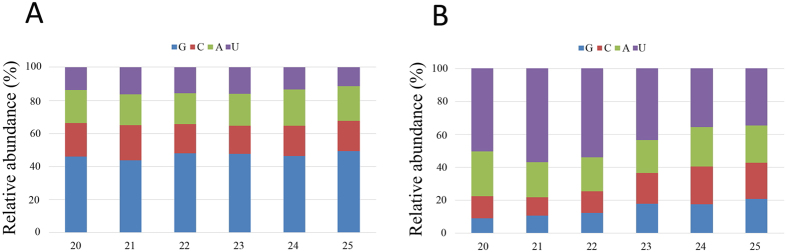
Identity of the 5′-terminal nucleotide among vsiRNAs. The identities of the 5′-terminal nucleotide of FgHV1-derived siRNAs (**A**) and FgHV2-derived siRNAs (**B**) were compared. The *y*-axis shows the percentages of 5′-terminal nucleotides consisting of G/C/A/U among the 20- to 25-nt vsiRNA class, and the *x*-axis represents the length distribution.

Previous reports demonstrated that the loading of sRNA onto an AGO-containing effector complex is guided by the 5′-terminal nucleotide of the sRNA. In *Arabidopsis,* AGO2 and AGO4 preferentially, but not exclusively, bind sRNAs beginning with a 5′-terminal adenosine (A), whereas AGO1 and AGO5 harbour microRNAs with 5′-terminal U and cytosine (C) residues, respectively^26^. The selectivity of AGO proteins is different in *Drosophila*, where AGO1 recruits sRNAs beginning with U, whereas sRNAs binding to AGO2 most frequently begin with C^27^. To examine the specificity of *F. graminearum*-encoded AGOs, the identities of the 5′-terminal nucleotides of 20- to 25-nt FgHV1-and FgHV2-derived siRNAs were analysed. Our bioinformatics analyses revealed that G and U were the most abundant nucleotides at the 5′-terminal position of FgHV1 and FgHV2 siRNAs, respectively (Fig. 6). Our results also indicated that the 5′-terminal nucleotides of the host sRNAs showed a preference for U (data not shown). These results indicate that different AGOs may be activated in *F. graminearum* by infection with FgHV1 and FgHV2 and that the antiviral silencing system of fungi may utilize AGO components that are distinct from those in plants and animals.

### Host genes targeted by vsiRNAs have varied functions, including functions related to purine ribonucleoside binding, oxidative stress and response to stimuli

**Figure 7:**
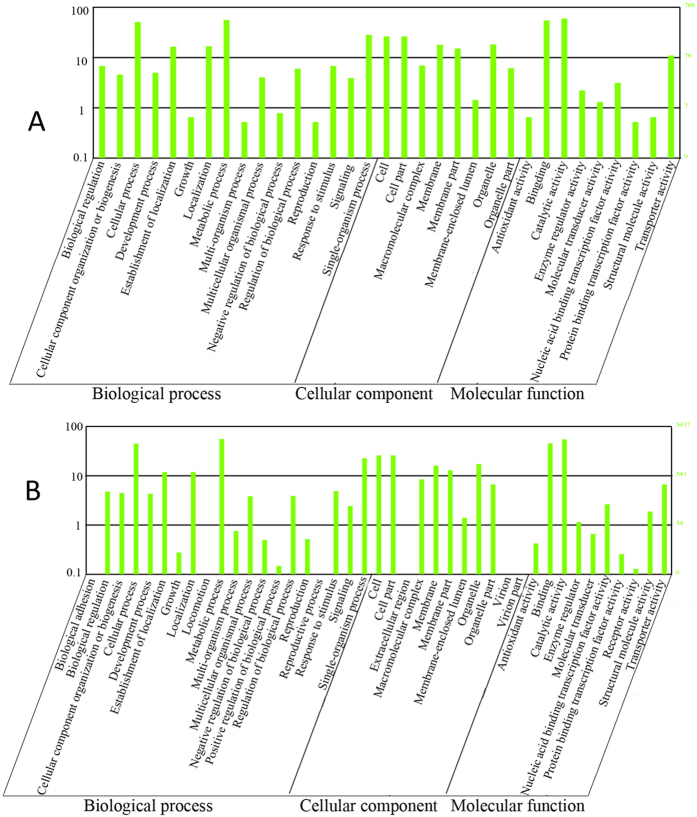
Histogram of the GO classification of genes putatively targeted by vsiRNAs. The GO terms for FgHV1-derived siRNA (**A**) and FgHV2-derived siRNA (**B**) putative target genes were classified into three ontologies: biological process, cellular component and molecular function. The *x*-axis indicates different GO terms. The left *y*-axis shows the percentage of genes in the main category, while the right *y*-axis represents the number of genes.

It has been experimentally shown that, during the interaction of vsiRNAs with host mRNAs, some vsiRNAs can guide host mRNA cleavage^28^,^29^. By targeting a host gene, vsiRNAs can post-transcriptionally modulate specific host genes using the host RNA silencing machinery, thereby modulating viral disease symptoms^30^. It was thus of great interest to examine the accumulation levels of transcripts that were targeted by FgHV1-derived sRNAs. We searched for vsiRNA-targeted mRNAs using MiRanda, which was developed to identify miRNA genomic targets^31^. As expected, a large number of potential transcripts were targeted by vsiRNAs; these genes are listed in Supplementary Tables S3 and S4. The alignment results showed that most of the hypovirus-derived siRNAs have single targets, while a portion are complementary to more than one transcript. Additionally, some host genes are targeted by several hypovirus-derived siRNAs at different base-pairing sites within the gene. Although most of the targeted genes encode hypothetical proteins, several genes that corresponded to a large number of FgHV1-derived siRNAs encode proteins involved in the nuclear envelope, the fungal-type vacuole, purine ribonucleoside binding, nucleoside-triphosphatase activity and cation-transporting ATPase activity, among other functions. In FgHV2-infected *F. graminearum*, genes that are related to the calcium channel complex, the mitochondrial proton-transporting ATP synthase complex, the peroxisomal membrane, translation factor activity, purine ribonucleoside binding, metal ion binding and amino acid synthase/transport, among others, were targeted by FgHV2-derived siRNAs. Notably, the RNA silencing-related genes FgDicer1 (FGSG_09025) and FgRdRp5 (FGSG_09076) were also predicted as potential targets of FgHV1- and FgHV2-derived siRNAs. In addition, we queried the Gene Ontology (GO) database with vsiRNA-targeted transcripts. The potential target genes of FgHV1- and FgHV2-derived siRNAs were classified into 33 and 41 GO term groups, respectively. These GO terms were classified within three ontologies, including biological process, cellular component and molecular function. The GO term analysis revealed that both the FgHV1- and FgHV2-regulated genes were significantly enriched for terms linked to nucleic acid-binding transcription factor activity, antioxidant activity and response to stimulus, among other categories (Fig. 7).

In the RNA silencing progress, vsiRNAs are loaded onto AGOs to form RISC complexes, which guide the degradation of viral RNA and result in specific gene silencing. To examine whether the vsiRNA-targeted host genes were down-regulated, we employed mRNA deep sequencing. The deep sequencing results revealed that the mRNA accumulation levels of some vsiRNA-targeted genes were down-regulated (Fig. S2). However, many target transcripts were not dramatically affected or even up-regulated. Based on these results, we suggest that hypovirus-derived siRNAs during infection may trigger vsiRNA-mediated host gene silencing via base pairing. However, the efficiency of vsiRNA-guided silencing is not closely correlated with vsiRNA abundance. It is reasonable to speculate that not all vsiRNAs can be recruited to guide AGO-containing RISC complexes during the cleavage of target genes. Furthermore, genes are often involved in multiple, distinct pathways and may be highly induced by non-RNA silencing-related pathways.

## Discussion

RNA silencing system plays a fundamental role as a defence mechanism against viruses, which uses sRNAs as key mediators to conduct gene silencing. In recent years, deep-sequencing technology has allowed researchers to analyse the sRNA profiles of a large variety of virus-infected tissues. Nevertheless, the majority of these studies have been restricted to virus-infected plants and insects, while large-scale vsiRNA investigation remains limited in fungi. Here, to elucidate the characteristics, origins and functions of fungal sRNAs, including milRNAs and vsiRNAs, we used Solexa-based deep sequencing of sRNAs. We obtained the first high-resolution vsiRNA maps for the hypoviruses FgHV1 and FgHV2, with 1,831,081 and 3,254,758 clean reads, respectively. The detection of large amounts of hypovirus-derived siRNAs directly demonstrated that mycoviral infection triggers fungal RNA silencing mechanisms. In a previous report, approximately 171 vsiRNAs were identified among the total sRNAs of *C. parasitica* infected with CHV1-EP713^16^. Although vsiRNAs accounted for the majority (73%) of the 233 cloned sRNAs from CHV1-EP713-infected *C. parasitica*, the relatively small number of vsiRNAs that were identified in *C. parasitica* may be not sufficient for understanding virus-triggered RNA silencing. In our study, the characterization of a much larger set of sRNAs may provide us with more accurate information regarding the properties of hypovirus-derived siRNAs and help us to understand the mechanism of RNA silencing in fungi.

The size distribution of the siRNAs reflected the pathways of viral genomic RNA cleavage, which relies on the hierarchical activity of DCLs. Notably, although the most dominant classes of FgHV1- and FgHV2-derived siRNAs were the 20-, 21-, 22-, 23- and 24-nt classes, these five classes did not form the majority of the total vsiRNA. This smaller proportion of 20- to 24-nt siRNAs in comparison to virus-infected plants suggests that DCLs in fungi may function differently from those in plants, which predominantly harbour DCL2, DCL 3 and DCL4 and generate a large proportion of 22-, 24- and 21-nt sRNAs^32^. In our study, the proportion of 24-nt-class sRNA reads in the FgHV1-infected library was much smaller than it was in the FgHV1-free library, as 24-nt sRNAs represented 11.58% of the FgHV1-infected library and 22.40% of the FgHV1-free library (Fig. 2). A large number of 24-nt FgHV1- and FgHV2-derived siRNAs were present, accounting for 8.10% and 8.64% of the total vsiRNAs, respectively (Fig. 4). The unique 24-nt vsiRNA generation mechanism in plants has been described, and it has also been reported that 24-nt vsiRNAs are generated in *Oryza sativa* and *Nicotiana benthamiana* but not in *Laodelphgax striatellus*^20^. Thus, it is of great interest to explore the relationship between the reduction of 24-nt sRNA accumulation and the generation of 24-nt vsiRNAs in virus-infected *F. graminearum.*

Previous work has demonstrated that the 5′-terminal nucleotides of sRNAs determine the sorting of sRNAs into different AGO-containing complexes^33^. In the present study, our bioinformatics analysis revealed that 20- to 25-nt FgHV2-derived siRNAs all tend to begin with U rather than with A, G or C residues. In addition, the 5′-terminal nucleotide of total *F. graminearum* sRNA showed a preference for U, suggesting that the *Arabidopsis* AGO1-related FgAGO protein may play a dominant role in the post-transcriptional gene silencing (PTGS) of *F. graminearum*. However, in contrast to FgHV2, the 5′-terminal nucleotide of FgHV1-derived siRNAs showed a bias towards G. Thus far, no AGOs in *Arabidopsis* or *Drosophila* have been demonstrated to show a preference for 5′-terminal G. This preference suggests that diverse AGO-containing RISC complexes may be involved in the incorporation of different virus-derived siRNAs. Another possibility is that not all of the generated vsiRNAs can be recruited to AGO-containing RISCs. The further sequencing of immunoprecipitated AGO complex-bound sRNAs will provide accurate information regarding the sequence preference properties of different AGOs in *F. graminearum.*

Our results indicated that there were more positive-strand-derived vsiRNAs than negative-strand-derived vsiRNAs in both the FgHV1- and FgHV2-infected libraries. Notably, a pattern of 60% positive and 40% negative polarity for vsiRNAs was also detected in a CHV1-EP713-infected sRNA library^16^. Previous reports have shown that different viruses display different vsiRNA sense/antisense ratios in the same plants, indicating that positive- and negative-strand vsiRNA production is not determined by the host, but rather, depends on the virus^20^,^34^. The observed similarities among the polarity ratios of vsiRNAs may result from the similar replication mechanism typically used by hypoviruses.

Previous studies have demonstrated that vsiRNA targeting and regulation of host-specific genes leads to the yellowing of virus-infected plants^30^. In our study, many host genes were predicted to be targets of FgHV1- and FgHV2-derived siRNAs. Strikingly, the RNA silencing-related genes FgDicer1 (FGSG_09025) and FgRdRp5 (FGSG_09076) were targeted by both FgHV1- and FgHV2-derived siRNAs. Phylogenetically, FgDicer1 is closely related to the Dicer1 proteins of *Magnaporthe oryzae*, *C. parasitica* and *N. crassa,* while *F. graminearum* FgRdRp5 is more closely related to *A. thaliana* RdRp2 and RdRp6^17^. Although FgDicer1 and FgRdRp5 may not play critical roles in the hairpin RNA (hpRNA)-mediated gene silencing process, they most likely play specific roles in the antiviral response of *F. graminearum*. Some vsiRNA-guided cleavage of host mRNAs has been reported in plant-virus interactions^28^,^29^, but the phenomenon of vsiRNAs targeting host mRNAs that encode genes related to RNA silencing has not to our knowledge been previously observed in virus-infected animals, plants, or fungi. RNA silencing system-generated viral sRNA may in turn direct the degradation of RNA silencing-related genes, thereby protecting itself from further degradation via the host RNA silencing mechanism. In addition to the viral suppressors of RNA silencing (VSR) strategy, this approach may be another anti-viral defence mechanism of hypovirus-infected *F. graminearum*.

Some reports have suggested that the RNA-silencing components of *F. graminearum* have roles in antiviral defence processes. For example, the expression levels of FGSG_08752 (agl1), FGSG_04408 (dcl2) and FGSG_01582 (rdr4) were significantly up-regulated at both 36 h and 5 d in the FgHV2-infected strain relative to the FgHV2-free strain^19^. Recently, Chen *et al.* reported that FgAgo1 and FgDicer2 may play critical roles in the hpRNA-mediated gene silencing process, whereas FgAgo2 and FgDicer1 may be required for the meiotic silencing via unpaired DNA (MSUD) pathway in *F. graminearum*^17^. In the present study, we also found by qRT-PCR examination that the expression levels of certain DCLs, AGOs and RNA-directed RNA polymerases (RDRs) in FgHV1-infected *F. graminearum* were significantly up-regulated (data not shown). In addition to our analysis of the size distribution and 5′-terminal constitution of FgHV1- and FgHV2-derived siRNAs, we also found that FgAgo1 and FgDCL2 might play fundamental roles during hypoviral infection. As with Dicers and AGOs, the expression levels of RDRs were also elevated in FgHV1- and FgHV2-infected *F. graminearum.* However, it has been reported that *rdr* knock-out strains show no increased susceptibility to mycoviral infection by *C. parasitica*^35^. The roles of the RDRs of *F. graminearum* during infection with hypoviruses require further investigation.

Viruses possess counter-measures to escape host antiviral responses. Many RNA silencing suppressors, targeting different silencing stages, have been reported and are diverse in their amino acid sequences and protein structures^36^,^37^,^38^. Similarly to pathogenic viruses of mammals, insects and plants, hypoviruses also encode protein suppressors of RNAi. The papain-like protease p29, encoded by CHV1, functions as a suppressor of RNA silencing in the natural fungal host^39^. Another example of a mycovirus able to suppress RNA silencing is the Rosellinia necatrix mycoreovirus 3, whose S10 gene exhibits RNA silencing suppressor activity^40^. FgHV1 encodes two papain-like proteinases, P20 and P25, which are closely related to the CHV1-encoded RNA suppressor p29. FgHV2 also encodes a papain-like protease, consisting of 105 amino acids, which is closely related to P25. *Agrobacterium* transient expression assays in *N. benthamiana* for the definition of RNA suppressors indicated that FgHV1-encoded P20 is a potential RNAi suppressor (L.H. Guo, unpublished data). The suppression of 24-nt sRNA in the FgHV1-infected library might be related to the FgHV1-encoded VSR, which may interfere with the function of some Dicers.

## Methods

### Fungal isolates and culture conditions

We used the FgHV1-infected *F. graminearum* strain HN10 along with the virus-free strain HN10-11F, which is an isogenic strain that was derived from strain HN10 by protoplast isolation and regeneration. *F. graminearum* strain JS16 harboured FgHV2, while the virus-free strain JS16F was derived from strain JS16 by treatment with 80–100 mM ribavirin. All strains were preserved in our laboratory. Mycelial plugs and conidia were stored in 25% glycerol at –80 °C. *F. graminearum* was cultured on potato dextrose agar medium at 25 °C. Activated mycelial plugs of strains HN10, HN10-11F, JS16 and JS16F were placed into 100 ml of potato dextrose broth (PDB) and cultured for 4 days at 25 °C.

### RNA isolation and RNA (sRNA and mRNA) deep sequencing

Mycelial masses were harvested and immediately frozen in liquid nitrogen. Double-stranded RNA was extracted as previously described^18^. Total RNA was extracted using TRIzol reagent (Invitrogen, Carlsbad, USA) according to the manufacturer’s protocols. The presence or absence of viruses in HN10, HN10-11F, JS16 and JS16F was detected by RT-PCR. Total RNA extracts from these four *F. graminearum* strains were used as templates with virus-specific primers.

For Illumina sequencing of the sRNA, we pooled the RNA from three replicates of each strain. Solexa sequencing was performed as previously described. The sRNAs that were shorter than 44 nt were purified from a 15% Tris-borate-EDTA (TBE)–urea polyacrylamide gel. Next, before sequencing, 5′- and 3′-adaptors were ligated onto each sRNA fragment according to the Illumina manufacturer’s instructions (BGI, Beijing, China). The sRNAs were then sequenced using the Illumina Solexa platform. As a control, we sequenced the isolated sRNAs from the virus-free strains.

Impurities in the raw data include 5′ primer contaminants, missing insert tags, oversized insertions, low-quality reads, poly(A) (adenine) tags and small tags. Among these, missing insert tags and 5′ primer contaminants are defined as adaptor contaminants, while oversized insertions manifest as missing 3′ primers. The data are processed with the following steps: 1) eliminating low-quality reads; 2) eliminating reads with 5′ primer contaminants; 3) eliminating reads without a 3′ primer; 4) eliminating reads without an insert tag; 5) eliminating reads with poly(A); 6) eliminating reads shorter than 18 nt; and 7) summarising the length distribution of the clean reads.

mRNA was isolated from the prepared RNA using oligo(dT) magnetic beads, mixed with fragmentation buffer and fragmented into short fragments. Next, cDNA was synthesized using the mRNA fragments as templates. Short fragments were purified and resolved with EB buffer for use in end repair and single-nucleotide A addition. The short fragments were then ligated to adapters. After agarose gel electrophoresis, suitable fragments were selected as templates for PCR amplification. During the quality-control (QC) steps, an Agilent 2100 Bioanalyzer and an ABI StepOnePlus Real-Time PCR System were used to quantify the samples and to assess the quality of the sample library. Finally, the libraries were sequenced using the Illumina HiSeq^TM^ 2000 system at BGI-Shenzhen. The raw reads produced from the Illumina HiSeq^TM^ 2000 were filtered to produce a set of clean reads. The data from the raw sRNA reads were deposited in the NCBI Sequence Read Archive (SRA, http://www.ncbi.nlm.nih.gov/sra/) under accession number SRA307939.

### Data analysis

The clean reads were aligned to the reference genome, namely the *F. graminearum* PH-1 genome, using the Short Oligonucleotide Analysis Package (SOAP) with the default parameters^41^. Nucleotide frequencies per position were displayed using the WebLogo program^42^. Subsequently, “MIREAP” software, which can identify novel candidates with a canonical hairpin structure (https://sourceforge.net/projects/mireap), was used to predict the milRNAs of *F. graminearum*. At the same time, miRNA conservation was considered, and we chose the miRNA with the highest expression for each mature miRNA family. The target genes of the potential miRNAs were predicted using the psRobot and TargetFinder programs. The expression levels of miRNAs were evaluated by summing the count of the tags that aligned to the temporary miRNA database with two or fewer mismatches.

To identify hypovirus-generated sRNAs, the clean reads from the four libraries were also aligned to the FgHV1 and FgHV2 genomes. The virus-derived reads were then analysed using Perl scripts and Microsoft Excel as previously described^43^. vsiRNA target prediction was conducted using previously developed criteria^44^. The prediction was implemented in the MIREAP program developed by BGI (Shenzhen, China). The rules used for target prediction were as follows: 1) no more than four mismatches between the sRNA and the potential target (G-U bases count as 0.5 mismatches); 2) no more than two adjacent mismatches in the miRNA/target duplex; 3) no adjacent mismatches at positions 2–12 of the miRNA/target duplex (5′ of miRNA); 4) no mismatches at positions 10–11 of the miRNA/target duplex; 5) no more than 2.5 mismatches in positions 1–12 of the of the miRNA/target duplex (5′ of miRNA); and 6) the minimum free energy (MFE) of the miRNA/target duplex must be ≥ 75% of the MFE of the miRNA bound to its perfect complement. All targeted transcripts were mapped to GO terms in the database (http://www.geneontology.org/), and gene numbers were calculated for every GO term. Significantly enriched GO terms were identified using a hypergeometric test on the input list of differentially expressed genes (DEGs). We developed a strict algorithm to perform the analysis, which was based on GO_TermFinder (http://smd.stanford.edu/help/GO-TermFinder/GO_TermFinder_help.shtml/). The calculated p-values were Bonferroni-corrected, and GO terms with corrected p-values ≤0.05 were considered to be significantly enriched in the DEGs. The secondary structure predictions for viral genomic RNA were performed using Mfold (version 2.3 energies) (http://mfold.rna.albany.edu/?q=mfold/RNA-Folding-Form2.3) with an assumed temperature of 25 °C^45^,^46^.

## Additional Information

**How to cite this article**: Wang, S. *et al.* Generation of a high resolution map of sRNAs from *Fusarium graminearum* and analysis of responses to viral infection. *Sci. Rep.* **6**, 26151; doi: 10.1038/srep26151 (2016).

## Supplementary Material

Supplementary Information

## Figures and Tables

**Table 1:** Summary statistics of small RNAs in the hypovirus-free and hypovirus-infected libraries.

**Library type**	**Total sRNA reads**	**Clean sRNA reads**	**Percent (Total reads)**	**Total FgHV1-derived sRNA reads**	**Total FgHV2-derived sRNA reads**	**Percent (Clean reads)**
FgHV1-free	12,108,968	11,693,092	96.56%	0	N/A	0
FgHV1-infected	11,423,975	11,090,406	97.08%	1,831,081	N/A	16.51%
FgHV2-free	13,392,162	12,778,696	95.42%	N/A	1,539	0.01%
FgHV2-infected	13,586,176	13,149,451	96.78%	N/A	3,254,758	24.75%
