# Supplementary material for: Generation of a high resolution map of sRNAs from Fusarium graminearum and analysis of responses to viral infection
Source: Sci Rep. 2016 May 18;6:26151. doi: 10.1038/srep26151 (PMC4870495; doi:10.1038/srep26151)
Supplement: Supplementary Information [file srep26151-s1.pdf]

Supplementary Information

Generation of a high resolution map of sRNAs from *Fusarium graminearum* and analysis of responses to viral infection

Shuangchao Wang, Pengfei Li, Jingze Zhang, Dewen Qiu, Lihua Guo*

State Key Laboratory for Biology of Plant Disease and Insect Pests, Institute of Plant Protection, Chinese Academy of Agricultural Science, Beijing, 100081, China

* Corresponding author

E-mail: guolihua72@yahoo.com (L.-H. Guo)

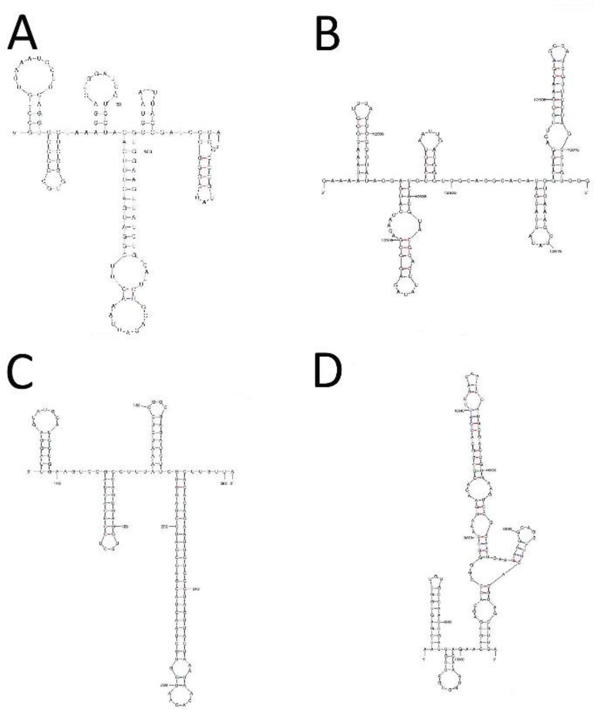

Fig. S1. Secondary structure of viral positive-strand RNA in the 5'- and 3'-terminal regions. The numbers represent the nucleotide positions in the FgHV1 and FgHV2 genomes. A, 5'-terminal region from nt 1 to nt 134 in the FgHV1 genome. B, 3'-terminal region from nt 12,877 to nt 13,023 of the FgHV1 genome. C, 5'-terminal region of the FgHV2 genome from nt 130 to nt 270. D, 3'-terminal region of the FgHV2 genome from nt 9,961 to nt 10,100.

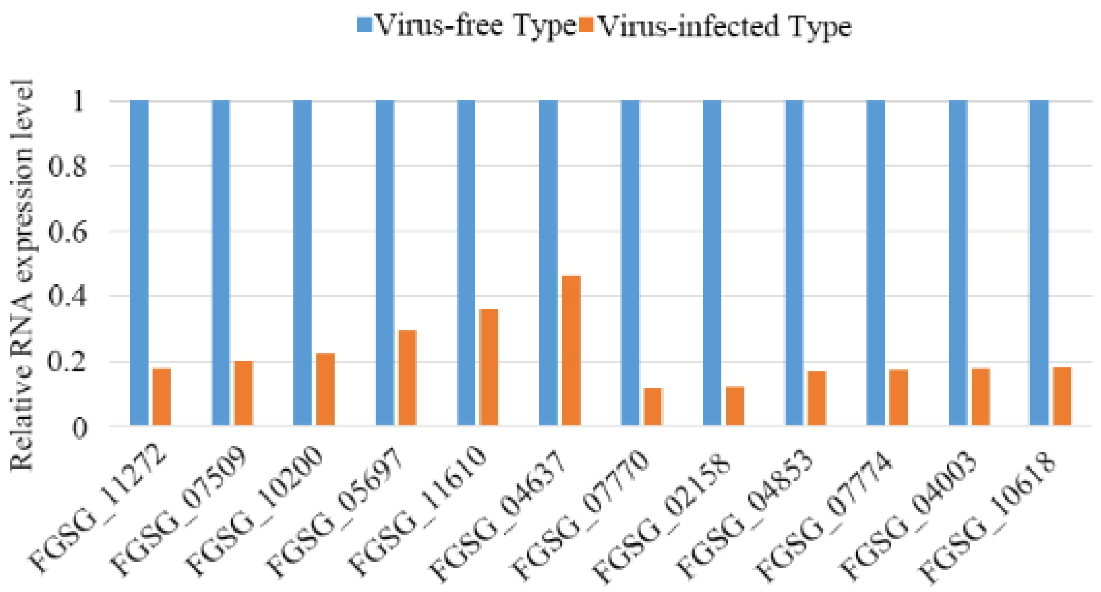

Fig. S2. The relative expression levels of selected down-regulated genes whose transcripts were targeted by FgHV1- and FgHV2-derived siRNAs, as assessed by high-throughput sequencing. FGSG_11272, FGSG_07509, FGSG_10200, FGSG_05697, GSG_11610 and FGSG_04637 were targeted by FgHV1-derived siRNAs, while FGSG_07770, FGSG_02158, FGSG_04853, FGSG_07774, FGSG_04003 and FGSG_10618 were targeted by FgHV2-derived siRNAs.

Table S1. The milRNA candidates identified in FgHV1-free and FgHV1–infected *F. graminearum* types.

milRNA	milRNA_seq	Length (nt)	HN10-11F expression	HN10 expression	Location	Mfe (kcal/mol)	Targets
**Fg-HN-milR 1**	AGGGACAAGCCUUG CGGCAGCU	22	5	0	CM000574:3520752:3520824:+	-27.60	FGSG_12210 FGSG_05411
**Fg-HN-milR 2**	UAGGAAAGGCAGUU AACUAGGA	22	66280	4575	CM000574:5515558:5515661:+	-66.00	NA
**Fg-HN-milR 3**	AAGAAAAUGGGAGC GAGCACA	21	21	0	CM000574:6007796:6007889:+	-25.6	FGSG_01263 FGSG_10946 FGSG_04253
**Fg-HN-milR 4**	AGGAACGGGGUACU GCAGUCUAC	23	5	0	CM000574:10743730:10743840:+	-21.7	NA
**Fg-HN-milR 5**	UGAGGUUGUAGCGG AGGUCGA	21	13	0	CM000574:11676586:11676680:+	-32.20	FGSG_02181 FGSG_02882 FGSG_04153 FGSG_05085 FGSG_06351 FGSG_06531 FGSG_07083 FGSG_10713 FGSG_10799 FGSG_09706 FGSG_02963 FGSG_09344

milRNA	milRNA_seq	Length (nt)	HN10-11F expression	HN10 expression	Location	Mfe (kcal/mol)	Targets
**Fg-HN-milR 6**	UUACUGUCAAGCUC AUAUCUC	21	100	0	CM000574:2786882:2786989:-	-27.9	FGSG_02657<br>FGSG_10558<br>FGSG_12278<br>FGSG_12891<br>FGSG_12963<br>FGSG_09085<br>FGSG_05520<br>FGSG_05577
**Fg-HN-milR 7**	UCGGUUGUAGGAAA GCAAGGAA	22	6	0	CM000574:3600517:3600861:-	-84.60	FGSG_07288<br>FGSG_11559<br>FGSG_09031
**Fg-HN-milR 8**	UUAUAUAUUUUUUG ACUGUCAA	22	499	28	CM000574:5381073:5381226:-	-74.20	NA
**Fg-HN-milR 9**	GAUAAGGAGUGGUC GAGCGGGA	22	142	882	CM000574:10112530:10112662:-	-41.17	FGSG_05902
**Fg-HN-milR 10**	UGGAGACGAAUGCG AUCUUU	20	6	0	CM000574:10724128:10724276:	-64.1	FGSG_06166<br>FGSG_13172<br>FGSG_09998<br>FGSG_04752<br>FGSG_03386<br>FGSG_00985<br>FGSG_05533<br>FGSG_02540<br>FGSG_05695

milRNA	milRNA_seq	Length (nt)	HN10-11F expression	HN10 expression	Location	Mfe (kcal/mol)	Targets
							FGSG_00281
**Fg-HN-milR 11**	UGGCAUGUUGGACA AAAGGUGUA	23	5	0	CM000575:2257626:2257769:+	-40.90	NA
**Fg-HN-milR 12**	UGUGACGGUUGGGU UUCUAA	20	6	0	CM000575:3418672:3418918:+	-57.10	FGSG_05270<br>FGSG_08920<br>FGSG_09051<br>FGSG_10059<br>FGSG_04629
**Fg-HN-milR 13**	UGGAAUGCUGCAUA UACUCAC	21	5	0	CM000575:2620855:2621202:-	-92.70	FGSG_03362<br>FGSG_04672
**Fg-HN-milR 14**	UCUUGAUAGUGAAU AGAGCGG	21	43	0	CM000575:3418255:3418361:-	-27.1	FGSG_00862<br>FGSG_04336<br>FGSG_12834<br>FGSG_07084
**Fg-HN-milR 15**	AAGCCUUCUCAAUU UACUCAAA	22	2521	0	CM000575:4472900:4472973:-	-37.3	FGSG_04063
**Fg-HN-milR 16**	UGGGAACUUGGAUG AAGCGA	20	7	0	CM000575:8881160:8881347:-	-61.7	FGSG_04019<br>FGSG_04287<br>FGSG_05224<br>FGSG_06633<br>FGSG_09374<br>FGSG_10848<br>FGSG_11518<br>FGSG_08490

milRNA	milRNA_seq	Length (nt)	HN10-11F expression	HN10 expression	Location	Mfe (kcal/mol)	Targets
							FGSG_11019
**Fg-HN-milR 17**	UGAGUAGAAUGUAA AGGGUAA	21	5	0	CM000576:96045:96372:+	-81.32	FGSG_00096<br>FGSG_12590
**Fg-HN-milR 18**	UGGGCUGAGGUGGU GGUGGGCA	22	5	0	CM000576:3930306:3930461:+	-53.2	FGSG_00660<br>FGSG_00987<br>FGSG_05904<br>FGSG_06397<br>FGSG_06687<br>FGSG_07554<br>FGSG_01349
**Fg-HN-milR 19**	ACAUACGACCAUAG CUCGCAG	21	151	52	CM000576:6228266:6228364:+	-26.20	NA
**Fg-HN-milR 20**	AGGGAAAGAUGGGU AGAUGCA	21	11	0	CM000576:6298440:6298508:+	-27.70	FGSG_04092<br>FGSG_10353<br>FGSG_11195<br>FGSG_00410<br>FGSG_13573
**Fg-HN-milR 21**	AGGGAAAGAUGGGU AGAUGCA	21	11	14	CM000576:6298440:6298508:+	-37.48	FGSG_04092<br>FGSG_10353<br>FGSG_11195<br>FGSG_00410<br>FGSG_13573
**Fg-HN-milR 22**	AUCGCUGUGGAAGA AACGUGGA	22	10	0	CM000576:1015298:1015387:-	-20.20	FGSG_06914

milRNA	milRNA_seq	Length (nt)	HN10-11F expression	HN10 expression	Location	Mfe (kcal/mol)	Targets
**Fg-HN-milR 23**	UGGAGAACAGUGUA GACAGCCC	21	19	0	CM000576:3376564:3376644:-	-29.4	FGSG_05784
**Fg-HN-milR 24**	UGGCUUGGAAGACA CGGGCAUA	21	6	0	CM000576:5381979:5382219:-	-54.80	FGSG_02233<br>FGSG_13355<br>FGSG_05249<br>FGSG_11200
**Fg-HN-milR 25**	UCUCAAACUUUAGC GAUGUGUGA	22	2721	0	CM000576:6274314:6274458:-	-95.10	FGSG_13893
**Fg-HN-milR 26**	AGGCAACUUGGUCU UGGGCUU	20	8	0	CM000577:572153:572283:+	-83.40	FGSG_01194<br>FGSG_05229<br>FGSG_07068<br>FGSG_11398<br>FGSG_12312<br>FGSG_12734<br>FGSG_13004<br>FGSG_12717<br>FGSG_01460<br>FGSG_02585<br>FGSG_06498
**Fg-HN-milR 27**	GCACAUGACUGUGG CCAUGGCU	21	6	0	CM000577:3157595:3157691:+	-44.30	FGSG_06080<br>FGSG_05653
**Fg-HN-milR 28**	UGAUGGACUUGGAC GUCGGCAA	21	8	0	CM000577:6510865:6511200:+	-113.6	FGSG_02409<br>FGSG_08851
**Fg-HN-milR 29**	GUUGAACGAACCUU GGCCGAA	20	20	0	CM000577:7772806:7772899:+	-19.85	FGSG_08632

milRNA	milRNA_seq	Length (nt)	HN10-11F expression	HN10 expression	Location	Mfe (kcal/mol)	Targets
**Fg-HN-milR 30**	GGGUCUGUGUAACC UCCCGGUUU	23	42	92	CM000577:265595:265684:-	-37.20	FGSG_07081<br>FGSG_10189
**Fg-HN-milR 31**	UGGAGAAAAUUGAG AAGGCUCGA	23	102	0	CM000577:7080427:7080536:-	-79.80	FGSG_04063<br>FGSG_12651
**Fg-HN-milR 32**	UCUGGCAUUGUUGU GAUGAA	20	5	0	CM000577:7214543:7214828:-	-142.34	FGSG_09213<br>FGSG_11111<br>FGSG_11141<br>FGSG_13582<br>FGSG_04615<br>FGSG_03303<br>FGSG_12092<br>FGSG_06097<br>FGSG_00423<br>FGSG_03619
**Fg-HN-milR 33**	UCAGAGCGGUGAUU GAUCAAGAU	23	25	0	CM000577:7215152:7215334:-	-76.70	NA
**Fg-HN-milR 34**	CGGGCUGGGUCUUG GGGAGGGG	22	0	10	CM000574:1084492:1084801:+	-115.40	FGSG_01111<br>FGSG_06658<br>FGSG_05367
**Fg-HN-milR 35**	AGUCAACGUAUGGA GCUAUGGCU	23	0	19	CM000574:1351324:1351394:+	-42.10	FGSG_13231
**Fg-HN-milR 36**	GCUGGUGCUGGUGC AGAUUUG	20	0	7	CM000574:6007701:6007771:+	-33.50	FGSG_03742<br>FGSG_05325<br>FGSG_05475

milRNA	milRNA_seq	Length (nt)	HN10-11F expression	HN10 expression	Location	Mfe (kcal/mol)	Targets
							FGSG_05590
							FGSG_07168
							FGSG_07386
							FGSG_09997
							FGSG_11378
							FGSG_12109
							FGSG_13646
							FGSG_13928
							FGSG_04554
							FGSG_06975
							FGSG_11210
							FGSG_11848
							FGSG_09574
							FGSG_03378
							FGSG_13677
							FGSG_05418
							FGSG_11819
							FGSG_10883
							FGSG_02463
							FGSG_13904
							FGSG_02952
							FGSG_11956
							FGSG_00723

milRNA	milRNA_seq	Length (nt)	HN10-11F expression	HN10 expression	Location	Mfe (kcal/mol)	Targets
							FGSG_12768<br>FGSG_13583
**Fg-HN-milR 37**	UGUGAAAUGUGAGG GGCUC	20	0	15	CM000574:8092964:8093291:+	-63.63	FGSG_03603<br>FGSG_03749<br>FGSG_05306<br>FGSG_06840<br>FGSG_09912<br>FGSG_04303<br>FGSG_09581<br>FGSG_03979
**Fg-HN-milR 38**	UCGAGCUAGGAACG GAAGCCA	21	0	5	CM000574:9382922:9383069:+	-47.60	NA
**Fg-HN-milR 39**	GUUCUUUGCGAUCG UCUCAAGCA	23	0	12	CM000574:1521613:1521808:-	-48.90	FGSG_05325
**Fg-HN-milR 40**	GAGGGAGGAGGUCU AAAGAUU	21	0	7	CM000574:6821114:6821192:-	-25.77	FGSG_00666<br>FGSG_07031<br>FGSG_05358<br>FGSG_03431
**Fg-HN-milR 41**	UGGGUCUGGGAGUG GUAGGGGA	22	0	8	CM000574:7889020:7889356:-	-114.71	FGSG_11269
**Fg-HN-milR 42**	CGUAGGAUUCUGAG CGACGACC	22	0	8	CM000574:9370170:9370445:-	-91.00	FGSG_09986
**Fg-HN-milR 43**	UGGGAGGGUGUGGA GAGGAA	20	0	23	CM000575:893840:893965:+	-45.20	FGSG_00951<br>FGSG_04384<br>FGSG_04465

milRNA	milRNA_seq	Length (nt)	HN10-11F expression	HN10 expression	Location	Mfe (kcal/mol)	Targets
							FGSG_05274<br>FGSG_05658<br>FGSG_06799<br>FGSG_07177<br>FGSG_07484<br>FGSG_08474<br>FGSG_07863<br>FGSG_09183<br>FGSG_02191<br>FGSG_07623<br>FGSG_05788<br>FGSG_00859<br>FGSG_00121<br>FGSG_01200
**Fg-HN-milR 44**	GUGGAUGGGAACUG GGUGGGUU	22	0	18	CM000575:3861705:3861869:+	-46.60	FGSG_09744<br>FGSG_06772<br>FGSG_08207
**Fg-HN-milR 45**	UGUCUCUGGAUCUG UUGACGA	21	0	6	CM000575:6472000:6472181:+	-32.3	FGSG_11270<br>FGSG_10205<br>FGSG_06694<br>FGSG_09070<br>FGSG_01219
**Fg-HN-milR 46**	GGAGGAGGCGACUG CGACUGGG	22	0	5	CM000575:6679624:6679700:+	-34	FGSG_00278

milRNA	milRNA_seq	Length (nt)	HN10-11F expression	HN10 expression	Location	Mfe (kcal/mol)	Targets
							FGSG_03525<br>FGSG_06123<br>FGSG_01082<br>FGSG_02719<br>FGSG_06769<br>FGSG_00332<br>FGSG_08445
**Fg-HN-milR 47**	UCAGAACUCAGAAG ACUCUGUA	22	0	8	CM000576:3313332:3313505:+	-52.61	FGSG_12695
**Fg-HN-milR 48**	GACAAGGAGUGGUC GAGCGGU	21	0	14	CM000576:6749680:6749823:+	-37.48	FGSG_00610<br>FGSG_09076<br>FGSG_01164<br>FGSG_11970<br>FGSG_01819<br>FGSG_13019
**Fg-HN-milR 49**	UGGGAAUCUAGAUU GACUCGCA	22	0	5	CM000576:381967:382157:-	-61.70	FGSG_06788
**Fg-HN-milR 50**	GGGGAGGUCAGGUA UGAGAUUGA	23	0	9	CM000576:1383044:1383145:-	-32.60	NA
**Fg-HN-milR 51**	UCUCAAACUUUAGC GAUGUGUGA	23	0	523	CM000576:6274325:6274448:-	-82.9	FGSG_13893
**Fg-HN-milR 52**	GAGGGAGGCUAGUG GGAAGGAC	22	0	14	CM000577:2896023:2896243:+	-59.90	FGSG_07075<br>FGSG_07543<br>FGSG_10510
**Fg-HN-milR 53**	GCACAUGACUGUGG CCAUGGCUU	23	0	6	CM000577:3157650:3157898:+	-60.40	FGSG_06080

milRNA	milRNA_seq	Length (nt)	HN10-11F expression	HN10 expression	Location	Mfe (kcal/mol)	Targets
**Fg-HN-milR 54**	UAGCAGAAAAGUUG ACCUCUUA	22	0	10	CM000577:3543035:3543180:+	-39.80	FGSG_06605 FGSG_12422 FGSG_03868 FGSG_06552
**Fg-HN-milR 55**	AAGCUAUUGCUGUC AUCGUCGU	22	0	6	CM000577:7036605:7036676:-	-23.20	FGSG_02983 FGSG_04619 FGSG_13541 FGSG_05606 FGSG_11769
**Fg-HN-milR 56**	GAGGAGAAGUGUUU UGGAAUGU	22	0	17	CM000577:7378306:7378387:-	-24.90	FGSG_04907 FGSG_07258 FGSG_09424 FGSG_13521 FGSG_14026
**Fg-HN-milR 57**	AGGGGGAAGAAAUA GUAGAA	20	50	0	CM000575:4554:4844:+	-84.74	FGSG_04764 FGSG_05972 FGSG_07915 FGSG_09063 FGSG_13783 FGSG_08933 FGSG_12735 FGSG_12849 FGSG_13654
**Fg-HN-milR 58**	UGUUGUUACGAUCU GCUGAG	19	4396	0	CM000575:2186569:2186767:+	-57.10	FGSG_12355 FGSG_01690 FGSG_13987
**Fg-HN-milR 59**	UUUGGGAUGGCAAA UGGAUAAGA	22	1580	1343	CM000576:6528158:6528420:+	-119.31	FGSG_05332

milRNA	milRNA_seq	Length (nt)	HN10-11F expression	HN10 expression	Location	Mfe (kcal/mol)	Targets
**Fg-HN-milR 60**	GGCAGCAUUGUUGA CAGGGCC	19	81	0	CM000577:7772484:7772835:-	-108.10	FGSG_01637<br>FGSG_01834<br>FGSG_03598<br>FGSG_06942<br>FGSG_09366<br>FGSG_09966<br>FGSG_06452<br>FGSG_09028<br>FGSG_10551

Table S2. The milRNA candidates identified in FgHV2-free and FgHV2–infected *F. graminearum* types.

milRNA	milRNA_seq	Length (nt)	JS16F expression	JS16 expression	Location	Mfe (kcal/mol)	Targets
**Fg-JS-milR 1**	GACAACGGGCCGAGGGA	21	974	596	Supercontig_3.1:5018990:5019096:+	-35.60	FGSG_12557<br>FGSG_07439
**Fg-JS-milR 2**	AGGAAAGGCAGAACAGGA	22	31885	46689	Supercontig_3.1:5482953:5483056:+	-66.60	NA
**Fg-JS-milR 3**	AGAGCAAGAAAAGGGCA	21	13969	11194	Supercontig_3.1:5755961:5756071:-	-61.10	FGSG_13512
**Fg-JS-milR 4**	GGCAACGGACGGAGGGA	21	3860	1905	Supercontig_3.7:1804571:1804926:+	-89.30	FGSG_05095<br>FGSG_04575
**Fg-JS-milR 5**	AAACGAAGCCGAAGCCCA	23	136	570	Supercontig_3.2:3942251:3942392:-	-56.00	NA
**Fg-JS-milR 6**	CGCAAAGACGAAGAGA	21	11929	7817	Supercontig_3.4:2740356:2740644:-	-67.20	FGSG_12963<br>FGSG_12844
**Fg-JS-milR 7**	GCGGGAGCCAGGGGAGA	23	827	371	Supercontig_3.5:1501580:1501684:-	-23.50	NA
**Fg-JS-milR 8**	GACAAGGAGGGCGAGCGG	21	23	41	Supercontig_3.8:1398664:1398807:+	-37.13	FGSG_11970<br>FGSG_01819<br>FGSG_13019<br>FGSG_01164<br>FGSG_09076<br>FGSG_00610
**Fg-JS-milR 9**	GAAGGACCACGA	20	95	393	Supercontig_3.3:5257246:5257364:+	-27.10	FGSG_08249<br>FGSG_12963

milRNA	milRNA_seq	Length (nt)	JS16F expression	JS16 expression	Location	Mfe (kcal/mol)	Targets
							FGSG_13474<br>FGSG_05084<br>FGSG_12951<br>FGSG_03343<br>FGSG_12126
**Fg-JS-milR 10**	GGGCGGAACCCCCGG	23	11	20	Supercontig_3.4:252091:252177:-	-37.60	FGSG_07081<br>FGSG_10189
**Fg-JS-milR 11**	CGCCGGGGCCAAG	20	8	18	Supercontig_3.5:2114277:2114386:+	-75.10	FGSG_03964<br>FGSG_01248<br>FGSG_10580<br>FGSG_08720<br>FGSG_12286
**Fg-JS-milR 12**	CCGGAGGGAGGGCA	21	532142	320022	Supercontig_3.1:6635370:6635617:+	-68.50	FGSG_08434
**Fg-JS-milR 13**	GAACGAAGAGAGAACAA	22	21	30	Supercontig_3.2:375355:375467:-	-55.40	FGSG_00710
**Fg-JS-milR 14**	AGGCGGCGGAGGGCC	22	35	14	Supercontig_3.3:1221753:1221840:+	-28.80	NA
**Fg-JS-milR 15**	GGACGAACCGGCGAGCA	23	19	27	Supercontig_3.5:1083430:1083519:+	-32.50	NA
**Fg-JS-milR 16**	GAGGGAAAGGAAGCACA	23	0	1794	Supercontig_3.13:11371:11487:+	-41.10	NA
**Fg-JS-milR 17**	GCGAGGCGAGGAGAGG	21	0	2023	Supercontig_3.6:683369:683475:-	-45.70	FGSG_06884<br>FGSG_06970<br>FGSG_04890<br>FGSG_04875

milRNA	milRNA_seq	Length (nt)	JS16F expression	JS16 expression	Location	Mfe (kcal/mol)	Targets
							FGSG_00284<br>FGSG_10523
**Fg-JS-milR 18**	AGGAACAGAAAAAAAGCC	22	0	30	Supercontig_3.2:3018610:3018759:+	-34.60	FGSG_08099
**Fg-JS-milR 19**	AAGGAGGGCGAGCGGGAA	22	0	9	Supercontig_3.7:1147520:1147649:-	-39.48	FGSG_05587<br>FGSG_05571
**Fg-JS-milR 20**	CAGAGCGGGAGACAAGA	22	0	6	Supercontig_3.6:806259:806439:+	-77.90	NA
**Fg-JS-milR 21**	GGGACGGGAGAGAGCCA	22	0	7	Supercontig_3.3:4452261:4452578:-	-95.10	FGSG_07285<br>FGSG_06746
**Fg-JS-milR 22**	CGGCGGGGACCGGCGG	20	0	5	Supercontig_3.1:817870:817957:+	-42.70	FGSG_01050<br>FGSG_05093<br>FGSG_12002<br>FGSG_09783<br>FGSG_02077<br>FGSG_05691<br>FGSG_10357
**Fg-JS-milR 23**	GCAGACGAGACGAC	21	0	7	Supercontig_3.1:4461613:4461681:+	-21.20	FGSG_01358
**Fg-JS-milR 24**	ACACGAGGCGGCGCCA	21	0	10	Supercontig_3.1:8214042:8214354:+	-107.80	FGSG_11210<br>FGSG_10676<br>FGSG_06554<br>FGSG_02565
**Fg-JS-milR 25**	AGGAGAACGAGGAC	21	0	5	Supercontig_3.1:1561062:1561193:-	-27.30	FGSG_00512

milRNA	milRNA_seq	Length (nt)	JS16F expression	JS16 expression	Location	Mfe (kcal/mol)	Targets
**Fg-JS-milR 26**	GGAGCAGAGGGC	23	0	24	Supercontig_3.1:8517266:8517380:-	-32.90	NA
**Fg-JS-milR 27**	GCGGAGGGACGAA	20	0	7	Supercontig_3.2:2713747:2713930:-	-54.10	FGSG_09515<br>FGSG_08568<br>FGSG_12961<br>FGSG_10874<br>FGSG_12715<br>FGSG_09939
**Fg-JS-milR 28**	ACGACGACGGAAGGCGGCG	22	0	10	Supercontig_3.4:3421372:3421547:-	-53.50	FGSG_07405<br>FGSG_08489
**Fg-JS-milR 29**	AAGCGGACGGAGAGGCC	20	0	8	Supercontig_3.5:1051773:1051902:-	-54.00	FGSG_01120<br>FGSG_02209<br>FGSG_08907<br>FGSG_04645<br>FGSG_05410<br>FGSG_10754
**Fg-JS-milR 30**	GGAGACAAGACGGAGCCA	20	0	6	Supercontig_3.5:1802196:1802297:-	-30.20	FGSG_08658<br>FGSG_08494<br>FGSG_00226<br>FGSG_10781<br>FGSG_00820<br>FGSG_09339<br>FGSG_09544<br>FGSG_04919

milRNA	milRNA_seq	Length (nt)	JS16F expression	JS16 expression	Location	Mfe (kcal/mol)	Targets
							FGSG_11955<br>FGSG_12493<br>FGSG_09427<br>FGSG_04331<br>FGSG_01117<br>FGSG_06557<br>FGSG_04552<br>FGSG_09343<br>FGSG_00579<br>FGSG_01790<br>FGSG_08352<br>FGSG_13457<br>FGSG_05095<br>FGSG_13583<br>FGSG_06774
**Fg-JS-milR 31**	AACGAAGAGGGCCGAGGGA A	23	0	10	Supercontig_3.7:1146907:1147023:-	-34.29	NA
**Fg-JS-milR 32**	CGAGAAGCGGCAGAAGA	23	0	5	Supercontig_3.8:1534421:1534545:+	-31.60	FGSG_03165<br>FGSG_02216
**Fg-JS-milR 33**	GGGGAGAGAGAGACACGG	21	0	9	Supercontig_3.8:1037582:1037674:-	-27.10	FGSG_02598<br>FGSG_10885<br>FGSG_07186<br>FGSG_00416<br>FGSG_05016

milRNA	milRNA_seq	Length (nt)	JS16F expression	JS16 expression	Location	Mfe (kcal/mol)	Targets
							FGSG_05431<br>FGSG_13171<br>FGSG_03547<br>FGSG_05276
**Fg-JS-milR 34**	GACGGAACCAAGGGGAGCA	22	0	5	Supercontig_3.8:1128961:1129034:-	-32.20	FGSG_09197<br>FGSG_04188<br>FGSG_09916<br>FGSG_13903
**Fg-JS-milR 35**	CGACACGCAGAACCAAGCC	23	0	6	Supercontig_3.1:7355730:7355831:+	-41.80	FGSG_02281
**Fg-JS-milR 36**	GACGAGGGAGCAGGGA	22	0	17	Supercontig_3.1:2223170:2223434:-	-60.20	FGSG_01186<br>FGSG_11240<br>FGSG_04611
**Fg-JS-milR 37**	AAGAAAGGAGCAACCAGGA A	23	0	22	Supercontig_3.1:5482952:5483055:-	-57.80	NA
**Fg-JS-milR 38**	GGAGGAGGGGAGGAGGAGG A	22	0	8	Supercontig_3.1:8122895:8123225:-	-93.80	FGSG_12931<br>FGSG_02542<br>FGSG_12766<br>FGSG_09410<br>FGSG_01858<br>FGSG_06205<br>FGSG_12901<br>FGSG_07482<br>FGSG_01335

milRNA	milRNA_seq	Length (nt)	JS16F expression	JS16 expression	Location	Mfe (kcal/mol)	Targets
							FGSG_12974
							FGSG_00562
							FGSG_08321
							FGSG_10017
							FGSG_04431
							FGSG_04278
							FGSG_08290
							FGSG_05373
							FGSG_02536
							FGSG_01867
							FGSG_01763
							FGSG_05556
							FGSG_06238
							FGSG_08301
							FGSG_05453
							FGSG_08775
							FGSG_11465
							FGSG_02704
							FGSG_10088
							FGSG_05205
							FGSG_04366
							FGSG_10112
							FGSG_08656
							FGSG_06944

milRNA	milRNA_seq	Length (nt)	JS16F expression	JS16 expression	Location	Mfe (kcal/mol)	Targets
							FGSG_10835
							FGSG_05329
							FGSG_08432
							FGSG_00552
							FGSG_12982
							FGSG_05891
							FGSG_11578
							FGSG_10391
							FGSG_01332
							FGSG_09330
							FGSG_09016
							FGSG_01950
							FGSG_06550
							FGSG_05042
							FGSG_06052
							FGSG_08560
							FGSG_04494
							FGSG_01893
							FGSG_05751
							FGSG_09782
							FGSG_04574
							FGSG_01316
							FGSG_00374
							FGSG_00996

milRNA	milRNA_seq	Length (nt)	JS16F expression	JS16 expression	Location	Mfe (kcal/mol)	Targets
							FGSG_03687
							FGSG_09746
							FGSG_05659
							FGSG_06015
							FGSG_06945
							FGSG_13622
							FGSG_00188
							FGSG_05558
							FGSG_06678
							FGSG_05591
							FGSG_07278
							FGSG_10686
							FGSG_04203
							FGSG_04172
							FGSG_01434
							FGSG_00442
							FGSG_04080
							FGSG_08908
							FGSG_03744
							FGSG_00803
							FGSG_13904
							FGSG_01070
							FGSG_05216
							FGSG_09455

milRNA	milRNA_seq	Length (nt)	JS16F expression	JS16 expression	Location	Mfe (kcal/mol)	Targets
**Fg-JS-milR 39**	GGAGAAGAGGAGAAAG	23	0	15	Supercontig_3.2:982840:983174:+	-87.90	FGSG_02817<br>FGSG_04527
**Fg-JS-milR 40**	GCGAAGGGGAAGAAGGGC	21	0	9	Supercontig_3.2:2012140:2012350:+	-59.70	FGSG_02374<br>FGSG_11998<br>FGSG_07279<br>FGSG_01194<br>FGSG_07704<br>FGSG_06871<br>FGSG_03980<br>FGSG_03964
**Fg-JS-milR 41**	GAGCGGGCGAGAGACGCG	22	0	5	Supercontig_3.2:604068:604323:-	-88.90	FGSG_08904<br>FGSG_08758
**Fg-JS-milR 42**	CGGAGAACGGACGGCGC	20	0	5	Supercontig_3.3:3563155:3563241:+	-24.70	FGSG_08309<br>FGSG_09986<br>FGSG_05852<br>FGSG_08650<br>FGSG_07383<br>FGSG_10449<br>FGSG_06915<br>FGSG_13137
**Fg-JS-milR 43**	GGGAAGAAGAAGACGG	22	0	5	Supercontig_3.3:545536:545656:-	-25.00	FGSG_08061<br>FGSG_11830
**Fg-JS-milR 44**	CGAGGAGCAGAACAACGGG	23	0	7	Supercontig_3.5:311968:312045:-	-38.00	FGSG_08875<br>FGSG_00610

milRNA	milRNA_seq	Length (nt)	JS16F expression	JS16 expression	Location	Mfe (kcal/mol)	Targets
							FGSG_04184
**Fg-JS-milR 45**	GGCGGACGGGCACAGAG	22	0	6	Supercontig_3.5:2015780:2015887:-	-38.40	NA
**Fg-JS-milR 46**	CACGCAACCGGGGAGAA	22	0	10	Supercontig_3.6:2968516:2968676:-	-67.21	NA
**Fg-JS-milR 47**	GCCCCCAGGAGCCGGAC	22	0	5	Supercontig_3.7:945744:945815:+	-22.20	FGSG_10132
**Fg-JS-milR 48**	AGCGACGAAGCGACGG	23	0	279	Supercontig_3.7:1759107:1759278:+	-80.00	NA
**Fg-JS-milR 49**	GCGAGACGCGAAGAGGCG	21	0	23	Supercontig_3.7:1801246:1801325:+	-30.70	FGSG_09960<br>FGSG_11367<br>FGSG_00230<br>FGSG_01854<br>FGSG_13012<br>FGSG_08249
**Fg-JS-milR 50**	CCCGCGAACCAACGAGA	20	5	0	Supercontig_3.1:6118448:6118546:-	-48.90	FGSG_05548<br>FGSG_06437<br>FGSG_10232<br>FGSG_13676<br>FGSG_07012<br>FGSG_04842<br>FGSG_07867<br>FGSG_12745<br>FGSG_08719<br>FGSG_03963

milRNA	milRNA_seq	Length (nt)	JS16F expression	JS16 expression	Location	Mfe (kcal/mol)	Targets
							FGSG_11963<br>FGSG_02052<br>FGSG_05091
**Fg-JS-milR 51**	CCGACAACAACGAGAC	23	26	0	Supercontig_3.2:2763531:2763740:-	-51.10	FGSG_01371
**Fg-JS-milR 52**	AAGGGGGGGGAGAAGA	22	55	0	Supercontig_3.3:528354:528669:+	-118.70	FGSG_06952<br>FGSG_09801<br>FGSG_01850<br>FGSG_06282
**Fg-JS-milR 53**	GAGACAGAGGAAGA	21	7	0	Supercontig_3.3:724159:724398:-	-53.70	FGSG_03415<br>FGSG_00617<br>FGSG_06833<br>FGSG_12579
**Fg-JS-milR 54**	CCGGACAGGCGAGAA	20	5	0	Supercontig_3.5:3256872:3256953:+	-25.80	FGSG_13245<br>FGSG_11593<br>FGSG_03757<br>FGSG_10048<br>FGSG_05953<br>FGSG_08923<br>FGSG_10399<br>FGSG_02551<br>FGSG_12479<br>FGSG_03866<br>FGSG_09238

milRNA	milRNA_seq	Length (nt)	JS16F expression	JS16 expression	Location	Mfe (kcal/mol)	Targets
							FGSG_01199
							FGSG_03805
							FGSG_13581
							FGSG_09886
**Fg-JS-milR 55**	GCAGGACAGAGACGACGA	22	8	0	Supercontig_3.5:163798:163989:-	-56.60	FGSG_01162
							FGSG_01899
							FGSG_13172
							FGSG_00592
							FGSG_07035
							FGSG_05925
							FGSG_02823
							FGSG_09419
							FGSG_12275
							FGSG_03756
**Fg-JS-milR 56**	GAGGGGGGGAAGAGG	20	243	0	Supercontig_3.8:1361188:1361257:-	-33.10	FGSG_06687
							FGSG_05276
							FGSG_11479
							FGSG_04378
							FGSG_06506
							FGSG_05021
							FGSG_00504
							FGSG_10925
							FGSG_04226
							FGSG_10882

milRNA	milRNA_seq	Length (nt)	JS16F expression	JS16 expression	Location	Mfe (kcal/mol)	Targets
							FGSG_06799<br>FGSG_13740<br>FGSG_06035<br>FGSG_09236<br>FGSG_11882
**Fg-JS-milR 57**	GGACACAGACAGACAGGACA	23	5	0	Supercontig_3.1:2768198:2768275:+	-21.80	NA
**Fg-JS-milR 58**	GCGAAGAGAGGGGG	21	7	0	Supercontig_3.1:4790505:4790871:+	-122.50	FGSG_00765<br>FGSG_01268<br>FGSG_00564<br>FGSG_06054
**Fg-JS-milR 59**	AGCGACACACCAGAACGAGC	23	15	0	Supercontig_3.1:3284400:3284744:-	-102.40	NA
**Fg-JS-milR 60**	GAAGCGACGGGCAA	22	11	0	Supercontig_3.2:1926977:1927150:+	-96.20	FGSG_10991
**Fg-JS-milR 61**	GGGACGGGACGGGACAGGGA	22	7	0	Supercontig_3.4:4768080:4768432:-	-88.20	FGSG_07312<br>FGSG_10402<br>FGSG_13639<br>FGSG_01915<br>FGSG_04155<br>FGSG_02643<br>FGSG_03747<br>FGSG_13634
**Fg-JS-milR 62**	GCCGACGGGCGAACAC	23	9	0	Supercontig_3.7:384048:384175:+	-36.70	FGSG_09980

milRNA	milRNA_seq	Length (nt)	JS16F expression	JS16 expression	Location	Mfe (kcal/mol)	Targets
**Fg-JS-milR 63**	AACGGGACCGGACGCGGC	23	10	0	Supercontig_3.7:1446382:1446449:+	-25.10	NA
**Fg-JS-milR 64**	GGGAGGCAAAGGAAAGA	22	25	12	CM000576:6528158:6528420:+	-119.31	FGSG_05332

Table S3. Potential target transcripts of FgHV1-derived siRNAs.

FgHV1-derived siRNAs	vsiRNA-seq	vsiRNA-length	vsiRNA-num	geneID	KEGG Orthology
siRNA-t0000278	GCAGAGCGTGTAGGGCTGTCCCA	23	2591	FgHV1gp2	-
siRNA-t0000336	GCAGAGCGTGTAGGGCTGTCCC	22	2175	FgHV1gp2	-
siRNA-t0000349	GGCTGGACTGGAGGCGCGTTTT	22	2101	FGSG_13084	K09650|1|2e-06|53.9|nfi:NFIA_016490|rhomboid-like protein [EC:3.4.21.105]
siRNA-t0000361	GGTGCGAGCAGGATTGAAGTGT	22	2063	FgHV1gp2	-
siRNA-t0000521	AGAATGAGGGAAGAAAGGGAACA	23	1494	FGSG_01483	-
siRNA-t0000547	CGAAGGAGGCGGAACGTGGGA	21	1432	FGSG_12896	K09184|1|5e-37|156|uma:UM04252.1|GATA-binding protein, other eukaryote
siRNA-t0000552	TCTCGTGGGATTGGGCGTGTGTT	23	1414	FgHV1gp2	-
siRNA-t0000611	GAAGAATGAGGGAAGAAAGGGAACA	25	1279	FGSG_01483	-
siRNA-t0000631	GAGCGAGTGGAGCGTCGGCA	20	1244	FGSG_04319	K15283|1|0.0|1065|fgr:FG04319.1|solute carrier family 35, member E1
siRNA-t0000644	GGCCCTGGGATTGTATGGTA	20	1220	FgHV1gp2	-
siRNA-t0000689	TGCTGCAAGATGGACTGGCCT	21	1162	FgHV1gp2	-
siRNA-t0000706	GGCTGGACTGGAGGCGCGTTT	21	1142	FGSG_13084	K09650|1|2e-06|53.9|nfi:NFIA_016490|rhomboid-like protein [EC:3.4.21.105]
siRNA-t0000712	CAGGACGATAGTGGGAGCGAA	21	1129	FGSG_13051	-
siRNA-t0000759	TGGACGGGTGTGAAGCTTGGA	21	1063	FGSG_00840	K00624|1|0.0|1728|fgr:FG00840.1|carnitine O-acetyltransferase [EC:2.3.1.7]
siRNA-t0000765	GGGACTATACATGATGGCTGA	21	1057	FGSG_00552	-
siRNA-t0000845	GCCCTGGGATTGTATGGTAATTGC	24	970	FgHV1gp2	-
siRNA-t0000877	GTTTCTCGTGGGATTGGGCGTGT	23	937	FgHV1gp2	-
siRNA-t0000913	GCTGTTCTCTGTCGGCATGTT	21	910	FGSG_11657	K09184|1|5e-13|75.9|bfu:BC1G_09852|GATA-binding protein, other eukaryote
siRNA-t0000918	CAGTGTCGAACCATACTGGA	20	906	FGSG_07164	K02328|1|0.0|980|fgr:FG07164.1|DNA polymerase delta subunit 2
siRNA-t0001057	AGCTGTTCTCTGTCGGCATGT	21	800	FgHV1gp2	-
siRNA-t0001058	GGCGACACCGACGGTACGTGGA	22	799	FgHV1gp2	-
siRNA-t0001085	GGGCCCAGTCATGAATTTTCGGT	23	782	FgHV1gp2	-
siRNA-t0001091	AGGCTGGACTGGAGGCGCGTT	21	778	FGSG_00786	K00924|1|0.0|755|mgr:MGG_08547|[EC:2.7.1.-];K08286|4|2e-96|352|kla:KLLA0F12188g|protein-serine/threonine kinase [EC:2.7.11.-]
siRNA-t0001093	GCGTGAGTGCGTAGATAAAGGTT	23	777	FgHV1gp2	-
siRNA-t0001109	AAATGATTTGCGACGTGCTTG	21	760	FgHV1gp2	-
siRNA-t0001144	GGGACTATACATGATGGCTG	20	742	FGSG_00552	-
siRNA-t0001145	GCTGTTCTCTGTCGGCATGT	20	741	FGSG_11657	K09184|1|5e-13|75.9|bfu:BC1G_09852|GATA-binding protein, other eukaryote
siRNA-t0001184	AAGGCGAAGTATGAGGGAGA	20	725	FGSG_07669	K09885|1|4e-23|108|ani:AN7168.2|aquaporin rerated protein, other eukaryote;K03293|2|1e-07|57.4|uma:UM03325.1|amino acid transporter, AAT family
siRNA-t0001200	TGCCATGGGATCGAAGGTTA	20	718	FGSG_11659	K01932|1|0.0|1431|afm:AFUA_5G12730|[EC:6.3.2.-]
siRNA-t0001416	GAAGGAGGCGGAACGTGGGA	20	631	FGSG_12896	K09184|1|5e-37|156|uma:UM04252.1|GATA-binding protein, other eukaryote
siRNA-t0001524	GGGGATGTTTGCTTGGTTTCGGGA	24	595	FgHV1gp2	-
siRNA-t0001580	GAAAGTGTCGTAGGAAAGGA	20	572	FGSG_10577	K08712|1|0.0|792|pic:PICST_57529|ATP-binding cassette, subfamily G (WHITE), member 2, SNQ2
siRNA-t0001594	TGCACGAGGGACGAGCTTGGC	21	569	FgHV1gp2	-
siRNA-t0001597	GGGTGACGTCAACAGTGAGCAA	22	567	FGSG_13887	K01824|1|4e-180|629|fgr:FG10931.1|cholestenol delta-isomerase [EC:5.3.3.5]
siRNA-t0001699	AGCTGTTCTCTGTCGGCATGTT	22	536	FgHV1gp2	-
siRNA-t0001707	TGTGCTGCAAGATGGACTGGCCT	23	535	FgHV1gp2	-
siRNA-t0001723	GTGTCTGACTGTTGCTTGATGGT	23	530	FgHV1gp2	-
siRNA-t0001729	GTAAGCAGAGCGTGTAGGGCT	21	529	FgHV1gp2	-
siRNA-t0001770	GGCCAAATCGTCATACATTGCA	22	517	FgHV1gp2	-
siRNA-t0001807	AGGCTGGACTGGAGGCGCGT	20	506	FGSG_05337	K09291|1|0.0|3327|fgr:FG05337.1|nucleoprotein TPR
siRNA-t0001823	ATGCCATGGGATCGAAGGTTA	21	501	FGSG_11659	K01932|1|0.0|1431|afm:AFUA_5G12730|[EC:6.3.2.-]
siRNA-t0001828	CTGACTGTTGCTTGATGGTT	20	500	FgHV1gp2	-
siRNA-t0001879	GCTGTTCTCTGTCGGCATGTTG	22	487	FGSG_11657	K09184|1|5e-13|75.9|bfu:BC1G_09852|GATA-binding protein, other eukaryote
siRNA-t0001933	CTGACTGTTGCTTGATGGTTGGCA	24	475	FgHV1gp2	-
siRNA-t0001935	TGCGAGCAGGATTGAAGTGTA	21	474	FgHV1gp2	-
siRNA-t0002046	TGGAACAACGTGAGCCAGAA	20	451	FgHV1gp2	-
siRNA-t0002066	CAGAACGGATGCGAACACTT	20	448	FGSG_06933	K11132|1|1e-08|62.0|sce:YDR206W|telomere elongation protein [EC:2.7.7.-]
siRNA-t0002128	AGACCGAAGAAAGACCACATCCA	23	438	FgHV1gp2	-
siRNA-t0002140	TCTGACTGTTGCTTGATGGTTG	22	436	FgHV1gp2	-
siRNA-t0002165	GCAAGCGTGACGAAGATCCCA	21	431	FGSG_07746	-
siRNA-t0002196	GGAGATCCGGTAAAGCATTGAA	22	426	FGSG_06511	-
siRNA-t0002235	GGAGGCACAAGAACATCAGGCA	22	419	FGSG_04287	K13830|1|0.0|3204|fgr:FG04287.1|pentafunctional AROM polypeptide [EC:4.2.3.4 4.2.1.10 1.1.1.25 2.7.1.71 2.5.1.19]
siRNA-t0002268	GTTTCTCGTGGGATTGGGCGT	21	415	FgHV1gp2	-
siRNA-t0002294	CAACGGTTCTTGGAAAGTTGGA	22	411	FgHV1gp2	-
siRNA-t0002312	AGGTGCGAGCAGGATTGAAGTGTA	24	409	FgHV1gp2	-
siRNA-t0002354	GGCCCTGGGATTGTATGGTAA	21	403	FgHV1gp2	-
siRNA-t0002387	TCGTGTCTGACTGTTGCTTGATGGT	25	397	FgHV1gp2	-
siRNA-t0002435	GCAGGATTGAAGTGTAATGCGTTGA	25	390	FgHV1gp2	-
siRNA-t0002436	TGGTGATCCTAAACATGTCGGT	22	390	FGSG_11659	K01932|1|0.0|1431|afm:AFUA_5G12730|[EC:6.3.2.-]
siRNA-t0002503	CGCGCGCTGTACTGAAGGTGGT	22	382	FgHV1gp2	-
siRNA-t0002516	GTGGAGCGTCGGCAAATTTGGCC	23	381	FGSG_01049	-
siRNA-t0002521	CGTGTCTGACTGTTGCTTGATGGT	24	379	FgHV1gp2	-
siRNA-t0002571	TTCTCGTGGGATTGGGCGTGT	21	372	FgHV1gp2	-
siRNA-t0002643	GGCCCTGGGATTGTATGGTAAT	22	364	FgHV1gp2	-
siRNA-t0002692	GGTCTTTCTTCGGTCTAGGT	20	357	FGSG_14002	-
siRNA-t0002851	GGCCCTGGGATTGTATGGTAATTG	24	342	FgHV1gp2	-
siRNA-t0002866	ACCAGCTGTTCTCTGTCGGCAT	22	341	FGSG_01569	-
siRNA-t0002876	TGACACTGGAGGGAACGAGA	20	340	FGSG_10577	K08712|1|0.0|792|pic:PICST_57529|ATP-binding cassette, subfamily G (WHITE), member 2, SNQ2
siRNA-t0002896	GGCGACACCGACGGTACGTGGAA	23	339	FgHV1gp2	-
siRNA-t0002918	TGGATCGGTGGCACACGCATA	21	336	FgHV1gp2	-
siRNA-t0002955	GGGGATGTTTGCTTGGTTTCGGGAC	25	333	FgHV1gp2	-
siRNA-t0002968	GAACGTCTGACTGTAGTGTGCCT	23	331	FgHV1gp2	-
siRNA-t0002991	GCCAAATCGTCATACATTGCA	21	330	FgHV1gp2	-
siRNA-t0002996	GCGTGTTGTACGGCGTGGTCT	21	329	FgHV1gp2	-
siRNA-t0003045	CCAGCTGTTCTCTGTCGGCATGT	23	323	FgHV1gp2	-
siRNA-t0003051	GCTGTTCTCTGTCGGCATGTTGC	23	323	FGSG_11657	K09184|1|5e-13|75.9|bfu:BC1G_09852|GATA-binding protein, other eukaryote
siRNA-t0003147	GTGATGATGAGATCACGTTCGGA	23	314	FGSG_01870	K14563|1|2e-142|504|fgr:FG01870.1|rRNA 2'-O-methyltransferase fibrillarin [EC:2.1.1.-]
siRNA-t0003155	ACGAAGAACATGCTACTGGA	20	313	FGSG_01442	K06867|1|1e-28|129|kla:KLLA0E17953g|
siRNA-t0003182	CGCGCTGTACTGAAGGTGGT	20	312	FgHV1gp2	-
siRNA-t0003308	AGGGACGTCTTCGATGGATGCTGCG	25	300	FgHV1gp2	-
siRNA-t0003319	AAGCAGAGCGTGTAGGGCTGTCC	23	299	FgHV1gp2	-
siRNA-t0003323	GTGAAAGTGTCGTAGGAAAGGA	22	299	FgHV1gp1	-
siRNA-t0003340	GGGGATGTTTGCTTGGTTTCGGG	23	298	FgHV1gp2	-
siRNA-t0003375	CAGGACGATAGTGGGAGCGAAA	22	296	FGSG_13051	-
siRNA-t0003370	GGTGTGGACATCGGAGCGGA	20	296	FGSG_08980	K00002|1|0.0|716|fgr:FG08980.1|alcohol dehydrogenase (NADP+) [EC:1.1.1.2]

siRNA-t0003401	GGATTGGGCGTGTGTTCTTTAT	22	294	FgHV1gp2	-
siRNA-t0003449	GGGCCCAGTCATGAATTTTCGGTA	24	291	FgHV1gp2	-
siRNA-t0003461	AGGACGATAGTGGGAGCGAA	20	290	FGSG_11843	K00135|1|0.0|959|fgr:FG00854.1|succinate-semialdehyde dehydrogenase (NADP+) [EC:1.2.1.16]
siRNA-t0003479	TGACGATGAATAGGCCTCCCA	21	289	FGSG_09707	K05658|1|4e-38|160|fgr:FG01684.1|ATP-binding cassette, subfamily B (MDR/TAP), member 1
siRNA-t0003535	GCCCTGGGATTGTATGGTAAT	21	285	FGSG_10267	K00288|1|0.0|1759|fgr:FG10267.1|methylenetetrahydrofolate dehydrogenase (NADP+) / methenyltetrahydrofolate cyclohydrolase / formyltetrahydrofolate synthetase [EC:1.5.1.5 3.5.4.9 6.3.4.3]
siRNA-t0003579	GTGGACCTGAGAGCATGAAG	20	282	FGSG_08097	K00688|1|7e-16|87.0|pno:SNOG_13460|starch phosphorylase [EC:2.4.1.1]
siRNA-t0003603	GACCAGCTGTTCTCTGTCGGCA	22	280	FgHV1gp2	-
siRNA-t0003623	CCATGAGTGGGTCGAGGATGCA	22	279	FGSG_11308	-
siRNA-t0003730	CAGTATGGTTCGACACTGGA	20	271	FGSG_05213	-
siRNA-t0003760	CGTTGAATCGATCGTCATGGT	21	270	FGSG_05002	K12818|1|0.0|2187|fgr:FG05002.1|ATP-dependent RNA helicase DHX8/PRP22 [EC:3.6.4.13]
siRNA-t0003794	ACACGCAGCAGAATTCTAGCCT	22	268	FgHV1gp2	-
siRNA-t0003815	GGTGGATGGTACGTGGCTCA	20	267	FGSG_01049	-
siRNA-t0003830	CGTGAGTGCGTAGATAAAGGTT	22	266	FgHV1gp2	-
siRNA-t0003827	GCACGAGGGACGAGCTTGGC	20	266	FgHV1gp2	-
siRNA-t0003892	CACGCAGGACGATAGTGGGA	20	262	FGSG_07294	K06176|1|0.0|1486|fgr:FG07294.1|tRNA pseudouridine13 synthase [EC:5.4.99.27]
siRNA-t0003914	GAGTGCTGAATGCTCTGCCTCGGT	24	261	FgHV1gp2	-
siRNA-t0003941	CTCGTGGGATTGGGCGTGTGT	21	260	FgHV1gp2	-
siRNA-t0003994	TCTCGTGGGATTGGGCGTGTG	21	257	FgHV1gp2	-
siRNA-t0004011	TGGACGCCTATAAGGAAGCTG	21	256	FGSG_05387	K03255|1|0.0|2231|fgr:FG05387.1|protein TIF31
siRNA-t0004065	GAGTGCCATGACGTACGACC	20	253	FgHV1gp1	-
siRNA-t0004080	CAAAATGCCATGGGATCGAAGGT	23	252	FGSG_09064	-
siRNA-t0004133	GGACAAGCCGTCAAACATAGACA	23	249	FgHV1gp2	-
siRNA-t0004161	GGCGTGAGTGCGTAGATAAAGGTT	24	248	FgHV1gp2	-
siRNA-t0004173	AGGCCCTGGGATTGTATGGTA	21	247	FgHV1gp2	-
siRNA-t0004216	TGGCAGGAGTTGGGAACGAG	20	245	FGSG_11941	K08741|1|0.0|1730|fgr:FG01367.1|DNA mismatch repair protein MSH5
siRNA-t0004236	CGGTTCTTGGAAAGTTGGAGCC	22	244	FgHV1gp2	-
siRNA-t0004251	GTGCGAGCAGGATTGAAGTGTA	22	244	FgHV1gp2	-
siRNA-t0004234	TCTGACTGTTGCTTGATGGTTGG	23	244	FgHV1gp2	-
siRNA-t0004362	GATCCTGACACTGACAAGCA	20	238	FGSG_05067	-
siRNA-t0004367	GCATGTACTGTTCGGCAACA	20	238	FGSG_10913	K14541|1|0.0|3625|fgr:FG10913.1|urea carboxylase / allophanate hydrolase [EC:6.3.4.6 3.5.1.54]
siRNA-t0004413	GCAGCAGAATTCTAGCCTGGTT	22	236	FgHV1gp2	-
siRNA-t0004433	AGAAGAATTAGCCTTTGAATGCCA	24	235	FgHV1gp2	-
siRNA-t0004430	GATCCACCCGGATGACTTGGCT	22	235	FgHV1gp2	-
siRNA-t0004437	GGTTTCGGGACGCGACAAGCA	21	235	FgHV1gp2	-
siRNA-t0004467	GGTGATCCTAAACATGTCGGT	21	233	FGSG_11659	K01932|1|0.0|1431|afm:AFUA_5G12730|[EC:6.3.2.-]
siRNA-t0004489	GACCAGCTGTTCTCTGTCGGCAT	23	232	FgHV1gp2	-
siRNA-t0004540	GAGCAGGATTGAAGTGTAATGCGT	24	229	FgHV1gp2	-
siRNA-t0004589	TGAGTGCGTAGATAAAGGTTA	21	227	FgHV1gp2	-
siRNA-t0004596	GGAAGTCCACGATTGTAACGAA	22	226	FGSG_05306	K08333|1|0.0|3035|fgr:FG05306.1|phosphoinositide-3-kinase, regulatory subunit 4, p150 [EC:2.7.11.1]
siRNA-t0004665	GGGAACATCATACCTTTGAGCT	22	224	FgHV1gp2	-
siRNA-t0004691	ATGCCATGGGATCGAAGGTT	20	222	FGSG_11659	K01932|1|0.0|1431|afm:AFUA_5G12730|[EC:6.3.2.-]
siRNA-t0004727	GCATTCAGTAGTCGGGGTCTC	21	221	FgHV1gp1	-
siRNA-t0004753	CCAAGTAAGCAGAGCGTGTAGGGCT	25	219	FgHV1gp2	-
siRNA-t0004785	GCCATGGGATCGAAGGTTAGA	21	218	FGSG_11659	K01932|1|0.0|1431|afm:AFUA_5G12730|[EC:6.3.2.-]
siRNA-t0004779	TCGTTGAATCGATCGTCATGGT	22	218	FgHV1gp2	-
siRNA-t0004799	GGTTCAAGTATCGTCACTTCT	21	217	FgHV1gp2	-
siRNA-t0004821	GTGAGTGCGTAGATAAAGGTT	21	217	FGSG_01801	-
siRNA-t0004845	CGGGACGCGACAAGCAACAA	20	216	FgHV1gp2	-
siRNA-t0004884	AATCGATCGTCATGGTGATACC	22	214	FgHV1gp2	-
siRNA-t0004893	GCTGGGGATGTTTGCTTGGT	20	214	FGSG_06971	K09272|1|0.0|856|mgr:MGG_11344|structure-specific recognition protein 1
siRNA-t0004909	GGCTTGTCGTCGGGAGAATCCA	22	213	FgHV1gp2	-
siRNA-t0004967	AAAATGCCATGGGATCGAAGGTTA	24	211	FGSG_09064	-
siRNA-t0004963	GGGATTGGGCGTGTGTTCTTTAT	23	211	FgHV1gp2	-
siRNA-t0004995	AAGGTGCGAGCAGGATTGAAGTGTA	25	210	FgHV1gp2	-
siRNA-t0004972	GGAAATCATCGGTCTCGCCA	20	210	FGSG_05013	K11786|1|9e-69|261|uma:UM01183.1|ATP-dependent helicase STH1/SNF2 [EC:3.6.4.-];K01509|4|3e-67|256|pic:PICST_30157|adenosinetriphosphatase [EC:3.6.1.3]
siRNA-t0005011	AAGTAAGCAGAGCGTGTAGGGCT	23	209	FgHV1gp2	-
siRNA-t0005005	TAAGCAGAGCGTGTAGGGCT	20	209	FgHV1gp2	-
siRNA-t0005044	AGTGCTGAATGCTCTGCCTCGGTCA	25	208	FgHV1gp2	-
siRNA-t0005073	GGGATGTTTGCTTGGTTTCGGGAC	24	207	FgHV1gp2	-
siRNA-t0005070	GTGTCGGCTGCCAAGTAAGCA	21	207	FGSG_12344	K01620|1|2e-117|421|act:ACLA_008260|threonine aldolase [EC:4.1.2.5]
siRNA-t0005121	CGGTGCTGATGATCATGACGT	21	205	FGSG_04439	K01051|1|2e-45|185|lbc:LACBIDRAFT_245379|pectinesterase [EC:3.1.1.11]
siRNA-t0005150	ACCAGCTGTTCTCTGTCGGCATGT	24	204	FgHV1gp2	-
siRNA-t0005146	AGCAGGATTGAAGTGTAATGCGT	23	204	FgHV1gp2	-
siRNA-t0005181	GAAAGTGTCGTAGGAAAGGAAAA	23	202	FgHV1gp1	-
siRNA-t0005242	GGATTGGGCGTGTGTTCTTTA	21	200	FgHV1gp2	-
siRNA-t0005280	AGCGAGTGGAGCGTCGGCAA	20	198	FGSG_12808	K15223|1|8e-119|425|fgr:FG05774.1|upstream activation factor subunit UAF30
siRNA-t0005288	CGACCGATGGAAGTGATGGCA	21	198	FGSG_08209	K01932|1|0.0|1305|afm:AFUA_5G12730|[EC:6.3.2.-]
siRNA-t0005330	GGATACGTTCTGAGAAGTCTCC	22	197	FgHV1gp2	-
siRNA-t0005376	GCAGGATTGAAGTGTAATGCGT	22	195	FgHV1gp2	-
siRNA-t0005369	TGGCTACTGGACGAAGACTTG	21	195	FGSG_00366	-
siRNA-t0005463	AAAGGTGCGAGCAGGATTGAAGTG	24	192	FgHV1gp2	-
siRNA-t0005447	GGATTAATCAATGTACCGTTCGGCA	25	192	FgHV1gp2	-
siRNA-t0005514	GGCGAGACCGATGATTTCCA	20	190	FGSG_12379	K03293|1|3e-136|484|cal:CaO19.6993|amino acid transporter, AAT family
siRNA-t0005531	GGGTCGTACGTCATGGCACTC	21	190	FGSG_10228	K03114|1|0.0|934|fgr:FG10228.1|mitosis inhibitor protein kinase SWE1 [EC:2.7.11.1]
siRNA-t0005518	TCGGGACGCGACAAGCAACAATT	23	190	FgHV1gp2	-
siRNA-t0005542	GAATCGATCGTCATGGTGATACCCA	25	189	FgHV1gp2	-
siRNA-t0005548	GCAGCAGAATTCTAGCCTGGT	21	189	FgHV1gp2	-
siRNA-t0005589	CGGTTCTTGGAAAGTTGGAGCCCTT	25	188	FgHV1gp2	-
siRNA-t0005591	GCAGGATTGAAGTGTAATGC	20	188	FGSG_05135	K08860|1|0.0|3130|fgr:FG05135.1|eukaryotic translation initiation factor 2-alpha kinase [EC:2.7.11.1]
siRNA-t0005678	CAACGGTTCTTGGAAAGTTGG	21	185	FgHV1gp2	-
siRNA-t0005691	GAATCGATCGTCATGGTGAT	20	185	FGSG_05519	K04728|1|0.0|5526|fgr:FG05519.1|ataxia telangiectasia mutated family protein [EC:2.7.11.1]
siRNA-t0005697	AGGCCCTGGGATTGTATGGTAA	22	184	FgHV1gp2	-
siRNA-t0005706	GCGTGTTGTACGGCGTGGTC	20	184	FgHV1gp2	-
siRNA-t0005700	GGATTGGGCGTGTGTTCTTT	20	184	FgHV1gp2	-
siRNA-t0005771	CGTTGAATCGATCGTCATGGTGA	23	182	FgHV1gp2	-
siRNA-t0005804	ACATTGCACGAGGGACGAGCTT	22	181	FgHV1gp2	-
siRNA-t0005835	GAATCGATCGTCATGGTGATACC	23	181	FgHV1gp2	-
siRNA-t0005864	GCGTGAGTGCGTAGATAAAGGTTA	24	180	FgHV1gp2	-
siRNA-t0005879	CCCGGCAGCGTTTATGGAACA	21	179	FGSG_12361	-
siRNA-t0005885	TGCAGCGGCAAATACAGGACC	21	179	FgHV1gp2	-
siRNA-t0005914	CCTGGTGGTTGGCGGCATTGT	21	178	FGSG_08786	K12182|1|4e-06|54.7|clu:CLUG_00966|hepatocyte growth factor-regulated tyrosine kinase substrate
siRNA-t0005912	GATGTGGTGAGGTATGACGTGTTCA	25	178	FgHV1gp2	-

<table>
<tr><td>siRNA-t0005948</td><td>CAGGCCCTGGGATTGTATGGT</td><td>21</td><td>177</td><td>FgHV1gp2</td><td>-</td></tr>
<tr><td>siRNA-t0005962</td><td>TGTGGTGAGGTATGACGTGTTCA</td><td>23</td><td>177</td><td>FgHV1gp2</td><td>-</td></tr>
<tr><td>siRNA-t0006162</td><td>GCGAGGGGCATTGCTGGCGAG</td><td>21</td><td>172</td><td>FGSG_02080</td><td>K13095|1|0.0|3053|fgr:FG02080.1|splicing factor 1</td></tr>
<tr><td>siRNA-t0006196</td><td>GAGCGAGTGGAGCGTCGGCAA</td><td>21</td><td>171</td><td>FGSG_04319</td><td>K15283|1|0.0|1065|fgr:FG04319.1|solute carrier family 35, member E1</td></tr>
<tr><td>siRNA-t0006179</td><td>TGAAAGTGTCGTAGGAAAGGAA</td><td>22</td><td>171</td><td>FgHV1gp1</td><td>-</td></tr>
<tr><td>siRNA-t0006202</td><td>ATAGATGGTGATTGTGGTCATTTG</td><td>24</td><td>170</td><td>FgHV1gp2</td><td>-</td></tr>
<tr><td>siRNA-t0006205</td><td>GGCGTGCTGAGATCAAGTAT</td><td>20</td><td>170</td><td>FGSG_10790</td><td>K15389|1|3e-101|368|ure:UREG_05875|acyl-CoA dehydrogenase;K00257|2|1e-100|366|afm:AFUA_6G10880|[EC:1.3.99.-]</td></tr>
<tr><td>siRNA-t0006300</td><td>GGTGATTGTGGTCATTTGGT</td><td>20</td><td>168</td><td>FGSG_13582</td><td>-</td></tr>
<tr><td>siRNA-t0006352</td><td>AAAGGTGCGAGCAGGATTGAA</td><td>21</td><td>167</td><td>FgHV1gp2</td><td>-</td></tr>
<tr><td>siRNA-t0006341</td><td>GAGTGGGATCTTCGTCACGC</td><td>20</td><td>167</td><td>FGSG_05729</td><td>-</td></tr>
<tr><td>siRNA-t0006378</td><td>GAGAACGCTGAAATCGAGGGTGTTG</td><td>25</td><td>166</td><td>FgHV1gp2</td><td>-</td></tr>
<tr><td>siRNA-t0006419</td><td>AAGAGCGAGTGGAGCGTCGGC</td><td>21</td><td>165</td><td>FGSG_01621</td><td>K01179|1|0.0|662|fgr:FG01621.1|endoglucanase [EC:3.2.1.4]</td></tr>
<tr><td>siRNA-t0006520</td><td>GGAGATGATGTTGGTTAGAA</td><td>20</td><td>163</td><td>FGSG_08118</td><td>-</td></tr>
<tr><td>siRNA-t0006552</td><td>CGTGTCTGACTGTTGCTTGATGGTT</td><td>25</td><td>162</td><td>FgHV1gp2</td><td>-</td></tr>
<tr><td>siRNA-t0006568</td><td>CCCTGGGATTGTATGGTAATT</td><td>21</td><td>161</td><td>FgHV1gp2</td><td>-</td></tr>
<tr><td>siRNA-t0006579</td><td>GGAGTTGGGAACGAGGCATC</td><td>20</td><td>161</td><td>FGSG_09910</td><td>-</td></tr>
<tr><td>siRNA-t0006697</td><td>AAAATGCCATGGGATCGAAGGT</td><td>22</td><td>158</td><td>FGSG_09064</td><td>-</td></tr>
<tr><td>siRNA-t0006747</td><td>GCACGAGGGACGAGCTTGGCC</td><td>21</td><td>157</td><td>FgHV1gp2</td><td>-</td></tr>
<tr><td>siRNA-t0006801</td><td>AAGAACGTAGATGTGATGCCTTG</td><td>23</td><td>156</td><td>FgHV1gp2</td><td>-</td></tr>
<tr><td>siRNA-t0006764</td><td>CGGGACCGACGTTTTGGCGA</td><td>20</td><td>156</td><td>FgHV1gp2</td><td>-</td></tr>
<tr><td>siRNA-t0006852</td><td>GATCAATGTTGGGCATCGGCT</td><td>21</td><td>155</td><td>FGSG_05974</td><td>-</td></tr>
<tr><td>siRNA-t0006830</td><td>GATGGATGCTGCGAAACCCAA</td><td>21</td><td>155</td><td>FgHV1gp2</td><td>-</td></tr>
<tr><td>siRNA-t0006850</td><td>GCGCGCTGTACTGAAGGTGGTGTCA</td><td>25</td><td>155</td><td>FgHV1gp2</td><td>-</td></tr>
<tr><td>siRNA-t0006869</td><td>GGGATGTTTGCTTGGTTTCGGGA</td><td>23</td><td>154</td><td>FgHV1gp2</td><td>-</td></tr>
<tr><td>siRNA-t0006896</td><td>GGTTGGCGGCATTGTATGGT</td><td>20</td><td>154</td><td>FGSG_12985</td><td>K15192|1|0.0|3430|nhe:NECHADRAFT_92843|TATA-binding protein-associated factor [EC:3.6.4.-]</td></tr>
<tr><td>siRNA-t0006983</td><td>ATGAGGGAAGAAAGGGAACA</td><td>20</td><td>152</td><td>FGSG_11569</td><td>-</td></tr>
<tr><td>siRNA-t0006976</td><td>CAGCTGTTCTCTGTCGGCATGT</td><td>22</td><td>152</td><td>FgHV1gp2</td><td>-</td></tr>
<tr><td>siRNA-t0006945</td><td>GAAGAACGTAGATGTGATGCC</td><td>21</td><td>152</td><td>FGSG_13130</td><td>K01505|1|0.0|665|ani:AN8210.2|1-aminocyclopropane-1-carboxylate deaminase [EC:3.5.99.7]</td></tr>
<tr><td>siRNA-t0006996</td><td>GCGCGCTGTACTGAAGGTGGTGTC</td><td>24</td><td>152</td><td>FgHV1gp2</td><td>-</td></tr>
<tr><td>siRNA-t0006940</td><td>TCCAATGTAAGTGACGAGGCCTC</td><td>23</td><td>152</td><td>FgHV1gp2</td><td>-</td></tr>
<tr><td>siRNA-t0006963</td><td>TGCCATGGGATCGAAGGTTAG</td><td>21</td><td>152</td><td>FGSG_11659</td><td>K01932|1|0.0|1431|afm:AFUA_5G12730|[EC:6.3.2.-]</td></tr>
<tr><td>siRNA-t0007049</td><td>ACGCAGCAGAATTCTAGCCTGGTT</td><td>24</td><td>150</td><td>FgHV1gp2</td><td>-</td></tr>
<tr><td>siRNA-t0007048</td><td>AGGGAACAATGAAGGCGTGT</td><td>20</td><td>150</td><td>FgHV1gp2</td><td>-</td></tr>
<tr><td>siRNA-t0007088</td><td>GGCGTGCTGAGATCAAGTATA</td><td>21</td><td>150</td><td>FGSG_10790</td><td>K15389|1|3e-101|368|ure:UREG_05875|acyl-CoA dehydrogenase;K00257|2|1e-100|366|afm:AFUA_6G10880|[EC:1.3.99.-]</td></tr>
<tr><td>siRNA-t0007102</td><td>CATCGGGAATCATGTCGGTG</td><td>20</td><td>149</td><td>FGSG_12126</td><td>K15418|1|0.0|4445|fgr:FG02396.1|zearalenone synthase, highly reducing iterative type I polyketide synthase;K15419|3|3e-172|606|fgr:FG08208.1|emericellamide biosynthesis, highly reducing iterative type I polyketide synthase</td></tr>
<tr><td>siRNA-t0007111</td><td>GATGTGGTGAGGTATGACGT</td><td>20</td><td>149</td><td>FGSG_06181</td><td>-</td></tr>
<tr><td>siRNA-t0007172</td><td>GTGACCAGCTGTTCTCTGTCGGCA</td><td>24</td><td>148</td><td>FgHV1gp2</td><td>-</td></tr>
<tr><td>siRNA-t0007181</td><td>CCAGCTGTTCTCTGTCGGCAT</td><td>21</td><td>147</td><td>FgHV1gp2</td><td>-</td></tr>
<tr><td>siRNA-t0007186</td><td>GCTGATGATCATGACGTGGA</td><td>20</td><td>147</td><td>FGSG_11121</td><td>-</td></tr>
<tr><td>siRNA-t0007226</td><td>ACGGGACCGACGTTTTGGCGA</td><td>21</td><td>146</td><td>FgHV1gp2</td><td>-</td></tr>
<tr><td>siRNA-t0007292</td><td>GGCGGGACCAGGATTCTTCTCCAC</td><td>24</td><td>145</td><td>FGSG_08844</td><td>K01183|1|1e-06|55.1|pan:PODANSg4492|chitinase [EC:3.2.1.14]</td></tr>
<tr><td>siRNA-t0007343</td><td>AGAGTCTTGCGGTGTGGGTCT</td><td>21</td><td>144</td><td>FgHV1gp2</td><td>-</td></tr>
<tr><td>siRNA-t0007387</td><td>AGAACCCATATGGATCGGTGGCACA</td><td>25</td><td>143</td><td>FgHV1gp2</td><td>-</td></tr>
<tr><td>siRNA-t0007398</td><td>GCGACACCGACGGTACGTGGAA</td><td>22</td><td>143</td><td>FgHV1gp2</td><td>-</td></tr>
<tr><td>siRNA-t0007396</td><td>GGAGGCACAAGAACATCAGGC</td><td>21</td><td>143</td><td>FGSG_04287</td><td>K13830|1|0.0|3204|fgr:FG04287.1|pentafunctional AROM polypeptide [EC:4.2.3.4 4.2.1.10 1.1.1.25 2.7.1.71 2.5.1.19]</td></tr>
<tr><td>siRNA-t0007374</td><td>TCTCAAGGATTTGTAAGGGGA</td><td>21</td><td>143</td><td>FgHV1gp2</td><td>-</td></tr>
<tr><td>siRNA-t0007427</td><td>GCGTACCTGCAGTCACAACTT</td><td>21</td><td>142</td><td>FGSG_01790</td><td>K00680|1|0.0|2387|afm:AFUA_3G14700|[EC:2.3.1.-];K15419|2|0.0|1077|ani:AN2547.2|emericellamide biosynthesis, highly reducing iterative type I polyketide synthase;K15384|5|0.0|762|afv:AFLA_066840|aspyridone synthetase, hybrid polyketide synthase / nonribosomal peptide synthetase</td></tr>
<tr><td>siRNA-t0007505</td><td>AACACGCAGCAGAATTCTAGCCT</td><td>23</td><td>141</td><td>FgHV1gp2</td><td>-</td></tr>
<tr><td>siRNA-t0007511</td><td>CTGACTGTTGCTTGATGGTTGG</td><td>22</td><td>141</td><td>FgHV1gp2</td><td>-</td></tr>
<tr><td>siRNA-t0007490</td><td>TTGTGACTGCAGGTACGCAA</td><td>20</td><td>141</td><td>FGSG_00016</td><td>-</td></tr>
<tr><td>siRNA-t0007581</td><td>GGTGACGTCAACAGTGAGCA</td><td>20</td><td>140</td><td>FGSG_05990</td><td>K02486|1|4e-52|207|spo:SPAC27E2.09|two-component system, unclassified family, sensor kinase [EC:2.7.13.3];K00936|3|5e-34|147|afm:AFUA_6G10240|[EC:2.7.3.-]</td></tr>
<tr><td>siRNA-t0007642</td><td>CCCTGGGATTGTATGGTAATTGC</td><td>23</td><td>139</td><td>FgHV1gp2</td><td>-</td></tr>
<tr><td>siRNA-t0007605</td><td>TCCCGGCAGCGTTTATGGAACA</td><td>22</td><td>139</td><td>FGSG_12361</td><td>-</td></tr>
<tr><td>siRNA-t0007665</td><td>AGGAGTGGCAGTGTAATGGA</td><td>20</td><td>138</td><td>FgHV1gp2</td><td>-</td></tr>
<tr><td>siRNA-t0007695</td><td>CCAAACTGTTCATTTCGGCG</td><td>20</td><td>138</td><td>FGSG_04004</td><td>K15196|1|0.0|1224|fgr:FG04004.1|transcription factor IIIB 90 kDa subunit</td></tr>
<tr><td>siRNA-t0007693</td><td>CGGTAGTCATCGGGAATCATGT</td><td>22</td><td>138</td><td>FgHV1gp2</td><td>-</td></tr>
<tr><td>siRNA-t0007699</td><td>GCCATGACGTACGACCCCGAA</td><td>21</td><td>138</td><td>FgHV1gp1</td><td>-</td></tr>
<tr><td>siRNA-t0007760</td><td>GGCCAAATCGTCATACATTGCAC</td><td>23</td><td>137</td><td>FgHV1gp2</td><td>-</td></tr>
<tr><td>siRNA-t0007784</td><td>GGATACGTTCTGAGAAGTCTCCGA</td><td>24</td><td>136</td><td>FgHV1gp2</td><td>-</td></tr>
<tr><td>siRNA-t0007819</td><td>GGATGTAGGTCTGGCATAGGAA</td><td>22</td><td>136</td><td>FGSG_00368</td><td>-</td></tr>
<tr><td>siRNA-t0007838</td><td>GCTGGACTGGAGGCGCGTTT</td><td>20</td><td>135</td><td>FGSG_13084</td><td>K09650|1|2e-06|53.9|nfi:NFIA_016490|rhomboid-like protein [EC:3.4.21.105]</td></tr>
<tr><td>siRNA-t0007942</td><td>AGGGACGTCTTCGATGGATGCTG</td><td>23</td><td>134</td><td>FgHV1gp2</td><td>-</td></tr>
<tr><td>siRNA-t0007909</td><td>GAACGTCTGACTGTAGTGTGCCTT</td><td>24</td><td>134</td><td>FgHV1gp2</td><td>-</td></tr>
<tr><td>siRNA-t0007935</td><td>TGTGGTCTTTCTTCGGTCTA</td><td>20</td><td>134</td><td>FGSG_14002</td><td>-</td></tr>
<tr><td>siRNA-t0007973</td><td>GTGTCTGACTGTTGCTTGATGGTT</td><td>24</td><td>133</td><td>FgHV1gp2</td><td>-</td></tr>
<tr><td>siRNA-t0008036</td><td>TCGTGAACTGCTGCGCTATGGTGTG</td><td>25</td><td>132</td><td>FgHV1gp2</td><td>-</td></tr>
<tr><td>siRNA-t0008070</td><td>TGGCGCATACACGGAACCCA</td><td>20</td><td>132</td><td>FGSG_11027</td><td>-</td></tr>
<tr><td>siRNA-t0008097</td><td>AAAGGTGCGAGCAGGATTGAAGTGT</td><td>25</td><td>131</td><td>FgHV1gp2</td><td>-</td></tr>
<tr><td>siRNA-t0008084</td><td>GCATGGGTCGCAGTTGGAATAT</td><td>22</td><td>131</td><td>FGSG_08364</td><td>-</td></tr>
<tr><td>siRNA-t0008089</td><td>TGAGAACGCTGAAATCGAGGGT</td><td>22</td><td>131</td><td>FgHV1gp2</td><td>-</td></tr>
<tr><td>siRNA-t0008162</td><td>AGCGTGTTGTACGGCGTGGTCT</td><td>22</td><td>130</td><td>FgHV1gp2</td><td>-</td></tr>
<tr><td>siRNA-t0008170</td><td>CAGTGTCGAACCATACTGGACCCA</td><td>24</td><td>130</td><td>FgHV1gp2</td><td>-</td></tr>
<tr><td>siRNA-t0008148</td><td>GGATTGGGCGTGTGTTCTTTATG</td><td>23</td><td>130</td><td>FgHV1gp2</td><td>-</td></tr>
<tr><td>siRNA-t0008160</td><td>TGAAATGATTTGCGACGTGCT</td><td>21</td><td>130</td><td>FgHV1gp2</td><td>-</td></tr>
<tr><td>siRNA-t0008164</td><td>TGGACCCAGATATGACCCAT</td><td>20</td><td>130</td><td>FGSG_06631</td><td>-</td></tr>
<tr><td>siRNA-t0008235</td><td>GGGTCTTGACTTGGGCTGTACC</td><td>22</td><td>129</td><td>FGSG_09304</td><td>-</td></tr>
<tr><td>siRNA-t0008371</td><td>CGTTGAATCGATCGTCATGGTG</td><td>22</td><td>127</td><td>FgHV1gp2</td><td>-</td></tr>
<tr><td>siRNA-t0008342</td><td>GAGCAGGATTGAAGTGTAATGC</td><td>22</td><td>127</td><td>FgHV1gp2</td><td>-</td></tr>
<tr><td>siRNA-t0008441</td><td>AGCTGTTCTCTGTCGGCATG</td><td>20</td><td>126</td><td>FGSG_11026</td><td>K01932|1|0.0|1527|afm:AFUA_1G17200|[EC:6.3.2.-]</td></tr>
<tr><td>siRNA-t0008409</td><td>CTCACTGTTGACGTCACCCTTTG</td><td>23</td><td>126</td><td>FgHV1gp2</td><td>-</td></tr>
<tr><td>siRNA-t0008457</td><td>CTGTTCTCTGTCGGCATGTT</td><td>20</td><td>126</td><td>FGSG_11657</td><td>K09184|1|5e-13|75.9|bfu:BC1G_09852|GATA-binding protein, other eukaryote</td></tr>
<tr><td>siRNA-t0008445</td><td>GAGCAGGATCTCGTAAGCTGC</td><td>21</td><td>126</td><td>FGSG_00789</td><td>-</td></tr>
<tr><td>siRNA-t0008465</td><td>AAGAACGTAGATGTGATGCCTTGTC</td><td>25</td><td>125</td><td>FgHV1gp2</td><td>-</td></tr>
<tr><td>siRNA-t0008565</td><td>CCTGGGATTGTATGGTAATTGC</td><td>22</td><td>124</td><td>FgHV1gp2</td><td>-</td></tr>
<tr><td>siRNA-t0008581</td><td>CGTGTCTGACTGTTGCTTGATGG</td><td>23</td><td>124</td><td>FgHV1gp2</td><td>-</td></tr>
<tr><td>siRNA-t0008572</td><td>GTGCTGCAAGATGGACTGGC</td><td>20</td><td>124</td><td>FgHV1gp2</td><td>-</td></tr>
<tr><td>siRNA-t0008609</td><td>AAATGATTTGCGACGTGCTT</td><td>20</td><td>123</td><td>FgHV1gp2</td><td>-</td></tr>
<tr><td>siRNA-t0008632</td><td>GGTGGTTGGCGGCATTGTAT</td><td>20</td><td>123</td><td>FGSG_01429</td><td>K14309|1|0.0|2016|fgr:FG01429.1|nuclear pore complex protein Nup93</td></tr>
<tr><td>siRNA-t0008730</td><td>CACTGTTGACGTCACCCTTTGGA</td><td>23</td><td>122</td><td>FgHV1gp2</td><td>-</td></tr>
<tr><td>siRNA-t0008691</td><td>CAGGCCCTGGGATTGTATGGTAA</td><td>23</td><td>122</td><td>FgHV1gp2</td><td>-</td></tr>
</table>

siRNA-t0008809	AATTTTTGGTCTTCGACGGGT	21	121	FGSG_13308	-
siRNA-t0008759	GAACGCATTCCGGACGGTGA	20	121	FGSG_04266	-
siRNA-t0008800	GGGGTCGTACGTCATGGCACTC	22	121	FGSG_10228	K03114|1|0.0|934|fgr:FG10228.1|mitosis inhibitor protein kinase SWE1 [EC:2.7.11.1]
siRNA-t0008855	GGGTCGTACGTCATGGCACT	20	120	FGSG_10228	K03114|1|0.0|934|fgr:FG10228.1|mitosis inhibitor protein kinase SWE1 [EC:2.7.11.1]
siRNA-t0008912	ATGGATCGGTGGCACACGCAT	21	119	FgHV1gp2	-
siRNA-t0008896	CTCACTGTTGACGTCACCCTTT	22	119	FgHV1gp2	-
siRNA-t0008951	GAGCAGGATTGAAGTGTAATGCGTT	25	119	FgHV1gp2	-
siRNA-t0008950	GGGATGTTTGCTTGGTTTCGGGACG	25	119	FgHV1gp2	-
siRNA-t0008920	TGTCTGACTGTTGCTTGATGGT	22	119	FGSG_04580	K08711|1|0.0|1709|cne:CNB05770|ATP-binding cassette, subfamily G (WHITE), member 2, PDR
siRNA-t0008976	GGGACGTCTTCGATGGATGCT	21	118	FGSG_00834	K10743|1|0.0|670|fgr:FG00834.1|ribonuclease H2 subunit A [EC:3.1.26.4]
siRNA-t0008983	TCAGGCATGTATGGGTCGTT	20	118	FGSG_05189	K00936|1|6e-75|282|afm:AFUA_8G06140|[EC:2.7.3.-];K11233|2|6e-15|83.2|ppl:POSPLDRAFT_134813|osomolarity two-component system, response regulator SSK1
siRNA-t0009082	GCTCACTGTTGACGTCACCC	20	117	FGSG_09884	K00472|1|2e-09|63.9|fgr:FG05473.1|prolyl 4-hydroxylase [EC:1.14.11.2]
siRNA-t0009189	GTTTCTCGTGGGATTGGGCGTGTG	24	116	FgHV1gp2	-
siRNA-t0009247	AAGAACGTAGATGTGATGCCT	21	115	FgHV1gp2	-
siRNA-t0009207	ACCAGCTGTTCTCTGTCGGCATGTT	25	115	FgHV1gp2	-
siRNA-t0009209	AGACCGAAGAAAGACCACAT	20	115	FGSG_09329	K08711|1|0.0|1646|cne:CNB05770|ATP-binding cassette, subfamily G (WHITE), member 2, PDR
siRNA-t0009205	AGAGCGTGTAGGGCTGTCCC	20	115	FGSG_10021	-
siRNA-t0009264	CTCACTGTTGACGTCACCCT	20	115	FGSG_07798	K15384|1|0.0|2498|afv:AFLA_066840|aspyridone synthetase, hybrid polyketide synthase / nonribosomal peptide synthetase;K15394|3|0.0|2447|cpw:CPC735_030880|hybrid polyketide synthase / nonribosomal peptide synthetase ACE1
siRNA-t0009238	GATGAACCTGGAAGAATCATA	21	115	FGSG_11026	K01932|1|0.0|1527|afm:AFUA_1G17200|[EC:6.3.2.-]
siRNA-t0009260	GTGGATCGTCCTACACTTTC	20	115	FGSG_02288	K11701|1|8e-53|209|spo:SPCC1739.03|helicase required for RNAi-mediated heterochromatin assembly 1 [EC:3.6.4.13]
siRNA-t0009311	AGGCTGGAATGGAAGAGGCC	20	114	FGSG_00362	K08286|1|8e-58|224|kla:KLLA0E21693g|protein-serine/threonine kinase [EC:2.7.11.-]
siRNA-t0009361	GACGAAGACTTGCGTACCTGC	21	114	FGSG_04439	K01051|1|2e-45|185|lbc:LACBIDRAFT_245379|pectinesterase [EC:3.1.1.11]
siRNA-t0009349	GAGTGGGTCGAGGATGCAAA	20	114	FGSG_04281	-
siRNA-t0009293	GCTGGCATAAAGGAATCCCA	20	114	FGSG_07516	K05658|1|0.0|2566|fgr:FG07516.1|ATP-binding cassette, subfamily B (MDR/TAP), member 1
siRNA-t0009305	TGATGAGATCACGTTCGGAG	20	114	FGSG_11059	-
siRNA-t0009429	AAGTGAAAGTGTCGTAGGAAAGGAA	25	113	FgHV1gp1	-
siRNA-t0009423	ACGTAGACGTCGGCACCAAC	20	113	FGSG_01589	K00777|1|3e-17|91.3|afm:AFUA_7G06290|[EC:2.4.2.-];K12823|3|3e-11|71.6|ure:UREG_02211|ATP-dependent RNA helicase DDX5/DBP2 [EC:3.6.4.13]
siRNA-t0009418	CTGGACCCAGATATGACCCA	20	113	FGSG_06631	-
siRNA-t0009376	GCAGCGGCAAATACAGGACC	20	113	FgHV1gp2	-
siRNA-t0009595	CGTGACCAGCTGTTCTCTGTCGGCA	25	111	FgHV1gp2	-
siRNA-t0009563	CGTTGAATCGATCGTCATGG	20	111	FgHV1gp2	-
siRNA-t0009557	TGGATGATATTCGGTATTGTCGA	23	111	FgHV1gp2	-
siRNA-t0009644	ACAGAACGGATGCGAACACT	20	110	FGSG_01431	-
siRNA-t0009679	GAGAACGCTGAAATCGAGGGTG	22	110	FgHV1gp2	-
siRNA-t0009695	GCAGGATTGAAGTGTAATGCGTT	23	110	FgHV1gp2	-
siRNA-t0009668	GGGAAGAAAGGGAACAGAGAA	21	110	FGSG_06101	K14780|1|0.0|2204|fgr:FG06101.1|ATP-dependent RNA helicase DHX37/DHR1 [EC:3.6.4.13]
siRNA-t0009681	GGGATTGGGCGTGTGTTCTTT	21	110	FgHV1gp2	-
siRNA-t0009744	CAGCTGTTCTCTGTCGGCAT	20	109	FgHV1gp2	-
siRNA-t0009769	CATGTAAGGATCTTTTGGCAT	21	109	FgHV1gp2	-
siRNA-t0009766	CTCACTGTTGACGTCACCCTT	21	109	FGSG_07798	K15384|1|0.0|2498|afv:AFLA_066840|aspyridone synthetase, hybrid polyketide synthase / nonribosomal peptide synthetase;K15394|3|0.0|2447|cpw:CPC735_030880|hybrid polyketide synthase / nonribosomal peptide synthetase ACE1
siRNA-t0009782	GCTGCAGCAACTGTCGCCTGGA	22	109	FgHV1gp1	-
siRNA-t0009720	GTGTCTGACTGTTGCTTGATGG	22	109	FgHV1gp2	-
siRNA-t0009804	GGCTTGTCGTCGGGAGAATCC	21	108	FgHV1gp2	-
siRNA-t0009973	GAGTGGGATGGGTTTCTGGT	20	107	FGSG_05683	K00485|1|5e-159|560|mgr:MGG_03330|dimethylaniline monooxygenase (N-oxide forming) [EC:1.14.13.8]
siRNA-t0009902	GTCCTTCGTTGAATCGATCGTC	22	107	FgHV1gp2	-
siRNA-t0009983	TAGACCGAAGAAAGACCACAT	21	107	FgHV1gp2	-
siRNA-t0009927	TGGGAGACGGCAGGTAACTCCT	22	107	FGSG_11154	-
siRNA-t0010006	GCAGGATCGTTATTCTTCTT	20	106	FGSG_05596	K13339|1|0.0|1840|fgr:FG05596.1|peroxin-6
siRNA-t0009994	GGGTCTTCTGACGAATTTGGACA	23	106	FgHV1gp2	-
siRNA-t0010067	TCCTCAAGGAGCTCAAAGGTAT	22	106	FGSG_09306	K11644|1|0.0|2881|fgr:FG09306.1|paired amphipathic helix protein Sin3a
siRNA-t0010091	GTGCTGAATGCTCTGCCTCGGT	22	105	FgHV1gp2	-
siRNA-t0010128	GTGGAAATCTTCAGGCCCTGGGA	23	105	FgHV1gp2	-
siRNA-t0010099	TCCTCAAGGAGCTCAAAGGTA	21	105	FGSG_09306	K11644|1|0.0|2881|fgr:FG09306.1|paired amphipathic helix protein Sin3a
siRNA-t0010213	GCGCATACACGGAACCCATTGAT	23	104	FgHV1gp2	-
siRNA-t0010184	GGTGTGGACATCGGAGCGGAG	21	104	FGSG_08980	K00002|1|0.0|716|fgr:FG08980.1|alcohol dehydrogenase (NADP+) [EC:1.1.1.2]
siRNA-t0010215	TCTGACGAATTTGGACAAGTA	21	104	FgHV1gp2	-
siRNA-t0010319	ACTTTCGGGGTCGTACGTCATGG	23	103	FGSG_02394	K01932|1|0.0|942|afm:AFUA_1G10380|[EC:6.3.2.-]
siRNA-t0010344	GAAAGTGTCGTAGGAAAGGAAAAGT	25	103	FgHV1gp1	-
siRNA-t0010263	GAGGAAAGGAAATATCGGAAAATTG	25	103	FgHV1gp2	-
siRNA-t0010316	GCATTCCGGACGGTGACCAT	20	103	FGSG_02632	K05349|1|0.0|1666|fgr:FG02632.1|beta-glucosidase [EC:3.2.1.21]
siRNA-t0010308	GGCGGGACCAGGATTCTTCTCCA	23	103	FGSG_08844	K01183|1|1e-06|55.1|pan:PODANSg4492|chitinase [EC:3.2.1.14]
siRNA-t0010271	TCAGGCCCTGGGATTGTATGG	21	103	FgHV1gp2	-
siRNA-t0010403	TGTTTCTCGTGGGATTGGGCGTGTG	25	102	FgHV1gp2	-
siRNA-t0010487	AGATGGACTGGCCTGTTGTCACCTT	25	101	FgHV1gp2	-
siRNA-t0010470	GCCCTGGGATTGTATGGTAA	20	101	FGSG_10267	K00288|1|0.0|1759|fgr:FG10267.1|methylenetetrahydrofolate dehydrogenase (NADP+) / methenyltetrahydrofolate cyclohydrolase / formyltetrahydrofolate synthetase [EC:1.5.1.5 3.5.4.9 6.3.4.3]
siRNA-t0010531	TCTGGCCGGTTGAAAATGTTCCA	23	101	FgHV1gp2	-
siRNA-t0010641	CAGTGTCGAACCATACTGGAC	21	100	FgHV1gp2	-
siRNA-t0010571	CCATAACCTCGTTGGGACCTT	21	100	FgHV1gp2	-
siRNA-t0010615	GCCCTGGGATTGTATGGTAATTGCG	25	100	FgHV1gp2	-
siRNA-t0010616	TCTTGGAAAGTTGGAGCCCTT	21	100	FgHV1gp2	-
siRNA-t0010638	TGTTCTCTGTCGGCATGTTG	20	100	FGSG_11657	K09184|1|5e-13|75.9|bfu:BC1G_09852|GATA-binding protein, other eukaryote
siRNA-t0010723	AGGGACGTCTTCGATGGATGCTGC	24	99	FgHV1gp2	-
siRNA-t0010689	ATTGGCTACTGGACGAAGACTTG	23	99	FGSG_00366	-
siRNA-t0010704	GGACCGACGTTTTGGCGAGACCGA	24	99	FgHV1gp2	-
siRNA-t0010679	GGGATTGGGCGTGTGTTCTTTA	22	99	FgHV1gp2	-
siRNA-t0010657	GTGGGATTGGGCGTGTGTTC	20	99	FgHV1gp2	-
siRNA-t0010806	AGTGCTGAATGCTCTGCCTCGGT	23	98	FgHV1gp2	-
siRNA-t0010865	CAAACTGTTCATTTCGGCGAG	21	98	FGSG_05794	K15419|1|7e-100|366|ani:AN2547.2|emericellamide biosynthesis, highly reducing iterative type I polyketide synthase
siRNA-t0010884	CAAGTAAGCAGAGCGTGTAGGGCT	24	98	FgHV1gp2	-
siRNA-t0010845	CGGAAAATTGTGTTGATTGCGCCAT	25	98	FgHV1gp2	-
siRNA-t0010850	CGTAGTCATCGTCAGGGTACGT	22	98	FGSG_09801	K03164|1|0.0|2817|fgr:FG09801.1|DNA topoisomerase II [EC:5.99.1.3]
siRNA-t0010881	GGACGGGTGTGAAGCTTGGA	20	98	FGSG_09780	K15436|1|0.0|1914|fgr:FG09780.1|transportin-3
siRNA-t0010836	GGGATGTTTGCTTGGTTTCGGG	22	98	FgHV1gp2	-
siRNA-t0010801	TCTGACTGTTGCTTGATGGTT	21	98	FgHV1gp2	-

<table>
<tr><td>siRNA-t0010971</td><td>AGCTGGGGATGTTTGCTTGGTTT</td><td>23</td><td>97</td><td>FgHV1gp2</td><td>-</td></tr>
<tr><td>siRNA-t0010969</td><td>CCAAACTGTTCATTTCGGCGA</td><td>21</td><td>97</td><td>FGSG_04004</td><td>K15196|1|0.0|1224|fgr:FG04004.1|transcription factor IIIB 90 kDa subunit</td></tr>
<tr><td>siRNA-t0010925</td><td>GGGATGTTTGCTTGGTTTCGG</td><td>21</td><td>97</td><td>FgHV1gp2</td><td>-</td></tr>
<tr><td>siRNA-t0010975</td><td>TCGTTGAATCGATCGTCATGGTGA</td><td>24</td><td>97</td><td>FgHV1gp2</td><td>-</td></tr>
<tr><td>siRNA-t0010995</td><td>CAGGCCCTGGGATTGTATGGTA</td><td>22</td><td>96</td><td>FgHV1gp2</td><td>-</td></tr>
<tr><td>siRNA-t0011004</td><td>GAGCTGGGGATGTTTGCTTGGT</td><td>22</td><td>96</td><td>FgHV1gp2</td><td>-</td></tr>
<tr><td>siRNA-t0011023</td><td>GAGTGCTGAATGCTCTGCCTCGGTC</td><td>25</td><td>96</td><td>FgHV1gp2</td><td>-</td></tr>
<tr><td>siRNA-t0011055</td><td>TCTGACTGTAGTGTGCCTTGT</td><td>21</td><td>96</td><td>FGSG_11659</td><td>K01932|1|0.0|1431|afm:AFUA_5G12730|[EC:6.3.2.-]</td></tr>
<tr><td>siRNA-t0011122</td><td>GATGATGAGATCACGTTCGGAG</td><td>22</td><td>95</td><td>FGSG_01870</td><td>K14563|1|2e-142|504|fgr:FG01870.1|rRNA 2'-O-methyltransferase fibrillarin [EC:2.1.1.-]</td></tr>
<tr><td>siRNA-t0011129</td><td>GCCATGGGATCGAAGGTTAG</td><td>20</td><td>95</td><td>FGSG_11659</td><td>K01932|1|0.0|1431|afm:AFUA_5G12730|[EC:6.3.2.-]</td></tr>
<tr><td>siRNA-t0011198</td><td>CAGGCCCTGGGATTGTATGG</td><td>20</td><td>94</td><td>FGSG_01287</td><td>-</td></tr>
<tr><td>siRNA-t0011214</td><td>CTGGACTGGAGGCGCGTTTTG</td><td>21</td><td>94</td><td>FGSG_08403</td><td>-</td></tr>
<tr><td>siRNA-t0011179</td><td>GACATATTCGGACTCATCCCA</td><td>21</td><td>94</td><td>FgHV1gp2</td><td>-</td></tr>
<tr><td>siRNA-t0011224</td><td>GCCTATTCATCGTCAATGACCA</td><td>22</td><td>94</td><td>FGSG_08065</td><td>K00100|1|1e-113|408|afm:AFUA_7G04850|[EC:1.1.1.-]</td></tr>
<tr><td>siRNA-t0011364</td><td>ACTTGTTTCTCGTGGGATTGGGCGT</td><td>25</td><td>93</td><td>FgHV1gp2</td><td>-</td></tr>
<tr><td>siRNA-t0011298</td><td>AGAAGAAGTCTGTTGCATTGCT</td><td>22</td><td>93</td><td>FGSG_08645</td><td>K03681|1|4e-120|429|fgr:FG08645.1|exosome complex component RRP40</td></tr>
<tr><td>siRNA-t0011396</td><td>CTGGACTGGAGGCGCGTTTTGG</td><td>22</td><td>93</td><td>FGSG_08403</td><td>-</td></tr>
<tr><td>siRNA-t0011306</td><td>GGAAGGGTTCATACTATCGTTG</td><td>22</td><td>93</td><td>FGSG_12536</td><td>K07050|1|0.0|709|fgr:FG02991.1|</td></tr>
<tr><td>siRNA-t0011329</td><td>TTGGCTACTGGACGAAGACT</td><td>20</td><td>93</td><td>FGSG_00366</td><td>-</td></tr>
<tr><td>siRNA-t0011491</td><td>GAAATGATTTGCGACGTGCT</td><td>20</td><td>92</td><td>FGSG_03245</td><td>K01932|1|1e-53|211|afm:AFUA_3G12920|[EC:6.3.2.-]</td></tr>
<tr><td>siRNA-t0011439</td><td>TCGGACTCATCCCACATGATGGT</td><td>23</td><td>92</td><td>FgHV1gp2</td><td>-</td></tr>
<tr><td>siRNA-t0011616</td><td>AGCGTGTTGTACGGCGTGGT</td><td>20</td><td>91</td><td>FgHV1gp2</td><td>-</td></tr>
<tr><td>siRNA-t0011649</td><td>CGGGACGCGACAAGCAACAAT</td><td>21</td><td>91</td><td>FgHV1gp2</td><td>-</td></tr>
<tr><td>siRNA-t0011576</td><td>CGTAGACGTCGGCACCAACTT</td><td>21</td><td>91</td><td>FGSG_08722</td><td>K15979|1|0.0|1721|fgr:FG08722.1|staphylococcal nuclease domain-containing protein 1</td></tr>
<tr><td>siRNA-t0011586</td><td>GAGTGCTCATCGTTGTGCCA</td><td>20</td><td>91</td><td>FGSG_05527</td><td>K05663|1|5e-114|411|cci:CC1G_07837|mitochondrial ABC transporter ATM</td></tr>
<tr><td>siRNA-t0011638</td><td>GCGCTGTACTGAAGGTGGTG</td><td>20</td><td>91</td><td>FGSG_00974</td><td>-</td></tr>
<tr><td>siRNA-t0011553</td><td>GGGTTCTGGTAGAAGGTGAA</td><td>20</td><td>91</td><td>FGSG_10041</td><td>-</td></tr>
<tr><td>siRNA-t0011604</td><td>GGTGATTGTGGTCATTTGGTT</td><td>21</td><td>91</td><td>FgHV1gp2</td><td>-</td></tr>
<tr><td>siRNA-t0011655</td><td>GTGCGAGCAGGATTGAAGTGTAA</td><td>23</td><td>91</td><td>FgHV1gp2</td><td>-</td></tr>
<tr><td>siRNA-t0011634</td><td>GTTTCTCGTGGGATTGGGCG</td><td>20</td><td>91</td><td>FGSG_09491</td><td>-</td></tr>
<tr><td>siRNA-t0011691</td><td>AGTGACGATACTTGAACCAA</td><td>20</td><td>90</td><td>FGSG_07561</td><td>-</td></tr>
<tr><td>siRNA-t0011769</td><td>CGCGCTGTACTGAAGGTGGTG</td><td>21</td><td>90</td><td>FgHV1gp2</td><td>-</td></tr>
<tr><td>siRNA-t0011696</td><td>CGCTGTACTGAAGGTGGTGTCA</td><td>22</td><td>90</td><td>FgHV1gp2</td><td>-</td></tr>
<tr><td>siRNA-t0011740</td><td>GGTATTGATGAAAGGAGTGCA</td><td>21</td><td>90</td><td>FGSG_13106</td><td>-</td></tr>
<tr><td>siRNA-t0011842</td><td>CCTTCGAACTAGATGGTGTGGCG</td><td>23</td><td>89</td><td>FgHV1gp2</td><td>-</td></tr>
<tr><td>siRNA-t0011790</td><td>GACAATTGTCGTGCGTTCCGT</td><td>21</td><td>89</td><td>FGSG_01049</td><td>-</td></tr>
<tr><td>siRNA-t0011806</td><td>GCATGGGTCGCAGTTGGAATATA</td><td>23</td><td>89</td><td>FGSG_08364</td><td>-</td></tr>
<tr><td>siRNA-t0011968</td><td>ACGCAGCAGAATTCTAGCCT</td><td>20</td><td>88</td><td>FgHV1gp2</td><td>-</td></tr>
<tr><td>siRNA-t0011956</td><td>AGCTGGGGATGTTTGCTTGGTT</td><td>22</td><td>88</td><td>FgHV1gp2</td><td>-</td></tr>
<tr><td>siRNA-t0012064</td><td>AGGAAAGCAGGATCGTTATT</td><td>20</td><td>87</td><td>FGSG_08795</td><td>K15418|1|0.0|743|pcs:Pc21g12440|zearalenone synthase, highly reducing iterative type I polyketide synthase;K15419|3|0.0|713|ani:AN2547.2|emericellamide biosynthesis, highly reducing iterative type I polyketide synthase</td></tr>
<tr><td>siRNA-t0012098</td><td>CCATGGGATCGAAGGTTAGA</td><td>20</td><td>87</td><td>FGSG_11659</td><td>K01932|1|0.0|1431|afm:AFUA_5G12730|[EC:6.3.2.-]</td></tr>
<tr><td>siRNA-t0012042</td><td>CCCTGGGATTGTATGGTAAT</td><td>20</td><td>87</td><td>FGSG_10267</td><td>K00288|1|0.0|1759|fgr:FG10267.1|methylenetetrahydrofolate dehydrogenase (NADP+) / methenyltetrahydrofolate cyclohydrolase / formyltetrahydrofolate synthetase [EC:1.5.1.5 3.5.4.9 6.3.4.3]</td></tr>
<tr><td>siRNA-t0012086</td><td>CCGGGTGGATCTGGGGTCTG</td><td>20</td><td>87</td><td>FGSG_11110</td><td>-</td></tr>
<tr><td>siRNA-t0012099</td><td>CTGCGGTACGATATTTGAGT</td><td>20</td><td>87</td><td>FGSG_11234</td><td>K15505|1|7e-25|115|ssl:SS1G_03212|DNA repair protein RAD5 [EC:3.6.4.-];K15711|5|2e-21|103|tve:TRV_04890|SWI/SNF-related matrix-associated actin-dependent regulator of chromatin subfamily A3 [EC:3.6.4.- 6.3.2.19]</td></tr>
<tr><td>siRNA-t0012054</td><td>GAATCGATCGTCATGGTGATACCC</td><td>24</td><td>87</td><td>FgHV1gp2</td><td>-</td></tr>
<tr><td>siRNA-t0012177</td><td>AAGTGTAGGACGATCCACTC</td><td>20</td><td>86</td><td>FGSG_06513</td><td>K07241|1|0.0|761|fgr:FG06513.1|high-affinity nickel-transport protein</td></tr>
<tr><td>siRNA-t0012246</td><td>AATGGCGCATACACGGAACCCA</td><td>22</td><td>86</td><td>FgHV1gp2</td><td>-</td></tr>
<tr><td>siRNA-t0012134</td><td>AGTGAAAGTGTCGTAGGAAAGGAA</td><td>24</td><td>86</td><td>FgHV1gp1</td><td>-</td></tr>
<tr><td>siRNA-t0012240</td><td>GAGATCACGTTCGGAGACTT</td><td>20</td><td>86</td><td>FGSG_10464</td><td>K15394|1|0.0|1754|act:ACLA_078660|hybrid polyketide synthase / nonribosomal peptide synthetase ACE1</td></tr>
<tr><td>siRNA-t0012149</td><td>GAGTGCCATGACGTACGACCC</td><td>21</td><td>86</td><td>FgHV1gp1</td><td>-</td></tr>
<tr><td>siRNA-t0012225</td><td>GCAGCAACTGTCGCCTGGAC</td><td>20</td><td>86</td><td>FGSG_10995</td><td>K05658|1|4e-24|113|yli:YALI0F20174g|ATP-binding cassette, subfamily B (MDR/TAP), member 1;K05663|4|2e-23|111|bfu:BC1G_07740|mitochondrial ABC transporter ATM</td></tr>
<tr><td>siRNA-t0012194</td><td>GCTGATGATCATGACGTGGAGA</td><td>22</td><td>86</td><td>FGSG_11121</td><td>-</td></tr>
<tr><td>siRNA-t0012153</td><td>GGACCGACGTTTTGGCGAGACCGAT</td><td>25</td><td>86</td><td>FgHV1gp2</td><td>-</td></tr>
<tr><td>siRNA-t0012138</td><td>GGAGACGGCAGGTAACTCCTTCA</td><td>23</td><td>86</td><td>FGSG_11154</td><td>-</td></tr>
<tr><td>siRNA-t0012223</td><td>GGATGATATTCGGTATTGTCGAG</td><td>23</td><td>86</td><td>FgHV1gp2</td><td>-</td></tr>
<tr><td>siRNA-t0012131</td><td>GGCGAGACCGATGATTTCCAT</td><td>21</td><td>86</td><td>FGSG_12379</td><td>K03293|1|3e-136|484|cal:CaO19.6993|amino acid transporter, AAT family</td></tr>
<tr><td>siRNA-t0012160</td><td>TCACTGTTGACGTCACCCTTT</td><td>21</td><td>86</td><td>FgHV1gp2</td><td>-</td></tr>
<tr><td>siRNA-t0012336</td><td>ACGGGTCCAGATGTTGTGCTTT</td><td>22</td><td>85</td><td>FGSG_07939</td><td>K05236|1|0.0|2545|fgr:FG07939.1|coatomer protein complex, subunit alpha (xenin)</td></tr>
<tr><td>siRNA-t0012346</td><td>AGAGCTGGGGATGTTTGCTTGGTT</td><td>24</td><td>85</td><td>FgHV1gp2</td><td>-</td></tr>
<tr><td>siRNA-t0012266</td><td>AGGACAAGCCGTCAAACATAGACA</td><td>24</td><td>85</td><td>FgHV1gp2</td><td>-</td></tr>
<tr><td>siRNA-t0012378</td><td>GAGGGCCCAGTCATGAATTTTCGGT</td><td>25</td><td>85</td><td>FgHV1gp2</td><td>-</td></tr>
<tr><td>siRNA-t0012262</td><td>GCCAAATCGTCATACATTGCAC</td><td>22</td><td>85</td><td>FgHV1gp2</td><td>-</td></tr>
<tr><td>siRNA-t0012322</td><td>GCGCTGTACTGAAGGTGGTGTC</td><td>22</td><td>85</td><td>FgHV1gp2</td><td>-</td></tr>
<tr><td>siRNA-t0012448</td><td>GCAGGATCTCTGACATATTCGGA</td><td>23</td><td>84</td><td>FgHV1gp2</td><td>-</td></tr>
<tr><td>siRNA-t0012457</td><td>GGGGTCGTACGTCATGGCACT</td><td>21</td><td>84</td><td>FGSG_10228</td><td>K03114|1|0.0|934|fgr:FG10228.1|mitosis inhibitor protein kinase SWE1 [EC:2.7.11.1]</td></tr>
<tr><td>siRNA-t0012514</td><td>ATGAACGTCTGACTGTAGTGTGCCT</td><td>25</td><td>83</td><td>FgHV1gp2</td><td>-</td></tr>
<tr><td>siRNA-t0012543</td><td>CGGACTCATCCCACATGATGGT</td><td>22</td><td>83</td><td>FgHV1gp2</td><td>-</td></tr>
<tr><td>siRNA-t0012628</td><td>GAGGATTTGGCCGTCTGGAG</td><td>20</td><td>83</td><td>FGSG_00819</td><td>K14403|1|0.0|1546|fgr:FG00819.1|cleavage and polyadenylation specificity factor subunit 3 [EC:3.1.27.-]</td></tr>
<tr><td>siRNA-t0012760</td><td>CGCAGCAGAATTCTAGCCTGGTT</td><td>23</td><td>82</td><td>FgHV1gp2</td><td>-</td></tr>
<tr><td>siRNA-t0012774</td><td>GAAGAACGTAGATGTGATGCCTTG</td><td>24</td><td>82</td><td>FgHV1gp2</td><td>-</td></tr>
<tr><td>siRNA-t0012635</td><td>GGTTCAAGTATCGTCACTTCTT</td><td>22</td><td>82</td><td>FgHV1gp2</td><td>-</td></tr>
<tr><td>siRNA-t0012841</td><td>AGGCCCTGGGATTGTATGGTAATT</td><td>24</td><td>81</td><td>FgHV1gp2</td><td>-</td></tr>
<tr><td>siRNA-t0012912</td><td>GACGATAGTGGGAGCGAAAGC</td><td>21</td><td>81</td><td>FGSG_08335</td><td>K02873|1|8e-89|325|fgr:FG08335.1|large subunit ribosomal protein L13e</td></tr>
<tr><td>siRNA-t0012817</td><td>GGGACGTCTTCGATGGATGCTGC</td><td>23</td><td>81</td><td>FgHV1gp2</td><td>-</td></tr>
<tr><td>siRNA-t0013032</td><td>ACGATAGTGGGAGCGAAAGCGA</td><td>22</td><td>80</td><td>FGSG_13563</td><td>-</td></tr>
<tr><td>siRNA-t0013062</td><td>AGAAGTCTGTTGCATTGCTT</td><td>20</td><td>80</td><td>FGSG_08719</td><td>K10352|1|0.0|4469|fgr:FG08719.1|myosin heavy chain</td></tr>
<tr><td>siRNA-t0013014</td><td>CAGAGCTGGGGATGTTTGCTT</td><td>21</td><td>80</td><td>FGSG_10105</td><td>K12864|1|8e-17|88.2|cne:CNH03260|beta-catenin-like protein 1</td></tr>
<tr><td>siRNA-t0012933</td><td>CCTAGACCGAAGAAAGACCACAT</td><td>23</td><td>80</td><td>FgHV1gp2</td><td>-</td></tr>
<tr><td>siRNA-t0012955</td><td>GCTGAATGCTCTGCCTCGGT</td><td>20</td><td>80</td><td>FGSG_09306</td><td>K11644|1|0.0|2881|fgr:FG09306.1|paired amphipathic helix protein Sin3a</td></tr>
<tr><td>siRNA-t0012983</td><td>GTCGACAGGCGTGGCTGAGAGAA</td><td>23</td><td>80</td><td>FgHV1gp2</td><td>-</td></tr>
<tr><td>siRNA-t0013051</td><td>TCGTGGGATTGGGCGTGTGTTCTTT</td><td>25</td><td>80</td><td>FgHV1gp2</td><td>-</td></tr>
<tr><td>siRNA-t0013176</td><td>ACGTGGACGGGTGTGAAGCT</td><td>20</td><td>79</td><td>FGSG_12200</td><td>K04718|1|0.0|682|fgr:FG02775.1|sphingosine kinase [EC:2.7.1.91]</td></tr>
<tr><td>siRNA-t0013090</td><td>CGGGACCGACGTTTTGGCGAGA</td><td>22</td><td>79</td><td>FgHV1gp2</td><td>-</td></tr>
<tr><td>siRNA-t0013136</td><td>GCGCGCTGTACTGAAGGTGGTG</td><td>22</td><td>79</td><td>FgHV1gp2</td><td>-</td></tr>
<tr><td>siRNA-t0013143</td><td>GCTTGATGGTTGGCACAACGA</td><td>21</td><td>79</td><td>FgHV1gp2</td><td>-</td></tr>
<tr><td>siRNA-t0013070</td><td>TCACTGTTGACGTCACCCTT</td><td>20</td><td>79</td><td>FGSG_08155</td><td>K00777|1|4e-53|209|afm:AFUA_7G06290|[EC:2.4.2.-];K15110|5|6e-16|86.3|ani:AN1299.2|solute carrier family 25 (mitochondrial 2-oxodicarboxylate transporter), member 21</td></tr>
<tr><td>siRNA-t0013114</td><td>TGCAAGCGTGACGAAGATCCC</td><td>21</td><td>79</td><td>FGSG_11395</td><td>K01932|1|0.0|1024|afm:AFUA_1G10380|[EC:6.3.2.-]</td></tr>
<tr><td>siRNA-t0013081</td><td>TGGACCTGAGAGCATGAAGGC</td><td>21</td><td>79</td><td>FGSG_08097</td><td>K00688|1|7e-16|87.0|pno:SNOG_13460|starch phosphorylase [EC:2.4.1.1]</td></tr>
</table>

siRNA-t0013283	CTTGAAGGCGAAGTATGAGGGAGA	24	78	FgHV1gp2	-
siRNA-t0013317	GATGAGATCACGTTCGGAGA	20	78	FGSG_11059	-
siRNA-t0013370	AAGAACGTAGATGTGATGCCTT	22	77	FgHV1gp2	-
siRNA-t0013484	ATTGGCTACTGGACGAAGACT	21	77	FGSG_00366	-
siRNA-t0013451	CAAAATGCCATGGGATCGAAGGTT	24	77	FGSG_09064	-
siRNA-t0013507	GATGTAGGCTCCTCCGACAATGGCG	25	77	FgHV1gp2	-
siRNA-t0013457	GCGTGACGAAGATCCCACTCT	21	77	FgHV1gp1	-
siRNA-t0013514	GGAGTGGCAGTGTAATGGAGGACG	24	77	FgHV1gp2	-
siRNA-t0013394	GTGCCATGACGTACGACCCCGAA	23	77	FgHV1gp1	-
siRNA-t0013650	AGAAGATTGAGTTCATTTGTTCCAT	25	76	FgHV1gp2	-
siRNA-t0013586	CCGTTTGTGCTGCAAGATGGACT	23	76	FgHV1gp2	-
siRNA-t0013529	CTGTTCTCTGTCGGCATGTTG	21	76	FGSG_11657	K09184|1|5e-13|75.9|bfu:BC1G_09852|GATA-binding protein, other eukaryote
siRNA-t0013575	TCACTGTTGACGTCACCCTTTG	22	76	FgHV1gp2	-
siRNA-t0013792	AGAAGATTGAGTTCATTTGTTCCA	24	75	FgHV1gp2	-
siRNA-t0013788	GAGCAGGATTGAAGTGTAATGCG	23	75	FgHV1gp2	-
siRNA-t0013756	GTCATGATCATCAGCACCGTACGG	24	75	FgHV1gp2	-
siRNA-t0013850	CACGCAGCAGAATTCTAGCCT	21	74	FgHV1gp2	-
siRNA-t0013899	CCCGAGAAAAACGCCCCTGGACGGT	25	74	FgHV1gp2	-
siRNA-t0013920	GCGCTGTACTGAAGGTGGTGTCAT	24	74	FgHV1gp2	-
siRNA-t0013844	GGTTCAAGTATCGTCACTTCTTC	23	74	FgHV1gp2	-
siRNA-t0013914	TGGTGGATGGTACGTGGCTC	20	74	FGSG_01251	K01175|1|3e-63|243|afm:AFUA_7G01550|[EC:3.1.-.-]
siRNA-t0014022	AAGTAAGCAGAGCGTGTAGGGC	22	73	FgHV1gp2	-
siRNA-t0014081	AGAAGAAGTCTGTTGCATTG	20	73	FGSG_11272	K08711|1|0.0|1632|cne:CNB05770|ATP-binding cassette, subfamily G (WHITE), member 2, PDR
siRNA-t0014019	CACTGTTGACGTCACCCTTTG	21	73	FgHV1gp2	-
siRNA-t0014121	CCAGAACCCAGCACCTTTCCCT	22	73	FgHV1gp2	-
siRNA-t0014097	CCTGAGAGCATGAAGGCACAA	21	73	FGSG_08778	K09595|1|0.0|1018|fgr:FG08778.1|minor histocompatibility antigen H13 [EC:3.4.23.-]
siRNA-t0014144	GACATATTCGGACTCATCCCAC	22	73	FgHV1gp2	-
siRNA-t0014091	GGTGAGAAGATTGAGTTCATTTGTT	25	73	FgHV1gp2	-
siRNA-t0014079	GGTGATCCTAAACATGTCGGTG	22	73	FGSG_11659	K01932|1|0.0|1431|afm:AFUA_5G12730|[EC:6.3.2.-]
siRNA-t0014181	AAGAACCCATATGGATCGGTGGCA	24	72	FgHV1gp2	-
siRNA-t0014238	AGAACGCTGAAATCGAGGGTGTT	23	72	FgHV1gp2	-
siRNA-t0014278	AGAGTCTTGCGGTGTGGGTCTT	22	72	FgHV1gp2	-
siRNA-t0014322	CGAGCAGGATTGAAGTGTAATG	22	72	FgHV1gp2	-
siRNA-t0014301	GAAGGCGAAGTATGAGGGAGA	21	72	FgHV1gp2	-
siRNA-t0014243	GCGAGCAGGATTGAAGTGTAAT	22	72	FgHV1gp2	-
siRNA-t0014237	GGGTCTTGACTTGGGCTGTA	20	72	FGSG_13911	K09424|1|1e-28|128|lth:KLTH0C07788g|Myb-like DNA-binding protein REB1
siRNA-t0014231	GTGACCAGCTGTTCTCTGTCGGCAT	25	72	FgHV1gp2	-
siRNA-t0014251	GTGCTGAATGCTCTGCCTCGGTC	23	72	FgHV1gp2	-
siRNA-t0014221	TATGGATCGGTGGCACACGCAT	22	72	FgHV1gp2	-
siRNA-t0014497	ATGTGGTGAGGTATGACGTGTTCA	24	71	FgHV1gp2	-
siRNA-t0014377	CAGCTGTTCTCTGTCGGCATG	21	71	FgHV1gp2	-
siRNA-t0014358	GAGCAAAGGCGATCGCTGGA	20	71	FgHV1gp2	-
siRNA-t0014597	AAGTGTCGTAGGAAAGGAAAAGT	23	70	FgHV1gp1	-
siRNA-t0014638	ACCAATCACACGCGGAACTGT	21	70	FGSG_09787	-
siRNA-t0014688	CCGCGGCAGCATGGAAGGCG	20	70	FGSG_04637	K03293|1|2e-111|402|zro:ZYRO0D03762g|amino acid transporter, AAT family
siRNA-t0014627	CTTGAAGGCGAAGTATGAGGGAG	23	70	FgHV1gp2	-
siRNA-t0014685	GATGTGGTGAGGTATGACGTGTTC	24	70	FgHV1gp2	-
siRNA-t0014678	GCAAGCGTGACGAAGATCCC	20	70	FGSG_07746	-
siRNA-t0014581	GCGCTGTACTGAAGGTGGTGTCA	23	70	FgHV1gp2	-
siRNA-t0014859	ACCAATCACACGCGGAACTG	20	69	FGSG_09787	-
siRNA-t0014881	AGAGACAGAGCTCGCGCCTTCCA	23	69	FgHV1gp2	-
siRNA-t0014889	CCGAAACGTCGTTCTGGCAT	20	69	FGSG_08781	K00936|1|4e-23|110|afm:AFUA_6G10240|[EC:2.7.3.-];K11231|2|1e-20|102|ure:UREG_06918|osomolarity two-component system, sensor histidine kinase SLN1 [EC:2.7.13.3];K02486|4|1e-17|92.4|spo:SPAC27E2.09|two-component system, unclassified family, sensor kinase [EC:2.7.13.3]
siRNA-t0014821	CGACAGTTGCTGCAGCTGGT	20	69	FGSG_05306	K08333|1|0.0|3035|fgr:FG05306.1|phosphoinositide-3-kinase, regulatory subunit 4, p150 [EC:2.7.11.1]
siRNA-t0014863	GGCTTGTCGTCGGGAGAATC	20	69	FGSG_09338	K03125|1|0.0|2056|fgr:FG09338.1|transcription initiation factor TFIID subunit 1
siRNA-t0014853	TCTGACGAATTTGGACAAGTACTC	24	69	FgHV1gp2	-
siRNA-t0014907	AGGGACGTCTTCGATGGATGCT	22	68	FgHV1gp2	-
siRNA-t0015058	CACTGTTGACGTCACCCTTT	20	68	FGSG_09884	K00472|1|2e-09|63.9|fgr:FG05473.1|prolyl 4-hydroxylase [EC:1.14.11.2]
siRNA-t0015020	CAGCTGTTCTCTGTCGGCATGTT	23	68	FgHV1gp2	-
siRNA-t0015082	GAAATGATTTGCGACGTGCTTG	22	68	FgHV1gp2	-
siRNA-t0014917	GCGTGAGTGCGTAGATAAAGG	21	68	FGSG_03839	-
siRNA-t0014919	GGTGCGAGCAGGATTGAAGTGTAAT	25	68	FgHV1gp2	-
siRNA-t0015039	TGAGTGCGTAGATAAAGGTT	20	68	FGSG_01801	-
siRNA-t0015037	TGCTGCATGTACTGTTCGGCA	21	68	FGSG_01434	-
siRNA-t0014961	TGGAAATCATCGGTCTCGCCA	21	68	FGSG_08343	K01535|1|0.0|1497|nhe:NECHADRAFT_77500|H+-transporting ATPase [EC:3.6.3.6]
siRNA-t0015168	ACGGGTCCAGATGTTGTGCTT	21	67	FGSG_07939	K05236|1|0.0|2545|fgr:FG07939.1|coatomer protein complex, subunit alpha (xenin)
siRNA-t0015256	ATTGGCTACTGGACGAAGACTT	22	67	FGSG_00366	-
siRNA-t0015120	CGGGACCGACGTTTTGGCGAGAC	23	67	FgHV1gp2	-
siRNA-t0015255	GCATTCAGTAGTCGGGGTCTCC	22	67	FgHV1gp1	-
siRNA-t0015099	GGATACGTTCTGAGAAGTCTCCGAA	25	67	FgHV1gp2	-
siRNA-t0015182	GTGATTGTGGTCATTTGGTTTGTGA	25	67	FgHV1gp2	-
siRNA-t0015129	TGCTGATGATCATGACGTGGAGA	23	67	FGSG_11121	-
siRNA-t0015444	AAAGTGTCGTAGGAAAGGAA	20	66	FgHV1gp1	-
siRNA-t0015302	AGGCGCATCGGCTTACCCTCGCA	23	66	FGSG_05873	-
siRNA-t0015489	ATCAGGCATGTATGGGTCGTT	21	66	FGSG_03750	K11244|1|7e-07|55.5|fgr:FG10787.1|cell wall integrity and stress response component
siRNA-t0015447	CAAGCGTGACGAAGATCCCACTCT	24	66	FgHV1gp1	-
siRNA-t0015343	CGACATGTTTAGGATCACCAA	21	66	FGSG_03933	K12618|1|1e-55|215|ani:AN8185.2|5'-3' exoribonuclease 1 [EC:3.1.13.-]
siRNA-t0015433	CGGTGTGGACATCGGAGCGG	20	66	FGSG_08980	K00002|1|0.0|716|fgr:FG08980.1|alcohol dehydrogenase (NADP+) [EC:1.1.1.2]
siRNA-t0015451	CTATAGGAGTACCAACAGCGGA	22	66	FGSG_08853	-
siRNA-t0015355	GCAGACACTTGTTTCTCGTGGGAT	24	66	FgHV1gp2	-
siRNA-t0015383	GTGGTGAGGTATGACGTGTT	20	66	FGSG_08757	K01180|1|0.0|667|afm:AFUA_1G04260|endo-1,3(4)-beta-glucanase [EC:3.2.1.6]
siRNA-t0015325	TCGGACTCATCCCACATGATGGTGT	25	66	FgHV1gp2	-
siRNA-t0015488	TCGTGAACTGCTGCGCTATGGT	22	66	FgHV1gp2	-
siRNA-t0015522	TTCGCGCGCTGTACTGAAGGTGGT	24	66	FgHV1gp2	-
siRNA-t0015611	CACGCAGCAGAATTCTAGCCTGGT	24	65	FgHV1gp2	-
siRNA-t0015674	CTCAAGGATTTGTAAGGGGAA	21	65	FGSG_00542	-
siRNA-t0015666	CTTTCGGGGTCGTACGTCATGG	22	65	FGSG_02394	K01932|1|0.0|942|afm:AFUA_1G10380|[EC:6.3.2.-]
siRNA-t0015672	GCCCTCAGTAGTCGACATGTC	21	65	FGSG_02945	K13095|1|6e-68|257|fgr:FG02080.1|splicing factor 1;K08192|2|2e-64|245|dha:DEHA2C12276g|MFS transporter, ACS family, allantoate permease
siRNA-t0015620	GCTGCAGCAACTGTCGCCTGGACA	24	65	FgHV1gp1	-
siRNA-t0015572	GGCTACTGGACGAAGACTTG	20	65	FGSG_05101	K06685|1|9e-09|60.8|pcs:Pc20g14730|maintenance of ploidy protein MOB1 (MPS1 binder 1)
siRNA-t0015892	CATATGGATCGGTGGCACACGCAT	24	64	FgHV1gp2	-

siRNA-t0015871	CCATGAGTGGGTCGAGGATGCAA	23	64	FGSG_11308	-
siRNA-t0015923	CGGTACGTGGAAATCTTCAGGCCCT	25	64	FgHV1gp2	-
siRNA-t0015944	GCGTTTGTGGAGCCATGGGT	20	64	FGSG_01348	K10771|1|0.0|826|fgr:FG01348.1|AP endonuclease 1 [EC:4.2.99.18]
siRNA-t0015727	GGAGACTAAACGCTTTGGGCA	21	64	FgHV1gp1	-
siRNA-t0015917	TCATCGGGAATCATGTCGGT	20	64	FGSG_08874	-
siRNA-t0015909	TCTGGATGTGGGTGCGCCGA	20	64	FGSG_11355	K01824|1|8e-09|60.5|nhe:NECHADRAFT_68940|cholestenol delta-isomerase [EC:5.3.3.5];K09246|3|3e-07|55.1|ago:AGOS_AAL175W|arginine metabolism regulation protein II
siRNA-t0016030	CTGAGGATTTGGCCGTCTGGA	21	63	FGSG_00819	K14403|1|0.0|1546|fgr:FG00819.1|cleavage and polyadenylation specificity factor subunit 3 [EC:3.1.27.-]
siRNA-t0016161	GGAGATCCGGTAAAGCATTG	20	63	FGSG_10354	-
siRNA-t0016136	GGGTCTTGACTTGGGCTGTAC	21	63	FGSG_09304	-
siRNA-t0016061	GTTGAATCGATCGTCATGGTGA	22	63	FgHV1gp2	-
siRNA-t0016110	TCTGACTGTTGCTTGATGGT	20	63	FGSG_05424	K12878|1|0.0|1596|fgr:FG05424.1|THO complex subunit 1
siRNA-t0016095	TGCTGGGTTCTGGTAGAAGGT	21	63	FGSG_06860	K12836|1|5e-113|405|fgr:FG06860.1|splicing factor U2AF 35 kDa subunit
siRNA-t0016316	CACTGTTGACGTCACCCTTTGG	22	62	FGSG_06368	-
siRNA-t0016231	CAGCAGAATTCTAGCCTGGT	20	62	FgHV1gp2	-
siRNA-t0016276	CGCTGTACTGAAGGTGGTGT	20	62	FGSG_13828	K09240|1|3e-08|59.3|sce:YMR280C|transcriptional regulatory protein CAT8;K09241|2|9e-07|54.7|ani:AN4785.2|transcriptional regulatory protein GAL4
siRNA-t0016398	CTCACTGTTGACGTCACCCTTTGGA	25	62	FgHV1gp2	-
siRNA-t0016380	GGGTCTTCTGACGAATTTGGACAA	24	62	FgHV1gp2	-
siRNA-t0016277	TCTGGCCGGTTGAAAATGTTCCAC	24	62	FgHV1gp2	-
siRNA-t0016309	TGTTGCTTGATGGTTGGCACA	21	62	FgHV1gp2	-
siRNA-t0016581	CCAGCTGTTCTCTGTCGGCA	20	61	FGSG_12508	-
siRNA-t0016483	TCGTTGAATCGATCGTCATGGTG	23	61	FgHV1gp2	-
siRNA-t0016731	AGGCTTGTCGTCGGGAGAATCCA	23	60	FgHV1gp2	-
siRNA-t0016830	CAAACTGTTCATTTCGGCGA	20	60	FGSG_05794	K15419|1|7e-100|366|ani:AN2547.2|emericellamide biosynthesis, highly reducing iterative type I polyketide synthase
siRNA-t0016832	GATGGTACGTGGCTCACCCT	20	60	FGSG_09454	K14293|1|0.0|1735|fgr:FG09454.1|importin subunit beta-1
siRNA-t0016683	GCCATGACGTACGACCCCGA	20	60	FgHV1gp1	-
siRNA-t0016695	GGGACGCGACAAGCAACAATTT	22	60	FgHV1gp2	-
siRNA-t0016819	GTGAAAGTGTCGTAGGAAAGGAAA	24	60	FgHV1gp1	-
siRNA-t0017044	ATGCAGCGGCAAATACAGGACC	22	59	FgHV1gp2	-
siRNA-t0016993	ATGTGGTGAGGTATGACGTGTT	22	59	FgHV1gp2	-
siRNA-t0016992	CGAGGCCATAGGGAAGATCAA	21	59	FGSG_02663	-
siRNA-t0017095	GAAGTTCTCTCTGACCTTGAT	21	59	FGSG_06807	-
siRNA-t0017011	GAGTCTTGCGGTGTGGGTCTTCT	23	59	FgHV1gp2	-
siRNA-t0016990	TGGCGCATACACGGAACCCAT	21	59	FgHV1gp2	-
siRNA-t0017274	ACGCAGCAGAATTCTAGCCTGGT	23	58	FgHV1gp2	-
siRNA-t0017323	AGGTTTGAACCCTGACAATACCAA	24	58	FgHV1gp2	-
siRNA-t0017155	CGGAACTGTCTCAAGGTCATG	21	58	FGSG_06420	K02202|1|3e-17|89.7|ssl:SS1G_06051|cyclin-dependent kinase 7 [EC:2.7.11.22 2.7.11.23]
siRNA-t0017237	GACCTGAGAGCATGAAGGCA	20	58	FGSG_08097	K00688|1|7e-16|87.0|pno:SNOG_13460|starch phosphorylase [EC:2.4.1.1]
siRNA-t0017330	GAGTCTTGCGGTGTGGGTCT	20	58	FGSG_00874	K11400|1|0.0|972|fgr:FG00874.1|actin-related protein 4
siRNA-t0017118	GCTGAATGCTCTGCCTCGGTCA	22	58	FgHV1gp2	-
siRNA-t0017164	GGCAACGGAAGCGATATTTGCA	22	58	FgHV1gp2	-
siRNA-t0017302	GTACTTGCCGCGCGTCTCACTGA	23	58	FgHV1gp2	-
siRNA-t0017143	TTGAGAACGCTGAAATCGAGGGTG	24	58	FgHV1gp2	-
siRNA-t0017186	TTGGCTACTGGACGAAGACTTG	22	58	FGSG_00366	-
siRNA-t0017624	AACTGGACAGAAGTTTTGTGGTA	23	57	FGSG_08662	K06657|1|7e-08|58.5|lel:LELG_00705|G1/S-specific cyclin PLC2
siRNA-t0017626	AGAAGAAGTCTGTTGCATTGC	21	57	FGSG_08645	K03681|1|4e-120|429|fgr:FG08645.1|exosome complex component RRP40
siRNA-t0017475	AGTTCTCTCTGACCTTGATGGCGT	24	57	FGSG_01555	K00784|1|2e-34|146|ani:AN0951.2|ribonuclease Z [EC:3.1.26.11]
siRNA-t0017562	ATGGCGCATACACGGAACCCAT	22	57	FgHV1gp2	-
siRNA-t0017419	CGTAGTCATCGTCAGGGTACG	21	57	FGSG_09801	K03164|1|0.0|2817|fgr:FG09801.1|DNA topoisomerase II [EC:5.99.1.3]
siRNA-t0017381	GAGTGCCATGACGTACGACCCC	22	57	FgHV1gp1	-
siRNA-t0017702	ATCGATCGTCATGGTGATACC	21	56	FgHV1gp2	-
siRNA-t0017723	CCATAACCTCGTTGGGACCTTT	22	56	FgHV1gp2	-
siRNA-t0017720	CCGAGAAAAACGCCCCTGGACGGTA	25	56	FgHV1gp2	-
siRNA-t0017737	CCTAGACCGAAGAAAGACCACA	22	56	FgHV1gp2	-
siRNA-t0017903	GAGAGACGTCTGTTCTACAC	20	56	FGSG_09856	K09506|1|4e-63|241|cim:CIMG_07253|DnaJ homolog subfamily A member 5
siRNA-t0017690	GCGTGAGTGCGTAGATAAAGGTTAT	25	56	FgHV1gp2	-
siRNA-t0017773	GGTCGAAACGGATGGTGTAG	20	56	FGSG_08343	K01535|1|0.0|1497|nhe:NECHADRAFT_77500|H+-transporting ATPase [EC:3.6.3.6]
siRNA-t0017788	GTCCAATGTAAGTGACGAGGCCTCT	25	56	FgHV1gp2	-
siRNA-t0017840	TGCGACCCATGCTTTCGACGT	21	56	FgHV1gp2	-
siRNA-t0018000	AATGGCGCATACACGGAACCCAT	23	55	FgHV1gp2	-
siRNA-t0018079	ACCAGCTGTTCTCTGTCGGCA	21	55	FGSG_12508	-
siRNA-t0018139	ACGCAGGACGATAGTGGGAG	20	55	FGSG_01223	-
siRNA-t0018025	AGGAGCTCAAAGGTATGATGT	21	55	FGSG_08060	K00688|1|4e-11|69.3|pno:SNOG_13460|starch phosphorylase [EC:2.4.1.1]
siRNA-t0018050	CCGACGGTACGTGGAAATCTTCA	23	55	FgHV1gp2	-
siRNA-t0017960	CGTCATGATCATCAGCACCGTACGG	25	55	FgHV1gp2	-
siRNA-t0017922	GGAAGTCCACGATTGTAACGA	21	55	FGSG_05306	K08333|1|0.0|3035|fgr:FG05306.1|phosphoinositide-3-kinase, regulatory subunit 4, p150 [EC:2.7.11.1]
siRNA-t0018097	GGTTTCGGGACGCGACAAGCAA	22	55	FgHV1gp2	-
siRNA-t0017982	TGGAAATCTTCAGGCCCTGGGA	22	55	FgHV1gp2	-
siRNA-t0018386	ACTTTCGGGGTCGTACGTCAT	21	54	FGSG_02394	K01932|1|0.0|942|afm:AFUA_1G10380|[EC:6.3.2.-]
siRNA-t0018364	AGAAGCTTGACACGAATGATGTAC	24	54	FgHV1gp2	-
siRNA-t0018457	AGAATGTCGAGATACTCTTC	20	54	FGSG_13169	K00750|1|4e-16|84.7|cdu:CD36_86420|glycogenin glucosyltransferase [EC:2.4.1.186]
siRNA-t0018469	AGTGCTGAATGCTCTGCCTCGGTC	24	54	FgHV1gp2	-
siRNA-t0018280	CAGAGCTGGGGATGTTTGCTTGGT	24	54	FgHV1gp2	-
siRNA-t0018346	CGTTACAATCGTGGACTTCCT	21	54	FgHV1gp2	-
siRNA-t0018335	CTCGTGGGATTGGGCGTGTGTTC	23	54	FgHV1gp2	-
siRNA-t0018205	GATGATCATGACGTGGAGAAGAA	23	54	FGSG_01407	-
siRNA-t0018297	GCACGGTCGTCGCACAGGAT	20	54	FGSG_01199	K00820|1|0.0|1319|fgr:FG01199.1|glucosamine--fructose-6-phosphate aminotransferase (isomerizing) [EC:2.6.1.16]
siRNA-t0018303	TCGTTGAATCGATCGTCATG	20	54	FGSG_11372	K00777|1|5e-21|103|afm:AFUA_7G06290|[EC:2.4.2.-];K12823|4|1e-10|69.7|ure:UREG_02211|ATP-dependent RNA helicase DDX5/DBP2 [EC:3.6.4.13]
siRNA-t0018376	TTCGGACTCATCCCACATGATGGT	24	54	FgHV1gp2	-
siRNA-t0018492	AGAGGACCCCTGATGCCTGG	20	53	FGSG_07373	K08498|1|2e-111|402|ani:AN8171.2|syntaxin 6 K08499 member of the syntaxin family of t-SNAREs
siRNA-t0018544	AGAGTCTTGCGGTGTGGGTCTTC	23	53	FgHV1gp2	-
siRNA-t0018495	AGCAGAATTCTAGCCTGGTTGA	22	53	FgHV1gp2	-
siRNA-t0018731	AGTATGAACCCTTCCACGATTGGCG	25	53	FgHV1gp2	-
siRNA-t0018660	ATGTAGGTCTGGCATAGGAA	20	53	FGSG_00368	-
siRNA-t0018631	CAGCAGAATTCTAGCCTGGTT	21	53	FgHV1gp2	-
siRNA-t0018673	CGACGTACTTAGGGTTGTTGA	21	53	FgHV1gp2	-
siRNA-t0018532	GCGGCAAGTACAAACTCCCT	20	53	FGSG_01163	-
siRNA-t0018621	GGCAACGGAAGCGATATTTGCAC	23	53	FgHV1gp2	-
siRNA-t0018769	TAGACCGAAGAAAGACCACATCCA	24	53	FgHV1gp2	-
siRNA-t0018633	TGGAGTGGCATATTCAAGAATCA	23	53	FgHV1gp2	-
siRNA-t0019066	AGGACCAGTTCCTGCTGAGTGTGGT	25	52	FgHV1gp2	-
siRNA-t0018784	AGGTGGGTTCTTGAAGTTGGTG	22	52	FgHV1gp2	-
siRNA-t0019007	CAACGGTTCTTGGAAAGTTGGAG	23	52	FgHV1gp2	-
siRNA-t0018823	GGCGGGACCAGGATTCTTCTCC	22	52	FGSG_08844	K01183|1|1e-06|55.1|pan:PODANSg4492|chitinase [EC:3.2.1.14]

siRNA-t0019119	GGTGCTGATGATCATGACGTG	21	52	FGSG_11121	-
siRNA-t0018968	TACTTGATCTCAGCACGCCGGT	22	52	FgHV1gp2	-
siRNA-t0018907	TGCCATGACGTACGACCCCGA	21	52	FgHV1gp1	-
siRNA-t0018849	TGGGTTCTTGAAGTTGGTGCCGA	23	52	FgHV1gp2	-
siRNA-t0019401	AGAGCTGGGGATGTTTGCTT	20	51	FGSG_13242	-
siRNA-t0019245	GCGGAGATCCGGTAAAGCATT	21	51	FGSG_06511	-
siRNA-t0019207	GGACCGACGTTTTGGCGAGAC	21	51	FgHV1gp2	-
siRNA-t0019264	GTGGTGAGGTATGACGTGTTCA	22	51	FgHV1gp2	-
siRNA-t0019148	TAACACGCAGCAGAATTCTAGCCT	24	51	FgHV1gp2	-
siRNA-t0019333	TAGCTTGCTAACTTCTGGAT	20	51	FGSG_02315	K01932|1|0.0|2455|afm:AFUA_1G10380|[EC:6.3.2.-]
siRNA-t0019258	TGCTGCAAGATGGACTGGCCTG	22	51	FgHV1gp2	-
siRNA-t0019191	TGGGGATGTTTGCTTGGTTTCGGG	24	51	FgHV1gp2	-
siRNA-t0019608	AAAGGTGCGAGCAGGATTGA	20	50	FgHV1gp2	-
siRNA-t0019709	AGGCCCTGGGATTGTATGGTAATTG	25	50	FgHV1gp2	-
siRNA-t0019482	CAGGATTGAAGTGTAATGCGTTGA	24	50	FgHV1gp2	-
siRNA-t0019724	CTCACTGTTGACGTCACCCTTTGG	24	50	FgHV1gp2	-
siRNA-t0019715	GCCCTCAGTAGTCGACATGT	20	50	FGSG_09197	K00021|1|0.0|2147|fgr:FG09197.1|hydroxymethylglutaryl-CoA reductase (NADPH) [EC:1.1.1.34]
siRNA-t0019588	GGGGTCTTGACTTGGGCTGTA	21	50	FGSG_13911	K09424|1|1e-28|128|lth:KLTH0C07788g|Myb-like DNA-binding protein REB1
siRNA-t0019682	GGTGCTGGGTTCTGGTAGAA	20	50	FGSG_07312	-
siRNA-t0019477	TCCTTCGTTGAATCGATCGTCA	22	50	FgHV1gp2	-
siRNA-t0019427	TGTGGTGAGGTATGACGTGTTCATT	25	50	FgHV1gp2	-
siRNA-t0019778	CAGAGCGTGTAGGGCTGTCCCA	22	49	FgHV1gp2	-
siRNA-t0020052	CCGGCAGCGTTTATGGAACA	20	49	FGSG_06161	K14850|1|0.0|818|fgr:FG06161.1|ribosomal RNA-processing protein 8 [EC:2.1.1.-]
siRNA-t0020092	CGCTGATCTGGCTCGTGACTTT	22	49	FgHV1gp2	-
siRNA-t0020019	CGGTGTCGCCATTGATGGGA	20	49	FGSG_11815	K10866|1|0.0|1009|fgr:FG00711.1|DNA repair protein RAD50 [EC:3.6.-.-];K13095|3|2e-94|346|fgr:FG02080.1|splicing factor 1;K01952|4|2e-33|143|pno:SNOG_10851|phosphoribosylformylglycinamidine synthase [EC:6.3.5.3];K08197|5|3e-10|66.2|cim:CIMG_07855|MFS transporter, SIT family, siderophore-iron:H+ symporter
siRNA-t0019950	GCAAGCGTGACGAAGATCCCACTCT	25	49	FgHV1gp1	-
siRNA-t0019824	GCCATGACGTACGACCCCGAAA	22	49	FgHV1gp1	-
siRNA-t0019854	GGAGACTAAACGCTTTGGGCACAT	24	49	FgHV1gp1	-
siRNA-t0019958	TCGTTACAATCGTGGACTTC	20	49	FGSG_12985	K15192|1|0.0|3430|nhe:NECHADRAFT_92843|TATA-binding protein-associated factor [EC:3.6.4.-]
siRNA-t0020123	AGGCCCTGGGATTGTATGGT	20	48	FgHV1gp2	-
siRNA-t0020112	AGGTGACGACGCTATGTGGA	20	48	FGSG_13413	K15285|1|0.0|656|fgr:FG08241.1|solute carrier family 35, member E3
siRNA-t0020278	ATACAAACCGGAATGCACTTT	21	48	FgHV1gp2	-
siRNA-t0020420	ATGGAACAACGTGAGCCAGAA	21	48	FgHV1gp2	-
siRNA-t0020196	CCATGACGTACGACCCCGAA	20	48	FgHV1gp1	-
siRNA-t0020095	CCCTGGGATTGTATGGTAATTG	22	48	FgHV1gp2	-
siRNA-t0020383	CGAGCAGGATTGAAGTGTAA	20	48	FGSG_06885	K01613|1|5e-08|60.5|pte:PTT_10674|phosphatidylserine decarboxylase [EC:4.1.1.65]
siRNA-t0020250	CGCTGAAATCGAGGGTGTTGG	21	48	FgHV1gp2	-
siRNA-t0020131	GCATTCAGTAGTCGGGGTCT	20	48	FgHV1gp1	-
siRNA-t0020409	GGACCGACGTTTTGGCGAGACC	22	48	FgHV1gp2	-
siRNA-t0020174	GTGCTGATGATCATGACGTG	20	48	FGSG_12230	-
siRNA-t0020165	TCGTTACAATCGTGGACTTCCT	22	48	FgHV1gp2	-
siRNA-t0020377	TCTAGTTCGAAGGAGGCGGAA	21	48	FGSG_11206	-
siRNA-t0020516	AGCAGGATTGAAGTGTAATGCGTT	24	47	FgHV1gp2	-
siRNA-t0020653	AGGCCCTGGGATTGTATGGTAAT	23	47	FgHV1gp2	-
siRNA-t0020647	AGTTCTCTCTGACCTTGATGGCG	23	47	FGSG_11656	K00668|1|0.0|1439|nfi:NFIA_006490|fatty acid synthase subunit beta, fungi type [EC:2.3.1.86]
siRNA-t0020452	CCCAACACATTTTTGAAGATCGT	23	47	FgHV1gp2	-
siRNA-t0020607	GCTGAAATCGAGGGTGTTGGGG	22	47	FGSG_00677	K03097|1|0.0|653|fgr:FG00677.1|casein kinase II subunit alpha [EC:2.7.11.1]
siRNA-t0020778	GGACAAGCCGTCAAACATAGACACT	25	47	FgHV1gp2	-
siRNA-t0020637	TTGAATCGATCGTCATGGTGA	21	47	FgHV1gp2	-
siRNA-t0020904	AAGTGAAAGTGTCGTAGGAA	20	46	FgHV1gp1	-
siRNA-t0020962	ACACGCAGCAGAATTCTAGCC	21	46	FgHV1gp2	-
siRNA-t0021149	CCAAATCGTCATACATTGCA	20	46	FGSG_04859	K00777|1|2e-86|320|afm:AFUA_7G07100|[EC:2.4.2.-];K14963|2|8e-15|82.8|dha:DEHA2E05016g|COMPASS component SWD3;K06666|3|2e-14|81.6|pcs:Pc12g04590|glucose repression regulatory protein TUP1;K03361|5|5e-14|80.5|yli:YALI0A09658g|F-box and WD-40 domain protein CDC4
siRNA-t0020996	CCATCAGGACCCGAGAAAAACGCC	24	46	FgHV1gp2	-
siRNA-t0020936	GAAGTGACGATACTTGAACC	20	46	FGSG_13783	K01932|1|0.0|1526|afm:AFUA_5G12730|[EC:6.3.2.-]
siRNA-t0021088	GAGCTGGGGATGTTTGCTTGGTT	23	46	FgHV1gp2	-
siRNA-t0021002	GATAGGAGATGATGTTGGTTA	21	46	FGSG_11742	K00921|1|0.0|4415|fgr:FG00290.1|1-phosphatidylinositol-3-phosphate 5-kinase [EC:2.7.1.150]
siRNA-t0021003	GGTGACGACGCTATGTGGAG	20	46	FGSG_09921	K09240|1|2e-26|120|ppa:PAS_chr2-1_0757|transcriptional regulatory protein CAT8
siRNA-t0021188	GTCATGATCATCAGCACCGTACGGA	25	46	FgHV1gp2	-
siRNA-t0020979	TAATCAATGTACCGTTCGGCA	21	46	FgHV1gp2	-
siRNA-t0021396	CAACGGTTCTTGGAAAGTTG	20	45	FgHV1gp2	-
siRNA-t0021281	CATATGGATCGGTGGCACACGCA	23	45	FgHV1gp2	-
siRNA-t0021333	CCAGCTGTTCTCTGTCGGCATGTTG	25	45	FgHV1gp2	-
siRNA-t0021224	CGGAAAATTGTGTTGATTGCGCC	23	45	FgHV1gp2	-
siRNA-t0021210	CGGACTCATCCCACATGATGGTG	23	45	FgHV1gp2	-
siRNA-t0021277	GAGATCAACGTCAGAGGCCTC	21	45	FGSG_10040	K01262|1|0.0|1198|afm:AFUA_1G07720|Xaa-Pro aminopeptidase [EC:3.4.11.9]
siRNA-t0021518	GCTGAATGCTCTGCCTCGGTC	21	45	FgHV1gp2	-
siRNA-t0021488	GGGACCGACGTTTTGGCGAGA	21	45	FgHV1gp2	-
siRNA-t0021530	GGGAGACAGAGCCTGAGTCA	20	45	FGSG_02870	K08712|1|0.0|1038|pic:PICST_57529|ATP-binding cassette, subfamily G (WHITE), member 2, SNQ2;K08711|5|0.0|964|cne:CNB05770|ATP-binding cassette, subfamily G (WHITE), member 2, PDR
siRNA-t0021509	GGTGCTGGGTTCTGGTAGAAG	21	45	FGSG_07312	-
siRNA-t0021377	GTTCTCTTTGTTGGCCTGGT	20	45	FGSG_09560	-
siRNA-t0021297	TGCATGTACTGTTCGGCAACA	21	45	FGSG_04886	K00309|1|6e-94|343|afm:AFUA_6G08760|[EC:1.5.3.-];K00318|4|2e-35|149|pgu:PGUG_01270|proline dehydrogenase [EC:1.5.99.8]
siRNA-t0021533	TGGATCGGTGGCACACGCATAAG	23	45	FgHV1gp2	-
siRNA-t0021748	AACGGTTCTTGGAAAGTTGGA	21	44	FgHV1gp2	-
siRNA-t0021792	ACACGCAGCAGAATTCTAGCCTGGT	25	44	FgHV1gp2	-
siRNA-t0021901	AGGGTACGTGACATTAGGTGAA	22	44	FgHV1gp2	-
siRNA-t0021924	AGTGCTCATCGTTGTGCCAA	20	44	FGSG_13651	K00924|1|3e-72|271|afm:AFUA_2G15700|[EC:2.7.1.-];K00844|4|2e-26|119|tve:TRV_07869|hexokinase [EC:2.7.1.1]
siRNA-t0021766	ATGATGAGATCACGTTCGGA	20	44	FGSG_01870	K14563|1|2e-142|504|fgr:FG01870.1|rRNA 2'-O-methyltransferase fibrillarin [EC:2.1.1.-]
siRNA-t0021630	ATGATTTGCGACGTGCTTGAAGGCG	25	44	FgHV1gp2	-
siRNA-t0021685	CGTGAGTGCGTAGATAAAGGTTA	23	44	FgHV1gp2	-
siRNA-t0021632	CTTCGAACTAGATGGTGTGGCG	22	44	FgHV1gp2	-
siRNA-t0021975	GCTGAAATCGAGGGTGTTGGGGT	23	44	FgHV1gp2	-
siRNA-t0021720	GCTTGTCAGTGTCAGGATCCG	21	44	FGSG_06354	K08157|1|2e-60|232|nhe:NECHADRAFT_101392|MFS transporter, DHA1 family, multidrug resistance protein
siRNA-t0021811	GCTTGTCAGTGTCAGGATCCGTCC	24	44	FgHV1gp2	-
siRNA-t0021911	GGATGATATTCGGTATTGTCG	21	44	FgHV1gp2	-
siRNA-t0021800	GGGAACAATGAAGGCGTGTT	20	44	FgHV1gp2	-
siRNA-t0021727	GTAGTCATCGTCAGGGTACGT	21	44	FGSG_09801	K03164|1|0.0|2817|fgr:FG09801.1|DNA topoisomerase II [EC:5.99.1.3]

siRNA-t0021804	GTGCCATGACGTACGACCCCGA	22	44	FgHV1gp1	-
siRNA-t0021669	GTTCTCTGTCGGCATGTTGC	20	44	FGSG_11657	K09184|1|5e-13|75.9|bfu:BC1G_09852|GATA-binding protein, other eukaryote
siRNA-t0022432	AACGGGACCGACGTTTTGGCGA	22	43	FgHV1gp2	-
siRNA-t0022286	ACCTTGTTCAGGAATCACGACTTT	24	43	FgHV1gp2	-
siRNA-t0022403	ACGATAGTGGGAGCGAAAGCGAT	23	43	FGSG_08335	K02873|1|8e-89|325|fgr:FG08335.1|large subunit ribosomal protein L13e
siRNA-t0022465	AGCGTGACGAAGATCCCACT	20	43	FGSG_13783	K01932|1|0.0|1526|afm:AFUA_5G12730|[EC:6.3.2.-]
siRNA-t0022183	CCATAACCTCGTTGGGACCT	20	43	FGSG_10765	K10729|1|0.0|873|fgr:FG10765.1|DNA replication regulator SLD2
siRNA-t0022302	CTTGTTTCTCGTGGGATTGGGCG	23	43	FgHV1gp2	-
siRNA-t0022198	GACGTGGACGGGTGTGAAGC	20	43	FGSG_12200	K04718|1|0.0|682|fgr:FG02775.1|sphingosine kinase [EC:2.7.1.91]
siRNA-t0022474	GCAGATTCGAAATGGCTGAC	20	43	FGSG_04298	K15451|1|0.0|2019|fgr:FG04298.1|tRNA wybutosine-synthesizing protein 4 [EC:2.1.1.-]
siRNA-t0022199	GCATTCCGGACGGTGACCATT	21	43	FGSG_02632	K05349|1|0.0|1666|fgr:FG02632.1|beta-glucosidase [EC:3.2.1.21]
siRNA-t0022042	GGATGTTTGCTTGGTTTCGGGACGC	25	43	FgHV1gp2	-
siRNA-t0022399	TGATGTGGTGAGGTATGACGTGT	23	43	FgHV1gp2	-
siRNA-t0022187	TGGGACCATCATGATGAAACCGAA	24	43	FgHV1gp2	-
siRNA-t0022155	TGTTGCTTGATGGTTGGCACAA	22	43	FgHV1gp2	-
siRNA-t0022055	TTCGGGACGCGACAAGCAACAAT	23	43	FgHV1gp2	-
siRNA-t0022903	AAGAACCACAACGATAGTAT	20	42	FGSG_11856	K10846|1|0.0|1600|nhe:NECHADRAFT_65808|DNA excision repair protein ERCC-5
siRNA-t0022825	AGAGCGTGTAGGGCTGTCCCA	21	42	FGSG_05297	K14676|1|0.0|2814|fgr:FG05297.1|lysophospholipid hydrolase [EC:3.1.1.5]
siRNA-t0022615	AGGACCAGTTCCTGCTGAGTGT	22	42	FgHV1gp2	-
siRNA-t0022854	CCCGGCAGCGTTTATGGAACAA	22	42	FGSG_12361	-
siRNA-t0022881	CGACAATGGCGCATACACGGAACC	24	42	FgHV1gp2	-
siRNA-t0022808	CGGGACGCGACAAGCAACAATTT	23	42	FgHV1gp2	-
siRNA-t0022861	CGGTAGTCATCGGGAATCAT	20	42	FGSG_03388	-
siRNA-t0022862	GATCATCAGCACCGTACGGATCCA	24	42	FgHV1gp2	-
siRNA-t0022496	GGCGACACCGACGGTACGTGG	21	42	FgHV1gp2	-
siRNA-t0022883	GGGACCGACGTTTTGGCGAGAC	22	42	FgHV1gp2	-
siRNA-t0022490	GGTGGGTTCTTGAAGTTGGTG	21	42	FGSG_13018	-
siRNA-t0022754	GTGCTGATGATCATGACGTGG	21	42	FGSG_11121	-
siRNA-t0022555	GTGGTGAGGTATGACGTGTTC	21	42	FgHV1gp2	-
siRNA-t0022725	TGCAGCAACTGTCGCCTGGAC	21	42	FgHV1gp1	-
siRNA-t0022592	TGCGACGTGCTTGAAGGCGAA	21	42	FgHV1gp2	-
siRNA-t0023003	ACCTAGACCGAAGAAAGACCACAT	24	41	FgHV1gp2	-
siRNA-t0023100	ACTTGCTAGGACTGCCGGCC	20	41	FGSG_11372	K00777|1|5e-21|103|afm:AFUA_7G06290|[EC:2.4.2.-];K12823|4|1e-10|69.7|ure:UREG_02211|ATP-dependent RNA helicase DDX5/DBP2 [EC:3.6.4.13]
siRNA-t0022944	AGACCGAAGAAAGACCACATC	21	41	FGSG_09329	K08711|1|0.0|1646|cne:CNB05770|ATP-binding cassette, subfamily G (WHITE), member 2, PDR
siRNA-t0023201	AGAGTCTTGCGGTGTGGGTCTTCT	24	41	FgHV1gp2	-
siRNA-t0023046	AGGAACTCCACATAGCGTCGT	21	41	FgHV1gp2	-
siRNA-t0023092	AGGACCCGAGAAAAACGCCC	20	41	FGSG_01234	K01768|1|0.0|3295|fgr:FG01234.1|adenylate cyclase [EC:4.6.1.1]
siRNA-t0023150	ATCCAAACTGTTCATTTCGGCGA	23	41	FgHV1gp2	-
siRNA-t0023353	CGCAACCACAAGCCACTGCAGTT	23	41	FgHV1gp2	-
siRNA-t0023160	CTGATGTGGTGAGGTATGACGTGTT	25	41	FgHV1gp2	-
siRNA-t0022956	CTTGTCAGTGTCAGGATCCGTCC	23	41	FgHV1gp2	-
siRNA-t0023064	GAAGGCGAAGTATGAGGGAG	20	41	FgHV1gp2	-
siRNA-t0022912	GATTCAACGAAGGACATCACA	21	41	FGSG_04694	K15419|1|0.0|1075|ani:AN2547.2|emericellamide biosynthesis, highly reducing iterative type I polyketide synthase;K00680|3|0.0|812|afm:AFUA_3G14700|[EC:2.3.1.-]
siRNA-t0022926	GATTTGCGACGTGCTTGAAGGCG	23	41	FgHV1gp2	-
siRNA-t0023315	GCAGGATTGAAGTGTAATGCG	21	41	FgHV1gp2	-
siRNA-t0023016	GGCGCATCGGCTTACCCTCGC	21	41	FGSG_05873	-
siRNA-t0023191	GGTGCTGATGATCATGACGT	20	41	FGSG_12230	-
siRNA-t0023085	TCCAAACTGTTCATTTCGGCGA	22	41	FgHV1gp2	-
siRNA-t0023178	TCTGACGAATTTGGACAAGTAC	22	41	FgHV1gp2	-
siRNA-t0023133	TGGCCAAATCGTCATACATTGCA	23	41	FgHV1gp2	-
siRNA-t0023355	TGGGTTCTTGAAGTTGGTGCCG	22	41	FgHV1gp2	-
siRNA-t0022965	TTGAATCGATCGTCATGGTG	20	41	FGSG_05002	K12818|1|0.0|2187|fgr:FG05002.1|ATP-dependent RNA helicase DHX8/PRP22 [EC:3.6.4.13]
siRNA-t0023749	AAAATGCCATGGGATCGAAGGTT	23	40	FGSG_09064	-
siRNA-t0023835	AAAGAACCCATATGGATCGGTGGCA	25	40	FgHV1gp2	-
siRNA-t0023551	ACGGTTCTTGGAAAGTTGGA	20	40	FGSG_11505	-
siRNA-t0023798	ACTTGATCTCAGCACGCCGGT	21	40	FGSG_00633	K10592|1|0.0|5185|fgr:FG00633.1|E3 ubiquitin-protein ligase HUWE1 [EC:6.3.2.19]
siRNA-t0023466	AGAACGTAGATGTGATGCCT	20	40	FgHV1gp2	-
siRNA-t0023422	AGGCTCCTCCGACAATGGCGCATA	24	40	FgHV1gp2	-
siRNA-t0023486	AGGTTTGAACCCTGACAATACCAAA	25	40	FgHV1gp2	-
siRNA-t0023775	CAAGTAAGCAGAGCGTGTAGGGC	23	40	FgHV1gp2	-
siRNA-t0023792	CACTGGAAGAGAATGACGAT	20	40	FGSG_04197	-
siRNA-t0023849	CGATAGTGGGAGCGAAAGCGAT	22	40	FGSG_08335	K02873|1|8e-89|325|fgr:FG08335.1|large subunit ribosomal protein L13e
siRNA-t0023438	CTTGTCAGTGTCAGGATCCGTCCA	24	40	FgHV1gp2	-
siRNA-t0023591	CTTTCAGTAGGCTTCAAATT	20	40	FGSG_06066	-
siRNA-t0023556	GACGACCGTGCGAAATTGTTT	21	40	FgHV1gp2	-
siRNA-t0023554	GACGATAGTGGGAGCGAAAGCG	22	40	FGSG_08335	K02873|1|8e-89|325|fgr:FG08335.1|large subunit ribosomal protein L13e
siRNA-t0023563	GACGATGAATAGGCCTCCCA	20	40	FGSG_09707	K05658|1|4e-38|160|fgr:FG01684.1|ATP-binding cassette, subfamily B (MDR/TAP), member 1
siRNA-t0023602	GGGTCTTCTGACGAATTTGGAC	22	40	FgHV1gp2	-
siRNA-t0023720	GTATGAAAACTATAGTCGGT	20	40	FGSG_01536	-
siRNA-t0023662	GTTTCGGGACGCGACAAGCAA	21	40	FgHV1gp2	-
siRNA-t0023806	TGCCATGACGTACGACCCCGAA	22	40	FgHV1gp1	-
siRNA-t0023593	TGGATGCTGCGAAACCCAAT	20	40	FGSG_11848	-
siRNA-t0023698	TTCTCGTGGGATTGGGCGTGTGTT	24	40	FgHV1gp2	-
siRNA-t0023416	TTCTGCGGAGATCTCTAGGAT	21	40	FGSG_09806	-
siRNA-t0024178	AACAGAGCTGGGGATGTTTGC	21	39	FgHV1gp2	-
siRNA-t0024107	ACCTATACTTGATCTCAGCACGCC	24	39	FgHV1gp2	-
siRNA-t0024116	AGAGAAGAAGTCTGTTGCAT	20	39	FGSG_10617	K14859|1|2e-98|359|scm:SCHCODRAFT_255991|ribosome biogenesis protein SSF1/2;K00143|2|1e-14|82.0|zro:ZYRO0C16566g|L-aminoadipate-semialdehyde dehydrogenase [EC:1.2.1.31]
siRNA-t0023942	AGCGTGATGTGGTGTTATTT	20	39	FGSG_02315	K01932|1|0.0|2455|afm:AFUA_1G10380|[EC:6.3.2.-]
siRNA-t0024118	ATCCAAACTGTTCATTTCGGCG	22	39	FgHV1gp2	-
siRNA-t0023948	CCGAGAAAAACGCCCCTGGACGGT	24	39	FgHV1gp2	-
siRNA-t0023891	CCTGAGAGCATGAAGGCACAAT	22	39	FGSG_08778	K09595|1|0.0|1018|fgr:FG08778.1|minor histocompatibility antigen H13 [EC:3.4.23.-]
siRNA-t0024285	CTGACTGTAGTGTGCCTTGT	20	39	FGSG_11659	K01932|1|0.0|1431|afm:AFUA_5G12730|[EC:6.3.2.-]
siRNA-t0023988	CTTCAGGCCCTGGGATTGTATGGT	24	39	FgHV1gp2	-
siRNA-t0024257	GAATCGATCGTCATGGTGATA	21	39	FgHV1gp2	-
siRNA-t0024110	GCATGACTTTACAAGCTGGT	20	39	FgHV1gp2	-
siRNA-t0023921	GCTGCAAGATGGACTGGCCTGTT	23	39	FgHV1gp2	-
siRNA-t0024070	GTGTGTCGCGTCTACCTTGTT	21	39	FGSG_00721	K10084|1|2e-106|387|spo:SPAC23A1.04c|ER degradation enhancer, mannosidase alpha-like 1
siRNA-t0024592	AAGGGAGCCGGATATGTTTT	20	38	FGSG_11364	K15385|1|2e-33|143|ure:UREG_06498|aspyridone synthetase trans-acting enoyl reductase
siRNA-t0024814	AATACAGGACCGCGCGATGGCG	22	38	FgHV1gp2	-
siRNA-t0024356	ACAATTGTCGTGCGTTCCGT	20	38	FGSG_01049	-
siRNA-t0024872	AGAAGAATGAGGGAAGAAAGGGAAC	25	38	FGSG_01483	-

siRNA-t0024416	AGCAGAATTCTAGCCTGGTT	20	38	FgHV1gp2	-
siRNA-t0024546	ATACTTGATCTCAGCACGCCGGT	23	38	FgHV1gp2	-
siRNA-t0024460	ATGTGGTGAGGTATGACGTGTTC	23	38	FgHV1gp2	-
siRNA-t0024836	CAGAGCGTGTAGGGCTGTCCC	21	38	FgHV1gp2	-
siRNA-t0024691	CAGAGCTGGGGATGTTTGCTTGGTT	25	38	FgHV1gp2	-
siRNA-t0024593	CGATTCAACGAAGGACATCAC	21	38	FGSG_10327	K00698|1|0.0|1847|fgr:FG10327.1|chitin synthase [EC:2.4.1.16]
siRNA-t0024497	CGGTACGTGGAAATCTTCAGGCC	23	38	FgHV1gp2	-
siRNA-t0024748	CGTAGTCATCGTCAGGGTAC	20	38	FGSG_09801	K03164|1|0.0|2817|fgr:FG09801.1|DNA topoisomerase II [EC:5.99.1.3]
siRNA-t0024806	GAAATGATTTGCGACGTGCTT	21	38	FgHV1gp2	-
siRNA-t0024666	GAAGAAGTCTGTTGCATTGCT	21	38	FGSG_08645	K03681|1|4e-120|429|fgr:FG08645.1|exosome complex component RRP40
siRNA-t0024477	GATGATATTCGGTATTGTCGA	21	38	FgHV1gp2	-
siRNA-t0024643	GCTCGATTCTGGCGGCGTCA	20	38	FGSG_01910	K06636|1|0.0|2339|fgr:FG01910.1|structural maintenance of chromosome 1
siRNA-t0024733	GGAATGACGTTTGTAAAGACCACTG	25	38	FgHV1gp2	-
siRNA-t0024532	GTTGAATCGATCGTCATGGTG	21	38	FgHV1gp2	-
siRNA-t0024568	TCTGTGAATTGGCCCTGTTG	21	38	FGSG_00633	K10592|1|0.0|5185|fgr:FG00633.1|E3 ubiquitin-protein ligase HUWE1 [EC:6.3.2.19]
siRNA-t0024764	TGATCATCAGCACCGTACGGATCCA	25	38	FgHV1gp2	-
siRNA-t0024563	TGTGGTGAGGTATGACGTGTTCAT	24	38	FgHV1gp2	-
siRNA-t0025064	AACCTATACTTGATCTCAGCACGCC	25	37	FgHV1gp2	-
siRNA-t0025010	ACTTGTTTCTCGTGGGATTGGGCG	24	37	FgHV1gp2	-
siRNA-t0025168	AGAACGCTGAAATCGAGGGTG	21	37	FgHV1gp2	-
siRNA-t0024919	AGCTGTTCTCTGTCGGCATGTTG	23	37	FgHV1gp2	-
siRNA-t0024992	AGGTGAGAAGATTGAGTTCATT	22	37	FgHV1gp2	-
siRNA-t0025321	CAGGACCCGAGAAAAACGCC	20	37	FGSG_00307	-
siRNA-t0025449	CCAGTTCTCTTTGTTGGCCTG	21	37	FGSG_04690	-
siRNA-t0024950	CCATATGGATCGGTGGCACAC	21	37	FgHV1gp2	-
siRNA-t0025450	GACCAGCTGTTCTCTGTCGGCATG	24	37	FgHV1gp2	-
siRNA-t0025142	GGCTTGTCGTCGGGAGAATCCAG	23	37	FgHV1gp2	-
siRNA-t0025203	GGGACGCGACAAGCAACAATTTG	23	37	FgHV1gp2	-
siRNA-t0025193	TGCCATCCTATAGATCGCGCGT	22	37	FgHV1gp2	-
siRNA-t0025169	TGGCGCATACACGGAACCCATTG	23	37	FgHV1gp2	-
siRNA-t0025172	TGGTTTGTGAAATTTATGGCAT	22	37	FgHV1gp2	-
siRNA-t0025299	TGTAGGAGTGGCAGTGTAATGGAG	24	37	FgHV1gp2	-
siRNA-t0025303	TGTTGACGTCACCCTTTGGA	20	37	FGSG_13865	K00128|1|1e-104|379|ncr:NCU03415|aldehyde dehydrogenase (NAD+) [EC:1.2.1.3]
siRNA-t0025786	AACGGGTCCAGATGTTGTGCTT	22	36	FGSG_07939	K05236|1|0.0|2545|fgr:FG07939.1|coatomer protein complex, subunit alpha (xenin)
siRNA-t0025536	ACCAGAACCCAGCACCTTTCCCT	23	36	FgHV1gp2	-
siRNA-t0026012	ACCGATGGAAGTGATGGCAAT	21	36	FGSG_08209	K01932|1|0.0|1305|afm:AFUA_5G12730|[EC:6.3.2.-]
siRNA-t0025557	ACTTGATCTCAGCACGCCGGTT	22	36	FGSG_00633	K10592|1|0.0|5185|fgr:FG00633.1|E3 ubiquitin-protein ligase HUWE1 [EC:6.3.2.19]
siRNA-t0025471	CCAAGTAAGCAGAGCGTGTAGGGC	24	36	FgHV1gp2	-
siRNA-t0025884	CCTGTTGTTGGTCTTCCAGGCA	22	36	FgHV1gp2	-
siRNA-t0025891	CTAGACCGAAGAAAGACCACATCCA	25	36	FgHV1gp2	-
siRNA-t0025676	CTCGGGTCCTGATGGTTTCAG	21	36	FGSG_06016	K05727|1|1e-46|188|mpr:MPER_07660|dedicator of cytokinesis 3/4/5
siRNA-t0025858	GACAATTGTCGTGCGTTCCGTG	22	36	FGSG_01049	-
siRNA-t0025585	GACGATAGTGGGAGCGAAAGCGA	23	36	FGSG_08335	K02873|1|8e-89|325|fgr:FG08335.1|large subunit ribosomal protein L13e
siRNA-t0025929	GCCTATTCATCGTCAATGACC	21	36	FGSG_08065	K00100|1|1e-113|408|afm:AFUA_7G04850|[EC:1.1.1.-]
siRNA-t0025830	GTTCAAGTATCGTCACTTCT	20	36	FGSG_05959	-
siRNA-t0025509	TCGAAACGGATGGTGTAGAA	20	36	FGSG_01433	K00264|1|0.0|4368|fgr:FG01433.1|glutamate synthase (NADPH/NADH) [EC:1.4.1.13 1.4.1.14]
siRNA-t0025494	TCGTTGAATCGATCGTCATGGTGAT	25	36	FgHV1gp2	-
siRNA-t0025694	TGCTGCAAGATGGACTGGCCTGT	23	36	FgHV1gp2	-
siRNA-t0026351	AACGTCTGACTGTAGTGTGCCTT	23	35	FgHV1gp2	-
siRNA-t0026312	AATGATTTGCGACGTGCTTG	20	35	FGSG_02169	-
siRNA-t0026480	ACAGAGCTGGGGATGTTTGC	20	35	FgHV1gp2	-
siRNA-t0026461	ACGGCGGCATTCATGAACTG	20	35	FGSG_03062	-
siRNA-t0026318	AGACATTTTCGCGCGCTGTA	20	35	FGSG_02394	K01932|1|0.0|942|afm:AFUA_1G10380|[EC:6.3.2.-]
siRNA-t0026341	AGCAGGATTGAAGTGTAATGCG	22	35	FgHV1gp2	-
siRNA-t0026149	CAGAGTCTTGCGGTGTGGGTCTT	23	35	FgHV1gp2	-
siRNA-t0026166	CGACCCATGCTTTCGACGTACTT	23	35	FgHV1gp2	-
siRNA-t0026592	CGGGTCCAGATGTTGTGCTTT	21	35	FgHV1gp2	-
siRNA-t0026500	CTGAGAGCATGAAGGCACAA	20	35	FGSG_08778	K09595|1|0.0|1018|fgr:FG08778.1|minor histocompatibility antigen H13 [EC:3.4.23.-]
siRNA-t0026566	GAACCTGATCCCGGAATTAT	20	35	FGSG_10966	K11702|1|1e-47|190|cne:CNL04750|beta-1,2-xylosyltransferase [EC:2.4.2.-]
siRNA-t0026616	GAGCAGGATTGAAGTGTAATG	21	35	FgHV1gp2	-
siRNA-t0026173	GATACAAACCGGAATGCACT	20	35	FgHV1gp2	-
siRNA-t0026491	GATCCACCCGGATGACTTGGCTC	23	35	FgHV1gp2	-
siRNA-t0026423	GGATACGTTCTGAGAAGTCTCCG	23	35	FgHV1gp2	-
siRNA-t0026245	GGGACCATCATGATGAAACCGAA	23	35	FgHV1gp2	-
siRNA-t0026164	GGTTCAATATCGGTACCAGAC	21	35	FGSG_08426	K12659|1|0.0|960|fgr:FG08426.1|N-acetyl-gamma-glutamyl-phosphate reductase / acetylglutamate kinase [EC:1.2.1.38 2.7.2.8]
siRNA-t0026302	GTCCTTCGTTGAATCGATCGTCAT	24	35	FgHV1gp2	-
siRNA-t0026196	TGGACGGGTGTGAAGCTTGGATG	23	35	FGSG_00840	K00624|1|0.0|1728|fgr:FG00840.1|carnitine O-acetyltransferase [EC:2.3.1.7]
siRNA-t0026439	TGGGCGTGTGTTCTTTATGGGCT	23	35	FGSG_05370	K09240|1|8e-06|52.0|cdu:CD36_07720|transcriptional regulatory protein CAT8
siRNA-t0026441	TGGTTCAAGTATCGTCACTTCT	22	35	FgHV1gp2	-
siRNA-t0027144	ACGATAGTGGGAGCGAAAGC	20	34	FGSG_08335	K02873|1|8e-89|325|fgr:FG08335.1|large subunit ribosomal protein L13e
siRNA-t0027211	ACTGTTGACGTCACCCTTTGGA	22	34	FgHV1gp2	-
siRNA-t0026810	AGGAGTGCAGATGAACAATG	20	34	FGSG_01442	K06867|1|1e-28|129|kla:KLLA0E17953g|
siRNA-t0026879	AGGCGTGTCTGAATGGAATA	20	34	FGSG_13878	K01932|1|0.0|1450|afm:AFUA_5G12730|[EC:6.3.2.-]
siRNA-t0027151	AGTGTTCGCATCCGTTCTGT	20	34	FGSG_08999	-
siRNA-t0026997	ATGAACGTCTGACTGTAGTGTGCC	24	34	FgHV1gp2	-
siRNA-t0027113	CAGGCCCTGGGATTGTATGGTAAT	24	34	FgHV1gp2	-
siRNA-t0026754	CGCTCCCACTATCGTCCTGCGT	22	34	FGSG_01294	K00799|1|1e-34|146|afv:AFLA_022030|glutathione S-transferase [EC:2.5.1.18]
siRNA-t0027254	CGGAATGCGTTCACACGATTCC	22	34	FgHV1gp2	-
siRNA-t0027241	CGGGACCGACGTTTTGGCGAGACC	24	34	FgHV1gp2	-
siRNA-t0027137	CGTCATGATCATCAGCACCGT	21	34	FGSG_11998	-
siRNA-t0026686	CTTGCGGTGTGGGTCTTCTGAC	22	34	FgHV1gp2	-
siRNA-t0027014	CTTGGTTTCGGGACGCGACAAGCA	24	34	FgHV1gp2	-
siRNA-t0027159	GAATCATGTCGGTGAACTGTT	21	34	FGSG_07266	K00643|1|0.0|1118|nhe:NECHADRAFT_68614|5-aminolevulinate synthase [EC:2.3.1.37]
siRNA-t0027160	GCATGGCCGGCAGTCCTAGCA	21	34	FgHV1gp2	-
siRNA-t0027193	GGGTTCTTGAAGTTGGTGCCGA	22	34	FgHV1gp2	-
siRNA-t0026766	GGTCGTACGTCATGGCACTC	20	34	FGSG_10228	K03114|1|0.0|934|fgr:FG10228.1|mitosis inhibitor protein kinase SWE1 [EC:2.7.11.1]
siRNA-t0027069	GTTATAAGGCTTGTCGTCGGGA	22	34	FGSG_08614	K01874|1|0.0|1256|fgr:FG08614.1|methionyl-tRNA synthetase [EC:6.1.1.10]
siRNA-t0027171	GTTGTATGAAGCTGATGGAATGCT	24	34	FgHV1gp2	-
siRNA-t0026716	TGATACAAACCGGAATGCACTTT	23	34	FgHV1gp2	-
siRNA-t0027031	TGCACGAGGGACGAGCTTGG	20	34	FgHV1gp2	-
siRNA-t0027083	TGGGGTCTTGACTTGGGCTGTA	22	34	FGSG_09304	-
siRNA-t0026807	TTGTGCTGCAAGATGGACTGGCCT	24	34	FgHV1gp2	-
siRNA-t0027361	AACGGGTCCAGATGTTGTGCT	21	33	FGSG_07939	K05236|1|0.0|2545|fgr:FG07939.1|coatomer protein complex, subunit alpha (xenin)

siRNA-t0027955	ACGGGTCCAGATGTTGTGCT	20	33	FGSG_07939	K05236|1|0.0|2545|fgr:FG07939.1|coatomer protein complex, subunit alpha (xenin)
siRNA-t0027869	AGAACGTAGATGTGATGCCTTGTC	24	33	FgHV1gp2	-
siRNA-t0027756	AGCGTGACGAAGATCCCACTC	21	33	FGSG_13783	K01932|1|0.0|1526|afm:AFUA_5G12730|[EC:6.3.2.-]
siRNA-t0027738	ATCGATCGTCATGGTGATACCCA	23	33	FgHV1gp2	-
siRNA-t0027854	ATGTGGTGAGGTATGACGTGTTCAT	25	33	FgHV1gp2	-
siRNA-t0027579	CTTGTTCTGGAACGTCAAATC	21	33	FGSG_06754	K06669|1|0.0|2432|fgr:FG06754.1|structural maintenance of chromosome 3 (chondroitin sulfate proteoglycan 6)
siRNA-t0027625	GAAAAACGCCCCTGGACGGTA	21	33	FgHV1gp2	-
siRNA-t0027539	GACGGTACGTGGAAATCTTCA	21	33	FgHV1gp2	-
siRNA-t0027483	GCAGCAACTGTCGCCTGGACA	21	33	FGSG_10995	K05658|1|4e-24|113|yli:YALI0F20174g|ATP-binding cassette, subfamily B (MDR/TAP), member 1;K05663|4|2e-23|111|bfu:BC1G_07740|mitochondrial ABC transporter ATM
siRNA-t0027904	GCGCATCGGCTTACCCTCGCA	21	33	FGSG_05873	-
siRNA-t0027466	GGGACGTCTTCGATGGATGCTGCGA	25	33	FgHV1gp2	-
siRNA-t0027927	TCTAGTTCGAAGGAGGCGGA	20	33	FGSG_11206	-
siRNA-t0027746	TGGAAATCTTCAGGCCCTGGG	21	33	FGSG_09607	K10413|1|0.0|8782|fgr:FG09607.1|dynein heavy chain 1, cytosolic
siRNA-t0027762	TGGTGGTTGGCGGCATTGTA	20	33	FGSG_00950	K08486|1|0.0|650|fgr:FG00950.1|syntaxin 1B/2/3
siRNA-t0028432	ACGGGACCGACGTTTTGGCG	20	32	FgHV1gp2	-
siRNA-t0027983	ATATGGATCGGTGGCACACGCAT	23	32	FgHV1gp2	-
siRNA-t0028303	CAAGGAGCTCAAAGGTATGAT	21	32	FGSG_11489	-
siRNA-t0028071	CACGCAGCAGAATTCTAGCCTGGTT	25	32	FgHV1gp2	-
siRNA-t0028107	CCAGATGCGAAGCGAATATCGGA	23	32	FGSG_03912	K09246|1|3e-07|55.8|lth:KLTH0E16500g|arginine metabolism regulation protein II
siRNA-t0028221	CGGTAGTCATCGGGAATCATGTC	23	32	FgHV1gp2	-
siRNA-t0028113	GCAGACACTTGTTTCTCGTGGGATT	25	32	FgHV1gp2	-
siRNA-t0027965	GCATAACACGCAGCAGAATTCTAG	24	32	FgHV1gp2	-
siRNA-t0027976	GGAGACTAAACGCTTTGGGCAC	22	32	FgHV1gp1	-
siRNA-t0028128	GGCGACACCGACGGTACGTGGAAA	24	32	FgHV1gp2	-
siRNA-t0028541	GGGACGCGACAAGCAACAAT	20	32	FgHV1gp2	-
siRNA-t0028585	GGGATTGGGCGTGTGTTCTTTATG	24	32	FgHV1gp2	-
siRNA-t0028334	GTGAAAGTGTCGTAGGAAAGGAAAA	25	32	FgHV1gp1	-
siRNA-t0028544	TACTTGATCTCAGCACGCCGG	21	32	FGSG_00633	K10592|1|0.0|5185|fgr:FG00633.1|E3 ubiquitin-protein ligase HUWE1 [EC:6.3.2.19]
siRNA-t0028389	TCTGGGGTCTGACCGAGGCA	20	32	FGSG_04361	K11373|1|0.0|2514|fgr:FG04361.1|elongator complex protein 1
siRNA-t0028307	TGCCATCCTATAGATCGCGCGTA	23	32	FgHV1gp2	-
siRNA-t0028644	TGCTGAATGCTCTGCCTCGGTCA	23	32	FgHV1gp2	-
siRNA-t0028163	TGCTGATGATCATGACGTGGAG	22	32	FGSG_11121	-
siRNA-t0028289	TGCTGTAGTGTCTGAAGATCTCAGA	25	32	FgHV1gp2	-
siRNA-t0028189	TGGAAATCATCGGTCTCGCC	20	32	FGSG_08343	K01535|1|0.0|1497|nhe:NECHADRAFT_77500|H+-transporting ATPase [EC:3.6.3.6]
siRNA-t0029005	AATCATCATCGTAGTCATCGTCA	23	31	FGSG_09900	-
siRNA-t0029088	ACAGAGCTGGGGATGTTTGCTTGGT	25	31	FgHV1gp2	-
siRNA-t0028791	AGAGCTGGGGATGTTTGCTTG	21	31	FgHV1gp2	-
siRNA-t0028775	AGCAGACACTTGTTTCTCGT	20	31	FGSG_11036	K09252|1|5e-06|50.4|afm:AFUA_7G02380|feruloyl esterase [EC:3.1.1.73]
siRNA-t0029327	AGGCTTGTCGTCGGGAGAATC	21	31	FGSG_06305	K01230|1|0.0|1876|fgr:FG06305.1|mannosyl-oligosaccharide alpha-1,2-mannosidase [EC:3.2.1.113]
siRNA-t0028733	AGGGTTGTTGAGAACGCTGA	20	31	FGSG_06772	-
siRNA-t0029013	CAATGGCGCATACACGGAACCCA	23	31	FgHV1gp2	-
siRNA-t0029014	CCATAACCTCGTTGGGACCTTTC	23	31	FgHV1gp2	-
siRNA-t0029313	CGGTACGTGGAAATCTTCAGGCCC	24	31	FgHV1gp2	-
siRNA-t0029207	GAACGTAGATGTGATGCCTTGT	22	31	FgHV1gp2	-
siRNA-t0028935	GAGTGCTCATCGTTGTGCCAA	21	31	FGSG_04332	-
siRNA-t0028790	GCAGAGAAGAAGTCTGTTGCAT	22	31	FGSG_10617	K14859|1|2e-98|359|scm:SCHCODRAFT_255991|ribosome biogenesis protein SSF1/2;K00143|2|1e-14|82.0|zro:ZYRO0C16566g|L-aminoadipate-semialdehyde dehydrogenase [EC:1.2.1.31]
siRNA-t0029134	GCCATTCGTTACAATCGTGGACT	23	31	FgHV1gp2	-
siRNA-t0029288	GCGACGACCGTGCGAAATTGT	21	31	FgHV1gp2	-
siRNA-t0029197	GCTTGAAGGCGAAGTATGAGGGAGA	25	31	FgHV1gp2	-
siRNA-t0029289	GCTTGTCAGTGTCAGGATCCGTC	23	31	FgHV1gp2	-
siRNA-t0029016	GTGATACAAACCGGAATGCACTTT	24	31	FgHV1gp2	-
siRNA-t0028711	GTGGAGAAGGACAAGAAAACT	21	31	FGSG_06580	K11262|1|0.0|4510|fgr:FG06580.1|acetyl-CoA carboxylase / biotin carboxylase [EC:6.4.1.2 6.3.4.14]
siRNA-t0029155	TCTGACTGTTGCTTGATGGTTGGCA	25	31	FgHV1gp2	-
siRNA-t0028933	TGCTGGGTTCTGGTAGAAGG	20	31	FGSG_07312	-
siRNA-t0029367	TGTACCGTTCGGCAACAGATCT	22	31	FgHV1gp2	-
siRNA-t0029722	AACAATTTATCGTGCGCTTCTG	22	30	FgHV1gp2	-
siRNA-t0029807	AAGTATCGTCACTTCTTCGTA	21	30	FGSG_09917	K01115|1|0.0|3466|fgr:FG09917.1|phospholipase D [EC:3.1.4.4]
siRNA-t0029623	AGCTGCAGCAACTGTCGCCT	20	30	FGSG_12712	K10884|1|0.0|1132|fgr:FG05317.1|ATP-dependent DNA helicase 2 subunit 1
siRNA-t0029434	AGGCGCATCGGCTTACCCTCGC	22	30	FGSG_05873	-
siRNA-t0029507	ATCCACCCGGATGACTTGGCTC	22	30	FgHV1gp2	-
siRNA-t0030005	CAGGATCTCTGACATATTCGGA	22	30	FgHV1gp2	-
siRNA-t0029891	CCAGAACCCAGCACCTTTCCC	21	30	FgHV1gp2	-
siRNA-t0029637	CGCAGCAGAATTCTAGCCTGGT	22	30	FgHV1gp2	-
siRNA-t0029956	CGGTAGTCATCGGGAATCATGTCGG	25	30	FgHV1gp2	-
siRNA-t0030107	CGTGAACTGCTGCGCTATGGTG	22	30	FgHV1gp2	-
siRNA-t0029795	CTGTTGACGTCACCCTTTGGA	21	30	FgHV1gp2	-
siRNA-t0029920	GACGGTACGTGGAAATCTTCAGGC	24	30	FgHV1gp2	-
siRNA-t0030056	GAGAAAAACGCCCCTGGACGGTA	23	30	FgHV1gp2	-
siRNA-t0029997	GCGACAGTTGCTGCAGCTGGT	21	30	FGSG_05306	K08333|1|0.0|3035|fgr:FG05306.1|phosphoinositide-3-kinase, regulatory subunit 4, p150 [EC:2.7.11.1]
siRNA-t0029511	GCTTGTCAGTGTCAGGATCCGTCCA	25	30	FgHV1gp2	-
siRNA-t0029882	GGCCCAGTCATGAATTTTCGGT	22	30	FgHV1gp2	-
siRNA-t0029477	GGGACGCGACAAGCAACAATT	21	30	FgHV1gp2	-
siRNA-t0029846	GGTCTTCGACGGGTACGATGT	21	30	FGSG_07258	K14572|1|0.0|8396|fgr:FG07258.1|midasin
siRNA-t0029835	GGTTCAAGTATCGTCACTTCTTCG	24	30	FgHV1gp2	-
siRNA-t0030101	GGTTCAAGTATCGTCACTTCTTCGT	25	30	FgHV1gp2	-
siRNA-t0029805	GGTTGGCGGCATTGTATGGTG	21	30	FGSG_05152	K04648|1|0.0|2319|fgr:FG05152.1|dynactin 1
siRNA-t0029599	GTTGGCGGCATTGTATGGTG	20	30	FGSG_13829	K00456|1|4e-65|246|aor:AOR_1_1144084|cysteine dioxygenase [EC:1.13.11.20]
siRNA-t0029389	GTTGTCACCTTCGGTACCAA	20	30	FGSG_10089	-
siRNA-t0029900	TCGGTAGTCATCGGGAATCATGT	23	30	FgHV1gp2	-
siRNA-t0030039	TCGTAGTCATCGTCAGGGTA	20	30	FGSG_10269	K01509|1|0.0|1066|pic:PICST_30157|adenosinetriphosphatase [EC:3.6.1.3];K11367|3|5e-128|458|cal:CaO19.10553|chromodomain-helicase-DNA-binding protein 1 [EC:3.6.4.12];K11786|5|1e-127|457|ure:UREG_04333|ATP-dependent helicase STH1/SNF2 [EC:3.6.4.-]
siRNA-t0029652	TCGTTACAATCGTGGACTTCC	21	30	FgHV1gp2	-
siRNA-t0029503	TGAGGATTTGGCCGTCTGGA	20	30	FGSG_01321	K05760|1|1e-21|104|mgl:MGL_4132|paxillin
siRNA-t0030667	AAGGATTTGTAAGGGGAAAC	20	29	FGSG_12660	-
siRNA-t0030547	ACCTATACTTGATCTCAGCACGC	23	29	FgHV1gp2	-
siRNA-t0030688	AGAACGTAGATGTGATGCCTTGTCT	25	29	FgHV1gp2	-
siRNA-t0030401	AGCTGGGGATGTTTGCTTGGTTTC	24	29	FgHV1gp2	-
siRNA-t0030755	AGGGATACCAACTTGATTTGAC	22	29	FGSG_11680	K06694|1|1e-13|77.8|clu:CLUG_01956|26S proteasome non-ATPase regulatory subunit 10
siRNA-t0030387	AGTTCTCTTTGTTGGCCTGGT	21	29	FGSG_09560	-
siRNA-t0030150	ATACTTGATCTCAGCACGCCGGTT	24	29	FgHV1gp2	-
siRNA-t0030708	ATATGGATCGGTGGCACACGCATA	24	29	FgHV1gp2	-

siRNA-t0030351	CAATCACACGCGGAACTGTC	20	29	FGSG_02482	K00381|1|0.0|3097|fgr:FG02482.1|sulfite reductase (NADPH) hemoprotein beta-component [EC:1.8.1.2]
siRNA-t0030491	CCAGTTGGGGTCTTGACTTGG	21	29	FGSG_01977	-
siRNA-t0030819	CGGAACGCACGACAATTGTC	20	29	FGSG_07466	K14951|1|0.0|2603|fgr:FG07466.1|cation-transporting ATPase 13A3/4/5 [EC:3.6.3.-]
siRNA-t0030849	CGGGACCGACGTTTTGGCGAG	21	29	FgHV1gp2	-
siRNA-t0030314	CGGTAGTCATCGGGAATCATG	21	29	FgHV1gp2	-
siRNA-t0030529	CGTTGAATCGATCGTCATGGTGAT	24	29	FgHV1gp2	-
siRNA-t0030605	CTGACTGTTGCTTGATGGTTGGC	23	29	FgHV1gp2	-
siRNA-t0030234	GAAATGATTTGCGACGTGCTTGAA	24	29	FgHV1gp2	-
siRNA-t0030839	GAAGAGACAGAGCTCGCGCCTTCCA	25	29	FgHV1gp2	-
siRNA-t0030282	GACAAGGTCGAGTGGCCCGAA	21	29	FGSG_12193	K12622|1|4e-42|168|nhe:NECHADRAFT_61990|U6 snRNA-associated Sm-like protein LSm3
siRNA-t0030723	GACGACCGTGCGAAATTGTT	20	29	FgHV1gp2	-
siRNA-t0030815	GATAAATGAACGTCTGACTGTA	22	29	FgHV1gp2	-
siRNA-t0030953	GCAGGATCTCTGACATATTCGGACT	25	29	FgHV1gp2	-
siRNA-t0030452	GCGAGACCGATGATTTCCAT	20	29	FGSG_12863	K02324|1|0.0|3986|fgr:FG06065.1|DNA polymerase epsilon subunit 1 [EC:2.7.7.7]
siRNA-t0030343	GCTGCATGTACTGTTCGGCA	20	29	FGSG_08421	K13126|1|0.0|636|fgr:FG08421.1|polyadenylate-binding protein
siRNA-t0030182	GCTGCCGCGGATAACCTATACTT	23	29	FgHV1gp2	-
siRNA-t0030943	GCTTGTCAGTGTCAGGATCCGT	22	29	FgHV1gp2	-
siRNA-t0030381	GGCATGAACTCGGTCAACGAAA	22	29	FgHV1gp2	-
siRNA-t0030718	GGTGAGAAGATTGAGTTCATT	21	29	FgHV1gp2	-
siRNA-t0030575	GTTTCTCGTGGGATTGGGCGTG	22	29	FgHV1gp2	-
siRNA-t0030615	TCTGTGAATTGGGCCCTGTTGT	22	29	FGSG_00633	K10592|1|0.0|5185|fgr:FG00633.1|E3 ubiquitin-protein ligase HUWE1 [EC:6.3.2.19]
siRNA-t0030710	TGATGATCATGACGTGGAGAA	21	29	FGSG_02870	K08712|1|0.0|1038|pic:PICST_57529|ATP-binding cassette, subfamily G (WHITE), member 2, SNQ2;K08711|5|0.0|964|cne:CNB05770|ATP-binding cassette, subfamily G (WHITE), member 2, PDR
siRNA-t0030882	TGGCGAGACCGATGATTTCCA	21	29	FGSG_08533	-
siRNA-t0030592	TGGCGCATACACGGAACCCATT	22	29	FgHV1gp2	-
siRNA-t0030295	TGGTGAGGTATGACGTGTTCA	21	29	FgHV1gp2	-
siRNA-t0030763	TGGTTGGCGGCATTGTATGGTG	22	29	FGSG_05152	K04648|1|0.0|2319|fgr:FG05152.1|dynactin 1
siRNA-t0030706	TTAACCATGGATCTGATTAT	20	29	FGSG_07843	-
siRNA-t0030721	TTGCTGCATGTACTGTTCGGCA	22	29	FGSG_01434	-
siRNA-t0031666	AACGGGTCCAGATGTTGTGCTTT	23	28	FgHV1gp2	-
siRNA-t0031417	AACTGGACAGAAGTTTTGTGG	21	28	FGSG_08662	K06657|1|7e-08|58.5|lel:LELG_00705|G1/S-specific cyclin PLC2
siRNA-t0031230	AAGGCGTGTCTGAATGGAATA	21	28	FGSG_13878	K01932|1|0.0|1450|afm:AFUA_5G12730|[EC:6.3.2.-]
siRNA-t0031849	AATCGATCGTCATGGTGATACCCA	24	28	FgHV1gp2	-
siRNA-t0031024	ACTTCCATCGGTCGAAACGG	20	28	FGSG_09575	-
siRNA-t0031775	AGCAGGATCTCGTAAGCTGC	20	28	FGSG_09857	K09506|1|0.0|927|fgr:FG09857.1|DnaJ homolog subfamily A member 5
siRNA-t0031671	AGGCTCCTCCGACAATGGCGCAT	23	28	FgHV1gp2	-
siRNA-t0031127	ATAACACGCAGCAGAATTCT	20	28	FgHV1gp2	-
siRNA-t0031576	CCCTGGACGGTACTTCTGCT	20	28	FGSG_05337	K09291|1|0.0|3327|fgr:FG05337.1|nucleoprotein TPR
siRNA-t0031300	CGGATACGTTCTGAGAAGTCTCC	23	28	FgHV1gp2	-
siRNA-t0031139	GAAACAAGTGTCTGCTGGAG	20	28	FGSG_06925	K03857|1|8e-22|106|uma:UM05234.1|phosphatidylinositol glycan, class A [EC:2.4.1.198]
siRNA-t0031461	GCCATCCTATAGATCGCGCGTA	22	28	FgHV1gp2	-
siRNA-t0031219	GCCATTCGTTACAATCGTGGACTTC	25	28	FgHV1gp2	-
siRNA-t0031231	GGTGAGAAGATTGAGTTCATTTGT	24	28	FgHV1gp2	-
siRNA-t0031509	GGTGTCGCCATTGATGGGATA	21	28	FGSG_12445	K01175|1|2e-12|73.2|afm:AFUA_4G12720|[EC:3.1.-.-]
siRNA-t0031337	GGTTCACGAGTGGGATGGGT	20	28	FGSG_08874	-
siRNA-t0031342	TCGGACTCATCCCACATGATGGTG	24	28	FgHV1gp2	-
siRNA-t0031213	TCTGTGAATTGGGCCCTGTT	20	28	FGSG_00633	K10592|1|0.0|5185|fgr:FG00633.1|E3 ubiquitin-protein ligase HUWE1 [EC:6.3.2.19]
siRNA-t0031505	TGCGGAGATCTCTAGGATGA	20	28	FGSG_09806	-
siRNA-t0031120	TGCTGATGATCATGACGTGG	20	28	FGSG_11121	-
siRNA-t0031118	TGGATGCTGCGAAACCCAATT	21	28	FgHV1gp2	-
siRNA-t0032424	AACTGTTCATTTCGGCGAGTCT	22	27	FgHV1gp2	-
siRNA-t0032038	AATGTACCGTTCGGCAACAGA	21	27	FGSG_07974	K05989|1|0.0|1786|fgr:FG07974.1|alpha-L-rhamnosidase [EC:3.2.1.40]
siRNA-t0032569	AGACACTTGTTTCTCGTGGGATT	23	27	FGSG_07509	K08157|1|4e-46|185|kla:KLLA0F18106g|MFS transporter, DHA1 family, multidrug resistance protein
siRNA-t0032242	ATAGATGGTGATTGTGGTCAT	21	27	FGSG_10402	-
siRNA-t0031867	CAAACATAGACACTTCAACAT	21	27	FgHV1gp2	-
siRNA-t0032722	CCCACTCATGGGCAAAAGACAGAT	24	27	FgHV1gp2	-
siRNA-t0032538	CGACATGTTTAGGATCACCA	20	27	FGSG_03933	K12618|1|1e-55|215|ani:AN8185.2|5'-3' exoribonuclease 1 [EC:3.1.13.-]
siRNA-t0032239	CGACGTACTTAGGGTTGTTGAG	22	27	FgHV1gp2	-
siRNA-t0032090	CGAGAAACAAGTGTCTGCTG	20	27	FGSG_06925	K03857|1|8e-22|106|uma:UM05234.1|phosphatidylinositol glycan, class A [EC:2.4.1.198]
siRNA-t0032301	CGATAGTGGGAGCGAAAGCGA	21	27	FGSG_13563	-
siRNA-t0032642	CGGGTGGATCTGGGGTCTGA	20	27	FGSG_11110	-
siRNA-t0032752	CTGCGACCCATGCTTTCGACGTA	23	27	FgHV1gp2	-
siRNA-t0032512	CTGGCCGGTTGAAAATGTTCCAC	23	27	FgHV1gp2	-
siRNA-t0032721	GCGACACCGACGGTACGTGG	20	27	FgHV1gp2	-
siRNA-t0032655	GGCCTGGTAATACCTGATGGCA	22	27	FgHV1gp2	-
siRNA-t0032489	GGCGAACGGGACCGACGTTTT	21	27	FgHV1gp2	-
siRNA-t0032625	GTCTTCTGACGAATTTGGACAA	22	27	FgHV1gp2	-
siRNA-t0032228	TCCAAACTGTTCATTTCGGCG	21	27	FGSG_04004	K15196|1|0.0|1224|fgr:FG04004.1|transcription factor IIIB 90 kDa subunit
siRNA-t0032690	TGCTTTCGACGTACTTGGGTT	21	27	FgHV1gp2	-
siRNA-t0031901	TGGAAGAGAATGACGATATT	20	27	FGSG_09076	K14326|1|0.0|3542|fgr:FG09076.1|regulator of nonsense transcripts 1 [EC:3.6.4.-]
siRNA-t0031882	TGGTGATCCTAAACATGTCGGTG	23	27	FGSG_11659	K01932|1|0.0|1431|afm:AFUA_5G12730|[EC:6.3.2.-]
siRNA-t0032328	TGGTGGATGGTACGTGGCTCA	21	27	FGSG_01049	-
siRNA-t0031894	TGTCTGACTGTTGCTTGATGGTT	23	27	FgHV1gp2	-
siRNA-t0032297	TTCGGGGTCGTACGTCATGG	20	27	FGSG_02394	K01932|1|0.0|942|afm:AFUA_1G10380|[EC:6.3.2.-]
siRNA-t0032496	TTGCGACGTGCTTGAAGGCG	20	27	FGSG_08795	K15418|1|0.0|743|pcs:Pc21g12440|zearalenone synthase, highly reducing iterative type I polyketide synthase;K15419|3|0.0|713|ani:AN2547.2|emericellamide biosynthesis, highly reducing iterative type I polyketide synthase
siRNA-t0033396	AAAATGCCATGGGATCGAAGG	21	26	FGSG_09064	-
siRNA-t0033343	AAATCTGGGTGGCGATGGATTGA	23	26	FgHV1gp2	-
siRNA-t0032993	AAGGTGAGAAGATTGAGTTCATT	23	26	FgHV1gp2	-
siRNA-t0033316	ACTTTCGGGGTCGTACGTCATGGC	24	26	FGSG_02394	K01932|1|0.0|942|afm:AFUA_1G10380|[EC:6.3.2.-]
siRNA-t0033772	AGAAGTCTCCGAACGTGATCTC	22	26	FgHV1gp2	-
siRNA-t0033172	AGAAGTGACGATACTTGAAC	20	26	FGSG_13783	K01932|1|0.0|1526|afm:AFUA_5G12730|[EC:6.3.2.-]
siRNA-t0033325	AGGACCAGTTCCTGCTGAGTG	21	26	FgHV1gp2	-
siRNA-t0033016	AGTCTTGCGGTGTGGGTCTT	20	26	FgHV1gp2	-
siRNA-t0033706	CGAGTGGAGCGTCGGCAAAT	20	26	FGSG_02536	K12856|1|0.0|4746|fgr:FG02536.1|pre-mRNA-processing factor 8
siRNA-t0032909	CGGGACGCGACAAGCAACAATTTGC	25	26	FgHV1gp2	-
siRNA-t0033342	CGTTATGCCAGAACGACGTTT	21	26	FGSG_04376	-
siRNA-t0033019	CTTGGTTCAAGTATCGTCACT	21	26	FgHV1gp2	-
siRNA-t0033287	GAAAGGTGCTGGGTTCTGGT	20	26	FGSG_01515	K11230|1|7e-12|72.0|ani:AN1180.2|mitogen-activated protein kinase kinase kinase [EC:2.7.11.25];K00472|2|6e-08|58.9|nhe:NECHADRAFT_71135|prolyl 4-hydroxylase [EC:1.14.11.2]
siRNA-t0033218	GAACGCTGAAATCGAGGGTGTT	22	26	FgHV1gp2	-
siRNA-t0033586	GAAGGGTTCATACTATCGTTGT	22	26	FGSG_12536	K07050|1|0.0|709|fgr:FG02991.1|

<table>
<tr><td>siRNA-t0033282</td><td>GAGTCTTGCGGTGTGGGTCTT</td><td>21</td><td>26</td><td>FGSG_00874</td><td>K11400|1|0.0|972|fgr:FG00874.1|actin-related protein 4</td></tr>
<tr><td>siRNA-t0033415</td><td>GCAAGCGTGACGAAGATCCCACT</td><td>23</td><td>26</td><td>FgHV1gp1</td><td>-</td></tr>
<tr><td>siRNA-t0033178</td><td>GCACTTGTTTGCCTGCATCGAT</td><td>22</td><td>26</td><td>FgHV1gp2</td><td>-</td></tr>
<tr><td>siRNA-t0033249</td><td>GCGACGACCGTGCGAAATTGTT</td><td>22</td><td>26</td><td>FgHV1gp2</td><td>-</td></tr>
<tr><td>siRNA-t0033555</td><td>GCGCCTTCCATGCTGCCGCGGAT</td><td>23</td><td>26</td><td>FGSG_04587</td><td>K00777|1|7e-77|289|afm:AFUA_7G07100|[EC:2.4.2.-];K14855|4|5e-55|216|lbc:LACBIDRAFT_296068|ribosome assembly protein 4</td></tr>
<tr><td>siRNA-t0032985</td><td>GCTGCATGTACTGTTCGGCAACA</td><td>23</td><td>26</td><td>FGSG_01434</td><td>-</td></tr>
<tr><td>siRNA-t0033414</td><td>GGGACGTCTTCGATGGATGCTGCG</td><td>24</td><td>26</td><td>FgHV1gp2</td><td>-</td></tr>
<tr><td>siRNA-t0033683</td><td>TCAACGTCAGAGGCCTCGTC</td><td>20</td><td>26</td><td>FGSG_13336</td><td>K15505|1|2e-49|197|pno:SNOG_10260|DNA repair protein RAD5 [EC:3.6.4.-]</td></tr>
<tr><td>siRNA-t0033499</td><td>TCGCATTCAGTAGTCGGGGT</td><td>20</td><td>26</td><td>FGSG_01690</td><td>K10395|1|5e-59|229|mpr:MPER_12592|kinesin family member 4/7/21/27;K10398|4|5e-42|173|tml:GSTUM_00008588001|kinesin family member 11</td></tr>
<tr><td>siRNA-t0033369</td><td>TCGGGTCCTGATGGTTTCAG</td><td>20</td><td>26</td><td>FGSG_06016</td><td>K05727|1|1e-46|188|mpr:MPER_07660|dedicator of cytokinesis 3/4/5</td></tr>
<tr><td>siRNA-t0032882</td><td>TCGGGTCCTGATGGTTTCAGAA</td><td>22</td><td>26</td><td>FGSG_06016</td><td>K05727|1|1e-46|188|mpr:MPER_07660|dedicator of cytokinesis 3/4/5</td></tr>
<tr><td>siRNA-t0033662</td><td>TCGTAGTCATCGTCAGGGTACG</td><td>22</td><td>26</td><td>FGSG_09801</td><td>K03164|1|0.0|2817|fgr:FG09801.1|DNA topoisomerase II [EC:5.99.1.3]</td></tr>
<tr><td>siRNA-t0033636</td><td>TGGACCTGAGAGCATGAAGG</td><td>20</td><td>26</td><td>FGSG_08097</td><td>K00688|1|7e-16|87.0|pno:SNOG_13460|starch phosphorylase [EC:2.4.1.1]</td></tr>
<tr><td>siRNA-t0032866</td><td>TTCGAACTAGATGGTGTGGCG</td><td>21</td><td>26</td><td>FgHV1gp2</td><td>-</td></tr>
<tr><td>siRNA-t0032826</td><td>TTGCTTGATGGTTGGCACAA</td><td>20</td><td>26</td><td>FGSG_12571</td><td>-</td></tr>
<tr><td>siRNA-t0033312</td><td>TTGGCCAAATCTCGGACCGCT</td><td>21</td><td>26</td><td>FgHV1gp2</td><td>-</td></tr>
<tr><td>siRNA-t0033460</td><td>TTGTTTCTCGTGGGATTGGGCGTG</td><td>24</td><td>26</td><td>FgHV1gp2</td><td>-</td></tr>
<tr><td>siRNA-t0034051</td><td>AACGGTTCTTGGAAAGTTGG</td><td>20</td><td>25</td><td>FgHV1gp2</td><td>-</td></tr>
<tr><td>siRNA-t0034381</td><td>AGAACGTCGTTATGCCAGAA</td><td>20</td><td>25</td><td>FGSG_09575</td><td>-</td></tr>
<tr><td>siRNA-t0034291</td><td>AGTGAAAGTGTCGTAGGAAA</td><td>20</td><td>25</td><td>FgHV1gp1</td><td>-</td></tr>
<tr><td>siRNA-t0034231</td><td>CAGGCATCAGGGGTCCTCTT</td><td>20</td><td>25</td><td>FGSG_06514</td><td>-</td></tr>
<tr><td>siRNA-t0034136</td><td>CATACAACTCGAACCAGTTCG</td><td>21</td><td>25</td><td>FGSG_08728</td><td>K02179|1|6e-11|69.7|cgr:CAGL0F06391g|cell cycle arrest protein BUB2</td></tr>
<tr><td>siRNA-t0034629</td><td>CCGAAGGAACTCCACATAGCGTCGT</td><td>25</td><td>25</td><td>FgHV1gp2</td><td>-</td></tr>
<tr><td>siRNA-t0033872</td><td>CGTTACAATCGTGGACTTCC</td><td>20</td><td>25</td><td>FgHV1gp2</td><td>-</td></tr>
<tr><td>siRNA-t0034150</td><td>GAGTGTGTCGCGTCTACCTTGTT</td><td>23</td><td>25</td><td>FgHV1gp2</td><td>-</td></tr>
<tr><td>siRNA-t0034044</td><td>GCATGAACTCGGTCAACGAAA</td><td>21</td><td>25</td><td>FgHV1gp2</td><td>-</td></tr>
<tr><td>siRNA-t0034317</td><td>GCTTGACAACAGGTTGGAATCTGCG</td><td>25</td><td>25</td><td>FgHV1gp2</td><td>-</td></tr>
<tr><td>siRNA-t0034262</td><td>GGGACCAGGATTCTTCTCCACGTC</td><td>24</td><td>25</td><td>FGSG_10617</td><td>K14859|1|2e-98|359|scm:SCHCODRAFT_255991|ribosome biogenesis protein SSF1/2;K00143|2|1e-14|82.0|zro:ZYRO0C16566g|L-aminoadipate-semialdehyde dehydrogenase [EC:1.2.1.31]</td></tr>
<tr><td>siRNA-t0034631</td><td>GGGAGGCCTATTCATCGTCA</td><td>20</td><td>25</td><td>FgHV1gp2</td><td>-</td></tr>
<tr><td>siRNA-t0034886</td><td>GGTGGGTTCTTGAAGTTGGTGC</td><td>22</td><td>25</td><td>FgHV1gp2</td><td>-</td></tr>
<tr><td>siRNA-t0034391</td><td>GGTTCGACACTGGAAGAGAAT</td><td>21</td><td>25</td><td>FGSG_05503</td><td>K03785|1|1e-17|91.7|ani:AN4350.2|3-dehydroquinate dehydratase I [EC:4.2.1.10];K00472|2|1e-14|81.6|nhe:NECHADRAFT_71135|prolyl 4-hydroxylase [EC:1.14.11.2];K09241|3|3e-08|60.5|sce:YPL248C|transcriptional regulatory protein GAL4;K09247|4|1e-06|55.5|sce:YLR256W|heme activator protein 1;K09248|5|2e-06|54.3|kla:KLLA0E18129g|proline utilization trans-activator</td></tr>
<tr><td>siRNA-t0034555</td><td>GGTTTGAACCCTGACAATACCAAA</td><td>24</td><td>25</td><td>FgHV1gp2</td><td>-</td></tr>
<tr><td>siRNA-t0034174</td><td>GTCCTTCGTTGAATCGATCGTCATG</td><td>25</td><td>25</td><td>FgHV1gp2</td><td>-</td></tr>
<tr><td>siRNA-t0033877</td><td>GTGATAAATGAACGTCTGACTGTA</td><td>24</td><td>25</td><td>FgHV1gp2</td><td>-</td></tr>
<tr><td>siRNA-t0033808</td><td>GTGGGATTGGGCGTGTGTTCTTT</td><td>23</td><td>25</td><td>FgHV1gp2</td><td>-</td></tr>
<tr><td>siRNA-t0033857</td><td>GTGGGTCTTCTGACGAATTTGGACA</td><td>25</td><td>25</td><td>FgHV1gp2</td><td>-</td></tr>
<tr><td>siRNA-t0033811</td><td>GTGGTGAGGTATGACGTGTTCAT</td><td>23</td><td>25</td><td>FgHV1gp2</td><td>-</td></tr>
<tr><td>siRNA-t0033991</td><td>TCGCTCCCACTATCGTCCTGCG</td><td>22</td><td>25</td><td>FGSG_01294</td><td>K00799|1|1e-34|146|afv:AFLA_022030|glutathione S-transferase [EC:2.5.1.18]</td></tr>
<tr><td>siRNA-t0033908</td><td>TGATTTGCGACGTGCTTGAAGGCG</td><td>24</td><td>25</td><td>FgHV1gp2</td><td>-</td></tr>
<tr><td>siRNA-t0034492</td><td>TGTTGTATGAAGCTGATGGAATGC</td><td>24</td><td>25</td><td>FgHV1gp2</td><td>-</td></tr>
<tr><td>siRNA-t0035824</td><td>AAGTATCGTCACTTCTTCGT</td><td>20</td><td>24</td><td>FGSG_13663</td><td>K03018|1|0.0|1747|fgr:FG09860.1|DNA-directed RNA polymerase III subunit RPC1 [EC:2.7.7.6];K15508|2|0.0|906|ncr:NCU03987|21S rRNA (uridine2791-2’-O)-methyltransferase [EC:2.1.1.168]</td></tr>
<tr><td>siRNA-t0036009</td><td>ACCATCAGGACCCGAGAAAAACGCC</td><td>25</td><td>24</td><td>FgHV1gp2</td><td>-</td></tr>
<tr><td>siRNA-t0036044</td><td>ACGGGACCGACGTTTTGGCGAG</td><td>22</td><td>24</td><td>FgHV1gp2</td><td>-</td></tr>
<tr><td>siRNA-t0035699</td><td>ACTGTTGACGTCACCCTTTG</td><td>20</td><td>24</td><td>FgHV1gp2</td><td>-</td></tr>
<tr><td>siRNA-t0035376</td><td>ACTTTCGGGGTCGTACGTCATG</td><td>22</td><td>24</td><td>FGSG_02394</td><td>K01932|1|0.0|942|afm:AFUA_1G10380|[EC:6.3.2.-]</td></tr>
<tr><td>siRNA-t0035744</td><td>AGAGATCTCCGCAGAAGAAT</td><td>20</td><td>24</td><td>FGSG_06836</td><td>K01259|1|7e-121|433|afm:AFUA_2G05000|proline iminopeptidase [EC:3.4.11.5]</td></tr>
<tr><td>siRNA-t0035453</td><td>AGGAACTCCACATAGCGTCGTC</td><td>22</td><td>24</td><td>FgHV1gp2</td><td>-</td></tr>
<tr><td>siRNA-t0036041</td><td>AGTATGAACCCTTCCACGATTGGC</td><td>24</td><td>24</td><td>FgHV1gp2</td><td>-</td></tr>
<tr><td>siRNA-t0035785</td><td>ATCGATCGTCATGGTGATAC</td><td>20</td><td>24</td><td>FGSG_00248</td><td>-</td></tr>
<tr><td>siRNA-t0035109</td><td>ATGGAGGCGCTGGGACTGTA</td><td>20</td><td>24</td><td>FGSG_07041</td><td>K15414|1|9e-138|488|fgr:FG07041.1|complement component 1 Q subcomponent-binding protein, mitochondrial</td></tr>
<tr><td>siRNA-t0035097</td><td>CAACGTCAGAGGCCTCGTCA</td><td>20</td><td>24</td><td>FGSG_08487</td><td>K14297|1|0.0|2640|fgr:FG08487.1|nuclear pore complex protein Nup98-Nup96</td></tr>
<tr><td>siRNA-t0035975</td><td>CAGACACTTGTTTCTCGTGGGAT</td><td>23</td><td>24</td><td>FgHV1gp2</td><td>-</td></tr>
<tr><td>siRNA-t0034889</td><td>CCAAATCGTCATACATTGCAC</td><td>21</td><td>24</td><td>FgHV1gp2</td><td>-</td></tr>
<tr><td>siRNA-t0035135</td><td>CCTAGACCGAAGAAAGACCAC</td><td>21</td><td>24</td><td>FgHV1gp2</td><td>-</td></tr>
<tr><td>siRNA-t0035737</td><td>CGGTGTGGGTCTTCTGACGAA</td><td>21</td><td>24</td><td>FGSG_07081</td><td>-</td></tr>
<tr><td>siRNA-t0035643</td><td>CGTACTTAGGGTTGTTGAGAACGCT</td><td>25</td><td>24</td><td>FgHV1gp2</td><td>-</td></tr>
<tr><td>siRNA-t0035304</td><td>CGTGATAAATGAACGTCTGACTGTA</td><td>25</td><td>24</td><td>FgHV1gp2</td><td>-</td></tr>
<tr><td>siRNA-t0035408</td><td>CTGAACCCAGTTGCTGTATCTT</td><td>22</td><td>24</td><td>FGSG_06379</td><td>K14289|1|0.0|2450|fgr:FG06379.1|exportin-5</td></tr>
<tr><td>siRNA-t0035413</td><td>CTGAGAGCATGAAGGCACAAT</td><td>21</td><td>24</td><td>FGSG_08778</td><td>K09595|1|0.0|1018|fgr:FG08778.1|minor histocompatibility antigen H13 [EC:3.4.23.-]</td></tr>
<tr><td>siRNA-t0035193</td><td>CTTTCGGGGTCGTACGTCATG</td><td>21</td><td>24</td><td>FGSG_02394</td><td>K01932|1|0.0|942|afm:AFUA_1G10380|[EC:6.3.2.-]</td></tr>
<tr><td>siRNA-t0035415</td><td>GAAGAACATGCTACTGGAGA</td><td>20</td><td>24</td><td>FGSG_03523</td><td>K06901|1|0.0|912|fgr:FG03523.1|putative MFS transporter, AGZA family, xanthine/uracil permease</td></tr>
<tr><td>siRNA-t0035724</td><td>GACCGAAGAAAGACCACATCCA</td><td>22</td><td>24</td><td>FgHV1gp2</td><td>-</td></tr>
<tr><td>siRNA-t0035185</td><td>GAGCTCTGTGACTCCAGCCAAA</td><td>22</td><td>24</td><td>FgHV1gp2</td><td>-</td></tr>
<tr><td>siRNA-t0035360</td><td>GAGTGGCATATTCAAGAATCAATGA</td><td>25</td><td>24</td><td>FgHV1gp2</td><td>-</td></tr>
<tr><td>siRNA-t0035803</td><td>GAGTGTGTCGCGTCTACCTT</td><td>20</td><td>24</td><td>FgHV1gp2</td><td>-</td></tr>
<tr><td>siRNA-t0036046</td><td>GATTTGCGACGTGCTTGAAGGCGAA</td><td>25</td><td>24</td><td>FgHV1gp2</td><td>-</td></tr>
<tr><td>siRNA-t0034938</td><td>GCCATTCGTTACAATCGTGGAC</td><td>22</td><td>24</td><td>FgHV1gp2</td><td>-</td></tr>
<tr><td>siRNA-t0035421</td><td>GCCCAGTCATGAATTTTCGGTA</td><td>22</td><td>24</td><td>FgHV1gp2</td><td>-</td></tr>
<tr><td>siRNA-t0035790</td><td>GCGGCAAGTACAAACTCCCTT</td><td>21</td><td>24</td><td>FGSG_01163</td><td>-</td></tr>
<tr><td>siRNA-t0035979</td><td>GCTGAACCCAGTTGCTGTATCT</td><td>22</td><td>24</td><td>FGSG_06379</td><td>K14289|1|0.0|2450|fgr:FG06379.1|exportin-5</td></tr>
<tr><td>siRNA-t0035868</td><td>GCTGTACTGAAGGTGGTGTCA</td><td>21</td><td>24</td><td>FgHV1gp2</td><td>-</td></tr>
<tr><td>siRNA-t0036053</td><td>GGGATACCAACTTGATTTGA</td><td>20</td><td>24</td><td>FGSG_13640</td><td>K07300|1|0.0|3608|fgr:FG09771.1|Ca2+:H+ antiporter</td></tr>
<tr><td>siRNA-t0034993</td><td>GTCTGACTGTTGCTTGATGGT</td><td>21</td><td>24</td><td>FGSG_04580</td><td>K08711|1|0.0|1709|cne:CNB05770|ATP-binding cassette, subfamily G (WHITE), member 2, PDR</td></tr>
<tr><td>siRNA-t0036067</td><td>GTGAACTGCTGCGCTATGGTGTGAG</td><td>25</td><td>24</td><td>FgHV1gp2</td><td>-</td></tr>
<tr><td>siRNA-t0034995</td><td>GTGATGGAGACTAAACGCTTTGG</td><td>23</td><td>24</td><td>FgHV1gp1</td><td>-</td></tr>
<tr><td>siRNA-t0036055</td><td>GTGTCGTAGGAAAGGAAAAGT</td><td>21</td><td>24</td><td>FgHV1gp1</td><td>-</td></tr>
<tr><td>siRNA-t0035470</td><td>GTTACAATCGTGGACTTCCT</td><td>20</td><td>24</td><td>FgHV1gp2</td><td>-</td></tr>
<tr><td>siRNA-t0035173</td><td>TAGATCTTGCTGTAGTGTCTGAA</td><td>23</td><td>24</td><td>FgHV1gp2</td><td>-</td></tr>
<tr><td>siRNA-t0035018</td><td>TATTCGGACTCATCCCACATGATGG</td><td>25</td><td>24</td><td>FgHV1gp2</td><td>-</td></tr>
<tr><td>siRNA-t0035503</td><td>TCTCGGGTCCTGATGGTTTCAG</td><td>22</td><td>24</td><td>FGSG_06016</td><td>K05727|1|1e-46|188|mpr:MPER_07660|dedicator of cytokinesis 3/4/5</td></tr>
<tr><td>siRNA-t0035120</td><td>TGCTGAATGCTCTGCCTCGGTC</td><td>22</td><td>24</td><td>FgHV1gp2</td><td>-</td></tr>
<tr><td>siRNA-t0035679</td><td>TGGAGCGTCGGCAAATTTGGC</td><td>21</td><td>24</td><td>FGSG_02536</td><td>K12856|1|0.0|4746|fgr:FG02536.1|pre-mRNA-processing factor 8</td></tr>
<tr><td>siRNA-t0035871</td><td>TGGATCAATGTCAGCAGTGTTCA</td><td>23</td><td>24</td><td>FgHV1gp2</td><td>-</td></tr>
<tr><td>siRNA-t0035387</td><td>TGGTGAGGTATGACGTGTTCAT</td><td>22</td><td>24</td><td>FgHV1gp2</td><td>-</td></tr>
<tr><td>siRNA-t0035601</td><td>TGTGGTGAGGTATGACGTGTTC</td><td>22</td><td>24</td><td>FgHV1gp2</td><td>-</td></tr>
<tr><td>siRNA-t0036389</td><td>AAATTTTTGGTCTTCGACGGGTA</td><td>23</td><td>23</td><td>FGSG_13308</td><td>-</td></tr>
<tr><td>siRNA-t0036205</td><td>AACACGCAGCAGAATTCTAGCC</td><td>22</td><td>23</td><td>FgHV1gp2</td><td>-</td></tr>
</table>

siRNA-t0036219	AAGGAGCTCAAAGGTATGATG	21	23	FGSG_08060	K00688|1|4e-11|69.3|pno:SNOG_13460|starch phosphorylase [EC:2.4.1.1]
siRNA-t0036942	AAGTGTCGTAGGAAAGGAAA	20	23	FGSG_07217	K05542|1|0.0|998|fgr:FG07217.1|tRNA-dihydrouridine synthase 1 [EC:1.3.1.88]
siRNA-t0036941	AATCAATGTACCGTTCGGCA	20	23	FGSG_03931	K13336|1|2e-47|189|afv:AFLA_048740|peroxin-3;K03448|2|8e-40|164|ani:AN2663.2|MFS transporter, ACS family, pantothenate transporter
siRNA-t0037245	ACAAGCTGGTCTGGGTGCTGA	21	23	FgHV1gp2	-
siRNA-t0036211	AGAACGTAGATGTGATGCCTTG	22	23	FgHV1gp2	-
siRNA-t0036492	AGAATTCTGCTGCGTGTTAT	20	23	FGSG_09750	-
siRNA-t0036184	AGGACAAGCCGTCAAACATAGACAC	25	23	FgHV1gp2	-
siRNA-t0036918	AGGACCCGAGAAAAACGCCCC	21	23	FgHV1gp2	-
siRNA-t0036723	AGGGACGTCTTCGATGGATG	20	23	FGSG_03170	K00698|1|4e-24|112|cci:CC1G_10992|chitin synthase [EC:2.4.1.16]
siRNA-t0036406	AGTTCTCTTTGTTGGCCTGG	20	23	FGSG_09560	-
siRNA-t0036852	CAAGAACATCAGGCATGTAT	20	23	FGSG_07245	K12830|1|0.0|2446|fgr:FG07245.1|splicing factor 3B subunit 3
siRNA-t0036109	CCGACAATGGCGCATACACGGAACC	25	23	FgHV1gp2	-
siRNA-t0037036	CCGATGGAAGTGATGGCAAT	20	23	FGSG_08209	K01932|1|0.0|1305|afm:AFUA_5G12730|[EC:6.3.2.-]
siRNA-t0037127	CGGTTCTTGGAAAGTTGGAG	20	23	FGSG_02449	K00936|1|2e-45|184|afm:AFUA_8G06140|[EC:2.7.3.-];K11233|3|7e-17|89.4|cgr:CAGL0D02882g|osomolarity two-component system, response regulator SSK1
siRNA-t0036385	CGTGCGCTTCTGGATCAATGTC	22	23	FgHV1gp2	-
siRNA-t0037210	CTGAGAGCATGAAGGCACAATA	22	23	FGSG_08778	K09595|1|0.0|1018|fgr:FG08778.1|minor histocompatibility antigen H13 [EC:3.4.23.-]
siRNA-t0036244	CTGATGTGGTGAGGTATGACGTG	23	23	FgHV1gp2	-
siRNA-t0036847	GAGTGCCATGACGTACGACCCCGAA	25	23	FgHV1gp1	-
siRNA-t0036172	GAGTGCGTAGATAAAGGTTAT	21	23	FGSG_01801	-
siRNA-t0037076	GAGTGGCATATTCAAGAATCA	21	23	FGSG_11486	K11369|1|2e-49|196|ani:AN3929.2|retrograde regulation protein 2;K13336|2|6e-49|194|afv:AFLA_048740|peroxin-3;K03448|3|2e-42|172|mgr:MGG_02319|MFS transporter, ACS family, pantothenate transporter
siRNA-t0037050	GCATGTTACTAAGAAGAGCGA	21	23	FgHV1gp2	-
siRNA-t0036520	GCCATCCTATAGATCGCGCGT	21	23	FgHV1gp2	-
siRNA-t0036984	GCCATTCGTTACAATCGTGGACTT	24	23	FgHV1gp2	-
siRNA-t0036985	GCTGAACCCAGTTGCTGTAT	20	23	FGSG_06379	K14289|1|0.0|2450|fgr:FG06379.1|exportin-5
siRNA-t0037163	GCTGATGATCATGACGTGGAG	21	23	FGSG_11121	-
siRNA-t0036630	GCTGCCGCGGATAACCTATACT	22	23	FgHV1gp2	-
siRNA-t0037242	GCTTGAAGGCGAAGTATGAGGGAG	24	23	FgHV1gp2	-
siRNA-t0036856	GGACCGACGTTTTGGCGAGACCG	23	23	FgHV1gp2	-
siRNA-t0036156	GGGGTCGTACGTCATGGCAC	20	23	FGSG_10228	K03114|1|0.0|934|fgr:FG10228.1|mitosis inhibitor protein kinase SWE1 [EC:2.7.11.1]
siRNA-t0036108	TCGACAGGCGTGGCTGAGAG	20	23	FgHV1gp2	-
siRNA-t0037166	TCGACAGGCGTGGCTGAGAGAA	22	23	FgHV1gp2	-
siRNA-t0036199	TCTACCAGAACCCAGCACCTT	21	23	FgHV1gp2	-
siRNA-t0036855	TCTGGCCGGTTGAAAATGTTC	21	23	FgHV1gp2	-
siRNA-t0036072	TGGCGACACCGACGGTACGTGGA	23	23	FgHV1gp2	-
siRNA-t0036305	TGGTGAGGTATGACGTGTTC	20	23	FgHV1gp2	-
siRNA-t0037131	TTGACCCCGAGCGTGATGTG	20	23	FGSG_10835	-
siRNA-t0037855	AAACGCCCCTGGACGGTACT	20	22	FgHV1gp2	-
siRNA-t0038491	AATGATTTGCGACGTGCTTGA	21	22	FgHV1gp2	-
siRNA-t0038541	AATGTACCGTTCGGCAACAG	20	22	FGSG_07974	K05989|1|0.0|1786|fgr:FG07974.1|alpha-L-rhamnosidase [EC:3.2.1.40]
siRNA-t0037343	ACATAGCGTCGTCACCTTGA	20	22	FGSG_13835	-
siRNA-t0038415	ACCTAGACCGAAGAAAGACCAC	22	22	FgHV1gp2	-
siRNA-t0038072	AGGCTTGTCGTCGGGAGAATCCAG	24	22	FgHV1gp2	-
siRNA-t0038290	AGGTACCTGAGGTTTGAACCCT	22	22	FgHV1gp2	-
siRNA-t0037789	AGTGAAAGTGTCGTAGGAAAGGAAA	25	22	FgHV1gp1	-
siRNA-t0038613	ATGCATGGAGGCGCTGGGAC	20	22	FGSG_09604	-
siRNA-t0037574	CAAGGATTTGTAAGGGGAAAC	21	22	FGSG_12660	-
siRNA-t0037790	CATACAACTCGAACCAGTTCGT	22	22	FGSG_08728	K02179|1|6e-11|69.7|cgr:CAGL0F06391g|cell cycle arrest protein BUB2
siRNA-t0037646	CCAGAATCGAGCTAAACGAACT	22	22	FgHV1gp2	-
siRNA-t0038012	CGCTGATCTGGCTCGTGACTT	21	22	FgHV1gp2	-
siRNA-t0037570	CTGAAATCGAGGGTGTTGGGGTC	23	22	FgHV1gp2	-
siRNA-t0037821	CTGGATGTGGGTGCGCCGAA	20	22	FgHV1gp2	-
siRNA-t0038614	GAAACGTCGTTCTGGCATAAC	21	22	FGSG_01049	-
siRNA-t0038075	GAGCAGGATCTCGTAAGCTG	20	22	FGSG_10913	K14541|1|0.0|3625|fgr:FG10913.1|urea carboxylase / allophanate hydrolase [EC:6.3.4.6 3.5.1.54]
siRNA-t0038109	GAGTGCCATGACGTACGACCCCG	23	22	FgHV1gp1	-
siRNA-t0038020	GAGTGCCATGACGTACGACCCCGA	24	22	FgHV1gp1	-
siRNA-t0038346	GCAGAAGAATTAGCCTTTGAA	21	22	FGSG_12776	K12879|1|0.0|2720|fgr:FG05657.1|THO complex subunit 2
siRNA-t0037742	GCGACGACCGTGCGAAATTG	20	22	FgHV1gp2	-
siRNA-t0037825	GCTGAACACCTTTGGCCTCAT	21	22	FGSG_09466	K14416|1|0.0|1385|fgr:FG09466.1|elongation factor 1 alpha-like protein
siRNA-t0038157	GCTGATCTGGCTCGTGACTTT	21	22	FgHV1gp2	-
siRNA-t0037567	GCTTACCTAGACCGAAGAAAGACCA	25	22	FgHV1gp2	-
siRNA-t0037868	GGATTAATCAATGTACCGTTCGG	23	22	FgHV1gp2	-
siRNA-t0037753	GGCGAGACCGATGATTTCCATCCC	24	22	FgHV1gp2	-
siRNA-t0037588	GGCGTGCTGAGATCAAGTATAG	22	22	FGSG_10790	K15389|1|3e-101|368|ure:UREG_05875|acyl-CoA dehydrogenase;K00257|2|1e-100|366|afm:AFUA_6G10880|[EC:1.3.99.-]
siRNA-t0037976	GTGCCATGACGTACGACCCC	20	22	FgHV1gp1	-
siRNA-t0037841	TACTTGATCTCAGCACGCCGGTT	23	22	FgHV1gp2	-
siRNA-t0038535	TAGCGTCGTCACCTTGATTG	20	22	FGSG_10511	K11236|1|0.0|1846|fgr:FG10511.1|cell division control protein 24
siRNA-t0037315	TCAATGATGGCACGTCGACAGGC	23	22	FgHV1gp2	-
siRNA-t0037635	TCCTTCGTTGAATCGATCGTC	21	22	FgHV1gp2	-
siRNA-t0037438	TCGCTCCCACTATCGTCCTG	20	22	FGSG_10648	-
siRNA-t0037446	TCGTAGTCATCGTCAGGGTAC	21	22	FGSG_09801	K03164|1|0.0|2817|fgr:FG09801.1|DNA topoisomerase II [EC:5.99.1.3]
siRNA-t0037571	TGAACCACAGTCGAATGAAATG	22	22	FgHV1gp2	-
siRNA-t0038527	TGCAGCGGCAAATACAGGAC	20	22	FGSG_06870	K06927|1|2e-149|529|mgr:MGG_00500|
siRNA-t0038377	TTCGTTGAATCGATCGTCATGGTGA	25	22	FgHV1gp2	-
siRNA-t0038100	TTCTCTCTGACCTTGATGGCG	21	22	FGSG_09454	K14293|1|0.0|1735|fgr:FG09454.1|importin subunit beta-1
siRNA-t0038302	TTGCGACGTGCTTGAAGGCGAA	22	22	FgHV1gp2	-
siRNA-t0040043	AAATGCCATGGGATCGAAGG	20	21	FGSG_06511	-
siRNA-t0038792	AATCGATCGTCATGGTGATAC	21	21	FGSG_00248	-
siRNA-t0039699	ACATGATGAGTAAGCGGGCTTG	22	21	FGSG_03736	K14859|1|6e-27|122|scm:SCHCODRAFT_255991|ribosome biogenesis protein SSF1/2
siRNA-t0039977	ACATGCATGGAGGCGCTGGG	20	21	FGSG_13574	-
siRNA-t0039767	ACGGTACGTGGAAATCTTCAGGCCC	25	21	FgHV1gp2	-
siRNA-t0040022	AGATCAACGTCAGAGGCCTC	20	21	FGSG_10040	K01262|1|0.0|1198|afm:AFUA_1G07720|Xaa-Pro aminopeptidase [EC:3.4.11.9]
siRNA-t0039274	AGGCGTCGACACGATGATACCCT	23	21	FgHV1gp2	-
siRNA-t0039885	AGGCTTGTCGTCGGGAGAAT	20	21	FGSG_08614	K01874|1|0.0|1256|fgr:FG08614.1|methionyl-tRNA synthetase [EC:6.1.1.10]
siRNA-t0038623	ATCTGGCCGGTTGAAAATGTTCCAC	25	21	FgHV1gp2	-
siRNA-t0039835	ATGTGGTGAGGTATGACGTGT	21	21	FgHV1gp2	-
siRNA-t0038976	CAAGCAACAGTCAGACACGAT	21	21	FGSG_08389	-
siRNA-t0038706	CAATCGTGGACTTCCTTTAGGA	22	21	FgHV1gp2	-
siRNA-t0039804	CAGTTCTCTTTGTTGGCCTG	20	21	FGSG_09560	-
siRNA-t0039817	CCCACTCGTGAACCTGTGGT	20	21	FGSG_12776	K12879|1|0.0|2720|fgr:FG05657.1|THO complex subunit 2
siRNA-t0039821	CCCACTCTAGCAATGCATCACCA	23	21	FgHV1gp1	-
siRNA-t0038740	CCTATTCATCGTCAATGACCA	21	21	FGSG_08065	K00100|1|1e-113|408|afm:AFUA_7G04850|[EC:1.1.1.-]
siRNA-t0039997	CGCCAAAACGTCGGTCCCGT	20	21	FGSG_02781	K12599|1|0.0|2405|fgr:FG02781.1|antiviral helicase SKI2 [EC:3.6.4.-]
siRNA-t0039760	CGCGGTGTGGACATCGGAGCGG	22	21	FGSG_08980	K00002|1|0.0|716|fgr:FG08980.1|alcohol dehydrogenase (NADP+) [EC:1.1.1.2]

siRNA-t0038789	CGCGTGTCGTTAGAAGCTTGA	21	21	FGSG_05325	-
siRNA-t0039523	CGGTGCTGATGATCATGACG	20	21	FGSG_05944	K11644|1|1e-10|68.6|ssl:SS1G_06217|paired amphipathic helix protein Sin3a
siRNA-t0039746	CTCAGAGTCTTGCGGTGTGGGT	22	21	FgHV1gp2	-
siRNA-t0039227	GAAGTCCACGATTGTAACGAA	21	21	FGSG_05306	K08333|1|0.0|3035|fgr:FG05306.1|phosphoinositide-3-kinase, regulatory subunit 4, p150 [EC:2.7.11.1]
siRNA-t0039020	GCCGTTTGTGCTGCAAGATGGACTG	25	21	FgHV1gp2	-
siRNA-t0040037	GCTTGGTTTCGGGACGCGACA	21	21	FgHV1gp2	-
siRNA-t0039460	GGCGCATACACGGAACCCATTGAT	24	21	FgHV1gp2	-
siRNA-t0039154	TAATATCGTCATTCTCTTCCAG	22	21	FGSG_10105	K12864|1|8e-17|88.2|cne:CNH03260|beta-catenin-like protein 1
siRNA-t0039936	TACAGGACCGCGCGATGGCGC	21	21	FgHV1gp2	-
siRNA-t0039948	TAGAATGTCGAGATACTCTT	20	21	FGSG_04220	K06867|1|5e-81|301|spo:SPBC336.12c|;K06648|2|2e-51|203|ago:AGOS_AFR690C|regulatory protein SWI6
siRNA-t0038810	TAGTATGAACCCTTCCACGATTGGC	25	21	FgHV1gp2	-
siRNA-t0039162	TGAGCGTTTGTGGAGCCATG	20	21	FGSG_11234	K15505|1|7e-25|115|ssl:SS1G_03212|DNA repair protein RAD5 [EC:3.6.4.-];K15711|5|2e-21|103|tve:TRV_04890|SWI/SNF-related matrix-associated actin-dependent regulator of chromatin subfamily A3 [EC:3.6.4.- 6.3.2.19]
siRNA-t0038927	TGATACAAACCGGAATGCACTT	22	21	FgHV1gp2	-
siRNA-t0039600	TGATCTGGCCGGTTGAAAATGTT	23	21	FgHV1gp2	-
siRNA-t0039930	TGATTTGCGACGTGCTTGAAGGCGA	25	21	FgHV1gp2	-
siRNA-t0039707	TGTCAGTGTCAGGATCCGTC	20	21	FGSG_09917	K01115|1|0.0|3466|fgr:FG09917.1|phospholipase D [EC:3.1.4.4]
siRNA-t0039625	TTCGTTACAATCGTGGACTTC	21	21	FGSG_04284	K12867|1|0.0|1709|fgr:FG04284.1|pre-mRNA-splicing factor SYF1
siRNA-t0039907	TTGGTTTCGGGACGCGACAAGCAA	24	21	FgHV1gp2	-
siRNA-t0040465	AACATATTGAACCACAGTCGAATGA	25	20	FgHV1gp2	-
siRNA-t0041324	AATCGATCGTCATGGTGATACCCAT	25	20	FgHV1gp2	-
siRNA-t0040639	AGAGCGTGTAGGGCTGTCCCAT	22	20	FGSG_05297	K14676|1|0.0|2814|fgr:FG05297.1|lysophospholipid hydrolase [EC:3.1.1.5]
siRNA-t0041142	AGCTGCTACTCGAACCGCCCT	21	20	FgHV1gp2	-
siRNA-t0040690	ATTGTCGAGTCGAAAGCGGT	20	20	FGSG_11279	-
siRNA-t0040844	CGTGAGTGCGTAGATAAAGGTTAT	24	20	FgHV1gp2	-
siRNA-t0041058	CTAAGAAGAGCGAGTGGAGCG	21	20	FGSG_00384	-
siRNA-t0041225	CTGAAACCATCAGGACCCGAGAA	23	20	FgHV1gp2	-
siRNA-t0041348	CTGGACGAAGACTTGCGTAC	20	20	FGSG_07962	K10875|1|0.0|1660|fgr:FG07962.1|DNA repair and recombination protein RAD54 and RAD54-like protein [EC:3.6.4.-]
siRNA-t0040940	GAAGTTTGCATCCTCGACCCACTCA	25	20	FgHV1gp2	-
siRNA-t0040951	GACGGGTTGGCCAAATCTCGGAC	23	20	FgHV1gp2	-
siRNA-t0040126	GAGCGTGTAGGGCTGTCCCATA	22	20	FGSG_05297	K14676|1|0.0|2814|fgr:FG05297.1|lysophospholipid hydrolase [EC:3.1.1.5]
siRNA-t0040207	GCCTGGGAGACAGAGCCTGAGTCA	24	20	FgHV1gp2	-
siRNA-t0040466	GGAAAGGAAAAGTTCTCGCCAGCA	24	20	FgHV1gp1	-
siRNA-t0040921	GGACAAGCCGTCAAACATAGACAC	24	20	FgHV1gp2	-
siRNA-t0040703	GGAGTGGCATATTCAAGAATCA	22	20	FgHV1gp2	-
siRNA-t0040984	GTCATCGGGAATCATGTCGGTG	22	20	FgHV1gp2	-
siRNA-t0040307	GTGCGAGCAGGATTGAAGTGTAAT	24	20	FgHV1gp2	-
siRNA-t0040068	GTGGAAATCTTCAGGCCCTGGG	22	20	FgHV1gp2	-
siRNA-t0040288	GTGGTCTTTCTTCGGTCTAGG	21	20	FGSG_14002	-
siRNA-t0040217	TAGATCTTGCTGTAGTGTCTGA	22	20	FgHV1gp2	-
siRNA-t0041449	TCGACAGGCGTGGCTGAGAGA	21	20	FgHV1gp2	-
siRNA-t0040681	TCGGTAGTCATCGGGAATCATG	22	20	FgHV1gp2	-
siRNA-t0040281	TGAAAGTGTCGTAGGAAAGG	20	20	FGSG_05232	-
siRNA-t0040195	TGCTGAATGCTCTGCCTCGGT	21	20	FGSG_09306	K11644|1|0.0|2881|fgr:FG09306.1|paired amphipathic helix protein Sin3a
siRNA-t0040052	TTCGGACTCATCCCACATGATGG	23	20	FgHV1gp2	-
siRNA-t0041138	TTGTGAACCTGATCCCGGAA	20	20	FGSG_10966	K11702|1|1e-47|190|cne:CNL04750|beta-1,2-xylosyltransferase [EC:2.4.2.-]
siRNA-t0041568	AAAACGCCCCTGGACGGTACTT	22	19	FgHV1gp2	-
siRNA-t0041654	AACACGCAGCAGAATTCTAG	20	19	FgHV1gp2	-
siRNA-t0042676	AACGGGTCCAGATGTTGTGCTTTT	24	19	FgHV1gp2	-
siRNA-t0042087	AAGAACCCATATGGATCGGTGGCAC	25	19	FgHV1gp2	-
siRNA-t0042291	ACAATGGCGCATACACGGAACCCA	24	19	FgHV1gp2	-
siRNA-t0042947	ACCTAGACCGAAGAAAGACCACA	23	19	FgHV1gp2	-
siRNA-t0041577	ACGGGTCCAGATGTTGTGCTTTT	23	19	FGSG_07939	K05236|1|0.0|2545|fgr:FG07939.1|coatomer protein complex, subunit alpha (xenin)
siRNA-t0043057	AGAGCTGGGGATGTTTGCTTGG	22	19	FgHV1gp2	-
siRNA-t0041974	AGAGTGCTCATCGTTGTGCCAA	22	19	FGSG_04332	-
siRNA-t0042095	AGCAGGATTGAAGTGTAATG	20	19	FGSG_05135	K08860|1|0.0|3130|fgr:FG05135.1|eukaryotic translation initiation factor 2-alpha kinase [EC:2.7.11.1]
siRNA-t0041980	AGCTGGGGATGTTTGCTTGG	20	19	FGSG_04916	-
siRNA-t0042418	AGGCTATCACGGATAATGTCAT	22	19	FGSG_12101	-
siRNA-t0041744	AGGCTCCTCCGACAATGGCGC	21	19	FGSG_10215	K15289|1|2e-09|62.8|yli:YALI0E22803g|solute carrier family 35, member F5;K15303|2|4e-09|62.0|ani:AN0675.2|aflatoxin B1 aldehyde reductase
siRNA-t0042682	ATAAATGAACGTCTGACTGT	20	19	FgHV1gp2	-
siRNA-t0042121	ATCGTAGTCATCGTCAGGGTA	21	19	FGSG_10269	K01509|1|0.0|1066|pic:PICST_30157|adenosinetriphosphatase [EC:3.6.1.3];K11367|3|5e-128|458|cal:CaO19.10553|chromodomain-helicase-DNA-binding protein 1 [EC:3.6.4.12];K11786|5|1e-127|457|ure:UREG_04333|ATP-dependent helicase STH1/SNF2 [EC:3.6.4.-]
siRNA-t0043127	CAAGAGACTTGAACATATTTCCT	23	19	FGSG_10353	K08052|1|1e-22|108|ssl:SS1G_13541|neurofibromin 1
siRNA-t0042411	CAAGCTGGTCTGGGTGCTGA	20	19	FGSG_06976	-
siRNA-t0042406	CAATGTAAGTGACGAGGCCTC	21	19	FGSG_01126	-
siRNA-t0042314	CAGCACCGTACGGATCCACA	20	19	FgHV1gp2	-
siRNA-t0042242	CAGGACCCGAGAAAAACGCCC	21	19	FgHV1gp2	-
siRNA-t0042546	CAGGACCCGAGAAAAACGCCCCTGG	25	19	FgHV1gp2	-
siRNA-t0042084	CAGGATTGAAGTGTAATGCGTTG	23	19	FgHV1gp2	-
siRNA-t0041987	CAGTGTCGAACCATACTGGACCC	23	19	FgHV1gp2	-
siRNA-t0041978	CCAGAAACCCATCCCACTCGT	21	19	FgHV1gp2	-
siRNA-t0041727	CGATAGTGGGAGCGAAAGCG	20	19	FGSG_13563	-
siRNA-t0042778	CTGACAATGGTTTGTTCTGA	20	19	FGSG_08209	K01932|1|0.0|1305|afm:AFUA_5G12730|[EC:6.3.2.-]
siRNA-t0043069	CTGCATGTACTGTTCGGCAACA	22	19	FGSG_01434	-
siRNA-t0043054	GAAGAAGTGACGATACTTGA	20	19	FGSG_02870	K08712|1|0.0|1038|pic:PICST_57529|ATP-binding cassette, subfamily G (WHITE), member 2, SNQ2;K08711|5|0.0|964|cne:CNB05770|ATP-binding cassette, subfamily G (WHITE), member 2, PDR
siRNA-t0042987	GAAGGAACTCCACATAGCGTCGT	23	19	FgHV1gp2	-
siRNA-t0042012	GAAGGGTTCATACTATCGTTG	21	19	FGSG_12536	K07050|1|0.0|709|fgr:FG02991.1|
siRNA-t0043049	GAGAAACAAGTGTCTGCTGG	20	19	FGSG_12904	K12600|1|0.0|2824|fgr:FG06322.1|superkiller protein 3
siRNA-t0042587	GAGCTGTGCGGCAAATGCGA	20	19	FGSG_08840	-
siRNA-t0043081	GATTAATCAATGTACCGTTCGGCA	24	19	FgHV1gp2	-
siRNA-t0042330	GCATTGTATGGTGCTCGGAG	20	19	FGSG_07312	-
siRNA-t0041771	GCGCATACACGGAACCCATT	20	19	FGSG_12002	K03457|1|0.0|1088|fgr:FG01732.1|nucleobase:cation symporter-1, NCS1 family;K09244|2|9e-10|64.7|fgr:FG06915.1|transcriptional regulatory protein AMDR
siRNA-t0041791	GCTCTGTGACTCCAGCCAAAGA	22	19	FgHV1gp2	-
siRNA-t0042707	GGAAGTCCACGATTGTAACG	20	19	FGSG_05306	K08333|1|0.0|3035|fgr:FG05306.1|phosphoinositide-3-kinase, regulatory subunit 4, p150 [EC:2.7.11.1]
siRNA-t0042508	GGATCGGTGGCACACGCATAA	21	19	FGSG_13987	-
siRNA-t0042011	GGATGTTTGCTTGGTTTCGGGACG	24	19	FgHV1gp2	-
siRNA-t0043159	GGGAACACGAATGGGTCTGG	20	19	FGSG_05372	K01932|1|0.0|759|afm:AFUA_1G17200|[EC:6.3.2.-]
siRNA-t0042986	GGGACCGACGTTTTGGCGAG	20	19	FgHV1gp2	-
siRNA-t0042819	GGTACGTCTATATTGTCTCTC	21	19	FgHV1gp2	-
siRNA-t0041634	GGTTCAAGTATCGTCACTTC	20	19	FgHV1gp2	-

siRNA-t0042500	GTACTTGCCGCGCGTCTCACTG	22	19	FGSG_06580	K11262|1|0.0|4510|fgr:FG06580.1|acetyl-CoA carboxylase / biotin carboxylase [EC:6.4.1.2 6.3.4.14]
siRNA-t0042477	GTCGACAGGCGTGGCTGAGA	20	19	FGSG_13189	K01238|1|2e-17|89.4|afm:AFUA_2G04480|[EC:3.2.1.-]
siRNA-t0041764	GTGATACAAACCGGAATGCACTT	23	19	FgHV1gp2	-
siRNA-t0043158	GTTCTCTTTGTTGGCCTGGTA	21	19	FGSG_09560	-
siRNA-t0043063	TACAGGACCGCGCGATGGCG	20	19	FgHV1gp2	-
siRNA-t0041887	TGCAAGAATAGGAAACGGGT	20	19	FGSG_07258	K14572|1|0.0|8396|fgr:FG07258.1|midasin
siRNA-t0042495	TGCATGTAGATCTTGCTGTA	20	19	FGSG_09221	-
siRNA-t0042293	TGCTGCATGTACTGTTCGGCAACA	24	19	FgHV1gp2	-
siRNA-t0042505	TTACAATCGTGGACTTCCTT	20	19	FgHV1gp2	-
siRNA-t0042148	TTAGCTCGATTCTGGCGGCG	20	19	FGSG_10061	-
siRNA-t0043328	AAATCTGGGTGGCGATGGAT	20	18	FGSG_00899	K11430|1|3e-39|163|bfu:BC1G_02412|enhancer of zeste [EC:2.1.1.43]
siRNA-t0043284	AACACGCAGCAGAATTCTAGCCTG	24	18	FgHV1gp2	-
siRNA-t0043540	AACGTGACCAGCTGTTCTCTG	21	18	FgHV1gp2	-
siRNA-t0043535	AAGGCTTGTCGTCGGGAGAATC	22	18	FgHV1gp2	-
siRNA-t0044119	ACAATCGTGGACTTCCTTTAGGA	23	18	FgHV1gp2	-
siRNA-t0044704	ACCCAACACATTTTTGAAGATCGT	24	18	FgHV1gp2	-
siRNA-t0043511	ACGGTACGTGGAAATCTTCAGGCC	24	18	FgHV1gp2	-
siRNA-t0044810	AGAAGATTGAGTTCATTTGTTC	22	18	FGSG_08247	-
siRNA-t0044407	AGAGCTGGGGATGTTTGCTTGGT	23	18	FgHV1gp2	-
siRNA-t0043581	AGATGGACTGGCCTGTTGTCAC	22	18	FgHV1gp2	-
siRNA-t0044448	AGCGAGTGGAGCGTCGGCAAA	21	18	FGSG_04319	K15283|1|0.0|1065|fgr:FG04319.1|solute carrier family 35, member E1
siRNA-t0044861	AGGTGCTGGGTTCTGGTAGA	20	18	FGSG_01321	K05760|1|1e-21|104|mgl:MGL_4132|paxillin
siRNA-t0043471	ATAACACGCAGCAGAATTCTAGCCT	25	18	FgHV1gp2	-
siRNA-t0043559	ATCGGGAATCATGTCGGTGA	20	18	FGSG_09217	K08065|1|2e-08|57.0|cci:CC1G_09213|nuclear transcription Y subunit beta;K14679|2|7e-07|51.6|ppl:POSPLDRAFT_101360|tRNA ligase [EC:6.5.1.3]
siRNA-t0043983	ATGGCGCATACACGGAACCCATTG	24	18	FgHV1gp2	-
siRNA-t0044062	ATTGTCGAGTCGAAAGCGGTC	21	18	FGSG_09146	K00777|1|3e-17|90.9|afm:AFUA_7G07100|[EC:2.4.2.-];K06653|3|7e-12|72.8|vpo:Kpol_1010p59|CDK inhibitor PHO81;K06694|4|2e-11|71.2|pno:SNOG_06697|26S proteasome non-ATPase regulatory subunit 10;K08803|5|2e-11|71.2|fgr:FG07816.1|death-associated protein kinase [EC:2.7.11.1]
siRNA-t0043805	CAGAAGAATTAGCCTTTGAATGCCA	25	18	FgHV1gp2	-
siRNA-t0043518	CAGACACTTGTTTCTCGTGGGATTG	25	18	FgHV1gp2	-
siRNA-t0044002	CCAGAAACCCATCCCACTCG	20	18	FgHV1gp2	-
siRNA-t0044506	CCATCAGGACCCGAGAAAAACGCCC	25	18	FgHV1gp2	-
siRNA-t0044626	CCGGTAAAGCATTGAAGAAAT	21	18	FGSG_00796	-
siRNA-t0044303	CGCAGCAGAATTCTAGCCTGGTTGA	25	18	FgHV1gp2	-
siRNA-t0044454	CGTCTTTTGGATTTGAGGTT	20	18	FGSG_06533	-
siRNA-t0043344	CGTGCGCTTCTGGATCAATGTCA	23	18	FgHV1gp2	-
siRNA-t0044708	CTACAAGGGCGGTTCGAGTAG	21	18	FGSG_05205	K01112|1|2e-178|625|afm:AFUA_1G06860|[EC:3.1.3.-];K01090|2|4e-95|348|spo:SPAC10F6.17c|protein phosphatase [EC:3.1.3.16]
siRNA-t0044186	CTCGATTTCGCAACCACAAGCCA	23	18	FgHV1gp2	-
siRNA-t0043708	CTGATGTGGTGAGGTATGACGTGT	24	18	FgHV1gp2	-
siRNA-t0043609	CTGGATCAATGTCAGCAGTGTTCAT	25	18	FgHV1gp2	-
siRNA-t0043454	CTTGGTTTCGGGACGCGACAAG	22	18	FgHV1gp2	-
siRNA-t0044437	GAATGGTCACCGTCCGGAAT	20	18	FGSG_07422	K15071|1|0.0|1752|fgr:FG07422.1|recyclin-1
siRNA-t0043434	GAGAACGCTGAAATCGAGGGTGTT	24	18	FgHV1gp2	-
siRNA-t0044191	GAGCAGGATTGAAGTGTAAT	20	18	FgHV1gp2	-
siRNA-t0044478	GAGCGTGTAGGGCTGTCCCAT	21	18	FGSG_05297	K14676|1|0.0|2814|fgr:FG05297.1|lysophospholipid hydrolase [EC:3.1.1.5]
siRNA-t0043276	GATCTGGCTCGTGACTTTTCCCT	23	18	FgHV1gp2	-
siRNA-t0044828	GATGATCATGACGTGGAGAA	20	18	FGSG_10530	-
siRNA-t0044614	GATTTGCGACGTGCTTGAAGGCGA	24	18	FgHV1gp2	-
siRNA-t0044872	GCAGTTGGTCCGCGAATTCTGC	22	18	FgHV1gp2	-
siRNA-t0043643	GCGGCAAATACAGGACCGCGCGAT	24	18	FgHV1gp2	-
siRNA-t0043935	GGAGACGGCAGGTAACTCCTTC	22	18	FGSG_11154	-
siRNA-t0043981	GGATCTCTGACATATTCGGACT	22	18	FgHV1gp2	-
siRNA-t0043691	GGATTGGGCGTGTGTTCTTTATGG	24	18	FgHV1gp2	-
siRNA-t0044705	GTCATGATCATCAGCACCGTAC	22	18	FGSG_04291	K02999|1|0.0|3375|fgr:FG04291.1|DNA-directed RNA polymerase I subunit RPA1 [EC:2.7.7.6]
siRNA-t0043461	GTCTTCTGACGAATTTGGACA	21	18	FGSG_10174	K11367|1|2e-107|390|scm:SCHCODRAFT_58011|chromodomain-helicase-DNA-binding protein 1 [EC:3.6.4.12]
siRNA-t0044784	GTGCCATGACGTACGACCCCGAAA	24	18	FgHV1gp1	-
siRNA-t0043205	GTGGAAATCTTCAGGCCCTGGGAT	24	18	FgHV1gp2	-
siRNA-t0043313	TCGGTAGTCATCGGGAATCAT	21	18	FgHV1gp2	-
siRNA-t0043615	TCTACCAGAACCCAGCACCTTTCCC	25	18	FgHV1gp2	-
siRNA-t0043711	TCTCGGGTCCTGATGGTTTCAGA	23	18	FGSG_06016	K05727|1|1e-46|188|mpr:MPER_07660|dedicator of cytokinesis 3/4/5
siRNA-t0043752	TCTTCGATGGATGCTGCGAAACCCA	25	18	FgHV1gp2	-
siRNA-t0044348	TCTTGAAATGATTTGCGACGTGCT	24	18	FgHV1gp2	-
siRNA-t0044731	TGCTGCATGTACTGTTCGGCAA	22	18	FgHV1gp2	-
siRNA-t0043923	TGGAGTGGCATATTCAAGAA	20	18	FGSG_05571	K05658|1|6e-45|182|scm:SCHCODRAFT_52512|ATP-binding cassette, subfamily B (MDR/TAP), member 1
siRNA-t0043293	TGGGATTGGGCGTGTGTTCTTTA	23	18	FgHV1gp2	-
siRNA-t0043618	TGGGTTGGTAATTGACAAAGGCCTA	25	18	FgHV1gp2	-
siRNA-t0044788	TGTTCTCTGTCGGCATGTTGC	21	18	FGSG_11657	K09184|1|5e-13|75.9|bfu:BC1G_09852|GATA-binding protein, other eukaryote
siRNA-t0043598	TTCTTGGAAAGTTGGAGCCC	20	18	FGSG_10683	-
siRNA-t0046143	AAATGATTTGCGACGTGCTTGAA	23	17	FgHV1gp2	-
siRNA-t0046112	AAGGCTTGTCGTCGGGAGAATCCA	24	17	FgHV1gp2	-
siRNA-t0046506	AAGTCTGGAATTCCTTGGGAGGC	23	17	FgHV1gp2	-
siRNA-t0045339	AATCGATCGTCATGGTGATA	20	17	FGSG_10464	K15394|1|0.0|1754|act:ACLA_078660|hybrid polyketide synthase / nonribosomal peptide synthetase ACE1
siRNA-t0047107	ACAGTTCCGCGTGTGATTGGTCTCT	25	17	FgHV1gp2	-
siRNA-t0045174	ACCTATACTTGATCTCAGCAC	21	17	FgHV1gp2	-
siRNA-t0046505	ACGATGACTACGATGATGATT	21	17	FGSG_13783	K01932|1|0.0|1526|afm:AFUA_5G12730|[EC:6.3.2.-]
siRNA-t0045098	ACGCAGCAGAATTCTAGCCTG	21	17	FgHV1gp2	-
siRNA-t0045867	AGAATGTCGAGATACTCTTCA	21	17	FGSG_13169	K00750|1|4e-16|84.7|cdu:CD36_86420|glycogenin glucosyltransferase [EC:2.4.1.186]
siRNA-t0046141	AGCGTGTAGGGCTGTCCCATA	21	17	FGSG_05297	K14676|1|0.0|2814|fgr:FG05297.1|lysophospholipid hydrolase [EC:3.1.1.5]
siRNA-t0046627	AGCGTGTTGTACGGCGTGGTCTCG	24	17	FgHV1gp2	-
siRNA-t0045801	AGGACCAGTTCCTGCTGAGTGTGG	24	17	FgHV1gp2	-
siRNA-t0046413	AGGGCCTGAAGATTTCCACG	20	17	FGSG_07104	K13338|1|0.0|2298|fgr:FG07104.1|peroxin-1
siRNA-t0045382	AGTGTTCGCATCCGTTCTGTG	21	17	FGSG_08999	-
siRNA-t0046123	ATCATCAGCACCGTACGGATCCA	23	17	FgHV1gp2	-
siRNA-t0045244	ATCTGGCCGGTTGAAAATGTT	21	17	FgHV1gp2	-
siRNA-t0046791	ATGCTTTCGACGTACTTGGGTT	22	17	FgHV1gp2	-
siRNA-t0046961	CAAAATGCCATGGGATCGAAGG	22	17	FGSG_09064	-
siRNA-t0046382	CAATCACACGCGGAACTGTCT	21	17	FGSG_02482	K00381|1|0.0|3097|fgr:FG02482.1|sulfite reductase (NADPH) hemoprotein beta-component [EC:1.8.1.2]
siRNA-t0046243	CAGAGTCTTGCGGTGTGGGTC	21	17	FgHV1gp2	-
siRNA-t0045363	CATACCTTTGAGCTCCTTGAG	21	17	FGSG_05519	K04728|1|0.0|5526|fgr:FG05519.1|ataxia telangiectasia mutated family protein [EC:2.7.11.1]
siRNA-t0046920	CCCGACTACTGAATGCGAGG	20	17	FGSG_13086	-
siRNA-t0045599	CCGACAATGGCGCATACACGGA	22	17	FgHV1gp2	-
siRNA-t0045624	CCGCAGTGATGGAGACTAAACG	22	17	FGSG_09869	K12669|1|0.0|647|fgr:FG09869.1|oligosaccharyltransferase complex subunit gamma
siRNA-t0046736	CCGGAATGCGTTCACACGATTCC	23	17	FgHV1gp2	-

siRNA-t0045755	CCGGTAAAGCATTGAAGAAA	20	17	FGSG_00796	-
siRNA-t0045991	CCTAGACCGAAGAAAGACCACATC	24	17	FgHV1gp2	-
siRNA-t0046434	CGAACCATACTGGACCCAGAT	21	17	FgHV1gp2	-
siRNA-t0046207	CGACAATGGCGCATACACGGAACCC	25	17	FgHV1gp2	-
siRNA-t0045765	CGAGGTTATGGCAGAATTCG	20	17	FGSG_08539	-
siRNA-t0045781	CGGTGTGGGTCTTCTGACGAAT	22	17	FgHV1gp2	-
siRNA-t0045707	CTAGACCGAAGAAAGACCACAT	22	17	FgHV1gp2	-
siRNA-t0046218	CTTCAGGCCCTGGGATTGTATGG	23	17	FgHV1gp2	-
siRNA-t0046114	GAACAATTTATCGTGCGCTTCTGG	24	17	FgHV1gp2	-
siRNA-t0046689	GAAGTCCACGATTGTAACGAAT	22	17	FGSG_05306	K08333|1|0.0|3035|fgr:FG05306.1|phosphoinositide-3-kinase, regulatory subunit 4, p150 [EC:2.7.11.1]
siRNA-t0045716	GATGATATTCGGTATTGTCGAG	22	17	FgHV1gp2	-
siRNA-t0046756	GCATGGATGGTGATAGACTTT	21	17	FGSG_08904	-
siRNA-t0046855	GCGACCCATGCTTTCGACGTACTTG	25	17	FgHV1gp2	-
siRNA-t0047104	GCGGAGATCCGGTAAAGCAT	20	17	FGSG_06511	-
siRNA-t0046811	GGAACTCCACATAGCGTCGTC	21	17	FgHV1gp2	-
siRNA-t0047119	GGCATGAACTCGGTCAACGAA	21	17	FgHV1gp2	-
siRNA-t0046341	GGGTCTAAAGGAGTGGATCGT	21	17	FGSG_04951	K14327|1|1e-87|322|ang:ANI_1_332064|regulator of nonsense transcripts 2
siRNA-t0045983	GGGTTCTTGAAGTTGGTGCCG	21	17	FgHV1gp2	-
siRNA-t0045840	GGTACGTCTATATTGTCTCTCA	22	17	FgHV1gp2	-
siRNA-t0046268	GGTTCAATATCGGTACCAGA	20	17	FGSG_08426	K12659|1|0.0|960|fgr:FG08426.1|N-acetyl-gamma-glutamyl-phosphate reductase / acetylglutamate kinase [EC:1.2.1.38 2.7.2.8]
siRNA-t0046907	GTAATCATCATCGTAGTCATCGTCA	25	17	FGSG_09900	-
siRNA-t0046371	GTCATGATCATCAGCACCGT	20	17	FGSG_11998	-
siRNA-t0045965	GTCATGATCATCAGCACCGTA	21	17	FGSG_04291	K02999|1|0.0|3375|fgr:FG04291.1|DNA-directed RNA polymerase I subunit RPA1 [EC:2.7.7.6]
siRNA-t0046313	GTGGGATTGGGCGTGTGTTCTTTAT	25	17	FgHV1gp2	-
siRNA-t0046045	GTGGTGAGGTATGACGTGTTCATT	24	17	FgHV1gp2	-
siRNA-t0045949	GTGGTGAGGTATGACGTGTTCATTA	25	17	FgHV1gp2	-
siRNA-t0046171	TCCAATGAGTTGTGGGAGCA	20	17	FGSG_11550	K03379|1|4e-100|364|mgr:MGG_06577|cyclohexanone monooxygenase [EC:1.14.13.22];K00540|4|2e-38|160|afm:AFUA_8G00440|[EC:1.-.-.-]
siRNA-t0045847	TCCATGCATGTAGATCTTGCTGT	23	17	FgHV1gp2	-
siRNA-t0046874	TCTTTGTTGGCCTGGTAATA	20	17	FGSG_14023	-
siRNA-t0045665	TGATAAATGAACGTCTGACTGTA	23	17	FgHV1gp2	-
siRNA-t0045131	TGTGTCGCGTCTACCTTGTT	20	17	FgHV1gp2	-
siRNA-t0045318	TGTTGCTTGATGGTTGGCAC	20	17	FgHV1gp2	-
siRNA-t0046828	TTCAGGCCCTGGGATTGTATGGT	23	17	FgHV1gp2	-
siRNA-t0046125	TTCTTGGAAAGTTGGAGCCCTT	22	17	FgHV1gp2	-
siRNA-t0046469	TTGCACGAGGGACGAGCTTG	20	17	FGSG_11987	K15441|1|2e-41|169|uma:UM00197.1|tRNA-specific adenosine deaminase 2 [EC:3.5.4.-]
siRNA-t0047246	AAACGCTGCCGGGATGTAATCA	22	16	FgHV1gp2	-
siRNA-t0048023	AAACTGTTCATTTCGGCGAG	20	16	FGSG_07722	K03448|1|2e-21|103|pcs:Pc22g12690|MFS transporter, ACS family, pantothenate transporter;K13336|2|8e-19|94.4|afv:AFLA_048740|peroxin-3;K15361|3|7e-17|88.2|cim:CIMG_09192|WD repeat-containing protein 48;K11369|4|1e-16|87.4|ani:AN3929.2|retrograde regulation protein 2
siRNA-t0047690	AAAGTGTCGTAGGAAAGGAAAA	22	16	FgHV1gp1	-
siRNA-t0048227	AACGGTTCTTGGAAAGTTGGAG	22	16	FgHV1gp2	-
siRNA-t0047798	AACTGCGACCCATGCTTTCGACGTA	25	16	FgHV1gp2	-
siRNA-t0047586	AATCGATCGTCATGGTGATACCC	23	16	FgHV1gp2	-
siRNA-t0049190	ACAATTGTCGTGCGTTCCGTG	21	16	FGSG_01049	-
siRNA-t0047925	ACACGCAGCAGAATTCTAGC	20	16	FgHV1gp2	-
siRNA-t0048437	ACACGCAGCAGAATTCTAGCCTG	23	16	FgHV1gp2	-
siRNA-t0049199	ACTGCAACAGTTCACCGACAT	21	16	FGSG_07469	K01553|1|0.0|1395|pic:PICST_75361|myosin ATPase [EC:3.6.4.1];K10352|2|1e-149|531|pno:SNOG_09711|myosin heavy chain
siRNA-t0049351	AGGACCCGAGAAAAACGCCCCTG	23	16	FgHV1gp2	-
siRNA-t0049029	AGGAGCTCAAAGGTATGATG	20	16	FGSG_08060	K00688|1|4e-11|69.3|pno:SNOG_13460|starch phosphorylase [EC:2.4.1.1]
siRNA-t0048905	AGTGTGTCGCGTCTACCTTGTT	22	16	FgHV1gp2	-
siRNA-t0047175	ATCTAGTTCGAAGGAGGCGGAA	22	16	FGSG_11206	-
siRNA-t0047504	ATGATTTGCGACGTGCTTGAA	21	16	FgHV1gp2	-
siRNA-t0049163	ATGGCGCATACACGGAACCCATT	23	16	FgHV1gp2	-
siRNA-t0048588	CAGCACCGTACGGATCCACAT	21	16	FgHV1gp2	-
siRNA-t0048025	CAGGATTGAAGTGTAATGCGTT	22	16	FgHV1gp2	-
siRNA-t0047174	CAGTTCTCTTTGTTGGCCTGG	21	16	FGSG_09560	-
siRNA-t0048214	CCAACACATTTTTGAAGATCGT	22	16	FgHV1gp2	-
siRNA-t0047359	CCACTCGTGAACCTGTGGTGTC	22	16	FgHV1gp2	-
siRNA-t0048608	CCCGAGAAAAACGCCCCTGGACGG	24	16	FgHV1gp2	-
siRNA-t0048708	CCCTCAGTAGTCGACATGTC	20	16	FGSG_09197	K00021|1|0.0|2147|fgr:FG09197.1|hydroxymethylglutaryl-CoA reductase (NADPH) [EC:1.1.1.34]
siRNA-t0048525	CCGTTTGTGCTGCAAGATGGACTGG	25	16	FgHV1gp2	-
siRNA-t0047220	CCTAGACCGAAGAAAGACCA	20	16	FgHV1gp2	-
siRNA-t0049221	CCTCGCATTCAGTAGTCGGG	20	16	FGSG_08719	K10352|1|0.0|4469|fgr:FG08719.1|myosin heavy chain
siRNA-t0047418	CGACCCATGCTTTCGACGTACTTG	24	16	FgHV1gp2	-
siRNA-t0048864	CGAGCAGGATTGAAGTGTAATGC	23	16	FgHV1gp2	-
siRNA-t0048289	CGCGCTGTACTGAAGGTGGTGTCA	24	16	FgHV1gp2	-
siRNA-t0048991	CGCTGATCTGGCTCGTGACTTTT	23	16	FgHV1gp2	-
siRNA-t0047729	CGGTACGTGGAAATCTTCAGGC	22	16	FGSG_11796	-
siRNA-t0048811	CGTGCGCTTCTGGATCAATGT	21	16	FGSG_04371	-
siRNA-t0047373	CGTGGGATTGGGCGTGTGTTCTTT	24	16	FgHV1gp2	-
siRNA-t0047593	CTATAGTAGATCGGGCAGAT	20	16	FGSG_04385	-
siRNA-t0047993	CTTGATCTCAGCACGCCGGT	20	16	FGSG_10904	K06681|1|1e-61|238|cdu:CD36_21160|Gdp/GTP exchange factor required for growth at low temperatures
siRNA-t0048313	CTTGGTTTCGGGACGCGACAAGC	23	16	FgHV1gp2	-
siRNA-t0048936	GAACGGGACCGACGTTTTGGCGA	23	16	FgHV1gp2	-
siRNA-t0047245	GAAGAAAGGTGCGAGCAGGA	20	16	FGSG_06342	K01027|1|0.0|1027|fgr:FG06342.1|3-oxoacid CoA-transferase [EC:2.8.3.5]
siRNA-t0047620	GACCCATGCTTTCGACGTACTT	22	16	FgHV1gp2	-
siRNA-t0048504	GAGACGGCAGGTAACTCCTTC	21	16	FGSG_11154	-
siRNA-t0048131	GAGCGTGATGTGGTGTTATT	20	16	FGSG_02315	K01932|1|0.0|2455|afm:AFUA_1G10380|[EC:6.3.2.-]
siRNA-t0048884	GAGCGTGTAGGGCTGTCCCA	20	16	FGSG_10021	-
siRNA-t0048544	GAGCTGGGGATGTTTGCTTGGTTT	24	16	FgHV1gp2	-
siRNA-t0048921	GAGTGTGTCGCGTCTACCTTGT	22	16	FgHV1gp2	-
siRNA-t0048987	GATACAAACCGGAATGCACTT	21	16	FgHV1gp2	-
siRNA-t0049089	GCAAAAGACAGATAGGTCGCA	21	16	FgHV1gp2	-
siRNA-t0048971	GCAACGAGGGCCCAGTCATGAATTT	25	16	FgHV1gp2	-
siRNA-t0047763	GCCGAACAGTACATGCAGCA	20	16	FGSG_06900	K03639|1|0.0|1291|fgr:FG06900.1|molybdenum cofactor biosynthesis protein
siRNA-t0047609	GCTGTAGTGTCTGAAGATCTCAGAA	25	16	FgHV1gp2	-
siRNA-t0047982	GGCCAAATCGTCATACATTGC	21	16	FgHV1gp2	-
siRNA-t0047250	GGGGTCTTGACTTGGGCTGTACC	23	16	FGSG_09304	-
siRNA-t0047945	GGGTTCAATATCGGTACCAGA	21	16	FGSG_08426	K12659|1|0.0|960|fgr:FG08426.1|N-acetyl-gamma-glutamyl-phosphate reductase / acetylglutamate kinase [EC:1.2.1.38 2.7.2.8]
siRNA-t0047286	GGGTTCTTGAAGTTGGTGCCGAC	23	16	FgHV1gp2	-
siRNA-t0047515	GGTTTGAACCCTGACAATACCAA	23	16	FgHV1gp2	-
siRNA-t0048600	GTACTTGTCCAAATTCGTCAGAA	23	16	FGSG_09244	-
siRNA-t0048017	GTAGGCTCCTCCGACAATGGCGCAT	25	16	FgHV1gp2	-
siRNA-t0049117	GTCATGATCATCAGCACCGTACG	23	16	FgHV1gp2	-
siRNA-t0048716	TAGCCATTCGTTACAATCGTGGAC	24	16	FgHV1gp2	-
siRNA-t0049414	TCTTGCGGTGTGGGTCTTCTGAC	23	16	FgHV1gp2	-

siRNA-t0049281	TGCTGCATGTACTGTTCGGCAAC	23	16	FgHV1gp2	-
siRNA-t0047886	TGCTTGATGGTTGGCACAACGAT	23	16	FgHV1gp2	-
siRNA-t0048280	TGCTTGGTTTCGGGACGCGACAAGC	25	16	FgHV1gp2	-
siRNA-t0048494	TGCTTTCGACGTACTTGGGTTCATA	25	16	FgHV1gp2	-
siRNA-t0047666	TGGATGTGGGTGCGCCGAAT	20	16	FGSG_06562	-
siRNA-t0047190	TGGCCAAATCGTCATACATTGCAC	24	16	FgHV1gp2	-
siRNA-t0047376	TGGTTCAAGTATCGTCACTTCTTC	24	16	FgHV1gp2	-
siRNA-t0049173	TTGGCATAGAATCGCGCTTG	20	16	FGSG_06468	K11369|1|1e-56|220|ani:AN3929.2|retrograde regulation protein 2;K13336|2|4e-56|218|afv:AFLA_048740|peroxin-3;K03448|3|2e-42|172|cpw:CPC735_001260|MFS transporter, ACS family, pantothenate transporter
siRNA-t0051113	AAGTAAGCAGAGCGTGTAGGGCTG	24	15	FgHV1gp2	-
siRNA-t0050151	ACTGCGACCCATGCTTTCGACGT	23	15	FgHV1gp2	-
siRNA-t0049949	ACTGCGGTACGATATTTGAG	20	15	FGSG_01883	-
siRNA-t0051245	AGAAGGGCTCCAACTTTCCA	20	15	FGSG_12634	-
siRNA-t0051325	AGACACTTGTTTCTCGTGGGATTGG	25	15	FgHV1gp2	-
siRNA-t0050911	AGAGACAGAGCTCGCGCCTT	20	15	FGSG_13273	-
siRNA-t0052006	AGATGTAGGCTCCTCCGACAAT	22	15	FGSG_11268	-
siRNA-t0049894	AGCAGAATTCTAGCCTGGTTG	21	15	FgHV1gp2	-
siRNA-t0049865	AGCCATTCGTTACAATCGTGGACT	24	15	FgHV1gp2	-
siRNA-t0050909	AGGATCTCTGACATATTCGGACT	23	15	FgHV1gp2	-
siRNA-t0050637	AGTCTCTTGTTCTGGAACGTCA	22	15	FGSG_04168	K12494|1|4e-11|70.9|cnb:CNBD1140|PH and SEC7 domain-containing protein
siRNA-t0051549	AGTGTCGTAGGAAAGGAAAAGT	22	15	FgHV1gp1	-
siRNA-t0049997	ATGATGGCACGTCGACAGGC	20	15	FgHV1gp2	-
siRNA-t0051181	ATGCTTTCGACGTACTTGGGT	21	15	FgHV1gp2	-
siRNA-t0051998	CAAGGATTTGTAAGGGGAAACG	22	15	FGSG_06885	K01613|1|5e-08|60.5|pte:PTT_10674|phosphatidylserine decarboxylase [EC:4.1.1.65]
siRNA-t0051731	CAATCGTGGACTTCCTTTAGGAA	23	15	FgHV1gp2	-
siRNA-t0050404	CAATGGCGACACCGACGGTA	20	15	FGSG_05709	K13830|1|2e-68|259|lbc:LACBIDRAFT_233717|pentafunctional AROM polypeptide [EC:4.2.3.4 4.2.1.10 1.1.1.25 2.7.1.71 2.5.1.19]
siRNA-t0051737	CAGGACCCGAGAAAAACGCCCCT	23	15	FgHV1gp2	-
siRNA-t0051089	CATTCAGTAGTCGGGGTCTC	20	15	FgHV1gp1	-
siRNA-t0051226	CCATGCAGCGGCAAATACAGGACC	24	15	FgHV1gp2	-
siRNA-t0049750	CCATGCTTTCGACGTACTTGGGT	23	15	FgHV1gp2	-
siRNA-t0051453	CCATTGCTGCATGTACTGTT	20	15	FGSG_05795	-
siRNA-t0049775	CCGGCGTGCTGAGATCAAGTA	21	15	FGSG_02554	-
siRNA-t0049764	CGAAGACCAAAAATTTCTTCA	21	15	FgHV1gp1	-
siRNA-t0050361	CGGACTCATCCCACATGATGG	21	15	FGSG_02113	K14338|1|2e-18|94.0|mgr:MGG_10879|cytochrome P450 / NADPH-cytochrome P450 reductase [EC:1.14.14.1 1.6.2.4];K00495|4|2e-17|90.5|uma:UM06473.1|[EC:1.14.14.-]
siRNA-t0051912	CGGATACGTTCTGAGAAGTCTCCGA	25	15	FgHV1gp2	-
siRNA-t0051935	CGGCAAATACAGGACCGCGCGAT	23	15	FgHV1gp2	-
siRNA-t0050231	CGGTGTGGGTCTTCTGACGA	20	15	FGSG_07081	-
siRNA-t0050313	CGTGAACCAGATCATAACCCGCAT	24	15	FgHV1gp2	-
siRNA-t0049544	CTGACATATTCGGACTCATCCCA	23	15	FgHV1gp2	-
siRNA-t0050284	CTGGACGAAGACTTGCGTACC	21	15	FGSG_07962	K10875|1|0.0|1660|fgr:FG07962.1|DNA repair and recombination protein RAD54 and RAD54-like protein [EC:3.6.4.-]
siRNA-t0050648	CTGTTGACGTCACCCTTTGG	20	15	FgHV1gp2	-
siRNA-t0051762	CTTGGTTTCGGGACGCGACAA	21	15	FgHV1gp2	-
siRNA-t0050508	GAAAACTATAGTCGGTCAGG	20	15	FGSG_04627	K00936|1|6e-80|298|afm:AFUA_8G06140|[EC:2.7.3.-];K02486|2|4e-15|83.6|spo:SPAC27E2.09|two-component system, unclassified family, sensor kinase [EC:2.7.13.3];K11233|5|7e-12|72.8|cgr:CAGL0D02882g|osomolarity two-component system, response regulator SSK1
siRNA-t0051099	GAAATGATTTGCGACGTGCTTGA	23	15	FgHV1gp2	-
siRNA-t0050560	GAAGTTGGTGCCGACGTCTA	20	15	FGSG_10168	-
siRNA-t0051652	GACATATTCGGACTCATCCC	20	15	FgHV1gp2	-
siRNA-t0049580	GACGATGACTACGATGATGAT	21	15	FGSG_13783	K01932|1|0.0|1526|afm:AFUA_5G12730|[EC:6.3.2.-]
siRNA-t0049855	GAGTGGCAGTGTAATGGAGGACG	23	15	FgHV1gp2	-
siRNA-t0051801	GAGTGTGTCGCGTCTACCTTGTTC	24	15	FgHV1gp2	-
siRNA-t0051024	GATCATCAGCACCGTACGGATC	22	15	FgHV1gp2	-
siRNA-t0050895	GCAAGAAGTTTGCATCCTCGACCCA	25	15	FgHV1gp2	-
siRNA-t0051316	GCAGAAGAATTAGCCTTTGAAT	22	15	FGSG_12776	K12879|1|0.0|2720|fgr:FG05657.1|THO complex subunit 2
siRNA-t0051831	GCCTGTGCCGGTGTCGGCTGCC	22	15	FgHV1gp2	-
siRNA-t0051510	GCGACGACCGTGCGAAATTGTTT	23	15	FgHV1gp2	-
siRNA-t0049583	GCTGCATGTACTGTTCGGCAA	21	15	FGSG_08421	K13126|1|0.0|636|fgr:FG08421.1|polyadenylate-binding protein
siRNA-t0051383	GGAAATATCGGAAAATTGTGTTGAT	25	15	FgHV1gp2	-
siRNA-t0051169	GGATGTTTGCTTGGTTTCGGGA	22	15	FgHV1gp2	-
siRNA-t0051871	GGGGTCTCCGATATTCGCTT	20	15	FGSG_06583	-
siRNA-t0051896	GGTACGTCTATATTGTCTCT	20	15	FgHV1gp2	-
siRNA-t0051443	GTACGTGGAAATCTTCAGGCC	21	15	FgHV1gp2	-
siRNA-t0051660	GTAGATCTTGCTGTAGTGTCTGA	23	15	FgHV1gp2	-
siRNA-t0050040	GTCTTTCTTCGGTCTAGGTA	20	15	FGSG_00643	-
siRNA-t0051571	GTGGGTCTTCTGACGAATTTGGAC	24	15	FgHV1gp2	-
siRNA-t0050984	TACCTAGACCGAAGAAAGACCA	22	15	FgHV1gp2	-
siRNA-t0050759	TCCGAATATGTCAGAGATCCTGCT	24	15	FGSG_09665	-
siRNA-t0050645	TCGCTCCCACTATCGTCCTGC	21	15	FGSG_01294	K00799|1|1e-34|146|afv:AFLA_022030|glutathione S-transferase [EC:2.5.1.18]
siRNA-t0050757	TGATCATGACGTGGAGAAGA	20	15	FGSG_07536	K03386|1|2e-83|307|ang:ANI_1_984094|peroxiredoxin (alkyl hydroperoxide reductase subunit C) [EC:1.11.1.15]
siRNA-t0050726	TGCGTACCTGCAGTCACAACTT	22	15	FGSG_01790	K00680|1|0.0|2387|afm:AFUA_3G14700|[EC:2.3.1.-];K15419|2|0.0|1077|ani:AN2547.2|emericellamide biosynthesis, highly reducing iterative type I polyketide synthase;K15384|5|0.0|762|afv:AFLA_066840|aspyridone synthetase, hybrid polyketide synthase / nonribosomal peptide synthetase
siRNA-t0051346	TGCTGCAAGATGGACTGGCCTGTTG	25	15	FgHV1gp2	-
siRNA-t0051361	TGCTTGGTTTCGGGACGCGACA	22	15	FgHV1gp2	-
siRNA-t0050229	TGGCCGGTTGAAAATGTTCCAC	22	15	FgHV1gp2	-
siRNA-t0051570	TGGCGACACCGACGGTACGTGGAA	24	15	FgHV1gp2	-
siRNA-t0051531	TGTGTGAAGTAGTGCCCGTC	20	15	FGSG_04474	-
siRNA-t0053200	AAAGCTTACCTAGACCGAAGAA	22	14	FgHV1gp2	-
siRNA-t0052400	AAAGGTGCGAGCAGGATTGAAG	22	14	FgHV1gp2	-
siRNA-t0052812	AACACGCAGCAGAATTCTAGC	21	14	FgHV1gp2	-
siRNA-t0052088	AAGGTGCTGGGTTCTGGTAGA	21	14	FGSG_01321	K05760|1|1e-21|104|mgl:MGL_4132|paxillin
siRNA-t0052201	AAGTCTCTTGTTCTGGAACGTC	22	14	FgHV1gp2	-
siRNA-t0052766	ACGACAATTGTCTCGAGCAA	20	14	FGSG_03589	K01904|1|2e-154|545|afm:AFUA_1G13110|4-coumarate--CoA ligase [EC:6.2.1.12];K14818|2|1e-74|280|pno:SNOG_03246|ribosome assembly protein SQT1;K01897|3|2e-32|140|afm:AFUA_1G17190|long-chain acyl-CoA synthetase [EC:6.2.1.3];K01976|5|2e-26|120|ago:AGOS_AAR168C|[EC:6.-.-.-]
siRNA-t0052855	ACGCAGCAGAATTCTAGCCTGG	22	14	FgHV1gp2	-
siRNA-t0053362	ACGTACCGTCGGTGTCGCCA	20	14	FGSG_08320	K00495|1|4e-93|341|afm:AFUA_1G01690|[EC:1.14.14.-]
siRNA-t0054419	ACGTGGAAATCTTCAGGCCCT	21	14	FgHV1gp2	-
siRNA-t0053218	ACTGTTGACGTCACCCTTTGGAG	23	14	FgHV1gp2	-
siRNA-t0053605	AGAACATCAGGCATGTATGGGT	22	14	FGSG_01948	K14327|1|0.0|2080|fgr:FG01948.1|regulator of nonsense transcripts 2
siRNA-t0053085	AGAAGAATTAGCCTTTGAATGCCAG	25	14	FgHV1gp2	-
siRNA-t0052476	AGCCATTCGTTACAATCGTGGACTT	25	14	FgHV1gp2	-

siRNA-t0053439	AGGCTTGTCGTCGGGAGAATCC	22	14	FgHV1gp2	-
siRNA-t0053302	AGGGTTCATACTATCGTTGT	20	14	FGSG_12536	K07050|1|0.0|709|fgr:FG02991.1|
siRNA-t0053417	AGTTATCGTGTCTGACTGTTGCTT	24	14	FgHV1gp2	-
siRNA-t0053739	ATCATTCCATTGATAATCGGCA	22	14	FgHV1gp2	-
siRNA-t0054761	CAACGTGGATTTTGCTACCAG	21	14	FgHV1gp1	-
siRNA-t0054659	CACTCGTGAACCTGTGGTGTC	21	14	FgHV1gp2	-
siRNA-t0053123	CAGCAGAATTCTAGCCTGGTTG	22	14	FgHV1gp2	-
siRNA-t0054286	CAGTGAGACGCGCGGCAAGT	20	14	FGSG_07469	K01553|1|0.0|1395|pic:PICST_75361|myosin ATPase [EC:3.6.4.1];K10352|2|1e-149|531|pno:SNOG_09711|myosin heavy chain
siRNA-t0054517	CCAAGTAAGCAGAGCGTGTAGGG	23	14	FgHV1gp2	-
siRNA-t0052464	CCAGATCTTATAGACAGGCCC	21	14	FgHV1gp2	-
siRNA-t0052309	CCAGTGTCGAACCATACTGGA	21	14	FgHV1gp2	-
siRNA-t0052992	CCGACATGTTTAGGATCACC	20	14	FGSG_03933	K12618|1|1e-55|215|ani:AN8185.2|5'-3' exoribonuclease 1 [EC:3.1.13.-]
siRNA-t0053756	CCGAGAAAAACGCCCCTGGACGG	23	14	FgHV1gp2	-
siRNA-t0053606	CCGGAATGCGTTCACACGATTCCC	24	14	FgHV1gp2	-
siRNA-t0052085	CCGTTGTGTGCTGCAAGATGGAC	22	14	FgHV1gp2	-
siRNA-t0052727	CGAAGAAGTGACGATACTTGAA	22	14	FGSG_03153	-
siRNA-t0052131	CGACGGTACGTGGAAATCTTCA	22	14	FgHV1gp2	-
siRNA-t0053348	CGAGAAAAACGCCCCTGGACGG	22	14	FgHV1gp2	-
siRNA-t0053014	CTCGGGTCCTGATGGTTTCAGA	22	14	FGSG_06016	K05727|1|1e-46|188|mpr:MPER_07660|dedicator of cytokinesis 3/4/5
siRNA-t0052255	CTGCGACCCATGCTTTCGACGT	22	14	FgHV1gp2	-
siRNA-t0052155	GACAAGCCGTCAAACATAGACA	22	14	FgHV1gp2	-
siRNA-t0054171	GACGGTACGTGGAAATCTTCAGGCC	25	14	FgHV1gp2	-
siRNA-t0052163	GAGTTGGGAACGAGGCATCG	20	14	FGSG_09910	-
siRNA-t0053836	GAGTTGTGGGAGCAAGAGAA	20	14	FGSG_13987	-
siRNA-t0053319	GATACAAACCGGAATGCACTTT	22	14	FgHV1gp2	-
siRNA-t0054738	GCAAGCGTGACGAAGATCCCACTC	24	14	FgHV1gp1	-
siRNA-t0054418	GCAGAATTCTAGCCTGGTTGAGA	23	14	FgHV1gp2	-
siRNA-t0052245	GCATGTAGATCTTGCTGTAGT	21	14	FgHV1gp2	-
siRNA-t0052449	GGGATCAGGTTCACAATTGCA	21	14	FgHV1gp2	-
siRNA-t0053012	GGGATTGGGCGTGTGTTCTTTATGG	25	14	FgHV1gp2	-
siRNA-t0054708	GGGTCTTCTGACGAATTTGGA	21	14	FgHV1gp2	-
siRNA-t0054116	GTGGGATTGGGCGTGTGTTCTTTA	24	14	FgHV1gp2	-
siRNA-t0052401	GTTCTTGGAAAGTTGGAGCCCT	22	14	FgHV1gp2	-
siRNA-t0052465	TACGTAAGGCGTCGACACGAT	21	14	FgHV1gp2	-
siRNA-t0053141	TCTGGCATAACGACGTTCTT	20	14	FGSG_13662	K03018|1|0.0|2938|fgr:FG09860.1|DNA-directed RNA polymerase III subunit RPC1 [EC:2.7.7.6]
siRNA-t0053166	TGCGAGGCCATAGGGAAGAT	20	14	FGSG_07267	K10875|1|8e-73|275|mgr:MGG_03549|DNA repair and recombination protein RAD54 and RAD54-like protein [EC:3.6.4.-]
siRNA-t0052799	TGCTGCAAGATGGACTGGCCTGTT	24	14	FgHV1gp2	-
siRNA-t0052770	TGGAGTGGCATATTCAAGAATCAA	24	14	FgHV1gp2	-
siRNA-t0053408	TGGCCCCAGCAGACTTGGAA	20	14	FGSG_12904	K12600|1|0.0|2824|fgr:FG06322.1|superkiller protein 3
siRNA-t0054347	TGGGTCGAGGATGCAAACTT	20	14	FGSG_07064	-
siRNA-t0054580	TGTTGAGAACGCTGAAATCGAGGG	24	14	FgHV1gp2	-
siRNA-t0054184	TTCGTTGAATCGATCGTCATGG	22	14	FgHV1gp2	-
siRNA-t0054587	TTCGTTGAATCGATCGTCATGGT	23	14	FgHV1gp2	-
siRNA-t0052471	TTGAATCGATCGTCATGGTGATACC	25	14	FgHV1gp2	-
siRNA-t0054458	TTGTCAGTGTCAGGATCCGTCC	22	14	FGSG_05487	K14318|1|2e-25|117|cci:CC1G_01275|nuclear pore complex protein Nup88
siRNA-t0054545	TTGTCAGTGTCAGGATCCGTCCA	23	14	FgHV1gp2	-
siRNA-t0056579	AACAGAGCTGGGGATGTTTG	20	13	FGSG_11307	-
siRNA-t0057680	AAGGTGCGAGCAGGATTGAAG	21	13	FgHV1gp2	-
siRNA-t0056893	AAGTCTGGAATTCCTTGGGAGGCCT	25	13	FgHV1gp2	-
siRNA-t0055303	AATCTTCAGGCCCTGGGATTGTA	23	13	FgHV1gp2	-
siRNA-t0055484	ACACTAATATCGTCATTCTCTT	22	13	FgHV1gp2	-
siRNA-t0057293	AGAAGCTTGACACGAATGATGTA	23	13	FgHV1gp2	-
siRNA-t0055781	AGAATTCTGCTGCGTGTTATG	21	13	FGSG_09750	-
siRNA-t0056647	AGCTGGGGATGTTTGCTTGGTTTCG	25	13	FgHV1gp2	-
siRNA-t0054816	AGTCTTGCGGTGTGGGTCTTCT	22	13	FgHV1gp2	-
siRNA-t0056838	AGTTCACGAAGGGAGAGGCTAT	22	13	FGSG_10561	K01183|1|0.0|635|afm:AFUA_5G03960|chitinase [EC:3.2.1.14]
siRNA-t0056741	ATACAGGACCGCGCGATGGCGCG	23	13	FgHV1gp2	-
siRNA-t0057541	CAATGTAAGTGACGAGGCCT	20	13	FGSG_01126	-
siRNA-t0056553	CACATCACGCTCGGGGTCAA	20	13	FGSG_12112	-
siRNA-t0056900	CAGACCCCAGATCCACCCGGA	21	13	FgHV1gp2	-
siRNA-t0056663	CAGACCCCAGATCCACCCGGAT	22	13	FgHV1gp2	-
siRNA-t0056334	CATACACGGAACCCATTGAT	20	13	FGSG_08294	-
siRNA-t0055558	CCACATCCAGATCTTATAGACA	22	13	FgHV1gp2	-
siRNA-t0056794	CCAGAAACCCATCCCACTCGTG	22	13	FgHV1gp2	-
siRNA-t0056844	CCATATGGATCGGTGGCACACGCA	24	13	FgHV1gp2	-
siRNA-t0056136	CCATGCTTTCGACGTACTTGGGTT	24	13	FgHV1gp2	-
siRNA-t0057838	CCATGGAGACTGCCGATTTGG	21	13	FGSG_03184	-
siRNA-t0055888	CCGAATATGTCAGAGATCCT	20	13	FGSG_12247	K00777|1|4e-08|59.3|afm:AFUA_7G07100|[EC:2.4.2.-]
siRNA-t0056340	CCGGAATGCGTTCACACGATTCCCT	25	13	FgHV1gp2	-
siRNA-t0057900	CGAATTCTGCCATAACCTCGTT	22	13	FgHV1gp2	-
siRNA-t0055386	CGCTCCCACTATCGTCCTGCG	21	13	FGSG_01294	K00799|1|1e-34|146|afv:AFLA_022030|glutathione S-transferase [EC:2.5.1.18]
siRNA-t0056990	CGGCATGAACTCGGTCAACGAAA	23	13	FgHV1gp2	-
siRNA-t0055158	CGGCGTGCTGAGATCAAGTA	20	13	FGSG_10790	K15389|1|3e-101|368|ure:UREG_05875|acyl-CoA dehydrogenase;K00257|2|1e-100|366|afm:AFUA_6G10880|[EC:1.3.99.-]
siRNA-t0055877	CGGGTCCAGATGTTGTGCTTTTT	23	13	FgHV1gp2	-
siRNA-t0054995	CGTGAGTCGGTGGTTGATAT	20	13	FGSG_04617	-
siRNA-t0055902	CTCGGGTCCTGATGGTTTCAGAA	23	13	FGSG_06016	K05727|1|1e-46|188|mpr:MPER_07660|dedicator of cytokinesis 3/4/5
siRNA-t0056799	CTCTTGTTCTGGAACGTCAAATC	23	13	FgHV1gp2	-
siRNA-t0055058	CTGAACCCAGTTGCTGTATCT	21	13	FGSG_06379	K14289|1|0.0|2450|fgr:FG06379.1|exportin-5
siRNA-t0057936	CTGACAAATCTGGGTGGCGATGGAT	25	13	FgHV1gp2	-
siRNA-t0055280	CTGACATATTCGGACTCATCCCAC	24	13	FgHV1gp2	-
siRNA-t0057472	CTGACTGTTGCTTGATGGTTGGCAC	25	13	FgHV1gp2	-
siRNA-t0056418	CTTGAAATGATTTGCGACGTGCTT	24	13	FgHV1gp2	-
siRNA-t0056573	GAACTCCACATAGCGTCGTC	20	13	FgHV1gp2	-
siRNA-t0056643	GAAGGAACTCCACATAGCGTC	21	13	FgHV1gp2	-
siRNA-t0055722	GACGTCGGCACCAACTTCAA	20	13	FGSG_11354	-
siRNA-t0055494	GAGTCTTGCGGTGTGGGTCTTC	22	13	FgHV1gp2	-
siRNA-t0054908	GAGTTTGAACCTGCTAAACT	20	13	FGSG_05163	-
siRNA-t0055577	GATATTCACGCATGTACTGT	20	13	FGSG_04622	K03380|1|8e-44|178|fgr:FG11194.1|phenol 2-monooxygenase [EC:1.14.13.7]
siRNA-t0056766	GCCTCTCCCTTCGTGAACTGCT	22	13	FgHV1gp2	-
siRNA-t0054920	GCGAATTCTGCCATAACCTCGTT	23	13	FgHV1gp2	-
siRNA-t0057373	GGGACGCGACAAGCAACAATTTGC	24	13	FgHV1gp2	-
siRNA-t0057303	GGGAGGCCTATTCATCGTCAA	21	13	FgHV1gp2	-
siRNA-t0057850	GGGATTCCTTTATGCCAGCA	20	13	FGSG_04126	K00365|1|1e-179|627|fgr:FG04126.1|urate oxidase [EC:1.7.3.3]
siRNA-t0055810	GGTGTCGGCTGCCAAGTAAG	20	13	FGSG_12344	K01620|1|2e-117|421|act:ACLA_008260|threonine aldolase [EC:4.1.2.5]
siRNA-t0055168	GTAGTCATCGTCAGGGTACG	20	13	FGSG_09801	K03164|1|0.0|2817|fgr:FG09801.1|DNA topoisomerase II [EC:5.99.1.3]
siRNA-t0057677	GTCCTTCGTTGAATCGATCGT	21	13	FgHV1gp2	-
siRNA-t0055808	GTGAACTGCTGCGCTATGGTGTG	23	13	FgHV1gp2	-
siRNA-t0057425	GTGTGTCGCGTCTACCTTGTTCA	23	13	FgHV1gp2	-
siRNA-t0057591	GTTCACGAAGGGAGAGGCTAT	21	13	FGSG_10561	K01183|1|0.0|635|afm:AFUA_5G03960|chitinase [EC:3.2.1.14]
siRNA-t0056120	GTTCCATAAACGCTGCCGGGA	21	13	FgHV1gp2	-
siRNA-t0056451	TACTATCGTTGTGGTTCTTT	20	13	FGSG_07258	K14572|1|0.0|8396|fgr:FG07258.1|midasin

siRNA-t0057347	TCATTGGCATAGAATCGCGC	20	13	FGSG_11240	K08711|1|0.0|1162|cne:CNB05770|ATP-binding cassette, subfamily G (WHITE), member 2, PDR
siRNA-t0057360	TCCAGATCTTATAGACAGGCCCAA	24	13	FgHV1gp2	-
siRNA-t0055990	TCGCTGATCTGGCTCGTGACTTT	23	13	FgHV1gp2	-
siRNA-t0057430	TCGGTAGTCATCGGGAATCA	20	13	FgHV1gp2	-
siRNA-t0056639	TCTACCAGAACCCAGCACCT	20	13	FGSG_12463	-
siRNA-t0056632	TCTCTTGTTCTGGAACGTCA	20	13	FGSG_13060	-
siRNA-t0057691	TCTCTTTGTTGGCCTGGTAA	20	13	FGSG_13186	-
siRNA-t0055740	TCTGGTACCGATATTGAACCCT	22	13	FgHV1gp2	-
siRNA-t0055767	TGAGCACTTGTTTGCCTGCA	20	13	FgHV1gp2	-
siRNA-t0057910	TGATGATCATGACGTGGAGA	20	13	FGSG_06097	-
siRNA-t0056089	TGCGACGACCGTGCGAAATTGTT	23	13	FgHV1gp2	-
siRNA-t0056576	TGCTCACTGTTGACGTCACCC	21	13	FGSG_07798	K15384|1|0.0|2498|afv:AFLA_066840|aspyridone synthetase, hybrid polyketide synthase / nonribosomal peptide synthetase;K15394|3|0.0|2447|cpw:CPC735_030880|hybrid polyketide synthase / nonribosomal peptide synthetase ACE1
siRNA-t0056329	TGGAAATCTTCAGGCCCTGG	20	13	FGSG_09607	K10413|1|0.0|8782|fgr:FG09607.1|dynein heavy chain 1, cytosolic
siRNA-t0056711	TGTAGAACTTTTGCCACTGGGAT	23	13	FgHV1gp2	-
siRNA-t0057442	TGTTCGCATCCGTTCTGTGAA	21	13	FGSG_08999	-
siRNA-t0055448	TTCACGAAGGGAGAGGCTAT	20	13	FGSG_10561	K01183|1|0.0|635|afm:AFUA_5G03960|chitinase [EC:3.2.1.14]
siRNA-t0060421	AAAGTGTCGTAGGAAAGGAAAAGTT	25	12	FgHV1gp1	-
siRNA-t0058662	AAATACAGGACCGCGCGATGGCG	23	12	FgHV1gp2	-
siRNA-t0058300	AACATGCCGACAGAGAACAGC	21	12	FGSG_10935	K08711|1|1e-79|298|kla:KLLA0D03476g|ATP-binding cassette, subfamily G (WHITE), member 2, PDR
siRNA-t0060566	AACCAAGAGTGCTCATCGTTGT	22	12	FGSG_10431	-
siRNA-t0061280	AAGTGTCGTAGGAAAGGAAAAGTTC	25	12	FgHV1gp1	-
siRNA-t0059010	ACACGCAGCAGAATTCTAGCCTGG	24	12	FgHV1gp2	-
siRNA-t0059020	ACATGATGGCTGATGCGACCGCT	23	12	FGSG_13018	-
siRNA-t0059881	ACCAGAACCCAGCACCTTTCCC	22	12	FgHV1gp2	-
siRNA-t0060126	ACGGTTCTTGGAAAGTTGGAG	21	12	FgHV1gp2	-
siRNA-t0060376	AGAAGCTTGACACGAATGATG	21	12	FgHV1gp2	-
siRNA-t0060878	AGACCGAAGAAAGACCACATCCAGA	25	12	FgHV1gp2	-
siRNA-t0061642	AGATCTCTAGGATGATGCCA	20	12	FGSG_11200	K00688|1|1e-06|55.5|pno:SNOG_13460|starch phosphorylase [EC:2.4.1.1]
siRNA-t0058778	AGCCTCTCCCTTCGTGAACTGCT	23	12	FgHV1gp2	-
siRNA-t0058142	AGGATCTCGTAAGCTGCTAC	20	12	FGSG_05596	K13339|1|0.0|1840|fgr:FG05596.1|peroxin-6
siRNA-t0059732	AGGTGAAAACCCTGACAAATCTGGG	25	12	FgHV1gp2	-
siRNA-t0060224	AGTGGCATATTCAAGAATCA	20	12	FgHV1gp2	-
siRNA-t0061379	AGTGGGTCGAGGATGCAAAC	20	12	FGSG_07064	-
siRNA-t0059330	AGTTCCGCGTGTGATTGGTCTCT	23	12	FGSG_03919	-
siRNA-t0061463	ATGGCATGACTTTACAAGCT	20	12	FgHV1gp2	-
siRNA-t0060223	ATTTTTGGTCTTCGACGGGTA	21	12	FGSG_13308	-
siRNA-t0058166	CAAATCTGGGTGGCGATGGA	20	12	FgHV1gp2	-
siRNA-t0060101	CAAGCGTGACGAAGATCCCA	20	12	FGSG_07746	-
siRNA-t0058723	CAGGCCAACAAAGAGAACTGGCG	23	12	FGSG_06226	K15084|1|0.0|743|fgr:FG06226.1|solute carrier family 25 (mitochondrial carrier protein), member 16
siRNA-t0058384	CCAAACTGTTCATTTCGGCGAG	22	12	FGSG_02771	K05393|1|1e-82|305|uma:UM03490.1|chloride channel, other eukaryote;K06867|2|2e-14|79.7|spo:SPBC2F12.05c|
siRNA-t0060372	CCAATGTAAGTGACGAGGCCTC	22	12	FGSG_01126	-
siRNA-t0058821	CCACACTCAGCAGGAACTGGT	21	12	FGSG_05573	-
siRNA-t0059496	CCGAAGGAACTCCACATAGCGTC	23	12	FgHV1gp2	-
siRNA-t0059947	CCGGGATCAGGTTCACAATTGCACA	25	12	FgHV1gp2	-
siRNA-t0059180	CCTATAGATCGCGCGTAAGCTT	22	12	FgHV1gp2	-
siRNA-t0058582	CGAACCTCAAATCCAAAAGACGCG	24	12	FgHV1gp2	-
siRNA-t0060995	CGAAGACCAAAAATTTCTTC	20	12	FgHV1gp1	-
siRNA-t0059803	CGAAGTATGAGGGAGAGGAA	20	12	FGSG_09380	K09503|1|7e-21|101|lbc:LACBIDRAFT_159402|DnaJ homolog subfamily A member 2;K09510|5|1e-18|94.0|act:ACLA_010590|DnaJ homolog subfamily B member 4
siRNA-t0061324	CGACCCATGCTTTCGACGTA	20	12	FGSG_08372	K10838|1|0.0|1448|fgr:FG08372.1|xeroderma pigmentosum group C-complementing protein
siRNA-t0058343	CGACGGTACGTGGAATCTTCAGGC	25	12	FgHV1gp2	-
siRNA-t0060047	CGTGTTGTACGGCGTGGTCTC	21	12	FgHV1gp2	-
siRNA-t0058680	CTGGTGGTTGGCGGCATTGTA	21	12	FGSG_00969	K00602|1|0.0|1213|fgr:FG00969.1|phosphoribosylaminoimidazolecarboxamide formyltransferase / IMP cyclohydrolase [EC:2.1.2.3 3.5.4.10]
siRNA-t0061248	CTGTTCTCTGTCGGCATGTTGC	22	12	FGSG_11657	K09184|1|5e-13|75.9|bfu:BC1G_09852|GATA-binding protein, other eukaryote
siRNA-t0060053	CTGTTGCTTGATGGTTGGCACAA	23	12	FgHV1gp2	-
siRNA-t0059980	CTTACCTAGACCGAAGAAAGACCA	24	12	FgHV1gp2	-
siRNA-t0060603	CTTCATTTGGGTGGCTTTGAAGTT	24	12	FgHV1gp2	-
siRNA-t0059104	CTTGATCTCAGCACGCCGGTT	21	12	FGSG_10904	K06681|1|1e-61|238|cdu:CD36_21160|Gdp/GTP exchange factor required for growth at low temperatures
siRNA-t0059530	CTTTTGGATTTGAGGTTCGCT	21	12	FGSG_10717	-
siRNA-t0059429	GAAGAAGTGACGATACTTGAA	21	12	FGSG_02870	K08712|1|0.0|1038|pic:PICST_57529|ATP-binding cassette, subfamily G (WHITE), member 2, SNQ2;K08711|5|0.0|964|cne:CNB05770|ATP-binding cassette, subfamily G (WHITE), member 2, PDR
siRNA-t0058405	GAGGGCCCAGTCATGAATTTTCGG	24	12	FgHV1gp2	-
siRNA-t0058742	GATCTTGCTGTAGTGTCTGAA	21	12	FgHV1gp2	-
siRNA-t0058386	GATGATCATGACGTGGAGAAG	21	12	FGSG_01407	-
siRNA-t0058368	GCGACGTGCTTGAAGGCGAAGTA	23	12	FgHV1gp2	-
siRNA-t0058712	GCGCATACACGGAACCCATTG	21	12	FgHV1gp2	-
siRNA-t0059802	GCGGTCCATGGTGGGGTCACA	21	12	FGSG_11800	-
siRNA-t0059290	GGCGAGACCGATGATTTCCATCCCC	25	12	FgHV1gp2	-
siRNA-t0060416	GGCTTGTCGTCGGGAGAATCCAGA	24	12	FgHV1gp2	-
siRNA-t0058669	GGGTCTTCTGACGAATTTGGACAAG	25	12	FgHV1gp2	-
siRNA-t0060239	GTGCGACAGTTTCTACACCA	20	12	FGSG_06395	K01529|1|0.0|4061|fgr:FG06395.1|[EC:3.6.1.-]
siRNA-t0058751	GTGGGTCTTCTGACGAATTTGGA	23	12	FgHV1gp2	-
siRNA-t0058841	GTGGTCTTTCTTCGGTCTAG	20	12	FGSG_14002	-
siRNA-t0060463	GTGTCGAACCATACTGGACCC	21	12	FGSG_08097	K00688|1|7e-16|87.0|pno:SNOG_13460|starch phosphorylase [EC:2.4.1.1]
siRNA-t0058920	GTTACAATCGTGGACTTCCTT	21	12	FgHV1gp2	-
siRNA-t0058417	TACGTGGAAATCTTCAGGCC	20	12	FgHV1gp2	-
siRNA-t0061209	TCCCATAGAATGTCGAGATACT	22	12	FGSG_13878	K01932|1|0.0|1450|afm:AFUA_5G12730|[EC:6.3.2.-]
siRNA-t0059212	TCCCTTCGTGAACTGCTGCGCTAT	24	12	FgHV1gp2	-
siRNA-t0061456	TCGCCTTCAAGCACGTCGCAA	21	12	FGSG_10936	-
siRNA-t0060060	TCTCGGGTCCTGATGGTTTCAGAA	24	12	FGSG_06016	K05727|1|1e-46|188|mpr:MPER_07660|dedicator of cytokinesis 3/4/5
siRNA-t0058727	TCTGACTGTTGCTTGATGGTTGGC	24	12	FgHV1gp2	-
siRNA-t0059996	TCTGGTACCGATATTGAACCCTC	23	12	FgHV1gp2	-
siRNA-t0061038	TCTTCTGACGAATTTGGACAAGT	23	12	FgHV1gp2	-
siRNA-t0059251	TGAAAGTGTCGTAGGAAAGGAAAA	24	12	FgHV1gp1	-
siRNA-t0060850	TGCTCACTGTTGACGTCACC	20	12	FGSG_07798	K15384|1|0.0|2498|afv:AFLA_066840|aspyridone synthetase, hybrid polyketide synthase / nonribosomal peptide synthetase;K15394|3|0.0|2447|cpw:CPC735_030880|hybrid polyketide synthase / nonribosomal peptide synthetase ACE1
siRNA-t0058120	TGGTTCAAGTATCGTCACTTCTTCG	25	12	FgHV1gp2	-
siRNA-t0059672	TGTCGGCTGCCAAGTAAGCA	20	12	FGSG_12344	K01620|1|2e-117|421|act:ACLA_008260|threonine aldolase [EC:4.1.2.5]
siRNA-t0061172	TGTGATTGGTCTCTTGCAGTGCA	23	12	FgHV1gp2	-
siRNA-t0059513	TTCGGACTCATCCCACATGATGGTG	25	12	FgHV1gp2	-
siRNA-t0060797	TTGTGAACCTGATCCCGGAATT	22	12	FGSG_10966	K11702|1|1e-47|190|cne:CNL04750|beta-1,2-xylosyltransferase [EC:2.4.2.-]

siRNA-t0063707	AAAGAACGTCGTTATGCCAG	20	11	FGSG_01539	K15218|1|0.0|1956|fgr:FG01539.1|RNA polymerase I-specific transcription initiation factor RRN6
siRNA-t0064909	AACTGCGACCCATGCTTTCGACG	23	11	FgHV1gp2	-
siRNA-t0065505	AAGGCTTGTCGTCGGGAGAAT	21	11	FGSG_08614	K01874|1|0.0|1256|fgr:FG08614.1|methionyl-tRNA synthetase [EC:6.1.1.10]
siRNA-t0061800	AAGTCGAAAGTGAAAGTGTCGTA	23	11	FgHV1gp1	-
siRNA-t0063286	AAGTCTCTTGTTCTGGAACGTCA	23	11	FgHV1gp2	-
siRNA-t0065520	AAGTTTTGTGGTACAATGGTATTGG	25	11	FGSG_13758	K06867|1|3e-09|62.0|ago:AGOS_ADL244W|
siRNA-t0064047	ACCTATACTTGATCTCAGCACGCCG	25	11	FgHV1gp2	-
siRNA-t0065756	ACGACACGCGTCTTTTGGATTT	22	11	FGSG_05472	K06910|1|4e-117|418|fgr:FG05472.1|
siRNA-t0062942	ACGATAGTGGGAGCGAAAGCG	21	11	FGSG_08335	K02873|1|8e-89|325|fgr:FG08335.1|large subunit ribosomal protein L13e
siRNA-t0063793	ACGCTGAAATCGAGGGTGTTG	21	11	FGSG_04870	-
siRNA-t0062386	AGAAGATTGAGTTCATTTGTTCC	23	11	FgHV1gp2	-
siRNA-t0063756	AGAATGTCGAGATACTCTTCAG	22	11	FGSG_13169	K00750|1|4e-16|84.7|cdu:CD36_86420|glycogenin glucosyltransferase [EC:2.4.1.186]
siRNA-t0064436	AGAGCTGGGGATGTTTGCTTGGTTT	25	11	FgHV1gp2	-
siRNA-t0064682	AGATCAACGTCAGAGGCCTCG	21	11	FGSG_06054	K01784|1|3e-65|249|fgr:FG07983.1|UDP-glucose 4-epimerase [EC:5.1.3.2]
siRNA-t0063432	AGCCATTCGTTACAATCGTGGAC	23	11	FgHV1gp2	-
siRNA-t0063223	AGGCCTCGTCACTTACATTG	20	11	FGSG_12610	-
siRNA-t0065400	AGGCGCATCGGCTTACCCTC	20	11	FGSG_13700	-
siRNA-t0063630	AGGGCCCAGTCATGAATTTTC	21	11	FgHV1gp2	-
siRNA-t0064230	AGTGGGATCTTCGTCACGCT	20	11	FGSG_05729	-
siRNA-t0064493	AGTTCCGCGTGTGATTGGTCTCTT	24	11	FGSG_03919	-
siRNA-t0065320	ATACAGGACCGCGCGATGGCG	21	11	FgHV1gp2	-
siRNA-t0063762	ATACTTGATCTCAGCACGCCG	21	11	FgHV1gp2	-
siRNA-t0064564	ATGTACTGTTCGGCAACAGAAT	22	11	FgHV1gp2	-
siRNA-t0062641	ATGTAGGCTCCTCCGACAATGGCGC	25	11	FgHV1gp2	-
siRNA-t0063243	CAAATACAGGACCGCGCGAT	20	11	FgHV1gp2	-
siRNA-t0065526	CAATGCAACAGACTTCTTCTC	21	11	FgHV1gp2	-
siRNA-t0064313	CACATTTTTGAAGATCGTGAA	21	11	FGSG_02394	K01932|1|0.0|942|afm:AFUA_1G10380|[EC:6.3.2.-]
siRNA-t0062385	CAGTTCCGCGTGTGATTGGTCTCT	24	11	FGSG_03919	-
siRNA-t0061775	CATTCGTTACAATCGTGGAC	20	11	FgHV1gp2	-
siRNA-t0062726	CATTTTATTGTAAGTCTGGA	20	11	FgHV1gp2	-
siRNA-t0063662	CCAAAGAGAGGTGCGTAGCAT	21	11	FgHV1gp2	-
siRNA-t0065280	CCACCCGGATGACTTGGCTC	20	11	FGSG_06041	K00143|1|0.0|2052|fgr:FG06041.1|L-aminoadipate-semialdehyde dehydrogenase [EC:1.2.1.31]
siRNA-t0063496	CCAGCAGACACTTGTTTCTCGT	22	11	FgHV1gp2	-
siRNA-t0063703	CCAGGGGCGTTTTTCTCGGG	20	11	FGSG_07182	K02358|1|0.0|889|fgr:FG07182.1|elongation factor Tu
siRNA-t0065664	CCGAACAGTACATGCAGCAA	20	11	FGSG_06900	K03639|1|0.0|1291|fgr:FG06900.1|molybdenum cofactor biosynthesis protein
siRNA-t0065291	CGGCGTGCTGAGATCAAGTAT	21	11	FGSG_10790	K15389|1|3e-101|368|ure:UREG_05875|acyl-CoA dehydrogenase;K00257|2|1e-100|366|afm:AFUA_6G10880|[EC:1.3.99.-]
siRNA-t0063226	CGTACCCGTCGAAGACCAAAAAT	23	11	FgHV1gp1	-
siRNA-t0062655	CGTCATACATTGCACGAGGGACG	23	11	FgHV1gp2	-
siRNA-t0061957	CGTTACAATCGTGGACTTCCTT	22	11	FgHV1gp2	-
siRNA-t0064847	CTACCAGAACCCAGCACCTTTCCCT	25	11	FgHV1gp2	-
siRNA-t0064197	CTGAAACCATCAGGACCCGAGAAA	24	11	FgHV1gp2	-
siRNA-t0064571	CTGCCATAACCTCGTTGGGA	20	11	FGSG_06651	-
siRNA-t0062485	CTGGATCAATGTCAGCAGTGTTCA	24	11	FgHV1gp2	-
siRNA-t0062512	CTGGCCGGTTGAAAATGTTC	20	11	FGSG_05462	K01072|1|2e-159|564|afm:AFUA_2G08440|ubiquitin thiolesterase [EC:3.1.2.15];K11838|3|3e-41|171|clu:CLUG_02963|ubiquitin carboxyl-terminal hydrolase 7 [EC:3.1.2.15]
siRNA-t0065608	GAACCCATATGGATCGGTGGCACAC	25	11	FgHV1gp2	-
siRNA-t0062063	GAACCCTTCCACGATTGGCGT	21	11	FgHV1gp2	-
siRNA-t0064486	GAATGTCGAGATACTCTTCAGCGTA	25	11	FgHV1gp2	-
siRNA-t0065420	GACCGATGGAAGTGATGGCAA	21	11	FGSG_08209	K01932|1|0.0|1305|afm:AFUA_5G12730|[EC:6.3.2.-]
siRNA-t0063020	GACGAGGCCTCTGACGTTGATCTCT	25	11	FgHV1gp2	-
siRNA-t0063749	GATCGATTCAACGAAGGACA	20	11	FGSG_05691	K11121|1|9e-70|265|ani:AN8450.2|NAD-dependent histone deacetylase SIR2 [EC:3.5.1.-]
siRNA-t0064143	GCAACAGACTTCTTCTCTGCT	21	11	FgHV1gp2	-
siRNA-t0065452	GCAGACACTTGTTTCTCGTGGGA	23	11	FgHV1gp2	-
siRNA-t0064543	GCAGCAGAATTCTAGCCTGG	20	11	FGSG_02733	-
siRNA-t0065649	GCAGGATCGTTATTCTTCTTC	21	11	FGSG_00698	-
siRNA-t0064399	GCCCAGTCATGAATTTTCGGT	21	11	FgHV1gp2	-
siRNA-t0064060	GCGCACATATCTCCTTGAACTT	22	11	FgHV1gp2	-
siRNA-t0063925	GCTGATCTGGCTCGTGACTT	20	11	FgHV1gp2	-
siRNA-t0061959	GCTGCTTGTCAGTGTCAGGATC	22	11	FgHV1gp2	-
siRNA-t0062840	GCTGCTTGTCAGTGTCAGGATCCGT	25	11	FgHV1gp2	-
siRNA-t0063544	GCTTGATGGTTGGCACAACGATGAG	25	11	FgHV1gp2	-
siRNA-t0064056	GGAGTGGCATATTCAAGAATCAATG	25	11	FgHV1gp2	-
siRNA-t0063766	GGATCTTCGTCACGCTTGCA	20	11	FGSG_06837	-
siRNA-t0062601	GGCCTCTGACGTTGATCTCT	20	11	FgHV1gp2	-
siRNA-t0063008	GGGACCATCATGATGAAACCGAAAT	25	11	FgHV1gp2	-
siRNA-t0064448	GGGACCGACGTTTTGGCGAGACC	23	11	FgHV1gp2	-
siRNA-t0064672	GGGTCGAGGATGCAAACTTCTT	22	11	FGSG_07064	-
siRNA-t0063435	GGGTTGGCCAAATCTCGGACCGCT	24	11	FgHV1gp2	-
siRNA-t0064492	GGTACGTGGAAATCTTCAGGCC	22	11	FgHV1gp2	-
siRNA-t0064625	GGTCTGGAAAGAACGTCGTTA	21	11	FGSG_04647	-
siRNA-t0063908	GTAAAGTTATCGTGTCTGAC	20	11	FgHV1gp2	-
siRNA-t0064561	GTATTGATGAAAGGAGTGCA	20	11	FGSG_10456	-
siRNA-t0065169	GTCGACAGGCGTGGCTGAGAGAAG	24	11	FgHV1gp2	-
siRNA-t0062741	GTCTTCTGACGAATTTGGAC	20	11	FGSG_10174	K11367|1|2e-107|390|scm:SCHCODRAFT_58011|chromodomain-helicase-DNA-binding protein 1 [EC:3.6.4.12]
siRNA-t0061668	GTGGCCGTAAGTAGCAGATT	20	11	FgHV1gp2	-
siRNA-t0065170	GTGGGTGCGCCGAATGAAAGCCCA	24	11	FgHV1gp2	-
siRNA-t0062217	GTGTCGTAGGAAAGGAAAAGTTC	23	11	FgHV1gp1	-
siRNA-t0063349	GTGTGTCGCGTCTACCTTGT	20	11	FGSG_00721	K10084|1|2e-106|387|spo:SPAC23A1.04c|ER degradation enhancer, mannosidase alpha-like 1
siRNA-t0064675	TACAATCGTGGACTTCCTTTAGGAA	25	11	FgHV1gp2	-
siRNA-t0062917	TACAGCAACTGGGTTCAGCA	20	11	FGSG_01144	-
siRNA-t0063875	TACCAGAACCCAGCACCTTTC	21	11	FgHV1gp2	-
siRNA-t0064928	TAGGCTCCTCCGACAATGGCGCAT	24	11	FgHV1gp2	-
siRNA-t0063573	TATACTTGATCTCAGCACGCCGG	23	11	FgHV1gp2	-
siRNA-t0063603	TCCCTTCGTGAACTGCTGCGCTA	23	11	FgHV1gp2	-
siRNA-t0063765	TCTCTCTGACCTTGATGGCG	20	11	FGSG_11656	K00668|1|0.0|1439|nfi:NFIA_006490|fatty acid synthase subunit beta, fungi type [EC:2.3.1.86]
siRNA-t0062835	TCTGACATATTCGGACTCATCCCA	24	11	FgHV1gp2	-
siRNA-t0064825	TCTTGGAAAGTTGGAGCCCTTC	22	11	FgHV1gp2	-
siRNA-t0062860	TCTTTAACAGAGCTGGGGATGT	22	11	FGSG_05241	-
siRNA-t0062514	TGCAGCAACTGTCGCCTGGACA	22	11	FgHV1gp1	-
siRNA-t0062357	TGCCATGACGTACGACCCCG	20	11	FgHV1gp1	-
siRNA-t0064127	TGCCATGACGTACGACCCCGAAA	23	11	FgHV1gp1	-
siRNA-t0065293	TGTACTTGCCGCGCGTCTCAC	21	11	FgHV1gp2	-
siRNA-t0065120	TGTAGATCTTGCTGTAGTGTCTGAA	25	11	FgHV1gp2	-
siRNA-t0064644	TGTCGAACCATACTGGACCCAGAT	24	11	FgHV1gp2	-
siRNA-t0063118	TTAATCAATGTACCGTTCGGCA	22	11	FgHV1gp2	-
siRNA-t0061920	TTCGCTCCCACTATCGTCCT	20	11	FGSG_10648	-

siRNA-t0063742	TTGTGAACCTGATCCCGGAAT	21	11	FGSG_10966	K11702|1|1e-47|190|cne:CNL04750|beta-1,2-xylosyltransferase [EC:2.4.2.-]
siRNA-t0068225	AAAACGCCCCTGGACGGTACTTC	23	10	FgHV1gp2	-
siRNA-t0065933	AAAGAGAAGAATGAGGGAAGA	21	10	FGSG_02232	-
siRNA-t0070796	AAATCTGGGTGGCGATGGATTG	22	10	FGSG_00899	K11430|1|3e-39|163|bfu:BC1G_02412|enhancer of zeste [EC:2.1.1.43]
siRNA-t0066191	AAATTTTTGGTCTTCGACGGGTAC	24	10	FGSG_13308	-
siRNA-t0068492	AACAGAGCTGGGGATGTTTGCTT	23	10	FgHV1gp2	-
siRNA-t0068314	AACGCCCCTGGACGGTACTTC	21	10	FGSG_13878	K01932|1|0.0|1450|afm:AFUA_5G12730|[EC:6.3.2.-]
siRNA-t0068558	AACTGCGACCCATGCTTTCGACGT	24	10	FgHV1gp2	-
siRNA-t0067954	ACAAAGGTGGAGTACTTGTC	20	10	FGSG_05392	-
siRNA-t0070179	ACCAAGAGTGCTCATCGTTGT	21	10	FGSG_10431	-
siRNA-t0070157	ACCAGAAACCCATCCCACTCGT	22	10	FgHV1gp2	-
siRNA-t0067387	ACCATTGCTGCATGTACTGTT	21	10	FGSG_05795	-
siRNA-t0069602	ACGGGTCCAGATGTTGTGCTTTTT	24	10	FgHV1gp2	-
siRNA-t0067698	ACGTTGATCTCTTATGCGCGG	21	10	FGSG_08308	K05658|1|1e-27|125|pte:PTT_08742|ATP-binding cassette, subfamily B (MDR/TAP), member 1;K05663|2|3e-24|114|cci:CC1G_07837|mitochondrial ABC transporter ATM
siRNA-t0067764	ACTAATATCGTCATTCTCTT	20	10	FGSG_00284	-
siRNA-t0068067	ACTTGCCGCGCGTCTCACTGA	21	10	FgHV1gp2	-
siRNA-t0066834	AGACCGAAGAAAGACCACATCCAG	24	10	FgHV1gp2	-
siRNA-t0067435	AGAGACAGAGCTCGCGCCTTCC	22	10	FgHV1gp2	-
siRNA-t0068869	AGAGTCTTGCGGTGTGGGTC	20	10	FgHV1gp2	-
siRNA-t0065927	AGGACCAGTTCCTGCTGAGTGTG	23	10	FgHV1gp2	-
siRNA-t0067119	AGGGACGTCTTCGATGGATGC	21	10	FGSG_00834	K10743|1|0.0|670|fgr:FG00834.1|ribonuclease H2 subunit A [EC:3.1.26.4]
siRNA-t0070839	AGGTACGTCTATATTGTCTCTCA	23	10	FgHV1gp2	-
siRNA-t0069637	AGTACATGCGTGAATATCTC	20	10	FGSG_02939	-
siRNA-t0066107	AGTGCCATGACGTACGACCCCGA	23	10	FgHV1gp1	-
siRNA-t0069591	AGTGCGTAGATAAAGGTTAT	20	10	FgHV1gp2	-
siRNA-t0067939	AGTTCCGCGTGTGATTGGTCTCTTG	25	10	FgHV1gp2	-
siRNA-t0066833	ATACAGGACCGCGCGATGGCGC	22	10	FgHV1gp2	-
siRNA-t0067645	ATACCTTTGAGCTCCTTGAGGAA	23	10	FgHV1gp2	-
siRNA-t0066695	ATACGTTCTGAGAAGTCTCC	20	10	FgHV1gp2	-
siRNA-t0068541	ATGATTTGCGACGTGCTTGAAGG	23	10	FgHV1gp2	-
siRNA-t0070724	ATGCAAGCGTGACGAAGATC	20	10	FGSG_11395	K01932|1|0.0|1024|afm:AFUA_1G10380|[EC:6.3.2.-]
siRNA-t0066133	ATTGCTGCATGTACTGTTCGGCAA	24	10	FgHV1gp2	-
siRNA-t0067071	CAAATCTGGGTGGCGATGGATT	22	10	FgHV1gp2	-
siRNA-t0067569	CAAGTCTCTTGTTCTGGAACGT	22	10	FGSG_02381	-
siRNA-t0069711	CAAGTTAGACAAATCTTGGA	20	10	FGSG_10835	-
siRNA-t0067480	CAATGGCGACACCGACGGTACGTGG	25	10	FgHV1gp2	-
siRNA-t0066637	CAGCAACTAGAGCCAAGTCAT	21	10	FGSG_06101	K14780|1|0.0|2204|fgr:FG06101.1|ATP-dependent RNA helicase DHX37/DHR1 [EC:3.6.4.13]
siRNA-t0070308	CAGGATTGAAGTGTAATGCGT	21	10	FgHV1gp2	-
siRNA-t0069083	CAGTGGCTTGTGGTTGCGAA	20	10	FGSG_05477	K01104|1|1e-12|74.7|bfu:BC1G_07812|protein-tyrosine phosphatase [EC:3.1.3.48]
siRNA-t0069915	CATACCTTTGAGCTCCTTGAGGAA	24	10	FgHV1gp2	-
siRNA-t0068351	CATTCGTTACAATCGTGGACTT	22	10	FgHV1gp2	-
siRNA-t0066222	CCACGTACCGTCGGTGTCGCC	21	10	FGSG_08320	K00495|1|4e-93|341|afm:AFUA_1G01690|[EC:1.14.14.-]
siRNA-t0069475	CCAGATCTTATAGACAGGCC	20	10	FgHV1gp2	-
siRNA-t0069296	CCGAAGGAACTCCACATAGCGT	22	10	FgHV1gp2	-
siRNA-t0066006	CCGCAGTGATGGAGACTAAAC	21	10	FGSG_09869	K12669|1|0.0|647|fgr:FG09869.1|oligosaccharyltransferase complex subunit gamma
siRNA-t0068606	CCGCTCCGATGTCCACACCGCGTT	24	10	FgHV1gp1	-
siRNA-t0066774	CCGGAATGCGTTCACACGAT	20	10	FgHV1gp2	-
siRNA-t0069802	CCGTACGGATCCACATGAGCT	21	10	FgHV1gp2	-
siRNA-t0067376	CCGTTGATGGCTTCCGTCCCT	21	10	FGSG_05634	-
siRNA-t0067044	CCTATACTTGATCTCAGCACGCCGG	25	10	FgHV1gp2	-
siRNA-t0066174	CGACACCGACGGTACGTGGA	20	10	FgHV1gp2	-
siRNA-t0068786	CGCCAGAATCGAGCTAAACGAACT	24	10	FgHV1gp2	-
siRNA-t0068797	CGGCACCAACTTCAAGAACC	20	10	FGSG_04694	K15419|1|0.0|1075|ani:AN2547.2|emericellamide biosynthesis, highly reducing iterative type I polyketide synthase;K00680|3|0.0|812|afm:AFUA_3G14700|[EC:2.3.1.-]
siRNA-t0065879	CGGTAACATGCAATTGAGTGCCAT	24	10	FgHV1gp1	-
siRNA-t0066582	CGTCATACATTGCACGAGGGACGA	24	10	FgHV1gp2	-
siRNA-t0065976	CTGAATGCTCTGCCTCGGTC	20	10	FGSG_09306	K11644|1|0.0|2881|fgr:FG09306.1|paired amphipathic helix protein Sin3a
siRNA-t0068980	CTGGGGATGTTTGCTTGGTTT	21	10	FgHV1gp2	-
siRNA-t0066786	CTGTCGACGTGCCATCATTG	20	10	FGSG_09894	K15541|1|2e-29|128|aor:AOR_1_1332164|protein MPE1
siRNA-t0067160	CTTGAAATGATTTGCGACGTGCT	23	10	FgHV1gp2	-
siRNA-t0069752	CTTGATCTCAGCACGCCGGTTACCA	25	10	FgHV1gp2	-
siRNA-t0070326	CTTGGTTTCGGGACGCGACAAGCAA	25	10	FgHV1gp2	-
siRNA-t0067233	CTTGTCAGTGTCAGGATCCGTC	22	10	FgHV1gp2	-
siRNA-t0068874	GAAGATCCCACTCTAGCAATGCAT	24	10	FgHV1gp1	-
siRNA-t0067469	GAAGCTTGACACGAATGATGTACCT	25	10	FgHV1gp2	-
siRNA-t0069673	GACAATGGCGCATACACGGAACCCA	25	10	FgHV1gp2	-
siRNA-t0069182	GACGATAGTGGGAGCGAAAG	20	10	FGSG_08335	K02873|1|8e-89|325|fgr:FG08335.1|large subunit ribosomal protein L13e
siRNA-t0068061	GAGGCGCTGGGACTGTACAA	20	10	FGSG_06992	K12494|1|2e-51|204|cnb:CNBD1140|PH and SEC7 domain-containing protein
siRNA-t0070017	GATAAATGAACGTCTGACTGTAGT	24	10	FgHV1gp2	-
siRNA-t0070333	GATAATCGGCATGAACTCGGTCAAC	25	10	FgHV1gp2	-
siRNA-t0066842	GATAGTGGGAGCGAAAGCGA	20	10	FGSG_13563	-
siRNA-t0065857	GATCATCAGCACCGTACGGAT	21	10	FgHV1gp2	-
siRNA-t0067648	GATCATCAGCACCGTACGGATCCAC	25	10	FgHV1gp2	-
siRNA-t0067891	GATCCCACTCTAGCAATGCATCACC	25	10	FgHV1gp1	-
siRNA-t0066878	GCAACATGCCGACAGAGAACA	21	10	FGSG_10935	K08711|1|1e-79|298|kla:KLLA0D03476g|ATP-binding cassette, subfamily G (WHITE), member 2, PDR
siRNA-t0067537	GCAGAATTCTAGCCTGGTTGAGAT	24	10	FgHV1gp2	-
siRNA-t0067190	GCATGGCCGGCAGTCCTAGCAA	22	10	FgHV1gp2	-
siRNA-t0069952	GCATGGTCATTGACGATGAAT	21	10	FGSG_13231	K05841|1|0.0|2541|fgr:FG07850.1|sterol 3beta-glucosyltransferase [EC:2.4.1.173]
siRNA-t0066304	GCCAGGTAGATGACCGACAACA	22	10	FgHV1gp2	-
siRNA-t0067400	GCCATAACCTCGTTGGGACCTTTC	24	10	FgHV1gp2	-
siRNA-t0070230	GCCTGGGAGACAGAGCCTGAGTCAT	25	10	FgHV1gp2	-
siRNA-t0068868	GCGAACGGGACCGACGTTTTGGC	23	10	FgHV1gp2	-
siRNA-t0070134	GCGACCCATGCTTTCGACGTA	21	10	FGSG_08372	K10838|1|0.0|1448|fgr:FG08372.1|xeroderma pigmentosum group C-complementing protein
siRNA-t0066964	GCGACGTGCTTGAAGGCGAA	20	10	FGSG_10824	-
siRNA-t0068463	GCTGAACACCTTTGGCCTCA	20	10	FGSG_10353	K08052|1|1e-22|108|ssl:SS1G_13541|neurofibromin 1
siRNA-t0068425	GCTTGGTTTCGGGACGCGACAAGC	24	10	FgHV1gp2	-
siRNA-t0069944	GCTTGTCGTCGGGAGAATCCA	21	10	FgHV1gp2	-
siRNA-t0069662	GGAACGCACGACAATTGTCTC	21	10	FGSG_08389	-
siRNA-t0068784	GGAATAGCGGTGTCTTATAGGAC	24	10	FgHV1gp2	-
siRNA-t0069358	GGAGCAAGAGAATAGGACAAG	21	10	FGSG_07466	K14951|1|0.0|2603|fgr:FG07466.1|cation-transporting ATPase 13A3/4/5 [EC:3.6.3.-]
siRNA-t0070558	GGAGCCGGATATGTTTTCAA	20	10	FGSG_06265	K14005|1|0.0|1761|fgr:FG06265.1|protein transport protein SEC31
siRNA-t0065872	GGAGGCCTATTCATCGTCAA	20	10	FgHV1gp2	-
siRNA-t0070106	GGATGTTTGCTTGGTTTCGGGAC	23	10	FgHV1gp2	-
siRNA-t0069851	GGCGCATACACGGAACCCATT	21	10	FgHV1gp2	-
siRNA-t0068014	GGGCATTGCCTTGACCGCGTT	21	10	FGSG_11988	K05658|1|0.0|2439|fgr:FG01684.1|ATP-binding cassette, subfamily B (MDR/TAP), member 1

<table>
<tr><td>siRNA-t0070586</td><td>GGGCCCAGTCATGAATTTTCGGTAG</td><td>25</td><td>10</td><td>FgHV1gp2</td><td>-</td></tr>
<tr><td>siRNA-t0066916</td><td>GGGTTGGCCAAATCTCGGAC</td><td>20</td><td>10</td><td>FGSG_11146</td><td>K11987|1|1e-83|311|aor:AOR_1_1372154|prostaglandin-endoperoxide synthase 2 [EC:1.14.99.1]</td></tr>
<tr><td>siRNA-t0066938</td><td>GGTACGTGGAAATCTTCAGGC</td><td>21</td><td>10</td><td>FGSG_11796</td><td>-</td></tr>
<tr><td>siRNA-t0065932</td><td>GGTGGGGTCACATATCTCGAT</td><td>21</td><td>10</td><td>FgHV1gp2</td><td>-</td></tr>
<tr><td>siRNA-t0069196</td><td>GTACCGATATTGAACCCTCCT</td><td>21</td><td>10</td><td>FGSG_01583</td><td>K00489|1|7e-11|67.8|bfu:BC1G_07665|cytochrome P450, family 7, subfamily A (cholesterol 7alpha-monooxygenase) [EC:1.14.13.17];K00517|3|2e-06|53.5|afm:AFUA_2G14430|[EC:1.14.-.-];K14338|4|8e-06|51.2|mgr:MGG_10879|cytochrome P450 / NADPH-cytochrome P450 reductase [EC:1.14.14.1 1.6.2.4]</td></tr>
<tr><td>siRNA-t0070298</td><td>GTACCGTCGGTGTCGCCATT</td><td>20</td><td>10</td><td>FGSG_04902</td><td>K01469|1|0.0|2377|nhe:NECHADRAFT_72144|5-oxoprolinase (ATP-hydrolysing) [EC:3.5.2.9]</td></tr>
<tr><td>siRNA-t0068343</td><td>GTAGAACAGACGTCTCTCAC</td><td>20</td><td>10</td><td>FgHV1gp2</td><td>-</td></tr>
<tr><td>siRNA-t0067792</td><td>GTCTCTTGTTCTGGAACGTCAAAT</td><td>24</td><td>10</td><td>FgHV1gp2</td><td>-</td></tr>
<tr><td>siRNA-t0066474</td><td>GTGATCCTAAACATGTCGGTG</td><td>21</td><td>10</td><td>FGSG_11659</td><td>K01932|1|0.0|1431|afm:AFUA_5G12730|[EC:6.3.2.-]</td></tr>
<tr><td>siRNA-t0068347</td><td>GTGATTGTGGTCATTTGGTTTGTG</td><td>24</td><td>10</td><td>FgHV1gp2</td><td>-</td></tr>
<tr><td>siRNA-t0066868</td><td>TAGAGATCTCCGCAGAAGAA</td><td>20</td><td>10</td><td>FGSG_06836</td><td>K01259|1|7e-121|433|afm:AFUA_2G05000|proline iminopeptidase [EC:3.4.11.5]</td></tr>
<tr><td>siRNA-t0069333</td><td>TAGAGATCTCCGCAGAAGAATT</td><td>22</td><td>10</td><td>FGSG_06836</td><td>K01259|1|7e-121|433|afm:AFUA_2G05000|proline iminopeptidase [EC:3.4.11.5]</td></tr>
<tr><td>siRNA-t0067754</td><td>TAGGACAAGCCGTCAAACATAGACA</td><td>25</td><td>10</td><td>FgHV1gp2</td><td>-</td></tr>
<tr><td>siRNA-t0068060</td><td>TATTGAACCACAGTCGAATGA</td><td>21</td><td>10</td><td>FgHV1gp2</td><td>-</td></tr>
<tr><td>siRNA-t0070462</td><td>TCCTAGAGATCTCCGCAGAAGAAT</td><td>24</td><td>10</td><td>FGSG_00352</td><td>K08064|1|2e-78|291|mgr:MGG_00354|nuclear transcription factor Y, alpha</td></tr>
<tr><td>siRNA-t0066353</td><td>TCCTTCGGTAATCACAGGGCA</td><td>21</td><td>10</td><td>FgHV1gp2</td><td>-</td></tr>
<tr><td>siRNA-t0068732</td><td>TCGAACCATACTGGACCCAG</td><td>20</td><td>10</td><td>FgHV1gp2</td><td>-</td></tr>
<tr><td>siRNA-t0066340</td><td>TCTACCAGAACCCAGCACCTTTCC</td><td>24</td><td>10</td><td>FgHV1gp2</td><td>-</td></tr>
<tr><td>siRNA-t0067586</td><td>TCTGGGTCCAGTATGGTTCGA</td><td>21</td><td>10</td><td>FGSG_13905</td><td>-</td></tr>
<tr><td>siRNA-t0069148</td><td>TCTTCGTCACGCTTGCATTGCT</td><td>22</td><td>10</td><td>FGSG_00633</td><td>K10592|1|0.0|5185|fgr:FG00633.1|E3 ubiquitin-protein ligase HUWE1 [EC:6.3.2.19]</td></tr>
<tr><td>siRNA-t0068246</td><td>TGAACCAGATCATAACCCGCA</td><td>21</td><td>10</td><td>FgHV1gp2</td><td>-</td></tr>
<tr><td>siRNA-t0068702</td><td>TGCTGCCGCGGATAACCTATACT</td><td>23</td><td>10</td><td>FgHV1gp2</td><td>-</td></tr>
<tr><td>siRNA-t0068203</td><td>TGGACCGCAATTACCATACA</td><td>20</td><td>10</td><td>FGSG_12292</td><td>-</td></tr>
<tr><td>siRNA-t0067473</td><td>TGTACCGTTCGGCAACAGAT</td><td>20</td><td>10</td><td>FGSG_07974</td><td>K05989|1|0.0|1786|fgr:FG07974.1|alpha-L-rhamnosidase [EC:3.2.1.40]</td></tr>
<tr><td>siRNA-t0069248</td><td>TGTGGGTCTTCTGACGAATTTGGAC</td><td>25</td><td>10</td><td>FgHV1gp2</td><td>-</td></tr>
<tr><td>siRNA-t0068666</td><td>TTCTCTCTGACCTTGATGGC</td><td>20</td><td>10</td><td>FGSG_09454</td><td>K14293|1|0.0|1735|fgr:FG09454.1|importin subunit beta-1</td></tr>
<tr><td>siRNA-t0070159</td><td>TTGGTAATTGACAAAGGCCTA</td><td>21</td><td>10</td><td>FgHV1gp2</td><td>-</td></tr>
<tr><td>siRNA-t0072563</td><td>AAACGCGCCTCCAGTCCAGCCTT</td><td>23</td><td>9</td><td>FgHV1gp2</td><td>-</td></tr>
<tr><td>siRNA-t0075774</td><td>AAAGTGTCGTAGGAAAGGAAA</td><td>21</td><td>9</td><td>FgHV1gp1</td><td>-</td></tr>
<tr><td>siRNA-t0074494</td><td>AACGACCCCTGAAAGCCTCAT</td><td>21</td><td>9</td><td>FgHV1gp1</td><td>-</td></tr>
<tr><td>siRNA-t0075636</td><td>AAGTCTGGAATTCCTTGGGAG</td><td>21</td><td>9</td><td>FgHV1gp2</td><td>-</td></tr>
<tr><td>siRNA-t0074336</td><td>AAGTTCTCTCTGACCTTGATG</td><td>21</td><td>9</td><td>FGSG_11656</td><td>K00668|1|0.0|1439|nfi:NFIA_006490|fatty acid synthase subunit beta, fungi type [EC:2.3.1.86]</td></tr>
<tr><td>siRNA-t0071738</td><td>AATTGTCGTGCGTTCCGTGC</td><td>20</td><td>9</td><td>FGSG_04366</td><td>-</td></tr>
<tr><td>siRNA-t0075308</td><td>ACAACAGGTTGGAATCTGCG</td><td>20</td><td>9</td><td>FGSG_06666</td><td>-</td></tr>
<tr><td>siRNA-t0071201</td><td>ACACATTTTTGAAGATCGTGAA</td><td>22</td><td>9</td><td>FgHV1gp2</td><td>-</td></tr>
<tr><td>siRNA-t0075434</td><td>ACATCGTACCCGTCGAAGACC</td><td>21</td><td>9</td><td>FgHV1gp1</td><td>-</td></tr>
<tr><td>siRNA-t0071507</td><td>ACATGCCGACAGAGAACAGC</td><td>20</td><td>9</td><td>FGSG_10935</td><td>K08711|1|1e-79|298|kla:KLLA0D03476g|ATP-binding cassette, subfamily G (WHITE), member 2, PDR</td></tr>
<tr><td>siRNA-t0076314</td><td>ACCAGACAGAAAGGGGCCAA</td><td>20</td><td>9</td><td>FGSG_07594</td><td>K08141|1|4e-43|175|pcs:Pc20g12640|MFS transporter, SP family, general alpha glucoside:H+ symporter</td></tr>
<tr><td>siRNA-t0075962</td><td>ACCTAGACCGAAGAAAGACCA</td><td>21</td><td>9</td><td>FgHV1gp2</td><td>-</td></tr>
<tr><td>siRNA-t0075012</td><td>ACCTAGACCGAAGAAAGACCACATC</td><td>25</td><td>9</td><td>FgHV1gp2</td><td>-</td></tr>
<tr><td>siRNA-t0075172</td><td>ACGAGTGTGTCGCGTCTACCTTGTT</td><td>25</td><td>9</td><td>FgHV1gp2</td><td>-</td></tr>
<tr><td>siRNA-t0076846</td><td>ACGTGGAAATCTTCAGGCCCTGGGA</td><td>25</td><td>9</td><td>FgHV1gp2</td><td>-</td></tr>
<tr><td>siRNA-t0073841</td><td>ACGTGGATTTTGCTACCAGCTGC</td><td>23</td><td>9</td><td>FgHV1gp1</td><td>-</td></tr>
<tr><td>siRNA-t0072185</td><td>ACTGTTGACGTCACCCTTTGG</td><td>21</td><td>9</td><td>FgHV1gp2</td><td>-</td></tr>
<tr><td>siRNA-t0075656</td><td>AGCAGAGAAGAAGTCTGTTGC</td><td>21</td><td>9</td><td>FGSG_10617</td><td>K14859|1|2e-98|359|scm:SCHCODRAFT_255991|ribosome biogenesis protein SSF1/2;K00143|2|1e-14|82.0|zro:ZYRO0C16566g|L-aminoadipate-semialdehyde dehydrogenase [EC:1.2.1.31]</td></tr>
<tr><td>siRNA-t0073121</td><td>AGCATGGCCGGCAGTCCTAGCA</td><td>22</td><td>9</td><td>FgHV1gp2</td><td>-</td></tr>
<tr><td>siRNA-t0074217</td><td>AGCCTCTCCCTTCGTGAACTGCTG</td><td>24</td><td>9</td><td>FgHV1gp2</td><td>-</td></tr>
<tr><td>siRNA-t0076673</td><td>AGCGCGCGAAAATGTCTGGT</td><td>20</td><td>9</td><td>FGSG_00581</td><td>K01598|1|2e-13|77.0|uma:UM05916.1|phosphopantothenoylcysteine decarboxylase [EC:4.1.1.36]</td></tr>
<tr><td>siRNA-t0073804</td><td>AGCTGTTCTCTGTCGGCATGTTGC</td><td>24</td><td>9</td><td>FgHV1gp2</td><td>-</td></tr>
<tr><td>siRNA-t0071067</td><td>AGGAGGGTTCAATATCGGTA</td><td>20</td><td>9</td><td>FGSG_11234</td><td>K15505|1|7e-25|115|ssl:SS1G_03212|DNA repair protein RAD5 [EC:3.6.4.-];K15711|5|2e-21|103|tve:TRV_04890|SWI/SNF-related matrix-associated actin-dependent regulator of chromatin subfamily A3 [EC:3.6.4.- 6.3.2.19]</td></tr>
<tr><td>siRNA-t0073136</td><td>AGGCTTGTCGTCGGGAGAATCCAGA</td><td>25</td><td>9</td><td>FgHV1gp2</td><td>-</td></tr>
<tr><td>siRNA-t0073718</td><td>AGGGAGTTTGTACTTGCCGCGC</td><td>22</td><td>9</td><td>FGSG_02478</td><td>K14840|1|0.0|696|fgr:FG02478.1|nucleolar protein 53</td></tr>
<tr><td>siRNA-t0071210</td><td>AGTCGAATGAAATGGAACAACGTG</td><td>24</td><td>9</td><td>FgHV1gp2</td><td>-</td></tr>
<tr><td>siRNA-t0073724</td><td>AGTGTGTCGCGTCTACCTTG</td><td>20</td><td>9</td><td>FgHV1gp2</td><td>-</td></tr>
<tr><td>siRNA-t0071156</td><td>ATAAGGTGAGAAGATTGAGTTC</td><td>22</td><td>9</td><td>FgHV1gp2</td><td>-</td></tr>
<tr><td>siRNA-t0073025</td><td>ATAGATGGTGATTGTGGTCATTTGG</td><td>25</td><td>9</td><td>FgHV1gp2</td><td>-</td></tr>
<tr><td>siRNA-t0076317</td><td>ATAGCGTCGTCACCTTGATT</td><td>20</td><td>9</td><td>FGSG_13835</td><td>-</td></tr>
<tr><td>siRNA-t0071051</td><td>ATTGAACCACAGTCGAATGAA</td><td>21</td><td>9</td><td>FgHV1gp2</td><td>-</td></tr>
<tr><td>siRNA-t0073771</td><td>CAAATCTGGGTGGCGATGGAT</td><td>21</td><td>9</td><td>FgHV1gp2</td><td>-</td></tr>
<tr><td>siRNA-t0074710</td><td>CAAGGAGCTCAAAGGTATGA</td><td>20</td><td>9</td><td>FGSG_11489</td><td>-</td></tr>
<tr><td>siRNA-t0073376</td><td>CAATGGCGACACCGACGGTAC</td><td>21</td><td>9</td><td>FGSG_05709</td><td>K13830|1|2e-68|259|lbc:LACBIDRAFT_233717|pentafunctional AROM polypeptide [EC:4.2.3.4 4.2.1.10 1.1.1.25 2.7.1.71 2.5.1.19]</td></tr>
<tr><td>siRNA-t0074051</td><td>CAATGGCGACACCGACGGTACG</td><td>22</td><td>9</td><td>FGSG_05709</td><td>K13830|1|2e-68|259|lbc:LACBIDRAFT_233717|pentafunctional AROM polypeptide [EC:4.2.3.4 4.2.1.10 1.1.1.25 2.7.1.71 2.5.1.19]</td></tr>
<tr><td>siRNA-t0071046</td><td>CACGCAGCAGAATTCTAGCCTGG</td><td>23</td><td>9</td><td>FgHV1gp2</td><td>-</td></tr>
<tr><td>siRNA-t0074263</td><td>CACTGTTGACGTCACCCTTTGGAG</td><td>24</td><td>9</td><td>FgHV1gp2</td><td>-</td></tr>
<tr><td>siRNA-t0070967</td><td>CAGCGGCAAATACAGGACCGCGC</td><td>23</td><td>9</td><td>FgHV1gp2</td><td>-</td></tr>
<tr><td>siRNA-t0071350</td><td>CAGCTGCAGCAACTGTCGCCT</td><td>21</td><td>9</td><td>FGSG_12712</td><td>K10884|1|0.0|1132|fgr:FG05317.1|ATP-dependent DNA helicase 2 subunit 1</td></tr>
<tr><td>siRNA-t0071577</td><td>CAGGATCTCTGACATATTCGGACT</td><td>24</td><td>9</td><td>FgHV1gp2</td><td>-</td></tr>
<tr><td>siRNA-t0075820</td><td>CCACTCTAGCAATGCATCACCA</td><td>22</td><td>9</td><td>FgHV1gp1</td><td>-</td></tr>
<tr><td>siRNA-t0071452</td><td>CCAGAACCCAGCACCTTTCCCTA</td><td>23</td><td>9</td><td>FgHV1gp2</td><td>-</td></tr>
<tr><td>siRNA-t0072085</td><td>CCAGCTGCAGCAACTGTCGCCTG</td><td>23</td><td>9</td><td>FgHV1gp1</td><td>-</td></tr>
<tr><td>siRNA-t0074891</td><td>CCATAACCTCGTTGGGACCTTTCC</td><td>24</td><td>9</td><td>FgHV1gp2</td><td>-</td></tr>
<tr><td>siRNA-t0074142</td><td>CCATCAGGACCCGAGAAAAACGC</td><td>23</td><td>9</td><td>FgHV1gp2</td><td>-</td></tr>
<tr><td>siRNA-t0076049</td><td>CCATGACGTACGACCCCGAAA</td><td>21</td><td>9</td><td>FgHV1gp1</td><td>-</td></tr>
<tr><td>siRNA-t0071749</td><td>CCCATGCTTTCGACGTACTTG</td><td>21</td><td>9</td><td>FGSG_09930</td><td>K14326|1|0.0|2138|fgr:FG09930.1|regulator of nonsense transcripts 1 [EC:3.6.4.-]</td></tr>
<tr><td>siRNA-t0076098</td><td>CCCGAAAACAACACGCTTCCGGC</td><td>23</td><td>9</td><td>FgHV1gp2</td><td>-</td></tr>
<tr><td>siRNA-t0076539</td><td>CCGGACGGTGACCATTCTGA</td><td>20</td><td>9</td><td>FGSG_07499</td><td>K03032|1|0.0|2046|fgr:FG07499.1|26S proteasome regulatory subunit N2</td></tr>
<tr><td>siRNA-t0072782</td><td>CCGGATACGTTCTGAGAAGTCTCC</td><td>24</td><td>9</td><td>FgHV1gp2</td><td>-</td></tr>
<tr><td>siRNA-t0072142</td><td>CCGTACGGATCCACATGAGC</td><td>20</td><td>9</td><td>FGSG_01632</td><td>-</td></tr>
<tr><td>siRNA-t0075474</td><td>CGACAATGGCGCATACACGGAAC</td><td>23</td><td>9</td><td>FgHV1gp2</td><td>-</td></tr>
<tr><td>siRNA-t0072308</td><td>CGACCCATGCTTTCGACGTACT</td><td>22</td><td>9</td><td>FgHV1gp2</td><td>-</td></tr>
<tr><td>siRNA-t0072481</td><td>CGACGTACTTAGGGTTGTTG</td><td>20</td><td>9</td><td>FgHV1gp2</td><td>-</td></tr>
<tr><td>siRNA-t0072147</td><td>CGCTCCCACTATCGTCCTGC</td><td>20</td><td>9</td><td>FGSG_01294</td><td>K00799|1|1e-34|146|afv:AFLA_022030|glutathione S-transferase [EC:2.5.1.18]</td></tr>
<tr><td>siRNA-t0072674</td><td>CGGATGTAGGTCTGGCATAG</td><td>20</td><td>9</td><td>FGSG_01035</td><td>K03515|1|0.0|2355|fgr:FG01035.1|DNA repair protein REV1 [EC:2.7.7.-]</td></tr>
<tr><td>siRNA-t0075268</td><td>CGGTGTGGGTCTTCTGACGAATT</td><td>23</td><td>9</td><td>FgHV1gp2</td><td>-</td></tr>
<tr><td>siRNA-t0075620</td><td>CGTGGAAGGGTTCATACTAT</td><td>20</td><td>9</td><td>FGSG_07525</td><td>K11805|1|0.0|853|fgr:FG07525.1|WD repeat-containing protein 68</td></tr>
<tr><td>siRNA-t0076436</td><td>CGTGGGATTGGGCGTGTGTTCTTTA</td><td>25</td><td>9</td><td>FgHV1gp2</td><td>-</td></tr>
</table>

siRNA-t0075378	CGTTACAATCGTGGACTTCCTTT	23	9	FgHV1gp2	-
siRNA-t0075849	CTCCCACTATCGTCCTGCGT	20	9	FGSG_10572	-
siRNA-t0071595	CTGAAACCATCAGGACCCGAGAAAA	25	9	FgHV1gp2	-
siRNA-t0072433	CTGAATGCTCTGCCTCGGTCA	21	9	FgHV1gp2	-
siRNA-t0075127	CTGCGACCCATGCTTTCGACG	21	9	FgHV1gp2	-
siRNA-t0073737	CTGCGACCCATGCTTTCGACGTACT	25	9	FgHV1gp2	-
siRNA-t0075085	CTTGCTGTAGTGTCTGAAGATCT	23	9	FgHV1gp2	-
siRNA-t0071462	CTTGTTCTGGAACGTCAAATCAAGT	25	9	FgHV1gp2	-
siRNA-t0071786	GAAGGCGTGTTTTTCAATGA	20	9	FGSG_08209	K01932|1|0.0|1305|afm:AFUA_5G12730|[EC:6.3.2.-]
siRNA-t0071057	GACACCGACGGTACGTGGAA	20	9	FgHV1gp2	-
siRNA-t0071325	GACCAGCTGTTCTCTGTCGGC	21	9	FgHV1gp2	-
siRNA-t0073063	GACGCGTGTCGTTAGAAGCTTG	22	9	FgHV1gp2	-
siRNA-t0076598	GACGCGTGTCGTTAGAAGCTTGACA	25	9	FgHV1gp2	-
siRNA-t0072296	GACGTACTTAGGGTTGTTGA	20	9	FGSG_10940	K14564|1|0.0|942|fgr:FG10940.1|nucleolar protein 56
siRNA-t0071112	GAGCGTTTGTGGAGCCATGG	20	9	FGSG_11234	K15505|1|7e-25|115|ssl:SS1G_03212|DNA repair protein RAD5 [EC:3.6.4.-];K15711|5|2e-21|103|tve:TRV_04890|SWI/SNF-related matrix-associated actin-dependent regulator of chromatin subfamily A3 [EC:3.6.4.- 6.3.2.19]
siRNA-t0071971	GAGTGCTGAATGCTCTGCCTCGG	23	9	FgHV1gp2	-
siRNA-t0073463	GAGTGGGATGGGTTTCTGGTT	21	9	FGSG_05683	K00485|1|5e-159|560|mgr:MGG_03330|dimethylaniline monooxygenase (N-oxide forming) [EC:1.14.13.8]
siRNA-t0076368	GATCATCAGCACCGTACGGATCC	23	9	FgHV1gp2	-
siRNA-t0076876	GATCCCACTCTAGCAATGCATCAC	24	9	FgHV1gp1	-
siRNA-t0071879	GATGGACTGGCCTGTTGTCAC	21	9	FgHV1gp2	-
siRNA-t0074969	GCAACGGAAGCGATATTTGCACCAT	25	9	FgHV1gp2	-
siRNA-t0070991	GCAAGCGTGACGAAGATCCCAC	22	9	FgHV1gp1	-
siRNA-t0071474	GCACAAGAACATCAGGCATG	20	9	FGSG_12863	K02324|1|0.0|3986|fgr:FG06065.1|DNA polymerase epsilon subunit 1 [EC:2.7.7.7]
siRNA-t0074984	GCAGAATTCTAGCCTGGTTGAGATC	25	9	FgHV1gp2	-
siRNA-t0072407	GCATAACACGCAGCAGAATTCTAGC	25	9	FgHV1gp2	-
siRNA-t0073174	GCCAAATCGTCATACATTGC	20	9	FGSG_11659	K01932|1|0.0|1431|afm:AFUA_5G12730|[EC:6.3.2.-]
siRNA-t0074575	GCCATGACGTACGACCCCGAAAG	23	9	FgHV1gp1	-
siRNA-t0073011	GCCATGACGTACGACCCCGAAAGT	24	9	FgHV1gp1	-
siRNA-t0072839	GCGACACCGACGGTACGTGGAAATC	25	9	FgHV1gp2	-
siRNA-t0073747	GCGACCCATGCTTTCGACGTACTT	24	9	FgHV1gp2	-
siRNA-t0071178	GCTGCCGCGGATAACCTATACTTG	24	9	FgHV1gp2	-
siRNA-t0075212	GCTGTAGTGTCTGAAGATCTCAG	23	9	FgHV1gp2	-
siRNA-t0072538	GCTGTTCTCTGTCGGCATGTTGCC	24	9	FGSG_11657	K09184|1|5e-13|75.9|bfu:BC1G_09852|GATA-binding protein, other eukaryote
siRNA-t0073662	GCTGTTGGTACTCCTATAGC	20	9	FGSG_01424	K15560|1|0.0|1154|fgr:FG01424.1|protein NRD1
siRNA-t0072354	GCTTGTCGTCGGGAGAATCC	20	9	FGSG_05462	K01072|1|2e-159|564|afm:AFUA_2G08440|ubiquitin thiolesterase [EC:3.1.2.15];K11838|3|3e-41|171|clu:CLUG_02963|ubiquitin carboxyl-terminal hydrolase 7 [EC:3.1.2.15]
siRNA-t0075767	GGACGTTGTGGACGCCTATAA	21	9	FGSG_05908	K01528|1|5e-14|79.3|cpw:CPC735_039510|dynamin GTPase [EC:3.6.5.5]
siRNA-t0074495	GGATCTCTGACATATTCGGACTCAT	25	9	FgHV1gp2	-
siRNA-t0074620	GGCCCAGTCATGAATTTTCGGTA	23	9	FgHV1gp2	-
siRNA-t0074652	GGGAAGGAGACACCATCATG	20	9	FGSG_05844	K05236|1|2e-59|230|spo:SPBPJ4664.04|coatomer protein complex, subunit alpha (xenin)
siRNA-t0072691	GGGGTGTGTATCCAATGAGTTGTGG	25	9	FgHV1gp2	-
siRNA-t0071220	GGTAGTCATCGGGAATCATGTC	22	9	FgHV1gp2	-
siRNA-t0071920	GGTGGTTCATTGCGATGTAATCT	23	9	FgHV1gp2	-
siRNA-t0072062	GGTTCTTGGAAAGTTGGAGCCCT	23	9	FgHV1gp2	-
siRNA-t0073292	GTAAGCTGCTACTCGAACCGCCCT	24	9	FgHV1gp2	-
siRNA-t0076152	GTACCATTGCTGCATGTACTGTT	23	9	FgHV1gp2	-
siRNA-t0076105	GTACGTGGAAATCTTCAGGC	20	9	FGSG_11796	-
siRNA-t0075793	GTCGAAAGTGAAAGTGTCGTAGGAA	25	9	FgHV1gp1	-
siRNA-t0075349	GTCTCTTGTTCTGGAACGTCA	21	9	FGSG_13060	-
siRNA-t0074738	GTCTTGCGGTGTGGGTCTTCTGAC	24	9	FgHV1gp2	-
siRNA-t0075076	GTGCGTAGCATCGATGAACCT	21	9	FGSG_08634	K09184|1|0.0|1634|fgr:FG08634.1|GATA-binding protein, other eukaryote
siRNA-t0076187	GTGTGTCGCGTCTACCTTGTTCAGG	25	9	FgHV1gp2	-
siRNA-t0075100	GTTATAAGGCTTGTCGTCGGGAG	23	9	FgHV1gp2	-
siRNA-t0074803	GTTCAAGTATCGTCACTTCTT	21	9	FGSG_05959	-
siRNA-t0073309	GTTCCATAAACGCTGCCGGGAT	22	9	FgHV1gp2	-
siRNA-t0075630	GTTCTCTGTCGGCATGTTGCC	21	9	FGSG_11657	K09184|1|5e-13|75.9|bfu:BC1G_09852|GATA-binding protein, other eukaryote
siRNA-t0072953	GTTCTGAAATTTGATGTTGTAGAAC	25	9	FgHV1gp2	-
siRNA-t0076526	TAATTGGTTCTTTAGGTTGAT	21	9	FgHV1gp2	-
siRNA-t0072009	TACAATCGTGGACTTCCTTT	20	9	FgHV1gp2	-
siRNA-t0074967	TACAGGACCGCGCGATGGCGCGC	23	9	FgHV1gp2	-
siRNA-t0073274	TACTTGATCTCAGCACGCCG	20	9	FGSG_01603	K01046|1|5e-177|620|afm:AFUA_8G02530|triacylglycerol lipase [EC:3.1.1.3];K01066|5|3e-43|175|mgr:MGG_00499|esterase / lipase [EC:3.1.1.-]
siRNA-t0071893	TAGTCATCGGGAATCATGTCGG	22	9	FgHV1gp2	-
siRNA-t0073537	TCAAGCAACAGTCAGACACGAT	22	9	FGSG_08389	-
siRNA-t0072226	TCAATGATGGCACGTCGACA	20	9	FgHV1gp2	-
siRNA-t0076845	TCCCATAGAATGTCGAGATA	20	9	FGSG_13878	K01932|1|0.0|1450|afm:AFUA_5G12730|[EC:6.3.2.-]
siRNA-t0071725	TCGTGAACCTGTGGTGTCAA	20	9	FGSG_11750	-
siRNA-t0070963	TCGTTAGAAGCTTGACACGAA	21	9	FgHV1gp2	-
siRNA-t0074666	TCTATGTTTGACGGCTTGTCCT	22	9	FGSG_08719	K10352|1|0.0|4469|fgr:FG08719.1|myosin heavy chain
siRNA-t0074102	TCTCTTGTTCTGGAACGTCAA	21	9	FGSG_13060	-
siRNA-t0074658	TCTGACTGTAGTGTGCCTTGTT	22	9	FGSG_07969	-
siRNA-t0075499	TCTGGAACGTCAAATCAAGTTGGT	24	9	FgHV1gp2	-
siRNA-t0073801	TCTGGCCGGTTGAAAATGTTCC	22	9	FgHV1gp2	-
siRNA-t0074267	TCTTGTTCTGGAACGTCAAATC	22	9	FGSG_06754	K06669|1|0.0|2432|fgr:FG06754.1|structural maintenance of chromosome 3 (chondroitin sulfate proteoglycan 6)
siRNA-t0071526	TGAAAAAGCACAACATCTGGA	21	9	FGSG_13783	K01932|1|0.0|1526|afm:AFUA_5G12730|[EC:6.3.2.-]
siRNA-t0071556	TGATGTTCTTGTGCCTCCGA	20	9	FGSG_12776	K12879|1|0.0|2720|fgr:FG05657.1|THO complex subunit 2
siRNA-t0075103	TGCATTACAAGAATCGGGGC	20	9	FGSG_12500	-
siRNA-t0071131	TGCGCCATTGTCGGAGGAGCC	21	9	FGSG_03859	-
siRNA-t0076803	TGGCGACACCGACGGTACGTGG	22	9	FgHV1gp2	-
siRNA-t0075664	TGGCGCATACACGGAACCCATTGAT	25	9	FgHV1gp2	-
siRNA-t0071999	TGGCTCGTGACTTTTCCCTC	20	9	FGSG_00581	K01598|1|2e-13|77.0|uma:UM05916.1|phosphopantothenoylcysteine decarboxylase [EC:4.1.1.36]
siRNA-t0073053	TGGGCGTGTGTTCTTTATGGGCTCA	25	9	FgHV1gp2	-
siRNA-t0075874	TGGTATTGTCAGGGTTCAAA	20	9	FGSG_00843	K03021|1|0.0|2410|fgr:FG00843.1|DNA-directed RNA polymerase III subunit RPC2 [EC:2.7.7.6]
siRNA-t0075566	TGTTATAAGGCTTGTCGTCGGGA	23	9	FGSG_08614	K01874|1|0.0|1256|fgr:FG08614.1|methionyl-tRNA synthetase [EC:6.1.1.10]
siRNA-t0074205	TTCCATTGATAATCGGCATGA	21	9	FGSG_00104	-
siRNA-t0074564	TTCTACCAGAACCCAGCACCTT	22	9	FgHV1gp2	-
siRNA-t0072428	TTCTCGTGGGATTGGGCGTGTGTTC	25	9	FgHV1gp2	-
siRNA-t0075432	TTGAATCGATCGTCATGGTGATAC	24	9	FgHV1gp2	-
siRNA-t0075015	TTGGGGTCTTGACTTGGGCTGT	22	9	FGSG_09304	-
siRNA-t0079438	AACACTAATATCGTCATTCTCTT	23	8	FgHV1gp2	-
siRNA-t0080780	AACGCTGCCGGGATGTAATCAA	22	8	FgHV1gp2	-
siRNA-t0083871	AACGGGTCCAGATGTTGTGC	20	8	FGSG_07939	K05236|1|0.0|2545|fgr:FG07939.1|coatomer protein complex, subunit alpha (xenin)
siRNA-t0078453	AAGGCGTGTCTGAATGGAAT	20	8	FGSG_13878	K01932|1|0.0|1450|afm:AFUA_5G12730|[EC:6.3.2.-]

siRNA-t0082770	AAGTCTCTTGTTCTGGAACGT	21	8	FGSG_02381	-
siRNA-t0078241	ACAATCGTGGACTTCCTTTA	20	8	FgHV1gp2	-
siRNA-t0082463	ACAATGGCGCATACACGGAACCC	23	8	FgHV1gp2	-
siRNA-t0078807	ACAGGCCTCATTGATTTTGTG	21	8	FgHV1gp2	-
siRNA-t0076940	ACATAGCGTCGTCACCTTGAT	21	8	FGSG_13835	-
siRNA-t0080697	ACATTTTATTGTAAGTCTGGAAT	23	8	FgHV1gp2	-
siRNA-t0078124	ACCCGTCGAAGACCAAAAATT	21	8	FgHV1gp1	-
siRNA-t0084277	ACCGATGGAAGTGATGGCAA	20	8	FGSG_08209	K01932|1|0.0|1305|afm:AFUA_5G12730|[EC:6.3.2.-]
siRNA-t0078395	AGAGAAGAAAGGTGCGAGCAG	21	8	FGSG_08773	K00966|1|0.0|712|fgr:FG08773.1|mannose-1-phosphate guanylyltransferase [EC:2.7.7.13]
siRNA-t0082460	AGCGTGAACCAGATCATAACC	21	8	FgHV1gp2	-
siRNA-t0080674	AGGGAAGGAGACACCATCAT	20	8	FGSG_05844	K05236|1|2e-59|230|spo:SPBPJ4664.04|coatomer protein complex, subunit alpha (xenin)
siRNA-t0081193	AGTCCACGATTGTAACGAAT	20	8	FGSG_09968	-
siRNA-t0078297	ATAGAATGTCGAGATACTCT	20	8	FGSG_01790	K00680|1|0.0|2387|afm:AFUA_3G14700|[EC:2.3.1.-];K15419|2|0.0|1077|ani:AN2547.2|emericellamide biosynthesis, highly reducing iterative type I polyketide synthase;K15384|5|0.0|762|afv:AFLA_066840|aspyridone synthetase, hybrid polyketide synthase / nonribosomal peptide synthetase
siRNA-t0082725	ATGCTTCCGGATTATGCCCAA	21	8	FgHV1gp2	-
siRNA-t0083077	CAAATACAGGACCGCGCGATGGCG	24	8	FgHV1gp2	-
siRNA-t0083588	CAACCACAAGCCACTGCAGTT	21	8	FgHV1gp2	-
siRNA-t0083554	CAACGTCAGAGGCCTCGTCAC	21	8	FGSG_07499	K03032|1|0.0|2046|fgr:FG07499.1|26S proteasome regulatory subunit N2
siRNA-t0079754	CAAGATGGACTGGCCTGTTGTCA	23	8	FgHV1gp2	-
siRNA-t0083604	CAAGCAACAGTCAGACACGA	20	8	FGSG_08389	-
siRNA-t0078492	CAAGGGACGGAAGCCATCAA	20	8	FGSG_09996	-
siRNA-t0082749	CACGCTCGGGGTCAATTTGATTGA	24	8	FgHV1gp2	-
siRNA-t0076957	CAGAAACCCATCCCACTCGTG	21	8	FgHV1gp2	-
siRNA-t0082386	CAGATGCGAAGCGAATATCGGA	22	8	FGSG_03912	K09246|1|3e-07|55.8|lth:KLTH0E16500g|arginine metabolism regulation protein II
siRNA-t0079966	CAGCACCGTACGGATCCACATG	22	8	FgHV1gp2	-
siRNA-t0079910	CAGGACCCGAGAAAAACGCCCC	22	8	FgHV1gp2	-
siRNA-t0080276	CAGGCCAACAAAGAGAACTG	20	8	FGSG_06226	K15084|1|0.0|743|fgr:FG06226.1|solute carrier family 25 (mitochondrial carrier protein), member 16
siRNA-t0082901	CAGTTCATGAATGCCGCCGTA	21	8	FGSG_12253	-
siRNA-t0079606	CCAGTGTCGAACCATACTGGACCC	24	8	FgHV1gp2	-
siRNA-t0080781	CCATCAACGGTTCTTGGAAAGTT	23	8	FgHV1gp2	-
siRNA-t0081395	CCCATGCTTTCGACGTACTTGG	22	8	FgHV1gp2	-
siRNA-t0078331	CCCGTCGAAGACCAAAAATTTCT	23	8	FgHV1gp1	-
siRNA-t0080073	CCCTTTGGAGACTTGGCAGTG	21	8	FgHV1gp2	-
siRNA-t0081100	CCGAAGGAACTCCACATAGCGTCG	24	8	FgHV1gp2	-
siRNA-t0077063	CCGAGATTTGGCCAACCCGT	20	8	FGSG_12230	-
siRNA-t0083038	CCGCAGAAGAATTAGCCTTGAA	23	8	FgHV1gp2	-
siRNA-t0078995	CCGCGAATTCTGCCATAACCTCGTT	25	8	FgHV1gp2	-
siRNA-t0080545	CCGGAATGCGTTCACACGATT	21	8	FgHV1gp2	-
siRNA-t0079350	CCTAGAGATCTCCGCAGAAGAATT	24	8	FgHV1gp2	-
siRNA-t0080212	CCTAGCAAGTGAAACTGTTCGCCA	24	8	FgHV1gp2	-
siRNA-t0079372	CCTATACTTGATCTCAGCACGCC	23	8	FgHV1gp2	-
siRNA-t0080018	CCTATAGATCGCGCGTAAGCT	21	8	FgHV1gp2	-
siRNA-t0077087	CCTCAAGGGACGGAAGCCATC	21	8	FGSG_09996	-
siRNA-t0080863	CGAAGAAGTGACGATACTTGA	21	8	FGSG_03153	-
siRNA-t0076930	CGCGTGTCGTTAGAAGCTTGAC	22	8	FgHV1gp2	-
siRNA-t0078799	CGGGTCCTGATGGTTTCAGA	20	8	FGSG_02471	-
siRNA-t0077913	CGTGAACCAGATCATAACCGCATA	25	8	FgHV1gp2	-
siRNA-t0080717	CTCGATCGCATATCCTTCGGTA	22	8	FgHV1gp2	-
siRNA-t0079918	CTCTGTGACTCCAGCCAAAGA	21	8	FgHV1gp2	-
siRNA-t0083208	CTCTTGTTCTGGAACGTCAA	20	8	FGSG_13060	-
siRNA-t0077395	CTGAAACCATCAGGACCCGAGA	22	8	FgHV1gp2	-
siRNA-t0079931	CTGACATATTCGGACTCATCCCACA	25	8	FgHV1gp2	-
siRNA-t0078680	CTGACGATGACTACGATGAT	20	8	FGSG_11298	K03379|1|3e-24|112|uma:UM03415.1|cyclohexanone monooxygenase [EC:1.14.13.22];K00540|3|7e-17|87.8|afm:AFUA_8G00440|[EC:1.-.-.-];K00485|4|2e-16|86.3|tml:GSTUM_00002301001|dimethylaniline monooxygenase (N-oxide forming) [EC:1.14.13.8]
siRNA-t0078744	CTGATGATCATGACGTGGAG	20	8	FGSG_11121	-
siRNA-t0078107	CTGCGGAGATCTCTAGGATG	20	8	FGSG_10548	K15418|1|0.0|1191|pcs:Pc21g12440|zearalenone synthase, highly reducing iterative type I polyketide synthase;K15419|4|0.0|740|ani:AN2547.2|emericellamide biosynthesis, highly reducing iterative type I polyketide synthase
siRNA-t0080966	CTGGATGTGGGTGCGCCGAATGAA	24	8	FgHV1gp2	-
siRNA-t0082988	CTTAGAATGACGGCGCTCCGTG	22	8	FgHV1gp2	-
siRNA-t0082133	CTTAGAATGACGGCGCTCCGTGA	23	8	FgHV1gp2	-
siRNA-t0080199	CTTCAGGCCCTGGGATTGTATGGTA	25	8	FgHV1gp2	-
siRNA-t0077284	CTTGAAGGCGAAGTATGAGG	20	8	FGSG_03202	K01537|1|0.0|1056|pcs:Pc13g12970|Ca2+-transporting ATPase [EC:3.6.3.8]
siRNA-t0082052	CTTGGTTCAAGTATCGTCAC	20	8	FgHV1gp2	-
siRNA-t0078762	CTTGTTTCTCGTGGGATTGGG	21	8	FgHV1gp2	-
siRNA-t0078527	GAAAAACGCCCCTGGACGGTACT	23	8	FgHV1gp2	-
siRNA-t0080559	GAACGCTGAAATCGAGGGTGTTGG	24	8	FgHV1gp2	-
siRNA-t0081919	GAAGAGACAGAGCTCGCGCCT	21	8	FgHV1gp2	-
siRNA-t0077628	GACCTTGAGACAGTTCCGCGTGT	23	8	FgHV1gp2	-
siRNA-t0081030	GACGACCGTGCGAAATTGTTTA	22	8	FgHV1gp2	-
siRNA-t0081617	GATCTCAGCACGCCGGTTACCAA	23	8	FgHV1gp2	-
siRNA-t0078760	GATTGTGGTCATTTGGTTTGT	21	8	FgHV1gp2	-
siRNA-t0077225	GCAGGATCGTTATTCTTCTTCT	22	8	FGSG_00698	-
siRNA-t0083322	GCATGTAGATCTTGCTGTAG	20	8	FGSG_04561	-
siRNA-t0079271	GCCAGAATCGAGCTAAACGAAC	22	8	FgHV1gp2	-
siRNA-t0081325	GCCGGCAGTCCTAGCAAGTGA	21	8	FGSG_06071	K11771|1|0.0|1014|fgr:FG06071.1|SWI/SNF chromatin-remodeling complex subunit SWI1
siRNA-t0081657	GCCTCTCCCTTCGTGAACTGC	21	8	FGSG_06193	K08773|1|2e-20|101|cci:CC1G_05669|RalA-binding protein 1
siRNA-t0081208	GCCTGGGAGACAGAGCCTGAGTC	23	8	FgHV1gp2	-
siRNA-t0080373	GCGAATTCTGCCATAACCTCGT	22	8	FgHV1gp2	-
siRNA-t0077288	GCGACCCATGCTTTCGACGTAC	22	8	FgHV1gp2	-
siRNA-t0078974	GCGAGACCGATGATTTCCATCCCCT	25	8	FgHV1gp2	-
siRNA-t0079387	GCGATGGCTTATGCTGGCAT	20	8	FGSG_02429	-
siRNA-t0077315	GCGCCTTCCATGCTGCCGCGGA	22	8	FGSG_04587	K00777|1|7e-77|289|afm:AFUA_7G07100|[EC:2.4.2.-];K14855|4|5e-55|216|lbc:LACBIDRAFT_296068|ribosome assembly protein 4
siRNA-t0077474	GCTGCTACTCGAACCGCCCT	20	8	FgHV1gp2	-
siRNA-t0078902	GCTGTACTGAAGGTGGTGTC	20	8	FGSG_13828	K09240|1|3e-08|59.3|sce:YMR280C|transcriptional regulatory protein CAT8;K09241|2|9e-07|54.7|ani:AN4785.2|transcriptional regulatory protein GAL4
siRNA-t0080353	GCTTGGTTTCGGGACGCGACAAGCA	25	8	FgHV1gp2	-
siRNA-t0078967	GCTTTCGACGTACTTGGGTTCATA	24	8	FgHV1gp2	-
siRNA-t0082329	GGAAAGGAAAAGTTCTCGCCAGC	23	8	FgHV1gp1	-
siRNA-t0082388	GGAACTCCACATAGCGTCGT	20	8	FgHV1gp2	-
siRNA-t0078725	GGAGGGATACCAACTTGATT	20	8	FGSG_11680	K06694|1|1e-13|77.8|clu:CLUG_01956|26S proteasome non-ATPase regulatory subunit 10
siRNA-t0079080	GGATCTTTTGGCATCATCCT	20	8	FGSG_09406	K03128|1|0.0|2761|fgr:FG09406.1|transcription initiation factor TFIID subunit 2

siRNA-t0084300	GGCAGTCCTAGCAAGTGAAACTGT	24	8	FgHV1gp2	-
siRNA-t0081993	GGCCTCTGACGTTGATCTCTTAT	23	8	FgHV1gp2	-
siRNA-t0084247	GGCGAGACCGATGATTTCCATCC	23	8	FgHV1gp2	-
siRNA-t0083634	GGCGCATACACGGAACCCAT	20	8	FgHV1gp2	-
siRNA-t0080447	GGCGTTTTTCTCGGGTCCTGA	21	8	FGSG_03762	K01175｜1｜1e-18｜94.0｜afm:AFUA_4G12720｜[EC:3.1.-.-]
siRNA-t0079894	GGCTTGTCGTCGGGAGAATCCAGAA	25	8	FgHV1gp2	-
siRNA-t0081962	GGGGCCACCTGAAGGATTTAA	21	8	FGSG_09133	-
siRNA-t0081474	GGGTGGATCTGGGGTCTGAC	20	8	FGSG_09943	-
siRNA-t0079175	GGGTGGTTGCAGCGTGAACCAG	22	8	FgHV1gp2	-
siRNA-t0077762	GGGTTGGCCAAATCTCGGACC	21	8	FgHV1gp2	-
siRNA-t0081845	GGTGGGTTCTTGAAGTTGGTGCCGA	25	8	FgHV1gp2	-
siRNA-t0083939	GGTTATGGCAGAATTCGCGG	20	8	FGSG_00890	K14552｜1｜3e-92｜338｜ani:AN9058.2｜NET1-associated nuclear protein 1 (U3 small nucleolar RNA-associated protein 17)
siRNA-t0080008	GTCCCATAGAATGTCGAGATA	21	8	FGSG_13878	K01932｜1｜0.0｜1450｜afm:AFUA_5G12730｜[EC:6.3.2.-]
siRNA-t0082662	GTCTGACTGTTGCTTGATGGTT	22	8	FgHV1gp2	-
siRNA-t0082339	GTGAACCAGATCATAACCCGCAT	23	8	FgHV1gp2	-
siRNA-t0080681	GTGATACAAACCGGAATGCACT	22	8	FgHV1gp2	-
siRNA-t0080170	GTGGGATCTTCGTCACGCTT	20	8	FGSG_07460	-
siRNA-t0080890	GTGTCGGCTGCCAAGTAAGC	20	8	FGSG_12344	K01620｜1｜2e-117｜421｜act:ACLA_008260｜threonine aldolase [EC:4.1.2.5]
siRNA-t0083951	GTTATCGTGTCTGACTGTTGCTT	23	8	FgHV1gp2	-
siRNA-t0083645	GTTCCTTCGGAAAGTATGGT	20	8	FGSG_10283	-
siRNA-t0079902	GTTCTCTCTGACCTTGATGGCG	22	8	FGSG_01555	K00784｜1｜2e-34｜146｜ani:AN0951.2｜ribonuclease Z [EC:3.1.26.11]
siRNA-t0077550	GTTCTGTGAACACTAATATCGTC	23	8	FgHV1gp2	-
siRNA-t0083186	GTTCTGTGAACACTAATATCGTCAT	25	8	FgHV1gp2	-
siRNA-t0078736	TAATATCGTCATTCTCTTCCAGT	23	8	FgHV1gp2	-
siRNA-t0081717	TAGCCATTCGTTACAATCGTGGACT	25	8	FgHV1gp2	-
siRNA-t0078584	TCAAGGAGCTCAAAGGTATGA	21	8	FGSG_11489	-
siRNA-t0082967	TCAAGGATTTGTAAGGGGAAA	21	8	FGSG_12660	-
siRNA-t0081520	TCCGGACGGTGACCATTCTG	20	8	FGSG_02632	K05349｜1｜0.0｜1666｜fgr:FG02632.1｜beta-glucosidase [EC:3.2.1.21]
siRNA-t0081085	TCCTGACACTGACAAGCAGC	20	8	FGSG_07119	K01613｜1｜5e-09｜63.5｜ure:UREG_00179｜phosphatidylserine decarboxylase [EC:4.1.1.65]
siRNA-t0080569	TCGAAACGTTGATCAGTGCAA	21	8	FGSG_11416	-
siRNA-t0078157	TCGCTGATCTGGCTCGTGACTT	22	8	FgHV1gp2	-
siRNA-t0082751	TCGGGGTCGTACGTCATGGC	20	8	FGSG_11097	K00799｜1｜2e-43｜177｜pno:SNOG_00220｜glutathione S-transferase [EC:2.5.1.18];K04618｜2｜5e-12｜72.8｜bfu:BC1G_12145｜galactose oxidase [EC:1.1.3.9];K01238｜5｜1e-10｜68.2｜afm:AFUA_3G01650｜[EC:3.2.1.-]
siRNA-t0079550	TCGGGTCCTGATGGTTTCAGA	21	8	FGSG_06016	K05727｜1｜1e-46｜188｜mpr:MPER_07660｜dedicator of cytokinesis 3/4/5
siRNA-t0079943	TCGTCATACATTGCACGAGGGACGA	25	8	FgHV1gp2	-
siRNA-t0079016	TCTCTTGTTCTGGAACGTCAAAT	23	8	FgHV1gp2	-
siRNA-t0079220	TCTGAATGGAATAGATTCACG	21	8	FGSG_05512	K11397｜1｜0.0｜2685｜fgr:FG05512.1｜chromatin modification-related protein VID21
siRNA-t0077627	TCTGACATATTCGGACTCATCCCAC	25	8	FgHV1gp2	-
siRNA-t0079000	TCTGACTGTAGTGTGCCTTG	20	8	FGSG_11659	K01932｜1｜0.0｜1431｜afm:AFUA_5G12730｜[EC:6.3.2.-]
siRNA-t0077975	TCTGACTGTAGTGTGCCTTGTTT	23	8	FGSG_07969	-
siRNA-t0083998	TCTTCTCCACGTCATGATCATCA	23	8	FGSG_01569	-
siRNA-t0078117	TCTTCTGACGAATTTGGACAAGTA	24	8	FgHV1gp2	-
siRNA-t0079203	TCTTGCGGTGTGGGTCTTCTGA	22	8	FgHV1gp2	-
siRNA-t0079320	TCTTGGAAAGTTGGAGCCCTTCT	23	8	FgHV1gp2	-
siRNA-t0082197	TCTTTCCTCTTGATGCGCACCAT	23	8	FgHV1gp2	-
siRNA-t0083146	TGAAACCATCAGGACCCGAG	20	8	FGSG_10402	-
siRNA-t0080998	TGAACCACAGTCGAATGAAATGGAA	25	8	FgHV1gp2	-
siRNA-t0077596	TGATCTCAGCACGCCGGTTACCAA	24	8	FgHV1gp2	-
siRNA-t0078379	TGCGCTTCCCATGACTGCTA	20	8	FGSG_07358	K01282｜1｜1e-19｜98.2｜ago:AGOS_ACR073C｜dipeptidyl aminopeptidase [EC:3.4.14.-];K01278｜2｜6e-19｜95.5｜ure:UREG_01669｜dipeptidyl-peptidase 4 [EC:3.4.14.5]
siRNA-t0078020	TGCTTGGTTTCGGGACGCGAC	21	8	FgHV1gp2	-
siRNA-t0084297	TGCTTTCGACGTACTTGGGT	20	8	FgHV1gp2	-
siRNA-t0082535	TGCTTTGGTTGAGACGCATT	20	8	FGSG_01480	K15326｜1｜0.0｜773｜fgr:FG01480.1｜tRNA-splicing endonuclease subunit Sen54
siRNA-t0078930	TGGCGACACCGACGGTACGT	20	8	FgHV1gp2	-
siRNA-t0077455	TGTCCCATAGAATGTCGAGA	20	8	FGSG_13153	K01932｜1｜2e-65｜249｜afm:AFUA_3G12920｜[EC:6.3.2.-];K12743｜5｜1e-55｜217｜afv:AFLA_070860｜N-(5-amino-5-carboxypentanoyl)-L-cysteinyl-D-valine synthase [EC:6.3.2.26]
siRNA-t0083026	TGTCGAACCATACTGGACCC	20	8	FGSG_08097	K00688｜1｜7e-16｜87.0｜pno:SNOG_13460｜starch phosphorylase [EC:2.4.1.1]
siRNA-t0077245	TGTGCTCATCGATGCAGGCA	20	8	FGSG_07628	K00480｜1｜1e-49｜196｜nfi:NFIA_002580｜salicylate hydroxylase [EC:1.14.13.1]
siRNA-t0077931	TGTTCATTTCGGCGAGTCTA	20	8	FgHV1gp2	-
siRNA-t0077537	TGTTTGCTTGGTTTCGGGACGC	22	8	FgHV1gp2	-
siRNA-t0080889	TTATGGCAGAATTCGCGGAC	20	8	FGSG_10154	-
siRNA-t0079628	TTCAGGCCCTGGGATTGTATGGTA	24	8	FgHV1gp2	-
siRNA-t0077068	TTCAGGCCCTGGGATTGTATGGTAA	25	8	FgHV1gp2	-
siRNA-t0079094	TTCTACCAGAACCCAGCACCTTT	23	8	FgHV1gp2	-
siRNA-t0077141	TTGTAGTAAAGTTATCGTGTCTGAC	25	8	FgHV1gp2	-
siRNA-t0079323	TTGTCGAGTCGAAAGCGGTC	20	8	FGSG_11279	-
siRNA-t0078426	TTTACAAGCTGGTCTGGGTGCTG	23	8	FgHV1gp2	-
siRNA-t0084509	AAACATAGACACTTCAACAT	20	7	FgHV1gp2	-
siRNA-t0088707	AAGTCTGGAATTCCTTGGGAGGCC	24	7	FgHV1gp2	-
siRNA-t0086366	AATCGGCATGAACTCGGTCAAC	22	7	FgHV1gp2	-
siRNA-t0087545	ACAAATCTGGGTGGCGATGGA	21	7	FGSG_00637	K02147｜1｜0.0｜1045｜fgr:FG00637.1｜V-type H+-transporting ATPase subunit B [EC:3.6.3.14]
siRNA-t0091916	ACAAATCTGGGTGGCGATGGAT	22	7	FgHV1gp2	-
siRNA-t0090756	ACAGAGCTGGGGATGTTTGCTT	22	7	FgHV1gp2	-
siRNA-t0086978	ACAGGCTAGGAACGATGCCTCGT	23	7	FGSG_02233	-
siRNA-t0086450	ACAGGCTTTGGTTCCAATTG	20	7	FGSG_08999	-
siRNA-t0088114	ACATCACGCTCGGGGTCAATT	21	7	FGSG_04102	K00654｜1｜0.0｜1186｜fgr:FG04102.1｜serine palmitoyltransferase [EC:2.3.1.50]
siRNA-t0088185	ACATGATGAGTAAGCGGGCT	20	7	FGSG_03736	K14859｜1｜6e-27｜122｜scm:SCHCODRAFT_255991｜ribosome biogenesis protein SSF1/2
siRNA-t0085306	ACATTGATCCAGAAGCGCACGA	22	7	FGSG_01364	-
siRNA-t0092759	ACATTGCACGAGGGACGAGCTTGG	24	7	FgHV1gp2	-
siRNA-t0093196	ACATTGCACGAGGGACGAGCTTGGC	25	7	FgHV1gp2	-
siRNA-t0092752	ACCATACTTTCCGAAGGAACTCC	23	7	FgHV1gp2	-
siRNA-t0090126	ACGAGGCCTCTGACGTTGATCTCT	24	7	FgHV1gp2	-
siRNA-t0085566	ACGGGACCGACGTTTTGGCGAGAC	24	7	FgHV1gp2	-
siRNA-t0092624	ACGTCATGATCATCAGCACCGTA	23	7	FgHV1gp2	-
siRNA-t0084719	ACGTGGAAATCTTCAGGCCCTG	22	7	FgHV1gp2	-
siRNA-t0084505	ACGTGGAAATCTTCAGGCCCTGGG	24	7	FgHV1gp2	-
siRNA-t0090049	ACTAATATCGTCATTCTCTTCCA	23	7	FGSG_09836	-
siRNA-t0085499	ACTAATATCGTCATTCTCTTCCAGT	25	7	FgHV1gp2	-
siRNA-t0093436	ACTTGCCGCGCGTCTCACTG	20	7	FgHV1gp2	-
siRNA-t0090138	AGAGGCCTCGTCACTTACATT	21	7	FGSG_12610	-
siRNA-t0091037	AGATCCACCCGGATGACTTGGCT	23	7	FgHV1gp2	-
siRNA-t0092332	AGATCCACCCGGATGACTTGGCTC	24	7	FgHV1gp2	-
siRNA-t0089029	AGATCCCACTCTAGCAATGCAT	22	7	FgHV1gp1	-
siRNA-t0086970	AGATCTCAGAATGGTCACCGT	21	7	FGSG_05023	K00006｜1｜0.0｜869｜fgr:FG05023.1｜glycerol-3-phosphate dehydrogenase (NAD+) [EC:1.1.1.8]
siRNA-t0090005	AGATCTTGCTGTAGTGTCTGA	21	7	FgHV1gp2	-
siRNA-t0089044	AGATGTAGGCTCCTCCGACAA	21	7	FGSG_11268	-

siRNA-t0087290	AGCAATGCAACAGACTTCTTCTCT	24	7	FgHV1gp2	-
siRNA-t0093142	AGCGTGTTGTACGGCGTGGTCTC	23	7	FgHV1gp2	-
siRNA-t0088983	AGCTGCTACTCGAACCGCCCTT	22	7	FgHV1gp2	-
siRNA-t0088214	AGCTTACCTAGACCGAAGAA	20	7	FgHV1gp2	-
siRNA-t0086464	AGCTTACCTAGACCGAAGAAA	21	7	FgHV1gp2	-
siRNA-t0090794	AGGACAAGCCGTCAAACATAGAC	23	7	FgHV1gp2	-
siRNA-t0091163	AGGACCCGAGAAAAACGCCCCTGG	24	7	FgHV1gp2	-
siRNA-t0093631	AGGCCCTCAGTAGTCGACATG	21	7	FGSG_09197	K00021|1|0.0|2147|fgr:FG09197.1|hydroxymethylglutaryl-CoA reductase (NADPH) [EC:1.1.1.34]
siRNA-t0087725	AGGCCTCGTCACTTACATTGG	21	7	FGSG_12610	-
siRNA-t0092681	AGGCGTGTTTTTCAATGATGG	21	7	FGSG_11031	-
siRNA-t0085019	AGGTACGTCTATATTGTCTCT	21	7	FgHV1gp2	-
siRNA-t0091072	AGTGTGTCGCGTCTACCTTGT	21	7	FgHV1gp2	-
siRNA-t0087172	ATACTTGATCTCAGCACGCCGG	22	7	FgHV1gp2	-
siRNA-t0092195	ATAGAATGTCGAGATACTCTT	21	7	FGSG_01790	K00680|1|0.0|2387|afm:AFUA_3G14700|[EC:2.3.1.-];K15419|2|0.0|1077|ani:AN2547.2|emericellamide biosynthesis, highly reducing iterative type I polyketide synthase;K15384|5|0.0|762|afv:AFLA_066840|aspyridone synthetase, hybrid polyketide synthase / nonribosomal peptide synthetase
siRNA-t0092552	ATGCAACAGACTTCTTCTCT	20	7	FGSG_06563	K00521|1|4e-62|238|cpw:CPC735_011850|ferric-chelate reductase [EC:1.16.1.7];K05920|2|7e-22|105|afm:AFUA_6G02170|[EC:1.16.1.-]
siRNA-t0093692	ATGCAGCGGCAAATACAGGAC	21	7	FgHV1gp2	-
siRNA-t0084715	ATGGCTGATGCGACCGCTTT	20	7	FGSG_06342	K01027|1|0.0|1027|fgr:FG06342.1|3-oxoacid CoA-transferase [EC:2.8.3.5]
siRNA-t0092199	ATTAACCATGGATCTGATTAT	21	7	FgHV1gp1	-
siRNA-t0085569	ATTCGTTACAATCGTGGACTT	21	7	FgHV1gp2	-
siRNA-t0092165	ATTGAACCACAGTCGAATGA	20	7	FGSG_06486	K05546|1|3e-37|157|ncr:NCU04203|alpha 1,3-glucosidase [EC:3.2.1.84]
siRNA-t0091581	ATTTGCGACGTGCTTGAAGGCGA	23	7	FgHV1gp2	-
siRNA-t0084384	CAAACATAGACACTTCAACATG	22	7	FgHV1gp2	-
siRNA-t0087906	CAAACATAGACACTTCAACATGGG	24	7	FgHV1gp2	-
siRNA-t0088968	CAAGAACATCAGGCATGTATG	21	7	FGSG_07245	K12830|1|0.0|2446|fgr:FG07245.1|splicing factor 3B subunit 3
siRNA-t0089708	CAAGATGGACTGGCCTGTTGTC	22	7	FgHV1gp2	-
siRNA-t0085749	CAATGATGGCACGTCGACAGGC	22	7	FgHV1gp2	-
siRNA-t0092175	CACGACCACGCAAGGATGGCGA	22	7	FGSG_01196	K01537|1|0.0|1397|ncr:NCU05154|Ca2+-transporting ATPase [EC:3.6.3.8]
siRNA-t0091260	CAGAATTCTAGCCTGGTTGAGA	22	7	FgHV1gp2	-
siRNA-t0085576	CAGACACTTGTTTCTCGTGGGA	22	7	FgHV1gp2	-
siRNA-t0090454	CAGCACGCCGGTTACCAAGGT	21	7	FgHV1gp2	-
siRNA-t0093435	CAGGACCCGAGAAAAACGCCCCTG	24	7	FgHV1gp2	-
siRNA-t0088316	CATACAATCCCAGGGCCTGA	20	7	FGSG_04433	K07393|1|0.0|711|fgr:FG04433.1|putative glutathione S-transferase
siRNA-t0088292	CATACCTTTGAGCTCCTTGA	20	7	FGSG_09364	K13953|1|5e-39|160|cal:CaO19.12579|alcohol dehydrogenase, propanol-preferring [EC:1.1.1.1]
siRNA-t0088480	CCACGAAACGTGGGGTGTGTAT	22	7	FgHV1gp2	-
siRNA-t0084740	CCAGAACCCAGCACCTTTCC	20	7	FgHV1gp2	-
siRNA-t0088727	CCAGTATGGTTCGACACTGGA	21	7	FGSG_05213	-
siRNA-t0086583	CCAGTGTCGAACCATACTGGACCCA	25	7	FgHV1gp2	-
siRNA-t0085127	CCATGACGTACGACCCCGAAAGT	23	7	FgHV1gp1	-
siRNA-t0085308	CCCATACATGCCTGATGTTCTTGT	24	7	FgHV1gp2	-
siRNA-t0087904	CCCCAGCAGACTTGGAAGTT	20	7	FGSG_07934	-
siRNA-t0093521	CCCTTCGTGAACTGCTGCGCTAT	23	7	FgHV1gp2	-
siRNA-t0085002	CCGAAGGAGACATTTGAGGGA	21	7	FGSG_09910	-
siRNA-t0092186	CCGACAATGGCGCATACACGGAA	23	7	FgHV1gp2	-
siRNA-t0084931	CCGAGAAAAACGCCCCTGGAC	21	7	FgHV1gp2	-
siRNA-t0091488	CCGGCTATAGGAGTACCAAC	20	7	FGSG_01541	K02906|1|2e-37|155|cnb:CNBE2710|large subunit ribosomal protein L3
siRNA-t0084572	CGAAGGAACTCCACATAGCGTCGT	24	7	FgHV1gp2	-
siRNA-t0087963	CGAATTGCAACGAGGGCCCAG	21	7	FGSG_07277	K01976|1|3e-32|139|sce:YBR222C|[EC:6.-.-.-];K01895|4|2e-19|96.7|mgr:MGG_04590|acetyl-CoA synthetase [EC:6.2.1.1];K01897|5|2e-19|96.7|ncr:NCU06063|long-chain acyl-CoA synthetase [EC:6.2.1.3]
siRNA-t0087565	CGACAATGGCGCATACACGGAA	22	7	FgHV1gp2	-
siRNA-t0089290	CGAGAAAAACGCCCCTGGAC	20	7	FGSG_01234	K01768|1|0.0|3295|fgr:FG01234.1|adenylate cyclase [EC:4.6.1.1]
siRNA-t0086765	CGCGCGCTGTACTGAAGGTG	20	7	FGSG_10470	K09238|1|1e-08|61.6|clu:CLUG_01494|metallothionein expression activator;K09202|2|2e-08|60.8|yli:YALI0E16973g|regulatory protein SWI5
siRNA-t0087924	CGCTGTTGGTACTCCTATAGC	21	7	FGSG_01424	K15560|1|0.0|1154|fgr:FG01424.1|protein NRD1
siRNA-t0086315	CGGAACCCATTGATCATTCTCCTCA	25	7	FgHV1gp2	-
siRNA-t0088062	CGGATACGTTCTGAGAAGTCT	21	7	FgHV1gp2	-
siRNA-t0092907	CGGATACGTTCTGAGAAGTCTC	22	7	FgHV1gp2	-
siRNA-t0090307	CGGCATGAACTCGGTCAACGAAAAC	25	7	FgHV1gp2	-
siRNA-t0089826	CGGCGTGCTGAGATCAAGTATA	22	7	FGSG_10790	K15389|1|3e-101|368|ure:UREG_05875|acyl-CoA dehydrogenase;K00257|2|1e-100|366|afm:AFUA_6G10880|[EC:1.3.99.-]
siRNA-t0086225	CGGGTCCAGATGTTGTGCTTTT	22	7	FgHV1gp2	-
siRNA-t0086847	CGGTGTCGCCATTGATGGGAT	21	7	FGSG_11815	K10866|1|0.0|1009|fgr:FG00711.1|DNA repair protein RAD50 [EC:3.6.-.-];K13095|3|2e-94|346|fgr:FG02080.1|splicing factor 1;K01952|4|2e-33|143|pno:SNOG_10851|phosphoribosylformylglycinamidine synthase [EC:6.3.5.3];K08197|5|3e-10|66.2|cim:CIMG_07855|MFS transporter, SIT family, siderophore-iron:H+ symporter
siRNA-t0092429	CGTAAGCTGCTACTCGAACCGCCCT	25	7	FgHV1gp2	-
siRNA-t0086218	CGTCATGATCATCAGCACCG	20	7	FGSG_11998	-
siRNA-t0089504	CGTCTTCGATGGATGCTGCG	20	7	FGSG_09845	K13076|1|0.0|1144|fgr:FG09845.1|delta8-fatty-acid desaturase [EC:1.14.19.4]
siRNA-t0085380	CGTGACGAAGATCCCACTCT	20	7	FGSG_13783	K01932|1|0.0|1526|afm:AFUA_5G12730|[EC:6.3.2.-]
siRNA-t0091289	CGTGTAGAAAGTCTTTTCAGC	21	7	FGSG_12799	-
siRNA-t0090161	CGTTGAAAATCAGAGAGCAGGA	22	7	FgHV1gp2	-
siRNA-t0087456	CTACCAGAACCCAGCACCTTTC	22	7	FgHV1gp2	-
siRNA-t0092461	CTAGACCGAAGAAAGACCACATC	23	7	FgHV1gp2	-
siRNA-t0093213	CTGACGAATTTGGACAAGTACTC	23	7	FgHV1gp2	-
siRNA-t0090137	CTGCAGCAACTGTCGCCTGGA	21	7	FgHV1gp1	-
siRNA-t0089991	CTGCATGTACTGTTCGGCAA	20	7	FGSG_08421	K13126|1|0.0|636|fgr:FG08421.1|polyadenylate-binding protein
siRNA-t0092734	CTGGAATTCCTTGGGAGGCCT	21	7	FgHV1gp2	-
siRNA-t0092833	CTGTTCTCTGTCGGCATGTTGCC	23	7	FGSG_11657	K09184|1|5e-13|75.9|bfu:BC1G_09852|GATA-binding protein, other eukaryote
siRNA-t0088825	CTTCAGGCCCTGGGATTGTA	20	7	FGSG_01442	K06867|1|1e-28|129|kla:KLLA0E17953g|
siRNA-t0091435	CTTGAAGTTGGTGCCGACGTCTA	23	7	FgHV1gp2	-
siRNA-t0089360	CTTGGAAAGTTGGAGCCCTTC	21	7	FgHV1gp2	-
siRNA-t0086613	CTTTCGGGGTCGTACGTCATGGC	23	7	FGSG_02394	K01932|1|0.0|942|afm:AFUA_1G10380|[EC:6.3.2.-]
siRNA-t0090617	GAAAAAGCACAACATCTGGA	20	7	FGSG_13783	K01932|1|0.0|1526|afm:AFUA_5G12730|[EC:6.3.2.-]
siRNA-t0089349	GAAACCATCAGGACCCGAGAAA	22	7	FgHV1gp2	-
siRNA-t0092000	GAAATATCGGAAAATTGTGTTGA	23	7	FgHV1gp2	-
siRNA-t0085867	GAACCACAGTCGAATGAAATGGAAC	25	7	FgHV1gp2	-
siRNA-t0086647	GAACCTGATCCCGGAATTATG	21	7	FGSG_08568	K11236|1|1e-09|65.5|uma:UM02422.1|cell division control protein 24
siRNA-t0092638	GAACTGCTGCGCTATGGTGTGA	22	7	FgHV1gp2	-
siRNA-t0086461	GAAGACGCAAGCCCGCTTACTCA	23	7	FgHV1gp2	-
siRNA-t0092635	GAAGGATATGCGATCGAGTTG	21	7	FGSG_01364	-
siRNA-t0093212	GAAGTTGGTGCCGACGTCTACGTA	24	7	FgHV1gp2	-
siRNA-t0087726	GAATGGTCTGCCTAATCTTTCCTC	24	7	FgHV1gp2	-
siRNA-t0085692	GAATTGCAACGAGGGCCCAGTCA	23	7	FgHV1gp2	-
siRNA-t0088822	GACAGTTCCGCGTGTGATTGGT	22	7	FgHV1gp2	-
siRNA-t0093313	GACGGGTGTGAAGCTTGGAT	20	7	FGSG_09780	K15436|1|0.0|1914|fgr:FG09780.1|transportin-3

siRNA-t0090939	GACGTTCCAGAACAAGAGACT	21	7	FGSG_00625	K15385|1|1e-33|144|ure:UREG_06498|aspyridone synthetase trans-acting enoyl reductase;K09241|2|7e-06|52.4|pcs:Pc20g03000|transcriptional regulatory protein GAL4
siRNA-t0092016	GATACGTTCTGAGAAGTCTCCGA	23	7	FgHV1gp2	-
siRNA-t0087651	GATCTCAGCACGCCGGTTACCAAGG	25	7	FgHV1gp2	-
siRNA-t0088790	GATCTTCGTCACGCTTGCATT	21	7	FGSG_06837	-
siRNA-t0084593	GATGTTTGCTTGGTTTCGGGA	21	7	FGSG_13866	K08139|1|2e-62|239|dha:DEHA2C11374g|MFS transporter, SP family, sugar:H+ symporter
siRNA-t0091234	GATTAATCAATGTACCGTTCGGCAA	25	7	FgHV1gp2	-
siRNA-t0087210	GCAACGAGGGCCCAGTCATGAA	22	7	FgHV1gp2	-
siRNA-t0091817	GCAACGAGGGCCCAGTCATGAATT	24	7	FgHV1gp2	-
siRNA-t0087367	GCAAGAAGTTTGCATCCTCGA	21	7	FGSG_09793	-
siRNA-t0087057	GCAAGATGGACTGGCCTGTT	20	7	FGSG_07818	-
siRNA-t0085704	GCACCAACTTCAAGAACCCAC	21	7	FGSG_04694	K15419|1|0.0|1075|ani:AN2547.2|emericellamide biosynthesis, highly reducing iterative type I polyketide synthase;K00680|3|0.0|812|afm:AFUA_3G14700|[EC:2.3.1.-]
siRNA-t0091065	GCAGCAGAATTCTAGCCTGGTTGAG	25	7	FgHV1gp2	-
siRNA-t0092883	GCAGTGTTCATGTGCACATT	20	7	FGSG_05238	K13336|1|2e-53|209|afv:AFLA_048740|peroxin-3;K11369|2|6e-48|191|ani:AN3929.2|retrograde regulation protein 2;K15361|3|1e-43|176|cim:CIMG_09192|WD repeat-containing protein 48;K03448|4|1e-31|137|act:ACLA_078170|MFS transporter, ACS family, pantothenate transporter;K08192|5|1e-31|136|spo:SPCC417.10|MFS transporter, ACS family, allantoate permease
siRNA-t0091174	GCATCCGCCTATGCTTGTCAGCCTT	25	7	FgHV1gp2	-
siRNA-t0087164	GCCACACCCTTAGGACCAGTT	21	7	FGSG_08147	K00777|1|9e-20|98.6|afm:AFUA_2G00910|[EC:2.4.2.-]
siRNA-t0087781	GCCTGGGAGACAGAGCCTGAGT	22	7	FgHV1gp2	-
siRNA-t0090708	GCGCACATATCTCCTTGAACT	21	7	FgHV1gp2	-
siRNA-t0086143	GCGGATAACCTATACTTGATCTC	23	7	FgHV1gp2	-
siRNA-t0086144	GCGGGCTTGCGTCTTCATTGT	21	7	FGSG_02391	-
siRNA-t0086595	GCTCTGTGACTCCAGCCAAAGAGA	24	7	FgHV1gp2	-
siRNA-t0086019	GCTTCTGGATCAATGTCAGCA	21	7	FGSG_04743	K12828|1|3e-13|77.0|ssl:SS1G_03093|splicing factor 3B subunit 1
siRNA-t0084305	GGAAGATTTGCCTGTGCCGGTG	22	7	FGSG_11341	K14458|1|2e-64|246|afv:AFLA_097500|2-acylglycerol O-acyltransferase 1 [EC:2.3.1.22]
siRNA-t0092028	GGATGTGGGTGCGCCGAATGAA	22	7	FgHV1gp2	-
siRNA-t0090421	GGCCTCTGACGTTGATCTCTT	21	7	FgHV1gp2	-
siRNA-t0091302	GGCGACACCGACGGTACGTG	20	7	FGSG_06461	K11369|1|1e-36|153|ani:AN3929.2|retrograde regulation protein 2;K13095|2|7e-35|147|fgr:FG02080.1|splicing factor 1;K13336|3|2e-34|146|afv:AFLA_048740|peroxin-3;K03448|4|5e-32|138|ncr:NCU07820|MFS transporter, ACS family, pantothenate transporter
siRNA-t0090235	GGCTCCTCCGACAATGGCGCAT	22	7	FGSG_10215	K15289|1|2e-09|62.8|yli:YALI0E22803g|solute carrier family 35, member F5;K15303|2|4e-09|62.0|ani:AN0675.2|aflatoxin B1 aldehyde reductase
siRNA-t0091772	GGCTCGTGACTTTTCCCTCA	20	7	FGSG_00581	K01598|1|2e-13|77.0|uma:UM05916.1|phosphopantothenoylcysteine decarboxylase [EC:4.1.1.36]
siRNA-t0086344	GGGATGCAAAACCGGTGCGA	20	7	FgHV1gp2	-
siRNA-t0090475	GTAGAACAGACGTCTCTCACG	21	7	FgHV1gp2	-
siRNA-t0090180	GTCTTCTGACGAATTTGGACAAG	23	7	FgHV1gp2	-
siRNA-t0091290	GTGACGAGGCCTCTGACGTTGAT	23	7	FgHV1gp2	-
siRNA-t0088258	GTGACTTTTCCCTCACAGGCT	21	7	FgHV1gp2	-
siRNA-t0088124	GTGATAAATGAACGTCTGACTGTAG	25	7	FgHV1gp2	-
siRNA-t0087974	GTGGGTTCTTGAAGTTGGTGCCG	23	7	FgHV1gp2	-
siRNA-t0087089	GTGTGGGTCTTCTGACGAATTTGGA	25	7	FgHV1gp2	-
siRNA-t0093169	GTTATGATCTGGTTCACGCT	20	7	FGSG_11696	K11701|1|3e-52|207|tml:GSTUM_00000065001|helicase required for RNAi-mediated heterochromatin assembly 1 [EC:3.6.4.13]
siRNA-t0090805	GTTCAAGTATCGTCACTTCTTC	22	7	FGSG_05959	-
siRNA-t0085950	GTTCAAGTATCGTCACTTCTTCGT	24	7	FgHV1gp2	-
siRNA-t0085708	GTTGAATCGATCGTCATGGTGAT	23	7	FgHV1gp2	-
siRNA-t0085678	TAAGTCTGGAATTCCTTGGGAG	22	7	FgHV1gp2	-
siRNA-t0088031	TACCAGAACCCAGCACCTTTCCC	23	7	FgHV1gp2	-
siRNA-t0087957	TACCAGAACCCAGCACCTTTCCCT	24	7	FgHV1gp2	-
siRNA-t0091641	TACCTGTTGTTGGTCTTCCAGGC	23	7	FgHV1gp2	-
siRNA-t0089111	TACGTAAGGCGTCGACACGATGAT	24	7	FgHV1gp2	-
siRNA-t0084328	TACTTGATCTCAGCACGCCGGTTA	24	7	FgHV1gp2	-
siRNA-t0090329	TAGAACAGACGTCTCTCACGC	21	7	FgHV1gp2	-
siRNA-t0093539	TAGAACAGACGTCTCTCACGCTC	23	7	FgHV1gp2	-
siRNA-t0086896	TAGAAGCTTGACACGAATGAT	21	7	FgHV1gp2	-
siRNA-t0088290	TAGACCGAAGAAAGACCACATCCAG	25	7	FgHV1gp2	-
siRNA-t0091999	TAGCAGCTTACGAGATCCTG	20	7	FGSG_02743	K09466|1|8e-22|105|ppa:PAS_chr4_0487|zinc finger protein ADR1
siRNA-t0091412	TATCCGTGATAGCCTCTCCCT	21	7	FGSG_12196	-
siRNA-t0090266	TCAACGGTTCTTGGAAAGTTGGAG	24	7	FgHV1gp2	-
siRNA-t0090712	TCAAGCAACAGTCAGACACG	20	7	FGSG_08389	-
siRNA-t0092212	TCATGGGCAAAAGACAGATAGGT	23	7	FgHV1gp2	-
siRNA-t0089146	TCCCACTCTAGCAATGCATCACC	23	7	FgHV1gp1	-
siRNA-t0087672	TCCGAAGGAACTCCACATAGCGTC	24	7	FgHV1gp2	-
siRNA-t0092445	TCGTGTAGAAAGTCTTTTCAGC	22	7	FgHV1gp2	-
siRNA-t0088767	TCTGTCTATGAGCACTTGTTT	21	7	FgHV1gp2	-
siRNA-t0086728	TCTTCAGCGTAACGTGACCAGCT	23	7	FgHV1gp2	-
siRNA-t0091019	TCTTCGGATGACCGTACCAT	20	7	FGSG_10523	K01932|1|4e-178|626|afm:AFUA_6G12080|[EC:6.3.2.-]
siRNA-t0084769	TCTTGAAGTTGGTGCCGACGTCTA	24	7	FgHV1gp2	-
siRNA-t0086534	TGAAACCATCAGGACCCGAGAA	22	7	FgHV1gp2	-
siRNA-t0090242	TGATCTGGTTCACGCTGCAAC	21	7	FGSG_11536	K00517|1|4e-44|178|afm:AFUA_6G13945|[EC:1.14.-.-];K05917|4|5e-17|88.6|lel:LELG_03738|cytochrome P450, family 51 (sterol 14-demethylase) [EC:1.14.13.70]
siRNA-t0086960	TGGATCAATGTCAGCAGTGTTC	22	7	FgHV1gp2	-
siRNA-t0087408	TGGATGTGGGTGCGCCGAATGAA	23	7	FgHV1gp2	-
siRNA-t0087002	TGGCCGGCAGTCCTAGCAAGTGA	23	7	FgHV1gp2	-
siRNA-t0091245	TGGTTCAAGTATCGTCACTT	20	7	FgHV1gp2	-
siRNA-t0092989	TGGTTCGACACTGGAAGAGA	20	7	FGSG_05503	K03785|1|1e-17|91.7|ani:AN4350.2|3-dehydroquinate dehydratase I [EC:4.2.1.10];K00472|2|1e-14|81.6|nhe:NECHADRAFT_71135|prolyl 4-hydroxylase [EC:1.14.11.2];K09241|3|3e-08|60.5|sce:YPL248C|transcriptional regulatory protein GAL4;K09247|4|1e-06|55.5|sce:YLR256W|heme activator protein 1;K09248|5|2e-06|54.3|kla:KLLA0E18129g|proline utilization trans-activator
siRNA-t0087488	TGGTTTGTTCTGAAATTTGATGTT	24	7	FgHV1gp2	-
siRNA-t0090365	TGTACTTGCCGCGCGTCTCACTGA	24	7	FgHV1gp2	-
siRNA-t0084382	TGTCGTAGGAAAGGAAAAGTT	21	7	FGSG_01049	-
siRNA-t0085456	TGTGTCGCGTCTACCTTGTTCA	22	7	FgHV1gp2	-
siRNA-t0091403	TGTTCGCATCCGTTCTGTGA	20	7	FGSG_08999	-
siRNA-t0087286	TGTTCTGGAACGTCAAATCAAGTT	24	7	FgHV1gp2	-
siRNA-t0088823	TGTTGACGTCACCCTTTGGAG	21	7	FGSG_13865	K00128|1|1e-104|379|ncr:NCU03415|aldehyde dehydrogenase (NAD+) [EC:1.2.1.3]
siRNA-t0086674	TTCAAGATACAGCAACTGGGTT	22	7	FGSG_09526	-
siRNA-t0087586	TTCCGAAGGAACTCCACATAGCGTC	25	7	FgHV1gp2	-
siRNA-t0086131	TTCGCTCCCACTATCGTCCTGC	22	7	FGSG_01294	K00799|1|1e-34|146|afv:AFLA_022030|glutathione S-transferase [EC:2.5.1.18]
siRNA-t0090874	TTCGGTATTGTCGAGTCGAA	20	7	FGSG_01049	-
siRNA-t0087721	TTCTACCAGAACCCAGCACC	20	7	FGSG_12463	-
siRNA-t0092812	TTCTACCAGAACCCAGCACCT	21	7	FGSG_12463	-

<table>
<tr><td>siRNA-t0089444</td><td>TTCTTCTGCAGTAAAACGTT</td><td>20</td><td>7</td><td>FGSG_00550</td><td>-</td></tr>
<tr><td>siRNA-t0091132</td><td>TTGAGAACGCTGAAATCGAGG</td><td>21</td><td>7</td><td>FgHV1gp2</td><td>-</td></tr>
<tr><td>siRNA-t0084530</td><td>TTGCGACGTGCTTGAAGGCGAAGT</td><td>24</td><td>7</td><td>FgHV1gp2</td><td>-</td></tr>
<tr><td>siRNA-t0102042</td><td>AAACGCTGCCGGGATGTAATC</td><td>21</td><td>6</td><td>FgHV1gp2</td><td>-</td></tr>
<tr><td>siRNA-t0101252</td><td>AAAGACAGATAGGTCGCAGGCG</td><td>22</td><td>6</td><td>FgHV1gp2</td><td>-</td></tr>
<tr><td>siRNA-t0102774</td><td>AACATATCCGGCTCCCTTTC</td><td>20</td><td>6</td><td>FGSG_06595</td><td>-</td></tr>
<tr><td>siRNA-t0103668</td><td>AAGGCGCATCGGCTTACCCTC</td><td>21</td><td>6</td><td>FGSG_05873</td><td>-</td></tr>
<tr><td>siRNA-t0103772</td><td>AAGTATCGTCACTTCTTCGTAGT</td><td>23</td><td>6</td><td>FgHV1gp2</td><td>-</td></tr>
<tr><td>siRNA-t0105272</td><td>AAGTCCACGATTGTAACGAAT</td><td>21</td><td>6</td><td>FGSG_05306</td><td>K08333|1|0.0|3035|fgr:FG05306.1|phosphoinositide-3-kinase, regulatory subunit 4, p150 [EC:2.7.11.1]</td></tr>
<tr><td>siRNA-t0097521</td><td>AAGTTACAGGCCTCATTGATTT</td><td>22</td><td>6</td><td>FgHV1gp2</td><td>-</td></tr>
<tr><td>siRNA-t0097868</td><td>AATCGGGGCGGTGACACTGGAGG</td><td>23</td><td>6</td><td>FGSG_11044</td><td>K05393|1|1e-45|182|uma:UM03490.1|chloride channel, other eukaryote;K06867|2|2e-11|69.3|sce:YDL019C|</td></tr>
<tr><td>siRNA-t0104459</td><td>AATCGTGGACTTCCTTTAGGA</td><td>21</td><td>6</td><td>FgHV1gp2</td><td>-</td></tr>
<tr><td>siRNA-t0098851</td><td>AATGATGGCACGTCGACAGGC</td><td>21</td><td>6</td><td>FgHV1gp2</td><td>-</td></tr>
<tr><td>siRNA-t0098837</td><td>AATGGAAGAGGCCAGTTGGGGT</td><td>22</td><td>6</td><td>FGSG_00259</td><td>-</td></tr>
<tr><td>siRNA-t0099452</td><td>AATGGCGACACCGACGGTACG</td><td>21</td><td>6</td><td>FGSG_05709</td><td>K13830|1|2e-68|259|lbc:LACBIDRAFT_233717|pentafunctional AROM polypeptide [EC:4.2.3.4 4.2.1.10 1.1.1.25 2.7.1.71 2.5.1.19]</td></tr>
<tr><td>siRNA-t0101721</td><td>ACACCACATCACGCTCGGGGT</td><td>21</td><td>6</td><td>FgHV1gp2</td><td>-</td></tr>
<tr><td>siRNA-t0096287</td><td>ACAGCTCCAATAATCGTTGAA</td><td>21</td><td>6</td><td>FgHV1gp2</td><td>-</td></tr>
<tr><td>siRNA-t0095214</td><td>ACCGAGGCAGAGCATTCAGCA</td><td>21</td><td>6</td><td>FGSG_04842</td><td>-</td></tr>
<tr><td>siRNA-t0098180</td><td>ACCTTGTTCAGGAATCACGACTT</td><td>23</td><td>6</td><td>FgHV1gp2</td><td>-</td></tr>
<tr><td>siRNA-t0098556</td><td>ACGAGGCCTCTGACGTTGATCTC</td><td>23</td><td>6</td><td>FgHV1gp2</td><td>-</td></tr>
<tr><td>siRNA-t0096871</td><td>ACGAGTGTGTCGCGTCTACCTT</td><td>22</td><td>6</td><td>FgHV1gp2</td><td>-</td></tr>
<tr><td>siRNA-t0100061</td><td>ACGTCGAAAGCATGGGTCGCA</td><td>21</td><td>6</td><td>FGSG_10548</td><td>K15418|1|0.0|1191|pcs:Pc21g12440|zearalenone synthase, highly reducing iterative type I polyketide synthase;K15419|4|0.0|740|ani:AN2547.2|emericellamide biosynthesis, highly reducing iterative type I polyketide synthase</td></tr>
<tr><td>siRNA-t0095340</td><td>AGACACTTGTTTCTCGTGGGA</td><td>21</td><td>6</td><td>FGSG_07509</td><td>K08157|1|4e-46|185|kla:KLLA0F18106g|MFS transporter, DHA1 family, multidrug resistance protein</td></tr>
<tr><td>siRNA-t0103808</td><td>AGACAGATAGGTCGCAGGCGC</td><td>21</td><td>6</td><td>FgHV1gp2</td><td>-</td></tr>
<tr><td>siRNA-t0096024</td><td>AGACTAAACGCTTTGGGCACAT</td><td>22</td><td>6</td><td>FgHV1gp1</td><td>-</td></tr>
<tr><td>siRNA-t0098729</td><td>AGAGTATCTCGACATTCTAT</td><td>20</td><td>6</td><td>FGSG_05303</td><td>K05393|1|0.0|1444|fgr:FG05303.1|chloride channel, other eukaryote</td></tr>
<tr><td>siRNA-t0099337</td><td>AGATTTGCCTGTGCCGGTGTCGGCT</td><td>25</td><td>6</td><td>FgHV1gp2</td><td>-</td></tr>
<tr><td>siRNA-t0094226</td><td>AGCATGAAGGCACAATATCGCT</td><td>22</td><td>6</td><td>FGSG_01216</td><td>K14015|1|0.0|1330|fgr:FG01216.1|nuclear protein localization protein 4 homolog</td></tr>
<tr><td>siRNA-t0105990</td><td>AGCGATCTTCCAAGCGGTCA</td><td>20</td><td>6</td><td>FGSG_06300</td><td>K15109|1|1e-177|620|fgr:FG06300.1|solute carrier family 25 (mitochondrial carnitine/acylcarnitine transporter), member 20/29</td></tr>
<tr><td>siRNA-t0101861</td><td>AGCTGCTACTCGAACCGCCC</td><td>20</td><td>6</td><td>FgHV1gp2</td><td>-</td></tr>
<tr><td>siRNA-t0097238</td><td>AGGCTAATTCTTCTGCGGAG</td><td>20</td><td>6</td><td>FGSG_01338</td><td>K06694|1|5e-128|455|fgr:FG01338.1|26S proteasome non-ATPase regulatory subunit 10</td></tr>
<tr><td>siRNA-t0105730</td><td>AGTCATCGGGAATCATGTCGGT</td><td>22</td><td>6</td><td>FgHV1gp2</td><td>-</td></tr>
<tr><td>siRNA-t0100876</td><td>AGTGATGGTACCATTGCTGCAT</td><td>22</td><td>6</td><td>FGSG_05452</td><td>-</td></tr>
<tr><td>siRNA-t0105096</td><td>AGTGCCATGACGTACGACCCC</td><td>21</td><td>6</td><td>FgHV1gp1</td><td>-</td></tr>
<tr><td>siRNA-t0098466</td><td>AGTGCCATGACGTACGACCCCG</td><td>22</td><td>6</td><td>FgHV1gp1</td><td>-</td></tr>
<tr><td>siRNA-t0096762</td><td>AGTGTCGAACCATACTGGACCC</td><td>22</td><td>6</td><td>FgHV1gp2</td><td>-</td></tr>
<tr><td>siRNA-t0103916</td><td>ATAACACGCAGCAGAATTCTAGC</td><td>23</td><td>6</td><td>FgHV1gp2</td><td>-</td></tr>
<tr><td>siRNA-t0094656</td><td>ATACACGACGTACTTAGGGTT</td><td>21</td><td>6</td><td>FgHV1gp2</td><td>-</td></tr>
<tr><td>siRNA-t0101840</td><td>ATACAGGACCGCGCGATGGCGCGCG</td><td>25</td><td>6</td><td>FgHV1gp2</td><td>-</td></tr>
<tr><td>siRNA-t0099076</td><td>ATAGCGTCGTCACCTTGATTG</td><td>21</td><td>6</td><td>FGSG_10511</td><td>K11236|1|0.0|1846|fgr:FG10511.1|cell division control protein 24</td></tr>
<tr><td>siRNA-t0104214</td><td>ATGATCATCAGCACCGTACGGAT</td><td>23</td><td>6</td><td>FgHV1gp2</td><td>-</td></tr>
<tr><td>siRNA-t0098610</td><td>ATGGATCTGATTATCTCCATG</td><td>21</td><td>6</td><td>FgHV1gp1</td><td>-</td></tr>
<tr><td>siRNA-t0099438</td><td>ATGGGCAAAAGACAGATAGGTCGC</td><td>24</td><td>6</td><td>FgHV1gp2</td><td>-</td></tr>
<tr><td>siRNA-t0105820</td><td>ATTCCGGACGGTGACCATTC</td><td>20</td><td>6</td><td>FGSG_02632</td><td>K05349|1|0.0|1666|fgr:FG02632.1|beta-glucosidase [EC:3.2.1.21]</td></tr>
<tr><td>siRNA-t0095908</td><td>ATTGTTATAAGGCTTGTCGTCGGGA</td><td>25</td><td>6</td><td>FgHV1gp2</td><td>-</td></tr>
<tr><td>siRNA-t0099578</td><td>ATTTATTCGTCACCTTTACC</td><td>20</td><td>6</td><td>FGSG_01362</td><td>-</td></tr>
<tr><td>siRNA-t0098627</td><td>CAAAAGACCAATCCCTGGATTAG</td><td>23</td><td>6</td><td>FgHV1gp2</td><td>-</td></tr>
<tr><td>siRNA-t0095854</td><td>CAACTCGATCGCATATCCTT</td><td>20</td><td>6</td><td>FgHV1gp2</td><td>-</td></tr>
<tr><td>siRNA-t0100697</td><td>CAAGCGTGACGAAGATCCCAC</td><td>21</td><td>6</td><td>FGSG_07746</td><td>-</td></tr>
<tr><td>siRNA-t0103434</td><td>CAAGCGTGACGAAGATCCCACT</td><td>22</td><td>6</td><td>FgHV1gp1</td><td>-</td></tr>
<tr><td>siRNA-t0104496</td><td>CAATTGCACATGGGACCATCA</td><td>21</td><td>6</td><td>FgHV1gp2</td><td>-</td></tr>
<tr><td>siRNA-t0101555</td><td>CAATTGCACATGGGACCATCAT</td><td>22</td><td>6</td><td>FgHV1gp2</td><td>-</td></tr>
<tr><td>siRNA-t0103057</td><td>CAGCGTAACGTGACCAGCTGTT</td><td>22</td><td>6</td><td>FgHV1gp2</td><td>-</td></tr>
<tr><td>siRNA-t0099148</td><td>CAGCTGCAGCAACTGTCGCC</td><td>20</td><td>6</td><td>FGSG_12712</td><td>K10884|1|0.0|1132|fgr:FG05317.1|ATP-dependent DNA helicase 2 subunit 1</td></tr>
<tr><td>siRNA-t0095526</td><td>CAGGCAAACAAGTGCTCATA</td><td>20</td><td>6</td><td>FGSG_04033</td><td>-</td></tr>
<tr><td>siRNA-t0102201</td><td>CATAACACGCAGCAGAATTCTAG</td><td>23</td><td>6</td><td>FgHV1gp2</td><td>-</td></tr>
<tr><td>siRNA-t0104021</td><td>CATAGCGTCGTCACCTTGATT</td><td>21</td><td>6</td><td>FGSG_13835</td><td>-</td></tr>
<tr><td>siRNA-t0099249</td><td>CATCAGGACCCGAGAAAAACGCC</td><td>23</td><td>6</td><td>FgHV1gp2</td><td>-</td></tr>
<tr><td>siRNA-t0098098</td><td>CATTCAGTAGTCGGGGTCTCC</td><td>21</td><td>6</td><td>FgHV1gp1</td><td>-</td></tr>
<tr><td>siRNA-t0101610</td><td>CATTCCGGACGGTGACCATT</td><td>20</td><td>6</td><td>FGSG_02632</td><td>K05349|1|0.0|1666|fgr:FG02632.1|beta-glucosidase [EC:3.2.1.21]</td></tr>
<tr><td>siRNA-t0096147</td><td>CCAAAACGTCGGTCCCGTTC</td><td>20</td><td>6</td><td>FGSG_06582</td><td>K10661|1|0.0|2855|fgr:FG06582.1|E3 ubiquitin-protein ligase MARCH6 [EC:6.3.2.19]</td></tr>
<tr><td>siRNA-t0102424</td><td>CCAAACTGTTCATTTCGGCGAGTC</td><td>24</td><td>6</td><td>FgHV1gp2</td><td>-</td></tr>
<tr><td>siRNA-t0102415</td><td>CCAACTGCGACCCATGCTTTCGA</td><td>23</td><td>6</td><td>FgHV1gp2</td><td>-</td></tr>
<tr><td>siRNA-t0099983</td><td>CCAAGAACCGTTGATGGCTT</td><td>20</td><td>6</td><td>FGSG_02395</td><td>K15417|1|0.0|1702|fgr:FG02395.1|zearalenone synthase, nonreducing iterative type I polyketide synthase</td></tr>
<tr><td>siRNA-t0101027</td><td>CCAAGATTTGTCTAACTTGAT</td><td>21</td><td>6</td><td>FGSG_11080</td><td>-</td></tr>
<tr><td>siRNA-t0100974</td><td>CCACATCACGCTCGGGGTCAA</td><td>21</td><td>6</td><td>FgHV1gp2</td><td>-</td></tr>
<tr><td>siRNA-t0104253</td><td>CCACGTACCGTCGGTGTCGC</td><td>20</td><td>6</td><td>FGSG_12896</td><td>K09184|1|5e-37|156|uma:UM04252.1|GATA-binding protein, other eukaryote</td></tr>
<tr><td>siRNA-t0095139</td><td>CCAGAATCGAGCTAAACGAAC</td><td>21</td><td>6</td><td>FgHV1gp2</td><td>-</td></tr>
<tr><td>siRNA-t0095262</td><td>CCAGATCTTATAGACAGGCCCAA</td><td>23</td><td>6</td><td>FgHV1gp2</td><td>-</td></tr>
<tr><td>siRNA-t0100507</td><td>CCAGGATTCTTCTCCACGTCA</td><td>21</td><td>6</td><td>FGSG_08844</td><td>K01183|1|1e-06|55.1|pan:PODANSg4492|chitinase [EC:3.2.1.14]</td></tr>
<tr><td>siRNA-t0096333</td><td>CCAGTGTCGAACCATACTGGAC</td><td>22</td><td>6</td><td>FgHV1gp2</td><td>-</td></tr>
<tr><td>siRNA-t0096974</td><td>CCATAAACGCTGCCGGGATGT</td><td>21</td><td>6</td><td>FgHV1gp2</td><td>-</td></tr>
<tr><td>siRNA-t0096255</td><td>CCATACAATGCCGCCAACCACCA</td><td>23</td><td>6</td><td>FgHV1gp2</td><td>-</td></tr>
<tr><td>siRNA-t0097680</td><td>CCATGACGTACGACCCCGAAAG</td><td>22</td><td>6</td><td>FgHV1gp1</td><td>-</td></tr>
<tr><td>siRNA-t0099163</td><td>CCATTCGTTACAATCGTGGACTTC</td><td>24</td><td>6</td><td>FgHV1gp2</td><td>-</td></tr>
<tr><td>siRNA-t0099114</td><td>CCATTTCATTCGACTGTGGT</td><td>20</td><td>6</td><td>FGSG_01312</td><td>K12767|1|0.0|3537|fgr:FG01312.1|serine/threonine-protein kinase RIM15 [EC:2.7.11.1]</td></tr>
<tr><td>siRNA-t0098240</td><td>CCCGTCGAAGACCAAAAATT</td><td>20</td><td>6</td><td>FgHV1gp1</td><td>-</td></tr>
<tr><td>siRNA-t0104179</td><td>CCGAAGGAGACATTTGAGGG</td><td>20</td><td>6</td><td>FGSG_09910</td><td>-</td></tr>
<tr><td>siRNA-t0098936</td><td>CCGACGTTTTGGCGAGACCGAT</td><td>22</td><td>6</td><td>FgHV1gp2</td><td>-</td></tr>
<tr><td>siRNA-t0101694</td><td>CCGAGCACCATACAATGCCG</td><td>20</td><td>6</td><td>FGSG_05627</td><td>K03002|1|0.0|2456|fgr:FG05627.1|DNA-directed RNA polymerase I subunit RPA2 [EC:2.7.7.6]</td></tr>
<tr><td>siRNA-t0098431</td><td>CCGCGTGTGATTGGTCTCTT</td><td>20</td><td>6</td><td>FGSG_03276</td><td>-</td></tr>
<tr><td>siRNA-t0094611</td><td>CCTATAGATCGCGCGTAAGC</td><td>20</td><td>6</td><td>FgHV1gp2</td><td>-</td></tr>
<tr><td>siRNA-t0100708</td><td>CGAAGGAACTCCACATAGCGTCGTC</td><td>25</td><td>6</td><td>FgHV1gp2</td><td>-</td></tr>
<tr><td>siRNA-t0099380</td><td>CGACAATGGCGCATACACGGA</td><td>21</td><td>6</td><td>FgHV1gp2</td><td>-</td></tr>
<tr><td>siRNA-t0105482</td><td>CGACACCGACGGTACGTGGAA</td><td>21</td><td>6</td><td>FgHV1gp2</td><td>-</td></tr>
<tr><td>siRNA-t0103867</td><td>CGACGACCGTGCGAAATTGT</td><td>20</td><td>6</td><td>FGSG_13129</td><td>-</td></tr>
<tr><td>siRNA-t0095766</td><td>CGACGTACTTAGGGTTGTTGAGA</td><td>23</td><td>6</td><td>FgHV1gp2</td><td>-</td></tr>
<tr><td>siRNA-t0102921</td><td>CGACGTACTTAGGGTTGTTGAGAA</td><td>24</td><td>6</td><td>FgHV1gp2</td><td>-</td></tr>
<tr><td>siRNA-t0105932</td><td>CGGAACCCATTGATCATTCTCCT</td><td>23</td><td>6</td><td>FgHV1gp2</td><td>-</td></tr>
<tr><td>siRNA-t0103600</td><td>CGGTTACCAAGGTGTGTGAAGTA</td><td>23</td><td>6</td><td>FgHV1gp2</td><td>-</td></tr>
<tr><td>siRNA-t0096392</td><td>CGTAGATGTGATGCCTTGTCT</td><td>21</td><td>6</td><td>FgHV1gp2</td><td>-</td></tr>
<tr><td>siRNA-t0099471</td><td>CGTGAACCAGATCATAACCCGCA</td><td>23</td><td>6</td><td>FgHV1gp2</td><td>-</td></tr>
<tr><td>siRNA-t0103575</td><td>CGTGATAAATGAACGTCTGACT</td><td>22</td><td>6</td><td>FgHV1gp2</td><td>-</td></tr>
</table>

<table>
<tr><td>siRNA-t0097087</td><td>CGTGGAAATCTTCAGGCCCTGGGA</td><td>24</td><td>6</td><td>FgHV1gp2</td><td>-</td></tr>
<tr><td>siRNA-t0095841</td><td>CTAATATCGTCATTCTCTTCCA</td><td>22</td><td>6</td><td>FGSG_09836</td><td>-</td></tr>
<tr><td>siRNA-t0099939</td><td>CTAGACCGAAGAAAGACCACATCC</td><td>24</td><td>6</td><td>FgHV1gp2</td><td>-</td></tr>
<tr><td>siRNA-t0098953</td><td>CTAGTTCGAAGGAGGCGGAA</td><td>20</td><td>6</td><td>FGSG_11206</td><td>-</td></tr>
<tr><td>siRNA-t0100464</td><td>CTATGTTTGACGGCTTGTCCT</td><td>21</td><td>6</td><td>FGSG_13806</td><td>K01187|1|0.0|649|fgr:FG10662.1|alpha-glucosidase [EC:3.2.1.20]</td></tr>
<tr><td>siRNA-t0103009</td><td>CTCAAGGGACGGAAGCCATCA</td><td>21</td><td>6</td><td>FGSG_09996</td><td>-</td></tr>
<tr><td>siRNA-t0094781</td><td>CTGACATATTCGGACTCATCC</td><td>21</td><td>6</td><td>FGSG_13252</td><td>-</td></tr>
<tr><td>siRNA-t0094223</td><td>CTGATCTGGCTCGTGACTTT</td><td>20</td><td>6</td><td>FgHV1gp2</td><td>-</td></tr>
<tr><td>siRNA-t0104361</td><td>CTGCCGCGGATAACCTATACTTG</td><td>23</td><td>6</td><td>FgHV1gp2</td><td>-</td></tr>
<tr><td>siRNA-t0103676</td><td>CTGGAAGAATCATAACCGACG</td><td>21</td><td>6</td><td>FgHV1gp2</td><td>-</td></tr>
<tr><td>siRNA-t0104730</td><td>CTGGATGTGGGTGCGCCGAATGA</td><td>23</td><td>6</td><td>FgHV1gp2</td><td>-</td></tr>
<tr><td>siRNA-t0098758</td><td>CTTGAGACAGTTCCGCGTGTGA</td><td>22</td><td>6</td><td>FgHV1gp2</td><td>-</td></tr>
<tr><td>siRNA-t0095699</td><td>CTTGAGACAGTTCCGCGTGTGATT</td><td>24</td><td>6</td><td>FgHV1gp2</td><td>-</td></tr>
<tr><td>siRNA-t0105737</td><td>CTTGCCGCGCGTCTCACTGA</td><td>20</td><td>6</td><td>FgHV1gp2</td><td>-</td></tr>
<tr><td>siRNA-t0096440</td><td>CTTGCGGTGTGGGTCTTCTGA</td><td>21</td><td>6</td><td>FGSG_08904</td><td>-</td></tr>
<tr><td>siRNA-t0095396</td><td>GAAATCGAGGGTGTTGGGGTC</td><td>21</td><td>6</td><td>FgHV1gp2</td><td>-</td></tr>
<tr><td>siRNA-t0097232</td><td>GAAGAGAGGTGAAATGGGAT</td><td>20</td><td>6</td><td>FGSG_07193</td><td>-</td></tr>
<tr><td>siRNA-t0098731</td><td>GAATGAGATCGTCCTCCATTAC</td><td>22</td><td>6</td><td>FGSG_01547</td><td>-</td></tr>
<tr><td>siRNA-t0097671</td><td>GACATTTTCGCGCGCTGTACT</td><td>21</td><td>6</td><td>FGSG_02394</td><td>K01932|1|0.0|942|afm:AFUA_1G10380|[EC:6.3.2.-]</td></tr>
<tr><td>siRNA-t0102759</td><td>GACCCACACCGCAAGACTCT</td><td>20</td><td>6</td><td>FGSG_06054</td><td>K01784|1|3e-65|249|fgr:FG07983.1|UDP-glucose 4-epimerase [EC:5.1.3.2]</td></tr>
<tr><td>siRNA-t0095989</td><td>GACCGAAGAAAGACCACATCC</td><td>21</td><td>6</td><td>FgHV1gp2</td><td>-</td></tr>
<tr><td>siRNA-t0100472</td><td>GACGGTACGTGGAAATCTTC</td><td>20</td><td>6</td><td>FgHV1gp2</td><td>-</td></tr>
<tr><td>siRNA-t0096899</td><td>GACGTACAAACAAATGCTCA</td><td>20</td><td>6</td><td>FGSG_13253</td><td>-</td></tr>
<tr><td>siRNA-t0100307</td><td>GAGTGCTCATCGTTGTGCCAAC</td><td>22</td><td>6</td><td>FGSG_04332</td><td>-</td></tr>
<tr><td>siRNA-t0095756</td><td>GAGTGGCATATTCAAGAATCAAT</td><td>23</td><td>6</td><td>FGSG_11486</td><td>K11369|1|2e-49|196|ani:AN3929.2|retrograde regulation protein 2;K13336|2|6e-49|194|afv:AFLA_048740|peroxin-3;K03448|3|2e-42|172|mgr:MGG_02319|MFS transporter, ACS family, pantothenate transporter</td></tr>
<tr><td>siRNA-t0099479</td><td>GAGTGTGTCGCGTCTACCTTG</td><td>21</td><td>6</td><td>FgHV1gp2</td><td>-</td></tr>
<tr><td>siRNA-t0095544</td><td>GAGTGTGTCGCGTCTACCTTGTTCA</td><td>25</td><td>6</td><td>FgHV1gp2</td><td>-</td></tr>
<tr><td>siRNA-t0104310</td><td>GATACTCTTCAGCGTAACGTGACC</td><td>24</td><td>6</td><td>FgHV1gp2</td><td>-</td></tr>
<tr><td>siRNA-t0100614</td><td>GATCAACGTCAGAGGCCTCG</td><td>20</td><td>6</td><td>FGSG_06054</td><td>K01784|1|3e-65|249|fgr:FG07983.1|UDP-glucose 4-epimerase [EC:5.1.3.2]</td></tr>
<tr><td>siRNA-t0099503</td><td>GATCTGGCTCGTGACTTTTCCCTCA</td><td>25</td><td>6</td><td>FgHV1gp2</td><td>-</td></tr>
<tr><td>siRNA-t0097566</td><td>GCAATGCAACAGACTTCTTCTC</td><td>22</td><td>6</td><td>FgHV1gp2</td><td>-</td></tr>
<tr><td>siRNA-t0098557</td><td>GCAGTTGGTCCGCGAATTCTGCC</td><td>23</td><td>6</td><td>FgHV1gp2</td><td>-</td></tr>
<tr><td>siRNA-t0097589</td><td>GCATCCTCGACCCACTCATGGGCA</td><td>24</td><td>6</td><td>FgHV1gp2</td><td>-</td></tr>
<tr><td>siRNA-t0094125</td><td>GCATGACTTTACAAGCTGGTCTG</td><td>23</td><td>6</td><td>FgHV1gp2</td><td>-</td></tr>
<tr><td>siRNA-t0097407</td><td>GCATTCCGGTTTGTATCACC</td><td>20</td><td>6</td><td>FGSG_00120</td><td>-</td></tr>
<tr><td>siRNA-t0099058</td><td>GCCATCGCGCGGTCCTGTAT</td><td>20</td><td>6</td><td>FGSG_04913</td><td>K05349|1|0.0|1664|fgr:FG04913.1|beta-glucosidase [EC:3.2.1.21]</td></tr>
<tr><td>siRNA-t0094645</td><td>GCCCCAGCAGACTTGGAAGTT</td><td>21</td><td>6</td><td>FGSG_05337</td><td>K09291|1|0.0|3327|fgr:FG05337.1|nucleoprotein TPR</td></tr>
<tr><td>siRNA-t0097017</td><td>GCCCCGGAGCGCAAAGGTAA</td><td>20</td><td>6</td><td>FGSG_02139</td><td>K05658|1|9e-34|145|smp:SMAC_06012|ATP-binding cassette, subfamily B (MDR/TAP), member 1</td></tr>
<tr><td>siRNA-t0102336</td><td>GCGAATTGCAACGAGGGCCCAG</td><td>22</td><td>6</td><td>FGSG_07277</td><td>K01976|1|3e-32|139|sce:YBR222C|[EC:6.-.-.-];K01895|4|2e-19|96.7|mgr:MGG_04590|acetyl-CoA synthetase [EC:6.2.1.1];K01897|5|2e-19|96.7|ncr:NCU06063|long-chain acyl-CoA synthetase [EC:6.2.1.3]</td></tr>
<tr><td>siRNA-t0102502</td><td>GCGACACCGACGGTACGTGGAAA</td><td>23</td><td>6</td><td>FgHV1gp2</td><td>-</td></tr>
<tr><td>siRNA-t0094246</td><td>GCGGCATTGTATGGTGCTCGG</td><td>21</td><td>6</td><td>FGSG_10874</td><td>K11274|1|2e-29|128|ani:AN5139.2|chromosome transmission fidelity protein 4</td></tr>
<tr><td>siRNA-t0095378</td><td>GCGGTACGATATTTGAGTATCT</td><td>22</td><td>6</td><td>FGSG_05462</td><td>K01072|1|2e-159|564|afm:AFUA_2G08440|ubiquitin thiolesterase [EC:3.1.2.15];K11838|3|3e-41|171|clu:CLUG_02963|ubiquitin carboxyl-terminal hydrolase 7 [EC:3.1.2.15]</td></tr>
<tr><td>siRNA-t0103472</td><td>GCTGCTACTCGAACCGCCCTT</td><td>21</td><td>6</td><td>FgHV1gp2</td><td>-</td></tr>
<tr><td>siRNA-t0102210</td><td>GCTTCCCATGACTGCTAGAA</td><td>20</td><td>6</td><td>FGSG_09177</td><td>-</td></tr>
<tr><td>siRNA-t0103165</td><td>GGAAAGGAAAAGTTCTCGCCAGCAA</td><td>25</td><td>6</td><td>FgHV1gp1</td><td>-</td></tr>
<tr><td>siRNA-t0096964</td><td>GGACCGACGTTTTGGCGAGA</td><td>20</td><td>6</td><td>FGSG_01778</td><td>-</td></tr>
<tr><td>siRNA-t0099527</td><td>GGACTGGCCTGTTGTCACCTT</td><td>21</td><td>6</td><td>FgHV1gp2</td><td>-</td></tr>
<tr><td>siRNA-t0098770</td><td>GGACTTGAAATCCTGGGCGCTAAC</td><td>24</td><td>6</td><td>FgHV1gp2</td><td>-</td></tr>
<tr><td>siRNA-t0094951</td><td>GGAGGCACAAGAACATCAGG</td><td>20</td><td>6</td><td>FGSG_04287</td><td>K13830|1|0.0|3204|fgr:FG04287.1|pentafunctional AROM polypeptide [EC:4.2.3.4 4.2.1.10 1.1.1.25 2.7.1.71 2.5.1.19]</td></tr>
<tr><td>siRNA-t0105472</td><td>GGCCGGCAGTCCTAGCAAGT</td><td>20</td><td>6</td><td>FGSG_10569</td><td>K06694|1|2e-07|58.2|zro:ZYRO0B15114g|26S proteasome non-ATPase regulatory subunit 10</td></tr>
<tr><td>siRNA-t0103548</td><td>GGCGCATACACGGAACCCATTGA</td><td>23</td><td>6</td><td>FgHV1gp2</td><td>-</td></tr>
<tr><td>siRNA-t0104605</td><td>GGCGGTTCGAGTAGCAGCTT</td><td>20</td><td>6</td><td>FGSG_05041</td><td>-</td></tr>
<tr><td>siRNA-t0105215</td><td>GGGTGCTCATGAGGTTGGCAT</td><td>21</td><td>6</td><td>FGSG_04349</td><td>K13210|1|0.0|903|fgr:FG04349.1|far upstream element-binding protein</td></tr>
<tr><td>siRNA-t0105987</td><td>GGTGGTTCATTGCGATGTAATCTT</td><td>24</td><td>6</td><td>FgHV1gp2</td><td>-</td></tr>
<tr><td>siRNA-t0096161</td><td>GGTGTCAAGATTGGATTTCTTCC</td><td>23</td><td>6</td><td>FgHV1gp2</td><td>-</td></tr>
<tr><td>siRNA-t0094344</td><td>GGTTCGACACTGGAAGAGAA</td><td>20</td><td>6</td><td>FGSG_05996</td><td>K00936|1|3e-61|238|afm:AFUA_6G10240|[EC:2.7.3.-];K11231|2|6e-39|163|afm:AFUA_2G00660|osomolarity two-component system, sensor histidine kinase SLN1 [EC:2.7.13.3];K02486|3|2e-38|161|spo:SPAC27E2.09|two-component system, unclassified family, sensor kinase [EC:2.7.13.3]</td></tr>
<tr><td>siRNA-t0101429</td><td>GTAAGTGACGAGGCCTCTGA</td><td>20</td><td>6</td><td>FGSG_07534</td><td>-</td></tr>
<tr><td>siRNA-t0097617</td><td>GTACGTGGAAATCTTCAGGCCCTGG</td><td>25</td><td>6</td><td>FgHV1gp2</td><td>-</td></tr>
<tr><td>siRNA-t0100670</td><td>GTATGAACCCTTCCACGATTGGCGT</td><td>25</td><td>6</td><td>FgHV1gp2</td><td>-</td></tr>
<tr><td>siRNA-t0098451</td><td>GTCTCCGAACGTGATCTCATCA</td><td>22</td><td>6</td><td>FgHV1gp2</td><td>-</td></tr>
<tr><td>siRNA-t0095639</td><td>GTCTTGCGGTGTGGGTCTTC</td><td>20</td><td>6</td><td>FgHV1gp2</td><td>-</td></tr>
<tr><td>siRNA-t0096485</td><td>GTGCTGGGTTCTGGTAGAAG</td><td>20</td><td>6</td><td>FGSG_07312</td><td>-</td></tr>
<tr><td>siRNA-t0096510</td><td>GTGGGTTCTTGAAGTTGGTGCCGA</td><td>24</td><td>6</td><td>FgHV1gp2</td><td>-</td></tr>
<tr><td>siRNA-t0099732</td><td>GTGTAGAAACTGTCGCACTC</td><td>20</td><td>6</td><td>FGSG_02035</td><td>K00777|1|1e-24|115|afm:AFUA_7G06290|[EC:2.4.2.-]</td></tr>
<tr><td>siRNA-t0099813</td><td>GTGTCGAACCATACTGGACC</td><td>20</td><td>6</td><td>FGSG_08097</td><td>K00688|1|7e-16|87.0|pno:SNOG_13460|starch phosphorylase [EC:2.4.1.1]</td></tr>
<tr><td>siRNA-t0094860</td><td>GTGTGTCGCGTCTACCTTGTTC</td><td>22</td><td>6</td><td>FgHV1gp2</td><td>-</td></tr>
<tr><td>siRNA-t0104809</td><td>GTGTTCGCATCCGTTCTGTG</td><td>20</td><td>6</td><td>FGSG_08999</td><td>-</td></tr>
<tr><td>siRNA-t0105835</td><td>GTGTTCTGAAACCATCAGGAC</td><td>21</td><td>6</td><td>FgHV1gp2</td><td>-</td></tr>
<tr><td>siRNA-t0102811</td><td>GTTGAAAATCAGAGAGCAGGAT</td><td>22</td><td>6</td><td>FgHV1gp2</td><td>-</td></tr>
<tr><td>siRNA-t0094265</td><td>TAATCATCATCGTAGTCATCGTC</td><td>23</td><td>6</td><td>FGSG_13700</td><td>-</td></tr>
<tr><td>siRNA-t0101267</td><td>TAATCATCATCGTAGTCATCGTCA</td><td>24</td><td>6</td><td>FGSG_12871</td><td>-</td></tr>
<tr><td>siRNA-t0101137</td><td>TAGAACAGACGTCTCTCACG</td><td>20</td><td>6</td><td>FgHV1gp2</td><td>-</td></tr>
<tr><td>siRNA-t0096261</td><td>TATACTTGATCTCAGCACGCCGGTT</td><td>25</td><td>6</td><td>FgHV1gp2</td><td>-</td></tr>
<tr><td>siRNA-t0095668</td><td>TCAAATCAAGTTGGTATCCCTC</td><td>22</td><td>6</td><td>FgHV1gp2</td><td>-</td></tr>
<tr><td>siRNA-t0097847</td><td>TCAAGATACAGCAACTGGGTT</td><td>21</td><td>6</td><td>FGSG_09526</td><td>-</td></tr>
<tr><td>siRNA-t0095446</td><td>TCAATGGCGACACCGACGGTA</td><td>21</td><td>6</td><td>FGSG_05709</td><td>K13830|1|2e-68|259|lbc:LACBIDRAFT_233717|pentafunctional AROM polypeptide [EC:4.2.3.4 4.2.1.10 1.1.1.25 2.7.1.71 2.5.1.19]</td></tr>
<tr><td>siRNA-t0096357</td><td>TCCAACTGCGACCCATGCTTTC</td><td>22</td><td>6</td><td>FgHV1gp2</td><td>-</td></tr>
<tr><td>siRNA-t0099318</td><td>TCCACATAGCGTCGTCACCT</td><td>20</td><td>6</td><td>FGSG_12057</td><td>-</td></tr>
<tr><td>siRNA-t0096779</td><td>TCCAGGGGCGTTTTTCTCGGG</td><td>21</td><td>6</td><td>FGSG_07182</td><td>K02358|1|0.0|889|fgr:FG07182.1|elongation factor Tu</td></tr>
<tr><td>siRNA-t0096640</td><td>TCCATAAACGCTGCCGGGATGTA</td><td>23</td><td>6</td><td>FgHV1gp2</td><td>-</td></tr>
<tr><td>siRNA-t0103430</td><td>TCCGAACGTGATCTCATCAT</td><td>20</td><td>6</td><td>FGSG_02786</td><td>K05658|1|0.0|2468|fgr:FG02786.1|ATP-binding cassette, subfamily B (MDR/TAP), member 1</td></tr>
<tr><td>siRNA-t0096817</td><td>TCCGAAGGAACTCCACATAGCGT</td><td>23</td><td>6</td><td>FgHV1gp2</td><td>-</td></tr>
<tr><td>siRNA-t0100050</td><td>TCCGACAATGGCGCATACACGGA</td><td>23</td><td>6</td><td>FgHV1gp2</td><td>-</td></tr>
<tr><td>siRNA-t0101838</td><td>TCCTAGAGATCTCCGCAGAAGAA</td><td>23</td><td>6</td><td>FGSG_00352</td><td>K08064|1|2e-78|291|mgr:MGG_00354|nuclear transcription factor Y, alpha</td></tr>
<tr><td>siRNA-t0102654</td><td>TCCTGGTTAGTTCTTGGGTA</td><td>20</td><td>6</td><td>FGSG_05462</td><td>K01072|1|2e-159|564|afm:AFUA_2G08440|ubiquitin thiolesterase [EC:3.1.2.15];K11838|3|3e-41|171|clu:CLUG_02963|ubiquitin carboxyl-terminal hydrolase 7 [EC:3.1.2.15]</td></tr>
<tr><td>siRNA-t0101096</td><td>TCGAAAGTGAAAGTGTCGTAGGA</td><td>23</td><td>6</td><td>FgHV1gp1</td><td>-</td></tr>
<tr><td>siRNA-t0096846</td><td>TCGGCAAATTTGGCCAAATCGTCA</td><td>24</td><td>6</td><td>FgHV1gp2</td><td>-</td></tr>
<tr><td>siRNA-t0105245</td><td>TCGTCATACATTGCACGAGGGACG</td><td>24</td><td>6</td><td>FgHV1gp2</td><td>-</td></tr>
</table>

siRNA-t0104532	TCGTGCGCTTCTGGATCAATGT	22	6	FgHV1gp2	-
siRNA-t0101972	TGCAGCGGCAAATACAGGACCG	22	6	FgHV1gp2	-
siRNA-t0094686	TGCAGTTGGTCCGCGAATTCTGC	23	6	FgHV1gp2	-
siRNA-t0104594	TGCCTGGGAGACAGAGCCTGA	21	6	FgHV1gp2	-
siRNA-t0102062	TGCGACGACCGTGCGAAATTGT	22	6	FgHV1gp2	-
siRNA-t0094224	TGCTGCCGCGGATAACCTATACTTG	25	6	FgHV1gp2	-
siRNA-t0095264	TGCTTGTCAGTGTCAGGATCCG	22	6	FgHV1gp2	-
siRNA-t0105983	TGGAGTGGCATATTCAAGAATCAAT	25	6	FgHV1gp2	-
siRNA-t0105696	TGGATTTTGCTACCAGCTGCAGCA	24	6	FgHV1gp1	-
siRNA-t0105503	TGGCGAGACCGATGATTTCCAT	22	6	FgHV1gp2	-
siRNA-t0096404	TGGGATGGGTTTCTGGTTCCA	21	6	FGSG_13796	K14338|1|8e-09|60.8|fgr:FG01972.1|cytochrome P450 / NADPH-cytochrome P450 reductase [EC:1.14.14.1 1.6.2.4];K00517|2|4e-08|58.9|afm:AFUA_3G14760|[EC:1.14.-.-];K00493|4|1e-07|56.6|pic:PICST_35590|unspecific monooxygenase [EC:1.14.14.1]
siRNA-t0103725	TGGGTTCTTGAAGTTGGTGCCGAC	24	6	FgHV1gp2	-
siRNA-t0105792	TGGTATTGTCAGGGTTCAAAC	21	6	FGSG_00843	K03021|1|0.0|2410|fgr:FG00843.1|DNA-directed RNA polymerase III subunit RPC2 [EC:2.7.7.6]
siRNA-t0100235	TGGTGAGGTATGACGTGTTCATTA	24	6	FgHV1gp2	-
siRNA-t0100679	TGTAATGGAGGACGATCTCATTCGT	25	6	FgHV1gp2	-
siRNA-t0105544	TGTAGATCTTGCTGTAGTGTCTG	23	6	FgHV1gp2	-
siRNA-t0094389	TGTTGTTGGTCTTCCAGGCATC	22	6	FGSG_03064	-
siRNA-t0098541	TTACAATCGTGGACTTCCTTTA	22	6	FgHV1gp2	-
siRNA-t0097721	TTAGAAGCTTGACACGAATGATGT	24	6	FgHV1gp2	-
siRNA-t0097162	TTCAAGTATCGTCACTTCTT	20	6	FgHV1gp2	-
siRNA-t0104334	TTCAATGATGGCACGTCGACAGGC	24	6	FgHV1gp2	-
siRNA-t0105939	TTCCATAAACGCTGCCGGGATG	22	6	FgHV1gp2	-
siRNA-t0094722	TTCGATGGATGCTGCGAAACCCAA	24	6	FgHV1gp2	-
siRNA-t0094938	TTCTGGAACGTCAAATCAAGTTGGT	25	6	FgHV1gp2	-
siRNA-t0105551	TTGCACGAGGGACGAGCTTGG	21	6	FgHV1gp2	-
siRNA-t0100675	TTGGGTGGCTTTGAAGTTGATCT	23	6	FgHV1gp2	-
siRNA-t0101709	TTGGTATTGTCAGGGTTCAAA	21	6	FGSG_10523	K01932|1|4e-178|626|afm:AFUA_6G12080|[EC:6.3.2.-]
siRNA-t0105430	TTGGTCTCTTGCAGTGCAATACCA	24	6	FGSG_08418	-
siRNA-t0106021	TTGTCAGTGTCAGGATCCGTC	21	6	FGSG_05487	K14318|1|2e-25|117|cci:CC1G_01275|nuclear pore complex protein Nup88
siRNA-t0103418	TTGTGGGAGCAAGAGAATAG	20	6	FGSG_00615	-
siRNA-t0103203	TTTCAATGATGGCACGTCGACAGGC	25	6	FgHV1gp2	-
siRNA-t0114583	AAACCACACTCAGCAGGAACT	21	5	FGSG_07104	K13338|1|0.0|2298|fgr:FG07104.1|peroxin-1
siRNA-t0122745	AAATGAACGTCTGACTGTAG	20	5	FgHV1gp2	-
siRNA-t0114401	AAATTTTTGGTCTTCGACGGGTACG	25	5	FGSG_13308	-
siRNA-t0112715	AACACGCAGCAGAATTCTAGCCTGG	25	5	FgHV1gp2	-
siRNA-t0118350	AAGGCTTGTCGTCGGGAGAATCCAG	25	5	FgHV1gp2	-
siRNA-t0114741	AAGTATCGTCACTTCTTCGTAG	22	5	FgHV1gp2	-
siRNA-t0107114	AATGAAATGTATGATTCGAT	20	5	FGSG_11505	-
siRNA-t0118375	AATTTGATTGATTCTAGCTT	20	5	FgHV1gp2	-
siRNA-t0122432	AATTTGGCCCCAGCAGACTTGG	22	5	FgHV1gp2	-
siRNA-t0115807	ACAATGAAGACGCAAGCCCGCT	22	5	FgHV1gp2	-
siRNA-t0108279	ACAATTTATCGTGCGCTTCTGGAT	24	5	FgHV1gp2	-
siRNA-t0114955	ACAGCAAGATCTACATGCAT	20	5	FGSG_06489	-
siRNA-t0119906	ACAGGCTAGGAACGATGCCTCG	22	5	FGSG_02233	-
siRNA-t0117204	ACATATTCGGACTCATCCCAC	21	5	FgHV1gp2	-
siRNA-t0121565	ACATGATGAGTAAGCGGGCTT	21	5	FGSG_03736	K14859|1|6e-27|122|scm:SCHCODRAFT_255991|ribosome biogenesis protein SSF1/2
siRNA-t0115591	ACCCATGCTTTCGACGTACTT	21	5	FgHV1gp2	-
siRNA-t0119023	ACCCTTTGGAGACTTGGCAGTG	22	5	FgHV1gp2	-
siRNA-t0106141	ACCGAGGCAGAGCATTCAGC	20	5	FGSG_04842	-
siRNA-t0113527	ACGCAGCAGAATTCTAGCCTGGTTG	25	5	FgHV1gp2	-
siRNA-t0120826	ACGCGTGTCGTTAGAAGCTTGA	22	5	FgHV1gp2	-
siRNA-t0113364	ACGCTGCCGGGATGTAATCAAT	22	5	FgHV1gp2	-
siRNA-t0116282	ACGTACTTAGGGTTGTTGAGAACGC	25	5	FgHV1gp2	-
siRNA-t0117175	ACTAATATCGTCATTCTCTTCC	22	5	FgHV1gp2	-
siRNA-t0106479	ACTGCGACCCATGCTTTCGACG	22	5	FgHV1gp2	-
siRNA-t0121086	AGAGAAGAATGAGGGAAGAAA	21	5	FGSG_01483	-
siRNA-t0117498	AGATCCCACTCTAGCAATGCATC	23	5	FgHV1gp1	-
siRNA-t0106193	AGATCCCACTCTAGCAATGCATCAC	25	5	FgHV1gp1	-
siRNA-t0120517	AGATTCCAACCTGTTGTCAA	20	5	FGSG_11514	-
siRNA-t0121313	AGCAGAGAAGAAGTCTGTTGCAT	23	5	FGSG_10617	K14859|1|2e-98|359|scm:SCHCODRAFT_255991|ribosome biogenesis protein SSF1/2;K00143|2|1e-14|82.0|zro:ZYRO0C16566g|L-aminoadipate-semialdehyde dehydrogenase [EC:1.2.1.31]
siRNA-t0109928	AGCAGGATCTCTGACATATTCG	22	5	FgHV1gp2	-
siRNA-t0120812	AGCCATTCGTTACAATCGTGGA	22	5	FgHV1gp2	-
siRNA-t0114052	AGCGGTCGCATCAGCCATCA	20	5	FGSG_06089	K08874|1|0.0|7852|fgr:FG06089.1|transformation/transcription domain-associated protein
siRNA-t0109527	AGCGTGACGAAGATCCCACTCTA	23	5	FgHV1gp1	-
siRNA-t0113208	AGCGTGTAGGGCTGTCCCAT	20	5	FGSG_05297	K14676|1|0.0|2814|fgr:FG05297.1|lysophospholipid hydrolase [EC:3.1.1.5]
siRNA-t0123174	AGGCCTCTGACGTTGATCTCT	21	5	FgHV1gp2	-
siRNA-t0108106	AGGCGTCGACACGATGATACCCTC	24	5	FgHV1gp2	-
siRNA-t0111963	AGTCTCTTGTTCTGGAACGTC	21	5	FGSG_04168	K12494|1|4e-11|70.9|cnb:CNBD1140|PH and SEC7 domain-containing protein
siRNA-t0117443	AGTCTTGCGGTGTGGGTCTTC	21	5	FgHV1gp2	-
siRNA-t0111824	AGTGAAAGTGTCGTAGGAAAG	21	5	FgHV1gp1	-
siRNA-t0108463	AGTGGCGCATTTCTTTCTTACCCT	24	5	FgHV1gp2	-
siRNA-t0117006	AGTGTCGTAGGAAAGGAAAAGTT	23	5	FgHV1gp1	-
siRNA-t0107041	AGTTCACGAAGGGAGAGGCTA	21	5	FGSG_10561	K01183|1|0.0|635|afm:AFUA_5G03960|chitinase [EC:3.2.1.14]
siRNA-t0110244	ATAACACGCAGCAGAATTCTAGCC	24	5	FgHV1gp2	-
siRNA-t0112702	ATACAAACCGGAATGCACTT	20	5	FgHV1gp2	-
siRNA-t0115280	ATGATCATCAGCACCGTACGGATCC	25	5	FgHV1gp2	-
siRNA-t0112551	ATGCTGGCATAAAGGAATCCC	21	5	FGSG_10767	-
siRNA-t0115691	ATGCTTCCGGATTATGCCCA	20	5	FGSG_07795	K12600|1|7e-10|63.5|fgr:FG06322.1|superkiller protein 3
siRNA-t0120060	ATGGCAACGGAAGCGATATTTG	22	5	FGSG_06631	-
siRNA-t0119518	ATGGTAATTGCGGTCCATGG	20	5	FGSG_09681	K01533|1|0.0|2289|fgr:FG09681.1|Cu2+-exporting ATPase [EC:3.6.3.4]
siRNA-t0116177	ATGTGGGTGCGCCGAATGAA	20	5	FGSG_06168	-
siRNA-t0116510	ATGTTTGCTTGGTTTCGGGACGC	23	5	FgHV1gp2	-
siRNA-t0115721	ATTACGGCGGCATTCATGAA	20	5	FGSG_08312	K08711|1|0.0|1726|cne:CNB05770|ATP-binding cassette, subfamily G (WHITE), member 2, PDR
siRNA-t0121620	ATTCGTTACAATCGTGGACTTCCTT	25	5	FgHV1gp2	-
siRNA-t0116069	CAAATACAGGACCGCGCGATGGCGC	25	5	FgHV1gp2	-
siRNA-t0109996	CAACCACAAGCCACTGCAGTTT	22	5	FgHV1gp2	-
siRNA-t0118634	CAAGTAAGCAGAGCGTGTAGGGCTG	25	5	FgHV1gp2	-
siRNA-t0110038	CAATCGTGGACTTCCTTTAGGAAA	24	5	FgHV1gp2	-
siRNA-t0121088	CACGATTGGCGTGAGTGCGTA	21	5	FgHV1gp2	-
siRNA-t0117294	CACGCAGCAGAATTCTAGCCTG	22	5	FgHV1gp2	-
siRNA-t0110751	CAGCAATGGTACCATCACTCA	21	5	FGSG_10283	-
siRNA-t0112932	CAGCATGGCCGGCAGTCCTAGCA	23	5	FgHV1gp2	-
siRNA-t0119725	CAGCCCAAGTCAAGACCCCA	20	5	FGSG_06099	-
siRNA-t0113926	CAGCTGTTCTCTGTCGGCATGTTG	24	5	FgHV1gp2	-
siRNA-t0113231	CAGGATCTCTGACATATTCGGAC	23	5	FgHV1gp2	-
siRNA-t0121011	CAGGATTGAAGTGTAATGCG	20	5	FgHV1gp2	-
siRNA-t0114439	CAGTCCCAGCGCCTCCATGCA	21	5	FGSG_13398	K00632|1|0.0|813|fgr:FG08287.1|acetyl-CoA acyltransferase [EC:2.3.1.16]
siRNA-t0118077	CAGTTCCGCGTGTGATTGGTCTCTT	25	5	FGSG_03919	-

siRNA-t0122262	CAGTTCCTGCTGAGTGTGGTT	21	5	FgHV1gp2	-
siRNA-t0108104	CATACCTTTGAGCTCCTTGAGGAAT	25	5	FgHV1gp2	-
siRNA-t0121429	CATAGCGTCGTCACCTTGAT	20	5	FGSG_13835	-
siRNA-t0111686	CATTTCGGCGAGTCTAATCTT	21	5	FgHV1gp2	-
siRNA-t0106601	CATTTGCGCACATATCTCCTTG	22	5	FgHV1gp2	-
siRNA-t0115515	CCAACGAGGTTATGGCAGAA	20	5	FGSG_05145	K12605|1|1e-17|89.7|fgr:FG05100.1|CCR4-NOT transcription complex subunit 2;K14640|2|2e-12|72.8|ani:AN2812.2|solute carrier family 20 (sodium-dependent phosphate transporter)
siRNA-t0120117	CCAACTGCGACCCATGCTTTCG	22	5	FgHV1gp2	-
siRNA-t0106765	CCAAGCCAAGCGGGATCATCCAT	23	5	FgHV1gp1	-
siRNA-t0112609	CCAAGCTCGTCCCTCGTGCAA	21	5	FGSG_02315	K01932|1|0.0|2455|afm:AFUA_1G10380|[EC:6.3.2.-]
siRNA-t0107406	CCAAGTCTGCTGGGGCCAAA	20	5	FGSG_13921	-
siRNA-t0120029	CCAGAAACCCATCCCACTCGTGAA	24	5	FgHV1gp2	-
siRNA-t0123035	CCAGAAACCCATCCCACTCGTGAAC	25	5	FgHV1gp2	-
siRNA-t0122035	CCAGACCCATTCGTGTTCCCGA	22	5	FgHV1gp2	-
siRNA-t0108607	CCAGATCCACCCGGATGACTTGGCT	25	5	FgHV1gp2	-
siRNA-t0113061	CCAGCTGCAGCAACTGTCGCCT	22	5	FGSG_06533	-
siRNA-t0109443	CCAGTATGGTTCGACACTGG	20	5	FGSG_05213	-
siRNA-t0114364	CCATACAATGCCGCCAACCA	20	5	FgHV1gp2	-
siRNA-t0116198	CCATGCAGCGGCAAATACAGGAC	23	5	FgHV1gp2	-
siRNA-t0107834	CCATGCTTTCGACGTACTTG	20	5	FGSG_09930	K14326|1|0.0|2138|fgr:FG09930.1|regulator of nonsense transcripts 1 [EC:3.6.4.-]
siRNA-t0122498	CCATTCACGAACGCTAAGAGGAA	23	5	FgHV1gp2	-
siRNA-t0118573	CCATTGCTGCATGTACTGTTC	21	5	FGSG_05795	-
siRNA-t0109881	CCCACTCTAGCAATGCATCACCAA	24	5	FgHV1gp1	-
siRNA-t0121227	CCCAGTGGCGCATTTCTTTCT	21	5	FgHV1gp2	-
siRNA-t0113005	CCCATTTGCGCACATATCTCCT	22	5	FgHV1gp2	-
siRNA-t0111180	CCCGTCGAAGACCAAAAATTT	21	5	FgHV1gp1	-
siRNA-t0115935	CCCGTCGAAGACCAAAAATTTCTT	24	5	FgHV1gp1	-
siRNA-t0118294	CCGAATACCATACTTTCCGA	20	5	FgHV1gp2	-
siRNA-t0115118	CCGACGGTACGTGGAAATCTT	21	5	FgHV1gp2	-
siRNA-t0121266	CCGACGTTTTGGCGAGACCG	20	5	FgHV1gp2	-
siRNA-t0120549	CCGCCAGAATCGAGCTAAACGAACT	25	5	FgHV1gp2	-
siRNA-t0115088	CCGCCTATGCTTGTCAGCCT	20	5	FgHV1gp2	-
siRNA-t0111452	CCGCGTTAGTGATTTGTGATTT	22	5	FgHV1gp2	-
siRNA-t0108808	CCGGCGTGCTGAGATCAAGT	20	5	FGSG_02554	-
siRNA-t0113373	CCTAGAGATCTCCGCAGAAGAA	22	5	FGSG_00352	K08064|1|2e-78|291|mgr:MGG_00354|nuclear transcription factor Y, alpha
siRNA-t0116853	CCTCAGGGTAGGCAAAGTTT	20	5	FGSG_05329	K03686|1|0.0|978|ani:AN6194.2|molecular chaperone DnaJ;K12200|2|2e-39|164|cpw:CPC735_002480|programmed cell death 6-interacting protein
siRNA-t0119362	CCTCCTTCGAACTAGATGGTG	21	5	FgHV1gp2	-
siRNA-t0120253	CGAATTCTGCCATAACCTCGT	21	5	FGSG_04291	K02999|1|0.0|3375|fgr:FG04291.1|DNA-directed RNA polymerase I subunit RPA1 [EC:2.7.7.6]
siRNA-t0112259	CGAATTGCAACGAGGGCCCAGT	22	5	FGSG_07277	K01976|1|3e-32|139|sce:YBR222C|[EC:6.-.-.-];K01895|4|2e-19|96.7|mgr:MGG_04590|acetyl-CoA synthetase [EC:6.2.1.1];K01897|5|2e-19|96.7|ncr:NCU06063|long-chain acyl-CoA synthetase [EC:6.2.1.3]
siRNA-t0118399	CGACGGGTTGGCCAAATCTCGGA	23	5	FgHV1gp2	-
siRNA-t0111369	CGGGTTGGCCAAATCTCGGAC	21	5	FgHV1gp2	-
siRNA-t0107616	CGTAACGTGACCAGCTGTTC	20	5	FGSG_13617	K00273|1|0.0|759|fgr:FG09652.1|D-amino-acid oxidase [EC:1.4.3.3]
siRNA-t0116042	CGTACCGTCGGTGTCGCCAT	20	5	FGSG_08320	K00495|1|4e-93|341|afm:AFUA_1G01690|[EC:1.14.14.-]
siRNA-t0122752	CGTCATGATCATCAGCACCGTA	22	5	FGSG_04291	K02999|1|0.0|3375|fgr:FG04291.1|DNA-directed RNA polymerase I subunit RPA1 [EC:2.7.7.6]
siRNA-t0112231	CGTCCCGCTGTTTCACTTGTAGGAG	25	5	FgHV1gp2	-
siRNA-t0111657	CGTCGAAAGCATGGGTCGCA	20	5	FGSG_08278	-
siRNA-t0112363	CGTGTGATTGGTCTCTTGCAGTGCA	25	5	FgHV1gp2	-
siRNA-t0122695	CGTTCTGTGAACACTAATATCGTCA	25	5	FgHV1gp2	-
siRNA-t0120405	CGTTGAAAATCAGAGAGCAGGAT	23	5	FgHV1gp2	-
siRNA-t0119716	CGTTGAATCGATCGTCATGGTGATA	25	5	FgHV1gp2	-
siRNA-t0116434	CGTTGATCTCTTATGCGCGG	20	5	FGSG_08591	-
siRNA-t0107843	CTATACTTGATCTCAGCACGCC	22	5	FgHV1gp2	-
siRNA-t0106575	CTCAAGGAGCTCAAAGGTATG	21	5	FGSG_11489	-
siRNA-t0113123	CTCAGCACGCCGGTTACCAAGGT	23	5	FgHV1gp2	-
siRNA-t0112287	CTCCGACAATGGCGCATACACGGA	24	5	FgHV1gp2	-
siRNA-t0122456	CTCCTTGAACTTGAGCATTTG	21	5	FGSG_11988	K05658|1|0.0|2439|fgr:FG01684.1|ATP-binding cassette, subfamily B (MDR/TAP), member 1
siRNA-t0116815	CTCTTGTTCTGGAACGTCAAA	21	5	FGSG_05697	K16055|1|0.0|1844|fgr:FG05697.1|trehalose 6-phosphate synthase/phosphatase [EC:2.4.1.15 3.1.3.12]
siRNA-t0119082	CTGATCTGGCTCGTGACTTTTCCCT	25	5	FgHV1gp2	-
siRNA-t0106289	CTTCGTTGAATCGATCGTCATG	22	5	FgHV1gp2	-
siRNA-t0109712	CTTCGTTGAATCGATCGTCATGGT	24	5	FgHV1gp2	-
siRNA-t0115964	CTTCGTTGAATCGATCGTCATGGTG	25	5	FgHV1gp2	-
siRNA-t0118277	CTTGAGACAGTTCCGCGTGTGAT	23	5	FgHV1gp2	-
siRNA-t0107625	CTTGATCTCAGCACGCCGGTTA	22	5	FGSG_00633	K10592|1|0.0|5185|fgr:FG00633.1|E3 ubiquitin-protein ligase HUWE1 [EC:6.3.2.19]
siRNA-t0122715	CTTGGTTCAAGTATCGTCACTTCTT	25	5	FgHV1gp2	-
siRNA-t0110154	CTTGTCAGTGTCAGGATCCGT	21	5	FgHV1gp2	-
siRNA-t0118489	CTTGTTCAGGAATCACGACTTT	22	5	FgHV1gp2	-
siRNA-t0107387	GAAACCCATCCCACTCGTGAACC	23	5	FgHV1gp2	-
siRNA-t0110857	GAAAGTTGGAGCCCTTCTTA	20	5	FGSG_01921	-
siRNA-t0122216	GAACACTAATATCGTCATTCTCTTC	25	5	FgHV1gp2	-
siRNA-t0106492	GAACATCAGGCATGTATGGGTC	22	5	FGSG_03750	K11244|1|7e-07|55.5|fgr:FG10787.1|cell wall integrity and stress response component
siRNA-t0114995	GAACCAGTTCGTTAACTATT	20	5	FGSG_03838	K00059|1|3e-15|81.6|fgr:FG03438.1|3-oxoacyl-[acyl-carrier protein] reductase [EC:1.1.1.100]
siRNA-t0118248	GAACCCTTCCACGATTGGCG	20	5	FGSG_05504	K02365|1|0.0|2227|fgr:FG05504.1|separase [EC:3.4.22.49]
siRNA-t0114812	GAACGCTAAGAGGAAAGGAAATAT	24	5	FgHV1gp2	-
siRNA-t0109985	GACCCCAGATCCACCCGGATGACT	24	5	FgHV1gp2	-
siRNA-t0123346	GATAACCTATACTTGATCTCAGCAC	25	5	FgHV1gp2	-
siRNA-t0117730	GATATTGAACCCTCCTTCGT	20	5	FgHV1gp2	-
siRNA-t0116360	GATCTCATCATCACCAGACTCTA	23	5	FGSG_01210	K12854|1|0.0|4128|fgr:FG01210.1|pre-mRNA-splicing helicase BRR2 [EC:3.6.4.13]
siRNA-t0121649	GATCTCTGACATATTCGGACT	21	5	FgHV1gp2	-
siRNA-t0112966	GATGTTTGCTTGGTTTCGGGACGC	24	5	FgHV1gp2	-
siRNA-t0108481	GCAACGAGGGCCCAGTCATGAAT	23	5	FgHV1gp2	-
siRNA-t0109439	GCACCGTACGGATCCACATGAGCT	24	5	FgHV1gp2	-
siRNA-t0122634	GCAGCGTGAACCAGATCATAACCCG	25	5	FgHV1gp2	-
siRNA-t0115033	GCAGGATCTCGTAAGCTGCT	20	5	FGSG_09857	K09506|1|0.0|927|fgr:FG09857.1|DnaJ homolog subfamily A member 5
siRNA-t0123225	GCAGTTGGTCCGCGAATTCTG	21	5	FgHV1gp2	-
siRNA-t0108061	GCATGTACTGTTCGGCAACAG	21	5	FgHV1gp2	-
siRNA-t0115224	GCATTCAGTAGTCGGGGTCTCCGA	24	5	FgHV1gp1	-
siRNA-t0112182	GCCAAAACGCGCCTCCAGTCCA	22	5	FgHV1gp2	-
siRNA-t0110596	GCCATCCTATAGATCGCGCGTAA	23	5	FgHV1gp2	-
siRNA-t0122067	GCCGCGGATAACCTATACTTGAT	23	5	FgHV1gp2	-
siRNA-t0121413	GCCTGATGTTCTTGTGCCTCCGA	23	5	FgHV1gp2	-
siRNA-t0112422	GCGACCCATGCTTTCGACGTACT	23	5	FgHV1gp2	-
siRNA-t0107757	GCGCTTCTGGATCAATGTCAGCA	23	5	FgHV1gp2	-

<table>
<tr><td>siRNA-t0115143</td><td>GCGGTCAAGGCAATGCCCAA</td><td>20</td><td>5</td><td>FGSG_05996</td><td>K00936|1|3e-61|238|afm:AFUA_6G10240|[EC:2.7.3.-];K11231|2|6e-39|163|afm:AFUA_2G00660|osomolarity two-component system, sensor histidine kinase SLN1 [EC:2.7.13.3];K02486|3|2e-38|161|spo:SPAC27E2.09|two-component system, unclassified family, sensor kinase [EC:2.7.13.3]</td></tr>
<tr><td>siRNA-t0114480</td><td>GCGGTCCATGGTGGGGTCAC</td><td>20</td><td>5</td><td>FGSG_11800</td><td>-</td></tr>
<tr><td>siRNA-t0107795</td><td>GCTGATCTGGCTCGTGACTTTT</td><td>22</td><td>5</td><td>FgHV1gp2</td><td>-</td></tr>
<tr><td>siRNA-t0108950</td><td>GCTGCTTGTCAGTGTCAGGAT</td><td>21</td><td>5</td><td>FGSG_02400</td><td>K01537|1|0.0|1155|pno:SNOG_08638|Ca2+-transporting ATPase [EC:3.6.3.8]</td></tr>
<tr><td>siRNA-t0123121</td><td>GCTGTAGTGTCTGAAGATCTC</td><td>21</td><td>5</td><td>FGSG_05705</td><td>K08157|1|4e-68|258|lel:LELG_05567|MFS transporter, DHA1 family, multidrug resistance protein</td></tr>
<tr><td>siRNA-t0118166</td><td>GCTTCCCATGACTGCTAGAAT</td><td>21</td><td>5</td><td>FGSG_09177</td><td>-</td></tr>
<tr><td>siRNA-t0122010</td><td>GCTTGGATGAGCGATATTGTGC</td><td>22</td><td>5</td><td>FGSG_10523</td><td>K01932|1|4e-178|626|afm:AFUA_6G12080|[EC:6.3.2.-]</td></tr>
<tr><td>siRNA-t0121820</td><td>GCTTTCGACGTACTTGGGTT</td><td>20</td><td>5</td><td>FGSG_00630</td><td>-</td></tr>
<tr><td>siRNA-t0117088</td><td>GGAAAAGTTCTCGCCAGCAATGCCC</td><td>25</td><td>5</td><td>FgHV1gp1</td><td>-</td></tr>
<tr><td>siRNA-t0116757</td><td>GGAACAATGAAGGCGTGTTTTT</td><td>22</td><td>5</td><td>FgHV1gp2</td><td>-</td></tr>
<tr><td>siRNA-t0116041</td><td>GGAACGCACGACAATTGTCT</td><td>20</td><td>5</td><td>FGSG_08389</td><td>-</td></tr>
<tr><td>siRNA-t0117213</td><td>GGAATAGCGGTTGTCTTATA</td><td>20</td><td>5</td><td>FgHV1gp2</td><td>-</td></tr>
<tr><td>siRNA-t0111345</td><td>GGAATGACGTTTGTAAAGACCA</td><td>22</td><td>5</td><td>FgHV1gp2</td><td>-</td></tr>
<tr><td>siRNA-t0113900</td><td>GGAATGACGTTTGTAAAGACCACT</td><td>24</td><td>5</td><td>FgHV1gp2</td><td>-</td></tr>
<tr><td>siRNA-t0107150</td><td>GGCATAATCCGGAAGCATGA</td><td>20</td><td>5</td><td>FGSG_06945</td><td>-</td></tr>
<tr><td>siRNA-t0109867</td><td>GGCCCTCAGTAGTCGACATG</td><td>20</td><td>5</td><td>FGSG_09197</td><td>K00021|1|0.0|2147|fgr:FG09197.1|hydroxymethylglutaryl-CoA reductase (NADPH) [EC:1.1.1.34]</td></tr>
<tr><td>siRNA-t0112791</td><td>GGCCTCCCACGTTCCGCCTCCT</td><td>22</td><td>5</td><td>FGSG_01190</td><td>-</td></tr>
<tr><td>siRNA-t0118032</td><td>GGCCTCTGACGTTGATCTCTTA</td><td>22</td><td>5</td><td>FgHV1gp2</td><td>-</td></tr>
<tr><td>siRNA-t0119648</td><td>GGCGAACGGGACCGACGTTTTGG</td><td>23</td><td>5</td><td>FgHV1gp2</td><td>-</td></tr>
<tr><td>siRNA-t0114626</td><td>GGCGTTTTTCTCGGGTCCTG</td><td>20</td><td>5</td><td>FGSG_03762</td><td>K01175|1|1e-18|94.0|afm:AFUA_4G12720|[EC:3.1.-.-]</td></tr>
<tr><td>siRNA-t0111126</td><td>GGCTCCTCCGACAATGGCGCA</td><td>21</td><td>5</td><td>FGSG_10215</td><td>K15289|1|2e-09|62.8|yli:YALI0E22803g|solute carrier family 35, member F5;K15303|2|4e-09|62.0|ani:AN0675.2|aflatoxin B1 aldehyde reductase</td></tr>
<tr><td>siRNA-t0120883</td><td>GGCTGGTAAGGACCCCAACAC</td><td>21</td><td>5</td><td>FGSG_08215</td><td>K00777|1|2e-06|54.3|afm:AFUA_7G07100|[EC:2.4.2.-]</td></tr>
<tr><td>siRNA-t0115928</td><td>GGGGTCAGCCATTTCGAATCTGCCC</td><td>25</td><td>5</td><td>FgHV1gp2</td><td>-</td></tr>
<tr><td>siRNA-t0111910</td><td>GGTGTCAAGATTGGATTTCTT</td><td>21</td><td>5</td><td>FGSG_01977</td><td>-</td></tr>
<tr><td>siRNA-t0119867</td><td>GGTGTGTATCCAATGAGTTGTGG</td><td>23</td><td>5</td><td>FgHV1gp2</td><td>-</td></tr>
<tr><td>siRNA-t0106423</td><td>GGTGTGTATCCAATGAGTTGTGGG</td><td>24</td><td>5</td><td>FgHV1gp2</td><td>-</td></tr>
<tr><td>siRNA-t0115065</td><td>GGTGTGTATCCAATGAGTTGTGGGA</td><td>25</td><td>5</td><td>FgHV1gp2</td><td>-</td></tr>
<tr><td>siRNA-t0120923</td><td>GGTTCATTGCGATGTAATCTTGAA</td><td>24</td><td>5</td><td>FgHV1gp2</td><td>-</td></tr>
<tr><td>siRNA-t0111882</td><td>GGTTCTTGGAAAGTTGGAGCCCTT</td><td>24</td><td>5</td><td>FgHV1gp2</td><td>-</td></tr>
<tr><td>siRNA-t0115100</td><td>GGTTGGCACAACGATGAGCA</td><td>20</td><td>5</td><td>FgHV1gp2</td><td>-</td></tr>
<tr><td>siRNA-t0109441</td><td>GTACCATTGCTGCATGTACTGTTC</td><td>24</td><td>5</td><td>FgHV1gp2</td><td>-</td></tr>
<tr><td>siRNA-t0110648</td><td>GTACCGTCGGTGTCGCCATTG</td><td>21</td><td>5</td><td>FGSG_00143</td><td>K01187|1|6e-35|149|pno:SNOG_11356|alpha-glucosidase [EC:3.2.1.20];K05546|2|2e-33|144|ppa:PAS_chr2-1_0778|alpha 1,3-glucosidase [EC:3.2.1.84]</td></tr>
<tr><td>siRNA-t0123284</td><td>GTAGAACAGACGTCTCTCACGCTC</td><td>24</td><td>5</td><td>FgHV1gp2</td><td>-</td></tr>
<tr><td>siRNA-t0113085</td><td>GTATGAACCCTTCCACGATTGGCG</td><td>24</td><td>5</td><td>FgHV1gp2</td><td>-</td></tr>
<tr><td>siRNA-t0115017</td><td>GTCATCGGGAATCATGTCGGT</td><td>21</td><td>5</td><td>FgHV1gp2</td><td>-</td></tr>
<tr><td>siRNA-t0114755</td><td>GTCGTACGTCATGGCACTCA</td><td>20</td><td>5</td><td>FGSG_06089</td><td>K08874|1|0.0|7852|fgr:FG06089.1|transformation/transcription domain-associated protein</td></tr>
<tr><td>siRNA-t0123159</td><td>GTCTGACTGTAGTGTGCCTT</td><td>20</td><td>5</td><td>FGSG_11659</td><td>K01932|1|0.0|1431|afm:AFUA_5G12730|[EC:6.3.2.-]</td></tr>
<tr><td>siRNA-t0121345</td><td>GTCTGACTGTAGTGTGCCTTGT</td><td>22</td><td>5</td><td>FGSG_11659</td><td>K01932|1|0.0|1431|afm:AFUA_5G12730|[EC:6.3.2.-]</td></tr>
<tr><td>siRNA-t0116039</td><td>GTCTGACTGTTGCTTGATGGTTGG</td><td>24</td><td>5</td><td>FgHV1gp2</td><td>-</td></tr>
<tr><td>siRNA-t0118821</td><td>GTGACGACGCTATGTGGAGT</td><td>20</td><td>5</td><td>FGSG_13413</td><td>K15285|1|0.0|656|fgr:FG08241.1|solute carrier family 35, member E3</td></tr>
<tr><td>siRNA-t0117536</td><td>GTGATTGTGGTCATTTGGTTTG</td><td>22</td><td>5</td><td>FgHV1gp2</td><td>-</td></tr>
<tr><td>siRNA-t0114465</td><td>GTGCTCATCGTTGTGCCAACC</td><td>21</td><td>5</td><td>FGSG_04332</td><td>-</td></tr>
<tr><td>siRNA-t0110434</td><td>GTGTCAAGCTTCTAACGACA</td><td>20</td><td>5</td><td>FGSG_06728</td><td>-</td></tr>
<tr><td>siRNA-t0109272</td><td>GTGTCTGACTGTTGCTTGATGGTTG</td><td>25</td><td>5</td><td>FgHV1gp2</td><td>-</td></tr>
<tr><td>siRNA-t0118476</td><td>GTGTGAAGCTTGGATGAGCGAT</td><td>22</td><td>5</td><td>FGSG_00324</td><td>-</td></tr>
<tr><td>siRNA-t0121500</td><td>GTTCATTTCGGCGAGTCTAATCTT</td><td>24</td><td>5</td><td>FgHV1gp2</td><td>-</td></tr>
<tr><td>siRNA-t0109824</td><td>GTTCTGTGAACACTAATATCGTCA</td><td>24</td><td>5</td><td>FgHV1gp2</td><td>-</td></tr>
<tr><td>siRNA-t0107812</td><td>GTTGGCACAACGATGAGCAC</td><td>20</td><td>5</td><td>FgHV1gp2</td><td>-</td></tr>
<tr><td>siRNA-t0106137</td><td>TAATATCGTCATTCTCTTCCA</td><td>21</td><td>5</td><td>FGSG_10105</td><td>K12864|1|8e-17|88.2|cne:CNH03260|beta-catenin-like protein 1</td></tr>
<tr><td>siRNA-t0121155</td><td>TAATTTGGCCCCAGCAGACTTGG</td><td>23</td><td>5</td><td>FgHV1gp2</td><td>-</td></tr>
<tr><td>siRNA-t0107624</td><td>TACATGCCTGATGTTCTTGTGC</td><td>22</td><td>5</td><td>FgHV1gp2</td><td>-</td></tr>
<tr><td>siRNA-t0112304</td><td>TACCCGTCGAAGACCAAAAATT</td><td>22</td><td>5</td><td>FgHV1gp1</td><td>-</td></tr>
<tr><td>siRNA-t0110847</td><td>TACGTGGAAATCTTCAGGCCCT</td><td>22</td><td>5</td><td>FgHV1gp2</td><td>-</td></tr>
<tr><td>siRNA-t0122045</td><td>TACTCTTCAGCGTAACGTGACCA</td><td>23</td><td>5</td><td>FgHV1gp2</td><td>-</td></tr>
<tr><td>siRNA-t0112096</td><td>TATACTTGATCTCAGCACGCC</td><td>21</td><td>5</td><td>FgHV1gp2</td><td>-</td></tr>
<tr><td>siRNA-t0108122</td><td>TATCGTACCGCAGTGATGGA</td><td>20</td><td>5</td><td>FGSG_08521</td><td>K07512|1|0.0|855|fgr:FG08521.1|mitochondrial trans-2-enoyl-CoA reductase [EC:1.3.1.38]</td></tr>
<tr><td>siRNA-t0111406</td><td>TCAAGTATCGTCACTTCTTC</td><td>20</td><td>5</td><td>FgHV1gp2</td><td>-</td></tr>
<tr><td>siRNA-t0117036</td><td>TCCAAACTGTTCATTTCGGCGAGT</td><td>24</td><td>5</td><td>FgHV1gp2</td><td>-</td></tr>
<tr><td>siRNA-t0119118</td><td>TCCAACTGCGACCCATGCTTTCG</td><td>23</td><td>5</td><td>FgHV1gp2</td><td>-</td></tr>
<tr><td>siRNA-t0116555</td><td>TCCAGATCTTATAGACAGGCC</td><td>21</td><td>5</td><td>FgHV1gp2</td><td>-</td></tr>
<tr><td>siRNA-t0116679</td><td>TCCCACTCTAGCAATGCATCACCA</td><td>24</td><td>5</td><td>FgHV1gp1</td><td>-</td></tr>
<tr><td>siRNA-t0110225</td><td>TCCGAAGGAACTCCACATAGCGTCG</td><td>25</td><td>5</td><td>FgHV1gp2</td><td>-</td></tr>
<tr><td>siRNA-t0115836</td><td>TCCGGGATCAGGTTCACAATTGCA</td><td>24</td><td>5</td><td>FgHV1gp2</td><td>-</td></tr>
<tr><td>siRNA-t0109292</td><td>TCGAAAGTGAAAGTGTCGTAGGAA</td><td>24</td><td>5</td><td>FgHV1gp1</td><td>-</td></tr>
<tr><td>siRNA-t0107686</td><td>TCGACGTACTTGGGTTCATA</td><td>20</td><td>5</td><td>FgHV1gp2</td><td>-</td></tr>
<tr><td>siRNA-t0111931</td><td>TCGGTAGTCATCGGGAATCATGTC</td><td>24</td><td>5</td><td>FgHV1gp2</td><td>-</td></tr>
<tr><td>siRNA-t0115424</td><td>TCTGGTACCGATATTGAACCC</td><td>21</td><td>5</td><td>FgHV1gp2</td><td>-</td></tr>
<tr><td>siRNA-t0122735</td><td>TCTTCGTCCAGTAGCCAATGA</td><td>21</td><td>5</td><td>FGSG_06448</td><td>-</td></tr>
<tr><td>siRNA-t0121931</td><td>TCTTGGAAAGTTGGAGCCCTTCTT</td><td>24</td><td>5</td><td>FgHV1gp2</td><td>-</td></tr>
<tr><td>siRNA-t0107276</td><td>TCTTGTTCTGGAACGTCAAAT</td><td>21</td><td>5</td><td>FGSG_06754</td><td>K06669|1|0.0|2432|fgr:FG06754.1|structural maintenance of chromosome 3 (chondroitin sulfate proteoglycan 6)</td></tr>
<tr><td>siRNA-t0106424</td><td>TCTTTCCTCTTGATGCGCACCA</td><td>22</td><td>5</td><td>FgHV1gp2</td><td>-</td></tr>
<tr><td>siRNA-t0106554</td><td>TGAAACCATCAGGACCCGAGA</td><td>21</td><td>5</td><td>FGSG_10402</td><td>-</td></tr>
<tr><td>siRNA-t0113730</td><td>TGAAAGTGTCGTAGGAAAGGAAAAG</td><td>25</td><td>5</td><td>FgHV1gp1</td><td>-</td></tr>
<tr><td>siRNA-t0110318</td><td>TGAACCCAAGTACGTCGAAA</td><td>20</td><td>5</td><td>FGSG_13824</td><td>K03380|1|1e-16|88.2|fgr:FG11194.1|phenol 2-monooxygenase [EC:1.14.13.7];K00777|2|6e-07|55.8|afm:AFUA_7G07100|[EC:2.4.2.-]</td></tr>
<tr><td>siRNA-t0113570</td><td>TGATACAAACCGGAATGCACTTTA</td><td>24</td><td>5</td><td>FgHV1gp2</td><td>-</td></tr>
<tr><td>siRNA-t0112750</td><td>TGATGCATTGCTAGAGTGGG</td><td>20</td><td>5</td><td>FGSG_07262</td><td>K14550|1|0.0|3445|fgr:FG07262.1|U3 small nucleolar RNA-associated protein 10</td></tr>
<tr><td>siRNA-t0108644</td><td>TGATTTCATGACCTTGAGACAGTT</td><td>24</td><td>5</td><td>FgHV1gp2</td><td>-</td></tr>
<tr><td>siRNA-t0110663</td><td>TGATTTGCGACGTGCTTGAAG</td><td>21</td><td>5</td><td>FGSG_08200</td><td>-</td></tr>
<tr><td>siRNA-t0121424</td><td>TGCAACGAGGGCCCAGTCATGAATT</td><td>25</td><td>5</td><td>FgHV1gp2</td><td>-</td></tr>
<tr><td>siRNA-t0109649</td><td>TGCACGAGGGACGAGCTTGGCCCCT</td><td>25</td><td>5</td><td>FgHV1gp2</td><td>-</td></tr>
<tr><td>siRNA-t0107506</td><td>TGCAGCGTGAACCAGATCAT</td><td>20</td><td>5</td><td>FGSG_03391</td><td>K01593|1|3e-37|155|nhe:NECHADRAFT_50494|aromatic-L-amino-acid decarboxylase [EC:4.1.1.28]</td></tr>
<tr><td>siRNA-t0114621</td><td>TGCCGCGGATAACCTATACTT</td><td>21</td><td>5</td><td>FgHV1gp2</td><td>-</td></tr>
<tr><td>siRNA-t0114091</td><td>TGCGAATGTTTCACTCCTCCT</td><td>21</td><td>5</td><td>FgHV1gp2</td><td>-</td></tr>
<tr><td>siRNA-t0110333</td><td>TGCGACCCATGCTTTCGACGTAC</td><td>23</td><td>5</td><td>FgHV1gp2</td><td>-</td></tr>
<tr><td>siRNA-t0108574</td><td>TGCGACGTGCTTGAAGGCGAAGT</td><td>23</td><td>5</td><td>FgHV1gp2</td><td>-</td></tr>
<tr><td>siRNA-t0118527</td><td>TGCTCTGCCTCGGTCAGACCCCA</td><td>23</td><td>5</td><td>FgHV1gp2</td><td>-</td></tr>
<tr><td>siRNA-t0120777</td><td>TGCTGCCGCGGATAACCTATA</td><td>21</td><td>5</td><td>FgHV1gp2</td><td>-</td></tr>
<tr><td>siRNA-t0111635</td><td>TGCTTTCGACGTACTTGGGTTC</td><td>22</td><td>5</td><td>FgHV1gp2</td><td>-</td></tr>
<tr><td>siRNA-t0120546</td><td>TGCTTTTTGCCTGGGAGACA</td><td>20</td><td>5</td><td>FGSG_08637</td><td>K15163|1|0.0|2805|fgr:FG08637.1|mediator of RNA polymerase II transcription subunit 12, fungi type</td></tr>
<tr><td>siRNA-t0122047</td><td>TGGCCGGCAGTCCTAGCAAGTG</td><td>22</td><td>5</td><td>FgHV1gp2</td><td>-</td></tr>
</table>

siRNA-t0111832	TGGGATGGGTTTCTGGTTCCAA	22	5	FGSG_13796	K14338|1|8e-09|60.8|fgr:FG01972.1|cytochrome P450 / NADPH-cytochrome P450 reductase [EC:1.14.14.1 1.6.2.4];K00517|2|4e-08|58.9|afm:AFUA_3G14760|[EC:1.14.-.-];K00493|4|1e-07|56.6|pic:PICST_35590|unspecific monooxygenase [EC:1.14.14.1]
siRNA-t0114546	TGGGTCTTCTGACGAATTTGGACA	24	5	FgHV1gp2	-
siRNA-t0113448	TGGTGAGGTATGACGTGTTCATT	23	5	FgHV1gp2	-
siRNA-t0115432	TGGTGAGGTATGACGTGTTCATTAT	25	5	FgHV1gp2	-
siRNA-t0120890	TGTAAGTGACGAGGCCTCTGACGTT	25	5	FgHV1gp2	-
siRNA-t0119072	TGTACTTGCCGCGCGTCTCACT	22	5	FgHV1gp2	-
siRNA-t0113789	TGTAGGAGTGGCAGTGTAAT	20	5	FgHV1gp2	-
siRNA-t0113174	TGTGTCGCGTCTACCTTGTTCAGGA	25	5	FgHV1gp2	-
siRNA-t0117403	TGTTATAAGGCTTGTCGTCGGGAG	24	5	FgHV1gp2	-
siRNA-t0108378	TGTTCATTTCGGCGAGTCTAA	21	5	FgHV1gp2	-
siRNA-t0120618	TTCCAACCTGTTGTCAAGCTT	21	5	FGSG_03264	K00517|1|3e-44|178|afm:AFUA_3G03450|[EC:1.14.-.-];K07824|3|1e-42|172|pno:SNOG_04264|benzoate 4-monooxygenase [EC:1.14.13.12];K07047|4|1e-41|169|fgr:FG08008.1|
siRNA-t0121110	TTCCAGAACAAGAGACTTGAA	21	5	FGSG_10661	K08141|1|0.0|858|ncr:NCU06522|MFS transporter, SP family, general alpha glucoside:H+ symporter
siRNA-t0121320	TTCCAGTGTCGAACCATACTGGAC	24	5	FgHV1gp2	-
siRNA-t0108556	TTCGTTGAATCGATCGTCAT	20	5	FGSG_11372	K00777|1|5e-21|103|afm:AFUA_7G06290|[EC:2.4.2.-];K12823|4|1e-10|69.7|ure:UREG_02211|ATP-dependent RNA helicase DDX5/DBP2 [EC:3.6.4.13]
siRNA-t0113472	TTCTCCAGTAGCATGTTCTTCGTG	24	5	FgHV1gp2	-
siRNA-t0110167	TTCTGAAATTTGATGTTGTAGA	22	5	FgHV1gp2	-
siRNA-t0115915	TTCTGCCATAACCTCGTTGGGACCT	25	5	FgHV1gp2	-
siRNA-t0122721	TTGAACCACAGTCGAATGAA	20	5	FgHV1gp2	-
siRNA-t0110665	TTGAATCGATCGTCATGGTGATA	23	5	FgHV1gp2	-
siRNA-t0111900	TTGATAGGGTCAGGAGTAGG	20	5	FGSG_02618	-
siRNA-t0109911	TTGCTGCATGTACTGTTCGGCAA	23	5	FgHV1gp2	-
siRNA-t0118790	TTGTGCTGCAAGATGGACTGGC	22	5	FgHV1gp2	-
siRNA-t0112838	TTGTTTCTCGTGGGATTGGGC	21	5	FgHV1gp2	-
siRNA-t0108178	TTTACAAGCTGGTCTGGGTGCT	22	5	FgHV1gp2	-
siRNA-t0107270	TTTATTAACCATGGATCTGAT	21	5	FgHV1gp1	-
siRNA-t0135005	AAACATAGACACTTCAACATG	21	4	FgHV1gp2	-
siRNA-t0134577	AAACCACACTCAGCAGGAACTG	22	4	FGSG_07104	K13338|1|0.0|2298|fgr:FG07104.1|peroxin-1
siRNA-t0143584	AAACCATCAGGACCCGAGAAAAACG	25	4	FgHV1gp2	-
siRNA-t0145892	AAACCCATCCCACTCGTGAACCTG	24	4	FgHV1gp2	-
siRNA-t0139789	AAAGATTAGGCAGACCATTC	20	4	FGSG_11505	-
siRNA-t0143803	AAAGTCACGAGCCAGATCAGC	21	4	FGSG_04598	K05393|1|7e-104|377|aor:AOR_1_1352054|chloride channel, other eukaryote
siRNA-t0149292	AAATACAGGACCGCGCGATGGCGC	24	4	FgHV1gp2	-
siRNA-t0126726	AAATGGAACAACGTGAGCCAG	21	4	FgHV1gp2	-
siRNA-t0138039	AACCACAAGCCACTGCAGTT	20	4	FgHV1gp2	-
siRNA-t0150279	AAGGATTAATCAATGTACCGTT	22	4	FgHV1gp2	-
siRNA-t0124723	AATACAGGACCGCGCGATGGCGCGC	25	4	FgHV1gp2	-
siRNA-t0134253	AATCAATGTACCGTTCGGCAA	21	4	FGSG_03931	K13336|1|2e-47|189|afv:AFLA_048740|peroxin-3;K03448|2|8e-40|164|ani:AN2663.2|MFS transporter, ACS family, pantothenate transporter
siRNA-t0141348	AATCATCATCGTAGTCATCGTCAGG	25	4	FGSG_07312	-
siRNA-t0148564	AATGATTTGCGACGTGCTTGAAGG	24	4	FgHV1gp2	-
siRNA-t0141385	AATGTTATCATGAGCGTTTG	20	4	FGSG_06438	K03380|1|2e-57|223|fgr:FG11194.1|phenol 2-monooxygenase [EC:1.14.13.7];K00540|2|5e-52|205|afm:AFUA_1G17630|[EC:1.-.-.-];K15418|3|3e-27|122|fgr:FG02396.1|zearalenone synthase, highly reducing iterative type I polyketide synthase;K01501|4|6e-24|112|ssl:SS1G_10951|nitrilase [EC:3.5.5.1];K00309|5|7e-07|55.1|afm:AFUA_6G14050|[EC:1.5.3.-]
siRNA-t0147338	ACAATTGCACATGGGACCATCA	22	4	FgHV1gp2	-
siRNA-t0127477	ACACCACATCACGCTCGGGG	20	4	FgHV1gp2	-
siRNA-t0140395	ACACGACGTACTTAGGGTTG	20	4	FgHV1gp2	-
siRNA-t0127506	ACATCCAGATCTTATAGACA	20	4	FGSG_13783	K01932|1|0.0|1526|afm:AFUA_5G12730|[EC:6.3.2.-]
siRNA-t0149957	ACATGATGGCTGATGCGACCGC	22	4	FGSG_13018	-
siRNA-t0131853	ACATGCAATTGAGTGCCATGACGT	24	4	FgHV1gp1	-
siRNA-t0134261	ACATGCCTGATGTTCTTGTGCCT	23	4	FgHV1gp2	-
siRNA-t0137325	ACATTTTTGAAGATCGTGAA	20	4	FGSG_02394	K01932|1|0.0|942|afm:AFUA_1G10380|[EC:6.3.2.-]
siRNA-t0139854	ACCAGAACCCAGCACCTTTC	20	4	FgHV1gp2	-
siRNA-t0134200	ACCAGACATTTTCGCGCGCTGT	22	4	FgHV1gp2	-
siRNA-t0136119	ACCATACAATGCCGCCAACCAC	22	4	FgHV1gp2	-
siRNA-t0135427	ACCCAGTTGCTGTATCTTGAA	21	4	FGSG_08583	K01386|1|5e-52|204|afm:AFUA_6G05350|[EC:3.4.23.-];K06005|2|2e-37|156|ctp:CTRG_02113|candidapepsin [EC:3.4.23.24];K06009|3|2e-36|152|vpo:Kpol_543p61|yapsin 1 [EC:3.4.23.41]
siRNA-t0142398	ACCCGTCGAAGACCAAAAATTTCTT	25	4	FgHV1gp1	-
siRNA-t0125827	ACCGAAGGAGACATTTGAGG	20	4	FGSG_11069	K13336|1|1e-52|206|afv:AFLA_048740|peroxin-3;K11369|2|1e-47|189|ani:AN3929.2|retrograde regulation protein 2;K15361|3|4e-31|135|cim:CIMG_09192|WD repeat-containing protein 48;K08192|4|1e-30|134|spo:SPBC1773.15|MFS transporter, ACS family, allantoate permease
siRNA-t0140432	ACGCCGCCAGAATCGAGCTAA	21	4	FgHV1gp2	-
siRNA-t0129540	ACGGAACCCATTGATCATTCTCCTC	25	4	FgHV1gp2	-
siRNA-t0144675	ACGGATCCACATGAGCTTCAG	21	4	FgHV1gp2	-
siRNA-t0132257	ACGGTACGTGGAAATCTTCA	20	4	FgHV1gp2	-
siRNA-t0126717	ACGGTACGTGGAAATCTTCAGGC	23	4	FgHV1gp2	-
siRNA-t0124053	ACGTACCCTGACGATGACTAC	21	4	FGSG_08056	-
siRNA-t0130537	ACGTACCGTCGGTGTCGCCAT	21	4	FGSG_08320	K00495|1|4e-93|341|afm:AFUA_1G01690|[EC:1.14.14.-]
siRNA-t0138838	ACGTCATGATCATCAGCACCGT	22	4	FgHV1gp2	-
siRNA-t0127693	ACTGTTGACGTCACCCTTTGGAGAC	25	4	FgHV1gp2	-
siRNA-t0146198	AGACATTTTCGCGCGCTGTACT	22	4	FGSG_02394	K01932|1|0.0|942|afm:AFUA_1G10380|[EC:6.3.2.-]
siRNA-t0141298	AGAGCGACTTGGAACAGGTC	20	4	FGSG_06785	-
siRNA-t0127869	AGAGGAAAGGAAATATCGGAAAA	23	4	FgHV1gp2	-
siRNA-t0136866	AGATCTCTAGGATGATGCCAA	21	4	FGSG_03014	K00844|1|2e-139|494|ncr:NCU02542|hexokinase [EC:2.7.1.1]
siRNA-t0147383	AGATCTTGCTGTAGTGTCTG	20	4	FGSG_10970	-
siRNA-t0129667	AGCAATGCAACAGACTTCTTCT	22	4	FgHV1gp2	-
siRNA-t0131605	AGCACTTGTTTGCCTGCATCGA	22	4	FGSG_08526	-
siRNA-t0136180	AGCAGACACTTGTTTCTCGTG	21	4	FgHV1gp2	-
siRNA-t0128212	AGGACTTGAAATCCTGGGCGCTAAC	25	4	FgHV1gp2	-
siRNA-t0126256	AGGATCGTTATTCTTCTTCTCA	22	4	FGSG_11029	K08197|1|1e-36|153|nhe:NECHADRAFT_101741|MFS transporter, SIT family, siderophore-iron:H+ symporter
siRNA-t0145973	AGGATCTCTGACATATTCGGAC	22	4	FgHV1gp2	-
siRNA-t0123569	AGGCGTCGACACGATGATACC	21	4	FgHV1gp2	-
siRNA-t0130143	AGGCGTCGACACGATGATACCC	22	4	FgHV1gp2	-
siRNA-t0137514	AGTCATCGGGAATCATGTCGG	21	4	FgHV1gp2	-
siRNA-t0135706	AGTCGAAAGCGGTCGCATCA	20	4	FGSG_13878	K01932|1|0.0|1450|afm:AFUA_5G12730|[EC:6.3.2.-]
siRNA-t0132308	AGTCTCCGAACGTGATCTCA	20	4	FgHV1gp2	-
siRNA-t0148641	AGTCTCCGAACGTGATCTCAT	21	4	FgHV1gp2	-
siRNA-t0130672	AGTCTTCGTCCAGTAGCCAAT	21	4	FgHV1gp2	-
siRNA-t0128593	AGTGAAGTCGAAAGTGAAAGTGT	23	4	FgHV1gp1	-
siRNA-t0126201	AGTGCCATGACGTACGACCCCGAAA	25	4	FgHV1gp1	-
siRNA-t0148879	AGTGTGTCGCGTCTACCTTGTTCA	24	4	FgHV1gp2	-
siRNA-t0139875	AGTTTGCATCCTCGACCCACT	21	4	FGSG_11200	K00688|1|1e-06|55.5|pno:SNOG_13460|starch phosphorylase [EC:2.4.1.1]
siRNA-t0142812	ATAATCGTTGAAAATCAGAGAGCA	24	4	FgHV1gp2	-

<table>
<tr><td></td><td></td><td></td><td></td><td></td><td></td></tr>
<tr><td>siRNA-t0142477</td><td>ATACCTTTGAGCTCCTTGAG</td><td>20</td><td>4</td><td>FGSG_06517</td><td>K01687|1|0.0|1239|fgr:FG06517.1|dihydroxy-acid dehydratase [EC:4.2.1.9]</td></tr>
<tr><td>siRNA-t0138340</td><td>ATACCTTTGAGCTCCTTGAGGAATG</td><td>25</td><td>4</td><td>FgHV1gp2</td><td>-</td></tr>
<tr><td>siRNA-t0140173</td><td>ATACTTTCCGAAGGAACTCCACAT</td><td>24</td><td>4</td><td>FgHV1gp2</td><td>-</td></tr>
<tr><td>siRNA-t0143394</td><td>ATAGATCGCGCGTAAGCTTA</td><td>20</td><td>4</td><td>FGSG_04986</td><td>-</td></tr>
<tr><td>siRNA-t0146834</td><td>ATGAGCGTTTGTGGAGCCAT</td><td>20</td><td>4</td><td>FGSG_09145</td><td>-</td></tr>
<tr><td>siRNA-t0131526</td><td>ATGATGGCACGTCGACAGGCG</td><td>21</td><td>4</td><td>FgHV1gp2</td><td>-</td></tr>
<tr><td>siRNA-t0146426</td><td>ATGATTTGCGACGTGCTTGA</td><td>20</td><td>4</td><td>FGSG_02169</td><td>-</td></tr>
<tr><td>siRNA-t0142567</td><td>ATGCCATCCTATAGATCGCG</td><td>20</td><td>4</td><td>FgHV1gp2</td><td>-</td></tr>
<tr><td>siRNA-t0131915</td><td>ATGCTCTGCCTCGGTCAGACCCC</td><td>23</td><td>4</td><td>FgHV1gp2</td><td>-</td></tr>
<tr><td>siRNA-t0125676</td><td>ATGTCCACACCGCGTTTCGCT</td><td>21</td><td>4</td><td>FgHV1gp1</td><td>-</td></tr>
<tr><td>siRNA-t0131003</td><td>ATGTTTCGCCGTTGAATGTT</td><td>20</td><td>4</td><td>FGSG_05482</td><td>K04518|1|1e-179|627|fgr:FG05482.1|prephenate dehydratase [EC:4.2.1.51]</td></tr>
<tr><td>siRNA-t0146825</td><td>ATTCGGACTCATCCCACATGA</td><td>21</td><td>4</td><td>FGSG_02113</td><td>K14338|1|2e-18|94.0|mgr:MGG_10879|cytochrome P450 / NADPH-cytochrome P450 reductase [EC:1.14.14.1 1.6.2.4];K00495|4|2e-17|90.5|uma:UM06473.1|[EC:1.14.14.-]</td></tr>
<tr><td>siRNA-t0144254</td><td>ATTCGTTACAATCGTGGACT</td><td>20</td><td>4</td><td>FgHV1gp2</td><td>-</td></tr>
<tr><td>siRNA-t0132763</td><td>ATTCTGCCATAACCTCGTTGG</td><td>21</td><td>4</td><td>FgHV1gp2</td><td>-</td></tr>
<tr><td>siRNA-t0125272</td><td>ATTGACGGGTCGCACGCACA</td><td>20</td><td>4</td><td>FGSG_09772</td><td>K06672|1|0.0|3586|fgr:FG09772.1|cohesin loading factor subunit SCC2</td></tr>
<tr><td>siRNA-t0125155</td><td>ATTTCGCACGGTCGTCGCAC</td><td>20</td><td>4</td><td>FGSG_09513</td><td>K08876|1|3e-10|66.6|smp:SMAC_07848|SCY1-like</td></tr>
<tr><td>siRNA-t0143405</td><td>ATTTGCGACGTGCTTGAAGGCG</td><td>22</td><td>4</td><td>FgHV1gp2</td><td>-</td></tr>
<tr><td>siRNA-t0146808</td><td>ATTTGGCCCCAGCAGACTTG</td><td>20</td><td>4</td><td>FGSG_07453</td><td>K01528|1|0.0|668|uma:UM02907.1|dynamin GTPase [EC:3.6.5.5]</td></tr>
<tr><td>siRNA-t0133947</td><td>CAAACATAGACACTTCAACATGGGG</td><td>25</td><td>4</td><td>FgHV1gp2</td><td>-</td></tr>
<tr><td>siRNA-t0137160</td><td>CAACGGTAGTACGTCGCGTG</td><td>20</td><td>4</td><td>FGSG_00188</td><td>K12870|1|2e-122|437|fgr:FG00188.1|pre-mRNA-splicing factor ISY1</td></tr>
<tr><td>siRNA-t0125798</td><td>CAACGGTTCTTGGAAAGTTGGAGC</td><td>24</td><td>4</td><td>FgHV1gp2</td><td>-</td></tr>
<tr><td>siRNA-t0140517</td><td>CAAGAAGTTTGCATCCTCGACCCA</td><td>24</td><td>4</td><td>FgHV1gp2</td><td>-</td></tr>
<tr><td>siRNA-t0127439</td><td>CAAGCAACAGTCAGACACGATA</td><td>22</td><td>4</td><td>FGSG_08389</td><td>-</td></tr>
<tr><td>siRNA-t0130570</td><td>CAAGCGTGACGAAGATCCCACTCTA</td><td>25</td><td>4</td><td>FgHV1gp1</td><td>-</td></tr>
<tr><td>siRNA-t0128420</td><td>CAAGTATCGTCACTTCTTCGTAGT</td><td>24</td><td>4</td><td>FgHV1gp2</td><td>-</td></tr>
<tr><td>siRNA-t0133139</td><td>CAATAATCGTTGAAAATCAGA</td><td>21</td><td>4</td><td>FgHV1gp2</td><td>-</td></tr>
<tr><td>siRNA-t0141177</td><td>CAATCAAGGTGACGACGCTA</td><td>20</td><td>4</td><td>FGSG_00307</td><td>-</td></tr>
<tr><td>siRNA-t0136091</td><td>CACAATTGCACATGGGACCATCA</td><td>23</td><td>4</td><td>FgHV1gp2</td><td>-</td></tr>
<tr><td>siRNA-t0142645</td><td>CACAGCTCCAATAATCGTTGAA</td><td>22</td><td>4</td><td>FgHV1gp2</td><td>-</td></tr>
<tr><td>siRNA-t0139766</td><td>CACATAGCGTCGTCACCTTG</td><td>20</td><td>4</td><td>FGSG_13835</td><td>-</td></tr>
<tr><td>siRNA-t0127552</td><td>CACCAGATGCGAAGCGAATAT</td><td>21</td><td>4</td><td>FGSG_03912</td><td>K09246|1|3e-07|55.8|lth:KLTH0E16500g|arginine metabolism regulation protein II</td></tr>
<tr><td>siRNA-t0142603</td><td>CACCGGCACAGGCAAATCTT</td><td>20</td><td>4</td><td>FGSG_04401</td><td>-</td></tr>
<tr><td>siRNA-t0130847</td><td>CACGCCGGTTACCAAGGTGT</td><td>20</td><td>4</td><td>FGSG_01497</td><td>K05767|1|0.0|3219|fgr:FG01497.1|IQ motif containing GTPase activating protein</td></tr>
<tr><td>siRNA-t0142421</td><td>CAGCAGACACTTGTTTCTCGT</td><td>21</td><td>4</td><td>FgHV1gp2</td><td>-</td></tr>
<tr><td>siRNA-t0127824</td><td>CAGCCATTTCGAATCTGCCC</td><td>20</td><td>4</td><td>FgHV1gp2</td><td>-</td></tr>
<tr><td>siRNA-t0147746</td><td>CAGCCATTTCGAATCTGCCCG</td><td>21</td><td>4</td><td>FgHV1gp2</td><td>-</td></tr>
<tr><td>siRNA-t0124359</td><td>CAGCTGCAGCAACTGTCGCCTG</td><td>22</td><td>4</td><td>FGSG_12712</td><td>K10884|1|0.0|1132|fgr:FG05317.1|ATP-dependent DNA helicase 2 subunit 1</td></tr>
<tr><td>siRNA-t0134493</td><td>CAGGCCTCATTGATTTTGTGTC</td><td>22</td><td>4</td><td>FgHV1gp2</td><td>-</td></tr>
<tr><td>siRNA-t0131232</td><td>CATCAGGACCCGAGAAAAACG</td><td>21</td><td>4</td><td>FgHV1gp2</td><td>-</td></tr>
<tr><td>siRNA-t0148371</td><td>CATTTGCGCACATATCTCCTTGA</td><td>23</td><td>4</td><td>FgHV1gp2</td><td>-</td></tr>
<tr><td>siRNA-t0145792</td><td>CCAACAGCGGAGATATTCAC</td><td>20</td><td>4</td><td>FGSG_00989</td><td>K06036|1|3e-170|597|afm:AFUA_4G03780|[EC:4.2.2.-]</td></tr>
<tr><td>siRNA-t0149585</td><td>CCAAGTAAGCAGAGCGTGTA</td><td>20</td><td>4</td><td>FgHV1gp2</td><td>-</td></tr>
<tr><td>siRNA-t0142604</td><td>CCAAGTAAGCAGAGCGTGTAGG</td><td>22</td><td>4</td><td>FgHV1gp2</td><td>-</td></tr>
<tr><td>siRNA-t0144553</td><td>CCAAGTACGTCGAAAGCATG</td><td>20</td><td>4</td><td>FGSG_04321</td><td>-</td></tr>
<tr><td>siRNA-t0142862</td><td>CCACAGTCGAATGAAATGGAA</td><td>21</td><td>4</td><td>FgHV1gp2</td><td>-</td></tr>
<tr><td>siRNA-t0138253</td><td>CCACATCACGCTCGGGGTCA</td><td>20</td><td>4</td><td>FGSG_04102</td><td>K00654|1|0.0|1186|fgr:FG04102.1|serine palmitoyltransferase [EC:2.3.1.50]</td></tr>
<tr><td>siRNA-t0136138</td><td>CCACGTACCGTCGGTGTCGCCA</td><td>22</td><td>4</td><td>FGSG_08320</td><td>K00495|1|4e-93|341|afm:AFUA_1G01690|[EC:1.14.14.-]</td></tr>
<tr><td>siRNA-t0144746</td><td>CCAGAAACCCATCCCACTCGTGA</td><td>23</td><td>4</td><td>FgHV1gp2</td><td>-</td></tr>
<tr><td>siRNA-t0131511</td><td>CCAGACATTTTCGCGCGCTGTA</td><td>22</td><td>4</td><td>FgHV1gp2</td><td>-</td></tr>
<tr><td>siRNA-t0143532</td><td>CCAGACGGCCAAATCCTCAG</td><td>20</td><td>4</td><td>FGSG_06161</td><td>K14850|1|0.0|818|fgr:FG06161.1|ribosomal RNA-processing protein 8 [EC:2.1.1.-]</td></tr>
<tr><td>siRNA-t0141995</td><td>CCATAACCTCGTTGGGACCTTTCCA</td><td>25</td><td>4</td><td>FgHV1gp2</td><td>-</td></tr>
<tr><td>siRNA-t0126259</td><td>CCATCTTGCAGCACAAACGGCC</td><td>22</td><td>4</td><td>FGSG_05511</td><td>K12041|1|0.0|1103|fgr:FG05511.1|solute carrier family 9 (sodium/hydrogen exchanger), member 6/7</td></tr>
<tr><td>siRNA-t0123990</td><td>CCATTCGTTACAATCGTGGACTT</td><td>23</td><td>4</td><td>FgHV1gp2</td><td>-</td></tr>
<tr><td>siRNA-t0142727</td><td>CCCACTCTAGCAATGCATCACC</td><td>22</td><td>4</td><td>FgHV1gp1</td><td>-</td></tr>
<tr><td>siRNA-t0147195</td><td>CCCAGTCATGAATTTTCGGT</td><td>20</td><td>4</td><td>FGSG_06089</td><td>K08874|1|0.0|7852|fgr:FG06089.1|transformation/transcription domain-associated protein</td></tr>
<tr><td>siRNA-t0141107</td><td>CCCATCCCACTCGTGAACCTGT</td><td>22</td><td>4</td><td>FgHV1gp2</td><td>-</td></tr>
<tr><td>siRNA-t0144130</td><td>CCCATTTCACCTCTCTTCGA</td><td>20</td><td>4</td><td>FGSG_13547</td><td>K15271|1|9e-42|169|ssl:SS1G_11638|ATP-dependent DNA helicase HFM1/MER3 [EC:3.6.4.12]</td></tr>
<tr><td>siRNA-t0130568</td><td>CCCTGACAATACCAAATATCC</td><td>21</td><td>4</td><td>FGSG_03735</td><td>K08711|1|0.0|1404|cne:CNB05770|ATP-binding cassette, subfamily G (WHITE), member 2, PDR</td></tr>
<tr><td>siRNA-t0126844</td><td>CCGAACGTGATCTCATCATCACCA</td><td>24</td><td>4</td><td>FgHV1gp2</td><td>-</td></tr>
<tr><td>siRNA-t0131672</td><td>CCGAAGAAAGACCACATCCAGATCT</td><td>25</td><td>4</td><td>FgHV1gp2</td><td>-</td></tr>
<tr><td>siRNA-t0148025</td><td>CCGACAATGGCGCATACACGG</td><td>21</td><td>4</td><td>FgHV1gp2</td><td>-</td></tr>
<tr><td>siRNA-t0124701</td><td>CCGACGGTACGTGGAAATCT</td><td>20</td><td>4</td><td>FgHV1gp2</td><td>-</td></tr>
<tr><td>siRNA-t0132993</td><td>CCGACGGTACGTGGAAATCTTC</td><td>22</td><td>4</td><td>FgHV1gp2</td><td>-</td></tr>
<tr><td>siRNA-t0136478</td><td>CCGCCTCCTTCGAACTAGATGG</td><td>22</td><td>4</td><td>FgHV1gp2</td><td>-</td></tr>
<tr><td>siRNA-t0143379</td><td>CCGCCTCCTTCGAACTAGATGGT</td><td>23</td><td>4</td><td>FgHV1gp2</td><td>-</td></tr>
<tr><td>siRNA-t0148721</td><td>CCGCGAATTCTGCCATAACCTCGT</td><td>24</td><td>4</td><td>FgHV1gp2</td><td>-</td></tr>
<tr><td>siRNA-t0141100</td><td>CCGGATACGTTCTGAGAAGTCTCCG</td><td>25</td><td>4</td><td>FgHV1gp2</td><td>-</td></tr>
<tr><td>siRNA-t0134633</td><td>CCGGGATCAGGTTCACAATTGCA</td><td>23</td><td>4</td><td>FgHV1gp2</td><td>-</td></tr>
<tr><td>siRNA-t0128189</td><td>CCGTAATCATCATCGTAGTCATCGT</td><td>25</td><td>4</td><td>FGSG_09900</td><td>-</td></tr>
<tr><td>siRNA-t0149265</td><td>CCTATACTTGATCTCAGCAC</td><td>20</td><td>4</td><td>FgHV1gp2</td><td>-</td></tr>
<tr><td>siRNA-t0142541</td><td>CCTATAGATCGCGCGTAAGCTTA</td><td>23</td><td>4</td><td>FgHV1gp2</td><td>-</td></tr>
<tr><td>siRNA-t0137125</td><td>CCTCGTTGGGACCTTTCCAAGAT</td><td>23</td><td>4</td><td>FgHV1gp2</td><td>-</td></tr>
<tr><td>siRNA-t0138650</td><td>CCTCTCCCTTCGTGAACTGCT</td><td>21</td><td>4</td><td>FgHV1gp2</td><td>-</td></tr>
<tr><td>siRNA-t0137549</td><td>CCTCTGTCTATGAGCACTTGTTT</td><td>23</td><td>4</td><td>FgHV1gp2</td><td>-</td></tr>
<tr><td>siRNA-t0144884</td><td>CCTGACAAATCTGGGTGGCGATGG</td><td>24</td><td>4</td><td>FgHV1gp2</td><td>-</td></tr>
<tr><td>siRNA-t0129829</td><td>CGAACCTCAAATCCAAAAGACGC</td><td>23</td><td>4</td><td>FgHV1gp2</td><td>-</td></tr>
<tr><td>siRNA-t0140403</td><td>CGAACCTCAAATCCAAAAGACGCGT</td><td>25</td><td>4</td><td>FgHV1gp2</td><td>-</td></tr>
<tr><td>siRNA-t0146632</td><td>CGAAGGAACTCCACATAGCGT</td><td>21</td><td>4</td><td>FgHV1gp2</td><td>-</td></tr>
<tr><td>siRNA-t0144899</td><td>CGACGACCGTGCGAAATTGTT</td><td>21</td><td>4</td><td>FgHV1gp2</td><td>-</td></tr>
<tr><td>siRNA-t0125052</td><td>CGCCTCCATGCATGTAGATCTT</td><td>22</td><td>4</td><td>FgHV1gp2</td><td>-</td></tr>
<tr><td>siRNA-t0144367</td><td>CGCCTTCCATGCTGCCGCGGA</td><td>21</td><td>4</td><td>FGSG_04587</td><td>K00777|1|7e-77|289|afm:AFUA_7G07100|[EC:2.4.2.-];K14855|4|5e-55|216|lbc:LACBIDRAFT_296068|ribosome assembly protein 4</td></tr>
<tr><td>siRNA-t0132783</td><td>CGCGCTGTACTGAAGGTGGTGTCAT</td><td>25</td><td>4</td><td>FgHV1gp2</td><td>-</td></tr>
<tr><td>siRNA-t0139680</td><td>CGGAATGCGTTCACACGATTCCC</td><td>23</td><td>4</td><td>FgHV1gp2</td><td>-</td></tr>
<tr><td>siRNA-t0125931</td><td>CGGAGATCTCTAGGATGATG</td><td>20</td><td>4</td><td>FGSG_10210</td><td>K01714|1|2e-63|241|pic:PICST_64442|dihydrodipicolinate synthase [EC:4.2.1.52];K01726|2|5e-35|147|afm:AFUA_3G11920|[EC:4.2.1.-]</td></tr>
<tr><td>siRNA-t0145879</td><td>CGGGAATCATGTCGGTGAAC</td><td>20</td><td>4</td><td>FGSG_09217</td><td>K08065|1|2e-08|57.0|cci:CC1G_09213|nuclear transcription Y subunit beta;K14679|2|7e-07|51.6|ppl:POSPLDRAFT_101360|tRNA ligase [EC:6.5.1.3]</td></tr>
<tr><td>siRNA-t0147509</td><td>CGGGCCACTCGACCTTGTCGCTG</td><td>23</td><td>4</td><td>FgHV1gp2</td><td>-</td></tr>
<tr><td>siRNA-t0148439</td><td>CGGTAACATGCAATTGAGTGCC</td><td>22</td><td>4</td><td>FgHV1gp1</td><td>-</td></tr>
<tr><td>siRNA-t0125828</td><td>CGGTCCATGGTGGGGTCACA</td><td>20</td><td>4</td><td>FGSG_11800</td><td>-</td></tr>
<tr><td>siRNA-t0148426</td><td>CGTAATCATCATCGTAGTCATCGTC</td><td>25</td><td>4</td><td>FGSG_09900</td><td>-</td></tr>
<tr><td>siRNA-t0124610</td><td>CGTACCGTCGGTGTCGCCATT</td><td>21</td><td>4</td><td>FGSG_04985</td><td>-</td></tr>
<tr><td>siRNA-t0126806</td><td>CGTAGATGTGATGCCTTGTCTA</td><td>22</td><td>4</td><td>FgHV1gp2</td><td>-</td></tr>
<tr><td>siRNA-t0136570</td><td>CGTCATACATTGCACGAGGGAC</td><td>22</td><td>4</td><td>FgHV1gp2</td><td>-</td></tr>
<tr><td>siRNA-t0137317</td><td>CGTCTTCGATGGATGCTGCGAA</td><td>22</td><td>4</td><td>FgHV1gp2</td><td>-</td></tr>
<tr><td>siRNA-t0146332</td><td>CGTGATAAATGAACGTCTGA</td><td>20</td><td>4</td><td>FgHV1gp2</td><td>-</td></tr>
</table>

<table>
<tr><td></td><td></td><td></td><td></td><td></td><td></td></tr>
<tr><td>siRNA-t0133182</td><td>CGTGGAAATCTTCAGGCCCTGG</td><td>22</td><td>4</td><td>FgHV1gp2</td><td>-</td></tr>
<tr><td>siRNA-t0140589</td><td>CGTGTTTTTCAATGATGGCAC</td><td>21</td><td>4</td><td>FgHV1gp2</td><td>-</td></tr>
<tr><td>siRNA-t0128598</td><td>CGTTAGAAGCTTGACACGAA</td><td>20</td><td>4</td><td>FGSG_04030</td><td>-</td></tr>
<tr><td>siRNA-t0138884</td><td>CGTTAGAAGCTTGACACGAATGA</td><td>23</td><td>4</td><td>FgHV1gp2</td><td>-</td></tr>
<tr><td>siRNA-t0148535</td><td>CTCAGCACGCCGGTTACCAAGG</td><td>22</td><td>4</td><td>FgHV1gp2</td><td>-</td></tr>
<tr><td>siRNA-t0127774</td><td>CTCAGCAGGAACTGGTCCTAA</td><td>21</td><td>4</td><td>FGSG_02329</td><td>-</td></tr>
<tr><td>siRNA-t0142163</td><td>CTCCGCAGAAGAATTAGCCTTTGA</td><td>24</td><td>4</td><td>FgHV1gp2</td><td>-</td></tr>
<tr><td>siRNA-t0139608</td><td>CTCGATCGCATATCCTTCGGT</td><td>21</td><td>4</td><td>FgHV1gp2</td><td>-</td></tr>
<tr><td>siRNA-t0126191</td><td>CTCGTGACTTTTCCCTCACAGGCT</td><td>24</td><td>4</td><td>FgHV1gp2</td><td>-</td></tr>
<tr><td>siRNA-t0133841</td><td>CTCTGGTGGTTCATTGCGATG</td><td>21</td><td>4</td><td>FGSG_05512</td><td>K11397|1|0.0|2685|fgr:FG05512.1|chromatin modification-related protein VID21</td></tr>
<tr><td>siRNA-t0138709</td><td>CTCTGTCTATGAGCACTTGT</td><td>20</td><td>4</td><td>FGSG_09118</td><td>K01183|1|0.0|1040|ssl:SS1G_00773|chitinase [EC:3.2.1.14]</td></tr>
<tr><td>siRNA-t0143220</td><td>CTGACATATTCGGACTCATCCC</td><td>22</td><td>4</td><td>FgHV1gp2</td><td>-</td></tr>
<tr><td>siRNA-t0138492</td><td>CTGACGATGACTACGATGATG</td><td>21</td><td>4</td><td>FGSG_06562</td><td>-</td></tr>
<tr><td>siRNA-t0133931</td><td>CTGCCATAACCTCGTTGGGACCTT</td><td>24</td><td>4</td><td>FgHV1gp2</td><td>-</td></tr>
<tr><td>siRNA-t0136347</td><td>CTGCCTCGGTCAGACCCCAGATC</td><td>23</td><td>4</td><td>FgHV1gp2</td><td>-</td></tr>
<tr><td>siRNA-t0148826</td><td>CTGCGGTACGATATTTGAGTA</td><td>21</td><td>4</td><td>FGSG_11234</td><td>K15505|1|7e-25|115|ssl:SS1G_03212|DNA repair protein RAD5 [EC:3.6.4.-];K15711|5|2e-21|103|tve:TRV_04890|SWI/SNF-related matrix-associated actin-dependent regulator of chromatin subfamily A3 [EC:3.6.4.- 6.3.2.19]</td></tr>
<tr><td>siRNA-t0141773</td><td>CTGCTGAGTGTGGTTTGAAAACAT</td><td>24</td><td>4</td><td>FgHV1gp2</td><td>-</td></tr>
<tr><td>siRNA-t0136253</td><td>CTGCTTGTCAGTGTCAGGATCCGT</td><td>24</td><td>4</td><td>FgHV1gp2</td><td>-</td></tr>
<tr><td>siRNA-t0127121</td><td>CTGGAACGTCAAATCAAGTTGG</td><td>22</td><td>4</td><td>FgHV1gp2</td><td>-</td></tr>
<tr><td>siRNA-t0145854</td><td>CTGGATCAATGTCAGCAGTGTT</td><td>22</td><td>4</td><td>FgHV1gp2</td><td>-</td></tr>
<tr><td>siRNA-t0132348</td><td>CTTCGTCACGCTTGCATTGCT</td><td>21</td><td>4</td><td>FGSG_00633</td><td>K10592|1|0.0|5185|fgr:FG00633.1|E3 ubiquitin-protein ligase HUWE1 [EC:6.3.2.19]</td></tr>
<tr><td>siRNA-t0137022</td><td>CTTCGTCCAGTAGCCAATGAGAA</td><td>23</td><td>4</td><td>FgHV1gp2</td><td>-</td></tr>
<tr><td>siRNA-t0134262</td><td>CTTCTGACGAATTTGGACAAGT</td><td>22</td><td>4</td><td>FGSG_11537</td><td>K00681|1|3e-08|57.8|aor:AOR_1_316174|gamma-glutamyltranspeptidase [EC:2.3.2.2]</td></tr>
<tr><td>siRNA-t0124409</td><td>CTTGGTTCAAGTATCGTCACTTCT</td><td>24</td><td>4</td><td>FgHV1gp2</td><td>-</td></tr>
<tr><td>siRNA-t0130278</td><td>CTTGTCAGTGTCAGGATCCGTCCAG</td><td>25</td><td>4</td><td>FgHV1gp2</td><td>-</td></tr>
<tr><td>siRNA-t0133735</td><td>CTTTACAAGCTGGTCTGGGTGCTG</td><td>24</td><td>4</td><td>FgHV1gp2</td><td>-</td></tr>
<tr><td>siRNA-t0149215</td><td>GAAACCATCAGGACCCGAGA</td><td>20</td><td>4</td><td>FGSG_07934</td><td>-</td></tr>
<tr><td>siRNA-t0128623</td><td>GAAATGGAACAACGTGAGCCA</td><td>21</td><td>4</td><td>FgHV1gp2</td><td>-</td></tr>
<tr><td>siRNA-t0138206</td><td>GAACCACAACGATAGTATGAACCCT</td><td>25</td><td>4</td><td>FgHV1gp2</td><td>-</td></tr>
<tr><td>siRNA-t0127897</td><td>GAACCAGATCATAACCCGCA</td><td>20</td><td>4</td><td>FgHV1gp2</td><td>-</td></tr>
<tr><td>siRNA-t0150142</td><td>GAACCTCCGATGAATCCTCTTCC</td><td>23</td><td>4</td><td>FgHV1gp2</td><td>-</td></tr>
<tr><td>siRNA-t0138165</td><td>GAACGCTGAAATCGAGGGTGTTG</td><td>23</td><td>4</td><td>FgHV1gp2</td><td>-</td></tr>
<tr><td>siRNA-t0136655</td><td>GACAATGGCGCATACACGGAA</td><td>21</td><td>4</td><td>FgHV1gp2</td><td>-</td></tr>
<tr><td>siRNA-t0132285</td><td>GACAGAAGTTTTGTGGTACA</td><td>20</td><td>4</td><td>FGSG_08662</td><td>K06657|1|7e-08|58.5|lel:LELG_00705|G1/S-specific cyclin PLC2</td></tr>
<tr><td>siRNA-t0146417</td><td>GACAGATAGGTCGCAGGCGC</td><td>20</td><td>4</td><td>FGSG_11146</td><td>K11987|1|1e-83|311|aor:AOR_1_1372154|prostaglandin-endoperoxide synthase 2 [EC:1.14.99.1]</td></tr>
<tr><td>siRNA-t0126919</td><td>GATAAATGAACGTCTGACTGTAG</td><td>23</td><td>4</td><td>FgHV1gp2</td><td>-</td></tr>
<tr><td>siRNA-t0124817</td><td>GATACTCTTCAGCGTAACGT</td><td>20</td><td>4</td><td>FGSG_08791</td><td>K11215|1|2e-12|74.3|cnb:CNBD0580|transcription factor STE12</td></tr>
<tr><td>siRNA-t0148812</td><td>GATCATGACGTGGAGAAGAA</td><td>20</td><td>4</td><td>FGSG_07536</td><td>K03386|1|2e-83|307|ang:ANI_1_984094|peroxiredoxin (alkyl hydroperoxide reductase subunit C) [EC:1.11.1.15]</td></tr>
<tr><td>siRNA-t0135828</td><td>GATCCCACTCTAGCAATGCAT</td><td>21</td><td>4</td><td>FgHV1gp1</td><td>-</td></tr>
<tr><td>siRNA-t0135529</td><td>GATCGGTGGCACACGCATAA</td><td>20</td><td>4</td><td>FGSG_13987</td><td>-</td></tr>
<tr><td>siRNA-t0137780</td><td>GATCTCAGAATGGTCACCGTC</td><td>21</td><td>4</td><td>FGSG_05023</td><td>K00006|1|0.0|869|fgr:FG05023.1|glycerol-3-phosphate dehydrogenase (NAD+) [EC:1.1.1.8]</td></tr>
<tr><td>siRNA-t0149415</td><td>GATCTCTTATGCGCGGGCCT</td><td>20</td><td>4</td><td>FGSG_00345</td><td>K08192|1|3e-63|242|dha:DEHA2C12276g|MFS transporter, ACS family, allantoate permease</td></tr>
<tr><td>siRNA-t0128043</td><td>GATCTGGCTCGTGACTTTTCCCTC</td><td>24</td><td>4</td><td>FgHV1gp2</td><td>-</td></tr>
<tr><td>siRNA-t0129443</td><td>GATGGTTGGCACAACGATGAGCAC</td><td>24</td><td>4</td><td>FgHV1gp2</td><td>-</td></tr>
<tr><td>siRNA-t0140098</td><td>GATGTTTGCTTGGTTTCGGGACG</td><td>23</td><td>4</td><td>FGSG_09801</td><td>K03164|1|0.0|2817|fgr:FG09801.1|DNA topoisomerase II [EC:5.99.1.3]</td></tr>
<tr><td>siRNA-t0125923</td><td>GCAACAGACTTCTTCTCTGC</td><td>20</td><td>4</td><td>FGSG_06563</td><td>K00521|1|4e-62|238|cpw:CPC735_011850|ferric-chelate reductase [EC:1.16.1.7];K05920|2|7e-22|105|afm:AFUA_6G02170|[EC:1.16.1.-]</td></tr>
<tr><td>siRNA-t0147660</td><td>GCAATGCAACAGACTTCTTCTCT</td><td>23</td><td>4</td><td>FgHV1gp2</td><td>-</td></tr>
<tr><td>siRNA-t0132804</td><td>GCAATGGTACCATCACTCAGGA</td><td>22</td><td>4</td><td>FGSG_02315</td><td>K01932|1|0.0|2455|afm:AFUA_1G10380|[EC:6.3.2.-]</td></tr>
<tr><td>siRNA-t0125807</td><td>GCACCTTTCCCTATTTTCTTCGGT</td><td>24</td><td>4</td><td>FgHV1gp2</td><td>-</td></tr>
<tr><td>siRNA-t0136080</td><td>GCACGAGGGACGAGCTTGGCCCCT</td><td>24</td><td>4</td><td>FgHV1gp2</td><td>-</td></tr>
<tr><td>siRNA-t0128364</td><td>GCAGTCCTAGCAAGTGAAACT</td><td>21</td><td>4</td><td>FGSG_02676</td><td>-</td></tr>
<tr><td>siRNA-t0139935</td><td>GCATACACGGAACCCATTGA</td><td>20</td><td>4</td><td>FGSG_08294</td><td>-</td></tr>
<tr><td>siRNA-t0142788</td><td>GCATGCTTTGGTTGAGACGCAT</td><td>22</td><td>4</td><td>FgHV1gp2</td><td>-</td></tr>
<tr><td>siRNA-t0123504</td><td>GCATGGCCGGCAGTCCTAGC</td><td>20</td><td>4</td><td>FgHV1gp2</td><td>-</td></tr>
<tr><td>siRNA-t0137685</td><td>GCATTTGCCGCACAGCTCCA</td><td>20</td><td>4</td><td>FgHV1gp2</td><td>-</td></tr>
<tr><td>siRNA-t0138316</td><td>GCCATCCTTGCGTGGTCGTGTG</td><td>22</td><td>4</td><td>FGSG_00671</td><td>K02949|1|8e-88|320|fgr:FG00671.1|small subunit ribosomal protein S11e</td></tr>
<tr><td>siRNA-t0128864</td><td>GCCATCGCGCGGTCCTGTATT</td><td>21</td><td>4</td><td>FGSG_04913</td><td>K05349|1|0.0|1664|fgr:FG04913.1|beta-glucosidase [EC:3.2.1.21]</td></tr>
<tr><td>siRNA-t0125424</td><td>GCCGAATGAAAGCCCAGTGGCGCA</td><td>24</td><td>4</td><td>FgHV1gp2</td><td>-</td></tr>
<tr><td>siRNA-t0143531</td><td>GCCTCTCCCTTCGTGAACTGCTG</td><td>23</td><td>4</td><td>FgHV1gp2</td><td>-</td></tr>
<tr><td>siRNA-t0149971</td><td>GCCTGATGTTCTTGTGCCTCCGAG</td><td>24</td><td>4</td><td>FgHV1gp2</td><td>-</td></tr>
<tr><td>siRNA-t0136953</td><td>GCGAATGTTTCACTCCTCCTTCCGT</td><td>25</td><td>4</td><td>FgHV1gp2</td><td>-</td></tr>
<tr><td>siRNA-t0130428</td><td>GCGAGACCGATGATTTCCATCCCC</td><td>24</td><td>4</td><td>FgHV1gp2</td><td>-</td></tr>
<tr><td>siRNA-t0140770</td><td>GCGCCTTCCATGCTGCCGCGG</td><td>21</td><td>4</td><td>FGSG_04587</td><td>K00777|1|7e-77|289|afm:AFUA_7G07100|[EC:2.4.2.-];K14855|4|5e-55|216|lbc:LACBIDRAFT_296068|ribosome assembly protein 4</td></tr>
<tr><td>siRNA-t0143404</td><td>GCGCCTTCCATGCTGCCGCGGATA</td><td>24</td><td>4</td><td>FGSG_04587</td><td>K00777|1|7e-77|289|afm:AFUA_7G07100|[EC:2.4.2.-];K14855|4|5e-55|216|lbc:LACBIDRAFT_296068|ribosome assembly protein 4</td></tr>
<tr><td>siRNA-t0138523</td><td>GCGCTTCTGGATCAATGTCA</td><td>20</td><td>4</td><td>FGSG_08541</td><td>K15134|1|0.0|1199|fgr:FG08541.1|mediator of RNA polymerase II transcription subunit 17, fungi type</td></tr>
<tr><td>siRNA-t0132650</td><td>GCGGTCATAAGTGACAAAAGCA</td><td>22</td><td>4</td><td>FgHV1gp2</td><td>-</td></tr>
<tr><td>siRNA-t0137407</td><td>GCTGAGTGTGGTTTGAAAAC</td><td>20</td><td>4</td><td>FGSG_10263</td><td>-</td></tr>
<tr><td>siRNA-t0145194</td><td>GCTGCATGTACTGTTCGGCAAC</td><td>22</td><td>4</td><td>FGSG_01434</td><td>-</td></tr>
<tr><td>siRNA-t0130807</td><td>GCTGCCGCGGATAACCTATAC</td><td>21</td><td>4</td><td>FgHV1gp2</td><td>-</td></tr>
<tr><td>siRNA-t0143894</td><td>GCTGCTTGTCAGTGTCAGGATCC</td><td>23</td><td>4</td><td>FgHV1gp2</td><td>-</td></tr>
<tr><td>siRNA-t0134991</td><td>GCTTGACAACAGGTTGGAATCTG</td><td>23</td><td>4</td><td>FgHV1gp2</td><td>-</td></tr>
<tr><td>siRNA-t0141247</td><td>GCTTGACAACAGGTTGGAATCTGC</td><td>24</td><td>4</td><td>FgHV1gp2</td><td>-</td></tr>
<tr><td>siRNA-t0124537</td><td>GGAACAATGAAGGCGTGTTT</td><td>20</td><td>4</td><td>FGSG_09871</td><td>K14649|1|4e-07|54.7|sce:YML114C|transcription initiation factor TFIID subunit 8</td></tr>
<tr><td>siRNA-t0130898</td><td>GGACAAGCCGTCAAACATAGAC</td><td>22</td><td>4</td><td>FgHV1gp2</td><td>-</td></tr>
<tr><td>siRNA-t0141004</td><td>GGACCCGAGAAAAACGCCCC</td><td>20</td><td>4</td><td>FgHV1gp2</td><td>-</td></tr>
<tr><td>siRNA-t0139641</td><td>GGACGTCTTCGATGGATGCTGCGA</td><td>24</td><td>4</td><td>FgHV1gp2</td><td>-</td></tr>
<tr><td>siRNA-t0142160</td><td>GGAGTGGCATATTCAAGAATCAAT</td><td>24</td><td>4</td><td>FgHV1gp2</td><td>-</td></tr>
<tr><td>siRNA-t0140115</td><td>GGATTTGAGGGCGAGTGGAGCTA</td><td>23</td><td>4</td><td>FgHV1gp2</td><td>-</td></tr>
<tr><td>siRNA-t0144968</td><td>GGCAAAAGACAGATAGGTCGCA</td><td>22</td><td>4</td><td>FgHV1gp2</td><td>-</td></tr>
<tr><td>siRNA-t0150607</td><td>GGCAAATTTGGCCAAATCGTCATAC</td><td>25</td><td>4</td><td>FgHV1gp2</td><td>-</td></tr>
<tr><td>siRNA-t0137338</td><td>GGCCAAATCGTCATACATTGCACG</td><td>24</td><td>4</td><td>FgHV1gp2</td><td>-</td></tr>
<tr><td>siRNA-t0128607</td><td>GGCCCCAGCAGACTTGGAAGT</td><td>21</td><td>4</td><td>FgHV1gp2</td><td>-</td></tr>
<tr><td>siRNA-t0128237</td><td>GGGGTCTCCGATATTCGCTTCGCAT</td><td>25</td><td>4</td><td>FgHV1gp1</td><td>-</td></tr>
<tr><td>siRNA-t0143037</td><td>GGGTGGATCTGGGGTCTGACCG</td><td>22</td><td>4</td><td>FGSG_09943</td><td>-</td></tr>
<tr><td>siRNA-t0130047</td><td>GGGTGTGTATCCAATGAGTTGTGGG</td><td>25</td><td>4</td><td>FgHV1gp2</td><td>-</td></tr>
<tr><td>siRNA-t0126032</td><td>GGTAATTGCGGTCCATGGTG</td><td>20</td><td>4</td><td>FGSG_06420</td><td>K02202|1|3e-17|89.7|ssl:SS1G_06051|cyclin-dependent kinase 7 [EC:2.7.11.22 2.7.11.23]</td></tr>
<tr><td>siRNA-t0137816</td><td>GGTTCTTGAAGTTGGTGCCGAC</td><td>22</td><td>4</td><td>FgHV1gp2</td><td>-</td></tr>
<tr><td>siRNA-t0131925</td><td>GGTTCTTGGAAAGTTGGAGCCCTTC</td><td>25</td><td>4</td><td>FgHV1gp2</td><td>-</td></tr>
<tr><td>siRNA-t0142756</td><td>GGTTGGCACAACGATGAGCAC</td><td>21</td><td>4</td><td>FgHV1gp2</td><td>-</td></tr>
<tr><td>siRNA-t0131283</td><td>GGTTTGAAGGTGGAACTCAT</td><td>20</td><td>4</td><td>FGSG_08851</td><td>K01720|1|0.0|1076|fgr:FG08851.1|2-methylcitrate dehydratase [EC:4.2.1.79]</td></tr>
<tr><td>siRNA-t0142263</td><td>GTAAGCAGAGCGTGTAGGGCTG</td><td>22</td><td>4</td><td>FgHV1gp2</td><td>-</td></tr>
</table>

siRNA-t0138516	GTAAGTCTGGAATTCCTTGGGAGGC	25	4	FgHV1gp2	-
siRNA-t0124028	GTACTTGCCGCGCGTCTCACTGAC	24	4	FgHV1gp2	-
siRNA-t0136289	GTATCCCATAGTGCCCGGCC	20	4	FgHV1gp2	-
siRNA-t0131551	GTCATACATTGCACGAGGGACGA	23	4	FgHV1gp2	-
siRNA-t0138745	GTCGAAAGCATGGGTCGCAGT	21	4	FGSG_08278	-
siRNA-t0136301	GTCGAAAGTGAAAGTGTCGTAGG	23	4	FgHV1gp1	-
siRNA-t0143543	GTCTCACTGACTTCGTAACGGGT	23	4	FgHV1gp2	-
siRNA-t0146895	GTCTTCGACGGGTACGATGT	20	4	FGSG_07258	K14572|1|0.0|8396|fgr:FG07258.1|midasin
siRNA-t0127663	GTGAACCAGATCATAACCCGCATA	24	4	FgHV1gp2	-
siRNA-t0141577	GTGAACCTGTGGTGTCAAGAT	21	4	FGSG_02348	-
siRNA-t0138205	GTGACCAGCTGTTCTCTGTCGGC	23	4	FgHV1gp2	-
siRNA-t0139665	GTGCGCTTCTGGATCAATGTC	21	4	FgHV1gp2	-
siRNA-t0139687	GTGCTCATCGTTGTGCCAACCA	22	4	FGSG_04332	-
siRNA-t0143014	GTGTAGGACGATCCACTCCT	20	4	FGSG_12039	K00698|1|0.0|2441|nhe:NECHADRAFT_123231|chitin synthase [EC:2.4.1.16]
siRNA-t0125708	GTGTATCCAATGAGTTGTGGGA	22	4	FgHV1gp2	-
siRNA-t0147925	GTGTGATTGGTCTCTTGCAGTGCA	24	4	FgHV1gp2	-
siRNA-t0142524	GTTCAAGTATCGTCACTTCTTCGTA	25	4	FgHV1gp2	-
siRNA-t0134893	GTTCCATAAACGCTGCCGGGATGTA	25	4	FgHV1gp2	-
siRNA-t0133470	GTTCCTGCTGAGTGTGGTTTGA	22	4	FgHV1gp2	-
siRNA-t0131750	GTTCTCTTGTTGGCCTGGTAA	22	4	FGSG_04785	-
siRNA-t0138195	GTTGATCTTCCCTATGGCCT	20	4	FGSG_12003	K03457|1|0.0|1005|fgr:FG01732.1|nucleobase:cation symporter-1, NCS1 family
siRNA-t0136519	GTTGCCGAACGGTACATTGATT	22	4	FGSG_06540	K01876|1|1e-10|64.3|fgr:FG04934.1|aspartyl-tRNA synthetase [EC:6.1.1.12]
siRNA-t0128688	TAATATCGTCATTCTCTTCC	20	4	FGSG_11597	K03065|1|0.0|817|nhe:NECHADRAFT_69981|26S proteasome regulatory subunit T5
siRNA-t0129500	TAATCAATGTACCGTTCGGCAAC	23	4	FgHV1gp2	-
siRNA-t0147437	TACAGGCCTCATTGATTTTGT	21	4	FgHV1gp2	-
siRNA-t0147121	TACCGATATTGAACCCTCCTT	21	4	FGSG_01583	K00489|1|7e-11|67.8|bfu:BC1G_07665|cytochrome P450, family 7, subfamily A (cholesterol 7alpha-monooxygenase) [EC:1.14.13.17];K00517|3|2e-06|53.5|afm:AFUA_2G14430|[EC:1.14.-.-];K14338|4|8e-06|51.2|mgr:MGG_10879|cytochrome P450 / NADPH-cytochrome P450 reductase [EC:1.14.14.1 1.6.2.4]
siRNA-t0130120	TACTTGCCGCGCGTCTCACTGA	22	4	FgHV1gp2	-
siRNA-t0147145	TACTTGGCAGCCGACACCGG	20	4	FGSG_06645	K14674|1|3e-62|239|cdu:CD36_80370|TAG lipase / steryl ester hydrolase / phospholipase A2 / LPA acyltransferase [EC:3.1.1.3 3.1.1.13 3.1.1.4 2.3.1.51]
siRNA-t0137636	TAGAATGACGGCGCTCCGTG	20	4	FgHV1gp2	-
siRNA-t0141417	TAGACCGAAGAAAGACCACA	20	4	FGSG_12692	K14209|1|0.0|1296|fgr:FG05148.1|solute carrier family 36 (proton-coupled amino acid transporter)
siRNA-t0126677	TAGCGCCCAGGATTTCAAGT	20	4	FGSG_10882	K14571|1|0.0|1543|fgr:FG10882.1|ribosome biogenesis ATPase
siRNA-t0138610	TAGCGTCGTCACCTTGATTGG	21	4	FGSG_10511	K11236|1|0.0|1846|fgr:FG10511.1|cell division control protein 24
siRNA-t0127512	TAGGACCAGTTCCTGCTGAGTGT	23	4	FgHV1gp2	-
siRNA-t0139343	TAGGACGATCCACTCCTTTA	20	4	FGSG_08795	K15418|1|0.0|743|pcs:Pc21g12440|zearalenone synthase, highly reducing iterative type I polyketide synthase;K15419|3|0.0|713|ani:AN2547.2|emericellamide biosynthesis, highly reducing iterative type I polyketide synthase
siRNA-t0124025	TAGTGCCCGGCCGTTTGTGCT	21	4	FgHV1gp2	-
siRNA-t0132923	TATAGATCGCGCGTAAGCTTA	21	4	FgHV1gp2	-
siRNA-t0128399	TATAGGAGTACCAACAGCGGA	21	4	FGSG_08853	-
siRNA-t0130721	TATCCGTGATAGCCTCTCCCTT	22	4	FGSG_12196	-
siRNA-t0126441	TATCGTGTCTGACTGTTGCT	20	4	FgHV1gp2	-
siRNA-t0124951	TATTCCAACTGCGACCCATGCTT	23	4	FgHV1gp2	-
siRNA-t0133559	TCAAAGGCTAATTCTTCTGCGG	22	4	FGSG_01338	K06694|1|5e-128|455|fgr:FG01338.1|26S proteasome non-ATPase regulatory subunit 10
siRNA-t0124973	TCAACAATGAAGACGCAAGCCCGCT	25	4	FgHV1gp2	-
siRNA-t0146282	TCATTCCATTGATAATCGGCAT	22	4	FgHV1gp2	-
siRNA-t0141771	TCCAAACTGTTCATTTCGGCGAGTC	25	4	FgHV1gp2	-
siRNA-t0127361	TCCAACTGCGACCCATGCTTTCGA	24	4	FgHV1gp2	-
siRNA-t0137033	TCCAAGATTTGTCTAACTTGAT	22	4	FGSG_11080	-
siRNA-t0130941	TCCCCTTACCTTTGCGCTCC	20	4	FGSG_04474	-
siRNA-t0138378	TCCGGGATCAGGTTCACAATT	21	4	FGSG_08396	K01443|1|0.0|921|fgr:FG08396.1|N-acetylglucosamine-6-phosphate deacetylase [EC:3.5.1.25]
siRNA-t0140112	TCCGGGATCAGGTTCACAATTGCAC	25	4	FgHV1gp2	-
siRNA-t0130812	TCCTAGAGATCTCCGCAGAA	20	4	FGSG_07084	-
siRNA-t0135392	TCCTATAGATCGCGCGTAAGC	21	4	FgHV1gp2	-
siRNA-t0136042	TCCTTCGTTGAATCGATCGTCAT	23	4	FgHV1gp2	-
siRNA-t0136395	TCCTTCGTTGAATCGATCGTCATGG	25	4	FgHV1gp2	-
siRNA-t0129206	TCGAACCATACTGGACCCAGAT	22	4	FgHV1gp2	-
siRNA-t0137809	TCTGGGGTCTGACCGAGGCAGA	22	4	FGSG_04361	K11373|1|0.0|2514|fgr:FG04361.1|elongator complex protein 1
siRNA-t0148126	TCTTCGATGGATGCTGCGAAA	21	4	FgHV1gp2	-
siRNA-t0133080	TCTTCTGACGAATTTGGACA	20	4	FGSG_10174	K11367|1|2e-107|390|scm:SCHCODRAFT_58011|chromodomain-helicase-DNA-binding protein 1 [EC:3.6.4.12]
siRNA-t0136510	TCTTCTTTGTGAACATCTGGAT	22	4	FGSG_13766	-
siRNA-t0148469	TCTTGAAGTTGGTGCCGACGT	21	4	FGSG_09454	K14293|1|0.0|1735|fgr:FG09454.1|importin subunit beta-1
siRNA-t0136736	TCTTGCGGTGTGGGTCTTCT	20	4	FGSG_01790	K00680|1|0.0|2387|afm:AFUA_3G14700|[EC:2.3.1.-];K15419|2|0.0|1077|ani:AN2547.2|emericellamide biosynthesis, highly reducing iterative type I polyketide synthase;K15384|5|0.0|762|afv:AFLA_066840|aspyridone synthetase, hybrid polyketide synthase / nonribosomal peptide synthetase
siRNA-t0131905	TGAGCTCTGTGACTCCAGCCA	21	4	FgHV1gp2	-
siRNA-t0130220	TGATAAATGAACGTCTGACTGTAG	24	4	FgHV1gp2	-
siRNA-t0142587	TGATCCATGCCAAACCAACTTA	22	4	FgHV1gp2	-
siRNA-t0124707	TGCACCAGGATTTGCTTCTT	20	4	FgHV1gp2	-
siRNA-t0136721	TGCAGTTGGTCCGCGAATTCTG	22	4	FgHV1gp2	-
siRNA-t0145535	TGCAGTTGGTCCGCGAATTCTGCC	24	4	FgHV1gp2	-
siRNA-t0149552	TGCCATGACGTACGACCCCGAAAG	24	4	FgHV1gp1	-
siRNA-t0129075	TGCCTGGGAGACAGAGCCTGAGTCA	25	4	FgHV1gp2	-
siRNA-t0132913	TGCGACCCATGCTTTCGACGTACTT	25	4	FgHV1gp2	-
siRNA-t0135462	TGCGCTTCTGGATCAATGTCAGC	23	4	FgHV1gp2	-
siRNA-t0149911	TGCTCATGAGGTTGGCATTG	20	4	FGSG_12742	K00472|1|0.0|1452|fgr:FG05473.1|prolyl 4-hydroxylase [EC:1.14.11.2];K09241|3|4e-07|56.2|ago:AGOS_AFL160C|transcriptional regulatory protein GAL4;K09247|5|2e-06|54.3|vpo:Kpol_467p1|heme activator protein 1
siRNA-t0136077	TGCTCTGCCTCGGTCAGACCCC	22	4	FgHV1gp2	-
siRNA-t0129668	TGCTTTACCGGATCTCCGCTCC	22	4	FgHV1gp1	-
siRNA-t0127211	TGGCTTGTGGTTGCGAAATC	20	4	FGSG_06635	K00784|1|0.0|1690|fgr:FG06635.1|ribonuclease Z [EC:3.1.26.11]
siRNA-t0149576	TGGGCGTGTGTTCTTTATGGGCTC	24	4	FgHV1gp2	-
siRNA-t0145385	TGGGGTGTGTATCCAATGAGTTGTG	25	4	FgHV1gp2	-
siRNA-t0128702	TGGGTCGAGGATGCAAACTTCT	22	4	FGSG_07064	-
siRNA-t0134971	TGGGTCTTCTGACGAATTTGGACAA	25	4	FgHV1gp2	-
siRNA-t0129612	TGGTACCGATATTGAACCCT	20	4	FgHV1gp2	-
siRNA-t0142602	TGGTACCGATATTGAACCCTC	21	4	FgHV1gp2	-
siRNA-t0143712	TGGTACCGATATTGAACCCTCC	22	4	FgHV1gp2	-
siRNA-t0127052	TGGTCCGCGAATTCTGCCAT	20	4	FgHV1gp2	-
siRNA-t0126421	TGGTGATGCATTGCTAGAGT	20	4	FGSG_10561	K01183|1|0.0|635|afm:AFUA_5G03960|chitinase [EC:3.2.1.14]
siRNA-t0130512	TGGTTGAGGCTTCTTCATGA	20	4	FGSG_10996	-
siRNA-t0126494	TGTACGTCCGAATACCATACTTT	23	4	FgHV1gp2	-

siRNA-t0125371	TGTACGTCCGAATACCATACTTTCC	25	4	FgHV1gp2	-
siRNA-t0140934	TGTAGGACGATCCACTCCTT	20	4	FGSG_12039	K00698|1|0.0|2441|nhe:NECHADRAFT_123231|chitin synthase [EC:2.4.1.16]
siRNA-t0133116	TGTGGTCTTTCTTCGGTCTAG	21	4	FGSG_14002	-
siRNA-t0148889	TGTGTCGCGTCTACCTTGTTC	21	4	FgHV1gp2	-
siRNA-t0146659	TGTTGTTGGTCTTCCAGGCAT	21	4	FGSG_08534	-
siRNA-t0128999	TTACCTAGACCGAAGAAAGACCA	23	4	FgHV1gp2	-
siRNA-t0132248	TTAGAATGACGGCGCTCCGTG	21	4	FgHV1gp2	-
siRNA-t0135535	TTATAAGGCTTGTCGTCGGGAG	22	4	FGSG_08614	K01874|1|0.0|1256|fgr:FG08614.1|methionyl-tRNA synthetase [EC:6.1.1.10]
siRNA-t0128614	TTCGGTATTGTCGAGTCGAAA	21	4	FgHV1gp2	-
siRNA-t0148050	TTCTCCACGTCATGATCATCA	21	4	FGSG_01569	-
siRNA-t0137162	TTCTGACGAATTTGGACAAGTA	22	4	FgHV1gp2	-
siRNA-t0123724	TTGAAATCCTGGGCGCTAAC	20	4	FgHV1gp2	-
siRNA-t0136132	TTGAATCGATCGTCATGGTGAT	22	4	FgHV1gp2	-
siRNA-t0124387	TTGACGATGAATAGGCCTCC	20	4	FGSG_09707	K05658|1|4e-38|160|fgr:FG01684.1|ATP-binding cassette, subfamily B (MDR/TAP), member 1
siRNA-t0128629	TTGATCTCAGCACGCCGGTTACCA	24	4	FgHV1gp2	-
siRNA-t0149436	TTGCAACGAGGGCCCAGTCA	20	4	FgHV1gp2	-
siRNA-t0127845	TTGCAGTTGGTCCGCGAATTCTG	23	4	FgHV1gp2	-
siRNA-t0148454	TTGGTATTGTCAGGGTTCAA	20	4	FGSG_10523	K01932|1|4e-178|626|afm:AFUA_6G12080|[EC:6.3.2.-]
siRNA-t0123746	TTGGTCTCTTGCAGTGCAATACC	23	4	FGSG_08418	-
siRNA-t0134693	TTGTTCCATAAACGCTGCCGGGATG	25	4	FgHV1gp2	-
siRNA-t0191107	AAACATATCCGGCTCCCTTT	20	3	FGSG_06595	-
siRNA-t0165577	AAACGCTGCCGGGATGTAAT	20	3	FgHV1gp2	-
siRNA-t0180977	AAACGCTGCCGGGATGTAATCAAT	24	3	FgHV1gp2	-
siRNA-t0181772	AAACTGCAGTGGCTTGTGGT	20	3	FGSG_11240	K08711|1|0.0|1162|cne:CNB05770|ATP-binding cassette, subfamily G (WHITE), member 2, PDR
siRNA-t0190822	AAAGCATGGCGATGCACTTT	20	3	FGSG_11083	-
siRNA-t0195234	AAAGTTCTCGCCAGCAATGCCCCT	24	3	FgHV1gp1	-
siRNA-t0175696	AAATCAATGAGGCCTGTAACT	21	3	FGSG_12450	-
siRNA-t0177847	AAATGATTTGCGACGTGCTTGAAG	24	3	FgHV1gp2	-
siRNA-t0158072	AAATTTGCCGACGCTCCACT	20	3	FGSG_09965	K14300|1|0.0|2662|fgr:FG09965.1|nuclear pore complex protein Nup133
siRNA-t0164137	AACATAGACACTTCAACATGGG	22	3	FGSG_07383	K08712|1|0.0|1020|pic:PICST_57529|ATP-binding cassette, subfamily G (WHITE), member 2, SNQ2;K08711|4|0.0|952|cne:CNB05770|ATP-binding cassette, subfamily G (WHITE), member 2, PDR
siRNA-t0156241	AACCAAGAGTGCTCATCGTT	20	3	FGSG_10431	-
siRNA-t0165381	AACCAAGAGTGCTCATCGTTG	21	3	FGSG_10431	-
siRNA-t0159933	AAGGGACGGAAGCCATCAAC	20	3	FGSG_09996	-
siRNA-t0190933	AAGGGTGACGTCAACAGTGA	20	3	FGSG_10200	K00121|1|0.0|752|fgr:FG10200.1|S-(hydroxymethyl)glutathione dehydrogenase / alcohol dehydrogenase [EC:1.1.1.284 1.1.1.1]
siRNA-t0162884	AAGGTACGTCTATATTGTCT	20	3	FgHV1gp2	-
siRNA-t0153813	AAGGTACGTCTATATTGTCTCTCA	24	3	FgHV1gp2	-
siRNA-t0193238	AAGTAGCAGATTTTACAAAGTCAT	24	3	FgHV1gp2	-
siRNA-t0156223	AATATCGTACCGCAGTGATGGA	22	3	FGSG_08521	K07512|1|0.0|855|fgr:FG08521.1|mitochondrial trans-2-enoyl-CoA reductase [EC:1.3.1.38]
siRNA-t0182242	AATATCGTCATTCTCTTCCAGT	22	3	FGSG_10105	K12864|1|8e-17|88.2|cne:CNH03260|beta-catenin-like protein 1
siRNA-t0180586	AATCATCATCGTAGTCATCGTCAG	24	3	FGSG_07312	-
siRNA-t0194269	AATCCCTGGATTAGCCATTCGTT	23	3	FgHV1gp2	-
siRNA-t0152485	AATCGTGGACTTCCTTTAGGAAAGC	25	3	FgHV1gp2	-
siRNA-t0155996	AATGGCGACACCGACGGTACGT	22	3	FgHV1gp2	-
siRNA-t0169931	AATTACGGACAGTACATGCGT	21	3	FGSG_00916	K03006|1|0.0|3167|fgr:FG00916.1|DNA-directed RNA polymerase II subunit RPB1 [EC:2.7.7.6]
siRNA-t0183900	AATTTGATTGATTCTAGCTTGT	22	3	FgHV1gp2	-
siRNA-t0153605	ACAATCGTGGACTTCCTTTAGGAA	24	3	FgHV1gp2	-
siRNA-t0156384	ACACCACATCACGCTCGGGGTCAA	24	3	FgHV1gp2	-
siRNA-t0199907	ACACGCCCAATCCCACGAGAA	21	3	FGSG_10982	K01278|1|0.0|1529|fgr:FG10982.1|dipeptidyl-peptidase 4 [EC:3.4.14.5]
siRNA-t0187957	ACAGCTCCAATAATCGTTGA	20	3	FgHV1gp2	-
siRNA-t0187384	ACAGGACCGCGCGATGGCGC	20	3	FGSG_12361	-
siRNA-t0190911	ACAGGCTAGGAACGATGCCTC	21	3	FGSG_02233	-
siRNA-t0172603	ACATATCTCCTTGAACTTGAGC	22	3	FGSG_11146	K11987|1|1e-83|311|aor:AOR_1_1372154|prostaglandin-endoperoxide synthase 2 [EC:1.14.99.1]
siRNA-t0181681	ACATATCTCGATTTCGCAACCAC	23	3	FgHV1gp2	-
siRNA-t0166871	ACATCACGCTCGGGGTCAATTT	22	3	FgHV1gp2	-
siRNA-t0181237	ACATTCAACGGCGAAACATC	20	3	FGSG_13700	-
siRNA-t0193702	ACATTGATCCAGAAGCGCACG	21	3	FGSG_01364	-
siRNA-t0153122	ACCACAACGATAGTATGAACCCT	23	3	FgHV1gp2	-
siRNA-t0192134	ACCACAGTCGAATGAAATGGAAC	23	3	FgHV1gp2	-
siRNA-t0171774	ACCAGAAACCCATCCCACTCG	21	3	FgHV1gp2	-
siRNA-t0168772	ACCATCAGGACCCGAGAAAAA	21	3	FgHV1gp2	-
siRNA-t0155652	ACCATCAGGACCCGAGAAAAACG	23	3	FgHV1gp2	-
siRNA-t0183403	ACCATCAGGACCCGAGAAAAACGC	24	3	FgHV1gp2	-
siRNA-t0198152	ACCATCTGTTCTACGTTTGA	20	3	FGSG_05607	K03848|1|0.0|1049|fgr:FG05607.1|alpha-1,3-glucosyltransferase [EC:2.4.1.267]
siRNA-t0159682	ACCCACACCGCAAGACTCTG	20	3	FGSG_06054	K01784|1|3e-65|249|fgr:FG07983.1|UDP-glucose 4-epimerase [EC:5.1.3.2]
siRNA-t0197813	ACCCATGCTTTCGACGTACTTG	22	3	FgHV1gp2	-
siRNA-t0159100	ACCCATTGATCATTCTCCTCA	21	3	FgHV1gp2	-
siRNA-t0197076	ACCGACGGTACGTGGAAATC	20	3	FgHV1gp2	-
siRNA-t0165171	ACCGACGGTACGTGGAAATCT	21	3	FgHV1gp2	-
siRNA-t0185443	ACCGATATTGAACCCTCCTTCGTGA	25	3	FgHV1gp2	-
siRNA-t0192236	ACGCCGCCAGAATCGAGCTA	20	3	FgHV1gp2	-
siRNA-t0201084	ACGGAACCCATTGATCATTCTCCT	24	3	FgHV1gp2	-
siRNA-t0167299	ACGGATCCACATGAGCTTCAGCT	23	3	FgHV1gp2	-
siRNA-t0186051	ACGGATCCACATGAGCTTCAGCTTC	25	3	FgHV1gp2	-
siRNA-t0190184	ACGTACCCTGACGATGACTA	20	3	FGSG_08056	-
siRNA-t0151065	ACGTACCCTGACGATGACTACGA	23	3	FGSG_08056	-
siRNA-t0162205	ACGTAGATGTGATGCCTTGTCTA	23	3	FgHV1gp2	-
siRNA-t0158948	ACGTGACCAGCTGTTCTCTG	20	3	FgHV1gp2	-
siRNA-t0200473	ACGTGACCAGCTGTTCTCTGT	21	3	FgHV1gp2	-
siRNA-t0167957	ACGTGCCATCATTGAAAAACA	21	3	FGSG_05542	K14311|1|0.0|3550|fgr:FG05542.1|nuclear pore complex protein Nup188
siRNA-t0172955	ACGTGGAAATCTTCAGGCCC	20	3	FgHV1gp2	-
siRNA-t0159665	AGATCTTGCTGTAGTGTCTGAAGAT	25	3	FgHV1gp2	-
siRNA-t0161888	AGATTAGGCAGACCATTCAA	20	3	FGSG_00044	K01567|1|7e-35|147|afm:AFUA_3G01280|[EC:3.-.-.-]
siRNA-t0175269	AGCACCGTACGGATCCACATGA	22	3	FgHV1gp2	-
siRNA-t0188798	AGCACCGTACGGATCCACATGAGCT	25	3	FgHV1gp2	-
siRNA-t0164897	AGCACTTGTTTGCCTGCATCGAT	23	3	FgHV1gp2	-
siRNA-t0178791	AGCATTTGTTTGTACGTCCG	20	3	FgHV1gp2	-
siRNA-t0169069	AGCCATTTCGAATCTGCCCGA	21	3	FgHV1gp2	-
siRNA-t0172273	AGCCATTTCGAATCTGCCCGATC	23	3	FgHV1gp2	-
siRNA-t0192484	AGGACCCGAGAAAAACGCCCCTGGA	25	3	FgHV1gp2	-
siRNA-t0152088	AGGCCAAAGGTGTTCAGCTT	20	3	FgHV1gp2	-
siRNA-t0193117	AGGCCTCATTGATTTTGTGT	20	3	FgHV1gp2	-
siRNA-t0186614	AGGCCTCATTGATTTTGTGTCGC	23	3	FgHV1gp2	-
siRNA-t0153034	AGGCCTCTGACGTTGATCTC	20	3	FGSG_13878	K01932|1|0.0|1450|afm:AFUA_5G12730|[EC:6.3.2.-]
siRNA-t0173272	AGGCTCCTCCGACAATGGCG	20	3	FGSG_10215	K15289|1|2e-09|62.8|yli:YALI0E22803g|solute carrier family 35, member F5;K15303|2|4e-09|62.0|ani:AN0675.2|aflatoxin B1 aldehyde reductase
siRNA-t0173527	AGTAATTGGTTCTTTAGGTTGATT	24	3	FgHV1gp2	-
siRNA-t0188472	AGTATAGGTTATCCGCGGCA	20	3	FGSG_00742	K13621|1|0.0|1687|fgr:FG00742.1|betaine lipid synthase

siRNA-t0165286	AGTCACGCTCTAAAGCACGAGG	22	3	FGSG_08309	K08712|1|0.0|884|sce:YDR011W|ATP-binding cassette, subfamily G (WHITE), member 2, SNQ2;K08711|4|0.0|875|cgr:CAGL0M01760g|ATP-binding cassette, subfamily G (WHITE), member 2, PDR
siRNA-t0155746	AGTCGAATGAAATGGAACAACGT	23	3	FgHV1gp2	-
siRNA-t0157792	AGTCTCCGAACGTGATCTCATCAT	24	3	FgHV1gp2	-
siRNA-t0153720	AGTCTTCGTCCAGTAGCCAATGA	23	3	FgHV1gp2	-
siRNA-t0162441	AGTGACGATACTTGAACCAAG	21	3	FGSG_07561	-
siRNA-t0168321	AGTGTCGAACCATACTGGAC	20	3	FgHV1gp2	-
siRNA-t0168199	AGTGTCGTAGGAAAGGAAAA	20	3	FGSG_13645	-
siRNA-t0196804	ATACATGCCTGATGTTCTTGTG	22	3	FgHV1gp2	-
siRNA-t0196448	ATACCATTCACGAACGCTAA	20	3	FgHV1gp2	-
siRNA-t0157740	ATACCTGATGGCAGTTGACCTTA	23	3	FgHV1gp2	-
siRNA-t0169511	ATACCTTTGAGCTCCTTGAGGAAT	24	3	FgHV1gp2	-
siRNA-t0190858	ATACGTTCTGAGAAGTCTCCG	21	3	FgHV1gp2	-
siRNA-t0168583	ATAGGAGATGATGTTGGTTAG	21	3	FGSG_06933	K11132|1|1e-08|62.0|sce:YDR206W|telomere elongation protein [EC:2.7.7.-]
siRNA-t0192771	ATGATCATCAGCACCGTACGGA	22	3	FgHV1gp2	-
siRNA-t0195406	ATGATCATCAGCACCGTACGGATC	24	3	FgHV1gp2	-
siRNA-t0167109	ATGATCATGACGTGGAGAAGA	21	3	FGSG_01407	-
siRNA-t0174037	ATGATCATGACGTGGAGAAGAA	22	3	FGSG_01407	-
siRNA-t0175770	ATGATCTGGTTCACGCTGCA	20	3	FGSG_11536	K00517|1|4e-44|178|afm:AFUA_6G13945|[EC:1.14.-.-];K05917|4|5e-17|88.6|lel:LELG_03738|cytochrome P450, family 51 (sterol 14-demethylase) [EC:1.14.13.70]
siRNA-t0190876	ATGCATGTAGATCTTGCTGTA	21	3	FGSG_03410	K05349|1|1e-174|613|nhe:NECHADRAFT_70519|beta-glucosidase [EC:3.2.1.21]
siRNA-t0151474	ATGCCAACCTCATGAGCACCC	21	3	FGSG_05519	K04728|1|0.0|5526|fgr:FG05519.1|ataxia telangiectasia mutated family protein [EC:2.7.11.1]
siRNA-t0188167	ATGCCTGATGTTCTTGTGCCTCC	23	3	FgHV1gp2	-
siRNA-t0189483	ATGCTCTGCCTCGGTCAGACCCCA	24	3	FgHV1gp2	-
siRNA-t0200342	ATGGACTGGCCTGTTGTCACCTTC	24	3	FgHV1gp2	-
siRNA-t0185782	ATGGCCGGCAGTCCTAGCAAGTG	23	3	FgHV1gp2	-
siRNA-t0156745	ATGGCCGGCAGTCCTAGCAAGTGA	24	3	FgHV1gp2	-
siRNA-t0153442	ATGGGTCGCAGTTGGAATAT	20	3	FGSG_08364	-
siRNA-t0159600	ATGTAAGTGACGAGGCCTCT	20	3	FgHV1gp2	-
siRNA-t0193530	ATGTTGTAGAACTTTTGCCACTG	23	3	FgHV1gp2	-
siRNA-t0156131	ATTCAGTAGTCGGGGTCTCC	20	3	FGSG_03062	-
siRNA-t0185091	ATTCCAACTGCGACCCATGCTT	22	3	FgHV1gp2	-
siRNA-t0160191	ATTCCTGAAGTATTTCTCCCCGA	23	3	FgHV1gp2	-
siRNA-t0154499	ATTCGAAATGGCTGACCCCA	20	3	FGSG_03518	-
siRNA-t0171683	ATTGACGATGAATAGGCCTC	20	3	FGSG_09707	K05658|1|4e-38|160|fgr:FG01684.1|ATP-binding cassette, subfamily B (MDR/TAP), member 1
siRNA-t0172409	ATTGCTGCATGTACTGTTCGGCA	23	3	FgHV1gp2	-
siRNA-t0152715	ATTGGAACCAAAGCCTGTGA	20	3	FGSG_10381	K00924|1|0.0|1042|afm:AFUA_4G06750|[EC:2.7.1.-];K06683|4|1e-58|228|cal:CaO19.11029|cell division control protein CDC15 [EC:2.7.11.1]
siRNA-t0181169	ATTTCTCCAGTAGCATGTTCTTCGT	25	3	FgHV1gp2	-
siRNA-t0165420	ATTTGCGACGTGCTTGAAGGCGAA	24	3	FgHV1gp2	-
siRNA-t0153936	ATTTGGCCAAATCGTCATACA	21	3	FGSG_01010	K13093|1|0.0|674|fgr:FG01010.1|HIV Tat-specific factor 1
siRNA-t0174149	ATTTGGCCCCAGCAGACTTGG	21	3	FgHV1gp2	-
siRNA-t0196783	ATTTGTTTGTACGTCCGAATACCA	24	3	FgHV1gp2	-
siRNA-t0169927	ATTTTATTGTAAGTCTGGAA	20	3	FGSG_05095	-
siRNA-t0197041	CAAAGAACCCATATGGATCGG	21	3	FgHV1gp2	-
siRNA-t0163505	CAAATCAAGTTGGTATCCCTCC	22	3	FgHV1gp2	-
siRNA-t0171822	CAACAATGAAGACGCAAGCCCGCT	24	3	FgHV1gp2	-
siRNA-t0165507	CAAGAAGTTTGCATCCTCGA	20	3	FGSG_09793	-
siRNA-t0173869	CAAGTATCGTCACTTCTTCGTA	22	3	FgHV1gp2	-
siRNA-t0189814	CAAGTTGGTATCCCTCCGTGG	21	3	FgHV1gp2	-
siRNA-t0163860	CAAGTTGTGACTGCAGGTAC	20	3	FGSG_04640	-
siRNA-t0168739	CAATACCTGTGTCTTTATACC	21	3	FGSG_03507	K05909|1|1e-70|266|afm:AFUA_1G15670|[EC:1.10.3.2]
siRNA-t0191321	CAATCGTGGACTTCCTTTAGG	21	3	FgHV1gp2	-
siRNA-t0176079	CAATGATGGCACGTCGACAGGCG	23	3	FgHV1gp2	-
siRNA-t0161014	CAATGCAACAGACTTCTTCT	20	3	FGSG_13783	K01932|1|0.0|1526|afm:AFUA_5G12730|[EC:6.3.2.-]
siRNA-t0159558	CAATGCAACAGACTTCTTCTCT	22	3	FgHV1gp2	-
siRNA-t0177390	CAATGCCGCCAACCACCAGG	20	3	FGSG_03062	-
siRNA-t0156896	CAATGTACCGTTCGGCAACAG	21	3	FgHV1gp2	-
siRNA-t0172933	CACAATTGCACATGGGACCATC	22	3	FgHV1gp2	-
siRNA-t0156506	CACCACATCACGCTCGGGGTCA	22	3	FgHV1gp2	-
siRNA-t0159321	CACCGACGGTACGTGGAAATCTTCA	25	3	FgHV1gp2	-
siRNA-t0167722	CACCGTCCGGAATGCGTTCA	20	3	FGSG_02766	-
siRNA-t0158457	CACGAACGCTAAGAGGAAAGGA	22	3	FgHV1gp2	-
siRNA-t0199448	CACGATCTTCAAAAATGTGT	20	3	FGSG_10508	-
siRNA-t0170140	CACGGAACCCATTGATCATTCTCCT	25	3	FgHV1gp2	-
siRNA-t0199649	CACGTACCGTCGGTGTCGCC	20	3	FGSG_08320	K00495|1|4e-93|341|afm:AFUA_1G01690|[EC:1.14.14.-]
siRNA-t0173703	CAGATTCCAACCTGTTGTCA	20	3	FGSG_11514	-
siRNA-t0196863	CAGCACCGTACGGATCCACATGA	23	3	FgHV1gp2	-
siRNA-t0191270	CAGCAGAATTCTAGCCTGGTTGAG	24	3	FgHV1gp2	-
siRNA-t0200186	CAGCGTAACGTGACCAGCTGTTC	23	3	FgHV1gp2	-
siRNA-t0167426	CAGCTCCAATAATCGTTGAA	20	3	FGSG_05996	K00936|1|3e-61|238|afm:AFUA_6G10240|[EC:2.7.3.-];K11231|2|6e-39|163|afm:AFUA_2G00660|osomolarity two-component system, sensor histidine kinase SLN1 [EC:2.7.13.3];K02486|3|2e-38|161|spo:SPAC27E2.09|two-component system, unclassified family, sensor kinase [EC:2.7.13.3]
siRNA-t0190340	CAGCTGTTCTCTGTCGGCATGTTGC	25	3	FgHV1gp2	-
siRNA-t0179440	CAGGCCTCATTGATTTTGTGT	21	3	FgHV1gp2	-
siRNA-t0174176	CAGGGGCGTTTTTCTCGGGT	20	3	FGSG_08097	K00688|1|7e-16|87.0|pno:SNOG_13460|starch phosphorylase [EC:2.4.1.1]
siRNA-t0199137	CAGTAGGCTTCAAATTAGAC	20	3	FGSG_09628	K00586|1|3e-168|589|fgr:FG09628.1|diphthine synthase [EC:2.1.1.98]
siRNA-t0179285	CAGTCCTAGCAAGTGAAACTGTT	23	3	FgHV1gp2	-
siRNA-t0184064	CAGTGGCTTGTGGTTGCGAAA	21	3	FGSG_05477	K01104|1|1e-12|74.7|bfu:BC1G_07812|protein-tyrosine phosphatase [EC:3.1.3.48]
siRNA-t0175303	CAGTGGCTTGTGGTTGCGAAAT	22	3	FGSG_05477	K01104|1|1e-12|74.7|bfu:BC1G_07812|protein-tyrosine phosphatase [EC:3.1.3.48]
siRNA-t0186093	CAGTGTCAGGATCCGTCCAGGGAA	24	3	FgHV1gp2	-
siRNA-t0171411	CAGTTCCGCGTGTGATTGGT	20	3	FGSG_03919	-
siRNA-t0166739	CATACAATGCCGCCAACCACCA	22	3	FgHV1gp2	-
siRNA-t0152927	CATCAACGGTTCTTGGAAAGT	21	3	FgHV1gp2	-
siRNA-t0195867	CATCAGCACCGTACGGATCC	20	3	FGSG_07266	K00643|1|0.0|1118|nhe:NECHADRAFT_68614|5-aminolevulinate synthase [EC:2.3.1.37]
siRNA-t0198713	CATTCACGAACGCTAAGAGGA	21	3	FgHV1gp2	-
siRNA-t0191414	CATTGATCCAGAAGCGCACG	20	3	FGSG_03153	-
siRNA-t0197613	CATTGATCCAGAAGCGCACGA	21	3	FGSG_01364	-
siRNA-t0162277	CATTGCACGAGGGACGAGCTTGG	23	3	FgHV1gp2	-
siRNA-t0170505	CCAAAACGCGCCTCCAGTCCA	21	3	FgHV1gp2	-
siRNA-t0176482	CCAACGAGGTTATGGCAGAAT	21	3	FGSG_05145	K12605|1|1e-17|89.7|fgr:FG05100.1|CCR4-NOT transcription complex subunit 2;K14640|2|2e-12|72.8|ani:AN2812.2|solute carrier family 20 (sodium-dependent phosphate transporter)
siRNA-t0196902	CCAAGATTTGTCTAACTTGA	20	3	FGSG_11080	-
siRNA-t0161931	CCAAGTAAGCAGAGCGTGTAG	21	3	FgHV1gp2	-
siRNA-t0180624	CCAATAATCGTTGAAAATCAGAGA	24	3	FgHV1gp2	-

siRNA-t0170266	CCAATGTAAGTGACGAGGCCTCT	23	3	FgHV1gp2	-
siRNA-t0167223	CCACAGTCGAATGAAATGGAACA	23	3	FgHV1gp2	-
siRNA-t0169517	CCACGATTGGCGTGAGTGCGTA	22	3	FgHV1gp2	-
siRNA-t0162026	CCACTGCGAATGTTTCACTCCTCCT	25	3	FgHV1gp2	-
siRNA-t0160007	CCAGAACCCAGCACCTTTCCCTAT	24	3	FgHV1gp2	-
siRNA-t0191004	CCAGATGCGAAGCGAATATCGG	22	3	FGSG_03912	K09246|1|3e-07|55.8|lth:KLTH0E16500g|arginine metabolism regulation protein II
siRNA-t0181889	CCAGGCCAACAAAGAGAACT	20	3	FGSG_06226	K15084|1|0.0|743|fgr:FG06226.1|solute carrier family 25 (mitochondrial carrier protein), member 16
siRNA-t0163893	CCAGTAGCATGTTCTTCGTG	20	3	FGSG_12034	K06877|1|0.0|2299|fgr:FG01945.1|DEAD/DEAH box helicase domain-containing protein
siRNA-t0191498	CCAGTAGCATGTTCTTCGTGTAGA	24	3	FgHV1gp2	-
siRNA-t0198455	CCAGTGGCGCATTTCTTTCTTA	22	3	FgHV1gp2	-
siRNA-t0167833	CCATAAACGCTGCCGGGATGTA	22	3	FgHV1gp2	-
siRNA-t0158968	CCATAAACGCTGCCGGGATGTAA	23	3	FgHV1gp2	-
siRNA-t0166058	CCATACTTTCCGAAGGAACTCCACA	25	3	FgHV1gp2	-
siRNA-t0188807	CCATAGTGCCCGGCCGTTTGTGCT	24	3	FgHV1gp2	-
siRNA-t0155793	CCATCCTATAGATCGCGCGTAA	22	3	FgHV1gp2	-
siRNA-t0165653	CCATCGCGCGGTCCTGTATT	20	3	FGSG_05512	K11397|1|0.0|2685|fgr:FG05512.1|chromatin modification-related protein VID21
siRNA-t0156519	CCATGCTTTCGACGTACTTGGG	22	3	FgHV1gp2	-
siRNA-t0191036	CCATTCGTTACAATCGTGGACT	22	3	FgHV1gp2	-
siRNA-t0182329	CCATTGATAATCGGCATGAAC	21	3	FGSG_00104	-
siRNA-t0196897	CCATTTCATTCGACTGTGGTT	21	3	FGSG_00546	K12869|1|8e-42|171|ure:UREG_03429|crooked neck
siRNA-t0200822	CCCGGCAGCGTTTATGGAAC	20	3	FGSG_12361	-
siRNA-t0201008	CCCTCAAATGTCTCCTTCGGTT	22	3	FgHV1gp2	-
siRNA-t0163012	CCCTCGCATTTGCCGCACAGCT	22	3	FgHV1gp2	-
siRNA-t0152242	CCCTCGCATTTGCCGCACAGCTCCA	25	3	FgHV1gp2	-
siRNA-t0197713	CCCTTCGTGAACTGCTGCGCTA	22	3	FgHV1gp2	-
siRNA-t0178154	CCGACGGTACGTGGAAATCTTCAGG	25	3	FgHV1gp2	-
siRNA-t0154935	CCGACGTTTTGGCGAGACCGA	21	3	FgHV1gp2	-
siRNA-t0199628	CCGAGAAAAACGCCCCTGGA	20	3	FGSG_12940	-
siRNA-t0196741	CCGCGAATTCTGCCATAACC	20	3	FgHV1gp2	-
siRNA-t0173489	CCGCGTGTGATTGGTCTCTTG	21	3	FGSG_06161	K14850|1|0.0|818|fgr:FG06161.1|ribosomal RNA-processing protein 8 [EC:2.1.1.-]
siRNA-t0192850	CCGCTTGGCTTGGTGATGCAT	21	3	FGSG_06090	K01907|1|0.0|1438|fgr:FG06090.1|acetoacetyl-CoA synthetase [EC:6.2.1.16]
siRNA-t0154027	CCGGCCCTCATTGATTCTTG	20	3	FGSG_09004	K00995|1|0.0|975|fgr:FG09004.1|CDP-diacylglycerol--glycerol-3-phosphate 3-phosphatidyltransferase [EC:2.7.8.5]
siRNA-t0170587	CCGGGATCAGGTTCACAATTGCAC	24	3	FgHV1gp2	-
siRNA-t0183939	CCGGTGTCGGCTGCCAAGTAAGC	23	3	FgHV1gp2	-
siRNA-t0166289	CCGGTTGAAAATGTTCCACA	20	3	FGSG_05462	K01072|1|2e-159|564|afm:AFUA_2G08440|ubiquitin thiolesterase [EC:3.1.2.15];K11838|3|3e-41|171|clu:CLUG_02963|ubiquitin carboxyl-terminal hydrolase 7 [EC:3.1.2.15]
siRNA-t0151174	CCGTTCGGCAACAGATCTTGTA	22	3	FGSG_03119	-
siRNA-t0163607	CCTAGAGATCTCCGCAGAAGA	21	3	FGSG_00352	K08064|1|2e-78|291|mgr:MGG_00354|nuclear transcription factor Y, alpha
siRNA-t0158563	CCTAGAGATCTCCGCAGAAGAAT	23	3	FGSG_00352	K08064|1|2e-78|291|mgr:MGG_00354|nuclear transcription factor Y, alpha
siRNA-t0200274	CCTATACTTGATCTCAGCACGC	22	3	FgHV1gp2	-
siRNA-t0156753	CCTATAGATCGCGCGTAAGCTTAGC	25	3	FgHV1gp2	-
siRNA-t0193405	CCTCAGAGTCTTGCGGTGTGG	21	3	FGSG_10283	-
siRNA-t0201281	CCTCTCCCTTCGTGAACTGCTGC	23	3	FgHV1gp2	-
siRNA-t0151412	CCTCTGATATATTCCAACTGCGAC	24	3	FgHV1gp2	-
siRNA-t0157454	CCTCTGTCTATGAGCACTTG	20	3	FGSG_09118	K01183|1|0.0|1040|ssl:SS1G_00773|chitinase [EC:3.2.1.14]
siRNA-t0153958	CCTGAAGTTATGAGCTCTGTGA	22	3	FgHV1gp2	-
siRNA-t0168929	CGAACGGGACCGACGTTTTGGCGA	24	3	FgHV1gp2	-
siRNA-t0182741	CGAAGAAAGACCACATCCAGATCT	24	3	FgHV1gp2	-
siRNA-t0182321	CGAAGGAACTCCACATAGCGTCG	23	3	FgHV1gp2	-
siRNA-t0169835	CGACAGGCGTGGCTGAGAGA	20	3	FgHV1gp2	-
siRNA-t0183389	CGACGACAAGCCTTATAACA	20	3	FGSG_09607	K10413|1|0.0|8782|fgr:FG09607.1|dynein heavy chain 1, cytosolic
siRNA-t0173976	CGACGACAAGCCTTATAACAAT	22	3	FGSG_09607	K10413|1|0.0|8782|fgr:FG09607.1|dynein heavy chain 1, cytosolic
siRNA-t0182942	CGACGGTACGTGGAAATCTTCAG	23	3	FgHV1gp2	-
siRNA-t0201785	CGCAGTGATGGAGACTAAACG	21	3	FGSG_09869	K12669|1|0.0|647|fgr:FG09869.1|oligosaccharyltransferase complex subunit gamma
siRNA-t0153080	CGCCAGAATCGAGCTAAACGAACTT	25	3	FgHV1gp2	-
siRNA-t0183669	CGCCCCGATTCTTGTAATGCATGT	24	3	FgHV1gp2	-
siRNA-t0171227	CGCCGCCAGAATCGAGCTAAACGAA	25	3	FgHV1gp2	-
siRNA-t0171428	CGCCTTCAAGCACGTCGCAA	20	3	FGSG_10936	-
siRNA-t0183731	CGCCTTCCATGCTGCCGCGGAT	22	3	FGSG_04587	K00777|1|7e-77|289|afm:AFUA_7G07100|[EC:2.4.2.-];K14855|4|5e-55|216|lbc:LACBIDRAFT_296068|ribosome assembly protein 4
siRNA-t0170063	CGCGCCTTCCATGCTGCCGC	20	3	FGSG_04587	K00777|1|7e-77|289|afm:AFUA_7G07100|[EC:2.4.2.-];K14855|4|5e-55|216|lbc:LACBIDRAFT_296068|ribosome assembly protein 4
siRNA-t0199070	CGCGTCTACCTTGTTCAGGA	20	3	FgHV1gp2	-
siRNA-t0162501	CGCGTCTACCTTGTTCAGGAAT	22	3	FgHV1gp2	-
siRNA-t0177153	CGCTTCCCATGACTGCTAGA	20	3	FGSG_09177	-
siRNA-t0192700	CGGATCTCCGCTCCGATGTCCACA	24	3	FgHV1gp1	-
siRNA-t0173842	CGGCCGTTTGTGCTGCAAGATGGAC	25	3	FgHV1gp2	-
siRNA-t0198520	CGGCTATAGGAGTACCAACA	20	3	FGSG_01541	K02906|1|2e-37|155|cnb:CNBE2710|large subunit ribosomal protein L3
siRNA-t0170467	CGGCTTACCCTCGCATTTGCC	21	3	FgHV1gp2	-
siRNA-t0174369	CGGCTTACCCTCGCATTTGCCGCAC	25	3	FgHV1gp2	-
siRNA-t0176112	CGGGATCAGGTTCACAATTG	20	3	FGSG_08396	K01443|1|0.0|921|fgr:FG08396.1|N-acetylglucosamine-6-phosphate deacetylase [EC:3.5.1.25]
siRNA-t0164612	CGGGATCAGGTTCACAATTGCA	22	3	FgHV1gp2	-
siRNA-t0172992	CGGGATCAGGTTCACAATTGCAC	23	3	FgHV1gp2	-
siRNA-t0184898	CGGTTACCAAGGTGTGTGAA	20	3	FgHV1gp2	-
siRNA-t0155554	CGTAACGGGTCCAGATGTTGT	21	3	FgHV1gp2	-
siRNA-t0164968	CGTAACGGGTCCAGATGTTGTGCTT	25	3	FgHV1gp2	-
siRNA-t0187011	CGTACCGCAGTGATGGAGACTAA	23	3	FGSG_09869	K12669|1|0.0|647|fgr:FG09869.1|oligosaccharyltransferase complex subunit gamma
siRNA-t0154916	CGTACCTGCAGTCACAACTTGA	22	3	FGSG_01743	K01895|1|0.0|1310|fgr:FG01743.1|acetyl-CoA synthetase [EC:6.2.1.1]
siRNA-t0199520	CGTAGATGTGATGCCTTGTC	20	3	FgHV1gp2	-
siRNA-t0178636	CGTCAAATCAAGTTGGTATCCCTC	24	3	FgHV1gp2	-
siRNA-t0166068	CGTCATGATCATCAGCACCGTAC	23	3	FGSG_04291	K02999|1|0.0|3375|fgr:FG04291.1|DNA-directed RNA polymerase I subunit RPA1 [EC:2.7.7.6]
siRNA-t0199574	CGTCCGAATACCATACTTTCCGAA	24	3	FgHV1gp2	-
siRNA-t0151699	CGTGAACCAGATCATAACCCG	21	3	FgHV1gp2	-
siRNA-t0166332	CGTGACCAGCTGTTCTCTGTCGGC	24	3	FgHV1gp2	-
siRNA-t0156312	CGTGCGCTTCTGGATCAATGTCAG	24	3	FgHV1gp2	-
siRNA-t0166553	CGTGGAAATCTTCAGGCCCTG	21	3	FGSG_10199	-
siRNA-t0183272	CGTGGATTTTGCTACCAGCTGCAG	24	3	FgHV1gp1	-
siRNA-t0158282	CGTGTCGTTAGAAGCTTGAC	20	3	FgHV1gp2	-
siRNA-t0171998	CGTTAGAAGCTTGACACGAATGAT	24	3	FgHV1gp2	-
siRNA-t0194366	CTGAACCCAGTTGCTGTATC	20	3	FGSG_09660	K02677|1|0.0|1989|fgr:FG09660.1|classical protein kinase C [EC:2.7.11.13]
siRNA-t0168565	CTGATATATTCCAACTGCGACCCAT	25	3	FgHV1gp2	-
siRNA-t0179951	CTGATCTGGCTCGTGACTTTT	21	3	FgHV1gp2	-
siRNA-t0166498	CTGCAAGATGGACTGGCCTGTT	22	3	FgHV1gp2	-
siRNA-t0166814	CTGCCATAACCTCGTTGGGACCTTT	25	3	FgHV1gp2	-

siRNA-t0196295	CTGCCGCGGATAACCTATACT	21	3	FgHV1gp2	-
siRNA-t0199985	CTGCCGCGGATAACCTATACTT	22	3	FgHV1gp2	-
siRNA-t0189008	CTGCCGCGGATAACCTATACTTGA	24	3	FgHV1gp2	-
siRNA-t0168140	CTGCTGCGCTATGGTGTGAGAT	22	3	FGSG_08808	-
siRNA-t0166584	CTGCTTGTCAGTGTCAGGATC	21	3	FGSG_12704	K05658|1|0.0|1949|nhe:NECHADRAFT_96330|ATP-binding cassette, subfamily B (MDR/TAP), member 1
siRNA-t0195901	CTGCTTGTCAGTGTCAGGATCCGTC	25	3	FgHV1gp2	-
siRNA-t0153519	CTGGATCAATGTCAGCAGTGT	21	3	FGSG_06089	K08874|1|0.0|7852|fgr:FG06089.1|transformation/transcription domain-associated protein
siRNA-t0184970	CTGGCAATTGTGCGTTCGCG	20	3	FGSG_05002	K12818|1|0.0|2187|fgr:FG05002.1|ATP-dependent RNA helicase DHX8/PRP22 [EC:3.6.4.13]
siRNA-t0151441	GAAACCATCAGGACCCGAGAAAA	23	3	FgHV1gp2	-
siRNA-t0195742	GAAACCATCAGGACCCGAGAAAAA	24	3	FgHV1gp2	-
siRNA-t0157579	GAAACGACCCATACATGCCT	20	3	FGSG_03170	K00698|1|4e-24|112|cci:CC1G_10992|chitin synthase [EC:2.4.1.16]
siRNA-t0188332	GAAAGGAAATATCGGAAAATTGTG	24	3	FgHV1gp2	-
siRNA-t0171172	GAAAGTGAAAGTGTCGTAGG	20	3	FGSG_08013	-
siRNA-t0197027	GAACCACAACGATAGTATGAACC	23	3	FgHV1gp2	-
siRNA-t0160442	GAACCACAACGATAGTATGAACCC	24	3	FgHV1gp2	-
siRNA-t0169656	GAACCCAAGTACGTCGAAAG	20	3	FGSG_13824	K03380|1|1e-16|88.2|fgr:FG11194.1|phenol 2-monooxygenase [EC:1.14.13.7];K00777|2|6e-07|55.8|afm:AFUA_7G07100|[EC:2.4.2.-]
siRNA-t0156434	GAACCCTGACAATACCAAATATCCC	25	3	FgHV1gp2	-
siRNA-t0164800	GAACCCTTCCACGATTGGCGTGA	23	3	FgHV1gp2	-
siRNA-t0199209	GAATAAGCCACACCCTTAGGACCA	24	3	FgHV1gp2	-
siRNA-t0176169	GAATTCTAGCCTGGTTGAGATCCAT	25	3	FgHV1gp2	-
siRNA-t0169644	GAATTGCAACGAGGGCCCAGT	21	3	FGSG_07277	K01976|1|3e-32|139|sce:YBR222C|[EC:6.-.-.-];K01895|4|2e-19|96.7|mgr:MGG_04590|acetyl-CoA synthetase [EC:6.2.1.1];K01897|5|2e-19|96.7|ncr:NCU06063|long-chain acyl-CoA synthetase [EC:6.2.1.3]
siRNA-t0190372	GAATTTGGACAAGTACTCCACCT	23	3	FgHV1gp2	-
siRNA-t0180535	GACAATGGCGCATACACGGAACC	23	3	FgHV1gp2	-
siRNA-t0195394	GACAGTTCCGCGTGTGATTGGTCT	24	3	FgHV1gp2	-
siRNA-t0181454	GACATATTCGGACTCATCCCACATG	25	3	FgHV1gp2	-
siRNA-t0156880	GACATTTTCGCGCGCTGTAC	20	3	FGSG_02394	K01932|1|0.0|942|afm:AFUA_1G10380|[EC:6.3.2.-]
siRNA-t0195481	GACCCATGCTTTCGACGTAC	20	3	FGSG_08372	K10838|1|0.0|1448|fgr:FG08372.1|xeroderma pigmentosum group C-complementing protein
siRNA-t0190100	GACCCCAGATCCACCCGGATGAC	23	3	FgHV1gp2	-
siRNA-t0182733	GACCCCAGATCCACCCGGATGACTT	25	3	FgHV1gp2	-
siRNA-t0184097	GATACGTTCTGAGAAGTCTCC	21	3	FgHV1gp2	-
siRNA-t0176831	GATAGCCTCTCCCTTCGTGAACTG	24	3	FgHV1gp2	-
siRNA-t0166188	GATATTCGGTATTGTCGAGTCGAA	24	3	FgHV1gp2	-
siRNA-t0185883	GATATTGAACCCTCCTTCGTG	21	3	FgHV1gp2	-
siRNA-t0176844	GATCATCAGCACCGTACGGA	20	3	FGSG_04439	K01051|1|2e-45|185|lbc:LACBIDRAFT_245379|pectinesterase [EC:3.1.1.11]
siRNA-t0171632	GATCATGACGTGGAGAAGAAT	21	3	FGSG_07536	K03386|1|2e-83|307|ang:ANI_1_984094|peroxiredoxin (alkyl hydroperoxide reductase subunit C) [EC:1.11.1.15]
siRNA-t0201577	GATCCACCCGGATGACTTGGC	21	3	FgHV1gp2	-
siRNA-t0157886	GATCCTAAACATGTCGGTGG	20	3	FGSG_13878	K01932|1|0.0|1450|afm:AFUA_5G12730|[EC:6.3.2.-]
siRNA-t0194488	GATCTCTAGGATGATGCCAAAA	22	3	FGSG_11200	K00688|1|1e-06|55.5|pno:SNOG_13460|starch phosphorylase [EC:2.4.1.1]
siRNA-t0158059	GATCTCTGACATATTCGGAC	20	3	FgHV1gp2	-
siRNA-t0160282	GATCTCTGACATATTCGGACTCAT	24	3	FgHV1gp2	-
siRNA-t0198094	GATCTTCCAAGCGGTCATAAGT	22	3	FgHV1gp2	-
siRNA-t0171045	GATCTTCGTCACGCTTGCAT	20	3	FGSG_06837	-
siRNA-t0180115	GATGTAGGCTCCTCCGACAA	20	3	FGSG_11268	-
siRNA-t0189022	GATGTGGGTGCGCCGAATGAAAGCC	25	3	FgHV1gp2	-
siRNA-t0201203	GATGTTGTAGAACTTTTGCCACT	23	3	FgHV1gp2	-
siRNA-t0193692	GATTAATCAATGTACCGTTCGGC	23	3	FgHV1gp2	-
siRNA-t0191888	GATTTCGCAACCACAAGCCACTG	23	3	FgHV1gp2	-
siRNA-t0198968	GCAAAAGTCTATCACCATCCA	21	3	FGSG_04127	K02880|1|0.0|697|cci:CC1G_14988|large subunit ribosomal protein L17e
siRNA-t0160312	GCAACCACAAGCCACTGCAGT	21	3	FgHV1gp2	-
siRNA-t0160306	GCAACGGAAGCGATATTTGCACCA	24	3	FgHV1gp2	-
siRNA-t0158283	GCAAGTCTTCGTCCAGTAGCCAA	23	3	FgHV1gp2	-
siRNA-t0158533	GCAATGCAACAGACTTCTTC	20	3	FGSG_13783	K01932|1|0.0|1526|afm:AFUA_5G12730|[EC:6.3.2.-]
siRNA-t0181531	GCAATGGTACCATCACTCAGG	21	3	FGSG_07504	K11215|1|3e-11|70.1|pcs:Pc12g07060|transcription factor STE12
siRNA-t0187341	GCACATGAACACTGCTGACATTG	23	3	FGSG_06191	K01759|1|9e-06|52.4|uma:UM06328.1|lactoylglutathione lyase [EC:4.4.1.5]
siRNA-t0185704	GCACCAGGATTTGCTTCTTGCA	22	3	FgHV1gp2	-
siRNA-t0166128	GCACCATACAATGCCGCCAA	20	3	FgHV1gp2	-
siRNA-t0200587	GCACGAGGGACGAGCTTGGCCC	22	3	FgHV1gp2	-
siRNA-t0157553	GCAGACACTTGTTTCTCGTGG	21	3	FgHV1gp2	-
siRNA-t0163509	GCAGACACTTGTTTCTCGTGGG	22	3	FgHV1gp2	-
siRNA-t0160717	GCAGCGGCAAATACAGGACCGCGC	24	3	FgHV1gp2	-
siRNA-t0201675	GCAGGGTGCTCATGAGGTTGGCAT	24	3	FgHV1gp2	-
siRNA-t0178220	GCAGTGTTCATGTGCACATTC	21	3	FGSG_02315	K01932|1|0.0|2455|afm:AFUA_1G10380|[EC:6.3.2.-]
siRNA-t0192332	GCAGTTGGTCCGCGAATTCTGCCAT	25	3	FgHV1gp2	-
siRNA-t0161817	GCATGGCCGGCAGTCCTAGCAAGTG	25	3	FgHV1gp2	-
siRNA-t0185066	GCATGTTCTTCGTGTAGAAAGT	22	3	FgHV1gp2	-
siRNA-t0173959	GCATTCCTCAAGGAGCTCAAA	21	3	FGSG_09306	K11644|1|0.0|2881|fgr:FG09306.1|paired amphipathic helix protein Sin3a
siRNA-t0171335	GCATTTGTTTGTACGTCCGAA	21	3	FGSG_11659	K01932|1|0.0|1431|afm:AFUA_5G12730|[EC:6.3.2.-]
siRNA-t0168954	GCCAGCATAAGCCATCGCAT	20	3	FGSG_00232	-
siRNA-t0182040	GCCAGGTAGATGACCGACAACAA	23	3	FgHV1gp2	-
siRNA-t0150821	GCCATAGCGCTGGTGTTGAA	20	3	FgHV1gp2	-
siRNA-t0195586	GCCATCAACGGTTCTTGGAAAGT	23	3	FgHV1gp2	-
siRNA-t0157934	GCCATCAACGGTTCTTGGAAAGTT	24	3	FgHV1gp2	-
siRNA-t0190318	GCCATCATGTATAGTCCCTCA	21	3	FgHV1gp2	-
siRNA-t0164208	GCCATCCTATAGATCGCGCGTAAG	24	3	FgHV1gp2	-
siRNA-t0165878	GCCATTCGTTACAATCGTGGA	21	3	FgHV1gp2	-
siRNA-t0155208	GCCATTGTCGGAGGAGCCTA	20	3	FGSG_13700	-
siRNA-t0179368	GCCCAGTCATGAATTTTCGGTAG	23	3	FgHV1gp2	-
siRNA-t0175285	GCCCTCAGTAGTCGACATGTCA	22	3	FGSG_02945	K13095|1|6e-68|257|fgr:FG02080.1|splicing factor 1;K08192|2|2e-64|245|dha:DEHA2C12276g|MFS transporter, ACS family, allantoate permease
siRNA-t0185214	GCCGACGCTCCACTCGCTCT	20	3	FGSG_09991	K11204|1|0.0|1353|fgr:FG09991.1|glutamate--cysteine ligase catalytic subunit [EC:6.3.2.2]
siRNA-t0165158	GCCGACGTCTACGTAAGGCGTCGA	24	3	FgHV1gp2	-
siRNA-t0176720	GCCGCACAGCTCCAATAATCGT	22	3	FgHV1gp2	-
siRNA-t0154426	GCCGGCAGTCCTAGCAAGTGAA	22	3	FgHV1gp2	-
siRNA-t0175177	GCCGGTTACCAAGGTGTGTGAA	22	3	FgHV1gp2	-
siRNA-t0179443	GCCGTAAGTAGCAGATTTTACAA	23	3	FgHV1gp2	-
siRNA-t0157403	GCCTATTCATCGTCAATGAC	20	3	FGSG_08065	K00100|1|1e-113|408|afm:AFUA_7G04850|[EC:1.1.1.-]
siRNA-t0175718	GCCTCTCCCTTCGTGAACTG	20	3	FGSG_08897	K12819|1|0.0|766|fgr:FG08897.1|pre-mRNA-processing factor SLU7
siRNA-t0193696	GCCTGATGTTCTTGTGCCTCC	21	3	FGSG_02044	-
siRNA-t0165973	GCGAACGGGACCGACGTTTTGGCG	24	3	FgHV1gp2	-
siRNA-t0159280	GCGAATTCTGCCATAACCTC	20	3	FgHV1gp2	-
siRNA-t0152755	GCGAGACCGATGATTTCCATC	21	3	FGSG_12863	K02324|1|0.0|3986|fgr:FG06065.1|DNA polymerase epsilon subunit 1 [EC:2.7.7.7]
siRNA-t0179527	GCGCTTCCCATGACTGCTAG	20	3	FGSG_05958	K01684|1|2e-11|70.5|ani:AN4828.2|galactonate dehydratase [EC:4.2.1.6];K08773|2|5e-06|52.4|fgr:FG01071.1|RalA-binding protein 1
siRNA-t0172521	GCGGCAAATACAGGACCGCGCGA	23	3	FgHV1gp2	-
siRNA-t0156911	GCGGGACCAGGATTCTTCTC	20	3	FGSG_13060	-
siRNA-t0177364	GCGGGACCAGGATTCTTCTCCA	22	3	FGSG_08844	K01183|1|1e-06|55.1|pan:PODANSg4492|chitinase [EC:3.2.1.14]

siRNA-t0161007	GCTCGCACCTTTCTTCTCTCA	21	3	FGSG_04522	-
siRNA-t0150942	GCTGATCTGGCTCGTGACTTTTCC	24	3	FgHV1gp2	-
siRNA-t0200118	GCTGCATGTACTGTTCGGCAACAGA	25	3	FgHV1gp2	-
siRNA-t0184145	GCTGCCGCGGATAACCTATA	20	3	FgHV1gp2	-
siRNA-t0166514	GCTGTAGTGTCTGAAGATCTCA	22	3	FgHV1gp2	-
siRNA-t0182913	GCTTACCCTCGCATTTGCCGCACA	24	3	FgHV1gp2	-
siRNA-t0152845	GCTTCACACCCGTCCACGTCT	21	3	FGSG_02315	K01932|1|0.0|2455|afm:AFUA_1G10380|[EC:6.3.2.-]
siRNA-t0189509	GCTTTCGACGTACTTGGGTTCAT	23	3	FgHV1gp2	-
siRNA-t0200723	GGAAATCTTCAGGCCCTGGGATTG	24	3	FgHV1gp2	-
siRNA-t0194062	GGACAAGCCGTCAAACATAGA	21	3	FgHV1gp2	-
siRNA-t0197387	GGACTTGAAATCCTGGGCGCTAA	23	3	FgHV1gp2	-
siRNA-t0174558	GGAGACAGAGCCTGAGTCAT	20	3	FGSG_06397	K01238|1|2e-48|191|afm:AFUA_8G06830|[EC:3.2.1.-];K01179|4|3e-15|81.6|afm:AFUA_3G03870|endoglucanase [EC:3.2.1.4]
siRNA-t0160582	GGATCGGTGGCACACGCATAAG	22	3	FGSG_13987	-
siRNA-t0171555	GGATGGTCATGATATGGAAA	20	3	FGSG_06013	-
siRNA-t0163781	GGATGTTTGCTTGGTTTCGG	20	3	FGSG_11770	-
siRNA-t0158274	GGATTAGCCATTCGTTACAATCGTG	25	3	FgHV1gp2	-
siRNA-t0150946	GGATTCCTTTATGCCAGCAT	20	3	FGSG_04859	K00777|1|2e-86|320|afm:AFUA_7G07100|[EC:2.4.2.-];K14963|2|8e-15|82.8|dha:DEHA2E05016g|COMPASS component SWD3;K06666|3|2e-14|81.6|pcs:Pc12g04590|glucose repression regulatory protein TUP1;K03361|5|5e-14|80.5|yli:YALI0A09658g|F-box and WD-40 domain protein CDC4
siRNA-t0171369	GGATTTGAGGGCGAGTGGAGCTAC	24	3	FgHV1gp2	-
siRNA-t0197737	GGCAACGGAAGCGATATTTGCACC	24	3	FgHV1gp2	-
siRNA-t0177423	GGCACCAACTTCAAGAACCC	20	3	FGSG_04694	K15419|1|0.0|1075|ani:AN2547.2|emericellamide biosynthesis, highly reducing iterative type I polyketide synthase;K00680|3|0.0|812|afm:AFUA_3G14700|[EC:2.3.1.-]
siRNA-t0162468	GGCAGTCCTAGCAAGTGAAACTG	23	3	FgHV1gp2	-
siRNA-t0193337	GGCCAAATCGTCATACATTG	20	3	FgHV1gp2	-
siRNA-t0160591	GGCGGGACCAGGATTCTTCT	20	3	FGSG_08844	K01183|1|1e-06|55.1|pan:PODANSg4492|chitinase [EC:3.2.1.14]
siRNA-t0192153	GGCGGTTCGAGTAGCAGCTTA	21	3	FGSG_05041	-
siRNA-t0165280	GGCGTCGACACGATGATACCCTC	23	3	FgHV1gp2	-
siRNA-t0155501	GGCTCGTGACTTTTCCCTCACA	22	3	FGSG_00581	K01598|1|2e-13|77.0|uma:UM05916.1|phosphopantothenoylcysteine decarboxylase [EC:4.1.1.36]
siRNA-t0154586	GGGAAATTAGTCTTCGGATG	20	3	FGSG_10464	K15394|1|0.0|1754|act:ACLA_078660|hybrid polyketide synthase / nonribosomal peptide synthetase ACE1
siRNA-t0188681	GGGTCTCCGATATTCGCTTCGCAT	24	3	FgHV1gp1	-
siRNA-t0193656	GGGTGCGCCGAATGAAAGCCCAGT	24	3	FgHV1gp2	-
siRNA-t0185999	GGGTGCTCATGAGGTTGGCA	20	3	FGSG_07519	-
siRNA-t0153120	GGGTTCTTGAAGTTGGTGCCGACG	24	3	FgHV1gp2	-
siRNA-t0167269	GGTAACGACCCCTGAAAGCC	20	3	FGSG_10464	K15394|1|0.0|1754|act:ACLA_078660|hybrid polyketide synthase / nonribosomal peptide synthetase ACE1
siRNA-t0172650	GGTAACGACCCCTGAAAGCCT	21	3	FgHV1gp1	-
siRNA-t0170028	GGTAATCCAGAGCTCTTGGCA	21	3	FGSG_10548	K15418|1|0.0|1191|pcs:Pc21g12440|zearalenone synthase, highly reducing iterative type I polyketide synthase;K15419|4|0.0|740|ani:AN2547.2|emericellamide biosynthesis, highly reducing iterative type I polyketide synthase
siRNA-t0199992	GGTTATCCGCGGCAGCATGG	20	3	FGSG_13033	K14766|1|0.0|1149|nhe:NECHADRAFT_37909|nucleolar protein 14
siRNA-t0198036	GGTTATGATCTGGTTCACGCT	21	3	FGSG_11696	K11701|1|3e-52|207|tml:GSTUM_00000065001|helicase required for RNAi-mediated heterochromatin assembly 1 [EC:3.6.4.13]
siRNA-t0172221	GGTTCTTGAAGTTGGTGCCGA	21	3	FgHV1gp2	-
siRNA-t0178454	GGTTTGAATGGTCTGCCTAATCT	23	3	FGSG_08405	K03657|1|0.0|1811|fgr:FG08405.1|DNA helicase II / ATP-dependent DNA helicase PcrA [EC:3.6.4.12]
siRNA-t0187430	GTAAGCTGCTACTCGAACCGCCC	23	3	FgHV1gp2	-
siRNA-t0170950	GTAAGTGACGAGGCCTCTGACGT	23	3	FgHV1gp2	-
siRNA-t0196984	GTACCAACAGCGGAGATATTCACG	24	3	FGSG_00989	K06036|1|3e-170|597|afm:AFUA_4G03780|[EC:4.2.2.-]
siRNA-t0160553	GTACCGTTCGGCAACAGATC	20	3	FgHV1gp2	-
siRNA-t0196346	GTACGTCCGAATACCATACTTT	22	3	FgHV1gp2	-
siRNA-t0177556	GTACGTGGAAATCTTCAGGCCCTG	24	3	FgHV1gp2	-
siRNA-t0174870	GTCATTCTCTTCCAGTGTCGAACCA	25	3	FgHV1gp2	-
siRNA-t0168458	GTCCGAATACCATACTTTCCGA	22	3	FgHV1gp2	-
siRNA-t0169000	GTCTGACTGTTGCTTGATGGTTG	23	3	FgHV1gp2	-
siRNA-t0161985	GTGAACCAGATCATAACCCGCA	22	3	FgHV1gp2	-
siRNA-t0159918	GTGAACTTACGTATCGCTGATCTG	24	3	FgHV1gp2	-
siRNA-t0192526	GTGACGATACTTGAACCAAG	20	3	FGSG_13783	K01932|1|0.0|1526|afm:AFUA_5G12730|[EC:6.3.2.-]
siRNA-t0197292	GTGATGCATTGCTAGAGTGGG	21	3	FGSG_07262	K14550|1|0.0|3445|fgr:FG07262.1|U3 small nucleolar RNA-associated protein 10
siRNA-t0181574	GTGATTGGTCTCTTGCAGTGCA	22	3	FgHV1gp2	-
siRNA-t0177505	GTGCGACTTCCGCAACGACGGGTT	24	3	FgHV1gp2	-
siRNA-t0150978	GTGGCCGTAAGTAGCAGATTTTA	23	3	FgHV1gp2	-
siRNA-t0151593	GTGGGGTCACATATCTCGAT	20	3	FgHV1gp2	-
siRNA-t0177868	GTGTGGGTCTTCTGACGAATTT	22	3	FgHV1gp2	-
siRNA-t0182699	GTGTGTATCCAATGAGTTGTGGGA	24	3	FgHV1gp2	-
siRNA-t0160463	GTTACAATCGTGGACTTCCTTT	22	3	FgHV1gp2	-
siRNA-t0182871	GTTAGAAGCTTGACACGAATGAT	23	3	FgHV1gp2	-
siRNA-t0174981	GTTCACGAAGGGAGAGGCTA	20	3	FGSG_10561	K01183|1|0.0|635|afm:AFUA_5G03960|chitinase [EC:3.2.1.14]
siRNA-t0184399	GTTCATTGCGATGTAATCTTGAA	23	3	FgHV1gp2	-
siRNA-t0159006	GTTCCATAAACGCTGCCGGGATGT	24	3	FgHV1gp2	-
siRNA-t0182688	GTTCCGCGTGTGATTGGTCTCTTG	24	3	FgHV1gp2	-
siRNA-t0170218	GTTCGCATCCGTTCTGTGAA	20	3	FGSG_08999	-
siRNA-t0163982	GTTCTTCGTGTAGAAAGTCTTTT	23	3	FgHV1gp2	-
siRNA-t0186291	GTTGAAGAGTGAGACTTTCG	20	3	FGSG_06379	K14289|1|0.0|2450|fgr:FG06379.1|exportin-5
siRNA-t0161391	GTTGAGAACGCTGAAATCGAG	21	3	FgHV1gp2	-
siRNA-t0161754	GTTGCGAAATCGAGATATGTGAC	23	3	FGSG_00034	K08141|1|2e-129|462|dha:DEHA2B16104g|MFS transporter, SP family, general alpha glucoside:H+ symporter
siRNA-t0155106	TAAGTCTGGAATTCCTTGGGAGG	23	3	FgHV1gp2	-
siRNA-t0155266	TAAGTCTGGAATTCCTTGGGAGGC	24	3	FgHV1gp2	-
siRNA-t0172147	TAAGTCTGGAATTCCTTGGGAGGCC	25	3	FgHV1gp2	-
siRNA-t0195204	TAATCGTTGAAAATCAGAGAGCA	23	3	FgHV1gp2	-
siRNA-t0186669	TAATGCATGTTACTAAGAAGAGCGA	25	3	FgHV1gp2	-
siRNA-t0176678	TACACGACGTACTTAGGGTT	20	3	FgHV1gp2	-
siRNA-t0166878	TACAGGACCGCGCGATGGCGCGCG	24	3	FgHV1gp2	-
siRNA-t0188216	TACATGCCTGATGTTCTTGT	20	3	FgHV1gp2	-
siRNA-t0182417	TACCGATATTGAACCCTCCTTC	22	3	FgHV1gp2	-
siRNA-t0151812	TACGGATGTAGGTCTGGCATA	21	3	FGSG_04545	K03380|1|3e-09|63.9|fgr:FG11194.1|phenol 2-monooxygenase [EC:1.14.13.7]
siRNA-t0185969	TACTATCGTTGTGGTTCTTTGG	22	3	FGSG_07258	K14572|1|0.0|8396|fgr:FG07258.1|midasin
siRNA-t0163525	TACTTGATCTCAGCACGCCGGTTAC	25	3	FgHV1gp2	-
siRNA-t0154395	TACTTGCCGCGCGTCTCACT	20	3	FGSG_06580	K11262|1|0.0|4510|fgr:FG06580.1|acetyl-CoA carboxylase / biotin carboxylase [EC:6.4.1.2 6.3.4.14]
siRNA-t0186581	TAGAGATCTCCGCAGAAGAAT	21	3	FGSG_06836	K01259|1|7e-121|433|afm:AFUA_2G05000|proline iminopeptidase [EC:3.4.11.5]
siRNA-t0183240	TAGAGCCAAGTCATCCGGGT	20	3	FGSG_12996	K00428|1|9e-23|105|ani:AN1630.2|cytochrome c peroxidase [EC:1.11.1.5]
siRNA-t0178709	TAGCCATTCGTTACAATCGTGGA	23	3	FgHV1gp2	-
siRNA-t0180929	TAGCCTCTCCCTTCGTGAACTGCT	24	3	FgHV1gp2	-
siRNA-t0161995	TAGCCTGGTTGAGATCCATGC	21	3	FGSG_10524	K15458|1|0.0|2091|fgr:FG10524.1|SIT4-associating protein SAP185/190
siRNA-t0181468	TAGTAAAGTTATCGTGTCTGA	21	3	FgHV1gp2	-
siRNA-t0181757	TAGTAAAGTTATCGTGTCTGAC	22	3	FgHV1gp2	-

siRNA-t0163105	TAGTCATCGTCAGGGTACGT	20	3	FGSG_09801	K03164|1|0.0|2817|fgr:FG09801.1|DNA topoisomerase II [EC:5.99.1.3]
siRNA-t0185364	TAGTCTCCATCACTGCGGTA	20	3	FGSG_04033	-
siRNA-t0172151	TATCGAAACTGGCAGGAGTT	20	3	FGSG_01429	K14309|1|0.0|2016|fgr:FG01429.1|nuclear pore complex protein Nup93
siRNA-t0178272	TATCTACGCACTCACGCCAA	20	3	FGSG_11797	K06681|1|5e-08|59.3|pgu:PGUG_03987|Gdp/GTP exchange factor required for growth at low temperatures
siRNA-t0167346	TATGAACCCTTCCACGATTGGCGT	24	3	FgHV1gp2	-
siRNA-t0189449	TATGTGGAGTTCCTTCGGAA	20	3	FGSG_04291	K02999|1|0.0|3375|fgr:FG04291.1|DNA-directed RNA polymerase I subunit RPA1 [EC:2.7.7.6]
siRNA-t0155221	TATGTTTGACGGCTTGTCCTA	21	3	FGSG_13806	K01187|1|0.0|649|fgr:FG10662.1|alpha-glucosidase [EC:3.2.1.20]
siRNA-t0161581	TCAAATCAAGTTGGTATCCCTCC	23	3	FgHV1gp2	-
siRNA-t0166645	TCAACACCAGCGCTATGGCTA	21	3	FGSG_07497	-
siRNA-t0182381	TCAAGGATTTGTAAGGGGAAAC	22	3	FgHV1gp2	-
siRNA-t0152410	TCATGGGCAAAAGACAGATAGGTC	24	3	FgHV1gp2	-
siRNA-t0197239	TCATTCGACTGTGGTTCAAT	20	3	FGSG_09607	K10413|1|0.0|8782|fgr:FG09607.1|dynein heavy chain 1, cytosolic
siRNA-t0199765	TCATTGCGATGTAATCTTGAA	21	3	FgHV1gp2	-
siRNA-t0183089	TCATTGCGATGTAATCTTGAATA	23	3	FgHV1gp2	-
siRNA-t0173457	TCCAAACTGTTCATTTCGGCGAG	23	3	FgHV1gp2	-
siRNA-t0179567	TCCAGACGGCCAAATCCTCA	20	3	FGSG_06161	K14850|1|0.0|818|fgr:FG06161.1|ribosomal RNA-processing protein 8 [EC:2.1.1.-]
siRNA-t0157626	TCCAGATCTTATAGACAGGCCCAAA	25	3	FgHV1gp2	-
siRNA-t0162042	TCCAGTGTCGAACCATACTGG	21	3	FgHV1gp2	-
siRNA-t0155667	TCCATAAACGCTGCCGGGATGT	22	3	FgHV1gp2	-
siRNA-t0157413	TCCCTCATACTTCGCCTTCA	20	3	FGSG_03607	K05349|1|0.0|637|pte:PTT_17365|beta-glucosidase [EC:3.2.1.21]
siRNA-t0193253	TCCCTTCGTGAACTGCTGCGCT	22	3	FgHV1gp2	-
siRNA-t0165294	TCCGCGAATTCTGCCATAACCTCGT	25	3	FgHV1gp2	-
siRNA-t0158719	TCCGGGATCAGGTTCACAATTGC	23	3	FgHV1gp2	-
siRNA-t0193916	TCCGTACGGTGCTGATGATC	20	3	FGSG_11890	K08773|1|0.0|1645|fgr:FG01071.1|RalA-binding protein 1
siRNA-t0189215	TCCTAGAGATCTCCGCAGAAGAATT	25	3	FGSG_00352	K08064|1|2e-78|291|mgr:MGG_00354|nuclear transcription factor Y, alpha
siRNA-t0158500	TCCTAGCAAGTGAAACTGTTCGCC	24	3	FgHV1gp2	-
siRNA-t0170874	TCCTAGCAAGTGAAACTGTTCGCCA	25	3	FgHV1gp2	-
siRNA-t0163992	TCCTCTGATATATTCCAACT	20	3	FgHV1gp2	-
siRNA-t0153357	TCCTTGCGTGGTCGTGTGAG	20	3	FGSG_11123	K01130|1|9e-25|114|yli:YALI0D26488g|arylsulfatase [EC:3.1.6.1]
siRNA-t0164630	TCCTTTCCTACGACACTTTCA	21	3	FGSG_01388	K05681|1|0.0|1551|mgr:MGG_01563|ATP-binding cassette, subfamily G (WHITE), member 2;K08711|2|7e-43|175|cne:CNB05770|ATP-binding cassette, subfamily G (WHITE), member 2, PDR
siRNA-t0160535	TCGAAGGTTAGAAGTTCTCT	20	3	FGSG_05996	K00936|1|3e-61|238|afm:AFUA_6G10240|[EC:2.7.3.-];K11231|2|6e-39|163|afm:AFUA_2G00660|osomolarity two-component system, sensor histidine kinase SLN1 [EC:2.7.13.3];K02486|3|2e-38|161|spo:SPAC27E2.09|two-component system, unclassified family, sensor kinase [EC:2.7.13.3]
siRNA-t0197630	TCTTCAGCGTAACGTGACCAG	21	3	FgHV1gp2	-
siRNA-t0158555	TCTTCAGGCCCTGGGATTGT	20	3	FGSG_04694	K15419|1|0.0|1075|ani:AN2547.2|emericellamide biosynthesis, highly reducing iterative type I polyketide synthase;K00680|3|0.0|812|afm:AFUA_3G14700|[EC:2.3.1.-]
siRNA-t0176825	TCTTCGATGGATGCTGCGAA	20	3	FGSG_07924	-
siRNA-t0156207	TCTTCGTCCAGTAGCCAATGAGA	23	3	FgHV1gp2	-
siRNA-t0168560	TCTTCTTTGTGAACATCTGGA	21	3	FGSG_13766	-
siRNA-t0165980	TGAAACCATCAGGACCCGAGAAA	23	3	FgHV1gp2	-
siRNA-t0153800	TGAGTGCCATGACGTACGACCC	22	3	FgHV1gp1	-
siRNA-t0153026	TGATACAAACCGGAATGCAC	20	3	FgHV1gp2	-
siRNA-t0199421	TGATCATCAGCACCGTACGGATCC	24	3	FgHV1gp2	-
siRNA-t0193424	TGATCCTTCCACTTCCCTGGA	21	3	FGSG_10042	K11231|1|0.0|2053|fgr:FG10042.1|osomolarity two-component system, sensor histidine kinase SLN1 [EC:2.7.13.3]
siRNA-t0189450	TGATCTGGCCGGTTGAAAATG	21	3	FgHV1gp2	-
siRNA-t0161019	TGATGGCACGTCGACAGGCG	20	3	FgHV1gp2	-
siRNA-t0151313	TGATGGTTGGCACAACGATGAGCAC	25	3	FgHV1gp2	-
siRNA-t0192091	TGATTGATTCTAGCTTGTGCA	21	3	FgHV1gp2	-
siRNA-t0159669	TGATTGATTCTAGCTTGTGCAA	22	3	FgHV1gp2	-
siRNA-t0184727	TGATTTCCAAAGGTACCTGA	20	3	FGSG_02035	K00777|1|1e-24|115|afm:AFUA_7G06290|[EC:2.4.2.-]
siRNA-t0200402	TGATTTCCAAAGGTACCTGAG	21	3	FgHV1gp2	-
siRNA-t0201685	TGCAAAAATGACTCAGGCTCTG	22	3	FGSG_01437	-
siRNA-t0165041	TGCAGCGTGAACCAGATCATA	21	3	FgHV1gp2	-
siRNA-t0152073	TGCAGTTGGTCCGCGAATTC	20	3	FGSG_07715	K12584|1|3e-39|161|ure:UREG_00738|scavenger mRNA-decapping enzyme DcpS [EC:3.-.-.-]
siRNA-t0183621	TGCCGACGCTCCACTCGCTCT	21	3	FGSG_09965	K14300|1|0.0|2662|fgr:FG09965.1|nuclear pore complex protein Nup133
siRNA-t0163435	TGCCTCGTTCCCAACTCCTGC	21	3	FGSG_03996	K01113|1|1e-132|473|cim:CIMG_03455|alkaline phosphatase D [EC:3.1.3.1];K10402|2|1e-36|154|lbc:LACBIDRAFT_379819|kinesin family member 20/23
siRNA-t0194948	TGCGACGACCGTGCGAAATTG	21	3	FgHV1gp2	-
siRNA-t0170709	TGCGACGACCGTGCGAAATTGTTT	24	3	FgHV1gp2	-
siRNA-t0192110	TGCGCACATATCTCCTTGAA	20	3	FgHV1gp2	-
siRNA-t0173812	TGCGCACATATCTCCTTGAACT	22	3	FgHV1gp2	-
siRNA-t0188415	TGCGCACATATCTCCTTGAACTT	23	3	FgHV1gp2	-
siRNA-t0190605	TGCGCTTCTGGATCAATGTCA	21	3	FGSG_08541	K15134|1|0.0|1199|fgr:FG08541.1|mediator of RNA polymerase II transcription subunit 17, fungi type
siRNA-t0182021	TGCGTACCTGCAGTCACAACTTG	23	3	FGSG_01743	K01895|1|0.0|1310|fgr:FG01743.1|acetyl-CoA synthetase [EC:6.2.1.1]
siRNA-t0174842	TGCGTTCACACGATTCCCTGA	21	3	FgHV1gp2	-
siRNA-t0201183	TGCTGAATGCTCTGCCTCGG	20	3	FGSG_09306	K11644|1|0.0|2881|fgr:FG09306.1|paired amphipathic helix protein Sin3a
siRNA-t0173895	TGCTTGAAGGCGAAGTATGAGGGAG	25	3	FgHV1gp2	-
siRNA-t0173908	TGCTTTCGACGTACTTGGGTTCA	23	3	FgHV1gp2	-
siRNA-t0186459	TGGAACATTTTCAACCGGCC	20	3	FgHV1gp2	-
siRNA-t0174580	TGGAAGAATCATAACCGACG	20	3	FGSG_09801	K03164|1|0.0|2817|fgr:FG09801.1|DNA topoisomerase II [EC:5.99.1.3]
siRNA-t0186014	TGGAAGTGTTCGCATCCGTT	20	3	FGSG_10136	-
siRNA-t0167662	TGGAATAGCGGTTGTCTTATA	21	3	FgHV1gp2	-
siRNA-t0163308	TGGAGCGTCGGCAAATTTGG	20	3	FGSG_11659	K01932|1|0.0|1431|afm:AFUA_5G12730|[EC:6.3.2.-]
siRNA-t0182618	TGGCCAAATCGTCATACATTGCACG	25	3	FgHV1gp2	-
siRNA-t0173097	TGGCCGGCAGTCCTAGCAAGTGAA	24	3	FgHV1gp2	-
siRNA-t0152842	TGGCGGGACCAGGATTCTTCTCCA	24	3	FGSG_08844	K01183|1|1e-06|55.1|pan:PODANSg4492|chitinase [EC:3.2.1.14]
siRNA-t0199349	TGGCTCCACAAACGCTCATGA	21	3	FGSG_13896	-
siRNA-t0200362	TGGGACCATCATGATGAAACCGAAA	25	3	FgHV1gp2	-
siRNA-t0175984	TGGGATGGGTTTCTGGTTCCAAT	23	3	FGSG_13796	K14338|1|8e-09|60.8|fgr:FG01972.1|cytochrome P450 / NADPH-cytochrome P450 reductase [EC:1.14.14.1 1.6.2.4];K00517|2|4e-08|58.9|afm:AFUA_3G14760|[EC:1.14.-.-];K00493|4|1e-07|56.6|pic:PICST_35590|unspecific monooxygenase [EC:1.14.14.1]
siRNA-t0197278	TGGGATTGGGCGTGTGTTCTTT	22	3	FgHV1gp2	-
siRNA-t0158367	TGGGCGTGTGTTCTTTATGGGC	22	3	FGSG_05370	K09240|1|8e-06|52.0|cdu:CD36_07720|transcriptional regulatory protein CAT8
siRNA-t0196869	TGGGCTTTCATTCGGCGCAC	20	3	FGSG_07689	K01190|1|0.0|1059|bfu:BC1G_06897|beta-galactosidase [EC:3.2.1.23]
siRNA-t0196952	TGGGGTCTGACCGAGGCAGA	20	3	FGSG_10464	K15394|1|0.0|1754|act:ACLA_078660|hybrid polyketide synthase / nonribosomal peptide synthetase ACE1
siRNA-t0159921	TGGTACCATTGCTGCATGTACTGTT	25	3	FgHV1gp2	-
siRNA-t0184651	TGGTCTTTCTTCGGTCTAGG	20	3	FGSG_14002	-
siRNA-t0200176	TGGTTGGCACAACGATGAGCAC	22	3	FgHV1gp2	-
siRNA-t0153823	TGTAAGTGACGAGGCCTCTGA	21	3	FgHV1gp2	-
siRNA-t0169471	TGTACGTCCGAATACCATACT	21	3	FgHV1gp2	-
siRNA-t0172802	TGTCAGTGTCAGGATCCGTCC	21	3	FGSG_05487	K14318|1|2e-25|117|cci:CC1G_01275|nuclear pore complex protein Nup88
siRNA-t0159092	TGTCGAGATACTCTTCAGCGT	21	3	FgHV1gp2	-

siRNA-t0196123	TGTCTATTGAAGGTGGGTAATT	22	3	FGSG_10824	-
siRNA-t0162347	TGTTATAAGGCTTGTCGTCGGG	22	3	FGSG_08614	K01874|1|0.0|1256|fgr:FG08614.1|methionyl-tRNA synthetase [EC:6.1.1.10]
siRNA-t0150893	TGTTGCTTGATGGTTGGCACAACGA	25	3	FgHV1gp2	-
siRNA-t0189318	TGTTGTAGAACTTTTGCCACT	21	3	FGSG_07747	-
siRNA-t0188185	TTAATCAATGTACCGTTCGGCAA	23	3	FgHV1gp2	-
siRNA-t0191246	TTCGTTGAATCGATCGTCATG	21	3	FGSG_11372	K00777|1|5e-21|103|afm:AFUA_7G06290|[EC:2.4.2.-];K12823|4|1e-10|69.7|ure:UREG_02211|ATP-dependent RNA helicase DDX5/DBP2 [EC:3.6.4.13]
siRNA-t0158739	TTCTACCAGAACCCAGCACCTTTCC	25	3	FgHV1gp2	-
siRNA-t0182693	TTCTCTCTGACCTTGATGGCGTCA	24	3	FGSG_09454	K14293|1|0.0|1735|fgr:FG09454.1|importin subunit beta-1
siRNA-t0170609	TTCTCTTTGTTGGCCTGGTA	20	3	FGSG_09560	-
siRNA-t0156800	TTCTGAAACCATCAGGACCCGA	22	3	FgHV1gp2	-
siRNA-t0180899	TTCTGAAATTTGATGTTGTAG	21	3	FGSG_09445	K01210|1|0.0|1595|fgr:FG09445.1|glucan 1,3-beta-glucosidase [EC:3.2.1.58]
siRNA-t0189033	TTCTGACGAATTTGGACAAGTAC	23	3	FgHV1gp2	-
siRNA-t0182694	TTCTGCCATAACCTCGTTGGGA	22	3	FgHV1gp2	-
siRNA-t0183688	TTCTGTCTGGTACCGATATT	20	3	FGSG_13162	-
siRNA-t0152929	TTCTTGGAAAGTTGGAGCCCTTC	23	3	FgHV1gp2	-
siRNA-t0177853	TTGAAATCCTGGGCGCTAACAA	22	3	FgHV1gp2	-
siRNA-t0190826	TTGAACCCTGACAATACCAAAT	22	3	FgHV1gp2	-
siRNA-t0189314	TTGAACCCTGACAATACCAAATA	23	3	FgHV1gp2	-
siRNA-t0153331	TTGAGACAGTTCCGCGTGTGA	21	3	FgHV1gp2	-
siRNA-t0186070	TTGATCTCAGCACGCCGGTT	20	3	FGSG_10904	K06681|1|1e-61|238|cdu:CD36_21160|Gdp/GTP exchange factor required for growth at low temperatures
siRNA-t0169936	TTGATCTCAGCACGCCGGTTAC	22	3	FgHV1gp2	-
siRNA-t0176653	TTGCACGAGGGACGAGCTTGGC	22	3	FgHV1gp2	-
siRNA-t0171865	TTGCCTGGGAGACAGAGCCTG	21	3	FgHV1gp2	-
siRNA-t0162333	TTGCTTGGTTTCGGGACGCGA	21	3	FgHV1gp2	-
siRNA-t0188686	TTGGCCCCAGCAGACTTGGA	20	3	FGSG_12904	K12600|1|0.0|2824|fgr:FG06322.1|superkiller protein 3
siRNA-t0181326	TTGGCCCCAGCAGACTTGGAA	21	3	FGSG_12904	K12600|1|0.0|2824|fgr:FG06322.1|superkiller protein 3
siRNA-t0184895	TTGGGCGTGTGTTCTTTATGGGCTC	25	3	FgHV1gp2	-
siRNA-t0158569	TTGGTCCGCGAATTCTGCCA	20	3	FgHV1gp2	-
siRNA-t0170403	TTGTACTTGCCGCGCGTCTCACTGA	25	3	FgHV1gp2	-
siRNA-t0179370	TTGTAGAAATAACCACATTTTCCCG	25	3	FgHV1gp2	-
siRNA-t0189085	TTGTCAGTGTCAGGATCCGTCCAGG	25	3	FgHV1gp2	-
siRNA-t0186213	TTGTTCTGGAACGTCAAATCAAGTT	25	3	FgHV1gp2	-
siRNA-t0161359	TTGTTGGTCTTCCAGGCATCA	21	3	FGSG_11505	-
siRNA-t0204414	AAAACGCGCCTCCAGTCCAGCCTT	24	2	FgHV1gp2	-
siRNA-t0329942	AAAAGTCACGAGCCAGATCAGC	22	2	FGSG_04598	K05393|1|7e-104|377|aor:AOR_1_1352054|chloride channel, other eukaryote
siRNA-t0267281	AAACCCATCCCACTCGTGAACCT	23	2	FgHV1gp2	-
siRNA-t0211124	AAACGCCCCTGGACGGTACTTCTG	24	2	FgHV1gp2	-
siRNA-t0202169	AAACGCGCCTCCAGTCCAGCCTTT	24	2	FgHV1gp2	-
siRNA-t0208859	AAACGCGCTAAGTTACAGGCCTCA	24	2	FgHV1gp2	-
siRNA-t0314303	AAACGCTGCCGGGATGTAATCAA	23	2	FgHV1gp2	-
siRNA-t0357663	AAACGCTTTGGGCACATCGTAC	22	2	FgHV1gp1	-
siRNA-t0349967	AAACTGTCGCACTCTCTTCAT	21	2	FGSG_07593	K01195|1|0.0|1252|fgr:FG07593.1|beta-glucuronidase [EC:3.2.1.31]
siRNA-t0365385	AAAGACACAGGTATTGATGA	20	2	FGSG_02315	K01932|1|0.0|2455|afm:AFUA_1G10380|[EC:6.3.2.-]
siRNA-t0246165	AAAGACTTTCTACACGAAGA	20	2	FGSG_13631	K02320|1|2e-29|129|aor:AOR_1_946054|DNA polymerase alpha subunit A [EC:2.7.7.7]
siRNA-t0308403	AAAGGTACGTCTATATTGTCTCTCA	25	2	FgHV1gp2	-
siRNA-t0254523	AAATCAAGTTGGTATCCCTC	20	2	FgHV1gp2	-
siRNA-t0366879	AAATCATCGGTCTCGCCAAAAC	22	2	FGSG_00669	K05658|1|4e-39|163|yli:YALI0F20174g|ATP-binding cassette, subfamily B (MDR/TAP), member 1;K05663|5|5e-39|163|cpw:CPC735_047650|mitochondrial ABC transporter ATM
siRNA-t0336681	AAATCTCGGACCGCTTGAATA	21	2	FGSG_00836	-
siRNA-t0223456	AAATGAACTCAATCTTCTCA	20	2	FGSG_07575	-
siRNA-t0360182	AAATTTCAGAACAAACCATT	20	2	FGSG_05519	K04728|1|0.0|5526|fgr:FG05519.1|ataxia telangiectasia mutated family protein [EC:2.7.11.1]
siRNA-t0339932	AACAATGAAGACGCAAGCCCGCTTA	25	2	FgHV1gp2	-
siRNA-t0302341	AACACCACATCACGCTCGGGGTC	23	2	FgHV1gp2	-
siRNA-t0315189	AACAGACTTCTTCTCTGCTAT	21	2	FGSG_08421	K13126|1|0.0|636|fgr:FG08421.1|polyadenylate-binding protein
siRNA-t0232246	AACATCAGGCATGTATGGGTCG	22	2	FGSG_03750	K11244|1|7e-07|55.5|fgr:FG10787.1|cell wall integrity and stress response component
siRNA-t0230963	AACATGCAATTGAGTGCCATGACG	24	2	FgHV1gp1	-
siRNA-t0206247	AACATGCCGACAGAGAACAG	20	2	FGSG_10935	K08711|1|1e-79|298|kla:KLLA0D03476g|ATP-binding cassette, subfamily G (WHITE), member 2, PDR
siRNA-t0279637	AACCACAAGCCACTGCAGTTTGTAC	25	2	FgHV1gp2	-
siRNA-t0214372	AACCACACTCAGCAGGAACT	20	2	FGSG_09541	K08744|1|8e-35|147|spo:SPAC22A12.08c|cardiolipin synthase [EC:2.7.8.-]
siRNA-t0335930	AAGGATTAATCAATGTACCGTTC	23	2	FgHV1gp2	-
siRNA-t0289397	AAGGCGATCGCTGGAAGAAT	20	2	FGSG_07214	K13100|1|0.0|1057|fgr:FG07214.1|pre-mRNA-splicing factor CWC22
siRNA-t0254769	AAGGTACGTCTATATTGTCTCT	22	2	FgHV1gp2	-
siRNA-t0251825	AAGGTGACAACAGGCCAGTC	20	2	FGSG_04012	K00540|1|4e-117|420|mgr:MGG_03823|[EC:1.-.-.-];K09580|5|2e-21|102|fgr:FG01246.1|protein disulfide-isomerase A1 [EC:5.3.4.1]
siRNA-t0236271	AAGGTGCTGGGTTCTGGTAG	20	2	FGSG_01321	K05760|1|1e-21|104|mgl:MGL_4132|paxillin
siRNA-t0292380	AAGTACCGTCCAGGGGCGTTT	21	2	FGSG_06653	-
siRNA-t0244689	AAGTCCACGATTGTAACGAA	20	2	FGSG_09968	-
siRNA-t0221565	AAGTCTCCGAACGTGATCTCA	21	2	FgHV1gp2	-
siRNA-t0220496	AAGTCTCTTGTTCTGGAACG	20	2	FGSG_02381	-
siRNA-t0341117	AAGTCTGGAATTCCTTGGGAGG	22	2	FgHV1gp2	-
siRNA-t0292872	AAGTCTTCGTCCAGTAGCCAA	21	2	FgHV1gp2	-
siRNA-t0219061	AAGTCTTCGTCCAGTAGCCAAT	22	2	FgHV1gp2	-
siRNA-t0298389	AAGTGACAAAAGCATGGCGATGCAC	25	2	FgHV1gp2	-
siRNA-t0299546	AAGTTATGAGCTCTGTGACTCCA	23	2	FgHV1gp2	-
siRNA-t0239802	AAGTTCTCGCCAGCAATGCCCCT	23	2	FgHV1gp1	-
siRNA-t0291373	AAGTTGATCTTCCCTATGGCCTCGC	25	2	FgHV1gp2	-
siRNA-t0299033	AAGTTTAGCAGGTTCAAACTCAA	23	2	FgHV1gp2	-
siRNA-t0215012	AAGTTTGCATCCTCGACCCACT	22	2	FgHV1gp2	-
siRNA-t0206282	AAGTTTTGTGGTACAATGGTATTG	24	2	FGSG_13758	K06867|1|3e-09|62.0|ago:AGOS_ADL244W|
siRNA-t0252642	AATACCATTCACGAACGCTA	20	2	FGSG_01910	K06636|1|0.0|2339|fgr:FG01910.1|structural maintenance of chromosome 1
siRNA-t0253677	AATATAGACGTACCTTTCGGT	21	2	FGSG_06016	K05727|1|1e-46|188|mpr:MPER_07660|dedicator of cytokinesis 3/4/5
siRNA-t0240096	AATCATCATCGTAGTCATCGTC	22	2	FGSG_13700	-
siRNA-t0225829	AATCCTGCTCGCACCTTTCT	20	2	FGSG_08737	K03263|1|4e-08|58.9|afv:AFLA_028800|translation initiation factor 5A
siRNA-t0269461	AATCGTCATACATTGCACGAG	21	2	FgHV1gp2	-
siRNA-t0294357	AATCGTGGACTTCCTTTAGGAAA	23	2	FgHV1gp2	-
siRNA-t0315260	AATCTTCAGGCCCTGGGATTGT	22	2	FgHV1gp2	-
siRNA-t0253732	AATGAAGACGCAAGCCCGCTT	21	2	FgHV1gp2	-
siRNA-t0272646	AATGATGGCACGTCGACAGGCG	22	2	FgHV1gp2	-
siRNA-t0330080	AATGCAACAGACTTCTTCTCT	21	2	FgHV1gp2	-
siRNA-t0264937	AATGGAACAACGTGAGCCAG	20	2	FGSG_05974	-
siRNA-t0257671	AATGGAGGACGATCTCATTCGT	22	2	FgHV1gp2	-
siRNA-t0286402	AATGGTCACCGTCCGGAATG	20	2	FGSG_10638	-
siRNA-t0215645	AATGGTCTGCCTAATCTTTCCTC	23	2	FgHV1gp2	-
siRNA-t0221244	AATGTAAGTGACGAGGCCTCT	21	2	FgHV1gp2	-
siRNA-t0290322	AATGTCAGCAGTGTTCATGTG	21	2	FGSG_07118	K11231|1|5e-39|162|afv:AFLA_039130|osomolarity two-component system, sensor histidine kinase SLN1 [EC:2.7.13.3];K02486|5|3e-33|143|spo:SPAC27E2.09|two-component system, unclassified family, sensor kinase [EC:2.7.13.3]

<table>
<tr><td>siRNA-t0325160</td><td>AATTCCGGGATCAGGTTCACA</td><td>21</td><td>2</td><td>FGSG_08396</td><td>K01443|1|0.0|921|fgr:FG08396.1|N-acetylglucosamine-6-phosphate deacetylase [EC:3.5.1.25]</td></tr>
<tr><td>siRNA-t0228699</td><td>AATTCTGCCATAACCTCGTT</td><td>20</td><td>2</td><td>FGSG_08139</td><td>-</td></tr>
<tr><td>siRNA-t0250617</td><td>AATTGCACATGGGACCATCAT</td><td>21</td><td>2</td><td>FgHV1gp2</td><td>-</td></tr>
<tr><td>siRNA-t0210928</td><td>ACAAAACTTCTGTCCAGTTGAGA</td><td>23</td><td>2</td><td>FGSG_12624</td><td>K00777|1|3e-30|133|afm:AFUA_7G07100|[EC:2.4.2.-];K14855|2|7e-15|82.4|lbc:LACBIDRAFT_296068|ribosome assembly protein 4;K06666|3|6e-10|66.2|cal:CaO19.13528|glucose repression regulatory protein TUP1</td></tr>
<tr><td>siRNA-t0333073</td><td>ACAAATCTGGGTGGCGATGGATT</td><td>23</td><td>2</td><td>FgHV1gp2</td><td>-</td></tr>
<tr><td>siRNA-t0280886</td><td>ACAACAGGAATGACGTTTGTAA</td><td>22</td><td>2</td><td>FgHV1gp2</td><td>-</td></tr>
<tr><td>siRNA-t0362779</td><td>ACAACAGGTTGGAATCTGCGTTT</td><td>23</td><td>2</td><td>FgHV1gp2</td><td>-</td></tr>
<tr><td>siRNA-t0364622</td><td>ACAAGCCGTCAAACATAGACA</td><td>21</td><td>2</td><td>FgHV1gp2</td><td>-</td></tr>
<tr><td>siRNA-t0202070</td><td>ACAATCGTGGACTTCCTTTAG</td><td>21</td><td>2</td><td>FgHV1gp2</td><td>-</td></tr>
<tr><td>siRNA-t0218788</td><td>ACAATGAGTCCCAAATCGGCA</td><td>21</td><td>2</td><td>FGSG_02139</td><td>K05658|1|9e-34|145|smp:SMAC_06012|ATP-binding cassette, subfamily B (MDR/TAP), member 1</td></tr>
<tr><td>siRNA-t0331559</td><td>ACAATGGCGCATACACGGAAC</td><td>21</td><td>2</td><td>FgHV1gp2</td><td>-</td></tr>
<tr><td>siRNA-t0326641</td><td>ACACCACATCACGCTCGGGGTC</td><td>22</td><td>2</td><td>FgHV1gp2</td><td>-</td></tr>
<tr><td>siRNA-t0286677</td><td>ACACCGTCAAGTTGTGACTG</td><td>20</td><td>2</td><td>FGSG_11704</td><td>-</td></tr>
<tr><td>siRNA-t0337833</td><td>ACACGACGTACTTAGGGTTGTTG</td><td>23</td><td>2</td><td>FgHV1gp2</td><td>-</td></tr>
<tr><td>siRNA-t0364103</td><td>ACACGCCTTCATTGTTCCCT</td><td>20</td><td>2</td><td>FGSG_01046</td><td>K03349|1|0.0|1692|fgr:FG01046.1|anaphase-promoting complex subunit 2</td></tr>
<tr><td>siRNA-t0307758</td><td>ACACGCTCTGCTTACTTGGC</td><td>20</td><td>2</td><td>FGSG_10381</td><td>K00924|1|0.0|1042|afm:AFUA_4G06750|[EC:2.7.1.-];K06683|4|1e-58|228|cal:CaO19.11029|cell division control protein CDC15 [EC:2.7.11.1]</td></tr>
<tr><td>siRNA-t0333414</td><td>ACACGCTCTGCTTACTTGGCA</td><td>21</td><td>2</td><td>FGSG_05329</td><td>K03686|1|0.0|978|ani:AN6194.2|molecular chaperone DnaJ;K12200|2|2e-39|164|cpw:CPC735_002480|programmed cell death 6-interacting protein</td></tr>
<tr><td>siRNA-t0355653</td><td>ACAGGCCTCATTGATTTTGTGTC</td><td>23</td><td>2</td><td>FgHV1gp2</td><td>-</td></tr>
<tr><td>siRNA-t0277135</td><td>ACATATTCGGACTCATCCCACA</td><td>22</td><td>2</td><td>FgHV1gp2</td><td>-</td></tr>
<tr><td>siRNA-t0219197</td><td>ACATATTCGGACTCATCCCACAT</td><td>23</td><td>2</td><td>FgHV1gp2</td><td>-</td></tr>
<tr><td>siRNA-t0286631</td><td>ACATCCAGATCTTATAGACAGGC</td><td>23</td><td>2</td><td>FgHV1gp2</td><td>-</td></tr>
<tr><td>siRNA-t0251607</td><td>ACATCGTACCCGTCGAAGAC</td><td>20</td><td>2</td><td>FGSG_07797</td><td>-</td></tr>
<tr><td>siRNA-t0275040</td><td>ACATCGTACCCGTCGAAGACCA</td><td>22</td><td>2</td><td>FgHV1gp1</td><td>-</td></tr>
<tr><td>siRNA-t0240342</td><td>ACATGCCTGATGTTCTTGTG</td><td>20</td><td>2</td><td>FgHV1gp2</td><td>-</td></tr>
<tr><td>siRNA-t0353764</td><td>ACATGCCTGATGTTCTTGTGCC</td><td>22</td><td>2</td><td>FgHV1gp2</td><td>-</td></tr>
<tr><td>siRNA-t0316230</td><td>ACATGCCTGATGTTCTTGTGCCTC</td><td>24</td><td>2</td><td>FgHV1gp2</td><td>-</td></tr>
<tr><td>siRNA-t0244798</td><td>ACATGCCTGATGTTCTTGTGCCTCC</td><td>25</td><td>2</td><td>FgHV1gp2</td><td>-</td></tr>
<tr><td>siRNA-t0304798</td><td>ACATTGATCCAGAAGCGCAC</td><td>20</td><td>2</td><td>FGSG_06957</td><td>K08286|1|0.0|1283|fgr:FG06957.1|protein-serine/threonine kinase [EC:2.7.11.-]</td></tr>
<tr><td>siRNA-t0359043</td><td>ACATTGGATGATATTCGGTATT</td><td>22</td><td>2</td><td>FgHV1gp2</td><td>-</td></tr>
<tr><td>siRNA-t0234208</td><td>ACCACACTCAGCAGGAACTG</td><td>20</td><td>2</td><td>FGSG_09541</td><td>K08744|1|8e-35|147|spo:SPAC22A12.08c|cardiolipin synthase [EC:2.7.8.-]</td></tr>
<tr><td>siRNA-t0351175</td><td>ACCAGAAACCCATCCCACTCGTG</td><td>23</td><td>2</td><td>FgHV1gp2</td><td>-</td></tr>
<tr><td>siRNA-t0291467</td><td>ACCAGAACCCAGCACCTTTCCCTA</td><td>24</td><td>2</td><td>FgHV1gp2</td><td>-</td></tr>
<tr><td>siRNA-t0216061</td><td>ACCAGAACCCAGCACCTTTCCCTAT</td><td>25</td><td>2</td><td>FgHV1gp2</td><td>-</td></tr>
<tr><td>siRNA-t0348473</td><td>ACCAGATCATAACCCGCATAA</td><td>21</td><td>2</td><td>FgHV1gp2</td><td>-</td></tr>
<tr><td>siRNA-t0325288</td><td>ACCAGGATTGATAGGGTCAGG</td><td>21</td><td>2</td><td>FGSG_11489</td><td>-</td></tr>
<tr><td>siRNA-t0313329</td><td>ACCAGGATTTGCTTCTTGCACT</td><td>22</td><td>2</td><td>FgHV1gp2</td><td>-</td></tr>
<tr><td>siRNA-t0267802</td><td>ACCATTCACGAACGCTAAGAGG</td><td>22</td><td>2</td><td>FgHV1gp2</td><td>-</td></tr>
<tr><td>siRNA-t0310624</td><td>ACCCAACACATTTTTGAAGATCG</td><td>23</td><td>2</td><td>FgHV1gp2</td><td>-</td></tr>
<tr><td>siRNA-t0313871</td><td>ACCCATACATGCCTGATGTTCT</td><td>22</td><td>2</td><td>FgHV1gp2</td><td>-</td></tr>
<tr><td>siRNA-t0328260</td><td>ACCCATGCTTTCGACGTACT</td><td>20</td><td>2</td><td>FgHV1gp2</td><td>-</td></tr>
<tr><td>siRNA-t0262209</td><td>ACCCATTCGTGTTCCCGAAAACAAC</td><td>25</td><td>2</td><td>FgHV1gp2</td><td>-</td></tr>
<tr><td>siRNA-t0213697</td><td>ACCCGAGAAAAACGCCCCTGG</td><td>21</td><td>2</td><td>FgHV1gp2</td><td>-</td></tr>
<tr><td>siRNA-t0359091</td><td>ACCCGTCGAAGACCAAAAATTT</td><td>22</td><td>2</td><td>FgHV1gp1</td><td>-</td></tr>
<tr><td>siRNA-t0331645</td><td>ACCCTGACGATGACTACGAT</td><td>20</td><td>2</td><td>FGSG_08056</td><td>-</td></tr>
<tr><td>siRNA-t0219594</td><td>ACCGACATGTTTAGGATCAC</td><td>20</td><td>2</td><td>FGSG_03933</td><td>K12618|1|1e-55|215|ani:AN8185.2|5'-3' exoribonuclease 1 [EC:3.1.13.-]</td></tr>
<tr><td>siRNA-t0345502</td><td>ACCGACGGTACGTGGAAATCTTCA</td><td>24</td><td>2</td><td>FgHV1gp2</td><td>-</td></tr>
<tr><td>siRNA-t0248015</td><td>ACCGAGGCAGAGCATTCAGCAC</td><td>22</td><td>2</td><td>FGSG_04842</td><td>-</td></tr>
<tr><td>siRNA-t0251029</td><td>ACCGATATTGAACCCTCCTT</td><td>20</td><td>2</td><td>FGSG_01583</td><td>K00489|1|7e-11|67.8|bfu:BC1G_07665|cytochrome P450, family 7, subfamily A (cholesterol 7alpha-monooxygenase) [EC:1.14.13.17];K00517|3|2e-06|53.5|afm:AFUA_2G14430|[EC:1.14.-.-];K14338|4|8e-06|51.2|mgr:MGG_10879|cytochrome P450 / NADPH-cytochrome P450 reductase [EC:1.14.14.1 1.6.2.4]</td></tr>
<tr><td>siRNA-t0259534</td><td>ACCGATATTGAACCCTCCTTCGT</td><td>23</td><td>2</td><td>FgHV1gp2</td><td>-</td></tr>
<tr><td>siRNA-t0324445</td><td>ACGCATGGATGGTGATAGAC</td><td>20</td><td>2</td><td>FGSG_08904</td><td>-</td></tr>
<tr><td>siRNA-t0366710</td><td>ACGCCCCTGGACGGTACTTCT</td><td>21</td><td>2</td><td>FgHV1gp2</td><td>-</td></tr>
<tr><td>siRNA-t0258821</td><td>ACGCGACAAGCAACAATTTGCC</td><td>22</td><td>2</td><td>FGSG_00104</td><td>-</td></tr>
<tr><td>siRNA-t0224603</td><td>ACGCGCCTCCAGTCCAGCCT</td><td>20</td><td>2</td><td>FgHV1gp2</td><td>-</td></tr>
<tr><td>siRNA-t0230293</td><td>ACGCGCCTCCAGTCCAGCCTTT</td><td>22</td><td>2</td><td>FgHV1gp2</td><td>-</td></tr>
<tr><td>siRNA-t0241703</td><td>ACGCGCCTCCAGTCCAGCCTTTT</td><td>23</td><td>2</td><td>FgHV1gp2</td><td>-</td></tr>
<tr><td>siRNA-t0254428</td><td>ACGCGCTAAGTTACAGGCCTCA</td><td>22</td><td>2</td><td>FgHV1gp2</td><td>-</td></tr>
<tr><td>siRNA-t0253164</td><td>ACGCGCTAAGTTACAGGCCTCAT</td><td>23</td><td>2</td><td>FgHV1gp2</td><td>-</td></tr>
<tr><td>siRNA-t0359883</td><td>ACGGAACCCATTGATCATTCTCC</td><td>23</td><td>2</td><td>FgHV1gp2</td><td>-</td></tr>
<tr><td>siRNA-t0365034</td><td>ACGGATCCACATGAGCTTCAGC</td><td>22</td><td>2</td><td>FgHV1gp2</td><td>-</td></tr>
<tr><td>siRNA-t0362577</td><td>ACGGTGCTGATGATCATGACG</td><td>21</td><td>2</td><td>FGSG_00470</td><td>-</td></tr>
<tr><td>siRNA-t0215773</td><td>ACGTACTTAGGGTTGTTGAGAACG</td><td>24</td><td>2</td><td>FgHV1gp2</td><td>-</td></tr>
<tr><td>siRNA-t0345566</td><td>ACGTCATACCTCACCACATCA</td><td>21</td><td>2</td><td>FGSG_13878</td><td>K01932|1|0.0|1450|afm:AFUA_5G12730|[EC:6.3.2.-]</td></tr>
<tr><td>siRNA-t0221774</td><td>ACGTCGAAAGCATGGGTCGC</td><td>20</td><td>2</td><td>FGSG_10548</td><td>K15418|1|0.0|1191|pcs:Pc21g12440|zearalenone synthase, highly reducing iterative type I polyketide synthase;K15419|4|0.0|740|ani:AN2547.2|emericellamide biosynthesis, highly reducing iterative type I polyketide synthase</td></tr>
<tr><td>siRNA-t0227025</td><td>ACGTGGAAATCTTCAGGCCCTGG</td><td>23</td><td>2</td><td>FgHV1gp2</td><td>-</td></tr>
<tr><td>siRNA-t0322136</td><td>AGATCCCACTCTAGCAATGCATCA</td><td>24</td><td>2</td><td>FgHV1gp1</td><td>-</td></tr>
<tr><td>siRNA-t0301138</td><td>AGATCTCAGAATGGTCACCG</td><td>20</td><td>2</td><td>FGSG_05023</td><td>K00006|1|0.0|869|fgr:FG05023.1|glycerol-3-phosphate dehydrogenase (NAD+) [EC:1.1.1.8]</td></tr>
<tr><td>siRNA-t0251410</td><td>AGATGGTGATTGTGGTCATTT</td><td>21</td><td>2</td><td>FGSG_06743</td><td>K14802|1|0.0|2681|fgr:FG06743.1|phospholipid-transporting ATPase [EC:3.6.3.1]</td></tr>
<tr><td>siRNA-t0333381</td><td>AGATTTGCCTGTGCCGGTGT</td><td>20</td><td>2</td><td>FGSG_11372</td><td>K00777|1|5e-21|103|afm:AFUA_7G06290|[EC:2.4.2.-];K12823|4|1e-10|69.7|ure:UREG_02211|ATP-dependent RNA helicase DDX5/DBP2 [EC:3.6.4.13]</td></tr>
<tr><td>siRNA-t0290861</td><td>AGATTTGCCTGTGCCGGTGTCGG</td><td>23</td><td>2</td><td>FgHV1gp2</td><td>-</td></tr>
<tr><td>siRNA-t0316378</td><td>AGATTTGTCAGGGTTTTCACC</td><td>21</td><td>2</td><td>FGSG_13878</td><td>K01932|1|0.0|1450|afm:AFUA_5G12730|[EC:6.3.2.-]</td></tr>
<tr><td>siRNA-t0204575</td><td>AGCAACTAGAGCCAAGTCAT</td><td>20</td><td>2</td><td>FGSG_06101</td><td>K14780|1|0.0|2204|fgr:FG06101.1|ATP-dependent RNA helicase DHX37/DHR1 [EC:3.6.4.13]</td></tr>
<tr><td>siRNA-t0247672</td><td>AGCAAGTGAAACTGTTCGCCA</td><td>21</td><td>2</td><td>FgHV1gp2</td><td>-</td></tr>
<tr><td>siRNA-t0232283</td><td>AGCAATGCAACAGACTTCTT</td><td>20</td><td>2</td><td>FgHV1gp2</td><td>-</td></tr>
<tr><td>siRNA-t0228652</td><td>AGCACCATACAATGCCGCCAA</td><td>21</td><td>2</td><td>FgHV1gp2</td><td>-</td></tr>
<tr><td>siRNA-t0265937</td><td>AGCACCGTACGGATCCACAT</td><td>20</td><td>2</td><td>FgHV1gp2</td><td>-</td></tr>
<tr><td>siRNA-t0261064</td><td>AGCACCGTACGGATCCACATGAGC</td><td>24</td><td>2</td><td>FgHV1gp2</td><td>-</td></tr>
<tr><td>siRNA-t0270921</td><td>AGCAGAATTCTAGCCTGGTTGAGAT</td><td>25</td><td>2</td><td>FgHV1gp2</td><td>-</td></tr>
<tr><td>siRNA-t0314037</td><td>AGCATGGCCGGCAGTCCTAGC</td><td>21</td><td>2</td><td>FgHV1gp2</td><td>-</td></tr>
<tr><td>siRNA-t0210794</td><td>AGCATGGCCGGCAGTCCTAGCAA</td><td>23</td><td>2</td><td>FgHV1gp2</td><td>-</td></tr>
<tr><td>siRNA-t0327090</td><td>AGCATGTTCTTCGTGTAGAA</td><td>20</td><td>2</td><td>FGSG_01137</td><td>K08866|1|0.0|1514|fgr:FG01137.1|serine/threonine-protein kinase TTK/MPS1 [EC:2.7.12.1]</td></tr>
<tr><td>siRNA-t0354404</td><td>AGCCATTTCGAATCTGCCCG</td><td>20</td><td>2</td><td>FgHV1gp2</td><td>-</td></tr>
<tr><td>siRNA-t0229593</td><td>AGCCATTTCGAATCTGCCCGATCTA</td><td>25</td><td>2</td><td>FgHV1gp2</td><td>-</td></tr>
<tr><td>siRNA-t0347054</td><td>AGGACGTTCTGGTCTTCGATG</td><td>21</td><td>2</td><td>FgHV1gp2</td><td>-</td></tr>
<tr><td>siRNA-t0238015</td><td>AGGCCAAAGGTGTTCAGCTTC</td><td>21</td><td>2</td><td>FgHV1gp2</td><td>-</td></tr>
<tr><td>siRNA-t0228435</td><td>AGGCCTCTGACGTTGATCTCTT</td><td>22</td><td>2</td><td>FgHV1gp2</td><td>-</td></tr>
<tr><td>siRNA-t0246119</td><td>AGGCGTCGACACGATGATAC</td><td>20</td><td>2</td><td>FGSG_10919</td><td>K08192|1|7e-64|244|dha:DEHA2C12276g|MFS transporter, ACS family, allantoate permease</td></tr>
</table>

siRNA-t0327843	AGGCTAGGAACGATGCCTCGT	21	2	FGSG_02233	-
siRNA-t0227821	AGTATCGTCACTTCTTCGTA	20	2	FGSG_09917	K01115|1|0.0|3466|fgr:FG09917.1|phospholipase D [EC:3.1.4.4]
siRNA-t0210345	AGTATGAACCCTTCCACGAT	20	2	FGSG_12434	-
siRNA-t0344384	AGTCAGACGTTCATTTATCA	20	2	FGSG_12602	-
siRNA-t0297575	AGTCTGGAATTCCTTGGGAGGC	22	2	FgHV1gp2	-
siRNA-t0332252	AGTCTTCGTCCAGTAGCCAA	20	2	FGSG_07372	-
siRNA-t0288331	AGTCTTCGTCCAGTAGCCAATG	22	2	FgHV1gp2	-
siRNA-t0223042	AGTCTTGCGGTGTGGGTCTTCTG	23	2	FgHV1gp2	-
siRNA-t0229065	AGTCTTGCGGTGTGGGTCTTCTGAC	25	2	FgHV1gp2	-
siRNA-t0243479	AGTGACGAGGCCTCTGACGTTG	22	2	FgHV1gp2	-
siRNA-t0359632	AGTGACGAGGCCTCTGACGTTGAT	24	2	FgHV1gp2	-
siRNA-t0211425	AGTGACGAGGCCTCTGACGTTGATC	25	2	FgHV1gp2	-
siRNA-t0299057	AGTGATGGTACCATTGCTGCA	21	2	FGSG_05452	-
siRNA-t0312224	AGTGCCATGACGTACGACCCCGAA	24	2	FgHV1gp1	-
siRNA-t0233180	AGTGCCCGGCCGTTTGTGCTGC	22	2	FgHV1gp2	-
siRNA-t0325155	AGTGCGACAGTTTCTACACC	20	2	FGSG_06395	K01529|1|0.0|4061|fgr:FG06395.1|[EC:3.6.1.-]
siRNA-t0344698	AGTGGCATATTCAAGAATCAAT	22	2	FgHV1gp2	-
siRNA-t0206455	AGTGGCGCATTTCTTTCTTACCCTC	25	2	FgHV1gp2	-
siRNA-t0288997	AGTGGGAGCGAAAGCGATATC	21	2	FGSG_06193	K08773|1|2e-20|101|cci:CC1G_05669|RalA-binding protein 1
siRNA-t0205965	AGTGGGATCTTCGTCACGCTT	21	2	FGSG_05729	-
siRNA-t0209058	AGTGGGATGGGTTTCTGGTT	20	2	FGSG_05683	K00485|1|5e-159|560|mgr:MGG_03330|dimethylaniline monooxygenase (N-oxide forming) [EC:1.14.13.8]
siRNA-t0239897	AGTGTCGTAGGAAAGGAAAAG	21	2	FGSG_13645	-
siRNA-t0236228	AGTTCATGAATGCCGCCGTA	20	2	FGSG_12253	-
siRNA-t0219012	AGTTCCGCGTGTGATTGGTCT	21	2	FGSG_03919	-
siRNA-t0340752	AGTTCCTGCTGAGTGTGGTTT	21	2	FgHV1gp2	-
siRNA-t0363183	AGTTGGTGCCGACGTCTACGTAAGG	25	2	FgHV1gp2	-
siRNA-t0267914	ATAAATGAACGTCTGACTGTA	21	2	FgHV1gp2	-
siRNA-t0326120	ATAAATGAACGTCTGACTGTAGT	23	2	FgHV1gp2	-
siRNA-t0270477	ATAACCTCGTTGGGACCTTTCC	22	2	FgHV1gp2	-
siRNA-t0234532	ATAATCGGCATGAACTCGGTCAA	23	2	FgHV1gp2	-
siRNA-t0300905	ATAATCGTTGAAAATCAGAGAGC	23	2	FgHV1gp2	-
siRNA-t0282692	ATACTCTTCAGCGTAACGTGAC	22	2	FgHV1gp2	-
siRNA-t0236569	ATAGATCTTATTGCCAGGTAGA	22	2	FgHV1gp2	-
siRNA-t0248886	ATAGTGCCCGGCCGTTTGTGC	21	2	FgHV1gp2	-
siRNA-t0292494	ATGAGCGTTTGTGGAGCCATG	21	2	FGSG_09145	-
siRNA-t0257859	ATGATGAGTAAGCGGGCTTG	20	2	FGSG_03736	K14859|1|6e-27|122|scm:SCHCODRAFT_255991|ribosome biogenesis protein SSF1/2
siRNA-t0242206	ATGCAGCGGCAAATACAGGACCG	23	2	FgHV1gp2	-
siRNA-t0269165	ATGCCAAAAGATCCTTACAT	20	2	FGSG_07604	-
siRNA-t0235908	ATGCCTGATGTTCTTGTGCC	20	2	FgHV1gp2	-
siRNA-t0259769	ATGGATCTGATTATCTCCAT	20	2	FgHV1gp1	-
siRNA-t0286079	ATGGCGACACCGACGGTACG	20	2	FGSG_05709	K13830|1|2e-68|259|lbc:LACBIDRAFT_233717|pentafunctional AROM polypeptide [EC:4.2.3.4 4.2.1.10 1.1.1.25 2.7.1.71 2.5.1.19]
siRNA-t0204108	ATGGCTCCACAAACGCTCAT	20	2	FGSG_13896	-
siRNA-t0313451	ATGGTCTGCCTAATCTTTCC	20	2	FgHV1gp2	-
siRNA-t0303252	ATGTACTGTTCGGCAACAGAA	21	2	FgHV1gp2	-
siRNA-t0313306	ATTAATCAATGTACCGTTCGGC	22	2	FgHV1gp2	-
siRNA-t0218835	ATTACGGACAGTACATGCGT	20	2	FGSG_00916	K03006|1|0.0|3167|fgr:FG00916.1|DNA-directed RNA polymerase II subunit RPB1 [EC:2.7.7.6]
siRNA-t0294378	ATTCACGAACGCTAAGAGGAA	21	2	FgHV1gp2	-
siRNA-t0314871	ATTCCAACTGCGACCCATGCTTTCG	25	2	FgHV1gp2	-
siRNA-t0269795	ATTCTTCCAGCGATCGCCTTT	21	2	FGSG_06429	K01183|1|2e-54|213|afm:AFUA_5G03960|chitinase [EC:3.2.1.14]
siRNA-t0206806	ATTGCACGAGGGACGAGCTTGGCC	24	2	FgHV1gp2	-
siRNA-t0338645	ATTGTTGCTTGTCGCGTCCC	20	2	FGSG_03032	K05663|1|3e-96|352|lbc:LACBIDRAFT_185384|mitochondrial ABC transporter ATM
siRNA-t0226991	ATTTGCGACGTGCTTGAAGGCGAAG	25	2	FgHV1gp2	-
siRNA-t0310185	ATTTGGCCCCAGCAGACTTGGA	22	2	FgHV1gp2	-
siRNA-t0218147	ATTTGGGTGGCTTTGAAGTTGA	22	2	FgHV1gp2	-
siRNA-t0266485	ATTTTCGCGCGCTGTACTGA	20	2	FgHV1gp2	-
siRNA-t0248531	ATTTTCGGTAGTCATCGGGAATCAT	25	2	FgHV1gp2	-
siRNA-t0236927	CAAAAGACCAATCCCTGGATTA	22	2	FgHV1gp2	-
siRNA-t0231133	CAAATCAAGTTGGTATCCCTCCGT	24	2	FgHV1gp2	-
siRNA-t0259842	CAAATTTGCCGACGCTCCACT	21	2	FGSG_09965	K14300|1|0.0|2662|fgr:FG09965.1|nuclear pore complex protein Nup133
siRNA-t0275538	CAACAATGAAGACGCAAGCCCGCTT	25	2	FgHV1gp2	-
siRNA-t0355889	CAACAGTGAGCAAGTTCGTT	20	2	FGSG_05818	-
siRNA-t0208124	CAACGCAATCGGGTTCACAA	20	2	FGSG_06582	K10661|1|0.0|2855|fgr:FG06582.1|E3 ubiquitin-protein ligase MARCH6 [EC:6.3.2.19]
siRNA-t0276458	CAACTTGATTTGACGTTCCA	20	2	FGSG_11425	K00777|1|3e-69|263|afm:AFUA_7G06290|[EC:2.4.2.-];K15110|4|1e-19|98.6|ani:AN1299.2|solute carrier family 25 (mitochondrial 2-oxodicarboxylate transporter), member 21
siRNA-t0273124	CAACTTTCCAAGAACCGTTG	20	2	FGSG_06395	K01529|1|0.0|4061|fgr:FG06395.1|[EC:3.6.1.-]
siRNA-t0282952	CAAGAAGTTTGCATCCTCGACCCAC	25	2	FgHV1gp2	-
siRNA-t0364306	CAAGAGCTCTGGATTACCAGA	21	2	FGSG_06982	K11996|1|2e-44|180|uma:UM02725.1|adenylyltransferase and sulfurtransferase
siRNA-t0236407	CAAGCGTGACGAAGATCCCACTC	23	2	FgHV1gp1	-
siRNA-t0238501	CAAGTACTCCACCTTTGTGA	20	2	FgHV1gp2	-
siRNA-t0311692	CAAGTCTGCTGGGGCCAAAT	20	2	FGSG_11513	-
siRNA-t0260809	CAAGTCTGCTGGGGCCAAATT	21	2	FGSG_11513	-
siRNA-t0313395	CAAGTTGGTATCCCTCCGTGGG	22	2	FgHV1gp2	-
siRNA-t0248891	CAATGAAGACGCAAGCCCGCTT	22	2	FgHV1gp2	-
siRNA-t0297432	CAATGATGGCACGTCGACAGGCGT	24	2	FgHV1gp2	-
siRNA-t0357577	CAATGCATCACCAAGCCAAGCG	22	2	FgHV1gp1	-
siRNA-t0303567	CAATTTGATTGATTCTAGCTTGTG	24	2	FgHV1gp2	-
siRNA-t0326989	CACAAGAACATCAGGCATGT	20	2	FGSG_04287	K13830|1|0.0|3204|fgr:FG04287.1|pentafunctional AROM polypeptide [EC:4.2.3.4 4.2.1.10 1.1.1.25 2.7.1.71 2.5.1.19]
siRNA-t0244659	CACACGACCACGCAAGGATG	20	2	FGSG_00433	K02831|1|6e-85|315|tml:GSTUM_00011396001|ser/thr/tyr protein kinase RAD53 [EC:2.7.11.-]
siRNA-t0352608	CACAGTCGAATGAAATGGAA	20	2	FgHV1gp2	-
siRNA-t0301085	CACATCACGCTCGGGGTCAATTTG	24	2	FgHV1gp2	-
siRNA-t0273346	CACATCCAGATCTTATAGACA	21	2	FGSG_13783	K01932|1|0.0|1526|afm:AFUA_5G12730|[EC:6.3.2.-]
siRNA-t0210205	CACATCCAGATCTTATAGACAG	22	2	FgHV1gp2	-
siRNA-t0331622	CACATCGTACCCGTCGAAGACCA	23	2	FgHV1gp1	-
siRNA-t0205210	CACCGTACGGATCCACATGAG	21	2	FgHV1gp2	-
siRNA-t0232984	CACCGTCAAGTTGTGACTGCAGGT	24	2	FgHV1gp2	-
siRNA-t0346000	CACCTTTCCCTATTTTCTTCGGT	23	2	FgHV1gp2	-
siRNA-t0302269	CACCTTTCCCTATTTTCTTCGGTG	24	2	FgHV1gp2	-
siRNA-t0253006	CACCTTTCCCTATTTTCTTCGGTGA	25	2	FgHV1gp2	-
siRNA-t0220997	CACGACGTACTTAGGGTTGTTG	22	2	FgHV1gp2	-
siRNA-t0253509	CACGACGTACTTAGGGTTGTTGA	23	2	FgHV1gp2	-
siRNA-t0218473	CACGACTTTGAGTGCTGAAT	20	2	FGSG_10088	K12840|1|0.0|674|fgr:FG10088.1|splicing factor 45
siRNA-t0247636	CACGAGGGACGAGCTTGGCCCCTT	24	2	FgHV1gp2	-
siRNA-t0250491	CACGTCATGATCATCAGCAC	20	2	FGSG_11578	K01183|1|2e-16|87.8|afm:AFUA_6G13720|chitinase [EC:3.2.1.14];K01238|3|1e-11|72.4|afm:AFUA_7G08350|[EC:3.2.1.-]
siRNA-t0327925	CAGATGCGAAGCGAATATCGGAG	23	2	FGSG_03912	K09246|1|3e-07|55.8|1th:KLTH0E16500g|arginine metabolism regulation protein II

<table>
<tr><td>siRNA-t0289071</td><td>CAGCAATGGTACCATCACTC</td><td>20</td><td>2</td><td>FGSG_10995</td><td>K05658|1|4e-24|113|yli:YALI0F20174g|ATP-binding cassette, subfamily B (MDR/TAP), member 1;K05663|4|2e-23|111|bfu:BC1G_07740|mitochondrial ABC transporter ATM</td></tr>
<tr><td>siRNA-t0361190</td><td>CAGCACCGTACGGATCCACATGAG</td><td>24</td><td>2</td><td>FgHV1gp2</td><td>-</td></tr>
<tr><td>siRNA-t0287277</td><td>CAGCAGACACTTGTTTCTCG</td><td>20</td><td>2</td><td>FGSG_11703</td><td>-</td></tr>
<tr><td>siRNA-t0326011</td><td>CAGCATCCATCGAAGACGTC</td><td>20</td><td>2</td><td>FGSG_01977</td><td>-</td></tr>
<tr><td>siRNA-t0271171</td><td>CAGCATGGAAGGCGCGAGCTC</td><td>21</td><td>2</td><td>FGSG_01429</td><td>K14309|1|0.0|2016|fgr:FG01429.1|nuclear pore complex protein Nup93</td></tr>
<tr><td>siRNA-t0302663</td><td>CAGCATGGCCGGCAGTCCTAGCAA</td><td>24</td><td>2</td><td>FgHV1gp2</td><td>-</td></tr>
<tr><td>siRNA-t0297014</td><td>CAGCGAATTGCAACGAGGGCC</td><td>21</td><td>2</td><td>FGSG_02659</td><td>K03010|1|0.0|2320|nhe:NECHADRAFT_70495|DNA-directed RNA polymerase II subunit RPB2 [EC:2.7.7.6]</td></tr>
<tr><td>siRNA-t0343219</td><td>CAGCGTGAACCAGATCATAACCCGC</td><td>25</td><td>2</td><td>FgHV1gp2</td><td>-</td></tr>
<tr><td>siRNA-t0260013</td><td>CAGCTCCAATAATCGTTGAAAAT</td><td>23</td><td>2</td><td>FgHV1gp2</td><td>-</td></tr>
<tr><td>siRNA-t0278101</td><td>CAGCTCCAATAATCGTTGAAAATCA</td><td>25</td><td>2</td><td>FgHV1gp2</td><td>-</td></tr>
<tr><td>siRNA-t0298129</td><td>CAGCTGCAGCAACTGTCGCCTGGAC</td><td>25</td><td>2</td><td>FgHV1gp1</td><td>-</td></tr>
<tr><td>siRNA-t0364312</td><td>CAGGCAAAAAGCATTCCTCA</td><td>20</td><td>2</td><td>FGSG_13783</td><td>K01932|1|0.0|1526|afm:AFUA_5G12730|[EC:6.3.2.-]</td></tr>
<tr><td>siRNA-t0340348</td><td>CAGGCCTCATTGATTTTGTG</td><td>20</td><td>2</td><td>FgHV1gp2</td><td>-</td></tr>
<tr><td>siRNA-t0231730</td><td>CAGGCTAGGAACGATGCCTCGT</td><td>22</td><td>2</td><td>FGSG_02233</td><td>-</td></tr>
<tr><td>siRNA-t0326904</td><td>CAGGGTGCTCATGAGGTTGGCA</td><td>22</td><td>2</td><td>FgHV1gp2</td><td>-</td></tr>
<tr><td>siRNA-t0257115</td><td>CAGTTGGGGTCTTGACTTGG</td><td>20</td><td>2</td><td>FGSG_01977</td><td>-</td></tr>
<tr><td>siRNA-t0232636</td><td>CATAAGCCATCGCATGAAGCAA</td><td>22</td><td>2</td><td>FgHV1gp2</td><td>-</td></tr>
<tr><td>siRNA-t0234601</td><td>CATACAACTCGAACCAGTTC</td><td>20</td><td>2</td><td>FGSG_08728</td><td>K02179|1|6e-11|69.7|cgr:CAGL0F06391g|cell cycle arrest protein BUB2</td></tr>
<tr><td>siRNA-t0249565</td><td>CATACAATGCCGCCAACCAC</td><td>20</td><td>2</td><td>FgHV1gp2</td><td>-</td></tr>
<tr><td>siRNA-t0241194</td><td>CATCACGCTCGGGGTCAATTT</td><td>21</td><td>2</td><td>FgHV1gp2</td><td>-</td></tr>
<tr><td>siRNA-t0253184</td><td>CATCAGGACCCGAGAAAAACGC</td><td>22</td><td>2</td><td>FgHV1gp2</td><td>-</td></tr>
<tr><td>siRNA-t0291807</td><td>CATTCGTTACAATCGTGGACTTC</td><td>23</td><td>2</td><td>FgHV1gp2</td><td>-</td></tr>
<tr><td>siRNA-t0333932</td><td>CATTCTCTTCCAGTGTCGAACCA</td><td>23</td><td>2</td><td>FgHV1gp2</td><td>-</td></tr>
<tr><td>siRNA-t0270421</td><td>CCAACACATTTTTGAAGATCG</td><td>21</td><td>2</td><td>FgHV1gp2</td><td>-</td></tr>
<tr><td>siRNA-t0316383</td><td>CCAACTGCGACCCATGCTTTC</td><td>21</td><td>2</td><td>FGSG_01439</td><td>-</td></tr>
<tr><td>siRNA-t0226292</td><td>CCAACTTTCCAAGAACCGTT</td><td>20</td><td>2</td><td>FGSG_08732</td><td>-</td></tr>
<tr><td>siRNA-t0203894</td><td>CCACAAGCCACTGCAGTTTGT</td><td>21</td><td>2</td><td>FGSG_13855</td><td>K01183|1|3e-09|61.6|afm:AFUA_5G01400|chitinase [EC:3.2.1.14]</td></tr>
<tr><td>siRNA-t0299296</td><td>CCACACTCAGCAGGAACTGG</td><td>20</td><td>2</td><td>FGSG_05573</td><td>-</td></tr>
<tr><td>siRNA-t0247333</td><td>CCACAGTCGAATGAAATGGAACAA</td><td>24</td><td>2</td><td>FgHV1gp2</td><td>-</td></tr>
<tr><td>siRNA-t0340850</td><td>CCACTCGTGAACCTGTGGTGTCAA</td><td>24</td><td>2</td><td>FgHV1gp2</td><td>-</td></tr>
<tr><td>siRNA-t0208359</td><td>CCACTCTAGCAATGCATCACC</td><td>21</td><td>2</td><td>FgHV1gp1</td><td>-</td></tr>
<tr><td>siRNA-t0213122</td><td>CCAGACATTTTCGCGCGCTGT</td><td>21</td><td>2</td><td>FgHV1gp2</td><td>-</td></tr>
<tr><td>siRNA-t0239961</td><td>CCAGACATTTTCGCGCGCTGTACTG</td><td>25</td><td>2</td><td>FgHV1gp2</td><td>-</td></tr>
<tr><td>siRNA-t0306170</td><td>CCAGCATGGCCGGCAGTCCTAGCA</td><td>24</td><td>2</td><td>FgHV1gp2</td><td>-</td></tr>
<tr><td>siRNA-t0268834</td><td>CCAGCGCCTCCATGCATGTAGATCT</td><td>25</td><td>2</td><td>FgHV1gp2</td><td>-</td></tr>
<tr><td>siRNA-t0202056</td><td>CCAGTAGCATGTTCTTCGTGT</td><td>21</td><td>2</td><td>FGSG_12034</td><td>K06877|1|0.0|2299|fgr:FG01945.1|DEAD/DEAH box helicase domain-containing protein</td></tr>
<tr><td>siRNA-t0299547</td><td>CCAGTGTCGAACCATACTGG</td><td>20</td><td>2</td><td>FgHV1gp2</td><td>-</td></tr>
<tr><td>siRNA-t0290805</td><td>CCAGTTCCTGCTGAGTGTGGTT</td><td>22</td><td>2</td><td>FgHV1gp2</td><td>-</td></tr>
<tr><td>siRNA-t0334517</td><td>CCATACACGCAAGCACGCAT</td><td>20</td><td>2</td><td>FGSG_07382</td><td>K11838|1|0.0|2335|fgr:FG07382.1|ubiquitin carboxyl-terminal hydrolase 7 [EC:3.1.2.15]</td></tr>
<tr><td>siRNA-t0209816</td><td>CCATACTTTCCGAAGGAACTCCA</td><td>23</td><td>2</td><td>FgHV1gp2</td><td>-</td></tr>
<tr><td>siRNA-t0252639</td><td>CCATCAACGGTTCTTGGAAAG</td><td>21</td><td>2</td><td>FgHV1gp2</td><td>-</td></tr>
<tr><td>siRNA-t0311144</td><td>CCATCAAGGTCAGAGAGAACT</td><td>21</td><td>2</td><td>FGSG_13883</td><td>K12373|1|0.0|810|nfi:NFIA_032880|hexosaminidase [EC:3.2.1.52]</td></tr>
<tr><td>siRNA-t0264397</td><td>CCATCAGGTATTACCAGGCCAA</td><td>22</td><td>2</td><td>FGSG_09582</td><td>-</td></tr>
<tr><td>siRNA-t0307121</td><td>CCATCCCACTCGTGAACCTG</td><td>20</td><td>2</td><td>FGSG_10526</td><td>K13103|1|0.0|1310|fgr:FG10526.1|tuftelin-interacting protein 11</td></tr>
<tr><td>siRNA-t0306667</td><td>CCATCGCCACCCAGATTTGT</td><td>20</td><td>2</td><td>FGSG_01569</td><td>-</td></tr>
<tr><td>siRNA-t0310027</td><td>CCATCTTGCAGCACAAACGGCCGGG</td><td>25</td><td>2</td><td>FGSG_05511</td><td>K12041|1|0.0|1103|fgr:FG05511.1|solute carrier family 9 (sodium/hydrogen exchanger), member 6/7</td></tr>
<tr><td>siRNA-t0348911</td><td>CCATTCACGAACGCTAAGAGG</td><td>21</td><td>2</td><td>FgHV1gp2</td><td>-</td></tr>
<tr><td>siRNA-t0254354</td><td>CCATTCGTTACAATCGTGGACTTCC</td><td>25</td><td>2</td><td>FgHV1gp2</td><td>-</td></tr>
<tr><td>siRNA-t0362847</td><td>CCCTCGCATTTGCCGCACAGCTCC</td><td>24</td><td>2</td><td>FgHV1gp2</td><td>-</td></tr>
<tr><td>siRNA-t0221119</td><td>CCCTTCGTGAACTGCTGCGC</td><td>20</td><td>2</td><td>FGSG_07169</td><td>-</td></tr>
<tr><td>siRNA-t0346271</td><td>CCCTTCGTGAACTGCTGCGCTATG</td><td>24</td><td>2</td><td>FgHV1gp2</td><td>-</td></tr>
<tr><td>siRNA-t0221846</td><td>CCGAACAGTACATGCAGCAAT</td><td>21</td><td>2</td><td>FGSG_06900</td><td>K03639|1|0.0|1291|fgr:FG06900.1|molybdenum cofactor biosynthesis protein</td></tr>
<tr><td>siRNA-t0206427</td><td>CCGAAGAAAGACCACATCCA</td><td>20</td><td>2</td><td>FgHV1gp2</td><td>-</td></tr>
<tr><td>siRNA-t0343882</td><td>CCGAAGGAACTCCACATAGCG</td><td>21</td><td>2</td><td>FGSG_06097</td><td>-</td></tr>
<tr><td>siRNA-t0354131</td><td>CCGAATACCATACTTTCCGAA</td><td>21</td><td>2</td><td>FgHV1gp2</td><td>-</td></tr>
<tr><td>siRNA-t0251999</td><td>CCGAATGAAAGCCCAGTGGCGCA</td><td>23</td><td>2</td><td>FgHV1gp2</td><td>-</td></tr>
<tr><td>siRNA-t0204517</td><td>CCGACGGTACGTGGAAATCTTCAG</td><td>24</td><td>2</td><td>FgHV1gp2</td><td>-</td></tr>
<tr><td>siRNA-t0205889</td><td>CCGGCAGTCCTAGCAAGTGA</td><td>20</td><td>2</td><td>FgHV1gp2</td><td>-</td></tr>
<tr><td>siRNA-t0212298</td><td>CCGGGATCAGGTTCACAATT</td><td>20</td><td>2</td><td>FGSG_08396</td><td>K01443|1|0.0|921|fgr:FG08396.1|N-acetylglucosamine-6-phosphate deacetylase [EC:3.5.1.25]</td></tr>
<tr><td>siRNA-t0318273</td><td>CCGTACGGATCCACATGAGCTT</td><td>22</td><td>2</td><td>FgHV1gp2</td><td>-</td></tr>
<tr><td>siRNA-t0337677</td><td>CCTACAAGTGAAACAGCGGGAC</td><td>22</td><td>2</td><td>FGSG_06881</td><td>K05658|1|0.0|899|cim:CIMG_00780|ATP-binding cassette, subfamily B (MDR/TAP), member 1</td></tr>
<tr><td>siRNA-t0255817</td><td>CCTACCCTGAGGTTTTCCATATCAT</td><td>25</td><td>2</td><td>FgHV1gp2</td><td>-</td></tr>
<tr><td>siRNA-t0220894</td><td>CCTAGCAAGTGAAACTGTTCGCC</td><td>23</td><td>2</td><td>FgHV1gp2</td><td>-</td></tr>
<tr><td>siRNA-t0365885</td><td>CCTCAAATCCAAAAGACGCGTG</td><td>22</td><td>2</td><td>FgHV1gp2</td><td>-</td></tr>
<tr><td>siRNA-t0318143</td><td>CCTCAAATCCAAAAGACGCGTGTCG</td><td>25</td><td>2</td><td>FgHV1gp2</td><td>-</td></tr>
<tr><td>siRNA-t0240654</td><td>CCTCAAATGTCTCCTTCGGTT</td><td>21</td><td>2</td><td>FgHV1gp2</td><td>-</td></tr>
<tr><td>siRNA-t0223250</td><td>CCTCAGAGTCTTGCGGTGTG</td><td>20</td><td>2</td><td>FGSG_10283</td><td>-</td></tr>
<tr><td>siRNA-t0276239</td><td>CCTCCAGTCCAGCCTTTTGCCT</td><td>22</td><td>2</td><td>FgHV1gp2</td><td>-</td></tr>
<tr><td>siRNA-t0236335</td><td>CCTCGATTTCAGCGTTCTCA</td><td>20</td><td>2</td><td>FGSG_06089</td><td>K08874|1|0.0|7852|fgr:FG06089.1|transformation/transcription domain-associated protein</td></tr>
<tr><td>siRNA-t0352612</td><td>CCTCGTATTTTGAACTCACGA</td><td>21</td><td>2</td><td>FGSG_08144</td><td>K00777|1|1e-98|360|afm:AFUA_7G07100|[EC:2.4.2.-]</td></tr>
<tr><td>siRNA-t0357849</td><td>CCTCTCCCTCATACTTCGCC</td><td>20</td><td>2</td><td>FGSG_01251</td><td>K01175|1|3e-63|243|afm:AFUA_7G01550|[EC:3.1.-.-]</td></tr>
<tr><td>siRNA-t0364672</td><td>CCTCTCCCTTCGTGAACTGCTG</td><td>22</td><td>2</td><td>FgHV1gp2</td><td>-</td></tr>
<tr><td>siRNA-t0244607</td><td>CCTCTGATATATTCCAACTG</td><td>20</td><td>2</td><td>FgHV1gp2</td><td>-</td></tr>
<tr><td>siRNA-t0279105</td><td>CCTCTGATATATTCCAACTGC</td><td>21</td><td>2</td><td>FgHV1gp2</td><td>-</td></tr>
<tr><td>siRNA-t0329812</td><td>CGAACGCTAAGAGGAAAGGA</td><td>20</td><td>2</td><td>FGSG_11028</td><td>K05658|1|2e-34|147|yli:YALI0A11473g|ATP-binding cassette, subfamily B (MDR/TAP), member 1</td></tr>
<tr><td>siRNA-t0328292</td><td>CGAACGCTAAGAGGAAAGGAA</td><td>21</td><td>2</td><td>FgHV1gp2</td><td>-</td></tr>
<tr><td>siRNA-t0224596</td><td>CGAACGGGACCGACGTTTTG</td><td>20</td><td>2</td><td>FGSG_02211</td><td>-</td></tr>
<tr><td>siRNA-t0313020</td><td>CGAACGGGACCGACGTTTTGGCG</td><td>23</td><td>2</td><td>FgHV1gp2</td><td>-</td></tr>
<tr><td>siRNA-t0215727</td><td>CGAACGTGATCTCATCATCA</td><td>20</td><td>2</td><td>FGSG_12667</td><td>-</td></tr>
<tr><td>siRNA-t0264326</td><td>CGAAGAAGTGACGATACTTG</td><td>20</td><td>2</td><td>FGSG_03153</td><td>-</td></tr>
<tr><td>siRNA-t0235619</td><td>CGAAGGATATGCGATCGAGTT</td><td>21</td><td>2</td><td>FGSG_08637</td><td>K15163|1|0.0|2805|fgr:FG08637.1|mediator of RNA polymerase II transcription subunit 12, fungi type</td></tr>
<tr><td>siRNA-t0253104</td><td>CGACAATGAAAGATCCTAAATCTT</td><td>24</td><td>2</td><td>FgHV1gp2</td><td>-</td></tr>
<tr><td>siRNA-t0235387</td><td>CGACAATGGCGCATACACGG</td><td>20</td><td>2</td><td>FgHV1gp2</td><td>-</td></tr>
<tr><td>siRNA-t0222848</td><td>CGACGACCGTGCGAAATTGTTT</td><td>22</td><td>2</td><td>FgHV1gp2</td><td>-</td></tr>
<tr><td>siRNA-t0243429</td><td>CGAGACCGATGATTTCCATC</td><td>20</td><td>2</td><td>FGSG_12863</td><td>K02324|1|0.0|3986|fgr:FG06065.1|DNA polymerase epsilon subunit 1 [EC:2.7.7.7]</td></tr>
<tr><td>siRNA-t0363708</td><td>CGAGACCGATGATTTCCATCCCCTT</td><td>25</td><td>2</td><td>FgHV1gp2</td><td>-</td></tr>
<tr><td>siRNA-t0233268</td><td>CGCATTTCTTTCTTACCCTCGATGT</td><td>25</td><td>2</td><td>FgHV1gp2</td><td>-</td></tr>
<tr><td>siRNA-t0307849</td><td>CGCCAGTTCTCTTTGTTGGCCT</td><td>22</td><td>2</td><td>FGSG_04690</td><td>-</td></tr>
<tr><td>siRNA-t0274271</td><td>CGCCGCCAGAATCGAGCTAA</td><td>20</td><td>2</td><td>FgHV1gp2</td><td>-</td></tr>
<tr><td>siRNA-t0319069</td><td>CGCCTATATCATCACCTCCA</td><td>20</td><td>2</td><td>FGSG_12441</td><td>-</td></tr>
<tr><td>siRNA-t0253485</td><td>CGCCTATGCTTGTCAGCCTTG</td><td>21</td><td>2</td><td>FgHV1gp2</td><td>-</td></tr>
<tr><td>siRNA-t0231670</td><td>CGCCTCCAGTCCAGCCTTTTGCCT</td><td>24</td><td>2</td><td>FgHV1gp2</td><td>-</td></tr>
<tr><td>siRNA-t0240095</td><td>CGCCTGGACAGTGATTTGTTAC</td><td>22</td><td>2</td><td>FgHV1gp1</td><td>-</td></tr>
<tr><td>siRNA-t0269975</td><td>CGCGAATTCTGCCATAACCTCGT</td><td>23</td><td>2</td><td>FgHV1gp2</td><td>-</td></tr>
</table>

siRNA-t0223627	CGCGCTAAGTTACAGGCCTCA	21	2	FgHV1gp2	-
siRNA-t0230100	CGCTAAGAGGAAAGGAAATAT	21	2	FgHV1gp2	-
siRNA-t0303535	CGCTGCTTGTCAGTGTCAGGATCC	24	2	FgHV1gp2	-
siRNA-t0242652	CGCTTCCCATGACTGCTAGAA	21	2	FGSG_09177	-
siRNA-t0205342	CGCTTGGAAGATCGCTCTTT	20	2	FGSG_05891	K01106|1|4e-43|176|uma:UM02595.1|inositol-1,4,5-trisphosphate 5-phosphatase [EC:3.1.3.56]
siRNA-t0312049	CGGAAGCGTGTTGTTTTCGG	20	2	FGSG_10889	K06681|1|3e-15|84.3|ppa:PAS_chr3_0758|Gdp/GTP exchange factor required for growth at low temperatures
siRNA-t0205622	CGGATACGTTCTGAGAAGTC	20	2	FgHV1gp2	-
siRNA-t0234970	CGGATACGTTCTGAGAAGTCTCCG	24	2	FgHV1gp2	-
siRNA-t0246517	CGGATCCACATGAGCTTCAGCT	22	2	FgHV1gp2	-
siRNA-t0266875	CGGATCTCCGCTCCGATGTCCACAC	25	2	FgHV1gp1	-
siRNA-t0271143	CGGATGGTGTAGAAACTGTC	20	2	FGSG_09638	K11541|1|0.0|4538|fgr:FG09638.1|carbamoyl-phosphate synthase / aspartate carbamoyltransferase [EC:6.3.5.5 2.1.3.2]
siRNA-t0301006	CGGCACCAACTTCAAGAACCC	21	2	FGSG_04694	K15419|1|0.0|1075|ani:AN2547.2|emericellamide biosynthesis, highly reducing iterative type I polyketide synthase;K00680|3|0.0|812|afm:AFUA_3G14700|[EC:2.3.1.-]
siRNA-t0356176	CGGCAGTCCTAGCAAGTGAAACTGT	25	2	FgHV1gp2	-
siRNA-t0224580	CGGCTATAGGAGTACCAACAGC	22	2	FGSG_08853	-
siRNA-t0269754	CGGCTTACCCTCGCATTTGCCGCA	24	2	FgHV1gp2	-
siRNA-t0267060	CGGGACCAGGATTCTTCTCCAC	22	2	FGSG_10617	K14859|1|2e-98|359|scm:SCHCODRAFT_255991|ribosome biogenesis protein SSF1/2;K00143|2|1e-14|82.0|zro:ZYRO0C16566g|L-aminoadipate-semialdehyde dehydrogenase [EC:1.2.1.31]
siRNA-t0317051	CGGGATCAGGTTCACAATTGC	21	2	FgHV1gp2	-
siRNA-t0320481	CGGGATCAGGTTCACAATTGCACAT	25	2	FgHV1gp2	-
siRNA-t0282660	CGGGCCACTCGACCTTGTCGCTGC	24	2	FgHV1gp2	-
siRNA-t0232189	CGGGGTCTCCGATATTCGCTTC	22	2	FGSG_06583	-
siRNA-t0304092	CGGGTTATGATCTGGTTCAC	20	2	FGSG_11471	-
siRNA-t0317503	CGGTCAGACCCCAGATCCACC	21	2	FgHV1gp2	-
siRNA-t0259807	CGGTCAGACCCCAGATCCACCCGG	24	2	FgHV1gp2	-
siRNA-t0332041	CGGTCAGACCCCAGATCCACCCGGA	25	2	FgHV1gp2	-
siRNA-t0253587	CGGTGTGGGTCTTCTGACGAATTT	24	2	FgHV1gp2	-
siRNA-t0249711	CGTAAGCTGCTACTCGAACCGCCC	24	2	FgHV1gp2	-
siRNA-t0307281	CGTACCTGCAGTCACAACTT	20	2	FGSG_01790	K00680|1|0.0|2387|afm:AFUA_3G14700|[EC:2.3.1.-];K15419|2|0.0|1077|ani:AN2547.2|emericellamide biosynthesis, highly reducing iterative type I polyketide synthase;K15384|5|0.0|762|afv:AFLA_066840|aspyridone synthetase, hybrid polyketide synthase / nonribosomal peptide synthetase
siRNA-t0260360	CGTACCTGCAGTCACAACTTG	21	2	FGSG_01743	K01895|1|0.0|1310|fgr:FG01743.1|acetyl-CoA synthetase [EC:6.2.1.1]
siRNA-t0269970	CGTACGGTGCTGATGATCAT	20	2	FGSG_08587	K01183|1|0.0|732|fgr:FG08587.1|chitinase [EC:3.2.1.14]
siRNA-t0262391	CGTACTACCGTTGGTATCCC	20	2	FGSG_01049	-
siRNA-t0244719	CGTAGATGTGATGCCTTGTCTAT	23	2	FgHV1gp2	-
siRNA-t0219897	CGTAGATGTGATGCCTTGTCTATT	24	2	FgHV1gp2	-
siRNA-t0277092	CGTCAAATCAAGTTGGTATCCCTCC	25	2	FgHV1gp2	-
siRNA-t0230316	CGTCAAGTTGTGACTGCAGGTA	22	2	FgHV1gp2	-
siRNA-t0308634	CGTCCGAATACCATACTTTCCG	22	2	FgHV1gp2	-
siRNA-t0323951	CGTCGTCACCTTGATTGGACAT	22	2	FGSG_09156	K01341|1|0.0|1484|fgr:FG09156.1|kexin [EC:3.4.21.61]
siRNA-t0352316	CGTCTCACTGACTTCGTAACGGGT	24	2	FgHV1gp2	-
siRNA-t0363927	CGTGACCAGCTGTTCTCTGT	20	2	FGSG_12508	-
siRNA-t0262386	CGTGACCAGCTGTTCTCTGTC	21	2	FgHV1gp2	-
siRNA-t0289896	CGTGCGCTTCTGGATCAATG	20	2	FGSG_09585	K07117|1|0.0|1683|fgr:FG09585.1|
siRNA-t0331698	CGTGCGCTTCTGGATCAATGTCAGC	25	2	FgHV1gp2	-
siRNA-t0228492	CGTGGAAATCTTCAGGCCCTGGGAT	25	2	FgHV1gp2	-
siRNA-t0270038	CGTGTCGTTAGAAGCTTGACA	21	2	FgHV1gp2	-
siRNA-t0204004	CGTGTCTGACTGTTGCTTGA	20	2	FgHV1gp2	-
siRNA-t0228352	CGTGTCTGACTGTTGCTTGATG	22	2	FgHV1gp2	-
siRNA-t0248031	CGTGTGATTGGTCTCTTGCAGT	22	2	FgHV1gp2	-
siRNA-t0281237	CGTGTGATTGGTCTCTTGCAGTGC	24	2	FgHV1gp2	-
siRNA-t0222505	CGTTACAATCGTGGACTTCCTTTA	24	2	FgHV1gp2	-
siRNA-t0320624	CGTTACGCTGAAGAGTATCTC	21	2	FGSG_06678	K09527|1|0.0|1154|fgr:FG06678.1|DnaJ homolog subfamily C member 7
siRNA-t0337589	CGTTCGGCAACAGATCTTGT	20	2	FGSG_03119	-
siRNA-t0287464	CTCAGAGTCTTGCGGTGTGG	20	2	FGSG_10283	-
siRNA-t0286570	CTCAGAGTCTTGCGGTGTGGG	21	2	FgHV1gp2	-
siRNA-t0215853	CTCAGAGTCTTGCGGTGTGGGTC	23	2	FgHV1gp2	-
siRNA-t0291403	CTCAGAGTCTTGCGGTGTGGGTCT	24	2	FgHV1gp2	-
siRNA-t0257276	CTCAGCCACGCCTGTCGACG	20	2	FGSG_07258	K14572|1|0.0|8396|fgr:FG07258.1|midasin
siRNA-t0254877	CTCATCGTTGTGCCAACCAT	20	2	FGSG_09976	-
siRNA-t0204030	CTCCACAAACGCTCATGATAACAT	24	2	FgHV1gp2	-
siRNA-t0293519	CTCCACGTCATGATCATCAGC	21	2	FGSG_10904	K06681|1|1e-61|238|cdu:CD36_21160|Gdp/GTP exchange factor required for growth at low temperatures
siRNA-t0279972	CTCCCAAGGAATTCCAGACT	20	2	FGSG_08422	K10589|1|0.0|2246|fgr:FG08422.1|ubiquitin-protein ligase E3 C [EC:6.3.2.19]
siRNA-t0329215	CTCCCTCATACTTCGCCTTCA	21	2	FGSG_03607	K05349|1|0.0|637|pte:PTT_17365|beta-glucosidase [EC:3.2.1.21]
siRNA-t0222722	CTCCCTTCGTGAACTGCTGC	20	2	FGSG_00541	K05658|1|0.0|659|cci:CC1G_03025|ATP-binding cassette, subfamily B (MDR/TAP), member 1
siRNA-t0365989	CTCCGATGAATCCTCTTCCGAT	22	2	FgHV1gp2	-
siRNA-t0366046	CTCCGCAGAAGAATTAGCCTTTGAA	25	2	FgHV1gp2	-
siRNA-t0235179	CTCGATTTCGCAACCACAAGCCAC	24	2	FgHV1gp2	-
siRNA-t0352805	CTCTGACATATTCGGACTCATCCCA	25	2	FgHV1gp2	-
siRNA-t0316319	CTCTGTGACTCCAGCCAAAGAGA	23	2	FgHV1gp2	-
siRNA-t0356059	CTCTTCAGCGTAACGTGACC	20	2	FgHV1gp2	-
siRNA-t0206982	CTCTTCAGCGTAACGTGACCAGC	23	2	FgHV1gp2	-
siRNA-t0345322	CTCTTCCAGTGTCGAACCATACT	23	2	FgHV1gp2	-
siRNA-t0306180	CTCTTTGTTGGCCTGGTAAT	20	2	FGSG_14023	-
siRNA-t0279870	CTGACAATGGTTTGTTCTGAA	21	2	FgHV1gp2	-
siRNA-t0340701	CTGAGTCATTTTTGCACCAGGA	22	2	FgHV1gp2	-
siRNA-t0220682	CTGATATATTCCAACTGCGACCCA	24	2	FgHV1gp2	-
siRNA-t0291504	CTGCAGTGGCTTGTGGTTGC	20	2	FGSG_12506	-
siRNA-t0341438	CTGCCGTCTCCCAAAAAGGCAT	22	2	FGSG_00767	K05349|1|0.0|656|pno:SNOG_04685|beta-glucosidase [EC:3.2.1.21]
siRNA-t0271125	CTGCTACTCGAACCGCCCTTGTAGC	25	2	FgHV1gp2	-
siRNA-t0257991	CTGGAACGTCAAATCAAGTTG	21	2	FgHV1gp2	-
siRNA-t0229788	GAAAAGTTCTCGCCAGCAATGCCC	24	2	FgHV1gp1	-
siRNA-t0218789	GAAACCATCAGGACCCGAGAA	21	2	FgHV1gp2	-
siRNA-t0285908	GAAACCATCAGGACCCGAGAAAAAC	25	2	FgHV1gp2	-
siRNA-t0350425	GAAAGACCACATCCAGATCTT	21	2	FgHV1gp2	-
siRNA-t0306247	GAAAGCCTCATCAATGTGGTATA	23	2	FgHV1gp1	-
siRNA-t0236517	GAAAGGAAAAGTTCTCGCCA	20	2	FgHV1gp1	-
siRNA-t0283807	GAAAGGGGCCAAGCTCGTCC	20	2	FGSG_10003	K00223|1|0.0|1142|fgr:FG10003.1|delta24(24(1))-sterol reductase [EC:1.3.1.71]
siRNA-t0251219	GAACATCTGGATGTGGGTGC	20	2	FGSG_11613	-
siRNA-t0269697	GAACCACAACGATAGTATGAAC	22	2	FgHV1gp2	-
siRNA-t0341401	GAACCTCCGATGAATCCTCTT	21	2	FgHV1gp2	-
siRNA-t0364551	GAATCCTTTTGTACCCTTCTGT	22	2	FGSG_06101	K14780|1|0.0|2204|fgr:FG06101.1|ATP-dependent RNA helicase DHX37/DHR1 [EC:3.6.4.13]
siRNA-t0304196	GAATGGTCTGCCTAATCTTTC	21	2	FgHV1gp2	-
siRNA-t0220670	GAATTCTAGCCTGGTTGAGATC	22	2	FgHV1gp2	-

<table>
<tr><td></td><td></td><td></td><td></td><td></td><td></td></tr>
<tr><td>siRNA-t0291163</td><td>GACAACAGGCCAGTCCATCT</td><td>20</td><td>2</td><td>FGSG_09329</td><td>K08711|1|0.0|1646|cne:CNB05770|ATP-binding cassette, subfamily G (WHITE), member 2, PDR</td></tr>
<tr><td>siRNA-t0225525</td><td>GACAATGGCGCATACACGGAACCC</td><td>24</td><td>2</td><td>FgHV1gp2</td><td>-</td></tr>
<tr><td>siRNA-t0299770</td><td>GACACCGACGGTACGTGGAAA</td><td>21</td><td>2</td><td>FgHV1gp2</td><td>-</td></tr>
<tr><td>siRNA-t0256789</td><td>GACACTTGTTTCTCGTGGGA</td><td>20</td><td>2</td><td>FGSG_07509</td><td>K08157|1|4e-46|185|kla:KLLA0F18106g|MFS transporter, DHA1 family, multidrug resistance protein</td></tr>
<tr><td>siRNA-t0282002</td><td>GACAGTTCCGCGTGTGATTGG</td><td>21</td><td>2</td><td>FgHV1gp2</td><td>-</td></tr>
<tr><td>siRNA-t0253260</td><td>GACATATAATAGACAAGGCATCA</td><td>23</td><td>2</td><td>FGSG_09129</td><td>K12823|1|6e-08|60.5|ure:UREG_02211|ATP-dependent RNA helicase DDX5/DBP2 [EC:3.6.4.13];K06694|4|7e-08|60.1|vpo:Kpol_1050p62|26S proteasome non-ATPase regulatory subunit 10</td></tr>
<tr><td>siRNA-t0331403</td><td>GACCCATGCTTTCGACGTACTTG</td><td>23</td><td>2</td><td>FgHV1gp2</td><td>-</td></tr>
<tr><td>siRNA-t0251623</td><td>GAGTGCTGAATGCTCTGCCTC</td><td>21</td><td>2</td><td>FgHV1gp2</td><td>-</td></tr>
<tr><td>siRNA-t0264807</td><td>GAGTTGTAGAAATAACCACATTT</td><td>23</td><td>2</td><td>FgHV1gp2</td><td>-</td></tr>
<tr><td>siRNA-t0219796</td><td>GATACTCAAATATCGTACCGCA</td><td>22</td><td>2</td><td>FGSG_12710</td><td>-</td></tr>
<tr><td>siRNA-t0212029</td><td>GATAGTGGGAGCGAAAGCGAT</td><td>21</td><td>2</td><td>FGSG_08335</td><td>K02873|1|8e-89|325|fgr:FG08335.1|large subunit ribosomal protein L13e</td></tr>
<tr><td>siRNA-t0311223</td><td>GATCCCACTCTAGCAATGCATC</td><td>22</td><td>2</td><td>FgHV1gp1</td><td>-</td></tr>
<tr><td>siRNA-t0315695</td><td>GATCTCAGCACGCCGGTTACC</td><td>21</td><td>2</td><td>FgHV1gp2</td><td>-</td></tr>
<tr><td>siRNA-t0366117</td><td>GATCTCATCATCACCAGACTCTAA</td><td>24</td><td>2</td><td>FGSG_01210</td><td>K12854|1|0.0|4128|fgr:FG01210.1|pre-mRNA-splicing helicase BRR2 [EC:3.6.4.13]</td></tr>
<tr><td>siRNA-t0227287</td><td>GATCTCCGCTCCGATGTCCACAC</td><td>23</td><td>2</td><td>FgHV1gp1</td><td>-</td></tr>
<tr><td>siRNA-t0273659</td><td>GATCTGGCCGGTTGAAAATG</td><td>20</td><td>2</td><td>FgHV1gp2</td><td>-</td></tr>
<tr><td>siRNA-t0361623</td><td>GATCTTCCCTATGGCCTCGCAT</td><td>22</td><td>2</td><td>FgHV1gp2</td><td>-</td></tr>
<tr><td>siRNA-t0299291</td><td>GATCTTGCTGTAGTGTCTGAAG</td><td>22</td><td>2</td><td>FgHV1gp2</td><td>-</td></tr>
<tr><td>siRNA-t0252428</td><td>GATCTTGCTGTAGTGTCTGAAGATC</td><td>25</td><td>2</td><td>FgHV1gp2</td><td>-</td></tr>
<tr><td>siRNA-t0316911</td><td>GATGGAAGTGATGGCAATCA</td><td>20</td><td>2</td><td>FGSG_13065</td><td>K13800|1|0.0|912|fgr:FG07124.1|UMP-CMP kinase [EC:2.7.4.- 2.7.4.14]</td></tr>
<tr><td>siRNA-t0343453</td><td>GATGGTGATAGACTTTTGCTA</td><td>21</td><td>2</td><td>FGSG_10379</td><td>K01930|1|2e-73|276|ncr:NCU01337|folylpolyglutamate synthase [EC:6.3.2.17]</td></tr>
<tr><td>siRNA-t0236206</td><td>GATGTAGGCTCCTCCGACAATGG</td><td>23</td><td>2</td><td>FgHV1gp2</td><td>-</td></tr>
<tr><td>siRNA-t0326121</td><td>GATGTGGGTGCGCCGAATGAA</td><td>21</td><td>2</td><td>FgHV1gp2</td><td>-</td></tr>
<tr><td>siRNA-t0362051</td><td>GATGTTCTTGTGCCTCCGAGCA</td><td>22</td><td>2</td><td>FgHV1gp2</td><td>-</td></tr>
<tr><td>siRNA-t0341775</td><td>GATTGAAGTGTAATGCGTTGATA</td><td>23</td><td>2</td><td>FgHV1gp2</td><td>-</td></tr>
<tr><td>siRNA-t0229909</td><td>GATTGGCGTGAGTGCGTAGATAA</td><td>23</td><td>2</td><td>FgHV1gp2</td><td>-</td></tr>
<tr><td>siRNA-t0239895</td><td>GATTGGTCTCTTGCAGTGCA</td><td>20</td><td>2</td><td>FGSG_11026</td><td>K01932|1|0.0|1527|afm:AFUA_1G17200|[EC:6.3.2.-]</td></tr>
<tr><td>siRNA-t0219461</td><td>GATTGGTGTCCAATGTAAGTGACG</td><td>24</td><td>2</td><td>FgHV1gp2</td><td>-</td></tr>
<tr><td>siRNA-t0285207</td><td>GATTTGCCTGTGCCGGTGTC</td><td>20</td><td>2</td><td>FGSG_11372</td><td>K00777|1|5e-21|103|afm:AFUA_7G06290|[EC:2.4.2.-];K12823|4|1e-10|69.7|ure:UREG_02211|ATP-dependent RNA helicase DDX5/DBP2 [EC:3.6.4.13]</td></tr>
<tr><td>siRNA-t0229961</td><td>GATTTTACAAAGTCATTGTTCATC</td><td>24</td><td>2</td><td>FgHV1gp2</td><td>-</td></tr>
<tr><td>siRNA-t0266035</td><td>GATTTTGTGTCGCCTCCAGACG</td><td>22</td><td>2</td><td>FgHV1gp2</td><td>-</td></tr>
<tr><td>siRNA-t0232747</td><td>GATTTTGTGTCGCCTCCAGACGG</td><td>23</td><td>2</td><td>FgHV1gp2</td><td>-</td></tr>
<tr><td>siRNA-t0302222</td><td>GCAAATTGTAGCTTGCTAACT</td><td>21</td><td>2</td><td>FGSG_04948</td><td>K00507|1|4e-31|137|cci:CC1G_15704|stearoyl-CoA desaturase (delta-9 desaturase) [EC:1.14.19.1]</td></tr>
<tr><td>siRNA-t0337411</td><td>GCAACAGACTTCTTCTCTGCTAT</td><td>23</td><td>2</td><td>FgHV1gp2</td><td>-</td></tr>
<tr><td>siRNA-t0263396</td><td>GCAAGTCTTCGTCCAGTAGCCA</td><td>22</td><td>2</td><td>FgHV1gp2</td><td>-</td></tr>
<tr><td>siRNA-t0340875</td><td>GCAAGTCTTCGTCCAGTAGCCAAT</td><td>24</td><td>2</td><td>FgHV1gp2</td><td>-</td></tr>
<tr><td>siRNA-t0250485</td><td>GCAATGCAACAGACTTCTTCT</td><td>21</td><td>2</td><td>FGSG_13783</td><td>K01932|1|0.0|1526|afm:AFUA_5G12730|[EC:6.3.2.-]</td></tr>
<tr><td>siRNA-t0243854</td><td>GCAATGCATCACCAAGCCAA</td><td>20</td><td>2</td><td>FGSG_04351</td><td>K15361|1|0.0|1859|fgr:FG04351.1|WD repeat-containing protein 48</td></tr>
<tr><td>siRNA-t0286731</td><td>GCACCGTACGGATCCACATG</td><td>20</td><td>2</td><td>FgHV1gp2</td><td>-</td></tr>
<tr><td>siRNA-t0295898</td><td>GCACCGTACGGATCCACATGA</td><td>21</td><td>2</td><td>FgHV1gp2</td><td>-</td></tr>
<tr><td>siRNA-t0221183</td><td>GCACCGTACGGATCCACATGAGCTT</td><td>25</td><td>2</td><td>FgHV1gp2</td><td>-</td></tr>
<tr><td>siRNA-t0314667</td><td>GCATAGCCCCGGATCGGTAA</td><td>20</td><td>2</td><td>FGSG_08185</td><td>-</td></tr>
<tr><td>siRNA-t0244663</td><td>GCATCCTCGACCCACTCATGGGC</td><td>23</td><td>2</td><td>FgHV1gp2</td><td>-</td></tr>
<tr><td>siRNA-t0256988</td><td>GCATGGCCGGCAGTCCTAGCAAGT</td><td>24</td><td>2</td><td>FgHV1gp2</td><td>-</td></tr>
<tr><td>siRNA-t0278374</td><td>GCATGTACTGTCCGTAATTG</td><td>20</td><td>2</td><td>FGSG_08568</td><td>K11236|1|1e-09|65.5|uma:UM02422.1|cell division control protein 24</td></tr>
<tr><td>siRNA-t0325775</td><td>GCCAAAGAGAGGTGCGTAGCAT</td><td>22</td><td>2</td><td>FGSG_03964</td><td>K15316|1|3e-170|599|ani:AN7825.2|sterigmatocystin polyketide synthase;K15321|4|6e-165|582|nfi:NFIA_093000|naphtho-gamma-pyrone polyketide synthase</td></tr>
<tr><td>siRNA-t0313886</td><td>GCCAGTCCATCTTGCAGCACA</td><td>21</td><td>2</td><td>FGSG_01433</td><td>K00264|1|0.0|4368|fgr:FG01433.1|glutamate synthase (NADPH/NADH) [EC:1.4.1.13 1.4.1.14]</td></tr>
<tr><td>siRNA-t0355013</td><td>GCCAGTTCTCTTTGTTGGCCTG</td><td>22</td><td>2</td><td>FGSG_04690</td><td>-</td></tr>
<tr><td>siRNA-t0358057</td><td>GCCATAGCGCTGGTGTTGAAT</td><td>21</td><td>2</td><td>FgHV1gp2</td><td>-</td></tr>
<tr><td>siRNA-t0335556</td><td>GCCCAGTCATGAATTTTCGG</td><td>20</td><td>2</td><td>FgHV1gp2</td><td>-</td></tr>
<tr><td>siRNA-t0266073</td><td>GCCCGCTTACTCATCATGTAA</td><td>21</td><td>2</td><td>FgHV1gp2</td><td>-</td></tr>
<tr><td>siRNA-t0312826</td><td>GCCCGCTTACTCATCATGTAAGGAT</td><td>25</td><td>2</td><td>FgHV1gp2</td><td>-</td></tr>
<tr><td>siRNA-t0323469</td><td>GCCCTACACGCTCTGCTTAC</td><td>20</td><td>2</td><td>FGSG_05049</td><td>K14191|1|0.0|673|fgr:FG05049.1|18S rRNA (adenine1779-N6/adenine1780-N6)-dimethyltransferase [EC:2.1.1.183]</td></tr>
<tr><td>siRNA-t0302633</td><td>GCCGCGGATAACCTATACTT</td><td>20</td><td>2</td><td>FgHV1gp2</td><td>-</td></tr>
<tr><td>siRNA-t0348107</td><td>GCCGGCAGTCCTAGCAAGTGAAACT</td><td>25</td><td>2</td><td>FgHV1gp2</td><td>-</td></tr>
<tr><td>siRNA-t0217510</td><td>GCCTCCCACGTTCCGCCTCCTTC</td><td>23</td><td>2</td><td>FGSG_01190</td><td>-</td></tr>
<tr><td>siRNA-t0259354</td><td>GCCTCCTTCGAACTAGATGGTGT</td><td>23</td><td>2</td><td>FgHV1gp2</td><td>-</td></tr>
<tr><td>siRNA-t0240590</td><td>GCCTCGGTCAGACCCCAGAT</td><td>20</td><td>2</td><td>FgHV1gp2</td><td>-</td></tr>
<tr><td>siRNA-t0277424</td><td>GCCTCTGACGTTGATCTCTTA</td><td>21</td><td>2</td><td>FgHV1gp2</td><td>-</td></tr>
<tr><td>siRNA-t0350272</td><td>GCCTGCGACCTATCTGTCTT</td><td>20</td><td>2</td><td>FGSG_02026</td><td>K11665|1|0.0|3015|fgr:FG02026.1|DNA helicase INO80 [EC:3.6.4.12]</td></tr>
<tr><td>siRNA-t0227292</td><td>GCCTGGACAGTGATTTGTTACC</td><td>22</td><td>2</td><td>FgHV1gp1</td><td>-</td></tr>
<tr><td>siRNA-t0349419</td><td>GCCTGGGCAAGTGGTTCACC</td><td>20</td><td>2</td><td>FGSG_13878</td><td>K01932|1|0.0|1450|afm:AFUA_5G12730|[EC:6.3.2.-]</td></tr>
<tr><td>siRNA-t0219475</td><td>GCCTTCCATGCTGCCGCGGAT</td><td>21</td><td>2</td><td>FGSG_04587</td><td>K00777|1|7e-77|289|afm:AFUA_7G07100|[EC:2.4.2.-];K14855|4|5e-55|216|lbc:LACBIDRAFT_296068|ribosome assembly protein 4</td></tr>
<tr><td>siRNA-t0339738</td><td>GCGAACCTCAAATCCAAAAGACGCG</td><td>25</td><td>2</td><td>FgHV1gp2</td><td>-</td></tr>
<tr><td>siRNA-t0310018</td><td>GCGAACGGGACCGACGTTTTG</td><td>21</td><td>2</td><td>FGSG_02211</td><td>-</td></tr>
<tr><td>siRNA-t0230342</td><td>GCGAACGGGACCGACGTTTTGG</td><td>22</td><td>2</td><td>FGSG_02211</td><td>-</td></tr>
<tr><td>siRNA-t0228451</td><td>GCGAACGGGACCGACGTTTTGGCGA</td><td>25</td><td>2</td><td>FgHV1gp2</td><td>-</td></tr>
<tr><td>siRNA-t0340387</td><td>GCGAATGTTTCACTCCTCCTT</td><td>21</td><td>2</td><td>FgHV1gp2</td><td>-</td></tr>
<tr><td>siRNA-t0288909</td><td>GCGACGTGCTTGAAGGCGAAG</td><td>21</td><td>2</td><td>FgHV1gp2</td><td>-</td></tr>
<tr><td>siRNA-t0297802</td><td>GCGAGACCGATGATTTCCATCCC</td><td>23</td><td>2</td><td>FgHV1gp2</td><td>-</td></tr>
<tr><td>siRNA-t0272148</td><td>GCGAGTCTAATCTTCTTTGT</td><td>20</td><td>2</td><td>FgHV1gp2</td><td>-</td></tr>
<tr><td>siRNA-t0255027</td><td>GCGCATCGGCTTACCCTCGC</td><td>20</td><td>2</td><td>FGSG_05873</td><td>-</td></tr>
<tr><td>siRNA-t0240273</td><td>GCGCATTTCTTTCTTACCCTCGAT</td><td>24</td><td>2</td><td>FgHV1gp2</td><td>-</td></tr>
<tr><td>siRNA-t0273954</td><td>GCGCCGAATGAAAGCCCAGTGGC</td><td>23</td><td>2</td><td>FgHV1gp2</td><td>-</td></tr>
<tr><td>siRNA-t0297584</td><td>GCGCCGGATACGTTCTGAGAAG</td><td>22</td><td>2</td><td>FgHV1gp2</td><td>-</td></tr>
<tr><td>siRNA-t0310742</td><td>GCGCCTCCATGCATGTAGATCTTG</td><td>24</td><td>2</td><td>FgHV1gp2</td><td>-</td></tr>
<tr><td>siRNA-t0356061</td><td>GCGCGTCTCACTGACTTCGTAA</td><td>22</td><td>2</td><td>FgHV1gp2</td><td>-</td></tr>
<tr><td>siRNA-t0223989</td><td>GCGGGTTATGATCTGGTTCA</td><td>20</td><td>2</td><td>FGSG_03610</td><td>K15385|1|4e-17|88.2|nfi:NFIA_001560|aspyridone synthetase trans-acting enoyl reductase;K00344|4|1e-13|76.6|pcs:Pc21g06300|NADPH2:quinone reductase [EC:1.6.5.5]</td></tr>
<tr><td>siRNA-t0350628</td><td>GCGGTCATAAGTGACAAAAGCATGG</td><td>25</td><td>2</td><td>FgHV1gp2</td><td>-</td></tr>
<tr><td>siRNA-t0215244</td><td>GCTCCGATGTCCACACCGCGT</td><td>21</td><td>2</td><td>FGSG_11148</td><td>-</td></tr>
<tr><td>siRNA-t0247309</td><td>GCTCCGATGTCCACACCGCGTT</td><td>22</td><td>2</td><td>FGSG_11148</td><td>-</td></tr>
<tr><td>siRNA-t0322945</td><td>GCTCGTCCCTCGTGCAATGTAT</td><td>22</td><td>2</td><td>FGSG_03927</td><td>-</td></tr>
<tr><td>siRNA-t0234419</td><td>GCTCGTGACTTTTCCCTCACAG</td><td>22</td><td>2</td><td>FgHV1gp2</td><td>-</td></tr>
<tr><td>siRNA-t0310493</td><td>GCTCTGCCTCGGTCAGACCCCA</td><td>22</td><td>2</td><td>FgHV1gp2</td><td>-</td></tr>
<tr><td>siRNA-t0293094</td><td>GCTCTGTGACTCCAGCCAAAGAG</td><td>23</td><td>2</td><td>FgHV1gp2</td><td>-</td></tr>
<tr><td>siRNA-t0248477</td><td>GCTGAACCCAGTTGCTGTATC</td><td>21</td><td>2</td><td>FGSG_06379</td><td>K14289|1|0.0|2450|fgr:FG06379.1|exportin-5</td></tr>
<tr><td>siRNA-t0218731</td><td>GCTGAGATCAAGTATAGGTT</td><td>20</td><td>2</td><td>FGSG_10790</td><td>K15389|1|3e-101|368|ure:UREG_05875|acyl-CoA dehydrogenase;K00257|2|1e-100|366|afm:AFUA_6G10880|[EC:1.3.99.-]</td></tr>
<tr><td>siRNA-t0263957</td><td>GCTGAGATCAAGTATAGGTTA</td><td>21</td><td>2</td><td>FGSG_01936</td><td>K09244|1|1e-68|260|cpw:CPC735_071800|transcriptional regulatory protein AMDR</td></tr>
<tr><td>siRNA-t0211142</td><td>GCTGCTACTCGAACCGCCCTTGT</td><td>23</td><td>2</td><td>FgHV1gp2</td><td>-</td></tr>
</table>

siRNA-t0255480	GCTTAGCGTGTTGTACGGCG	20	2	FGSG_12323	-
siRNA-t0255357	GCTTTACCGGATCTCCGCTC	20	2	FgHV1gp1	-
siRNA-t0305740	GCTTTACCGGATCTCCGCTCC	21	2	FgHV1gp1	-
siRNA-t0263890	GCTTTACCGGATCTCCGCTCCGATG	25	2	FgHV1gp1	-
siRNA-t0210321	GCTTTCGACGTACTTGGGTTC	21	2	FgHV1gp2	-
siRNA-t0277192	GGAAAGGAAAAGTTCTCGCC	20	2	FgHV1gp1	-
siRNA-t0217359	GGAAGTTTTCTTGTCCTTCTCCA	23	2	FGSG_02751	K00469|1|5e-176|615|fgr:FG02751.1|inositol oxygenase [EC:1.13.99.1]
siRNA-t0266407	GGAATAAGCCACACCCTTAGGACC	24	2	FgHV1gp2	-
siRNA-t0339875	GGACCCGAGAAAAACGCCCCTGGA	24	2	FgHV1gp2	-
siRNA-t0296067	GGACGCGACAAGCAACAATTTG	22	2	FgHV1gp2	-
siRNA-t0310381	GGACGCGACAAGCAACAATTTGCCA	25	2	FgHV1gp2	-
siRNA-t0290508	GGACGTTCTGGTCTTCGATGG	21	2	FgHV1gp2	-
siRNA-t0326247	GGAGCTCAAAGGTATGATGT	20	2	FGSG_08060	K00688|1|4e-11|69.3|pno:SNOG_13460|starch phosphorylase [EC:2.4.1.1]
siRNA-t0353181	GGAGTGGCATATTCAAGAATC	21	2	FgHV1gp2	-
siRNA-t0218913	GGATCCGTCCAGGGAAGTGG	20	2	FGSG_03452	K01133|1|3e-06|53.5|afv:AFLA_033500|choline-sulfatase [EC:3.1.6.6]
siRNA-t0334929	GGATCGTTATTCTTCTTCTC	20	2	FGSG_09236	-
siRNA-t0294825	GGATGCAAAACCGGTGCGATA	21	2	FgHV1gp2	-
siRNA-t0253649	GGATGCTTTATCCTTGGCTT	20	2	FGSG_09236	-
siRNA-t0205150	GGATTAATCAATGTACCGTTCG	22	2	FgHV1gp2	-
siRNA-t0288438	GGCAGTCCTAGCAAGTGAAACTGTT	25	2	FgHV1gp2	-
siRNA-t0266785	GGCATTGCCTTGACCGCGTT	20	2	FGSG_11988	K05658|1|0.0|2439|fgr:FG01684.1|ATP-binding cassette, subfamily B (MDR/TAP), member 1
siRNA-t0267898	GGCCAAATCCTCAGAGTCTTGC	22	2	FGSG_11099	-
siRNA-t0214562	GGCCAAATCGTCATACATTGCACGA	25	2	FgHV1gp2	-
siRNA-t0249123	GGCCGTAAGTAGCAGATTTTACAA	24	2	FgHV1gp2	-
siRNA-t0224250	GGCCTGGTAATACCTGATGGCAGT	24	2	FgHV1gp2	-
siRNA-t0316984	GGCGAGTCTAATCTTCTTTG	20	2	FgHV1gp2	-
siRNA-t0295346	GGCGTCGACACGATGATACCC	21	2	FgHV1gp2	-
siRNA-t0211264	GGCGTGAGTGCGTAGATAAA	20	2	FgHV1gp2	-
siRNA-t0315853	GGCGTGGTCTCGTTCCCTCC	20	2	FGSG_13479	-
siRNA-t0252435	GGCTCCACAAACGCTCATGA	20	2	FGSG_13896	-
siRNA-t0257472	GGCTCCACAAACGCTCATGAT	21	2	FgHV1gp2	-
siRNA-t0361382	GGCTCCTCCGACAATGGCGCATA	23	2	FGSG_10215	K15289|1|2e-09|62.8|yli:YALI0E22803g|solute carrier family 35, member F5;K15303|2|4e-09|62.0|ani:AN0675.2|aflatoxin B1 aldehyde reductase
siRNA-t0207588	GGGGTCCTCTTATTGATATTAGCCC	25	2	FgHV1gp2	-
siRNA-t0260358	GGGGTGTGTATCCAATGAGTTGTG	24	2	FgHV1gp2	-
siRNA-t0269398	GGGTCTAAAGGAGTGGATCG	20	2	FGSG_04951	K14327|1|1e-87|322|ang:ANI_1_332064|regulator of nonsense transcripts 2
siRNA-t0202047	GGGTCTCCGATATTCGCTTCGCA	23	2	FgHV1gp1	-
siRNA-t0235729	GGGTGCGCCGAATGAAAGCCCAG	23	2	FgHV1gp2	-
siRNA-t0299195	GGGTGCTCATGAGGTTGGCATTG	23	2	FgHV1gp2	-
siRNA-t0308204	GGGTGGATCTGGGGTCTGACC	21	2	FGSG_09943	-
siRNA-t0232140	GGGTTGGTAATTGACAAAGGCCTAT	25	2	FgHV1gp2	-
siRNA-t0348586	GGTGGTTCATTGCGATGTAATCTTG	25	2	FgHV1gp2	-
siRNA-t0252894	GGTGTGAAGCTTGGATGAGC	20	2	FGSG_06572	K14644|1|4e-06|53.1|lth:KLTH0A06094g|subtilase-type proteinase RRT12 [EC:3.4.21.-];K01341|3|7e-06|52.4|mgl:MGL_3817|kexin [EC:3.4.21.61]
siRNA-t0300590	GGTGTGAAGCTTGGATGAGCGA	22	2	FGSG_09637	-
siRNA-t0246182	GGTGTGGGTCTTCTGACGAATT	22	2	FgHV1gp2	-
siRNA-t0206063	GGTTACCAAGGTGTGTGAAGTA	22	2	FGSG_09801	K03164|1|0.0|2817|fgr:FG09801.1|DNA topoisomerase II [EC:5.99.1.3]
siRNA-t0334838	GGTTCGAGTTGTATGAAAAC	20	2	FGSG_06589	-
siRNA-t0331138	GGTTGAGGCTTCTTCATGACAT	22	2	FGSG_01847	-
siRNA-t0340545	GGTTTGAATGGTCTGCCTAATCTT	24	2	FgHV1gp2	-
siRNA-t0266418	GTAAAGAGTTGCGATAGTGTCAT	23	2	FGSG_01589	K00777|1|3e-17|91.3|afm:AFUA_7G06290|[EC:2.4.2.-];K12823|3|3e-11|71.6|ure:UREG_02211|ATP-dependent RNA helicase DDX5/DBP2 [EC:3.6.4.13]
siRNA-t0218891	GTAACGGGTCCAGATGTTGTGCT	23	2	FgHV1gp2	-
siRNA-t0345184	GTAAGCAGAGCGTGTAGGGCTGT	23	2	FgHV1gp2	-
siRNA-t0304626	GTAAGCTGCTACTCGAACCGCCCTT	25	2	FgHV1gp2	-
siRNA-t0331015	GTAAGGATCTTTTGGCATCA	20	2	FGSG_01839	K02685|1|0.0|1045|fgr:FG01839.1|DNA primase large subunit [EC:2.7.7.-]
siRNA-t0261951	GTAAGTCTGGAATTCCTTGGG	21	2	FgHV1gp2	-
siRNA-t0270601	GTAATACCTGATGGCAGTTGACC	23	2	FgHV1gp2	-
siRNA-t0257074	GTAATGGAGGACGATCTCATTCGT	24	2	FgHV1gp2	-
siRNA-t0359097	GTACAGTCCCAGCGCCTCCA	20	2	FGSG_13581	-
siRNA-t0226078	GTACCATTGCTGCATGTACTGTTCG	25	2	FgHV1gp2	-
siRNA-t0234244	GTACCGCAGTGATGGAGACT	20	2	FGSG_09869	K12669|1|0.0|647|fgr:FG09869.1|oligosaccharyltransferase complex subunit gamma
siRNA-t0238204	GTACCGCAGTGATGGAGACTA	21	2	FGSG_09869	K12669|1|0.0|647|fgr:FG09869.1|oligosaccharyltransferase complex subunit gamma
siRNA-t0245123	GTACCGTTCGGCAACAGATCT	21	2	FgHV1gp2	-
siRNA-t0278456	GTACCTGTTGTTGGTCTTCCAGGC	24	2	FgHV1gp2	-
siRNA-t0338184	GTACGGATCCACATGAGCTTCA	22	2	FgHV1gp2	-
siRNA-t0345015	GTACGGATCCACATGAGCTTCAGC	24	2	FgHV1gp2	-
siRNA-t0338212	GTACTTGCCGCGCGTCTCAC	20	2	FGSG_06580	K11262|1|0.0|4510|fgr:FG06580.1|acetyl-CoA carboxylase / biotin carboxylase [EC:6.4.1.2 6.3.4.14]
siRNA-t0257809	GTACTTGCCGCGCGTCTCACT	21	2	FGSG_06580	K11262|1|0.0|4510|fgr:FG06580.1|acetyl-CoA carboxylase / biotin carboxylase [EC:6.4.1.2 6.3.4.14]
siRNA-t0358686	GTAGAACAGACGTCTCTCACGC	22	2	FgHV1gp2	-
siRNA-t0298955	GTATCACCATGACGATCGAT	20	2	FGSG_13662	K03018|1|0.0|2938|fgr:FG09860.1|DNA-directed RNA polymerase III subunit RPC1 [EC:2.7.7.6]
siRNA-t0331517	GTATCCCATAGTGCCCGGCCGTT	23	2	FgHV1gp2	-
siRNA-t0361852	GTATGAACCCTTCCACGATTGG	22	2	FgHV1gp2	-
siRNA-t0339692	GTCAAATCAAGTTGGTATCCCTC	23	2	FgHV1gp2	-
siRNA-t0311545	GTCACCCTTTGGAGACTTGGCAGT	24	2	FgHV1gp2	-
siRNA-t0305456	GTCACTCCCGCTCGATCTGCG	21	2	FGSG_00618	-
siRNA-t0256692	GTCAGTGTCAGGATCCGTCCAGGGA	25	2	FgHV1gp2	-
siRNA-t0352557	GTCATGAAGAAGCCTCAACCA	21	2	FGSG_06462	K01976|1|6e-46|186|ago:AGOS_AAR168C|[EC:6.-.-.-];K01897|4|3e-21|104|afm:AFUA_1G17190|long-chain acyl-CoA synthetase [EC:6.2.1.3]
siRNA-t0270701	GTCATGAATTTTCGGTAGTCATC	23	2	FgHV1gp2	-
siRNA-t0294403	GTCCAATGTAAGTGACGAGGCCTC	24	2	FgHV1gp2	-
siRNA-t0312949	GTCCACACCGCGTTTCGCTTGCCA	24	2	FgHV1gp1	-
siRNA-t0262021	GTCCCGCTGTTTCACTTGTAGGA	23	2	FgHV1gp2	-
siRNA-t0227490	GTCCGGAATGCGTTCACACG	20	2	FgHV1gp2	-
siRNA-t0273693	GTCCGGAATGCGTTCACACGAT	22	2	FgHV1gp2	-
siRNA-t0210797	GTCGAAAGTGAAAGTGTCGTAG	22	2	FgHV1gp1	-
siRNA-t0361841	GTCGGCCTTGGAATCGGTCAT	21	2	FGSG_08386	K01764|1|7e-171|598|fgr:FG08386.1|cytochrome c heme-lyase [EC:4.4.1.17]
siRNA-t0359011	GTCTCCGAACGTGATCTCAT	20	2	FgHV1gp2	-
siRNA-t0288970	GTCTCTTGTTCTGGAACGTCAAA	23	2	FgHV1gp2	-
siRNA-t0257808	GTCTTCGTCCAGTAGCCAATGA	22	2	FGSG_06448	-
siRNA-t0312727	GTCTTCGTCCAGTAGCCAATGAGA	24	2	FgHV1gp2	-
siRNA-t0305416	GTCTTCTGACAATGGTTTGTTCTG	24	2	FgHV1gp2	-
siRNA-t0349166	GTCTTGCGGTGTGGGTCTTCTGA	23	2	FgHV1gp2	-
siRNA-t0222822	GTGACAAAAGCATGGCGATGCACT	24	2	FgHV1gp2	-
siRNA-t0360883	GTGACGAGGCCTCTGACGTTGA	22	2	FgHV1gp2	-
siRNA-t0313753	GTGATACCCATTTGCGCACAT	21	2	FgHV1gp2	-
siRNA-t0244241	GTGATCCTAAACATGTCGGT	20	2	FGSG_11659	K01932|1|0.0|1431|afm:AFUA_5G12730|[EC:6.3.2.-]
siRNA-t0318670	GTGCCAACCATCAAGCAACA	20	2	FGSG_10611	K00309|1|3e-33|142|afm:AFUA_7G01560|[EC:1.5.3.-]
siRNA-t0230762	GTGCCATGACGTACGACCCCG	21	2	FgHV1gp1	-
siRNA-t0260396	GTGCGACGACCGTGCGAAATT	21	2	FGSG_13129	-

siRNA-t0202197	GTGCGCCGAATGAAAGCCCAGT	22	2	FgHV1gp2	-
siRNA-t0284565	GTGCGCTTCTGGATCAATGTCA	22	2	FgHV1gp2	-
siRNA-t0240998	GTGCTCATCGTTGTGCCAAC	20	2	FGSG_04332	-
siRNA-t0300859	GTGGAAATCTTCAGGCCCTG	20	2	FGSG_10199	-
siRNA-t0268178	GTGGACGGGTGTGAAGCTTGG	21	2	FGSG_00840	K00624|1|0.0|1728|fgr:FG00840.1|carnitine O-acetyltransferase [EC:2.3.1.7]
siRNA-t0279723	GTGGCGGGACCAGGATTCTTCTC	23	2	FGSG_08844	K01183|1|1e-06|55.1|pan:PODANSg4492|chitinase [EC:3.2.1.14]
siRNA-t0358201	GTGGCGGGACCAGGATTCTTCTCCA	25	2	FGSG_08844	K01183|1|1e-06|55.1|pan:PODANSg4492|chitinase [EC:3.2.1.14]
siRNA-t0357730	GTGGGTGCGCCGAATGAAAGCCC	23	2	FgHV1gp2	-
siRNA-t0205971	GTGGGTGCGCCGAATGAAAGCCCAG	25	2	FgHV1gp2	-
siRNA-t0247416	GTGGTTTTTGAACCCATGGCTCCA	24	2	FGSG_08608	K02486|1|4e-24|113|spo:SPAC27E2.09|two-component system, unclassified family, sensor kinase [EC:2.7.13.3];K00936|3|6e-19|95.9|afm:AFUA_6G10240|[EC:2.7.3.-]
siRNA-t0312758	GTGTAGGACGATCCACTCCTT	21	2	FGSG_12039	K00698|1|0.0|2441|nhe:NECHADRAFT_123231|chitin synthase [EC:2.4.1.16]
siRNA-t0250838	GTGTCAGGATCCGTCCAGGGAAG	23	2	FgHV1gp2	-
siRNA-t0248718	GTGTCCAATGTAAGTGACGAG	21	2	FGSG_07383	K08712|1|0.0|1020|pic:PICST_57529|ATP-binding cassette, subfamily G (WHITE), member 2, SNQ2;K08711|4|0.0|952|cne:CNB05770|ATP-binding cassette, subfamily G (WHITE), member 2, PDR
siRNA-t0286129	GTGTCGCGTCTACCTTGTTC	20	2	FGSG_13640	K07300|1|0.0|3608|fgr:FG09771.1|Ca2+:H+ antiporter
siRNA-t0350799	GTGTGATTGGTCTCTTGCAG	20	2	FgHV1gp2	-
siRNA-t0287219	GTGTGATTGGTCTCTTGCAGT	21	2	FgHV1gp2	-
siRNA-t0275133	GTGTGATTGGTCTCTTGCAGTG	22	2	FgHV1gp2	-
siRNA-t0314014	GTGTGGGTCTTCTGACGAATT	21	2	FgHV1gp2	-
siRNA-t0313296	GTGTGTATCCAATGAGTTGTG	21	2	FGSG_08373	K05658|1|1e-16|89.0|mgr:MGG_02348|ATP-binding cassette, subfamily B (MDR/TAP), member 1
siRNA-t0291049	GTGTGTATCCAATGAGTTGTGG	22	2	FgHV1gp2	-
siRNA-t0281524	GTGTTCGCATCCGTTCTGTGA	21	2	FGSG_06421	-
siRNA-t0332540	GTTACAATCGTGGACTTCCTTTA	23	2	FgHV1gp2	-
siRNA-t0222721	GTTAGAAGCTTGACACGAATGA	22	2	FgHV1gp2	-
siRNA-t0210488	GTTATAAGGCTTGTCGTCGGG	21	2	FGSG_08614	K01874|1|0.0|1256|fgr:FG08614.1|methionyl-tRNA synthetase [EC:6.1.1.10]
siRNA-t0267810	GTTCAAACCTCAGGTACCTTT	21	2	FGSG_08823	K05658|1|0.0|2440|fgr:FG08823.1|ATP-binding cassette, subfamily B (MDR/TAP), member 1
siRNA-t0252586	GTTCATCGATGCTACGCACCT	21	2	FGSG_01598	-
siRNA-t0273541	GTTCCCGAAAACAACACGCTT	21	2	FgHV1gp2	-
siRNA-t0204429	GTTCCGCGTGTGATTGGTCT	20	2	FGSG_09306	K11644|1|0.0|2881|fgr:FG09306.1|paired amphipathic helix protein Sin3a
siRNA-t0225598	GTTCCTGCTGAGTGTGGTTT	20	2	FGSG_02288	K11701|1|8e-53|209|spo:SPCC1739.03|helicase required for RNAi-mediated heterochromatin assembly 1 [EC:3.6.4.13]
siRNA-t0265171	GTTCGACACTGGAAGAGAAT	20	2	FGSG_05503	K03785|1|1e-17|91.7|ani:AN4350.2|3-dehydroquinate dehydratase I [EC:4.2.1.10];K00472|2|1e-14|81.6|nhe:NECHADRAFT_71135|prolyl 4-hydroxylase [EC:1.14.11.2];K09241|3|3e-08|60.5|sce:YPL248C|transcriptional regulatory protein GAL4;K09247|4|1e-06|55.5|sce:YLR256W|heme activator protein 1;K09248|5|2e-06|54.3|kla:KLLA0E18129g|proline utilization trans-activator
siRNA-t0227804	GTTCTGAAACCATCAGGACCCGA	23	2	FgHV1gp2	-
siRNA-t0332254	GTTCTTCGTGTAGAAAGTCTTTTC	24	2	FgHV1gp2	-
siRNA-t0208694	GTTGACGTCACCCTTTGGAGA	21	2	FgHV1gp2	-
siRNA-t0244599	GTTGCCGAACGGTACATTGA	20	2	FGSG_06540	K01876|1|1e-10|64.3|fgr:FG04934.1|aspartyl-tRNA synthetase [EC:6.1.1.12]
siRNA-t0315530	GTTGCTTGATGGTTGGCACAAC	22	2	FgHV1gp2	-
siRNA-t0333556	GTTGGTAATTGACAAAGGCCTA	22	2	FgHV1gp2	-
siRNA-t0303458	TAAGTTACAGGCCTCATTGATT	22	2	FgHV1gp2	-
siRNA-t0299257	TAATAATTGTACATCATTCCAT	22	2	FgHV1gp2	-
siRNA-t0279923	TAATCATCATCGTAGTCATCGTCAG	25	2	FGSG_12871	-
siRNA-t0319422	TACAAACCGGAATGCACTTTA	21	2	FgHV1gp2	-
siRNA-t0243650	TACAAACTGCAGTGGCTTGT	20	2	FGSG_12573	K07511|1|5e-23|107|cne:CNI00240|enoyl-CoA hydratase [EC:4.2.1.17]
siRNA-t0318745	TACACGGAACCCATTGATCATTCT	24	2	FgHV1gp2	-
siRNA-t0282675	TACCAGAACCCAGCACCTTTCCCTA	25	2	FgHV1gp2	-
siRNA-t0309837	TACCAGGCCAACAAAGAGAA	20	2	FGSG_02677	K01536|1|0.0|1037|afm:AFUA_6G03690|Na+-exporting ATPase [EC:3.6.3.7];K01537|3|0.0|879|cal:CaO19.6070|Ca2+-transporting ATPase [EC:3.6.3.8]
siRNA-t0359007	TACCATACTTTCCGAAGGAACTCC	24	2	FgHV1gp2	-
siRNA-t0337031	TACCATACTTTCCGAAGGAACTCCA	25	2	FgHV1gp2	-
siRNA-t0224125	TACCCGTCGAAGACCAAAAATTTCT	25	2	FgHV1gp1	-
siRNA-t0225768	TACGGATCCACATGAGCTTCA	21	2	FGSG_02857	-
siRNA-t0253007	TACGTAAGGCGTCGACACGATGA	23	2	FgHV1gp2	-
siRNA-t0299811	TACGTGGAAATCTTCAGGCCCTGG	24	2	FgHV1gp2	-
siRNA-t0252512	TACTCAAATATCGTACCGCA	20	2	FgHV1gp1	-
siRNA-t0324154	TACTCCTGACCCTATCAATCCT	22	2	FGSG_13493	-
siRNA-t0328779	TACTGAATGCGAGGGGCATTG	21	2	FGSG_00634	K02991|1|2e-135|480|fgr:FG00634.1|small subunit ribosomal protein S6e
siRNA-t0229727	TACTTGCCGCGCGTCTCACTG	21	2	FGSG_06580	K11262|1|0.0|4510|fgr:FG06580.1|acetyl-CoA carboxylase / biotin carboxylase [EC:6.4.1.2 6.3.4.14]
siRNA-t0270799	TACTTGCCGCGCGTCTCACTGACTT	25	2	FgHV1gp2	-
siRNA-t0312980	TAGAATGTCGAGATACTCTTC	21	2	FgHV1gp2	-
siRNA-t0270918	TAGCCTCTCCCTTCGTGAACTGCTG	25	2	FgHV1gp2	-
siRNA-t0319484	TAGTCCCTCAAATGTCTCCTTCGGT	25	2	FgHV1gp2	-
siRNA-t0326183	TAGTGCCCGGCCGTTTGTGC	20	2	FgHV1gp2	-
siRNA-t0316761	TATAAGGCTTGTCGTCGGGAGA	22	2	FgHV1gp2	-
siRNA-t0285935	TATACTTGATCTCAGCACGCCG	22	2	FgHV1gp2	-
siRNA-t0327349	TATACTTGATCTCAGCACGCCGGT	24	2	FgHV1gp2	-
siRNA-t0329591	TATAGTCCCTCAAATGTCTCC	21	2	FgHV1gp2	-
siRNA-t0220222	TATATTCCAACTGCGACCCAT	21	2	FgHV1gp2	-
siRNA-t0285172	TATCCTTCGGTAATCACAGGGCA	23	2	FgHV1gp2	-
siRNA-t0297176	TATCTCGACATTCTATGGGACA	22	2	FGSG_03407	-
siRNA-t0349507	TATGAACCCTTCCACGATTGGCGTG	25	2	FgHV1gp2	-
siRNA-t0217993	TATTCGGTATTGTCGAGTCG	20	2	FgHV1gp2	-
siRNA-t0317031	TCAAAAGAATGCTGTCTAGG	20	2	FGSG_05929	-
siRNA-t0234982	TCAAACGCAGATTCCAACCT	20	2	FGSG_08139	-
siRNA-t0213815	TCAAATCAAGTTGGTATCCCTCCGT	25	2	FgHV1gp2	-
siRNA-t0277355	TCAACAGTGAGCAAGTTCGT	20	2	FGSG_05818	-
siRNA-t0235395	TCAAGTATCGTCACTTCTTCG	21	2	FgHV1gp2	-
siRNA-t0308818	TCAAGTATCGTCACTTCTTCGTAGT	25	2	FgHV1gp2	-
siRNA-t0204616	TCAATGATGGCACGTCGACAG	21	2	FgHV1gp2	-
siRNA-t0253832	TCAATGGCGACACCGACGGTAC	22	2	FGSG_05709	K13830|1|2e-68|259|lbc:LACBIDRAFT_233717|pentafunctional AROM polypeptide [EC:4.2.3.4 4.2.1.10 1.1.1.25 2.7.1.71 2.5.1.19]
siRNA-t0321790	TCATTCGTGTCAAGCTTCTA	20	2	FGSG_03980	K00777|1|4e-68|259|afm:AFUA_7G06290|[EC:2.4.2.-]
siRNA-t0215286	TCATTGTTCATCTGCACTCCT	21	2	FGSG_12863	K02324|1|0.0|3986|fgr:FG06065.1|DNA polymerase epsilon subunit 1 [EC:2.7.7.7]
siRNA-t0247514	TCATTTCGGCGAGTCTAATCTT	22	2	FgHV1gp2	-
siRNA-t0231059	TCCAACTGCGACCCATGCTTT	21	2	FgHV1gp2	-
siRNA-t0277659	TCCAACTGCGACCCATGCTTTCGAC	25	2	FgHV1gp2	-
siRNA-t0290606	TCCAATAATCGTTGAAAATCAG	22	2	FgHV1gp2	-
siRNA-t0281896	TCCACCTCGTATTTTGAACTCACGA	25	2	FgHV1gp2	-
siRNA-t0360972	TCCAGTAGCATGTTCTTCGT	20	2	FgHV1gp2	-
siRNA-t0279159	TCCAGTAGCATGTTCTTCGTG	21	2	FgHV1gp2	-
siRNA-t0345023	TCCAGTATGGTTCGACACTG	20	2	FGSG_05213	-
siRNA-t0223073	TCCAGTGTCGAACCATACTGGAC	23	2	FgHV1gp2	-

siRNA-t0218393	TCCATAAACGCTGCCGGGAT	20	2	FGSG_09930	K14326|1|0.0|2138|fgr:FG09930.1|regulator of nonsense transcripts 1 [EC:3.6.4.-]
siRNA-t0253751	TCCATAAACGCTGCCGGGATG	21	2	FgHV1gp2	-
siRNA-t0206118	TCCATTCCAGCCTAGACAGCA	21	2	FGSG_03882	K08711|1|0.0|1456|cne:CNB05770|ATP-binding cassette, subfamily G (WHITE), member 2, PDR;K08712|3|0.0|1276|pic:PICST_57529|ATP-binding cassette, subfamily G (WHITE), member 2, SNQ2
siRNA-t0290960	TCCCACTCTAGCAATGCATCA	21	2	FgHV1gp1	-
siRNA-t0361736	TCCCATAGTGCCCGGCCGTTTGTGC	25	2	FgHV1gp2	-
siRNA-t0217523	TCCCATCAATGGCGACACCGA	21	2	FGSG_07053	-
siRNA-t0262246	TCCCGGCAGCGTTTATGGAA	20	2	FGSG_12361	-
siRNA-t0327327	TCCCTCAAATGTCTCCTTCGGT	22	2	FgHV1gp2	-
siRNA-t0289232	TCCGAATATGTCAGAGATCCTGC	23	2	FGSG_09665	-
siRNA-t0255579	TCCGCAGAAGAATTAGCCTTTGAA	24	2	FgHV1gp2	-
siRNA-t0299552	TCCGCAGAAGAATTAGCCTTTGAAT	25	2	FgHV1gp2	-
siRNA-t0258140	TCCGCGTGTGATTGGTCTCTT	21	2	FGSG_09394	-
siRNA-t0247727	TCCGGAATGCGTTCACACGATT	22	2	FgHV1gp2	-
siRNA-t0339461	TCCGGATTATGCCCAAAAGGAT	22	2	FgHV1gp2	-
siRNA-t0228055	TCCTATAGATCGCGCGTAAG	20	2	FgHV1gp2	-
siRNA-t0356025	TCCTTCCACTTCCCTGGACGG	21	2	FGSG_08097	K00688|1|7e-16|87.0|pno:SNOG_13460|starch phosphorylase [EC:2.4.1.1]
siRNA-t0299199	TCCTTTCCTACGACACTTTC	20	2	FGSG_01388	K05681|1|0.0|1551|mgr:MGG_01563|ATP-binding cassette, subfamily G (WHITE), member 2;K08711|2|7e-43|175|cne:CNB05770|ATP-binding cassette, subfamily G (WHITE), member 2, PDR
siRNA-t0269547	TCGAACCATACTGGACCCAGA	21	2	FgHV1gp2	-
siRNA-t0270431	TCGACAATGAAAGATCCTAA	20	2	FgHV1gp2	-
siRNA-t0356105	TCGACACGATGATACCCTCT	20	2	FGSG_06655	K01267|1|0.0|969|fgr:FG06655.1|aspartyl aminopeptidase [EC:3.4.11.21]
siRNA-t0217806	TCTGGCTCGTGACTTTTCCCTC	22	2	FgHV1gp2	-
siRNA-t0322476	TCTGTTGCCGAACGGTACAT	20	2	FGSG_06540	K01876|1|1e-10|64.3|fgr:FG04934.1|aspartyl-tRNA synthetase [EC:6.1.1.12]
siRNA-t0254575	TCTTCCAAGCGGTCATAAGTGAC	23	2	FgHV1gp2	-
siRNA-t0241191	TCTTCCAGGTTCATCGATGCTA	22	2	FGSG_05080	K01133|1|2e-07|57.4|afv:AFLA_033500|choline-sulfatase [EC:3.1.6.6];K01565|3|4e-07|56.6|ang:ANI_1_1460064|N-sulfoglucosamine sulfohydrolase [EC:3.10.1.1]
siRNA-t0242035	TCTTCCAGTGTCGAACCATA	20	2	FgHV1gp2	-
siRNA-t0215511	TCTTCGTCCAGTAGCCAATGAG	22	2	FGSG_06448	-
siRNA-t0292448	TCTTCGTGTAGAAAGTCTTTT	21	2	FgHV1gp2	-
siRNA-t0203451	TCTTCGTGTAGAAAGTCTTTTCA	23	2	FgHV1gp2	-
siRNA-t0251365	TCTTCTCCACGTCATGATCAT	21	2	FGSG_01910	K06636|1|0.0|2339|fgr:FG01910.1|structural maintenance of chromosome 1
siRNA-t0208451	TCTTGTTCTGGAACGTCAAA	20	2	FGSG_13660	-
siRNA-t0310315	TGAACCACTTGCCCAGGCCT	20	2	FGSG_13385	K00100|1|1e-42|172|afm:AFUA_2G01410|[EC:1.1.1.-]
siRNA-t0204236	TGAGCTCCTTGAGGAATGCTT	21	2	FGSG_09241	K10590|1|0.0|3046|fgr:FG09241.1|E3 ubiquitin-protein ligase TRIP12 [EC:6.3.2.19]
siRNA-t0257384	TGAGCTCTGTGACTCCAGCCAA	22	2	FgHV1gp2	-
siRNA-t0220410	TGAGCTCTGTGACTCCAGCCAAA	23	2	FgHV1gp2	-
siRNA-t0211387	TGAGGCCAAAGGTGTTCAGCT	21	2	FGSG_08098	K01066|1|2e-53|208|afm:AFUA_4G07720|esterase / lipase [EC:3.1.1.-];K01046|2|3e-30|132|afm:AFUA_8G02530|triacylglycerol lipase [EC:3.1.1.3]
siRNA-t0351682	TGAGTGCCATGACGTACGACCCCG	24	2	FgHV1gp1	-
siRNA-t0293708	TGATAGATCTTATTGCCAGGTAGAT	25	2	FgHV1gp2	-
siRNA-t0269153	TGATCCATGCCAAACCAACTT	21	2	FgHV1gp2	-
siRNA-t0277890	TGATCCTAAACATGTCGGTG	20	2	FGSG_11659	K01932|1|0.0|1431|afm:AFUA_5G12730|[EC:6.3.2.-]
siRNA-t0310043	TGATCTGGCTCGTGACTTTT	20	2	FGSG_09838	K12614|1|5e-143|508|ani:AN3460.2|ATP-dependent RNA helicase DDX6/DHH1 [EC:3.6.4.13];K01553|2|2e-86|320|pic:PICST_31021|myosin ATPase [EC:3.6.4.1];K06675|5|1e-05|52.4|tml:GSTUM_00006213001|structural maintenance of chromosome 4
siRNA-t0290095	TGATGAGTAAGCGGGCTTGC	20	2	FGSG_03736	K14859|1|6e-27|122|scm:SCHCODRAFT_255991|ribosome biogenesis protein SSF1/2
siRNA-t0356510	TGATGGTACCATTGCTGCATG	21	2	FgHV1gp2	-
siRNA-t0285560	TGATTTCATGACCTTGAGACAG	22	2	FGSG_07175	K02327|1|0.0|2272|fgr:FG07175.1|DNA polymerase delta subunit 1 [EC:2.7.7.7]
siRNA-t0237113	TGCAACGAGGGCCCAGTCATG	21	2	FgHV1gp2	-
siRNA-t0205955	TGCAGCGGCAAATACAGGACCGCGC	25	2	FgHV1gp2	-
siRNA-t0359451	TGCCGAACAGTACATGCAGC	20	2	FGSG_06900	K03639|1|0.0|1291|fgr:FG06900.1|molybdenum cofactor biosynthesis protein
siRNA-t0360440	TGCCGACGCTCCACTCGCTCTTCT	24	2	FGSG_09965	K14300|1|0.0|2662|fgr:FG09965.1|nuclear pore complex protein Nup133
siRNA-t0247734	TGCCGCGCGTCTCACTGACT	20	2	FgHV1gp2	-
siRNA-t0302064	TGCCGCGCGTCTCACTGACTT	21	2	FgHV1gp2	-
siRNA-t0332892	TGCCTGGGAGACAGAGCCTGAGTC	24	2	FgHV1gp2	-
siRNA-t0242601	TGCGCACATATCTCCTTGAAC	21	2	FgHV1gp2	-
siRNA-t0271482	TGCGCTTCTGGATCAATGTC	20	2	FGSG_08541	K15134|1|0.0|1199|fgr:FG08541.1|mediator of RNA polymerase II transcription subunit 17, fungi type
siRNA-t0277668	TGCGCTTCTGGATCAATGTCAG	22	2	FgHV1gp2	-
siRNA-t0286538	TGCGTACCTGCAGTCACAACT	21	2	FGSG_01790	K00680|1|0.0|2387|afm:AFUA_3G14700|[EC:2.3.1.-];K15419|2|0.0|1077|ani:AN2547.2|emericellamide biosynthesis, highly reducing iterative type I polyketide synthase;K15384|5|0.0|762|afv:AFLA_066840|aspyridone synthetase, hybrid polyketide synthase / nonribosomal peptide synthetase
siRNA-t0272861	TGCTCAAGTTCAAGGAGATA	20	2	FGSG_06193	K08773|1|2e-20|101|cci:CC1G_05669|RalA-binding protein 1
siRNA-t0308543	TGCTGCCGCGGATAACCTAT	20	2	FGSG_09049	K00652|1|0.0|782|fgr:FG09049.1|8-amino-7-oxononanoate synthase [EC:2.3.1.47]
siRNA-t0302455	TGCTTTCGACGTACTTGGGTTCAT	24	2	FgHV1gp2	-
siRNA-t0244445	TGGAAATCTTCAGGCCCTGGGATTG	25	2	FgHV1gp2	-
siRNA-t0310089	TGGAAGTGTTCGCATCCGTTC	21	2	FGSG_10136	-
siRNA-t0356070	TGGAGTGGCATATTCAAGAAT	21	2	FgHV1gp2	-
siRNA-t0233761	TGGAGTGGCATATTCAAGAATC	22	2	FgHV1gp2	-
siRNA-t0275833	TGGATCTGGGGTCTGACCGAG	21	2	FGSG_13315	-
siRNA-t0312834	TGGCCAAATCGTCATACATTG	21	2	FGSG_07453	K01528|1|0.0|668|uma:UM02907.1|dynamin GTPase [EC:3.6.5.5]
siRNA-t0365877	TGGCCAAATCGTCATACATTGC	22	2	FGSG_07453	K01528|1|0.0|668|uma:UM02907.1|dynamin GTPase [EC:3.6.5.5]
siRNA-t0357335	TGGCCGGCAGTCCTAGCAAGT	21	2	FgHV1gp2	-
siRNA-t0280899	TGGCCGGTTGAAAATGTTCCACA	23	2	FgHV1gp2	-
siRNA-t0324402	TGGCCGGTTGAAAATGTTCCACAG	24	2	FgHV1gp2	-
siRNA-t0301993	TGGCGAGACCGATGATTTCC	20	2	FGSG_12863	K02324|1|0.0|3986|fgr:FG06065.1|DNA polymerase epsilon subunit 1 [EC:2.7.7.7]
siRNA-t0292762	TGGCGAGACCGATGATTTCCATCCC	25	2	FgHV1gp2	-
siRNA-t0297799	TGGCGCATTTCTTTCTTACCCTC	23	2	FgHV1gp2	-
siRNA-t0347703	TGGGAGGCCTATTCATCGTCAA	22	2	FgHV1gp2	-
siRNA-t0349211	TGGGATGCAAAACCGGTGCG	20	2	FgHV1gp2	-
siRNA-t0247016	TGGGATGCAAAACCGGTGCGATA	23	2	FgHV1gp2	-
siRNA-t0288397	TGGGATTGGGCGTGTGTTCTTTAT	24	2	FgHV1gp2	-
siRNA-t0311044	TGGGGTCACATATCTCGATT	20	2	FGSG_01184	K12585|1|0.0|1961|fgr:FG01184.1|exosome complex exonuclease DIS3/RRP44 [EC:3.1.13.-]
siRNA-t0274076	TGGGGTCAGCCATTTCGAATCTG	23	2	FgHV1gp2	-
siRNA-t0218771	TGGGTCTTCTGACGAATTTGGA	22	2	FgHV1gp2	-
siRNA-t0318508	TGGGTGCGCCGAATGAAAGCCCAG	24	2	FgHV1gp2	-
siRNA-t0244813	TGGGTTTCGCAGCATCCATC	20	2	FGSG_11610	-
siRNA-t0282569	TGGTATCCCATAGTGCCCGG	20	2	FGSG_04074	-
siRNA-t0246070	TGGTATCCCATAGTGCCCGGCC	22	2	FgHV1gp2	-
siRNA-t0343140	TGGTCCTAAGGGTGTGGCTT	20	2	FGSG_11051	K09241|1|2e-09|63.5|aor:AOR_1_568084|transcriptional regulatory protein GAL4

<table>
<tr><td></td><td></td><td></td><td></td><td></td><td></td></tr>
<tr><td>siRNA-t0282830</td><td>TGGTCTTCCAGGCATCAGGGGTC</td><td>23</td><td>2</td><td>FGSG_08601</td><td>K01610|1|0.0|1128|fgr:FG08601.1|phosphoenolpyruvate carboxykinase (ATP) [EC:4.1.1.49]</td></tr>
<tr><td>siRNA-t0207092</td><td>TGGTTCGAGTTGTATGAAAA</td><td>20</td><td>2</td><td>FGSG_04089</td><td>-</td></tr>
<tr><td>siRNA-t0312709</td><td>TGGTTGCAGCGTGAACCAGATCAT</td><td>24</td><td>2</td><td>FgHV1gp2</td><td>-</td></tr>
<tr><td>siRNA-t0328711</td><td>TGGTTTGAATGGTCTGCCTAATCTT</td><td>25</td><td>2</td><td>FgHV1gp2</td><td>-</td></tr>
<tr><td>siRNA-t0282591</td><td>TGTAAGTGACGAGGCCTCTGAC</td><td>22</td><td>2</td><td>FgHV1gp2</td><td>-</td></tr>
<tr><td>siRNA-t0283787</td><td>TGTACTTGCCGCGCGTCTCACTG</td><td>23</td><td>2</td><td>FgHV1gp2</td><td>-</td></tr>
<tr><td>siRNA-t0220795</td><td>TGTAGATCTTGCTGTAGTGTC</td><td>21</td><td>2</td><td>FgHV1gp2</td><td>-</td></tr>
<tr><td>siRNA-t0237158</td><td>TGTCCAAATTCGTCAGAAGA</td><td>20</td><td>2</td><td>FGSG_09793</td><td>-</td></tr>
<tr><td>siRNA-t0360911</td><td>TGTCGAGATACTCTTCAGCGTA</td><td>22</td><td>2</td><td>FgHV1gp2</td><td>-</td></tr>
<tr><td>siRNA-t0250835</td><td>TGTGAAGCTTGGATGAGCGAT</td><td>21</td><td>2</td><td>FGSG_00324</td><td>-</td></tr>
<tr><td>siRNA-t0346526</td><td>TGTGGCGGGACCAGGATTCTTC</td><td>22</td><td>2</td><td>FGSG_08844</td><td>K01183|1|1e-06|55.1|pan:PODANSg4492|chitinase [EC:3.2.1.14]</td></tr>
<tr><td>siRNA-t0252525</td><td>TGTTCATCTGCACTCCTTTCATCA</td><td>24</td><td>2</td><td>FgHV1gp2</td><td>-</td></tr>
<tr><td>siRNA-t0341373</td><td>TGTTCCATAAACGCTGCCGGGATG</td><td>24</td><td>2</td><td>FgHV1gp2</td><td>-</td></tr>
<tr><td>siRNA-t0282092</td><td>TGTTGACGTCACCCTTTGGAGA</td><td>22</td><td>2</td><td>FgHV1gp2</td><td>-</td></tr>
<tr><td>siRNA-t0338921</td><td>TTAATCAATGTACCGTTCGGC</td><td>21</td><td>2</td><td>FgHV1gp2</td><td>-</td></tr>
<tr><td>siRNA-t0261108</td><td>TTACAGGCCTCATTGATTTT</td><td>20</td><td>2</td><td>FGSG_02786</td><td>K05658|1|0.0|2468|fgr:FG02786.1|ATP-binding cassette, subfamily B (MDR/TAP), member 1</td></tr>
<tr><td>siRNA-t0245101</td><td>TTACAGGCCTCATTGATTTTG</td><td>21</td><td>2</td><td>FGSG_02786</td><td>K05658|1|0.0|2468|fgr:FG02786.1|ATP-binding cassette, subfamily B (MDR/TAP), member 1</td></tr>
<tr><td>siRNA-t0265898</td><td>TTACAGGCCTCATTGATTTTGT</td><td>22</td><td>2</td><td>FgHV1gp2</td><td>-</td></tr>
<tr><td>siRNA-t0216336</td><td>TTACCCTCGCATTTGCCGCAC</td><td>21</td><td>2</td><td>FgHV1gp2</td><td>-</td></tr>
<tr><td>siRNA-t0277532</td><td>TTATCCGTGATAGCCTCTCCCT</td><td>22</td><td>2</td><td>FGSG_12196</td><td>-</td></tr>
<tr><td>siRNA-t0351951</td><td>TTATGATCTGGTTCACGCTG</td><td>20</td><td>2</td><td>FGSG_13338</td><td>-</td></tr>
<tr><td>siRNA-t0282363</td><td>TTATTGTAAGTCTGGAATTCC</td><td>21</td><td>2</td><td>FgHV1gp2</td><td>-</td></tr>
<tr><td>siRNA-t0297438</td><td>TTCAAGTATCGTCACTTCTTC</td><td>21</td><td>2</td><td>FgHV1gp2</td><td>-</td></tr>
<tr><td>siRNA-t0224933</td><td>TTCGTGAACTGCTGCGCTATGGTG</td><td>24</td><td>2</td><td>FgHV1gp2</td><td>-</td></tr>
<tr><td>siRNA-t0229358</td><td>TTCTAGCCTGGTTGAGATCC</td><td>20</td><td>2</td><td>FGSG_07798</td><td>K15384|1|0.0|2498|afv:AFLA_066840|aspyridone synthetase, hybrid polyketide synthase / nonribosomal peptide synthetase;K15394|3|0.0|2447|cpw:CPC735_030880|hybrid polyketide synthase / nonribosomal peptide synthetase ACE1</td></tr>
<tr><td>siRNA-t0266486</td><td>TTCTCTTTGTTGGCCTGGTAA</td><td>21</td><td>2</td><td>FGSG_04785</td><td>-</td></tr>
<tr><td>siRNA-t0255640</td><td>TTCTGCCATAACCTCGTTGG</td><td>20</td><td>2</td><td>FGSG_05163</td><td>-</td></tr>
<tr><td>siRNA-t0221653</td><td>TTCTGGAACGTCAAATCAAG</td><td>20</td><td>2</td><td>FGSG_10558</td><td>K02320|1|2e-32|140|aor:AOR_1_946054|DNA polymerase alpha subunit A [EC:2.7.7.7]</td></tr>
<tr><td>siRNA-t0255716</td><td>TTCTGTGAACACTAATATCGTCAT</td><td>24</td><td>2</td><td>FgHV1gp2</td><td>-</td></tr>
<tr><td>siRNA-t0236952</td><td>TTCTTCTGCGGAGATCTCTA</td><td>20</td><td>2</td><td>FGSG_06912</td><td>K11835|1|0.0|3046|fgr:FG06912.1|ubiquitin carboxyl-terminal hydrolase 4/11/15 [EC:3.1.2.15]</td></tr>
<tr><td>siRNA-t0271287</td><td>TTCTTTCTTACCCTCGATGT</td><td>20</td><td>2</td><td>FGSG_05542</td><td>K14311|1|0.0|3550|fgr:FG05542.1|nuclear pore complex protein Nup188</td></tr>
<tr><td>siRNA-t0260070</td><td>TTGAACTTGAGCATTTGTTTG</td><td>21</td><td>2</td><td>FGSG_00270</td><td>K14548|1|0.0|1696|fgr:FG00270.1|U3 small nucleolar RNA-associated protein 4</td></tr>
<tr><td>siRNA-t0245068</td><td>TTGAGTGCCATGACGTACGACCCCG</td><td>25</td><td>2</td><td>FgHV1gp1</td><td>-</td></tr>
<tr><td>siRNA-t0245984</td><td>TTGATCTCAGCACGCCGGTTA</td><td>21</td><td>2</td><td>FGSG_00633</td><td>K10592|1|0.0|5185|fgr:FG00633.1|E3 ubiquitin-protein ligase HUWE1 [EC:6.3.2.19]</td></tr>
<tr><td>siRNA-t0226252</td><td>TTGCCGAACAGTACATGCAG</td><td>20</td><td>2</td><td>FGSG_01049</td><td>-</td></tr>
<tr><td>siRNA-t0235570</td><td>TTGCGCACATATCTCCTTGAA</td><td>21</td><td>2</td><td>FgHV1gp2</td><td>-</td></tr>
<tr><td>siRNA-t0344950</td><td>TTGCTGCATGTACTGTTCGGCAAC</td><td>24</td><td>2</td><td>FgHV1gp2</td><td>-</td></tr>
<tr><td>siRNA-t0225618</td><td>TTGGCCAAATCGTCATACATTG</td><td>22</td><td>2</td><td>FgHV1gp2</td><td>-</td></tr>
<tr><td>siRNA-t0310242</td><td>TTGGCCCCAGCAGACTTGGAAG</td><td>22</td><td>2</td><td>FgHV1gp2</td><td>-</td></tr>
<tr><td>siRNA-t0358667</td><td>TTGGCCCCTTTCTGTCTGGT</td><td>20</td><td>2</td><td>FGSG_03825</td><td>-</td></tr>
<tr><td>siRNA-t0209881</td><td>TTGGTGCCGACGTCTACGTAA</td><td>21</td><td>2</td><td>FGSG_08913</td><td>-</td></tr>
<tr><td>siRNA-t0238551</td><td>TTGGTTCAAGTATCGTCACT</td><td>20</td><td>2</td><td>FgHV1gp2</td><td>-</td></tr>
<tr><td>siRNA-t0211007</td><td>TTGGTTCAAGTATCGTCACTTCTT</td><td>24</td><td>2</td><td>FgHV1gp2</td><td>-</td></tr>
<tr><td>siRNA-t0258059</td><td>TTGTACTTGCCGCGCGTCTCACT</td><td>23</td><td>2</td><td>FgHV1gp2</td><td>-</td></tr>
<tr><td>siRNA-t0333078</td><td>TTGTAGAACTTTTGCCACTGGGATT</td><td>25</td><td>2</td><td>FgHV1gp2</td><td>-</td></tr>
<tr><td>siRNA-t0315263</td><td>TTGTAGTAAAGTTATCGTGTCTGA</td><td>24</td><td>2</td><td>FgHV1gp2</td><td>-</td></tr>
<tr><td>siRNA-t0297618</td><td>TTGTCAGTGTCAGGATCCGT</td><td>20</td><td>2</td><td>FGSG_09917</td><td>K01115|1|0.0|3466|fgr:FG09917.1|phospholipase D [EC:3.1.4.4]</td></tr>
<tr><td>siRNA-t0243192</td><td>TTGTTCATCAAGTTAGACAA</td><td>20</td><td>2</td><td>FGSG_01544</td><td>K06653|1|0.0|2065|fgr:FG01544.1|CDK inhibitor PHO81</td></tr>
<tr><td>siRNA-t0359928</td><td>TTGTTCCATAAACGCTGCCGGGA</td><td>23</td><td>2</td><td>FgHV1gp2</td><td>-</td></tr>
<tr><td>siRNA-t0657590</td><td>AAAAATGACTCAGGCTCTGTC</td><td>21</td><td>1</td><td>FGSG_01964</td><td>K00698|1|0.0|3795|fgr:FG01964.1|chitin synthase [EC:2.4.1.16]</td></tr>
<tr><td>siRNA-t0617685</td><td>AAAACGCGCCTCCAGTCCAGCC</td><td>22</td><td>1</td><td>FgHV1gp2</td><td>-</td></tr>
<tr><td>siRNA-t0421438</td><td>AAAACGCGCCTCCAGTCCAGCCTTT</td><td>25</td><td>1</td><td>FgHV1gp2</td><td>-</td></tr>
<tr><td>siRNA-t0850905</td><td>AAAACTTCTGTCCAGTTGAGA</td><td>21</td><td>1</td><td>FGSG_09379</td><td>-</td></tr>
<tr><td>siRNA-t0893430</td><td>AAAAGCATGGCGATGCACTTTAT</td><td>23</td><td>1</td><td>FgHV1gp2</td><td>-</td></tr>
<tr><td>siRNA-t0656130</td><td>AAAATTGTGTTGATTGCGCCA</td><td>21</td><td>1</td><td>FGSG_01976</td><td>K01876|1|0.0|1969|fgr:FG01976.1|aspartyl-tRNA synthetase [EC:6.1.1.12]</td></tr>
<tr><td>siRNA-t0641579</td><td>AAACAACACGCTTCCGGCTA</td><td>20</td><td>1</td><td>FgHV1gp2</td><td>-</td></tr>
<tr><td>siRNA-t0671250</td><td>AAACATATCCGGCTCCCTTTC</td><td>21</td><td>1</td><td>FGSG_06595</td><td>-</td></tr>
<tr><td>siRNA-t0910805</td><td>AAACATATTGAACCACAGTC</td><td>20</td><td>1</td><td>FgHV1gp2</td><td>-</td></tr>
<tr><td>siRNA-t0603470</td><td>AAACATATTGAACCACAGTCGA</td><td>22</td><td>1</td><td>FgHV1gp2</td><td>-</td></tr>
<tr><td>siRNA-t1022547</td><td>AAACATATTGAACCACAGTCGAA</td><td>23</td><td>1</td><td>FgHV1gp2</td><td>-</td></tr>
<tr><td>siRNA-t1072360</td><td>AAACATTTTCGGATGTAATG</td><td>20</td><td>1</td><td>FgHV1gp2</td><td>-</td></tr>
<tr><td>siRNA-t0770707</td><td>AAACCATCAGGACCCGAGAAAAA</td><td>23</td><td>1</td><td>FgHV1gp2</td><td>-</td></tr>
<tr><td>siRNA-t0574143</td><td>AAACCCATCCCACTCGTGAA</td><td>20</td><td>1</td><td>FGSG_07946</td><td>K00706|1|0.0|3025|pan:PODANSg1678|1,3-beta-glucan synthase [EC:2.4.1.34]</td></tr>
<tr><td>siRNA-t1255457</td><td>AAACCCATCCCACTCGTGAAC</td><td>21</td><td>1</td><td>FgHV1gp2</td><td>-</td></tr>
<tr><td>siRNA-t1300020</td><td>AAACGCGCCTCCAGTCCAGCC</td><td>21</td><td>1</td><td>FgHV1gp2</td><td>-</td></tr>
<tr><td>siRNA-t0565566</td><td>AAACGCGCCTCCAGTCCAGCCT</td><td>22</td><td>1</td><td>FgHV1gp2</td><td>-</td></tr>
<tr><td>siRNA-t1140553</td><td>AAACGCGCTAAGTTACAGGCCT</td><td>22</td><td>1</td><td>FgHV1gp2</td><td>-</td></tr>
<tr><td>siRNA-t0911809</td><td>AAACGCTCATGATAACATTGGA</td><td>22</td><td>1</td><td>FgHV1gp2</td><td>-</td></tr>
<tr><td>siRNA-t1126986</td><td>AAACGCTCATGATAACATTGGAT</td><td>23</td><td>1</td><td>FgHV1gp2</td><td>-</td></tr>
<tr><td>siRNA-t0986781</td><td>AAACTGCAGTGGCTTGTGGTT</td><td>21</td><td>1</td><td>FGSG_11181</td><td>K05349|1|7e-149|527|pno:SNOG_04685|beta-glucosidase [EC:3.2.1.21]</td></tr>
<tr><td>siRNA-t0882715</td><td>AAACTTCTGTCCAGTTGAGACA</td><td>22</td><td>1</td><td>FGSG_09379</td><td>-</td></tr>
<tr><td>siRNA-t0754044</td><td>AAAGAACCCATATGGATCGGTG</td><td>22</td><td>1</td><td>FgHV1gp2</td><td>-</td></tr>
<tr><td>siRNA-t1099275</td><td>AAAGACCACATCCAGATCTTA</td><td>21</td><td>1</td><td>FgHV1gp2</td><td>-</td></tr>
<tr><td>siRNA-t0578320</td><td>AAAGGGTGACGTCAACAGTGA</td><td>21</td><td>1</td><td>FGSG_07884</td><td>K09246|1|8e-08|58.2|lth:KLTH0E16500g|arginine metabolism regulation protein II</td></tr>
<tr><td>siRNA-t0649525</td><td>AAAGGTGCTGGGTTCTGGTA</td><td>20</td><td>1</td><td>FGSG_11822</td><td>K01887|1|5e-114|410|mgr:MGG_09080|arginyl-tRNA synthetase [EC:6.1.1.19]</td></tr>
<tr><td>siRNA-t1415874</td><td>AAAGTCTCACTCTTCAACGT</td><td>20</td><td>1</td><td>FGSG_09135</td><td>K14848|1|6e-08|59.7|nhe:NECHADRAFT_100888|ribosome assembly protein RRB1;K10752|2|7e-08|59.7|pan:PODANSg8997|histone-binding protein RBBP4;K14549|3|1e-07|58.9|cci:CC1G_00329|U3 small nucleolar RNA-associated protein 15;K14005|4|1e-07|58.9|nhe:NECHADRAFT_68119|protein transport protein SEC31</td></tr>
<tr><td>siRNA-t1023332</td><td>AAAGTTGGAGCCCTTCTTAAG</td><td>21</td><td>1</td><td>FgHV1gp2</td><td>-</td></tr>
<tr><td>siRNA-t0912309</td><td>AAATAACACCACATCACGCTCGG</td><td>23</td><td>1</td><td>FgHV1gp2</td><td>-</td></tr>
<tr><td>siRNA-t1315958</td><td>AAATCAATGAGGCCTGTAAC</td><td>20</td><td>1</td><td>FGSG_12450</td><td>-</td></tr>
<tr><td>siRNA-t0582242</td><td>AAATCATCGGTCTCGCCAAA</td><td>20</td><td>1</td><td>FGSG_00669</td><td>K05658|1|4e-39|163|yli:YALI0F20174g|ATP-binding cassette, subfamily B (MDR/TAP), member 1;K05663|5|5e-39|163|cpw:CPC735_047650|mitochondrial ABC transporter ATM</td></tr>
<tr><td>siRNA-t0477096</td><td>AAATCCAAAAGACGCGTGTCG</td><td>21</td><td>1</td><td>FgHV1gp2</td><td>-</td></tr>
<tr><td>siRNA-t1409745</td><td>AAATCCTGGGCGCTAACAAATAG</td><td>23</td><td>1</td><td>FgHV1gp2</td><td>-</td></tr>
<tr><td>siRNA-t1199330</td><td>AAATCCTGGGCGCTAACAAATAGTT</td><td>25</td><td>1</td><td>FgHV1gp2</td><td>-</td></tr>
<tr><td>siRNA-t0923155</td><td>AAATCGTCATACATTGCACG</td><td>20</td><td>1</td><td>FgHV1gp2</td><td>-</td></tr>
<tr><td>siRNA-t1029808</td><td>AAATCTTCAGGCCCTGGGATTGT</td><td>23</td><td>1</td><td>FgHV1gp2</td><td>-</td></tr>
<tr><td>siRNA-t1334547</td><td>AAATTCAACCTGACCTGTTCCAA</td><td>23</td><td>1</td><td>FgHV1gp2</td><td>-</td></tr>
<tr><td>siRNA-t0603044</td><td>AAATTTGGCCAAATCGTCATACATT</td><td>25</td><td>1</td><td>FgHV1gp2</td><td>-</td></tr>
<tr><td>siRNA-t0806404</td><td>AACAAATGCTCAAGTTCAAGG</td><td>21</td><td>1</td><td>FGSG_06193</td><td>K08773|1|2e-20|101|cci:CC1G_05669|RalA-binding protein 1</td></tr>
<tr><td>siRNA-t0608901</td><td>AACAACACGCTTCCGGCTATG</td><td>21</td><td>1</td><td>FgHV1gp2</td><td>-</td></tr>
</table>

siRNA-t1057167	AACACTAATATCGTCATTCTCTTC	24	1	FgHV1gp2	-
siRNA-t1113034	AACAGACGTCTCTCACGCTC	20	1	FgHV1gp2	-
siRNA-t0717759	AACAGGAATGACGTTTGTAAA	21	1	FgHV1gp2	-
siRNA-t0495890	AACAGGTTGGAATCTGCGTTTG	22	1	FgHV1gp2	-
siRNA-t0649224	AACATATTGAACCACAGTCGAAT	23	1	FgHV1gp2	-
siRNA-t0505186	AACATCCCCAGCTCTGTTAA	20	1	FGSG_02078	-
siRNA-t0735592	AACCACAACGATAGTATGAACC	22	1	FgHV1gp2	-
siRNA-t0922144	AACCACAAGCCACTGCAGTTT	21	1	FgHV1gp2	-
siRNA-t0487866	AACCACATTTTCCCGTTCCAC	21	1	FGSG_13700	-
siRNA-t1015338	AACCACATTTTCCCGTTCCACA	22	1	FGSG_06781	K03355|1|0.0|1239|fgr:FG06781.1|anaphase-promoting complex subunit 8
siRNA-t0774931	AACCAGATCATAACCCGCAT	20	1	FgHV1gp2	-
siRNA-t0649362	AACCATCAGGACCCGAGAAAA	21	1	FgHV1gp2	-
siRNA-t0442353	AAGTCCAAGCCACCAGTGGGA	21	1	FGSG_01433	K00264|1|0.0|4368|fgr:FG01433.1|glutamate synthase (NADPH/NADH) [EC:1.4.1.13 1.4.1.14]
siRNA-t0452426	AAGTCTCCGAACGTGATCTCATCA	24	1	FgHV1gp2	-
siRNA-t1231571	AATGTTTCACTCCTCCTTCCGTA	23	1	FgHV1gp2	-
siRNA-t0801734	AATTCCGGGATCAGGTTCAC	20	1	FGSG_08396	K01443|1|0.0|921|fgr:FG08396.1|N-acetylglucosamine-6-phosphate deacetylase [EC:3.5.1.25]
siRNA-t1346435	AATTCCGGGATCAGGTTCACAAT	23	1	FGSG_08396	K01443|1|0.0|921|fgr:FG08396.1|N-acetylglucosamine-6-phosphate deacetylase [EC:3.5.1.25]
siRNA-t0637465	AATTCGTGTATCATCTGTGCGACT	24	1	FgHV1gp2	-
siRNA-t0637111	AATTCTAGCCTGGTTGAGAT	20	1	FgHV1gp2	-
siRNA-t0857614	AATTCTAGCCTGGTTGAGATCCA	23	1	FgHV1gp2	-
siRNA-t0968114	AATTCTAGCCTGGTTGAGATCCAT	24	1	FgHV1gp2	-
siRNA-t1042271	AATTCTGCTGCGTGTTATGC	20	1	FGSG_09750	-
siRNA-t1129791	AATTGACCCCGAGCGTGATG	20	1	FGSG_11871	-
siRNA-t1295523	AATTGAGTGCCATGACGTACGACCC	25	1	FgHV1gp1	-
siRNA-t0419967	AATTGCAACGAGGGCCCAGT	20	1	FGSG_07277	K01976|1|3e-32|139|sce:YBR222C|[EC:6.-.-.-];K01895|4|2e-19|96.7|mgr:MGG_04590|acetyl-CoA synthetase [EC:6.2.1.1];K01897|5|2e-19|96.7|ncr:NCU06063|long-chain acyl-CoA synthetase [EC:6.2.1.3]
siRNA-t0934773	AATTGCAACGAGGGCCCAGTCATG	24	1	FgHV1gp2	-
siRNA-t1064281	AATTTCTCCAGTAGCATGTTC	21	1	FGSG_11448	K01843|1|7e-40|164|afv:AFLA_005130|lysine 2,3-aminomutase [EC:5.4.3.2];K06694|2|3e-12|72.8|cim:CIMG_08979|26S proteasome non-ATPase regulatory subunit 10;K06653|3|3e-12|72.8|ppa:PAS_c121_0015|CDK inhibitor PHO81;K06867|4|5e-12|72.4|spo:SPAC2F7.10|
siRNA-t1069049	ACAAAACTTCTGTCCAGTTGA	21	1	FGSG_12624	K00777|1|3e-30|133|afm:AFUA_7G07100|[EC:2.4.2.-];K14855|2|7e-15|82.4|lbc:LACBIDRAFT_296068|ribosome assembly protein 4;K06666|3|6e-10|66.2|cal:CaO19.13528|glucose repression regulatory protein TUP1
siRNA-t0941710	ACAAAACTTCTGTCCAGTTGAG	22	1	FGSG_12624	K00777|1|3e-30|133|afm:AFUA_7G07100|[EC:2.4.2.-];K14855|2|7e-15|82.4|lbc:LACBIDRAFT_296068|ribosome assembly protein 4;K06666|3|6e-10|66.2|cal:CaO19.13528|glucose repression regulatory protein TUP1
siRNA-t0884079	ACAAAGTCATTGTTCATCTGC	21	1	FGSG_10189	K10392|1|5e-61|236|zro:ZYRO0D12650g|kinesin family member 1/13/14;K10401|3|1e-57|225|afv:AFLA_092580|kinesin family member 18/19;K10398|5|2e-57|224|pgu:PGUG_05568|kinesin family member 11
siRNA-t0908186	ACAACCGCTATTCCAAAAGA	20	1	FGSG_09782	-
siRNA-t0510241	ACAACGATAGTATGAACCCTTC	22	1	FgHV1gp2	-
siRNA-t0801677	ACAAGCCACTGCAGTTTGTA	20	1	FGSG_08249	K01580|1|5e-08|59.7|ctp:CTRG_03478|glutamate decarboxylase [EC:4.1.1.15]
siRNA-t0788676	ACAAGCCGTCAAACATAGACACTTC	25	1	FgHV1gp2	-
siRNA-t1286989	ACAATCCTTACCCAAGATTT	20	1	FgHV1gp2	-
siRNA-t0729027	ACAATCGTGGACTTCCTTTAGG	22	1	FgHV1gp2	-
siRNA-t1235601	ACAATCGTGGACTTCCTTTAGGAAA	25	1	FgHV1gp2	-
siRNA-t1240508	ACAATGCCGCCAACCACCAG	20	1	FGSG_03735	K08711|1|0.0|1404|cne:CNB05770|ATP-binding cassette, subfamily G (WHITE), member 2, PDR
siRNA-t0584675	ACAATGGCGCATACACGGAACCCAT	25	1	FgHV1gp2	-
siRNA-t1148340	ACACACCTTGGTAACCGGCGT	21	1	FGSG_08931	K15116|1|0.0|773|fgr:FG08931.1|solute carrier family 25, member 33/36
siRNA-t1276788	ACACCACATCACGCTCGGGGTCA	23	1	FgHV1gp2	-
siRNA-t0828387	ACACGCGGAACTGTCTCAAG	20	1	FGSG_05990	K02486|1|4e-52|207|spo:SPAC27E2.09|two-component system, unclassified family, sensor kinase [EC:2.7.13.3];K00936|3|5e-34|147|afm:AFUA_6G10240|[EC:2.7.3.-]
siRNA-t0986850	ACACTCGTCGGTTATGATTC	20	1	FGSG_08139	-
siRNA-t0750587	ACAGCCCAAGTCAAGACCCC	20	1	FGSG_06099	-
siRNA-t0595105	ACAGCTCCAATAATCGTTGAAAAT	24	1	FgHV1gp2	-
siRNA-t1188212	ACAGGACCGCGCGATGGCGCGC	22	1	FgHV1gp2	-
siRNA-t1293142	ACAGGCTTTGGTTCCAATTGA	21	1	FGSG_03159	K01684|1|5e-68|258|ani:AN4828.2|galactonate dehydratase [EC:4.2.1.6];K09241|3|9e-09|61.6|ani:AN4785.2|transcriptional regulatory protein GAL4
siRNA-t0948105	ACAGTTCCGCGTGTGATTGGT	21	1	FgHV1gp2	-
siRNA-t1433436	ACATAGACACTTCAACATGGG	21	1	FGSG_07383	K08712|1|0.0|1020|pic:PICST_57529|ATP-binding cassette, subfamily G (WHITE), member 2, SNQ2;K08711|4|0.0|952|cne:CNB05770|ATP-binding cassette, subfamily G (WHITE), member 2, PDR
siRNA-t0411110	ACATATCTCCTTGAACTTGAG	21	1	FGSG_11146	K11987|1|1e-83|311|aor:AOR_1_1372154|prostaglandin-endoperoxide synthase 2 [EC:1.14.99.1]
siRNA-t0877922	ACATCACGCTCGGGGTCAATTTG	23	1	FgHV1gp2	-
siRNA-t1396635	ACATCACGCTCGGGGTCAATTTGA	24	1	FgHV1gp2	-
siRNA-t0547830	ACATCACGCTCGGGGTCAATTTGAT	25	1	FgHV1gp2	-
siRNA-t1410376	ACATCAGGCATGTATGGGTC	20	1	FGSG_03750	K11244|1|7e-07|55.5|fgr:FG10787.1|cell wall integrity and stress response component
siRNA-t0936694	ACATCAGGGAATCGTGTGAAC	21	1	FGSG_09678	K00226|1|0.0|844|fgr:FG09678.1|dihydroorotate dehydrogenase (fumarate) [EC:1.3.98.1]
siRNA-t0784088	ACATCATTCGTGTCAAGCTTCTAA	24	1	FGSG_03747	K01932|1|0.0|677|afm:AFUA_1G10380|[EC:6.3.2.-]
siRNA-t0407098	ACATCCAGATCTTATAGACAGGCC	24	1	FgHV1gp2	-
siRNA-t0670456	ACATCCAGATCTTATAGACAGGCCC	25	1	FgHV1gp2	-
siRNA-t1019977	ACATCGTACCCGTCGAAGACCAA	23	1	FgHV1gp1	-
siRNA-t0703701	ACATGAGGCCAAAGGTGTTCAGCT	24	1	FgHV1gp2	-
siRNA-t0511819	ACATGCCTGATGTTCTTGTGC	21	1	FgHV1gp2	-
siRNA-t0774391	ACCAAAGCCAAGGATAAAGCAT	22	1	FgHV1gp2	-
siRNA-t1269471	ACCAACAGCGGAGATATTCAC	21	1	FGSG_00989	K06036|1|3e-170|597|afm:AFUA_4G03780|[EC:4.2.2.-]
siRNA-t0595618	ACCAACATCATCTCCTATCTA	21	1	FGSG_11372	K00777|1|5e-21|103|afm:AFUA_7G06290|[EC:2.4.2.-];K12823|4|1e-10|69.7|ure:UREG_02211|ATP-dependent RNA helicase DDX5/DBP2 [EC:3.6.4.13]
siRNA-t0540881	ACCAAGAGTGCTCATCGTTG	20	1	FGSG_10431	-
siRNA-t1305176	ACCACAACGATAGTATGAACCCTT	24	1	FgHV1gp2	-
siRNA-t1061959	ACCACAGTCGAATGAAATGGAACA	24	1	FgHV1gp2	-
siRNA-t0492773	ACCACATCACGCTCGGGGTCAATT	24	1	FgHV1gp2	-
siRNA-t0455889	ACCACTGCGAATGTTTCACT	20	1	FgHV1gp2	-
siRNA-t1417248	ACCACTGCGAATGTTTCACTCCTCC	25	1	FgHV1gp2	-
siRNA-t0557743	ACCAGAAACCCATCCCACTC	20	1	FgHV1gp2	-
siRNA-t1430288	ACCAGGCCAACAAAGAGAAC	20	1	FGSG_06226	K15084|1|0.0|743|fgr:FG06226.1|solute carrier family 25 (mitochondrial carrier protein), member 16
siRNA-t0454320	ACCAGTTCCTGCTGAGTGTGGTTTG	25	1	FgHV1gp2	-
siRNA-t0497540	ACCATACAATCCCAGGGCT	20	1	FGSG_01248	K01868|1|0.0|1444|fgr:FG01248.1|threonyl-tRNA synthetase [EC:6.1.1.3]
siRNA-t1243817	ACCATACAATGCCGCCAACC	20	1	FgHV1gp2	-
siRNA-t1248073	ACCATACAATGCCGCCAACCA	21	1	FgHV1gp2	-
siRNA-t0606521	ACCATACTTTCCGAAGGAACT	21	1	FgHV1gp2	-
siRNA-t0439265	ACCATACTTTCCGAAGGAACTC	22	1	FgHV1gp2	-
siRNA-t0867315	ACCATTCACGAACGCTAAGAG	21	1	FgHV1gp2	-
siRNA-t0899444	ACCATTCACGAACGCTAAGAGGA	23	1	FgHV1gp2	-

siRNA-t0775043	ACCATTCACGAACGCTAAGAGGAAA	25	1	FgHV1gp2	-
siRNA-t0577988	ACCCACATCCAGATGTTCAC	20	1	FGSG_02677	K01536|1|0.0|1037|afm:AFUA_6G03690|Na+-exporting ATPase [EC:3.6.3.7];K01537|3|0.0|879|cal:CaO19.6070|Ca2+-transporting ATPase [EC:3.6.3.8]
siRNA-t0710263	ACCCACCTGATGTGCTCATC	20	1	FGSG_00689	-
siRNA-t0971709	ACCCAGCACCTTTCCCTATTTT	22	1	FgHV1gp2	-
siRNA-t1263538	ACCCATCCCACTCGTGAACCTG	22	1	FGSG_10526	K13103|1|0.0|1310|fgr:FG10526.1|tuftelin-interacting protein 11
siRNA-t0385646	ACCCATTTGCGCACATATCTCCT	23	1	FgHV1gp2	-
siRNA-t1001667	ACCCCAGATCCACCCGGATGA	21	1	FgHV1gp2	-
siRNA-t0662688	ACCCGTCGAAGACCAAAAATTTCT	24	1	FgHV1gp1	-
siRNA-t0990540	ACCCGTTTCCTATTCTTGCACCCGG	25	1	FgHV1gp2	-
siRNA-t0765903	ACCCTTCCACGATTGGCGTGAGTGC	25	1	FgHV1gp2	-
siRNA-t1417227	ACCGAAGAAAGACCACATCCA	21	1	FgHV1gp2	-
siRNA-t0603579	ACCGAAGAAAGACCACATCCAGAT	24	1	FgHV1gp2	-
siRNA-t0849188	ACCGACGGTACGTGGAAATCTTCAG	25	1	FgHV1gp2	-
siRNA-t0837021	ACCGACGTTTTGGCGAGACCGA	22	1	FgHV1gp2	-
siRNA-t1433855	ACCGACGTTTTGGCGAGACCGATG	24	1	FgHV1gp2	-
siRNA-t1021997	ACCGACGTTTTGGCGAGACCGATGA	25	1	FgHV1gp2	-
siRNA-t1332729	ACCGATATTGAACCCTCCTTCGTG	24	1	FgHV1gp2	-
siRNA-t0578870	ACGCCGGTTACCAAGGTGTGT	21	1	FgHV1gp2	-
siRNA-t1331529	ACGCCTTCATTGTTCCCTGA	20	1	FGSG_10530	-
siRNA-t1449376	ACGCGACAAGCAACAATTTGCCAA	24	1	FgHV1gp2	-
siRNA-t1240648	ACGCGACAAGCAACAATTTGCCAAG	25	1	FgHV1gp2	-
siRNA-t1053710	ACGCGCCTCCAGTCCAGCCTT	21	1	FgHV1gp2	-
siRNA-t1392355	ACGCGCGATCTATAGGATGG	20	1	FGSG_08540	K15710|1|4e-49|196|yli:YALI0F30261g|E3 ubiquitin-protein ligase SHPRH [EC:3.6.4.- 6.3.2.19];K15083|3|3e-47|190|nhe:NECHADRAFT_123411|DNA repair protein RAD16
siRNA-t0469890	ACGCTATGTGGAGTTCCTTCG	21	1	FGSG_00820	-
siRNA-t0600427	ACGCTCGGGGTCAATTTGATT	21	1	FgHV1gp2	-
siRNA-t0939729	ACGCTCGGGGTCAATTTGATTGA	23	1	FgHV1gp2	-
siRNA-t0564273	ACGCTGCCGGGATGTAATCAA	21	1	FgHV1gp2	-
siRNA-t0796568	ACGGAACCCATTGATCATTCTC	22	1	FgHV1gp2	-
siRNA-t0639279	ACGGAAGCCATCAACGGTTCTTG	23	1	FgHV1gp2	-
siRNA-t0466697	ACGGATCCACATGAGCTTCA	20	1	FGSG_02857	-
siRNA-t0865965	ACGGATCCACATGAGCTTCAGCTT	24	1	FgHV1gp2	-
siRNA-t0816375	ACGGGTTGGCCAAATCTCGGACCG	24	1	FgHV1gp2	-
siRNA-t0750124	ACGTAAGGCGTCGACACGATGA	22	1	FgHV1gp2	-
siRNA-t0399728	ACGTAAGGCGTCGACACGATGAT	23	1	FgHV1gp2	-
siRNA-t0735594	ACGTACGACCCCGAAAGTCTCA	22	1	FgHV1gp1	-
siRNA-t0774834	ACGTACTTAGGGTTGTTGAG	20	1	FGSG_10940	K14564|1|0.0|942|fgr:FG10940.1|nucleolar protein 56
siRNA-t0454311	ACGTACTTAGGGTTGTTGAGA	21	1	FGSG_10239	K14803|1|0.0|858|fgr:FG10239.1|protein phosphatase 2C homolog 2/3 [EC:3.1.3.16]
siRNA-t0613206	ACGTACTTAGGGTTGTTGAGAA	22	1	FGSG_10239	K14803|1|0.0|858|fgr:FG10239.1|protein phosphatase 2C homolog 2/3 [EC:3.1.3.16]
siRNA-t1004411	ACGTCAACAGTGAGCAAGTT	20	1	FGSG_00470	-
siRNA-t1142561	ACGTCATGATCATCAGCACC	20	1	FGSG_11578	K01183|1|2e-16|87.8|afm:AFUA_6G13720|chitinase [EC:3.2.1.14];K01238|3|1e-11|72.4|afm:AFUA_7G08350|[EC:3.2.1.-]
siRNA-t0624349	ACGTCATGATCATCAGCACCG	21	1	FgHV1gp2	-
siRNA-t1425081	ACGTCATGATCATCAGCACCGTAC	24	1	FgHV1gp2	-
siRNA-t0926078	ACGTCCGAATACCATACTTTCCG	23	1	FgHV1gp2	-
siRNA-t1013131	ACGTCCGAATACCATACTTTCCGAA	25	1	FgHV1gp2	-
siRNA-t0997713	ACGTGACCAGCTGTTCTCTGTC	22	1	FgHV1gp2	-
siRNA-t0835590	ACGTGACCAGCTGTTCTCTGTCGG	24	1	FgHV1gp2	-
siRNA-t1342263	AGATCCACCCGGATGACTTGGC	22	1	FgHV1gp2	-
siRNA-t0971514	AGATCCCACTCTAGCAATGCA	21	1	FGSG_00355	K05767|1|0.0|1528|fgr:FG00355.1|IQ motif containing GTPase activating protein
siRNA-t0908558	AGATCTCCGCAGAAGAATTAGCCT	24	1	FgHV1gp2	-
siRNA-t1039325	AGATCTCCGCAGAAGAATTAGCCTT	25	1	FgHV1gp2	-
siRNA-t0445558	AGATGTGATGCCTTGTCTATTATA	24	1	FgHV1gp2	-
siRNA-t0736773	AGATTGAGTTCATTTGTTCCAT	22	1	FgHV1gp2	-
siRNA-t0724607	AGATTGAGTTCATTTGTTCCATAAA	25	1	FgHV1gp2	-
siRNA-t0395860	AGCAAGTGAAACTGTTCGCCAG	22	1	FgHV1gp2	-
siRNA-t1090058	AGCACATCAGGTGGGTTCTTG	21	1	FGSG_06089	K08874|1|0.0|7852|fgr:FG06089.1|transformation/transcription domain-associated protein
siRNA-t0535779	AGCACCATACAATGCCGCCAACCAC	25	1	FgHV1gp2	-
siRNA-t0626270	AGCACCCTGCGAAACAAGGC	20	1	FGSG_05870	K00100|1|7e-90|330|afm:AFUA_5G10790|[EC:1.1.1.-]
siRNA-t0800680	AGCACCGTACGGATCCACATG	21	1	FgHV1gp2	-
siRNA-t1223248	AGCACTTGTTTGCCTGCATCGATGA	25	1	FgHV1gp2	-
siRNA-t0622585	AGCAGACACTTGTTTCTCGTGGGA	24	1	FgHV1gp2	-
siRNA-t1003447	AGCAGGATCTCTGACATATTC	21	1	FGSG_10353	K08052|1|1e-22|108|ssl:SS1G_13541|neurofibromin 1
siRNA-t0698953	AGCCAAACGCGCTAAGTTACAGGCC	25	1	FgHV1gp2	-
siRNA-t0650724	AGCCAAAGAGAGGTGCGTAGCAT	23	1	FgHV1gp2	-
siRNA-t1360947	AGCCACACCCTTAGGACCAGT	21	1	FGSG_08147	K00777|1|9e-20|98.6|afm:AFUA_2G00910|[EC:2.4.2.-]
siRNA-t0511262	AGCCATCATGTATAGTCCCTC	21	1	FgHV1gp2	-
siRNA-t0922081	AGCCATTCGTTACAATCGTGG	21	1	FgHV1gp2	-
siRNA-t0706460	AGCCATTTCGAATCTGCCCGAT	22	1	FgHV1gp2	-
siRNA-t0756839	AGCCCAGTGGCGCATTTCTTTCTTA	25	1	FgHV1gp2	-
siRNA-t0407099	AGGATCGTTATTCTTCTTCT	20	1	FGSG_00698	-
siRNA-t0525646	AGGATCGTTATTCTTCTTCTC	21	1	FgHV1gp2	-
siRNA-t0588593	AGGCCTCTGACGTTGATCTCTTA	23	1	FgHV1gp2	-
siRNA-t0754862	AGGCTTCTTCATGACATGAG	20	1	FGSG_08589	-
siRNA-t0667793	AGGCTTCTTCATGACATGAGGC	22	1	FgHV1gp2	-
siRNA-t1000230	AGTAATTGGTTCTTTAGGTTGAT	23	1	FgHV1gp2	-
siRNA-t1078329	AGTAATTGGTTCTTTAGGTTGATTG	25	1	FgHV1gp2	-
siRNA-t0860920	AGTAGCATGTTCTTCGTGTAGA	22	1	FgHV1gp2	-
siRNA-t0605796	AGTATCGTCACTTCTTCGTAGT	22	1	FgHV1gp2	-
siRNA-t0742388	AGTATCTCGACATTCTATGG	20	1	FGSG_08898	K02350|1|0.0|3330|fgr:FG08898.1|DNA polymerase zeta subunit [EC:2.7.7.7]
siRNA-t1100627	AGTATGAACCCTTCCACGATT	21	1	FgHV1gp2	-
siRNA-t1146322	AGTATTTTCTTCCTGACACATA	22	1	FgHV1gp2	-
siRNA-t1363911	AGTCCCAAATCGGCAGTCTC	20	1	FGSG_09327	-
siRNA-t0795217	AGTCCCTCAAATGTCTCCTTCGGT	24	1	FgHV1gp2	-
siRNA-t1043509	AGTCCCTCAAATGTCTCCTTCGGTT	25	1	FgHV1gp2	-
siRNA-t0754533	AGTCCTAGCAAGTGAAACTGT	21	1	FgHV1gp2	-
siRNA-t0702051	AGTCCTAGCAAGTGAAACTGTTC	23	1	FgHV1gp2	-
siRNA-t1129369	AGTCCTAGCAAGTGAAACTGTTCG	24	1	FgHV1gp2	-
siRNA-t1084488	AGTCCTGAGTGATGGTACCA	20	1	FGSG_12439	-
siRNA-t0631721	AGTCGAATGAAATGGAACAACG	22	1	FgHV1gp2	-
siRNA-t1442709	AGTCTATCACCATCCATGCG	20	1	FGSG_07055	K14556|1|0.0|1839|fgr:FG07055.1|U3 small nucleolar RNA-associated protein 12
siRNA-t0485066	AGTCTCTTGTTCTGGAACGTCAA	23	1	FgHV1gp2	-
siRNA-t0528846	AGTCTCTTGTTCTGGAACGTCAAA	24	1	FgHV1gp2	-
siRNA-t1162953	AGTCTTCGTCCAGTAGCCAATGAGA	25	1	FgHV1gp2	-
siRNA-t1428752	AGTCTTTTCAGCGAATTGCA	20	1	FGSG_11240	K08711|1|0.0|1162|cne:CNB05770|ATP-binding cassette, subfamily G (WHITE), member 2, PDR
siRNA-t0623036	AGTGAAGTCGAAAGTGAAAGTGTCG	25	1	FgHV1gp1	-
siRNA-t0478890	AGTGCCATGACGTACGACCC	20	1	FgHV1gp1	-

<table>
<tr><th></th><th></th><th></th><th></th><th></th><th></th></tr>
<tr><td>siRNA-t0590798</td><td>AGTGCCCGTCAATATTGATGGA</td><td>22</td><td>1</td><td>FGSG_09146</td><td>K00777|1|3e-17|90.9|afm:AFUA_7G07100|[EC:2.4.2.-];K06653|3|7e-12|72.8|vpo:Kpol_1010p59|CDK inhibitor PHO81;K06694|4|2e-11|71.2|pno:SNOG_06697|26S proteasome non-ATPase regulatory subunit 10;K08803|5|2e-11|71.2|fgr:FG07816.1|death-associated protein kinase [EC:2.7.11.1]</td></tr>
<tr><td>siRNA-t1365871</td><td>AGTGCTCATCGTTGTGCCAAC</td><td>21</td><td>1</td><td>FGSG_04332</td><td>-</td></tr>
<tr><td>siRNA-t0370042</td><td>AGTGCTCATCGTTGTGCCAACC</td><td>22</td><td>1</td><td>FGSG_04332</td><td>-</td></tr>
<tr><td>siRNA-t1077370</td><td>AGTGGCTTGTGGTTGCGAAA</td><td>20</td><td>1</td><td>FGSG_05477</td><td>K01104|1|1e-12|74.7|bfu:BC1G_07812|protein-tyrosine phosphatase [EC:3.1.3.48]</td></tr>
<tr><td>siRNA-t0441762</td><td>AGTGTCACCGCCCCGATTCTT</td><td>21</td><td>1</td><td>FgHV1gp2</td><td>-</td></tr>
<tr><td>siRNA-t0502751</td><td>AGTGTCACCGCCCCGATTCTTGTAA</td><td>25</td><td>1</td><td>FgHV1gp2</td><td>-</td></tr>
<tr><td>siRNA-t0626846</td><td>AGTTATCGTGTCTGACTGTTGCTTG</td><td>25</td><td>1</td><td>FgHV1gp2</td><td>-</td></tr>
<tr><td>siRNA-t0936794</td><td>AGTTCATGAATGCCGCCGTAA</td><td>21</td><td>1</td><td>FGSG_05016</td><td>K10908|1|0.0|2607|fgr:FG05016.1|DNA-directed RNA polymerase, mitochondrial [EC:2.7.7.6]</td></tr>
<tr><td>siRNA-t1439299</td><td>AGTTCATGAATGCCGCCGTAAT</td><td>22</td><td>1</td><td>FGSG_05016</td><td>K10908|1|0.0|2607|fgr:FG05016.1|DNA-directed RNA polymerase, mitochondrial [EC:2.7.7.6]</td></tr>
<tr><td>siRNA-t1159790</td><td>AGTTCCTGCTGAGTGTGGTT</td><td>20</td><td>1</td><td>FgHV1gp2</td><td>-</td></tr>
<tr><td>siRNA-t0613845</td><td>AGTTCCTGCTGAGTGTGGTTTGAA</td><td>24</td><td>1</td><td>FgHV1gp2</td><td>-</td></tr>
<tr><td>siRNA-t1153999</td><td>AGTTGATCTTCCCTATGGCCTCG</td><td>23</td><td>1</td><td>FgHV1gp2</td><td>-</td></tr>
<tr><td>siRNA-t0483420</td><td>AGTTGATCTTCCCTATGGCCTCGCA</td><td>25</td><td>1</td><td>FgHV1gp2</td><td>-</td></tr>
<tr><td>siRNA-t1128425</td><td>AGTTGGGGTCTTGACTTGGG</td><td>20</td><td>1</td><td>FGSG_07020</td><td>-</td></tr>
<tr><td>siRNA-t0816563</td><td>AGTTGGTCCGCGAATTCTGC</td><td>20</td><td>1</td><td>FgHV1gp2</td><td>-</td></tr>
<tr><td>siRNA-t0479349</td><td>AGTTGGTCCGCGAATTCTGCC</td><td>21</td><td>1</td><td>FgHV1gp2</td><td>-</td></tr>
<tr><td>siRNA-t1349564</td><td>AGTTGGTCCGCGAATTCTGCCA</td><td>22</td><td>1</td><td>FgHV1gp2</td><td>-</td></tr>
<tr><td>siRNA-t0503304</td><td>AGTTTGCATCCTCGACCCAC</td><td>20</td><td>1</td><td>FGSG_11200</td><td>K00688|1|1e-06|55.5|pno:SNOG_13460|starch phosphorylase [EC:2.4.1.1]</td></tr>
<tr><td>siRNA-t0717804</td><td>AGTTTGCATCCTCGACCCACTCAT</td><td>24</td><td>1</td><td>FgHV1gp2</td><td>-</td></tr>
<tr><td>siRNA-t0802472</td><td>AGTTTGCATCCTCGACCCACTCATG</td><td>25</td><td>1</td><td>FgHV1gp2</td><td>-</td></tr>
<tr><td>siRNA-t0704772</td><td>AGTTTGTACTTGCCGCGCGT</td><td>20</td><td>1</td><td>FGSG_02478</td><td>K14840|1|0.0|696|fgr:FG02478.1|nucleolar protein 53</td></tr>
<tr><td>siRNA-t0842176</td><td>AGTTTGTACTTGCCGCGCGTCTC</td><td>23</td><td>1</td><td>FgHV1gp2</td><td>-</td></tr>
<tr><td>siRNA-t0585426</td><td>AGTTTGTACTTGCCGCGCGTCTCA</td><td>24</td><td>1</td><td>FgHV1gp2</td><td>-</td></tr>
<tr><td>siRNA-t1405492</td><td>ATAAACGCTGCCGGGATGTAAT</td><td>22</td><td>1</td><td>FgHV1gp2</td><td>-</td></tr>
<tr><td>siRNA-t1270533</td><td>ATAAATGAACGTCTGACTGTAG</td><td>22</td><td>1</td><td>FgHV1gp2</td><td>-</td></tr>
<tr><td>siRNA-t1247978</td><td>ATAACACCACATCACGCTCG</td><td>20</td><td>1</td><td>FgHV1gp2</td><td>-</td></tr>
<tr><td>siRNA-t0646138</td><td>ATAACACCACATCACGCTCGGGGT</td><td>24</td><td>1</td><td>FgHV1gp2</td><td>-</td></tr>
<tr><td>siRNA-t1308690</td><td>ATAACACGCAGCAGAATTCTAG</td><td>22</td><td>1</td><td>FgHV1gp2</td><td>-</td></tr>
<tr><td>siRNA-t1087350</td><td>ATAACCTCGTTGGGACCTTTC</td><td>21</td><td>1</td><td>FGSG_05063</td><td>K00480|1|0.0|875|fgr:FG05063.1|salicylate hydroxylase [EC:1.14.13.1]</td></tr>
<tr><td>siRNA-t0506569</td><td>ATAACGACGTTCTTTCCAGACCCAT</td><td>25</td><td>1</td><td>FgHV1gp2</td><td>-</td></tr>
<tr><td>siRNA-t1014466</td><td>ATAATCGGCATGAACTCGGTCAAC</td><td>24</td><td>1</td><td>FgHV1gp2</td><td>-</td></tr>
<tr><td>siRNA-t1262667</td><td>ATACAACTCGAACCAGTTCG</td><td>20</td><td>1</td><td>FGSG_08728</td><td>K02179|1|6e-11|69.7|cgr:CAGL0F06391g|cell cycle arrest protein BUB2</td></tr>
<tr><td>siRNA-t0565948</td><td>ATACAATGCCGCCAACCACCA</td><td>21</td><td>1</td><td>FgHV1gp2</td><td>-</td></tr>
<tr><td>siRNA-t0495274</td><td>ATACACGACGTACTTAGGGTTG</td><td>22</td><td>1</td><td>FgHV1gp2</td><td>-</td></tr>
<tr><td>siRNA-t1099855</td><td>ATACACGACGTACTTAGGGTTGTT</td><td>24</td><td>1</td><td>FgHV1gp2</td><td>-</td></tr>
<tr><td>siRNA-t0803575</td><td>ATACACGACGTACTTAGGGTTGTTG</td><td>25</td><td>1</td><td>FgHV1gp2</td><td>-</td></tr>
<tr><td>siRNA-t0790630</td><td>ATACATGCCTGATGTTCTTG</td><td>20</td><td>1</td><td>FgHV1gp2</td><td>-</td></tr>
<tr><td>siRNA-t1432036</td><td>ATACCATACTTTCCGAAGGAACTCC</td><td>25</td><td>1</td><td>FgHV1gp2</td><td>-</td></tr>
<tr><td>siRNA-t0447825</td><td>ATACCATTCACGAACGCTAAGA</td><td>22</td><td>1</td><td>FgHV1gp2</td><td>-</td></tr>
<tr><td>siRNA-t1163876</td><td>ATACCATTCACGAACGCTAAGAG</td><td>23</td><td>1</td><td>FgHV1gp2</td><td>-</td></tr>
<tr><td>siRNA-t0996916</td><td>ATACCATTCACGAACGCTAAGAGG</td><td>24</td><td>1</td><td>FgHV1gp2</td><td>-</td></tr>
<tr><td>siRNA-t0721990</td><td>ATACCCATTTGCGCACATATCTCCT</td><td>25</td><td>1</td><td>FgHV1gp2</td><td>-</td></tr>
<tr><td>siRNA-t0893317</td><td>ATACCTTTGAGCTCCTTGAGG</td><td>21</td><td>1</td><td>FGSG_06517</td><td>K01687|1|0.0|1239|fgr:FG06517.1|dihydroxy-acid dehydratase [EC:4.2.1.9]</td></tr>
<tr><td>siRNA-t1231723</td><td>ATACGTTCTGAGAAGTCTCCGA</td><td>22</td><td>1</td><td>FgHV1gp2</td><td>-</td></tr>
<tr><td>siRNA-t1363106</td><td>ATAGCCTCTCCCTTCGTGAACTG</td><td>23</td><td>1</td><td>FgHV1gp2</td><td>-</td></tr>
<tr><td>siRNA-t0737522</td><td>ATAGCGGTTGTCTTATAGGACTTG</td><td>24</td><td>1</td><td>FgHV1gp2</td><td>-</td></tr>
<tr><td>siRNA-t0749613</td><td>ATAGCGTCGTCACCTTGATTGG</td><td>22</td><td>1</td><td>FGSG_10511</td><td>K11236|1|0.0|1846|fgr:FG10511.1|cell division control protein 24</td></tr>
<tr><td>siRNA-t1187200</td><td>ATAGTCCCTCAAATGTCTCC</td><td>20</td><td>1</td><td>FgHV1gp2</td><td>-</td></tr>
<tr><td>siRNA-t0616969</td><td>ATAGTCCCTCAAATGTCTCCT</td><td>21</td><td>1</td><td>FgHV1gp2</td><td>-</td></tr>
<tr><td>siRNA-t1037993</td><td>ATAGTGCCCGGCCGTTTGTG</td><td>20</td><td>1</td><td>FgHV1gp2</td><td>-</td></tr>
<tr><td>siRNA-t0573024</td><td>ATGGTCTGCCTAATCTTTCCT</td><td>21</td><td>1</td><td>FgHV1gp2</td><td>-</td></tr>
<tr><td>siRNA-t1459866</td><td>ATGGTCTGCCTAATCTTTCCTC</td><td>22</td><td>1</td><td>FgHV1gp2</td><td>-</td></tr>
<tr><td>siRNA-t0709580</td><td>ATGGTGATTGTGGTCATTTGGTT</td><td>23</td><td>1</td><td>FgHV1gp2</td><td>-</td></tr>
<tr><td>siRNA-t1141944</td><td>ATGGTGGGGTCACATATCTCGAT</td><td>23</td><td>1</td><td>FgHV1gp2</td><td>-</td></tr>
<tr><td>siRNA-t0591434</td><td>ATGGTTGGCACAACGATGAGCAC</td><td>23</td><td>1</td><td>FgHV1gp2</td><td>-</td></tr>
<tr><td>siRNA-t1422352</td><td>ATGTACCGTTCGGCAACAGATC</td><td>22</td><td>1</td><td>FgHV1gp2</td><td>-</td></tr>
<tr><td>siRNA-t1167556</td><td>ATGTACCGTTCGGCAACAGATCT</td><td>23</td><td>1</td><td>FgHV1gp2</td><td>-</td></tr>
<tr><td>siRNA-t1133498</td><td>ATGTAGGCTCCTCCGACAAT</td><td>20</td><td>1</td><td>FGSG_12246</td><td>K06867|1|3e-06|52.8|vpo:Kpol_1039p26|</td></tr>
<tr><td>siRNA-t0422762</td><td>ATGTATAGTCCCTCAAATGTCTCCT</td><td>25</td><td>1</td><td>FgHV1gp2</td><td>-</td></tr>
<tr><td>siRNA-t0973569</td><td>ATGTCAGCAGTGTTCATGTGCA</td><td>22</td><td>1</td><td>FgHV1gp2</td><td>-</td></tr>
<tr><td>siRNA-t1317183</td><td>ATGTCAGCAGTGTTCATGTGCAC</td><td>23</td><td>1</td><td>FgHV1gp2</td><td>-</td></tr>
<tr><td>siRNA-t0640435</td><td>ATGTGGGTGCGCCGAATGAAAG</td><td>22</td><td>1</td><td>FgHV1gp2</td><td>-</td></tr>
<tr><td>siRNA-t0995968</td><td>ATGTGGTCTTTCTTCGGTCTA</td><td>21</td><td>1</td><td>FGSG_14002</td><td>-</td></tr>
<tr><td>siRNA-t1341150</td><td>ATTAGCCATTCGTTACAATCGT</td><td>22</td><td>1</td><td>FgHV1gp2</td><td>-</td></tr>
<tr><td>siRNA-t0947845</td><td>ATTCATGACTTTAAGGTATGAT</td><td>22</td><td>1</td><td>FgHV1gp2</td><td>-</td></tr>
<tr><td>siRNA-t0397514</td><td>ATTCCAACTGCGACCCATGC</td><td>20</td><td>1</td><td>FGSG_01589</td><td>K00777|1|3e-17|91.3|afm:AFUA_7G06290|[EC:2.4.2.-];K12823|3|3e-11|71.6|ure:UREG_02211|ATP-dependent RNA helicase DDX5/DBP2 [EC:3.6.4.13]</td></tr>
<tr><td>siRNA-t0672861</td><td>ATTCCAACTGCGACCCATGCT</td><td>21</td><td>1</td><td>FgHV1gp2</td><td>-</td></tr>
<tr><td>siRNA-t1368419</td><td>ATTCCAACTGCGACCCATGCTTT</td><td>23</td><td>1</td><td>FgHV1gp2</td><td>-</td></tr>
<tr><td>siRNA-t1218129</td><td>ATTCCGGGATCAGGTTCACA</td><td>20</td><td>1</td><td>FGSG_09704</td><td>K01196|1|0.0|3174|fgr:FG09704.1|glycogen debranching enzyme [EC:2.4.1.25 3.2.1.33]</td></tr>
<tr><td>siRNA-t1313807</td><td>ATTCCTTGGGAGGCCTATTCAT</td><td>22</td><td>1</td><td>FgHV1gp2</td><td>-</td></tr>
<tr><td>siRNA-t1415378</td><td>ATTCGACTGTGGTTCAATAT</td><td>20</td><td>1</td><td>FGSG_09607</td><td>K10413|1|0.0|8782|fgr:FG09607.1|dynein heavy chain 1, cytosolic</td></tr>
<tr><td>siRNA-t0615375</td><td>ATTCGCTTCGCATCTGGTGGT</td><td>21</td><td>1</td><td>FgHV1gp1</td><td>-</td></tr>
<tr><td>siRNA-t1340719</td><td>ATTCGGTATTGTCGAGTCGAA</td><td>21</td><td>1</td><td>FgHV1gp2</td><td>-</td></tr>
<tr><td>siRNA-t0869251</td><td>ATTCTCTTGCTCCCACAACT</td><td>20</td><td>1</td><td>FGSG_01049</td><td>-</td></tr>
<tr><td>siRNA-t0707372</td><td>ATTCTGCCATAACCTCGTTGGGAC</td><td>24</td><td>1</td><td>FgHV1gp2</td><td>-</td></tr>
<tr><td>siRNA-t0591589</td><td>ATTCTGCCATAACCTCGTTGGGACC</td><td>25</td><td>1</td><td>FgHV1gp2</td><td>-</td></tr>
<tr><td>siRNA-t0526448</td><td>ATTCTTGAATATGCCACTCC</td><td>20</td><td>1</td><td>FGSG_05671</td><td>K14312|1|0.0|2711|fgr:FG05671.1|nuclear pore complex protein Nup155</td></tr>
<tr><td>siRNA-t0661110</td><td>ATTGAACGGGACTCTTATGGAGA</td><td>23</td><td>1</td><td>FgHV1gp2</td><td>-</td></tr>
<tr><td>siRNA-t1339398</td><td>ATTGATCCATGCCAAACCAACT</td><td>22</td><td>1</td><td>FgHV1gp2</td><td>-</td></tr>
<tr><td>siRNA-t1325136</td><td>ATTGCAACGAGGGCCCAGTCAT</td><td>22</td><td>1</td><td>FgHV1gp2</td><td>-</td></tr>
<tr><td>siRNA-t0766108</td><td>ATTGCAACGAGGGCCCAGTCATGAA</td><td>25</td><td>1</td><td>FgHV1gp2</td><td>-</td></tr>
<tr><td>siRNA-t1049686</td><td>ATTGCCATCACTTCCATCGG</td><td>20</td><td>1</td><td>FGSG_12486</td><td>-</td></tr>
<tr><td>siRNA-t1458228</td><td>ATTGCGATGTAATCTTGAATA</td><td>21</td><td>1</td><td>FGSG_01321</td><td>K05760|1|1e-21|104|mgl:MGL_4132|paxillin</td></tr>
<tr><td>siRNA-t0470721</td><td>ATTGCGATGTAATCTTGAATAAG</td><td>23</td><td>1</td><td>FgHV1gp2</td><td>-</td></tr>
<tr><td>siRNA-t0860127</td><td>ATTGGGCGTGTGTTCTTTATGGGCT</td><td>25</td><td>1</td><td>FgHV1gp2</td><td>-</td></tr>
<tr><td>siRNA-t1186287</td><td>ATTTCGGCGAGTCTAATCTTC</td><td>21</td><td>1</td><td>FgHV1gp2</td><td>-</td></tr>
<tr><td>siRNA-t1478512</td><td>ATTTCGGCGAGTCTAATCTTCTT</td><td>23</td><td>1</td><td>FgHV1gp2</td><td>-</td></tr>
<tr><td>siRNA-t1093558</td><td>ATTTCTCCAGTAGCATGTTCTT</td><td>22</td><td>1</td><td>FGSG_07023</td><td>K01580|1|0.0|990|fgr:FG07023.1|glutamate decarboxylase [EC:4.1.1.15]</td></tr>
<tr><td>siRNA-t0862357</td><td>ATTTCTTTCTTACCCTCGATGT</td><td>22</td><td>1</td><td>FgHV1gp2</td><td>-</td></tr>
<tr><td>siRNA-t0603416</td><td>ATTTGATTGATTCTAGCTTGTGCAA</td><td>25</td><td>1</td><td>FgHV1gp2</td><td>-</td></tr>
<tr><td>siRNA-t1483604</td><td>ATTTGCCTGTGCCGGTGTCGGCT</td><td>23</td><td>1</td><td>FgHV1gp2</td><td>-</td></tr>
<tr><td>siRNA-t1197126</td><td>ATTTGCGCACATATCTCCTTG</td><td>21</td><td>1</td><td>FgHV1gp2</td><td>-</td></tr>
<tr><td>siRNA-t0891422</td><td>ATTTGCGCACATATCTCCTTGAAC</td><td>24</td><td>1</td><td>FgHV1gp2</td><td>-</td></tr>
<tr><td>siRNA-t0876287</td><td>ATTTGGCCCCAGCAGACTTGGAA</td><td>23</td><td>1</td><td>FgHV1gp2</td><td>-</td></tr>
<tr><td>siRNA-t1058766</td><td>ATTTGTTTGTACGTCCGAATACCAT</td><td>25</td><td>1</td><td>FgHV1gp2</td><td>-</td></tr>
<tr><td>siRNA-t0713589</td><td>ATTTTCGCGCGCTGTACTGAA</td><td>21</td><td>1</td><td>FgHV1gp2</td><td>-</td></tr>
<tr><td>siRNA-t1316746</td><td>ATTTTTGGTCTTCGACGGGTAC</td><td>22</td><td>1</td><td>FGSG_13308</td><td>-</td></tr>
</table>

<table>
<tr><td>siRNA-t1321701</td><td>CAAAACGCGCCTCCAGTCCAGCC</td><td>23</td><td>1</td><td>FgHV1gp2</td><td>-</td></tr>
<tr><td>siRNA-t1421943</td><td>CAAAAGCATGGCGATGCACTTT</td><td>22</td><td>1</td><td>FgHV1gp2</td><td>-</td></tr>
<tr><td>siRNA-t0904265</td><td>CAAACTGCAGTGGCTTGTGG</td><td>20</td><td>1</td><td>FGSG_12573</td><td>K07511|1|5e-23|107|cne:CNI00240|enoyl-CoA hydratase [EC:4.2.1.17]</td></tr>
<tr><td>siRNA-t0565998</td><td>CAAAGCCACCCAAATGAAGGA</td><td>21</td><td>1</td><td>FGSG_10617</td><td>K14859|1|2e-98|359|scm:SCHCODRAFT_255991|ribosome biogenesis protein SSF1/2;K00143|2|1e-14|82.0|zro:ZYRO0C16566g|L-aminoadipate-semialdehyde dehydrogenase [EC:1.2.1.31]</td></tr>
<tr><td>siRNA-t1460759</td><td>CAAAGGCTAATTCTTCTGCGG</td><td>21</td><td>1</td><td>FGSG_01338</td><td>K06694|1|5e-128|455|fgr:FG01338.1|26S proteasome non-ATPase regulatory subunit 10</td></tr>
<tr><td>siRNA-t0371285</td><td>CAAAGGTGTTCAGCTTCCTTA</td><td>21</td><td>1</td><td>FgHV1gp2</td><td>-</td></tr>
<tr><td>siRNA-t1116747</td><td>CAAAGTCGTGATTCCTGAAC</td><td>20</td><td>1</td><td>FGSG_05054</td><td>-</td></tr>
<tr><td>siRNA-t0408836</td><td>CAAATAACACCACATCACGCTCGG</td><td>24</td><td>1</td><td>FgHV1gp2</td><td>-</td></tr>
<tr><td>siRNA-t0371753</td><td>CAAATCAAGTTGGTATCCCTC</td><td>21</td><td>1</td><td>FgHV1gp2</td><td>-</td></tr>
<tr><td>siRNA-t0674037</td><td>CAACAATGAAGACGCAAGCCCG</td><td>22</td><td>1</td><td>FgHV1gp2</td><td>-</td></tr>
<tr><td>siRNA-t0554709</td><td>CAACACCAGCGCTATGGCTA</td><td>20</td><td>1</td><td>FGSG_10713</td><td>-</td></tr>
<tr><td>siRNA-t1358554</td><td>CAACCAGGCTAGAATTCTGC</td><td>20</td><td>1</td><td>FGSG_05011</td><td>K13336|1|4e-48|191|afv:AFLA_048740|peroxin-3;K11369|2|1e-44|180|ani:AN3929.2|retrograde regulation protein 2;K08192|3|2e-33|142|dha:DEHA2C12276g|MFS transporter, ACS family, allantoate permease;K03448|4|5e-33|141|act:ACLA_078170|MFS transporter, ACS family, pantothenate transporter</td></tr>
<tr><td>siRNA-t0717041</td><td>CAACCATCAAGCAACAGTCA</td><td>20</td><td>1</td><td>FGSG_08858</td><td>-</td></tr>
<tr><td>siRNA-t1385484</td><td>CAACGAGGGCCCAGTCATGAA</td><td>21</td><td>1</td><td>FgHV1gp2</td><td>-</td></tr>
<tr><td>siRNA-t0961138</td><td>CAACGATAGTATGAACCCTTCCA</td><td>23</td><td>1</td><td>FgHV1gp2</td><td>-</td></tr>
<tr><td>siRNA-t0999254</td><td>CAACGATAGTATGAACCCTTCCAC</td><td>24</td><td>1</td><td>FgHV1gp2</td><td>-</td></tr>
<tr><td>siRNA-t0865706</td><td>CAACGGAAGCGATATTTGCAC</td><td>21</td><td>1</td><td>FgHV1gp2</td><td>-</td></tr>
<tr><td>siRNA-t0803239</td><td>CAACTCGATCGCATATCCTTCGG</td><td>23</td><td>1</td><td>FgHV1gp2</td><td>-</td></tr>
<tr><td>siRNA-t1006587</td><td>CAACTGCGACCCATGCTTTCGACGT</td><td>25</td><td>1</td><td>FgHV1gp2</td><td>-</td></tr>
<tr><td>siRNA-t0521263</td><td>CAAGAACCGTTGATGGCTTCCG</td><td>22</td><td>1</td><td>FGSG_01790</td><td>K00680|1|0.0|2387|afm:AFUA_3G14700|[EC:2.3.1.-];K15419|2|0.0|1077|ani:AN2547.2|emericellamide biosynthesis, highly reducing iterative type I polyketide synthase;K15384|5|0.0|762|afv:AFLA_066840|aspyridone synthetase, hybrid polyketide synthase / nonribosomal peptide synthetase</td></tr>
<tr><td>siRNA-t1304030</td><td>CAAGAAGCAAATCCTGGTGCAA</td><td>22</td><td>1</td><td>FGSG_05149</td><td>K01530|1|0.0|2461|fgr:FG05149.1|phospholipid-translocating ATPase [EC:3.6.3.1]</td></tr>
<tr><td>siRNA-t0659711</td><td>CAAGATTTGTCTAACTTGAT</td><td>20</td><td>1</td><td>FGSG_11080</td><td>-</td></tr>
<tr><td>siRNA-t1367993</td><td>CAAGCCAAGCGGGATCATCCAT</td><td>22</td><td>1</td><td>FgHV1gp1</td><td>-</td></tr>
<tr><td>siRNA-t0990456</td><td>CAAGGAATTCCAGACTTACAA</td><td>21</td><td>1</td><td>FGSG_11988</td><td>K05658|1|0.0|2439|fgr:FG01684.1|ATP-binding cassette, subfamily B (MDR/TAP), member 1</td></tr>
<tr><td>siRNA-t1065679</td><td>CAAGGACGTTCTGGTCTTCGA</td><td>21</td><td>1</td><td>FGSG_09801</td><td>K03164|1|0.0|2817|fgr:FG09801.1|DNA topoisomerase II [EC:5.99.1.3]</td></tr>
<tr><td>siRNA-t1276042</td><td>CAAGTAAGCAGAGCGTGTAG</td><td>20</td><td>1</td><td>FgHV1gp2</td><td>-</td></tr>
<tr><td>siRNA-t0434557</td><td>CAAGTATCGTCACTTCTTCGT</td><td>21</td><td>1</td><td>FgHV1gp2</td><td>-</td></tr>
<tr><td>siRNA-t0525025</td><td>CAAGTATCGTCACTTCTTCGTAG</td><td>23</td><td>1</td><td>FgHV1gp2</td><td>-</td></tr>
<tr><td>siRNA-t0381241</td><td>CAAGTCTCTTGTTCTGGAACG</td><td>21</td><td>1</td><td>FGSG_02381</td><td>-</td></tr>
<tr><td>siRNA-t1127927</td><td>CAAGTCTTCGTCCAGTAGCCA</td><td>21</td><td>1</td><td>FgHV1gp2</td><td>-</td></tr>
<tr><td>siRNA-t0436110</td><td>CAAGTCTTCGTCCAGTAGCCAA</td><td>22</td><td>1</td><td>FgHV1gp2</td><td>-</td></tr>
<tr><td>siRNA-t1401727</td><td>CAAGTCTTCGTCCAGTAGCCAAT</td><td>23</td><td>1</td><td>FgHV1gp2</td><td>-</td></tr>
<tr><td>siRNA-t1417680</td><td>CAATACCATTCACGAACGCT</td><td>20</td><td>1</td><td>FGSG_10435</td><td>K01238|1|4e-09|64.3|afm:AFUA_3G01650|[EC:3.2.1.-];K11244|3|7e-06|53.5|vpo:Kpol_322p6|cell wall integrity and stress response component</td></tr>
<tr><td>siRNA-t0690481</td><td>CAATCGTGGACTTCCTTTAGGAAAG</td><td>25</td><td>1</td><td>FgHV1gp2</td><td>-</td></tr>
<tr><td>siRNA-t0776907</td><td>CAATGAAGACGCAAGCCCGCTTAC</td><td>24</td><td>1</td><td>FgHV1gp2</td><td>-</td></tr>
<tr><td>siRNA-t1370484</td><td>CAATGCATCACCAAGCCAAGC</td><td>21</td><td>1</td><td>FgHV1gp1</td><td>-</td></tr>
<tr><td>siRNA-t0413007</td><td>CAATGGGTTCCGTGTATGCG</td><td>20</td><td>1</td><td>FGSG_08819</td><td>-</td></tr>
<tr><td>siRNA-t1081496</td><td>CAATGTAAGTGACGAGGCCTCT</td><td>22</td><td>1</td><td>FgHV1gp2</td><td>-</td></tr>
<tr><td>siRNA-t0771099</td><td>CAATGTAAGTGACGAGGCCTCTG</td><td>23</td><td>1</td><td>FgHV1gp2</td><td>-</td></tr>
<tr><td>siRNA-t0770193</td><td>CAATGTCAGCAGTGTTCATGTGCA</td><td>24</td><td>1</td><td>FgHV1gp2</td><td>-</td></tr>
<tr><td>siRNA-t1006812</td><td>CAATTCGCTGAAAAGACTTT</td><td>20</td><td>1</td><td>FGSG_13506</td><td>-</td></tr>
<tr><td>siRNA-t1190379</td><td>CACAAAACTTCTGTCCAGTTGAGA</td><td>24</td><td>1</td><td>FGSG_12624</td><td>K00777|1|3e-30|133|afm:AFUA_7G07100|[EC:2.4.2.-];K14855|2|7e-15|82.4|lbc:LACBIDRAFT_296068|ribosome assembly protein 4;K06666|3|6e-10|66.2|cal:Ca019.13528|glucose repression regulatory protein TUP1</td></tr>
<tr><td>siRNA-t0608145</td><td>CACAAACGCTCATGATAACAT</td><td>21</td><td>1</td><td>FgHV1gp2</td><td>-</td></tr>
<tr><td>siRNA-t0556248</td><td>CACAAACGCTCATGATAACATT</td><td>22</td><td>1</td><td>FgHV1gp2</td><td>-</td></tr>
<tr><td>siRNA-t1161184</td><td>CACAAGCCACTGCAGTTTGT</td><td>20</td><td>1</td><td>FGSG_13855</td><td>K01183|1|3e-09|61.6|afm:AFUA_5G01400|chitinase [EC:3.2.1.14]</td></tr>
<tr><td>siRNA-t0495913</td><td>CACACACCTTGGTAACCGGC</td><td>20</td><td>1</td><td>FGSG_01156</td><td>K14303|1|0.0|1353|nhe:NECHADRAFT_89992|nuclear pore complex protein Nup160</td></tr>
<tr><td>siRNA-t1129774</td><td>CACACCCGTCCACGTCTTAA</td><td>20</td><td>1</td><td>FGSG_02238</td><td>-</td></tr>
<tr><td>siRNA-t0394629</td><td>CACACGATTCCCTGATGTGGTG</td><td>22</td><td>1</td><td>FgHV1gp2</td><td>-</td></tr>
<tr><td>siRNA-t1285991</td><td>CACACTCAGCAGGAACTGGT</td><td>20</td><td>1</td><td>FGSG_05573</td><td>-</td></tr>
<tr><td>siRNA-t1016689</td><td>CACAGCTCCAATAATCGTTGAAA</td><td>23</td><td>1</td><td>FgHV1gp2</td><td>-</td></tr>
<tr><td>siRNA-t0924779</td><td>CACAGTCGAATGAAATGGAACA</td><td>22</td><td>1</td><td>FgHV1gp2</td><td>-</td></tr>
<tr><td>siRNA-t1309865</td><td>CACATATCTCCTTGAACTTGAG</td><td>22</td><td>1</td><td>FGSG_11146</td><td>K11987|1|1e-83|311|aor:AOR_1_1372154|prostaglandin-endoperoxide synthase 2 [EC:1.14.99.1]</td></tr>
<tr><td>siRNA-t1299614</td><td>CACATATCTCCTTGAACTTGAGC</td><td>23</td><td>1</td><td>FGSG_11146</td><td>K11987|1|1e-83|311|aor:AOR_1_1372154|prostaglandin-endoperoxide synthase 2 [EC:1.14.99.1]</td></tr>
<tr><td>siRNA-t0448328</td><td>CACATATCTCGATTTCGCAACCA</td><td>23</td><td>1</td><td>FgHV1gp2</td><td>-</td></tr>
<tr><td>siRNA-t1041574</td><td>CACATGAACACTGCTGACATTG</td><td>22</td><td>1</td><td>FGSG_06191</td><td>K01759|1|9e-06|52.4|uma:UM06328.1|lactoylglutathione lyase [EC:4.4.1.5]</td></tr>
<tr><td>siRNA-t1263189</td><td>CACATGAGCTTCAGCTTCGAAG</td><td>22</td><td>1</td><td>FGSG_11244</td><td>-</td></tr>
<tr><td>siRNA-t0559794</td><td>CACCAAGCCAAGCGGGATCATC</td><td>22</td><td>1</td><td>FgHV1gp1</td><td>-</td></tr>
<tr><td>siRNA-t1375890</td><td>CACCAAGCCAAGCGGGATCATCCAT</td><td>25</td><td>1</td><td>FgHV1gp1</td><td>-</td></tr>
<tr><td>siRNA-t0751534</td><td>CACCACATCACGCTCGGGGTC</td><td>21</td><td>1</td><td>FgHV1gp2</td><td>-</td></tr>
<tr><td>siRNA-t0432817</td><td>CACCATACAATGCCGCCAACCAC</td><td>23</td><td>1</td><td>FgHV1gp2</td><td>-</td></tr>
<tr><td>siRNA-t1191682</td><td>CACCCGGATGACTTGGCTCTAG</td><td>22</td><td>1</td><td>FgHV1gp2</td><td>-</td></tr>
<tr><td>siRNA-t1207886</td><td>CACCGCCCCGATTCTTGTAAT</td><td>21</td><td>1</td><td>FgHV1gp2</td><td>-</td></tr>
<tr><td>siRNA-t1452719</td><td>CACCGCGTTTCGCTTGCCAT</td><td>20</td><td>1</td><td>FGSG_13336</td><td>K15505|1|2e-49|197|pno:SNOG_10260|DNA repair protein RAD5 [EC:3.6.4.-]</td></tr>
<tr><td>siRNA-t1145668</td><td>CACCGTACGGATCCACATGA</td><td>20</td><td>1</td><td>FgHV1gp2</td><td>-</td></tr>
<tr><td>siRNA-t0617505</td><td>CACCGTACGGATCCACATGAGC</td><td>22</td><td>1</td><td>FgHV1gp2</td><td>-</td></tr>
<tr><td>siRNA-t1099759</td><td>CACCGTACGGATCCACATGAGCTT</td><td>24</td><td>1</td><td>FgHV1gp2</td><td>-</td></tr>
<tr><td>siRNA-t1036437</td><td>CACCTTCTACCAGAACCCAG</td><td>20</td><td>1</td><td>FGSG_10327</td><td>K00698|1|0.0|1847|fgr:FG10327.1|chitin synthase [EC:2.4.1.16]</td></tr>
<tr><td>siRNA-t1446920</td><td>CACCTTCTACCAGAACCCAGC</td><td>21</td><td>1</td><td>FGSG_10327</td><td>K00698|1|0.0|1847|fgr:FG10327.1|chitin synthase [EC:2.4.1.16]</td></tr>
<tr><td>siRNA-t0802637</td><td>CACCTTCTACCAGAACCCAGCAC</td><td>23</td><td>1</td><td>FgHV1gp2</td><td>-</td></tr>
<tr><td>siRNA-t1393909</td><td>CACCTTCTACCAGAACCCAGCACCT</td><td>25</td><td>1</td><td>FgHV1gp2</td><td>-</td></tr>
<tr><td>siRNA-t1027484</td><td>CACGACGTACTTAGGGTTGT</td><td>20</td><td>1</td><td>FgHV1gp2</td><td>-</td></tr>
<tr><td>siRNA-t0755274</td><td>CACGACGTACTTAGGGTTGTT</td><td>21</td><td>1</td><td>FgHV1gp2</td><td>-</td></tr>
<tr><td>siRNA-t0559801</td><td>CACGAGCCAGATCAGCGATAC</td><td>21</td><td>1</td><td>FGSG_13445</td><td>K07824|1|0.0|1051|fgr:FG08086.1|benzoate 4-monooxygenase [EC:1.14.13.12]</td></tr>
<tr><td>siRNA-t0937858</td><td>CACGAGGGACGAGCTTGGCCC</td><td>21</td><td>1</td><td>FgHV1gp2</td><td>-</td></tr>
<tr><td>siRNA-t0810922</td><td>CACGAGGGACGAGCTTGGCCCC</td><td>22</td><td>1</td><td>FgHV1gp2</td><td>-</td></tr>
<tr><td>siRNA-t0515947</td><td>CACGATTCCCTGATGTGGTG</td><td>20</td><td>1</td><td>FGSG_13060</td><td>-</td></tr>
<tr><td>siRNA-t0474808</td><td>CACGCTCGGGGTCAATTTGATT</td><td>22</td><td>1</td><td>FgHV1gp2</td><td>-</td></tr>
<tr><td>siRNA-t1065892</td><td>CACGCTCTGCTTACTTGGCA</td><td>20</td><td>1</td><td>FGSG_05329</td><td>K03686|1|0.0|978|ani:AN6194.2|molecular chaperone DnaJ;K12200|2|2e-39|164|cpw:CPC735_002480|programmed cell death 6-interacting protein</td></tr>
<tr><td>siRNA-t0652031</td><td>CACGGAACCCATTGATCATTCT</td><td>22</td><td>1</td><td>FGSG_05847</td><td>K01452|1|1e-93|342|mgr:MGG_09159|chitin deacetylase [EC:3.5.1.41]</td></tr>
<tr><td>siRNA-t0465824</td><td>CAGCAAGATCTACATGCATG</td><td>20</td><td>1</td><td>FGSG_06489</td><td>-</td></tr>
<tr><td>siRNA-t0494859</td><td>CAGCAGACACTTGTTTCTCGTG</td><td>22</td><td>1</td><td>FgHV1gp2</td><td>-</td></tr>
<tr><td>siRNA-t0699733</td><td>CAGCAGACACTTGTTTCTCGTGG</td><td>23</td><td>1</td><td>FgHV1gp2</td><td>-</td></tr>
<tr><td>siRNA-t0826988</td><td>CAGCCATCATGTATAGTCCCTCAA</td><td>24</td><td>1</td><td>FgHV1gp2</td><td>-</td></tr>
<tr><td>siRNA-t0488109</td><td>CAGCCATTTCGAATCTGCCCGAT</td><td>23</td><td>1</td><td>FgHV1gp2</td><td>-</td></tr>
<tr><td>siRNA-t0675286</td><td>CAGCGCCTCCATGCATGTAGATC</td><td>23</td><td>1</td><td>FgHV1gp2</td><td>-</td></tr>
</table>

siRNA-t0523990	CAGCGCCTCCATGCATGTAGATCTT	25	1	FgHV1gp2	-
siRNA-t0927281	CAGCGTGAACCAGATCATAA	20	1	FGSG_11372	K00777|1|5e-21|103|afm:AFUA_7G06290|[EC:2.4.2.-];K12823|4|1e-10|69.7|ure:UREG_02211|ATP-dependent RNA helicase DDX5/DBP2 [EC:3.6.4.13]
siRNA-t1197028	CAGCTGCAGCAACTGTCGCCTGG	23	1	FgHV1gp1	-
siRNA-t1195066	CAGGACCGCGCGATGGCGCGCGT	23	1	FgHV1gp2	-
siRNA-t1279951	CAGGATTCTTCTCCACGTCA	20	1	FGSG_08844	K01183|1|1e-06|55.1|pan:PODANSg4492|chitinase [EC:3.2.1.14]
siRNA-t0396651	CAGGCCAACAAAGAGAACTGGC	22	1	FGSG_06226	K15084|1|0.0|743|fgr:FG06226.1|solute carrier family 25 (mitochondrial carrier protein), member 16
siRNA-t0504021	CAGGCCAGTCCATCTTGCAG	20	1	FGSG_08137	K01423|1|3e-08|58.2|uma:UM00094.1|[EC:3.4.-.-]
siRNA-t0566666	CAGGCCTCATTGATTTTGTGTCG	23	1	FgHV1gp2	-
siRNA-t0761890	CAGGCCTCATTGATTTTGTGTCGC	24	1	FgHV1gp2	-
siRNA-t0552372	CAGGCTAGAATTCTGCTGCG	20	1	FGSG_05306	K08333|1|0.0|3035|fgr:FG05306.1|phosphoinositide-3-kinase, regulatory subunit 4, p150 [EC:2.7.11.1]
siRNA-t0814217	CAGGCTAGGAACGATGCCTCG	21	1	FGSG_02233	-
siRNA-t0968673	CAGTACATGCAGCAATGGTA	20	1	FGSG_02482	K00381|1|0.0|3097|fgr:FG02482.1|sulfite reductase (NADPH) hemoprotein beta-component [EC:1.8.1.2]
siRNA-t1450601	CATGTTGAAGTGTCTATGTT	20	1	FGSG_09660	K02677|1|0.0|1989|fgr:FG09660.1|classical protein kinase C [EC:2.7.11.13]
siRNA-t0751413	CATGTTTAGGATCACCAACGCCAA	24	1	FgHV1gp2	-
siRNA-t1318504	CATTCACGAACGCTAAGAGGAAAG	24	1	FgHV1gp2	-
siRNA-t1184469	CATTCCATTGATAATCGGCAT	21	1	FgHV1gp2	-
siRNA-t1134123	CATTCGACTGTGGTTCAATA	20	1	FGSG_09607	K10413|1|0.0|8782|fgr:FG09607.1|dynein heavy chain 1, cytosolic
siRNA-t0685691	CATTCGACTGTGGTTCAATAT	21	1	FGSG_09607	K10413|1|0.0|8782|fgr:FG09607.1|dynein heavy chain 1, cytosolic
siRNA-t1192697	CATTCGTGTCAAGCTTCTAA	20	1	FGSG_13755	K00309|1|9e-07|54.3|afm:AFUA_3G12950|[EC:1.5.3.-]
siRNA-t1052825	CATTCGTTACAATCGTGGACT	21	1	FgHV1gp2	-
siRNA-t1313362	CATTCGTTACAATCGTGGACTTCC	24	1	FgHV1gp2	-
siRNA-t1058901	CATTCGTTACAATCGTGGACTTCCT	25	1	FgHV1gp2	-
siRNA-t0480172	CATTCTCTTCCAGTGTCGAACCATA	25	1	FgHV1gp2	-
siRNA-t1320501	CATTGATCCATGCCAAACCAACTT	24	1	FgHV1gp2	-
siRNA-t1074028	CATTGATGAGGCTTTCAGGGG	21	1	FGSG_03095	K03927|1|3e-38|159|fgr:FG05935.1|carboxylesterase 2 [EC:3.1.1.1 3.1.1.84 3.1.1.56];K01046|2|3e-30|132|mgr:MGG_02130|triacylglycerol lipase [EC:3.1.1.3];K01066|3|3e-28|125|afm:AFUA_1G01310|esterase / lipase [EC:3.1.1.-]
siRNA-t0468444	CATTGATTTTGTGTCGCCTCC	21	1	FgHV1gp2	-
siRNA-t0697219	CATTGATTTTGTGTCGCCTCCAGAC	25	1	FgHV1gp2	-
siRNA-t1149903	CATTGCGATGTAATCTTGAAT	21	1	FgHV1gp2	-
siRNA-t1446277	CATTGTTCATCTGCACTCCTTT	22	1	FgHV1gp2	-
siRNA-t1042920	CATTGTTGATGTTTTCCCTC	20	1	FGSG_06272	K12232|1|0.0|2246|fgr:FG06272.1|E3 ubiquitin-protein ligase HECTD2 [EC:6.3.2.19]
siRNA-t0634718	CATTTCTTTCTTACCCTCGATGT	23	1	FgHV1gp2	-
siRNA-t0954383	CATTTGCCGCACAGCTCCAA	20	1	FgHV1gp2	-
siRNA-t1142381	CATTTGTTCCATAAACGCTGCCGGG	25	1	FgHV1gp2	-
siRNA-t1084455	CATTTGTTTGTACGTCCGAATACCA	25	1	FgHV1gp2	-
siRNA-t1378369	CATTTTCGCGCGCTGTACTG	20	1	FgHV1gp2	-
siRNA-t1454130	CATTTTTGCACCAGGATTTGCT	22	1	FGSG_02369	K03379|1|1e-24|110|mgr:MGG_06577|cyclohexanone monooxygenase [EC:1.14.13.22]
siRNA-t1228163	CCAAAACGCGCCTCCAGTCC	20	1	FgHV1gp2	-
siRNA-t1428443	CCAAAACGCGCCTCCAGTCCAGCC	24	1	FgHV1gp2	-
siRNA-t1299602	CCAAAACGCGCCTCCAGTCCAGCCT	25	1	FgHV1gp2	-
siRNA-t1137385	CCAAACAGAGCAAAGGCGAT	20	1	FGSG_09159	K01188|1|2e-24|113|afm:AFUA_5G07080|beta-glucosidase [EC:3.2.1.21];K01207|2|4e-16|85.9|scm:SCHCODRAFT_76670|beta-N-acetylhexosaminidase [EC:3.2.1.52];K15920|5|1e-12|73.9|ani:AN8401.2|beta-D-xylosidase 4 [EC:3.2.1.37]
siRNA-t0669041	CCAAATCGTCATACATTGCACG	22	1	FgHV1gp2	-
siRNA-t0496889	CCAACACCCTCGATTTCAGCGT	22	1	FGSG_06291	K11359|1|0.0|1947|fgr:FG06291.1|transcriptional activator SPT7
siRNA-t0636719	CCAACCATCAAGCAACAGTCA	21	1	FGSG_08858	-
siRNA-t0511969	CCAACCTCATGAGCACCCTG	20	1	FGSG_06280	-
siRNA-t0770134	CCAACTGCGACCCATGCTTTCGAC	24	1	FgHV1gp2	-
siRNA-t1020543	CCAACTGCGACCCATGCTTTCGACG	25	1	FgHV1gp2	-
siRNA-t0736778	CCAAGAACCGTTGATGGCTTCC	22	1	FGSG_01790	K00680|1|0.0|2387|afm:AFUA_3G14700|[EC:2.3.1.-];K15419|2|0.0|1077|ani:AN2547.2|emericellamide biosynthesis, highly reducing iterative type I polyketide synthase;K15384|5|0.0|762|afv:AFLA_066840|aspyridone synthetase, hybrid polyketide synthase / nonribosomal peptide synthetase
siRNA-t0467847	CCAAGCCACCAGTGGGAAGT	20	1	FGSG_00428	K10415|1|0.0|1226|fgr:FG00428.1|dynein intermediate chain, cytosolic
siRNA-t0699902	CCAATAATCGTTGAAAATCAG	21	1	FgHV1gp2	-
siRNA-t0749549	CCACACCGCGTTTCGCTTGCCA	22	1	FgHV1gp1	-
siRNA-t0713876	CCACACCGCGTTTCGCTTGCCATTA	25	1	FgHV1gp1	-
siRNA-t0383599	CCACAGTCGAATGAAATGGAAC	22	1	FgHV1gp2	-
siRNA-t0497163	CCAGATCTTATAGACAGGCCCAAA	24	1	FgHV1gp2	-
siRNA-t0570119	CCAGCAGACACTTGTTTCTCGTG	23	1	FgHV1gp2	-
siRNA-t0539613	CCAGCAGACACTTGTTTCTCGTGG	24	1	FgHV1gp2	-
siRNA-t0569942	CCAGGATTCTTCTCCACGTCAT	22	1	FGSG_09163	K10949|1|1e-36|153|dha:DEHA2G17974g|ER lumen protein retaining receptor
siRNA-t0564317	CCAGGATTTGCTTCTTGCACT	21	1	FgHV1gp2	-
siRNA-t0767308	CCAGTCATGAATTTTCGGTAG	21	1	FgHV1gp2	-
siRNA-t0644672	CCAGTCATGAATTTTCGGTAGT	22	1	FgHV1gp2	-
siRNA-t0820834	CCAGTCCAGCCTTTTGCCTA	20	1	FGSG_12196	-
siRNA-t0588414	CCAGTCTCTCTTTATTAACCAT	22	1	FgHV1gp1	-
siRNA-t0754337	CCAGTGGCAAAAGTTCTACA	20	1	FGSG_08133	K07203|1|0.0|4876|fgr:FG08133.1|FKBP12-rapamycin complex-associated protein
siRNA-t0774482	CCAGTTCCTGCTGAGTGTGGT	21	1	FGSG_08983	-
siRNA-t1289102	CCAGTTCCTGCTGAGTGTGGTTT	23	1	FgHV1gp2	-
siRNA-t0763062	CCAGTTCCTGCTGAGTGTGGTTTGA	25	1	FgHV1gp2	-
siRNA-t1269527	CCATAAACGCTGCCGGGATG	20	1	FgHV1gp2	-
siRNA-t1141067	CCATACAATGCCGCCAACCAC	21	1	FgHV1gp2	-
siRNA-t0535769	CCATACAATGCCGCCAACCACC	22	1	FgHV1gp2	-
siRNA-t0830706	CCATACATGCCTGATGTTCTTG	22	1	FgHV1gp2	-
siRNA-t0982471	CCATACATGCCTGATGTTCTTGT	23	1	FgHV1gp2	-
siRNA-t0889118	CCATACTTTCCGAAGGACTCC	22	1	FgHV1gp2	-
siRNA-t1427922	CCATACTTTCCGAAGGAACTCCAC	24	1	FgHV1gp2	-
siRNA-t1463814	CCATAGCGCTGGTGTTGAATG	21	1	FgHV1gp2	-
siRNA-t0907391	CCATAGTGCCCGGCCGTTTG	20	1	FgHV1gp2	-
siRNA-t1353348	CCATAGTGCCCGGCCGTTTGT	21	1	FgHV1gp2	-
siRNA-t0949309	CCATCAACGGTTCTTGGAAAGT	22	1	FgHV1gp2	-
siRNA-t1443503	CCATCAACGGTTCTTGGAAAGTTG	24	1	FgHV1gp2	-
siRNA-t0790422	CCATCAAGGTCAGAGAGAAC	20	1	FGSG_13883	K12373|1|0.0|810|nfi:NFIA_032880|hexosaminidase [EC:3.2.1.52]
siRNA-t0625500	CCATCAATGGCGACACCGAC	20	1	FGSG_06108	K15280|1|4e-24|111|yli:YALI0E24959g|solute carrier family 35, member C2
siRNA-t0888640	CCATCAGGACCCGAGAAAAACG	22	1	FgHV1gp2	-
siRNA-t1198552	CCATCATGTATAGTCCCTCAAAT	23	1	FgHV1gp2	-
siRNA-t0622919	CCATCCCACTCGTGAACCTGT	21	1	FgHV1gp2	-
siRNA-t1027716	CCATCCTTGCGTGGTCGTGTGAG	23	1	FgHV1gp2	-
siRNA-t0658750	CCATCGAAGACCAGAACGTCCT	22	1	FGSG_07262	K14550|1|0.0|3445|fgr:FG07262.1|U3 small nucleolar RNA-associated protein 10
siRNA-t0409387	CCATCGAAGACCAGAACGTCCTT	23	1	FGSG_05216	K11238|1|0.0|1960|fgr:FG05216.1|cytokinesis protein
siRNA-t1262478	CCATCGCCACCCAGATTTGTC	21	1	FGSG_00055	-
siRNA-t0505527	CCATCGCGCGGTCCTGTATTT	21	1	FGSG_05512	K11397|1|0.0|2685|fgr:FG05512.1|chromatin modification-related protein VID21

siRNA-t1451954	CCATGATCAGGTGAGATAAA	20	1	FGSG_00502	K02212|1|0.0|773|ure:UREG_00964|minichromosome maintenance protein 4 (cell division control protein 54);K06653|2|2e-09|64.3|yli:YALI0A00759g|CDK inhibitor PHO81
siRNA-t0963748	CCATGCAGCGGCAAATACAGGACCG	25	1	FgHV1gp2	-
siRNA-t1007130	CCATGCTGCCGCGGATAACC	20	1	FGSG_06168	-
siRNA-t1327612	CCATGCTTTCGACGTACTTGG	21	1	FgHV1gp2	-
siRNA-t0391354	CCATGGCTCCACAAACGCTC	20	1	FGSG_11115	K14708|1|4e-75|282|pcs:Pc16g10020|solute carrier family 26 (sodium-independent sulfate anion transporter), member 11
siRNA-t0509276	CCATTCACGAACGCTAAGAG	20	1	FGSG_02928	-
siRNA-t0839038	CCATTCACGAACGCTAAGAGGAAA	24	1	FgHV1gp2	-
siRNA-t1321728	CCATTCGTTACAATCGTGGAC	21	1	FgHV1gp2	-
siRNA-t0808171	CCATTGCTGCATGTACTGTTCGGCA	25	1	FgHV1gp2	-
siRNA-t0713249	CCATTGGTTTGAATGGTCTGC	21	1	FgHV1gp2	-
siRNA-t1363399	CCATTGTCGGAGGAGCCTAC	20	1	FGSG_09198	K10254|1|2e-83|309|ang:ANI_1_1112164|myosin-crossreactive antigen
siRNA-t0407784	CCCGGATGACTTGGCTCTAG	20	1	FGSG_13853	-
siRNA-t0454258	CCCGTCGTTGCGGAAGTCGCACAG	24	1	FGSG_06361	-
siRNA-t1045916	CCCGTTTCCTATTCTTGCACCCGG	24	1	FgHV1gp2	-
siRNA-t0898712	CCCTCAAATGTCTCCTTCGGT	21	1	FgHV1gp2	-
siRNA-t1053728	CCCTCAGTAGTCGACATGTCA	21	1	FGSG_09197	K00021|1|0.0|2147|fgr:FG09197.1|hydroxymethylglutaryl-CoA reductase (NADPH) [EC:1.1.1.34]
siRNA-t0743990	CCCTCATACTTCGCCTTCAA	20	1	FGSG_03607	K05349|1|0.0|637|pte:PTT_17365|beta-glucosidase [EC:3.2.1.21]
siRNA-t1334485	CCCTCCAGTGTCACCGCCCC	20	1	FgHV1gp2	-
siRNA-t0898934	CCCTCGCATTCAGTAGTCGG	20	1	FGSG_08719	K10352|1|0.0|4469|fgr:FG08719.1|myosin heavy chain
siRNA-t1303140	CCCTCGCATTTGCCGCACAGC	21	1	FgHV1gp2	-
siRNA-t0905555	CCCTCTGTCTATGAGCACTTG	21	1	FgHV1gp2	-
siRNA-t0948266	CCCTGACAATACCAAATATCCC	22	1	FgHV1gp2	-
siRNA-t0660238	CCCTGAGGTTTTCCATATCATG	22	1	FGSG_09872	-
siRNA-t1076502	CCCTGGATTAGCCATTCGTTACA	23	1	FgHV1gp2	-
siRNA-t1186142	CCCTTCTTAAGGCACCCTATCCGTA	25	1	FgHV1gp2	-
siRNA-t0767151	CCCTTTCGGGCCACTCGACCTTGT	24	1	FgHV1gp2	-
siRNA-t1486141	CCGAAATTCAACCTGACCTGT	21	1	FgHV1gp2	-
siRNA-t1121231	CCGAAATTCAACCTGACCTGTTCC	24	1	FgHV1gp2	-
siRNA-t0515334	CCGAAATTCAACCTGACCTGTTCCA	25	1	FgHV1gp2	-
siRNA-t0585846	CCGAACGTGATCTCATCATC	20	1	FgHV1gp2	-
siRNA-t0881126	CCGAACGTGATCTCATCATCAC	22	1	FgHV1gp2	-
siRNA-t1210543	CCGAAGAAAGACCACATCCAG	21	1	FgHV1gp2	-
siRNA-t0369327	CCGAAGGAACTCCACATAGC	20	1	FGSG_13665	K03235|1|1e-21|105|mgl:MGL_0347|elongation factor 3
siRNA-t1473916	CCGAATACCATACTTTCCGAAG	22	1	FgHV1gp2	-
siRNA-t0419866	CCGAATACCATACTTTCCGAAGGAA	25	1	FgHV1gp2	-
siRNA-t0578560	CCGAATATCATCCAATGTTATC	22	1	FGSG_09851	K01509|1|2e-109|396|pic:PICST_86369|adenosinetriphosphatase [EC:3.6.1.3];K12196|3|5e-59|228|cgr:CAGL0I06402g|vacuolar protein-sorting-associated protein 4
siRNA-t0803805	CCGAATGAAAGCCCAGTGGCGC	22	1	FGSG_12348	K00224|1|2e-95|348|afm:AFUA_6G00230|[EC:1.3.1.-]
siRNA-t1234192	CCGACAATGGCGCATACACGGAAC	24	1	FgHV1gp2	-
siRNA-t1020935	CCGACGCTCCACTCGCTCTT	20	1	FGSG_10266	-
siRNA-t0857376	CCGTACAACACGCTAAGCTT	20	1	FGSG_11195	K08008|1|9e-24|111|scm:SCHCODRAFT_16162|NADPH oxidase
siRNA-t0639106	CCGTCAAGTTGTGACTGCAGG	21	1	FgHV1gp2	-
siRNA-t1108318	CCGTCGAAGACCAAAAATTTC	21	1	FgHV1gp1	-
siRNA-t1461939	CCGTGATAGCCTCTCCCTTCGT	22	1	FgHV1gp2	-
siRNA-t1408576	CCGTTTCCTATTCTTGCACC	20	1	FgHV1gp2	-
siRNA-t0829108	CCTAATCTTTCCTCTTGATGCGCA	24	1	FgHV1gp2	-
siRNA-t0708919	CCTAATCTTTCCTCTTGATGCGCAC	25	1	FgHV1gp2	-
siRNA-t1139113	CCTAGAGATCTCCGCAGAAG	20	1	FGSG_00352	K08064|1|2e-78|291|mgr:MGG_00354|nuclear transcription factor Y, alpha
siRNA-t1240919	CCTAGCAAGTGAAACTGTTCG	21	1	FgHV1gp2	-
siRNA-t0730941	CCTAGCAAGTGAAACTGTTCGC	22	1	FgHV1gp2	-
siRNA-t1340028	CCTATACTTGATCTCAGCACGCCG	24	1	FgHV1gp2	-
siRNA-t0613121	CCTCAAATCCAAAAGACGCG	20	1	FgHV1gp2	-
siRNA-t0530406	CCTCAAATCCAAAAGACGCGT	21	1	FgHV1gp2	-
siRNA-t0641066	CCTCAAATCCAAAAGACGCGTGTC	24	1	FgHV1gp2	-
siRNA-t1406739	CCTCAAATGTCTCCTTCGGT	20	1	FGSG_09073	K05989|1|0.0|1831|fgr:FG09073.1|alpha-L-rhamnosidase [EC:3.2.1.40]
siRNA-t1202114	CCTCCGAGCACCATACAATGCC	22	1	FgHV1gp2	-
siRNA-t0810347	CCTCCGAGCACCATACAATGCCG	23	1	FgHV1gp2	-
siRNA-t1086593	CCTCCGATGAATCCTCTTCCGAT	23	1	FgHV1gp2	-
siRNA-t1271314	CCTCCTTCCGTAATCATCATCGT	23	1	FgHV1gp2	-
siRNA-t0628254	CCTCCTTCGAACTAGATGGT	20	1	FgHV1gp2	-
siRNA-t0574317	CCTCCTTCGTGATAAATGAACGTC	24	1	FgHV1gp2	-
siRNA-t0435141	CCTCGGTCAGACCCCAGATC	20	1	FgHV1gp2	-
siRNA-t0818682	CCTCTGACGTTGATCTCTTATGC	23	1	FgHV1gp2	-
siRNA-t0937683	CCTCTGACGTTGATCTCTTATGCGC	25	1	FgHV1gp2	-
siRNA-t1476056	CCTCTGATATATTCCAACTGCG	22	1	FgHV1gp2	-
siRNA-t1067877	CCTCTGTCTATGAGCACTTGT	21	1	FGSG_09118	K01183|1|0.0|1040|ssl:SS1G_00773|chitinase [EC:3.2.1.14]
siRNA-t0466077	CCTCTGTCTATGAGCACTTGTT	22	1	FgHV1gp2	-
siRNA-t0524082	CCTCTTATTGATATTAGCCCCCTG	24	1	FgHV1gp2	-
siRNA-t0690965	CCTCTTCCATTCCAGCCTAG	20	1	FGSG_07258	K14572|1|0.0|8396|fgr:FG07258.1|midasin
siRNA-t0952038	CCTGAAAGCCTCATCAATGTG	21	1	FGSG_04898	-
siRNA-t1199199	CGAACCTCAAATCCAAAAGAC	21	1	FgHV1gp2	-
siRNA-t0783104	CGAACGGGACCGACGTTTTGG	21	1	FGSG_02211	-
siRNA-t1204222	CGAACGGGACCGACGTTTTGGC	22	1	FgHV1gp2	-
siRNA-t0497582	CGAACGGGACCGACGTTTTGGCGAG	25	1	FgHV1gp2	-
siRNA-t1135068	CGAACGTGATCTCATCATCACCA	23	1	FgHV1gp2	-
siRNA-t0856779	CGAACGTGATCTCATCATCACCAG	24	1	FgHV1gp2	-
siRNA-t0900395	CGAACTAGATGGTGTGGCGGG	21	1	FgHV1gp2	-
siRNA-t1448291	CGAAGAAAGACCACATCCAGAT	22	1	FgHV1gp2	-
siRNA-t1356886	CGAAGATCCCACTCTAGCAATGCAT	25	1	FgHV1gp1	-
siRNA-t0954854	CGAAGGAACTCCACATAGCG	20	1	FGSG_09607	K10413|1|0.0|8782|fgr:FG09607.1|dynein heavy chain 1, cytosolic
siRNA-t1435559	CGAAGGAGACATTTGAGGGA	20	1	FGSG_00248	-
siRNA-t0647010	CGAATACCATACTTTCCGAA	20	1	FgHV1gp2	-
siRNA-t1232953	CGAATATGTCAGAGATCCTGCT	22	1	FGSG_04858	-
siRNA-t1033209	CGAATCATACATTTCATTGG	20	1	FgHV1gp2	-
siRNA-t1393121	CGAATCATACATTTCATTGGCATA	24	1	FgHV1gp2	-
siRNA-t1342470	CGAATTCTGCCATAACCTCGTTG	23	1	FgHV1gp2	-
siRNA-t0979729	CGACACCGACGGTACGTGGAAAT	23	1	FgHV1gp2	-
siRNA-t0491636	CGACCCATGCTTTCGACGTAC	21	1	FgHV1gp2	-
siRNA-t1389015	CGACCCCTGAAAGCCTCATCAAT	23	1	FgHV1gp1	-
siRNA-t1002080	CGACCTATCTGTCTTTTGCCC	21	1	FGSG_01271	K00924|1|8e-93|341|afm:AFUA_8G05680|[EC:2.7.1.-];K06660|3|3e-70|266|vpo:Kpol_1037p20|cell cycle serine/threonine-protein kinase CDC5/MSD2 [EC:2.7.11.21]
siRNA-t1019623	CGACCTATCTGTCTTTTGCCCA	22	1	FGSG_01271	K00924|1|8e-93|341|afm:AFUA_8G05680|[EC:2.7.1.-];K06660|3|3e-70|266|vpo:Kpol_1037p20|cell cycle serine/threonine-protein kinase CDC5/MSD2 [EC:2.7.11.21]
siRNA-t1250818	CGACGACAAGCCTTATAACAA	21	1	FGSG_09607	K10413|1|0.0|8782|fgr:FG09607.1|dynein heavy chain 1, cytosolic
siRNA-t0584000	CGACGAGTGTGTCGCGTCTACC	22	1	FgHV1gp2	-
siRNA-t1246985	CGACGCTCCACTCGCTCTTC	20	1	FGSG_09991	K11204|1|0.0|1353|fgr:FG09991.1|glutamate--cysteine ligase catalytic subunit [EC:6.3.2.2]
siRNA-t0819453	CGACGCTCCACTCGCTCTTCT	21	1	FGSG_09965	K14300|1|0.0|2662|fgr:FG09965.1|nuclear pore complex protein Nup133
siRNA-t1219020	CGACGGGTTGGCCAAATCTCGGAC	24	1	FgHV1gp2	-
siRNA-t1128131	CGACGGTACGTGGAAATCTT	20	1	FgHV1gp2	-

siRNA-t0371261	CGACGGTACGTGGAAATCTTC	21	1	FgHV1gp2	-
siRNA-t0507718	CGACGTGCTTGAAGGCGAAGT	21	1	FGSG_10435	K01238|1|4e-09|64.3|afm:AFUA_3G01650|[EC:3.2.1.-];K11244|3|7e-06|53.5|vpo:Kpol_322p6|cell wall integrity and stress response component
siRNA-t0872021	CGACGTGCTTGAAGGCGAAGTA	22	1	FgHV1gp2	-
siRNA-t1026470	CGACGTGCTTGAAGGCGAAGTAT	23	1	FgHV1gp2	-
siRNA-t1439058	CGACGTTTTGGCGAGACCGATGA	23	1	FGSG_12997	-
siRNA-t1036917	CGACGTTTTGGCGAGACCGATGAT	24	1	FgHV1gp2	-
siRNA-t0784678	CGACTATAGTTTTCATACAA	20	1	FGSG_10566	-
siRNA-t0787588	CGAGAAAAACGCCCCTGGACG	21	1	FgHV1gp2	-
siRNA-t0776354	CGAGACCGATGATTTCCATCCCC	23	1	FgHV1gp2	-
siRNA-t0510146	CGCTGATCTGGCTCGTGACTTTTC	24	1	FgHV1gp2	-
siRNA-t0800344	CGCTGCCGGGATGTAATCAAT	21	1	FgHV1gp2	-
siRNA-t1488139	CGCTTCTGGATCAATGTCAGCAG	23	1	FGSG_04743	K12828|1|3e-13|77.0|ssl:SS1G_03093|splicing factor 3B subunit 1
siRNA-t0516958	CGGAAGCCATCAACGGTTCTTGG	23	1	FgHV1gp2	-
siRNA-t0724594	CGGATCCACATGAGCTTCAGCTT	23	1	FgHV1gp2	-
siRNA-t1010708	CGGATCCACATGAGCTTCAGCTTCG	25	1	FgHV1gp2	-
siRNA-t1067017	CGGCAGGTAACTCCTTCAAAC	21	1	FGSG_11154	-
siRNA-t0798071	CGGCAGTCCTAGCAAGTGAAACT	23	1	FgHV1gp2	-
siRNA-t0738605	CGGCAGTCCTAGCAAGTGAAACTG	24	1	FgHV1gp2	-
siRNA-t1314945	CGGCGAGTCTAATCTTCTTTGT	22	1	FgHV1gp2	-
siRNA-t1386010	CGGCGAGTCTAATCTTCTTTGTGA	24	1	FgHV1gp2	-
siRNA-t1083564	CGGGACCAGGATTCTTCTCCA	21	1	FGSG_10617	K14859|1|2e-98|359|scm:SCHCODRAFT_255991|ribosome biogenesis protein SSF1/2;K00143|2|1e-14|82.0|zro:ZYRO0C16566g|L-aminoadipate-semialdehyde dehydrogenase [EC:1.2.1.31]
siRNA-t0738081	CGGGCCACTCGACCTTGTCGC	21	1	FGSG_06090	K01907|1|0.0|1438|fgr:FG06090.1|acetoacetyl-CoA synthetase [EC:6.2.1.16]
siRNA-t0402024	CGGGCTTGCGTCTTCATTGTTG	22	1	FGSG_01287	-
siRNA-t1005088	CGGTAATCACAGGGCAACTGAA	22	1	FgHV1gp2	-
siRNA-t1023070	CGGTACGATATTTGAGTATC	20	1	FGSG_05462	K01072|1|2e-159|564|afm:AFUA_2G08440|ubiquitin thiolesterase [EC:3.1.2.15];K11838|3|3e-41|171|clu:CLUG_02963|ubiquitin carboxyl-terminal hydrolase 7 [EC:3.1.2.15]
siRNA-t1354019	CGGTACGTGGAAATCTTCAG	20	1	FgHV1gp2	-
siRNA-t0762161	CGGTGTGGGTCTTCTGACGAATTTG	25	1	FgHV1gp2	-
siRNA-t0764335	CGGTTGAAAATGTTCCACAGT	21	1	FgHV1gp2	-
siRNA-t0685731	CGTAACGGGTCCAGATGTTGTGC	23	1	FgHV1gp2	-
siRNA-t1424337	CGTAACGGGTCCAGATGTTGTGCT	24	1	FgHV1gp2	-
siRNA-t1437210	CGTAACGTGACCAGCTGTTCTCTGT	25	1	FgHV1gp2	-
siRNA-t0414138	CGTAAGCTGCTACTCGAACC	20	1	FgHV1gp2	-
siRNA-t0956504	CGTAAGCTGCTACTCGAACCG	21	1	FgHV1gp2	-
siRNA-t0755587	CGTAAGCTGCTACTCGAACCGC	22	1	FgHV1gp2	-
siRNA-t0492811	CGTAATCATCATCGTAGTCATCGT	24	1	FGSG_09900	-
siRNA-t1225134	CGTAGCATCGATGAACCTGG	20	1	FGSG_12904	K12600|1|0.0|2824|fgr:FG06322.1|superkiller protein 3
siRNA-t1012787	CGTAGGAAAGGAAAAGTTCTCGC	23	1	FgHV1gp1	-
siRNA-t1179322	CGTCATACATTGCACGAGGG	20	1	FgHV1gp2	-
siRNA-t1345310	CGTCATACATTGCACGAGGGA	21	1	FgHV1gp2	-
siRNA-t1463451	CGTCATGATCATCAGCACCGTACG	24	1	FgHV1gp2	-
siRNA-t1009728	CGTCATTCTCTTCCAGTGTC	20	1	FGSG_10911	K05663|1|0.0|1379|fgr:FG10911.1|mitochondrial ABC transporter ATM
siRNA-t1427750	CGTCATTCTCTTCCAGTGTCGA	22	1	FgHV1gp2	-
siRNA-t0795150	CGTCCACCTCGTATTTTGAACTC	23	1	FgHV1gp2	-
siRNA-t1401342	CGTCCAGTAGCCAATGAGAA	20	1	FGSG_08898	K02350|1|0.0|3330|fgr:FG08898.1|DNA polymerase zeta subunit [EC:2.7.7.7]
siRNA-t0692022	CGTCGAAAGCATGGGTCGCAG	21	1	FGSG_08278	-
siRNA-t0613598	CGTGAACCAGATCATAACCC	20	1	FgHV1gp2	-
siRNA-t0470193	CGTGATAGCCTCTCCCTTCGTG	22	1	FgHV1gp2	-
siRNA-t0802427	CGTGATAGCCTCTCCCTTCGTGA	23	1	FgHV1gp2	-
siRNA-t1273540	CGTGCCATCATTGAAAAACA	20	1	FGSG_05542	K14311|1|0.0|3550|fgr:FG05542.1|nuclear pore complex protein Nup188
siRNA-t0421078	CGTGCTGAGATCAAGTATAGG	21	1	FGSG_10790	K15389|1|3e-101|368|ure:UREG_05875|acyl-CoA dehydrogenase;K00257|2|1e-100|366|afm:AFUA_6G10880|[EC:1.3.99.-]
siRNA-t0704498	CGTGGAAATCTTCAGGCCCTGGG	23	1	FgHV1gp2	-
siRNA-t1090463	CGTGTCAAGCTTCTAACGAC	20	1	FGSG_06728	-
siRNA-t1314228	CGTGTCTGACTGTTGCTTGAT	21	1	FgHV1gp2	-
siRNA-t1422169	CGTGTTCTGAAACCATCAGGA	21	1	FgHV1gp2	-
siRNA-t0784623	CGTGTTCTGAAACCATCAGGAC	22	1	FgHV1gp2	-
siRNA-t0367582	CGTGTTGTACGGCGTGGTCTCG	22	1	FgHV1gp2	-
siRNA-t0832326	CGTGTTGTACGGCGTGGTCTCGTT	24	1	FgHV1gp2	-
siRNA-t1383438	CGTTACAATCGTGGACTTCCTTTAG	25	1	FgHV1gp2	-
siRNA-t0883602	CGTTAGAAGCTTGACACGAAT	21	1	FGSG_04030	-
siRNA-t0539722	CGTTAGTGATTTGTGATTTGACA	23	1	FgHV1gp2	-
siRNA-t1346113	CGTTCACACGATTCCCTGATGT	22	1	FgHV1gp2	-
siRNA-t0799656	CGTTCCAGAACAAGAGACTT	20	1	FGSG_00625	K15385|1|1e-33|144|ure:UREG_06498|aspyridone synthetase trans-acting enoyl reductase;K09241|2|7e-06|52.4|pcs:Pc20g03000|transcriptional regulatory protein GAL4
siRNA-t1202998	CGTTCTGTGAACACTAATATCGTC	24	1	FgHV1gp2	-
siRNA-t1135661	CGTTGACAGGCGATAAGGGA	20	1	FGSG_02439	K09885|1|3e-39|162|ani:AN7168.2|aquaporin rerated protein, other eukaryote;K03293|2|2e-07|57.0|lbc:LACBIDRAFT_300838|amino acid transporter, AAT family
siRNA-t0551394	CGTTGACCGAGTTCATGCCGA	21	1	FGSG_10548	K15418|1|0.0|1191|pcs:Pc21g12440|zearalenone synthase, highly reducing iterative type I polyketide synthase;K15419|4|0.0|740|ani:AN2547.2|emericellamide biosynthesis, highly reducing iterative type I polyketide synthase
siRNA-t0506463	CGTTGATGGCTTCCGTCCCT	20	1	FGSG_05634	-
siRNA-t0383298	CTCAGAGTCTTGCGGTGTGGGTCTT	25	1	FgHV1gp2	-
siRNA-t1357892	CTCAGCACGCCGGTTACCAA	20	1	FGSG_08373	K05658|1|1e-16|89.0|mgr:MGG_02348|ATP-binding cassette, subfamily B (MDR/TAP), member 1
siRNA-t1123587	CTCAGCACGCCGGTTACCAAG	21	1	FgHV1gp2	-
siRNA-t1073048	CTCATCATGTAAGGATCTTTT	21	1	FgHV1gp2	-
siRNA-t1080314	CTCCAATAATCGTTGAAAATCAGAG	25	1	FgHV1gp2	-
siRNA-t0957759	CTCCACGTCATGATCATCAG	20	1	FGSG_10904	K06681|1|1e-61|238|cdu:CD36_21160|Gdp/GTP exchange factor required for growth at low temperatures
siRNA-t1130926	CTCCAGTAGCATGTTCTTCGT	21	1	FGSG_09025	K11592|1|0.0|3073|fgr:FG09025.1|endoribonuclease Dicer [EC:3.1.26.-]
siRNA-t0999532	CTCCAGTAGCATGTTCTTCGTG	22	1	FGSG_09025	K11592|1|0.0|3073|fgr:FG09025.1|endoribonuclease Dicer [EC:3.1.26.-]
siRNA-t0430524	CTCCCACGTTCCGCCTCCTTCGA	23	1	FgHV1gp2	-
siRNA-t1348791	CTCCGAACGTGATCTCATCATC	22	1	FgHV1gp2	-
siRNA-t1457986	CTCCGACAATGGCGCATACAC	21	1	FgHV1gp2	-
siRNA-t0533182	CTCCGACAATGGCGCATACACG	22	1	FgHV1gp2	-
siRNA-t0901710	CTCCGACAATGGCGCATACACGGAA	25	1	FgHV1gp2	-
siRNA-t1292165	CTCCGAGCACCATACAATGCCGCC	24	1	FgHV1gp2	-
siRNA-t0900646	CTCCGCAGAAGAATTAGCCTTTG	23	1	FgHV1gp2	-
siRNA-t0514432	CTCCGCTCCGATGTCCACACCGCGT	25	1	FgHV1gp1	-
siRNA-t0845472	CTCCTCCGACAATGGCGCATA	21	1	FGSG_10215	K15289|1|2e-09|62.8|yli:YALI0E22803g|solute carrier family 35, member F5;K15303|2|4e-09|62.0|ani:AN0675.2|aflatoxin B1 aldehyde reductase
siRNA-t1242399	CTCCTCCTTCCGTAATCATCAT	22	1	FgHV1gp2	-
siRNA-t1007078	CTCCTCCTTCCGTAATCATCATC	23	1	FgHV1gp2	-
siRNA-t1103453	CTCCTCCTTCCGTAATCATCATCGT	25	1	FgHV1gp2	-

siRNA-t0391111	CTCCTCTGATATATTCCAAC	20	1	FGSG_03677	K08803|1|8e-17|89.7|fgr:FG07816.1|death-associated protein kinase [EC:2.7.11.1];K00777|4|1e-12|75.5|afm:AFUA_7G06290|[EC:2.4.2.-];K06867|5|4e-12|74.3|sce:YOR034C|
siRNA-t1387615	CTCCTCTGATATATTCCAACT	21	1	FgHV1gp2	-
siRNA-t0392042	CTCCTCTGATATATTCCAACTGCG	24	1	FgHV1gp2	-
siRNA-t0370004	CTCCTCTGATATATTCCAACTGCGA	25	1	FgHV1gp2	-
siRNA-t0525710	CTCCTGCCAGTTTCGATATA	20	1	FGSG_03407	-
siRNA-t1040135	CTCCTTCGAACTAGATGGTG	20	1	FGSG_11057	K08139|1|4e-36|152|sce:YEL069C|MFS transporter, SP family, sugar:H+ symporter;K05841|5|2e-34|146|fgr:FG07850.1|sterol 3beta-glucosyltransferase [EC:2.4.1.173]
siRNA-t0491115	CTCCTTCGAACTAGATGGTGT	21	1	FgHV1gp2	-
siRNA-t0921481	CTCGATCGCATATCCTTCGGTAA	23	1	FgHV1gp2	-
siRNA-t0673874	CTCGATTTCGCAACCACAAGCCACT	25	1	FgHV1gp2	-
siRNA-t0701501	CTCGCCAAAACGTCGGTCCCG	21	1	FGSG_02781	K12599|1|0.0|2405|fgr:FG02781.1|antiviral helicase SKI2 [EC:3.6.4.-]
siRNA-t1266585	CTCGTAAGCTGCTACTCGAAC	21	1	FgHV1gp2	-
siRNA-t0555430	CTCGTTCCCTCCAGTGTCACC	21	1	FGSG_07818	-
siRNA-t0508895	CTCTAGCAATGCATCACCAAGC	22	1	FgHV1gp1	-
siRNA-t0460262	CTCTAGCAATGCATCACCAAGCCA	24	1	FgHV1gp1	-
siRNA-t1423877	CTCTAGCAATGCATCACCAAGCCAA	25	1	FgHV1gp1	-
siRNA-t0874835	CTCTCTGACCTTGATGGCGTC	21	1	FGSG_09454	K14293|1|0.0|1735|fgr:FG09454.1|importin subunit beta-1
siRNA-t0479747	CTCTCTGACCTTGATGGCGTCAAT	24	1	FGSG_09454	K14293|1|0.0|1735|fgr:FG09454.1|importin subunit beta-1
siRNA-t0958081	CTCTGATATATTCCAACTGCG	21	1	FgHV1gp2	-
siRNA-t1213815	CTCTGTGACTCCAGCCAAAGAGAG	24	1	FgHV1gp2	-
siRNA-t1239206	CTCTGTTCCCTTTCTTCCCT	20	1	FGSG_03800	K14950|1|1e-10|66.6|ani:AN3146.2|cation-transporting ATPase 13A1 [EC:3.6.3.-];K01613|2|5e-10|64.7|ani:AN9039.2|phosphatidylserine decarboxylase [EC:4.1.1.65];K02920|3|1e-07|56.6|ani:AN4452.2|large subunit ribosomal protein L36e
siRNA-t0826621	CTCTGTTCCCTTTCTTCCCTC	21	1	FGSG_03800	K14950|1|1e-10|66.6|ani:AN3146.2|cation-transporting ATPase 13A1 [EC:3.6.3.-];K01613|2|5e-10|64.7|ani:AN9039.2|phosphatidylserine decarboxylase [EC:4.1.1.65];K02920|3|1e-07|56.6|ani:AN4452.2|large subunit ribosomal protein L36e
siRNA-t0818956	CTCTTCCAGTGTCGAACCAT	20	1	FGSG_12118	-
siRNA-t0615419	CTCTTCCAGTGTCGAACCATA	21	1	FgHV1gp2	-
siRNA-t0496775	CTCTTGCTCCCACAACTCAT	20	1	FGSG_09851	K01509|1|2e-109|396|pic:PICST_86369|adenosinetriphosphatase [EC:3.6.1.3];K12196|3|5e-59|228|cgr:CAGL0I06402g|vacuolar protein-sorting-associated protein 4
siRNA-t0959642	CTCTTTTGGAATAGCGGTTGT	21	1	FgHV1gp2	-
siRNA-t0732984	CTCTTTTGGAATAGCGGTTGTC	22	1	FgHV1gp2	-
siRNA-t1482789	CTGAAATTTGATGTTGTAGA	20	1	FGSG_09566	K09359|1|2e-07|57.8|sce:YCR039C|mating-type protein A2
siRNA-t0678383	CTGAAGGTGGTGTCATGTGTAA	22	1	FgHV1gp2	-
siRNA-t1086024	CTGAAGTTATGAGCTCTGTG	20	1	FgHV1gp2	-
siRNA-t0772260	CTGAAGTTATGAGCTCTGTGA	21	1	FgHV1gp2	-
siRNA-t1279386	CTGACATATTCGGACTCATC	20	1	FGSG_13252	-
siRNA-t0614144	CTGACGAATTTGGACAAGTACTCC	24	1	FgHV1gp2	-
siRNA-t0813751	CTGACGAATTTGGACAAGTACTCCA	25	1	FgHV1gp2	-
siRNA-t0873776	CTGACGTTGATCTCTTATGCGC	22	1	FgHV1gp2	-
siRNA-t0471051	CTGAGAGAAGAAAGGTGCGA	20	1	FGSG_10468	K09246|1|3e-07|55.5|lel:LELG_03589|arginine metabolism regulation protein II
siRNA-t1472343	CTGATATATTCCAACTGCGACC	22	1	FgHV1gp2	-
siRNA-t1136914	CTGATCTGGCTCGTGACTTTTC	22	1	FgHV1gp2	-
siRNA-t1467903	CTGATGTTCTTGTGCCTCCGA	21	1	FgHV1gp2	-
siRNA-t0940146	CTGATGTTCTTGTGCCTCCGAG	22	1	FgHV1gp2	-
siRNA-t0800653	CTGATGTTCTTGTGCCTCCGAGC	23	1	FgHV1gp2	-
siRNA-t0440232	CTGCAGAAGAAATACAAAAT	20	1	FGSG_00338	-
siRNA-t1207914	CTGCAGCAACTGTCGCCTGG	20	1	FgHV1gp1	-
siRNA-t1343508	CTGCAGTAAAACGTTCACAT	20	1	FGSG_00055	-
siRNA-t1448855	CTGCATGTACTGTTCGGCAAC	21	1	FGSG_01434	-
siRNA-t1305337	CTGCATGTACTGTTCGGCAACAG	23	1	FGSG_01434	-
siRNA-t1400426	CTGCATGTACTGTTCGGCAACAGAA	25	1	FgHV1gp2	-
siRNA-t1336433	CTGCCAAGTCTCCAAAGGGT	20	1	FGSG_02577	-
siRNA-t1312430	CTGCCATAACCTCGTTGGGACC	22	1	FgHV1gp2	-
siRNA-t1256355	CTGCCGCGGATAACCTATAC	20	1	FgHV1gp2	-
siRNA-t0453607	CTGCCGCGGATAACCTATACTTGAT	25	1	FgHV1gp2	-
siRNA-t1031219	CTGCCTCGGTCAGACCCCAGAT	22	1	FgHV1gp2	-
siRNA-t1086747	CTGCGAATGTTTCACTCCTCC	21	1	FgHV1gp2	-
siRNA-t1027564	CTGCGAATGTTTCACTCCTCCT	22	1	FgHV1gp2	-
siRNA-t1396306	CTGCGAATGTTTCACTCCTCCTTCC	25	1	FgHV1gp2	-
siRNA-t0635429	CTGCGACCCATGCTTTCGAC	20	1	FGSG_09206	K13726|1|1e-132|473|nfi:NFIA_027170|metallopeptidase MepB [EC:3.4.24.-]
siRNA-t1182401	CTGCTACTCGAACCGCCCTT	20	1	FGSG_00738	-
siRNA-t1134864	CTGCTATGGCAACGGAAGCGA	21	1	FgHV1gp2	-
siRNA-t0855781	CTGCTCGCACCTTTCTTCTC	20	1	FGSG_12776	K12879|1|0.0|2720|fgr:FG05657.1|THO complex subunit 2
siRNA-t0944692	CTGCTGCGCTATGGTGTGAGA	21	1	FGSG_08808	-
siRNA-t0565872	CTGGAAGAATCATAACCGAC	20	1	FgHV1gp2	-
siRNA-t0705333	GAAAACATAGTCTTCTGACAA	21	1	FGSG_09190	-
siRNA-t0570052	GAAAACTTCCAAGTCTGCTGG	21	1	FGSG_07012	K06892|1|6e-22|103|ani:AN2390.2|;K04125|3|1e-18|93.6|pic:PICST_51972|gibberellin 2-oxidase [EC:1.14.11.13]
siRNA-t1372150	GAAACAGTGAAAGGTAGAACCGCC	24	1	FgHV1gp2	-
siRNA-t0678655	GAAACCCATCCCACTCGTGAACCT	24	1	FgHV1gp2	-
siRNA-t1196236	GAAACGGATGGTGTAGAAAC	20	1	FGSG_08830	K08712|1|0.0|1006|cgr:CAGL0I04862g|ATP-binding cassette, subfamily G (WHITE), member 2, SNQ2
siRNA-t0892496	GAAAGGAAAAGTTCTCGCCAGCAA	24	1	FgHV1gp1	-
siRNA-t0666638	GAAATCGAGGGTGTTGGGGTCC	22	1	FgHV1gp2	-
siRNA-t1094665	GAAATCTTCAGGCCCTGGGATT	22	1	FgHV1gp2	-
siRNA-t0539928	GAAATCTTCAGGCCCTGGGATTGT	24	1	FgHV1gp2	-
siRNA-t0762733	GAAATCTTCAGGCCCTGGGATTGTA	25	1	FgHV1gp2	-
siRNA-t0631220	GAAATGATTTGCGACGTGCTTGAAG	25	1	FgHV1gp2	-
siRNA-t0641156	GAACAAGAGACTTGAACATA	20	1	FGSG_10353	K08052|1|1e-22|108|ssl:SS1G_13541|neurofibromin 1
siRNA-t0768245	GAACAATGAAGGCGTGTTTTTCAA	24	1	FgHV1gp2	-
siRNA-t0979984	GAACAGATGGTGCGCATCAA	20	1	FGSG_04676	K08139|1|9e-49|194|dha:DEHA2C11374g|MFS transporter, SP family, sugar:H+ symporter;K05841|2|9e-44|177|fgr:FG07850.1|sterol 3beta-glucosyltransferase [EC:2.4.1.173]
siRNA-t0579838	GAACCCAGCACCTTTCCCTATTTTC	25	1	FgHV1gp2	-
siRNA-t0791588	GAACCCATTGATCATTCTCCT	21	1	FgHV1gp2	-
siRNA-t1383027	GAACCCATTGATCATTCTCCTCA	23	1	FgHV1gp2	-
siRNA-t1014119	GAACCCATTGATCATTCTCCTCAT	24	1	FgHV1gp2	-
siRNA-t1064805	GAACCCTGACAATACCAAAT	20	1	FgHV1gp2	-
siRNA-t1213424	GAACCCTGACAATACCAAATA	21	1	FgHV1gp2	-
siRNA-t1446789	GAACCTCAAATCCAAAAGACGCG	23	1	FgHV1gp2	-
siRNA-t1144079	GAACGCACGACAATTGTCTC	20	1	FGSG_08389	-
siRNA-t0644420	GAATAAGCCACACCCTTAGGACC	23	1	FgHV1gp2	-
siRNA-t1046419	GAATAGCGGTTGTCTTATAGGACT	24	1	FgHV1gp2	-
siRNA-t0987470	GAATATCTCCGCTGTTGGTA	20	1	FGSG_06198	-
siRNA-t1126254	GAATATCTCCGCTGTTGGTAC	21	1	FGSG_06198	-
siRNA-t0509661	GAATCTGCCCGATCTACTATA	21	1	FgHV1gp2	-
siRNA-t0667700	GAATGACGTTTGTAAAGACCACTG	24	1	FgHV1gp2	-
siRNA-t0471997	GAATGATGTACCTCCCATGC	20	1	FGSG_04336	K05658|1|0.0|692|ssl:SS1G_07287|ATP-binding cassette, subfamily B (MDR/TAP), member 1

siRNA-t0810170	GAATGCTCTGCCTCGGTCAG	20	1	FGSG_03252	-
siRNA-t0756296	GAATGCTCTGCCTCGGTCAGACCCC	25	1	FgHV1gp2	-
siRNA-t1287959	GAATGGTCTGCCTAATCTTT	20	1	FgHV1gp2	-
siRNA-t0787115	GAATGGTCTGCCTAATCTTTCCT	23	1	FgHV1gp2	-
siRNA-t0624036	GAATGTCGAGATACTCTTCAG	21	1	FGSG_13169	K00750|1|4e-16|84.7|cdu:CD36_86420|glycogenin glucosyltransferase [EC:2.4.1.186]
siRNA-t0396002	GAATTCTGCCATAACCTCGTT	21	1	FGSG_12745	K01362|1|0.0|1150|afm:AFUA_1G15270|[EC:3.4.21.-];K01509|2|5e-166|584|pic:PICST_54293|adenosinetriphosphatase [EC:3.6.1.3];K03695|3|1e-161|570|cpw:CPC735_033400|ATP-dependent Clp protease ATP-binding subunit ClpB
siRNA-t0422490	GAATTTCTCCAGTAGCATGTTC	22	1	FgHV1gp2	-
siRNA-t1338429	GAATTTCTCCAGTAGCATGTTCT	23	1	FGSG_07023	K01580|1|0.0|990|fgr:FG07023.1|glutamate decarboxylase [EC:4.1.1.15]
siRNA-t0556649	GAATTTGGACAAGTACTCCACCTTT	25	1	FgHV1gp2	-
siRNA-t0698694	GAATTTCGGTAGTCATCGGGA	22	1	FgHV1gp2	-
siRNA-t1311363	GACAAAGTCACGCTCTAAAG	20	1	FGSG_06743	K14802|1|0.0|2681|fgr:FG06743.1|phospholipid-transporting ATPase [EC:3.6.3.1]
siRNA-t0512829	GACAACAGGTTGGAATCTGC	20	1	FgHV1gp2	-
siRNA-t1027768	GACAAGCCGTCAAACATAGAC	21	1	FgHV1gp2	-
siRNA-t1223976	GACAAGCCGTCAAACATAGACAC	23	1	FgHV1gp2	-
siRNA-t0862752	GACAAGCCGTCAAACATAGACACTT	25	1	FgHV1gp2	-
siRNA-t1351418	GACAATGAAAGATCCTAAAT	20	1	FgHV1gp2	-
siRNA-t0929418	GACAATGAAAGATCCTAAATC	21	1	FgHV1gp2	-
siRNA-t1448869	GACAATGGCGCATACACGGA	20	1	FGSG_05519	K04728|1|0.0|5526|fgr:FG05519.1|ataxia telangiectasia mutated family protein [EC:2.7.11.1]
siRNA-t1092772	GACAATTGTCGTGCGTTCCG	20	1	FGSG_04366	-
siRNA-t0806741	GACAGAGAACAGCTGGTCAC	20	1	FGSG_12230	-
siRNA-t1039786	GACAGTTCCGCGTGTGATTG	20	1	FGSG_07178	K06030|1|6e-59|228|pno:SNOG_10786|mitofusin [EC:3.6.5.-]
siRNA-t0493069	GACCACATCCAGATCTTATA	20	1	FGSG_13130	K01505|1|0.0|665|ani:AN8210.2|1-aminocyclopropane-1-carboxylate deaminase [EC:3.5.99.7]
siRNA-t1130721	GACCAGCTGTTCTCTGTCGG	20	1	FGSG_09020	K01530|1|0.0|2928|fgr:FG09020.1|phospholipid-translocating ATPase [EC:3.6.3.1]
siRNA-t1026368	GACCAGTTCCTGCTGAGTGTGGT	23	1	FgHV1gp2	-
siRNA-t0523170	GACCAGTTCCTGCTGAGTGTGGTTT	25	1	FgHV1gp2	-
siRNA-t1446732	GACCCATACATGCCTGATGTT	21	1	FgHV1gp2	-
siRNA-t1353368	GACCCATACATGCCTGATGTTCTT	24	1	FgHV1gp2	-
siRNA-t0950197	GACCCATGCTTTCGACGTACTTGGG	25	1	FgHV1gp2	-
siRNA-t0636544	GACCCATTCGTGTTCCCGAAA	21	1	FgHV1gp2	-
siRNA-t1019658	GACCCATTCGTGTTCCCGAAAA	22	1	FgHV1gp2	-
siRNA-t0979462	GACCCATTCGTGTTCCCGAAAAC	23	1	FgHV1gp2	-
siRNA-t0698012	GACCCATTCGTGTTCCCGAAAACA	24	1	FgHV1gp2	-
siRNA-t0992704	GACCCCAGATCCACCCGGAT	20	1	FGSG_06580	K11262|1|0.0|4510|fgr:FG06580.1|acetyl-CoA carboxylase / biotin carboxylase [EC:6.4.1.2 6.3.4.14]
siRNA-t1268863	GAGTGCTGAATGCTCTGCCTCG	22	1	FgHV1gp2	-
siRNA-t0488124	GAGTGGAGCTACCTCTTTTGG	21	1	FGSG_00007	K00517|1|4e-47|187|afm:AFUA_8G00760|[EC:1.14.-.-];K10438|4|2e-25|115|aor:AOR_1_2894174|3-hydroxyphenylacetate 6-hydroxylase [EC:1.14.13.63]
siRNA-t0589581	GAGTTTGTACTTGCCGCGCGTC	22	1	FgHV1gp2	-
siRNA-t0776118	GAGTTTGTACTTGCCGCGCGTCTC	24	1	FgHV1gp2	-
siRNA-t1310505	GATAAATGAACGTCTGACTGTAGTG	25	1	FgHV1gp2	-
siRNA-t0890725	GATAACCTTTATCTACGCACTC	22	1	FGSG_01589	K00777|1|3e-17|91.3|afm:AFUA_7G06290|[EC:2.4.2.-];K12823|3|3e-11|71.6|ure:UREG_02211|ATP-dependent RNA helicase DDX5/DBP2 [EC:3.6.4.13]
siRNA-t1243830	GATACGTTCTGAGAAGTCTCCGAA	24	1	FgHV1gp2	-
siRNA-t0894116	GATACTCTTCAGCGTAACGTG	21	1	FgHV1gp2	-
siRNA-t0382403	GATAGCCTCTCCCTTCGTGAACT	23	1	FgHV1gp2	-
siRNA-t1168416	GATATATTCCAACTGCGACCC	21	1	FgHV1gp2	-
siRNA-t0961700	GATATTCGCTTCGCATCTGGTG	22	1	FgHV1gp1	-
siRNA-t1307355	GATATTGAACCCTCCTTCGTGATAA	25	1	FgHV1gp2	-
siRNA-t0800094	GATATTTGCACCATTGCCACTAG	23	1	FgHV1gp2	-
siRNA-t0747414	GATCAATGTCAGCAGTGTTCATGTG	25	1	FgHV1gp2	-
siRNA-t0877543	GATCAATGTTGGGCATCGGCTCT	23	1	FGSG_08390	K12571|1|0.0|1293|fgr:FG08390.1|PAB-dependent poly(A)-specific ribonuclease subunit 2 [EC:3.1.13.4]
siRNA-t0750379	GATCAGGTTCACAATTGCACAT	22	1	FgHV1gp2	-
siRNA-t1467681	GATCATCTTCCCTCTGTCTATGA	23	1	FgHV1gp2	-
siRNA-t0830861	GATCCATGCCAAACCAACTTA	21	1	FgHV1gp2	-
siRNA-t0426589	GATCGGTGGCACACGCATAAG	21	1	FGSG_13987	-
siRNA-t0607011	GATCGGTGGCACACGCATAAGGGCC	25	1	FgHV1gp2	-
siRNA-t1157039	GATCTCAGCACGCCGGTTACCAAG	24	1	FgHV1gp2	-
siRNA-t1039865	GATCTCCGCAGAAGAATTAGCCTT	24	1	FgHV1gp2	-
siRNA-t1444978	GATCTCCGCTCCGATGTCCACA	22	1	FgHV1gp1	-
siRNA-t1351648	GATCTCTAGGATGATGCCAA	20	1	FGSG_11200	K00688|1|1e-06|55.5|pno:SNOG_13460|starch phosphorylase [EC:2.4.1.1]
siRNA-t0720759	GATCTCTAGGATGATGCCAAA	21	1	FGSG_11200	K00688|1|1e-06|55.5|pno:SNOG_13460|starch phosphorylase [EC:2.4.1.1]
siRNA-t1249270	GATCTCTGACATATTCGGACTCATC	25	1	FgHV1gp2	-
siRNA-t0730544	GATCTGGCCGGTTGAAAATGTTC	23	1	FgHV1gp2	-
siRNA-t0757279	GATCTTCCAAGCGGTCATAAGTGAC	25	1	FgHV1gp2	-
siRNA-t0724831	GATCTTCCCTATGGCCTCGCA	21	1	FgHV1gp2	-
siRNA-t1237528	GATCTTGCTGTAGTGTCTGA	20	1	FGSG_07218	-
siRNA-t0949612	GATGAGTCCCATTCTAGCAG	20	1	FGSG_03323	K05658|1|0.0|931|cpw:CPC735_055210|ATP-binding cassette, subfamily B (MDR/TAP), member 1
siRNA-t0409954	GATGAGTCCGAATATGTCAG	20	1	FGSG_08827	-
siRNA-t1265768	GATGAGTCCGAATATGTCAGA	21	1	FGSG_08827	-
siRNA-t0624047	GATGATACCCTCTGGTGGTTCAT	23	1	FGSG_08858	-
siRNA-t1465266	GATGATGCCAAAAGATCCTT	20	1	FGSG_10066	K02218|1|0.0|938|fgr:FG10066.1|casein kinase 1 [EC:2.7.11.1]
siRNA-t0757173	GATGATGTTGGTTAGAACAC	20	1	FGSG_10559	-
siRNA-t0483393	GATGCCTTGTCTATTATATGTCA	23	1	FgHV1gp2	-
siRNA-t1182360	GATGCCTTGTCTATTATATGTCATG	25	1	FgHV1gp2	-
siRNA-t1450177	GATGTAGGCTCCTCCGACAAT	21	1	FGSG_11268	-
siRNA-t0540911	GATGTCCACACCGCGTTTCGCT	22	1	FgHV1gp1	-
siRNA-t1055549	GATGTGGGTGCGCCGAATGAAAG	23	1	FgHV1gp2	-
siRNA-t0918324	GATGTTCTTGTGCCTCCGAG	20	1	FGSG_08474	K12604|1|0.0|4179|fgr:FG08474.1|CCR4-NOT transcription complex subunit 1
siRNA-t0629970	GATGTTCTTGTGCCTCCGAGC	21	1	FgHV1gp2	-
siRNA-t1152867	GATGTTTGCTTGGTTTCGGGAC	22	1	FGSG_09801	K03164|1|0.0|2817|fgr:FG09801.1|DNA topoisomerase II [EC:5.99.1.3]
siRNA-t1430584	GATTCCTTTATGCCAGCATAA	21	1	FGSG_04126	K00365|1|1e-179|627|fgr:FG04126.1|urate oxidase [EC:1.7.3.3]
siRNA-t0604290	GATTGAGTTCATTTGTTCCAT	21	1	FgHV1gp2	-
siRNA-t0675756	GATTGGTGTCCAATGTAAGTGAC	23	1	FgHV1gp2	-
siRNA-t1112942	GATTTCGCAACCACAAGCCACT	22	1	FgHV1gp2	-
siRNA-t1318970	GATTTCGCAACCACAAGCCACTGCA	25	1	FgHV1gp2	-
siRNA-t0754898	GATTTGCCTGTGCCGGTGTCGG	22	1	FgHV1gp2	-
siRNA-t1376580	GATTTGCCTGTGCCGGTGTCGGC	23	1	FgHV1gp2	-
siRNA-t1116936	GATTTGCCTGTGCCGGTGTCGGCT	24	1	FgHV1gp2	-
siRNA-t0694125	GATTTGCGACGTGCTTGAAGG	21	1	FGSG_08200	-
siRNA-t0945386	GATTTGGCCGTCTGGAGGCG	20	1	FGSG_04440	K05658|1|1e-19|99.4|lth:KLTH0B01100g|ATP-binding cassette, subfamily B (MDR/TAP), member 1
siRNA-t1340678	GATTTTGTGTCGCCTCCAGA	20	1	FgHV1gp2	-
siRNA-t1329703	GATTTTGTGTCGCCTCCAGAC	21	1	FgHV1gp2	-
siRNA-t0727331	GCAAAAATGACTCAGGCTCTG	21	1	FGSG_01437	-

siRNA-t0554097	GCAAATATCGCTTCCGTTGCC	21	1	FGSG_05188	K15322|1|2e-51|203|ani:AN8255.2|tRNA-splicing endonuclease subunit Sen2 [EC:3.1.27.9];K06661|2|3e-47|190|afm:AFUA_5G04020|DNA repair protein RAD9;K00547|3|6e-43|176|cim:CIMG_06640|homocysteine S-methyltransferase [EC:2.1.1.10]
siRNA-t1089136	GCAAATTTGGCCAAATCGTCAT	22	1	FGSG_03633	-
siRNA-t1235585	GCAACAGACTTCTTCTCTGCTA	22	1	FgHV1gp2	-
siRNA-t0404525	GCAACAGTCAGACACGATAA	20	1	FGSG_08389	-
siRNA-t1127293	GCAACCACAAGCCACTGCAGTT	22	1	FgHV1gp2	-
siRNA-t1002896	GCAACGAGGGCCCAGTCATG	20	1	FgHV1gp2	-
siRNA-t1323999	GCAAGCCCGCTTACTCATCATGT	23	1	FgHV1gp2	-
siRNA-t1367536	GCACAGCTCCAATAATCGTTGAA	23	1	FgHV1gp2	-
siRNA-t0699762	GCACCATACAATGCCGCCAACCAC	24	1	FgHV1gp2	-
siRNA-t0779807	GCACCCACATCCAGATGTTCA	21	1	FGSG_02640	K11836|1|0.0|1573|fgr:FG02640.1|ubiquitin carboxyl-terminal hydrolase 5/13 [EC:3.1.2.15]
siRNA-t0930752	GCACCGTACGGATCCACATGAG	22	1	FgHV1gp2	-
siRNA-t0454578	GCACCGTACGGATCCACATGAGC	23	1	FgHV1gp2	-
siRNA-t0920127	GCACCTTTCCCTATTTTCTTCGGTG	25	1	FgHV1gp2	-
siRNA-t1394813	GCACGCCGGTTACCAAGGTGTG	22	1	FgHV1gp2	-
siRNA-t0800631	GCACTCTCTTCATTTGGCTT	20	1	FGSG_01049	-
siRNA-t0842304	GCACTGATCAACGTTTCGAG	20	1	FGSG_09227	-
siRNA-t0816280	GCAGACACTTGTTTCTCGTG	20	1	FgHV1gp2	-
siRNA-t1397420	GCAGCGGCAAATACAGGACCG	21	1	FgHV1gp2	-
siRNA-t1355297	GCAGCGTGAACCAGATCATAAC	22	1	FGSG_11372	K00777|1|5e-21|103|afm:AFUA_7G06290|[EC:2.4.2.-];K12823|4|1e-10|69.7|ure:UREG_02211|ATP-dependent RNA helicase DDX5/DBP2 [EC:3.6.4.13]
siRNA-t0669515	GCAGTCCTAGCAAGTGAAACTGTT	24	1	FgHV1gp2	-
siRNA-t0946848	GCATCACATCTACGTTCTTC	20	1	FGSG_09106	-
siRNA-t1159758	GCATCACCAAGCCAAGCGGGA	21	1	FGSG_13758	K06867|1|3e-09|62.0|ago:AGOS_ADL244W|
siRNA-t1231457	GCATCACCAAGCCAAGCGGGAT	22	1	FgHV1gp1	-
siRNA-t0728390	GCATCAGCCATCATGTATAGTCCCT	25	1	FgHV1gp2	-
siRNA-t0417699	GCATCCGCCTATGCTTGTCA	20	1	FgHV1gp2	-
siRNA-t0713518	GCATCCGCCTATGCTTGTCAGCCT	24	1	FgHV1gp2	-
siRNA-t1466607	GCATCCTCGACCCACTCATGGG	22	1	FgHV1gp2	-
siRNA-t1126040	GCATGTACTGTCCGTAATTGA	21	1	FGSG_08568	K11236|1|1e-09|65.5|uma:UM02422.1|cell division control protein 24
siRNA-t0518554	GCATGTATGGGTCGTTTCCC	20	1	FGSG_00842	-
siRNA-t0568173	GCATGTTCTTCGTGTAGAAA	20	1	FGSG_10548	K15418|1|0.0|1191|pcs:Pc21g12440|zearalenone synthase, highly reducing iterative type I polyketide synthase;K15419|4|0.0|740|ani:AN2547.2|emericellamide biosynthesis, highly reducing iterative type I polyketide synthase
siRNA-t0830986	GCATTTCTTTCTTACCCTCGAT	22	1	FgHV1gp2	-
siRNA-t0630365	GCATTTGCCGCACAGCTCCAATA	23	1	FgHV1gp2	-
siRNA-t0537913	GCCAAAACGCGCCTCCAGTCCAGCC	25	1	FgHV1gp2	-
siRNA-t0478885	GCCAAACGCGCTAAGTTACA	20	1	FgHV1gp2	-
siRNA-t0998714	GCCAAATCCTCAGAGTCTTGC	21	1	FGSG_11099	-
siRNA-t1188946	GCCAAATCGTCATACATTGCACG	23	1	FgHV1gp2	-
siRNA-t1032621	GCCAACCATCAAGCAACAGT	20	1	FGSG_08358	K00451|1|0.0|939|fgr:FG08358.1|homogentisate 1,2-dioxygenase [EC:1.13.11.5]
siRNA-t1112575	GCCAACCTCATGAGCACCCT	20	1	FGSG_09186	K00344|1|4e-42|171|afm:AFUA_8G01420|NADPH2:quinone reductase [EC:1.6.5.5];K15385|2|3e-09|62.0|aor:AOR_1_492164|aspyridone synthetase trans-acting enoyl reductase
siRNA-t0961972	GCCAAGCTCGTCCCTCGTGCA	21	1	FGSG_02315	K01932|1|0.0|2455|afm:AFUA_1G10380|[EC:6.3.2.-]
siRNA-t0894164	GCCAAGGATAAAGCATCCAT	20	1	FgHV1gp2	-
siRNA-t0840850	GCCAAGTCATCCGGGTGGAT	20	1	FGSG_12996	K00428|1|9e-23|105|ani:AN1630.2|cytochrome c peroxidase [EC:1.11.1.5]
siRNA-t1202972	GCCACACCCTTAGGACCAGT	20	1	FGSG_08147	K00777|1|9e-20|98.6|afm:AFUA_2G00910|[EC:2.4.2.-]
siRNA-t0444123	GCCAGAATCGAGCTAAACGAACTT	24	1	FgHV1gp2	-
siRNA-t0684617	GCCATAGGGAAGATCAACTT	20	1	FGSG_07150	K00924|1|6e-144|510|afm:AFUA_5G12870|[EC:2.7.1.-];K00858|2|8e-86|317|afm:AFUA_2G01350|NAD+ kinase [EC:2.7.1.23]
siRNA-t0430274	GCCATCAACGGTTCTTGGAA	20	1	FgHV1gp2	-
siRNA-t0551480	GCCATCACTTCCATCGGTCG	20	1	FGSG_01927	K00528|1|0.0|1076|fgr:FG01927.1|ferredoxin--NADP+ reductase [EC:1.18.1.2]
siRNA-t0693405	GCCATCCTATAGATCGCGCG	20	1	FgHV1gp2	-
siRNA-t0497779	GCCATCCTTGCGTGGTCGTGTGAG	24	1	FgHV1gp2	-
siRNA-t0769543	GCCATGCGTCAAAAACCTGA	20	1	FGSG_04907	K14401|1|0.0|2589|fgr:FG04907.1|cleavage and polyadenylation specificity factor subunit 1
siRNA-t0384886	GCCATTTCGAATCTGCCCGATC	22	1	FgHV1gp2	-
siRNA-t0447460	GCCATTTCGAATCTGCCCGATCTAC	25	1	FgHV1gp2	-
siRNA-t0607713	GCCCCGATTCTTGTAATGCATGTTA	25	1	FgHV1gp2	-
siRNA-t1006955	GCCCGCGCATAAGAGATCAA	20	1	FGSG_10548	K15418|1|0.0|1191|pcs:Pc21g12440|zearalenone synthase, highly reducing iterative type I polyketide synthase;K15419|4|0.0|740|ani:AN2547.2|emericellamide biosynthesis, highly reducing iterative type I polyketide synthase
siRNA-t0833619	GCCGACGCTCCACTCGCTCTT	21	1	FGSG_09991	K11204|1|0.0|1353|fgr:FG09991.1|glutamate--cysteine ligase catalytic subunit [EC:6.3.2.2]
siRNA-t0419956	GCCGCACAGCTCCAATAATCGTTGA	25	1	FgHV1gp2	-
siRNA-t1074346	GCCGGATACGTTCTGAGAAGTCT	23	1	FgHV1gp2	-
siRNA-t0541671	GCCGGTTACCAAGGTGTGTGA	21	1	FgHV1gp2	-
siRNA-t0665933	GCCGGTTGAAAATGTTCCACA	21	1	FgHV1gp2	-
siRNA-t0432566	GCCGGTTGAAAATGTTCCACAGTGA	25	1	FgHV1gp2	-
siRNA-t1144976	GCCGTAAGTAGCAGATTTTACAAA	24	1	FgHV1gp2	-
siRNA-t0583957	GCCTCCCACGTTCCGCCTCCT	21	1	FgHV1gp2	-
siRNA-t0816311	GCCTCGGTCAGACCCCAGATCCAC	24	1	FgHV1gp2	-
siRNA-t0559347	GCCTCGTCACTTACATTGGA	20	1	FGSG_12610	-
siRNA-t1181726	GCCTCTGACGTTGATCTCTT	20	1	FGSG_12977	-
siRNA-t0936102	GCCTCTGACGTTGATCTCTTATGC	24	1	FgHV1gp2	-
siRNA-t1094692	GCCTGATGTTCTTGTGCCTCCGAGC	25	1	FgHV1gp2	-
siRNA-t0430622	GCCTTCCATGCTGCCGCGGATA	22	1	FGSG_04587	K00777|1|7e-77|289|afm:AFUA_7G07100|[EC:2.4.2.-];K14855|4|5e-55|216|lbc:LACBIDRAFT_296068|ribosome assembly protein 4
siRNA-t0963156	GCCTTGTCTATTATATGTCATG	22	1	FgHV1gp2	-
siRNA-t0412602	GCGAACCTCAAATCCAAAAGACGC	24	1	FgHV1gp2	-
siRNA-t0726351	GCGAATGTTTCACTCCTCCT	20	1	FgHV1gp2	-
siRNA-t0453586	GCGAATGTTTCACTCCTCCTTCCG	24	1	FgHV1gp2	-
siRNA-t0576765	GCGACAAGCAACAATTTGCCAA	22	1	FgHV1gp2	-
siRNA-t0627690	GCGACAGTTGCTGCAGCTGG	20	1	FGSG_11200	K00688|1|1e-06|55.5|pno:SNOG_13460|starch phosphorylase [EC:2.4.1.1]
siRNA-t1401284	GCGAGAACTTTTCCTTTCCT	20	1	FGSG_04378	-
siRNA-t0829306	GCGATATTTGCACCATTGCCACT	23	1	FgHV1gp2	-
siRNA-t0853023	GCGCAATCAACACAATTTTCC	21	1	FGSG_04168	K12494|1|4e-11|70.9|cnb:CNBD1140|PH and SEC7 domain-containing protein
siRNA-t0946068	GCGCACATATCTCCTTGAAC	20	1	FgHV1gp2	-
siRNA-t1165019	GCGCATTTCTTTCTTACCCTCG	22	1	FgHV1gp2	-
siRNA-t1356031	GCGCCGAATGAAAGCCCAGT	20	1	FGSG_01606	K07300|1|0.0|2091|fgr:FG01606.1|Ca2+:H+ antiporter
siRNA-t1012487	GCGCCGAATGAAAGCCCAGTGG	22	1	FGSG_01606	K07300|1|0.0|2091|fgr:FG01606.1|Ca2+:H+ antiporter
siRNA-t0516387	GCGCCGGATACGTTCTGAGAA	21	1	FgHV1gp2	-
siRNA-t1174424	GCGCCTCCAGTCCAGCCTTT	20	1	FGSG_06947	K00326|1|0.0|637|fgr:FG06947.1|cytochrome-b5 reductase [EC:1.6.2.2];K00360|5|3e-138|490|ncr:NCU00216|nitrate reductase (NADH) [EC:1.7.1.1]
siRNA-t1078327	GCGCCTCCAGTCCAGCCTTTTGCC	24	1	FgHV1gp2	-
siRNA-t0803065	GCGCCTCCATGCATGTAGAT	20	1	FgHV1gp2	-

<table>
<tr><td>siRNA-t1430483</td><td>GCGCGAAAATGTCTGGTAAT</td><td>20</td><td>1</td><td>FGSG_03570</td><td>K01188|1|0.0|931|afm:AFUA_6G08700|beta-glucosidase [EC:3.2.1.21];K13115|2|0.0|855|ani:AN3904.2|coiled-coil domain-containing protein 130;K05349|3|0.0|647|mgr:MGG_09160|beta-glucosidase [EC:3.2.1.21]</td></tr>
<tr><td>siRNA-t0406130</td><td>GCGCGCGTGCCAATGTTTGTGCC</td><td>23</td><td>1</td><td>FgHV1gp2</td><td>-</td></tr>
<tr><td>siRNA-t0758506</td><td>GCGCGGCAAGTACAAACTCC</td><td>20</td><td>1</td><td>FGSG_08503</td><td>-</td></tr>
<tr><td>siRNA-t1242700</td><td>GCGGGACCAGGATTCTTCTCC</td><td>21</td><td>1</td><td>FGSG_08844</td><td>K01183|1|1e-06|55.1|pan:PODANSg4492|chitinase [EC:3.2.1.14]</td></tr>
<tr><td>siRNA-t0524412</td><td>GCTCCGATGTCCACACCGCG</td><td>20</td><td>1</td><td>FGSG_11148</td><td>-</td></tr>
<tr><td>siRNA-t1269150</td><td>GCTCGAGACAATTGTCGTGC</td><td>20</td><td>1</td><td>FGSG_01680</td><td>K01932|1|2e-101|370|afm:AFUA_1G10380|[EC:6.3.2.-]</td></tr>
<tr><td>siRNA-t0701598</td><td>GCTCGTGACTTTTCCCTCACAGGCT</td><td>25</td><td>1</td><td>FgHV1gp2</td><td>-</td></tr>
<tr><td>siRNA-t0901687</td><td>GCTCTGCCTCGGTCAGACCCCAGAT</td><td>25</td><td>1</td><td>FgHV1gp2</td><td>-</td></tr>
<tr><td>siRNA-t0665325</td><td>GCTCTGTGACTCCAGCCAAAGAGAG</td><td>25</td><td>1</td><td>FgHV1gp2</td><td>-</td></tr>
<tr><td>siRNA-t0874509</td><td>GCTCTTCTTAGTAACATGCAT</td><td>21</td><td>1</td><td>FGSG_01964</td><td>K00698|1|0.0|3795|fgr:FG01964.1|chitin synthase [EC:2.4.1.16]</td></tr>
<tr><td>siRNA-t1312118</td><td>GCTGAATGCTCTGCCTCGGTCAG</td><td>23</td><td>1</td><td>FgHV1gp2</td><td>-</td></tr>
<tr><td>siRNA-t1376546</td><td>GCTGATCTGGCTCGTGACTTTTC</td><td>23</td><td>1</td><td>FgHV1gp2</td><td>-</td></tr>
<tr><td>siRNA-t0979748</td><td>GCTGCATGGGAGGTACATCAT</td><td>21</td><td>1</td><td>FGSG_07279</td><td>-</td></tr>
<tr><td>siRNA-t1427160</td><td>GCTGCATGTACTGTTCGGCAACAG</td><td>24</td><td>1</td><td>FGSG_01434</td><td>-</td></tr>
<tr><td>siRNA-t0445539</td><td>GCTGCTACTCGAACCGCCCTTG</td><td>22</td><td>1</td><td>FgHV1gp2</td><td>-</td></tr>
<tr><td>siRNA-t0802484</td><td>GCTGTAGTGTCTGAAGATCTCAGA</td><td>24</td><td>1</td><td>FgHV1gp2</td><td>-</td></tr>
<tr><td>siRNA-t0497492</td><td>GCTTACCCTCGCATTTGCCGCAC</td><td>23</td><td>1</td><td>FgHV1gp2</td><td>-</td></tr>
<tr><td>siRNA-t0376102</td><td>GCTTACCTAGACCGAAGAAA</td><td>20</td><td>1</td><td>FgHV1gp2</td><td>-</td></tr>
<tr><td>siRNA-t1356289</td><td>GCTTACCTAGACCGAAGAAAGA</td><td>22</td><td>1</td><td>FgHV1gp2</td><td>-</td></tr>
<tr><td>siRNA-t0658763</td><td>GCTTACCTAGACCGAAGAAAGACC</td><td>24</td><td>1</td><td>FgHV1gp2</td><td>-</td></tr>
<tr><td>siRNA-t0429391</td><td>GCTTATTCCTCTCCCTCATACT</td><td>22</td><td>1</td><td>FGSG_11743</td><td>K00921|1|0.0|708|fgr:FG00290.1|1-phosphatidylinositol-3-phosphate 5-kinase [EC:2.7.1.150]</td></tr>
<tr><td>siRNA-t1110830</td><td>GCTTCCGGATTATGCCCAAAAG</td><td>22</td><td>1</td><td>FgHV1gp2</td><td>-</td></tr>
<tr><td>siRNA-t1365831</td><td>GCTTCCGTTGCCATAGCAGCA</td><td>21</td><td>1</td><td>FGSG_04015</td><td>-</td></tr>
<tr><td>siRNA-t1061633</td><td>GCTTCTGGATCAATGTCAGCAGT</td><td>23</td><td>1</td><td>FGSG_04743</td><td>K12828|1|3e-13|77.0|ssl:SS1G_03093|splicing factor 3B subunit 1</td></tr>
<tr><td>siRNA-t0847193</td><td>GCTTGACAACAGGTTGGAATC</td><td>21</td><td>1</td><td>FGSG_06666</td><td>-</td></tr>
<tr><td>siRNA-t0639051</td><td>GCTTGCGCCCAAGAAGGCCTAC</td><td>22</td><td>1</td><td>FgHV1gp2</td><td>-</td></tr>
<tr><td>siRNA-t1284655</td><td>GCTTGTCGTCGGGAGAATCCAG</td><td>22</td><td>1</td><td>FgHV1gp2</td><td>-</td></tr>
<tr><td>siRNA-t0440628</td><td>GCTTGTCGTCGGGAGAATCCAGAAC</td><td>25</td><td>1</td><td>FgHV1gp2</td><td>-</td></tr>
<tr><td>siRNA-t0749698</td><td>GCTTTATCCTTGGCTTTGGTGC</td><td>22</td><td>1</td><td>FGSG_08690</td><td>K07088|1|0.0|635|mgr:MGG_10269|;K00866|2|3e-146|517|pno:SNOG_02006|choline kinase [EC:2.7.1.32]</td></tr>
<tr><td>siRNA-t0493656</td><td>GCTTTCGACGTACTTGGGTTCA</td><td>22</td><td>1</td><td>FgHV1gp2</td><td>-</td></tr>
<tr><td>siRNA-t1472453</td><td>GGAAAGGAAAAGTTCTCGCCA</td><td>21</td><td>1</td><td>FgHV1gp1</td><td>-</td></tr>
<tr><td>siRNA-t0776840</td><td>GGAACAAATGAACTCAATCTT</td><td>21</td><td>1</td><td>FGSG_07466</td><td>K14951|1|0.0|2603|fgr:FG07466.1|cation-transporting ATPase 13A3/4/5 [EC:3.6.3.-]</td></tr>
<tr><td>siRNA-t0604331</td><td>GGAACAATGAAGGCGTGTTTTTCA</td><td>24</td><td>1</td><td>FgHV1gp2</td><td>-</td></tr>
<tr><td>siRNA-t0502463</td><td>GGAACCCATTGATCATTCTCCTCAT</td><td>25</td><td>1</td><td>FgHV1gp2</td><td>-</td></tr>
<tr><td>siRNA-t0716745</td><td>GGAAGAAAGGGAACAGAGAA</td><td>20</td><td>1</td><td>FGSG_06101</td><td>K14780|1|0.0|2204|fgr:FG06101.1|ATP-dependent RNA helicase DHX37/DHR1 [EC:3.6.4.13]</td></tr>
<tr><td>siRNA-t1225196</td><td>GGACCAGTTCCTGCTGAGTGTGGTT</td><td>25</td><td>1</td><td>FgHV1gp2</td><td>-</td></tr>
<tr><td>siRNA-t1454146</td><td>GGACCCGAGAAAAACGCCCCTG</td><td>22</td><td>1</td><td>FgHV1gp2</td><td>-</td></tr>
<tr><td>siRNA-t0668445</td><td>GGACGCGACAAGCAACAATT</td><td>20</td><td>1</td><td>FgHV1gp2</td><td>-</td></tr>
<tr><td>siRNA-t0905740</td><td>GGACGCGACAAGCAACAATTTGCC</td><td>24</td><td>1</td><td>FgHV1gp2</td><td>-</td></tr>
<tr><td>siRNA-t0577246</td><td>GGACGTCTTCGATGGATGCTGC</td><td>22</td><td>1</td><td>FgHV1gp2</td><td>-</td></tr>
<tr><td>siRNA-t0802300</td><td>GGACGTCTTCGATGGATGCTGCG</td><td>23</td><td>1</td><td>FgHV1gp2</td><td>-</td></tr>
<tr><td>siRNA-t0582726</td><td>GGACGTCTTCGATGGATGCTGCGAA</td><td>25</td><td>1</td><td>FgHV1gp2</td><td>-</td></tr>
<tr><td>siRNA-t0670133</td><td>GGACGTTCTGGTCTTCGATGGAC</td><td>23</td><td>1</td><td>FgHV1gp2</td><td>-</td></tr>
<tr><td>siRNA-t0775608</td><td>GGACTCATCCCACATGATGGT</td><td>21</td><td>1</td><td>FGSG_02113</td><td>K14338|1|2e-18|94.0|mgr:MGG_10879|cytochrome P450 / NADPH-cytochrome P450 reductase [EC:1.14.14.1 1.6.2.4];K00495|4|2e-17|90.5|uma:UM06473.1|[EC:1.14.14.-]</td></tr>
<tr><td>siRNA-t1420871</td><td>GGAGGGTTCAATATCGGTAC</td><td>20</td><td>1</td><td>FGSG_03219</td><td>K01824|1|2e-09|62.8|fgr:FG10931.1|cholestenol delta-isomerase [EC:5.3.3.5];K09246|3|1e-06|53.9|ang:ANI_1_2296104|arginine metabolism regulation protein II</td></tr>
<tr><td>siRNA-t1020482</td><td>GGAGTTTGTACTTGCCGCGC</td><td>20</td><td>1</td><td>FGSG_02478</td><td>K14840|1|0.0|696|fgr:FG02478.1|nucleolar protein 53</td></tr>
<tr><td>siRNA-t1031557</td><td>GGATACGTTCTGAGAAGTCTC</td><td>21</td><td>1</td><td>FGSG_12118</td><td>-</td></tr>
<tr><td>siRNA-t1260919</td><td>GGATCAATGTCAGCAGTGTTC</td><td>21</td><td>1</td><td>FGSG_10057</td><td>-</td></tr>
<tr><td>siRNA-t0485528</td><td>GGATCTCCGCTCCGATGTCCACA</td><td>23</td><td>1</td><td>FgHV1gp1</td><td>-</td></tr>
<tr><td>siRNA-t0873289</td><td>GGATCTCTGACATATTCGGAC</td><td>21</td><td>1</td><td>FgHV1gp2</td><td>-</td></tr>
<tr><td>siRNA-t1370634</td><td>GGATGACTTGGCTCTAGTTG</td><td>20</td><td>1</td><td>FGSG_04305</td><td>-</td></tr>
<tr><td>siRNA-t0665972</td><td>GGATGAGTCCGAATATGTCAG</td><td>21</td><td>1</td><td>FGSG_08827</td><td>-</td></tr>
<tr><td>siRNA-t0425589</td><td>GGATGCAAAACCGGTGCGAT</td><td>20</td><td>1</td><td>FgHV1gp2</td><td>-</td></tr>
<tr><td>siRNA-t0547544</td><td>GGATGGCATGGTCATTGACGA</td><td>21</td><td>1</td><td>FGSG_02366</td><td>K01078|1|1e-43|176|pno:SNOG_00663|acid phosphatase [EC:3.1.3.2];K00517|2|1e-19|97.1|afm:AFUA_2G04290|[EC:1.14.-.-];K14338|3|1e-18|93.6|fgr:FG07596.1|cytochrome P450 / NADPH-cytochrome P450 reductase [EC:1.14.14.1 1.6.2.4]</td></tr>
<tr><td>siRNA-t1448613</td><td>GGATTGAAGTGTAATGCGTTGAT</td><td>23</td><td>1</td><td>FgHV1gp2</td><td>-</td></tr>
<tr><td>siRNA-t0410587</td><td>GGCAAATTTGGCCAAATCGTCAT</td><td>23</td><td>1</td><td>FgHV1gp2</td><td>-</td></tr>
<tr><td>siRNA-t1220435</td><td>GGCAAGGCCCTCAGTAGTCGA</td><td>21</td><td>1</td><td>FgHV1gp2</td><td>-</td></tr>
<tr><td>siRNA-t1110323</td><td>GGCAAGTACAAACTCCCTTT</td><td>20</td><td>1</td><td>FGSG_00615</td><td>-</td></tr>
<tr><td>siRNA-t1223307</td><td>GGCAGCATGGAAGGCGCGAG</td><td>20</td><td>1</td><td>FGSG_01429</td><td>K14309|1|0.0|2016|fgr:FG01429.1|nuclear pore complex protein Nup93</td></tr>
<tr><td>siRNA-t0723418</td><td>GGCATCAGGGGTCCTCTTAT</td><td>20</td><td>1</td><td>FGSG_08910</td><td>-</td></tr>
<tr><td>siRNA-t0877781</td><td>GGCATGACTTTACAAGCTGGTCT</td><td>23</td><td>1</td><td>FgHV1gp2</td><td>-</td></tr>
<tr><td>siRNA-t1059598</td><td>GGCATGACTTTACAAGCTGGTCTG</td><td>24</td><td>1</td><td>FgHV1gp2</td><td>-</td></tr>
<tr><td>siRNA-t1261570</td><td>GGCATGTATGGGTCGTTTCC</td><td>20</td><td>1</td><td>FGSG_08097</td><td>K00688|1|7e-16|87.0|pno:SNOG_13460|starch phosphorylase [EC:2.4.1.1]</td></tr>
<tr><td>siRNA-t0868520</td><td>GGCCACTCGACCTTGTCGCTGC</td><td>22</td><td>1</td><td>FgHV1gp2</td><td>-</td></tr>
<tr><td>siRNA-t1165700</td><td>GGCCCAGTCATGAATTTTCGGTAG</td><td>24</td><td>1</td><td>FgHV1gp2</td><td>-</td></tr>
<tr><td>siRNA-t0692456</td><td>GGCCGGCAGTCCTAGCAAGTG</td><td>21</td><td>1</td><td>FgHV1gp2</td><td>-</td></tr>
<tr><td>siRNA-t0615421</td><td>GGCCGGCAGTCCTAGCAAGTGA</td><td>22</td><td>1</td><td>FgHV1gp2</td><td>-</td></tr>
<tr><td>siRNA-t1122011</td><td>GGCCTATTCATCGTCAATGA</td><td>20</td><td>1</td><td>FGSG_09801</td><td>K03164|1|0.0|2817|fgr:FG09801.1|DNA topoisomerase II [EC:5.99.1.3]</td></tr>
<tr><td>siRNA-t1233895</td><td>GGCCTCATTGATTTTGTGTCG</td><td>21</td><td>1</td><td>FGSG_10548</td><td>K15418|1|0.0|1191|pcs:Pc21g12440|zearalenone synthase, highly reducing iterative type I polyketide synthase;K15419|4|0.0|740|ani:AN2547.2|emericellamide biosynthesis, highly reducing iterative type I polyketide synthase</td></tr>
<tr><td>siRNA-t0986055</td><td>GGCCTCATTGATTTTGTGTCGC</td><td>22</td><td>1</td><td>FGSG_10548</td><td>K15418|1|0.0|1191|pcs:Pc21g12440|zearalenone synthase, highly reducing iterative type I polyketide synthase;K15419|4|0.0|740|ani:AN2547.2|emericellamide biosynthesis, highly reducing iterative type I polyketide synthase</td></tr>
<tr><td>siRNA-t1153457</td><td>GGCCTCCCACGTTCCGCCTC</td><td>20</td><td>1</td><td>FgHV1gp2</td><td>-</td></tr>
<tr><td>siRNA-t0787245</td><td>GGCCTCCCACGTTCCGCCTCC</td><td>21</td><td>1</td><td>FGSG_01190</td><td>-</td></tr>
<tr><td>siRNA-t1291199</td><td>GGCGCATTTCTTTCTTACCCT</td><td>21</td><td>1</td><td>FgHV1gp2</td><td>-</td></tr>
<tr><td>siRNA-t0607065</td><td>GGCGCATTTCTTTCTTACCCTC</td><td>22</td><td>1</td><td>FgHV1gp2</td><td>-</td></tr>
<tr><td>siRNA-t0794026</td><td>GGCGCATTTCTTTCTTACCCTCG</td><td>23</td><td>1</td><td>FgHV1gp2</td><td>-</td></tr>
<tr><td>siRNA-t0370615</td><td>GGCGCATTTCTTTCTTACCCTCGA</td><td>24</td><td>1</td><td>FgHV1gp2</td><td>-</td></tr>
<tr><td>siRNA-t0514152</td><td>GGCGCGCGTGCCAATGTTTGTG</td><td>22</td><td>1</td><td>FgHV1gp2</td><td>-</td></tr>
<tr><td>siRNA-t0934520</td><td>GGCGCGCGTGCCAATGTTTGTGC</td><td>23</td><td>1</td><td>FgHV1gp2</td><td>-</td></tr>
<tr><td>siRNA-t0888686</td><td>GGCGCGCGTGCCAATGTTTGTGCCA</td><td>25</td><td>1</td><td>FgHV1gp2</td><td>-</td></tr>
<tr><td>siRNA-t1283593</td><td>GGCGTCGACACGATGATACC</td><td>20</td><td>1</td><td>FgHV1gp2</td><td>-</td></tr>
<tr><td>siRNA-t1349845</td><td>GGCGTGTGTTCTTTATGGGCTC</td><td>22</td><td>1</td><td>FgHV1gp2</td><td>-</td></tr>
<tr><td>siRNA-t1007750</td><td>GGCTCCTCCGACAATGGCGC</td><td>20</td><td>1</td><td>FGSG_10215</td><td>K15289|1|2e-09|62.8|yli:YALI0E22803g|solute carrier family 35, member F5;K15303|2|4e-09|62.0|ani:AN0675.2|aflatoxin B1 aldehyde reductase</td></tr>
<tr><td>siRNA-t0774310</td><td>GGCTCCTCCGACAATGGCGCATAC</td><td>24</td><td>1</td><td>FgHV1gp2</td><td>-</td></tr>
<tr><td>siRNA-t0419144</td><td>GGCTGCCAAGTAAGCAGAGCGTGT</td><td>24</td><td>1</td><td>FgHV1gp2</td><td>-</td></tr>
<tr><td>siRNA-t1164021</td><td>GGCTTGGTGATGCATTGCTA</td><td>20</td><td>1</td><td>FGSG_04291</td><td>K02999|1|0.0|3375|fgr:FG04291.1|DNA-directed RNA polymerase I subunit RPA1 [EC:2.7.7.6]</td></tr>
<tr><td>siRNA-t0396266</td><td>GGGGTCACATATCTCGATTTC</td><td>21</td><td>1</td><td>FgHV1gp2</td><td>-</td></tr>
</table>

siRNA-t0563363	GGGGTCAGCCATTTCGAATCTGCC	24	1	FgHV1gp2	-
siRNA-t1175880	GGGGTGTGTATCCAATGAGTTG	22	1	FgHV1gp2	-
siRNA-t0703417	GGGTCACATATCTCGATTTC	20	1	FgHV1gp2	-
siRNA-t1172699	GGGTCAGCCATTTCGAATCT	20	1	FgHV1gp2	-
siRNA-t0925819	GGGTCAGCCATTTCGAATCTGCC	23	1	FgHV1gp2	-
siRNA-t0531869	GGGTCAGCCATTTCGAATCTGCCC	24	1	FgHV1gp2	-
siRNA-t1372208	GGGTCATATCTGGGTCCAGT	20	1	FGSG_08702	K01553|1|1e-94|347|pic:PICST_30460|myosin ATPase [EC:3.6.4.1];K12614|3|1e-14|82.0|ani:AN3460.2|ATP-dependent RNA helicase DDX6/DHH1 [EC:3.6.4.13];K06674|4|4e-08|60.1|lbc:LACBIDRAFT_191040|structural maintenance of chromosome 2;K06669|5|4e-08|60.1|pic:PICST_641|structural maintenance of chromosome 3 (chondroitin sulfate proteoglycan 6)
siRNA-t0935488	GGGTCCAGATGTTGTGCTTT	20	1	FGSG_12230	-
siRNA-t0375098	GGGTCCAGATGTTGTGCTTTTT	22	1	FgHV1gp2	-
siRNA-t0414637	GGGTCCAGTATGGTTCGACA	20	1	FGSG_09077	-
siRNA-t0980447	GGGTCGAGGATGCAAACTTC	20	1	FGSG_07064	-
siRNA-t0952052	GGGTGCAAGAATAGGAAACG	20	1	FGSG_09143	K01467|1|6e-07|55.1|pic:PICST_88817|beta-lactamase [EC:3.5.2.6]
siRNA-t1100686	GGGTGCGCCGAATGAAAGCCCAGTG	25	1	FgHV1gp2	-
siRNA-t0768831	GGTAACGACCCCTGAAAGCCTCA	23	1	FgHV1gp1	-
siRNA-t0805734	GGTGGTGTCATGTGTAATTGACA	23	1	FgHV1gp2	-
siRNA-t0948780	GGTGTGGCGGGACCAGGATT	20	1	FGSG_08904	-
siRNA-t0874581	GGTGTGGGTCTTCTGACGAAT	21	1	FGSG_04995	K01536|1|0.0|1046|afm:AFUA_2G01320|Na+-exporting ATPase [EC:3.6.3.7];K01537|4|0.0|761|spo:SPBC839.06|Ca2+-transporting ATPase [EC:3.6.3.8]
siRNA-t1269225	GGTGTGGGTCTTCTGACGAATTT	23	1	FgHV1gp2	-
siRNA-t1170260	GGTTCAAACCTCAGGTACCTT	21	1	FGSG_08823	K05658|1|0.0|2440|fgr:FG08823.1|ATP-binding cassette, subfamily B (MDR/TAP), member 1
siRNA-t1252322	GGTTCAAACCTCAGGTACCTTT	22	1	FGSG_08823	K05658|1|0.0|2440|fgr:FG08823.1|ATP-binding cassette, subfamily B (MDR/TAP), member 1
siRNA-t1339321	GGTTCACAATTGCACATGGG	20	1	FgHV1gp2	-
siRNA-t0495047	GGTTCGAGTTGTATGAAAACT	21	1	FGSG_04089	-
siRNA-t0434147	GGTTCTTGAAGTTGGTGCCGACG	23	1	FgHV1gp2	-
siRNA-t0429685	GGTTGGTAATTGACAAAGGCCT	22	1	FgHV1gp2	-
siRNA-t0527632	GGTTGGTAATTGACAAAGGCCTA	23	1	FgHV1gp2	-
siRNA-t1092262	GGTTTGAATGGTCTGCCTAA	20	1	FGSG_08405	K03657|1|0.0|1811|fgr:FG08405.1|DNA helicase II / ATP-dependent DNA helicase PcrA [EC:3.6.4.12]
siRNA-t1319769	GGTTTGTTCTGAAATTTGATGTT	23	1	FgHV1gp2	-
siRNA-t0962831	GGTTTTCCATATCATGACCATCCAT	25	1	FgHV1gp2	-
siRNA-t0855471	GTAAAGTTATCGTGTCTGACT	21	1	FgHV1gp2	-
siRNA-t0743400	GTAACGGGTCCAGATGTTGTG	21	1	FgHV1gp2	-
siRNA-t0735320	GTAAGCTGCTACTCGAACCGCC	22	1	FgHV1gp2	-
siRNA-t1401712	GTAATCATCATCGTAGTCATCGT	23	1	FGSG_09900	-
siRNA-t0630588	GTAATCATCATCGTAGTCATCGTC	24	1	FGSG_09900	-
siRNA-t1395694	GTAATTGCGGTCCATGGTGG	20	1	FGSG_06420	K02202|1|3e-17|89.7|ssl:SS1G_06051|cyclin-dependent kinase 7 [EC:2.7.11.22 2.7.11.23]
siRNA-t0693975	GTAATTGGTTCTTTAGGTTGAT	22	1	FgHV1gp2	-
siRNA-t1117060	GTACAGCGCGCGAAAATGTC	20	1	FGSG_13008	K09466|1|4e-09|63.2|ppa:PAS_chr4_0487|zinc finger protein ADR1;K11215|4|3e-08|60.5|ncr:NCU00340|transcription factor STE12
siRNA-t1478822	GTACCAACAGCGGAGATATTCACGC	25	1	FGSG_00989	K06036|1|3e-170|597|afm:AFUA_4G03780|[EC:4.2.2.-]
siRNA-t1200425	GTACCTGCAGTCACAACTTG	20	1	FGSG_01790	K00680|1|0.0|2387|afm:AFUA_3G14700|[EC:2.3.1.-];K15419|2|0.0|1077|ani:AN2547.2|emericellamide biosynthesis, highly reducing iterative type I polyketide synthase;K15384|5|0.0|762|afv:AFLA_066840|aspyridone synthetase, hybrid polyketide synthase / nonribosomal peptide synthetase
siRNA-t0616520	GTACCTGCAGTCACAACTTGA	21	1	FGSG_01743	K01895|1|0.0|1310|fgr:FG01743.1|acetyl-CoA synthetase [EC:6.2.1.1]
siRNA-t1045681	GTACGGATCCACATGAGCTTC	21	1	FgHV1gp2	-
siRNA-t1319590	GTACGGATCCACATGAGCTTCAG	23	1	FgHV1gp2	-
siRNA-t0519298	GTACGGATCCACATGAGCTTCAGCT	25	1	FgHV1gp2	-
siRNA-t1181122	GTACGTCCGAATACCATACTTTCC	24	1	FgHV1gp2	-
siRNA-t1410215	GTACGTCCGAATACCATACTTTCCG	25	1	FgHV1gp2	-
siRNA-t0667951	GTACGTGGAAATCTTCAGGCCC	22	1	FgHV1gp2	-
siRNA-t1045807	GTACTGAAGGTGGTGTCATGTGTAA	25	1	FgHV1gp2	-
siRNA-t0490087	GTACTTGCCGCGCGTCTCACTGACT	25	1	FgHV1gp2	-
siRNA-t0769028	GTAGCATGTTCTTCGTGTAGA	21	1	FgHV1gp2	-
siRNA-t1121833	GTAGTAAAGTTATCGTGTCTGACT	24	1	FgHV1gp2	-
siRNA-t0920153	GTAGTCATCGGGAATCATGT	20	1	FGSG_08236	-
siRNA-t1235805	GTATAGGTTATCCGCGGCAG	20	1	FGSG_00742	K13621|1|0.0|1687|fgr:FG00742.1|betaine lipid synthase
siRNA-t0528320	GTATAGTCCCTCAAATGTCTCCT	23	1	FgHV1gp2	-
siRNA-t0532055	GTATCATCTGTGCGACTTCCGC	22	1	FgHV1gp2	-
siRNA-t0449930	GTATCCCATAGTGCCCGGCCGTTT	24	1	FgHV1gp2	-
siRNA-t1414829	GTATTTCTTCTGCAGTAAAAC	21	1	FGSG_13534	-
siRNA-t1433794	GTCAAATCAAGTTGGTATCCCTCCG	25	1	FgHV1gp2	-
siRNA-t1008668	GTCACAACTTGACGGTGTCA	20	1	FGSG_08795	K15418|1|0.0|743|pcs:Pc21g12440|zearalenone synthase, highly reducing iterative type I polyketide synthase;K15419|3|0.0|713|ani:AN2547.2|emericellamide biosynthesis, highly reducing iterative type I polyketide synthase
siRNA-t0635413	GTCACATATCTCGATTTCGCAACC	24	1	FgHV1gp2	-
siRNA-t0843750	GTCACCGCCCCGATTCTTGT	20	1	FgHV1gp2	-
siRNA-t1455944	GTCACCGCCCCGATTCTTGTAAT	23	1	FgHV1gp2	-
siRNA-t0785811	GTCAGACCCCAGATCCACCCGG	22	1	FgHV1gp2	-
siRNA-t1432680	GTCAGACCCCAGATCCACCCGGA	23	1	FgHV1gp2	-
siRNA-t0796898	GTCAGACCCCAGATCCACCCGGAT	24	1	FgHV1gp2	-
siRNA-t0792049	GTCAGGGTACGTGACATTAGG	21	1	FgHV1gp2	-
siRNA-t0694097	GTCAGTGTCAGGATCCGTCCA	21	1	FGSG_05487	K14318|1|2e-25|117|cci:CC1G_01275|nuclear pore complex protein Nup88
siRNA-t1021299	GTCATACCTCACCACATCAG	20	1	FGSG_13878	K01932|1|0.0|1450|afm:AFUA_5G12730|[EC:6.3.2.-]
siRNA-t0771280	GTCATTGTTCATCTGCACTCCTTT	24	1	FgHV1gp2	-
siRNA-t0391824	GTCATTTTTGCACCAGGATTT	21	1	FGSG_09980	-
siRNA-t0370904	GTCCAAGCCACCAGTGGGAAGT	22	1	FGSG_00428	K10415|1|0.0|1226|fgr:FG00428.1|dynein intermediate chain, cytosolic
siRNA-t0570544	GTCCCGCTGTTTCACTTGTAGGAGT	25	1	FgHV1gp2	-
siRNA-t0401458	GTCCGGAATGCGTTCACACGA	21	1	FgHV1gp2	-
siRNA-t0503405	GTCGAATGAAATGGAACAACGTG	23	1	FgHV1gp2	-
siRNA-t0608220	GTCGAGGATGCAAACTTCTT	20	1	FGSG_07360	-
siRNA-t0584130	GTCGCAGTTGGAATATATCAG	21	1	FGSG_01144	-
siRNA-t0739116	GTCGCATCAGCCATCATGTATA	22	1	FgHV1gp2	-
siRNA-t0389956	GTCGGCACCAACTTCAAGAAC	21	1	FGSG_04588	K15321|1|1e-123|444|aor:AOR_1_898134|naphtho-gamma-pyrone polyketide synthase
siRNA-t0640577	GTCGTTAGAAGCTTGACACGAA	22	1	FgHV1gp2	-
siRNA-t0494075	GTCTATTATATGTCATGCGTGCT	23	1	FgHV1gp2	-
siRNA-t0510383	GTCTCCGAACGTGATCTCATCAT	23	1	FgHV1gp2	-
siRNA-t1322015	GTCTTCGTCCAGTAGCCAATGAG	23	1	FgHV1gp2	-
siRNA-t0376145	GTCTTGCGGTGTGGGTCTTCTGACG	25	1	FgHV1gp2	-
siRNA-t0584596	GTGAACCCGATTGCGTTGGT	20	1	FGSG_10062	K08968|1|4e-103|372|fgr:FG10062.1|L-methionine (R)-S-oxide reductase [EC:1.8.4.14]
siRNA-t1286453	GTGAATTTCTCCAGTAGCATGT	22	1	FGSG_07168	-
siRNA-t1389371	GTGAATTTCTCCAGTAGCATGTTCT	25	1	FgHV1gp2	-
siRNA-t1061863	GTGACAAAAGCATGGCGATGCA	22	1	FgHV1gp2	-
siRNA-t0988222	GTGACAAAAGCATGGCGATGCACTT	25	1	FgHV1gp2	-

siRNA-t0657424	GTGACCAGCTGTTCTCTGTC	20	1	FGSG_11026	K01932|1|0.0|1527|afm:AFUA_1G17200|[EC:6.3.2.-]
siRNA-t1285863	GTGACGAAGATCCCACTCTA	20	1	FGSG_13783	K01932|1|0.0|1526|afm:AFUA_5G12730|[EC:6.3.2.-]
siRNA-t0978272	GTGACGAAGATCCCACTCTAGCAA	24	1	FgHV1gp1	-
siRNA-t1002363	GTGACGAGGCCTCTGACGTTGATC	24	1	FgHV1gp2	-
siRNA-t1024955	GTGAGAAGATTGAGTTCATT	20	1	FGSG_08247	-
siRNA-t1190887	GTGAGAAGATTGAGTTCATTTGT	23	1	FgHV1gp2	-
siRNA-t0627604	GTGAGAAGATTGAGTTCATTTGTTC	25	1	FgHV1gp2	-
siRNA-t1064758	GTGAGGTATGACGTGTTCATT	21	1	FgHV1gp2	-
siRNA-t1230216	GTGATACAAACCGGAATGCAC	21	1	FgHV1gp2	-
siRNA-t1042524	GTGATAGATCTTATTGCCAGGTA	23	1	FgHV1gp2	-
siRNA-t0485810	GTGATAGCCTCTCCCTTCGT	20	1	FgHV1gp2	-
siRNA-t0791064	GTGATGCATTGCTAGAGTGG	20	1	FGSG_10561	K01183|1|0.0|635|afm:AFUA_5G03960|chitinase [EC:3.2.1.14]
siRNA-t0456795	GTGATGGTACCATTGCTGCA	20	1	FGSG_05452	-
siRNA-t1261896	GTGCCAATGTTTGTGCCAAGA	21	1	FGSG_09851	K01509|1|2e-109|396|pic:PICST_86369|adenosinetriphosphatase [EC:3.6.1.3];K12196|3|5e-59|228|cgr:CAGL0I06402g|vacuolar protein-sorting-associated protein 4
siRNA-t0504736	GTGCCAATGTTTGTGCCAAGAG	22	1	FGSG_09851	K01509|1|2e-109|396|pic:PICST_86369|adenosinetriphosphatase [EC:3.6.1.3];K12196|3|5e-59|228|cgr:CAGL0I06402g|vacuolar protein-sorting-associated protein 4
siRNA-t1087992	GTGCCCAAAGCGTTTAGTCT	20	1	FGSG_11006	K01238|1|9e-135|480|afm:AFUA_4G03350|[EC:3.2.1.-];K01210|2|8e-93|340|afm:AFUA_2G00430|glucan 1,3-beta-glucosidase [EC:3.2.1.58]
siRNA-t1436279	GTGCCCGGCCGTTTGTGCTGCA	22	1	FgHV1gp2	-
siRNA-t0874546	GTGCGACGACCGTGCGAAAT	20	1	FGSG_13129	-
siRNA-t1206473	GTGCGACGACCGTGCGAAATTGTTT	25	1	FgHV1gp2	-
siRNA-t0648296	GTGCGCAAATGGGTATCACCA	21	1	FGSG_07150	K00924|1|6e-144|510|afm:AFUA_5G12870|[EC:2.7.1.-];K00858|2|8e-86|317|afm:AFUA_2G01350|NAD+ kinase [EC:2.7.1.23]
siRNA-t1003931	GTGCGCTTCTGGATCAATGTCAG	23	1	FgHV1gp2	-
siRNA-t0571570	GTGCTCATGAGGTTGGCATTG	21	1	FGSG_04717	K00517|1|1e-80|299|afm:AFUA_2G18010|[EC:1.14.-.-];K15388|4|1e-12|73.6|ani:AN8411.2|cytochrome P450 monooxygenase;K05917|5|5e-12|71.6|dha:DEHA2E19272g|cytochrome P450, family 51 (sterol 14-demethylase) [EC:1.14.13.70]
siRNA-t0394798	GTGCTGAATGCTCTGCCTCGG	21	1	FgHV1gp2	-
siRNA-t1000086	GTGGAAATCTTCAGGCCCTGG	21	1	FgHV1gp2	-
siRNA-t1044740	GTGGAGTTCCTTCGGAAAGT	20	1	FGSG_10722	K01870|1|0.0|2196|fgr:FG10722.1|isoleucyl-tRNA synthetase [EC:6.1.1.5]
siRNA-t1353225	GTGGCCGTAAGTAGCAGATTTT	22	1	FgHV1gp2	-
siRNA-t0904894	GTGGCCGTAAGTAGCAGATTTTAC	24	1	FgHV1gp2	-
siRNA-t0461766	GTGGCGCATTTCTTTCTTACCCTC	24	1	FgHV1gp2	-
siRNA-t0850563	GTGGCGCATTTCTTTCTTACCCTCG	25	1	FgHV1gp2	-
siRNA-t0483617	GTGGCGGGACCAGGATTCTTCT	22	1	FGSG_08844	K01183|1|1e-06|55.1|pan:PODANSg4492|chitinase [EC:3.2.1.14]
siRNA-t1008328	GTGGGAGCGAAAGCGATATC	20	1	FGSG_06689	K00587|1|3e-144|509|fgr:FG06689.1|protein-S-isoprenylcysteine O-methyltransferase [EC:2.1.1.100]
siRNA-t0648453	GTGGGATGGGTTTCTGGTTC	20	1	FGSG_05683	K00485|1|5e-159|560|mgr:MGG_03330|dimethylaniline monooxygenase (N-oxide forming) [EC:1.14.13.8]
siRNA-t0854838	GTGGGGTCACATATCTCGATTTCG	24	1	FgHV1gp2	-
siRNA-t0789093	GTGGGGTGTGTATCCAATGAGTTG	24	1	FgHV1gp2	-
siRNA-t0788101	GTGGGTCTTCTGACGAATTT	20	1	FGSG_00956	-
siRNA-t0854816	GTGGGTTCTTGAAGTTGGTGCC	22	1	FgHV1gp2	-
siRNA-t1442026	GTGGGTTCTTGAAGTTGGTGCCGAC	25	1	FgHV1gp2	-
siRNA-t1386559	GTGGTTCATTGCGATGTAATCTT	23	1	FgHV1gp2	-
siRNA-t1436221	GTGTCAAGATTGGATTTCTTCCTC	24	1	FgHV1gp2	-
siRNA-t1163414	GTGTCACCGCCCCGATTCTTGT	22	1	FgHV1gp2	-
siRNA-t0670082	GTGTCGCGTCTACCTTGTTCA	21	1	FgHV1gp2	-
siRNA-t0989800	GTGTCGCGTCTACCTTGTTCAGGA	24	1	FgHV1gp2	-
siRNA-t0993325	GTGTCGCGTCTACCTTGTTCAGGAA	25	1	FgHV1gp2	-
siRNA-t0399855	GTGTCGTAGGAAAGGAAAAG	20	1	FGSG_13645	-
siRNA-t0509181	GTGTCGTTAGAAGCTTGACAC	21	1	FgHV1gp2	-
siRNA-t1284556	GTGTCTGACTGTTGCTTGAT	20	1	FgHV1gp2	-
siRNA-t0690509	GTGTGGCGGGACCAGGATTCTTC	23	1	FgHV1gp2	-
siRNA-t1197764	GTGTGGGTCTTCTGACGAATTTGG	24	1	FgHV1gp2	-
siRNA-t1090490	GTGTGTCGCGTCTACCTTGTTCAG	24	1	FgHV1gp2	-
siRNA-t1184334	TAAGTTACAGGCCTCATTGA	20	1	FgHV1gp2	-
siRNA-t0404839	TAAGTTACAGGCCTCATTGAT	21	1	FgHV1gp2	-
siRNA-t0631006	TAATATCGTCATTCTCTTCCAGTGT	25	1	FgHV1gp2	-
siRNA-t1038739	TAATCATCATCGTAGTCATCGT	22	1	FGSG_13700	-
siRNA-t0377899	TAATCTTTCCTCTTGATGCGCAC	23	1	FgHV1gp2	-
siRNA-t0611750	TAATCTTTCCTCTTGATGCGCACCA	25	1	FgHV1gp2	-
siRNA-t0635014	TAATGGAGGACGATCTCATTCGT	23	1	FgHV1gp2	-
siRNA-t1288681	TAATTCCGGGATCAGGTTCAC	21	1	FGSG_08396	K01443|1|0.0|921|fgr:FG08396.1|N-acetylglucosamine-6-phosphate deacetylase [EC:3.5.1.25]
siRNA-t1261616	TAATTGGTTCTTTAGGTTGATT	22	1	FgHV1gp2	-
siRNA-t1252774	TAATTGTACATCATTCCATTG	21	1	FgHV1gp2	-
siRNA-t0755834	TACAAACCGGAATGCACTTT	20	1	FgHV1gp2	-
siRNA-t1387359	TACAACATCAAATTTCAGAA	20	1	FGSG_06971	K09272|1|0.0|856|mgr:MGG_11344|structure-specific recognition protein 1
siRNA-t0990204	TACAATCCTTACCCAAGATTT	21	1	FgHV1gp2	-
siRNA-t1018536	TACAATCGTGGACTTCCTTTAGG	23	1	FgHV1gp2	-
siRNA-t1147050	TACACGACGTACTTAGGGTTGTT	23	1	FgHV1gp2	-
siRNA-t0559137	TACACTGCCACTCCTACAAG	20	1	FGSG_03197	-
siRNA-t1083849	TACAGGACCGCGCGATGGCGCG	22	1	FgHV1gp2	-
siRNA-t0630691	TACAGGCCTCATTGATTTTG	20	1	FgHV1gp2	-
siRNA-t1053226	TACAGGCCTCATTGATTTTGTGT	23	1	FgHV1gp2	-
siRNA-t0459648	TACATGATGGCTGATGCGACCG	22	1	FGSG_03527	-
siRNA-t1100280	TACATGCCTGATGTTCTTGTG	21	1	FgHV1gp2	-
siRNA-t0741952	TACATTTCATTGGCATAGAA	20	1	FGSG_05642	-
siRNA-t0954345	TACCACAAAACTTCTGTCCA	20	1	FGSG_12624	K00777|1|3e-30|133|afm:AFUA_7G07100|[EC:2.4.2.-];K14855|2|7e-15|82.4|lbc:LACBIDRAFT_296068|ribosome assembly protein 4;K06666|3|6e-10|66.2|cal:Ca019.13528|glucose repression regulatory protein TUP1
siRNA-t0617336	TACCACATTGATGAGGCTTTCAG	23	1	FGSG_01212	K02605|1|0.0|1358|fgr:FG01212.1|origin recognition complex subunit 3
siRNA-t0380374	TACCAGAACCCAGCACCTTT	20	1	FgHV1gp2	-
siRNA-t0462925	TACCAGACATTTTCGCGCGC	20	1	FGSG_10326	-
siRNA-t0404195	TACCATTCACGAACGCTAAGAG	22	1	FgHV1gp2	-
siRNA-t1249273	TACCATTCACGAACGCTAAGAGGA	24	1	FgHV1gp2	-
siRNA-t0645473	TACCCATTTGCGCACATATCTCCT	24	1	FgHV1gp2	-
siRNA-t1326552	TACCGCAGTGATGGAGACTAAAC	23	1	FGSG_09869	K12669|1|0.0|647|fgr:FG09869.1|oligosaccharyltransferase complex subunit gamma
siRNA-t0427035	TACCTGCAGTCACAACTTGAC	21	1	FGSG_01743	K01895|1|0.0|1310|fgr:FG01743.1|acetyl-CoA synthetase [EC:6.2.1.1]
siRNA-t0401883	TACCTTTGAGCTCCTTGAGGA	21	1	FGSG_01693	-
siRNA-t0658026	TACCTTTGAGCTCCTTGAGGAA	22	1	FgHV1gp2	-
siRNA-t0774331	TACCTTTGAGCTCCTTGAGGAAT	23	1	FgHV1gp2	-
siRNA-t1235282	TACGCCCTTCTAAACACTTT	20	1	FGSG_08795	K15418|1|0.0|743|pcs:Pc21g12440|zearalenone synthase, highly reducing iterative type I polyketide synthase;K15419|3|0.0|713|ani:AN2547.2|emericellamide biosynthesis, highly reducing iterative type I polyketide synthase
siRNA-t0815640	TACGGATCCACATGAGCTTCAGCT	24	1	FgHV1gp2	-
siRNA-t0477708	TACGTCCGAATACCATACTT	20	1	FgHV1gp2	-
siRNA-t0757594	TACGTCCGAATACCATACTTT	21	1	FgHV1gp2	-
siRNA-t1395317	TACGTCCGAATACCATACTTTCCG	24	1	FgHV1gp2	-
siRNA-t0475048	TACGTGGAAATCTTCAGGCCC	21	1	FgHV1gp2	-

siRNA-t1336996	TACGTGGAAATCTTCAGGCCCTG	23	1	FgHV1gp2	-
siRNA-t1283511	TACGTGGAAATCTTCAGGCCCTGGG	25	1	FgHV1gp2	-
siRNA-t0896831	TACTCCTGACCCTATCAATC	20	1	FGSG_13493	-
siRNA-t0849348	TACTTACGGCCACTTTGCAC	20	1	FGSG_12947	-
siRNA-t1489579	TAGAACTTTTGCCACTGGGA	20	1	FGSG_07715	K12584|1|3e-39|161|ure:UREG_00738|scavenger mRNA-decapping enzyme DcpS [EC:3.-.-.-]
siRNA-t1020930	TAGAAGCTTGACACGAATGA	20	1	FGSG_04030	-
siRNA-t1000845	TAGACAGCATTCTTTTGAAA	20	1	FGSG_12703	-
siRNA-t1132390	TAGATGTGATGCCTTGTCTA	20	1	FGSG_05514	K05841|1|1e-72|274|sce:YLR189C|sterol 3beta-glucosyltransferase [EC:2.4.1.173]
siRNA-t1408364	TAGATGTGATGCCTTGTCTATT	22	1	FGSG_05514	K05841|1|1e-72|274|sce:YLR189C|sterol 3beta-glucosyltransferase [EC:2.4.1.173]
siRNA-t0402369	TAGCATGTTCTTCGTGTAGAA	21	1	FGSG_01497	K05767|1|0.0|3219|fgr:FG01497.1|IQ motif containing GTPase activating protein
siRNA-t0716430	TAGCATGTTCTTCGTGTAGAAA	22	1	FgHV1gp2	-
siRNA-t0805787	TAGCCATTCGTTACAATCGT	20	1	FgHV1gp2	-
siRNA-t1099299	TAGCGCCCAGGATTTCAAGTC	21	1	FGSG_10882	K14571|1|0.0|1543|fgr:FG10882.1|ribosome biogenesis ATPase
siRNA-t0375887	TAGGACAAGCCGTCAAACATA	21	1	FGSG_00541	K05658|1|0.0|659|cci:CC1G_03025|ATP-binding cassette, subfamily B (MDR/TAP), member 1
siRNA-t0420774	TAGGACAAGCCGTCAAACATAG	22	1	FgHV1gp2	-
siRNA-t1097786	TAGGACCAGTTCCTGCTGAGT	21	1	FgHV1gp2	-
siRNA-t0940052	TAGGTCTGGCATAGGAAGGTT	21	1	FgHV1gp2	-
siRNA-t1395441	TAGTCATCGGGAATCATGTCGGTG	24	1	FgHV1gp2	-
siRNA-t1132588	TAGTGCCCGGCCGTTTGTGCTGC	23	1	FgHV1gp2	-
siRNA-t0726776	TAGTGCCCGTCAATATTGAT	20	1	FGSG_09146	K00777|1|3e-17|90.9|afm:AFUA_7G07100|[EC:2.4.2.-];K06653|3|7e-12|72.8|vpo:Kpol_1010p59|CDK inhibitor PHO81;K06694|4|2e-11|71.2|pno:SNOG_06697|26S proteasome non-ATPase regulatory subunit 10;K08803|5|2e-11|71.2|fgr:FG07816.1|death-associated protein kinase [EC:2.7.11.1]
siRNA-t1275714	TATAGATCGCGCGTAAGCTT	20	1	FgHV1gp2	-
siRNA-t0711637	TATAGGAGTACCAACAGCGG	20	1	FGSG_08853	-
siRNA-t0468053	TATCCGGCTCCCTTTCGGGCC	21	1	FGSG_01266	K11236|1|6e-07|56.2|mgl:MGL_0960|cell division control protein 24
siRNA-t0906518	TATCGCTTTCGCTCCCACTA	20	1	FGSG_04129	-
siRNA-t1098159	TATCGCTTTCGCTCCCACTAT	21	1	FGSG_04129	-
siRNA-t1466806	TATCGTCATTCTCTTCCAGT	20	1	FGSG_10105	K12864|1|8e-17|88.2|cne:CNH03260|beta-catenin-like protein 1
siRNA-t0650603	TATCGTCCTGCGTGATTGCC	20	1	FGSG_11389	K00517|1|0.0|734|afm:AFUA_8G00760|[EC:1.14.-.-];K10437|3|8e-44|177|uma:UM01424.1|phenylacetate 2-hydroxylase [EC:1.14.13.-];K10438|4|6e-43|174|fgr:FG06447.1|3-hydroxyphenylacetate 6-hydroxylase [EC:1.14.13.63]
siRNA-t0943639	TATCGTGCGCTTCTGGATCAATGT	24	1	FgHV1gp2	-
siRNA-t0457348	TATGACGATTTGGCCAAATTT	21	1	FGSG_10980	-
siRNA-t1092256	TATGCCCGCGAGCTTTGCTC	20	1	FGSG_09629	K12848|1|6e-88|322|fgr:FG09629.1|U4/U6.U5 tri-snRNP component SNU23
siRNA-t0565737	TATGGTAATTGCGGTCCATG	20	1	FGSG_07585	-
siRNA-t1338320	TATGTGGAGTTCCTTCGGAAA	21	1	FGSG_04291	K02999|1|0.0|3375|fgr:FG04291.1|DNA-directed RNA polymerase I subunit RPA1 [EC:2.7.7.6]
siRNA-t0833590	TATTCCAACTGCGACCCATGCT	22	1	FgHV1gp2	-
siRNA-t1418120	TATTGATATTAGCCCCCTGT	20	1	FgHV1gp2	-
siRNA-t0776467	TATTGATATTAGCCCCCTGTCTC	23	1	FgHV1gp2	-
siRNA-t0654789	TCAAATCCAAAAGACGCGTG	20	1	FgHV1gp2	-
siRNA-t0416453	TCAAATGTCTCCTTCGGTTC	20	1	FGSG_00678	-
siRNA-t1097074	TCAACGGTTCTTGGAAAGTTGGAGC	25	1	FgHV1gp2	-
siRNA-t1480146	TCAAGATTGGATTTCTTCCTCAA	23	1	FgHV1gp2	-
siRNA-t1014577	TCAAGGACGTTCTGGTCTTCGAT	23	1	FGSG_09801	K03164|1|0.0|2817|fgr:FG09801.1|DNA topoisomerase II [EC:5.99.1.3]
siRNA-t0622698	TCAAGGTCAGAGAGAACTTCT	21	1	FGSG_12863	K02324|1|0.0|3986|fgr:FG06065.1|DNA polymerase epsilon subunit 1 [EC:2.7.7.7]
siRNA-t0805132	TCAAGTCTCTTGTTCTGGAACG	22	1	FGSG_02381	-
siRNA-t0821334	TCAAGTTGGTATCCCTCCGT	20	1	FgHV1gp2	-
siRNA-t0565201	TCAATCCATCGCCACCCAGA	20	1	FGSG_12704	K05658|1|0.0|1949|nhe:NECHADRAFT_96330|ATP-binding cassette, subfamily B (MDR/TAP), member 1
siRNA-t0720092	TCAATCCTGCTCGCACCTTTC	21	1	FGSG_06699	-
siRNA-t0774274	TCAATGATGGCACGTCGACAGG	22	1	FgHV1gp2	-
siRNA-t1221073	TCAATGATGGCACGTCGACAGGCG	24	1	FgHV1gp2	-
siRNA-t1446590	TCAATGATGGCACGTCGACAGGCGT	25	1	FgHV1gp2	-
siRNA-t0650904	TCAATGTACCGTTCGGCAAC	20	1	FGSG_03931	K13336|1|2e-47|189|afv:AFLA_048740|peroxin-3;K03448|2|8e-40|164|ani:AN2663.2|MFS transporter, ACS family, pantothenate transporter
siRNA-t1431676	TCAATGTACCGTTCGGCAACAG	22	1	FGSG_00259	-
siRNA-t0838162	TCATGGGCAAAAGACAGATAGGTCG	25	1	FgHV1gp2	-
siRNA-t1156749	TCATTCGTGTCAAGCTTCTAA	21	1	FGSG_03747	K01932|1|0.0|677|afm:AFUA_1G10380|[EC:6.3.2.-]
siRNA-t0908708	TCATTCTCTTCCAGTGTCGAACCA	24	1	FgHV1gp2	-
siRNA-t0391190	TCATTCTCTTCCAGTGTCGAACCAT	25	1	FgHV1gp2	-
siRNA-t0709798	TCATTTCGGCGAGTCTAATCTTC	23	1	FgHV1gp2	-
siRNA-t1454568	TCATTTTTGCACCAGGATTTGCTTC	25	1	FgHV1gp2	-
siRNA-t0787123	TCCAAGTCGCTCTTTGTGAACCC	23	1	FgHV1gp2	-
siRNA-t1270158	TCCAATGTAAGTGACGAGGCCTCT	24	1	FgHV1gp2	-
siRNA-t0415500	TCCAATGTAAGTGACGAGGCCTCTG	25	1	FgHV1gp2	-
siRNA-t0871388	TCCACACCGCGTTTCGCTTGCCA	23	1	FgHV1gp1	-
siRNA-t1287250	TCCACCCGGATGACTTGGCTCTAG	24	1	FgHV1gp2	-
siRNA-t1348478	TCCACCTCGTATTTTGAACTC	21	1	FgHV1gp2	-
siRNA-t1196584	TCCACGTCATGATCATCAGC	20	1	FGSG_10904	K06681|1|1e-61|238|cdu:CD36_21160|Gdp/GTP exchange factor required for growth at low temperatures
siRNA-t0625859	TCCAGACCCATTCGTGTTCCCG	22	1	FgHV1gp2	-
siRNA-t1085904	TCCAGACCCATTCGTGTTCCCGA	23	1	FgHV1gp2	-
siRNA-t0833698	TCCAGATGTTGTGCTTTTTC	20	1	FGSG_08236	-
siRNA-t0617100	TCCAGTGTCACCGCCCCGATTCT	23	1	FgHV1gp2	-
siRNA-t1001576	TCCAGTGTCGAACCATACTGGACCC	25	1	FgHV1gp2	-
siRNA-t0747184	TCCATATCATGACCATCCAT	20	1	FgHV1gp2	-
siRNA-t0668064	TCCATCCCCTTACCTTTGCGCT	22	1	FGSG_01301	-
siRNA-t0688851	TCCATCGCCACCCAGATTTGTC	22	1	FGSG_00055	-
siRNA-t1308837	TCCATCTTGCAGCACAAACGGCC	23	1	FGSG_05511	K12041|1|0.0|1103|fgr:FG05511.1|solute carrier family 9 (sodium/hydrogen exchanger), member 6/7
siRNA-t1070666	TCCATGGTGGGGTCACATATCTC	23	1	FGSG_00910	-
siRNA-t1043705	TCCCACTCTAGCAATGCATC	20	1	FgHV1gp1	-
siRNA-t1424642	TCCCACTCTAGCAATGCATCACCAA	25	1	FgHV1gp1	-
siRNA-t0559840	TCCCATAGTGCCCGGCCGTTTGTG	24	1	FgHV1gp2	-
siRNA-t1140567	TCCCGGCAGCGTTTATGGAAC	21	1	FGSG_12361	-
siRNA-t0748135	TCCCTATATAATTTGGCCCCAGC	23	1	FgHV1gp2	-
siRNA-t1353032	TCCCTTTCTTCCCTCATTCTTCTCT	25	1	FgHV1gp2	-
siRNA-t0598126	TCCGAAGGAACTCCACATAGC	21	1	FgHV1gp2	-
siRNA-t1330849	TCCGAATACCATACTTTCCGA	21	1	FgHV1gp2	-
siRNA-t1174207	TCCGACAATGGCGCATACACG	21	1	FgHV1gp2	-
siRNA-t0690060	TCCGAGCACCATACAATGCCGC	22	1	FgHV1gp2	-
siRNA-t1364153	TCCGCAGAAGAATTAGCCTTTG	22	1	FgHV1gp2	-
siRNA-t0603449	TCCGCCTCCTTCGAACTAGATGG	23	1	FgHV1gp2	-
siRNA-t1279193	TCCGCCTCCTTCGAACTAGATGGT	24	1	FgHV1gp2	-
siRNA-t1020671	TCCGCGTGTGATTGGTCTCTTGCA	24	1	FgHV1gp2	-
siRNA-t1134874	TCCGGAATGCGTTCACACGAT	21	1	FgHV1gp2	-

siRNA-t1080562	TCCGGGATCAGGTTCACAATTG	22	1	FGSG_08396	K01443|1|0.0|921|fgr:FG08396.1|N-acetylglucosamine-6-phosphate deacetylase [EC:3.5.1.25]
siRNA-t1461353	TCCTAGAGATCTCCGCAGAAG	21	1	FGSG_00352	K08064|1|2e-78|291|mgr:MGG_00354|nuclear transcription factor Y, alpha
siRNA-t0837315	TCCTAGAGATCTCCGCAGAAGA	22	1	FGSG_00352	K08064|1|2e-78|291|mgr:MGG_00354|nuclear transcription factor Y, alpha
siRNA-t1435225	TCCTAGCAAGTGAAACTGTT	20	1	FgHV1gp2	-
siRNA-t1056282	TCCTAGCAAGTGAAACTGTTC	21	1	FgHV1gp2	-
siRNA-t0681105	TCCTATAGATCGCGCGTAAGCTTA	24	1	FgHV1gp2	-
siRNA-t1044851	TCCTATGCCAGACCTACATCC	21	1	FGSG_01645	K15731|1|7e-15|83.2|ani:AN0425.2|carboxy-terminal domain RNA polymerase II polypeptide A small phosphatase [EC:3.1.3.16];K01768|4|1e-14|82.4|cim:CIMG_01098|adenylate cyclase [EC:4.6.1.1]
siRNA-t0486588	TCCTCCTTCCGTAATCATCA	20	1	FGSG_02536	K12856|1|0.0|4746|fgr:FG02536.1|pre-mRNA-processing factor 8
siRNA-t1217293	TCCTCTCCCTCATACTTCGCC	21	1	FGSG_01251	K01175|1|3e-63|243|afm:AFUA_7G01550|[EC:3.1.-.-]
siRNA-t1133522	TCCTCTGATATATTCCAACTGCGA	24	1	FgHV1gp2	-
siRNA-t0771605	TCCTGCTCTCTGATTTTCAAC	21	1	FGSG_05188	K15322|1|2e-51|203|ani:AN8255.2|tRNA-splicing endonuclease subunit Sen2 [EC:3.1.27.9];K06661|2|3e-47|190|afm:AFUA_5G04020|DNA repair protein RAD9;K00547|3|6e-43|176|cim:CIMG_06640|homocysteine S-methyltransferase [EC:2.1.1.10]
siRNA-t0939898	TCCTGGTCCCGCCACACCATC	21	1	FGSG_12025	-
siRNA-t1208738	TCCTTCCGTAATCATCATCGTAGTC	25	1	FgHV1gp2	-
siRNA-t1330115	TCCTTCGAACTAGATGGTGTG	21	1	FgHV1gp2	-
siRNA-t0742686	TCCTTCGGTAATCACAGGGCAA	22	1	FgHV1gp2	-
siRNA-t0847283	TCCTTCGTTGAATCGATCGT	20	1	FGSG_03642	-
siRNA-t0920602	TCCTTCGTTGAATCGATCGTCATG	24	1	FgHV1gp2	-
siRNA-t0976694	TCCTTGGCTTTGGTGCATTGA	21	1	FGSG_08738	K07566|1|0.0|667|ani:AN6079.2|tRNA threonylcarbamoyladenosine biosynthesis protein
siRNA-t1306563	TCGAAAGTGAAAGTGTCGTAGG	22	1	FgHV1gp1	-
siRNA-t0807105	TCGACAATGAAAGATCCTAAA	21	1	FgHV1gp2	-
siRNA-t0770868	TCGACGTGCCATCATTGAAA	20	1	FGSG_09894	K15541|1|2e-29|128|aor:AOR_1_1332164|protein MPE1
siRNA-t0679038	TGAGCAAGTTCGTTTAGCTC	20	1	FGSG_10267	K00288|1|0.0|1759|fgr:FG10267.1|methylenetetrahydrofolate dehydrogenase (NADP+) / methenyltetrahydrofolate cyclohydrolase / formyltetrahydrofolate synthetase [EC:1.5.1.5 3.5.4.9 6.3.4.3]
siRNA-t0549794	TGAGCAAGTTCGTTTAGCTCGAT	23	1	FGSG_10267	K00288|1|0.0|1759|fgr:FG10267.1|methylenetetrahydrofolate dehydrogenase (NADP+) / methenyltetrahydrofolate cyclohydrolase / formyltetrahydrofolate synthetase [EC:1.5.1.5 3.5.4.9 6.3.4.3]
siRNA-t0728922	TGAGCACTTGTTTGCCTGCAT	21	1	FgHV1gp2	-
siRNA-t0806930	TGAGCACTTGTTTGCCTGCATCGA	24	1	FgHV1gp2	-
siRNA-t1483973	TGAGCTCTGTGACTCCAGCCAAAG	24	1	FgHV1gp2	-
siRNA-t1188703	TGAGGCCAAAGGTGTTCAGCTT	22	1	FGSG_08098	K01066|1|2e-53|208|afm:AFUA_4G07720|esterase / lipase [EC:3.1.1.-];K01046|2|3e-30|132|afm:AFUA_8G02530|triacylglycerol lipase [EC:3.1.1.3]
siRNA-t1434574	TGAGGTATGACGTGTTCATTA	21	1	FgHV1gp2	-
siRNA-t1225631	TGAGGTTTCCTCGTGCTTTA	20	1	FGSG_06939	K08286|1|0.0|709|mgr:MGG_06420|protein-serine/threonine kinase [EC:2.7.11.-]
siRNA-t0953560	TGATAGATCTTATTGCCAGGTAG	23	1	FgHV1gp2	-
siRNA-t0564088	TGATAGATCTTATTGCCAGGTAGA	24	1	FgHV1gp2	-
siRNA-t0403422	TGATAGCCTCTCCCTTCGTGA	21	1	FGSG_00363	K11137|1|0.0|1883|fgr:FG00363.1|telomere length regulation protein
siRNA-t0810449	TGATAGCCTCTCCCTTCGTGAA	22	1	FgHV1gp2	-
siRNA-t0788214	TGATCATGGTACGGTCATCC	20	1	FGSG_07258	K14572|1|0.0|8396|fgr:FG07258.1|midasin
siRNA-t1058886	TGATCCATGCCAAACCAACT	20	1	FGSG_07498	K01098|1|4e-18|92.8|cal:CaO19.10140|polynucleotide 5'-phosphatase [EC:3.1.3.33]
siRNA-t1477592	TGATCCTTCCACTTCCCTGG	20	1	FGSG_10042	K11231|1|0.0|2053|fgr:FG10042.1|osomolarity two-component system, sensor histidine kinase SLN1 [EC:2.7.13.3]
siRNA-t1419915	TGATCTGGCTCGTGACTTTTC	21	1	FgHV1gp2	-
siRNA-t0682274	TGATCTGGCTCGTGACTTTTCC	22	1	FGSG_09358	-
siRNA-t1203497	TGATGGCACGTCGACAGGCGT	21	1	FgHV1gp2	-
siRNA-t1321206	TGATGGCTTCCGTCCCTTGA	20	1	FGSG_13549	-
siRNA-t0676004	TGATGTTCTTGTGCCTCCGAG	21	1	FGSG_10734	-
siRNA-t1365289	TGATGTTCTTGTGCCTCCGAGCA	23	1	FgHV1gp2	-
siRNA-t0461941	TGATTGATTCTAGCTTGTGCAAA	23	1	FgHV1gp2	-
siRNA-t1098489	TGATTGGTCTCTTGCAGTGCA	21	1	FgHV1gp2	-
siRNA-t1193359	TGATTTGACGTTCCAGAACA	20	1	FGSG_07450	K12385|1|5e-14|79.7|yli:YALI0C20757g|Niemann-Pick C1 protein
siRNA-t1150011	TGATTTGCGACGTGCTTGAAGG	22	1	FGSG_08200	-
siRNA-t0681222	TGATTTTGTGTCGCCTCCAG	20	1	FgHV1gp2	-
siRNA-t1342700	TGATTTTGTGTCGCCTCCAGAC	22	1	FgHV1gp2	-
siRNA-t1274456	TGCACCAAAGCCAAGGATAAA	21	1	FGSG_03360	K10866|1|9e-49|194|fgr:FG00711.1|DNA repair protein RAD50 [EC:3.6.-.-];K13095|3|1e-25|117|fgr:FG02080.1|splicing factor 1;K01952|4|5e-22|105|pno:SNOG_10851|phosphoribosylformylglycinamidine synthase [EC:6.3.5.3];K08197|5|5e-11|68.6|fgr:FG05848.1|MFS transporter, SIT family, siderophore-iron:H+ symporter
siRNA-t1274222	TGCAGCGTGAACCAGATCATAA	22	1	FgHV1gp2	-
siRNA-t1396237	TGCAGCGTGAACCAGATCATAAC	23	1	FgHV1gp2	-
siRNA-t0737886	TGCAGTTGGTCCGCGAATTCTGCCA	25	1	FgHV1gp2	-
siRNA-t0741227	TGCATCACCAAGCCAAGCGGG	21	1	FGSG_09875	K13026|1|2e-90|333|ncr:NCU01143|ATP-dependent RNA helicase DHX57 [EC:3.6.4.13];K02974|4|3e-69|263|cci:CC1G_06717|small subunit ribosomal protein S24e;K12813|5|9e-55|215|cci:CC1G_02978|pre-mRNA-splicing factor ATP-dependent RNA helicase DHX16 [EC:3.6.4.13]
siRNA-t1208879	TGCATCCTCGACCCACTCAT	20	1	FGSG_03964	K15316|1|3e-170|599|ani:AN7825.2|sterigmatocystin polyketide synthase;K15321|4|6e-165|582|nfi:NFIA_093000|naphtho-gamma-pyrone polyketide synthase
siRNA-t0526871	TGCATCCTCGACCCACTCATGGGCA	25	1	FgHV1gp2	-
siRNA-t0515137	TGCATGTACTGTTCGGCAAC	20	1	FGSG_04886	K00309|1|6e-94|343|afm:AFUA_6G08760|[EC:1.5.3.-];K00318|4|2e-35|149|pgu:PGUG_01270|proline dehydrogenase [EC:1.5.99.8]
siRNA-t1487317	TGCCAGCATAAGCCATCGCA	20	1	FGSG_00232	-
siRNA-t1253760	TGCCAGGTAGATGACCGACAAC	22	1	FgHV1gp2	-
siRNA-t1253841	TGCCAGGTAGATGACCGACAACA	23	1	FgHV1gp2	-
siRNA-t1076821	TGCCATAACCTCGTTGGGACCTT	23	1	FgHV1gp2	-
siRNA-t1410831	TGCCATCACTTCCATCGGTC	20	1	FGSG_05736	K11886|1|0.0|3673|fgr:FG05736.1|proteasome component ECM29
siRNA-t0805970	TGCCATCCTATAGATCGCGCGTAA	24	1	FgHV1gp2	-
siRNA-t0596055	TGCCCAACATTGATCCATGC	20	1	FGSG_10627	K01427|1|0.0|846|ani:AN0431.2|urease [EC:3.5.1.5]
siRNA-t0478825	TGCCGCGCGTCTCACTGACTTC	22	1	FgHV1gp2	-
siRNA-t1268774	TGCCGCGGATAACCTATACTTG	22	1	FgHV1gp2	-
siRNA-t0932810	TGCCGGTGTCGGCTGCCAAGTAA	23	1	FgHV1gp2	-
siRNA-t0500067	TGCCTCGGTCAGACCCCAGA	20	1	FgHV1gp2	-
siRNA-t0430032	TGCCTCGGTCAGACCCCAGAT	21	1	FgHV1gp2	-
siRNA-t0612903	TGCCTCGGTCAGACCCCAGATC	22	1	FgHV1gp2	-
siRNA-t0408124	TGCCTCGTTCCCAACTCCTG	20	1	FGSG_03996	K01113|1|1e-132|473|cim:CIMG_03455|alkaline phosphatase D [EC:3.1.3.1];K10402|2|1e-36|154|lbc:LACBIDRAFT_379819|kinesin family member 20/23
siRNA-t1351347	TGCCTGATGTTCTTGTGCCT	20	1	FGSG_07575	-
siRNA-t1361463	TGCCTGATGTTCTTGTGCCTCCGA	24	1	FgHV1gp2	-
siRNA-t1191117	TGCGAATGTTTCACTCCTCC	20	1	FgHV1gp2	-
siRNA-t0536341	TGCGCTTCTGGATCAATGTCAGCA	24	1	FgHV1gp2	-
siRNA-t1305395	TGCGTCTCAACCAAAGCATG	20	1	FGSG_10187	-
siRNA-t0369331	TGCGTCTCAACCAAAGCATGCT	22	1	FGSG_10187	-
siRNA-t1418893	TGCTACTCGAACCGCCCTTGTAG	23	1	FgHV1gp2	-
siRNA-t0620311	TGCTACTCGAACCGCCCTTGTAGC	24	1	FgHV1gp2	-
siRNA-t0730062	TGCTTGGTTTCGGGACGCGACAAG	24	1	FgHV1gp2	-

siRNA-t0606233	TGCTTGTCAGTGTCAGGATC	20	1	FGSG_06354	K08157|1|2e-60|232|nhe:NECHADRAFT_101392|MFS transporter, DHA1 family, multidrug resistance protein
siRNA-t1410646	TGCTTGTCAGTGTCAGGATCCGTC	24	1	FgHV1gp2	-
siRNA-t0569959	TGCTTGTCAGTGTCAGGATCCGTCC	25	1	FgHV1gp2	-
siRNA-t1091888	TGCTTTACCGGATCTCCGCTC	21	1	FgHV1gp1	-
siRNA-t0812451	TGGAAATCTTCAGGCCCTGGGAT	23	1	FgHV1gp2	-
siRNA-t1044133	TGGAACGTCAAATCAAGTTG	20	1	FgHV1gp2	-
siRNA-t0776112	TGGACAAGTACTCCACCTTTG	21	1	FgHV1gp2	-
siRNA-t0910500	TGGAGTTCCTTCGGAAAGTA	20	1	FGSG_10722	K01870|1|0.0|2196|fgr:FG10722.1|isoleucyl-tRNA synthetase [EC:6.1.1.5]
siRNA-t0498917	TGGATGCTGCGAAACCCAATTT	22	1	FgHV1gp2	-
siRNA-t0880630	TGGATGTGGTCTTTCTTCGG	20	1	FGSG_07854	K06677|1|0.0|2375|fgr:FG07854.1|condensin complex subunit 1
siRNA-t0754685	TGGATTTGAGGGCGAGTGGAGCTAC	25	1	FgHV1gp2	-
siRNA-t1189311	TGGCAAGGCCCTCAGTAGTCGA	22	1	FgHV1gp2	-
siRNA-t1257165	TGGCATCATCCTAGAGATCT	20	1	FGSG_09073	K05989|1|0.0|1831|fgr:FG09073.1|alpha-L-rhamnosidase [EC:3.2.1.40]
siRNA-t0515753	TGGCCGTAAGTAGCAGATTT	20	1	FgHV1gp2	-
siRNA-t0421691	TGGCCTCTTCCATTCCAGCCTAGA	24	1	FGSG_01510	-
siRNA-t0404861	TGGCGACACCGACGGTACGTG	21	1	FgHV1gp2	-
siRNA-t1471152	TGGTGCCGACGTCTACGTAA	20	1	FgHV1gp2	-
siRNA-t0587071	TGGTGTCCAATGTAAGTGAC	20	1	FGSG_01194	K10625|1|0.0|4192|fgr:FG01194.1|E3 ubiquitin-protein ligase UBR1 [EC:6.3.2.19]
siRNA-t0373240	TGGTGTGGCGGGACCAGGATT	21	1	FGSG_08904	-
siRNA-t1171164	TGGTTCAAGTATCGTCACTTC	21	1	FgHV1gp2	-
siRNA-t0904251	TGGTTGAGGCTTCTTCATGACATG	24	1	FgHV1gp2	-
siRNA-t1090126	TGGTTTGAATGGTCTGCCTA	20	1	FGSG_12039	K00698|1|0.0|2441|nhe:NECHADRAFT_123231|chitin synthase [EC:2.4.1.16]
siRNA-t0512445	TGGTTTGAATGGTCTGCCTAA	21	1	FGSG_08405	K03657|1|0.0|1811|fgr:FG08405.1|DNA helicase II / ATP-dependent DNA helicase PcrA [EC:3.6.4.12]
siRNA-t0617172	TGGTTTGTTCTGAAATTTGA	20	1	FGSG_13662	K03018|1|0.0|2938|fgr:FG09860.1|DNA-directed RNA polymerase III subunit RPC1 [EC:2.7.7.6]
siRNA-t0852302	TGGTTTGTTCTGAAATTTGATGTTG	25	1	FgHV1gp2	-
siRNA-t0523711	TGTAAGTCTGGAATTCCTTG	20	1	FGSG_06874	K03163|1|0.0|1651|fgr:FG06874.1|DNA topoisomerase I [EC:5.99.1.2]
siRNA-t1333164	TGTACTTGCCGCGCGTCTCACTGAC	25	1	FgHV1gp2	-
siRNA-t0600976	TGTAGGCTCCTCCGACAATGG	21	1	FgHV1gp2	-
siRNA-t0604040	TGTAGTGTCTGAAGATCTCAGAA	23	1	FGSG_05705	K08157|1|4e-68|258|lel:LELG_05567|MFS transporter, DHA1 family, multidrug resistance protein
siRNA-t0405484	TGTCACCGCCCCGATTCTTGTAAT	24	1	FgHV1gp2	-
siRNA-t1445426	TGTCAGGATCCGTCCAGGGAA	21	1	FgHV1gp2	-
siRNA-t1439903	TGTCATCATCAGAAACAGTGA	21	1	FGSG_11008	K00108|1|0.0|1256|fgr:FG11008.1|choline dehydrogenase [EC:1.1.99.1]
siRNA-t1015132	TGTCCAATGTAAGTGACGAGGCCTC	25	1	FgHV1gp2	-
siRNA-t0510347	TGTCCACACCGCGTTTCGCT	20	1	FgHV1gp1	-
siRNA-t1289691	TGTCGCGTCTACCTTGTTCAG	21	1	FgHV1gp2	-
siRNA-t1230311	TGTCGTAGGAAAGGAAAAGT	20	1	FGSG_01049	-
siRNA-t0994789	TGTCTGACTGTTGCTTGATG	20	1	FGSG_12306	-
siRNA-t0590142	TGTGAAATTTATGGCATATGCA	22	1	FgHV1gp2	-
siRNA-t0893038	TGTGACTCCAGCCAAAGAGAGGTG	24	1	FgHV1gp2	-
siRNA-t0585879	TGTGCGACGACCGTGCGAAAT	21	1	FGSG_13129	-
siRNA-t0909979	TGTGCGACGACCGTGCGAAATTGT	24	1	FgHV1gp2	-
siRNA-t0405122	TGTGCGACGACCGTGCGAAATTGTT	25	1	FgHV1gp2	-
siRNA-t0967275	TGTGGCGGGACCAGGATTCTTCT	23	1	FGSG_08844	K01183|1|1e-06|55.1|pan:PODANSg4492|chitinase [EC:3.2.1.14]
siRNA-t1040627	TGTGGGTCTTCTGACGAATTTGG	23	1	FgHV1gp2	-
siRNA-t1179775	TGTGTCGCGTCTACCTTGTTCAGG	24	1	FgHV1gp2	-
siRNA-t1332963	TGTTACCAGTGAAGTCGAAAG	21	1	FGSG_01488	-
siRNA-t0457895	TGTTATAAGGCTTGTCGTCGG	21	1	FGSG_09681	K01533|1|0.0|2289|fgr:FG09681.1|Cu2+-exporting ATPase [EC:3.6.3.4]
siRNA-t1083156	TGTTATTTGATGAGTTCCAC	20	1	FGSG_06097	-
siRNA-t0594705	TGTTCAAGTCTCTTGTTCTG	20	1	FGSG_02460	K14950|1|0.0|2580|fgr:FG02460.1|cation-transporting ATPase 13A1 [EC:3.6.3.-]
siRNA-t0944064	TGTTCCATAAACGCTGCCGG	20	1	FgHV1gp2	-
siRNA-t0872535	TGTTCCATAAACGCTGCCGGGA	22	1	FgHV1gp2	-
siRNA-t1031831	TGTTCCATAAACGCTGCCGGGAT	23	1	FgHV1gp2	-
siRNA-t0942760	TGTTCCCGAAAACAACACGCTT	22	1	FgHV1gp2	-
siRNA-t0429114	TGTTCTGAAACCATCAGGACCCGA	24	1	FgHV1gp2	-
siRNA-t1326644	TGTTCTGAAATTTGATGTTGTAGAA	25	1	FgHV1gp2	-
siRNA-t0522962	TGTTCTGGAACGTCAAATCAAGTTG	25	1	FgHV1gp2	-
siRNA-t1451979	TGTTGCCGAACGGTACATTGA	21	1	FGSG_06540	K01876|1|1e-10|64.3|fgr:FG04934.1|aspartyl-tRNA synthetase [EC:6.1.1.12]
siRNA-t1381396	TGTTGGTCTTCCAGGCATCA	20	1	FGSG_11505	-
siRNA-t1479483	TGTTTCACTCCTCCTTCCGTAA	22	1	FgHV1gp2	-
siRNA-t0975903	TGTTTGACGGCTTGTCCTATT	21	1	FGSG_01533	-
siRNA-t0794827	TGTTTGCTTGGTTTCGGGACG	21	1	FgHV1gp2	-
siRNA-t0712224	TGTTTGCTTGGTTTCGGGACGCGA	24	1	FgHV1gp2	-
siRNA-t0681417	TGTTTGTACGTCCGAATACCATACT	25	1	FgHV1gp2	-
siRNA-t1063524	TTACAATCGTGGACTTCCTTT	21	1	FgHV1gp2	-
siRNA-t0424472	TTACAATCGTGGACTTCCTTTAGG	24	1	FgHV1gp2	-
siRNA-t0998658	TTACAGGCCTCATTGATTTTGTGT	24	1	FgHV1gp2	-
siRNA-t1482776	TTACATCCGAAAATGTTTCA	20	1	FGSG_06694	-
siRNA-t0727832	TTACCAGACATTTTCGCGCGC	21	1	FgHV1gp2	-
siRNA-t1022316	TTACCAGGCCAACAAAGAGA	20	1	FGSG_13878	K01932|1|0.0|1450|afm:AFUA_5G12730|[EC:6.3.2.-]
siRNA-t1371453	TTACCCTCGCATTTGCCGCA	20	1	FGSG_06834	K02349|1|0.0|1748|fgr:FG06834.1|DNA polymerase theta subunit [EC:2.7.7.7]
siRNA-t1015908	TTACCTAGACCGAAGAAAGA	20	1	FGSG_03616	-
siRNA-t1331056	TTACCTAGACCGAAGAAAGAC	21	1	FGSG_03616	-
siRNA-t1149378	TTACCTAGACCGAAGAAAGACC	22	1	FgHV1gp2	-
siRNA-t0434758	TTACTTGGCAGCCGACACCG	20	1	FGSG_08102	-
siRNA-t1057272	TTATAAGGCTTGTCGTCGGG	20	1	FGSG_08614	K01874|1|0.0|1256|fgr:FG08614.1|methionyl-tRNA synthetase [EC:6.1.1.10]
siRNA-t1336610	TTATATGTCATGCGTGCTTGCGT	23	1	FgHV1gp2	-
siRNA-t0516257	TTATCGACAATGAAAGATCC	20	1	FgHV1gp2	-
siRNA-t0685458	TTATCGTGTCTGACTGTTGCTT	22	1	FgHV1gp2	-
siRNA-t0933554	TTATGAGCTCTGTGACTCCA	20	1	FgHV1gp2	-
siRNA-t1154077	TTATTGATATTAGCCCCCTG	20	1	FgHV1gp2	-
siRNA-t1161667	TTCAACCTGACCTGTTCCAAGT	22	1	FgHV1gp2	-
siRNA-t0790204	TTCAACGTGGATTTTGCTACCAG	23	1	FgHV1gp1	-
siRNA-t0995966	TTCAAGTCTCTTGTTCTGGAAC	22	1	FGSG_02381	-
siRNA-t0422945	TTGCGCACATATCTCCTTGAAC	22	1	FgHV1gp2	-
siRNA-t0407219	TTGCGCACATATCTCCTTGAACTT	24	1	FgHV1gp2	-
siRNA-t0386334	TTGCGTACCTGCAGTCACAAC	21	1	FGSG_01743	K01895|1|0.0|1310|fgr:FG01743.1|acetyl-CoA synthetase [EC:6.2.1.1]
siRNA-t0712579	TTGCTGTAGTGTCTGAAGAT	20	1	FGSG_05705	K08157|1|4e-68|258|lel:LELG_05567|MFS transporter, DHA1 family, multidrug resistance protein
siRNA-t0708260	TTGGCACAAACATTGGCACGC	21	1	FGSG_08708	-
siRNA-t0698016	TTGGCATCATCCTAGAGATCT	21	1	FGSG_05079	-
siRNA-t0471893	TTGGGGTCTTGACTTGGGCT	20	1	FGSG_13911	K09424|1|1e-28|128|lth:KLTH0C07788g|Myb-like DNA-binding protein REB1
siRNA-t0746970	TTGGGGTCTTGACTTGGGCTG	21	1	FGSG_13911	K09424|1|1e-28|128|lth:KLTH0C07788g|Myb-like DNA-binding protein REB1
siRNA-t0569948	TTGGGTGGCTTTGAAGTTGA	20	1	FGSG_10617	K14859|1|2e-98|359|scm:SCHCODRAFT_255991|ribosome biogenesis protein SSF1/2;K00143|2|1e-14|82.0|zro:ZYRO0C16566g|L-aminoadipate-semialdehyde dehydrogenase [EC:1.2.1.31]
siRNA-t0816290	TTGGTCCGCGAATTCTGCCATA	22	1	FgHV1gp2	-
siRNA-t0517122	TTGGTCCGCGAATTCTGCCATAACC	25	1	FgHV1gp2	-
siRNA-t0489911	TTGGTTCAAGTATCGTCACTTC	22	1	FgHV1gp2	-

siRNA-t0739104	TTGTAAGTCTGGAATTCCTT	20	1	FGSG_06743	K14802|1|0.0|2681|fgr:FG06743.1|phospholipid-transporting ATPase [EC:3.6.3.1]
siRNA-t0433007	TTGTACTTGCCGCGCGTCTCACTG	24	1	FgHV1gp2	-
siRNA-t0459905	TTGTCCAAATTCGTCAGAAG	20	1	FGSG_09793	-
siRNA-t0672308	TTGTTATAAGGCTTGTCGTC	20	1	FGSG_09553	-
siRNA-t0461079	TTGTTATAAGGCTTGTCGTCGGG	23	1	FgHV1gp2	-
siRNA-t0507547	TTGTTCATCTGCACTCCTTT	20	1	FGSG_12863	K02324|1|0.0|3986|fgr:FG06065.1|DNA polymerase epsilon subunit 1 [EC:2.7.7.7]
siRNA-t0463524	TTGTTCTGAAATTTGATGTTGTA	23	1	FgHV1gp2	-

Table S4. Potential target transcripts of FgHV2-derived siRNAs.

```
query=JS16_t1324315, target=FGSG_05288, score=3, range=135-154, strand=-1

target  5’ CUGCG-ACGGUAAAGGUGAUG 3’
            :::: ::::::::.::::::
query   3’ UACGCAUGCCAUUUUCACUAC 5’

query=JS16_t1324315, target=FGSG_13253, score=4, range=3123-3143, strand=-1

target  5’ GUGCGUAUCGUGAAAGUGAUG 3’
           .::::::. ::.:::::::::
query   3’ UACGCAUGCCAUUUUCACUAC 5’

query=JS16_t3116780, target=FGSG_09876, score=3, range=2383-2401, strand=-1

target  5’ GUGCG-ACGAGCGCUCGGAU 3’
           :. :. ::::::::::::::
query   3’ CGAGUGUGCUCGCGAGCCUA 5’

query=JS16_t3116780, target=FGSG_11483, score=4, range=504-522, strand=1

target  5’ GUUCA-GCGAGCGCUUGGAG 3’
           :.::: .::::::::.:::
query   3’ CGAGUGUGCUCGCGAGCCUA 5’

query=JS16_t0240155, target=FGSG_01270, score=3, range=77-99, strand=-1

target  5’ AGGGUGAUCAUGAGCUUGAGCCU 3’
           :::: ::::::::::::::: :
query   3’ UCCCUCUAGUACUCGAACUCGCA 5’

query=JS16_t0240155, target=FGSG_12705, score=4, range=346-367, strand=-1

target  5’ AGGGAGGUU-UUAGCUUGAGCGU 3’
           ::::::.:.  : :::::::::::
query   3’ UCCCUCUAGUACUCGAACUCGCA 5’

No results for JS16_t1324394
query=JS16_t4013643, target=FGSG_13670, score=3, range=2306-2324, strand=-1

target  5’ UCGAAGGUAGUUUCGUGGC 3’
           ::: :::::.::::::::.:
query   3’ AGCCUCCAUUAAAGCACUG 5’

query=JS16_t4013643, target=FGSG_06820, score=3.5, range=1168-1185, strand=1

target  5’ UCCGAGGUAAUUUC-UGAU 3’
           :: ::::::::::: :::.
query   3’ AGCCUCCAUUAAAGCACUG 5’

query=JS16_t4013643, target=FGSG_00127, score=3.5, range=752-770, strand=-1

target  5’ UUGGUGGUAGUUUCGUGGC 3’
           :.:: ::::.::::::::.:
query   3’ AGCCUCCAUUAAAGCACUG 5’

query=JS16_t4013643, target=FGSG_08840, score=4, range=1155-1172, strand=-1

target  5’ UCGUCGGUAAUUUCG-GAC 3’
           :::  :::::::::: :::
query   3’ AGCCUCCAUUAAAGCACUG 5’

No results for JS16_t4013683
query=JS16_t4013697, target=FGSG_09304, score=4, range=980-1002, strand=1

target  5’ CUCAGCCCAACCUGGGAGAAGAG 3’
           : ::.:::::::::::: ::::.
query   3’ GCGUUGGGUUGGACCCUGUUCUU 5’

No results for JS16_t4013705
No results for JS16_t1324524
query=JS16_t2220959, target=FGSG_02654, score=4, range=277-297, strand=-1

target  5’ AUUGGAUGAAAGAUGGCUAUC 3’
            :::: : ::::::::::::
query   3’ CAACCCAAUUUCUACCGAUAC 5’

query=JS16_t3116998, target=FGSG_08922, score=3.5, range=383-399, strand=1

target  5’ UCA-GAGCCGAGCAGGGG 3’
           ::: :::::: ::::::.
query   3’ AGUACUCGGCCCGUCCCU 5’

query=JS16_t3116998, target=FGSG_12597, score=4, range=232-248, strand=1

target  5’ UCAUG-GCUGGGCGGGGA 3’
           ::::: ::.::::.::::
query   3’ AGUACUCGGCCCGUCCCU 5’
```

```
query=JS16_t1385666, target=FGSG_04694, score=4, range=7524-7541, strand=1

target  5’ UGAGCCAAGCAAGCCACU 3’
           :::::::::::: : :::
query   3’ ACUCGGUUCGUUAGCUGA 5’

query=JS16_t1385666, target=FGSG_11926, score=4, range=42-59, strand=1

target  5’ UGAGCCAAGCCAUCGCCU 3’
           :::::::::: :::: ::
query   3’ ACUCGGUUCGUUAGCUGA 5’

query=JS16_t1385666, target=FGSG_01862, score=4, range=597-614, strand=1

target  5’ UGUGCCGAGCAUUCGACU 3’
           :: :::.:::: ::::::
query   3’ ACUCGGUUCGUUAGCUGA 5’

query=JS16_t1385666, target=FGSG_08708, score=4, range=1812-1830, strand=1

target  5’ UGAGUCCGAGCACUCGACU 3’
           :::: ::.:::: ::::::
query   3’ ACUC-GGUUCGUUAGCUGA 5’

query=JS16_t1385666, target=FGSG_13824, score=4, range=1772-1789, strand=1

target  5’ UGAUCCAAGCAAUCGAAG 3’
           ::: ::::::::::::
query   3’ ACUCGGUUCGUUAGCUGA 5’

query=JS16_t1385666, target=FGSG_04159, score=4, range=557-573, strand=1

target  5’ UGAGCCAAGCAAUU-ACC 3’
           :::::::::::::. ::
query   3’ ACUCGGUUCGUUAGCUGA 5’

query=JS16_t1385666, target=FGSG_00243, score=4, range=1018-1035, strand=1

target  5’ UCAUCCAAGCGAUCGACC 3’
           : : ::::::.::::::
query   3’ ACUCGGUUCGUUAGCUGA 5’

query=JS16_t1385666, target=FGSG_07877, score=4, range=3207-3223, strand=1

target  5’ UGA-CCAAGCACUCGAUU 3’
           ::: ::::::: ::::.:
query   3’ ACUCGGUUCGUUAGCUGA 5’

query=JS16_t1385666, target=FGSG_00112, score=4, range=102-118, strand=1

target  5’ CAAG-CGAGCAAUCGACU 3’
             :: :.:::::::::::
query   3’ ACUCGGUUCGUUAGCUGA 5’

query=JS16_t1385666, target=FGSG_08735, score=4, range=1404-1422, strand=1

target  5’ UGAAGGCAAGAAAUCGACU 3’
           :: :: :::: ::::::::
query   3’ AC-UCGGUUCGUUAGCUGA 5’

query=JS16_t1385666, target=FGSG_10564, score=4, range=3788-3804, strand=1

target  5’ CGA-CCAAACAAUCGACU 3’
            :: :::: :::::::::
query   3’ ACUCGGUUCGUUAGCUGA 5’

query=JS16_t1385666, target=FGSG_02486, score=4, range=15-31, strand=1

target  5’ CAAGCCAA-CAAUCGACU 3’
             :::::: :::::::::
query   3’ ACUCGGUUCGUUAGCUGA 5’

query=JS16_t1385666, target=FGSG_03653, score=4, range=127-145, strand=1

target  5’ UCAGCAAAGCAAGUCGACU 3’
           : ::: :::::: ::::::
query   3’ ACUCGGUUCGUU-AGCUGA 5’

query=JS16_t1385666, target=FGSG_13961, score=4, range=584-601, strand=-1

target  5’ UGAACCAAGCAGUCGAUC 3’
           ::: :::::::.::::.
query   3’ ACUCGGUUCGUUAGCUGA 5’

query=JS16_t1385666, target=FGSG_02115, score=4, range=108-124, strand=-1

target  5’ UGAGCCAAGCGAU-GACC 3’
           ::::::::::.:: :::
```

```
query=JS16_t3116998, target=FGSG_02366, score=4, range=6-22, strand=1

target  5’ UC-UGAGGUGGGCAGGGA 3’
           :: :::: .:::::::::
query   3’ AGUACUCGGCCCGUCCCU 5’

query=JS16_t3116998, target=FGSG_09400, score=4, range=446-463, strand=-1

target  5’ GCAUGGGCCGGGUAGGGC 3’
            ::::.::::::.::::
query   3’ AGUACUCGGCCCGUCCCU 5’

query=JS16_t3116998, target=FGSG_00299, score=4, range=1279-1296, strand=-1

target  5’ CCAUGAGUCGGGCAUGGA 3’
            ::::::.:::::: :::
query   3’ AGUACUCGGCCCGUCCCU 5’

query=JS16_t3116998, target=FGSG_04468, score=4, range=259-277, strand=-1

target  5’ UCAGGACGCUGGGCAGGGA 3’
           ::: :: ::.:::::::::
query   3’ AGUACU-CGGCCCGUCCCU 5’

query=JS16_t3116998, target=FGSG_12278, score=4, range=339-355, strand=-1

target  5’ UCAUG-GCAGGGCAGGGA 3’
           ::::: :: :::::::::
query   3’ AGUACUCGGCCCGUCCCU 5’

query=JS16_t3116998, target=FGSG_10303, score=4, range=193-209, strand=-1

target  5’ GCA-GAGCCGGUCAGGGA 3’
            :: ::::::: ::::::
query   3’ AGUACUCGGCCCGUCCCU 5’

query=JS16_t4013832, target=FGSG_00152, score=3, range=1167-1184, strand=1

target  5’ UCGCGACUUGCUGCCCAG 3’
            :: :::::::::::::
query   3’ CGCUCUGAACGACGGGUG 5’

query=JS16_t4013832, target=FGSG_06551, score=3, range=293-309, strand=1

target  5’ GCGA-ACUUGCUGCCCGA 3’
           :::: :::::::::::.
query   3’ CGCUCUGAACGACGGGUG 5’

query=JS16_t4013832, target=FGSG_09464, score=3, range=358-376, strand=-1

target  5’ GUGAGGCUUGCUGCCACAC 3’
           :.:::.::::::::: :::
query   3’ CGCUCUGAACGACGG-GUG 5’

query=JS16_t4013832, target=FGSG_01194, score=3.5, range=305-322, strand=1

target  5’ ACGAGACUUGCUGCCUGU 3’
            ::::::::::::::...
query   3’ CGCUCUGAACGACGGGUG 5’

query=JS16_t4013832, target=FGSG_07182, score=3.5, range=479-497, strand=1

target  5’ GUGAGCACUUGCUGCUCGC 3’
           :.::: :::::::::.:.:
query   3’ CGCUC-UGAACGACGGGUG 5’

query=JS16_t4013832, target=FGSG_11576, score=3.5, range=50-68, strand=1

target  5’ ACGAGAUCUUGCUGCCCAU 3’
            ::::: :::::::::::.
query   3’ CGCUCU-GAACGACGGGUG 5’

query=JS16_t4013832, target=FGSG_01695, score=3.5, range=91-108, strand=-1

target  5’ GCGAGACUUGCUGCUCUU 3’
           ::::::::::::::.: .
query   3’ CGCUCUGAACGACGGGUG 5’

query=JS16_t4013832, target=FGSG_10148, score=3.5, range=823-840, strand=-1

target  5’ GUUCGACUUGCUGCUCAC 3’
           :.  ::::::::::.:::
query   3’ CGCUCUGAACGACGGGUG 5’

query=JS16_t4013832, target=FGSG_10094, score=3.5, range=3266-3283, strand=-1

target  5’ UUGCGACUUGCUGCCCAA 3’
            .: :::::::::::::
query   3’ CGCUCUGAACGACGGGUG 5’

query=JS16_t4013832, target=FGSG_00586, score=3.5, range=1328-1345, strand=-1
query   3’ ACUCGGUUCGUUAGCUGA 5’

query=JS16_t1385666, target=FGSG_05201, score=4, range=510-526, strand=-1

target  5’ UGAGCCAAGCAA-CGGCG 3’
           :::::::::::: ::.:
query   3’ ACUCGGUUCGUUAGCUGA 5’

query=JS16_t1385666, target=FGSG_01613, score=4, range=997-1013, strand=-1

target  5’ UGA-CCAAGCAGUCCACU 3’
           ::: :::::::.:: :::
query   3’ ACUCGGUUCGUUAGCUGA 5’

query=JS16_t1385666, target=FGSG_12239, score=4, range=557-573, strand=-1

target  5’ CCAGCCAAGCAAUCG-CU 3’
             ::::::::::::: ::
query   3’ ACUCGGUUCGUUAGCUGA 5’

query=JS16_t1385666, target=FGSG_10760, score=4, range=1112-1128, strand=-1

target  5’ CGAG-CAAGCAGUCGACA 3’
            ::: ::::::.:::::
query   3’ ACUCGGUUCGUUAGCUGA 5’

query=JS16_t1385666, target=FGSG_05389, score=4, range=1205-1223, strand=-1

target  5’ UGAGCCAGAGCAAUGGACU 3’
           ::::::: :::::: ::::
query   3’ ACUCGGU-UCGUUAGCUGA 5’

No results for JS16_t1385680
query=JS16_t3177944, target=FGSG_07921, score=1.5, range=501-518, strand=1

target  5’ CAACGUCGAUCCGGGAUG 3’
           ::: .:::::::::::::
query   3’ GUUUUAGCUAGGCCCUAC 5’

query=JS16_t3177944, target=FGSG_07245, score=2.5, range=1912-1929, strand=-1

target  5’ CAAAGUCGAUUCGGGAUC 3’
           ::::.:::::.::::::
query   3’ GUUUUAGCUAGGCCCUAC 5’

query=JS16_t3177944, target=FGSG_04620, score=3, range=340-358, strand=1

target  5’ CAAGGUCGAUCCGGAGAUG 3’
           :::..::::::::: ::::
query   3’ GUUUUAGCUAGGCC-CUAC 5’

query=JS16_t3177944, target=FGSG_13722, score=3, range=1384-1400, strand=-1

target  5’ CAAAAUCGAU-CGGGGUG 3’
           :::::::::: ::::.::
query   3’ GUUUUAGCUAGGCCCUAC 5’

query=JS16_t3177944, target=FGSG_03535, score=3.5, range=186-202, strand=1

target  5’ UGAAGUC-AUCCGGGAUG 3’
           ..::.:: ::::::::::
query   3’ GUUUUAGCUAGGCCCUAC 5’

query=JS16_t3177944, target=FGSG_12600, score=4, range=360-376, strand=1

target  5’ CAAGGUUGAU-CGGGAUG 3’
           :::..:.::: :::::::
query   3’ GUUUUAGCUAGGCCCUAC 5’

query=JS16_t3177944, target=FGSG_10702, score=4, range=283-299, strand=1

target  5’ GAAAAUCGAUCU-GGAUG 3’
            ::::::::::. :::::
query   3’ GUUUUAGCUAGGCCCUAC 5’

query=JS16_t3177944, target=FGSG_04807, score=4, range=51-67, strand=-1

target  5’ UAAAGUCGAUCUGGG-UG 3’
           .:::.::::::.::: ::
query   3’ GUUUUAGCUAGGCCCUAC 5’

query=JS16_t3177944, target=FGSG_04242, score=4, range=1179-1196, strand=-1

target  5’ AUAAAUCGAUCAGGGAUG 3’
             ::::::::: ::::::
query   3’ GUUUUAGCUAGGCCCUAC 5’

query=JS16_t0255336, target=FGSG_09818, score=3, range=2222-2239, strand=1

target  5’ GAAAC-UUUACCCCAGAGG 3’
           ::::: ::::::::::: :
query   3’ CUUUGCAAAUGGGGUCUAC 5’
```

```
target  5’ UCAUGACUUGCUGCCCAU 3’
            :  :::::::::::::.
query   3’ CGCUCUGAACGACGGGUG 5’

query=JS16_t4013832, target=FGSG_07433, score=4, range=1965-1983, strand=1

target  5’ UCGAGACUUGUUUGCCCAC 3’
            ::::::::: .:::::::
query   3’ CGCUCUGAAC-GACGGGUG 5’

query=JS16_t4013832, target=FGSG_10539, score=4, range=616-633, strand=1

target  5’ CCCAGACUUGUUGCCUAC 3’
            : :::::::.::::.::
query   3’ CGCUCUGAACGACGGGUG 5’

query=JS16_t4013832, target=FGSG_11110, score=4, range=1186-1203, strand=1

target  5’ UCCGGACUUGCUGUCCAU 3’
            : .:::::::::.:::.
query   3’ CGCUCUGAACGACGGGUG 5’

query=JS16_t4013832, target=FGSG_07944, score=4, range=833-851, strand=1

target  5’ GCGAGACUGGCUGGCCCAC 3’
           :::::::: ::: ::::::
query   3’ CGCUCUGAACGA-CGGGUG 5’

query=JS16_t4013832, target=FGSG_04060, score=4, range=119-135, strand=1

target  5’ GUG-GACUUGUUGCCUAU 3’
           :.: ::::::.::::.:.
query   3’ CGCUCUGAACGACGGGUG 5’

query=JS16_t4013832, target=FGSG_08604, score=4, range=368-386, strand=1

target  5’ GUGAUGACUUGCUGGCCAU 3’
           :.:: ::::::::: :::.
query   3’ CGCU-CUGAACGACGGGUG 5’

query=JS16_t4013832, target=FGSG_00883, score=4, range=918-934, strand=1

target  5’ ACGAG-CUUGCUGUCCAC 3’
            :::: :::::::.::::
query   3’ CGCUCUGAACGACGGGUG 5’

query=JS16_t4013832, target=FGSG_05297, score=4, range=2354-2370, strand=1

target  5’ GCG-GACUUGCUGGCUAC 3’
           ::: ::::::::: :.::
query   3’ CGCUCUGAACGACGGGUG 5’

query=JS16_t4013832, target=FGSG_11238, score=4, range=1517-1533, strand=1

target  5’ GCG-GACUUGAUGCCUAC 3’
           ::: :::::: ::::.::
query   3’ CGCUCUGAACGACGGGUG 5’

query=JS16_t4013832, target=FGSG_01723, score=4, range=34-50, strand=1

target  5’ GC-AGUCUUGCUGCUCAC 3’
           :: :: ::::::::.:::
query   3’ CGCUCUGAACGACGGGUG 5’

query=JS16_t4013832, target=FGSG_01970, score=4, range=205-222, strand=1

target  5’ GCUAAACUUGCUGCCCUC 3’
           :: : ::::::::::: :
query   3’ CGCUCUGAACGACGGGUG 5’

query=JS16_t4013832, target=FGSG_01434, score=4, range=181-198, strand=1

target  5’ GCUCGACUUGCUGCCGAC 3’
           ::  ::::::::::: ::
query   3’ CGCUCUGAACGACGGGUG 5’

query=JS16_t4013832, target=FGSG_07704, score=4, range=562-579, strand=1

target  5’ GCUACACUAGCUGCCCAC 3’
           :: : ::: :::::::::
query   3’ CGCUCUGAACGACGGGUG 5’

query=JS16_t4013832, target=FGSG_09329, score=4, range=2754-2772, strand=1

target  5’ GCAAGACUUGCAUGUCCAC 3’
           :: :::::::: ::.::::
query   3’ CGCUCUGAACG-ACGGGUG 5’

query=JS16_t4013832, target=FGSG_05477, score=4, range=1370-1387, strand=-1

target  5’ GCGAGACUUGCUGACUAG 3’
           ::::::::::::: :.:

query=JS16_t0255336, target=FGSG_05977, score=3.5, range=242-259, strand=1

target  5’ CAAAUGUUUACCCCAG-UG 3’
            :::.:::::::::::: ::
query   3’ CUUUGCAAAUGGGGUCUAC 5’

query=JS16_t0255336, target=FGSG_07936, score=3.5, range=165-182, strand=-1

target  5’ GAGA-AUUUACCCCAGAUU 3’
           ::.:  ::::::::::::
query   3’ CUUUGCAAAUGGGGUCUAC 5’

query=JS16_t0255336, target=FGSG_12556, score=4, range=247-265, strand=1

target  5’ GGAGAGUUUACUCCAGAUU 3’
           :.:. ::::::.::::::
query   3’ CUUUGCAAAUGGGGUCUAC 5’

query=JS16_t0255336, target=FGSG_06395, score=4, range=173-190, strand=1

target  5’ GGGAC-UUUAUCUCAGAUG 3’
           :..:: ::::.:.::::::
query   3’ CUUUGCAAAUGGGGUCUAC 5’

query=JS16_t0255336, target=FGSG_05990, score=4, range=5003-5022, strand=1

target  5’ GACAAGAUUUAUCCCAGAUG 3’
           :: ::  ::::.::::::::
query   3’ CU-UUGCAAAUGGGGUCUAC 5’

query=JS16_t0255336, target=FGSG_12280, score=4, range=430-447, strand=1

target  5’ GAUUCG-UUACCCCAGAUG 3’
           ::  :: ::::::::::::
query   3’ CUUUGCAAAUGGGGUCUAC 5’

query=JS16_t0255336, target=FGSG_10074, score=4, range=2314-2331, strand=1

target  5’ GCAAC-UUUACCCCAGAAG 3’
           : ::: ::::::::::: :
query   3’ CUUUGCAAAUGGGGUCUAC 5’

query=JS16_t0255336, target=FGSG_04899, score=4, range=1079-1098, strand=-1

target  5’ AAAGUACUUUACCCCAGAUG 3’
            ::..  :::::::::::::
query   3’ CUUUG-CAAAUGGGGUCUAC 5’

No results for JS16_t4074689
No results for JS16_t1385858
query=JS16_t2282020, target=FGSG_08728, score=2.5, range=2296-2313, strand=1

target  5’ GCU-UUGCUAUUCCUUUCA 3’
           .:: ::::::::::::::
query   3’ UGACAACGAUAAGGAAAGG 5’

query=JS16_t2282020, target=FGSG_07800, score=3, range=617-634, strand=1

target  5’ ACU-UUGCUGUUCCUUUCA 3’
           ::: :::::.::::::::
query   3’ UGACAACGAUAAGGAAAGG 5’

query=JS16_t2282020, target=FGSG_05973, score=3, range=908-925, strand=1

target  5’ AC-GGUGCUAUUCCUUUCA 3’
           :: : :::::::::::::
query   3’ UGACAACGAUAAGGAAAGG 5’

query=JS16_t2282020, target=FGSG_10738, score=3.5, range=179-196, strand=1

target  5’ GCU-UUGCUGUUCCUUUCG 3’
           .:: :::::.::::::::
query   3’ UGACAACGAUAAGGAAAGG 5’

query=JS16_t2282020, target=FGSG_11573, score=4, range=1030-1048, strand=1

target  5’ ACAGUUGCUAUUCUUGUCC 3’
           :: ::::::::::.: :::
query   3’ UGACAACGAUAAGGAAAGG 5’

query=JS16_t2282020, target=FGSG_06551, score=4, range=1408-1426, strand=1

target  5’ CCUGUUGCUAUUCCUGUCA 3’
            ::::::::::::: ::
query   3’ UGACAACGAUAAGGAAAGG 5’

query=JS16_t2282020, target=FGSG_04807, score=4, range=916-934, strand=1

target  5’ GUCGGAGCUAUUCCUUUCC 3’
           .. :  :::::::::::::
query   3’ UGACAACGAUAAGGAAAGG 5’
```

```
query    3' CGCUCUGAACGACGGGUG 5'

query=JS16_t4013832, target=FGSG_06580, score=4, range=2608-2625, strand=-1

target  5' GCGAGACUUGCUGCGCUC 3'
           :::::::::::::: : :
query    3' CGCUCUGAACGACGGGUG 5'

query=JS16_t4013832, target=FGSG_04241, score=4, range=366-383, strand=-1

target  5' UCGAGACUUUCUGCCCAG 3'
            :::::::: :::::::
query    3' CGCUCUGAACGACGGGUG 5'

query=JS16_t4013832, target=FGSG_08060, score=4, range=533-550, strand=-1

target  5' GAGCGACUUGUUGUCCAC 3'
           : : ::::::.::.::::
query    3' CGCUCUGAACGACGGGUG 5'

query=JS16_t4013832, target=FGSG_10090, score=4, range=539-556, strand=-1

target  5' CCGAUACUUGCUGUCCAA 3'
            ::: ::::::::.:::
query    3' CGCUCUGAACGACGGGUG 5'

query=JS16_t4013832, target=FGSG_12631, score=4, range=241-257, strand=-1

target  5' UCGGGACUUGCUG-CCAU 3'
            ::.::::::::: :::.
query    3' CGCUCUGAACGACGGGUG 5'

query=JS16_t4013832, target=FGSG_01910, score=4, range=148-166, strand=-1

target  5' GCGAAGACUUGAUGCCCAA 3'
           ::: ::::::: ::::::
query    3' CGC-UCUGAACGACGGGUG 5'

query=JS16_t4013832, target=FGSG_00849, score=4, range=372-390, strand=-1

target  5' GAGAGACUUGCUGGCCCAG 3'
           : :::::::::: :::::
query    3' CGCUCUGAACGA-CGGGUG 5'

query=JS16_t4013832, target=FGSG_09607, score=4, range=10495-10512, strand=-1

target  5' GAAAGACUUGCUGCCCUC 3'
           :  ::::::::::::: :
query    3' CGCUCUGAACGACGGGUG 5'

query=JS16_t4013832, target=FGSG_00203, score=4, range=558-575, strand=-1

target  5' GCAACACUUGCUGUCCAG 3'
           :: : ::::::::.:::
query    3' CGCUCUGAACGACGGGUG 5'

query=JS16_t4013832, target=FGSG_04595, score=4, range=638-655, strand=-1

target  5' GCAUGACUUGCUGUCCAA 3'
           ::  :::::::::.:::
query    3' CGCUCUGAACGACGGGUG 5'

query=JS16_t4013832, target=FGSG_02766, score=4, range=2584-2601, strand=-1

target  5' GCCAUACUUGCUGGCCAC 3'
           :: : :::::::: ::::
query    3' CGCUCUGAACGACGGGUG 5'

query=JS16_t4013832, target=FGSG_07245, score=4, range=3344-3361, strand=-1

target  5' GCAAAACUUCCUGCCCAC 3'
           :: : :::: ::::::::
query    3' CGCUCUGAACGACGGGUG 5'

query=JS16_t4013832, target=FGSG_07043, score=4, range=1393-1410, strand=-1

target  5' GCUUGACUUGAUGCCCAC 3'
           ::  :::::: :::::::
query    3' CGCUCUGAACGACGGGUG 5'

query=JS16_t4013832, target=FGSG_07834, score=4, range=1139-1156, strand=-1

target  5' GAGAAACUUGCUGCACAC 3'
           : :: ::::::::: :::
query    3' CGCUCUGAACGACGGGUG 5'

query=JS16_t4013832, target=FGSG_09994, score=4, range=1379-1397, strand=-1

target  5' GCGCCACUUGCUGCCGCAC 3'
           :::  :::::::::: :::
query    3' CGCUCUGAACGACGG-GUG 5'

query=JS16_t2282020, target=FGSG_13252, score=4, range=692-709, strand=1

target  5' AC-GUGGCUAUUCUUUUCA 3'
           :: :: :::::::.::::
query    3' UGACAACGAUAAGGAAAGG 5'

query=JS16_t2282020, target=FGSG_08425, score=4, range=527-544, strand=1

target  5' ACU-CUGCUAUUCCUAUCC 3'
           :::  :::::::::: :::
query    3' UGACAACGAUAAGGAAAGG 5'

query=JS16_t2282020, target=FGSG_08201, score=4, range=2015-2033, strand=-1

target  5' GCCGUUGCUAUGCCUUUCU 3'
           .: :::::::: ::::::.
query    3' UGACAACGAUAAGGAAAGG 5'

query=JS16_t2282020, target=FGSG_08778, score=4, range=1636-1653, strand=-1

target  5' AC-GGUGCUUUUCCUUUCC 3'
           :: : :::: :::::::::
query    3' UGACAACGAUAAGGAAAGG 5'

query=JS16_t1385991, target=FGSG_10632, score=2, range=691-709, strand=-1

target  5' UCUCUGGGUCGGUUGACUC 3'
           ::::: :.:::::::::::
query    3' AGAGA-CUAGCCAACUGAG 5'

query=JS16_t1385991, target=FGSG_13406, score=3.5, range=206-223, strand=1

target  5' UUUCUGACUGGUUGACUC 3'
           :.::::: .:::::::::
query    3' AGAGACUAGCCAACUGAG 5'

query=JS16_t1385991, target=FGSG_13783, score=3.5, range=12632-12649, strand=1

target  5' UGACUGAUCGGUUGGCUU 3'
           :  :::::::::::.::.
query    3' AGAGACUAGCCAACUGAG 5'

query=JS16_t1385991, target=FGSG_09470, score=3.5, range=972-989, strand=-1

target  5' UCUUUGAUCGGUUGGAUC 3'
           :::.::::::::::. ::
query    3' AGAGACUAGCCAACUGAG 5'

query=JS16_t1385991, target=FGSG_05725, score=3.5, range=743-760, strand=-1

target  5' UUCCUGAUCGGAUGACUC 3'
           :. :::::::: ::::::
query    3' AGAGACUAGCCAACUGAG 5'

query=JS16_t1385991, target=FGSG_08245, score=3.5, range=658-675, strand=-1

target  5' UUCCUGCUCGGUUGACUC 3'
           :. ::: :::::::::::
query    3' AGAGACUAGCCAACUGAG 5'

query=JS16_t1385991, target=FGSG_05963, score=3.5, range=1192-1208, strand=-1

target  5' UUACUGAU-GGUUGACUC 3'
           :. ::::: :::::::::
query    3' AGAGACUAGCCAACUGAG 5'

query=JS16_t1385991, target=FGSG_03647, score=4, range=604-621, strand=1

target  5' UUCUUGAUCGGUGGACUC 3'
           :. .:::::::: :::::
query    3' AGAGACUAGCCAACUGAG 5'

query=JS16_t1385991, target=FGSG_04280, score=4, range=269-286, strand=1

target  5' UUCCCGAUCGGUUGAUUU 3'
           :. : ::::::::::.:.
query    3' AGAGACUAGCCAACUGAG 5'

query=JS16_t1385991, target=FGSG_09691, score=4, range=772-789, strand=1

target  5' CCCCUGAUCGGUCGACUC 3'
            : ::::::::: :::::
query    3' AGAGACUAGCCAACUGAG 5'

query=JS16_t1385991, target=FGSG_02774, score=4, range=651-667, strand=1

target  5' CCUC-GAUCGGUUGGCUG 3'
            ::: :::::::::.::
query    3' AGAGACUAGCCAACUGAG 5'

query=JS16_t1385991, target=FGSG_01234, score=4, range=3493-3510, strand=1
```

```
query=JS16_t4013832, target=FGSG_10290, score=4, range=1791-1808, strand=-1

target  5' GCUUCACUUGUUGCCCAC 3'
           ::   :::::.:::::::
query   3' CGCUCUGAACGACGGGUG 5'

No results for JS16_t3117037
query=JS16_t0282991, target=FGSG_10048, score=2.5, range=1165-1182, strand=-1

target  5' GCUUA-GAUCUCCCUCGGC 3'
           :::.: ::::::::::.::
query   3' CGAGUACUAGAGGGAGUCG 5'

query=JS16_t0282991, target=FGSG_05700, score=3.5, range=1951-1968, strand=1

target  5' GCUCGUG-UCUCUCUCAGC 3'
           ::::.:: ::::.::::::
query   3' CGAGUACUAGAGGGAGUCG 5'

query=JS16_t0282991, target=FGSG_09312, score=3.5, range=253-270, strand=1

target  5' GCUCAUGAUUUCCC-CAGU 3'
           :::::::::.:::: :::.
query   3' CGAGUACUAGAGGGAGUCG 5'

query=JS16_t0282991, target=FGSG_03677, score=3.5, range=1723-1741, strand=1

target  5' CUUGAAGAUCUCCCUCAGC 3'
            .: : :::::::::::::
query   3' CGAGUACUAGAGGGAGUCG 5'

query=JS16_t0282991, target=FGSG_07947, score=3.5, range=631-649, strand=-1

target  5' CUUCGUGAUCUCCUUCAGU 3'
            .::.::::::::.::::.
query   3' CGAGUACUAGAGGGAGUCG 5'

query=JS16_t0282991, target=FGSG_05276, score=3.5, range=1086-1104, strand=-1

target  5' GUUCAUGAUCUCCUUCAUC 3'
           :.:::::::::::.::: :
query   3' CGAGUACUAGAGGGAGUCG 5'

query=JS16_t0282991, target=FGSG_02791, score=3.5, range=349-367, strand=-1

target  5' UAUCGUGAUCUUCCUCAGC 3'
             ::.::::::.:::::::
query   3' CGAGUACUAGAGGGAGUCG 5'

query=JS16_t0282991, target=FGSG_01580, score=4, range=609-627, strand=1

target  5' UGUCAUGAUCUUCUUCAGC 3'
             :::::::::.:.:::::
query   3' CGAGUACUAGAGGGAGUCG 5'

query=JS16_t0282991, target=FGSG_07584, score=4, range=1201-1218, strand=1

target  5' GC-CAUGAUCUUCCUUGGC 3'
           :: ::::::::.:::..::
query   3' CGAGUACUAGAGGGAGUCG 5'

query=JS16_t0282991, target=FGSG_04622, score=4, range=960-979, strand=1

target  5' ACUCAUUGAUCUUCCUCAGG 3'
            :::: ::::::.::::::
query   3' CGAGU-ACUAGAGGGAGUCG 5'

query=JS16_t0282991, target=FGSG_10300, score=4, range=1519-1538, strand=1

target  5' GUUCCUGGAUUUCCCUCAGU 3'
           :.:: : :::.::::::::.
query   3' CGAGUA-CUAGAGGGAGUCG 5'

query=JS16_t0282991, target=FGSG_09899, score=4, range=982-1000, strand=1

target  5' GCCCUUGAUCUCUCUCAGG 3'
           :: : :::::::.:::::
query   3' CGAGUACUAGAGGGAGUCG 5'

query=JS16_t0282991, target=FGSG_01768, score=4, range=30-48, strand=1

target  5' UCUCCAGAUCUUCCUCAGC 3'
            :::  :::::.:::::::
query   3' CGAGUACUAGAGGGAGUCG 5'

query=JS16_t0282991, target=FGSG_13056, score=4, range=895-912, strand=1

target  5' GCUGAUG-UCUUCCUCAGC 3'
           ::: ::: :::.:::::::
query   3' CGAGUACUAGAGGGAGUCG 5'

query=JS16_t0282991, target=FGSG_05325, score=4, range=2618-2635, strand=1

target  5' UCCCAGAUCGGUUGUCUC 3'
           :: : ::::::::: :::
query   3' AGAGACUAGCCAACUGAG 5'

query=JS16_t1385991, target=FGSG_01954, score=4, range=519-537, strand=1

target  5' UCUCGAAUCGGUUGAGCUC 3'
           ::::  ::::::::: :::
query   3' AGAGACUAGCCAACU-GAG 5'

query=JS16_t1385991, target=FGSG_02281, score=4, range=843-861, strand=1

target  5' UCACAUGAUCGGUCGACUC 3'
           :: : :::::::: :::::
query   3' AGAG-ACUAGCCAACUGAG 5'

query=JS16_t1385991, target=FGSG_07391, score=4, range=575-592, strand=-1

target  5' UCUCAGAACGGUUGAUUC 3'
           :::: :: :::::::.::
query   3' AGAGACUAGCCAACUGAG 5'

query=JS16_t1385991, target=FGSG_00664, score=4, range=136-153, strand=-1

target  5' CAUCUGAUCGCUUGACUC 3'
             :::::::: :::::::
query   3' AGAGACUAGCCAACUGAG 5'

query=JS16_t1385991, target=FGSG_05511, score=4, range=1451-1468, strand=-1

target  5' UUCCUGAUCGUUUGACUU 3'
           :. ::::::: ::::::.
query   3' AGAGACUAGCCAACUGAG 5'

query=JS16_t1385991, target=FGSG_00697, score=4, range=2782-2798, strand=-1

target  5' UC-CUUGUCGGUUGACUC 3'
           :: :: .:::::::::::
query   3' AGAGACUAGCCAACUGAG 5'

query=JS16_t1385991, target=FGSG_03410, score=4, range=1449-1465, strand=-1

target  5' UCU-UGGGCGGUUGACUC 3'
           ::: ::. ::::::::::
query   3' AGAGACUAGCCAACUGAG 5'

query=JS16_t1385991, target=FGSG_12565, score=4, range=93-109, strand=-1

target  5' UCUGUGAUCGGUU-AUUC 3'
           ::: ::::::::: :.::
query   3' AGAGACUAGCCAACUGAG 5'

query=JS16_t1385991, target=FGSG_13878, score=4, range=13025-13043, strand=-1

target  5' UCUCCAGAUCGGUAGACUC 3'
           ::: : ::::::: :::::
query   3' AGA-GACUAGCCAACUGAG 5'

query=JS16_t1385991, target=FGSG_02701, score=4, range=2708-2724, strand=-1

target  5' UCUCCG-UCGGUUGACUA 3'
           :::: : ::::::::::
query   3' AGAGACUAGCCAACUGAG 5'

No results for JS16_t0202610
No results for JS16_t3178241
query=JS16_t3178246, target=FGSG_10353, score=3, range=992-1010, strand=-1

target  5' GCAAUG-CAGUAUUCCUCAU 3'
           :: ::: :::.:::::::::
query   3' CGAUACAGUCGUAAGGAGUA 5'

query=JS16_t3178246, target=FGSG_03237, score=3.5, range=800-818, strand=1

target  5' UCUGUGUCAGCAUUCC-CAU 3'
            ::.:::::::::::: :::
query   3' CGAUACAGUCGUAAGGAGUA 5'

query=JS16_t3178246, target=FGSG_08821, score=3.5, range=1112-1130, strand=1

target  5' GC-GUGACAGCAUUCCUCAC 3'
           :: .:: ::::::::::::
query   3' CGAUACAGUCGUAAGGAGUA 5'

query=JS16_t3178246, target=FGSG_08595, score=3.5, range=1477-1496, strand=-1

target  5' GUUAUGCCAGCAUUCAUCAU 3'
           :.:::: :::::::: ::::
query   3' CGAUACAGUCGUAAGGAGUA 5'

query=JS16_t3178246, target=FGSG_11821, score=4, range=2543-2562, strand=1
```

```
target  5' CCUC-UGAUCUUCCUCAGG 3'
            ::: ::::::.::::::
query   3' CGAGUACUAGAGGGAGUCG 5'

query=JS16_t0282991, target=FGSG_03252, score=4, range=362-379, strand=1

target  5' ACCCA-GGUCUCCCUCAGC 3'
            : :: :.:::::::::::
query   3' CGAGUACUAGAGGGAGUCG 5'

query=JS16_t0282991, target=FGSG_01384, score=4, range=144-163, strand=1

target  5' CCUCAUGGACCUCCCUCAGC 3'
            ::::: :: ::::::::::
query   3' CGAGUA-CUAGAGGGAGUCG 5'

query=JS16_t0282991, target=FGSG_13867, score=4, range=202-220, strand=-1

target  5' GCUCGUGAUGUUCCUCAGU 3'
           ::::.:::: :.::::::.
query   3' CGAGUACUAGAGGGAGUCG 5'

query=JS16_t0282991, target=FGSG_07192, score=4, range=1612-1630, strand=-1

target  5' GUUCAUGGUCUACCUCAGU 3'
           :.:::::.::: ::::::.
query   3' CGAGUACUAGAGGGAGUCG 5'

query=JS16_t0282991, target=FGSG_10048, score=4, range=547-565, strand=-1

target  5' GCUCAACAUCUCCCUCGGC 3'
           :::::  :::::::::.::
query   3' CGAGUACUAGAGGGAGUCG 5'

query=JS16_t0282991, target=FGSG_01348, score=4, range=795-814, strand=-1

target  5' GCUCUAUGAUGUCCCUCAGG 3'
           :::: ::::: ::::::::
query   3' CGAG-UACUAGAGGGAGUCG 5'

query=JS16_t0282991, target=FGSG_04598, score=4, range=822-840, strand=-1

target  5' GCUGAGCAUCUCCCUCAGC 3'
           ::: :  ::::::::::::
query   3' CGAGUACUAGAGGGAGUCG 5'

query=JS16_t0282991, target=FGSG_10459, score=4, range=1066-1084, strand=-1

target  5' GAUAAAGAUCUCCUUCAGC 3'
           : : : :::::::.:::::
query   3' CGAGUACUAGAGGGAGUCG 5'

No results for JS16_t2221012
query=JS16_t1324635, target=FGSG_13443, score=3, range=89-106, strand=1

target  5' AGCGCGCCAACUGCAAGC 3'
            : :::::::::::::.:
query   3' ACACGCGGUUGACGUUUG 5'

query=JS16_t1324635, target=FGSG_04944, score=3, range=1194-1211, strand=1

target  5' AGUGGGCCAACUGCAAGC 3'
            ::: :::::::::::.:
query   3' ACACGCGGUUGACGUUUG 5'

query=JS16_t1324635, target=FGSG_11997, score=3, range=251-268, strand=1

target  5' UAUGCGCCGACUGCAAAG 3'
           : ::::::.::::::::
query   3' ACACGCGGUUGACGUUUG 5'

query=JS16_t1324635, target=FGSG_09994, score=3, range=2011-2028, strand=1

target  5' CCCGCGCCAACUGCAAAC 3'
              :::::::::::::::
query   3' ACACGCGGUUGACGUUUG 5'

query=JS16_t1324635, target=FGSG_06437, score=3, range=42-58, strand=-1

target  5' UG-GCGUCAACUGCAAAG 3'
           :: :::.::::::::::
query   3' ACACGCGGUUGACGUUUG 5'

query=JS16_t1324635, target=FGSG_04591, score=3, range=804-820, strand=-1

target  5' UGUAC-CCAACUGCAAAC 3'
           ::: : ::::::::::::
query   3' ACACGCGGUUGACGUUUG 5'

query=JS16_t1324635, target=FGSG_04758, score=3.5, range=927-943, strand=1

target  5' GCUCAGUCAGCGUUCCUCAA 3'
           :::  ::::::.:::::::
query   3' CGAUACAGUCGUAAGGAGUA 5'

query=JS16_t3178246, target=FGSG_06352, score=4, range=458-476, strand=-1

target  5' GCUUUGUCAGCA-UCCUCAA 3'
           ::: :::::::: ::::::
query   3' CGAUACAGUCGUAAGGAGUA 5'

query=JS16_t3178246, target=FGSG_08080, score=4, range=745-763, strand=-1

target  5' GCU-UGACGGCAUUCCUCAA 3'
           ::: :: :.::::::::::
query   3' CGAUACAGUCGUAAGGAGUA 5'

query=JS16_t0473973, target=FGSG_00097, score=2, range=1268-1285, strand=-1

target  5' CACUGGUUGGAGGAGUAG 3'
           :::::::: :::::::::
query   3' GUGACCAAACUCCUCAUC 5'

query=JS16_t0473973, target=FGSG_13867, score=2.5, range=277-294, strand=1

target  5' UACUGGUUUGAGGAGAAG 3'
           .:::::::::::::: ::
query   3' GUGACCAAACUCCUCAUC 5'

query=JS16_t0473973, target=FGSG_07590, score=3.5, range=489-507, strand=1

target  5' CAUUCAGUUUGAGGAGUAC 3'
           ::.:  ::::::::::::
query   3' GUGA-CCAAACUCCUCAUC 5'

query=JS16_t0473973, target=FGSG_09615, score=3.5, range=1259-1275, strand=1

target  5' UAC-AGUUUGAGGAGUAC 3'
           .::  ::::::::::::
query   3' GUGACCAAACUCCUCAUC 5'

query=JS16_t0473973, target=FGSG_05685, score=3.5, range=682-699, strand=-1

target  5' UACUUGUUUGAGGAGUGU 3'
           .::: :::::::::::.
query   3' GUGACCAAACUCCUCAUC 5'

query=JS16_t0473973, target=FGSG_05667, score=4, range=160-177, strand=1

target  5' CACUGGUUUGGGGACUGG 3'
           ::::::::::.::: :.:
query   3' GUGACCAAACUCCUCAUC 5'

query=JS16_t0473973, target=FGSG_00371, score=4, range=2367-2384, strand=1

target  5' CACUGGUUUGUGGGGUAU 3'
           :::::::::: ::.:::
query   3' GUGACCAAACUCCUCAUC 5'

query=JS16_t0473973, target=FGSG_09065, score=4, range=498-515, strand=1

target  5' GACUGGUUUCAGGAGUGG 3'
            :::::::: ::::::.:
query   3' GUGACCAAACUCCUCAUC 5'

query=JS16_t0473973, target=FGSG_05387, score=4, range=3364-3381, strand=1

target  5' CUCUGGUUUGAGGAGUCU 3'
           : ::::::::::::::
query   3' GUGACCAAACUCCUCAUC 5'

query=JS16_t0473973, target=FGSG_10523, score=4, range=2390-2408, strand=1

target  5' CACCGGCUUUGAGGAGUAU 3'
           ::: :: :::::::::::
query   3' GUGACC-AAACUCCUCAUC 5'

query=JS16_t0473973, target=FGSG_12693, score=4, range=759-777, strand=1

target  5' GACUGAGUUUGAGGAGAAG 3'
            :::: :::::::::: ::
query   3' GUGAC-CAAACUCCUCAUC 5'

query=JS16_t0473973, target=FGSG_00257, score=4, range=691-708, strand=1

target  5' AACGUGUUUGAGGAGUGG 3'
            ::  :::::::::::.:
query   3' GUGACCAAACUCCUCAUC 5'

query=JS16_t0473973, target=FGSG_01874, score=4, range=3253-3270, strand=1

target  5' CCCUCGUUUGAGGAGCAG 3'
```

```
target  5' UGU-CGUCGACUGCAAAU 3'
           ::: ::.:.::::::::.
query   3' ACACGCGGUUGACGUUUG 5'

query=JS16_t1324635, target=FGSG_08876, score=3.5, range=932-949, strand=1

target  5' UAUGUGCCAAGUGCAAAC 3'
           : ::.::::: :::::::
query   3' ACACGCGGUUGACGUUUG 5'

query=JS16_t1324635, target=FGSG_05366, score=3.5, range=378-395, strand=-1

target  5' UUUGUGCCAACUGCAGGC 3'
           : ::.:::::::::..:
query   3' ACACGCGGUUGACGUUUG 5'

query=JS16_t1324635, target=FGSG_08649, score=3.5, range=1155-1172, strand=-1

target  5' UGAGUGUCAACUGCAAAG 3'
           :: :.:.::::::::::
query   3' ACACGCGGUUGACGUUUG 5'

query=JS16_t1324635, target=FGSG_08066, score=3.5, range=890-908, strand=-1

target  5' UCUGUGCCAACUGCAACAC 3'
           : ::.::::::::::: ::
query   3' ACACGCGGUUGACGUU-UG 5'

query=JS16_t1324635, target=FGSG_08786, score=4, range=4035-4052, strand=1

target  5' UGUGCGCAAACUUCAAAC 3'
           ::::::: :::: :::::
query   3' ACACGCGGUUGACGUUUG 5'

query=JS16_t1324635, target=FGSG_08933, score=4, range=1226-1243, strand=1

target  5' UUUGCCCCAACUGCAAGC 3'
           : ::: ::::::::::.:
query   3' ACACGCGGUUGACGUUUG 5'

query=JS16_t1324635, target=FGSG_00469, score=4, range=152-169, strand=1

target  5' UGAGCGCCAACUGCAGCC 3'
           :: ::::::::::::. :
query   3' ACACGCGGUUGACGUUUG 5'

query=JS16_t1324635, target=FGSG_02368, score=4, range=50-67, strand=1

target  5' CUUGCGACAACUGCAAAC 3'
             :::: ::::::::::
query   3' ACACGCGGUUGACGUUUG 5'

query=JS16_t1324635, target=FGSG_09045, score=4, range=2055-2071, strand=1

target  5' CGUG-GUCAACUGCAGAC 3'
            ::: :.::::::::.::
query   3' ACACGCGGUUGACGUUUG 5'

query=JS16_t1324635, target=FGSG_09939, score=4, range=624-641, strand=1

target  5' UGAGCGCCAACGGCAAAA 3'
           :: :::::::: :::::
query   3' ACACGCGGUUGACGUUUG 5'

query=JS16_t1324635, target=FGSG_11672, score=4, range=134-151, strand=1

target  5' UAUGCGACAACUGCAAAA 3'
           : :::: ::::::::::
query   3' ACACGCGGUUGACGUUUG 5'

query=JS16_t1324635, target=FGSG_05421, score=4, range=676-693, strand=1

target  5' UCUUCGCCAGCUGCAAAA 3'
           : : :::::.:::::::
query   3' ACACGCGGUUGACGUUUG 5'

query=JS16_t1324635, target=FGSG_01463, score=4, range=1128-1146, strand=1

target  5' UGAGACGCCAGCUGCAAAG 3'
           :: : :::::.:::::::
query   3' ACAC-GCGGUUGACGUUUG 5'

query=JS16_t1324635, target=FGSG_07260, score=4, range=1232-1248, strand=1

target  5' UGU-CGCCAAUGGCAAAC 3'
           ::: ::::::. ::::::
query   3' ACACGCGGUUGACGUUUG 5'

query=JS16_t1324635, target=FGSG_00495, score=4, range=255-272, strand=1

target  5' UUCACGCCAACUGCAAAA 3'
           :   :::::::::::::
           : :: :::::::::: ::
query   3' GUGACCAAACUCCUCAUC 5'

query=JS16_t0473973, target=FGSG_00718, score=4, range=1383-1401, strand=1

target  5' UGCUGGUCUUGAGGAGUGG 3'
           ..::::: :::::::::.:
query   3' GUGACCA-AACUCCUCAUC 5'

query=JS16_t0473973, target=FGSG_11174, score=4, range=1137-1155, strand=1

target  5' CAAUGCUUUGAGGAUGUAG 3'
           :: :: :::::::: ::::
query   3' GUGACCAAACUCCU-CAUC 5'

query=JS16_t0473973, target=FGSG_12689, score=4, range=663-681, strand=1

target  5' CACUGCGUUUGAGGAGGAA 3'
           ::::: :::::::::: :
query   3' GUGAC-CAAACUCCUCAUC 5'

query=JS16_t0473973, target=FGSG_10043, score=4, range=82-100, strand=-1

target  5' CACUGAGUUUGAGGAGAGG 3'
           ::::: :::::::::: .:
query   3' GUGAC-CAAACUCCUCAUC 5'

query=JS16_t0473973, target=FGSG_01026, score=4, range=757-774, strand=-1

target  5' GCCUGGCUUGAGGAGUAG 3'
             :::: ::::::::::
query   3' GUGACCAAACUCCUCAUC 5'

query=JS16_t0473973, target=FGSG_02822, score=4, range=5-22, strand=-1

target  5' CGUGGUUUUGAGGAGUAG 3'
           :.. : :::::::::::
query   3' GUGACCAAACUCCUCAUC 5'

query=JS16_t0473973, target=FGSG_02937, score=4, range=635-652, strand=-1

target  5' CAGCGGUUUGAGGAGUGU 3'
           ::  ::::::::::::.
query   3' GUGACCAAACUCCUCAUC 5'

query=JS16_t0473973, target=FGSG_09665, score=4, range=1104-1120, strand=-1

target  5' CAC-GGUUUGAGGGGGAG 3'
           ::: :::::::::.: ::
query   3' GUGACCAAACUCCUCAUC 5'

query=JS16_t0473973, target=FGSG_07413, score=4, range=2626-2643, strand=-1

target  5' CAGGGGUUUCAGGAGUAG 3'
           ::  ::::: ::::::::
query   3' GUGACCAAACUCCUCAUC 5'

query=JS16_t0473973, target=FGSG_08941, score=4, range=334-352, strand=-1

target  5' CAUCAGGGUUGAGGAGUAG 3'
           :: : :: ::::::::::
query   3' GU-GACCAAACUCCUCAUC 5'

query=JS16_t0473973, target=FGSG_02117, score=4, range=647-663, strand=-1

target  5' GAGUGGUUUGAGGAG-AG 3'
            : ::::::::::: ::
query   3' GUGACCAAACUCCUCAUC 5'

query=JS16_t0473973, target=FGSG_05456, score=4, range=925-941, strand=-1

target  5' CCCU-GUUUGAGGAGAAG 3'
           : :: :::::::::: ::
query   3' GUGACCAAACUCCUCAUC 5'

query=JS16_t0473973, target=FGSG_08213, score=4, range=617-633, strand=-1

target  5' CUCU-GUUUGAGUAGUAG 3'
           : :: ::::::: :::::
query   3' GUGACCAAACUCCUCAUC 5'

query=JS16_t0473973, target=FGSG_10300, score=4, range=254-270, strand=-1

target  5' CACU-GUUUGAGGCGUAU 3'
           :::: :::::::: :::
query   3' GUGACCAAACUCCUCAUC 5'

No results for JS16_t2282183
No results for JS16_t3178306
No results for JS16_t3178340
query=JS16_t4074971, target=FGSG_10564, score=3.5, range=668-687, strand=-1
```

```
query    3' ACACGCGGUUGACGUUUG 5'

query=JS16_t1324635, target=FGSG_07622, score=4, range=1619-1636, strand=-1

target   5' UAUGUGCCAGCUGCAAGU 3'
            : ::.::::.::::::..
query    3' ACACGCGGUUGACGUUUG 5'

query=JS16_t1324635, target=FGSG_13865, score=4, range=1053-1070, strand=-1

target   5' UGUCCGCCAACGGCAAGC 3'
            ::: ::::::: ::::.:
query    3' ACACGCGGUUGACGUUUG 5'

query=JS16_t1324635, target=FGSG_07068, score=4, range=209-227, strand=-1

target   5' UGUCCAGCCGAUUGCAAAC 3'
            ::: : :::.:.:::::::
query    3' ACACG-CGGUUGACGUUUG 5'

query=JS16_t1324635, target=FGSG_00708, score=4, range=165-181, strand=-1

target   5' UG-GCGCCAAUUGCAAGA 3'
            :: :::::::.:::::.
query    3' ACACGCGGUUGACGUUUG 5'

query=JS16_t1324635, target=FGSG_01800, score=4, range=344-361, strand=-1

target   5' UUCGCGCCCACUGCAAAC 3'
            :  ::::: :::::::::
query    3' ACACGCGGUUGACGUUUG 5'

query=JS16_t1324635, target=FGSG_12989, score=4, range=166-184, strand=-1

target   5' UCAGCAGCCAACUGCAGAC 3'
            :  :: ::::::::::.::
query    3' ACACG-CGGUUGACGUUUG 5'

query=JS16_t1324635, target=FGSG_09441, score=4, range=2442-2459, strand=-1

target   5' UGAGACCCAACUGCAAAC 3'
            :: :  ::::::::::::
query    3' ACACGCGGUUGACGUUUG 5'

query=JS16_t2221032, target=FGSG_08439, score=4, range=932-949, strand=1

target   5' GCAUCAUUAAACUGUGGG 3'
            :  ::::::::::::.:
query    3' CACAGUAAUUUGACAUCA 5'

query=JS16_t2221032, target=FGSG_09878, score=4, range=100-118, strand=-1

target   5' GUCGUCGUUGAACUGUAGG 3'
            :: :::.::.::::::::
query    3' CA-CAGUAAUUUGACAUCA 5'

query=JS16_t2221032, target=FGSG_12602, score=4, range=608-626, strand=-1

target   5' GUGUCCAUUGAACUGGAGU 3'
            :::: ::::.::::: :::
query    3' CACA-GUAAUUUGACAUCA 5'

query=JS16_t2221032, target=FGSG_03830, score=4, range=249-266, strand=-1

target   5' CUACCAUUGAACUGUAGU 3'
             :  ::::.:::::::::
query    3' CACAGUAAUUUGACAUCA 5'

query=JS16_t2221032, target=FGSG_13851, score=4, range=261-279, strand=-1

target   5' AUGUGCAUUAAACUGUUGU 3'
             ::: :::::::::: ::
query    3' CACA-GUAAUUUGACAUCA 5'

query=JS16_t0268558, target=FGSG_06395, score=3, range=1578-1597, strand=1

target   5' AGUCAUGGUGUUUGUCCACU 3'
             :::: ::.::::::::::
query    3' GCAGU-CCGCAAACAGGUGA 5'

query=JS16_t0268558, target=FGSG_06284, score=3, range=2830-2848, strand=1

target   5' CGUGCGGCGUUUGUCCGCU 3'
            :::  :::::::::::.::
query    3' GCAGUCCGCAAACAGGUGA 5'

query=JS16_t0268558, target=FGSG_01162, score=4, range=1680-1699, strand=1

target   5' CGACAGAGCGUUUGUCCAUG 3'
            :: ::: :::::::::::.
query    3' GCAGUC-CGCAAACAGGUGA 5'

target   5' AAGGAAUACUCCACGAGGGA 3'
            ::::: .:::::::::::.
query    3' UUCCUAGUGAGGUGCUCCUC 5'

query=JS16_t4074971, target=FGSG_11428, score=3.5, range=240-258, strand=-1

target   5' GAGGAUGACUCCACGA-GAG 3'
            .::::: ::::::::: :::
query    3' UUCCUAGUGAGGUGCUCCUC 5'

query=JS16_t4074971, target=FGSG_00698, score=4, range=1073-1092, strand=1

target   5' GAUGGGCACUCCAUGAGGAG 3'
            .: :.  :::::::.::::::
query    3' UUCCUAGUGAGGUGCUCCUC 5'

query=JS16_t4074971, target=FGSG_08962, score=4, range=509-528, strand=-1

target   5' AGGGGUAACUCCUCGAGGAG 3'
            :.::.: ::::: :::::::
query    3' UUCCUAGUGAGGUGCUCCUC 5'

No results for JS16_t3178361
query=JS16_t3178363, target=FGSG_02863, score=3.5, range=345-365, strand=-1

target   5' GGAGCCUGAAGUCGAGUUCCA 3'
            :.:: : :::.::::::::::
query    3' CUUCAG-CUUUAGCUCAAGGU 5'

query=JS16_t3178363, target=FGSG_10456, score=4, range=673-692, strand=1

target   5' GAAGUCGAGGUCGAAUUCCA 3'
            ::::::::..:::: :::::
query    3' CUUCAGCUUUAGCUCAAGGU 5'

query=JS16_t3178363, target=FGSG_11140, score=4, range=1270-1289, strand=1

target   5' GAAGUACGAAUUGAGUUCCA 3'
            :::::  .:::.:::::::::
query    3' CUUCAGCUUUAGCUCAAGGU 5'

query=JS16_t3178363, target=FGSG_00253, score=4, range=160-179, strand=1

target   5' GAUGUUCAAAUUGAGUUCCG 3'
            :: ::.  ::::.::::::::.
query    3' CUUCAGCUUUAGCUCAAGGU 5'

query=JS16_t3178363, target=FGSG_12035, score=4, range=1172-1191, strand=-1

target   5' CAAAUCCAGAUCGAGUUCCA 3'
             :: :: :.::::::::::::
query    3' CUUCAGCUUUAGCUCAAGGU 5'

query=JS16_t3178363, target=FGSG_07860, score=4, range=343-361, strand=-1

target   5' GAAG-AGAAAUCGACUUCCA 3'
            ::::  :::::::: :::::
query    3' CUUCAGCUUUAGCUCAAGGU 5'

No results for JS16_t3178369
query=JS16_t0366666, target=FGSG_00070, score=4, range=439-463, strand=1

target   5' GCCUUCAUCUUCAUCACCUUCUUUG 3'
            ::::: ::: ::::::: :::::::
query    3' CGGAAUUAG-AGUAGUGCAAGAAAC 5'

query=JS16_t0220261, target=FGSG_07509, score=4, range=581-598, strand=1

target   5' GCG-GUUCUGUUUUUAGUU 3'
            ::: ::: ::::::::.:
query    3' CGCUCAACACAAAAAUCGA 5'

query=JS16_t0220261, target=FGSG_05269, score=4, range=1197-1215, strand=-1

target   5' UCGUGUUGUGUUUUUGGCG 3'
             :: :::::::::::.::
query    3' CGCUCAACACAAAAAUCGA 5'

query=JS16_t0220261, target=FGSG_09373, score=4, range=673-692, strand=-1

target   5' UCGAGGUUGUGUUUUUGGCC 3'
             ::: :::::::::::.::
query    3' CGCU-CAACACAAAAAUCGA 5'

query=JS16_t0220261, target=FGSG_10074, score=4, range=192-211, strand=-1

target   5' GCGAAGGUGUGUUUAUAGCU 3'
            ::: :: ::::::: :::::
query    3' CGC-UCAACACAAAAAUCGA 5'

query=JS16_t3178397, target=FGSG_00267, score=2.5, range=292-310, strand=-1
```

```
query=JS16_t0268558, target=FGSG_10416, score=4, range=1209-1226, strand=1

target  5’ UG-CGGGCGUUUUUCCACU 3’
           .: :.::::::: ::::::
query   3’ GCAGUCCGCAAACAGGUGA 5’

query=JS16_t0268558, target=FGSG_06653, score=4, range=3379-3397, strand=-1

target  5’ CCACAGGCGUUUGUCCAGU 3’
           :  ::::::::::::: :
query   3’ GCAGUCCGCAAACAGGUGA 5’

query=JS16_t0268558, target=FGSG_05751, score=4, range=4447-4465, strand=-1

target  5’ CAUCACGCGUUUGUCCAAU 3’
           : ::: ::::::::::: :
query   3’ GCAGUCCGCAAACAGGUGA 5’

query=JS16_t0268558, target=FGSG_06724, score=4, range=149-167, strand=-1

target  5’ CGGAAGGCCUUUGUCCACU 3’
           ::  :::: ::::::::::
query   3’ GCAGUCCGCAAACAGGUGA 5’

No results for JS16_t1324669
No results for JS16_t4013932
query=JS16_t3117139, target=FGSG_11545, score=3, range=541-558, strand=1

target  5’ UGGAA-GGAUGGUAAAGAU 3’
           :.::: :::::.::::::.
query   3’ AUCUUGCCUACUAUUUCUG 5’

query=JS16_t3117139, target=FGSG_10866, score=3, range=202-221, strand=-1

target  5’ UAGAGACGGGUGAUGAAGAC 3’
           :::: ::::.::::.:::::
query   3’ AUCU-UGCCUACUAUUUCUG 5’

query=JS16_t3117139, target=FGSG_10196, score=3, range=1075-1092, strand=-1

target  5’ UAG-ACGGAUGAUAAAGGG 3’
           ::: :::::::::::::.
query   3’ AUCUUGCCUACUAUUUCUG 5’

query=JS16_t3117139, target=FGSG_03675, score=3, range=1153-1170, strand=-1

target  5’ UA-AAAGGAUGAUGAAGAC 3’
           :: :: :::::::.:::::
query   3’ AUCUUGCCUACUAUUUCUG 5’

query=JS16_t3117139, target=FGSG_14016, score=3.5, range=639-657, strand=1

target  5’ UAGAGCGAAUGAUGAAGAC 3’
           ::::.:: :::::.:::::
query   3’ AUCUUGCCUACUAUUUCUG 5’

query=JS16_t3117139, target=FGSG_09939, score=3.5, range=728-747, strand=1

target  5’ UGGCAUCGGAUGAUGAAGAC 3’
           :.: : ::::::::.:::::
query   3’ AUC-UUGCCUACUAUUUCUG 5’

query=JS16_t3117139, target=FGSG_13625, score=3.5, range=735-753, strand=-1

target  5’ UAGAGCGGAUGAUAAAGUA 3’
           ::::.::::::::::::
query   3’ AUCUUGCCUACUAUUUCUG 5’

query=JS16_t3117139, target=FGSG_07202, score=3.5, range=118-137, strand=-1

target  5’ UAGGUCGGAUGAUAAAGCAC 3’
           :::. :::::::::::: ::
query   3’ AUCUUGCCUACUAUUUC-UG 5’

query=JS16_t3117139, target=FGSG_06776, score=3.5, range=1173-1190, strand=-1

target  5’ UGGAA-GGAUGAUAAAGCC 3’
           :.::: ::::::::::: :
query   3’ AUCUUGCCUACUAUUUCUG 5’

query=JS16_t3117139, target=FGSG_04368, score=4, range=990-1008, strand=1

target  5’ UGGAGCGGUUGGUAAAGAC 3’
           :.::.::: ::.:::::::
query   3’ AUCUUGCCUACUAUUUCUG 5’

query=JS16_t3117139, target=FGSG_11657, score=4, range=482-500, strand=1

target  5’ AAGAAGGGAUGAUGGAGAC 3’
            :::: :::::::..::::
query   3’ AUCUUGCCUACUAUUUCUG 5’

target  5’ GUAUAGGAAAGUCAUCGCU 3’
           :: .::::::::::::.::
query   3’ CAAGUCCUUUCAGUAGUGA 5’

query=JS16_t3178397, target=FGSG_14021, score=3, range=1240-1258, strand=-1

target  5’ CUUGAAGAAAGUCAUCACU 3’
            :: : :::::::::::::
query   3’ CAAGUCCUUUCAGUAGUGA 5’

query=JS16_t3178397, target=FGSG_10738, score=4, range=264-282, strand=1

target  5’ GUUCAAGAAAGUCAACGCU 3’
           ::::: :::::::: :.::
query   3’ CAAGUCCUUUCAGUAGUGA 5’

query=JS16_t3178397, target=FGSG_10189, score=4, range=314-331, strand=1

target  5’ GUUCUGGAAAGUC-UUACU 3’
           :::: :::::::: :.:::
query   3’ CAAGUCCUUUCAGUAGUGA 5’

query=JS16_t3178397, target=FGSG_04128, score=4, range=67-84, strand=1

target  5’ GAUCA-GAAAGUCAUUGCU 3’
           : ::: ::::::::::..::
query   3’ CAAGUCCUUUCAGUAGUGA 5’

query=JS16_t3178397, target=FGSG_12276, score=4, range=1000-1018, strand=-1

target  5’ AUUCAGGAAGGUCAUCGCC 3’
            ::::::::.:::::::.:
query   3’ CAAGUCCUUUCAGUAGUGA 5’

query=JS16_t3178397, target=FGSG_06070, score=4, range=686-703, strand=-1

target  5’ GUUCAGGAAGGUCGUC-CU 3’
           :::::::::.:::.:: ::
query   3’ CAAGUCCUUUCAGUAGUGA 5’

query=JS16_t3178397, target=FGSG_09673, score=4, range=1212-1229, strand=-1

target  5’ GUUC-GGAAAGUCAUUGUU 3’
           :::: ::::::::::...:
query   3’ CAAGUCCUUUCAGUAGUGA 5’

query=JS16_t3178397, target=FGSG_12890, score=4, range=247-266, strand=-1

target  5’ GUCUUGGGAAAGUCAUCUCU 3’
           :: :..:::::::::::: ::
query   3’ CA-AGUCCUUUCAGUAGUGA 5’

query=JS16_t3178397, target=FGSG_01629, score=4, range=1042-1060, strand=-1

target  5’ CUUCACGAGAGUCAUCACG 3’
            :::: ::.:::::::::
query   3’ CAAGUCCUUUCAGUAGUGA 5’

query=JS16_t3178397, target=FGSG_07848, score=4, range=316-333, strand=-1

target  5’ UUUCAGG-AAGUCAUCGCU 3’
            :::::: ::::::::.::
query   3’ CAAGUCCUUUCAGUAGUGA 5’

No results for JS16_t4075048
No results for JS16_t1386262
No results for JS16_t2282442
No results for JS16_t3178551
No results for JS16_t3178558
query=JS16_t0474027, target=FGSG_12499, score=2.5, range=242-262, strand=-1

target  5’ AUAUCGGCCCUUUACCCAGAA 3’
            .::::::::.::::::::::
query   3’ CGUAGCCGGGGAAUGGGUCUU 5’

query=JS16_t0474027, target=FGSG_06195, score=2.5, range=1127-1146, strand=-1

target  5’ GCAUUUG-CCCUUACCCAGAA 3’
           ::::. : :::::::::::::
query   3’ CGUAGCCGGGGAAUGGGUCUU 5’

query=JS16_t0474027, target=FGSG_08472, score=4, range=354-373, strand=1

target  5’ CCAU-GGCCCCUUGCCCAGAC 3’
            ::: ::::::::.::::::
query   3’ CGUAGCCGGGGAAUGGGUCUU 5’

query=JS16_t0474027, target=FGSG_04063, score=4, range=727-747, strand=-1

target  5’ GUAUUUGCCCGUUACCCAGAA 3’
           :.::. :::: ::::::::::
query   3’ CGUAGCCGGGGAAUGGGUCUU 5’
```

```
query=JS16_t3117139, target=FGSG_00716, score=4, range=1509-1527, strand=1

target  5’ UGGGUCGGGUGAUAAAGAG 3’
           :.:. :::.:::::::::
query   3’ AUCUUGCCUACUAUUUCUG 5’

query=JS16_t3117139, target=FGSG_00738, score=4, range=249-267, strand=1

target  5’ UGGAGAGGAUGAGAAAGAC 3’
           :.::. :::::: ::::::
query   3’ AUCUUGCCUACUAUUUCUG 5’

query=JS16_t3117139, target=FGSG_08316, score=4, range=1618-1635, strand=1

target  5’ GAGGA-GGAUGAUAGAGAU 3’
            ::.: ::::::::.:::.
query   3’ AUCUUGCCUACUAUUUCUG 5’

query=JS16_t3117139, target=FGSG_09112, score=4, range=699-717, strand=1

target  5’ CAGACCAGAUGAUAAAGAC 3’
            ::: : ::::::::::::
query   3’ AUCUUGCCUACUAUUUCUG 5’

query=JS16_t3117139, target=FGSG_00592, score=4, range=1294-1311, strand=1

target  5’ UUGAACGGA-GAUGAAGAC 3’
           : ::::::: :::.:::::
query   3’ AUCUUGCCUACUAUUUCUG 5’

query=JS16_t3117139, target=FGSG_10318, score=4, range=766-784, strand=-1

target  5’ AAGGACGGAUGGUGAAGAU 3’
            ::.:::::::.:.::::.
query   3’ AUCUUGCCUACUAUUUCUG 5’

query=JS16_t3117139, target=FGSG_01425, score=4, range=2431-2449, strand=-1

target  5’ UGGCACGGGUGAUGAAGAU 3’
           :.: ::::.::::.::::.
query   3’ AUCUUGCCUACUAUUUCUG 5’

query=JS16_t3117139, target=FGSG_08009, score=4, range=598-616, strand=-1

target  5’ UGGCAAGGAUGAUAGAGAU 3’
           :.: : ::::::::.:::.
query   3’ AUCUUGCCUACUAUUUCUG 5’

query=JS16_t3117139, target=FGSG_08840, score=4, range=2188-2206, strand=-1

target  5’ CGGCCUGGAUGAUAAAGAC 3’
            .:  .:::::::::::::
query   3’ AUCUUGCCUACUAUUUCUG 5’

query=JS16_t3117139, target=FGSG_02874, score=4, range=792-809, strand=-1

target  5’ GAGAA-GGAUGAUAGAGAA 3’
            :::: ::::::::.:::
query   3’ AUCUUGCCUACUAUUUCUG 5’

query=JS16_t3117139, target=FGSG_13275, score=4, range=742-761, strand=-1

target  5’ UACAACAUGAUGAUAAAGAC 3’
           :: :::  ::::::::::::
query   3’ AUCUUG-CCUACUAUUUCUG 5’

query=JS16_t3117139, target=FGSG_07213, score=4, range=154-171, strand=-1

target  5’ GAGAA-GGAUGAUAAUGAC 3’
            :::: ::::::::: :::
query   3’ AUCUUGCCUACUAUUUCUG 5’

query=JS16_t0466626, target=FGSG_08829, score=3.5, range=319-339, strand=-1

target  5’ UCCUGGUGAAGCUUGCGGGAA 3’
           :  :::.:::::::::::.::
query   3’ ACAACCGCUUCGAACGCCUUU 5’

query=JS16_t0466626, target=FGSG_07174, score=4, range=504-523, strand=-1

target  5’ GGUU-GCGAAGCUUGCGGUAA 3’
            ::: ::::::::::::: ::
query   3’ ACAACCGCUUCGAACGCCUUU 5’

query=JS16_t0466626, target=FGSG_10743, score=4, range=414-433, strand=-1

target  5’ UGUU-ACAGAGCUUGCGGAAA 3’
           ::::  : .::::::::::::
query   3’ ACAACCGCUUCGAACGCCUUU 5’

query=JS16_t0466626, target=FGSG_06139, score=4, range=1152-1171, strand=-1

No results for JS16_t2282512
No results for JS16_t0166637
No results for JS16_t3178653
query=JS16_t1386460, target=FGSG_13971, score=4, range=1465-1484, strand=1

target  5’ AACAGUGGACUUUC-CGAAAU 3’
           ::: :: ::::::: ::::::
query   3’ UUGGCAACUGAAAGAGCUUUA 5’

query=JS16_t1386460, target=FGSG_02781, score=4, range=3491-3511, strand=-1

target  5’ CGUCGUUGACUUUCUCGGAAA 3’
            ..::::::::::::::.::
query   3’ UUGGCAACUGAAAGAGCUUUA 5’

No results for JS16_t3178679
query=JS16_t2282587, target=FGSG_10339, score=3.5, range=360-379, strand=-1

target  5’ GAGUCGAACAGCGGUAUGGG 3’
           .:..:::::::::::::: :
query   3’ UUUGGCUUGUCGCCAUACGC 5’

query=JS16_t2282587, target=FGSG_06754, score=4, range=3405-3423, strand=1

target  5’ AUACCGAACAGCGGU-UGCA 3’
           : ::::::::::::: :::
query   3’ UUUGGCUUGUCGCCAUACGC 5’

query=JS16_t2282587, target=FGSG_06156, score=4, range=1022-1040, strand=-1

target  5’ AAAUUG-ACAGCGGUACGCG 3’
           :::..: ::::::::: :::
query   3’ UUUGGCUUGUCGCCAUACGC 5’

query=JS16_t2282619, target=FGSG_08858, score=3.5, range=37-56, strand=-1

target  5’ GCAGGUGAUCGAAAAGGUAA 3’
           . ::::::::: ::::::::
query   3’ UAUCCACUAGCCUUUCCAUU 5’

query=JS16_t2282619, target=FGSG_02833, score=4, range=1402-1421, strand=1

target  5’ GAGGGAGAUUGGAAAGGUAA 3’
           . .:: :::.::::::::::
query   3’ UAUCCACUAGCCUUUCCAUU 5’

query=JS16_t2282619, target=FGSG_00347, score=4, range=234-254, strand=-1

target  5’ AUAGGGCGAUCGGAAAGGUCA 3’
           ::: :: :::::::::::: :
query   3’ UAU-CCACUAGCCUUUCCAUU 5’

query=JS16_t4075433, target=FGSG_05757, score=4, range=562-581, strand=1

target  5’ GCCAAGGGUGGAUCUGCUUU 3’
           :: : :::::.:.:::::::
query   3’ CGCUGCCCACUUGGACGAAA 5’

query=JS16_t4075433, target=FGSG_01886, score=4, range=992-1010, strand=1

target  5’ CCGA-GGGUGAACCUGCUGU 3’
            ::: ::::::::::::: :
query   3’ CGCUGCCCACUUGGACGAAA 5’

No results for JS16_t4075446
No results for JS16_t0228937
query=JS16_t0166639, target=FGSG_00020, score=3, range=2446-2464, strand=1

target  5’ CAGUACGGUGAACUGGACA 3’
           ::::::::::::::.: ::
query   3’ GUCAUGCCACUUGAUCAGU 5’

query=JS16_t0166639, target=FGSG_10203, score=4, range=897-916, strand=1

target  5’ GAGUGAUGGUGGACUAGUCG 3’
            ::: :.::::.:::::::.
query   3’ GUCA-UGCCACUUGAUCAGU 5’

query=JS16_t0166639, target=FGSG_11725, score=4, range=188-205, strand=1

target  5’ CAG-ACGGUGAAUUAGGCA 3’
           ::: ::::::::.::: ::
query   3’ GUCAUGCCACUUGAUCAGU 5’

query=JS16_t0166639, target=FGSG_07512, score=4, range=534-551, strand=-1

target  5’ AAGUG-GGUGAACUGGUCG 3’
            :::. :::::::::.:::.
query   3’ GUCAUGCCACUUGAUCAGU 5’

query=JS16_t0166639, target=FGSG_05514, score=4, range=1710-1728, strand=-1
```

```
target  5’ UCUU-GUUAAGCUUGCGGAAG 3’
           : :: :. :::::::::::.
query   3’ ACAACCGCUUCGAACGCCUUU 5’

No results for JS16_t3117146
query=JS16_t4013978, target=FGSG_08812, score=2, range=2644-2661, strand=1

target  5’ GAGGAAAUCAAGAUCGAA 3’
           : ::::::::::::.::
query   3’ CGCCUUUAGUUCUAGUUU 5’

query=JS16_t4013978, target=FGSG_11250, score=2.5, range=1010-1028, strand=-1

target  5’ GCGGAAAUCAAGAUACAAG 3’
           ::::::::::::: :::.
query   3’ CGCCUUUAGUUCUA-GUUU 5’

query=JS16_t4013978, target=FGSG_01165, score=3, range=322-339, strand=1

target  5’ GCGGCAAUCAAGAUCACA 3’
           :::: ::::::::::: :
query   3’ CGCCUUUAGUUCUAGUUU 5’

query=JS16_t4013978, target=FGSG_11551, score=3, range=140-157, strand=1

target  5’ GUGUUAAUCAAGAUCAAG 3’
           :.:  :::::::::::.
query   3’ CGCCUUUAGUUCUAGUUU 5’

query=JS16_t4013978, target=FGSG_00142, score=3, range=542-558, strand=1

target  5’ GC-GCAAUCAAGAUCAAC 3’
           :: : ::::::::::::
query   3’ CGCCUUUAGUUCUAGUUU 5’

query=JS16_t4013978, target=FGSG_08758, score=3, range=1017-1034, strand=-1

target  5’ AUGGGAAUCAAGAUCAGA 3’
            .::.:::::::::::.:
query   3’ CGCCUUUAGUUCUAGUUU 5’

query=JS16_t4013978, target=FGSG_02359, score=3, range=595-611, strand=-1

target  5’ UCGGAAAU-AAGAUCAAA 3’
            ::::::: :::::::::
query   3’ CGCCUUUAGUUCUAGUUU 5’

query=JS16_t4013978, target=FGSG_12595, score=3.5, range=3040-3057, strand=1

target  5’ ACGGAAAUCAAGAUCGGG 3’
            ::::::::::::::...
query   3’ CGCCUUUAGUUCUAGUUU 5’

query=JS16_t4013978, target=FGSG_01251, score=3.5, range=1474-1491, strand=1

target  5’ CCGGAAGUCAAGAUCGAG 3’
            :::::.::::::::.:.
query   3’ CGCCUUUAGUUCUAGUUU 5’

query=JS16_t4013978, target=FGSG_02834, score=3.5, range=1207-1224, strand=1

target  5’ GUUGAAGUUAAGAUCAAA 3’
           :.  :::.:.:::::::::
query   3’ CGCCUUUAGUUCUAGUUU 5’

query=JS16_t4013978, target=FGSG_13662, score=3.5, range=601-618, strand=1

target  5’ CCGGAAAGCAAGAUCAAG 3’
            :::::: :::::::::.
query   3’ CGCCUUUAGUUCUAGUUU 5’

query=JS16_t4013978, target=FGSG_07041, score=3.5, range=200-216, strand=1

target  5’ GC-GAGAUCAAGAUCGAG 3’
           :: ::.::::::::::.:.
query   3’ CGCCUUUAGUUCUAGUUU 5’

query=JS16_t4013978, target=FGSG_07063, score=3.5, range=482-498, strand=1

target  5’ GC-GAAAUCGAGAUCGAG 3’
           :: ::::::.:::::.:.
query   3’ CGCCUUUAGUUCUAGUUU 5’

query=JS16_t4013978, target=FGSG_14013, score=3.5, range=104-121, strand=1

target  5’ GACGAGAUCAAGAUCAAG 3’
           :  ::.:::::::::::.
query   3’ CGCCUUUAGUUCUAGUUU 5’

query=JS16_t4013978, target=FGSG_02636, score=3.5, range=1402-1419, strand=1

target  5’ GCUGAAUUCAAGAUCAAG 3’
```

```
target  5’ CAUUUCGGUGAACUUGUCA 3’
           :: : ::::::::: ::::
query   3’ GUCAUGCCACUUGAUCAGU 5’

No results for JS16_t3178895
query=JS16_t0255357, target=FGSG_14031, score=4, range=484-505, strand=1

target  5’ AGCGCUCGUGAGGAUGAAGUUC 3’
            ::: :  ::::::::::::::
query   3’ ACGCCAUGACUCCUACUUCAAG 5’

query=JS16_t0255357, target=FGSG_06515, score=4, range=540-561, strand=-1

target  5’ UGCCAUGUCGAGGAUGAAGUUC 3’
           :::  :.. :::::::::::::
query   3’ ACGCCAUGACUCCUACUUCAAG 5’

No results for JS16_t3178965
query=JS16_t2282834, target=FGSG_03960, score=2.5, range=308-324, strand=-1

target  5’ UUUUCCAACA-CUCAGGC 3’
           :::.::::::: :::::::
query   3’ AAAGGGUUGUGGAGUCCG 5’

query=JS16_t2282834, target=FGSG_00615, score=3, range=520-537, strand=1

target  5’ CGUCCCAGCACCUCAGGC 3’
             :::::.::::::::::
query   3’ AAAGGGUUGUGGAGUCCG 5’

query=JS16_t2282834, target=FGSG_04202, score=3, range=1275-1292, strand=-1

target  5’ UUUCGCAGCACCUCGGGC 3’
           :::: ::.::::::.:::
query   3’ AAAGGGUUGUGGAGUCCG 5’

query=JS16_t2282834, target=FGSG_05233, score=3.5, range=2186-2204, strand=1

target  5’ CUUCUCAAUCACCUCAGGC 3’
            :::.::: ::::::::::
query   3’ AAAGGGUU-GUGGAGUCCG 5’

query=JS16_t2282834, target=FGSG_08422, score=3.5, range=73-91, strand=1

target  5’ UUUGCCAACACCUCAUGGU 3’
           ::: ::::::::::: ::.
query   3’ AAAGGGUUGUGGAGU-CCG 5’

query=JS16_t2282834, target=FGSG_07064, score=3.5, range=1644-1661, strand=-1

target  5’ UCUUCCAGCACCUCAGGG 3’
           : :.:::.:::::::::
query   3’ AAAGGGUUGUGGAGUCCG 5’

query=JS16_t2282834, target=FGSG_04748, score=3.5, range=324-342, strand=-1

target  5’ UUUCUGAACACCUCGAGGC 3’
           ::::. :::::::: ::::
query   3’ AAAGGGUUGUGGAG-UCCG 5’

query=JS16_t2282834, target=FGSG_02517, score=3.5, range=153-169, strand=-1

target  5’ UAUCCCAAC-CCUCAGGU 3’
           : ::::::: :::::::.
query   3’ AAAGGGUUGUGGAGUCCG 5’

query=JS16_t2282834, target=FGSG_11340, score=4, range=1132-1149, strand=1

target  5’ UUUCUCAGCACCUCAGAU 3’
           ::::.::.:::::::: .
query   3’ AAAGGGUUGUGGAGUCCG 5’

query=JS16_t2282834, target=FGSG_01184, score=4, range=433-451, strand=1

target  5’ UUUUUCAACGACUUCAGGC 3’
           :::..:::: ::.::::::
query   3’ AAAGGGUUG-UGGAGUCCG 5’

query=JS16_t2282834, target=FGSG_02651, score=4, range=654-672, strand=1

target  5’ UUUCCACAGCAUCUCGGGC 3’
           ::::: ::.::.:::.:::
query   3’ AAAGG-GUUGUGGAGUCCG 5’

query=JS16_t2282834, target=FGSG_01927, score=4, range=1372-1389, strand=1

target  5’ UUCCUUAACACCUCGGGU 3’
           :: :..::::::::.::.
query   3’ AAAGGGUUGUGGAGUCCG 5’

query=JS16_t2282834, target=FGSG_06944, score=4, range=405-422, strand=1
```

```
           :: ::: ::::::::::.
query   3' CGCCUUUAGUUCUAGUUU 5'

query=JS16_t4013978, target=FGSG_09801, score=3.5, range=1003-1020, strand=1

target  5' GAGCAAGUCAAGAUCAAG 3'
           : : ::.::::::::::.
query   3' CGCCUUUAGUUCUAGUUU 5'

query=JS16_t4013978, target=FGSG_01827, score=3.5, range=1336-1353, strand=1

target  5' GCAAGAAUCAAGAUCGAA 3'
           ::  .::::::::::.::
query   3' CGCCUUUAGUUCUAGUUU 5'

query=JS16_t4013978, target=FGSG_03525, score=3.5, range=92-109, strand=1

target  5' GCGACAAUCGAGAUCAAG 3'
           :::  ::::.:::::::.
query   3' CGCCUUUAGUUCUAGUUU 5'

query=JS16_t4013978, target=FGSG_11620, score=3.5, range=287-304, strand=1

target  5' CCAAAAAUCAAGAUCAAG 3'
            :  :::::::::::::.
query   3' CGCCUUUAGUUCUAGUUU 5'

query=JS16_t4013978, target=FGSG_13366, score=3.5, range=199-216, strand=-1

target  5' ACGGGAAUCAAGAUUGAA 3'
            :::.:::::::::..::
query   3' CGCCUUUAGUUCUAGUUU 5'

query=JS16_t4013978, target=FGSG_12433, score=3.5, range=328-345, strand=-1

target  5' GUCGAAAUCGGGAUCAAA 3'
           :. ::::::..:::::::
query   3' CGCCUUUAGUUCUAGUUU 5'

query=JS16_t4013978, target=FGSG_10712, score=3.5, range=127-144, strand=-1

target  5' GUGUAAAUCAAGAUCGAC 3'
           :.: :::::::::::.:
query   3' CGCCUUUAGUUCUAGUUU 5'

query=JS16_t4013978, target=FGSG_11294, score=3.5, range=487-503, strand=-1

target  5' GUGG-AGUCAAGAUCAAC 3'
           :.:: :.::::::::::
query   3' CGCCUUUAGUUCUAGUUU 5'

query=JS16_t4013978, target=FGSG_04008, score=4, range=361-378, strand=1

target  5' GCGGAGAUAGAGAUCAAA 3'
           :::::.:: .::::::::
query   3' CGCCUUUAGUUCUAGUUU 5'

query=JS16_t4013978, target=FGSG_13776, score=4, range=1064-1081, strand=1

target  5' GCGGCAGUCGAGAUCAGA 3'
           :::: :.::.::::::.:
query   3' CGCCUUUAGUUCUAGUUU 5'

query=JS16_t4013978, target=FGSG_13092, score=4, range=768-785, strand=1

target  5' GCGGGCAUCAAGAUCGAG 3'
           ::::.  :::::::::.:.
query   3' CGCCUUUAGUUCUAGUUU 5'

query=JS16_t4013978, target=FGSG_07521, score=4, range=382-399, strand=1

target  5' CCGGAAAUCAAGAUCGGU 3'
            ::::::::::::::..
query   3' CGCCUUUAGUUCUAGUUU 5'

query=JS16_t4013978, target=FGSG_06361, score=4, range=3049-3066, strand=1

target  5' GUCGAAGUCGAGAUCAAG 3'
           :. :::.::.:::::::.
query   3' CGCCUUUAGUUCUAGUUU 5'

query=JS16_t4013978, target=FGSG_03629, score=4, range=2419-2436, strand=1

target  5' GAGGGAGUCAAGAUUAAG 3'
           : ::.:.:::::::.::.
query   3' CGCCUUUAGUUCUAGUUU 5'

query=JS16_t4013978, target=FGSG_11840, score=4, range=310-327, strand=1

target  5' GUGGUGAUUAAGAUCAAG 3'
           :.:: .::.::::::::.
query   3' CGCCUUUAGUUCUAGUUU 5'

target  5' UUUCCCAACACCUCCUGC 3'
           ::::::::::::::  ::
query   3' AAAGGGUUGUGGAGUCCG 5'

query=JS16_t2282834, target=FGSG_13512, score=4, range=111-128, strand=1

target  5' UCGCCUAACACCUCGGGC 3'
           :  ::.::::::::.:::
query   3' AAAGGGUUGUGGAGUCCG 5'

query=JS16_t2282834, target=FGSG_11055, score=4, range=623-640, strand=1

target  5' UUGUUCAACACCUCAGCC 3'
           :: ..:::::::::: :
query   3' AAAGGGUUGUGGAGUCCG 5'

query=JS16_t2282834, target=FGSG_05028, score=4, range=1086-1104, strand=1

target  5' UCUCUGCAACAUCUCAGGU 3'
           : ::. :::::.::::::.
query   3' AAAGG-GUUGUGGAGUCCG 5'

query=JS16_t2282834, target=FGSG_04015, score=4, range=406-423, strand=1

target  5' UACCCCAACAACUCAGGC 3'
           :  ::::::: :::::::
query   3' AAAGGGUUGUGGAGUCCG 5'

query=JS16_t2282834, target=FGSG_06661, score=4, range=1501-1517, strand=1

target  5' UUCCUCAAC-CCUCAGGU 3'
           :: :.:::: :::::::.
query   3' AAAGGGUUGUGGAGUCCG 5'

query=JS16_t2282834, target=FGSG_09637, score=4, range=849-866, strand=1

target  5' UUUAACAACACCUCAGAC 3'
           :::  :::::::::: :
query   3' AAAGGGUUGUGGAGUCCG 5'

query=JS16_t2282834, target=FGSG_09550, score=4, range=37-54, strand=-1

target  5' CUUCCCAACGCCUUGGGC 3'
            ::::::::.:::..:::
query   3' AAAGGGUUGUGGAGUCCG 5'

query=JS16_t2282834, target=FGSG_03254, score=4, range=297-314, strand=-1

target  5' UUUACCAACAACUCAGGA 3'
           ::: :::::: ::::::
query   3' AAAGGGUUGUGGAGUCCG 5'

query=JS16_t2282834, target=FGSG_10380, score=4, range=603-619, strand=-1

target  5' UAUCCCAA-ACCUCAGGG 3'
           : :::::: ::::::::
query   3' AAAGGGUUGUGGAGUCCG 5'

query=JS16_t2282834, target=FGSG_13445, score=4, range=1296-1312, strand=-1

target  5' UCUCCCAACG-CUCAGGC 3'
           : :::::::. :::::::
query   3' AAAGGGUUGUGGAGUCCG 5'

No results for JS16_t0366688
No results for JS16_t2282869
query=JS16_t2282905, target=FGSG_03456, score=2, range=274-290, strand=1

target  5' GAUG-GCCAGGUUUUGCA 3'
           .:.: :::::::::::::
query   3' UUGCACGGUCCAAAACGU 5'

query=JS16_t2282905, target=FGSG_05587, score=2, range=837-853, strand=-1

target  5' GAUG-GCCAGGUUUUGCA 3'
           .:.: :::::::::::::
query   3' UUGCACGGUCCAAAACGU 5'

query=JS16_t2282905, target=FGSG_02748, score=2.5, range=1870-1887, strand=1

target  5' GUCGUGUCAGGUUUUGCA 3'
           .  ::::.::::::::::
query   3' UUGCACGGUCCAAAACGU 5'

query=JS16_t2282905, target=FGSG_12863, score=3, range=3739-3755, strand=1

target  5' AAC-UGGCAGGUUUUGCA 3'
           ::: :: :::::::::::
query   3' UUGCACGGUCCAAAACGU 5'

query=JS16_t2282905, target=FGSG_07093, score=3, range=666-682, strand=-1
```

```
query=JS16_t4013978, target=FGSG_06716, score=4, range=1816-1833, strand=1

target  5’ AUGGAAAUCAAGAUGAAG 3’
            .:::::::::::: ::.
query   3’ CGCCUUUAGUUCUAGUUU 5’

query=JS16_t4013978, target=FGSG_02630, score=4, range=751-768, strand=1

target  5’ UUGGGCAUCAAGAUCAAA 3’
            .::. ::::::::::::
query   3’ CGCCUUUAGUUCUAGUUU 5’

query=JS16_t4013978, target=FGSG_08101, score=4, range=877-894, strand=1

target  5’ AAAGAAGUCAAGAUCAAA 3’
              :::.:::::::::::
query   3’ CGCCUUUAGUUCUAGUUU 5’

query=JS16_t4013978, target=FGSG_12337, score=4, range=124-141, strand=1

target  5’ CAAGAAAUCAAGAUCAGA 3’
              :::::::::::::.:
query   3’ CGCCUUUAGUUCUAGUUU 5’

query=JS16_t4013978, target=FGSG_00612, score=4, range=643-661, strand=1

target  5’ ACGUGGGAUCAAGAUCAAG 3’
            :: :..:::::::::::.
query   3’ CGC-CUUUAGUUCUAGUUU 5’

query=JS16_t4013978, target=FGSG_01042, score=4, range=616-633, strand=1

target  5’ GAGGAGAUCAUGAUCAAA 3’
           : :::.:::: :::::::
query   3’ CGCCUUUAGUUCUAGUUU 5’

query=JS16_t4013978, target=FGSG_12390, score=4, range=493-510, strand=1

target  5’ GCUGAGAUCAAGAGCAAA 3’
           :: ::.::::::: ::::
query   3’ CGCCUUUAGUUCUAGUUU 5’

query=JS16_t4013978, target=FGSG_11170, score=4, range=256-273, strand=1

target  5’ GCUCAAAUCGAGGUCAAA 3’
           ::  :::::.::.:::::
query   3’ CGCCUUUAGUUCUAGUUU 5’

query=JS16_t4013978, target=FGSG_07631, score=4, range=955-973, strand=1

target  5’ GCUGGAAUCAAGGAUCAAG 3’
           :: :.:::::: ::::::.
query   3’ CGCCUUUAGUU-CUAGUUU 5’

query=JS16_t4013978, target=FGSG_00096, score=4, range=581-598, strand=1

target  5’ UCGAAGAUCAAGAUCAAU 3’
            :: :.:::::::::::
query   3’ CGCCUUUAGUUCUAGUUU 5’

query=JS16_t4013978, target=FGSG_13779, score=4, range=170-187, strand=1

target  5’ GUCAAGAUCAAGAUCAAG 3’
           :.  :.:::::::::::.
query   3’ CGCCUUUAGUUCUAGUUU 5’

query=JS16_t4013978, target=FGSG_01249, score=4, range=512-529, strand=1

target  5’ ACUUAGAUCAAGAUCAAA 3’
            :  :.::::::::::::
query   3’ CGCCUUUAGUUCUAGUUU 5’

query=JS16_t4013978, target=FGSG_00858, score=4, range=1320-1338, strand=1

target  5’ UCGUGAAAUGAAGAUCAAA 3’
            :: ::::: :::::::::
query   3’ CGC-CUUUAGUUCUAGUUU 5’

query=JS16_t4013978, target=FGSG_07569, score=4, range=116-132, strand=1

target  5’ GC-GAAAUCAAGACCGAA 3’
           :: :::::::::: :.::
query   3’ CGCCUUUAGUUCUAGUUU 5’

query=JS16_t4013978, target=FGSG_09710, score=4, range=344-360, strand=1

target  5’ GCGG-AAUCAAAGUCAAA 3’
           :::: :::::: .:::::
query   3’ CGCCUUUAGUUCUAGUUU 5’

query=JS16_t4013978, target=FGSG_13777, score=4, range=738-756, strand=1

target  5’ AAGGUGCCA-GUUUUGCA 3’
           :: :::::: ::::::::
query   3’ UUGCACGGUCCAAAACGU 5’

query=JS16_t2282905, target=FGSG_03606, score=3.5, range=1478-1495, strand=1

target  5’ AAAGUGCCAUGUUUUGCG 3’
           :: :::::: :::::::.
query   3’ UUGCACGGUCCAAAACGU 5’

query=JS16_t2282905, target=FGSG_05990, score=3.5, range=4344-4361, strand=1

target  5’ AAUCCGCCAGGUUUUGCU 3’
           ::.  ::::::::::::
query   3’ UUGCACGGUCCAAAACGU 5’

query=JS16_t2282905, target=FGSG_08664, score=3.5, range=191-208, strand=-1

target  5’ GACGAGACAGGUUUUGCA 3’
           .::: : :::::::::::
query   3’ UUGCACGGUCCAAAACGU 5’

query=JS16_t2282905, target=FGSG_04278, score=3.5, range=1295-1311, strand=-1

target  5’ AGC-UGCCAAGUUUUGCA 3’
           :.: ::::: ::::::::
query   3’ UUGCACGGUCCAAAACGU 5’

query=JS16_t2282905, target=FGSG_12236, score=4, range=358-375, strand=1

target  5’ AACGUGCCAGAUUUUGAA 3’
           :::::::::: ::::: :
query   3’ UUGCACGGUCCAAAACGU 5’

query=JS16_t2282905, target=FGSG_10030, score=4, range=176-193, strand=1

target  5’ GGCCUGCCAGGUUUUCCA 3’
           ..: ::::::::::: ::
query   3’ UUGCACGGUCCAAAACGU 5’

query=JS16_t2282905, target=FGSG_12059, score=4, range=814-831, strand=1

target  5’ AACAUGCCAGGUUUGGCC 3’
           ::: :::::::::: ::
query   3’ UUGCACGGUCCAAAACGU 5’

query=JS16_t2282905, target=FGSG_13419, score=4, range=739-756, strand=-1

target  5’ GAUGUGCCAGCUUUUGUA 3’
           .:.::::::: :::::.:
query   3’ UUGCACGGUCCAAAACGU 5’

query=JS16_t2282905, target=FGSG_07937, score=4, range=2517-2534, strand=-1

target  5’ UGCUUGCCAGGUUUUGUG 3’
            .: ::::::::::::..
query   3’ UUGCACGGUCCAAAACGU 5’

query=JS16_t2282905, target=FGSG_00658, score=4, range=524-541, strand=-1

target  5’ GCUGUGUCAGGUUUUGCU 3’
           . .:::.::::::::::
query   3’ UUGCACGGUCCAAAACGU 5’

query=JS16_t2282905, target=FGSG_04284, score=4, range=139-156, strand=-1

target  5’ ACCGUGCCGGGUUUUGAA 3’
           : ::::::.::::::: :
query   3’ UUGCACGGUCCAAAACGU 5’

query=JS16_t2282905, target=FGSG_12743, score=4, range=243-259, strand=-1

target  5’ AGUGU-CCAGGUUUUGCC 3’
           :..:: :::::::::::
query   3’ UUGCACGGUCCAAAACGU 5’

query=JS16_t2282905, target=FGSG_07177, score=4, range=2062-2078, strand=-1

target  5’ GAC-UGCCAGGUUUCGCG 3’
           .:: :::::::::: ::.
query   3’ UUGCACGGUCCAAAACGU 5’

query=JS16_t2282905, target=FGSG_00361, score=4, range=1926-1943, strand=-1

target  5’ AUCUUGCCAGGUGUUGCA 3’
           : : :::::::: :::::
query   3’ UUGCACGGUCCAAAACGU 5’

query=JS16_t2282905, target=FGSG_04803, score=4, range=1412-1429, strand=-1

target  5’ ACCUUGCCAGCUUUUGCA 3’
```

```
target  5' GCGAGCAAUCAAGAUCAGC 3'
           ::: : :::::::::::.
query   3' CGC-CUUUAGUUCUAGUUU 5'

query=JS16_t4013978, target=FGSG_09838, score=4, range=632-648, strand=1

target  5' GUGG-AUUCAAGAUCAAG 3'
           :.:: : ::::::::::.
query   3' CGCCUUUAGUUCUAGUUU 5'

query=JS16_t4013978, target=FGSG_13801, score=4, range=1271-1287, strand=1

target  5' GC-GUGAUCAAGAUCAGA 3'
           :: : .::::::::::.:
query   3' CGCCUUUAGUUCUAGUUU 5'

query=JS16_t4013978, target=FGSG_04973, score=4, range=113-129, strand=1

target  5' UCGCAAA-CAAGAUCAAA 3'
            :: ::: ::::::::::
query   3' CGCCUUUAGUUCUAGUUU 5'

query=JS16_t4013978, target=FGSG_04041, score=4, range=295-312, strand=-1

target  5' ACGGAAGUCGAGAUCAGA 3'
            :::::.::.::::::.:
query   3' CGCCUUUAGUUCUAGUUU 5'

query=JS16_t4013978, target=FGSG_02080, score=4, range=1035-1052, strand=-1

target  5' GCGGAAAUAGAGAUCGAA 3'
           :::::::: .:::::.::
query   3' CGCCUUUAGUUCUAGUUU 5'

query=JS16_t4013978, target=FGSG_09704, score=4, range=2761-2778, strand=-1

target  5' GAGGAAAUUGAGAUCAGA 3'
           : ::::::..::::::.:
query   3' CGCCUUUAGUUCUAGUUU 5'

query=JS16_t4013978, target=FGSG_09115, score=4, range=610-627, strand=-1

target  5' UCGGAAAUCAAGAUUCAA 3'
            :::::::::::::. ::
query   3' CGCCUUUAGUUCUAGUUU 5'

query=JS16_t4013978, target=FGSG_01698, score=4, range=1120-1137, strand=-1

target  5' GCGGAAAUCAACAUCAGC 3'
           ::::::::::: ::::.
query   3' CGCCUUUAGUUCUAGUUU 5'

query=JS16_t4013978, target=FGSG_05703, score=4, range=345-362, strand=-1

target  5' CUGGGAAACAAGAUCAAA 3'
            .::.:: ::::::::::
query   3' CGCCUUUAGUUCUAGUUU 5'

query=JS16_t4013978, target=FGSG_06183, score=4, range=3148-3165, strand=-1

target  5' GGGGAAAACAAGAUCGAA 3'
           : ::::: :::::::.::
query   3' CGCCUUUAGUUCUAGUUU 5'

query=JS16_t4013978, target=FGSG_12620, score=4, range=100-117, strand=-1

target  5' GCAGAUAUCGAGAUCAAA 3'
           :: :: :::.::::::::
query   3' CGCCUUUAGUUCUAGUUU 5'

query=JS16_t4013978, target=FGSG_05691, score=4, range=3516-3533, strand=-1

target  5' UCGCAAGUCAAGAUCAAU 3'
            :: ::.::::::::::
query   3' CGCCUUUAGUUCUAGUUU 5'

query=JS16_t4013978, target=FGSG_02128, score=4, range=609-625, strand=-1

target  5' GCUGAAAU-AAGAUCAGA 3'
           :: ::::: :::::::.:
query   3' CGCCUUUAGUUCUAGUUU 5'

query=JS16_t4013978, target=FGSG_03164, score=4, range=244-260, strand=-1

target  5' GCGUAGAUCAAGA-CAAA 3'
           ::: :.::::::: ::::
query   3' CGCCUUUAGUUCUAGUUU 5'

query=JS16_t4013978, target=FGSG_10564, score=4, range=8122-8139, strand=-1

target  5' GGGAAAAUCCAGAUCAAA 3'
           : : :::::: :::::::
query   3' UUGCACGGUCCAAAACGU 5'

query=JS16_t2282905, target=FGSG_02636, score=4, range=998-1016, strand=-1

target  5' AACGUCGACAGGUUUUGCU 3'
           ::::: : ::::::::::
query   3' UUGCA-CGGUCCAAAACGU 5'

No results for JS16_t3179076
No results for JS16_t0255361
query=JS16_t2282987, target=FGSG_02659, score=3, range=1027-1044, strand=-1

target  5' UUCUCUGUCUUGA-ACACA 3'
           :::: :::::::: :::::
query   3' AAGACACAGAACUGUGUGU 5'

query=JS16_t2282987, target=FGSG_03814, score=3.5, range=1006-1024, strand=1

target  5' CUUUGUGUCUUGACAAACA 3'
            :.:::::::::::: :::
query   3' AAGACACAGAACUGUGUGU 5'

query=JS16_t2282987, target=FGSG_05794, score=4, range=7448-7465, strand=1

target  5' UUAUGUGUCUUGACG-ACA 3'
           :: :::::::::::. :::
query   3' AAGACACAGAACUGUGUGU 5'

query=JS16_t2282987, target=FGSG_01572, score=4, range=368-385, strand=1

target  5' AUCUGAGUCUUGACA-ACA 3'
            :::: ::::::::: :::
query   3' AAGACACAGAACUGUGUGU 5'

query=JS16_t2282987, target=FGSG_06193, score=4, range=1989-2006, strand=1

target  5' UUCUGU-UCUCGACACACA 3'
           :::::: ::: ::::::::
query   3' AAGACACAGAACUGUGUGU 5'

query=JS16_t2282987, target=FGSG_04551, score=4, range=820-837, strand=-1

target  5' UUCUG-GUCUUGAUAUGCA 3'
           ::::: :::::::.:..::
query   3' AAGACACAGAACUGUGUGU 5'

query=JS16_t2282987, target=FGSG_06191, score=4, range=1266-1283, strand=-1

target  5' UUCU-UGUCUUGACAAGCA 3'
           :::: :::::::::: .::
query   3' AAGACACAGAACUGUGUGU 5'

query=JS16_t2282987, target=FGSG_09417, score=4, range=1313-1332, strand=-1

target  5' AACAGUGGUCUUGACACACA 3'
             : :: :::::::::::::
query   3' AAGACA-CAGAACUGUGUGU 5'

No results for JS16_t3179114
No results for JS16_t4075732
No results for JS16_t2283031
No results for JS16_t4075750
No results for JS16_t1386969
query=JS16_t1386985, target=FGSG_00012, score=4, range=1369-1387, strand=1

target  5' AUUCCUUUCUCUGCGGGUG 3'
           :::::::::.::.: ::::
query   3' UAAGGAAAGGGAUGGCCAC 5'

query=JS16_t1386985, target=FGSG_02563, score=4, range=1120-1138, strand=1

target  5' GGUUCUUUCUCUGCCGGUG 3'
           .  :.:::::.::.::::::
query   3' UAAGGAAAGGGAUGGCCAC 5'

query=JS16_t1386985, target=FGSG_08390, score=4, range=679-697, strand=1

target  5' AUUCCCUUCCCAGCCGGUG 3'
           ::::: ::::: .::::::
query   3' UAAGGAAAGGGAUGGCCAC 5'

query=JS16_t1386985, target=FGSG_06041, score=4, range=2242-2260, strand=1

target  5' GUUGUGUUCCUUACCGGUG 3'
           .:: .  ::::.::::::::
query   3' UAAGGAAAGGGAUGGCCAC 5'

query=JS16_t1386985, target=FGSG_06605, score=4, range=352-370, strand=1

target  5' AGCGCUUUCCCUACUGGUG 3'
           :   ::::::::::.::::
```

```
           : : ::::: ::::::::
query   3’ CGCCUUUAGUUCUAGUUU 5’

query=JS16_t4013978, target=FGSG_11125, score=4, range=636-654, strand=-1

target  5’ GUGGAAAUCAGUGAUCAAG 3’
           :.:::::::. ::::::.
query   3’ CGCCUUUAGUU-CUAGUUU 5’

No results for JS16_t0268559
query=JS16_t1324768, target=FGSG_09740, score=3.5, range=99-119, strand=1

target  5’ GGCUGAAAAGUCAGCAAGGAU 3’
           ::::. ::::::: :::::::
query   3’ CCGAUCUUUCAGUAGUUCCUA 5’

query=JS16_t1324768, target=FGSG_08193, score=3.5, range=222-243, strand=1

target  5’ UGUUGAGAAAGUCAUCAAGGAC 3’
            :.: ::::::::::::::::
query   3’ CCGA-UCUUUCAGUAGUUCCUA 5’

query=JS16_t1324768, target=FGSG_03020, score=3.5, range=1684-1704, strand=-1

target  5’ CGCAGUAAAGUCAUCAAGGAU 3’
            :: . :::::::::::::::
query   3’ CCGAUCUUUCAGUAGUUCCUA 5’

query=JS16_t1324768, target=FGSG_06287, score=4, range=69-89, strand=1

target  5’ GGCCACAAGGUCAUCAAGGAG 3’
           ::: : ::.:::::::::::
query   3’ CCGAUCUUUCAGUAGUUCCUA 5’

query=JS16_t1324768, target=FGSG_10561, score=4, range=3460-3480, strand=1

target  5’ GGGUCGAAAAUCAUCAAGGAU 3’
           :: : :::: ::::::::::
query   3’ CCGAUCUUUCAGUAGUUCCUA 5’

query=JS16_t1324768, target=FGSG_07284, score=4, range=1356-1377, strand=-1

target  5’ GGCGAUGUAAGUCAUCAGGGAU 3’
           ::: : : :::::::::.::::
query   3’ CCGAU-CUUUCAGUAGUUCCUA 5’

No results for JS16_t4014034
query=JS16_t1324814, target=FGSG_07821, score=3.5, range=4473-4492, strand=-1

target  5’ CUUGUGGCACGGUACGUCGU 3’
           :.:::: :::::::::: ::
query   3’ GGACACGGUGCCAUGCA-CA 5’

query=JS16_t1324814, target=FGSG_08474, score=4, range=5881-5899, strand=-1

target  5’ CCUGUGCCAUGGCAUGUGU 3’
           :::::::::.:: :.::::
query   3’ GGACACGGUGCCAUGCACA 5’

query=JS16_t1324814, target=FGSG_00179, score=4, range=149-167, strand=-1

target  5’ CCGGUGCCGUGGUAUGUGU 3’
           :: :::::..::::.::::
query   3’ GGACACGGUGCCAUGCACA 5’

query=JS16_t1324814, target=FGSG_04651, score=4, range=1907-1924, strand=-1

target  5’ GCU-UGCCACGGUACGUCU 3’
            :: ::::::::::::: :
query   3’ GGACACGGUGCCAUGCACA 5’

No results for JS16_t0364612
No results for JS16_t3117245
No results for JS16_t2221218
No results for JS16_t0466638
query=JS16_t4014076, target=FGSG_02118, score=3, range=1120-1138, strand=-1

target  5’ CCAGACCAUCAACAUUU-UG 3’
           ::::: :::::::::: ::
query   3’ GGUCUAGUAGUUGUAAAUAC 5’

query=JS16_t4014076, target=FGSG_10471, score=3.5, range=775-793, strand=1

target  5’ GUCGA-CAUCAACAUUUAUG 3’
            . :: ::::::::::::::
query   3’ GGUCUAGUAGUUGUAAAUAC 5’

query=JS16_t4014076, target=FGSG_01040, score=3.5, range=258-276, strand=-1

target  5’ CCAG-UUAUCAACAUUUGUA 3’
           :::: :.:::::::::.:
query   3’ GGUCUAGUAGUUGUAAAUAC 5’

query   3’ UAAGGAAAGGGAUGGCCAC 5’

query=JS16_t1386985, target=FGSG_05614, score=4, range=172-190, strand=1

target  5’ UUCCCUUUCCCUACCGUUG 3’
            : ::::::::::::: ::
query   3’ UAAGGAAAGGGAUGGCCAC 5’

No results for JS16_t0307380
query=JS16_t1387005, target=FGSG_13471, score=3.5, range=494-515, strand=1

target  5’ CUGGUUC-GCCAAUCGAGUGGUC 3’
            :::::: .:::::::::::::
query   3’ CACCAAGAUGGUUAGCUCACCAA 5’

No results for JS16_t0474113
No results for JS16_t2283229
query=JS16_t2283275, target=FGSG_11392, score=3, range=894-912, strand=1

target  5’ UGGUAGGCGAAAGGGAGAG 3’
           ::::: : :::::::::::
query   3’ ACCAU-CACUUUCCCUCUC 5’

query=JS16_t2283275, target=FGSG_02054, score=3, range=659-677, strand=1

target  5’ UGGAGAGUGAAAGGGAGAA 3’
           :::  ::::::::::::
query   3’ ACC-AUCACUUUCCCUCUC 5’

query=JS16_t2283275, target=FGSG_08489, score=3, range=17-35, strand=-1

target  5’ UGGUAGAUGAAGGGGAGAG 3’
           :::::: ::::.:::::::
query   3’ ACCAUC-ACUUUCCCUCUC 5’

query=JS16_t2283275, target=FGSG_02286, score=3, range=311-328, strand=-1

target  5’ CGGAUGUGAAAGGGAGAG 3’
            ::  ::::::::::::
query   3’ ACCAUCACUUUCCCUCUC 5’

query=JS16_t2283275, target=FGSG_08390, score=3.5, range=2679-2696, strand=1

target  5’ GGAUGGUGAAAGGGAGAC 3’
            : :.:::::::::::
query   3’ ACCAUCACUUUCCCUCUC 5’

query=JS16_t2283275, target=FGSG_01199, score=3.5, range=275-292, strand=-1

target  5’ UGGUGGUGGAAGGGGGAC 3’
           ::::.:::.:::::.::
query   3’ ACCAUCACUUUCCCUCUC 5’

query=JS16_t2283275, target=FGSG_01902, score=3.5, range=13-30, strand=-1

target  5’ UGGGGGUGAAGGGGGGAG 3’
           ::: .:::::.:::.:::
query   3’ ACCAUCACUUUCCCUCUC 5’

query=JS16_t2283275, target=FGSG_02021, score=3.5, range=129-146, strand=-1

target  5’ AGGUGGUGAAAGGAAGAG 3’
            :::.::::::: ::::
query   3’ ACCAUCACUUUCCCUCUC 5’

query=JS16_t2283275, target=FGSG_03075, score=3.5, range=104-121, strand=-1

target  5’ UCUUGGUGAAAGGGAGAU 3’
           :  :.:::::::::::
query   3’ ACCAUCACUUUCCCUCUC 5’

query=JS16_t2283275, target=FGSG_07143, score=4, range=801-818, strand=1

target  5’ UGGUAGUGAGAGGGCGAA 3’
           :::::::::.:::: ::
query   3’ ACCAUCACUUUCCCUCUC 5’

query=JS16_t2283275, target=FGSG_11956, score=4, range=330-347, strand=1

target  5’ UGGAAGGGAGAGGGAGAG 3’
           ::: :: ::.::::::::
query   3’ ACCAUCACUUUCCCUCUC 5’

query=JS16_t2283275, target=FGSG_05518, score=4, range=1802-1820, strand=1

target  5’ UGGCUAGUGAAAGUGAGAC 3’
           ::: ::::::::: ::::
query   3’ ACC-AUCACUUUCCCUCUC 5’

query=JS16_t2283275, target=FGSG_04627, score=4, range=135-152, strand=1

target  5’ UGUUCGUGAGAGGGAGAC 3’
```

```
query=JS16_t4014076, target=FGSG_13355, score=3.5, range=1308-1326, strand=-1

target  5’ UCA-AUCAUCAUCAUUUAUG 3’
           .:: ::::::: ::::::::
query   3’ GGUCUAGUAGUUGUAAAUAC 5’

query=JS16_t4014076, target=FGSG_03437, score=4, range=225-243, strand=1

target  5’ GCACAUCAUCAA-AUUUAUG 3’
            :: :::::::: :::::::
query   3’ GGUCUAGUAGUUGUAAAUAC 5’

query=JS16_t4014076, target=FGSG_11974, score=4, range=1032-1050, strand=-1

target  5’ CUUGAUU-UCAACAUUUAUG 3’
           :.  :::. :::::::::::
query   3’ GGUCUAGUAGUUGUAAAUAC 5’

query=JS16_t4014076, target=FGSG_06832, score=4, range=861-879, strand=-1

target  5’ CCAGAU-AUCAACCUUUGUG 3’
           :::::: :::::: :::.::
query   3’ GGUCUAGUAGUUGUAAAUAC 5’

No results for JS16_t3117268
No results for JS16_t3117299
No results for JS16_t1324895
No results for JS16_t0466656
query=JS16_t4014234, target=FGSG_02138, score=4, range=613-635, strand=1

target  5’ UUUGAUGAACUUGAUGAGCGUGA 3’
           .: .:::  ::::::::::::::
query   3’ GACUUACAAGAACUACUCGCACU 5’

query=JS16_t4014234, target=FGSG_05748, score=4, range=1607-1629, strand=-1

target  5’ GUGAAUCUUGUUGAUGAGCGUGU 3’
            ::::: :: ::::::::::::
query   3’ GACUUACAAGAACUACUCGCACU 5’

No results for JS16_t2221437
query=JS16_t3117503, target=FGSG_08891, score=3.5, range=817-840, strand=1

target  5’ UCUCCAUGGAACAACUUCAUCAGA 3’
             :::::.: ::::::::::::::
query   3’ CUAGGUAUC-UGUUGAAGUAGUCU 5’

query=JS16_t3117503, target=FGSG_10948, score=3.5, range=619-641, strand=-1

target  5’ GGUCCAUGGACAACUUCACCAGG 3’
           :.:::::.:::::::::: :::.
query   3’ CUAGGUAUCUGUUGAAGUAGUCU 5’

query=JS16_t3117503, target=FGSG_03257, score=4, range=2464-2485, strand=-1

target  5’ GAUCCGUUGACAACUUC-UCAGG 3’
           :::::.: ::::::::: ::::.
query   3’ CUAGGUAUCUGUUGAAGUAGUCU 5’

No results for JS16_t0793652
No results for JS16_t4014351
No results for JS16_t3117574
query=JS16_t2221557, target=FGSG_00558, score=3, range=983-1001, strand=1

target  5’ GUG-AUGAGGUUGAGUUCAU 3’
           ::.  :: ::::::::::::.
query   3’ CAUGUAGUCCAACUCAAGUG 5’

query=JS16_t2221557, target=FGSG_01347, score=3.5, range=3220-3239, strand=1

target  5’ GUCCCUGAGGUUGAGUUCAU 3’
           :: : : ::::::::::::.
query   3’ CAUGUAGUCCAACUCAAGUG 5’

query=JS16_t2221557, target=FGSG_02721, score=3.5, range=314-332, strand=-1

target  5’ GU-UGUAAGGUUGAGUUCAU 3’
           :: ..: ::::::::::::.
query   3’ CAUGUAGUCCAACUCAAGUG 5’

query=JS16_t2221557, target=FGSG_07268, score=4, range=460-479, strand=1

target  5’ GUUAAGCAGGUUGAGUUCAA 3’
           ::  : :::::::::::::
query   3’ CAUGUAGUCCAACUCAAGUG 5’

query=JS16_t2221557, target=FGSG_07104, score=4, range=458-477, strand=-1

target  5’ GGACAAAAGGUUGAGUUCAA 3’
           : :::  ::::::::::::
query   3’ CAUGUAGUCCAACUCAAGUG 5’

           :: : ::::.:::::::
query   3’ ACCAUCACUUUCCCUCUC 5’

query=JS16_t2283275, target=FGSG_03769, score=4, range=1250-1266, strand=1

target  5’ UGG-AGUGGAAGGGACAG 3’
           ::: ::::.:::::: ::
query   3’ ACCAUCACUUUCCCUCUC 5’

query=JS16_t2283275, target=FGSG_11009, score=4, range=1601-1617, strand=1

target  5’ CGGAAG-GAAAGGGAGAG 3’
            :: :: ::::::::::
query   3’ ACCAUCACUUUCCCUCUC 5’

query=JS16_t2283275, target=FGSG_08948, score=4, range=364-381, strand=-1

target  5’ AUGUAGUGAAGGGGAGAU 3’
             ::::::::.::::::
query   3’ ACCAUCACUUUCCCUCUC 5’

query=JS16_t2283275, target=FGSG_09143, score=4, range=145-161, strand=-1

target  5’ UGG-AGUGGAGGGGGGAG 3’
           ::: ::::.:.:::.:::
query   3’ ACCAUCACUUUCCCUCUC 5’

query=JS16_t2283275, target=FGSG_07238, score=4, range=1845-1862, strand=-1

target  5’ UGGAAGUGAGAGUGAGAG 3’
           ::: :::::.:: :::::
query   3’ ACCAUCACUUUCCCUCUC 5’

query=JS16_t2283275, target=FGSG_05336, score=4, range=1598-1616, strand=-1

target  5’ UGGGAGUGCAAGGGGAGAG 3’
           ::: :::: ::.:::::::
query   3’ ACCAUCAC-UUUCCCUCUC 5’

query=JS16_t2283275, target=FGSG_02658, score=4, range=329-346, strand=-1

target  5’ CGGUCGUGAAAGUGAGAG 3’
            ::: ::::::: :::::
query   3’ ACCAUCACUUUCCCUCUC 5’

query=JS16_t2283275, target=FGSG_03454, score=4, range=467-484, strand=-1

target  5’ UGGCAUUGAAAGGGAGAA 3’
           ::: : ::::::::::
query   3’ ACCAUCACUUUCCCUCUC 5’

query=JS16_t2283275, target=FGSG_12242, score=4, range=1976-1992, strand=-1

target  5’ UGGU-GUGAAAGGGGCAG 3’
           :::: :::::::::. ::
query   3’ ACCAUCACUUUCCCUCUC 5’

query=JS16_t2283275, target=FGSG_11126, score=4, range=6-22, strand=-1

target  5’ GGGUAGU-AAAGGGAGAC 3’
            :::::: :::::::::
query   3’ ACCAUCACUUUCCCUCUC 5’

No results for JS16_t3179404
query=JS16_t2283337, target=FGSG_05628, score=2, range=634-652, strand=-1

target  5’ UCUUUG-GAUGAAACUGGUA 3’
           ::: :: :::::::::::::
query   3’ AGACACACUACUUUGACCAU 5’

query=JS16_t2283337, target=FGSG_05337, score=3, range=1669-1687, strand=-1

target  5’ UUUGUGUGAUG-AACUGGUG 3’
           :.::::::::: :::::::.
query   3’ AGACACACUACUUUGACCAU 5’

query=JS16_t2283337, target=FGSG_03340, score=3, range=3210-3228, strand=-1

target  5’ UCUGAGUGAUGAAA-UGGUA 3’
           :::: ::::::::: :::::
query   3’ AGACACACUACUUUGACCAU 5’

query=JS16_t2283337, target=FGSG_13273, score=3, range=777-795, strand=-1

target  5’ UAUG-GUGAUGAGACUGGUA 3’
           : :: :::::::.:::::::
query   3’ AGACACACUACUUUGACCAU 5’

query=JS16_t2283337, target=FGSG_07659, score=3.5, range=1120-1138, strand=1

target  5’ ACUG-AUGAUGAAACUGGUG 3’
            :::  :::::::::::::.
```

```
query=JS16_t2221567, target=FGSG_02469, score=3, range=394-411, strand=1

target  5' GC-UUUCCUCCGUGAGGUU 3'
           :: ::: ::::::::::::
query   3' CGCAAAAGAGGCACUCCAA 5'

query=JS16_t2221567, target=FGSG_10357, score=4, range=1139-1158, strand=1

target  5' UCGUUGUCAUCCGUGAGGUU 3'
            :::: :: :::::::::::
query   3' CGCAAAAG-AGGCACUCCAA 5'

query=JS16_t2221567, target=FGSG_12276, score=4, range=2033-2052, strand=-1

target  5' CCGUUUUCUUCCGUGGGGUU 3'
            ::::::: ::::::.::::
query   3' CGCAAAAG-AGGCACUCCAA 5'

query=JS16_t2221567, target=FGSG_11781, score=4, range=1639-1656, strand=-1

target  5' GCGUUUUC-CCGUGACGUU 3'
           :::::::: :::::: :::
query   3' CGCAAAAGAGGCACUCCAA 5'

query=JS16_t2221567, target=FGSG_02796, score=4, range=321-338, strand=-1

target  5' GC-UCUUCUCUGUGAGGUG 3'
           :: : :::::.:::::::
query   3' CGCAAAAGAGGCACUCCAA 5'

query=JS16_t0793661, target=FGSG_00270, score=3.5, range=665-685, strand=1

target  5' GCGCGGACCUAGGCGGCGGUG 3'
           ::: :.:::::::::.:.:::
query   3' CGCACUUGGAUCCGCUGUCAC 5'

No results for JS16_t0335129
No results for JS16_t3117627
No results for JS16_t4014420
query=JS16_t4014499, target=FGSG_09640, score=3.5, range=63-80, strand=1

target  5' GUCAAUGGGCCCGUUGUC 3'
           .::: :::.::::::::
query   3' UAGUCACCUGGGCAACAA 5'

query=JS16_t4014499, target=FGSG_01054, score=3.5, range=556-573, strand=1

target  5' CUGGGUGGGCCCGUUGUU 3'
            : .::::.:::::::::
query   3' UAGUCACCUGGGCAACAA 5'

query=JS16_t4014499, target=FGSG_03191, score=3.5, range=191-207, strand=1

target  5' AAUA-UGGACCUGUUGUU 3'
           : .: ::::::.::::::
query   3' UAGUCACCUGGGCAACAA 5'

query=JS16_t4014499, target=FGSG_07200, score=4, range=1003-1020, strand=1

target  5' CUCAGUGGAUGCGUUGUU 3'
            ::::::::. :::::::
query   3' UAGUCACCUGGGCAACAA 5'

query=JS16_t4014499, target=FGSG_13151, score=4, range=1963-1980, strand=1

target  5' CUCAGUGGACCUGUUGAU 3'
            ::::::::::.:::: :
query   3' UAGUCACCUGGGCAACAA 5'

query=JS16_t4014499, target=FGSG_05896, score=4, range=613-630, strand=1

target  5' AUCAUUGGACUCGUGGUU 3'
           :::: :::::.::: :::
query   3' UAGUCACCUGGGCAACAA 5'

query=JS16_t4014499, target=FGSG_13665, score=4, range=1508-1526, strand=1

target  5' AUCAGUUGGAUCCUUUGUU 3'
           ::::: ::::.:: :::::
query   3' UAGUC-ACCUGGGCAACAA 5'

query=JS16_t4014499, target=FGSG_08419, score=4, range=905-921, strand=1

target  5' AUCA-AGGACUCGUUGUU 3'
           ::::  ::::.:::::::
query   3' UAGUCACCUGGGCAACAA 5'

query=JS16_t4014499, target=FGSG_03083, score=4, range=1484-1502, strand=1

target  5' AGGAGAUGGACCUGUUGUU 3'
           :  :: ::::::.::::::
query   3' AGACACACUACUUUGACCAU 5'

query=JS16_t2283337, target=FGSG_07705, score=4, range=2101-2119, strand=1

target  5' UCU-UCUGAUGAAACUGGGA 3'
           ::: : :::::::::::: :
query   3' AGACACACUACUUUGACCAU 5'

No results for JS16_t2283344
No results for JS16_t4076107
query=JS16_t4076200, target=FGSG_02044, score=3, range=834-854, strand=-1

target  5' UUGUUUUGGAUGAAUGGUGUA 3'
           ::: ::::::::::.:::::
query   3' AAC-AAACCUACUUGCCACAA 5'

query=JS16_t4076200, target=FGSG_03144, score=4, range=1184-1203, strand=1

target  5' UUGUCUGGAUGCACGGUGUC 3'
           :::: :::::: :::::::
query   3' AACAAACCUACUUGCCACAA 5'

No results for JS16_t2283444
query=JS16_t0801332, target=FGSG_10502, score=3.5, range=3096-3115, strand=1

target  5' ACAUACGGCGACUGAGUCGG 3'
           :: .:::::.::::::::.:
query   3' UG-GUGCCGUUGACUCAGUC 5'

query=JS16_t0801332, target=FGSG_13182, score=3.5, range=552-570, strand=1

target  5' GCGACAGCAAUUGAGUCAG 3'
           .: :: ::::.::::::::
query   3' UGGUGCCGUUGACUCAGUC 5'

query=JS16_t0801332, target=FGSG_01076, score=3.5, range=449-468, strand=1

target  5' ACAGAGUGCAACUGAGUCAG 3'
           :: .  : ::::::::::::
query   3' UGGUGC-CGUUGACUCAGUC 5'

query=JS16_t0801332, target=FGSG_10515, score=3.5, range=44-62, strand=-1

target  5' GCUUUGGCAGCUGAGUCAG 3'
           .:. .::::.:::::::::
query   3' UGGUGCCGUUGACUCAGUC 5'

query=JS16_t0801332, target=FGSG_02686, score=3.5, range=704-723, strand=-1

target  5' ACCAAUGGCACCUGAGUCAG 3'
           ::: :.:::: :::::::::
query   3' UGG-UGCCGUUGACUCAGUC 5'

query=JS16_t0801332, target=FGSG_02327, score=4, range=577-595, strand=1

target  5' GACAUGGCAACUGAGUUGG 3'
           .  ::.:::::::::::..:
query   3' UGGUGCCGUUGACUCAGUC 5'

query=JS16_t0801332, target=FGSG_02400, score=4, range=3259-3276, strand=1

target  5' AUCAUGGCAGCUG-GUCAG 3'
           :.::.::::.::: :::::
query   3' UGGUGCCGUUGACUCAGUC 5'

query=JS16_t0801332, target=FGSG_02185, score=4, range=117-135, strand=1

target  5' ACAACCGCAACUGAGUUAU 3'
           :: :: ::::::::::.:
query   3' UGGUGCCGUUGACUCAGUC 5'

query=JS16_t0801332, target=FGSG_06556, score=4, range=56-74, strand=1

target  5' ACCAAAGCAACUGUGUCAG 3'
           ::::  ::::::: :::::
query   3' UGGUGCCGUUGACUCAGUC 5'

query=JS16_t0801332, target=FGSG_04559, score=4, range=409-426, strand=1

target  5' ACCA-GACAACUGAGUCAA 3'
           :::: : :::::::::::
query   3' UGGUGCCGUUGACUCAGUC 5'

query=JS16_t0801332, target=FGSG_09550, score=4, range=152-170, strand=-1

target  5' ACCACGGUAUCUGAGUCGG 3'
           :::::::.: :::::::.:
query   3' UGGUGCCGUUGACUCAGUC 5'

query=JS16_t0801332, target=FGSG_13475, score=4, range=67-85, strand=-1

target  5' GCCAUAGCAACUGGGUCAA 3'
```

```
query   3’ UAGUC-ACCUGGGCAACAA 5’

query=JS16_t4014499, target=FGSG_10028, score=4, range=41-57, strand=1

target  5’ AUC-GUGGUCCCGUUGUC 3’
           ::: :::: ::::::::
query   3’ UAGUCACCUGGGCAACAA 5’

query=JS16_t4014499, target=FGSG_09626, score=4, range=301-317, strand=1

target  5’ AUC-GUGGACCCUUUGUC 3’
           ::: :::::::: ::::
query   3’ UAGUCACCUGGGCAACAA 5’

query=JS16_t4014499, target=FGSG_00661, score=4, range=356-372, strand=1

target  5’ AUCAGUGGAACC-UUGUU 3’
           ::::::::: :: :::::
query   3’ UAGUCACCUGGGCAACAA 5’

query=JS16_t4014499, target=FGSG_13890, score=4, range=7-24, strand=-1

target  5’ AUUGAUGGGUCCGUUGUU 3’
           ::.. :::..::::::::
query   3’ UAGUCACCUGGGCAACAA 5’

query=JS16_t4014499, target=FGSG_06701, score=4, range=115-132, strand=-1

target  5’ GUGGGUGGAUCCGUUGUC 3’
           .: .:::::.:::::::
query   3’ UAGUCACCUGGGCAACAA 5’

query=JS16_t4014499, target=FGSG_03883, score=4, range=1003-1020, strand=-1

target  5’ AUCAAUGGUCUCGUUGUU 3’
           :::: ::: :.:::::::
query   3’ UAGUCACCUGGGCAACAA 5’

query=JS16_t4014499, target=FGSG_10005, score=4, range=958-975, strand=-1

target  5’ AGUGGUGGACCAGUUGUU 3’
           : ..::::::: ::::::
query   3’ UAGUCACCUGGGCAACAA 5’

query=JS16_t4014499, target=FGSG_02883, score=4, range=766-783, strand=-1

target  5’ ACCAAUGGACCUGUUGUC 3’
           : :: ::::::.:::::
query   3’ UAGUCACCUGGGCAACAA 5’

query=JS16_t4014499, target=FGSG_03561, score=4, range=751-767, strand=-1

target  5’ CUCAGUGGACCC-UUGUC 3’
            ::::::::::: ::::
query   3’ UAGUCACCUGGGCAACAA 5’

query=JS16_t4014499, target=FGSG_12140, score=4, range=614-631, strand=-1

target  5’ AGCAAUGGACCCGUGGUU 3’
           : :: ::::::::: :::
query   3’ UAGUCACCUGGGCAACAA 5’

query=JS16_t4014499, target=FGSG_06475, score=4, range=449-465, strand=-1

target  5’ CUCACUGG-CCCGUUGUU 3’
            ::: ::: :::::::::
query   3’ UAGUCACCUGGGCAACAA 5’

query=JS16_t4014499, target=FGSG_11126, score=4, range=176-192, strand=-1

target  5’ AUCACUGG-CUCGUUGUU 3’
           :::: ::: :.:::::::
query   3’ UAGUCACCUGGGCAACAA 5’

No results for JS16_t4014526
No results for JS16_t0466705
No results for JS16_t1325456
No results for JS16_t1325459
No results for JS16_t4014665
No results for JS16_t3117875
No results for JS16_t3117909
No results for JS16_t2221891
No results for JS16_t0575749
query=JS16_t3117949, target=FGSG_12575, score=3.5, range=41-60, strand=1

target  5’ UGUUUGUUGGUUGGUUAGUU 3’
           .:::.:.:::: ::::::::
query   3’ GCAAGCGACCAUCCAAUCAA 5’

query=JS16_t3117949, target=FGSG_08835, score=3.5, range=235-254, strand=-1

target  5’ UGUUCAAUGGUAGGUUGGUU 3’
           .:::. :::::::.::::
query   3’ UGGUGCCGUUGACUCAGUC 5’

query=JS16_t0801332, target=FGSG_02432, score=4, range=629-647, strand=-1

target  5’ AAGACGGCGACUGAGUCAU 3’
           :  :::::.:::::::::
query   3’ UGGUGCCGUUGACUCAGUC 5’

query=JS16_t0801332, target=FGSG_14008, score=4, range=98-116, strand=-1

target  5’ ACCAGGGCAACUGAGACAA 3’
           :::: :::::::::: ::
query   3’ UGGUGCCGUUGACUCAGUC 5’

query=JS16_t0801332, target=FGSG_07725, score=4, range=275-292, strand=-1

target  5’ AUC-UGGUAACUGAGUCAU 3’
           :.: .::.::::::::::
query   3’ UGGUGCCGUUGACUCAGUC 5’

query=JS16_t0801332, target=FGSG_12579, score=4, range=130-147, strand=-1

target  5’ ACUG-AGCAACUGAGUUAG 3’
           ::..  ::::::::::.::
query   3’ UGGUGCCGUUGACUCAGUC 5’

query=JS16_t0801332, target=FGSG_11512, score=4, range=374-391, strand=-1

target  5’ AUGAUGG-AACUGAGUCAG 3’
           :.  :.:: :::::::::::
query   3’ UGGUGCCGUUGACUCAGUC 5’

No results for JS16_t4076251
query=JS16_t3179620, target=FGSG_05547, score=4, range=2804-2824, strand=-1

target  5’ CAUGCGAGGGAACGCUUCGUG 3’
            :..:::::::::::: ::::
query   3’ UUGUGCUCCCUUGCGACGCAC 5’

query=JS16_t1387394, target=FGSG_01698, score=4, range=818-837, strand=-1

target  5’ CUCAUCC-GUGAGACACGAGC 3’
            :::::: ::::.:::::::
query   3’ UAGUAGGACACUUUGUGCUCC 5’

No results for JS16_t3179698
No results for JS16_t0269575
No results for JS16_t0395989
query=JS16_t3179816, target=FGSG_01364, score=2.5, range=6114-6131, strand=1

target  5’ AGCUUUCAGUUUCGAGAU 3’
           :::::.:::::::::.:.
query   3’ UCGAAGGUCAAAGCUUUG 5’

query=JS16_t3179816, target=FGSG_04278, score=3, range=1032-1049, strand=1

target  5’ AGCUUUCAGUUUCGAGAA 3’
           :::::.:::::::::.:
query   3’ UCGAAGGUCAAAGCUUUG 5’

query=JS16_t3179816, target=FGSG_08135, score=3, range=1146-1163, strand=1

target  5’ ACCUUUUAGUUUCGAAAC 3’
           : :::..:::::::::::
query   3’ UCGAAGGUCAAAGCUUUG 5’

query=JS16_t3179816, target=FGSG_06133, score=3, range=1468-1485, strand=1

target  5’ ACCUUCCAGAUUCGAAAC 3’
           : ::::::: ::::::::
query   3’ UCGAAGGUCAAAGCUUUG 5’

query=JS16_t3179816, target=FGSG_03455, score=3, range=791-808, strand=1

target  5’ AAUUGCCAGUUUCGAAAU 3’
           : .: ::::::::::::.
query   3’ UCGAAGGUCAAAGCUUUG 5’

query=JS16_t3179816, target=FGSG_11268, score=3, range=1731-1748, strand=-1

target  5’ CGCUUUCGGUUUCGAAAC 3’
            ::::.:.::::::::::
query   3’ UCGAAGGUCAAAGCUUUG 5’

query=JS16_t3179816, target=FGSG_11668, score=3, range=291-309, strand=-1

target  5’ AGCUUCACAGUUUCGGAAC 3’
           :::::: ::::::::.:::
query   3’ UCGAAG-GUCAAAGCUUUG 5’

query=JS16_t3179816, target=FGSG_11671, score=3, range=1574-1592, strand=-1
```

```
           .::::  :::::::::.:::
query   3' GCAAGCGACCAUCCAAUCAA 5'

No results for JS16_t4014777
query=JS16_t1325596, target=FGSG_02138, score=2.5, range=640-656, strand=1

target  5' GCC-UGGAACGUGUACCC 3'
           .:: :::::::::::::
query   3' UGGUACCUUGCACAUGGC 5'

query=JS16_t1325596, target=FGSG_03691, score=3, range=2059-2076, strand=-1

target  5' GCCGAGGAACGUGUAUCG 3'
           .::. ::::::::::.::
query   3' UGGUACCUUGCACAUGGC 5'

query=JS16_t1325596, target=FGSG_00108, score=3.5, range=620-637, strand=1

target  5' UCUAUGGAGCGUGUACUG 3'
            :.:::::.:::::::.:
query   3' UGGUACCUUGCACAUGGC 5'

query=JS16_t1325596, target=FGSG_08513, score=3.5, range=808-824, strand=1

target  5' ACCGUGGAACGUG-ACCA 3'
           :::.::::::::: :::
query   3' UGGUACCUUGCACAUGGC 5'

query=JS16_t1325596, target=FGSG_12346, score=3.5, range=823-839, strand=1

target  5' AUC-UGGAACGUGUACUA 3'
           :.: ::::::::::::.
query   3' UGGUACCUUGCACAUGGC 5'

query=JS16_t1325596, target=FGSG_01677, score=3.5, range=780-797, strand=-1

target  5' GUCGUGGAAUGUGUACCA 3'
           ..:.:::::.:::::::
query   3' UGGUACCUUGCACAUGGC 5'

query=JS16_t1325596, target=FGSG_07554, score=4, range=153-169, strand=1

target  5' GUCAUGGAACGU-UGCCG 3'
           ..:::::::::: :.:::
query   3' UGGUACCUUGCACAUGGC 5'

query=JS16_t1325596, target=FGSG_05218, score=4, range=780-797, strand=1

target  5' ACCAUGGCAGGUGUACCG 3'
           ::::::: : ::::::::
query   3' UGGUACCUUGCACAUGGC 5'

query=JS16_t1325596, target=FGSG_05951, score=4, range=1335-1353, strand=1

target  5' ACCAUUGGGAGGUGUACCG 3'
           :::: :::.: ::::::::
query   3' UGGU-ACCUUGCACAUGGC 5'

query=JS16_t1325596, target=FGSG_11167, score=4, range=318-334, strand=1

target  5' ACCA-GGGACGUGUACUC 3'
           :::: ::.::::::::.
query   3' UGGUACCUUGCACAUGGC 5'

query=JS16_t1325596, target=FGSG_10982, score=4, range=1738-1754, strand=-1

target  5' ACCAUGGAACG-GUACUU 3'
           ::::::::::: ::::.
query   3' UGGUACCUUGCACAUGGC 5'

query=JS16_t1325596, target=FGSG_02337, score=4, range=174-191, strand=-1

target  5' CCCUUGGAAAGUGUACCG 3'
            :: ::::: ::::::::
query   3' UGGUACCUUGCACAUGGC 5'

query=JS16_t1325596, target=FGSG_03722, score=4, range=251-268, strand=-1

target  5' GCAGCGGAACGUGUACCA 3'
           .: .  ::::::::::::
query   3' UGGUACCUUGCACAUGGC 5'

query=JS16_t1325596, target=FGSG_12474, score=4, range=2-18, strand=-1

target  5' ACAAUGGAACGUG-ACCA 3'
           :: :::::::::: :::
query   3' UGGUACCUUGCACAUGGC 5'

query=JS16_t1325596, target=FGSG_03592, score=4, range=146-162, strand=-1

target  5' ACCAUGGAAC-UGUGCCA 3'
           :::::::::: :::.::

target  5' AGCUUGCUAGUUUUGAAAC 3'
           ::::: :.:::::.:::::
query   3' UCGAA-GGUCAAAGCUUUG 5'

query=JS16_t3179816, target=FGSG_03407, score=3, range=2540-2557, strand=-1

target  5' UGCUGCCAGUUUCGAAAG 3'
             ::: ::::::::::::
query   3' UCGAAGGUCAAAGCUUUG 5'

query=JS16_t3179816, target=FGSG_01008, score=3, range=20-36, strand=-1

target  5' AGC-UCCAGUUUCGGAAA 3'
           ::: ::::::::::.::
query   3' UCGAAGGUCAAAGCUUUG 5'

query=JS16_t3179816, target=FGSG_08209, score=3.5, range=1095-1112, strand=1

target  5' GGCUUCCACUUUCGGAAC 3'
           .::::::: :::::.:::
query   3' UCGAAGGUCAAAGCUUUG 5'

query=JS16_t3179816, target=FGSG_12072, score=3.5, range=265-281, strand=-1

target  5' GGUUUCCA-UUUCGAAAU 3'
           .:.::::: ::::::::.
query   3' UCGAAGGUCAAAGCUUUG 5'

query=JS16_t3179816, target=FGSG_02198, score=3.5, range=683-700, strand=-1

target  5' AGCAUCCAAUUUCGAAAU 3'
           ::: :::: ::::::::.
query   3' UCGAAGGUCAAAGCUUUG 5'

query=JS16_t3179816, target=FGSG_08831, score=3.5, range=521-538, strand=-1

target  5' GUCGUCCAGUUUCGAAGC 3'
           .  : ::::::::::::.:
query   3' UCGAAGGUCAAAGCUUUG 5'

query=JS16_t3179816, target=FGSG_02198, score=3.5, range=506-523, strand=-1

target  5' CGUCUCCAGUUUUGAAAC 3'
             :.  ::::::::.:::::
query   3' UCGAAGGUCAAAGCUUUG 5'

query=JS16_t3179816, target=FGSG_04413, score=4, range=1004-1022, strand=1

target  5' AGCGUGCUGGUUUCGAAAC 3'
           ::: : :..::::::::::
query   3' UCG-AAGGUCAAAGCUUUG 5'

query=JS16_t3179816, target=FGSG_13008, score=4, range=1001-1018, strand=1

target  5' AUCUUCCAGUGUCGAAAA 3'
           : :::::::: ::::::
query   3' UCGAAGGUCAAAGCUUUG 5'

query=JS16_t3179816, target=FGSG_05352, score=4, range=87-104, strand=1

target  5' AGCUCCCAGAUUCGAAAG 3'
           :::: :::: :::::::
query   3' UCGAAGGUCAAAGCUUUG 5'

query=JS16_t3179816, target=FGSG_04901, score=4, range=140-158, strand=1

target  5' AGAUUCCAGUUUCUGAAAG 3'
           :: :::::::::: ::::
query   3' UCGAAGGUCAAAG-CUUUG 5'

query=JS16_t3179816, target=FGSG_08651, score=4, range=172-188, strand=1

target  5' AGCUCCCAGUUUC-AAGC 3'
           :::: :::::::: ::.:
query   3' UCGAAGGUCAAAGCUUUG 5'

query=JS16_t3179816, target=FGSG_08379, score=4, range=139-155, strand=1

target  5' AG-UUCCUGUUUCGAGAC 3'
           :: :::: :::::::.::
query   3' UCGAAGGUCAAAGCUUUG 5'

query=JS16_t3179816, target=FGSG_04769, score=4, range=133-149, strand=1

target  5' UACUUCC-GUUUCGAAAC 3'
               ::::: :::::::::
query   3' UCGAAGGUCAAAGCUUUG 5'

query=JS16_t3179816, target=FGSG_10736, score=4, range=201-218, strand=1

target  5' AGAUCCCAGUUUCGACAC 3'
```

```
query   3' UGGUACCUUGCACAUGGC 5'

No results for JS16_t4014802
No results for JS16_t2222005
query=JS16_t0684377, target=FGSG_04916, score=3.5, range=1566-1589, strand=1

target  5' AGAUGAAC-CAUCCACCGCCAAGAG 3'
           :.:::::: :::: :::::::::::
query   3' UUUACUUGCGUAGUUGGCGGUUCUC 5'

No results for JS16_t3118014
query=JS16_t0393969, target=FGSG_05221, score=4, range=428-450, strand=1

target  5' AGGAGGCUAAGCGACUUCUUGGU 3'
           ::: :: :  ::::::::::::
query   3' UCCACCAAGGCGCUGAAGAACCA 5'

query=JS16_t1325640, target=FGSG_01138, score=3.5, range=107-127, strand=1

target  5' AAUUCAA-GGACUGUCUCAAGG 3'
             .:::: ::::::::::::::
query   3' GCGAGUUACCUGACAGAGUUCC 5'

query=JS16_t1325640, target=FGSG_05586, score=3.5, range=2598-2618, strand=1

target  5' CGUUCAAAGGACUG-CUCAAGG 3'
           ::.:::: :::::: :::::::
query   3' GCGAGUUACCUGACAGAGUUCC 5'

No results for JS16_t1325661
query=JS16_t0194384, target=FGSG_02982, score=4, range=1207-1226, strand=1

target  5' CUUCCUA-AUGGCGACUUUCU 3'
           ::::: : : :::::::::::
query   3' GAAGGUUGUCCCGCUGAAAGA 5'

query=JS16_t0194384, target=FGSG_10227, score=4, range=918-937, strand=-1

target  5' CUUCCAACAGGGC-AUUUUCC 3'
           ::::::::::::: :.::::
query   3' GAAGGUUGUCCCGCUGAAAGA 5'

No results for JS16_t3118082
query=JS16_t0228342, target=FGSG_04421, score=4, range=640-657, strand=1

target  5' GA-CUUUUCUGUUCUAAUG 3'
           :: ::::.::::: :::::
query   3' CUCGAAAGGACAAAAUUAC 5'

query=JS16_t0335149, target=FGSG_09706, score=1, range=129-146, strand=1

target  5' AUCAACCGCCAAGGGAAA 3'
           :::::::::::::.::::
query   3' UAGUUGGCGGUUCUCUUU 5'

query=JS16_t0335149, target=FGSG_06573, score=2, range=302-318, strand=1

target  5' GUC-ACCGCCAAGAGAAG 3'
           .:: :::::::::::::.
query   3' UAGUUGGCGGUUCUCUUU 5'

query=JS16_t0335149, target=FGSG_07351, score=2, range=251-268, strand=-1

target  5' GUCGACCGCCAAGAGGAA 3'
           .::.::::::::::.::
query   3' UAGUUGGCGGUUCUCUUU 5'

query=JS16_t0335149, target=FGSG_01144, score=2, range=2906-2922, strand=-1

target  5' GUU-ACCGCCAAGAGAAA 3'
           .:.  ::::::::::::::
query   3' UAGUUGGCGGUUCUCUUU 5'

query=JS16_t0335149, target=FGSG_05171, score=3, range=64-81, strand=1

target  5' AUCAACCGUCAGGAGAAU 3'
           ::::::::.::.:::::
query   3' UAGUUGGCGGUUCUCUUU 5'

query=JS16_t0335149, target=FGSG_08461, score=3, range=512-529, strand=1

target  5' ACCAACCGCCCAGAGAAA 3'
           : :::::::: :::::::
query   3' UAGUUGGCGGUUCUCUUU 5'

query=JS16_t0335149, target=FGSG_02815, score=3, range=715-732, strand=1

target  5' AUUCUCCGCCAAGAGAAG 3'
           ::.   ::::::::::::.
query   3' UAGUUGGCGGUUCUCUUU 5'

query=JS16_t0335149, target=FGSG_03987, score=3, range=121-137, strand=1

           :: : :::::::::: ::
query   3' UCGAAGGUCAAAGCUUUG 5'

query=JS16_t3179816, target=FGSG_01473, score=4, range=22-39, strand=1

target  5' AGAAGUCAGUUUCGAAAC 3'
           ::   .::::::::::::
query   3' UCGAAGGUCAAAGCUUUG 5'

query=JS16_t3179816, target=FGSG_13277, score=4, range=77-95, strand=1

target  5' AGAUUACAGAUUUCGAAAC 3'
           :: :: ::: :::::::::
query   3' UCGAAGGUC-AAAGCUUUG 5'

query=JS16_t3179816, target=FGSG_10651, score=4, range=732-748, strand=1

target  5' AUCUCCCAG-UUCGAAAC 3'
           : :: :::: ::::::::
query   3' UCGAAGGUCAAAGCUUUG 5'

query=JS16_t3179816, target=FGSG_00960, score=4, range=1489-1506, strand=-1

target  5' UUAUUCCAGUUUCGAAGC 3'
              :::::::::::::.:
query   3' UCGAAGGUCAAAGCUUUG 5'

query=JS16_t3179816, target=FGSG_03556, score=4, range=830-846, strand=-1

target  5' AG-UUUCAGUCUCGAAAC 3'
           :: ::.:::: :::::::
query   3' UCGAAGGUCAAAGCUUUG 5'

query=JS16_t3179816, target=FGSG_06116, score=4, range=790-806, strand=-1

target  5' UUCUUCCAGUUU-GAAAC 3'
             :::::::::: :::::
query   3' UCGAAGGUCAAAGCUUUG 5'

query=JS16_t3179816, target=FGSG_03455, score=4, range=799-815, strand=-1

target  5' AGCGGCCA-UUUCGAAAC 3'
           :::  ::: :::::::::
query   3' UCGAAGGUCAAAGCUUUG 5'

No results for JS16_t0337175
No results for JS16_t4076454
No results for JS16_t4076472
No results for JS16_t4076476
No results for JS16_t2283714
query=JS16_t0691754, target=FGSG_05205, score=4, range=557-580, strand=1

target  5' ACGACC-CAAUUGAGGAUGAUCACG 3'
           :::::: :  :::::::.:::::::
query   3' UGCUGGUGGCAACUCCUGCUAGUGC 5'

No results for JS16_t1387609
No results for JS16_t3179870
query=JS16_t1387649, target=FGSG_00379, score=2, range=399-416, strand=-1

target  5' ACUCUUUUGAUAGCAUCA 3'
           ::  ::::::::::::::
query   3' UGCCAAAACUAUCGUAGU 5'

query=JS16_t1387649, target=FGSG_00633, score=3, range=182-199, strand=1

target  5' ACCGAUUUGAUAGCAUCC 3'
           :: : ::::::::::::
query   3' UGCCAAAACUAUCGUAGU 5'

query=JS16_t1387649, target=FGSG_13177, score=3, range=271-287, strand=1

target  5' ACUGUUUUGA-AGCAUCA 3'
           :: ::::::: :::::::
query   3' UGCCAAAACUAUCGUAGU 5'

query=JS16_t1387649, target=FGSG_02899, score=3, range=372-389, strand=-1

target  5' GUGGUUGUGAUAGCAUCA 3'
           ..:::: :::::::::::
query   3' UGCCAAAACUAUCGUAGU 5'

query=JS16_t1387649, target=FGSG_06220, score=3, range=594-611, strand=-1

target  5' GCGGUCUUGAUAGCAUCG 3'
           .:::: :::::::::::.
query   3' UGCCAAAACUAUCGUAGU 5'

query=JS16_t1387649, target=FGSG_07348, score=3, range=495-512, strand=-1

target  5' AUUGUUUUGAUAGUAUCG 3'
           :. ::::::::::.:::.
```

```
target  5’ GUC-GCCGCCAAGAGAAU 3’
           .:: .:::::::::::::
query   3’ UAGUUGGCGGUUCUCUUU 5’

query=JS16_t0335149, target=FGSG_01690, score=3, range=1118-1134, strand=-1

target  5’ AUU-GCCGCCAAGAGAAU 3’
           ::. .:::::::::::::
query   3’ UAGUUGGCGGUUCUCUUU 5’

query=JS16_t0335149, target=FGSG_01488, score=3.5, range=132-149, strand=1

target  5’ CACAGCUGCCAAGAGAAA 3’
             ::.:.::::::::::::
query   3’ UAGUUGGCGGUUCUCUUU 5’

query=JS16_t0335149, target=FGSG_07008, score=3.5, range=33-50, strand=1

target  5’ UUCGCCCGCCAAGAGGAA 3’
            ::. ::::::::::.::
query   3’ UAGUUGGCGGUUCUCUUU 5’

query=JS16_t0335149, target=FGSG_04530, score=3.5, range=2008-2025, strand=-1

target  5’ UUCAGUCGCCAAGAGAAC 3’
            :::..:::::::::::
query   3’ UAGUUGGCGGUUCUCUUU 5’

query=JS16_t0335149, target=FGSG_04890, score=3.5, range=908-924, strand=-1

target  5’ GUU-GCCGCCAAGAGAGA 3’
           .:. .:::::::::::.:
query   3’ UAGUUGGCGGUUCUCUUU 5’

query=JS16_t0335149, target=FGSG_07934, score=3.5, range=428-445, strand=-1

target  5’ AGCUGCCGCCAGGAGAAA 3’
           : : .::::::.::::::
query   3’ UAGUUGGCGGUUCUCUUU 5’

query=JS16_t0335149, target=FGSG_05658, score=3.5, range=545-563, strand=-1

target  5’ AACAUGCCGCCAAGAGAAC 3’
           : :: .:::::::::::::
query   3’ UAGU-UGGCGGUUCUCUUU 5’

query=JS16_t0335149, target=FGSG_02895, score=4, range=176-193, strand=1

target  5’ AUCAACCGCCUAGGGAGA 3’
           :::::::::: ::.::.:
query   3’ UAGUUGGCGGUUCUCUUU 5’

query=JS16_t0335149, target=FGSG_05855, score=4, range=1530-1547, strand=1

target  5’ UCCAACUGCCGAGAGAAA 3’
             ::::.:::.:::::::
query   3’ UAGUUGGCGGUUCUCUUU 5’

query=JS16_t0335149, target=FGSG_06900, score=4, range=1238-1256, strand=1

target  5’ GUGCAGCCGUCAAGAGGAA 3’
           .: ::.:::.::::::.::
query   3’ UA-GUUGGCGGUUCUCUUU 5’

query=JS16_t0335149, target=FGSG_10033, score=4, range=2029-2045, strand=1

target  5’ AUCAACCG-CAAGGGAGA 3’
           :::::::: ::::.::.:
query   3’ UAGUUGGCGGUUCUCUUU 5’

query=JS16_t0335149, target=FGSG_09585, score=4, range=1742-1759, strand=1

target  5’ AACAACCGCCAAGAAAGA 3’
           : :::::::::::: :.:
query   3’ UAGUUGGCGGUUCUCUUU 5’

query=JS16_t0335149, target=FGSG_13630, score=4, range=826-844, strand=1

target  5’ ACCAACCGCCAGGCAGAAA 3’
           : :::::::::.: :::::
query   3’ UAGUUGGCGGUUC-UCUUU 5’

query=JS16_t0335149, target=FGSG_08400, score=4, range=1359-1376, strand=1

target  5’ GCCAGCAGCCAAGAGAAA 3’
           .  ::.: ::::::::::
query   3’ UAGUUGGCGGUUCUCUUU 5’

query=JS16_t0335149, target=FGSG_02595, score=4, range=60-78, strand=1

target  5’ GUCGUCCGCCAAGAUGAAA 3’
query   3’ UGCCAAAACUAUCGUAGU 5’

query=JS16_t1387649, target=FGSG_12938, score=3, range=249-266, strand=-1

target  5’ AUGGAUUUGAUAGCAUUG 3’
           :.:: :::::::::::..
query   3’ UGCCAAAACUAUCGUAGU 5’

query=JS16_t1387649, target=FGSG_08981, score=3, range=1026-1043, strand=-1

target  5’ ACAGUGUUGAUAGCAUCA 3’
           :: :: ::::::::::::
query   3’ UGCCAAAACUAUCGUAGU 5’

query=JS16_t1387649, target=FGSG_01352, score=3, range=279-296, strand=-1

target  5’ ACGGGUUUGAUAGCAGCA 3’
           :::: :::::::::: ::
query   3’ UGCCAAAACUAUCGUAGU 5’

query=JS16_t1387649, target=FGSG_00596, score=3, range=1314-1331, strand=-1

target  5’ AGGGCUUUGAUGGCAUCA 3’
           : :: ::::::.::::::
query   3’ UGCCAAAACUAUCGUAGU 5’

query=JS16_t1387649, target=FGSG_08555, score=3.5, range=970-987, strand=1

target  5’ GCCGCUUUGAUGGCAUCA 3’
           .: : ::::::.::::::
query   3’ UGCCAAAACUAUCGUAGU 5’

query=JS16_t1387649, target=FGSG_03208, score=3.5, range=1202-1219, strand=1

target  5’ CUAGUUUUGAUAGCAUCC 3’
            . ::::::::::::::
query   3’ UGCCAAAACUAUCGUAGU 5’

query=JS16_t1387649, target=FGSG_12561, score=3.5, range=1406-1423, strand=1

target  5’ AUAUCUUUGAUAGCAUCA 3’
           :.   :::::::::::::
query   3’ UGCCAAAACUAUCGUAGU 5’

query=JS16_t1387649, target=FGSG_11596, score=3.5, range=537-554, strand=-1

target  5’ GCGUUCUUGAUAGCAUCA 3’
           .:: : ::::::::::::
query   3’ UGCCAAAACUAUCGUAGU 5’

query=JS16_t1387649, target=FGSG_11822, score=3.5, range=180-196, strand=-1

target  5’ AU-GUUUGGAUAGCAUCA 3’
           :. :::: ::::::::::
query   3’ UGCCAAAACUAUCGUAGU 5’

query=JS16_t1387649, target=FGSG_09910, score=3.5, range=396-412, strand=-1

target  5’ GCCG-UUUGAUAGCGUCA 3’
           .: : :::::::::.:::
query   3’ UGCCAAAACUAUCGUAGU 5’

query=JS16_t1387649, target=FGSG_01645, score=4, range=1021-1039, strand=1

target  5’ ACGGGUUUUGAUGGCGUUA 3’
           :: :::::::::.::.:.:
query   3’ UG-CCAAAACUAUCGUAGU 5’

query=JS16_t1387649, target=FGSG_11181, score=4, range=1745-1762, strand=1

target  5’ AGGGUUUUGAUCGCGUCA 3’
           : ::::::::: ::.:::
query   3’ UGCCAAAACUAUCGUAGU 5’

query=JS16_t1387649, target=FGSG_03910, score=4, range=33-49, strand=1

target  5’ ACAG-UUUGGUAGCGUCA 3’
           :: : ::::.::::.:::
query   3’ UGCCAAAACUAUCGUAGU 5’

query=JS16_t1387649, target=FGSG_12583, score=4, range=83-100, strand=1

target  5’ ACCGCUUCGAUAGCAUCA 3’
           :: : :: ::::::::::
query   3’ UGCCAAAACUAUCGUAGU 5’

query=JS16_t1387649, target=FGSG_01286, score=4, range=324-340, strand=1

target  5’ ACCG-UCUGAUAGCAUCA 3’
           :: : : :::::::::::
query   3’ UGCCAAAACUAUCGUAGU 5’
```

```
           .::. ::::::::: ::::
query   3’ UAGUUGGCGGUUCU-CUUU 5’

query=JS16_t0335149, target=FGSG_01608, score=4, range=681-699, strand=1

target  5’ AUCACGCCGCCACGAGAAG 3’
           :::: .:::::: :::::.
query   3’ UAGU-UGGCGGUUCUCUUU 5’

query=JS16_t0335149, target=FGSG_11707, score=4, range=727-744, strand=1

target  5’ CUAAACCGCGAAGAGAAA 3’
            : :::::: ::::::::
query   3’ UAGUUGGCGGUUCUCUUU 5’

query=JS16_t0335149, target=FGSG_00737, score=4, range=2361-2377, strand=1

target  5’ AGCAGCCGCCAAG-GAAG 3’
           : ::.:::::::: :::.
query   3’ UAGUUGGCGGUUCUCUUU 5’

query=JS16_t0335149, target=FGSG_08579, score=4, range=1813-1829, strand=1

target  5’ AUU-GCCUCCAAGAGAAA 3’
           ::. .:: ::::::::::
query   3’ UAGUUGGCGGUUCUCUUU 5’

query=JS16_t0335149, target=FGSG_10362, score=4, range=966-983, strand=1

target  5’ CCCUCCCGCCAAGAGAAA 3’
             :  :::::::::::::
query   3’ UAGUUGGCGGUUCUCUUU 5’

query=JS16_t0335149, target=FGSG_02276, score=4, range=99-115, strand=1

target  5’ AGCAACUG-CAAGAGAAA 3’
           : ::::.: :::::::::
query   3’ UAGUUGGCGGUUCUCUUU 5’

query=JS16_t0335149, target=FGSG_05957, score=4, range=325-341, strand=1

target  5’ GUCCGCCG-CAAGAGAAA 3’
           .:: .::: :::::::::
query   3’ UAGUUGGCGGUUCUCUUU 5’

query=JS16_t0335149, target=FGSG_12110, score=4, range=46-63, strand=-1

target  5’ GCCAACUGUCAAGAGAAG 3’
           .  ::::.:.::::::::.
query   3’ UAGUUGGCGGUUCUCUUU 5’

query=JS16_t0335149, target=FGSG_06898, score=4, range=368-385, strand=-1

target  5’ ACGAACUGCCAGGAGAAA 3’
           :  :::.::::.::::::
query   3’ UAGUUGGCGGUUCUCUUU 5’

query=JS16_t0335149, target=FGSG_03606, score=4, range=83-100, strand=-1

target  5’ AUCCACCGCUAAGAGACA 3’
           ::: :::::.:::::: :
query   3’ UAGUUGGCGGUUCUCUUU 5’

query=JS16_t0335149, target=FGSG_02528, score=4, range=1264-1281, strand=-1

target  5’ ACCAAACGCCAAGAGGAA 3’
           : ::: :::::::::.::
query   3’ UAGUUGGCGGUUCUCUUU 5’

query=JS16_t0335149, target=FGSG_12941, score=4, range=132-149, strand=-1

target  5’ AGCAACCGACAGGAGAAA 3’
           : :::::: ::.::::::
query   3’ UAGUUGGCGGUUCUCUUU 5’

query=JS16_t0335149, target=FGSG_02020, score=4, range=1715-1732, strand=-1

target  5’ GACGACCGCCAAGAGCAA 3’
           .  :.:::::::::: ::
query   3’ UAGUUGGCGGUUCUCUUU 5’

query=JS16_t0335149, target=FGSG_10096, score=4, range=855-872, strand=-1

target  5’ AUGAUCCGCCAAGGGAGA 3’
           :: : ::::::::.::.:
query   3’ UAGUUGGCGGUUCUCUUU 5’

query=JS16_t0335149, target=FGSG_09952, score=4, range=322-339, strand=-1

target  5’ AACGACAGCCAAGAGAAG 3’
           : :.:: ::::::::::.
query   3’ UAGUUGGCGGUUCUCUUU 5’

query=JS16_t1387649, target=FGSG_05751, score=4, range=48-65, strand=-1

target  5’ UCGGUUUUUAUGGCAUCA 3’
            ::::::: ::.::::::
query   3’ UGCCAAAACUAUCGUAGU 5’

query=JS16_t1387649, target=FGSG_13017, score=4, range=665-682, strand=-1

target  5’ ACGGCUUUGAUACUAUCA 3’
           :::: ::::::: .::::
query   3’ UGCCAAAACUAUCGUAGU 5’

query=JS16_t1387649, target=FGSG_05705, score=4, range=1021-1038, strand=-1

target  5’ ACGUUUUUGAUAGUAUGA 3’
           ::: :::::::::.:: :
query   3’ UGCCAAAACUAUCGUAGU 5’

query=JS16_t1387649, target=FGSG_12041, score=4, range=2079-2096, strand=-1

target  5’ GUAGUUUGGAUAGCAUCA 3’
           .. :::: ::::::::::
query   3’ UGCCAAAACUAUCGUAGU 5’

query=JS16_t1387649, target=FGSG_13445, score=4, range=258-275, strand=-1

target  5’ AUAGUGUUGAUAGCAUCG 3’
           :. :: :::::::::::.
query   3’ UGCCAAAACUAUCGUAGU 5’

query=JS16_t1387649, target=FGSG_07192, score=4, range=2294-2312, strand=-1

target  5’ ACAGUGUUUGGUGGCAUCA 3’
           :: :: ::::.:.::::::
query   3’ UGCCA-AAACUAUCGUAGU 5’

query=JS16_t1387649, target=FGSG_02394, score=4, range=5577-5594, strand=-1

target  5’ ACAGUUUUGAUAGAAUCC 3’
           :: :::::::::: :::
query   3’ UGCCAAAACUAUCGUAGU 5’

query=JS16_t1387649, target=FGSG_00767, score=4, range=771-788, strand=-1

target  5’ CCGGCUUUGAUAGCAGCA 3’
            ::: :::::::::: ::
query   3’ UGCCAAAACUAUCGUAGU 5’

query=JS16_t1387649, target=FGSG_12237, score=4, range=186-204, strand=-1

target  5’ ACCGUUUUGGAUAGCAUCU 3’
           :: ::::: :::::::::
query   3’ UGCCAAAA-CUAUCGUAGU 5’

query=JS16_t1387649, target=FGSG_02217, score=4, range=906-922, strand=-1

target  5’ ACGG-CUUGGUAGCAUCA 3’
           ::::  :::.::::::::
query   3’ UGCCAAAACUAUCGUAGU 5’

query=JS16_t1387649, target=FGSG_01030, score=4, range=705-722, strand=-1

target  5’ AGAGUUGUGAUAGCAUCA 3’
           :  ::: ::::::::::
query   3’ UGCCAAAACUAUCGUAGU 5’

query=JS16_t1387649, target=FGSG_06881, score=4, range=2169-2186, strand=-1

target  5’ UUUGCUUUGAUAGCAUCG 3’
            . : ::::::::::::.
query   3’ UGCCAAAACUAUCGUAGU 5’

query=JS16_t1387649, target=FGSG_08465, score=4, range=501-517, strand=-1

target  5’ GCGG-UUUGAUCGCAUCG 3’
           .::: :::::: :::::.
query   3’ UGCCAAAACUAUCGUAGU 5’

query=JS16_t1387649, target=FGSG_08214, score=4, range=561-577, strand=-1

target  5’ ACUG-UUUGAUAGCUUCA 3’
           :: : ::::::::: :::
query   3’ UGCCAAAACUAUCGUAGU 5’

query=JS16_t3179959, target=FGSG_08067, score=3.5, range=364-384, strand=-1

target  5’ GUUCGUCGUGUGGAACUCAUC 3’
           ::::. : .:::::::::::::
query   3’ CAAGUUGAGCACCUUGAGUAG 5’

query=JS16_t3179959, target=FGSG_10127, score=4, range=48-68, strand=1
```

```
query=JS16_t0335149, target=FGSG_01702, score=4, range=1151-1167, strand=-1

target  5' AUC-ACUGCCAAGAGCAA 3'
           ::: ::.:::::::: ::
query   3' UAGUUGGCGGUUCUCUUU 5'

query=JS16_t0335149, target=FGSG_02665, score=4, range=485-501, strand=-1

target  5' AUC-ACCGCCAAGGGCAA 3'
           ::: :::::::::.: ::
query   3' UAGUUGGCGGUUCUCUUU 5'

query=JS16_t0335149, target=FGSG_08847, score=4, range=183-199, strand=-1

target  5' AUCCGCCGCCAAG-GAAG 3'
           ::: .:::::::: :::.
query   3' UAGUUGGCGGUUCUCUUU 5'

No results for JS16_t0136890
query=JS16_t1325745, target=FGSG_07798, score=3.5, range=2130-2149, strand=1

target  5' CACUAGC-ACGACUCUAUCAG 3'
           :: :::: :::::.::::::.
query   3' GUCAUCGUUGCUGGGAUAGUU 5'

query=JS16_t1325745, target=FGSG_13114, score=4, range=605-626, strand=1

target  5' CAUGUGCCGACGACCCUGUCAA 3'
           :: ::. :.::::::::.::::
query   3' GU-CAUCGUUGCUGGGAUAGUU 5'

query=JS16_t1325745, target=FGSG_06733, score=4, range=1871-1891, strand=-1

target  5' UGGUAACAACGACACUAUCAA 3'
           ..::: ::::::: :::::::
query   3' GUCAUCGUUGCUGGGAUAGUU 5'

No results for JS16_t1325754
No results for JS16_t2222160
query=JS16_t1325808, target=FGSG_00382, score=3, range=1620-1637, strand=1

target  5' CUCGGUUAGAUCGAGCCG 3'
           : : ::.:::::::::::
query   3' GCGACAGUCUAGCUCGGC 5'

query=JS16_t1325808, target=FGSG_08617, score=3, range=803-820, strand=-1

target  5' CGCUUUCAGAUCGAGCUU 3'
           :::: :::::::::::.
query   3' GCGACAGUCUAGCUCGGC 5'

query=JS16_t1325808, target=FGSG_01910, score=3, range=3323-3339, strand=-1

target  5' CGCU-UCAGAUCGAGUCA 3'
           :::: ::::::::::.:
query   3' GCGACAGUCUAGCUCGGC 5'

query=JS16_t1325808, target=FGSG_03574, score=3, range=545-561, strand=-1

target  5' CUCU-UCAGAUCGAGCUG 3'
           : :: :::::::::::.:
query   3' GCGACAGUCUAGCUCGGC 5'

query=JS16_t1325808, target=FGSG_06992, score=3.5, range=2403-2420, strand=-1

target  5' CGUUGUCGGAUCGGGCCC 3'
           ::.::::.:::::.:::
query   3' GCGACAGUCUAGCUCGGC 5'

query=JS16_t1325808, target=FGSG_07390, score=3.5, range=5-22, strand=-1

target  5' CGUAGUCAGAUCGAGCGG 3'
           ::. ::::::::::: :
query   3' GCGACAGUCUAGCUCGGC 5'

query=JS16_t1325808, target=FGSG_08056, score=3.5, range=401-417, strand=-1

target  5' UGC-GUCAGAUCGGGCCU 3'
           .:: :::::::::.:::
query   3' GCGACAGUCUAGCUCGGC 5'

query=JS16_t1325808, target=FGSG_13581, score=4, range=20-37, strand=1

target  5' CGAUGUCAGGUCUAGCCG 3'
           :: ::::::.:: :::::
query   3' GCGACAGUCUAGCUCGGC 5'

query=JS16_t1325808, target=FGSG_12645, score=4, range=1038-1055, strand=1

target  5' CGAUGUCAGAACGAGCCU 3'
           :: ::::::: ::::::
```

```
target  5' CUUUGACCCGUGGAACUCGUC 3'
             ::..:: ::::::::::.::
query   3' CAAGUUGAGCACCUUGAGUAG 5'

query=JS16_t3179959, target=FGSG_08245, score=4, range=1231-1251, strand=-1

target  5' GUUCAACUCGUAGACCUCAUC 3'
           ::::::::::: :: ::::::
query   3' CAAGUUGAGCACCUUGAGUAG 5'

query=JS16_t3179959, target=FGSG_10509, score=4, range=696-716, strand=-1

target  5' GUUGACCUUGUGGAACUCAUG 3'
           ::: : ::.::::::::::::
query   3' CAAGUUGAGCACCUUGAGUAG 5'

No results for JS16_t1387702
No results for JS16_t1387711
No results for JS16_t0801378
No results for JS16_t0366721
query=JS16_t4076667, target=FGSG_08773, score=3.5, range=39-58, strand=1

target  5' UCUUCGC-CCUCUCACCUUGA 3'
           .:::: : ::::::::::::
query   3' GGAAGAGUGGAGAGUGGAACC 5'

query=JS16_t4076667, target=FGSG_13740, score=3.5, range=1106-1126, strand=-1

target  5' CCUGCUCGCCUUUCACCUUGU 3'
           ::: :::.:::.::::::::
query   3' GGAAGAGUGGAGAGUGGAACC 5'

query=JS16_t4076667, target=FGSG_02147, score=4, range=21-40, strand=1

target  5' CCUCCUCACC-CUCGCCUUGG 3'
           ::: :::::: :::.::::::
query   3' GGAAGAGUGGAGAGUGGAACC 5'

query=JS16_t4076667, target=FGSG_11363, score=4, range=1857-1876, strand=-1

target  5' CCUUC-AACCUCUUACUUUGG 3'
           :::::  ::::::.::.::::
query   3' GGAAGAGUGGAGAGUGGAACC 5'

No results for JS16_t1387764
No results for JS16_t2283910
No results for JS16_t4076706
query=JS16_t0241127, target=FGSG_05386, score=4, range=496-515, strand=1

target  5' GCGUUUCAGUCAGUAUGGAC 3'
           :: .:.:::::: :::::::
query   3' CGAGAGGUCAGUGAUACCUG 5'

query=JS16_t0241127, target=FGSG_07760, score=4, range=181-201, strand=1

target  5' GCUGCCGCAGUCAUUAUGGAC 3'
           ::: :  ::::::.:::::::
query   3' CGA-GAGGUCAGUGAUACCUG 5'

No results for JS16_t3180171
No results for JS16_t0153546
No results for JS16_t4076819
No results for JS16_t0583198
No results for JS16_t1387932
query=JS16_t1387964, target=FGSG_01432, score=3, range=399-419, strand=-1

target  5' UUAA-UGAUCUUGAGGAAGCCA 3'
           ..:: :::::.:::::::::::
query   3' GGUUCACUAGGACUCCUUCGGU 5'

query=JS16_t1387964, target=FGSG_13158, score=3.5, range=217-237, strand=1

target  5' CCAAG-GGUUCUGAGGAAGCCC 3'
           ::::: :.:.:::::::::::
query   3' GGUUCACUAGGACUCCUUCGGU 5'

query=JS16_t1387964, target=FGSG_00551, score=3.5, range=4434-4455, strand=1

target  5' CCAAAAGAGCCUGAGGAAGCCG 3'
           ::::  :: :::::::::::.
query   3' GGUUCACUAGGACUCCUUCGGU 5'

query=JS16_t1387964, target=FGSG_02371, score=4, range=1374-1396, strand=1

target  5' CCCAGUGAUGCUGGAGGAAGCCA 3'
           :: :::::: :: ::::::::::
query   3' GGUUCACUAGGA-CUCCUUCGGU 5'

No results for JS16_t1387985
query=JS16_t0283971, target=FGSG_11460, score=4, range=270-290, strand=1

target  5' AGGCGCUAUGGGUGUAAUCUU 3'
```

```
query   3’ GCGACAGUCUAGCUCGGC 5’

query=JS16_t1325808, target=FGSG_06195, score=4, range=203-220, strand=1

target  5’ CGAUAUCAGAUCGAGCUU 3’
           :: : :::::::::::.
query   3’ GCGACAGUCUAGCUCGGC 5’

query=JS16_t1325808, target=FGSG_06052, score=4, range=860-876, strand=1

target  5’ GGCU-UCAGAUCGAGUCC 3’
            ::: ::::::::::.:
query   3’ GCGACAGUCUAGCUCGGC 5’

query=JS16_t1325808, target=FGSG_04346, score=4, range=938-955, strand=1

target  5’ CUCUAUCAGAUCGAGACG 3’
           : :: :::::::::: ::
query   3’ GCGACAGUCUAGCUCGGC 5’

query=JS16_t1325808, target=FGSG_03664, score=4, range=93-109, strand=1

target  5’ UGUUGU-AGAUCGAGCCU 3’
           .:.::: ::::::::::
query   3’ GCGACAGUCUAGCUCGGC 5’

query=JS16_t1325808, target=FGSG_12769, score=4, range=467-483, strand=1

target  5’ CGCCCUCAGAU-GAGCCG 3’
           :::  :::::: ::::::
query   3’ GCGACAGUCUAGCUCGGC 5’

query=JS16_t1325808, target=FGSG_11523, score=4, range=588-605, strand=-1

target  5’ CUCUGUCGGAUUGAGUCG 3’
           : :::::.:::.:::.::
query   3’ GCGACAGUCUAGCUCGGC 5’

query=JS16_t1325808, target=FGSG_06679, score=4, range=545-562, strand=-1

target  5’ CGCUAUCAGAUAGAGCUG 3’
           :::: :::::: ::::.:
query   3’ GCGACAGUCUAGCUCGGC 5’

query=JS16_t1325808, target=FGSG_11786, score=4, range=1661-1678, strand=-1

target  5’ ACCAGUCAGAUCGAGCUG 3’
             : ::::::::::::.:
query   3’ GCGACAGUCUAGCUCGGC 5’

query=JS16_t1325808, target=FGSG_13878, score=4, range=27503-27520, strand=-1

target  5’ CGCUCUCAGAUCCAGCCU 3’
           :::: ::::::: ::::
query   3’ GCGACAGUCUAGCUCGGC 5’

query=JS16_t1325808, target=FGSG_05874, score=4, range=209-225, strand=-1

target  5’ AGCU-UCAGAUCGGGCCU 3’
            ::: ::::::::.:::
query   3’ GCGACAGUCUAGCUCGGC 5’

query=JS16_t0170899, target=FGSG_08078, score=3, range=275-292, strand=1

target  5’ UGAUGGAUUGG-UUCUAUG 3’
           .::.::::::: :::::::
query   3’ GCUGCCUAACCAAAGAUAC 5’

query=JS16_t0170899, target=FGSG_10717, score=3, range=472-489, strand=1

target  5’ CCAC-GAUUGGUUUCUAUC 3’
           : :: :::::::::::::
query   3’ GCUGCCUAACCAAAGAUAC 5’

query=JS16_t0170899, target=FGSG_01977, score=4, range=3429-3448, strand=1

target  5’ CGACCCGAUUGGUCUCUAUG 3’
           ::: : ::::::: ::::::
query   3’ GCU-GCCUAACCAAAGAUAC 5’

query=JS16_t0170899, target=FGSG_02247, score=4, range=740-757, strand=1

target  5’ CUCCGGAUUGG-UUCUAUG 3’
           :  :::::::: :::::::
query   3’ GCUGCCUAACCAAAGAUAC 5’

query=JS16_t0170899, target=FGSG_06989, score=4, range=756-775, strand=-1

target  5’ CGACGUGGUUGGCUUCUAUG 3’
           ::::: :.:::: :::::::
query   3’ GCUGC-CUAACCAAAGAUAC 5’

           ::.: :.:::::::::: :::
query   3’ UCUG-GGUACCCACAUUCGAA 5’

query=JS16_t0283971, target=FGSG_11911, score=4, range=505-523, strand=1

target  5’ AGAACCAUGGGUG-GAGCUU 3’
           ::: ::::::::: .:::::
query   3’ UCUGGGUACCCACAUUCGAA 5’

query=JS16_t0283971, target=FGSG_03345, score=4, range=1603-1621, strand=1

target  5’ AGGGUCAUGGGUGU-AGCUU 3’
           ::. .::::::::: :::::
query   3’ UCUGGGUACCCACAUUCGAA 5’

query=JS16_t0283971, target=FGSG_03629, score=4, range=525-543, strand=-1

target  5’ AGAACCAUGGGU-UGAGCUU 3’
           ::: :::::::: :.:::::
query   3’ UCUGGGUACCCACAUUCGAA 5’

query=JS16_t2284127, target=FGSG_06215, score=3, range=23-41, strand=-1

target  5’ CGGCGGGUUUGCGCCUCGG 3’
            :::..::::::.::::::
query   3’ ACCGUUCAAACGUGGAGCC 5’

query=JS16_t2284127, target=FGSG_01631, score=3.5, range=731-749, strand=1

target  5’ UGCCGAGUUUGCACUUUGG 3’
           :: :.:::::::::.:.::
query   3’ ACCGUUCAAACGUGGAGCC 5’

query=JS16_t2284127, target=FGSG_01295, score=3.5, range=142-160, strand=1

target  5’ UGGGGCGUUUGCGCCUCGG 3’
           ::: . ::::::.::::::
query   3’ ACCGUUCAAACGUGGAGCC 5’

query=JS16_t2284127, target=FGSG_08538, score=3.5, range=416-434, strand=-1

target  5’ UUGCGAGUUUGCGCCUCGA 3’
           : ::.:::::::.:::::
query   3’ ACCGUUCAAACGUGGAGCC 5’

query=JS16_t2284127, target=FGSG_06369, score=4, range=649-667, strand=1

target  5’ UGGCAAGCUUUCACCUCGG 3’
           ::::::: :: ::::::::
query   3’ ACCGUUCAAACGUGGAGCC 5’

query=JS16_t2284127, target=FGSG_02680, score=4, range=1437-1455, strand=1

target  5’ UGUCAAGUUUGCAGCUUGG 3’
           :: :::::::::: ::.::
query   3’ ACCGUUCAAACGUGGAGCC 5’

query=JS16_t2284127, target=FGSG_13485, score=4, range=204-221, strand=1

target  5’ UGGCAAG-UUGCAUCUUGG 3’
           ::::::: :::::.::.::
query   3’ ACCGUUCAAACGUGGAGCC 5’

query=JS16_t2284127, target=FGSG_09129, score=4, range=4629-4647, strand=-1

target  5’ AUGCGGGUUUGCACCUCGU 3’
             ::..::::::::::::
query   3’ ACCGUUCAAACGUGGAGCC 5’

query=JS16_t2284127, target=FGSG_05650, score=4, range=98-116, strand=-1

target  5’ UGGCAUGUUUGCAGCUUGG 3’
           ::::: ::::::: ::.::
query   3’ ACCGUUCAAACGUGGAGCC 5’

query=JS16_t2284127, target=FGSG_13500, score=4, range=134-151, strand=-1

target  5’ UGGCACGUUUGCG-CUCGG 3’
           ::::: ::::::. :::::
query   3’ ACCGUUCAAACGUGGAGCC 5’

query=JS16_t2284127, target=FGSG_07713, score=4, range=95-112, strand=-1

target  5’ UGGU-GGCUUGCACCUCGG 3’
           :::. .: :::::::::::
query   3’ ACCGUUCAAACGUGGAGCC 5’

query=JS16_t2284127, target=FGSG_04418, score=4, range=27-44, strand=-1

target  5’ UGGC-CGUUUGCACCCCGG 3’
           ::::  ::::::::: :::
query   3’ ACCGUUCAAACGUGGAGCC 5’
```

```
No results for JS16_t3118282
No results for JS16_t0149809
query=JS16_t4015053, target=FGSG_07195, score=3.5, range=395-417, strand=1

target  5’ GUGCAGAAGACAUUCUCGACGAA 3’
           ::: :.: :::::::.:::::::
query   3’ CACAUUUGCUGUAAGGGCUGCUU 5’

No results for JS16_t0305374
query=JS16_t0575789, target=FGSG_07187, score=2.5, range=1793-1811, strand=1

target  5’ UGAAGAAGUAUGGCAAUGG 3’
           ::::::::::::: ::::.
query   3’ ACUUCUUCAUACC-UUACU 5’

query=JS16_t0575789, target=FGSG_10563, score=2.5, range=88-105, strand=-1

target  5’ GGGAGAGGUAUGGAAUGA 3’
            :.:::.:::::::::::
query   3’ ACUUCUUCAUACCUUACU 5’

query=JS16_t0575789, target=FGSG_00595, score=3, range=2738-2756, strand=1

target  5’ GGAAGAAAGUAUGGGAUGA 3’
            :::: ::::::::.::::
query   3’ ACUUC-UUCAUACCUUACU 5’

query=JS16_t0575789, target=FGSG_09394, score=3, range=1248-1264, strand=1

target  5’ CGAAG-AGUAUGGAAUGA 3’
            :::: ::::::::::::
query   3’ ACUUCUUCAUACCUUACU 5’

query=JS16_t0575789, target=FGSG_09086, score=3, range=342-359, strand=-1

target  5’ UGGAGAAGUAUGAAAUGG 3’
           ::.::::::::: ::::.
query   3’ ACUUCUUCAUACCUUACU 5’

query=JS16_t0575789, target=FGSG_04894, score=3.5, range=1296-1313, strand=1

target  5’ UGGAGAAGUAUCGAAUGU 3’
           ::.:::::::: :::::
query   3’ ACUUCUUCAUACCUUACU 5’

query=JS16_t0575789, target=FGSG_06536, score=3.5, range=353-371, strand=1

target  5’ UGAAGGAGUAUGAGAAUGC 3’
           :::::.:::::: :::::
query   3’ ACUUCUUCAUAC-CUUACU 5’

query=JS16_t0575789, target=FGSG_04622, score=3.5, range=993-1010, strand=1

target  5’ UGCAGAUGUAUGGAAUGG 3’
           :: ::: ::::::::::.
query   3’ ACUUCUUCAUACCUUACU 5’

query=JS16_t0575789, target=FGSG_11504, score=3.5, range=213-229, strand=1

target  5’ AGAGGAA-UAUGGAAUGA 3’
            ::.::: ::::::::::
query   3’ ACUUCUUCAUACCUUACU 5’

query=JS16_t0575789, target=FGSG_07251, score=3.5, range=1716-1732, strand=1

target  5’ UGAA-AAAUAUGGAAUGG 3’
           :::: :: :::::::::.
query   3’ ACUUCUUCAUACCUUACU 5’

query=JS16_t0575789, target=FGSG_04676, score=3.5, range=238-256, strand=-1

target  5’ UGAAGAAAGCAUGGAAUGG 3’
           ::::: ::: ::::::::.
query   3’ ACUUC-UUCAUACCUUACU 5’

query=JS16_t0575789, target=FGSG_08105, score=4, range=1229-1246, strand=1

target  5’ UGAAGGAGGAUGGAGUGA 3’
           :::::.:: :::::.:::
query   3’ ACUUCUUCAUACCUUACU 5’

query=JS16_t0575789, target=FGSG_05935, score=4, range=564-581, strand=1

target  5’ UGAAGGAGAAUGGAAUGU 3’
           :::::.:: ::::::::
query   3’ ACUUCUUCAUACCUUACU 5’

query=JS16_t0575789, target=FGSG_02346, score=4, range=1373-1390, strand=1

target  5’ CGGAGAAGUAUGGAAAGG 3’
            :.:::::::::::: :.
query   3’ ACUUCUUCAUACCUUACU 5’
query=JS16_t2284127, target=FGSG_11212, score=4, range=212-229, strand=-1

target  5’ AGGCAAGUUUG-AUCUCGG 3’
            :::::::::: :.::::
query   3’ ACCGUUCAAACGUGGAGCC 5’

No results for JS16_t1388013
query=JS16_t0150077, target=FGSG_13422, score=4, range=2138-2157, strand=1

target  5’ UCGAUCCUC-UUCUCGUUAAC 3’
           ::.::::.: ::::::::::
query   3’ AGUUAGGGGAAAGAGCAAUUA 5’

query=JS16_t0150077, target=FGSG_10446, score=4, range=1089-1109, strand=-1

target  5’ GUAAUCUCCUUUCUCGUCAAU 3’
            .::::.:::::::::: :::
query   3’ AGUUAGGGGAAAGAGCAAUUA 5’

No results for JS16_t2284155
query=JS16_t0801420, target=FGSG_06934, score=4, range=322-340, strand=1

target  5’ CAGGGCGAGCAUACUUACU 3’
           ::: ::.::::::::: :
query   3’ GUCACGUUCGUAUGAAUCA 5’

query=JS16_t0801420, target=FGSG_10021, score=4, range=917-935, strand=1

target  5’ CAGAACAAGUAUACUUGGU 3’
           :::  ::::.::::::.::
query   3’ GUCACGUUCGUAUGAAUCA 5’

query=JS16_t0801420, target=FGSG_11477, score=4, range=891-908, strand=-1

target  5’ GAGU-CAAGCAUACUUGGG 3’
            ::: :::::::::::.:
query   3’ GUCACGUUCGUAUGAAUCA 5’

query=JS16_t0474244, target=FGSG_01647, score=4, range=593-613, strand=-1

target  5’ UUACACCGGGUUUCUGCACCU 3’
           ::    :::::::::::::::
query   3’ AACACAGCCCAAAGACGUGGA 5’

No results for JS16_t3180491
No results for JS16_t2284341
No results for JS16_t1388263
query=JS16_t3180554, target=FGSG_13258, score=4, range=721-742, strand=-1

target  5’ AGGUCGAGCUCUCGAAGUUGGC 3’
           :: :: :: ::::::::::::
query   3’ UCAAGGUCCAGAGCUUCAACCA 5’

No results for JS16_t0801452
No results for JS16_t0801460
No results for JS16_t3180697
No results for JS16_t1388401
No results for JS16_t1388454
No results for JS16_t4077380
query=JS16_t0583264, target=FGSG_11026, score=4, range=13547-13570, strand=-1

target  5’ UUGGGAACGUCGCUUUGAACUGUG 3’
           :::::::::: :::::: ::::.:
query   3’ AACCCUUGCAACGAAAC-UGACGC 5’

query=JS16_t4077430, target=FGSG_10665, score=3, range=521-541, strand=1

target  5’ AAGGACGUCCUGCAUUUCAGA 3’
           :::.: .:::::::::::::
query   3’ UUCUUAUAGGACGUAAAGUCG 5’

query=JS16_t4077430, target=FGSG_01760, score=3, range=815-834, strand=1

target  5’ AACAAUA-CCUGUAUUUCAGC 3’
           :: :::: ::::.::::::::
query   3’ UUCUUAUAGGACGUAAAGUCG 5’

query=JS16_t4077430, target=FGSG_06970, score=4, range=3025-3044, strand=-1

target  5’ AAGAA-ACCCUGCAUUUCAAC 3’
           ::::: : ::::::::::: :
query   3’ UUCUUAUAGGACGUAAAGUCG 5’

query=JS16_t4077454, target=FGSG_08761, score=2.5, range=796-813, strand=-1

target  5’ ACGCUCGUCGAAGUAUUU 3’
           ::::.::::::::::.:
query   3’ UGCGGGCAGCUUCAUGAC 5’

query=JS16_t4077454, target=FGSG_01116, score=3, range=329-345, strand=1
```

```
query=JS16_t0575789, target=FGSG_05094, score=4, range=365-382, strand=1

target  5’ CGGAGAAGUAUGGAAAGG 3’
            :.:::::::::::: :.
query   3’ ACUUCUUCAUACCUUACU 5’

query=JS16_t0575789, target=FGSG_08372, score=4, range=738-754, strand=1

target  5’ UGAAGAGGUGUGGAA-GA 3’
           ::::::.::.::::: ::
query   3’ ACUUCUUCAUACCUUACU 5’

query=JS16_t0575789, target=FGSG_01007, score=4, range=279-296, strand=1

target  5’ GGAGCAGGUAUGGAAUGG 3’
            ::.  :.::::::::::.
query   3’ ACUUCUUCAUACCUUACU 5’

query=JS16_t0575789, target=FGSG_06071, score=4, range=1311-1327, strand=1

target  5’ UGAGGAAGUGUGG-AUGG 3’
           :::.:::::.::: :::.
query   3’ ACUUCUUCAUACCUUACU 5’

query=JS16_t0575789, target=FGSG_10343, score=4, range=1427-1444, strand=1

target  5’ UGAAGAAGUAUGGCAAGA 3’
           ::::::::::::: : ::
query   3’ ACUUCUUCAUACCUUACU 5’

query=JS16_t0575789, target=FGSG_10193, score=4, range=197-215, strand=1

target  5’ UGAAGAAGUAUGGCAAAGA 3’
           ::::::::::::: :: ::
query   3’ ACUUCUUCAUACC-UUACU 5’

query=JS16_t0575789, target=FGSG_06374, score=4, range=45-62, strand=1

target  5’ GGAACAACUAUGGAAUGA 3’
            ::: :: ::::::::::
query   3’ ACUUCUUCAUACCUUACU 5’

query=JS16_t0575789, target=FGSG_10398, score=4, range=227-244, strand=1

target  5’ CGAAAAAGUAUGGAAAGA 3’
            ::: :::::::::: ::
query   3’ ACUUCUUCAUACCUUACU 5’

query=JS16_t0575789, target=FGSG_00687, score=4, range=283-300, strand=1

target  5’ UUAAAAAGUAUGGACUGA 3’
           : :: ::::::::: :::
query   3’ ACUUCUUCAUACCUUACU 5’

query=JS16_t0575789, target=FGSG_12891, score=4, range=231-248, strand=1

target  5’ UCAUGAAGUAUGGAAGGA 3’
           : : ::::::::::: ::
query   3’ ACUUCUUCAUACCUUACU 5’

query=JS16_t0575789, target=FGSG_00340, score=4, range=652-668, strand=1

target  5’ AGAA-AAAUAUGGAAUGA 3’
            ::: :: ::::::::::
query   3’ ACUUCUUCAUACCUUACU 5’

query=JS16_t0575789, target=FGSG_05337, score=4, range=4124-4140, strand=1

target  5’ UGACCAAGUAUGGAA-GA 3’
           :::  :::::::::: ::
query   3’ ACUUCUUCAUACCUUACU 5’

query=JS16_t0575789, target=FGSG_00438, score=4, range=1074-1090, strand=1

target  5’ UGUUGAAG-AUGGAAUGA 3’
           ::  :::: :::::::::
query   3’ ACUUCUUCAUACCUUACU 5’

query=JS16_t0575789, target=FGSG_07064, score=4, range=231-248, strand=-1

target  5’ GGAGGGAGUGUGGAAUGG 3’
            ::.:.:::.:::::::.
query   3’ ACUUCUUCAUACCUUACU 5’

query=JS16_t0575789, target=FGSG_08483, score=4, range=152-169, strand=-1

target  5’ UGAAGAGGUAGGGGAUGA 3’
           ::::::.::: ::.::::
query   3’ ACUUCUUCAUACCUUACU 5’

query=JS16_t0575789, target=FGSG_09011, score=4, range=689-706, strand=-1

target  5’ ACGACC-UCGAAGUACUG 3’
           ::: :: :::::::::::
query   3’ UGCGGGCAGCUUCAUGAC 5’

query=JS16_t4077454, target=FGSG_00122, score=3, range=517-534, strand=-1

target  5’ GCGCUCGUCGAGGUACUU 3’
           .:::.::::::.:::::
query   3’ UGCGGGCAGCUUCAUGAC 5’

query=JS16_t4077454, target=FGSG_07007, score=3, range=451-467, strand=-1

target  5’ ACG-CCGUCGAGGUGCUG 3’
           ::: :::::::.::.:::
query   3’ UGCGGGCAGCUUCAUGAC 5’

query=JS16_t4077454, target=FGSG_02743, score=3.5, range=1769-1786, strand=1

target  5’ AUAUUCGUCGAAGUACUC 3’
           :.  ..:::::::::::
query   3’ UGCGGGCAGCUUCAUGAC 5’

query=JS16_t4077454, target=FGSG_00858, score=3.5, range=968-984, strand=1

target  5’ AC-CUUGUCGAAGUAUUG 3’
           :: :..:::::::::.::
query   3’ UGCGGGCAGCUUCAUGAC 5’

query=JS16_t4077454, target=FGSG_11317, score=3.5, range=14-31, strand=-1

target  5’ AUGCCUGUCGAACUACUG 3’
           :.:::.:::::: :::::
query   3’ UGCGGGCAGCUUCAUGAC 5’

query=JS16_t4077454, target=FGSG_04671, score=3.5, range=1131-1148, strand=-1

target  5’ AAGCUCGUCGAAGUGUUG 3’
           : ::.:::::::::..::
query   3’ UGCGGGCAGCUUCAUGAC 5’

query=JS16_t4077454, target=FGSG_09359, score=3.5, range=1138-1155, strand=-1

target  5’ GCGGACGUCGGAGUACUG 3’
           .::  :::::.:::::::
query   3’ UGCGGGCAGCUUCAUGAC 5’

query=JS16_t4077454, target=FGSG_04298, score=3.5, range=2251-2267, strand=-1

target  5’ UUGCCCGUCGAAGU-CUG 3’
            .:::::::::::: :::
query   3’ UGCGGGCAGCUUCAUGAC 5’

query=JS16_t4077454, target=FGSG_07198, score=3.5, range=1962-1978, strand=-1

target  5’ AU-CCCGUCGAAGUGCUC 3’
           :.  :::::::::::.::
query   3’ UGCGGGCAGCUUCAUGAC 5’

query=JS16_t4077454, target=FGSG_00916, score=3.5, range=4704-4720, strand=-1

target  5’ ACG-UCGGCGAAGUACUG 3’
           ::: .:: ::::::::::
query   3’ UGCGGGCAGCUUCAUGAC 5’

query=JS16_t4077454, target=FGSG_06999, score=4, range=697-714, strand=1

target  5’ ACGGCCGUAGAAGUAUUG 3’
           ::: :::: ::::::.::
query   3’ UGCGGGCAGCUUCAUGAC 5’

query=JS16_t4077454, target=FGSG_04700, score=4, range=1145-1162, strand=1

target  5’ UCGGCCGUCGAAGUAUUC 3’
            :: :::::::::::.:
query   3’ UGCGGGCAGCUUCAUGAC 5’

query=JS16_t4077454, target=FGSG_06361, score=4, range=2967-2984, strand=1

target  5’ GCAGUCGUUGAAGUACUG 3’
           .:  .:::.:::::::::
query   3’ UGCGGGCAGCUUCAUGAC 5’

query=JS16_t4077454, target=FGSG_03424, score=4, range=1301-1317, strand=1

target  5’ AC-CCCGUCGAAGUUCUC 3’
           :: ::::::::::: ::
query   3’ UGCGGGCAGCUUCAUGAC 5’

query=JS16_t4077454, target=FGSG_06013, score=4, range=993-1009, strand=1

target  5’ ACGCCC-UCGAACUACUG 3’
           :::::: ::::: :::::
```

```
target  5’ UGAUGAAGUACGGGAUGA 3’
           ::: :::::: ::.::::
query   3’ ACUUCUUCAUACCUUACU 5’

query=JS16_t0575789, target=FGSG_07847, score=4, range=1350-1367, strand=-1

target  5’ UGAUGAAGUAUGGAAUCU 3’
           ::: ::::::::::::
query   3’ ACUUCUUCAUACCUUACU 5’

query=JS16_t0575789, target=FGSG_03024, score=4, range=1289-1306, strand=-1

target  5’ CGUAGAAGUAUGUAAUGA 3’
            : ::::::::: :::::
query   3’ ACUUCUUCAUACCUUACU 5’

No results for JS16_t2222355
query=JS16_t0254369, target=FGSG_12487, score=2, range=362-379, strand=-1

target  5’ UCUUGGCGAUAGAAGUCU 3’
           :  :::::::::::::::
query   3’ AUCACCGCUAUCUUCAGA 5’

query=JS16_t0254369, target=FGSG_10262, score=3.5, range=912-929, strand=-1

target  5’ UGGAAGCGAUAGAAGUCC 3’
           :.:  ::::::::::::
query   3’ AUCACCGCUAUCUUCAGA 5’

query=JS16_t0254369, target=FGSG_04099, score=4, range=485-502, strand=1

target  5’ AAGUGGCGAUUGGAGUCU 3’
            ::::::::: :.:::::
query   3’ AUCACCGCUAUCUUCAGA 5’

query=JS16_t0254369, target=FGSG_09965, score=4, range=321-338, strand=1

target  5’ UAAAGGUGAUGGAAGUCU 3’
           ::  ::.:::.:::::::
query   3’ AUCACCGCUAUCUUCAGA 5’

query=JS16_t0254369, target=FGSG_08623, score=4, range=159-176, strand=1

target  5’ UACUCGCGAUAGAAGUUC 3’
           :: : :::::::::::.
query   3’ AUCACCGCUAUCUUCAGA 5’

query=JS16_t0254369, target=FGSG_04191, score=4, range=1404-1420, strand=1

target  5’ UAUUGG-GAUAGAAGUUU 3’
           :: ::: :::::::::.:
query   3’ AUCACCGCUAUCUUCAGA 5’

query=JS16_t0254369, target=FGSG_01538, score=4, range=755-771, strand=1

target  5’ UUGAGGCGAU-GAAGUCU 3’
           : : :::::: :::::::
query   3’ AUCACCGCUAUCUUCAGA 5’

query=JS16_t0254369, target=FGSG_12230, score=4, range=8270-8287, strand=-1

target  5’ GAGUGGUGGUAGGAGUCU 3’
            :::::.:.:::.:::::
query   3’ AUCACCGCUAUCUUCAGA 5’

query=JS16_t0254369, target=FGSG_02089, score=4, range=548-565, strand=-1

target  5’ UAGUGUCGAUAGAGGUUU 3’
           ::::: :::::::.::.:
query   3’ AUCACCGCUAUCUUCAGA 5’

query=JS16_t0254369, target=FGSG_02394, score=4, range=3125-3141, strand=-1

target  5’ UAGUGGCGAUGGGAG-CU 3’
           ::::::::::.:.:: ::
query   3’ AUCACCGCUAUCUUCAGA 5’

query=JS16_t0254369, target=FGSG_03399, score=4, range=617-634, strand=-1

target  5’ UAGUGUCGCUAGAAGUCU 3’
           ::::: :: :::::::::
query   3’ AUCACCGCUAUCUUCAGA 5’

query=JS16_t0254369, target=FGSG_07522, score=4, range=179-196, strand=-1

target  5’ UUGUGGCGGCAGAAGUCU 3’
           : :::::::. ::::::::
query   3’ AUCACCGCUAUCUUCAGA 5’

query=JS16_t0254369, target=FGSG_08456, score=4, range=470-488, strand=-1

query   3’ UGCGGGCAGCUUCAUGAC 5’

query=JS16_t4077454, target=FGSG_05797, score=4, range=778-794, strand=1

target  5’ CCGUUCGUCG-AGUACUG 3’
            ::..:::: :::::::
query   3’ UGCGGGCAGCUUCAUGAC 5’

query=JS16_t4077454, target=FGSG_03416, score=4, range=875-891, strand=1

target  5’ ACACCCGUC-AAGUGCUG 3’
           :: :::::: ::::.:::
query   3’ UGCGGGCAGCUUCAUGAC 5’

query=JS16_t4077454, target=FGSG_03267, score=4, range=154-170, strand=1

target  5’ ACGCCAGUC-AAGUACUG 3’
           ::::: ::: ::::::::
query   3’ UGCGGGCAGCUUCAUGAC 5’

query=JS16_t4077454, target=FGSG_13583, score=4, range=3567-3584, strand=-1

target  5’ GUGCCGGUUGAAGUACUG 3’
           ..::: ::.:::::::::
query   3’ UGCGGGCAGCUUCAUGAC 5’

query=JS16_t4077454, target=FGSG_05896, score=4, range=940-957, strand=-1

target  5’ UUGCUCGUCGAGGUACUC 3’
            .::.::::::.:::::
query   3’ UGCGGGCAGCUUCAUGAC 5’

query=JS16_t4077454, target=FGSG_12050, score=4, range=259-276, strand=-1

target  5’ GAGCUUGUCGAAGUACUC 3’
           . ::..:::::::::::
query   3’ UGCGGGCAGCUUCAUGAC 5’

query=JS16_t4077454, target=FGSG_12055, score=4, range=204-221, strand=-1

target  5’ AAACCCGUCGAAGUGCUC 3’
           :  :::::::::::.::
query   3’ UGCGGGCAGCUUCAUGAC 5’

query=JS16_t4077454, target=FGSG_04053, score=4, range=1310-1326, strand=-1

target  5’ AC-CCCGUCCAAGUGCUG 3’
           :: :::::: ::::.:::
query   3’ UGCGGGCAGCUUCAUGAC 5’

query=JS16_t4077454, target=FGSG_01556, score=4, range=454-470, strand=-1

target  5’ ACG-CCGUCGAGGUAGUG 3’
           ::: :::::::.::: ::
query   3’ UGCGGGCAGCUUCAUGAC 5’

query=JS16_t4077454, target=FGSG_03418, score=4, range=1534-1550, strand=-1

target  5’ UCG-CCGUGGAAGUACUG 3’
            :: :::: :::::::::
query   3’ UGCGGGCAGCUUCAUGAC 5’

query=JS16_t4077454, target=FGSG_10868, score=4, range=1060-1076, strand=-1

target  5’ ACGCCAG-CGAAGUACUG 3’
           ::::: : ::::::::::
query   3’ UGCGGGCAGCUUCAUGAC 5’

No results for JS16_t1388538
No results for JS16_t0691868
query=JS16_t4077490, target=FGSG_10451, score=3.5, range=459-478, strand=-1

target  5’ CUUCUCGCUUACCGUUCUUG 3’
            :::::.:: ::::::::::
query   3’ CAAGAGUGACUGGCAAGAAC 5’

query=JS16_t4077490, target=FGSG_07960, score=4, range=261-281, strand=1

target  5’ GUUCUCACUGACACGUUCUCG 3’
           :::::::::::: :::::: :
query   3’ CAAGAGUGACUG-GCAAGAAC 5’

query=JS16_t4077490, target=FGSG_09731, score=4, range=380-399, strand=-1

target  5’ GUACUUGUUGACCGUUCUUA 3’
           :: ::...:::::::::::
query   3’ CAAGAGUGACUGGCAAGAAC 5’

No results for JS16_t3180932
query=JS16_t3180966, target=FGSG_07670, score=2, range=553-570, strand=1

target  5’ GCCUAUGAGUCGG-GGACU 3’
```

```
target  5' UAGUUGGUGACAGAAGUCU 3'
           ::: :::.:: ::::::::
query   3' AUC-ACCGCUAUCUUCAGA 5'

query=JS16_t0254369, target=FGSG_08459, score=4, range=693-710, strand=-1

target  5' AACUGGCGAUAGAGGUCA 3'
            : ::::::::::.:::
query   3' AUCACCGCUAUCUUCAGA 5'

query=JS16_t0254369, target=FGSG_05927, score=4, range=1355-1372, strand=-1

target  5' UCUCGGUGAUAGAAGUCU 3'
           :   ::.::::::::::
query   3' AUCACCGCUAUCUUCAGA 5'

query=JS16_t0254369, target=FGSG_03517, score=4, range=362-379, strand=-1

target  5' GAUUCGCGAUAGAGGUCU 3'
            : : ::::::::.::::
query   3' AUCACCGCUAUCUUCAGA 5'

query=JS16_t0254369, target=FGSG_04372, score=4, range=171-188, strand=-1

target  5' UAGACGCGAUAGGAGUCG 3'
           :::  :::::::.::::
query   3' AUCACCGCUAUCUUCAGA 5'

query=JS16_t0254369, target=FGSG_04284, score=4, range=1460-1476, strand=-1

target  5' UUGUGGUGAUAG-AGUCU 3'
           : ::::.::::: :::::
query   3' AUCACCGCUAUCUUCAGA 5'

query=JS16_t0254369, target=FGSG_08887, score=4, range=1660-1677, strand=-1

target  5' UAUUUGAGAUAGAAGUCU 3'
           :: : : ::::::::::
query   3' AUCACCGCUAUCUUCAGA 5'

query=JS16_t0254369, target=FGSG_01573, score=4, range=1316-1333, strand=-1

target  5' UAGCAGCGAUAGCAGUCU 3'
           :::  ::::::: :::::
query   3' AUCACCGCUAUCUUCAGA 5'

query=JS16_t0254369, target=FGSG_00958, score=4, range=852-868, strand=-1

target  5' UAGCAGCGAUAG-AGUCU 3'
           :::  ::::::: :::::
query   3' AUCACCGCUAUCUUCAGA 5'

query=JS16_t0254369, target=FGSG_01882, score=4, range=456-472, strand=-1

target  5' UCAUGGC-AUAGAAGUCU 3'
           :  :::: ::::::::::
query   3' AUCACCGCUAUCUUCAGA 5'

No results for JS16_t4015201
No results for JS16_t0335162
No results for JS16_t1325986
No results for JS16_t2222411
query=JS16_t0254373, target=FGSG_13297, score=3, range=1240-1257, strand=1

target  5' GACCCAAAGAAAAUGCAC 3'
           ::: :: :::::::::::
query   3' CUGAGUCUCUUUUACGUG 5'

query=JS16_t0254373, target=FGSG_02743, score=3, range=2479-2496, strand=1

target  5' GAGACAGAGAAGAUGCAC 3'
           ::  :::::::.::::::
query   3' CUGAGUCUCUUUUACGUG 5'

query=JS16_t0254373, target=FGSG_07078, score=3, range=923-941, strand=1

target  5' GACAGCAGAGAAAGUGCAC 3'
           :::  ::::::::.:::::
query   3' CUG-AGUCUCUUUUACGUG 5'

query=JS16_t0254373, target=FGSG_13073, score=3, range=300-316, strand=1

target  5' CACU-AGAGAAAAUGCAG 3'
            ::: ::::::::::::
query   3' CUGAGUCUCUUUUACGUG 5'

query=JS16_t0254373, target=FGSG_00091, score=3, range=1552-1568, strand=-1

target  5' GAAUCAGAG-AAAUGCAC 3'
           :: :::::: ::::::::
query   3' CUGAGUCUCUUUUACGUG 5'
```

```
           ::::::::::::: :::::
query   3' CGGAUACUCAGCCACCUGA 5'

query=JS16_t3180966, target=FGSG_06548, score=3, range=887-905, strand=1

target  5' GUCUGGGAGUCGGUGGACA 3'
           :.::. ::::::::::::
query   3' CGGAUACUCAGCCACCUGA 5'

query=JS16_t3180966, target=FGSG_13987, score=3.5, range=1348-1366, strand=1

target  5' GCUCCUGAGUCGGUGGACC 3'
           ::.   :::::::::::::
query   3' CGGAUACUCAGCCACCUGA 5'

query=JS16_t3180966, target=FGSG_12745, score=4, range=2731-2749, strand=1

target  5' GUUGAUGAGUUGGUGGACG 3'
           :.. ::::::.:::::::
query   3' CGGAUACUCAGCCACCUGA 5'

query=JS16_t3180966, target=FGSG_08934, score=4, range=379-397, strand=1

target  5' GCCUCGGAGUUGGUGGACC 3'
           ::::  ::::.:::::::
query   3' CGGAUACUCAGCCACCUGA 5'

query=JS16_t3180966, target=FGSG_10088, score=4, range=1060-1077, strand=1

target  5' GCCGAUGAGUCGG-GGAUU 3'
           ::: ::::::::: :::.:
query   3' CGGAUACUCAGCCACCUGA 5'

query=JS16_t3180966, target=FGSG_00566, score=4, range=139-156, strand=1

target  5' GACGAUGAGUCGGUGG-CU 3'
           : : :::::::::::: ::
query   3' CGGAUACUCAGCCACCUGA 5'

query=JS16_t3180966, target=FGSG_08290, score=4, range=1530-1548, strand=-1

target  5' GCCUCUGGGUCGGUGGGCC 3'
           :::: ::.:::::::::.:
query   3' CGGAUACUCAGCCACCUGA 5'

query=JS16_t3180966, target=FGSG_04617, score=4, range=500-518, strand=-1

target  5' UCCCAUGAGUCGGUGGAUA 3'
            :: :::::::::::::.
query   3' CGGAUACUCAGCCACCUGA 5'

query=JS16_t3180966, target=FGSG_03614, score=4, range=1214-1231, strand=-1

target  5' GCUUGUGAGUC-GUGGACG 3'
           ::.:.:::::: ::::::
query   3' CGGAUACUCAGCCACCUGA 5'

query=JS16_t3180966, target=FGSG_06676, score=4, range=635-652, strand=-1

target  5' GCUCGU-AGUCGGUGGACU 3'
           ::. .: ::::::::::::
query   3' CGGAUACUCAGCCACCUGA 5'

query=JS16_t3180966, target=FGSG_06777, score=4, range=1968-1985, strand=-1

target  5' GCCU-CGAGUCGGUUGACU 3'
           ::::  :::::::: ::::
query   3' CGGAUACUCAGCCACCUGA 5'

No results for JS16_t0801489
No results for JS16_t2284853
No results for JS16_t3181026
query=JS16_t1388748, target=FGSG_12106, score=2.5, range=1158-1179, strand=-1

target  5' UCCAAACUUGAUGUUGAUGCCA 3'
           :::::: :.:::::::::::::
query   3' AGGUUU-AGCUACAACUACGGG 5'

query=JS16_t1388748, target=FGSG_06557, score=3, range=917-937, strand=1

target  5' UCCACAUCGAUGUUGAUCCCC 3'
           :::: :::::::::::: :::
query   3' AGGUUUAGCUACAACUACGGG 5'

query=JS16_t1388748, target=FGSG_03365, score=3.5, range=482-502, strand=1

target  5' ACCAAGGCGAUGUUGAUGCUC 3'
            ::::. ::::::::::::.:
query   3' AGGUUUAGCUACAACUACGGG 5'

query=JS16_t1388748, target=FGSG_13286, score=3.5, range=277-298, strand=1
```

```
query=JS16_t0254373, target=FGSG_07927, score=3.5, range=1383-1401, strand=1

target  5’ GAUUACGGAGAAAGUGCAC 3’
           ::.: :.::::::.:::::
query   3’ CUGA-GUCUCUUUUACGUG 5’

query=JS16_t0254373, target=FGSG_01490, score=3.5, range=894-910, strand=1

target  5’ GGCUCAGAG-AAAUGCAA 3’
           :.::::::: :::::::
query   3’ CUGAGUCUCUUUUACGUG 5’

query=JS16_t0254373, target=FGSG_11074, score=3.5, range=418-434, strand=1

target  5’ GAUACAGAGAAAAU-CAC 3’
           ::. :::::::::: :::
query   3’ CUGAGUCUCUUUUACGUG 5’

query=JS16_t0254373, target=FGSG_10679, score=3.5, range=986-1002, strand=1

target  5’ GGC-AAGAGAAAAUGUAC 3’
           :.:  ::::::::::.::
query   3’ CUGAGUCUCUUUUACGUG 5’

query=JS16_t0254373, target=FGSG_03251, score=3.5, range=505-522, strand=-1

target  5’ GACUGUGAGAAAAUGCAU 3’
           ::::  :::::::::::.
query   3’ CUGAGUCUCUUUUACGUG 5’

query=JS16_t0254373, target=FGSG_07689, score=3.5, range=1362-1379, strand=-1

target  5’ GACCAAGAGAAGAUGCAU 3’
           :::  ::::::.:::::.
query   3’ CUGAGUCUCUUUUACGUG 5’

query=JS16_t0254373, target=FGSG_12624, score=4, range=1957-1973, strand=1

target  5’ GACUCAG-GGGAAUGCAC 3’
           ::::::: :..:::::::
query   3’ CUGAGUCUCUUUUACGUG 5’

query=JS16_t0254373, target=FGSG_11328, score=4, range=445-463, strand=1

target  5’ GACUCGAGGGAGAAUGCAA 3’
           ::::: ::.::.::::::
query   3’ CUGAG-UCUCUUUUACGUG 5’

query=JS16_t0254373, target=FGSG_09367, score=4, range=266-282, strand=1

target  5’ GAUUUAGAGAAAA-GUAC 3’
           ::.:.:::::::: :.::
query   3’ CUGAGUCUCUUUUACGUG 5’

query=JS16_t0254373, target=FGSG_04987, score=4, range=217-234, strand=1

target  5’ GAUCUAGAGCAAAUGCAC 3’
           ::. .:::: ::::::::
query   3’ CUGAGUCUCUUUUACGUG 5’

query=JS16_t0254373, target=FGSG_01790, score=4, range=3520-3538, strand=1

target  5’ GACAUUAGAGCAAAUGCAU 3’
           ::: :.:::: :::::::.
query   3’ CUG-AGUCUCUUUUACGUG 5’

query=JS16_t0254373, target=FGSG_06071, score=4, range=1800-1818, strand=1

target  5’ GGUUGCAGAGAAAAUGCCC 3’
           :..: :::::::::::: :
query   3’ CUGA-GUCUCUUUUACGUG 5’

query=JS16_t0254373, target=FGSG_07679, score=4, range=358-375, strand=1

target  5’ CAGUCAGAGAAGAUGCAA 3’
            : ::::::::.:::::
query   3’ CUGAGUCUCUUUUACGUG 5’

query=JS16_t0254373, target=FGSG_04745, score=4, range=118-134, strand=1

target  5’ UACU-GGGGAAAAUGCAC 3’
            ::: .:.::::::::::
query   3’ CUGAGUCUCUUUUACGUG 5’

query=JS16_t0254373, target=FGSG_09912, score=4, range=1355-1372, strand=1

target  5’ GAGGCAGAGAAAAUGUAG 3’
           ::  ::::::::::::.:
query   3’ CUGAGUCUCUUUUACGUG 5’

query=JS16_t0254373, target=FGSG_09534, score=4, range=3142-3158, strand=1

target  5’ UCUGAUUCUGAUGUUGAUGCCU 3’
           ::..: :: ::::::::::::.
query   3’ AGGUUUAG-CUACAACUACGGG 5’

query=JS16_t1388748, target=FGSG_05322, score=4, range=1368-1389, strand=-1

target  5’ GCUAGAGUCGAUGUUGAUGUCC 3’
            :.: :.:::::::::::.::
query   3’ AGGU-UUAGCUACAACUACGGG 5’

query=JS16_t1388748, target=FGSG_01741, score=4, range=798-818, strand=-1

target  5’ GCCAACUUGAUGUUGAUGUCU 3’
            :::: :.::::::::::.:.
query   3’ AGGUUUAGCUACAACUACGGG 5’

query=JS16_t1388748, target=FGSG_06231, score=4, range=1671-1691, strand=-1

target  5’ UCGAGAUAGGUGUUGAUGCCU 3’
           :: :.:: :.::::::::::.
query   3’ AGGUUUAGCUACAACUACGGG 5’

query=JS16_t1388748, target=FGSG_11840, score=4, range=339-360, strand=-1

target  5’ UCACAAGCUGAUGUUGAUGCCG 3’
           :: :::. .::::::::::::
query   3’ AG-GUUUAGCUACAACUACGGG 5’

query=JS16_t3181059, target=FGSG_13851, score=3.5, range=258-279, strand=1

target  5’ UGAACAACAGUUUAAUGCACAU 3’
           ::::: ::::::::::: :::.
query   3’ ACUUGAUGUCAAAUUAC-UGUG 5’

query=JS16_t2284929, target=FGSG_09558, score=4, range=1758-1779, strand=1

target  5’ CAGACAACUUCAGGGUCCCGUU 3’
           :: :: :: :::.:::::::::
query   3’ GUAUGCUGUAGUUCCAGGGCAA 5’

No results for JS16_t0474315
query=JS16_t3181133, target=FGSG_10798, score=3, range=199-217, strand=1

target  5’ GGAGAUCCGGUUAUGCAAG 3’
           ..  ::::::::::.:::::
query   3’ UUACUAGGCCAAUGCGUUC 5’

query=JS16_t3181133, target=FGSG_08906, score=3.5, range=300-318, strand=-1

target  5’ GUUGAUUCGGUUGCGCAAG 3’
           .  ::::.:::::.::::::
query   3’ UUACUAGGCCAAUGCGUUC 5’

query=JS16_t3181133, target=FGSG_13790, score=4, range=31-48, strand=1

target  5’ AAU-UUCCGGUUAAGCAAG 3’
           :::  :::::::: :::::
query   3’ UUACUAGGCCAAUGCGUUC 5’

No results for JS16_t4077734
query=JS16_t0228964, target=FGSG_01049, score=3, range=127-145, strand=1

target  5’ UACUCGCGUCAGCAACCGC 3’
           .::::.:::::: ::::::
query   3’ GUGAGUGCAGUCUUUGGCG 5’

query=JS16_t0228964, target=FGSG_07508, score=3, range=159-177, strand=1

target  5’ CACUGCCGUCAGAAAUCGC 3’
           ::::  :::::::::.:::
query   3’ GUGAGUGCAGUCUUUGGCG 5’

query=JS16_t0228964, target=FGSG_02704, score=3, range=1019-1036, strand=1

target  5’ CAC-CCCGUCAGGAACCGC 3’
           ::: : ::::::.::::::
query   3’ GUGAGUGCAGUCUUUGGCG 5’

query=JS16_t0228964, target=FGSG_07267, score=3.5, range=1799-1817, strand=1

target  5’ CGCACACCUCAGAAACCGC 3’
           :.: ::: :::::::::::
query   3’ GUGAGUGCAGUCUUUGGCG 5’

query=JS16_t0228964, target=FGSG_06669, score=3.5, range=544-562, strand=-1

target  5’ UACUCCUGUCAGAAACCGA 3’
           .:::: .:::::::::::
query   3’ GUGAGUGCAGUCUUUGGCG 5’

query=JS16_t0228964, target=FGSG_07165, score=3.5, range=400-419, strand=-1
```

```
target  5’ CACGCAGAGAAAA-GCAC 3’
            :: ::::::::: ::::
query   3’ CUGAGUCUCUUUUACGUG 5’

query=JS16_t0254373, target=FGSG_03650, score=4, range=746-764, strand=1

target  5’ GACUCAGAGGACGAUGCAC 3’
           :::::::::.: .::::::
query   3’ CUGAGUCUCUU-UUACGUG 5’

query=JS16_t0254373, target=FGSG_04045, score=4, range=160-176, strand=1

target  5’ GACGCAGA-AAAAUGCAA 3’
           ::: :::: ::::::::
query   3’ CUGAGUCUCUUUUACGUG 5’

query=JS16_t0254373, target=FGSG_05597, score=4, range=548-565, strand=-1

target  5’ GACUCAGAAAGAAUGCGC 3’
           :::::::: :.:::::.:
query   3’ CUGAGUCUCUUUUACGUG 5’

query=JS16_t0254373, target=FGSG_07946, score=4, range=3612-3629, strand=-1

target  5’ UUCUCGGAGAAAAUGUAC 3’
             :::.:::::::::.::
query   3’ CUGAGUCUCUUUUACGUG 5’

query=JS16_t0254373, target=FGSG_02998, score=4, range=917-934, strand=-1

target  5’ GACGCAGAGGAAAUGCGA 3’
           ::: :::::.::::::.
query   3’ CUGAGUCUCUUUUACGUG 5’

query=JS16_t0254373, target=FGSG_11915, score=4, range=753-770, strand=-1

target  5’ GUCUCAGAGAAGAUGUAA 3’
           : :::::::::.:::.:
query   3’ CUGAGUCUCUUUUACGUG 5’

query=JS16_t0254373, target=FGSG_05263, score=4, range=732-748, strand=-1

target  5’ GAUUU-GAGAAAGUGCAC 3’
           ::.:. ::::::.:::::
query   3’ CUGAGUCUCUUUUACGUG 5’

query=JS16_t0254373, target=FGSG_09515, score=4, range=3034-3051, strand=-1

target  5’ GACCCAGAGAAGUUGCAC 3’
           ::: :::::::. :::::
query   3’ CUGAGUCUCUUUUACGUG 5’

query=JS16_t0254373, target=FGSG_01336, score=4, range=965-981, strand=-1

target  5’ GAC-CAGGGAAAAUUCAC 3’
           ::: :::.:::::: :::
query   3’ CUGAGUCUCUUUUACGUG 5’

query=JS16_t0254373, target=FGSG_12929, score=4, range=75-91, strand=-1

target  5’ GA-UGAGGGAAAAUGUAC 3’
           :: : ::.:::::::.::
query   3’ CUGAGUCUCUUUUACGUG 5’

query=JS16_t0254373, target=FGSG_08611, score=4, range=1105-1121, strand=-1

target  5’ GACAAAGAGAAAAUG-AC 3’
           :::  :::::::::: ::
query   3’ CUGAGUCUCUUUUACGUG 5’

No results for JS16_t4015255
No results for JS16_t4015296
No results for JS16_t0268578
query=JS16_t0335172, target=FGSG_11070, score=4, range=324-347, strand=-1

target  5’ ACGAUAAUCGGAUCAAAGUCUUUA 3’
           ::.:.  :: ::::::::::::::
query   3’ UGUUGAAAG-CUAGUUUCAGAAAU 5’

No results for JS16_t2222587
No results for JS16_t3118620
query=JS16_t4015417, target=FGSG_13610, score=3.5, range=190-210, strand=1

target  5’ UGGCUUUCAUUCGUUGUUCAU 3’
           : ::.:::::: :::::::::
query   3’ AGCGGAAGUAAACAACAAGUA 5’

query=JS16_t4015417, target=FGSG_06437, score=4, range=925-945, strand=1

target  5’ ACACCUUCAUUUGUUGUUGAU 3’
            : ::::::::::::::: ::
query   3’ AGCGGAAGUAAACAACAAGUA 5’
```

```
target  5’ CGCUCACCGUCAGAAACAGC 3’
           :.:::: :::::::::: ::
query   3’ GUGAGU-GCAGUCUUUGGCG 5’

query=JS16_t0228964, target=FGSG_06016, score=4, range=5519-5537, strand=1

target  5’ CAUUCGCGCCAGAAAUCGC 3’
           ::.::.:: ::::::.:::
query   3’ GUGAGUGCAGUCUUUGGCG 5’

query=JS16_t0228964, target=FGSG_00585, score=4, range=275-293, strand=1

target  5’ UCUUCACGUCAGAGACUGC 3’
           . .::::::::::.::.::
query   3’ GUGAGUGCAGUCUUUGGCG 5’

query=JS16_t0228964, target=FGSG_05041, score=4, range=453-471, strand=1

target  5’ CAAGUACGUCAGGAACCGU 3’
           ::  .:::::::.:::::.
query   3’ GUGAGUGCAGUCUUUGGCG 5’

query=JS16_t0228964, target=FGSG_03136, score=4, range=1295-1312, strand=1

target  5’ CACU-ACGUCAAAAACUGC 3’
           :::: :::::: ::::.::
query   3’ GUGAGUGCAGUCUUUGGCG 5’

query=JS16_t0228964, target=FGSG_03283, score=4, range=183-200, strand=1

target  5’ CA-UCAUGGCAGAAACCGC 3’
           :: :::.: :::::::::
query   3’ GUGAGUGCAGUCUUUGGCG 5’

query=JS16_t0228964, target=FGSG_05435, score=4, range=798-815, strand=1

target  5’ CAC-CACGUCAGAAACAGA 3’
           ::: ::::::::::: :
query   3’ GUGAGUGCAGUCUUUGGCG 5’

query=JS16_t0228964, target=FGSG_11991, score=4, range=371-388, strand=1

target  5’ CAC-CCCGUCGGAAACCGG 3’
           ::: : ::::.:::::::
query   3’ GUGAGUGCAGUCUUUGGCG 5’

query=JS16_t0228964, target=FGSG_02774, score=4, range=1380-1397, strand=1

target  5’ CACUCGCG-CAGAGACCGU 3’
           :::::.:: ::::.::::.
query   3’ GUGAGUGCAGUCUUUGGCG 5’

query=JS16_t0228964, target=FGSG_07645, score=4, range=256-274, strand=-1

target  5’ CACUCACGUCAGAGAUCUC 3’
           :::::::::::::.:.: :
query   3’ GUGAGUGCAGUCUUUGGCG 5’

query=JS16_t0228964, target=FGSG_10978, score=4, range=1106-1123, strand=-1

target  5’ CGCUUGCGUCAG-AACCGU 3’
           :.::..:::::: :::::.
query   3’ GUGAGUGCAGUCUUUGGCG 5’

query=JS16_t0228964, target=FGSG_01544, score=4, range=1297-1316, strand=-1

target  5’ UGAUCAGCGUCAGAAACCGG 3’
           ..  ::: ::::::::::::
query   3’ GUGAGU-GCAGUCUUUGGCG 5’

No results for JS16_t3181169
No results for JS16_t1388911
query=JS16_t2285030, target=FGSG_10530, score=4, range=5979-5997, strand=-1

target  5’ UG-ACUCUGUGAGCAAGUUC 3’
           :.  ::.::::.:::::::.:
query   3’ AUGUGGGACAUUCGUUCAGG 5’

No results for JS16_t0691909
query=JS16_t3181229, target=FGSG_06225, score=3, range=89-107, strand=1

target  5’ AAAGCCAGGUUCUCCCGUC 3’
           :::: ::::::: ::::::
query   3’ UUUC-GUCCAAGUGGGCAG 5’

query=JS16_t3181229, target=FGSG_01886, score=3, range=994-1010, strand=-1

target  5’ ACAGCAGGUUCACCC-UC 3’
           : ::::::::::::: ::
query   3’ UUUCGUCCAAGUGGGCAG 5’

query=JS16_t3181229, target=FGSG_06823, score=3.5, range=2286-2303, strand=-1
```

```
query=JS16_t4015417, target=FGSG_12742, score=4, range=1978-1998, strand=-1

target  5’ UCUUUUCCGUUUGUUGUUCAU 3’
           :: ..: :.:::::::::::
query   3’ AGCGGAAGUAAACAACAAGUA 5’

No results for JS16_t0240187
query=JS16_t0240190, target=FGSG_04261, score=4, range=1027-1045, strand=1

target  5’ UGGAUUGCUGCAGCCGA-GA 3’
            :::::::::::.:::: ::
query   3’ CCCUAACGACGUUGGCUACU 5’

query=JS16_t0240190, target=FGSG_01993, score=4, range=746-764, strand=1

target  5’ AGGA-UGCUGCAGCUGAUGA 3’
            ::: :::::::.:.:::::
query   3’ CCCUAACGACGUUGGCUACU 5’

query=JS16_t0240190, target=FGSG_13215, score=4, range=379-397, strand=1

target  5’ AGGAUUGC-GCAACCGAUGU 3’
            ::::::: ::::::::::
query   3’ CCCUAACGACGUUGGCUACU 5’

query=JS16_t0240190, target=FGSG_00669, score=4, range=3043-3062, strand=-1

target  5’ AUGAUUGCUGUAACUGAUGA 3’
             ::::::::.:::.:::::
query   3’ CCCUAACGACGUUGGCUACU 5’

No results for JS16_t1326351
No results for JS16_t3118834
No results for JS16_t1326398
No results for JS16_t3118888
query=JS16_t0254379, target=FGSG_12621, score=3, range=289-305, strand=1

target  5’ GUACCAGG-CUCGAAGUU 3’
           :: ::::: :::::::::
query   3’ CAAGGUCCAGAGCUUCAA 5’

query=JS16_t0254379, target=FGSG_11890, score=3, range=486-503, strand=-1

target  5’ GUUCCAGGCUUCGAAGUU 3’
           :::::::: .::::::::
query   3’ CAAGGUCCAGAGCUUCAA 5’

query=JS16_t0254379, target=FGSG_08209, score=3, range=4790-4807, strand=-1

target  5’ GUGCCAGGUCUUGAAGUC 3’
           :: ::::::::.:::::
query   3’ CAAGGUCCAGAGCUUCAA 5’

query=JS16_t0254379, target=FGSG_04896, score=3, range=449-466, strand=-1

target  5’ GCUCCAGGUCUCGAAGGU 3’
           : :::::::::::::: :
query   3’ CAAGGUCCAGAGCUUCAA 5’

query=JS16_t0254379, target=FGSG_09992, score=3, range=305-322, strand=-1

target  5’ GUACCAGGUCUCGAACUU 3’
           :: :::::::::::: ::
query   3’ CAAGGUCCAGAGCUUCAA 5’

query=JS16_t0254379, target=FGSG_03273, score=3.5, range=802-819, strand=1

target  5’ GAUUCAGGUCUCGAAGGU 3’
           : :.:::::::::::: :
query   3’ CAAGGUCCAGAGCUUCAA 5’

query=JS16_t0254379, target=FGSG_06830, score=3.5, range=192-209, strand=-1

target  5’ GUUCUAGGUUGCGAAGUU 3’
           ::::.::::. :::::::
query   3’ CAAGGUCCAGAGCUUCAA 5’

query=JS16_t0254379, target=FGSG_10464, score=4, range=5394-5411, strand=1

target  5’ GUUCCAGGUCUUGGAUUU 3’
           :::::::::::.:.: ::
query   3’ CAAGGUCCAGAGCUUCAA 5’

query=JS16_t0254379, target=FGSG_03214, score=4, range=390-407, strand=1

target  5’ CUUCCAGGUCUUCAAGUU 3’
            ::::::::::. :::::
query   3’ CAAGGUCCAGAGCUUCAA 5’

query=JS16_t0254379, target=FGSG_01035, score=4, range=2335-2352, strand=1

target  5’ GAAGCAGGUUCAUCCUUC 3’
           .::::::::::::.:: ::
query   3’ UUUCGUCCAAGUGGGCAG 5’

query=JS16_t3181229, target=FGSG_06148, score=4, range=102-120, strand=1

target  5’ AAAGCAGGUUCCGCUCGUC 3’
           :::::::::: :.:.::::
query   3’ UUUCGUCCAA-GUGGGCAG 5’

query=JS16_t3181229, target=FGSG_03597, score=4, range=594-611, strand=1

target  5’ AAAGAAGGGUCACCUGUC 3’
           :::: ::: :::::.:::
query   3’ UUUCGUCCAAGUGGGCAG 5’

query=JS16_t3181229, target=FGSG_04627, score=4, range=2638-2656, strand=-1

target  5’ AAAGCAGGUUCGCUCUGUC 3’
           :::::::::::.: :.:::
query   3’ UUUCGUCCAAGUG-GGCAG 5’

query=JS16_t3181229, target=FGSG_07379, score=4, range=1518-1534, strand=-1

target  5’ GAAG-AGGUUCGCUCGUU 3’
           .::: ::::::.:.:::.
query   3’ UUUCGUCCAAGUGGGCAG 5’

query=JS16_t3181229, target=FGSG_08505, score=4, range=201-218, strand=-1

target  5’ ACAGCAGGUUCUCCCGUG 3’
           : ::::::::: :::::
query   3’ UUUCGUCCAAGUGGGCAG 5’

query=JS16_t3181229, target=FGSG_13445, score=4, range=888-904, strand=-1

target  5’ CAAG-AGGUUCACCCUUC 3’
            ::: :::::::::: ::
query   3’ UUUCGUCCAAGUGGGCAG 5’

query=JS16_t3181229, target=FGSG_03246, score=4, range=1771-1789, strand=-1

target  5’ CAAGAAGGUUCAACCCGUC 3’
            ::: :::::: :::::::
query   3’ UUUCGUCCAAG-UGGGCAG 5’

No results for JS16_t0220280
No results for JS16_t2285067
query=JS16_t0583320, target=FGSG_04307, score=3.5, range=1382-1401, strand=-1

target  5’ GAGGUCGUACCGGAAGCCAA 3’
           :::.:  ::::::::.::::
query   3’ CUCUA-AAUGGCCUUUGGUU 5’

query=JS16_t0583320, target=FGSG_06193, score=4, range=83-102, strand=1

target  5’ GAGAUGUAUCCGGAAACCGA 3’
           ::::: :: :::::::::.:
query   3’ CUCUAAAU-GGCCUUUGGUU 5’

query=JS16_t0583320, target=FGSG_05076, score=4, range=186-204, strand=1

target  5’ GCAAUGUAUCGGAAACCAA 3’
           :  :: ::.::::::::::
query   3’ CUCUAAAUGGCCUUUGGUU 5’

query=JS16_t0583320, target=FGSG_05352, score=4, range=452-469, strand=-1

target  5’ GAGAGUUGCC-GAAACCAA 3’
           :::: ::.:: ::::::::
query   3’ CUCUAAAUGGCCUUUGGUU 5’

No results for JS16_t0691922
No results for JS16_t4077950
No results for JS16_t3181336
No results for JS16_t0113646
No results for JS16_t4078042
No results for JS16_t0801546
No results for JS16_t3181439
No results for JS16_t2285292
No results for JS16_t4078200
query=JS16_t2285410, target=FGSG_05576, score=2, range=197-216, strand=1

target  5’ CCGAG-UCUCGUCAAGUGCAC 3’
            :::: :::::::::::::::
query   3’ UGCUCAAGAGCAGUUCACGUG 5’

query=JS16_t2285410, target=FGSG_03605, score=4, range=625-644, strand=-1

target  5’ AC-AGUUGUCGUCAGGUGCAG 3’
           :: :::: ::::::.:::::
```

```
target  5' AUGCCAGGUCUCGGGGUU 3'
             : ::::::::::..:::
query   3' CAAGGUCCAGAGCUUCAA 5'

query=JS16_t0254379, target=FGSG_02315, score=4, range=13957-13974, strand=1

target  5' GUCCCAGGUCUCAAAGUC 3'
           :: ::::::::: ::::
query   3' CAAGGUCCAGAGCUUCAA 5'

query=JS16_t0254379, target=FGSG_10096, score=4, range=1270-1287, strand=1

target  5' AUUACAGGUGUCGAAGUU 3'
            :: ::::: ::::::::
query   3' CAAGGUCCAGAGCUUCAA 5'

query=JS16_t0254379, target=FGSG_02303, score=4, range=799-816, strand=1

target  5' CUGCCAGGUGUCGAAGUU 3'
            : :::::: ::::::::
query   3' CAAGGUCCAGAGCUUCAA 5'

query=JS16_t0254379, target=FGSG_03061, score=4, range=340-357, strand=1

target  5' GUUGAAGGUUUCGAAGUC 3'
           :::  ::::.:::::::
query   3' CAAGGUCCAGAGCUUCAA 5'

query=JS16_t0254379, target=FGSG_01254, score=4, range=873-890, strand=1

target  5' GAACAAGGUCUCGGAGUU 3'
           :  : ::::::::.::::
query   3' CAAGGUCCAGAGCUUCAA 5'

query=JS16_t0254379, target=FGSG_07877, score=4, range=2551-2568, strand=1

target  5' GACGAAGGUCUCGAAGUU 3'
           :    :::::::::::::
query   3' CAAGGUCCAGAGCUUCAA 5'

query=JS16_t0254379, target=FGSG_02615, score=4, range=194-210, strand=1

target  5' GUGGCA-GUCUCGAAGUU 3'
           ::  :: :::::::::::
query   3' CAAGGUCCAGAGCUUCAA 5'

query=JS16_t0254379, target=FGSG_04890, score=4, range=1946-1962, strand=1

target  5' GUCCCAGG-CUCGAGGUU 3'
           :: ::::: :::::.:::
query   3' CAAGGUCCAGAGCUUCAA 5'

query=JS16_t0254379, target=FGSG_02586, score=4, range=210-227, strand=-1

target  5' GUUCCAGGUCUUGAGUUU 3'
           :::::::::::.::. ::
query   3' CAAGGUCCAGAGCUUCAA 5'

query=JS16_t0254379, target=FGSG_03480, score=4, range=238-255, strand=-1

target  5' ACUCCAGGUCUUGAAGUC 3'
             :::::::::.:::::
query   3' CAAGGUCCAGAGCUUCAA 5'

query=JS16_t0254379, target=FGSG_09080, score=4, range=1223-1240, strand=-1

target  5' CAACCAGGUUUCGAAGUU 3'
              ::::::.::::::::
query   3' CAAGGUCCAGAGCUUCAA 5'

query=JS16_t0254379, target=FGSG_13878, score=4, range=28236-28253, strand=-1

target  5' GUUCCAGGUAACGAAGUU 3'
           :::::::::  :::::::
query   3' CAAGGUCCAGAGCUUCAA 5'

query=JS16_t0254379, target=FGSG_07798, score=4, range=1156-1173, strand=-1

target  5' GUUCAAGGUCUCGAAGAG 3'
           :::: :::::::::::
query   3' CAAGGUCCAGAGCUUCAA 5'

query=JS16_t0254379, target=FGSG_08185, score=4, range=619-636, strand=-1

target  5' GCGCCAGGUCUCGAGGUC 3'
           :  :::::::::::.::
query   3' CAAGGUCCAGAGCUUCAA 5'

query=JS16_t0254379, target=FGSG_09046, score=4, range=71-88, strand=-1

target  5' AUUCAAGGUCUCCAAGUU 3'
            ::: ::::::: :::::
query   3' UGCUCAAGAGCAGUUCACGUG 5'

No results for JS16_t2285417
query=JS16_t0283990, target=FGSG_13130, score=1.5, range=712-729, strand=-1

target  5' UCUUGCGCCAUAGCUAUG 3'
           ::::::::::::::.::.
query   3' AGAACGCGGUAUCGGUAU 5'

query=JS16_t0283990, target=FGSG_13556, score=2, range=1596-1612, strand=-1

target  5' UAUU-CGCCAUAGCCAUA 3'
           : :: :::::::::::::
query   3' AGAACGCGGUAUCGGUAU 5'

query=JS16_t0283990, target=FGSG_04621, score=3, range=757-774, strand=1

target  5' UCUUUCGCCAAAGCCAUA 3'
           :::: ::::: :::::::
query   3' AGAACGCGGUAUCGGUAU 5'

query=JS16_t0283990, target=FGSG_04188, score=3, range=1604-1620, strand=1

target  5' UCUU-CGCCAUGGCCAUC 3'
           :::: ::::::.:::::
query   3' AGAACGCGGUAUCGGUAU 5'

query=JS16_t0283990, target=FGSG_07454, score=3, range=1390-1407, strand=-1

target  5' ACUUCCGCCAUGGCCAUA 3'
            ::: ::::::.::::::
query   3' AGAACGCGGUAUCGGUAU 5'

query=JS16_t0283990, target=FGSG_07562, score=3, range=571-587, strand=-1

target  5' UCUU-CGCCAUGGCCGUA 3'
           :::: ::::::.:::.::
query   3' AGAACGCGGUAUCGGUAU 5'

query=JS16_t0283990, target=FGSG_10030, score=3, range=651-668, strand=-1

target  5' UCGAGCGCCAUGGCCAUA 3'
           ::  :::::::.::::::
query   3' AGAACGCGGUAUCGGUAU 5'

query=JS16_t0283990, target=FGSG_00369, score=3, range=997-1013, strand=-1

target  5' UCCU-CGCCAUAGCCGUA 3'
           :: : ::::::::::.::
query   3' AGAACGCGGUAUCGGUAU 5'

query=JS16_t0283990, target=FGSG_07059, score=3.5, range=37-54, strand=-1

target  5' UCCUCCGCCAUGGCCAUG 3'
           :: : ::::::.:::::.
query   3' AGAACGCGGUAUCGGUAU 5'

query=JS16_t0283990, target=FGSG_12921, score=3.5, range=727-743, strand=-1

target  5' UCUGGCGCCAUAG-CAUG 3'
           ::: ::::::::: :::.
query   3' AGAACGCGGUAUCGGUAU 5'

query=JS16_t0283990, target=FGSG_05950, score=4, range=702-719, strand=1

target  5' UUUUGCGCCAUGGCGAUG 3'
           :.:::::::::.:: ::.
query   3' AGAACGCGGUAUCGGUAU 5'

query=JS16_t0283990, target=FGSG_04167, score=4, range=766-783, strand=1

target  5' CCUCGUGCCAUGGCCAUA 3'
            :: :.:::::.::::::
query   3' AGAACGCGGUAUCGGUAU 5'

query=JS16_t0283990, target=FGSG_06570, score=4, range=116-132, strand=1

target  5' UCUU-UGCCAUGGCUAUA 3'
           :::: .:::::.::.:::
query   3' AGAACGCGGUAUCGGUAU 5'

query=JS16_t0283990, target=FGSG_03998, score=4, range=372-390, strand=1

target  5' UCUGUUCGUCAUGGCCAUA 3'
           ::: : ::.:::.::::::
query   3' AGA-ACGCGGUAUCGGUAU 5'

query=JS16_t0283990, target=FGSG_10847, score=4, range=314-330, strand=1

target  5' UCUU-CGUUAUAGCCAUC 3'
           :::: ::..:::::::::
query   3' AGAACGCGGUAUCGGUAU 5'
```

```
query   3' CAAGGUCCAGAGCUUCAA 5'

query=JS16_t0254379, target=FGSG_00935, score=4, range=418-435, strand=-1

target  5' GUCCCAGGUCACGAAGUA 3'
           :: ::::::: ::::::
query   3' CAAGGUCCAGAGCUUCAA 5'

query=JS16_t0254379, target=FGSG_13389, score=4, range=112-129, strand=-1

target  5' CUUGAAGGUUUCGAAGUU 3'
            ::  ::::.::::::::
query   3' CAAGGUCCAGAGCUUCAA 5'

query=JS16_t0254379, target=FGSG_13258, score=4, range=724-741, strand=-1

target  5' GGUCGAGCUCUCGAAGUU 3'
           : :: :: ::::::::::
query   3' CAAGGUCCAGAGCUUCAA 5'

query=JS16_t3118911, target=FGSG_08133, score=4, range=3386-3407, strand=1

target  5' UCAAGGCUGCCUGGGAUACAAA 3'
           .:::: : .::.::::::::::
query   3' GGUUCGGUUGGGCCCUAUGUUU 5'

No results for JS16_t4015742
No results for JS16_t1326557
query=JS16_t2222997, target=FGSG_08097, score=4, range=4706-4727, strand=1

target  5' CCCUUGAAUCGCCACGCGACUA 3'
            : ::::: :.::::::::::
query   3' AGUAACUU-GUGGUGCGCUGAU 5'

query=JS16_t3119037, target=FGSG_05539, score=3.5, range=466-484, strand=1

target  5' GAUGCC-GAAUUAGAACUCC 3'
            ::.:: ::::.::::::::
query   3' AUAUGGACUUAGUCUUGAGG 5'

query=JS16_t0364679, target=FGSG_00031, score=4, range=3241-3261, strand=1

target  5' UUCACUGGCAACCAAGACUAC 3'
           :::::. .::::::: :::::
query   3' AAGUGGAUGUUGGUUGUGAUG 5'

query=JS16_t0364679, target=FGSG_09513, score=4, range=2232-2252, strand=1

target  5' UCCACCUGCAAGCAACACUAU 3'
           : :::::.::: ::::::::.
query   3' AAGUGGAUGUUGGUUGUGAUG 5'

No results for JS16_t3119078
query=JS16_t0182369, target=FGSG_10588, score=4, range=74-92, strand=1

target  5' UCCAUGUUCUACUCAUAGC 3'
           .::: ...::::::::::
query   3' GGGUUUGGGAUGAGUAUCG 5'

query=JS16_t0182369, target=FGSG_07368, score=4, range=975-993, strand=1

target  5' AUCAAGGCCUACUCAUAGC 3'
            .:::. :::::::::::
query   3' GGGUUUGGGAUGAGUAUCG 5'

query=JS16_t0182369, target=FGSG_06596, score=4, range=219-237, strand=1

target  5' CCCUAACCCAACUUAUAGC 3'
           ::: ::::: :::.:::::
query   3' GGGUUUGGGAUGAGUAUCG 5'

query=JS16_t0182369, target=FGSG_07155, score=4, range=180-197, strand=1

target  5' CGCAACCCCUACUCA-AGC 3'
           : ::: ::::::::: :::
query   3' GGGUUUGGGAUGAGUAUCG 5'

query=JS16_t0182369, target=FGSG_00667, score=4, range=922-941, strand=-1

target  5' CCCAUACCACUGCUCAUAGC 3'
           :::: ::: ::.::::::::
query   3' GGGUUUGG-GAUGAGUAUCG 5'

query=JS16_t0182369, target=FGSG_06717, score=4, range=395-413, strand=-1

target  5' UCAGAACCCUUCUCAUAGC 3'
           .: .:::::: ::::::::
query   3' GGGUUUGGGAUGAGUAUCG 5'

No results for JS16_t4015947
No results for JS16_t3119187
query=JS16_t0111672, target=FGSG_07350, score=3.5, range=1134-1155, strand=1

query=JS16_t0283990, target=FGSG_04381, score=4, range=754-771, strand=1

target  5' UAUACCGCUAUAGCCAUA 3'
           : :  :::.::::::::::
query   3' AGAACGCGGUAUCGGUAU 5'

query=JS16_t0283990, target=FGSG_02732, score=4, range=1760-1776, strand=1

target  5' UC-UACGCCAUAGCCAAA 3'
           :: : ::::::::::: :
query   3' AGAACGCGGUAUCGGUAU 5'

query=JS16_t0283990, target=FGSG_07193, score=4, range=268-284, strand=1

target  5' UCUUG-GCCAUGGCCAUC 3'
           ::::: :::::.:::::
query   3' AGAACGCGGUAUCGGUAU 5'

query=JS16_t0283990, target=FGSG_04511, score=4, range=875-891, strand=1

target  5' UCUU-CGCCACAGCCAUU 3'
           :::: ::::: ::::::
query   3' AGAACGCGGUAUCGGUAU 5'

query=JS16_t0283990, target=FGSG_09686, score=4, range=1068-1085, strand=-1

target  5' UCUUGCGCCAUGGUCAAA 3'
           ::::::::::.:.:: :
query   3' AGAACGCGGUAUCGGUAU 5'

query=JS16_t0283990, target=FGSG_00782, score=4, range=604-621, strand=-1

target  5' CCUCGCGUCGUAGCCAUA 3'
            :: :::.:.::::::::
query   3' AGAACGCGGUAUCGGUAU 5'

query=JS16_t0283990, target=FGSG_01029, score=4, range=1861-1878, strand=-1

target  5' UCCUGCGCCAGAGCCGUA 3'
           :: ::::::: ::::.::
query   3' AGAACGCGGUAUCGGUAU 5'

query=JS16_t0283990, target=FGSG_08873, score=4, range=993-1010, strand=-1

target  5' UCGUGCUUCAUAGCCAUA 3'
           :: ::: .::::::::::
query   3' AGAACGCGGUAUCGGUAU 5'

query=JS16_t0283990, target=FGSG_02617, score=4, range=955-971, strand=-1

target  5' GCUUGCGCCAU-GUCAUA 3'
            :::::::::: :.::::
query   3' AGAACGCGGUAUCGGUAU 5'

query=JS16_t0283990, target=FGSG_04113, score=4, range=205-222, strand=-1

target  5' ACUUCCGCCAUAGCCAGA 3'
            ::: ::::::::::: :
query   3' AGAACGCGGUAUCGGUAU 5'

query=JS16_t0283990, target=FGSG_08662, score=4, range=1257-1274, strand=-1

target  5' GCCUGGGCCAUAGCCAUA 3'
            : :: ::::::::::::
query   3' AGAACGCGGUAUCGGUAU 5'

query=JS16_t0283990, target=FGSG_04768, score=4, range=808-826, strand=-1

target  5' UCAUGCUGCCAUAGCCAUC 3'
           :: ::: :::::::::::
query   3' AGAACG-CGGUAUCGGUAU 5'

query=JS16_t0283990, target=FGSG_05106, score=4, range=3324-3340, strand=-1

target  5' UC-UGCGCCAUGUCCAUA 3'
           :: ::::::::. :::::
query   3' AGAACGCGGUAUCGGUAU 5'

query=JS16_t0283990, target=FGSG_08237, score=4, range=1660-1676, strand=-1

target  5' UCUU-CGCCAUAGUCAAA 3'
           :::: ::::::::.:: :
query   3' AGAACGCGGUAUCGGUAU 5'

query=JS16_t0283990, target=FGSG_06316, score=4, range=2134-2150, strand=-1

target  5' UCUU-CGCCAUCGUCAUA 3'
           :::: :::::: :.::::
query   3' AGAACGCGGUAUCGGUAU 5'

query=JS16_t0283990, target=FGSG_03643, score=4, range=529-545, strand=-1
```

```
target  5’ CGGCGGACGUGUUGUUUUGGCG 3’
           :.: :::: .::::::::::::
query   3’ GUCACCUGGGCAACAAAACCGC 5’

query=JS16_t2223195, target=FGSG_03915, score=3, range=502-522, strand=1

target  5’ UUCUACACACGCAUCAAUGAU 3’
            :::  :::::::::::::::
query   3’ CAGAAUUGUGCGUAGUUACUA 5’

query=JS16_t2223195, target=FGSG_08928, score=3, range=328-347, strand=-1

target  5’ GUCGUAA-AUGCAUCAAUGAU 3’
           ::: ::: :.:::::::::::
query   3’ CAGAAUUGUGCGUAGUUACUA 5’

query=JS16_t2223195, target=FGSG_04175, score=4, range=2041-2061, strand=-1

target  5’ CUCUUGAUACGUAUCGAUGAU 3’
            ::::.:.:::.:::.:::::
query   3’ CAGAAUUGUGCGUAGUUACUA 5’

query=JS16_t2223195, target=FGSG_02910, score=4, range=382-402, strand=-1

target  5’ GUUCGGAAACGCAUCAAUGAU 3’
           ::.  .: :::::::::::::
query   3’ CAGAAUUGUGCGUAGUUACUA 5’

No results for JS16_t0466857
No results for JS16_t0149816
query=JS16_t0364688, target=FGSG_10575, score=2.5, range=680-697, strand=1

target  5’ CUAUCGACGAUCAAGAAG 3’
            .: ::::::::::::::
query   3’ CGUCGCUGCUAGUUCUUC 5’

query=JS16_t0364688, target=FGSG_02808, score=3, range=698-715, strand=1

target  5’ AUGGCGAUGAUCAAGAAG 3’
            ..::::.::::::::::
query   3’ CGUCGCUGCUAGUUCUUC 5’

query=JS16_t0364688, target=FGSG_01025, score=3, range=26-43, strand=1

target  5’ GCAGCGACGAUGAAGAGG 3’
           ::::::::::: ::::.:
query   3’ CGUCGCUGCUAGUUCUUC 5’

query=JS16_t0364688, target=FGSG_04486, score=3, range=167-184, strand=1

target  5’ GCAUCGAUGAUCAGGAAG 3’
           ::: :::.:::::.::::
query   3’ CGUCGCUGCUAGUUCUUC 5’

query=JS16_t0364688, target=FGSG_03805, score=3, range=2166-2184, strand=1

target  5’ GCAGACGACGAUUGAGAAG 3’
           :::: :::::::..:::::
query   3’ CGUC-GCUGCUAGUUCUUC 5’

query=JS16_t0364688, target=FGSG_01606, score=3, range=1102-1118, strand=1

target  5’ GUGG-GACGAUCGAGAAG 3’
           :..: :::::::.:::::
query   3’ CGUCGCUGCUAGUUCUUC 5’

query=JS16_t0364688, target=FGSG_07444, score=3, range=589-606, strand=1

target  5’ GCAGAGACGAUGAAGAAG 3’
           :::: :::::: ::::::
query   3’ CGUCGCUGCUAGUUCUUC 5’

query=JS16_t0364688, target=FGSG_07417, score=3, range=265-282, strand=1

target  5’ GAAACGACGAUCAAGGAG 3’
           : : :::::::::::.::
query   3’ CGUCGCUGCUAGUUCUUC 5’

query=JS16_t0364688, target=FGSG_00673, score=3, range=558-574, strand=1

target  5’ GCAG-GAGGAUCAAGAAG 3’
           :::: :: ::::::::::
query   3’ CGUCGCUGCUAGUUCUUC 5’

query=JS16_t0364688, target=FGSG_01693, score=3, range=3969-3986, strand=-1

target  5’ GCAGCGACGAUCUGGAAG 3’
           :::::::::::: .::::
query   3’ CGUCGCUGCUAGUUCUUC 5’

query=JS16_t0364688, target=FGSG_03412, score=3, range=654-671, strand=-1
```

```
target  5’ UCUUUCGCUAUAG-CAUA 3’
           :::: :::.:::: ::::
query   3’ AGAACGCGGUAUCGGUAU 5’

query=JS16_t0283990, target=FGSG_09193, score=4, range=658-675, strand=-1

target  5’ CGUUUCGCCAUAGCCAUU 3’
             :: ::::::::::::
query   3’ AGAACGCGGUAUCGGUAU 5’

query=JS16_t0283990, target=FGSG_01318, score=4, range=2410-2426, strand=-1

target  5’ UC-UCCGCCAGAGCCAUA 3’
           :: : ::::: :::::::
query   3’ AGAACGCGGUAUCGGUAU 5’

query=JS16_t0283990, target=FGSG_04504, score=4, range=904-920, strand=-1

target  5’ ACAUGCGCCAU-GCCAUA 3’
            : :::::::: ::::::
query   3’ AGAACGCGGUAUCGGUAU 5’

query=JS16_t0283990, target=FGSG_05645, score=4, range=154-170, strand=-1

target  5’ UCUU-CGCCAGAGCCAUU 3’
           :::: ::::: ::::::
query   3’ AGAACGCGGUAUCGGUAU 5’

query=JS16_t0283990, target=FGSG_04788, score=4, range=262-278, strand=-1

target  5’ UCUUCCGCCAUA-UCAUA 3’
           :::: ::::::: .::::
query   3’ AGAACGCGGUAUCGGUAU 5’

query=JS16_t0283990, target=FGSG_09386, score=4, range=174-190, strand=-1

target  5’ UCUUGC-UCAUAGCCGUA 3’
           :::::: .:::::::.::
query   3’ AGAACGCGGUAUCGGUAU 5’

No results for JS16_t1389325
query=JS16_t3181626, target=FGSG_04803, score=3.5, range=1067-1085, strand=1

target  5’ AGCUGCAGAGCAAUCGGUG 3’
            : :: ::::::::::::.
query   3’ CCCAC-UCUCGUUAGCCAU 5’

query=JS16_t3181626, target=FGSG_00633, score=3.5, range=5768-5784, strand=-1

target  5’ ACGU-AGAGCAAUCGGUG 3’
             :: ::::::::::::.
query   3’ CCCACUCUCGUUAGCCAU 5’

query=JS16_t3181626, target=FGSG_05656, score=3.5, range=518-534, strand=-1

target  5’ GGCUGA-AGCAAUCGGUG 3’
           :: ::: ::::::::::.
query   3’ CCCACUCUCGUUAGCCAU 5’

query=JS16_t3181626, target=FGSG_07793, score=3.5, range=42-60, strand=-1

target  5’ GGGAGAGAGCCAAUCGGUG 3’
           ::: ::::: ::::::::.
query   3’ CCCACUCUC-GUUAGCCAU 5’

query=JS16_t3181626, target=FGSG_11219, score=4, range=12-29, strand=1

target  5’ CGUUGAGAGUGAUCGGUA 3’
            : ::::::..:::::::
query   3’ CCCACUCUCGUUAGCCAU 5’

query=JS16_t3181626, target=FGSG_07944, score=4, range=855-871, strand=1

target  5’ GGGUGAUAGCAAU-GGUA 3’
           :::::: :::::: ::::
query   3’ CCCACUCUCGUUAGCCAU 5’

query=JS16_t3181626, target=FGSG_07557, score=4, range=1066-1083, strand=1

target  5’ GGAGGAGAGCAAUUGGUU 3’
           ::  :::::::::.:::
query   3’ CCCACUCUCGUUAGCCAU 5’

query=JS16_t3181626, target=FGSG_01106, score=4, range=720-736, strand=1

target  5’ GAGUGAGAGUAAU-GGUA 3’
           : :::::::.::: ::::
query   3’ CCCACUCUCGUUAGCCAU 5’

query=JS16_t3181626, target=FGSG_05780, score=4, range=1338-1354, strand=1
```

```
target  5’ ACAGCGACGAUCAUGAAG 3’
            :::::::::::: ::::
query   3’ CGUCGCUGCUAGUUCUUC 5’

query=JS16_t0364688, target=FGSG_03639, score=3, range=510-527, strand=-1

target  5’ GCAUCGAUGAUCAAGAAA 3’
           ::: :::.:::::::::
query   3’ CGUCGCUGCUAGUUCUUC 5’

query=JS16_t0364688, target=FGSG_09135, score=3, range=1804-1820, strand=-1

target  5’ GCA-CGACGAUCGAGGAG 3’
           ::: ::::::::.::.::
query   3’ CGUCGCUGCUAGUUCUUC 5’

query=JS16_t0364688, target=FGSG_09373, score=3, range=217-233, strand=-1

target  5’ GGAGCG-CGAUCAAGAAG 3’
           : :::: :::::::::::
query   3’ CGUCGCUGCUAGUUCUUC 5’

query=JS16_t0364688, target=FGSG_13994, score=3, range=247-263, strand=-1

target  5’ GCCGCGACGAU-AAGAAG 3’
           :: :::::::: ::::::
query   3’ CGUCGCUGCUAGUUCUUC 5’

query=JS16_t0364688, target=FGSG_03736, score=3, range=49-65, strand=-1

target  5’ GCA-AGACGAUCAAGAAU 3’
           :::  ::::::::::::
query   3’ CGUCGCUGCUAGUUCUUC 5’

query=JS16_t0364688, target=FGSG_09461, score=3.5, range=1513-1530, strand=1

target  5’ GCGGCGGCGCUCAAGAAG 3’
           ::.:::.:: ::::::::
query   3’ CGUCGCUGCUAGUUCUUC 5’

query=JS16_t0364688, target=FGSG_01925, score=3.5, range=251-268, strand=1

target  5’ GUACCGACGGUCAAGAAA 3’
           :.: :::::.:::::::
query   3’ CGUCGCUGCUAGUUCUUC 5’

query=JS16_t0364688, target=FGSG_05026, score=3.5, range=696-713, strand=1

target  5’ GCGACGACGAUCACGAAG 3’
           ::. ::::::::: ::::
query   3’ CGUCGCUGCUAGUUCUUC 5’

query=JS16_t0364688, target=FGSG_03442, score=3.5, range=748-765, strand=1

target  5’ GAGGAGGCGAUCAAGAAG 3’
           : .: :.:::::::::::
query   3’ CGUCGCUGCUAGUUCUUC 5’

query=JS16_t0364688, target=FGSG_05542, score=3.5, range=286-303, strand=1

target  5’ AUCGAGACGAUCAAGAAG 3’
            . : :::::::::::::
query   3’ CGUCGCUGCUAGUUCUUC 5’

query=JS16_t0364688, target=FGSG_07719, score=3.5, range=2969-2985, strand=1

target  5’ GUA-CGACUAUCAAGAAG 3’
           :.: :::: :::::::::
query   3’ CGUCGCUGCUAGUUCUUC 5’

query=JS16_t0364688, target=FGSG_05482, score=3.5, range=897-913, strand=1

target  5’ GUA-CGACGAUCCAGAAG 3’
           :.: :::::::: :::::
query   3’ CGUCGCUGCUAGUUCUUC 5’

query=JS16_t0364688, target=FGSG_10881, score=3.5, range=8-24, strand=1

target  5’ GCGGCGAU-AUCAAGAAG 3’
           ::.::::. :::::::::
query   3’ CGUCGCUGCUAGUUCUUC 5’

query=JS16_t0364688, target=FGSG_03599, score=3.5, range=15-32, strand=-1

target  5’ GCGGCGACGAUGAAGGAG 3’
           ::.:::::::: :::.::
query   3’ CGUCGCUGCUAGUUCUUC 5’

query=JS16_t0364688, target=FGSG_09805, score=3.5, range=270-286, strand=-1

target  5’ GUA-CGGCGAUCGAGAAG 3’

target  5’ GGGU-CGAGCGAUCGGUA 3’
           ::::  ::::.::::::
query   3’ CCCACUCUCGUUAGCCAU 5’

query=JS16_t3181626, target=FGSG_09135, score=4, range=3723-3739, strand=1

target  5’ GAGU-AGAGCAGUUGGUA 3’
           : :: :::::.:.::::
query   3’ CCCACUCUCGUUAGCCAU 5’

query=JS16_t3181626, target=FGSG_02874, score=4, range=1925-1943, strand=1

target  5’ CGGUGAGAGUAUAUCGGUA 3’
            ::::::::.: :::::::
query   3’ CCCACUCUCGU-UAGCCAU 5’

query=JS16_t3181626, target=FGSG_09121, score=4, range=232-248, strand=1

target  5’ UGGUGGGAG-AAUCGGUA 3’
            ::::.::: ::::::::
query   3’ CCCACUCUCGUUAGCCAU 5’

query=JS16_t3181626, target=FGSG_04824, score=4, range=1236-1253, strand=-1

target  5’ CGCUCAGGGCAAUCGGUA 3’
            : : ::.::::::::::
query   3’ CCCACUCUCGUUAGCCAU 5’

query=JS16_t3181626, target=FGSG_10978, score=4, range=17-34, strand=-1

target  5’ GUCUAAGAGCAAUUGGUA 3’
           :  : ::::::::.::::
query   3’ CCCACUCUCGUUAGCCAU 5’

query=JS16_t3181626, target=FGSG_04181, score=4, range=1047-1063, strand=-1

target  5’ GGGAGAGAGCAG-CGGUA 3’
           ::: ::::::::. :::::
query   3’ CCCACUCUCGUUAGCCAU 5’

No results for JS16_t4078304
No results for JS16_t3181675
No results for JS16_t2285514
No results for JS16_t0691970
No results for JS16_t4078393
No results for JS16_t3181794
No results for JS16_t3181847
query=JS16_t0241157, target=FGSG_00333, score=3, range=674-694, strand=1

target  5’ CGAGCGAAACUUUUGCAUUCG 3’
           :.::: ::::::::::::.:.
query   3’ GUUCG-UUUGAAAACGUAGGU 5’

query=JS16_t0241157, target=FGSG_11856, score=3.5, range=1441-1459, strand=-1

target  5’ CAA-CAGACUUUUGUGUCCA 3’
           ::: ::.:::::::..::::
query   3’ GUUCGUUUGAAAACGUAGGU 5’

query=JS16_t0241157, target=FGSG_03176, score=3.5, range=326-344, strand=-1

target  5’ UAA-CGGACUUUUGCAUCCC 3’
           .:: :..:::::::::::
query   3’ GUUCGUUUGAAAACGUAGGU 5’

query=JS16_t0241157, target=FGSG_11026, score=4, range=193-212, strand=1

target  5’ GGAGAGAGCUUUUGCAUCCA 3’
            .:: .:.::::::::::::
query   3’ GUUCGUUUGAAAACGUAGGU 5’

query=JS16_t0241157, target=FGSG_09250, score=4, range=1360-1379, strand=-1

target  5’ UGAGCGAACUUUUGCAUACG 3’
           ..:::.::::::::::: :.
query   3’ GUUCGUUUGAAAACGUAGGU 5’

query=JS16_t0241157, target=FGSG_10333, score=4, range=1057-1076, strand=-1

target  5’ UAAUUUAGCUUUUGCAUCCA 3’
           .:: . :.::::::::::::
query   3’ GUUCGUUUGAAAACGUAGGU 5’

No results for JS16_t0801613
query=JS16_t3182013, target=FGSG_02035, score=4, range=2835-2855, strand=1

target  5’ AACACCUUUGAUGCUCGCUAU 3’
           : :::  :::::::::::::
query   3’ UGGUGCUAACUACGAGCGAUC 5’

query=JS16_t3182013, target=FGSG_09558, score=4, range=418-437, strand=-1
```

```
           :.: ::.:::::.:::::
query   3’ CGUCGCUGCUAGUUCUUC 5’

query=JS16_t0364688, target=FGSG_05968, score=3.5, range=303-320, strand=-1

target  5’ GCCGUGACGAUGAAGAAG 3’
           :: :.:::::: ::::::
query   3’ CGUCGCUGCUAGUUCUUC 5’

query=JS16_t0364688, target=FGSG_03932, score=3.5, range=360-376, strand=-1

target  5’ CCA-UGACGAUCAGGAAG 3’
            :: .::::::::.::::
query   3’ CGUCGCUGCUAGUUCUUC 5’

query=JS16_t0364688, target=FGSG_10850, score=4, range=146-163, strand=1

target  5’ GUGGCGAUGAUGAAGAAG 3’
           :..::::.::: ::::::
query   3’ CGUCGCUGCUAGUUCUUC 5’

query=JS16_t0364688, target=FGSG_00797, score=4, range=763-780, strand=1

target  5’ GCCGCGAUGAUCAAGGAU 3’
           :: ::::.:::::::.:
query   3’ CGUCGCUGCUAGUUCUUC 5’

query=JS16_t0364688, target=FGSG_02177, score=4, range=244-261, strand=1

target  5’ GGAGCGAUGAUCGAGAAU 3’
           : :::::.::::.::::
query   3’ CGUCGCUGCUAGUUCUUC 5’

query=JS16_t0364688, target=FGSG_07214, score=4, range=1037-1054, strand=1

target  5’ GCAGCGACGAUGAAGAUG 3’
           ::::::::::: :::: :
query   3’ CGUCGCUGCUAGUUCUUC 5’

query=JS16_t0364688, target=FGSG_10262, score=4, range=869-885, strand=1

target  5’ GCAG-GAUGAUCGAGAGG 3’
           :::: ::.::::.:::.:
query   3’ CGUCGCUGCUAGUUCUUC 5’

query=JS16_t0364688, target=FGSG_06504, score=4, range=1658-1675, strand=1

target  5’ GGAGCGACGAGCAAGGAG 3’
           : :::::::: ::::.::
query   3’ CGUCGCUGCUAGUUCUUC 5’

query=JS16_t0364688, target=FGSG_11210, score=4, range=504-521, strand=1

target  5’ GCAGAGACGAUCCAGGAG 3’
           :::: ::::::: ::.::
query   3’ CGUCGCUGCUAGUUCUUC 5’

query=JS16_t0364688, target=FGSG_06123, score=4, range=1602-1620, strand=1

target  5’ GCAAGCGACGAUGAGGAAG 3’
           :: ::::::::: :.::::
query   3’ CG-UCGCUGCUAGUUCUUC 5’

query=JS16_t0364688, target=FGSG_07457, score=4, range=742-759, strand=1

target  5’ GCUGAGAUGAUCAAGGAG 3’
           :: : ::.:::::::.::
query   3’ CGUCGCUGCUAGUUCUUC 5’

query=JS16_t0364688, target=FGSG_13419, score=4, range=388-405, strand=1

target  5’ GCAGGGAAGAUCAAGAAA 3’
           :::: :: :::::::::
query   3’ CGUCGCUGCUAGUUCUUC 5’

query=JS16_t0364688, target=FGSG_03382, score=4, range=401-418, strand=1

target  5’ UCAGAGACGAUGAAGAAG 3’
            ::: :::::: ::::::
query   3’ CGUCGCUGCUAGUUCUUC 5’

query=JS16_t0364688, target=FGSG_09394, score=4, range=3244-3261, strand=1

target  5’ CCUGAGAUGAUCAAGAAG 3’
            : : ::.::::::::::
query   3’ CGUCGCUGCUAGUUCUUC 5’

query=JS16_t0364688, target=FGSG_10736, score=4, range=841-858, strand=1

target  5’ GCCGAGAUGAUCAAGAAC 3’
           :: : ::.:::::::::
query   3’ CGUCGCUGCUAGUUCUUC 5’
```

```
target  5’ GUCGUG-UUGAUGCUCGCUAU 3’
           ..:..: :::::::::::::
query   3’ UGGUGCUAACUACGAGCGAUC 5’

No results for JS16_t2285911
query=JS16_t1389826, target=FGSG_10122, score=2, range=1268-1286, strand=1

target  5’ GCACAUGGCAAUGCGAGAA 3’
           ::::::::::: :::::::
query   3’ CGUGUACCGUUUCGCUCUU 5’

query=JS16_t1389826, target=FGSG_04907, score=2.5, range=962-979, strand=-1

target  5’ GCGC-CGGCAAAGCGAGAA 3’
           ::.:  :::::::::::::
query   3’ CGUGUACCGUUUCGCUCUU 5’

query=JS16_t1389826, target=FGSG_02251, score=3, range=2198-2216, strand=-1

target  5’ UCACAAGGCAAAGCGAGGA 3’
            :::: :::::::::::.:
query   3’ CGUGUACCGUUUCGCUCUU 5’

query=JS16_t1389826, target=FGSG_06990, score=3, range=254-272, strand=-1

target  5’ GCAAAGGGUAAAGCGAGAA 3’
           ::: : ::.::::::::::
query   3’ CGUGUACCGUUUCGCUCUU 5’

query=JS16_t1389826, target=FGSG_00078, score=3.5, range=692-710, strand=1

target  5’ CCAAGUGGCAAGGCGAGAA 3’
            :: .::::::.:::::::
query   3’ CGUGUACCGUUUCGCUCUU 5’

query=JS16_t1389826, target=FGSG_05243, score=3.5, range=507-525, strand=-1

target  5’ GUACAUGGCAAAGCUAGAC 3’
           :.:::::::::::: :::
query   3’ CGUGUACCGUUUCGCUCUU 5’

query=JS16_t1389826, target=FGSG_07356, score=3.5, range=395-412, strand=-1

target  5’ UCGCA-GGCAAAGUGAGAA 3’
            :.:: :::::::.:::::
query   3’ CGUGUACCGUUUCGCUCUU 5’

query=JS16_t1389826, target=FGSG_10750, score=3.5, range=812-829, strand=-1

target  5’ GGAC-UGGCGAAGCGAGAG 3’
           : :: ::::.:::::::::.
query   3’ CGUGUACCGUUUCGCUCUU 5’

query=JS16_t1389826, target=FGSG_09367, score=3.5, range=1199-1216, strand=-1

target  5’ GCUUA-GGCAAAGCGAGGA 3’
           :: .: :::::::::::.:
query   3’ CGUGUACCGUUUCGCUCUU 5’

query=JS16_t1389826, target=FGSG_10094, score=3.5, range=2000-2019, strand=-1

target  5’ GAACGAGGGCAAAGCGAGAG 3’
           : :: : ::::::::::::.
query   3’ CGUG-UACCGUUUCGCUCUU 5’

query=JS16_t1389826, target=FGSG_06351, score=4, range=627-645, strand=1

target  5’ CAAAAUGGCGAAGCGAGAA 3’
             : :::::.::::::::::
query   3’ CGUGUACCGUUUCGCUCUU 5’

query=JS16_t1389826, target=FGSG_07968, score=4, range=959-977, strand=1

target  5’ GCGGACGGCAGAGCGAGAG 3’
           ::.  : ::::.::::::::.
query   3’ CGUGUACCGUUUCGCUCUU 5’

query=JS16_t1389826, target=FGSG_00459, score=4, range=687-706, strand=1

target  5’ UCAAAUGGCAAAGCGACGAA 3’
            :: :::::::::::: :::
query   3’ CGUGUACCGUUUCGCU-CUU 5’

query=JS16_t1389826, target=FGSG_05168, score=4, range=1032-1049, strand=1

target  5’ GGAC-UGGCAGAGCGAGAU 3’
           : :: :::::.:::::::
query   3’ CGUGUACCGUUUCGCUCUU 5’

query=JS16_t1389826, target=FGSG_00805, score=4, range=455-472, strand=1

target  5’ GC-CUUGGCAACGCGAGAA 3’
```

```
query=JS16_t0364688, target=FGSG_09985, score=4, range=68-85, strand=1

target  5’ ACAAAGAUGAUCAAGAAG 3’
            ::  ::.::::::::::
query   3’ CGUCGCUGCUAGUUCUUC 5’

query=JS16_t0364688, target=FGSG_11913, score=4, range=126-142, strand=1

target  5’ GC-GCGACGGUCAAGAUG 3’
           :: ::::::.:::::: :
query   3’ CGUCGCUGCUAGUUCUUC 5’

query=JS16_t0364688, target=FGSG_06106, score=4, range=488-504, strand=1

target  5’ GCAGAGACGA-CAAGGAG 3’
           :::: ::::: ::::.::
query   3’ CGUCGCUGCUAGUUCUUC 5’

query=JS16_t0364688, target=FGSG_10842, score=4, range=406-423, strand=1

target  5’ GAUGCGACAAUCAAGAAG 3’
           :  ::::: :::::::::
query   3’ CGUCGCUGCUAGUUCUUC 5’

query=JS16_t0364688, target=FGSG_11569, score=4, range=1674-1690, strand=1

target  5’ GGAG-GAUGAUCAGGAAG 3’
           : :: ::.:::::.::::
query   3’ CGUCGCUGCUAGUUCUUC 5’

query=JS16_t0364688, target=FGSG_02595, score=4, range=343-361, strand=1

target  5’ GAAGCAGACGAUCAUGAAG 3’
           : ::: :::::::: ::::
query   3’ CGUCG-CUGCUAGUUCUUC 5’

query=JS16_t0364688, target=FGSG_00074, score=4, range=1060-1077, strand=1

target  5’ GCCAAGGCGAUCAAGAAG 3’
           ::    :.::::::::::
query   3’ CGUCGCUGCUAGUUCUUC 5’

query=JS16_t0364688, target=FGSG_07157, score=4, range=180-196, strand=1

target  5’ CCAG-GACGACCAAGAAG 3’
            ::: ::::: :::::::
query   3’ CGUCGCUGCUAGUUCUUC 5’

query=JS16_t0364688, target=FGSG_10627, score=4, range=2320-2336, strand=1

target  5’ GAAG-GACGAUCAAGACG 3’
           : :: ::::::::::: :
query   3’ CGUCGCUGCUAGUUCUUC 5’

query=JS16_t0364688, target=FGSG_02216, score=4, range=2087-2103, strand=1

target  5’ GCA-AGACUAUCAAGAAG 3’
           :::  ::: :::::::::
query   3’ CGUCGCUGCUAGUUCUUC 5’

query=JS16_t0364688, target=FGSG_04376, score=4, range=732-748, strand=1

target  5’ GAAG-GACGAUGAAGAAG 3’
           : :: :::::: ::::::
query   3’ CGUCGCUGCUAGUUCUUC 5’

query=JS16_t0364688, target=FGSG_01386, score=4, range=1657-1673, strand=1

target  5’ GAAGCG-CGAUCAAGAAA 3’
           : :::: ::::::::::
query   3’ CGUCGCUGCUAGUUCUUC 5’

query=JS16_t0364688, target=FGSG_09909, score=4, range=400-416, strand=1

target  5’ GCAGCGACGA-CAGGAAC 3’
           :::::::::: ::.:::
query   3’ CGUCGCUGCUAGUUCUUC 5’

query=JS16_t0364688, target=FGSG_06553, score=4, range=848-864, strand=1

target  5’ UCAGCGA-GAUCAAGAAA 3’
            :::::: :::::::::
query   3’ CGUCGCUGCUAGUUCUUC 5’

query=JS16_t0364688, target=FGSG_05443, score=4, range=267-285, strand=1

target  5’ GAAGCGACGGAUCGAGAAG 3’
           : :::::: ::::.:::::
query   3’ CGUCGCUG-CUAGUUCUUC 5’

query=JS16_t0364688, target=FGSG_06095, score=4, range=658-676, strand=1
```

```
           :: : :::::: :::::::
query   3’ CGUGUACCGUUUCGCUCUU 5’

query=JS16_t1389826, target=FGSG_06661, score=4, range=706-725, strand=-1

target  5’ GCGACAUGGUGAAGCGAGAU 3’
           :: ::::::..::::::::
query   3’ CG-UGUACCGUUUCGCUCUU 5’

query=JS16_t1389826, target=FGSG_13282, score=4, range=764-783, strand=-1

target  5’ GCGGUAUGGCAAAUCGAGAA 3’
           :: ..:::::::: ::::::
query   3’ CG-UGUACCGUUUCGCUCUU 5’

query=JS16_t1389826, target=FGSG_03466, score=4, range=720-738, strand=-1

target  5’ GUUGGUGGCGAAGCGAGAA 3’
           :.  .::::.:::::::::
query   3’ CGUGUACCGUUUCGCUCUU 5’

query=JS16_t1389826, target=FGSG_05422, score=4, range=3000-3018, strand=-1

target  5’ GGACAUGGCAAAACGAGAC 3’
           : :::::::::: :::::
query   3’ CGUGUACCGUUUCGCUCUU 5’

query=JS16_t1389826, target=FGSG_04313, score=4, range=1302-1319, strand=-1

target  5’ GC-CAUGGCAAGGCGAUAA 3’
           :: ::::::::.:::: ::
query   3’ CGUGUACCGUUUCGCUCUU 5’

query=JS16_t1389826, target=FGSG_10844, score=4, range=1020-1037, strand=-1

target  5’ GGAC-UGGCAGAGCGAGAC 3’
           : :: :::::.:::::::
query   3’ CGUGUACCGUUUCGCUCUU 5’

query=JS16_t1389826, target=FGSG_11092, score=4, range=1523-1542, strand=-1

target  5’ GCCCAUGGCAAGAGCGAGAU 3’
           :: :::::::: :::::::
query   3’ CGUGUACCGUU-UCGCUCUU 5’

No results for JS16_t4078789
query=JS16_t3182120, target=FGSG_13751, score=3, range=290-307, strand=-1

target  5’ CAGGU-GUACAGUUCCAUG 3’
           : ::: .::::::::::::
query   3’ GACCAGUAUGUCAAGGUAC 5’

query=JS16_t3182120, target=FGSG_04604, score=3, range=145-162, strand=-1

target  5’ GCGGU-AUACAGUUCCAUG 3’
             ::: :::::::::::::
query   3’ GACCAGUAUGUCAAGGUAC 5’

query=JS16_t3182120, target=FGSG_04681, score=3.5, range=637-655, strand=-1

target  5’ CUGGUUGUACAGUACCAUG 3’
           :::::..:::::: :::::
query   3’ GACCAGUAUGUCAAGGUAC 5’

query=JS16_t3182120, target=FGSG_00946, score=3.5, range=1032-1050, strand=-1

target  5’ CUGGUUAUACAGUUCAGUG 3’
           :::::.::::::::: .::
query   3’ GACCAGUAUGUCAAGGUAC 5’

query=JS16_t3182120, target=FGSG_11875, score=3.5, range=254-272, strand=-1

target  5’ UUGGUCAUACAGUGCCAUU 3’
           .::::::::::::: ::::
query   3’ GACCAGUAUGUCAAGGUAC 5’

query=JS16_t3182120, target=FGSG_11381, score=4, range=129-147, strand=1

target  5’ CUGGCCAUACAUUUCCAUU 3’
           :::: :::::: ::::::
query   3’ GACCAGUAUGUCAAGGUAC 5’

query=JS16_t3182120, target=FGSG_13337, score=4, range=272-290, strand=1

target  5’ CUGUAGAUACAGUUCCAUC 3’
           :::    ::::::::::::
query   3’ GACCAGUAUGUCAAGGUAC 5’

query=JS16_t3182120, target=FGSG_05973, score=4, range=1202-1220, strand=-1

target  5’ CUGGAGAUAUAGUUCCAUC 3’
           ::::  :::.::::::::
```

```
target  5’ GCAGCAACAGAUCAAGGAG 3’
           ::::: :: :::::::.::
query   3’ CGUCGCUG-CUAGUUCUUC 5’

query=JS16_t0364688, target=FGSG_09289, score=4, range=788-804, strand=1

target  5’ GCAUCGAUG-UCAAGAAG 3’
           ::: :::.: ::::::::
query   3’ CGUCGCUGCUAGUUCUUC 5’

query=JS16_t0364688, target=FGSG_10299, score=4, range=482-498, strand=1

target  5’ UCGGUGAC-AUCAAGAAG 3’
            :.:.::: :::::::::
query   3’ CGUCGCUGCUAGUUCUUC 5’

query=JS16_t0364688, target=FGSG_06662, score=4, range=242-259, strand=-1

target  5’ GCAGCGGCGGUCCAGAAG 3’
           ::::::.::.:: :::::
query   3’ CGUCGCUGCUAGUUCUUC 5’

query=JS16_t0364688, target=FGSG_11812, score=4, range=1165-1182, strand=-1

target  5’ GCGGUUACGAUCGAGAAG 3’
           ::.:. ::::::.:::::
query   3’ CGUCGCUGCUAGUUCUUC 5’

query=JS16_t0364688, target=FGSG_00743, score=4, range=285-302, strand=-1

target  5’ GGAGCGACGGUCAAGGGG 3’
           : :::::::.:::::..:
query   3’ CGUCGCUGCUAGUUCUUC 5’

query=JS16_t0364688, target=FGSG_00887, score=4, range=1663-1681, strand=-1

target  5’ GCGGUGACGAUGCAAGAGG 3’
           ::.:.:::::: :::::.:
query   3’ CGUCGCUGCUA-GUUCUUC 5’

query=JS16_t0364688, target=FGSG_09128, score=4, range=16-33, strand=-1

target  5’ GAGGUGACGAUCGGGAAG 3’
           : .:.::::::::..::::
query   3’ CGUCGCUGCUAGUUCUUC 5’

query=JS16_t0364688, target=FGSG_08403, score=4, range=371-388, strand=-1

target  5’ CCAGCGACGGUCCAGAAG 3’
            ::::::::.:: :::::
query   3’ CGUCGCUGCUAGUUCUUC 5’

query=JS16_t0364688, target=FGSG_03744, score=4, range=718-734, strand=-1

target  5’ GCAGCGACGA-CAGGGAG 3’
           :::::::::: ::.:.::
query   3’ CGUCGCUGCUAGUUCUUC 5’

query=JS16_t0364688, target=FGSG_13299, score=4, range=192-209, strand=-1

target  5’ GCAGCGACGAACAAGACG 3’
           :::::::::: ::::: :
query   3’ CGUCGCUGCUAGUUCUUC 5’

query=JS16_t0364688, target=FGSG_02229, score=4, range=837-854, strand=-1

target  5’ GCAGCGCCGAUAAAGAAG 3’
           :::::: :::: ::::::
query   3’ CGUCGCUGCUAGUUCUUC 5’

query=JS16_t0364688, target=FGSG_04188, score=4, range=336-353, strand=-1

target  5’ GCACCGACGGUCAUGAAG 3’
           ::: :::::.::: ::::
query   3’ CGUCGCUGCUAGUUCUUC 5’

query=JS16_t0364688, target=FGSG_09539, score=4, range=65-82, strand=-1

target  5’ GCAGAGAAGAUCGAGAAG 3’
           :::: :: ::::.:::::
query   3’ CGUCGCUGCUAGUUCUUC 5’

query=JS16_t0364688, target=FGSG_10921, score=4, range=255-272, strand=-1

target  5’ GCACCGGCGAUCCAGAAG 3’
           ::: ::.::::: :::::
query   3’ CGUCGCUGCUAGUUCUUC 5’

query=JS16_t0364688, target=FGSG_00469, score=4, range=1173-1190, strand=-1

target  5’ GCAUCGGCGCUCAAGAAG 3’
query   3’ GACCAGUAUGUCAAGGUAC 5’

query=JS16_t2286028, target=FGSG_10291, score=2, range=187-209, strand=-1

target  5’ GCUUGCUGCCGCAAUUGUUCCUU 3’
           ::::::::::::::.::.:::::
query   3’ CGAACGACGGCGUUGACGAGGAA 5’

query=JS16_t2286028, target=FGSG_12548, score=4, range=37-58, strand=1

target  5’ GUUUG-UGCUGCAGCUGCUCCUG 3’
           :.::: :::.:::.::::::::
query   3’ CGAACGACGGCGUUGACGAGGAA 5’

query=JS16_t2286028, target=FGSG_02478, score=4, range=1078-1099, strand=-1

target  5’ GCUUGC-GCCGCAACUUCUCCUG 3’
           :::::: ::::::::: :::::
query   3’ CGAACGACGGCGUUGACGAGGAA 5’

query=JS16_t4078884, target=FGSG_01054, score=3.5, range=4-23, strand=-1

target  5’ ACUGUGUAUGCUUGCAAUGC 3’
           :::. ::::::::::: :::
query   3’ UGAUUCAUACGAACGUGACG 5’

query=JS16_t4078884, target=FGSG_03263, score=4, range=552-571, strand=-1

target  5’ AGUAAAUGUGCUUGCACUGG 3’
           : ::: :.:::::::::::
query   3’ UGAUUCAUACGAACGUGACG 5’

query=JS16_t4078884, target=FGSG_13085, score=4, range=50-68, strand=-1

target  5’ AUCAAG-AUGCUUGCGCUGU 3’
           :. ::: ::::::::.:::.
query   3’ UGAUUCAUACGAACGUGACG 5’

query=JS16_t4078884, target=FGSG_11953, score=4, range=211-229, strand=-1

target  5’ CCGAAGUA-GCUUGCACUGC 3’
            : ::::: :::::::::::
query   3’ UGAUUCAUACGAACGUGACG 5’

No results for JS16_t2286058
No results for JS16_t0307482
No results for JS16_t0801643
No results for JS16_t0366802
No results for JS16_t0801651
query=JS16_t1390124, target=FGSG_08596, score=3, range=1396-1415, strand=-1

target  5’ UUUGCGCGAAGAAGGGUGAG 3’
           .::::.:::::: :::::::
query   3’ GAACGUGCUUCUACCCACUC 5’

query=JS16_t1390124, target=FGSG_05514, score=4, range=1179-1197, strand=1

target  5’ CUUG-AAGAUGAUGGGUGAG 3’
           :::: : :: ::::::::::
query   3’ GAACGUGCUUCUACCCACUC 5’

query=JS16_t1390124, target=FGSG_09329, score=4, range=3631-3649, strand=-1

target  5’ CUUGGGUGAAGAU-GGUGAG 3’
           :::: ..:::::: ::::::
query   3’ GAACGUGCUUCUACCCACUC 5’

query=JS16_t1390124, target=FGSG_05765, score=4, range=339-357, strand=-1

target  5’ CUUGC-CGAAGAUGGGCGAC 3’
           ::::: :::::::::: ::
query   3’ GAACGUGCUUCUACCCACUC 5’

No results for JS16_t2286228
No results for JS16_t0307489
No results for JS16_t1390160
No results for JS16_t2286336
No results for JS16_t0801677
No results for JS16_t1390297
query=JS16_t3182569, target=FGSG_11505, score=3.5, range=1620-1637, strand=1

target  5’ UCUCGCAGCUUGGAAUCU 3’
           .::::::: :.:::::::
query   3’ GGAGCGUCUAGCCUUAGA 5’

query=JS16_t3182569, target=FGSG_02482, score=3.5, range=626-643, strand=1

target  5’ CUUCGCAGACUGGAAUCU 3’
           :.:::::::: .:::::::
query   3’ GGAGCGUCUAGCCUUAGA 5’

query=JS16_t3182569, target=FGSG_09325, score=3.5, range=783-799, strand=-1
```

```
           ::: ::.:: ::::::::
query   3’ CGUCGCUGCUAGUUCUUC 5’

query=JS16_t0364688, target=FGSG_09004, score=4, range=1072-1089, strand=-1

target  5’ GAAGCGACGGGCAAGAAG 3’
           : :::::::. :::::::
query   3’ CGUCGCUGCUAGUUCUUC 5’

query=JS16_t0364688, target=FGSG_12767, score=4, range=420-437, strand=-1

target  5’ GCACCGAUGAGCAAGAAG 3’
           ::: :::.:: :::::::
query   3’ CGUCGCUGCUAGUUCUUC 5’

query=JS16_t0364688, target=FGSG_09380, score=4, range=907-924, strand=-1

target  5’ GCUGAGACGGUCGAGAAG 3’
           :: : ::::.::.:::::
query   3’ CGUCGCUGCUAGUUCUUC 5’

query=JS16_t0364688, target=FGSG_13588, score=4, range=544-560, strand=-1

target  5’ GCA-CGACGAUCGGGAAU 3’
           ::: ::::::::..:::
query   3’ CGUCGCUGCUAGUUCUUC 5’

query=JS16_t0364688, target=FGSG_06272, score=4, range=2802-2819, strand=-1

target  5’ ACAACGCCGAUCAAGAAG 3’
            :: :: :::::::::::
query   3’ CGUCGCUGCUAGUUCUUC 5’

query=JS16_t0364688, target=FGSG_01375, score=4, range=355-373, strand=-1

target  5’ GCAGACGACGACCAAGAAU 3’
           :::: :::::: ::::::
query   3’ CGUC-GCUGCUAGUUCUUC 5’

query=JS16_t0364688, target=FGSG_05633, score=4, range=187-204, strand=-1

target  5’ GAAGAGGCGAUCAAGAAA 3’
           : :: :.::::::::::
query   3’ CGUCGCUGCUAGUUCUUC 5’

query=JS16_t0364688, target=FGSG_03681, score=4, range=640-657, strand=-1

target  5’ GCCUCGACGGUCAAGAAU 3’
           ::  :::::.:::::::
query   3’ CGUCGCUGCUAGUUCUUC 5’

query=JS16_t0364688, target=FGSG_11561, score=4, range=1096-1113, strand=-1

target  5’ ACUGAGACGAUUAAGAAG 3’
            : : ::::::.::::::
query   3’ CGUCGCUGCUAGUUCUUC 5’

query=JS16_t0364688, target=FGSG_13932, score=4, range=216-233, strand=-1

target  5’ GGAUCGAUGAUCAAGAAA 3’
           : : :::.:::::::::
query   3’ CGUCGCUGCUAGUUCUUC 5’

query=JS16_t0364688, target=FGSG_05335, score=4, range=319-335, strand=-1

target  5’ GCA-CGAUGAUCCAGAAG 3’
           ::: :::.:::: :::::
query   3’ CGUCGCUGCUAGUUCUUC 5’

query=JS16_t0364688, target=FGSG_03066, score=4, range=1534-1550, strand=-1

target  5’ AAAGCG-CGAUCAAGAAG 3’
             :::: ::::::::::
query   3’ CGUCGCUGCUAGUUCUUC 5’

query=JS16_t0364688, target=FGSG_05394, score=4, range=148-165, strand=-1

target  5’ UCUCCGACGAUCAAGAAC 3’
            :  ::::::::::::
query   3’ CGUCGCUGCUAGUUCUUC 5’

query=JS16_t0364688, target=FGSG_05532, score=4, range=274-291, strand=-1

target  5’ GAAGAGACGAUCAAGUAG 3’
           : :: ::::::::: ::
query   3’ CGUCGCUGCUAGUUCUUC 5’

query=JS16_t0364688, target=FGSG_05992, score=4, range=348-365, strand=-1

target  5’ GCUACGAGGAUCAAGAAG 3’
           ::  ::: ::::::::::
query   3’ CGUCGCUGCUAGUUCUUC 5’

target  5’ UCUCG-AGAUCGGGAUCU 3’
           .:::: :::::::.::::
query   3’ GGAGCGUCUAGCCUUAGA 5’

query=JS16_t3182569, target=FGSG_06168, score=4, range=1319-1336, strand=1

target  5’ AGACGCAGAUCGGAAUCA 3’
              :::::::::::::
query   3’ GGAGCGUCUAGCCUUAGA 5’

query=JS16_t3182569, target=FGSG_11624, score=4, range=218-234, strand=1

target  5’ CCUCG-AGAACGGAAUCU 3’
           ::::: ::: ::::::::
query   3’ GGAGCGUCUAGCCUUAGA 5’

query=JS16_t3182569, target=FGSG_04055, score=4, range=366-382, strand=1

target  5’ CUUUGC-GAUCGGAAUCG 3’
           :.:.:: :::::::::
query   3’ GGAGCGUCUAGCCUUAGA 5’

query=JS16_t3182569, target=FGSG_00418, score=4, range=1182-1200, strand=-1

target  5’ CCUGGGUAGAUCGGAGUCU 3’
           :::  :.::::::::.:::
query   3’ GGA-GCGUCUAGCCUUAGA 5’

query=JS16_t3182569, target=FGSG_11142, score=4, range=1079-1095, strand=-1

target  5’ AUUUGCAGAUC-GAAUCU 3’
            .:.::::::: ::::::
query   3’ GGAGCGUCUAGCCUUAGA 5’

query=JS16_t3182569, target=FGSG_10353, score=4, range=465-482, strand=-1

target  5’ CCACGCAGAUCGGAAACA 3’
           :: ::::::::::: :
query   3’ GGAGCGUCUAGCCUUAGA 5’

query=JS16_t3182569, target=FGSG_10835, score=4, range=4633-4650, strand=-1

target  5’ GCUGCCAGAUCGGAGUCU 3’
            ::  :::::::::.:::
query   3’ GGAGCGUCUAGCCUUAGA 5’

query=JS16_t3182569, target=FGSG_09982, score=4, range=463-480, strand=-1

target  5’ CGAGGCAGAUCGGAAUCC 3’
           :   :::::::::::::
query   3’ GGAGCGUCUAGCCUUAGA 5’

query=JS16_t3182569, target=FGSG_01049, score=4, range=21323-21341, strand=-1

target  5’ GCUCGAAGAUCGGCAAUCU 3’
            :::: ::::::: :::::
query   3’ GGAGCGUCUAGCC-UUAGA 5’

query=JS16_t3182569, target=FGSG_04998, score=4, range=984-1000, strand=-1

target  5’ CCUCG-AGGUCGGAAUCC 3’
           ::::: ::.::::::::
query   3’ GGAGCGUCUAGCCUUAGA 5’

No results for JS16_t2286423
query=JS16_t0307502, target=FGSG_02188, score=2.5, range=639-658, strand=-1

target  5’ ACGUGUGACGAGCACGUUUG 3’
           ::: .: ::::::::::::
query   3’ UGCUUAGUGCUCGUGCAAAC 5’

query=JS16_t0307502, target=FGSG_06279, score=3.5, range=1838-1858, strand=1

target  5’ GCGAAUCACAGAGCAUGUUUG 3’
           .:::::::: :::::.:::::
query   3’ UGCUUAGUG-CUCGUGCAAAC 5’

query=JS16_t0307502, target=FGSG_08060, score=4, range=848-867, strand=-1

target  5’ ACAAAUCACGGGCGUGUUUG 3’
           :: :::::::.::..:::::
query   3’ UGCUUAGUGCUCGUGCAAAC 5’

query=JS16_t0307502, target=FGSG_11624, score=4, range=996-1014, strand=-1

target  5’ ACGAAU-ACGGGCAUGUUUU 3’
           :::::: :::.:::.::::
query   3’ UGCUUAGUGCUCGUGCAAAC 5’

No results for JS16_t0255416
query=JS16_t3182705, target=FGSG_01449, score=3.5, range=852-872, strand=1
```

```
query=JS16_t0364688, target=FGSG_05404, score=4, range=966-982, strand=-1

target  5’ GGAG-GACGAUCAAGAGC 3’
           : :: :::::::::::.
query   3’ CGUCGCUGCUAGUUCUUC 5’

query=JS16_t0364688, target=FGSG_10684, score=4, range=1356-1372, strand=-1

target  5’ UCCGCGA-GAUCAAGAAG 3’
            : :::: ::::::::::
query   3’ CGUCGCUGCUAGUUCUUC 5’

query=JS16_t2223238, target=FGSG_08952, score=3.5, range=1532-1549, strand=1

target  5’ UGCAU-UCGGUGUUGAGCA 3’
           .:::: .:::::.::::::
query   3’ GCGUACGGCCACGACUCGU 5’

query=JS16_t2223238, target=FGSG_07581, score=3.5, range=194-212, strand=-1

target  5’ UGCAUGCGGGUGUUGAGCA 3’
           .:::::: ::::.::::::
query   3’ GCGUACGGCCACGACUCGU 5’

query=JS16_t2223238, target=FGSG_09826, score=4, range=727-746, strand=1

target  5’ CGUCAUCCUGGUGCUGAGCC 3’
           :: ::: :.::::::::::
query   3’ GC-GUACGGCCACGACUCGU 5’

query=JS16_t2223238, target=FGSG_03756, score=4, range=1098-1115, strand=1

target  5’ CG-AUUCUGGUGCUGAGUA 3’
           :: :: :.:::::::::.:
query   3’ GCGUACGGCCACGACUCGU 5’

query=JS16_t2223238, target=FGSG_01141, score=4, range=501-518, strand=1

target  5’ CG-AUGCCGCUGCUGAGCU 3’
           :: :::::: ::::::::
query   3’ GCGUACGGCCACGACUCGU 5’

query=JS16_t2223238, target=FGSG_05599, score=4, range=2846-2864, strand=1

target  5’ CUCAAUCCGGUGCUGAGCU 3’
           : ::  ::::::::::::
query   3’ GCGUACGGCCACGACUCGU 5’

query=JS16_t2223238, target=FGSG_02734, score=4, range=17-34, strand=-1

target  5’ CG-AUGCCGGUGUUAAGCA 3’
           :: :::::::::.: ::::
query   3’ GCGUACGGCCACGACUCGU 5’

query=JS16_t2223238, target=FGSG_10382, score=4, range=465-482, strand=-1

target  5’ CGC-UGCUGCUGCUGAGCA 3’
           ::: :::.: :::::::::
query   3’ GCGUACGGCCACGACUCGU 5’

query=JS16_t2223238, target=FGSG_11122, score=4, range=1172-1191, strand=-1

target  5’ CGCAUUACCGCUGCUGAGCA 3’
           :::: : ::: :::::::::
query   3’ GCGU-ACGGCCACGACUCGU 5’

query=JS16_t1326838, target=FGSG_02628, score=4, range=331-351, strand=1

target  5’ CUCGCUGCUGGCAAACAUGUU 3’
           :.:: .::::::::: :::::
query   3’ GGGCAGCGACCGUUU-UACAA 5’

query=JS16_t1326838, target=FGSG_08637, score=4, range=1005-1024, strand=1

target  5’ UGUGUGGCUGGCAAAAUGUG 3’
           . .:: :::::::::::::
query   3’ GGGCAGCGACCGUUUUACAA 5’

query=JS16_t1326838, target=FGSG_03758, score=4, range=1124-1143, strand=-1

target  5’ UCUGACACUGGCAAAAUGUA 3’
           .:.: : ::::::::::::
query   3’ GGGCAGCGACCGUUUUACAA 5’

No results for JS16_t4016169
No results for JS16_t0684558
No results for JS16_t3119373
No results for JS16_t0793875
No results for JS16_t4016225
No results for JS16_t2223372
No results for JS16_t1326965

target  5’ GAGAUGGGUUGAGGCUUACUU 3’
           ::.:: :::::::: ::::::
query   3’ CUUUA-CCAACUCCUAAUGAA 5’

query=JS16_t3182705, target=FGSG_05683, score=4, range=1235-1253, strand=1

target  5’ CGGAU-UUUGAGGAUUACUU 3’
            ..::  :::::::::::::
query   3’ CUUUACCAACUCCUAAUGAA 5’

query=JS16_t2286606, target=FGSG_00314, score=3, range=187-205, strand=1

target  5’ CAGGUUGCUUCCAAGCCCG 3’
           ::::..:::::::::: ::
query   3’ GUCCGGCGAAGGUUCGCGC 5’

query=JS16_t2286606, target=FGSG_05326, score=3, range=2497-2516, strand=1

target  5’ CAGGCUCGCUUCCAAGCGGG 3’
           ::::: :::::::::::: :
query   3’ GUCCG-GCGAAGGUUCGCGC 5’

query=JS16_t2286606, target=FGSG_00989, score=3.5, range=1027-1045, strand=1

target  5’ AAGGUCGCUUCCAAGAGCG 3’
            :::.:::::::::: :::
query   3’ GUCCGGCGAAGGUUCGCGC 5’

query=JS16_t2286606, target=FGSG_09227, score=3.5, range=2863-2880, strand=1

target  5’ CAGGCUGCUUCCAA-CGCU 3’
           :::::.:::::::: :::
query   3’ GUCCGGCGAAGGUUCGCGC 5’

query=JS16_t2286606, target=FGSG_09544, score=3.5, range=478-497, strand=-1

target  5’ AAGGUCGCCUUCCAAGCGCG 3’
            :::.:: ::::::::::::
query   3’ GUCCGGC-GAAGGUUCGCGC 5’

query=JS16_t2286606, target=FGSG_09026, score=4, range=1185-1203, strand=1

target  5’ UGCGCCGUUUCCAAGCGUG 3’
           .. ::::.:::::::::.:
query   3’ GUCCGGCGAAGGUUCGCGC 5’

query=JS16_t2286606, target=FGSG_05145, score=4, range=431-449, strand=1

target  5’ CAGCCCGCAUCCAGGCGCG 3’
           ::: :::: ::::.:::::
query   3’ GUCCGGCGAAGGUUCGCGC 5’

query=JS16_t2286606, target=FGSG_05344, score=4, range=1729-1747, strand=1

target  5’ GAGCCGGUUUCCAAGCGCG 3’
            :: : :.:::::::::::
query   3’ GUCCGGCGAAGGUUCGCGC 5’

query=JS16_t2286606, target=FGSG_00176, score=4, range=1309-1327, strand=1

target  5’ CAAGCAGCUACCAAGCGCG 3’
           :: :: ::: :::::::::
query   3’ GUCCGGCGAAGGUUCGCGC 5’

query=JS16_t2286606, target=FGSG_09638, score=4, range=4492-4510, strand=1

target  5’ CUGGAGGUUUCCAAGCGCG 3’
           : ::  :.:::::::::::
query   3’ GUCCGGCGAAGGUUCGCGC 5’

query=JS16_t2286606, target=FGSG_01245, score=4, range=625-643, strand=1

target  5’ CCUGACGCUUCCAAGCGCC 3’
           :  : :::::::::::::
query   3’ GUCCGGCGAAGGUUCGCGC 5’

query=JS16_t2286606, target=FGSG_05873, score=4, range=116-134, strand=1

target  5’ CACCCAGCUUCCAAGCGCC 3’
           ::  : ::::::::::::
query   3’ GUCCGGCGAAGGUUCGCGC 5’

query=JS16_t2286606, target=FGSG_02515, score=4, range=41-58, strand=1

target  5’ CAGACCG-UUCCAGGCGCG 3’
           ::: ::: :::::.:::::
query   3’ GUCCGGCGAAGGUUCGCGC 5’

query=JS16_t2286606, target=FGSG_08863, score=4, range=164-182, strand=-1

target  5’ GAUCCCGCUUCCAAGCGCA 3’
```

```
query=JS16_t4016276, target=FGSG_05911, score=3, range=1011-1028, strand=-1

target  5’ ACAUGGUAGUUCUUUGCA 3’
           ::: ::::.::::.::::
query   3’ UGUUCCAUUAAGAGACGU 5’

query=JS16_t4016276, target=FGSG_09105, score=3, range=1650-1667, strand=-1

target  5’ AUGAUGUAAUUCUCUGUA 3’
           :..: :::::::::::.:
query   3’ UGUUCCAUUAAGAGACGU 5’

query=JS16_t4016276, target=FGSG_03030, score=3.5, range=263-281, strand=-1

target  5’ AUAUGGGUAAUUCUUUGCG 3’
           :.: .:::::::::.:::.
query   3’ UGU-UCCAUUAAGAGACGU 5’

query=JS16_t4016276, target=FGSG_00040, score=3.5, range=2097-2114, strand=-1

target  5’ UCAGCGUAAUUCUCUGCU 3’
            ::. ::::::::::::
query   3’ UGUUCCAUUAAGAGACGU 5’

query=JS16_t4016276, target=FGSG_00667, score=4, range=254-271, strand=1

target  5’ GCAAUGUAAUUCUCUGGG 3’
           .::: ::::::::::: .
query   3’ UGUUCCAUUAAGAGACGU 5’

query=JS16_t4016276, target=FGSG_00252, score=4, range=791-808, strand=1

target  5’ AGAAGGCAAUUCUCUGCC 3’
           : :::: ::::::::::
query   3’ UGUUCCAUUAAGAGACGU 5’

query=JS16_t4016276, target=FGSG_06307, score=4, range=951-969, strand=-1

target  5’ CCAAUGGUACUUCUCUGCA 3’
            ::: :::: :::::::::
query   3’ UGUU-CCAUUAAGAGACGU 5’

No results for JS16_t4016277
query=JS16_t0335205, target=FGSG_06137, score=4, range=217-237, strand=-1

target  5’ CGCGCGCGGUGGCGUGCAAGU 3’
           : :. : .:::::::::::::
query   3’ GAGUAC-UCACCGCACGUUCA 5’

No results for JS16_t3119492
No results for JS16_t0466899
No results for JS16_t0394011
query=JS16_t2223558, target=FGSG_12251, score=4, range=508-530, strand=-1

target  5’ UUCAAGUAGUCGUAAGUUCCAGG 3’
           ::::: .:. :::::::::::.:
query   3’ AAGUUGGUUCGCAUUCAAGGUUC 5’

No results for JS16_t0684594
No results for JS16_t0194400
No results for JS16_t4016480
query=JS16_t3119695, target=FGSG_09534, score=2, range=2947-2967, strand=1

target  5’ GUCGAAAAGGCUGUCGAUCAU 3’
           :: ::: ::::::::::::::
query   3’ CAACUUAUCCGACAGCUAGUA 5’

query=JS16_t3119695, target=FGSG_11305, score=4, range=262-281, strand=1

target  5’ GUUGUAAAGGCUGUCGA-CAU 3’
           :::: : :::::::::: :::
query   3’ CAACUUAUCCGACAGCUAGUA 5’

query=JS16_t3119695, target=FGSG_11966, score=4, range=427-448, strand=-1

target  5’ GUUGACCAUGGCUGUUGAUCAU 3’
           :::::  : ::::::.::::::
query   3’ CAACUUAU-CCGACAGCUAGUA 5’

No results for JS16_t3119729
No results for JS16_t3119735
No results for JS16_t1327247
No results for JS16_t1327265
query=JS16_t2223688, target=FGSG_03856, score=3.5, range=249-267, strand=1

target  5’ UCUCG-GUCAUGCUUGCGGG 3’
           ::: : .::::::::::.::
query   3’ AGAUCUUAGUACGAACGUCC 5’

query=JS16_t2223688, target=FGSG_10429, score=3.5, range=308-327, strand=-1

target  5’ UUUAGAAGCAUGCUUGUAGG 3’
            :  ::::::::::::::
query   3’ GUCCGGCGAAGGUUCGCGC 5’

No results for JS16_t4079422
query=JS16_t0692097, target=FGSG_10608, score=4, range=12-29, strand=1

target  5’ UUCGUUUGACACUACGCC 3’
           :: ::::.::::::::.:
query   3’ AAACAAAUUGUGAUGUGU 5’

query=JS16_t0692097, target=FGSG_03018, score=4, range=741-758, strand=1

target  5’ UUACUUUAACCCUACACA 3’
           ::  :::::: :::::::
query   3’ AAACAAAUUGUGAUGUGU 5’

No results for JS16_t1390587
query=JS16_t2286666, target=FGSG_08265, score=3.5, range=91-109, strand=-1

target  5’ GUUGUUCGAAUCUGGCAGU 3’
           :.:: ::::::::: ::::
query   3’ CGACCAGCUUAGACAGUCA 5’

query=JS16_t2286666, target=FGSG_02305, score=4, range=832-850, strand=1

target  5’ GCUGGUAGAAUCCGUCAGU 3’
           :::::: ::::: ::::::
query   3’ CGACCAGCUUAGACAGUCA 5’

query=JS16_t2286666, target=FGSG_10111, score=4, range=484-501, strand=1

target  5’ GCU-GUCGAAUCUGUCGCU 3’
           ::: ::::::::::::. :
query   3’ CGACCAGCUUAGACAGUCA 5’

query=JS16_t2286666, target=FGSG_05195, score=4, range=1584-1603, strand=1

target  5’ GCAGGUCCGAGUCUGUCAGG 3’
           :: ::: :::.::::::::
query   3’ CGACCA-GCUUAGACAGUCA 5’

query=JS16_t2286666, target=FGSG_08338, score=4, range=262-280, strand=-1

target  5’ CCUUGUCGAGUCUGUCAGA 3’
            :: :::::.::::::::
query   3’ CGACCAGCUUAGACAGUCA 5’

query=JS16_t2286666, target=FGSG_13293, score=4, range=892-909, strand=-1

target  5’ GCU-GUCGGAUCUGUGAGU 3’
           ::: ::::.:::::: :::
query   3’ CGACCAGCUUAGACAGUCA 5’

query=JS16_t2286666, target=FGSG_04522, score=4, range=199-216, strand=-1

target  5’ GCU-UUUGAGUCUGUCAGU 3’
           :::  :.::.:::::::::
query   3’ CGACCAGCUUAGACAGUCA 5’

query=JS16_t2286666, target=FGSG_10123, score=4, range=289-308, strand=-1

target  5’ UCUGAGGCGAAUCUGUCAGC 3’
            ::: : ::::::::::::
query   3’ CGAC-CAGCUUAGACAGUCA 5’

query=JS16_t2286666, target=FGSG_09348, score=4, range=247-264, strand=-1

target  5’ GCU-GACGGAUCUGUCAGA 3’
           ::: : ::.::::::::::
query   3’ CGACCAGCUUAGACAGUCA 5’

query=JS16_t2286666, target=FGSG_04619, score=4, range=856-874, strand=-1

target  5’ GAUCGACGAAUCUGUCAGA 3’
           : : : ::::::::::::
query   3’ CGACCAGCUUAGACAGUCA 5’

No results for JS16_t2286668
query=JS16_t3182844, target=FGSG_04178, score=3, range=528-546, strand=1

target  5’ GGCCGUGGACUUGCACGCU 3’
           :.: : ::::::::::::.
query   3’ CUGCCUCCUGAACGUGCGG 5’

query=JS16_t3182844, target=FGSG_07212, score=3.5, range=987-1004, strand=-1

target  5’ GACCGAGGACUUGC-CGCU 3’
           ::: :::::::::: :::.
query   3’ CUGCCUCCUGAACGUGCGG 5’

query=JS16_t3182844, target=FGSG_09161, score=4, range=730-747, strand=1
```

```
           :.::::: ::::::::.:::
query   3’ AGAUCUUAGUACGAACGUCC 5’

query=JS16_t2223688, target=FGSG_05368, score=4, range=1237-1256, strand=1

target  5’ UAUCGAUUCAUGCUUGCAGC 3’
           : : :: ::::::::::::
query   3’ AGAUCUUAGUACGAACGUCC 5’

query=JS16_t2223688, target=FGSG_00595, score=4, range=3148-3166, strand=1

target  5’ UUUAAGAUCAUGC-UGCAGG 3’
           :.:: .::::::: ::::::
query   3’ AGAUCUUAGUACGAACGUCC 5’

query=JS16_t2223688, target=FGSG_10199, score=4, range=820-839, strand=-1

target  5’ UCGAGAAUCAUGUCUGCAGG 3’
           :: :::::::::. ::::::
query   3’ AGAUCUUAGUACGAACGUCC 5’

query=JS16_t2223688, target=FGSG_00431, score=4, range=223-241, strand=-1

target  5’ UCU-UAAUCGUGCUUGUAGG 3’
           :::  ::::.::::::.:::
query   3’ AGAUCUUAGUACGAACGUCC 5’

No results for JS16_t4016534
No results for JS16_t2223702
No results for JS16_t0305410
query=JS16_t4016551, target=FGSG_09168, score=3, range=688-707, strand=-1

target  5’ UGGUAAGUCAAGGUGAAGAU 3’
           ..:::::.:::::::::::
query   3’ GUCAUUCGGUUCCACUUCUU 5’

query=JS16_t4016551, target=FGSG_03890, score=3.5, range=1615-1634, strand=1

target  5’ CAGAAGGUCGAGGUGAAGAA 3’
           ::: :.:.:.::::::::::
query   3’ GUCAUUCGGUUCCACUUCUU 5’

query=JS16_t4016551, target=FGSG_00321, score=3.5, range=263-282, strand=1

target  5’ CGGCCAUCCAAGGUGAAGAA 3’
           :.:  : :::::::::::::
query   3’ GUCAUUCGGUUCCACUUCUU 5’

query=JS16_t4016551, target=FGSG_13911, score=4, range=1303-1322, strand=1

target  5’ CGGUCGGCCAAGGCGAAGAA 3’
           :.:: .::::::: ::::::
query   3’ GUCAUUCGGUUCCACUUCUU 5’

query=JS16_t4016551, target=FGSG_04069, score=4, range=647-665, strand=1

target  5’ CA-UUAGUCAAGGUGGAGAA 3’
           :: : ::.:::::::.::::
query   3’ GUCAUUCGGUUCCACUUCUU 5’

query=JS16_t4016551, target=FGSG_05556, score=4, range=992-1010, strand=1

target  5’ UGG-ACGCCGAGGUGAAGAA 3’
           ..: : :::.::::::::::
query   3’ GUCAUUCGGUUCCACUUCUU 5’

query=JS16_t4016551, target=FGSG_05398, score=4, range=2230-2249, strand=1

target  5’ GAUUAUUCCAAGGUGAAGAA 3’
            : ::  :::::::::::::
query   3’ GUCAUUCGGUUCCACUUCUU 5’

query=JS16_t4016551, target=FGSG_12354, score=4, range=384-404, strand=1

target  5’ CCGAAAGCGCAAGGUGAAGAA 3’
           : : :::: ::::::::::::
query   3’ GUCAUUCG-GUUCCACUUCUU 5’

query=JS16_t4016551, target=FGSG_09089, score=4, range=53-71, strand=1

target  5’ CAGUAA-CCAGAGUGAAGAA 3’
           :::::: :::. ::::::::
query   3’ GUCAUUCGGUUCCACUUCUU 5’

query=JS16_t4016551, target=FGSG_10350, score=4, range=335-355, strand=-1

target  5’ CAGGACAGCCAGGGUGAAGAC 3’
           ::: : :::::.::::::::
query   3’ GUCAU-UCGGUUCCACUUCUU 5’

query=JS16_t0166343, target=FGSG_11170, score=2, range=2005-2022, strand=-1

target  5’ GAC-GAGGACUUGUACGGC 3’
           ::: :::::::::.::: :
query   3’ CUGCCUCCUGAACGUGCGG 5’

query=JS16_t3182844, target=FGSG_09973, score=4, range=426-444, strand=-1

target  5’ GAUGGUGGGCUUGUACGCU 3’
           ::.:: ::.::::.::::.
query   3’ CUGCCUCCUGAACGUGCGG 5’

query=JS16_t3182844, target=FGSG_10261, score=4, range=1261-1279, strand=-1

target  5’ GUUGGAGGAUUUGCACGUU 3’
           : .::::::.::::::::..
query   3’ CUGCCUCCUGAACGUGCGG 5’

query=JS16_t3182844, target=FGSG_09373, score=4, range=853-872, strand=-1

target  5’ GUUGGAUGGAUUUGCACGCU 3’
           : .::: :::.::::::::.
query   3’ CUGCCU-CCUGAACGUGCGG 5’

query=JS16_t3182844, target=FGSG_03225, score=4, range=724-743, strand=-1

target  5’ AACGCGUGGACUUGCAUGCC 3’
            ::: : :::::::::.:::
query   3’ CUGC-CUCCUGAACGUGCGG 5’

query=JS16_t3182844, target=FGSG_05733, score=4, range=1075-1092, strand=-1

target  5’ GGUGGUGGACUUGC-CGCC 3’
           :..:: :::::::: ::::
query   3’ CUGCCUCCUGAACGUGCGG 5’

query=JS16_t3182844, target=FGSG_12811, score=4, range=437-454, strand=-1

target  5’ GA-GAAGGACUUGCACUCC 3’
           :: : ::::::::::: ::
query   3’ CUGCCUCCUGAACGUGCGG 5’

No results for JS16_t3182864
No results for JS16_t4079567
query=JS16_t2286742, target=FGSG_07407, score=4, range=708-725, strand=1

target  5’ GGAAU-GGCUCAGCGGAGG 3’
           .:::. :::::::::::.
query   3’ UCUUGCCCGAGUCGCCUUG 5’

query=JS16_t2286742, target=FGSG_06101, score=4, range=335-354, strand=1

target  5’ AGAAGGAGGCUCAGCGGAGG 3’
           :::: : :::::::::::.
query   3’ UCUUGC-CCGAGUCGCCUUG 5’

query=JS16_t2286742, target=FGSG_05462, score=4, range=3092-3109, strand=1

target  5’ AGGGC-GACUCAGCGGAAC 3’
           ::..: : :::::::::::
query   3’ UCUUGCCCGAGUCGCCUUG 5’

query=JS16_t2286742, target=FGSG_04789, score=4, range=510-529, strand=-1

target  5’ AGAACCGGCUGCAGCGGAGC 3’
           ::::: :::: ::::::::.:
query   3’ UCUUGCCCGA-GUCGCCUUG 5’

No results for JS16_t1390696
No results for JS16_t2286772
query=JS16_t0474548, target=FGSG_02757, score=3, range=801-819, strand=1

target  5’ AGCCAUGUUUGGUCGAGCA 3’
           :::  ::.:::::::::::
query   3’ UCGUAACGAACCAGCUCGU 5’

query=JS16_t0474548, target=FGSG_00464, score=3, range=235-253, strand=1

target  5’ ACCAGUGCUUGGUCGAGUA 3’
           : :: ::::::::::::.:
query   3’ UCGUAACGAACCAGCUCGU 5’

query=JS16_t0474548, target=FGSG_05902, score=3, range=1572-1589, strand=1

target  5’ AGAAUUG-UUGGUCGAGCA 3’
           :: :::: :::::::::::
query   3’ UCGUAACGAACCAGCUCGU 5’

query=JS16_t0474548, target=FGSG_01528, score=3, range=153-171, strand=-1

target  5’ AGUGCUGCUUGGUCGGGCA 3’
           ::.. ::::::::::.:::
query   3’ UCGUAACGAACCAGCUCGU 5’
```

```
target  5' GAUGUCGUCAACCAUGGG 3'
           :::::::::::::::.:
query   3' CUACAGCAGUUGGUAUCA 5'
```

query=JS16_t0166343, target=FGSG_00667, score=2.5, range=808-825, strand=1

```
target  5' AGUGUCGUCAGCCAUAGU 3'
            .::::::::.:::::::
query   3' CUACAGCAGUUGGUAUCA 5'
```

query=JS16_t0166343, target=FGSG_02677, score=3, range=2125-2142, strand=1

```
target  5' GAUGGCGUCAACGAUAGU 3'
           :::: ::::::: :::::
query   3' CUACAGCAGUUGGUAUCA 5'
```

query=JS16_t0166343, target=FGSG_06728, score=3, range=244-261, strand=-1

```
target  5' CUUGUCGUUAACCAUAGU 3'
             ::::::.:::::::::
query   3' CUACAGCAGUUGGUAUCA 5'
```

query=JS16_t0166343, target=FGSG_02640, score=3, range=373-390, strand=-1

```
target  5' GAUGCCGUCAACCAUGGG 3'
           :::: ::::::::::.:
query   3' CUACAGCAGUUGGUAUCA 5'
```

query=JS16_t0166343, target=FGSG_10523, score=3, range=1124-1140, strand=-1

```
target  5' CAUGUCGUCAACCA-AGU 3'
            ::::::::::::: :::
query   3' CUACAGCAGUUGGUAUCA 5'
```

query=JS16_t0166343, target=FGSG_03964, score=3.5, range=407-425, strand=1

```
target  5' CGUGUUCGUCAACCAUAGA 3'
            .:: :::::::::::::
query   3' CUAC-AGCAGUUGGUAUCA 5'
```

query=JS16_t0166343, target=FGSG_05522, score=3.5, range=1246-1264, strand=-1

```
target  5' GGUCGCCGUCAACCAUAGA 3'
           :.: : :::::::::::
query   3' CUA-CAGCAGUUGGUAUCA 5'
```

query=JS16_t0166343, target=FGSG_05571, score=4, range=3937-3954, strand=1

```
target  5' GAUUUUGUCAACUAUAGC 3'
           ::: :.::::::.::::
query   3' CUACAGCAGUUGGUAUCA 5'
```

query=JS16_t0166343, target=FGSG_05353, score=4, range=6-23, strand=1

```
target  5' CAUGAUGUCAACCAUGGU 3'
            ::: .:::::::::.::
query   3' CUACAGCAGUUGGUAUCA 5'
```

query=JS16_t0166343, target=FGSG_09981, score=4, range=2383-2400, strand=1

```
target  5' CAUGUCGUCAACGAUAGC 3'
            ::::::::::: ::::
query   3' CUACAGCAGUUGGUAUCA 5'
```

query=JS16_t0166343, target=FGSG_05083, score=4, range=568-584, strand=1

```
target  5' GAUAUCGUCAACCG-AGU 3'
           ::: :::::::::. :::
query   3' CUACAGCAGUUGGUAUCA 5'
```

query=JS16_t0166343, target=FGSG_07418, score=4, range=1046-1062, strand=1

```
target  5' CAGGUC-UCAACCAUAGU 3'
            : ::: :::::::::::
query   3' CUACAGCAGUUGGUAUCA 5'
```

query=JS16_t0166343, target=FGSG_04395, score=4, range=37-53, strand=1

```
target  5' GUUAUCGUCAACC-UAGU 3'
           : : ::::::::: ::::
query   3' CUACAGCAGUUGGUAUCA 5'
```

query=JS16_t0166343, target=FGSG_05685, score=4, range=586-603, strand=-1

```
target  5' GAUGUCGUCAACCAUGAA 3'
           :::::::::::::::.
query   3' CUACAGCAGUUGGUAUCA 5'
```

query=JS16_t0166343, target=FGSG_00297, score=4, range=1591-1608, strand=-1

```
target  5' GAUGGUGUUGACCAUAGU 3'
           :::: .::..::::::::
```

query=JS16_t0474548, target=FGSG_11599, score=3, range=3123-3140, strand=-1

```
target  5' AGC-UUGCUUGAUCGAGCA 3'
           ::: ::::::: :::::::
query   3' UCGUAACGAACCAGCUCGU 5'
```

query=JS16_t0474548, target=FGSG_05996, score=3.5, range=3157-3175, strand=1

```
target  5' AGUGUUGCUUGGUGGAGCG 3'
           ::..::::::::: ::::.
query   3' UCGUAACGAACCAGCUCGU 5'
```

query=JS16_t0474548, target=FGSG_00974, score=3.5, range=2859-2876, strand=1

```
target  5' AG-GUGGCUUGGUCGAGCU 3'
           :: .: ::::::::::::
query   3' UCGUAACGAACCAGCUCGU 5'
```

query=JS16_t0474548, target=FGSG_05775, score=3.5, range=1433-1451, strand=-1

```
target  5' AGCUUUGUUUGGUUGAGCG 3'
           ::: :::.:::::.::::.
query   3' UCGUAACGAACCAGCUCGU 5'
```

query=JS16_t0474548, target=FGSG_10597, score=3.5, range=519-536, strand=-1

```
target  5' AG-GUUGCUUGGUUGAGCC 3'
           :: .:::::::::.::::
query   3' UCGUAACGAACCAGCUCGU 5'
```

query=JS16_t0474548, target=FGSG_08416, score=4, range=106-124, strand=1

```
target  5' AGAACUGCUUGGUCAAGCA 3'
           :: : ::::::::: ::::
query   3' UCGUAACGAACCAGCUCGU 5'
```

query=JS16_t0474548, target=FGSG_01515, score=4, range=1101-1119, strand=1

```
target  5' AUCAAUGCUUGGUCGAUCA 3'
           : :: :::::::::: ::
query   3' UCGUAACGAACCAGCUCGU 5'
```

query=JS16_t0474548, target=FGSG_09988, score=4, range=692-710, strand=1

```
target  5' ACCAGUGCUUGGUCGAGGA 3'
           : :: ::::::::::: :
query   3' UCGUAACGAACCAGCUCGU 5'
```

query=JS16_t0474548, target=FGSG_00252, score=4, range=688-705, strand=1

```
target  5' AACA-UGCUUGGUCUAGCA 3'
           : :: ::::::::: ::::
query   3' UCGUAACGAACCAGCUCGU 5'
```

query=JS16_t0474548, target=FGSG_08403, score=4, range=1869-1886, strand=1

```
target  5' UGCCUUGC-UGGUCGAGCA 3'
            :: :::: ::::::::::
query   3' UCGUAACGAACCAGCUCGU 5'
```

query=JS16_t0474548, target=FGSG_06997, score=4, range=2103-2122, strand=1

```
target  5' AUUAUUGCUUGGCUCGAGCG 3'
           : .::::::::: ::::::.
query   3' UCGUAACGAACC-AGCUCGU 5'
```

query=JS16_t0474548, target=FGSG_06395, score=4, range=5198-5216, strand=-1

```
target  5' CGAAUUGCCUGGUCGAGCA 3'
            : ::::: ::::::::::
query   3' UCGUAACGAACCAGCUCGU 5'
```

query=JS16_t0474548, target=FGSG_01429, score=4, range=2423-2442, strand=-1

```
target  5' UGCAUCGCUUGGUCGGAGCA 3'
            :::: :::::::: :::::
query   3' UCGUAACGAACCAG-CUCGU 5'
```

query=JS16_t0474548, target=FGSG_00728, score=4, range=1931-1948, strand=-1

```
target  5' AGC-UUGCUUGGUUGAGGA 3'
           ::: :::::::::.::: :
query   3' UCGUAACGAACCAGCUCGU 5'
```

query=JS16_t0474548, target=FGSG_04381, score=4, range=2570-2587, strand=-1

```
target  5' GUCGUU-CUUGGUCGAGCA 3'
           . :.:: :::::::::::
query   3' UCGUAACGAACCAGCUCGU 5'
```

query=JS16_t0474548, target=FGSG_07564, score=4, range=1719-1737, strand=-1

```
query   3' CUACAGCAGUUGGUAUCA 5'

query=JS16_t0166343, target=FGSG_13543, score=4, range=3631-3648, strand=-1

target  5' GAUUUCAUCAAUCAUAGU 3'
           ::: :: ::::.::::::
query   3' CUACAGCAGUUGGUAUCA 5'

query=JS16_t0166343, target=FGSG_04590, score=4, range=1078-1095, strand=-1

target  5' GACGCCGUCAAUCAUGGU 3'
           :: : ::::::.:::.::
query   3' CUACAGCAGUUGGUAUCA 5'

query=JS16_t0166343, target=FGSG_02072, score=4, range=985-1002, strand=-1

target  5' GAUACCGUCAGCCGUAGU 3'
           :::  :::::.::.::::
query   3' CUACAGCAGUUGGUAUCA 5'

query=JS16_t0166343, target=FGSG_02051, score=4, range=536-552, strand=-1

target  5' GAUGUUGUCAA-CAUAGG 3'
           :::::.::::: :::::
query   3' CUACAGCAGUUGGUAUCA 5'

query=JS16_t0166343, target=FGSG_11356, score=4, range=1225-1241, strand=-1

target  5' GAU-UCGUCAGCCAUGGA 3'
           ::: ::::::.::::.:
query   3' CUACAGCAGUUGGUAUCA 5'

query=JS16_t0166343, target=FGSG_10162, score=4, range=790-807, strand=-1

target  5' GUCAUCGUCAGCCAUAGU 3'
           :   ::::::.:::::::
query   3' CUACAGCAGUUGGUAUCA 5'

query=JS16_t0166343, target=FGSG_01161, score=4, range=1868-1884, strand=-1

target  5' GAUGUCGUCAAGCA-AGU 3'
           ::::::::::: :: :::
query   3' CUACAGCAGUUGGUAUCA 5'

query=JS16_t0166343, target=FGSG_10436, score=4, range=1561-1577, strand=-1

target  5' GAUAUCGUCAACCA-AGA 3'
           ::: :::::::::: ::
query   3' CUACAGCAGUUGGUAUCA 5'

query=JS16_t0166343, target=FGSG_06379, score=4, range=2774-2790, strand=-1

target  5' GCAGUCGUCAACCA-AGU 3'
           :  ::::::::::: :::
query   3' CUACAGCAGUUGGUAUCA 5'

query=JS16_t0166343, target=FGSG_05764, score=4, range=1178-1194, strand=-1

target  5' GAUGUAGUCAA-CAUAGU 3'
           ::::: ::::: ::::::
query   3' CUACAGCAGUUGGUAUCA 5'

No results for JS16_t3119790
No results for JS16_t1327313
query=JS16_t2223762, target=FGSG_13338, score=3, range=735-757, strand=-1

target  5' CGUCUCGGAGAGCUUGGGUAGCC 3'
           :::::: :::::::::::::.:
query   3' GCAGAGACUCUCGAACCCAUUGA 5'

No results for JS16_t4016613
query=JS16_t1327365, target=FGSG_02550, score=2.5, range=296-313, strand=1

target  5' GUCC-UUGGAAGAAGUGGA 3'
           :::: .:::::::::.:::
query   3' CAGGUGACCUUCUUCGCCU 5'

query=JS16_t1327365, target=FGSG_09877, score=2.5, range=505-524, strand=-1

target  5' GUAUCAGUGGAAGAAGCGGA 3'
           :: .:: :::::::::::::
query   3' CA-GGUGACCUUCUUCGCCU 5'

query=JS16_t1327365, target=FGSG_11395, score=3, range=1755-1774, strand=1

target  5' UCCCACAUGGAAGAAGCGGA 3'
             :::: :::::::::::::
query   3' CAGGUG-ACCUUCUUCGCCU 5'

query=JS16_t1327365, target=FGSG_08555, score=3, range=146-163, strand=1

target  5' GUCCA-UGGAAGAAGCUGA 3'

target  5' ACCAUAGCUGGGUCGAGCA 3'
           : ::: ::: :::::::::
query   3' UCGUAACGAACCAGCUCGU 5'

query=JS16_t0474548, target=FGSG_02577, score=4, range=5708-5726, strand=-1

target  5' AUCCUGGCUUGGUUGAGCA 3'
           : : : :::::::.:::::
query   3' UCGUAACGAACCAGCUCGU 5'

query=JS16_t0474548, target=FGSG_04694, score=4, range=3551-3569, strand=-1

target  5' UGGAAGGCUUGGUCGAGCA 3'
            : :  :::::::::::::
query   3' UCGUAACGAACCAGCUCGU 5'

query=JS16_t0474548, target=FGSG_05190, score=4, range=528-545, strand=-1

target  5' AG-AAUGGUUGGUCGAGCA 3'
           :: : :: :::::::::::
query   3' UCGUAACGAACCAGCUCGU 5'

query=JS16_t0474548, target=FGSG_08391, score=4, range=668-685, strand=-1

target  5' AGC-UCGCUUGGUCGUGCA 3'
           ::: : ::::::::: :::
query   3' UCGUAACGAACCAGCUCGU 5'

query=JS16_t0474548, target=FGSG_04263, score=4, range=617-634, strand=-1

target  5' AGCAGAG-UUGGUCGAGCA 3'
           ::::  : :::::::::::
query   3' UCGUAACGAACCAGCUCGU 5'

query=JS16_t0474548, target=FGSG_11012, score=4, range=354-373, strand=-1

target  5' AGAAAUGCUUGGUCUGAGCA 3'
           :: : ::::::::: :::::
query   3' UCGUAACGAACCAG-CUCGU 5'

query=JS16_t0474548, target=FGSG_13859, score=4, range=1697-1714, strand=-1

target  5' AGUGUUGC-UGGUCGAGUA 3'
           ::..:::: ::::::::.:
query   3' UCGUAACGAACCAGCUCGU 5'

query=JS16_t0474548, target=FGSG_06636, score=4, range=867-884, strand=-1

target  5' ACCAGUGC-UGGUCGAGCA 3'
           : :: ::: ::::::::::
query   3' UCGUAACGAACCAGCUCGU 5'

query=JS16_t4079630, target=FGSG_04287, score=4, range=473-495, strand=-1

target  5' GACAAGAUUUUUGCCCAUUGGUG 3'
           :: : .: :.:::::::::::::
query   3' CUAUCUUCAGAACGGGUAACCAC 5'

No results for JS16_t2286825
query=JS16_t0176679, target=FGSG_01162, score=3.5, range=1085-1104, strand=-1

target  5' GACGA-GAACACCAAGAGGUU 3'
           ::::: : :::::::::.::.
query   3' CUGCUACCUGUGGUUCUUCAG 5'

query=JS16_t0176679, target=FGSG_04419, score=4, range=1021-1041, strand=1

target  5' GAGGAUGAGCACCAGGAAGUC 3'
           :: :::: .:::::.::::::
query   3' CUGCUACCUGUGGUUCUUCAG 5'

query=JS16_t0176679, target=FGSG_06032, score=4, range=102-122, strand=1

target  5' GCCGAAGAACACUAAGAAGUC 3'
           : ::: : ::::.::::::::
query   3' CUGCUACCUGUGGUUCUUCAG 5'

query=JS16_t0176679, target=FGSG_01436, score=4, range=325-344, strand=1

target  5' GAC-AGGGACACCAAGAUGUC 3'
           ::: : ::::::::::: :::
query   3' CUGCUACCUGUGGUUCUUCAG 5'

query=JS16_t0176679, target=FGSG_04731, score=4, range=278-297, strand=-1

target  5' GGC-AUGGACACCAAGAAGCU 3'
           :.: :::::::::::::: .
query   3' CUGCUACCUGUGGUUCUUCAG 5'

query=JS16_t0583530, target=FGSG_06388, score=2, range=1112-1128, strand=-1

target  5' CUCCAACUGGUU-CUCAU 3'
```

```
           ::::: :::::::::: ::
query   3' CAGGUGACCUUCUUCGCCU 5'

query=JS16_t1327365, target=FGSG_04755, score=3.5, range=258-277, strand=1

target  5' GACCAGCUGGAAGAAGUGGG 3'
           : ::: ::::::::::.::.
query   3' CAGGU-GACCUUCUUCGCCU 5'

query=JS16_t1327365, target=FGSG_04612, score=3.5, range=213-231, strand=-1

target  5' GUUCUCUGGAAGAAGUGGC 3'
           ::.: ::::::::::.::
query   3' CAGGUGACCUUCUUCGCCU 5'

query=JS16_t1327365, target=FGSG_05562, score=3.5, range=63-81, strand=-1

target  5' GGGUACUGGAAGAGGCGGA 3'
           :  .:::::::::.:::::
query   3' CAGGUGACCUUCUUCGCCU 5'

query=JS16_t1327365, target=FGSG_05728, score=3.5, range=428-445, strand=-1

target  5' GUCCGUU-GAAGAAGCGGG 3'
           ::::..: ::::::::::.
query   3' CAGGUGACCUUCUUCGCCU 5'

query=JS16_t1327365, target=FGSG_09477, score=4, range=1619-1637, strand=1

target  5' GUUUGUUGGACGAAGCGGA 3'
           ::....:::: ::::::::
query   3' CAGGUGACCUUCUUCGCCU 5'

query=JS16_t1327365, target=FGSG_10935, score=4, range=327-345, strand=1

target  5' UAUCAUUGGAGGAAGCGGA 3'
             .::.::::.::::::::
query   3' CAGGUGACCUUCUUCGCCU 5'

query=JS16_t1327365, target=FGSG_13305, score=4, range=355-373, strand=1

target  5' CUGCGCUGGAGGAAGCGGG 3'
            : :.:::::.:::::::.
query   3' CAGGUGACCUUCUUCGCCU 5'

query=JS16_t1327365, target=FGSG_08719, score=4, range=2094-2112, strand=1

target  5' CGCCACUGGAAGAAGCAGA 3'
             ::::::::::::: ::
query   3' CAGGUGACCUUCUUCGCCU 5'

query=JS16_t1327365, target=FGSG_05855, score=4, range=2846-2864, strand=1

target  5' GACUGCUGGAAGAAGCCGA 3'
           : :..:::::::::: ::
query   3' CAGGUGACCUUCUUCGCCU 5'

query=JS16_t1327365, target=FGSG_02366, score=4, range=417-436, strand=1

target  5' GUCAUUUUGGAAGAAGUGGA 3'
           ::: . .:::::::::.:::
query   3' CAG-GUGACCUUCUUCGCCU 5'

query=JS16_t1327365, target=FGSG_04709, score=4, range=144-163, strand=1

target  5' GCCGACAUGGAAGAAGUGGA 3'
           : : :: :::::::::.:::
query   3' CAGGUG-ACCUUCUUCGCCU 5'

query=JS16_t1327365, target=FGSG_07005, score=4, range=638-655, strand=1

target  5' GCACACU-GAAGAAGCGGA 3'
           :  :::: :::::::::::
query   3' CAGGUGACCUUCUUCGCCU 5'

query=JS16_t1327365, target=FGSG_05295, score=4, range=130-147, strand=-1

target  5' GUCU-UUGGGAGGAGCGGA 3'
           :::. .:::.::.::::::
query   3' CAGGUGACCUUCUUCGCCU 5'

query=JS16_t1327365, target=FGSG_01867, score=4, range=493-510, strand=-1

target  5' GUUC-UUGGAGGAAGUGGA 3'
           ::.: .::::.::::.:::
query   3' CAGGUGACCUUCUUCGCCU 5'

query=JS16_t1327365, target=FGSG_03065, score=4, range=200-218, strand=-1

target  5' AUCUCGUGGAAGAAGCGGG 3'
            ::.  ::::::::::::.
query   3' CAGGUGACCUUCUUCGCCU 5'
```

```
           :::::::::::: :::::
query   3' GAGGUUGACCAACGAGUA 5'

query=JS16_t0583530, target=FGSG_00294, score=3, range=627-643, strand=1

target  5' CUACAACUGGUU-CUCAU 3'
           :: ::::::::: :::::
query   3' GAGGUUGACCAACGAGUA 5'

query=JS16_t0583530, target=FGSG_13394, score=3.5, range=2159-2176, strand=1

target  5' UUCAAACUGGUUGUUCAC 3'
           .:: :::::::::.:::
query   3' GAGGUUGACCAACGAGUA 5'

query=JS16_t0583530, target=FGSG_00206, score=3.5, range=1-18, strand=-1

target  5' GUCCGGUUGGUUGCUCAU 3'
            :::...::::::::::
query   3' GAGGUUGACCAACGAGUA 5'

query=JS16_t0583530, target=FGSG_10095, score=3.5, range=1114-1131, strand=-1

target  5' CUCUACCUGGUUGUUCAU 3'
           :::.: :::::::.::::
query   3' GAGGUUGACCAACGAGUA 5'

query=JS16_t0583530, target=FGSG_13844, score=3.5, range=763-780, strand=-1

target  5' UUGCAACUGGUUGUUCAG 3'
           .: ::::::::::.:::
query   3' GAGGUUGACCAACGAGUA 5'

query=JS16_t0583530, target=FGSG_11871, score=3.5, range=397-413, strand=-1

target  5' CUGUAACUGGUUGCU-AU 3'
           :: .:::::::::: ::
query   3' GAGGUUGACCAACGAGUA 5'

query=JS16_t0583530, target=FGSG_11516, score=4, range=9-26, strand=1

target  5' CAUCGGCUGGUUGCUUAU 3'
           : .:..:::::::::.::
query   3' GAGGUUGACCAACGAGUA 5'

query=JS16_t0583530, target=FGSG_01870, score=4, range=765-782, strand=1

target  5' CGCCAACUGGUUCCUCAA 3'
           : :::::::::: ::::
query   3' GAGGUUGACCAACGAGUA 5'

query=JS16_t0583530, target=FGSG_04279, score=4, range=4019-4037, strand=1

target  5' CUGCAUACUGGUUGCCCAU 3'
           :: :: ::::::::: :::
query   3' GAGGU-UGACCAACGAGUA 5'

query=JS16_t0583530, target=FGSG_00831, score=4, range=1471-1487, strand=1

target  5' GUCCUACUGGUUG-UCAU 3'
            ::: :::::::: ::::
query   3' GAGGUUGACCAACGAGUA 5'

query=JS16_t0583530, target=FGSG_02996, score=4, range=1759-1775, strand=1

target  5' CUGGAACUGGUUGC-CAU 3'
           ::  :::::::::: :::
query   3' GAGGUUGACCAACGAGUA 5'

query=JS16_t0583530, target=FGSG_00982, score=4, range=635-652, strand=-1

target  5' CAUCGGCUGGUUGCUCGU 3'
           : .:..::::::::::.:
query   3' GAGGUUGACCAACGAGUA 5'

query=JS16_t0583530, target=FGSG_06335, score=4, range=1006-1023, strand=-1

target  5' CUUGGGCUGGUUGCUCAG 3'
           ::. ..:::::::::::
query   3' GAGGUUGACCAACGAGUA 5'

query=JS16_t0583530, target=FGSG_08209, score=4, range=9500-9517, strand=-1

target  5' CUCCAACUGUUUGAUCAU 3'
           ::::::::: ::: ::::
query   3' GAGGUUGACCAACGAGUA 5'

query=JS16_t0583530, target=FGSG_07240, score=4, range=714-731, strand=-1

target  5' CUCACACUGGUUGUUCGU 3'
           :::  ::::::::.::.:
query   3' GAGGUUGACCAACGAGUA 5'
```

```
No results for JS16_t2223841
query=JS16_t1327473, target=FGSG_09648, score=2, range=665-682, strand=1

target  5’ AUGGAGCUGCUGUUAUGG 3’
           :.:.:::.::::::::::
query   3’ UGCUUCGGCGACAAUACC 5’

query=JS16_t1327473, target=FGSG_08779, score=2, range=186-202, strand=1

target  5’ AC-AAGCCGCUGUUAUGA 3’
           :: ::::::::::::::
query   3’ UGCUUCGGCGACAAUACC 5’

query=JS16_t1327473, target=FGSG_07251, score=2.5, range=751-768, strand=-1

target  5’ ACGAGGCUGCUGUUGUGG 3’
           ::::.::.::::::.:::
query   3’ UGCUUCGGCGACAAUACC 5’

query=JS16_t1327473, target=FGSG_09815, score=3, range=869-886, strand=1

target  5’ ACCAAGCCGCUAUUAUGG 3’
           :: :::::::: ::::::
query   3’ UGCUUCGGCGACAAUACC 5’

query=JS16_t1327473, target=FGSG_09815, score=3, range=744-761, strand=-1

target  5’ GUCAAGCCGUUGUUAUGG 3’
           .. ::::::.::::::::
query   3’ UGCUUCGGCGACAAUACC 5’

query=JS16_t1327473, target=FGSG_04981, score=3, range=887-904, strand=-1

target  5’ ACGAUGCCGCUGUUAGGG 3’
           :::: :::::::::: ::
query   3’ UGCUUCGGCGACAAUACC 5’

query=JS16_t1327473, target=FGSG_08055, score=3.5, range=98-115, strand=1

target  5’ AUGAAGAUGCUGUUAUGG 3’
           :.:::: .::::::::::
query   3’ UGCUUCGGCGACAAUACC 5’

query=JS16_t1327473, target=FGSG_07128, score=3.5, range=1202-1219, strand=1

target  5’ GUGAGGGCGCUGUUAUGG 3’
           ..::.: :::::::::::
query   3’ UGCUUCGGCGACAAUACC 5’

query=JS16_t1327473, target=FGSG_09014, score=3.5, range=1298-1315, strand=1

target  5’ UGGAGGCUGCUGUUAUGG 3’
             ::.::.::::::::::
query   3’ UGCUUCGGCGACAAUACC 5’

query=JS16_t1327473, target=FGSG_08644, score=3.5, range=1556-1573, strand=1

target  5’ CUGAAGCCGCUGUUAAGG 3’
            .::::::::::::: ::
query   3’ UGCUUCGGCGACAAUACC 5’

query=JS16_t1327473, target=FGSG_06959, score=3.5, range=184-202, strand=1

target  5’ GCCAACGCCGCUGUUAUGC 3’
           .: :: ::::::::::::
query   3’ UGCUU-CGGCGACAAUACC 5’

query=JS16_t1327473, target=FGSG_06421, score=3.5, range=1490-1507, strand=-1

target  5’ GCGAAGCUGCUGUCAUGG 3’
           .::::::.::::: ::::
query   3’ UGCUUCGGCGACAAUACC 5’

query=JS16_t1327473, target=FGSG_03973, score=4, range=539-556, strand=1

target  5’ AUCGAGUUGCUGUUAUGG 3’
           :. .::..::::::::::
query   3’ UGCUUCGGCGACAAUACC 5’

query=JS16_t1327473, target=FGSG_13032, score=4, range=1154-1171, strand=1

target  5’ ACGAAGCCGUUGUCAUGA 3’
           :::::::::.::: :::
query   3’ UGCUUCGGCGACAAUACC 5’

query=JS16_t1327473, target=FGSG_11659, score=4, range=3620-3637, strand=1

target  5’ ACGACGCCGCUUUUGUGG 3’
           :::: :::::: ::.:::
query   3’ UGCUUCGGCGACAAUACC 5’

query=JS16_t0583530, target=FGSG_06790, score=4, range=685-703, strand=-1

target  5’ CUCCAGACUGGUUGCUCUC 3’
           ::::: :::::::::::
query   3’ GAGGU-UGACCAACGAGUA 5’

query=JS16_t0583530, target=FGSG_08884, score=4, range=91-108, strand=-1

target  5’ CUUGGACUGGUUUCUCAU 3’
           ::. .::::::: :::::
query   3’ GAGGUUGACCAACGAGUA 5’

query=JS16_t0583530, target=FGSG_10566, score=4, range=4262-4278, strand=-1

target  5’ UGUCAACUGGUUGC-CAU 3’
           . .::::::::::: :::
query   3’ GAGGUUGACCAACGAGUA 5’

query=JS16_t0583530, target=FGSG_09858, score=4, range=92-110, strand=-1

target  5’ CACCAGACUGGUUGAUCAU 3’
           : ::: :::::::: ::::
query   3’ GAGGU-UGACCAACGAGUA 5’

query=JS16_t0583530, target=FGSG_04572, score=4, range=764-780, strand=-1

target  5’ CUCCAAUUGG-UGCUCGU 3’
           ::::::.::: :::::.:
query   3’ GAGGUUGACCAACGAGUA 5’

No results for JS16_t2286953
No results for JS16_t2287070
No results for JS16_t1390978
No results for JS16_t0337302
No results for JS16_t0474585
query=JS16_t4079959, target=FGSG_09605, score=3, range=1557-1575, strand=1

target  5’ AAUUC-UCGUCGAUAUCAUC 3’
           : ::: :::.::::::::::
query   3’ UCAAGCAGCGGCUAUAGUAG 5’

query=JS16_t4079959, target=FGSG_09437, score=3, range=1120-1138, strand=-1

target  5’ AG-UCUUCGUCGAUAUCAUC 3’
           :: :: :::.::::::::::
query   3’ UCAAGCAGCGGCUAUAGUAG 5’

query=JS16_t4079959, target=FGSG_08785, score=3.5, range=416-434, strand=-1

target  5’ GGUUCGUCGCCGAUAUU-UC 3’
           .:::::::::::::::. ::
query   3’ UCAAGCAGCGGCUAUAGUAG 5’

query=JS16_t4079959, target=FGSG_01591, score=4, range=329-348, strand=1

target  5’ AGUUCUUCGCCGAUAUCGCC 3’
           ::::: :::::::::::. :
query   3’ UCAAGCAGCGGCUAUAGUAG 5’

query=JS16_t4079959, target=FGSG_07019, score=4, range=1425-1443, strand=1

target  5’ AGUU-GUUGCGGAUAUCAUC 3’
           :::: ::.:: :::::::::
query   3’ UCAAGCAGCGGCUAUAGUAG 5’

query=JS16_t4079959, target=FGSG_03838, score=4, range=237-255, strand=1

target  5’ AG-UCGUUGCCGAUAUCUUC 3’
           :: ::::.:::::::::: ::
query   3’ UCAAGCAGCGGCUAUAGUAG 5’

query=JS16_t4079959, target=FGSG_08352, score=4, range=917-936, strand=1

target  5’ AGGCCCUCGCCGAUAUCGUC 3’
           ::  : :::::::::::.::
query   3’ UCAAGCAGCGGCUAUAGUAG 5’

query=JS16_t4079959, target=FGSG_02061, score=4, range=225-243, strand=1

target  5’ AAUUC-ACGCCGAUAUCGUC 3’
           : :::  ::::::::::.::
query   3’ UCAAGCAGCGGCUAUAGUAG 5’

query=JS16_t4079959, target=FGSG_11328, score=4, range=152-170, strand=1

target  5’ AAUUCAUCGCC-AUAUCAUC 3’
           : ::: ::::: ::::::::
query   3’ UCAAGCAGCGGCUAUAGUAG 5’

query=JS16_t4079959, target=FGSG_12676, score=4, range=250-269, strand=-1
```

```
query=JS16_t1327473, target=FGSG_07665, score=4, range=1367-1384, strand=1

target  5’ ACCAAGUCGCUGUCAUGG 3’
           :: :::.:::::: ::::
query   3’ UGCUUCGGCGACAAUACC 5’

query=JS16_t1327473, target=FGSG_01927, score=4, range=578-595, strand=1

target  5’ AAGAAGCCGUUGUUAUCG 3’
           : :::::::.:::::: :
query   3’ UGCUUCGGCGACAAUACC 5’

query=JS16_t1327473, target=FGSG_05642, score=4, range=3470-3487, strand=1

target  5’ ACCAUGUCGCUGUUGUGG 3’
           :: : :.:::::::.:::
query   3’ UGCUUCGGCGACAAUACC 5’

query=JS16_t1327473, target=FGSG_01724, score=4, range=1720-1737, strand=1

target  5’ GAGGAGCCGCUGUUAUCG 3’
           .  :.::::::::::: :
query   3’ UGCUUCGGCGACAAUACC 5’

query=JS16_t1327473, target=FGSG_09869, score=4, range=876-892, strand=1

target  5’ AC-AAGUCGCUGUUAUCG 3’
           :: :::.::::::::: :
query   3’ UGCUUCGGCGACAAUACC 5’

query=JS16_t1327473, target=FGSG_10464, score=4, range=7244-7261, strand=1

target  5’ ACAAUGCCGCUGUUAUCG 3’
           :: : ::::::::::: :
query   3’ UGCUUCGGCGACAAUACC 5’

query=JS16_t1327473, target=FGSG_03691, score=4, range=293-311, strand=-1

target  5’ GCAGGUGCCGCUGUUGUGG 3’
           .: :.  :::::::::.:::
query   3’ UG-CUUCGGCGACAAUACC 5’

query=JS16_t1327473, target=FGSG_01189, score=4, range=515-532, strand=-1

target  5’ UCAAAGCCGCUGUUAUAG 3’
            : ::::::::::::: :
query   3’ UGCUUCGGCGACAAUACC 5’

query=JS16_t1327473, target=FGSG_01925, score=4, range=1059-1075, strand=-1

target  5’ AC-AUGCUGCUGUUGUGG 3’
           :: : ::.::::::.:::
query   3’ UGCUUCGGCGACAAUACC 5’

No results for JS16_t1327513
query=JS16_t4016854, target=FGSG_06025, score=4, range=1280-1301, strand=-1

target  5’ AUGAUCUCAGCUUCCUGCUCAG 3’
           :: :::: ::::.:::.:::::
query   3’ UA-UAGACUCGAGGGAUGAGUC 5’

No results for JS16_t2224013
No results for JS16_t2224028
No results for JS16_t2224034
No results for JS16_t0575995
No results for JS16_t4016919
No results for JS16_t3120138
No results for JS16_t2224072
query=JS16_t1327695, target=FGSG_09180, score=2.5, range=659-677, strand=1

target  5’ ACAAGAUAUCGGAGCCGAA 3’
           : ::.::::::::.:::::
query   3’ UCUUUUAUAGCCUUGGCUU 5’

query=JS16_t1327695, target=FGSG_04770, score=3, range=395-413, strand=1

target  5’ AGGAAAUAUCGGAACGGAG 3’
           ::.:::::::::::: ::.
query   3’ UCUUUUAUAGCCUUGGCUU 5’

query=JS16_t1327695, target=FGSG_05519, score=3, range=6587-6605, strand=1

target  5’ AUGAAGUAUCGGAACUGAA 3’
           : .::.::::::::.:::
query   3’ UCUUUUAUAGCCUUGGCUU 5’

query=JS16_t1327695, target=FGSG_07118, score=3, range=975-993, strand=1

target  5’ AAGAGAUGUCGGAACCGAA 3’
           : .:.::.:::::::::::
query   3’ UCUUUUAUAGCCUUGGCUU 5’

target  5’ CGUUCGUCGUCGCUAUCAUC 3’
            ::::::::.:: :::::::
query   3’ UCAAGCAGCGGCUAUAGUAG 5’

query=JS16_t4079959, target=FGSG_00584, score=4, range=1918-1938, strand=-1

target  5’ AGCUUCGUCGCCGAAAUCGUC 3’
           :: ::::::::::: :::.::
query   3’ UC-AAGCAGCGGCUAUAGUAG 5’

query=JS16_t4079959, target=FGSG_13499, score=4, range=1051-1070, strand=-1

target  5’ CAUGCGUCGUCGAUAUCAUC 3’
             : :::::.::::::::::
query   3’ UCAAGCAGCGGCUAUAGUAG 5’

query=JS16_t4079959, target=FGSG_13172, score=4, range=1851-1870, strand=-1

target  5’ GGAUUAUCGUCGAUAUCAUC 3’
           .: :.  :::.::::::::::
query   3’ UCAAGCAGCGGCUAUAGUAG 5’

query=JS16_t4079959, target=FGSG_13422, score=4, range=1366-1384, strand=-1

target  5’ AGUUC-UCGCCGAUUUUAUC 3’
           ::::: :::::::: :.:::
query   3’ UCAAGCAGCGGCUAUAGUAG 5’

query=JS16_t4079959, target=FGSG_00059, score=4, range=73-91, strand=-1

target  5’ AGGUCG-CGCUGAUGUCAUC 3’
           :: ::: :::.:::.:::::
query   3’ UCAAGCAGCGGCUAUAGUAG 5’

query=JS16_t0220299, target=FGSG_04396, score=3, range=451-470, strand=-1

target  5’ CAU-AAUGUUGGUGAUUUGGU 3’
           ::: :::::::.::::.::::
query   3’ GUAUUUACAACUACUAGACCA 5’

query=JS16_t0220299, target=FGSG_00362, score=3.5, range=1870-1889, strand=-1

target  5’ UGUGAA-GUUGAUGGUCUGGU 3’
           ..:.:: :::::::.::::::
query   3’ GUAUUUACAACUACUAGACCA 5’

query=JS16_t0220299, target=FGSG_06832, score=4, range=861-880, strand=1

target  5’ CACAAAGGUUGAU-AUCUGGU 3’
           :: ::: :::::: :::::::
query   3’ GUAUUUACAACUACUAGACCA 5’

No results for JS16_t3183295
No results for JS16_t3183308
No results for JS16_t2287209
No results for JS16_t2287238
No results for JS16_t0474607
query=JS16_t3183387, target=FGSG_10205, score=4, range=1590-1611, strand=1

target  5’ UGACUG-GAUUGACGACAUCAAG 3’
           .::::. : :::::::::::::
query   3’ GCUGAUACCAACUGCUGUAGUUG 5’

query=JS16_t3183387, target=FGSG_05083, score=4, range=565-586, strand=-1

target  5’ CCACU-CGGUUGACGAUAUCAAC 3’
           : :::  :::::::::.::::::
query   3’ GCUGAUACCAACUGCUGUAGUUG 5’

query=JS16_t1391155, target=FGSG_05058, score=3, range=175-193, strand=-1

target  5’ CGCG-CAUGUGGUCGUCACC 3’
           :::: :: ::::::::::::
query   3’ GCGCUGUUCACCAGCAGUGG 5’

query=JS16_t1391155, target=FGSG_07362, score=4, range=58-76, strand=1

target  5’ CGC-UCUGGUGGUCGUCACC 3’
           :::  : .::::::::::::
query   3’ GCGCUGUUCACCAGCAGUGG 5’

query=JS16_t1391155, target=FGSG_13575, score=4, range=369-387, strand=-1

target  5’ CGCGACUAGUGGUCG-CAUC 3’
           :::::: :::::::: ::.:
query   3’ GCGCUGUUCACCAGCAGUGG 5’

query=JS16_t1391155, target=FGSG_09949, score=4, range=1501-1519, strand=-1

target  5’ CGC-ACCAGUGGUUGUUACC 3’
           ::: :: ::::::.::.:::
query   3’ GCGCUGUUCACCAGCAGUGG 5’
```

```
query=JS16_t1327695, target=FGSG_08884, score=3, range=178-196, strand=-1

target  5’ AGAGAAUACCGGAACCGAG 3’
           :::.:::: :::::::::.
query   3’ UCUUUUAUAGCCUUGGCUU 5’

query=JS16_t1327695, target=FGSG_04702, score=3.5, range=756-774, strand=1

target  5’ AGAAAUAAUCGGAACCGAG 3’
           :::::  :::::::::::.
query   3’ UCUUUUAUAGCCUUGGCUU 5’

query=JS16_t1327695, target=FGSG_11948, score=3.5, range=235-253, strand=-1

target  5’ GUGGAAUAUCGGAAUCGAA 3’
           . ..::::::::::.::::
query   3’ UCUUUUAUAGCCUUGGCUU 5’

query=JS16_t1327695, target=FGSG_13194, score=4, range=603-621, strand=1

target  5’ AAAGGAUAUCGGAACUGAU 3’
           : :..::::::::::.::
query   3’ UCUUUUAUAGCCUUGGCUU 5’

query=JS16_t1327695, target=FGSG_08400, score=4, range=1901-1918, strand=1

target  5’ AGGAAG-AUCGGGACCGAA 3’
           ::.::. :::::.::::::
query   3’ UCUUUUAUAGCCUUGGCUU 5’

query=JS16_t1327695, target=FGSG_06982, score=4, range=1766-1784, strand=1

target  5’ CAAAAAUAUCUGAACCGAA 3’
             :::::::: ::::::::
query   3’ UCUUUUAUAGCCUUGGCUU 5’

query=JS16_t1327695, target=FGSG_12368, score=4, range=522-540, strand=1

target  5’ GGGACAUAUCGGAACCCAA 3’
           .:.: ::::::::::: ::
query   3’ UCUUUUAUAGCCUUGGCUU 5’

query=JS16_t1327695, target=FGSG_07635, score=4, range=777-795, strand=1

target  5’ CGGAGCUAUCGGAACCGAC 3’
            :.:. ::::::::::::
query   3’ UCUUUUAUAGCCUUGGCUU 5’

query=JS16_t1327695, target=FGSG_06126, score=4, range=403-421, strand=-1

target  5’ AGAAAAUGCCGGAACCGAU 3’
           :::::::. :::::::::
query   3’ UCUUUUAUAGCCUUGGCUU 5’

query=JS16_t1327695, target=FGSG_04876, score=4, range=874-892, strand=-1

target  5’ CGAGGAUACCGGAACCGAA 3’
            ::..::: ::::::::::
query   3’ UCUUUUAUAGCCUUGGCUU 5’

query=JS16_t1327695, target=FGSG_07433, score=4, range=708-727, strand=-1

target  5’ CGACAAUAUCGGAACCAGAA 3’
            :: ::::::::::: :::
query   3’ UCUUUUAUAGCCUUGG-CUU 5’

query=JS16_t1327695, target=FGSG_03678, score=4, range=840-858, strand=-1

target  5’ AGCAUAUAUCGGAAUCGAC 3’
           :: : :::::::::.:::
query   3’ UCUUUUAUAGCCUUGGCUU 5’

query=JS16_t3120173, target=FGSG_06108, score=3.5, range=443-462, strand=1

target  5’ UUAUCGCUUCAUGGGCAUGG 3’
           :::::.:::::::::: .::
query   3’ AAUAGUGAAGUACCCGCGCC 5’

query=JS16_t3120173, target=FGSG_08491, score=4, range=960-979, strand=1

target  5’ CUCUCGUUUCAUGGGCGUGG 3’
            : ::..::::::::::.::
query   3’ AAUAGUGAAGUACCCGCGCC 5’

query=JS16_t3120173, target=FGSG_03560, score=4, range=461-479, strand=1

target  5’ CUAUGACUU-AUGGGCGCGG 3’
            ::: :::: ::::::::::
query   3’ AAUAGUGAAGUACCCGCGCC 5’

query=JS16_t3120173, target=FGSG_05275, score=4, range=606-624, strand=-1

query=JS16_t1391155, target=FGSG_06745, score=4, range=154-172, strand=-1

target  5’ CACGA-AAGUGGUCGUCAUG 3’
           : ::: ::::::::::::.
query   3’ GCGCUGUUCACCAGCAGUGG 5’

query=JS16_t1391157, target=FGSG_11228, score=3.5, range=1793-1810, strand=1

target  5’ AGGCUAGAGUCUAUGGUG 3’
            :::. ::::::::::::
query   3’ CCCGGCCUCAGAUACCAC 5’

query=JS16_t1391157, target=FGSG_05343, score=3.5, range=34-51, strand=1

target  5’ GAGCUGGAGUUUAUGGUU 3’
           : ::.:::::.::::::
query   3’ CCCGGCCUCAGAUACCAC 5’

query=JS16_t1391157, target=FGSG_08128, score=3.5, range=2387-2404, strand=-1

target  5’ GGGCUGGAGUUUCUGGUG 3’
           ::::.::::::.: :::::
query   3’ CCCGGCCUCAGAUACCAC 5’

query=JS16_t1391157, target=FGSG_10377, score=4, range=441-457, strand=1

target  5’ GGGUUGGAGUUUA-GGUG 3’
           :::..:::::.:: ::::
query   3’ CCCGGCCUCAGAUACCAC 5’

query=JS16_t1391157, target=FGSG_07216, score=4, range=197-214, strand=1

target  5’ GUGCCGGAGGUUAUGGUG 3’
           : ::::::: .:::::::
query   3’ CCCGGCCUCAGAUACCAC 5’

query=JS16_t1391157, target=FGSG_13660, score=4, range=624-641, strand=1

target  5’ GGGCGGGGGUCUCUGGUG 3’
           :::: ::.:::: :::::
query   3’ CCCGGCCUCAGAUACCAC 5’

query=JS16_t1391157, target=FGSG_00184, score=4, range=397-414, strand=1

target  5’ GAGUUUGAGUCUAUGGUG 3’
           : :.. ::::::::::::
query   3’ CCCGGCCUCAGAUACCAC 5’

query=JS16_t1391157, target=FGSG_07955, score=4, range=1713-1730, strand=1

target  5’ GGUUUGGUGUCUAUGGUG 3’
           :: ..:: ::::::::::
query   3’ CCCGGCCUCAGAUACCAC 5’

query=JS16_t1391157, target=FGSG_08557, score=4, range=4235-4251, strand=1

target  5’ GGGUU-GAGUCUAUGGUU 3’
           :::.. :::::::::::
query   3’ CCCGGCCUCAGAUACCAC 5’

query=JS16_t1391157, target=FGSG_00573, score=4, range=1689-1706, strand=1

target  5’ UGACCGGAGUCUAAGGUG 3’
            : :::::::::: ::::
query   3’ CCCGGCCUCAGAUACCAC 5’

query=JS16_t1391157, target=FGSG_04870, score=4, range=945-962, strand=1

target  5’ UGACCUGAGUCUAUGGUG 3’
            : :: ::::::::::::
query   3’ CCCGGCCUCAGAUACCAC 5’

query=JS16_t1391157, target=FGSG_05754, score=4, range=537-554, strand=-1

target  5’ GAGCCGGAGUUGAUGGUG 3’
           : ::::::::. ::::::
query   3’ CCCGGCCUCAGAUACCAC 5’

query=JS16_t1391157, target=FGSG_06228, score=4, range=927-944, strand=-1

target  5’ GUGUUGGAGUCUCUGGUG 3’
           : :..::::::: :::::
query   3’ CCCGGCCUCAGAUACCAC 5’

query=JS16_t1391157, target=FGSG_00841, score=4, range=630-646, strand=-1

target  5’ GGGCGGGAG-CUGUGGUG 3’
           :::: :::: ::.:::::
query   3’ CCCGGCCUCAGAUACCAC 5’

query=JS16_t1391157, target=FGSG_00625, score=4, range=455-471, strand=-1
```

```
target  5’ UUGU-GCUUCUUGGGCGCGG 3’
           ::.: .:::: :::::::::
query   3’ AAUAGUGAAGUACCCGCGCC 5’

query=JS16_t1327737, target=FGSG_03776, score=3, range=70-88, strand=1

target  5’ AUUGAAGGUUUAUUGUCUU 3’
           ::..: :.::::::::::
query   3’ UAGUUACUAAAUAACAGAA 5’

query=JS16_t1327737, target=FGSG_06950, score=3, range=500-519, strand=-1

target  5’ AUCAAUGAUUUGUUGAUCUU 3’
           ::::::::::.::: ::::
query   3’ UAGUUACUAAAUAAC-AGAA 5’

query=JS16_t1327737, target=FGSG_10601, score=4, range=138-156, strand=-1

target  5’ AUCAAUGAUUUGCUGUCUA 3’
           ::::::::::. :::::
query   3’ UAGUUACUAAAUAACAGAA 5’

query=JS16_t1327737, target=FGSG_00208, score=4, range=209-226, strand=-1

target  5’ AUUGA-GAUUUGUUGUUUU 3’
           ::..: :::::.::::.::
query   3’ UAGUUACUAAAUAACAGAA 5’

query=JS16_t1327737, target=FGSG_13441, score=4, range=13-32, strand=-1

target  5’ UUCAAUGGAUUUGUUGUCUC 3’
            ::::: ::::.::::::
query   3’ UAGUUA-CUAAAUAACAGAA 5’

query=JS16_t1327737, target=FGSG_02323, score=4, range=248-266, strand=-1

target  5’ UUCCAUAAUUUAUUGUCUU 3’
            :: :: ::::::::::::
query   3’ UAGUUACUAAAUAACAGAA 5’

No results for JS16_t1327774
query=JS16_t0684676, target=FGSG_11098, score=2, range=1054-1071, strand=-1

target  5’ AAGCCAGGUAAAGAGAUA 3’
           :: :::::.:::::::::
query   3’ UUGGGUCCGUUUCUCUAU 5’

query=JS16_t0684676, target=FGSG_05432, score=2.5, range=2209-2226, strand=1

target  5’ AAUCUAGGCAAAGGGAUG 3’
           ::.:.:::::::.:::.
query   3’ UUGGGUCCGUUUCUCUAU 5’

query=JS16_t0684676, target=FGSG_03388, score=2.5, range=4525-4542, strand=-1

target  5’ AAUCCAGGUAAAGAGAUC 3’
           ::.:::::.::::::::
query   3’ UUGGGUCCGUUUCUCUAU 5’

query=JS16_t0684676, target=FGSG_00136, score=2.5, range=957-974, strand=-1

target  5’ AGACCAGGCAGAGAGAUA 3’
           :.  :::::::.:::::::
query   3’ UUGGGUCCGUUUCUCUAU 5’

query=JS16_t0684676, target=FGSG_07127, score=3, range=1795-1812, strand=1

target  5’ UUCCCAGGCAAAGAGAUU 3’
             ::::::::::::::
query   3’ UUGGGUCCGUUUCUCUAU 5’

query=JS16_t0684676, target=FGSG_09391, score=3, range=1966-1983, strand=1

target  5’ GAUGCAGGCAAAGAGAUU 3’
           .:.  ::::::::::::
query   3’ UUGGGUCCGUUUCUCUAU 5’

query=JS16_t0684676, target=FGSG_04279, score=3.5, range=4621-4638, strand=1

target  5’ GACGCAGGCGAAGAGAUU 3’
           .:: :::::.:::::::
query   3’ UUGGGUCCGUUUCUCUAU 5’

query=JS16_t0684676, target=FGSG_03197, score=3.5, range=1058-1075, strand=1

target  5’ AACUAAGGCAGAGAGAUC 3’
           :::.  :::::.::::::
query   3’ UUGGGUCCGUUUCUCUAU 5’

query=JS16_t0684676, target=FGSG_05308, score=3.5, range=481-498, strand=1

target  5’ AACUGGGGCAAAGAGAUC 3’
```

```
target  5’ GUGACGGAGUCU-UGGUG 3’
           : : :::::::: :::::
query   3’ CCCGGCCUCAGAUACCAC 5’

No results for JS16_t4080079
No results for JS16_t1391166
query=JS16_t0337305, target=FGSG_11115, score=2.5, range=62-79, strand=-1

target  5’ CACCGGCAUAUGAGGGGU 3’
           ::::.:::::.:::::.:
query   3’ GUGGUCGUAUGCUCCCUA 5’

query=JS16_t0337305, target=FGSG_06736, score=3, range=1598-1615, strand=1

target  5’ CACCAGCAUACGAGGCAG 3’
           :::::::::::::: :
query   3’ GUGGUCGUAUGCUCCCUA 5’

query=JS16_t0337305, target=FGSG_06167, score=3, range=626-642, strand=1

target  5’ CAUCGGCAUACGA-GGAU 3’
           ::.:.:::::::: ::::
query   3’ GUGGUCGUAUGCUCCCUA 5’

query=JS16_t0337305, target=FGSG_09740, score=3, range=541-558, strand=-1

target  5’ CGUCAGCAUGUGAGGGAU 3’
           :..::::::..:::::::
query   3’ GUGGUCGUAUGCUCCCUA 5’

query=JS16_t0337305, target=FGSG_05376, score=3.5, range=2161-2178, strand=1

target  5’ CGCCAGCUUAUGAGGGAU 3’
           :.::::: ::.:::::::
query   3’ GUGGUCGUAUGCUCCCUA 5’

query=JS16_t0337305, target=FGSG_11659, score=3.5, range=1296-1314, strand=1

target  5’ CGUCGUGCGUACGAGGGAU 3’
           :..:.  ::.::::::::::
query   3’ GUGGU-CGUAUGCUCCCUA 5’

query=JS16_t0337305, target=FGSG_13003, score=3.5, range=278-294, strand=-1

target  5’ UA-CAGCAUGUGAGGGAU 3’
           .: ::::::..:::::::
query   3’ GUGGUCGUAUGCUCCCUA 5’

query=JS16_t0337305, target=FGSG_12010, score=4, range=330-348, strand=1

target  5’ UGCCAGCAUACUGAGGGAA 3’
           ..:::::::: ::::::
query   3’ GUGGUCGUAUG-CUCCCUA 5’

query=JS16_t0337305, target=FGSG_07354, score=4, range=39-56, strand=1

target  5’ CACCAGCAUCCGAGAGAU 3’
           ::::::::: :::: :::
query   3’ GUGGUCGUAUGCUCCCUA 5’

query=JS16_t0337305, target=FGSG_02634, score=4, range=668-685, strand=1

target  5’ CAGCAGUAUACAAGGGAU 3’
           :: :::.:::: ::::::
query   3’ GUGGUCGUAUGCUCCCUA 5’

query=JS16_t0337305, target=FGSG_07553, score=4, range=2488-2506, strand=1

target  5’ CGUAAUGCAUACGAGGGGU 3’
           :..  : :::::::::::.:
query   3’ GUGGU-CGUAUGCUCCCUA 5’

query=JS16_t0337305, target=FGSG_13634, score=4, range=1312-1330, strand=-1

target  5’ CAUCGGCAUAUGAGGGCAU 3’
           ::.:.:::::.::::: ::
query   3’ GUGGUCGUAUGCUCCC-UA 5’

query=JS16_t0337305, target=FGSG_00052, score=4, range=113-130, strand=-1

target  5’ GGCUCGCAUGCGAGGGAU 3’
            .:.  ::::.::::::::
query   3’ GUGGUCGUAUGCUCCCUA 5’

query=JS16_t0337305, target=FGSG_04437, score=4, range=1034-1051, strand=-1

target  5’ CAGCAGCAUAUGAGUGAU 3’
           :: :::::::.::: :::
query   3’ GUGGUCGUAUGCUCCCUA 5’

query=JS16_t0337305, target=FGSG_00811, score=4, range=200-217, strand=-1
```

```
           :::. .:::::::::::
query   3' UUGGGUCCGUUUCUCUAU 5'

query=JS16_t0684676, target=FGSG_12296, score=3.5, range=153-170, strand=1

target  5' AUCCCAGGCAAAGCGAUG 3'
           : ::::::::::: :::.
query   3' UUGGGUCCGUUUCUCUAU 5'

query=JS16_t0684676, target=FGSG_03557, score=3.5, range=167-184, strand=1

target  5' ACCCCAUGCAAAGAGAUG 3'
           : :::: ::::::::::.
query   3' UUGGGUCCGUUUCUCUAU 5'

query=JS16_t0684676, target=FGSG_03162, score=3.5, range=579-595, strand=1

target  5' AAUCCAGGCAAA-AGAUC 3'
           ::.:::::::::: ::::
query   3' UUGGGUCCGUUUCUCUAU 5'

query=JS16_t0684676, target=FGSG_06403, score=3.5, range=543-561, strand=1

target  5' AGCCCAGAGCAGAGAGAUA 3'
           :.::::: :::.:::::::
query   3' UUGGGUC-CGUUUCUCUAU 5'

query=JS16_t0684676, target=FGSG_03337, score=3.5, range=2896-2912, strand=-1

target  5' AGUCCAGGCAAAG-GAUG 3'
           :..:::::::::: :::.
query   3' UUGGGUCCGUUUCUCUAU 5'

query=JS16_t0684676, target=FGSG_11553, score=3.5, range=321-338, strand=-1

target  5' AUCUCAGUCAAAGAGAUA 3'
           : :.::: ::::::::::
query   3' UUGGGUCCGUUUCUCUAU 5'

query=JS16_t0684676, target=FGSG_09077, score=4, range=318-335, strand=1

target  5' GGUCUAUGCAAAGAGAUA 3'
           ...:.: :::::::::::
query   3' UUGGGUCCGUUUCUCUAU 5'

query=JS16_t0684676, target=FGSG_02010, score=4, range=986-1003, strand=1

target  5' CGUCCAGGCCAAGAGAUA 3'
            ..::::::: ::::::::
query   3' UUGGGUCCGUUUCUCUAU 5'

query=JS16_t0684676, target=FGSG_03272, score=4, range=598-616, strand=1

target  5' UACCUAGGCAAAGAGAAUG 3'
            :::.:::::::::: ::.
query   3' UUGGGUCCGUUUCUC-UAU 5'

query=JS16_t0684676, target=FGSG_02724, score=4, range=3309-3326, strand=1

target  5' AGUACAGGUAAAGAGAUU 3'
           :..  ::::.::::::::
query   3' UUGGGUCCGUUUCUCUAU 5'

query=JS16_t0684676, target=FGSG_13829, score=4, range=325-341, strand=1

target  5' AACCCAGGC-AAGGGAUC 3'
           ::::::::: :::.:::
query   3' UUGGGUCCGUUUCUCUAU 5'

query=JS16_t0684676, target=FGSG_07549, score=4, range=2473-2490, strand=1

target  5' CAUGUAGGCAAAGAGAUC 3'
            :. .:::::::::::::
query   3' UUGGGUCCGUUUCUCUAU 5'

query=JS16_t0684676, target=FGSG_02286, score=4, range=329-345, strand=1

target  5' AACUU-GGCAAAGAGAUC 3'
           :::.. :::::::::::
query   3' UUGGGUCCGUUUCUCUAU 5'

query=JS16_t0684676, target=FGSG_13805, score=4, range=1174-1191, strand=1

target  5' UACCAAGGCCAAGAGAUA 3'
            ::: :::: ::::::::
query   3' UUGGGUCCGUUUCUCUAU 5'

query=JS16_t0684676, target=FGSG_12100, score=4, range=48-66, strand=-1

target  5' AACCCAGGCGACAGGGAUA 3'
           :::::::::.: ::.::::
query   3' UUGGGUCCGUU-UCUCUAU 5'

target  5' AACCAGCAUCCGAGGGAC 3'
            :::::::: :::::::
query   3' GUGGUCGUAUGCUCCCUA 5'

query=JS16_t0337305, target=FGSG_07924, score=4, range=1271-1288, strand=-1

target  5' UUUCAGGAUACGAGGGAU 3'
           . .::: :::::::::::
query   3' GUGGUCGUAUGCUCCCUA 5'

query=JS16_t0337305, target=FGSG_07264, score=4, range=1128-1146, strand=-1

target  5' CACGGAGCGUACGAGGGAA 3'
           :::  :::.:::::::::
query   3' GUG-GUCGUAUGCUCCCUA 5'

query=JS16_t0337305, target=FGSG_03129, score=4, range=296-312, strand=-1

target  5' CGUC-GCAUACGAGAGAU 3'
           :..: ::::::::: :::
query   3' GUGGUCGUAUGCUCCCUA 5'

No results for JS16_t1391196
query=JS16_t4080125, target=FGSG_07702, score=3, range=595-613, strand=1

target  5' UCGCUUGGU-ACCUAUUUCC 3'
           :: :::::: ::::::::::
query   3' AGGGAACCAUUGGAUAAAGG 5'

query=JS16_t4080125, target=FGSG_12326, score=3.5, range=19-38, strand=1

target  5' UUUCUUGGUCACCUAUUUCU 3'
           :..::::::: :::::::::.
query   3' AGGGAACCAUUGGAUAAAGG 5'

query=JS16_t4080125, target=FGSG_00015, score=3.5, range=483-503, strand=-1

target  5' UCCUUUGGGUAACCUAAUUCC 3'
           :::.:: ::::::::: ::::
query   3' AGGGAA-CCAUUGGAUAAAGG 5'

query=JS16_t0692183, target=FGSG_02767, score=3.5, range=587-608, strand=1

target  5' GCUUGACGGCAUACUUCAUUUU 3'
           .:.::  .::::::::::::::
query   3' UGGAC-CUCGUAUGAAGUAAAA 5'

query=JS16_t0692183, target=FGSG_00273, score=4, range=165-184, strand=1

target  5' ACCU-GAGCAUACUUCCUUUG 3'
           :::: ::::::::::: :::
query   3' UGGACCUCGUAUGAAGUAAAA 5'

query=JS16_t0692183, target=FGSG_10599, score=4, range=706-725, strand=-1

target  5' ACCUUGAGCAU-CUUCAUUUG 3'
           :::: :::::: ::::::::
query   3' UGGACCUCGUAUGAAGUAAAA 5'

No results for JS16_t4080158
query=JS16_t1391273, target=FGSG_00297, score=2.5, range=1807-1825, strand=1

target  5' CGAUCGCGCUCAGCAAGAA 3'
           :::::.:::::: ::::::
query   3' GCUAGUGCGAGUAGUUCUU 5'

query=JS16_t1391273, target=FGSG_13665, score=2.5, range=5099-5117, strand=1

target  5' UGGGCGCGCUCAUCAAGAA 3'
           .:.  :.:::::::::::::
query   3' GCUAGUGCGAGUAGUUCUU 5'

query=JS16_t1391273, target=FGSG_03528, score=2.5, range=239-257, strand=1

target  5' CGAGUGCGCUCAUCAAGAG 3'
           ::: ..::::::::::::.
query   3' GCUAGUGCGAGUAGUUCUU 5'

query=JS16_t1391273, target=FGSG_04244, score=2.5, range=651-669, strand=1

target  5' CGCUUACGCUCAUCAAGAU 3'
           :: :.:::::::::::::
query   3' GCUAGUGCGAGUAGUUCUU 5'

query=JS16_t1391273, target=FGSG_05556, score=2.5, range=1208-1225, strand=-1

target  5' CGGU-ACGCUCAUCGAGAA 3'
           ::.: :::::::::.::::
query   3' GCUAGUGCGAGUAGUUCUU 5'

query=JS16_t1391273, target=FGSG_06560, score=3, range=971-989, strand=1
```

```
query=JS16_t0684676, target=FGSG_10760, score=4, range=759-776, strand=-1

target  5’ AGCCCAGGCGAAGAGUUG 3’
           :.:::::::.::::: :.
query   3’ UUGGGUCCGUUUCUCUAU 5’

query=JS16_t0684676, target=FGSG_12484, score=4, range=31-48, strand=-1

target  5’ AAACCGGGCAGAGGGAUA 3’
           :: ::.::::.::.::::
query   3’ UUGGGUCCGUUUCUCUAU 5’

query=JS16_t0684676, target=FGSG_00775, score=4, range=394-411, strand=-1

target  5’ AACCCAGGCAAAGACGUU 3’
           :::::::::::::: .:
query   3’ UUGGGUCCGUUUCUCUAU 5’

query=JS16_t0684676, target=FGSG_10573, score=4, range=2832-2850, strand=-1

target  5’ GGCCCAGGCAAAGGAGAUC 3’
           ..:::::::::: :::::
query   3’ UUGGGUCCGUUU-CUCUAU 5’

query=JS16_t0684676, target=FGSG_06865, score=4, range=2119-2136, strand=-1

target  5’ AACACGGUCAAAGAGAUA 3’
           ::: :.: ::::::::::
query   3’ UUGGGUCCGUUUCUCUAU 5’

query=JS16_t0684676, target=FGSG_08546, score=4, range=228-245, strand=-1

target  5’ AAGAUAGGCAAAGGGAUG 3’
           ::  .::::::::.:::.
query   3’ UUGGGUCCGUUUCUCUAU 5’

query=JS16_t0684676, target=FGSG_12779, score=4, range=86-103, strand=-1

target  5’ UAGCCAGGCAAAGAAAUA 3’
            : ::::::::::: :::
query   3’ UUGGGUCCGUUUCUCUAU 5’

query=JS16_t0684676, target=FGSG_05701, score=4, range=337-353, strand=-1

target  5’ AACAUAGG-AAAGAGAUG 3’
           ::: .::: ::::::::.
query   3’ UUGGGUCCGUUUCUCUAU 5’

query=JS16_t4017077, target=FGSG_13956, score=3, range=182-200, strand=1

target  5’ GGGUGGUCUACAUCCAUGG 3’
            ::: :::::::::::.::
query   3’ ACCAACAGAUGUAGGUGCC 5’

query=JS16_t4017077, target=FGSG_09039, score=3, range=999-1016, strand=1

target  5’ UGGUCG-CUACAUCCACGG 3’
           :::: : ::::::::::::
query   3’ ACCAACAGAUGUAGGUGCC 5’

query=JS16_t4017077, target=FGSG_01902, score=4, range=1145-1163, strand=1

target  5’ CGCUUGUCUACAUGCACGG 3’
            : :::::::::: :::::
query   3’ ACCAACAGAUGUAGGUGCC 5’

query=JS16_t4017077, target=FGSG_05949, score=4, range=619-636, strand=1

target  5’ UGG-UGUCGACAUCCACGA 3’
           ::: :::: :::::::::
query   3’ ACCAACAGAUGUAGGUGCC 5’

query=JS16_t4017077, target=FGSG_03243, score=4, range=363-380, strand=1

target  5’ UGG-UCUCUUCAUCCACGG 3’
           ::: : ::: :::::::::
query   3’ ACCAACAGAUGUAGGUGCC 5’

query=JS16_t4017077, target=FGSG_06681, score=4, range=312-329, strand=1

target  5’ UGGUU-UCUACAUCGACGC 3’
           ::::: :::::::: :::
query   3’ ACCAACAGAUGUAGGUGCC 5’

query=JS16_t4017077, target=FGSG_09961, score=4, range=791-808, strand=-1

target  5’ UAGUUG-CUACAUCCAUGG 3’
           : :::: :::::::::.::
query   3’ ACCAACAGAUGUAGGUGCC 5’

query=JS16_t4017077, target=FGSG_04066, score=4, range=143-161, strand=-1
```

```
target  5’ CGAUCCCGUUUAUCAAGAA 3’
           ::::: ::.:.::::::::
query   3’ GCUAGUGCGAGUAGUUCUU 5’

query=JS16_t1391273, target=FGSG_08795, score=3, range=5970-5987, strand=1

target  5’ CGA-CAUGCUCGUCAAGAA 3’
           ::: ::.::::.:::::::
query   3’ GCUAGUGCGAGUAGUUCUU 5’

query=JS16_t1391273, target=FGSG_13783, score=3, range=12437-12455, strand=1

target  5’ CGACCUCGCUUAUCAAGAA 3’
           ::: : ::::.::::::::
query   3’ GCUAGUGCGAGUAGUUCUU 5’

query=JS16_t1391273, target=FGSG_06605, score=3, range=1632-1649, strand=1

target  5’ CGAUGA-GCUCAUCAAGAA 3’
           :::: : ::::::::::::
query   3’ GCUAGUGCGAGUAGUUCUU 5’

query=JS16_t1391273, target=FGSG_13743, score=3.5, range=324-342, strand=1

target  5’ UGAUGUCGUUCAUCAAGAA 3’
           .:::  ::.::::::::::
query   3’ GCUAGUGCGAGUAGUUCUU 5’

query=JS16_t1391273, target=FGSG_10733, score=3.5, range=219-236, strand=1

target  5’ UGUUCA-GCUCAUCAAGAA 3’
           .: ::: ::::::::::::
query   3’ GCUAGUGCGAGUAGUUCUU 5’

query=JS16_t1391273, target=FGSG_02240, score=3.5, range=1353-1372, strand=1

target  5’ CAGCCACCGCUCAUCAAGAA 3’
           : .  :: :::::::::::::
query   3’ GCUAGU-GCGAGUAGUUCUU 5’

query=JS16_t1391273, target=FGSG_05697, score=3.5, range=2120-2138, strand=-1

target  5’ GUGUUGCGCUCAUCAAGAA 3’
             .:..:::::::::::::
query   3’ GCUAGUGCGAGUAGUUCUU 5’

query=JS16_t1391273, target=FGSG_01049, score=4, range=4584-4603, strand=1

target  5’ CGAUCGACGAUCAUCAAGGA 3’
           ::::: ::: ::::::::.:
query   3’ GCUAG-UGCGAGUAGUUCUU 5’

query=JS16_t1391273, target=FGSG_06922, score=4, range=85-104, strand=1

target  5’ CGAUCAAUGCUGAUCAAGAA 3’
           ::::: :.::: ::::::::
query   3’ GCUAG-UGCGAGUAGUUCUU 5’

query=JS16_t1391273, target=FGSG_11812, score=4, range=854-872, strand=1

target  5’ CCAUUAAGCUCAUCAAGAG 3’
           : ::.: :::::::::::.
query   3’ GCUAGUGCGAGUAGUUCUU 5’

query=JS16_t1391273, target=FGSG_05933, score=4, range=560-578, strand=1

target  5’ CAACCACGUUCGUCAAGAA 3’
           : : ::::.::.:::::::
query   3’ GCUAGUGCGAGUAGUUCUU 5’

query=JS16_t1391273, target=FGSG_08436, score=4, range=1649-1667, strand=1

target  5’ AGAUCUCGCUCAUCGAGAC 3’
            :::: ::::::::.:::
query   3’ GCUAGUGCGAGUAGUUCUU 5’

query=JS16_t1391273, target=FGSG_05060, score=4, range=1556-1574, strand=1

target  5’ UGCGCAUGCUCAUCAAGAG 3’
           .:  ::.:::::::::::.
query   3’ GCUAGUGCGAGUAGUUCUU 5’

query=JS16_t1391273, target=FGSG_06492, score=4, range=537-555, strand=1

target  5’ CAAGCACGCUCGUCAAGAC 3’
           : : :::::::.::::::
query   3’ GCUAGUGCGAGUAGUUCUU 5’

query=JS16_t1391273, target=FGSG_06749, score=4, range=396-413, strand=1

target  5’ CGAU-ACCCUCAUCAAGGA 3’
```

```
target  5’ UCGUUUUCUCCAUCCACGG 3’
           : ::: ::: :::::::::
query   3’ ACCAACAGAUGUAGGUGCC 5’

query=JS16_t1327835, target=FGSG_00280, score=3.5, range=892-911, strand=1

target  5’ CUUCAGAACCACAACUCAGC 3’
           :.: :  :::::::::::::
query   3’ GGAUUAGUGGUGUUGAGUCG 5’

query=JS16_t1327835, target=FGSG_01505, score=3.5, range=457-475, strand=-1

target  5’ CCUUGUC-CCACAACUCAGC 3’
           ::: .:: ::::::::::::
query   3’ GGAUUAGUGGUGUUGAGUCG 5’

query=JS16_t1327835, target=FGSG_12505, score=4, range=325-344, strand=1

target  5’ CCUGGUGACUACAACUUAGC 3’
           :::..: ::.::::::.:::
query   3’ GGAUUAGUGGUGUUGAGUCG 5’

query=JS16_t1327835, target=FGSG_05260, score=4, range=108-126, strand=1

target  5’ CUUAG-CACCACGACUCGGC 3’
           :.::. ::::::.::::.::
query   3’ GGAUUAGUGGUGUUGAGUCG 5’

query=JS16_t1327835, target=FGSG_03004, score=4, range=1456-1476, strand=1

target  5’ CCCGAUCGACCACGACUCAGU 3’
           :: .::: :::::.::::::.
query   3’ GGAUUAG-UGGUGUUGAGUCG 5’

query=JS16_t1327835, target=FGSG_05424, score=4, range=153-173, strand=1

target  5’ ACGAAUCACCACAACUGCAGC 3’
            : ::::::::::::: ::::
query   3’ GGAUUAGUGGUGUUGA-GUCG 5’

query=JS16_t1327835, target=FGSG_04901, score=4, range=463-483, strand=1

target  5’ UCGAAUUACCACAAGCUCAGC 3’
           .: :::.:::::::: ::::::
query   3’ GGAUUAGUGGUGUU-GAGUCG 5’

query=JS16_t1327835, target=FGSG_03439, score=4, range=748-768, strand=-1

target  5’ CAUGAUUGACCACAGCUCAGC 3’
           : :.::. ::::::.::::::
query   3’ GGAUUAG-UGGUGUUGAGUCG 5’

query=JS16_t3120369, target=FGSG_10081, score=0.5, range=256-273, strand=1

target  5’ CAGAUUCGACCAGCCAAG 3’
           :::::::::::::::::.
query   3’ GUCUAAGCUGGUCGGUUU 5’

query=JS16_t3120369, target=FGSG_11614, score=2.5, range=634-651, strand=1

target  5’ UGGGAUCGACCAGCCAAA 3’
           ..:. :::::::::::::
query   3’ GUCUAAGCUGGUCGGUUU 5’

query=JS16_t3120369, target=FGSG_01790, score=2.5, range=4815-4832, strand=1

target  5’ UAUAUUCGACCAGCCAAU 3’
           .: ::::::::::::::
query   3’ GUCUAAGCUGGUCGGUUU 5’

query=JS16_t3120369, target=FGSG_12945, score=2.5, range=27-45, strand=1

target  5’ UAGUUAUCGACCAGCCAAA 3’
           .:: : :::::::::::::
query   3’ GUCUA-AGCUGGUCGGUUU 5’

query=JS16_t3120369, target=FGSG_12187, score=3, range=312-329, strand=1

target  5’ CAGAUUCGGCCCGCCAAA 3’
           ::::::::.:: ::::::
query   3’ GUCUAAGCUGGUCGGUUU 5’

query=JS16_t3120369, target=FGSG_01977, score=3, range=3146-3162, strand=1

target  5’ CA-AUUCGACAAGCCAAA 3’
           :: ::::::: :::::::
query   3’ GUCUAAGCUGGUCGGUUU 5’

query=JS16_t3120369, target=FGSG_05505, score=3, range=310-327, strand=-1

target  5’ UAGAUUCGACCAGCCUAG 3’
           :::: :: :::::::::.:
query   3’ GCUAGUGCGAGUAGUUCUU 5’

query=JS16_t1391273, target=FGSG_06397, score=4, range=399-416, strand=1

target  5’ CGA-CAAGCUCAUCAAGGA 3’
           ::: :: ::::::::::.:
query   3’ GCUAGUGCGAGUAGUUCUU 5’

query=JS16_t1391273, target=FGSG_10737, score=4, range=54-71, strand=1

target  5’ CGA-CACGCGCAUUAAGAA 3’
           ::: ::::: :::.:::::
query   3’ GCUAGUGCGAGUAGUUCUU 5’

query=JS16_t1391273, target=FGSG_12802, score=4, range=429-446, strand=1

target  5’ CGA-CAAGCUCAUCAAGGA 3’
           ::: :: ::::::::::.:
query   3’ GCUAGUGCGAGUAGUUCUU 5’

query=JS16_t1391273, target=FGSG_06958, score=4, range=525-542, strand=1

target  5’ CGG-CGAGCUCAUCAAGAA 3’
           ::. :. ::::::::::::
query   3’ GCUAGUGCGAGUAGUUCUU 5’

query=JS16_t1391273, target=FGSG_09659, score=4, range=652-669, strand=1

target  5’ CGAAC-UGCUCGUCAAGAA 3’
           ::: : .::::.:::::::
query   3’ GCUAGUGCGAGUAGUUCUU 5’

query=JS16_t1391273, target=FGSG_00926, score=4, range=444-461, strand=1

target  5’ CGA-CUUGCUUAUCAAGAA 3’
           ::: : .:::.::::::::
query   3’ GCUAGUGCGAGUAGUUCUU 5’

query=JS16_t1391273, target=FGSG_01576, score=4, range=716-733, strand=1

target  5’ AGAUC-CGAUCAUCAAGAA 3’
            :::: :: ::::::::::
query   3’ GCUAGUGCGAGUAGUUCUU 5’

query=JS16_t1391273, target=FGSG_03630, score=4, range=678-697, strand=1

target  5’ CGAUCAACGAUCAUCAAGAC 3’
           ::::: ::: :::::::::
query   3’ GCUAG-UGCGAGUAGUUCUU 5’

query=JS16_t1391273, target=FGSG_09740, score=4, range=929-947, strand=-1

target  5’ CGAUCACCUUCAUCGAGAA 3’
           ::::::: .:::::.::::
query   3’ GCUAGUGCGAGUAGUUCUU 5’

query=JS16_t1391273, target=FGSG_10999, score=4, range=71-89, strand=-1

target  5’ UCGUCACGCUCAUCGAGGA 3’
           . .:::::::::::.::.:
query   3’ GCUAGUGCGAGUAGUUCUU 5’

query=JS16_t1391273, target=FGSG_09318, score=4, range=115-134, strand=-1

target  5’ CGGUUGUGCUCAUCAACGAA 3’
           ::.:...::::::::: :::
query   3’ GCUAGUGCGAGUAGUU-CUU 5’

query=JS16_t1391273, target=FGSG_11118, score=4, range=377-395, strand=-1

target  5’ CGAUCUUGCUCAUCAAGGU 3’
           ::::: .::::::::::.
query   3’ GCUAGUGCGAGUAGUUCUU 5’

query=JS16_t1391273, target=FGSG_04022, score=4, range=878-896, strand=-1

target  5’ UUAUCGCGCUCGUCAAGAC 3’
           .  :::.:::::.::::::
query   3’ GCUAGUGCGAGUAGUUCUU 5’

query=JS16_t1391273, target=FGSG_05819, score=4, range=1280-1298, strand=-1

target  5’ UCUUUACGCUCAUCGAGAA 3’
           .   :.::::::::.::::
query   3’ GCUAGUGCGAGUAGUUCUU 5’

query=JS16_t1391273, target=FGSG_07274, score=4, range=368-386, strand=-1

target  5’ CGAUCCAGCUCAUCAGGAA 3’
           :::::  ::::::::.:::
query   3’ GCUAGUGCGAGUAGUUCUU 5’
```

```
           .:::::::::::::: :.
query   3’ GUCUAAGCUGGUCGGUUU 5’

query=JS16_t3120369, target=FGSG_09843, score=3, range=1449-1467, strand=-1

target  5’ CAGUAUUCGACCAGCCAUA 3’
           ::: ::::::::::::: :
query   3’ GUC-UAAGCUGGUCGGUUU 5’

query=JS16_t3120369, target=FGSG_04291, score=3, range=3684-3700, strand=-1

target  5’ CAGAGU-GACCAGCCAAA 3’
           :::: : :::::::::::
query   3’ GUCUAAGCUGGUCGGUUU 5’

query=JS16_t3120369, target=FGSG_01439, score=3, range=1154-1170, strand=-1

target  5’ UAGAUUC-ACCAGCCAAG 3’
           .:::::: :::::::::.
query   3’ GUCUAAGCUGGUCGGUUU 5’

query=JS16_t3120369, target=FGSG_06793, score=3.5, range=2214-2231, strand=1

target  5’ AGGAAUCGACCAGCCAGA 3’
            .:: :::::::::::.:
query   3’ GUCUAAGCUGGUCGGUUU 5’

query=JS16_t3120369, target=FGSG_03898, score=3.5, range=1090-1106, strand=1

target  5’ CAGG-UCAACCAGCCAAA 3’
           :::. :: ::::::::::
query   3’ GUCUAAGCUGGUCGGUUU 5’

query=JS16_t3120369, target=FGSG_13795, score=3.5, range=71-87, strand=1

target  5’ CA-AUUCUACCAGCCAAG 3’
           :: :::: :::::::::.
query   3’ GUCUAAGCUGGUCGGUUU 5’

query=JS16_t3120369, target=FGSG_05236, score=3.5, range=1476-1493, strand=-1

target  5’ CGGGUGCGACCAGCCAAG 3’
           :.:.: :::::::::::.
query   3’ GUCUAAGCUGGUCGGUUU 5’

query=JS16_t3120369, target=FGSG_02641, score=3.5, range=381-398, strand=-1

target  5’ AAGAAUCGGCCAGCCAAG 3’
            ::: :::.::::::::.
query   3’ GUCUAAGCUGGUCGGUUU 5’

query=JS16_t3120369, target=FGSG_09607, score=3.5, range=11559-11575, strand=

target  5’ CAG-UUUGACCAGCCAGG 3’
           ::: ::.::::::::::..
query   3’ GUCUAAGCUGGUCGGUUU 5’

query=JS16_t3120369, target=FGSG_02376, score=3.5, range=656-673, strand=-1

target  5’ CAGACUCGACCAGCAAAG 3’
           :::: ::::::::: ::.
query   3’ GUCUAAGCUGGUCGGUUU 5’

query=JS16_t3120369, target=FGSG_05706, score=3.5, range=534-550, strand=-1

target  5’ CA-AUUCGACCAGCCCAG 3’
           :: :::::::::::: :.
query   3’ GUCUAAGCUGGUCGGUUU 5’

query=JS16_t3120369, target=FGSG_11912, score=3.5, range=603-619, strand=-1

target  5’ CAG-CUCGACCAGUCAAG 3’
           :::  ::::::::.:::.
query   3’ GUCUAAGCUGGUCGGUUU 5’

query=JS16_t3120369, target=FGSG_03069, score=4, range=607-624, strand=1

target  5’ CAGAUUCCAUCAGCCGAA 3’
           ::::::: :.:::::.::
query   3’ GUCUAAGCUGGUCGGUUU 5’

query=JS16_t3120369, target=FGSG_08169, score=4, range=846-863, strand=1

target  5’ AGGGUUCAACCAGCCAAA 3’
            .:.::: ::::::::::
query   3’ GUCUAAGCUGGUCGGUUU 5’

query=JS16_t3120369, target=FGSG_10294, score=4, range=337-354, strand=1

target  5’ GGAAUUCGGCCAGCCAAG 3’
            . :::::.::::::::.
query   3’ GUCUAAGCUGGUCGGUUU 5’

query=JS16_t1391273, target=FGSG_03554, score=4, range=1163-1180, strand=-1

target  5’ UGAU-GCGCUCGUCAAGGA 3’
           .::: .:::::.:::::.:
query   3’ GCUAGUGCGAGUAGUUCUU 5’

query=JS16_t1391273, target=FGSG_09548, score=4, range=68-85, strand=-1

target  5’ UGG-CACGCUCGUUAAGAA 3’
           .:.  :::::::.:.:::::
query   3’ GCUAGUGCGAGUAGUUCUU 5’

query=JS16_t1391273, target=FGSG_01924, score=4, range=863-881, strand=-1

target  5’ UGAGCACGCUCCUCAAGAG 3’
           .:: ::::::: ::::::.
query   3’ GCUAGUGCGAGUAGUUCUU 5’

query=JS16_t1391273, target=FGSG_01790, score=4, range=329-346, strand=-1

target  5’ CGAU-ACUUUCAUCAAGAA 3’
           :::: :: .::::::::::
query   3’ GCUAGUGCGAGUAGUUCUU 5’

query=JS16_t1391273, target=FGSG_05671, score=4, range=2375-2392, strand=-1

target  5’ CGA-CACGCUCGUCAAAAA 3’
           ::: :::::::.:::: ::
query   3’ GCUAGUGCGAGUAGUUCUU 5’

query=JS16_t1391273, target=FGSG_04144, score=4, range=1343-1361, strand=-1

target  5’ CCCUCAAGCUCAUCAAGAA 3’
           :  ::: :::::::::::
query   3’ GCUAGUGCGAGUAGUUCUU 5’

query=JS16_t1391273, target=FGSG_01866, score=4, range=584-601, strand=-1

target  5’ UGA-CACGCUCCUCAAGAG 3’
           .:: ::::::: ::::::.
query   3’ GCUAGUGCGAGUAGUUCUU 5’

query=JS16_t1391273, target=FGSG_00604, score=4, range=1388-1405, strand=-1

target  5’ CGAAC-CGCUCAUCAAGGU 3’
           ::: : :::::::::::.
query   3’ GCUAGUGCGAGUAGUUCUU 5’

query=JS16_t1391273, target=FGSG_11119, score=4, range=77-94, strand=-1

target  5’ CGAGUGCGCUCA-CAAGAA 3’
           ::: ..:::::: ::::::
query   3’ GCUAGUGCGAGUAGUUCUU 5’

No results for JS16_t2287377
No results for JS16_t3183533
No results for JS16_t1391330
No results for JS16_t1391370
No results for JS16_t4080398
query=JS16_t0396131, target=FGSG_09293, score=3, range=565-585, strand=1

target  5’ UCCAGUAGUGGCGGUGGUAGC 3’
           :::::: ::::::::::: ::
query   3’ AGGUCAACACCGCCACCAACG 5’

query=JS16_t0396131, target=FGSG_01718, score=3.5, range=22-41, strand=-1

target  5’ UCCAG-UGUGGCGGUGGUCGU 3’
           ::::: :::::::::::: :.
query   3’ AGGUCAACACCGCCACCAACG 5’

query=JS16_t0396131, target=FGSG_04175, score=4, range=3013-3033, strand=1

target  5’ UCGAGUUGUGGUGGUGGUUCC 3’
           :: ::::::::.::::::: :
query   3’ AGGUCAACACCGCCACCAACG 5’

query=JS16_t0396131, target=FGSG_11166, score=4, range=211-231, strand=-1

target  5’ UGAAGUAGUGGUGGUGGUUGC 3’
           :  ::: ::::.:::::::::
query   3’ AGGUCAACACCGCCACCAACG 5’

query=JS16_t0396131, target=FGSG_09260, score=4, range=25-45, strand=-1

target  5’ UGCUGUUGUGGUGGUGGUUGA 3’
           : : :::::::.::::::::
query   3’ AGGUCAACACCGCCACCAACG 5’

query=JS16_t2287609, target=FGSG_04097, score=3.5, range=384-405, strand=1
```

```
query=JS16_t3120369, target=FGSG_07212, score=4, range=1794-1810, strand=1

target  5' UGGA-UCGACCGGCCAGA 3'
           ..:: ::::::.::::.:
query   3' GUCUAAGCUGGUCGGUUU 5'

query=JS16_t3120369, target=FGSG_01539, score=4, range=441-458, strand=1

target  5' CCGAAUUGACCAGUCAAA 3'
           : :: :.::::::.::::
query   3' GUCUAAGCUGGUCGGUUU 5'

query=JS16_t3120369, target=FGSG_08223, score=4, range=113-131, strand=1

target  5' UGGACUUCGACCAGCCAUA 3'
           ..:: :::::::::::: :
query   3' GUCU-AAGCUGGUCGGUUU 5'

query=JS16_t3120369, target=FGSG_13766, score=4, range=276-294, strand=1

target  5' CGCGUGUCGACCAGUCAAA 3'
           :. .: ::::::::.::::
query   3' GUCUA-AGCUGGUCGGUUU 5'

query=JS16_t3120369, target=FGSG_01488, score=4, range=594-612, strand=1

target  5' UAGAGGUCGGCCAGCCAAG 3'
           .:::  :::.::::::::.
query   3' GUCU-AAGCUGGUCGGUUU 5'

query=JS16_t3120369, target=FGSG_01965, score=4, range=433-450, strand=1

target  5' AAUCUUCGACCGGCCAAA 3'
            :  :::::::.::::::
query   3' GUCUAAGCUGGUCGGUUU 5'

query=JS16_t3120369, target=FGSG_05421, score=4, range=798-814, strand=1

target  5' CA-AUUUGACCAGCAAAA 3'
           :: :::.::::::: :::
query   3' GUCUAAGCUGGUCGGUUU 5'

query=JS16_t3120369, target=FGSG_09135, score=4, range=662-678, strand=1

target  5' CAG-UUCGACAAGUCAAA 3'
           ::: :::::: ::.::::
query   3' GUCUAAGCUGGUCGGUUU 5'

query=JS16_t3120369, target=FGSG_03201, score=4, range=1038-1054, strand=1

target  5' CACAUUCGACCA-UCAAA 3'
           :: ::::::::: .::::
query   3' GUCUAAGCUGGUCGGUUU 5'

query=JS16_t3120369, target=FGSG_06122, score=4, range=416-432, strand=1

target  5' CAGA-UCGAUCUGCCAAA 3'
           :::: ::::.: ::::::
query   3' GUCUAAGCUGGUCGGUUU 5'

query=JS16_t3120369, target=FGSG_03701, score=4, range=93-109, strand=1

target  5' CAGA-UCGACCGUCCAAA 3'
           :::: ::::::. :::::
query   3' GUCUAAGCUGGUCGGUUU 5'

query=JS16_t3120369, target=FGSG_10312, score=4, range=323-339, strand=1

target  5' CGGACUCGACC-GCCAAG 3'
           :.:: :::::: :::::.
query   3' GUCUAAGCUGGUCGGUUU 5'

query=JS16_t3120369, target=FGSG_13720, score=4, range=307-325, strand=1

target  5' CAGACACGGACCAGCCAAA 3'
           ::::  : :::::::::::
query   3' GUCUAAG-CUGGUCGGUUU 5'

query=JS16_t3120369, target=FGSG_10564, score=4, range=2004-2021, strand=-1

target  5' CAGAGUUGAUCAGUCAAA 3'
           :::: :.::.:::.::::
query   3' GUCUAAGCUGGUCGGUUU 5'

query=JS16_t3120369, target=FGSG_09148, score=4, range=1143-1160, strand=-1

target  5' AAGAUUCGACCAGCAAAC 3'
            ::::::::::::: ::
query   3' GUCUAAGCUGGUCGGUUU 5'

query=JS16_t3120369, target=FGSG_10795, score=4, range=945-963, strand=-1

target  5' GACCCUGAACCCUUACUCAUGG 3'
           :: : :: :::::::::::::.
query   3' CU-GAACAUGGGAAUGAGUACU 5'

query=JS16_t2287609, target=FGSG_00801, score=4, range=163-182, strand=1

target  5' AACUUGGACCCUUAC-CAUGA 3'
            ::::: :::::::: :::::
query   3' CUGAACAUGGGAAUGAGUACU 5'

query=JS16_t2287609, target=FGSG_09638, score=4, range=4379-4400, strand=-1

target  5' GUCUGGUAUCCCUUACUCAUGU 3'
           : :: ::: ::::::::::::
query   3' CUGAACAU-GGGAAUGAGUACU 5'

query=JS16_t2287609, target=FGSG_07399, score=4, range=836-855, strand=-1

target  5' AACUUGU-CCCUUGCUCAUGC 3'
            :::::: :::::.::::::
query   3' CUGAACAUGGGAAUGAGUACU 5'

No results for JS16_t2287611
No results for JS16_t3183850
No results for JS16_t4080506
query=JS16_t2287733, target=FGSG_10051, score=2.5, range=55-72, strand=-1

target  5' GGUGGUAGAGUCGUGCUU 3'
           :::::::::::::: ::.
query   3' CCACCAUCUCAGCAAGAG 5'

query=JS16_t2287733, target=FGSG_06133, score=3, range=676-693, strand=1

target  5' GGUGGUAGAGUUGUUGUC 3'
           :::::::::::.::: ::
query   3' CCACCAUCUCAGCAAGAG 5'

query=JS16_t2287733, target=FGSG_07284, score=3, range=2026-2043, strand=-1

target  5' AGUGGUAAAGUCGUUCUC 3'
            :::::: ::::::::::
query   3' CCACCAUCUCAGCAAGAG 5'

query=JS16_t2287733, target=FGSG_00044, score=3.5, range=789-806, strand=1

target  5' GGAGAUAGAGUCGUUUUU 3'
           :: : ::::::::::.:.
query   3' CCACCAUCUCAGCAAGAG 5'

query=JS16_t2287733, target=FGSG_10024, score=3.5, range=715-732, strand=-1

target  5' GGAGGCAGAGUCGUUCUU 3'
           :: :: :::::::::::.
query   3' CCACCAUCUCAGCAAGAG 5'

query=JS16_t2287733, target=FGSG_05031, score=3.5, range=199-216, strand=-1

target  5' GAUGGUAGAGUCGUUGUU 3'
           : ::::::::::::: :.
query   3' CCACCAUCUCAGCAAGAG 5'

query=JS16_t2287733, target=FGSG_00503, score=4, range=1063-1080, strand=1

target  5' GGUGGUUUAGUCGUUCUC 3'
           ::::::  ::::::::::
query   3' CCACCAUCUCAGCAAGAG 5'

query=JS16_t2287733, target=FGSG_05455, score=4, range=1782-1799, strand=1

target  5' GGAGGUAUGGUCGUUCUC 3'
           :: :::: .:::::::::
query   3' CCACCAUCUCAGCAAGAG 5'

query=JS16_t2287733, target=FGSG_03391, score=4, range=1315-1332, strand=1

target  5' CGAGGUAGAGUUGUUCUG 3'
            : ::::::::.:::::
query   3' CCACCAUCUCAGCAAGAG 5'

query=JS16_t2287733, target=FGSG_09362, score=4, range=1011-1028, strand=1

target  5' GGCUGUAGAGUCGCUCUC 3'
           ::  ::::::::: ::::
query   3' CCACCAUCUCAGCAAGAG 5'

query=JS16_t2287733, target=FGSG_07518, score=4, range=3305-3321, strand=1

target  5' GGGGGUGGAGUC-UUCUC 3'
           :: :::.::::: :::::
query   3' CCACCAUCUCAGCAAGAG 5'

query=JS16_t2287733, target=FGSG_06580, score=4, range=2098-2115, strand=-1
```

```
target  5’ CAUAUUUCGACCAGCUAGA 3’
           :: : ::::::::::.:.:
query   3’ GUCU-AAGCUGGUCGGUUU 5’

query=JS16_t3120369, target=FGSG_03177, score=4, range=1583-1601, strand=-1

target  5’ CAGAAGUUGGCCAGCCAAA 3’
           ::: : :.:.:::::::::
query   3’ GUC-UAAGCUGGUCGGUUU 5’

query=JS16_t3120369, target=FGSG_01108, score=4, range=1772-1789, strand=-1

target  5’ UGGCCUCGACCAGCCAGA 3’
           ..:  :::::::::::.:
query   3’ GUCUAAGCUGGUCGGUUU 5’

query=JS16_t3120369, target=FGSG_13720, score=4, range=106-123, strand=-1

target  5’ CAGCGUCGACCAGUCAAU 3’
           :::  ::::::::.:::
query   3’ GUCUAAGCUGGUCGGUUU 5’

query=JS16_t3120369, target=FGSG_10889, score=4, range=2547-2565, strand=-1

target  5’ GAGACGUCGACCAGCCGAA 3’
            :::  ::::::::::.::
query   3’ GUCU-AAGCUGGUCGGUUU 5’

query=JS16_t3120369, target=FGSG_03030, score=4, range=1257-1273, strand=-1

target  5’ CAGUUUC-ACCAGCCAGA 3’
           ::: ::: ::::::::.:
query   3’ GUCUAAGCUGGUCGGUUU 5’

query=JS16_t3120369, target=FGSG_00433, score=4, range=1294-1311, strand=-1

target  5’ CAUUUUCGACAAGCCAAA 3’
           ::  :::::: :::::::
query   3’ GUCUAAGCUGGUCGGUUU 5’

No results for JS16_t0394034
No results for JS16_t0283039
No results for JS16_t2224421
query=JS16_t4017391, target=FGSG_02269, score=2.5, range=288-304, strand=1

target  5’ CUCG-CUCUCACCUUGGA 3’
           :::. ::::::::::::
query   3’ GAGUGGAGAGUGGAACCA 5’

query=JS16_t4017391, target=FGSG_09341, score=3, range=325-342, strand=1

target  5’ CUCGUCUCUCGUCUUGGU 3’
           :::..:::::..::::::
query   3’ GAGUGGAGAGUGGAACCA 5’

query=JS16_t4017391, target=FGSG_03100, score=3, range=1551-1568, strand=1

target  5’ CUCAGCUCUCACCUUCGU 3’
           :::: :::::::::: ::
query   3’ GAGUGGAGAGUGGAACCA 5’

query=JS16_t4017391, target=FGSG_13334, score=3.5, range=165-182, strand=1

target  5’ CUCAUCUCUCGCCUUGUU 3’
           ::::.:::::.::::: :
query   3’ GAGUGGAGAGUGGAACCA 5’

query=JS16_t4017391, target=FGSG_02753, score=3.5, range=632-648, strand=1

target  5’ CUUA-CUCUCCCCUUGGU 3’
           ::.: ::::: :::::::
query   3’ GAGUGGAGAGUGGAACCA 5’

query=JS16_t4017391, target=FGSG_13110, score=3.5, range=642-660, strand=1

target  5’ CUUUCCUCUCGACCUUGGU 3’
           ::. :::::: ::::::::
query   3’ GAGUGGAGAG-UGGAACCA 5’

query=JS16_t4017391, target=FGSG_03608, score=3.5, range=1075-1092, strand=-1

target  5’ CUGAUCUCUCGCUUUGGU 3’
           :: :.:::::.:.:::::
query   3’ GAGUGGAGAGUGGAACCA 5’

query=JS16_t4017391, target=FGSG_08066, score=3.5, range=627-644, strand=-1

target  5’ CUUAACUUUCGCCUUGGU 3’
           ::.: ::.::.:::::::
query   3’ GAGUGGAGAGUGGAACCA 5’

target  5’ CUGGGUAGGGUCGUUCUC 3’
              :::::.:::::::::
query   3’ CCACCAUCUCAGCAAGAG 5’

query=JS16_t2287733, target=FGSG_08172, score=4, range=313-330, strand=-1

target  5’ GGUGGUAAAGUCGAUCUC 3’
           ::::::: ::::: ::::
query   3’ CCACCAUCUCAGCAAGAG 5’

query=JS16_t2287733, target=FGSG_00193, score=4, range=186-203, strand=-1

target  5’ CUUGGUAGAGUCAUUCUC 3’
             :::::::::: :::::
query   3’ CCACCAUCUCAGCAAGAG 5’

query=JS16_t2287733, target=FGSG_04951, score=4, range=955-972, strand=-1

target  5’ GAUGAUGGGGUCGUUCUC 3’
           : :: :.:.:::::::::
query   3’ CCACCAUCUCAGCAAGAG 5’

query=JS16_t2287733, target=FGSG_02950, score=4, range=1417-1434, strand=-1

target  5’ CUUGCUGGAGUCGUUCUC 3’
             :: :.:::::::::::
query   3’ CCACCAUCUCAGCAAGAG 5’

query=JS16_t2287733, target=FGSG_04186, score=4, range=2040-2056, strand=-1

target  5’ GGUGGUAGAGU-GUUUUG 3’
           ::::::::::: :::.:
query   3’ CCACCAUCUCAGCAAGAG 5’

query=JS16_t2287733, target=FGSG_05039, score=4, range=991-1008, strand=-1

target  5’ ACCCGUAGAGUCGUUCUC 3’
               ::::::::::::::
query   3’ CCACCAUCUCAGCAAGAG 5’

query=JS16_t2287733, target=FGSG_03252, score=4, range=2584-2600, strand=-1

target  5’ AGUGUUAGAGUC-UUCUC 3’
            ::: ::::::: :::::
query   3’ CCACCAUCUCAGCAAGAG 5’

query=JS16_t0182808, target=FGSG_13613, score=4, range=571-589, strand=-1

target  5’ UUGGUCGUCCAUAGGGUUC 3’
           :::: :.:: :::::::::
query   3’ AACCCGUAGAUAUCCCAAG 5’

query=JS16_t0134184, target=FGSG_07702, score=4, range=594-614, strand=1

target  5’ UUCGCUUGGU-ACCUAUUUCCA 3’
           ::: :::::: ::::::::::
query   3’ AAGGGAACCAUUGGAUAAAGGC 5’

query=JS16_t0284026, target=FGSG_07419, score=2, range=346-365, strand=1

target  5’ UUCAAGUUCAUUUUCCUCCU 3’
            :: ::::::::::::::::
query   3’ UAG-UCAAGUAAAAGGAGGA 5’

query=JS16_t0284026, target=FGSG_11231, score=3, range=355-372, strand=1

target  5’ AUCA-UUCAUUUUCCUCUG 3’
           :::: ::::::::::::.
query   3’ UAGUCAAGUAAAAGGAGGA 5’

query=JS16_t0284026, target=FGSG_11084, score=3, range=855-872, strand=1

target  5’ UUCA-AUCAUUUUCCUCCU 3’
            :::  :::::::::::::
query   3’ UAGUCAAGUAAAAGGAGGA 5’

query=JS16_t0284026, target=FGSG_13715, score=3, range=137-156, strand=-1

target  5’ AUCCAUUUCAUUUUCUUCCU 3’
           :: :: :::::::::.::::
query   3’ UA-GUCAAGUAAAAGGAGGA 5’

query=JS16_t0284026, target=FGSG_00018, score=3.5, range=905-923, strand=-1

target  5’ GGCAUCUCAUUUUCCUCCU 3’
           . ::  :::::::::::::
query   3’ UAGUCAAGUAAAAGGAGGA 5’

query=JS16_t0284026, target=FGSG_09401, score=4, range=727-746, strand=1

target  5’ AUCAGUUUAUUUGUCCUUCU 3’
```

```
query=JS16_t4017391, target=FGSG_11363, score=3.5, range=1856-1874, strand=-1

target  5’ UUCAACCUCUUACUUUGGU 3’
           .:: ::::::.::.:::::
query   3’ GAG-UGGAGAGUGGAACCA 5’

query=JS16_t4017391, target=FGSG_04233, score=3.5, range=705-723, strand=-1

target  5’ CUCACUUCUCAUCCUUGGG 3’
           :::::.::::: ::::::
query   3’ GAGUGGAGAGU-GGAACCA 5’

query=JS16_t4017391, target=FGSG_05427, score=3.5, range=1352-1370, strand=-1

target  5’ CUCAAUUCUCCACCUUGGU 3’
           :::: .::: :::::::::
query   3’ GAGUGGAGA-GUGGAACCA 5’

query=JS16_t4017391, target=FGSG_03429, score=4, range=481-498, strand=1

target  5’ CUCACCUUUGGCCUUGGU 3’
           :::::::.: .:::::::
query   3’ GAGUGGAGAGUGGAACCA 5’

query=JS16_t4017391, target=FGSG_00768, score=4, range=159-176, strand=1

target  5’ UUUAAUUCUCAUCUUGGU 3’
           .:.: .:::::.::::::
query   3’ GAGUGGAGAGUGGAACCA 5’

query=JS16_t4017391, target=FGSG_06404, score=4, range=437-454, strand=1

target  5’ UUGGCCUUUCAUCUUGGU 3’
           .: .:::.:::.::::::
query   3’ GAGUGGAGAGUGGAACCA 5’

query=JS16_t4017391, target=FGSG_12861, score=4, range=78-95, strand=1

target  5’ CUCACAUCUUACCUUGGA 3’
           ::::: :::.:::::::
query   3’ GAGUGGAGAGUGGAACCA 5’

query=JS16_t4017391, target=FGSG_09352, score=4, range=595-613, strand=1

target  5’ CUUGCUCUCUUACCUUGGG 3’
           ::..: ::::.:::::::
query   3’ GAGUG-GAGAGUGGAACCA 5’

query=JS16_t4017391, target=FGSG_01355, score=4, range=527-545, strand=1

target  5’ CUCUGUCUCUCAUCUUGGC 3’
           ::: ..::::::.:::::
query   3’ GAG-UGGAGAGUGGAACCA 5’

query=JS16_t4017391, target=FGSG_11091, score=4, range=586-602, strand=1

target  5’ CUCA-CUCUCGUCUUGGA 3’
           :::: :::::..:::::
query   3’ GAGUGGAGAGUGGAACCA 5’

query=JS16_t4017391, target=FGSG_04348, score=4, range=607-624, strand=1

target  5’ AUCAACUCUCACCUUGUU 3’
            ::: ::::::::::: :
query   3’ GAGUGGAGAGUGGAACCA 5’

query=JS16_t4017391, target=FGSG_07000, score=4, range=365-381, strand=1

target  5’ CUAACCUUUCA-CUUGGU 3’
           :: ::::.::: ::::::
query   3’ GAGUGGAGAGUGGAACCA 5’

query=JS16_t4017391, target=FGSG_03986, score=4, range=1175-1191, strand=1

target  5’ CUC-CCUCUCACCUUGAC 3’
           ::: ::::::::::::
query   3’ GAGUGGAGAGUGGAACCA 5’

query=JS16_t4017391, target=FGSG_09412, score=4, range=1418-1434, strand=1

target  5’ CUUG-CUCUCUCCUUGGU 3’
           ::.. ::::: :::::::
query   3’ GAGUGGAGAGUGGAACCA 5’

query=JS16_t4017391, target=FGSG_02077, score=4, range=325-341, strand=1

target  5’ GCCACCUC-CACCUUGGU 3’
             :::::: :::::::::
query   3’ GAGUGGAGAGUGGAACCA 5’

query=JS16_t4017391, target=FGSG_13607, score=4, range=1481-1497, strand=-1
           :::::::.:::: ::::.::
query   3’ UAGUCAAGUAAA-AGGAGGA 5’

query=JS16_t0284026, target=FGSG_05643, score=4, range=240-258, strand=1

target  5’ AUCACUUCAUAUUCCUCCA 3’
           :::: ::::: :::::::
query   3’ UAGUCAAGUAAAAGGAGGA 5’

query=JS16_t0284026, target=FGSG_06809, score=4, range=153-172, strand=1

target  5’ AUCACCUUCCUUUUCCUCCU 3’
           ::::  ::: ::::::::::
query   3’ UAGU-CAAGUAAAAGGAGGA 5’

query=JS16_t0284026, target=FGSG_04140, score=4, range=80-99, strand=1

target  5’ AGCAGGUCAUUUUCCAUCCU 3’
           : ::: ::::::::: ::::
query   3’ UAGUCAAGUAAAAGG-AGGA 5’

query=JS16_t0284026, target=FGSG_06539, score=4, range=38-56, strand=1

target  5’ AUCCUCUCAUUUUCCUCUU 3’
           :::   :::::::::::.:
query   3’ UAGUCAAGUAAAAGGAGGA 5’

query=JS16_t0284026, target=FGSG_09765, score=4, range=1841-1859, strand=1

target  5’ GACGCCUCAUUUUCCUCCU 3’
           . :.  :::::::::::::
query   3’ UAGUCAAGUAAAAGGAGGA 5’

query=JS16_t0284026, target=FGSG_05273, score=4, range=1530-1547, strand=1

target  5’ AUCCG-GCAUUUUCCUCCU 3’
           ::: :  ::::::::::::
query   3’ UAGUCAAGUAAAAGGAGGA 5’

query=JS16_t0284026, target=FGSG_01760, score=4, range=1259-1277, strand=-1

target  5’ AUCAGUUCGUUUACCUCCA 3’
           ::::::::.::: :::::
query   3’ UAGUCAAGUAAAAGGAGGA 5’

query=JS16_t0284026, target=FGSG_11660, score=4, range=236-254, strand=-1

target  5’ AUCUCUUCGUUUUCCUCCA 3’
           :::  :::.:::::::::
query   3’ UAGUCAAGUAAAAGGAGGA 5’

query=JS16_t0284026, target=FGSG_08582, score=4, range=1397-1415, strand=-1

target  5’ UUCUUUUCGUUUUCCUCCU 3’
            ::  :::.::::::::::
query   3’ UAGUCAAGUAAAAGGAGGA 5’

query=JS16_t0284026, target=FGSG_13240, score=4, range=1051-1069, strand=-1

target  5’ CUCUCUUCAUUUUCUUCCU 3’
            ::  :::::::::.::::
query   3’ UAGUCAAGUAAAAGGAGGA 5’

query=JS16_t0284026, target=FGSG_06631, score=4, range=5180-5198, strand=-1

target  5’ AUCUGGUCAUGUUCCUCCU 3’
           ::: : :::: ::::::::
query   3’ UAGUCAAGUAAAAGGAGGA 5’

query=JS16_t0284026, target=FGSG_06906, score=4, range=1322-1340, strand=-1

target  5’ AACAUUUCAUCUUCCUCCU 3’
           : :: ::::: ::::::::
query   3’ UAGUCAAGUAAAAGGAGGA 5’

No results for JS16_t2287833
No results for JS16_t2287858
query=JS16_t2287861, target=FGSG_04743, score=3.5, range=925-944, strand=-1

target  5’ UGUCCAAGAGGUGCGCCGAG 3’
           ::: :: ::.:::::::::.
query   3’ ACAAGUACUUCACGCGGCUU 5’

query=JS16_t2287861, target=FGSG_03711, score=3.5, range=244-263, strand=-1

target  5’ UAUGUAAGAAGUGCGCCGAA 3’
           : : .: ::::::::::::
query   3’ ACAAGUACUUCACGCGGCUU 5’

query=JS16_t3184063, target=FGSG_09285, score=2, range=366-388, strand=1

target  5’ ACCAUUAAUAGACUUUGAUCGAA 3’
```

```
target  5’ CUCA-CUCUUGUCUUGGU 3’
           :::: ::::...::::::
query   3’ GAGUGGAGAGUGGAACCA 5’

query=JS16_t4017391, target=FGSG_13254, score=4, range=1643-1659, strand=-1

target  5’ CUC-CUUCUCGCUUUGGU 3’
           ::: :.::::.:.:::::
query   3’ GAGUGGAGAGUGGAACCA 5’

query=JS16_t4017391, target=FGSG_05484, score=4, range=1384-1402, strand=-1

target  5’ CUCGUGUUCUCACCUUGGC 3’
           :::.. .:::::::::::
query   3’ GAGUG-GAGAGUGGAACCA 5’

query=JS16_t4017391, target=FGSG_09534, score=4, range=2332-2348, strand=-1

target  5’ CUU-UCUCUUGCCUUGGU 3’
           ::. .::::..:::::::
query   3’ GAGUGGAGAGUGGAACCA 5’

query=JS16_t4017391, target=FGSG_07835, score=4, range=698-715, strand=-1

target  5’ CCCAGCUCUCGUCUUGGU 3’
           : :: :::::..::::::
query   3’ GAGUGGAGAGUGGAACCA 5’

query=JS16_t4017391, target=FGSG_05096, score=4, range=107-125, strand=-1

target  5’ UUGGCACUCUCAUCUUGGU 3’
           .: .: ::::::.::::::
query   3’ GAGUG-GAGAGUGGAACCA 5’

query=JS16_t4017391, target=FGSG_09267, score=4, range=446-462, strand=-1

target  5’ CUCA-CUCUCAUCUUCGU 3’
           :::: ::::::.::: ::
query   3’ GAGUGGAGAGUGGAACCA 5’

query=JS16_t4017391, target=FGSG_03476, score=4, range=1102-1118, strand=-1

target  5’ CUGACUUC-CACCUUGGU 3’
           :: ::.:: :::::::::
query   3’ GAGUGGAGAGUGGAACCA 5’

query=JS16_t4017391, target=FGSG_08494, score=4, range=1139-1155, strand=-1

target  5’ UUGAUCUC-CACCUUGGU 3’
           .: :.::: :::::::::
query   3’ GAGUGGAGAGUGGAACCA 5’

query=JS16_t4017391, target=FGSG_08494, score=4, range=1190-1206, strand=-1

target  5’ UUGAUCUC-CACCUUGGU 3’
           .: :.::: :::::::::
query   3’ GAGUGGAGAGUGGAACCA 5’

query=JS16_t3120614, target=FGSG_12035, score=4, range=1907-1930, strand=-1

target  5’ GCUUCUCACGCUCCUUCUUGAGCU 3’
           :::.::::   ::::::::::::.
query   3’ CGAGGAGUAAAAGGAAGAACUCGG 5’

query=JS16_t3120614, target=FGSG_10277, score=4, range=299-322, strand=-1

target  5’ UCUCCUUCUUCUCCUUCUUGAGCU 3’
            :::::. :: ::::::::::::.
query   3’ CGAGGAGUAAAAGGAAGAACUCGG 5’

No results for JS16_t0576057
query=JS16_t2224555, target=FGSG_00184, score=4, range=646-666, strand=1

target  5’ GGUAUUACAUGGUCUACCUGG 3’
           :.:::. ::::: ::::::::
query   3’ CUAUAG-GUACCCGAUGGACC 5’

query=JS16_t2224555, target=FGSG_09129, score=4, range=2349-2369, strand=1

target  5’ AAUAUCUCCUGGGCUACCUGG 3’
            ::::: : ::::::::::::
query   3’ CUAUAG-GUACCCGAUGGACC 5’

query=JS16_t2224555, target=FGSG_11116, score=4, range=549-568, strand=1

target  5’ GAUUCCCACGGGCUACCUGG 3’
           :::  ::: :::::::::::
query   3’ CUAUAGGUACCCGAUGGACC 5’

query=JS16_t2224555, target=FGSG_08769, score=4, range=1620-1638, strand=-1

target  5’ GAAAUCAAUGGGCU-CCUGG 3’
           : :::::: ::::::::::::::
query   3’ UUGUAAUU-UCUGAAACUAGCUU 5’

query=JS16_t3184063, target=FGSG_04358, score=4, range=1297-1318, strand=1

target  5’ GUCGUUAAAGACUUUGAUCCAA 3’
           . :.::::::::::::::: ::
query   3’ UUGUAAUUUCUGAAACUAGCUU 5’

query=JS16_t2287897, target=FGSG_02898, score=3, range=791-808, strand=1

target  5’ CCAA-GUGUGGCAAGAGUA 3’
           :::: ::.::::::::::
query   3’ GGUUCCAUACCGUUCUCAC 5’

query=JS16_t2287897, target=FGSG_06580, score=3.5, range=4062-4081, strand=1

target  5’ CGAGGGUAUUGGCAAGAGUG 3’
           : :.:::: :::::::::::
query   3’ GGUUCCAU-ACCGUUCUCAC 5’

query=JS16_t2287897, target=FGSG_10756, score=4, range=2219-2237, strand=1

target  5’ CCAAGGUGUGGUAUGAGUG 3’
           :::::::.:::.: :::::
query   3’ GGUUCCAUACCGUUCUCAC 5’

query=JS16_t2287897, target=FGSG_00792, score=4, range=1820-1838, strand=1

target  5’ CCAAGGUGUUGCAAGAGUU 3’
           :::::::.: ::::::::
query   3’ GGUUCCAUACCGUUCUCAC 5’

query=JS16_t2287897, target=FGSG_07316, score=4, range=461-480, strand=1

target  5’ CCAAGGGUAUGGCAAUGGUG 3’
           :::: :::::::::: .:::
query   3’ GGUU-CCAUACCGUUCUCAC 5’

query=JS16_t2287897, target=FGSG_02342, score=4, range=236-253, strand=1

target  5’ CCAA-GUAUGGAAAGGGUG 3’
           :::: :::::: :::.:::
query   3’ GGUUCCAUACCGUUCUCAC 5’

query=JS16_t2287897, target=FGSG_07049, score=4, range=1457-1474, strand=1

target  5’ CCAA-GUAUGGCAAGAUUC 3’
           :::: ::::::::::: :
query   3’ GGUUCCAUACCGUUCUCAC 5’

query=JS16_t2287897, target=FGSG_00986, score=4, range=1263-1282, strand=1

target  5’ GCAACGUUAUGGCAAGAGUC 3’
            ::: : ::::::::::::
query   3’ GGUU-CCAUACCGUUCUCAC 5’

query=JS16_t2287897, target=FGSG_01748, score=4, range=1055-1072, strand=1

target  5’ CCAAGG-AUGGCAAGACUG 3’
           :::::: ::::::::: ::
query   3’ GGUUCCAUACCGUUCUCAC 5’

query=JS16_t2287897, target=FGSG_07569, score=4, range=1988-2005, strand=1

target  5’ CCAAGAU-UGGUAAGAGUG 3’
           ::::: : :::.:::::::
query   3’ GGUUCCAUACCGUUCUCAC 5’

query=JS16_t2287897, target=FGSG_04252, score=4, range=372-389, strand=1

target  5’ UCGAAGUAUGG-AAGAGUG 3’
           .:.: :::::: :::::::
query   3’ GGUUCCAUACCGUUCUCAC 5’

query=JS16_t2287897, target=FGSG_07338, score=4, range=1600-1618, strand=-1

target  5’ ACAAGGUAUGGCGAGAGAG 3’
            :::::::::::.:::: :
query   3’ GGUUCCAUACCGUUCUCAC 5’

query=JS16_t2287897, target=FGSG_09238, score=4, range=86-104, strand=-1

target  5’ CCCUGGUUUGGCAAGAGUG 3’
           ::  ::: :::::::::::
query   3’ GGUUCCAUACCGUUCUCAC 5’

query=JS16_t2287897, target=FGSG_03810, score=4, range=221-240, strand=-1

target  5’ CGAAGUUUGUGGCAAGAGUG 3’
           : :::  :.:::::::::::
query   3’ GGUUC-CAUACCGUUCUCAC 5’
```

```
           :: ::: ::::::: :::::
query   3’ CUAUAGGUACCCGAUGGACC 5’

query=JS16_t1328147, target=FGSG_06477, score=4, range=308-328, strand=1

target  5’ CUGCACACCAGCGCUAUACUG 3’
            ::: :  :::::::::::::
query   3’ AACGAGGUGUCGCGAUAUGAC 5’

No results for JS16_t3120658
No results for JS16_t3120685
No results for JS16_t3120686
No results for JS16_t1328196
No results for JS16_t0576068
query=JS16_t2224653, target=FGSG_13276, score=2.5, range=692-710, strand=1

target  5’ GCGGCAUGAACCAAGGUGG 3’
           :  :::::::::::::::.
query   3’ CAGCGUACUUGGUUCCACU 5’

query=JS16_t2224653, target=FGSG_09660, score=3, range=191-209, strand=1

target  5’ GCCGUGUGAACCAAGGUGU 3’
           : ::..::::::::::::
query   3’ CAGCGUACUUGGUUCCACU 5’

query=JS16_t2224653, target=FGSG_13587, score=3.5, range=1122-1140, strand=1

target  5’ CUCGCUUGAACCAGGGUGG 3’
            :::: :::::::.::::.
query   3’ CAGCGUACUUGGUUCCACU 5’

query=JS16_t2224653, target=FGSG_02974, score=3.5, range=2326-2344, strand=1

target  5’ GCUGCCUGGACCAAGGUGA 3’
           : .:: ::.::::::::::
query   3’ CAGCGUACUUGGUUCCACU 5’

query=JS16_t2224653, target=FGSG_10925, score=4, range=302-320, strand=1

target  5’ GUUACAAGAACCAAGGUGG 3’
           ::. :: :::::::::::.
query   3’ CAGCGUACUUGGUUCCACU 5’

query=JS16_t2224653, target=FGSG_09807, score=4, range=1359-1376, strand=1

target  5’ AUCAU-UGAACCAAGGUGG 3’
            :: . ::::::::::::.
query   3’ CAGCGUACUUGGUUCCACU 5’

query=JS16_t2224653, target=FGSG_08975, score=4, range=84-102, strand=-1

target  5’ GUUGCUUGAACCGGGGUGG 3’
           ::.:: ::::::..::::.
query   3’ CAGCGUACUUGGUUCCACU 5’

query=JS16_t2224653, target=FGSG_05080, score=4, range=676-695, strand=-1

target  5’ GUCGUGAUGAUCCAAGGUGG 3’
           ::::. :::: ::::::::.
query   3’ CAGCG-UACUUGGUUCCACU 5’

query=JS16_t2224653, target=FGSG_04666, score=4, range=1463-1480, strand=-1

target  5’ GUCG-AUGAACCAGGAUGA 3’
           :::: ::::::::.: :::
query   3’ CAGCGUACUUGGUUCCACU 5’

query=JS16_t2224653, target=FGSG_02191, score=4, range=1238-1255, strand=-1

target  5’ UGCGCAUGAACCAAGG-GA 3’
             :::::::::::::: ::
query   3’ CAGCGUACUUGGUUCCACU 5’

query=JS16_t2224653, target=FGSG_01432, score=4, range=1407-1424, strand=-1

target  5’ GUUGGGUGAACCAA-GUGA 3’
           ::.: .:::::::: ::::
query   3’ CAGCGUACUUGGUUCCACU 5’

query=JS16_t2224653, target=FGSG_00627, score=4, range=1967-1984, strand=-1

target  5’ GGUG-GUGAACCAAGGUGC 3’
           : .: .::::::::::::
query   3’ CAGCGUACUUGGUUCCACU 5’

query=JS16_t2224653, target=FGSG_02395, score=4, range=4217-4234, strand=-1

target  5’ GUC-CUUGAAGCAAGGUGA 3’
           ::: : :::: ::::::::
query   3’ CAGCGUACUUGGUUCCACU 5’

query=JS16_t2287897, target=FGSG_02908, score=4, range=675-692, strand=-1

target  5’ CCAAGGU-CGGCAAGAGUG 3’
           :::::::  ::::::::::
query   3’ GGUUCCAUACCGUUCUCAC 5’

No results for JS16_t1391790
No results for JS16_t1391795
query=JS16_t1391815, target=FGSG_01123, score=3.5, range=775-795, strand=1

target  5’ GAC-GUCAAGGAGGAAGAUGAG 3’
            :: ::::::::::::.::::.
query   3’ GUGUCAGUUCCUCCUUUUACUU 5’

query=JS16_t1391815, target=FGSG_11084, score=3.5, range=859-880, strand=-1

target  5’ UGUUGUCAAGGAGGAAAAUGAU 3’
           ... ::::::::::::::::
query   3’ GUGUCAGUUCCUCCUUUUACUU 5’

query=JS16_t1391815, target=FGSG_10242, score=4, range=192-213, strand=1

target  5’ CAAAGACAAGGAGGAGGAUGAA 3’
           :: :: :::::::::..:::::
query   3’ GUGUCAGUUCCUCCUUUUACUU 5’

query=JS16_t1391815, target=FGSG_05297, score=4, range=1381-1401, strand=1

target  5’ CGCAGU-GAGGAGGAAGAUGAC 3’
           :.:::: .::::::::.::::
query   3’ GUGUCAGUUCCUCCUUUUACUU 5’

query=JS16_t1391815, target=FGSG_07419, score=4, range=352-374, strand=-1

target  5’ GAGAGCCAGAGGAGGAAAAUGAA 3’
            : :: :: ::::::::::::::
query   3’ GUGUCAGU-UCCUCCUUUUACUU 5’

No results for JS16_t2287937
query=JS16_t1391847, target=FGSG_05751, score=4, range=3804-3825, strand=-1

target  5’ CGU-UGACUCCUCCUCAAACCAC 3’
           :.: :.::: ::::::::::::
query   3’ GUAUAUUGAUGAGGAGUUUGGUC 5’

query=JS16_t0337330, target=FGSG_08900, score=4, range=281-301, strand=1

target  5’ AAGCU-CUCGUCAACGACGGUG 3’
           ::.:: :::::: :::::::::.
query   3’ UUUGAUGAGCAGAUGCUGCCAU 5’

query=JS16_t2287982, target=FGSG_08121, score=4, range=1447-1467, strand=1

target  5’ GGGCCGCGCUGUUGGGAGAGC 3’
           ..: ::: :::::::.:::::
query   3’ UUCAGCGGGACAACCUUCUCG 5’

query=JS16_t2287982, target=FGSG_01644, score=4, range=375-394, strand=1

target  5’ AAGU-GCCCUGUUCGAAGAGG 3’
           :::: :::::::: ::::::
query   3’ UUCAGCGGGACAACCUUCUCG 5’

query=JS16_t2287982, target=FGSG_10855, score=4, range=768-787, strand=-1

target  5’ GGGUAG-CCUGUUGGAAGGGC 3’
           ..:: : :::::::::::.::
query   3’ UUCAGCGGGACAACCUUCUCG 5’

query=JS16_t2287982, target=FGSG_09646, score=4, range=1037-1057, strand=-1

target  5’ GUGUCGAACUGUUGGAAGAGU 3’
           . ::::  ::::::::::::.
query   3’ UUCAGCGGGACAACCUUCUCG 5’

query=JS16_t3184168, target=FGSG_08934, score=4, range=1189-1212, strand=1

target  5’ UUCGACGAAGAUCUCCGCGAUCUC 3’
           .::: :.:: :.::::::::::::
query   3’ GAGCGGUUUGUGGAGGCGCUAGAG 5’

query=JS16_t3184168, target=FGSG_02130, score=4, range=6328-6351, strand=-1

target  5’ CUUGACAAACGCCUUUGCGAUCUC 3’
           ::.: :::::.:::..::::::::
query   3’ GAGCGGUUUGUGGAGGCGCUAGAG 5’

query=JS16_t2287997, target=FGSG_06951, score=2, range=156-173, strand=1

target  5’ GCAUCUUGACGAGCUCGC 3’
           ::: :::::::::::::
```

```
query=JS16_t2224653, target=FGSG_00082, score=4, range=1341-1360, strand=-1

target  5' GAUGCGUGACACCAAGGUGA 3'
           : .::.::: ::::::::::
query   3' CAGCGUACU-UGGUUCCACU 5'

query=JS16_t3120759, target=FGSG_00003, score=4, range=280-300, strand=-1

target  5' AUC-UGGCCCGUUGGUCGUCAU 3'
           ::: : :: ::::::::.::::
query   3' UAGCAACGUGCAACCAGUAGUA 5'

query=JS16_t3120759, target=FGSG_00376, score=4, range=445-466, strand=-1

target  5' AACACUGCUCGUUGGUCAUCAU 3'
           : :  ::: :::::::::::::
query   3' UAGCAACGUGCAACCAGUAGUA 5'

query=JS16_t0305442, target=FGSG_10464, score=4, range=4285-4304, strand=-1

target  5' AAACCAAUGGCGUGUGUGUA 3'
           ::: :::::.::::::.::
query   3' UUUAGUUACUGCACACGCAC 5'

No results for JS16_t0794046
query=JS16_t1328303, target=FGSG_00715, score=4, range=361-381, strand=-1

target  5' GAAUUGUC-CCUCUCGUAGUAG 3'
           .: :.::: ::::::::::::
query   3' UUAAGCAGUGGAGAGCAUCAUG 5'

No results for JS16_t0364726
query=JS16_t0268604, target=FGSG_11119, score=3, range=794-812, strand=1

target  5' UUGGACCGAGCGACUGGCA 3'
           .:.:::: :::::::::::
query   3' GAUCUGGAUCGCUGACCGU 5'

query=JS16_t0268604, target=FGSG_10648, score=3, range=2563-2581, strand=-1

target  5' CUAGACCUAGUGACUUGCA 3'
           ::::::::::.:::: :::
query   3' GAUCUGGAUCGCUGACCGU 5'

query=JS16_t0268604, target=FGSG_01817, score=3.5, range=473-490, strand=1

target  5' CUGGGUC-AGCGACUGGCA 3'
           ::.:..: :::::::::::
query   3' GAUCUGGAUCGCUGACCGU 5'

query=JS16_t0268604, target=FGSG_10424, score=3.5, range=1023-1040, strand=-1

target  5' CUGGACCUGGCG-CUGGCA 3'
           ::.:::::.::: ::::::
query   3' GAUCUGGAUCGCUGACCGU 5'

query=JS16_t0268604, target=FGSG_06284, score=4, range=3110-3127, strand=1

target  5' CU-GGUUUAGCGACUGGCC 3'
           :: :...:::::::::::
query   3' GAUCUGGAUCGCUGACCGU 5'

query=JS16_t0268604, target=FGSG_04299, score=4, range=2534-2553, strand=1

target  5' CUAGACCUAUGCCACUGGCA 3'
           ::::::::: :: :::::::
query   3' GAUCUGGAU-CGCUGACCGU 5'

query=JS16_t0268604, target=FGSG_01480, score=4, range=213-231, strand=-1

target  5' CAUGACCUGGCGACUGGCC 3'
           :  :::::.:::::::::
query   3' GAUCUGGAUCGCUGACCGU 5'

No results for JS16_t0240227
No results for JS16_t1328360
No results for JS16_t3120905
No results for JS16_t0576086
No results for JS16_t1328404
query=JS16_t0240228, target=FGSG_09233, score=3, range=2277-2297, strand=1

target  5' AGCUCAGGGAGAUGAUGAGCU 3'
           :::: :::::::: :::::::
query   3' UCGACUCCCUCUAGUACUCGA 5'

query=JS16_t0240228, target=FGSG_10155, score=4, range=200-219, strand=-1

target  5' AGC-GAGUGAGAUCAUGUGCU 3'
           ::: ::: ::::::::: :::
query   3' UCGACUCCCUCUAGUACUCGA 5'

query=JS16_t2224883, target=FGSG_03517, score=3, range=383-401, strand=1
query   3' CGUUGAACUGCUCGAGCC 5'

query=JS16_t2287997, target=FGSG_09492, score=2, range=1551-1568, strand=-1

target  5' GCGCUUUGACGAGCUCGG 3'
           ::. .:::::::::::::
query   3' CGUUGAACUGCUCGAGCC 5'

query=JS16_t2287997, target=FGSG_01976, score=2.5, range=1728-1745, strand=1

target  5' GCGACUUGGCGAGCUCGU 3'
           ::.:::::.::::::::
query   3' CGUUGAACUGCUCGAGCC 5'

query=JS16_t2287997, target=FGSG_07960, score=2.5, range=980-998, strand=1

target  5' GCAGCUUUGGCGAGCUCGG 3'
           :::.: :::.:::::::::
query   3' CGUUG-AACUGCUCGAGCC 5'

query=JS16_t2287997, target=FGSG_05435, score=2.5, range=1140-1157, strand=1

target  5' CCAGGUUGACGAGCUCGG 3'
            ::. :::::::::::::
query   3' CGUUGAACUGCUCGAGCC 5'

query=JS16_t2287997, target=FGSG_01061, score=2.5, range=32-48, strand=1

target  5' GUAACUUGACGAGC-CGG 3'
           :.:::::::::::: :::
query   3' CGUUGAACUGCUCGAGCC 5'

query=JS16_t2287997, target=FGSG_06133, score=2.5, range=722-739, strand=-1

target  5' CCAGCUUGACGAGCUUGG 3'
            ::.:::::::::::.::
query   3' CGUUGAACUGCUCGAGCC 5'

query=JS16_t2287997, target=FGSG_01900, score=2.5, range=1307-1325, strand=-1

target  5' GCAGCCUUGACGAGUUCGG 3'
           :::. :::::::::.::::
query   3' CGUU-GAACUGCUCGAGCC 5'

query=JS16_t2287997, target=FGSG_08828, score=3, range=363-380, strand=1

target  5' UCAACUUGGCGAGCUUGG 3'
            :::::::.::::::.::
query   3' CGUUGAACUGCUCGAGCC 5'

query=JS16_t2287997, target=FGSG_10132, score=3, range=614-632, strand=1

target  5' GCGAACUUGACGAGCUUGC 3'
           :: ::::::::::::::.:
query   3' CG-UUGAACUGCUCGAGCC 5'

query=JS16_t2287997, target=FGSG_07084, score=3, range=4085-4102, strand=-1

target  5' GGAACUUGACGAGGUCGG 3'
           : ::::::::::: ::::
query   3' CGUUGAACUGCUCGAGCC 5'

query=JS16_t2287997, target=FGSG_08489, score=3, range=386-403, strand=-1

target  5' GAAUCUUGACGAGCUCGA 3'
           : : :::::::::::::
query   3' CGUUGAACUGCUCGAGCC 5'

query=JS16_t2287997, target=FGSG_05321, score=3.5, range=603-620, strand=1

target  5' GCCGCUUGAUGAGCUUGG 3'
           :: .:::::.:::::.::
query   3' CGUUGAACUGCUCGAGCC 5'

query=JS16_t2287997, target=FGSG_00483, score=3.5, range=1076-1094, strand=1

target  5' GCGACUUUGACGAGUUCGC 3'
           ::.:: ::::::::.:::
query   3' CGUUG-AACUGCUCGAGCC 5'

query=JS16_t2287997, target=FGSG_02421, score=3.5, range=537-554, strand=1

target  5' UAAAUUUGACGAGCUCGA 3'
             ::.::::::::::::
query   3' CGUUGAACUGCUCGAGCC 5'

query=JS16_t2287997, target=FGSG_07812, score=3.5, range=1429-1445, strand=1

target  5' GU-GUUUGACGAGCUUGG 3'
           :. ..::::::::::.::
query   3' CGUUGAACUGCUCGAGCC 5'
```

```
target  5’ GCGUUGAGUAUCUGGAUCU 3’
           : ::: :::::::::::
query   3’ CACAAAUCAUAGACCUAGC 5’

query=JS16_t2224883, target=FGSG_10661, score=4, range=985-1003, strand=1

target  5’ GUGAUUGGUAUCUGGCUCG 3’
           ::: ::.:::::::: :::
query   3’ CACAAAUCAUAGACCUAGC 5’

query=JS16_t2224883, target=FGSG_04919, score=4, range=2632-2650, strand=1

target  5’ GUCUAUGGUGUCUGGAUCG 3’
           :: : :.::.:::::::::
query   3’ CACAAAUCAUAGACCUAGC 5’

query=JS16_t2224883, target=FGSG_12926, score=4, range=342-361, strand=1

target  5’ AUGUCUUAGUAUCUGCAUCG 3’
            ::: :::::::::: ::::
query   3’ CACA-AAUCAUAGACCUAGC 5’

query=JS16_t2224883, target=FGSG_05477, score=4, range=2507-2525, strand=1

target  5’ UGGUGGAGUAUCUGGAUCG 3’
             ::  :::::::::::::
query   3’ CACAAAUCAUAGACCUAGC 5’

query=JS16_t2224883, target=FGSG_10014, score=4, range=320-338, strand=-1

target  5’ AUAUUUAGUAUCUGGGUUG 3’
            : ::::::::::::.:.:
query   3’ CACAAAUCAUAGACCUAGC 5’

query=JS16_t2224883, target=FGSG_03066, score=4, range=236-254, strand=-1

target  5’ GUGUUGAGUGUUUGGAUCA 3’
           ::::: :::.:.::::::
query   3’ CACAAAUCAUAGACCUAGC 5’

query=JS16_t2224883, target=FGSG_04859, score=4, range=3309-3327, strand=-1

target  5’ GACUUUAGUAUCUGGAUUC 3’
           :  ::::::::::::::.
query   3’ CACAAAUCAUAGACCUAGC 5’

query=JS16_t2224883, target=FGSG_12118, score=4, range=7710-7727, strand=-1

target  5’ AUGAUU-GUAUCUGGAUCG 3’
            :: :: :::::::::::
query   3’ CACAAAUCAUAGACCUAGC 5’

No results for JS16_t3120984
No results for JS16_t3120987
query=JS16_t4017771, target=FGSG_06238, score=4, range=2814-2834, strand=1

target  5’ UCUCUGUCUCUUUGGUUGCGC 3’
           : : :: ::::.:::::::::
query   3’ ACAAACCGAGAGACCAACGCG 5’

query=JS16_t4017771, target=FGSG_05855, score=4, range=830-850, strand=-1

target  5’ UUUUAGGCUCGCUGGUUGCGC 3’
           : :: ::::: ::::::::::
query   3’ ACAAACCGAGAGACCAACGCG 5’

No results for JS16_t2224910
query=JS16_t0254413, target=FGSG_05794, score=3, range=4819-4836, strand=1

target  5’ ACACAAAUGAUUGGCGAG 3’
            ::: ::::::.::::::
query   3’ CGUGAUUACUAGCCGCUC 5’

query=JS16_t0254413, target=FGSG_06792, score=3.5, range=433-450, strand=1

target  5’ GCGCCAAUGAUUGGUGAG 3’
           ::.: ::::::.::.:::
query   3’ CGUGAUUACUAGCCGCUC 5’

query=JS16_t0254413, target=FGSG_06506, score=3.5, range=353-369, strand=1

target  5’ AUACU-AUGAUCGGCGAG 3’
            .::: :::::::::::
query   3’ CGUGAUUACUAGCCGCUC 5’

query=JS16_t0254413, target=FGSG_10290, score=3.5, range=353-370, strand=-1

target  5’ GCAUUAAUGGUCGGUGAU 3’
           :::.:::::.::::.::
query   3’ CGUGAUUACUAGCCGCUC 5’

query=JS16_t2287997, target=FGSG_00846, score=3.5, range=1934-1951, strand=-1

target  5’ GCCGCUUGAUGAGCUUGG 3’
           :: .:::::.:::::.::
query   3’ CGUUGAACUGCUCGAGCC 5’

query=JS16_t2287997, target=FGSG_01210, score=3.5, range=6137-6154, strand=-1

target  5’ GCCGCUUGACGAGCUUGU 3’
           :: .:::::::::::.:
query   3’ CGUUGAACUGCUCGAGCC 5’

query=JS16_t2287997, target=FGSG_10307, score=3.5, range=527-544, strand=-1

target  5’ GCAUUUUGACGAGCUUGA 3’
           ::: .::::::::::.:
query   3’ CGUUGAACUGCUCGAGCC 5’

query=JS16_t2287997, target=FGSG_08388, score=3.5, range=65-83, strand=-1

target  5’ ACGACCUUGAUGAGCUCGG 3’
            :.: :::::.::::::::
query   3’ CGUU-GAACUGCUCGAGCC 5’

query=JS16_t2287997, target=FGSG_07963, score=3.5, range=389-407, strand=-1

target  5’ CUACACUUGAUGAGCUCGG 3’
            .: ::::::.::::::::
query   3’ CGU-UGAACUGCUCGAGCC 5’

query=JS16_t2287997, target=FGSG_02359, score=3.5, range=614-631, strand=-1

target  5’ GCAGGUUGACGAGCUCAG 3’
           :::.  ::::::::::: :
query   3’ CGUUGAACUGCUCGAGCC 5’

query=JS16_t2287997, target=FGSG_01372, score=3.5, range=2387-2405, strand=-1

target  5’ GCUGCUUGACGAGGCUCGG 3’
           :: .:::::::: ::::::
query   3’ CGUUGAACUGCU-CGAGCC 5’

query=JS16_t2287997, target=FGSG_00983, score=3.5, range=1267-1285, strand=-1

target  5’ GCGAGCUUGACGAGCUAGG 3’
           :: :.:::::::::: ::
query   3’ CG-UUGAACUGCUCGAGCC 5’

query=JS16_t2287997, target=FGSG_05546, score=3.5, range=215-232, strand=-1

target  5’ GAGUCUUGAUGAGCUCGG 3’
           : .  :::::.::::::::
query   3’ CGUUGAACUGCUCGAGCC 5’

query=JS16_t2287997, target=FGSG_06836, score=3.5, range=1328-1346, strand=-1

target  5’ GUACCCUUGACGAGCUUGG 3’
           :.:  :::::::::::.::
query   3’ CGU-UGAACUGCUCGAGCC 5’

query=JS16_t2287997, target=FGSG_09509, score=3.5, range=2471-2487, strand=-1

target  5’ GU-GUUUGACGAGCUCGU 3’
           :. ..::::::::::::
query   3’ CGUUGAACUGCUCGAGCC 5’

query=JS16_t2287997, target=FGSG_01866, score=3.5, range=104-120, strand=-1

target  5’ CUGG-UUGACGAGCUCGG 3’
            ...  :::::::::::::
query   3’ CGUUGAACUGCUCGAGCC 5’

query=JS16_t2287997, target=FGSG_06222, score=3.5, range=104-122, strand=-1

target  5’ GCAGCAUGACGAUGCUCGG 3’
           :::.: :::::: ::::::
query   3’ CGUUGAACUGCU-CGAGCC 5’

query=JS16_t2287997, target=FGSG_10035, score=3.5, range=281-297, strand=-1

target  5’ GCGACUUGA-GAGUUCGG 3’
           ::.:::::: :::.::::
query   3’ CGUUGAACUGCUCGAGCC 5’

query=JS16_t2287997, target=FGSG_07053, score=4, range=470-487, strand=1

target  5’ GUAAUUUCACGAGCUUGG 3’
           :.::.:: :::::::.::
query   3’ CGUUGAACUGCUCGAGCC 5’

query=JS16_t2287997, target=FGSG_09768, score=4, range=384-401, strand=1
```

```
query=JS16_t0254413, target=FGSG_09854, score=3.5, range=890-907, strand=-1

target  5’ GCCUUGAUGAUCGGCGAC 3’
           :: .:.:::::::::::
query   3’ CGUGAUUACUAGCCGCUC 5’

query=JS16_t0254413, target=FGSG_02192, score=3.5, range=574-591, strand=-1

target  5’ GUAAAAGUGAUCGGCGAG 3’
           :.:  :.:::::::::::
query   3’ CGUGAUUACUAGCCGCUC 5’

query=JS16_t0254413, target=FGSG_10578, score=3.5, range=663-681, strand=-1

target  5’ GUGACGAAUGAUUGGCGAG 3’
           :. :: ::::::.::::::
query   3’ CG-UGAUUACUAGCCGCUC 5’

query=JS16_t0254413, target=FGSG_00714, score=3.5, range=1076-1094, strand=-1

target  5’ GCGGUCAAUGAUCGGCGAA 3’
           ::. : ::::::::::::
query   3’ CGUGA-UUACUAGCCGCUC 5’

query=JS16_t0254413, target=FGSG_04031, score=4, range=1355-1372, strand=1

target  5’ GCACUGAUGGUCUGCGAG 3’
           :::::.:::.:: :::::
query   3’ CGUGAUUACUAGCCGCUC 5’

query=JS16_t0254413, target=FGSG_10283, score=4, range=3763-3780, strand=1

target  5’ GCACGGAUGAUUGGCGAU 3’
           :::: .:::::.:::::
query   3’ CGUGAUUACUAGCCGCUC 5’

query=JS16_t0254413, target=FGSG_12012, score=4, range=968-984, strand=1

target  5’ GUAUUAAUGAUU-GCGAG 3’
           :.:.:::::::. :::::
query   3’ CGUGAUUACUAGCCGCUC 5’

query=JS16_t0254413, target=FGSG_03530, score=4, range=335-351, strand=1

target  5’ CCACU-AUGAUCGGUGAG 3’
            :::: ::::::::.:::
query   3’ CGUGAUUACUAGCCGCUC 5’

query=JS16_t0254413, target=FGSG_06948, score=4, range=1760-1776, strand=1

target  5’ GCA-UAAAGAUUGGCGAG 3’
           ::: ::: :::.::::::
query   3’ CGUGAUUACUAGCCGCUC 5’

query=JS16_t0254413, target=FGSG_08559, score=4, range=347-363, strand=1

target  5’ GC-CUAAAGGUCGGCGAG 3’
           :: :::: :.::::::::
query   3’ CGUGAUUACUAGCCGCUC 5’

query=JS16_t0254413, target=FGSG_10876, score=4, range=350-366, strand=1

target  5’ GCAC-AGUGACCGGCGAG 3’
           :::: :.::: :::::::
query   3’ CGUGAUUACUAGCCGCUC 5’

query=JS16_t0254413, target=FGSG_03919, score=4, range=1722-1738, strand=1

target  5’ GC-CGAAUGGUUGGCGAG 3’
           :: : ::::.:.::::::
query   3’ CGUGAUUACUAGCCGCUC 5’

query=JS16_t0254413, target=FGSG_01198, score=4, range=166-183, strand=1

target  5’ GGACAAAUUAUCGGCGAG 3’
           : :: ::: :::::::::
query   3’ CGUGAUUACUAGCCGCUC 5’

query=JS16_t0254413, target=FGSG_00914, score=4, range=27-43, strand=1

target  5’ GC-CAAAUGAUCGUCGAG 3’
           :: : :::::::: ::::
query   3’ CGUGAUUACUAGCCGCUC 5’

query=JS16_t0254413, target=FGSG_05831, score=4, range=676-693, strand=-1

target  5’ UUGGUGAUGAUCGGCGAG 3’
            ..  :.:::::::::::
query   3’ CGUGAUUACUAGCCGCUC 5’

query=JS16_t0254413, target=FGSG_00903, score=4, range=886-903, strand=-1

target  5’ GCAGUUUGACCAGUUCGG 3’
           :::..::::: ::.::::
query   3’ CGUUGAACUGCUCGAGCC 5’

query=JS16_t2287997, target=FGSG_13519, score=4, range=1894-1911, strand=1

target  5’ GCAGUUUUACGAGCUUGG 3’
           :::..:: :::::::.::
query   3’ CGUUGAACUGCUCGAGCC 5’

query=JS16_t2287997, target=FGSG_13166, score=4, range=777-794, strand=1

target  5’ GCAGUUUGAUGAGCUCUG 3’
           :::..::::.:::::: :
query   3’ CGUUGAACUGCUCGAGCC 5’

query=JS16_t2287997, target=FGSG_02587, score=4, range=905-922, strand=1

target  5’ ACAACUUGACGAGUCCGG 3’
            ::::::::::::. :::
query   3’ CGUUGAACUGCUCGAGCC 5’

query=JS16_t2287997, target=FGSG_08098, score=4, range=1193-1210, strand=1

target  5’ GUGACUUGACGAGCUGGA 3’
           :..:::::::::::: :
query   3’ CGUUGAACUGCUCGAGCC 5’

query=JS16_t2287997, target=FGSG_05038, score=4, range=429-446, strand=1

target  5’ GGAGUUUGGCGAGCUCGA 3’
           : :..:::.::::::::
query   3’ CGUUGAACUGCUCGAGCC 5’

query=JS16_t2287997, target=FGSG_10819, score=4, range=2486-2504, strand=1

target  5’ GUGACUUUGACGGGCUCGA 3’
           :..:: ::::::.:::::
query   3’ CGUUG-AACUGCUCGAGCC 5’

query=JS16_t2287997, target=FGSG_00732, score=4, range=984-1001, strand=1

target  5’ GGGCUUUGACGAGCUUGG 3’
           : . .::::::::::.::
query   3’ CGUUGAACUGCUCGAGCC 5’

query=JS16_t2287997, target=FGSG_04343, score=4, range=907-923, strand=1

target  5’ GC-ACUUGAUGAGCUUGA 3’
           :: ::::::.:::::.:
query   3’ CGUUGAACUGCUCGAGCC 5’

query=JS16_t2287997, target=FGSG_12240, score=4, range=486-503, strand=1

target  5’ GCUUCUUGAUGAGCUCGA 3’
           ::  :::::.:::::::
query   3’ CGUUGAACUGCUCGAGCC 5’

query=JS16_t2287997, target=FGSG_08471, score=4, range=1137-1154, strand=1

target  5’ UGAAAUUGACGAGCUCGA 3’
             :: ::::::::::::
query   3’ CGUUGAACUGCUCGAGCC 5’

query=JS16_t2287997, target=FGSG_02504, score=4, range=75-92, strand=1

target  5’ UCACAUUGACGAGCUCGC 3’
            ::  ::::::::::::
query   3’ CGUUGAACUGCUCGAGCC 5’

query=JS16_t2287997, target=FGSG_00846, score=4, range=1048-1064, strand=1

target  5’ GCUA-UUGAUGAGCUCGA 3’
           :: : ::::.::::::::
query   3’ CGUUGAACUGCUCGAGCC 5’

query=JS16_t2287997, target=FGSG_11916, score=4, range=146-162, strand=1

target  5’ GCGAUUUGAC-AGCUCGA 3’
           ::.:.::::: ::::::
query   3’ CGUUGAACUGCUCGAGCC 5’

query=JS16_t2287997, target=FGSG_07245, score=4, range=1700-1718, strand=-1

target  5’ GCAACUUGAUGAGCAUUGG 3’
           :::::::::.:::: :.::
query   3’ CGUUGAACUGCUCG-AGCC 5’

query=JS16_t2287997, target=FGSG_06174, score=4, range=494-511, strand=-1

target  5’ GUAAUUCGACGAGUUCGG 3’
           :.::.: ::::::.::::
```

```
target  5’ GCCCUGAGGAUCGGCGAG 3’
           :: ::.: ::::::::::
query   3’ CGUGAUUACUAGCCGCUC 5’

query=JS16_t0254413, target=FGSG_04182, score=4, range=337-354, strand=-1

target  5’ ACACCAAUGACCGGCGAG 3’
            ::: ::::: :::::::
query   3’ CGUGAUUACUAGCCGCUC 5’

query=JS16_t0254413, target=FGSG_09749, score=4, range=221-239, strand=-1

target  5’ GCGACGGAUGAUCGGCGAA 3’
           :: :: .:::::::::::
query   3’ CG-UGAUUACUAGCCGCUC 5’

query=JS16_t0254413, target=FGSG_00508, score=4, range=172-189, strand=-1

target  5’ GGAGAAAUGAUCGGCGAC 3’
           : :  ::::::::::::
query   3’ CGUGAUUACUAGCCGCUC 5’

query=JS16_t0335245, target=FGSG_13834, score=4, range=193-212, strand=1

target  5’ AAGGA-GUUUGACUUUACUGG 3’
           ::::: : .::::::.:::::
query   3’ UUCCUCCCGACUGAAGUGACC 5’

query=JS16_t2224969, target=FGSG_11067, score=1.5, range=4252-4272, strand=-1

target  5’ AGUGUAUU-UUCGUACCCAUGU 3’
           :::::::. :::::::::::::
query   3’ UCACAUAGCAAGCAUGGGUACA 5’

query=JS16_t2224969, target=FGSG_00166, score=4, range=2574-2594, strand=1

target  5’ AGUGUAUCUUUCGUACCC-UGA 3’
           :::::::: ::::::::: ::
query   3’ UCACAUAGCAAGCAUGGGUACA 5’

query=JS16_t4017860, target=FGSG_01102, score=3, range=553-573, strand=1

target  5’ GGUGGCGGUGGUUGCG-UCGAG 3’
            ::::::::::::::: :::::
query   3’ ACACCGCCACCAACGCGAGCUC 5’

No results for JS16_t0684776
No results for JS16_t0794091
No results for JS16_t0364739
query=JS16_t1328788, target=FGSG_10718, score=3.5, range=1216-1235, strand=1

target  5’ GAGAACGUCGAUGGUUUCCU 3’
           ::::: :::.:.:::::::.
query   3’ CUCUUUCAGUUGCCAAAGGG 5’

query=JS16_t1328788, target=FGSG_07745, score=3.5, range=731-751, strand=-1

target  5’ GAGGAGGAUCAAUGGUUUCCU 3’
           :::.:.: ::::.:::::::.
query   3’ CUCUUUC-AGUUGCCAAAGGG 5’

query=JS16_t1328788, target=FGSG_09985, score=3.5, range=585-603, strand=-1

target  5’ CAGAAAGUCAAC-GUUUCCU 3’
            ::::::::::: ::::::.
query   3’ CUCUUUCAGUUGCCAAAGGG 5’

query=JS16_t1328788, target=FGSG_06945, score=4, range=1587-1607, strand=1

target  5’ CAGAAAGCUCAACGGUUACCC 3’
            :::::: ::::::::: :::
query   3’ CUCUUUC-AGUUGCCAAAGGG 5’

query=JS16_t1328788, target=FGSG_03886, score=4, range=1664-1683, strand=-1

target  5’ GAGGAAUUCGAUGGUUUCCU 3’
           :::.:: ::.:.:::::::.
query   3’ CUCUUUCAGUUGCCAAAGGG 5’

query=JS16_t1328788, target=FGSG_06084, score=4, range=380-399, strand=-1

target  5’ AAGAAACUCAGCGGUUUCUC 3’
            ::::: :::.:::::::.:
query   3’ CUCUUUCAGUUGCCAAAGGG 5’

query=JS16_t1328788, target=FGSG_04111, score=4, range=1262-1281, strand=-1

target  5’ GACAAGGUCAACGGCUUCCU 3’
           :: ::.:::::::: ::::.
query   3’ CUCUUUCAGUUGCCAAAGGG 5’

query=JS16_t1328788, target=FGSG_09022, score=4, range=1912-1932, strand=-1

query   3’ CGUUGAACUGCUCGAGCC 5’

query=JS16_t2287997, target=FGSG_01271, score=4, range=2051-2068, strand=-1

target  5’ AGAACUUGAUGGGCUCGG 3’
             :::::::.:.::::::
query   3’ CGUUGAACUGCUCGAGCC 5’

query=JS16_t2287997, target=FGSG_09723, score=4, range=686-703, strand=-1

target  5’ ACAUCUUGAUGGGCUCGG 3’
            :: :::::.:.::::::
query   3’ CGUUGAACUGCUCGAGCC 5’

query=JS16_t2287997, target=FGSG_08609, score=4, range=1598-1616, strand=-1

target  5’ AUAGCCUUGACGAGCUUGG 3’
            .:.  :::::::::::.::
query   3’ CGUU-GAACUGCUCGAGCC 5’

query=JS16_t2287997, target=FGSG_11922, score=4, range=23-41, strand=-1

target  5’ GUGACCUUGACGAGCUUGA 3’
           :..: :::::::::::.:
query   3’ CGUU-GAACUGCUCGAGCC 5’

query=JS16_t2287997, target=FGSG_06733, score=4, range=770-787, strand=-1

target  5’ GCCACUUGACGAACUUGG 3’
           :: ::::::::: ::.::
query   3’ CGUUGAACUGCUCGAGCC 5’

query=JS16_t2287997, target=FGSG_09716, score=4, range=378-395, strand=-1

target  5’ GCAUCUCGACGAGCUUGG 3’
           ::: :: ::::::::.::
query   3’ CGUUGAACUGCUCGAGCC 5’

query=JS16_t2287997, target=FGSG_10827, score=4, range=86-104, strand=-1

target  5’ GCAAGCUUGGCGAACUCGG 3’
           :::: ::::.::: :::::
query   3’ CGUU-GAACUGCUCGAGCC 5’

query=JS16_t2287997, target=FGSG_11821, score=4, range=776-793, strand=-1

target  5’ GUAUUUUGUCGAGCUCGG 3’
           :.: .::: :::::::::
query   3’ CGUUGAACUGCUCGAGCC 5’

query=JS16_t2287997, target=FGSG_06340, score=4, range=1067-1085, strand=-1

target  5’ GCGGCCUUGAAGAGCUCGG 3’
           ::.. ::::: ::::::::
query   3’ CGUU-GAACUGCUCGAGCC 5’

query=JS16_t2287997, target=FGSG_05962, score=4, range=896-914, strand=-1

target  5’ GCGCGCUUGAAGAGCUCGG 3’
           ::. .::::: ::::::::
query   3’ CGU-UGAACUGCUCGAGCC 5’

query=JS16_t2287997, target=FGSG_13196, score=4, range=230-248, strand=-1

target  5’ GUAGCCUUGACGAGAUCGG 3’
           :.:. ::::::::: ::::
query   3’ CGUU-GAACUGCUCGAGCC 5’

query=JS16_t2287997, target=FGSG_05931, score=4, range=266-283, strand=-1

target  5’ UUGAAUUGACGAGCUCGA 3’
            ..: ::::::::::::
query   3’ CGUUGAACUGCUCGAGCC 5’

query=JS16_t2287997, target=FGSG_09238, score=4, range=308-325, strand=-1

target  5’ CCUUCUUGACGGGCUCGG 3’
            :  :::::::.::::::
query   3’ CGUUGAACUGCUCGAGCC 5’

query=JS16_t2287997, target=FGSG_06039, score=4, range=1058-1074, strand=-1

target  5’ GUAG-UUGGCGAGCUCGU 3’
           :.:. :::.::::::::
query   3’ CGUUGAACUGCUCGAGCC 5’

query=JS16_t2287997, target=FGSG_02364, score=4, range=131-148, strand=-1

target  5’ GCAUCUUGACCAGCUCGU 3’
           ::: :::::: ::::::
query   3’ CGUUGAACUGCUCGAGCC 5’
```

```
target  5’ GAGAUACUCAAUCGGUUUCCC 3’
           :::: : :::: :::::::::
query   3’ CUCUUUCAGUU-GCCAAAGGG 5’

query=JS16_t1328788, target=FGSG_00388, score=4, range=141-159, strand=-1

target  5’ GAGAAUCUCAA-GGUUUCCC 3’
           :::::  :::: ::::::::
query   3’ CUCUUUCAGUUGCCAAAGGG 5’

query=JS16_t4018038, target=FGSG_10683, score=2, range=46-63, strand=1

target  5’ UUCCA-UGAGAAUGAUCCU 3’
           :.::: ::::::::::::.
query   3’ AGGGUCACUCUUACUAGGG 5’

query=JS16_t4018038, target=FGSG_10414, score=3, range=435-453, strand=1

target  5’ UCCUGGUGACAAUGAUCCC 3’
           :::..:::: :::::::::
query   3’ AGGGUCACUCUUACUAGGG 5’

query=JS16_t4018038, target=FGSG_01739, score=3, range=723-740, strand=1

target  5’ UCUUA-UGGGAAUGAUCCC 3’
           ::..: ::.::::::::::
query   3’ AGGGUCACUCUUACUAGGG 5’

query=JS16_t4018038, target=FGSG_11459, score=3.5, range=164-183, strand=1

target  5’ UAUUAGCUGAGAAUGAUCCU 3’
           : ..:: ::::::::::::.
query   3’ AGGGUC-ACUCUUACUAGGG 5’

query=JS16_t4018038, target=FGSG_12364, score=3.5, range=458-477, strand=1

target  5’ GUGCAGCUGAGAAUGAUCCC 3’
            . ::: :::::::::::::
query   3’ AGGGUC-ACUCUUACUAGGG 5’

query=JS16_t4018038, target=FGSG_03557, score=3.5, range=677-694, strand=-1

target  5’ UCCUAAUGAGAAUG-UCCC 3’
           :::.: :::::::: ::::
query   3’ AGGGUCACUCUUACUAGGG 5’

query=JS16_t4018038, target=FGSG_13503, score=4, range=312-330, strand=1

target  5’ CCCGAGUGAGGAUGAUCUC 3’
            :: ::::::.::::::.:
query   3’ AGGGUCACUCUUACUAGGG 5’

query=JS16_t4018038, target=FGSG_06265, score=4, range=234-252, strand=1

target  5’ UCCCAAUGAGGAUCAUCCC 3’
           ::::: ::::.:: :::::
query   3’ AGGGUCACUCUUACUAGGG 5’

query=JS16_t4018038, target=FGSG_08494, score=4, range=377-395, strand=1

target  5’ UCCCAAUGGGAAAGAUCCC 3’
           ::::: ::.::: ::::::
query   3’ AGGGUCACUCUUACUAGGG 5’

query=JS16_t4018038, target=FGSG_07188, score=4, range=1001-1020, strand=1

target  5’ UCAUCGUUGAGGAUGAUCCC 3’
           :: .:. ::::.::::::::
query   3’ AG-GGUCACUCUUACUAGGG 5’

query=JS16_t4018038, target=FGSG_03535, score=4, range=122-140, strand=1

target  5’ ACCCACUGAGAAAGAUCCC 3’
            :::: :::::: ::::::
query   3’ AGGGUCACUCUUACUAGGG 5’

query=JS16_t4018038, target=FGSG_00351, score=4, range=245-264, strand=1

target  5’ UCCCAGAUGAGAAUGAUCGA 3’
           :::::: :::::::::::
query   3’ AGGGUC-ACUCUUACUAGGG 5’

query=JS16_t4018038, target=FGSG_03120, score=4, range=283-302, strand=-1

target  5’ GCCCAGAUGGGAAUGAUCUC 3’
            ::::: ::.::::::::.:
query   3’ AGGGUC-ACUCUUACUAGGG 5’

query=JS16_t4018038, target=FGSG_13878, score=4, range=5253-5271, strand=-1

target  5’ GCUUCGUGAGAAUGAUUCC 3’
```

```
query=JS16_t2287997, target=FGSG_09744, score=4, range=695-713, strand=-1

target  5’ UCAAUCUUGACGAUCUCGG 3’
            ::: :::::::: :::::
query   3’ CGUU-GAACUGCUCGAGCC 5’

query=JS16_t2287997, target=FGSG_12183, score=4, range=2144-2162, strand=-1

target  5’ GCAUACUUGACGCGCUCGC 3’
           ::: :::::::: :::::
query   3’ CGU-UGAACUGCUCGAGCC 5’

query=JS16_t2287997, target=FGSG_10940, score=4, range=620-638, strand=-1

target  5’ ACAAUCUUGACGAGCUCAG 3’
            ::: :::::::::::: :
query   3’ CGUU-GAACUGCUCGAGCC 5’

query=JS16_t2287997, target=FGSG_12827, score=4, range=386-404, strand=-1

target  5’ ACAACGUUGACAAGCUCGG 3’
            :::: ::::: :::::::
query   3’ CGUUG-AACUGCUCGAGCC 5’

query=JS16_t2287997, target=FGSG_03372, score=4, range=15-33, strand=-1

target  5’ GCCACAUUGACGAGCUUGA 3’
           :: :: ::::::::::.:
query   3’ CGUUG-AACUGCUCGAGCC 5’

query=JS16_t2287997, target=FGSG_01828, score=4, range=272-288, strand=-1

target  5’ GUGAGUUGACGAG-UCGG 3’
           :..: :::::::: ::::
query   3’ CGUUGAACUGCUCGAGCC 5’

query=JS16_t2287997, target=FGSG_12179, score=4, range=50-68, strand=-1

target  5’ GUGUUCUUGACGAGCUCGU 3’
           :..  :::::::::::::
query   3’ CGU-UGAACUGCUCGAGCC 5’

query=JS16_t2287997, target=FGSG_00906, score=4, range=1097-1114, strand=-1

target  5’ GCUUCUUGACGAGCUCAG 3’
           ::  :::::::::::: :
query   3’ CGUUGAACUGCUCGAGCC 5’

query=JS16_t2287997, target=FGSG_10922, score=4, range=683-699, strand=-1

target  5’ GCUA-UUGACGGGCUCGU 3’
           :: : ::::::.:::::
query   3’ CGUUGAACUGCUCGAGCC 5’

query=JS16_t2287997, target=FGSG_04396, score=4, range=793-811, strand=-1

target  5’ GCCACUUGAACGAGCUCGC 3’
           :: ::::: :::::::::
query   3’ CGUUGAAC-UGCUCGAGCC 5’

No results for JS16_t0337333
query=JS16_t0366859, target=FGSG_12126, score=3, range=6169-6186, strand=1

target  5’ GAGGUGUUGGGAAUCCGC 3’
           ::::::::::::: :::
query   3’ CUCCACAACCCUUUGGCA 5’

query=JS16_t0366859, target=FGSG_00932, score=3, range=176-193, strand=-1

target  5’ GAGGGUUUGGGAAACCGU 3’
           ::::  ::::::::::::
query   3’ CUCCACAACCCUUUGGCA 5’

query=JS16_t0366859, target=FGSG_07166, score=3, range=1214-1230, strand=-1

target  5’ GCGGUGUUGGG-AACCGU 3’
           : ::::::::: ::::::
query   3’ CUCCACAACCCUUUGGCA 5’

query=JS16_t0366859, target=FGSG_10346, score=3, range=512-528, strand=-1

target  5’ GAGAUGUUGGG-AACCGU 3’
           ::: ::::::: ::::::
query   3’ CUCCACAACCCUUUGGCA 5’

query=JS16_t0366859, target=FGSG_03565, score=3.5, range=164-180, strand=-1

target  5’ GGGCUGUUGGG-AACCGU 3’
           :.: ::::::: ::::::
query   3’ CUCCACAACCCUUUGGCA 5’

query=JS16_t0366859, target=FGSG_10114, score=4, range=31-48, strand=1
```

```
            :.. :::::::::::.::
query   3’ AGGGUCACUCUUACUAGGG 5’

query=JS16_t4018038, target=FGSG_06881, score=4, range=2537-2554, strand=-1

target  5’ UUCC-GUGAGAAUGGUCUU 3’
           :.:: :::::::::.::..
query   3’ AGGGUCACUCUUACUAGGG 5’

No results for JS16_t3121283
query=JS16_t2225241, target=FGSG_00945, score=4, range=1041-1063, strand=1

target  5’ GUCCAUGCG-ACGAACGAUACACU 3’
           .. :::: : ::::::::::::::
query   3’ UGUGUACCCAUGCUUGCUAUGUGA 5’

No results for JS16_t0794112
No results for JS16_t1328936
query=JS16_t2225327, target=FGSG_01346, score=4, range=250-271, strand=-1

target  5’ CAACAGC-GGACUGGUCCUUGAC 3’
           :  :::: ..:::::::::::::
query   3’ GACGUCGUUUUGACCAGGAACUG 5’

No results for JS16_t4018278
No results for JS16_t3121522
query=JS16_t0576147, target=FGSG_13098, score=4, range=1899-1918, strand=1

target  5’ GUAUUUUUUCCCACUGGAUA 3’
           :::.:.::::: ::::::.:
query   3’ CAUGAGAAAGGCUGACCUGU 5’

query=JS16_t0576147, target=FGSG_01289, score=4, range=93-111, strand=1

target  5’ CUAC-CUUUCCGACUGGAUU 3’
            ::: :::::::::::::.
query   3’ CAUGAGAAAGGCUGACCUGU 5’

query=JS16_t0576147, target=FGSG_11395, score=4, range=2955-2974, strand=1

target  5’ GGACACUUUCCGUCUGGACA 3’
           : :: ::::::: :::::::
query   3’ CAUGAGAAAGGCUGACCUGU 5’

query=JS16_t0576147, target=FGSG_05662, score=4, range=420-439, strand=-1

target  5’ GUUCCCUUUCCGGCUGGAUA 3’
           :: : :::::::.:::::.:
query   3’ CAUGAGAAAGGCUGACCUGU 5’

query=JS16_t0576147, target=FGSG_00200, score=4, range=1080-1098, strand=-1

target  5’ GAACUC-UUCCGACUUGACA 3’
           : :::: :::::::: ::::
query   3’ CAUGAGAAAGGCUGACCUGU 5’

No results for JS16_t0794126
query=JS16_t2225486, target=FGSG_06799, score=4, range=580-601, strand=1

target  5’ AACUACAA-CCAUAACGUUCGGC 3’
           :  : ::: ::::::::::::::
query   3’ UCAAGGUUCGGUAUUGCAAGCCG 5’

query=JS16_t3121621, target=FGSG_06101, score=3.5, range=1390-1408, strand=-1

target  5’ CUCCAG-AUUUGUCGCCAAG 3’
           :  ::: ::::::::::::.
query   3’ GUCGUCAUAAACAGCGGUUU 5’

query=JS16_t3121621, target=FGSG_13164, score=4, range=511-531, strand=1

target  5’ CGGACAGAAUUUGUCGUCAAG 3’
           :.: ::: ::::::::.:::.
query   3’ GUC-GUCAUAAACAGCGGUUU 5’

query=JS16_t3121621, target=FGSG_11788, score=4, range=410-430, strand=1

target  5’ CAGCAAAUAUAUGUCGCCAAA 3’
           :::: : ::: ::::::::::
query   3’ GUCG-UCAUAAACAGCGGUUU 5’

query=JS16_t3121621, target=FGSG_08239, score=4, range=522-541, strand=-1

target  5’ AAGCGAUAUUUGUCGCCAGG 3’
            :::. ::::::::::::..
query   3’ GUCGUCAUAAACAGCGGUUU 5’

query=JS16_t3121621, target=FGSG_08313, score=4, range=767-787, strand=-1

target  5’ AACCAGUAAUUUGUCGCCAGA 3’
            : :::: :::::::::::.:
query   3’ GUCGUCA-UAAACAGCGGUUU 5’

target  5’ GUGGUGUUGGGAGACGGU 3’
           : ::::::::::.:: ::
query   3’ CUCCACAACCCUUUGGCA 5’

query=JS16_t0366859, target=FGSG_07364, score=4, range=1724-1740, strand=1

target  5’ GCGGUGUUGGGAAG-CGU 3’
           : :::::::::::. :::
query   3’ CUCCACAACCCUUUGGCA 5’

query=JS16_t0366859, target=FGSG_11392, score=4, range=811-827, strand=1

target  5’ GAGG-GUUGGGACACUGU 3’
           :::: ::::::: ::.::
query   3’ CUCCACAACCCUUUGGCA 5’

query=JS16_t0366859, target=FGSG_08192, score=4, range=148-165, strand=1

target  5’ GAGAAGUUGGGAAACCCU 3’
           :::  ::::::::::: :
query   3’ CUCCACAACCCUUUGGCA 5’

query=JS16_t0366859, target=FGSG_04764, score=4, range=332-348, strand=1

target  5’ CAGG-GUUGAGAAACCGU 3’
            ::: :::: ::::::::
query   3’ CUCCACAACCCUUUGGCA 5’

query=JS16_t0366859, target=FGSG_05273, score=4, range=883-901, strand=1

target  5’ GAGGAGUUGGAGAAACUGU 3’
           :::: ::::: :::::.::
query   3’ CUCCACAACC-CUUUGGCA 5’

query=JS16_t0366859, target=FGSG_11084, score=4, range=65-83, strand=1

target  5’ GAGGUGCUGUGGAAACCGU 3’
           :::::: :: :::::::::
query   3’ CUCCACAAC-CCUUUGGCA 5’

query=JS16_t0366859, target=FGSG_14027, score=4, range=220-238, strand=1

target  5’ GAAGAGUUGUGGAAACCGU 3’
           :: : :::: :::::::::
query   3’ CUCCACAAC-CCUUUGGCA 5’

query=JS16_t0366859, target=FGSG_09418, score=4, range=382-398, strand=-1

target  5’ GAGGUGUUGGG-GAUCGU 3’
           ::::::::::: .:.:::
query   3’ CUCCACAACCCUUUGGCA 5’

query=JS16_t0366859, target=FGSG_09581, score=4, range=136-153, strand=-1

target  5’ GAAGUGUUGGGAAACCAG 3’
           :: :::::::::::::
query   3’ CUCCACAACCCUUUGGCA 5’

query=JS16_t0366859, target=FGSG_09029, score=4, range=462-479, strand=-1

target  5’ GUGUUGUUGGGAAAUCGC 3’
           : : ::::::::::.::
query   3’ CUCCACAACCCUUUGGCA 5’

query=JS16_t0366859, target=FGSG_02147, score=4, range=387-404, strand=-1

target  5’ GAAUUGUUGGGAAACUGC 3’
           ::  ::::::::::::.:
query   3’ CUCCACAACCCUUUGGCA 5’

query=JS16_t0366859, target=FGSG_10106, score=4, range=388-404, strand=-1

target  5’ GAGAUGUUGGGGAA-CGU 3’
           ::: :::::::.:: :::
query   3’ CUCCACAACCCUUUGGCA 5’

query=JS16_t0366859, target=FGSG_03455, score=4, range=294-311, strand=-1

target  5’ UCGCUGUUGGGAAACCGG 3’
             : :::::::::::::
query   3’ CUCCACAACCCUUUGGCA 5’

query=JS16_t0366859, target=FGSG_05459, score=4, range=1570-1586, strand=-1

target  5’ GAUGUGUUGGG-AACCGC 3’
           :: :::::::: :::::
query   3’ CUCCACAACCCUUUGGCA 5’

No results for JS16_t0474700
No results for JS16_t0692282
No results for JS16_t3184298
```

```
query=JS16_t3121621, target=FGSG_02258, score=4, range=571-589, strand=-1

target  5’ UAGCGGU-UUUGUUGCCAAA 3’
           .:::.:: :::::.::::::
query   3’ GUCGUCAUAAACAGCGGUUU 5’

No results for JS16_t3121641
No results for JS16_t4018476
No results for JS16_t3121750
query=JS16_t1329291, target=FGSG_02732, score=4, range=1018-1037, strand=1

target  5’ UCUGCUGUGGCGAUAACUCC 3’
            ::.::..:::::::::::
query   3’ UGAUGAUGCCGCUAUUGAGC 5’

query=JS16_t1329291, target=FGSG_07413, score=4, range=3465-3483, strand=1

target  5’ AUUGC-ACGGCGAUGACUCA 3’
           :.:.: ::::::::.::::
query   3’ UGAUGAUGCCGCUAUUGAGC 5’

query=JS16_t1329291, target=FGSG_12230, score=4, range=8395-8413, strand=1

target  5’ ACUACUCCGGCGGUAA-UCG 3’
           :::::: :::::.::: :::
query   3’ UGAUGAUGCCGCUAUUGAGC 5’

query=JS16_t1329291, target=FGSG_11913, score=4, range=1066-1084, strand=-1

target  5’ AC-GCUGCGGCGAUAGUUCG 3’
           :: .::.::::::::..:::
query   3’ UGAUGAUGCCGCUAUUGAGC 5’

query=JS16_t1329291, target=FGSG_07026, score=4, range=151-169, strand=-1

target  5’ GCUGCU-CGGCGAUGGCUCG 3’
           .::.:: :::::::..::::
query   3’ UGAUGAUGCCGCUAUUGAGC 5’

query=JS16_t1329291, target=FGSG_11410, score=4, range=783-802, strand=-1

target  5’ ACUCUGGCGGCGAUGACUCG 3’
           ::: . .:::::::.:::::
query   3’ UGAUGAUGCCGCUAUUGAGC 5’

No results for JS16_t1329316
query=JS16_t1329358, target=FGSG_08909, score=3, range=1255-1271, strand=-1

target  5’ UUAAAAU-CCGCGCUGUU 3’
           ::::::: :::::::.::
query   3’ AAUUUUAGGGCGCGAUAA 5’

query=JS16_t1329358, target=FGSG_03353, score=4, range=798-815, strand=-1

target  5’ UUGAGAUCUCGCGCAAUU 3’
           ::.:.:::.::::: :::
query   3’ AAUUUUAGGGCGCGAUAA 5’

query=JS16_t1329358, target=FGSG_08176, score=4, range=45-62, strand=-1

target  5’ UUAAAGUCCCUCGCUAUG 3’
           :::::.:::: ::::::
query   3’ AAUUUUAGGGCGCGAUAA 5’

query=JS16_t1329358, target=FGSG_00503, score=4, range=1426-1443, strand=-1

target  5’ GUAAAAUCCCGCCCUGUU 3’
            ::::::::::: ::.::
query   3’ AAUUUUAGGGCGCGAUAA 5’

query=JS16_t1329358, target=FGSG_13662, score=4, range=1353-1370, strand=-1

target  5’ UUGAGAUCCCGAGCUAUA 3’
           ::.:.::::::: :::::
query   3’ AAUUUUAGGGCGCGAUAA 5’

query=JS16_t1329358, target=FGSG_06122, score=4, range=1020-1036, strand=-1

target  5’ UUAAUAUCUCGCGC-AUU 3’
           :::: :::.::::: :::
query   3’ AAUUUUAGGGCGCGAUAA 5’

query=JS16_t3121869, target=FGSG_13483, score=3, range=1030-1048, strand=1

target  5’ CGC-GAUUUCGAUCUCUUGG 3’
           ::: ::::::.:.:::::::
query   3’ GCGUCUAAAGUUGGAGAACC 5’

query=JS16_t3121869, target=FGSG_09793, score=3.5, range=1786-1805, strand=1

target  5’ CGCGACUUUCAACUUCUUGG 3’
```

```
No results for JS16_t4080967
No results for JS16_t1392058
query=JS16_t0366862, target=FGSG_04162, score=3.5, range=293-316, strand=1

target  5’ UUGAGGACUACGAAGAAAUUGGCG 3’
           .::: :: :: :::::::::::::
query   3’ GACUGCU-AUUCUUCUUUAACCGC 5’

No results for JS16_t2288193
No results for JS16_t2288247
query=JS16_t0474728, target=FGSG_01092, score=2.5, range=379-397, strand=1

target  5’ CUCGACGAGACAGAGCAGC 3’
           :.::::::.:::::::::
query   3’ GGGCUGCUUUGUCUCGUCU 5’

query=JS16_t0474728, target=FGSG_11573, score=2.5, range=48-66, strand=1

target  5’ CACGAUGGAACAGAGCAGA 3’
           : :::.:.:::::::::::
query   3’ GGGCUGCUUUGUCUCGUCU 5’

query=JS16_t0474728, target=FGSG_07495, score=3, range=1693-1711, strand=1

target  5’ CACGAGGAGACAGAGCAGA 3’
           : ::: ::.::::::::::
query   3’ GGGCUGCUUUGUCUCGUCU 5’

query=JS16_t0474728, target=FGSG_05420, score=3, range=1185-1203, strand=1

target  5’ CCAAACGAAACAGAGCAGU 3’
           ::  ::::::::::::::
query   3’ GGGCUGCUUUGUCUCGUCU 5’

query=JS16_t0474728, target=FGSG_07294, score=3.5, range=1885-1903, strand=1

target  5’ UCCGACGAUACGGAGCAGA 3’
           .::::::: ::.:::::::
query   3’ GGGCUGCUUUGUCUCGUCU 5’

query=JS16_t0474728, target=FGSG_03081, score=3.5, range=181-199, strand=1

target  5’ CUCGUUGAAACAGGGCAGG 3’
           :.:: .:::::::.::::.
query   3’ GGGCUGCUUUGUCUCGUCU 5’

query=JS16_t0474728, target=FGSG_07104, score=3.5, range=2947-2965, strand=-1

target  5’ CGGGACGAAGCAGAGCAGG 3’
           :  ::::::.::::::::.
query   3’ GGGCUGCUUUGUCUCGUCU 5’

query=JS16_t0474728, target=FGSG_00243, score=4, range=379-397, strand=1

target  5’ UCCAACGAGGCAGAGCAGG 3’
           .:: ::::..::::::::.
query   3’ GGGCUGCUUUGUCUCGUCU 5’

query=JS16_t0474728, target=FGSG_02544, score=4, range=787-805, strand=1

target  5’ ACCGACCGAACAGAGCAGA 3’
            ::::: .:::::::::::
query   3’ GGGCUGCUUUGUCUCGUCU 5’

query=JS16_t0474728, target=FGSG_04316, score=4, range=934-952, strand=1

target  5’ GCCGACGAGAAAGAGCAGA 3’
            :::::::.: ::::::::
query   3’ GGGCUGCUUUGUCUCGUCU 5’

query=JS16_t0474728, target=FGSG_01925, score=4, range=768-787, strand=1

target  5’ UCCGGCCAAAGCAGAGCAGA 3’
           .:::.: ::: :::::::::
query   3’ GGGCUGCUUU-GUCUCGUCU 5’

query=JS16_t0474728, target=FGSG_05894, score=4, range=1099-1117, strand=1

target  5’ ACCUACGAAACAGAGCGGU 3’
            :: ::::::::::::.:
query   3’ GGGCUGCUUUGUCUCGUCU 5’

query=JS16_t0474728, target=FGSG_04888, score=4, range=1591-1609, strand=1

target  5’ GCCGAGCAAACAGAGCAGA 3’
            ::::  ::::::::::::
query   3’ GGGCUGCUUUGUCUCGUCU 5’

query=JS16_t0474728, target=FGSG_00293, score=4, range=2452-2470, strand=1

target  5’ ACCAAGGGAACAGAGCAGA 3’
            :: : :.:::::::::::
```

```
          :::.  :::::::.::::::
query   3' GCGUCUAAAGUUGGAGAACC 5'

query=JS16_t3121869, target=FGSG_02935, score=3.5, range=88-106, strand=1

target  5' UGCAG-CUUCAACCUCUUGA 3'
          .::::  ::::::::::::
query   3' GCGUCUAAAGUUGGAGAACC 5'

query=JS16_t3121869, target=FGSG_08651, score=3.5, range=128-146, strand=-1

target  5' CGUCGA-UUCAGCCUCUUGG 3'
          ::. :: ::::.::::::::
query   3' GCGUCUAAAGUUGGAGAACC 5'

query=JS16_t3121869, target=FGSG_02063, score=4, range=214-234, strand=1

target  5' CGCGAGAUUGCAACCUCUUGU 3'
          ::: ::::: ::::::::::
query   3' GCG-UCUAAAGUUGGAGAACC 5'

query=JS16_t3121869, target=FGSG_03032, score=4, range=784-802, strand=1

target  5' CG-AGCUUUCAACAUCUUGG 3'
          :: :: ::::::: ::::::
query   3' GCGUCUAAAGUUGGAGAACC 5'

query=JS16_t2225754, target=FGSG_02075, score=3.5, range=34-55, strand=1

target  5' GGAUAUGGCAUGUCCGCCAAGA 3'
          :::::::. :::::::::::.
query   3' CCUAUACUCUACAGGCGGUUUG 5'

No results for JS16_t2225856
query=JS16_t0576200, target=FGSG_11268, score=3.5, range=1823-1840, strand=-1

target  5' UGUCUAGUUGGAAA-AGUA 3'
          ::::.:::.::::: ::::
query   3' ACAGGUCAGCCUUUCUCAU 5'

query=JS16_t0576200, target=FGSG_09371, score=4, range=557-576, strand=1

target  5' UGAUGCAGUCGGAGAGAGUU 3'
          :: : ::::::::.:::::
query   3' AC-AGGUCAGCCUUUCUCAU 5'

query=JS16_t0576200, target=FGSG_08381, score=4, range=150-169, strand=1

target  5' UGUCUCUGUCGGAAAGAGCA 3'
          :::: : ::::::::::: :
query   3' ACAG-GUCAGCCUUUCUCAU 5'

query=JS16_t0576200, target=FGSG_00200, score=4, range=1080-1097, strand=1

target  5' UGUCAAGUCGG-AAGAGUU 3'
          :::: :::::: ::::::
query   3' ACAGGUCAGCCUUUCUCAU 5'

query=JS16_t0576200, target=FGSG_02541, score=4, range=402-420, strand=-1

target  5' GGUAGAGUCGGAAGGAGUA 3'
           ::  ::::::::.:::::
query   3' ACAGGUCAGCCUUUCUCAU 5'

query=JS16_t0576200, target=FGSG_06647, score=4, range=342-361, strand=-1

target  5' UGGCCAUGUCGGGAAGAGUU 3'
          :: ::: :::::.::::::
query   3' ACAGGU-CAGCCUUUCUCAU 5'

query=JS16_t0576200, target=FGSG_01289, score=4, range=94-111, strand=-1

target  5' AAUCCAGUCGGAAAG-GUA 3'
            ::::::::::::: :::
query   3' ACAGGUCAGCCUUUCUCAU 5'

query=JS16_t0576200, target=FGSG_09623, score=4, range=372-391, strand=-1

target  5' UCUUGUAGUCGGAAAGAGUC 3'
          : :. .:::::::::::::
query   3' ACAG-GUCAGCCUUUCUCAU 5'

query=JS16_t0576200, target=FGSG_06810, score=4, range=820-837, strand=-1

target  5' AGUCC-GUUGGAAAGAGUC 3'
           :::: ::.:::::::::
query   3' ACAGGUCAGCCUUUCUCAU 5'

query=JS16_t0576200, target=FGSG_13068, score=4, range=344-362, strand=-1

target  5' UGAACAGUCGGCAAGAGUA 3'
          ::  ::::::: :::::::
query   3' GGGCUGCUUUGUCUCGUCU 5'

query=JS16_t0474728, target=FGSG_03951, score=4, range=928-945, strand=1

target  5' CCAGA-GGAGCAGAGCAGA 3'
          :: :: :.:.:::::::::
query   3' GGGCUGCUUUGUCUCGUCU 5'

query=JS16_t0474728, target=FGSG_04002, score=4, range=292-309, strand=1

target  5' UUCGA-GAAACAGAGCAAA 3'
          ..::: ::::::::::: :
query   3' GGGCUGCUUUGUCUCGUCU 5'

query=JS16_t0474728, target=FGSG_12227, score=4, range=222-240, strand=-1

target  5' CUGGAGGAAGCAGAGCAGG 3'
          :. :: :::.::::::::.
query   3' GGGCUGCUUUGUCUCGUCU 5'

query=JS16_t0474728, target=FGSG_08447, score=4, range=806-824, strand=-1

target  5' CGCGACGAAACAGAGCCGC 3'
          : ::::::::::::: :
query   3' GGGCUGCUUUGUCUCGUCU 5'

query=JS16_t1392257, target=FGSG_09014, score=2.5, range=883-900, strand=1

target  5' GUGGCUCUUCAAGCUUUG 3'
          .::::::::::.::::
query   3' UACCGAGAAGUUUGAAAG 5'

query=JS16_t1392257, target=FGSG_07889, score=2.5, range=62-79, strand=1

target  5' UUGGUCUCUCAGACUUUC 3'
           :::.::::::.::::::
query   3' UACCGAGAAGUUUGAAAG 5'

query=JS16_t1392257, target=FGSG_12041, score=3, range=155-171, strand=1

target  5' AUGG-UCUUCAAUCUUUC 3'
          :::: ::::::: :::::
query   3' UACCGAGAAGUUUGAAAG 5'

query=JS16_t1392257, target=FGSG_11425, score=3, range=2282-2299, strand=-1

target  5' AUCGCUCUUCAAACUUAC 3'
          :: ::::::::::::: :
query   3' UACCGAGAAGUUUGAAAG 5'

query=JS16_t1392257, target=FGSG_05451, score=3.5, range=1144-1161, strand=1

target  5' GUUGUUCUUCAAGCUUUU 3'
          .: :.:::::::.::::.
query   3' UACCGAGAAGUUUGAAAG 5'

query=JS16_t1392257, target=FGSG_11306, score=3.5, range=3-20, strand=1

target  5' GAGGUUCUUCAAGCUUUU 3'
          .  ::.:::::::.::::.
query   3' UACCGAGAAGUUUGAAAG 5'

query=JS16_t1392257, target=FGSG_05660, score=3.5, range=1967-1983, strand=1

target  5' AU-GUUCUUCAAACCUUC 3'
          :: :.::::::::: :::
query   3' UACCGAGAAGUUUGAAAG 5'

query=JS16_t1392257, target=FGSG_11840, score=3.5, range=250-266, strand=1

target  5' AU-GUUAUUCAAACUUUC 3'
          :: :.: ::::::::::
query   3' UACCGAGAAGUUUGAAAG 5'

query=JS16_t1392257, target=FGSG_13287, score=3.5, range=224-241, strand=-1

target  5' AUGGCUUUUCGGACUUUU 3'
          ::::::.:::..:::::.
query   3' UACCGAGAAGUUUGAAAG 5'

query=JS16_t1392257, target=FGSG_13420, score=3.5, range=605-622, strand=-1

target  5' CUUGCUCUUCAAGCUUUU 3'
           : :::::::::.::::.
query   3' UACCGAGAAGUUUGAAAG 5'

query=JS16_t1392257, target=FGSG_01614, score=3.5, range=1320-1337, strand=-1

target  5' GUAGCUCUUCCAACUUUC 3'
          .: ::::::: :::::::
query   3' UACCGAGAAGUUUGAAAG 5'
```

```
query   3' ACAGGUCAGCCUUUCUCAU 5'

query=JS16_t4018751, target=FGSG_08325, score=2, range=2993-3010, strand=-1

target  5' AAGA-UUUGAUCGAAGGUU 3'
           :::: :::::::::::.:::
query   3' UUCUGAAACUAGCUUUCAA 5'

query=JS16_t4018751, target=FGSG_03102, score=2.5, range=598-615, strand=-1

target  5' AGGACUUUGAUC-AAAGUU 3'
           :.:::::::::: ::::::
query   3' UUCUGAAACUAGCUUUCAA 5'

query=JS16_t4018751, target=FGSG_01739, score=3, range=308-326, strand=-1

target  5' GGGUCGUUGAUCGAAAGUU 3'
           ..: : :::::::::::::
query   3' UUCUGAAACUAGCUUUCAA 5'

query=JS16_t4018751, target=FGSG_11659, score=3.5, range=5204-5222, strand=-1

target  5' GAGUCUUUGAUCGAGAGUG 3'
           .:: ::::::::::.:::
query   3' UUCUGAAACUAGCUUUCAA 5'

query=JS16_t4018751, target=FGSG_01735, score=3.5, range=1260-1278, strand=-1

target  5' AAGCUCUUGAUCGAGAGUU 3'
           ::: . ::::::::.::::
query   3' UUCUGAAACUAGCUUUCAA 5'

query=JS16_t4018751, target=FGSG_09967, score=4, range=197-215, strand=1

target  5' AAUACUUUGGUCGCAAGUU 3'
           :: ::::::.::: :::::
query   3' UUCUGAAACUAGCUUUCAA 5'

query=JS16_t4018751, target=FGSG_09285, score=4, range=374-392, strand=1

target  5' UAGACUUUGAUCGAAAAUG 3'
            ::::::::::::::: :
query   3' UUCUGAAACUAGCUUUCAA 5'

query=JS16_t4018751, target=FGSG_01893, score=4, range=1003-1020, strand=1

target  5' AACACUUUGAUC-AAGGUU 3'
           :: ::::::::: ::.:::
query   3' UUCUGAAACUAGCUUUCAA 5'

query=JS16_t4018751, target=FGSG_05297, score=4, range=2935-2954, strand=1

target  5' AAGACAUUGAUGCGAAAGUC 3'
           ::::: ::::: :::::::
query   3' UUCUGAAACUA-GCUUUCAA 5'

query=JS16_t4018751, target=FGSG_08439, score=4, range=688-706, strand=-1

target  5' UAGACUUUGAUCGAGGGUC 3'
            :::::::::::::..::
query   3' UUCUGAAACUAGCUUUCAA 5'

query=JS16_t4018751, target=FGSG_09000, score=4, range=208-227, strand=-1

target  5' AAGACUUUGAUCGACAGGUC 3'
           :::::::::::::: :.::
query   3' UUCUGAAACUAGCU-UUCAA 5'

query=JS16_t4018751, target=FGSG_13692, score=4, range=1221-1239, strand=-1

target  5' AUCUCCUUGAUCGAAAGUU 3'
           :   : :::::::::::::
query   3' UUCUGAAACUAGCUUUCAA 5'

No results for JS16_t2225966
No results for JS16_t4018807
No results for JS16_t0364777
No results for JS16_t4018877
No results for JS16_t2226043
query=JS16_t0684890, target=FGSG_05105, score=3, range=258-277, strand=1

target  5' AGACACCAAGAAGUCGCCGA 3'
            :::::::::::::::: ::
query   3' CCUGUGGUUCUUCAGCGCCU 5'

query=JS16_t0684890, target=FGSG_09593, score=3.5, range=293-311, strand=1

target  5' GGGCA-CAAGAUGUCGCGGA 3'
           ::.:: ::::: ::::::::
query   3' CCUGUGGUUCUUCAGCGCCU 5'

query=JS16_t0684890, target=FGSG_00768, score=4, range=1263-1282, strand=1

query=JS16_t1392257, target=FGSG_01790, score=3.5, range=4423-4440, strand=-1

target  5' AACAUUCUUCAAACUUUC 3'
           :   .:::::::::::::
query   3' UACCGAGAAGUUUGAAAG 5'

query=JS16_t1392257, target=FGSG_10865, score=4, range=326-343, strand=1

target  5' AUGGCUUUUCAAGCUUGC 3'
           ::::::.:::::.::: :
query   3' UACCGAGAAGUUUGAAAG 5'

query=JS16_t1392257, target=FGSG_06352, score=4, range=1030-1047, strand=1

target  5' AUGGCUUUUCAAACAUUA 3'
           ::::::.::::::: ::
query   3' UACCGAGAAGUUUGAAAG 5'

query=JS16_t1392257, target=FGSG_03754, score=4, range=592-609, strand=1

target  5' AUCGCUCUUCAGGCUUUG 3'
           :: ::::::::..::::
query   3' UACCGAGAAGUUUGAAAG 5'

query=JS16_t1392257, target=FGSG_13254, score=4, range=532-549, strand=1

target  5' GCAGUUCUUCAAGCUUUC 3'
           .   :.::::::::.:::::
query   3' UACCGAGAAGUUUGAAAG 5'

query=JS16_t1392257, target=FGSG_01172, score=4, range=744-762, strand=1

target  5' AUGGGUCUUCAAGCUUAUC 3'
           :::: :::::::.::: ::
query   3' UACCGAGAAGUUUGAA-AG 5'

query=JS16_t1392257, target=FGSG_12355, score=4, range=2417-2433, strand=1

target  5' AUGG-UCUUCAAGUUUUA 3'
           :::: :::::::..:::
query   3' UACCGAGAAGUUUGAAAG 5'

query=JS16_t1392257, target=FGSG_11825, score=4, range=293-309, strand=1

target  5' AU-GCUCUUCAACUUUUC 3'
           :: ::::::::: .::::
query   3' UACCGAGAAGUUUGAAAG 5'

query=JS16_t1392257, target=FGSG_00013, score=4, range=1392-1408, strand=1

target  5' CUGGCUCUUC-AACUUUG 3'
            ::::::::: ::::::
query   3' UACCGAGAAGUUUGAAAG 5'

query=JS16_t1392257, target=FGSG_05646, score=4, range=143-160, strand=-1

target  5' AUAGCUCUUCGAGUUUUC 3'
           :: :::::::.:..::::
query   3' UACCGAGAAGUUUGAAAG 5'

query=JS16_t1392257, target=FGSG_10348, score=4, range=640-657, strand=-1

target  5' UUGGCUCUUCAAGCUAUC 3'
            :::::::::::.:: ::
query   3' UACCGAGAAGUUUGAAAG 5'

query=JS16_t1392257, target=FGSG_05780, score=4, range=848-864, strand=-1

target  5' GU-GCUCUUCGAGCUUUU 3'
           .: :::::::.:.::::.
query   3' UACCGAGAAGUUUGAAAG 5'

query=JS16_t1392257, target=FGSG_03000, score=4, range=698-715, strand=-1

target  5' CCGGCUCUGCAAACUUUC 3'
             :::::: :::::::::
query   3' UACCGAGAAGUUUGAAAG 5'

query=JS16_t1392257, target=FGSG_07685, score=4, range=1835-1852, strand=-1

target  5' CCAACUCUUCAAACUUUC 3'
               ::::::::::::::
query   3' UACCGAGAAGUUUGAAAG 5'

query=JS16_t1392257, target=FGSG_00695, score=4, range=1922-1939, strand=-1

target  5' UUUCCUUUUCAAACUUUC 3'
            :   ::.:::::::::::
query   3' UACCGAGAAGUUUGAAAG 5'

query=JS16_t1392257, target=FGSG_01224, score=4, range=368-385, strand=-1
```

```
target  5’ GGGCACAAAGAGGUUGCGGG 3’
           ::.::: ::::.::.::::.
query   3’ CCUGUGGUUCUUCAGCGCCU 5’

query=JS16_t0684890, target=FGSG_10284, score=4, range=1007-1025, strand=1

target  5’ GGA-ACCAGGAGGUCGUGGA 3’
           ::: ::::.::.::::.:::
query   3’ CCUGUGGUUCUUCAGCGCCU 5’

query=JS16_t0684890, target=FGSG_03158, score=4, range=1463-1483, strand=1

target  5’ GGUUGAUCAAGAAGUCGUGGA 3’
           :: . :.::::::::::.:::
query   3’ CCUG-UGGUUCUUCAGCGCCU 5’

query=JS16_t0684890, target=FGSG_08077, score=4, range=1222-1240, strand=1

target  5’ GGACA-CAAGAAGUCGCCGU 3’
           ::::: :::::::::: :
query   3’ CCUGUGGUUCUUCAGCGCCU 5’

query=JS16_t0684890, target=FGSG_07928, score=4, range=1198-1217, strand=-1

target  5’ GGCCAUUGAGAGGUCGCGGA 3’
           :: ::...:::.::::::::
query   3’ CCUGUGGUUCUUCAGCGCCU 5’

query=JS16_t0684890, target=FGSG_02807, score=4, range=1684-1702, strand=-1

target  5’ UGGUA-CAAGAAGUCGCGGU 3’
            :..: ::::::::::::
query   3’ CCUGUGGUUCUUCAGCGCCU 5’

No results for JS16_t2226066
query=JS16_t2226067, target=FGSG_01371, score=1.5, range=1111-1129, strand=1

target  5’ AUGGAUCUAGACGACAUCC 3’
           :::. :::::::::::::
query   3’ UACUAAGAUCUGCUGUAGG 5’

query=JS16_t2226067, target=FGSG_11690, score=2, range=3869-3886, strand=1

target  5’ AU-AUCCUAGACGACAUCC 3’
           :: :: ::::::::::::
query   3’ UACUAAGAUCUGCUGUAGG 5’

query=JS16_t2226067, target=FGSG_06097, score=2.5, range=3616-3634, strand=1

target  5’ GUGAUACUAGACGACAUCA 3’
           .:::: ::::::::::::
query   3’ UACUAAGAUCUGCUGUAGG 5’

query=JS16_t2226067, target=FGSG_01320, score=2.5, range=2283-2301, strand=-1

target  5’ ACGGUUCUGGACGACAUCC 3’
           : :.::::.::::::::::
query   3’ UACUAAGAUCUGCUGUAGG 5’

query=JS16_t2226067, target=FGSG_01713, score=3, range=268-286, strand=1

target  5’ AAGAAUCUGGACGACAUCC 3’
           : :: :::.::::::::::
query   3’ UACUAAGAUCUGCUGUAGG 5’

query=JS16_t2226067, target=FGSG_04263, score=4, range=3112-3130, strand=1

target  5’ CUGCUUUUGGACGACAUCC 3’
            :: ::.:.::::::::::
query   3’ UACUAAGAUCUGCUGUAGG 5’

query=JS16_t2226067, target=FGSG_11683, score=4, range=1537-1555, strand=1

target  5’ AUGUUUCUAGGCGACAUUA 3’
           ::: ::::::.::::::.
query   3’ UACUAAGAUCUGCUGUAGG 5’

query=JS16_t2226067, target=FGSG_09286, score=4, range=521-538, strand=1

target  5’ AUGAUCCUAG-CGACAUCG 3’
           ::::: :::: :::::::
query   3’ UACUAAGAUCUGCUGUAGG 5’

query=JS16_t2226067, target=FGSG_05326, score=4, range=846-864, strand=-1

target  5’ ACGAUUCUUGACGACAUCA 3’
           : :::::: :::::::::
query   3’ UACUAAGAUCUGCUGUAGG 5’

query=JS16_t0684892, target=FGSG_04246, score=3, range=546-566, strand=-1

target  5’ CUUCCUCUUCAGACUUUC 3’
            :  :::::::.::::::
query   3’ UACCGAGAAGUUUGAAAG 5’

query=JS16_t1392257, target=FGSG_12381, score=4, range=548-565, strand=-1

target  5’ AUGUCUCUUCACACUUUG 3’
           ::: ::::::: :::::
query   3’ UACCGAGAAGUUUGAAAG 5’

query=JS16_t1392257, target=FGSG_09445, score=4, range=1385-1403, strand=-1

target  5’ UUGAUAUCUUCAAACUUUU 3’
            :: . ::::::::::::.
query   3’ UACCG-AGAAGUUUGAAAG 5’

query=JS16_t1392257, target=FGSG_08710, score=4, range=737-754, strand=-1

target  5’ AGGUCUCUUCAAACUUGC 3’
           : : ::::::::::: :
query   3’ UACCGAGAAGUUUGAAAG 5’

query=JS16_t1392257, target=FGSG_13401, score=4, range=1927-1945, strand=-1

target  5’ AUCGCUUCUUCAAAAUUUC 3’
           :: :: :::::::: ::::
query   3’ UACCG-AGAAGUUUGAAAG 5’

query=JS16_t1392257, target=FGSG_02753, score=4, range=339-357, strand=-1

target  5’ AAGUCUCUUCAAACUCUUC 3’
           : : ::::::::::: :::
query   3’ UACCGAGAAGUUUGA-AAG 5’

query=JS16_t1392257, target=FGSG_04064, score=4, range=346-364, strand=-1

target  5’ ACGGCGCUUCAAACUUAUC 3’
           : ::: :::::::::: ::
query   3’ UACCGAGAAGUUUGAA-AG 5’

query=JS16_t1392257, target=FGSG_02944, score=4, range=1118-1135, strand=-1

target  5’ CUGAGUCUUCAAACUUUG 3’
            ::  ::::::::::::
query   3’ UACCGAGAAGUUUGAAAG 5’

query=JS16_t1392257, target=FGSG_08819, score=4, range=835-851, strand=-1

target  5’ AU-GCUCUUCAAACAUUG 3’
           :: ::::::::::: ::
query   3’ UACCGAGAAGUUUGAAAG 5’

query=JS16_t1392257, target=FGSG_07230, score=4, range=416-432, strand=-1

target  5’ AU-GAUCUUCAAACAUUC 3’
           :: : ::::::::: :::
query   3’ UACCGAGAAGUUUGAAAG 5’

query=JS16_t1392257, target=FGSG_09312, score=4, range=910-926, strand=-1

target  5’ AUGGCUC-UCAAACUCUC 3’
           ::::::: ::::::: ::
query   3’ UACCGAGAAGUUUGAAAG 5’

No results for JS16_t0366869
No results for JS16_t2288399
No results for JS16_t0801934
query=JS16_t1392382, target=FGSG_13479, score=3, range=623-640, strand=-1

target  5’ GUACGGGUAUGCGGACAA 3’
           ..:  :::::::::::::
query   3’ UGUCGCCAUACGCCUGUU 5’

query=JS16_t1392382, target=FGSG_06547, score=3.5, range=414-431, strand=-1

target  5’ UCAUUGGUAUGCGGACAU 3’
            :: .:::::::::::
query   3’ UGUCGCCAUACGCCUGUU 5’

query=JS16_t1392382, target=FGSG_06743, score=4, range=333-350, strand=1

target  5’ CCAGCGUUAUGCGGACGA 3’
            ::::: ::::::::.:
query   3’ UGUCGCCAUACGCCUGUU 5’

query=JS16_t1392382, target=FGSG_09607, score=4, range=5750-5767, strand=-1

target  5’ UAAGAGGUGUGCGGACAA 3’
             :: :::.::::::::
query   3’ UGUCGCCAUACGCCUGUU 5’

query=JS16_t1392382, target=FGSG_09106, score=4, range=957-974, strand=-1
```

```
target  5' CCAACAGGCGCAAGUUUACCA 3'
           ::::: : ::::::::::::
query   3' GGUUG-CGGCGUUCAAAUGGG 5'

query=JS16_t0684892, target=FGSG_09963, score=3.5, range=496-514, strand=1

target  5' ACAA-GUCGCAAGUUUACCG 3'
            ::: :.::::::::::::
query   3' GGUUGCGGCGUUCAAAUGGG 5'

query=JS16_t0684892, target=FGSG_11147, score=3.5, range=1565-1585, strand=1

target  5' UCAACGCUCGCAAGUUUACUA 3'
           .:::::: :::::::::::.
query   3' GGUUGCG-GCGUUCAAAUGGG 5'

query=JS16_t0684892, target=FGSG_09863, score=4, range=408-427, strand=1

target  5' UCGACGACGCAAGUUUGCCG 3'
           .:.::: :::::::::.::
query   3' GGUUGCGGCGUUCAAAUGGG 5'

query=JS16_t0684892, target=FGSG_04411, score=4, range=424-442, strand=1

target  5' CCGAC-CUGCAAGUUUACUU 3'
           ::.:: :.::::::::::..
query   3' GGUUGCGGCGUUCAAAUGGG 5'

query=JS16_t0684892, target=FGSG_13191, score=4, range=1-19, strand=1

target  5' AUGACG-CGCAAGUUUAUCC 3'
            ..::: :::::::::.::
query   3' GGUUGCGGCGUUCAAAUGGG 5'

query=JS16_t0684892, target=FGSG_10348, score=4, range=894-914, strand=-1

target  5' UCAGCACGCGCAAGUUUAUCC 3'
           .::.: : ::::::::::.::
query   3' GGUUGCG-GCGUUCAAAUGGG 5'

No results for JS16_t0684896
query=JS16_t0467219, target=FGSG_03421, score=4, range=907-929, strand=1

target  5' UUGGCACAAGCACUCGAUUGGCC 3'
           ::::::::: :::::::::::
query   3' AACCGUGUUGGUGAGCUAACCAU 5'

query=JS16_t0467219, target=FGSG_10582, score=4, range=162-184, strand=-1

target  5' UUGGAAUAGCGACUCGAUUGGUA 3'
           :::: :.:.: ::::::::::::
query   3' AACCGUGUUGGUGAGCUAACCAU 5'

No results for JS16_t3122277
No results for JS16_t1329746
query=JS16_t1329754, target=FGSG_03713, score=3.5, range=815-834, strand=1

target  5' CUGUCCAGUCCUCCGUCGAU 3'
            :: : :::::::::::::.
query   3' CAC-GUUCAGGAGGCAGCUG 5'

query=JS16_t1329754, target=FGSG_08481, score=3.5, range=2533-2551, strand=-1

target  5' UUCCGAGUCCUUCGUCGAC 3'
            : :.::::::.:::::::
query   3' CACGUUCAGGAGGCAGCUG 5'

query=JS16_t1329754, target=FGSG_03761, score=4, range=864-882, strand=1

target  5' CUGCACGUUCUCCGUCGGC 3'
            :::: ::.::::::::.:
query   3' CACGUUCAGGAGGCAGCUG 5'

query=JS16_t1329754, target=FGSG_08403, score=4, range=615-633, strand=1

target  5' GCGCAAGUCUUCCGCCGAC 3'
           : :::::::.:::: ::::
query   3' CACGUUCAGGAGGCAGCUG 5'

query=JS16_t1329754, target=FGSG_02893, score=4, range=1255-1274, strand=1

target  5' GUGUCACGUCCUCUGUCGGC 3'
           ::: :: ::::::.::::.:
query   3' CAC-GUUCAGGAGGCAGCUG 5'

query=JS16_t1329754, target=FGSG_06832, score=4, range=4-21, strand=1

target  5' GCGCAAGUCCUCC-UUGAC 3'
           : ::::::::::: :.:::
query   3' CACGUUCAGGAGGCAGCUG 5'

query=JS16_t1329754, target=FGSG_07635, score=4, range=1015-1033, strand=1
```

```
target  5' ACCCAGGUAUGUGGACAA 3'
           ::   ::::::.::::::
query   3' UGUCGCCAUACGCCUGUU 5'

query=JS16_t1392382, target=FGSG_07859, score=4, range=46-62, strand=-1

target  5' AGAGCGGUG-GCGGACAA 3'
           : ::::::. ::::::::
query   3' UGUCGCCAUACGCCUGUU 5'

No results for JS16_t4081246
query=JS16_t1392395, target=FGSG_09026, score=4, range=570-590, strand=-1

target  5' GCAACGUCUUCCCCGU-ACUCU 3'
           :::::.: :::::::: ::::.
query   3' CGUUGUAAAAGGGGCACUGAGG 5'

query=JS16_t1392416, target=FGSG_07543, score=3.5, range=750-771, strand=1

target  5' UGAACUCUUCACUGACAAGUCA 3'
           .  :::: ::::::::::::::
query   3' GAUUGA-AAGUGACUGUUCAGC 5'

query=JS16_t1392416, target=FGSG_07471, score=3.5, range=296-315, strand=1

target  5' CU-GCUUACACUGACAAGUCU 3'
           :: .::: ::::::::::::
query   3' GAUUGAAAGUGACUGUUCAGC 5'

No results for JS16_t1392426
query=JS16_t1392492, target=FGSG_09064, score=4, range=727-746, strand=1

target  5' GCAAUUGGCGCGCAA-UGUGA 3'
           ::: :::::::.::: :::::
query   3' CGUCAACCGCGUGUUGACACU 5'

query=JS16_t1392492, target=FGSG_08510, score=4, range=394-414, strand=-1

target  5' CUGGUUGACGCACAGCUGUGA 3'
            ..:::: ::::::.::::::
query   3' CGUCAACCGCGUGUUGACACU 5'

query=JS16_t1392497, target=FGSG_11815, score=3, range=427-446, strand=1

target  5' GC-GGCGGUCAUCUUCUGGAU 3'
           .: :.:::::::::::::.::
query   3' UGUCUGCCAGUAGAAGACUUA 5'

query=JS16_t1392497, target=FGSG_13912, score=3.5, range=103-122, strand=-1

target  5' GCAUGU-GUCAUCUUCUGAAU 3'
           .:: ..  ::::::::::::::
query   3' UGUCUGCCAGUAGAAGACUUA 5'

query=JS16_t1392497, target=FGSG_04587, score=4, range=656-677, strand=-1

target  5' ACAGGUUCGUCAUCUUCUGAGU 3'
           ::::. .  :::::::::::.:
query   3' UGUCU-GCCAGUAGAAGACUUA 5'

query=JS16_t1392497, target=FGSG_03718, score=4, range=1193-1212, strand=-1

target  5' ACA-AGGUUCAUCUUCUGAAC 3'
           ::: : : ::::::::::::
query   3' UGUCUGCCAGUAGAAGACUUA 5'

query=JS16_t3184823, target=FGSG_10635, score=3, range=192-211, strand=-1

target  5' AGCCGGGUUUAGUAUUGAAC 3'
           ::::::::::::::.::: :
query   3' UCGGCCCAAAUCAUGACUGG 5'

query=JS16_t3184823, target=FGSG_13683, score=3.5, range=437-457, strand=-1

target  5' AGCUGGGGUUUAGCACUGACC 3'
           :::.  :::::::: :::::::
query   3' UCGG-CCCAAAUCAUGACUGG 5'

query=JS16_t3184823, target=FGSG_02949, score=4, range=1157-1175, strand=-1

target  5' AG-UGGGUUUGGUGCUGACU 3'
           :: .::::::.::.:::::.
query   3' UCGGCCCAAAUCAUGACUGG 5'

No results for JS16_t2288684
No results for JS16_t3184864
No results for JS16_t2288728
No results for JS16_t0337367
query=JS16_t1392713, target=FGSG_11831, score=3, range=385-402, strand=1

target  5' UAUG-CUUUCGGCAUUGGC 3'
```

```
target  5’ GUUCUUGUCUUCCGUCGAC 3’
           :: :  :::.:::::::::
query   3’ CACGUUCAGGAGGCAGCUG 5’

query=JS16_t1329754, target=FGSG_05848, score=4, range=244-261, strand=1

target  5’ CUGC-AGUCCUCCGUCAAC 3’
            ::: ::::::::::: ::
query   3’ CACGUUCAGGAGGCAGCUG 5’

query=JS16_t1329754, target=FGSG_12746, score=4, range=208-225, strand=1

target  5’ GUGCAACUCC-CCGUCGAC 3’
           :::::: ::: ::::::::
query   3’ CACGUUCAGGAGGCAGCUG 5’

query=JS16_t1329754, target=FGSG_00885, score=4, range=754-773, strand=-1

target  5’ UUGCCAGCUCCUCCGUCGAC 3’
            ::: :: ::::::::::::
query   3’ CACGUUC-AGGAGGCAGCUG 5’

query=JS16_t1329754, target=FGSG_05555, score=4, range=2242-2259, strand=-1

target  5’ GGGCAA-UCCUCCGUCGAG 3’
           : :::: :::::::::::
query   3’ CACGUUCAGGAGGCAGCUG 5’

No results for JS16_t0240244
No results for JS16_t4019089
query=JS16_t3122390, target=FGSG_07857, score=3, range=2268-2287, strand=-1

target  5’ UGCGCGUUGUCGUUUCAAUG 3’
           :::: : ::.::::::::::
query   3’ ACGCACUACGGCAAAGUUAC 5’

query=JS16_t3122390, target=FGSG_06051, score=4, range=1313-1332, strand=1

target  5’ UGCACGAUGCCGUUACAAUG 3’
           :::  ::::::::: :::::
query   3’ ACGCACUACGGCAAAGUUAC 5’

query=JS16_t3122390, target=FGSG_06228, score=4, range=913-931, strand=-1

target  5’ UG-GUGAUGCCGUUUCGCUG 3’
           :: :::::::::::::. ::
query   3’ ACGCACUACGGCAAAGUUAC 5’

No results for JS16_t3122450
No results for JS16_t4019202
query=JS16_t0684947, target=FGSG_06212, score=2, range=1604-1621, strand=1

target  5’ AACAUACAUUACGUGUCA 3’
           :::  :::::::::::::
query   3’ UUGCUUGUAAUGCACAGU 5’

query=JS16_t0684947, target=FGSG_07332, score=4, range=1462-1479, strand=1

target  5’ AGCGGACAUUAUGUUUCA 3’
           :.::.::::::.:: :::
query   3’ UUGCUUGUAAUGCACAGU 5’

query=JS16_t0684947, target=FGSG_01439, score=4, range=1898-1915, strand=1

target  5’ UGCGAACAUUACUUGUCG 3’
            .:::::::::: ::::.
query   3’ UUGCUUGUAAUGCACAGU 5’

query=JS16_t0684947, target=FGSG_07121, score=4, range=1748-1764, strand=1

target  5’ AACGAACAUUGU-UGUCA 3’
           ::::::::::.. :::::
query   3’ UUGCUUGUAAUGCACAGU 5’

query=JS16_t0684947, target=FGSG_10823, score=4, range=472-488, strand=1

target  5’ AGCGAACAUUAUG-GUCG 3’
           :.:::::::::.: :::.
query   3’ UUGCUUGUAAUGCACAGU 5’

query=JS16_t0684947, target=FGSG_12976, score=4, range=332-348, strand=1

target  5’ AACG-ACAUGACGUGUUA 3’
           :::: :::: ::::::.:
query   3’ UUGCUUGUAAUGCACAGU 5’

query=JS16_t0684947, target=FGSG_03470, score=4, range=265-283, strand=1

target  5’ AACACACAUUCACGUGUCA 3’
           :::  ::::: ::::::::
query   3’ UUGCUUGUAA-UGCACAGU 5’
```

```
            ::: :::::::::::.::
query   3’ UUACAGAAAGCCGUAAUCG 5’

query=JS16_t1392713, target=FGSG_03131, score=3.5, range=52-69, strand=-1

target  5’ GAUGUCUUU-GGCAUUGGC 3’
           .:::::::: ::::::.::
query   3’ UUACAGAAAGCCGUAAUCG 5’

query=JS16_t1392713, target=FGSG_08739, score=3.5, range=1690-1708, strand=-1

target  5’ AACGUAUUUCGGCAUUGGU 3’
           :: :: ::::::::::.:.
query   3’ UUACAGAAAGCCGUAAUCG 5’

query=JS16_t1392713, target=FGSG_07307, score=3.5, range=1270-1288, strand=-1

target  5’ CGUGGGUUUCGGCAUUAGC 3’
            .::  :::::::::::::
query   3’ UUACAGAAAGCCGUAAUCG 5’

query=JS16_t1392713, target=FGSG_10548, score=4, range=5310-5328, strand=1

target  5’ ACGGGCUUUCGGCAUUAGG 3’
           :  : :::::::::::::
query   3’ UUACAGAAAGCCGUAAUCG 5’

query=JS16_t1392713, target=FGSG_02919, score=4, range=286-305, strand=-1

target  5’ AGUGGUCUUUCGGCAUCAGU 3’
           :.: :::::::::::: ::.
query   3’ UUA-CAGAAAGCCGUAAUCG 5’

query=JS16_t1392713, target=FGSG_12549, score=4, range=1620-1637, strand=-1

target  5’ ACUG-CUUUCGUCAUUAGC 3’
           : :: :::::: :::::::
query   3’ UUACAGAAAGCCGUAAUCG 5’

No results for JS16_t2288793
query=JS16_t1392786, target=FGSG_00737, score=3, range=3035-3053, strand=1

target  5’ UUGGUGU-UGGAACUCGAGC 3’
           :..:::: ::::::::::::
query   3’ AGUCACAUACCUUGAGCUCG 5’

query=JS16_t1392786, target=FGSG_06206, score=3.5, range=349-369, strand=-1

target  5’ UUCGUGUGAUGGAACUCGAGA 3’
           :. :::: ::::::::::::
query   3’ AGUCACA-UACCUUGAGCUCG 5’

query=JS16_t1392786, target=FGSG_04426, score=4, range=862-880, strand=-1

target  5’ GCAGU-AAUGGAACUUGAGC 3’
            ::::  ::::::::.::::
query   3’ AGUCACAUACCUUGAGCUCG 5’

query=JS16_t4081691, target=FGSG_05763, score=3.5, range=1690-1708, strand=1

target  5’ AUUUUUAGUUACUGUGGUG 3’
           ::::: :::::: :::::.
query   3’ UAAAAUUCAAUGCCACCAU 5’

query=JS16_t4081691, target=FGSG_11036, score=4, range=409-427, strand=1

target  5’ GGUUGGAGUUACGGUGGUU 3’
           .  :: .::::::::::::
query   3’ UAAAAUUCAAUGCCACCAU 5’

query=JS16_t4081691, target=FGSG_09558, score=4, range=323-340, strand=1

target  5’ AUCUU-GGUUAUGGUGGUA 3’
           :: :: .::::.:::::::
query   3’ UAAAAUUCAAUGCCACCAU 5’

query=JS16_t4081691, target=FGSG_09262, score=4, range=1518-1536, strand=1

target  5’ ACUUCAAGUUACGGUGGCA 3’
           : :: :::::::::::: :
query   3’ UAAAAUUCAAUGCCACCAU 5’

query=JS16_t4081691, target=FGSG_03158, score=4, range=1219-1237, strand=-1

target  5’ UGUUCAAGUUGCGGUGGUA 3’
             :: :::::.::::::::
query   3’ UAAAAUUCAAUGCCACCAU 5’

query=JS16_t2288936, target=FGSG_01485, score=3.5, range=158-179, strand=1

target  5’ GCUGGAAGGAUGCUUCAGGAAC 3’
            :.  ::: :::::::::::::
```

```
query=JS16_t0684947, target=FGSG_12656, score=4, range=32-50, strand=-1

target  5’ AACGCGACAUUGUGUGUCG 3’
           :::: .:::::..:::::.
query   3’ UUGC-UUGUAAUGCACAGU 5’

query=JS16_t0684947, target=FGSG_00018, score=4, range=197-213, strand=-1

target  5’ AAU-AGCAUUAUGUGUCG 3’
           ::. :.:::::.:::::.
query   3’ UUGCUUGUAAUGCACAGU 5’

query=JS16_t0684947, target=FGSG_02556, score=4, range=350-367, strand=-1

target  5’ CACCAACAUUACCUGUCA 3’
            :: :::::::: :::::
query   3’ UUGCUUGUAAUGCACAGU 5’

No results for JS16_t1329981
No results for JS16_t2226470
No results for JS16_t0194428
query=JS16_t3122655, target=FGSG_11428, score=2, range=846-866, strand=1

target  5’ UGUGCCUACUCAUGAAGACGA 3’
           ::..:: ::::::::::::::
query   3’ ACGUGG-UGAGUACUUCUGCU 5’

query=JS16_t3122655, target=FGSG_11659, score=2, range=359-378, strand=-1

target  5’ UGUGCCACUCGUGAAGACGA 3’
           ::..::::::.:::::::::
query   3’ ACGUGGUGAGUACUUCUGCU 5’

query=JS16_t3122655, target=FGSG_10283, score=2.5, range=1565-1583, strand=1

target  5’ UGUA-CACUCAUGAAGACGC 3’
           ::.: ::::::::::::::
query   3’ ACGUGGUGAGUACUUCUGCU 5’

query=JS16_t3122655, target=FGSG_08148, score=3.5, range=105-125, strand=1

target  5’ UGUUGUCACUCAUGAGGACGA 3’
           :: ...::::::::.:::::
query   3’ AC-GUGGUGAGUACUUCUGCU 5’

query=JS16_t3122655, target=FGSG_11258, score=3.5, range=369-389, strand=1

target  5’ UGUCAUCACUCUUGAAGACGA 3’
           :: ::.::::: :::::::::
query   3’ AC-GUGGUGAGUACUUCUGCU 5’

query=JS16_t3122655, target=FGSG_01525, score=3.5, range=374-393, strand=-1

target  5’ UGCACCGAUCAUGAAGAUGA 3’
           ::::::. :::::::::.::
query   3’ ACGUGGUGAGUACUUCUGCU 5’

query=JS16_t3122655, target=FGSG_00985, score=3.5, range=1056-1075, strand=-1

target  5’ UGCGCCUCUCACGAAGACGA 3’
           :::.:: :::: ::::::::
query   3’ ACGUGGUGAGUACUUCUGCU 5’

query=JS16_t3122655, target=FGSG_06233, score=4, range=453-473, strand=1

target  5’ UGCAUCGCUCAUGAAUGAUGA 3’
           ::::.:.:::::::: ::.::
query   3’ ACGUGGUGAGUACUU-CUGCU 5’

query=JS16_t3122655, target=FGSG_09696, score=4, range=622-640, strand=1

target  5’ UGC-CCACUCAUGGAGAAGA 3’
           ::: :::::::::.::: ::
query   3’ ACGUGGUGAGUACUUCUGCU 5’

query=JS16_t3122655, target=FGSG_08638, score=4, range=1764-1783, strand=-1

target  5’ UGUCCCGCUCAUAAAGACGA 3’
           ::. ::.::::: :::::::
query   3’ ACGUGGUGAGUACUUCUGCU 5’

query=JS16_t3122655, target=FGSG_03540, score=4, range=471-489, strand=-1

target  5’ UG-ACUGCUCAUGGAGACGC 3’
           :: ::..::::::.:::::
query   3’ ACGUGGUGAGUACUUCUGCU 5’

query=JS16_t3122655, target=FGSG_12077, score=4, range=266-284, strand=-1

target  5’ UUCAUU-CUCAUGGAGACGA 3’
           : ::.. ::::::.::::::
```

```
query   3’ AGGGCUUGCUACGAAGUCCUUG 5’

query=JS16_t2288936, target=FGSG_08062, score=4, range=1022-1043, strand=1

target  5’ UCACGAAAGAUGCUUCAGGAGA 3’
           :: :::: ::::::::::::.
query   3’ AGGGCUUGCUACGAAGUCCUUG 5’

query=JS16_t2288936, target=FGSG_00721, score=4, range=555-575, strand=1

target  5’ ACCCGAA-GAUGAUUCAGGAAC 3’
            :::::: :::: :::::::::
query   3’ AGGGCUUGCUACGAAGUCCUUG 5’

query=JS16_t2288936, target=FGSG_07897, score=4, range=474-494, strand=1

target  5’ UCCCGUA-GAUGCUUCAGGAGA 3’
           ::::: : ::::::::::::.
query   3’ AGGGCUUGCUACGAAGUCCUUG 5’

query=JS16_t3185126, target=FGSG_13396, score=2.5, range=1025-1042, strand=-1

target  5’ AAGAU-UCAUCAUUCCAUC 3’
           ::.:: ::::::::::::
query   3’ UUUUAUAGUAGUAAGGUAU 5’

query=JS16_t3185126, target=FGSG_01560, score=3, range=1212-1229, strand=-1

target  5’ AAAAUAUCAUCAUUCU-UA 3’
           :::::::::::::::. ::
query   3’ UUUUAUAGUAGUAAGGUAU 5’

query=JS16_t3185126, target=FGSG_02748, score=3, range=956-973, strand=-1

target  5’ AGGAUAUCAUC-UUCCAUA 3’
           :..::::::::: :::::::
query   3’ UUUUAUAGUAGUAAGGUAU 5’

query=JS16_t3185126, target=FGSG_06340, score=3.5, range=275-293, strand=1

target  5’ AUCGUAUCGUCAUUCCAUA 3’
           :  .::::.::::::::::
query   3’ UUUUAUAGUAGUAAGGUAU 5’

query=JS16_t3185126, target=FGSG_13622, score=3.5, range=971-990, strand=-1

target  5’ AAGAUCAUCAUCGUUCCAUC 3’
           ::.:: ::::::.::::::
query   3’ UUUUA-UAGUAGUAAGGUAU 5’

query=JS16_t3185126, target=FGSG_10294, score=4, range=122-140, strand=1

target  5’ AACGUGUCGUCAUUCCAUC 3’
           :: .:.::.:::::::::
query   3’ UUUUAUAGUAGUAAGGUAU 5’

query=JS16_t3185126, target=FGSG_01352, score=4, range=896-913, strand=1

target  5’ ACAAUAUCAUCA-UCCAUU 3’
           : :::::::::: :::::
query   3’ UUUUAUAGUAGUAAGGUAU 5’

query=JS16_t3185126, target=FGSG_00661, score=4, range=1546-1565, strand=-1

target  5’ AGAGUAUCAUCAUCUUCAUA 3’
           :.:.:::::::::: :.::::
query   3’ UUUUAUAGUAGUA-AGGUAU 5’

query=JS16_t3185126, target=FGSG_09219, score=4, range=508-525, strand=-1

target  5’ AAAAU-UCAUCUUUCCAUU 3’
           ::::: ::::: ::::::
query   3’ UUUUAUAGUAGUAAGGUAU 5’

No results for JS16_t4081804
No results for JS16_t0474798
query=JS16_t1392937, target=FGSG_01248, score=3.5, range=489-511, strand=1

target  5’ UGAGCGACGCUUCGGAUGCUACC 3’
           :: .:: :: :::::::::::::
query   3’ ACCUGCCGCCAAGCCUACGAUGG 5’

query=JS16_t1392948, target=FGSG_07067, score=3, range=25-44, strand=-1

target  5’ GCUCGCGAGACGCCUUUGCG 3’
           : ::::::::::::: ::::
query   3’ CAAGCGCUCUGCGGACACGC 5’

query=JS16_t1392948, target=FGSG_05523, score=3.5, range=1280-1298, strand=-1

target  5’ GUUU-CAAGACGCUUGUGCG 3’
           :::. : ::::::.::::::
```

```
query    3' ACGUGGUGAGUACUUCUGCU 5'

query=JS16_t0576291, target=FGSG_03032, score=3, range=225-244, strand=-1

target  5' CGAGAUGGACCGUCUGUGCU 3'
             .:::: ::::::::::::.
query    3' CUUCUA-CUGGCAGACACGG 5'

query=JS16_t0576291, target=FGSG_10442, score=3, range=381-399, strand=-1

target  5' GUCGAUGACCGUCUGUGUC 3'
            :  ::::::::::::::.:
query    3' CUUCUACUGGCAGACACGG 5'

query=JS16_t0576291, target=FGSG_13414, score=3.5, range=219-237, strand=-1

target  5' GGACCGGACCGUCUGUGCC 3'
            :.:    :::::::::::::
query    3' CUUCUACUGGCAGACACGG 5'

query=JS16_t0576291, target=FGSG_00404, score=4, range=1077-1095, strand=1

target  5' GAGGAUGACUGUCUCUGCU 3'
            ::.::::::.:::: :::.
query    3' CUUCUACUGGCAGACACGG 5'

query=JS16_t0576291, target=FGSG_13831, score=4, range=2175-2193, strand=1

target  5' GAGGAUGACUGUGUGUGCU 3'
            ::.::::::.:: :::::.
query    3' CUUCUACUGGCAGACACGG 5'

query=JS16_t0576291, target=FGSG_10909, score=4, range=141-158, strand=1

target  5' CAAGAUGACUGU-UGUGCC 3'
             ::::::::.:: ::::::
query    3' CUUCUACUGGCAGACACGG 5'

query=JS16_t0576291, target=FGSG_01129, score=4, range=523-540, strand=1

target  5' GAGGACGACCGUCUG-GCU 3'
            ::.:: ::::::::: ::.
query    3' CUUCUACUGGCAGACACGG 5'

query=JS16_t0576291, target=FGSG_13709, score=4, range=994-1011, strand=1

target  5' GAAGA-GACUGUCUGUGAC 3'
            ::::: :::.:::::::: :
query    3' CUUCUACUGGCAGACACGG 5'

query=JS16_t0576291, target=FGSG_09151, score=4, range=736-754, strand=-1

target  5' GUAGGUGAUCGUCUGUGUU 3'
            : ::.:::.::::::::..
query    3' CUUCUACUGGCAGACACGG 5'

query=JS16_t0576291, target=FGSG_10530, score=4, range=6234-6253, strand=-1

target  5' GAAGAUGGCCGUCCUGUGCA 3'
            :::::::.:::: ::::::
query    3' CUUCUACUGGCA-GACACGG 5'

query=JS16_t0576291, target=FGSG_06417, score=4, range=3772-3790, strand=-1

target  5' GAUGAGGAUCGUUUGUGCC 3'
            :: :: ::.:::.::::::
query    3' CUUCUACUGGCAGACACGG 5'

query=JS16_t0576291, target=FGSG_02048, score=4, range=1436-1453, strand=-1

target  5' GAAGAGGAUCGUCUG-GCC 3'
            ::::: ::.:::::: :::
query    3' CUUCUACUGGCAGACACGG 5'

query=JS16_t0576291, target=FGSG_10922, score=4, range=559-576, strand=-1

target  5' GACGAUG-CCGUUUGUGCC 3'
            :: :::: ::::.::::::
query    3' CUUCUACUGGCAGACACGG 5'

query=JS16_t0576291, target=FGSG_11205, score=4, range=124-141, strand=-1

target  5' GAUGA-GGCCGUUUGUGCC 3'
            :: :: :.::::.::::::
query    3' CUUCUACUGGCAGACACGG 5'

query=JS16_t0576291, target=FGSG_01811, score=4, range=343-360, strand=-1

target  5' GAAUAUGACC-UUUGUGCC 3'
            ::: :::::: :.::::::
query    3' CUUCUACUGGCAGACACGG 5'

query    3' CAAGCGCUCUGCGGACACGC 5'

query=JS16_t1392948, target=FGSG_13604, score=4, range=354-372, strand=1

target  5' GUUC-CAAGACGCCUGUGUU 3'
            :::: : :::::::::::.
query    3' CAAGCGCUCUGCGGACACGC 5'

No results for JS16_t1392974
No results for JS16_t3185269
query=JS16_t2289091, target=FGSG_11941, score=4, range=328-350, strand=-1

target  5' GAAAUGUCUUCUGAAACUUGAAG 3'
             :: .:.::::.:::::::::::
query    3' GUUAGCGGAAGGCUUUGAACUUC 5'

query=JS16_t0337383, target=FGSG_12798, score=3.5, range=1192-1212, strand=-1

target  5' UGCGUUAAUGAUUUUGCCACA 3'
            ..:::.: ::::::::::::
query    3' GUGCAGUAACUAAAACGGUGA 5'

query=JS16_t0337383, target=FGSG_07100, score=3.5, range=1159-1179, strand=-1

target  5' UCCGGCAUUGAUUUUGCCAUU 3'
            . :: ::::::::::::::.:
query    3' GUGCAGUAACUAAAACGGUGA 5'

query=JS16_t0337383, target=FGSG_00270, score=3.5, range=988-1008, strand=-1

target  5' AACGACGCUGAUUUUGCCACU 3'
             ::: :. :::::::::::::
query    3' GUGCAGUAACUAAAACGGUGA 5'

query=JS16_t2289149, target=FGSG_04589, score=2.5, range=707-724, strand=1

target  5' CCUGGUAUAUGGACGAUC 3'
            ::::.::::::: :::::
query    3' GGACUAUAUACCGGCUAG 5'

query=JS16_t2289149, target=FGSG_09617, score=3, range=641-657, strand=1

target  5' UCUGGUAUAUGGCC-AUC 3'
            .:::.:::::::::: :::
query    3' GGACUAUAUACCGGCUAG 5'

query=JS16_t2289149, target=FGSG_00915, score=3, range=325-343, strand=1

target  5' CCUGUAUAUAUGGCCGAGC 3'
            :::: :::::::::::: :
query    3' GGAC-UAUAUACCGGCUAG 5'

query=JS16_t2289149, target=FGSG_08004, score=3.5, range=801-818, strand=1

target  5' UUUGGUUUAUGGCCGAUC 3'
            ..::.: :::::::::::
query    3' GGACUAUAUACCGGCUAG 5'

query=JS16_t2289149, target=FGSG_03351, score=3.5, range=876-893, strand=1

target  5' GCUGAUAUAUGGCCCAUU 3'
             ::::::::::::: ::.
query    3' GGACUAUAUACCGGCUAG 5'

query=JS16_t2289149, target=FGSG_06202, score=3.5, range=721-738, strand=-1

target  5' AUCGAUAUAUGGCCGGUC 3'
             .  ::::::::::::.::
query    3' GGACUAUAUACCGGCUAG 5'

query=JS16_t2289149, target=FGSG_01848, score=4, range=619-636, strand=1

target  5' CCUGAUGUAUGGUCCAUC 3'
            ::::::.:::::.: :::
query    3' GGACUAUAUACCGGCUAG 5'

query=JS16_t2289149, target=FGSG_07764, score=4, range=1261-1278, strand=1

target  5' CCUCAUAUAUGGUGGAUC 3'
            ::: ::::::::. ::::
query    3' GGACUAUAUACCGGCUAG 5'

query=JS16_t2289149, target=FGSG_05322, score=4, range=2289-2307, strand=1

target  5' CCAGAUGUAUGGCCGUAUC 3'
            :: :::.:::::::: :::
query    3' GGACUAUAUACCGGC-UAG 5'

query=JS16_t2289149, target=FGSG_08105, score=4, range=1210-1227, strand=1

target  5' GCUGAUAUCUGGCCGAUG 3'
             ::::::: ::::::::
```

```
No results for JS16_t0364793
query=JS16_t0467279, target=FGSG_11228, score=1, range=925-942, strand=1

target  5' UUUGACGAUAAGAAGAAG 3'
           :.:::::::::::::::.
query   3' AGACUGCUAUUCUUCUUU 5'

query=JS16_t0467279, target=FGSG_09413, score=1.5, range=6-23, strand=-1

target  5' UCUGACGAUAAGAAGGAG 3'
           ::::::::::::::.:.
query   3' AGACUGCUAUUCUUCUUU 5'

query=JS16_t0467279, target=FGSG_02035, score=2, range=3218-3234, strand=1

target  5' UC-GAUGAUAAGAAGAAA 3'
           :: ::.:::::::::::
query   3' AGACUGCUAUUCUUCUUU 5'

query=JS16_t0467279, target=FGSG_07232, score=2.5, range=2017-2034, strand=1

target  5' UCUGGCGACAAGAAGAAA 3'
           ::::.::: :::::::::
query   3' AGACUGCUAUUCUUCUUU 5'

query=JS16_t0467279, target=FGSG_05329, score=2.5, range=572-588, strand=1

target  5' UC-GCCGAUAAGAAGAAG 3'
           :: : ::::::::::::.
query   3' AGACUGCUAUUCUUCUUU 5'

query=JS16_t0467279, target=FGSG_02749, score=2.5, range=1135-1152, strand=-1

target  5' ACUGGCGAUAAGGAGAAA 3'
            :::.:::::::.:::::
query   3' AGACUGCUAUUCUUCUUU 5'

query=JS16_t0467279, target=FGSG_05244, score=3, range=991-1008, strand=1

target  5' UCUGAUGAUGAGAAGGAA 3'
           :::::.:::.:::::.::
query   3' AGACUGCUAUUCUUCUUU 5'

query=JS16_t0467279, target=FGSG_13232, score=3, range=1003-1020, strand=1

target  5' GUUGGCGAUAAGAAGGAA 3'
            .::.:::::::::::.::
query   3' AGACUGCUAUUCUUCUUU 5'

query=JS16_t0467279, target=FGSG_13983, score=3, range=907-924, strand=1

target  5' UCUGCUGAUAAGAAGAGA 3'
           :::: .::::::::::.:
query   3' AGACUGCUAUUCUUCUUU 5'

query=JS16_t0467279, target=FGSG_09835, score=3, range=967-984, strand=1

target  5' UUGGAUGAUAAGAAGAAG 3'
           :.  ::.::::::::::::.
query   3' AGACUGCUAUUCUUCUUU 5'

query=JS16_t0467279, target=FGSG_04040, score=3, range=1672-1689, strand=1

target  5' UCCGACGAUGAGAAGAAU 3'
           :: ::::::.:::::::
query   3' AGACUGCUAUUCUUCUUU 5'

query=JS16_t0467279, target=FGSG_00710, score=3, range=1255-1272, strand=1

target  5' ACAGGCGAUAAGAAGAAG 3'
            : :.:::::::::::::.
query   3' AGACUGCUAUUCUUCUUU 5'

query=JS16_t0467279, target=FGSG_01036, score=3, range=473-489, strand=1

target  5' UCU-AUGGUAAGAAGAAA 3'
           ::: :.:.::::::::::
query   3' AGACUGCUAUUCUUCUUU 5'

query=JS16_t0467279, target=FGSG_02690, score=3, range=1105-1122, strand=1

target  5' UGGGAUGAUAAGAAGAAA 3'
           :  ::.::::::::::::
query   3' AGACUGCUAUUCUUCUUU 5'

query=JS16_t0467279, target=FGSG_02664, score=3, range=626-642, strand=1

target  5' UC-GGCGAUGAGAAGAAG 3'
           :: :.::::.:::::::.
query   3' AGACUGCUAUUCUUCUUU 5'

query=JS16_t0467279, target=FGSG_04274, score=3, range=370-387, strand=1
query   3' GGACUAUAUACCGGCUAG 5'

query=JS16_t2289149, target=FGSG_08169, score=4, range=941-957, strand=1

target  5' CAUUAUAUAUGGCCG-UC 3'
           : : ::::::::::: ::
query   3' GGACUAUAUACCGGCUAG 5'

query=JS16_t2289149, target=FGSG_09098, score=4, range=426-442, strand=1

target  5' CGGGAUAUAUGG-CGAUC 3'
           :  ::::::::: :::::
query   3' GGACUAUAUACCGGCUAG 5'

query=JS16_t2289149, target=FGSG_07758, score=4, range=235-251, strand=1

target  5' CCUGGUGU-UGGCCGAUU 3'
           ::::.:.: ::::::::.
query   3' GGACUAUAUACCGGCUAG 5'

query=JS16_t2289149, target=FGSG_10123, score=4, range=652-669, strand=-1

target  5' CUUGAUGUAUGGCCCAUU 3'
           :.::::.::::::: ::.
query   3' GGACUAUAUACCGGCUAG 5'

query=JS16_t2289149, target=FGSG_03480, score=4, range=1926-1943, strand=-1

target  5' UCUCGUGUAUGGCCGGUC 3'
           .:: .:.::::::::.::
query   3' GGACUAUAUACCGGCUAG 5'

query=JS16_t2289149, target=FGSG_06750, score=4, range=1180-1197, strand=-1

target  5' CAUGAUAUCUGGUCGAUC 3'
           : :::::: :::.:::::
query   3' GGACUAUAUACCGGCUAG 5'

query=JS16_t2289149, target=FGSG_03329, score=4, range=462-478, strand=-1

target  5' CCUGAUAGAUGG-CGAUC 3'
           ::::::: :::: :::::
query   3' GGACUAUAUACCGGCUAG 5'

No results for JS16_t0474829
query=JS16_t1393120, target=FGSG_03100, score=2, range=262-279, strand=-1

target  5' CAUACAUUGUCGAAUGGU 3'
           :::  :::::::::::::
query   3' GUACCUAACAGCUUACCA 5'

query=JS16_t1393120, target=FGSG_00880, score=3, range=1140-1157, strand=-1

target  5' CAUGGGUUGUCAAAUGGU 3'
           :::::.::::: ::::::
query   3' GUACCUAACAGCUUACCA 5'

query=JS16_t1393120, target=FGSG_09752, score=3, range=639-656, strand=-1

target  5' CAUGGAUUGCCGGAUGGU 3'
           ::::::::: ::.:::::
query   3' GUACCUAACAGCUUACCA 5'

query=JS16_t1393120, target=FGSG_04307, score=3, range=18-35, strand=-1

target  5' UGUUGGUUGUCGAAUGGU 3'
           ..: :.::::::::::::
query   3' GUACCUAACAGCUUACCA 5'

query=JS16_t1393120, target=FGSG_09072, score=3, range=175-193, strand=-1

target  5' CUUGGUAUUGUUGAAUGGU 3'
           : ::: :::::.:::::::
query   3' GUACC-UAACAGCUUACCA 5'

query=JS16_t1393120, target=FGSG_12643, score=3.5, range=769-787, strand=1

target  5' UAUGGUAUUGUUGAGUGGU 3'
           .:::: :::::.::.::::
query   3' GUACC-UAACAGCUUACCA 5'

query=JS16_t1393120, target=FGSG_05582, score=3.5, range=1535-1552, strand=1

target  5' UAUGGAUUGUGGAAUGGC 3'
           .::::::::: ::::::
query   3' GUACCUAACAGCUUACCA 5'

query=JS16_t1393120, target=FGSG_00545, score=3.5, range=674-691, strand=-1

target  5' CGUAGAUUGUCGAAGGGU 3'
           :.: :::::::::: :::
query   3' GUACCUAACAGCUUACCA 5'
```

```
target  5’ UCAGACGAUAAGAUGAAA 3’
           :: :::::::::: ::::
query   3’ AGACUGCUAUUCUUCUUU 5’

query=JS16_t0467279, target=FGSG_00445, score=3, range=757-774, strand=1

target  5’ UCUCACCAUAAGAAGAAA 3’
           ::: :: :::::::::::
query   3’ AGACUGCUAUUCUUCUUU 5’

query=JS16_t0467279, target=FGSG_06904, score=3, range=168-186, strand=1

target  5’ UGUGACAGAUAAGAAGAAA 3’
           : :::: ::::::::::::
query   3’ AGACUG-CUAUUCUUCUUU 5’

query=JS16_t0467279, target=FGSG_13299, score=3, range=283-300, strand=-1

target  5’ UUGGGCGAUAAGAAGAGA 3’
           :.  :.:::::::::::.:
query   3’ AGACUGCUAUUCUUCUUU 5’

query=JS16_t0467279, target=FGSG_08874, score=3, range=783-801, strand=-1

target  5’ ACUGAGCGAUGAGAAGAAA 3’
            :::: ::::.::::::::
query   3’ AGACU-GCUAUUCUUCUUU 5’

query=JS16_t0467279, target=FGSG_04424, score=3.5, range=973-990, strand=1

target  5’ UCUGACGAUGAGGAGGAG 3’
           :::::::::.::.::.:.
query   3’ AGACUGCUAUUCUUCUUU 5’

query=JS16_t0467279, target=FGSG_04635, score=3.5, range=1729-1746, strand=1

target  5’ CCUGAUGAUGAGAAGAAG 3’
            ::::.:::.:::::::.
query   3’ AGACUGCUAUUCUUCUUU 5’

query=JS16_t0467279, target=FGSG_10221, score=3.5, range=307-324, strand=1

target  5’ UUUGACAAUAAGAGGAAA 3’
           :.:::: ::::::.::::
query   3’ AGACUGCUAUUCUUCUUU 5’

query=JS16_t0467279, target=FGSG_02563, score=3.5, range=748-765, strand=1

target  5’ UUUGGCGAUAUGAAGAAG 3’
           :.::.::::: ::::::.
query   3’ AGACUGCUAUUCUUCUUU 5’

query=JS16_t0467279, target=FGSG_09392, score=3.5, range=372-390, strand=1

target  5’ UCGUGACGAUGAGGAGAAG 3’
           :: :::::::.::.::::.
query   3’ AG-ACUGCUAUUCUUCUUU 5’

query=JS16_t0467279, target=FGSG_08780, score=3.5, range=343-360, strand=1

target  5’ AAUGAUGAUAAGAAGAAG 3’
             :::.:::::::::::.
query   3’ AGACUGCUAUUCUUCUUU 5’

query=JS16_t0467279, target=FGSG_10564, score=3.5, range=5857-5874, strand=1

target  5’ CCUGAGGAUAAGAAGAAG 3’
            :::: :::::::::::.
query   3’ AGACUGCUAUUCUUCUUU 5’

query=JS16_t0467279, target=FGSG_02875, score=3.5, range=334-351, strand=1

target  5’ UUUGAAGAUAAGAAGAAU 3’
           :.::: :::::::::::
query   3’ AGACUGCUAUUCUUCUUU 5’

query=JS16_t0467279, target=FGSG_11268, score=3.5, range=8026-8043, strand=1

target  5’ CCUGCCGAUGAGAAGAAG 3’
            ::: ::::.:::::::.
query   3’ AGACUGCUAUUCUUCUUU 5’

query=JS16_t0467279, target=FGSG_02793, score=3.5, range=271-288, strand=1

target  5’ GCUGCCGAUGAGAAGAAG 3’
            ::: ::::.:::::::.
query   3’ AGACUGCUAUUCUUCUUU 5’

query=JS16_t0467279, target=FGSG_08447, score=3.5, range=469-486, strand=1

target  5’ GUUUACGAUAAGGAGAAA 3’

query=JS16_t1393120, target=FGSG_07162, score=3.5, range=423-440, strand=-1

target  5’ CGAGGAUCGUCGAAUGGU 3’
           :. :::: ::::::::::
query   3’ GUACCUAACAGCUUACCA 5’

query=JS16_t1393120, target=FGSG_06517, score=3.5, range=1173-1191, strand=-1

target  5’ CAGGGGUUUGUCGAAUGGU 3’
           :: ::. ::::::::::::
query   3’ GUACCU-AACAGCUUACCA 5’

query=JS16_t1393120, target=FGSG_10191, score=4, range=443-459, strand=1

target  5’ AAUGGAUUGUCGAG-GGU 3’
            ::::::::::::. :::
query   3’ GUACCUAACAGCUUACCA 5’

query=JS16_t1393120, target=FGSG_02764, score=4, range=737-754, strand=1

target  5’ CCAAGAUUGUCGAAUGGA 3’
           :    :::::::::::::
query   3’ GUACCUAACAGCUUACCA 5’

query=JS16_t1393120, target=FGSG_12781, score=4, range=1203-1221, strand=1

target  5’ CAAGGCAUUGACGAAUGGU 3’
           :: :: :::: ::::::::
query   3’ GUACC-UAACAGCUUACCA 5’

query=JS16_t1393120, target=FGSG_12495, score=4, range=617-634, strand=1

target  5’ AAGGUAUUGUCGAAUGGG 3’
            : : ::::::::::::
query   3’ GUACCUAACAGCUUACCA 5’

query=JS16_t1393120, target=FGSG_09477, score=4, range=1163-1179, strand=1

target  5’ CA-GAAUUAUCGAAUGGU 3’
           :: : ::: :::::::::
query   3’ GUACCUAACAGCUUACCA 5’

query=JS16_t1393120, target=FGSG_13686, score=4, range=115-132, strand=-1

target  5’ CAUGGAUUUUCGGAUGGC 3’
           :::::::: :::.::::
query   3’ GUACCUAACAGCUUACCA 5’

query=JS16_t1393120, target=FGSG_08405, score=4, range=1759-1777, strand=-1

target  5’ CAUGGAUUGUCGAGAUUGU 3’
           ::::::::::::: :: ::
query   3’ GUACCUAACAGCU-UACCA 5’

query=JS16_t1393120, target=FGSG_11216, score=4, range=1113-1129, strand=-1

target  5’ CAGGGAUUGUCGAG-GGU 3’
           :: ::::::::::. :::
query   3’ GUACCUAACAGCUUACCA 5’

query=JS16_t1393120, target=FGSG_02679, score=4, range=385-401, strand=-1

target  5’ CAUGUAUUGUCGAG-GGU 3’
           :::: ::::::::. :::
query   3’ GUACCUAACAGCUUACCA 5’

query=JS16_t1393120, target=FGSG_10095, score=4, range=2519-2536, strand=-1

target  5’ CAGGCAUUGUCGAAGGGU 3’
           :: : ::::::::: :::
query   3’ GUACCUAACAGCUUACCA 5’

query=JS16_t1393120, target=FGSG_09210, score=4, range=910-926, strand=-1

target  5’ CAUGGA-UGUAGAAUGGU 3’
           :::::: ::: :::::::
query   3’ GUACCUAACAGCUUACCA 5’

No results for JS16_t3185434
No results for JS16_t0692434
No results for JS16_t3185471
query=JS16_t1393243, target=FGSG_12078, score=3.5, range=140-162, strand=-1

target  5’ UGUCGGUGAGAUGGUGUUGAAUG 3’
           .::::::  :::::::::::::
query   3’ GCAGCCA-GCUACCACAACUUAG 5’

query=JS16_t1393243, target=FGSG_09516, score=4, range=129-149, strand=1

target  5’ CGUC-GUCCAUGGUGAUGAAUC 3’
           :::: ::: :::::: ::::::
```

```
           .: ::::::::.:::::
query   3’ AGACUGCUAUUCUUCUUU 5’

query=JS16_t0467279, target=FGSG_03184, score=3.5, range=319-336, strand=1

target  5’ CCGGAUGAUAAGAAGAAG 3’
            : ::.::::::::::.
query   3’ AGACUGCUAUUCUUCUUU 5’

query=JS16_t0467279, target=FGSG_05282, score=3.5, range=535-552, strand=1

target  5’ GACGACGAUAAGAAGAAG 3’
              ::::::::::::::.
query   3’ AGACUGCUAUUCUUCUUU 5’

query=JS16_t0467279, target=FGSG_06565, score=3.5, range=2247-2265, strand=1

target  5’ UCUGGCCGAUAAGACGAAA 3’
           ::::. :::::::: ::::
query   3’ AGACU-GCUAUUCUUCUUU 5’

query=JS16_t0467279, target=FGSG_02953, score=3.5, range=474-492, strand=1

target  5’ UUGGACCGAUGAGAAGAAA 3’
           :. :: ::::.::::::::
query   3’ AGACU-GCUAUUCUUCUUU 5’

query=JS16_t0467279, target=FGSG_06130, score=3.5, range=340-357, strand=1

target  5’ GUUGCCGAUAAGAAGAAC 3’
            .:: ::::::::::::
query   3’ AGACUGCUAUUCUUCUUU 5’

query=JS16_t0467279, target=FGSG_00292, score=3.5, range=263-279, strand=1

target  5’ UC-GACGACAAGAAGAAG 3’
           :: ::::: ::::::::.
query   3’ AGACUGCUAUUCUUCUUU 5’

query=JS16_t0467279, target=FGSG_01121, score=3.5, range=548-564, strand=1

target  5’ UC-GAGGAUAAGAAGAAG 3’
           :: :: ::::::::::.
query   3’ AGACUGCUAUUCUUCUUU 5’

query=JS16_t0467279, target=FGSG_05848, score=3.5, range=643-660, strand=-1

target  5’ ACUGACGAUGAGAGGAAG 3’
            ::::::::.:::.:::.
query   3’ AGACUGCUAUUCUUCUUU 5’

query=JS16_t0467279, target=FGSG_08127, score=3.5, range=211-228, strand=-1

target  5’ GUUGAUGAUAAGAAGAGA 3’
            .:::.::::::::::.:
query   3’ AGACUGCUAUUCUUCUUU 5’

query=JS16_t0467279, target=FGSG_01575, score=3.5, range=94-111, strand=-1

target  5’ GUUGGCGAUAGGAAGAAG 3’
            .::.:::::.::::::.
query   3’ AGACUGCUAUUCUUCUUU 5’

query=JS16_t0467279, target=FGSG_09607, score=3.5, range=9428-9445, strand=-1

target  5’ UCUCACGAUGAGAAGGAG 3’
           ::: :::::.:::::.:.
query   3’ AGACUGCUAUUCUUCUUU 5’

query=JS16_t0467279, target=FGSG_11357, score=4, range=369-386, strand=1

target  5’ UCUGAUGACGAGAAGAAA 3’
           :::::.:: .::::::::
query   3’ AGACUGCUAUUCUUCUUU 5’

query=JS16_t0467279, target=FGSG_01770, score=4, range=613-630, strand=1

target  5’ UCUGAUGAAGAGAAGAAA 3’
           :::::.:: .::::::::
query   3’ AGACUGCUAUUCUUCUUU 5’

query=JS16_t0467279, target=FGSG_10530, score=4, range=4252-4269, strand=1

target  5’ UCUGGCGACGAGAAGAAG 3’
           ::::.::: .:::::::.
query   3’ AGACUGCUAUUCUUCUUU 5’

query=JS16_t0467279, target=FGSG_02271, score=4, range=1582-1599, strand=1

target  5’ UUUGGCGAGAAGAAGGAA 3’
           :.::.::: ::::::.::
query   3’ AGACUGCUAUUCUUCUUU 5’

query   3’ GCAGCCAGCUACCACAACUUAG 5’

query=JS16_t1393243, target=FGSG_09786, score=4, range=275-295, strand=-1

target  5’ CGGCGAUC-AUGGUGUUGAGUC 3’
           :: :: :: ::::::::::.::
query   3’ GCAGCCAGCUACCACAACUUAG 5’

query=JS16_t3185537, target=FGSG_13321, score=3.5, range=698-717, strand=1

target  5’ GAGGGGGGUCAUCACUUUUU 3’
            :::...::::::::::::
query   3’ GUCCUUUCAGUAGUGAAAAU 5’

query=JS16_t3185537, target=FGSG_07615, score=3.5, range=1169-1188, strand=-1

target  5’ CAGCGAAGUCAUCACUUGUA 3’
           ::: .:::::::::::: ::
query   3’ GUCCUUUCAGUAGUGAAAAU 5’

query=JS16_t3185537, target=FGSG_11690, score=4, range=641-661, strand=1

target  5’ CGGUGAAAGUCAUCACUGUUG 3’
           :.: ::::::::::::: ::.
query   3’ GUC-CUUUCAGUAGUGAAAAU 5’

query=JS16_t3185537, target=FGSG_01275, score=4, range=2595-2614, strand=1

target  5’ CAACAAGGUCAUCAUUUUUG 3’
           ::  ::.:::::::.::::.
query   3’ GUCCUUUCAGUAGUGAAAAU 5’

query=JS16_t3185537, target=FGSG_12194, score=4, range=885-904, strand=1

target  5’ CGCAAAGGUUAUCACUUUUA 3’
           :.  ::.::.::::::::::
query   3’ GUCCUUUCAGUAGUGAAAAU 5’

query=JS16_t4082208, target=FGSG_09206, score=2, range=1066-1084, strand=-1

target  5’ CUUCAAGACUAUGGUUGAC 3’
            :::: :::::::::::::
query   3’ AAAGUGCUGAUACCAACUG 5’

query=JS16_t4082208, target=FGSG_05253, score=2.5, range=43-61, strand=-1

target  5’ GUUCAAGACUAUGGUUGAU 3’
            :::: ::::::::::::.
query   3’ AAAGUGCUGAUACCAACUG 5’

query=JS16_t4082208, target=FGSG_03390, score=3.5, range=639-657, strand=1

target  5’ UAUUGAGACUAUGGUUGAU 3’
           : :.. ::::::::::::.
query   3’ AAAGUGCUGAUACCAACUG 5’

query=JS16_t4082208, target=FGSG_09804, score=3.5, range=388-405, strand=1

target  5’ AUUCA-GACUAUGGUUGGU 3’
            :::: :::::::::::..
query   3’ AAAGUGCUGAUACCAACUG 5’

query=JS16_t4082208, target=FGSG_04865, score=4, range=126-144, strand=1

target  5’ UUGCACGACUGUGGUUGGG 3’
           :: :::::::.::::::.
query   3’ AAAGUGCUGAUACCAACUG 5’

query=JS16_t4082208, target=FGSG_01741, score=4, range=1731-1749, strand=1

target  5’ UCCCAUGACUAUGGUUGGU 3’
           :  ::.:::::::::::..
query   3’ AAAGUGCUGAUACCAACUG 5’

query=JS16_t4082208, target=FGSG_06769, score=4, range=1023-1041, strand=1

target  5’ UUUCAGGACUAUGCUUGAA 3’
           ::::: ::::::: ::::
query   3’ AAAGUGCUGAUACCAACUG 5’

query=JS16_t4082208, target=FGSG_13802, score=4, range=1324-1341, strand=1

target  5’ UUUGAUG-CUAUGGUUGAU 3’
           ::: :.: ::::::::::.
query   3’ AAAGUGCUGAUACCAACUG 5’

query=JS16_t4082208, target=FGSG_00427, score=4, range=229-246, strand=-1

target  5’ UUUCAUGGCUAUGG-UGAU 3’
           :::::.:.:::::: :::.
query   3’ AAAGUGCUGAUACCAACUG 5’
```

```
query=JS16_t0467279, target=FGSG_11626, score=4, range=100-117, strand=1

target  5’ UCUGGUGACAAGAAGAAG 3’
           ::::..:: ::::::::.
query   3’ AGACUGCUAUUCUUCUUU 5’

query=JS16_t0467279, target=FGSG_01332, score=4, range=2608-2626, strand=1

target  5’ UCUGAUGAUGAGCAAGAAG 3’
           :::::.:::.:: :::::.
query   3’ AGACUGCUAUUC-UUCUUU 5’

query=JS16_t0467279, target=FGSG_05427, score=4, range=2698-2715, strand=1

target  5’ GCUGACGACGAGAAGAAA 3’
            ::::::: .::::::::
query   3’ AGACUGCUAUUCUUCUUU 5’

query=JS16_t0467279, target=FGSG_09914, score=4, range=139-156, strand=1

target  5’ GCUGACGAAAAGAAGGAA 3’
            ::::::: ::::::.::
query   3’ AGACUGCUAUUCUUCUUU 5’

query=JS16_t0467279, target=FGSG_07665, score=4, range=46-63, strand=1

target  5’ UCUGACGAGGAGAAGAAC 3’
           :::::::: .:::::::
query   3’ AGACUGCUAUUCUUCUUU 5’

query=JS16_t0467279, target=FGSG_04182, score=4, range=7-24, strand=1

target  5’ UCUGACGAGGAGAAGAAC 3’
           :::::::: .:::::::
query   3’ AGACUGCUAUUCUUCUUU 5’

query=JS16_t0467279, target=FGSG_07141, score=4, range=1519-1536, strand=1

target  5’ UCCGACGAUGAGGAGAAU 3’
           :: ::::::.::.::::
query   3’ AGACUGCUAUUCUUCUUU 5’

query=JS16_t0467279, target=FGSG_10940, score=4, range=1468-1485, strand=1

target  5’ GCCGACGGUGAGAAGAAA 3’
            : ::::.:.::::::::
query   3’ AGACUGCUAUUCUUCUUU 5’

query=JS16_t0467279, target=FGSG_00538, score=4, range=883-900, strand=1

target  5’ AUUGACGAGAAGAAGAAG 3’
            .:::::: ::::::::.
query   3’ AGACUGCUAUUCUUCUUU 5’

query=JS16_t0467279, target=FGSG_07821, score=4, range=3736-3753, strand=1

target  5’ CCAGGCGAUGAGAAGAAG 3’
            : :.::::.:::::::.
query   3’ AGACUGCUAUUCUUCUUU 5’

query=JS16_t0467279, target=FGSG_12809, score=4, range=2575-2592, strand=1

target  5’ GCGGGCGGUAAGAAGAAG 3’
            : :.::.:::::::::.
query   3’ AGACUGCUAUUCUUCUUU 5’

query=JS16_t0467279, target=FGSG_12033, score=4, range=898-915, strand=1

target  5’ UCUGACGAGAAAAAGAAA 3’
           :::::::: :: ::::::
query   3’ AGACUGCUAUUCUUCUUU 5’

query=JS16_t0467279, target=FGSG_05334, score=4, range=1399-1416, strand=1

target  5’ UCUGCCGAAGAGAAGAAA 3’
           :::: ::: .::::::::
query   3’ AGACUGCUAUUCUUCUUU 5’

query=JS16_t0467279, target=FGSG_07584, score=4, range=90-108, strand=1

target  5’ UCUGGAGGAUGAGAAGAAA 3’
           ::: :: :::.::::::::
query   3’ AGA-CUGCUAUUCUUCUUU 5’

query=JS16_t0467279, target=FGSG_00504, score=4, range=2386-2403, strand=1

target  5’ ACUGCCGAUGAGAAGAAU 3’
            ::: ::::.:::::::
query   3’ AGACUGCUAUUCUUCUUU 5’

query=JS16_t0467279, target=FGSG_02232, score=4, range=1324-1341, strand=1
```

```
query=JS16_t4082208, target=FGSG_03385, score=4, range=506-523, strand=-1

target  5’ UUUCA-GGCUGUGGUUGGC 3’
           ::::: :.::.::::::.:
query   3’ AAAGUGCUGAUACCAACUG 5’

query=JS16_t4082208, target=FGSG_02894, score=4, range=502-519, strand=-1

target  5’ CUUCAGGAC-AUGGUUGAC 3’
            :::: ::: :::::::::
query   3’ AAAGUGCUGAUACCAACUG 5’

No results for JS16_t2289376
No results for JS16_t4082227
query=JS16_t3185668, target=FGSG_06653, score=2, range=3377-3394, strand=1

target  5’ AGACUGGACAAACGCCUG 3’
            :: ::::::::::::::
query   3’ GCUCACCUGUUUGCGGAC 5’

query=JS16_t3185668, target=FGSG_09666, score=3, range=417-433, strand=1

target  5’ CGAG-GGCCAAACGCCUG 3’
           :::: :: ::::::::::
query   3’ GCUCACCUGUUUGCGGAC 5’

query=JS16_t3185668, target=FGSG_02899, score=3, range=201-218, strand=-1

target  5’ CUCUUGGACAAACGCCUG 3’
           :   ::::::::::::::
query   3’ GCUCACCUGUUUGCGGAC 5’

query=JS16_t3185668, target=FGSG_09637, score=3.5, range=1400-1417, strand=1

target  5’ AGGGUGGACAAAAGCCUG 3’
            :.::::::::: :::::
query   3’ GCUCACCUGUUUGCGGAC 5’

query=JS16_t3185668, target=FGSG_08845, score=3.5, range=275-292, strand=1

target  5’ UGCGUGGACAAAAGCCUG 3’
           .: ::::::::: :::::
query   3’ GCUCACCUGUUUGCGGAC 5’

query=JS16_t3185668, target=FGSG_03692, score=3.5, range=1366-1383, strand=1

target  5’ CAGGCGGACAAAUGCCUG 3’
           : .: :::::::.:::::
query   3’ GCUCACCUGUUUGCGGAC 5’

query=JS16_t3185668, target=FGSG_02312, score=3.5, range=195-211, strand=1

target  5’ UGA-UGGACAAAUGCCUC 3’
           .:: :::::::::.::::
query   3’ GCUCACCUGUUUGCGGAC 5’

query=JS16_t3185668, target=FGSG_04282, score=3.5, range=1136-1153, strand=-1

target  5’ CGGGUGGAUAAGCGCUUG 3’
           ::.:::::.::.:::.::
query   3’ GCUCACCUGUUUGCGGAC 5’

query=JS16_t3185668, target=FGSG_09695, score=4, range=569-586, strand=1

target  5’ UGGGAGGACAAAUGUCUG 3’
           .:.: :::::::.:.:::
query   3’ GCUCACCUGUUUGCGGAC 5’

query=JS16_t3185668, target=FGSG_00324, score=4, range=3737-3754, strand=1

target  5’ AGAGUGGAUACACGCCUG 3’
            :::::::.: :::::::
query   3’ GCUCACCUGUUUGCGGAC 5’

query=JS16_t3185668, target=FGSG_09772, score=4, range=3942-3959, strand=1

target  5’ CGAGUGGACAAAUACCUU 3’
           ::::::::::::.  :::
query   3’ GCUCACCUGUUUGCGGAC 5’

query=JS16_t3185668, target=FGSG_09118, score=4, range=960-977, strand=1

target  5’ CAAGUGGACAGGCGCCUU 3’
           : ::::::::..:::::
query   3’ GCUCACCUGUUUGCGGAC 5’

query=JS16_t3185668, target=FGSG_04875, score=4, range=390-406, strand=1

target  5’ CGAGUGGGCAAAC-UCUG 3’
           :::::::.::::: .:::
query   3’ GCUCACCUGUUUGCGGAC 5’
```

```
target  5' UCGAGCGAUGAGAAGAAG 3'
           ::  .::::.:::::::.
query   3' AGACUGCUAUUCUUCUUU 5'

query=JS16_t0467279, target=FGSG_03307, score=4, range=404-420, strand=1

target  5' ACU-AUGAUGAGAAGAAA 3'
            :: :.:::.::::::::
query   3' AGACUGCUAUUCUUCUUU 5'

query=JS16_t0467279, target=FGSG_03186, score=4, range=598-615, strand=1

target  5' CCCGACGAUAACAAGAAA 3'
            : :::::::: ::::::
query   3' AGACUGCUAUUCUUCUUU 5'

query=JS16_t0467279, target=FGSG_07422, score=4, range=1399-1416, strand=1

target  5' UCGGCCGAUAAGAAGGAC 3'
           :: : ::::::::::.:
query   3' AGACUGCUAUUCUUCUUU 5'

query=JS16_t0467279, target=FGSG_01249, score=4, range=1597-1614, strand=1

target  5' UCCAACGAUAAGAAGGAC 3'
           ::  :::::::::::.:
query   3' AGACUGCUAUUCUUCUUU 5'

query=JS16_t0467279, target=FGSG_04287, score=4, range=1051-1068, strand=1

target  5' GGUGUCGAUAAGAAGAAC 3'
             :: ::::::::::::
query   3' AGACUGCUAUUCUUCUUU 5'

query=JS16_t0467279, target=FGSG_10040, score=4, range=2222-2238, strand=1

target  5' UC-GGCAAUAAGAAGAAG 3'
           :: :.: ::::::::::.
query   3' AGACUGCUAUUCUUCUUU 5'

query=JS16_t0467279, target=FGSG_10314, score=4, range=686-702, strand=1

target  5' UCU-CCGAUGAGAAGGAA 3'
           :::  ::::.:::::.::
query   3' AGACUGCUAUUCUUCUUU 5'

query=JS16_t0467279, target=FGSG_05182, score=4, range=236-252, strand=1

target  5' UC-GCCGAUAAGGAGAAU 3'
           :: : :::::::.::::
query   3' AGACUGCUAUUCUUCUUU 5'

query=JS16_t0467279, target=FGSG_13801, score=4, range=750-766, strand=1

target  5' GCUGUCGA-AAGAAGAAA 3'
            ::: ::: :::::::::
query   3' AGACUGCUAUUCUUCUUU 5'

query=JS16_t0467279, target=FGSG_01733, score=4, range=717-734, strand=-1

target  5' UUUUGCGAUAGGAAGAGA 3'
           :.: .:::::.:::::.:
query   3' AGACUGCUAUUCUUCUUU 5'

query=JS16_t0467279, target=FGSG_11495, score=4, range=475-493, strand=-1

target  5' UCUCGGCGAUGAGAAGAGG 3'
           ::: :.::::.:::::::..
query   3' AGA-CUGCUAUUCUUCUUU 5'

query=JS16_t0467279, target=FGSG_03534, score=4, range=915-932, strand=-1

target  5' GUUGGUGAUAAGAAGAAU 3'
            .::..::::::::::
query   3' AGACUGCUAUUCUUCUUU 5'

query=JS16_t0467279, target=FGSG_06461, score=4, range=493-510, strand=-1

target  5' ACUGACCAUAGGAAGAAA 3'
            ::::: :::.:::::::
query   3' AGACUGCUAUUCUUCUUU 5'

query=JS16_t0467279, target=FGSG_06843, score=4, range=586-603, strand=-1

target  5' ACCGGCGAUAAGGAGAAG 3'
            : :.::::::.::::.
query   3' AGACUGCUAUUCUUCUUU 5'

query=JS16_t0467279, target=FGSG_07830, score=4, range=39-56, strand=-1

target  5' GCGGGCGGUAAGAAGAAG 3'

query=JS16_t3185668, target=FGSG_10615, score=4, range=1305-1321, strand=1

target  5' CGA-UGGACAAGUGCUUG 3'
           ::: :::::::..::.::
query   3' GCUCACCUGUUUGCGGAC 5'

query=JS16_t3185668, target=FGSG_13805, score=4, range=1017-1034, strand=1

target  5' CGAGGGAACAGACGCCUG 3'
           :::: : :::.:::::::
query   3' GCUCACCUGUUUGCGGAC 5'

query=JS16_t3185668, target=FGSG_06260, score=4, range=1326-1344, strand=1

target  5' CGAGUCGGAGAAGCGCCUG 3'
           ::::: ::: ::.::::::
query   3' GCUCA-CCUGUUUGCGGAC 5'

query=JS16_t3185668, target=FGSG_04485, score=4, range=675-693, strand=1

target  5' CGAGCUCGACAAGCGCCUG 3'
           :::: : :::::.::::::
query   3' GCUC-ACCUGUUUGCGGAC 5'

query=JS16_t3185668, target=FGSG_04224, score=4, range=1480-1497, strand=1

target  5' CGAAUGGACAAACACCUC 3'
           ::: ::::::::: :::
query   3' GCUCACCUGUUUGCGGAC 5'

query=JS16_t3185668, target=FGSG_06724, score=4, range=147-164, strand=1

target  5' CAAGUGGACAAAGGCCUU 3'
           : :::::::::: ::::
query   3' GCUCACCUGUUUGCGGAC 5'

query=JS16_t3185668, target=FGSG_03353, score=4, range=930-946, strand=1

target  5' CGAGUGGACAAA-GCAUG 3'
           :::::::::::: :: ::
query   3' GCUCACCUGUUUGCGGAC 5'

query=JS16_t3185668, target=FGSG_04258, score=4, range=192-209, strand=1

target  5' CCAGAGGGCAAACGCCUA 3'
           : :: ::.:::::::::
query   3' GCUCACCUGUUUGCGGAC 5'

query=JS16_t3185668, target=FGSG_12946, score=4, range=738-754, strand=1

target  5' CGAG-GGUCAAGCGCCUG 3'
           :::: :: :::.::::::
query   3' GCUCACCUGUUUGCGGAC 5'

query=JS16_t3185668, target=FGSG_12935, score=4, range=639-655, strand=1

target  5' GGAG-GGACAGACGCCUU 3'
            ::: :::::.::::::
query   3' GCUCACCUGUUUGCGGAC 5'

query=JS16_t3185668, target=FGSG_05751, score=4, range=4444-4462, strand=1

target  5' CGUAUUGGACAAACGCGUG 3'
           :: : ::::::::::: ::
query   3' GC-UCACCUGUUUGCGGAC 5'

query=JS16_t3185668, target=FGSG_00472, score=4, range=109-126, strand=1

target  5' CUACAGGACAAACGCUUG 3'
           : :  ::::::::::.::
query   3' GCUCACCUGUUUGCGGAC 5'

query=JS16_t3185668, target=FGSG_04357, score=4, range=1704-1720, strand=1

target  5' CUUGUGGACAAAC-CCUG 3'
           :  :::::::::: ::::
query   3' GCUCACCUGUUUGCGGAC 5'

query=JS16_t3185668, target=FGSG_13294, score=4, range=919-937, strand=-1

target  5' CGAGUUGGACAAGUGCUUG 3'
           :::: :::::::..::.::
query   3' GCUC-ACCUGUUUGCGGAC 5'

query=JS16_t3185668, target=FGSG_11677, score=4, range=1627-1645, strand=-1

target  5' CGAGAUGGACAGACGUUUG 3'
           :::: ::::::.:::..::
query   3' GCUC-ACCUGUUUGCGGAC 5'

query=JS16_t3185668, target=FGSG_00083, score=4, range=509-526, strand=-1
```

```
            : :.::.:::::::::.
query   3’ AGACUGCUAUUCUUCUUU 5’

query=JS16_t0467279, target=FGSG_09033, score=4, range=542-559, strand=-1

target  5’ CCUUGCGAUAAGGAGAAG 3’
            :: .:::::::.::::.
query   3’ AGACUGCUAUUCUUCUUU 5’

query=JS16_t0467279, target=FGSG_06272, score=4, range=2689-2706, strand=-1

target  5’ UCUGACAAGAAGAAGAAA 3’
           :::::: : :::::::::
query   3’ AGACUGCUAUUCUUCUUU 5’

query=JS16_t0467279, target=FGSG_04100, score=4, range=514-532, strand=-1

target  5’ CUUGAUCGAUAAGGAGAAG 3’
            .::: :::::::.::::.
query   3’ AGACU-GCUAUUCUUCUUU 5’

query=JS16_t0467279, target=FGSG_07516, score=4, range=2326-2343, strand=-1

target  5’ UCCAAUGAUAAGGAGAAA 3’
           ::  :.::::::.:::::
query   3’ AGACUGCUAUUCUUCUUU 5’

query=JS16_t0467279, target=FGSG_08343, score=4, range=1177-1194, strand=-1

target  5’ ACCGACGAUGAGAAGAAC 3’
            : ::::::.:::::::
query   3’ AGACUGCUAUUCUUCUUU 5’

query=JS16_t0467279, target=FGSG_06015, score=4, range=1400-1416, strand=-1

target  5’ UCUGACGAUAA-AAGAGU 3’
           ::::::::::: ::::.
query   3’ AGACUGCUAUUCUUCUUU 5’

query=JS16_t0467279, target=FGSG_08320, score=4, range=963-980, strand=-1

target  5’ UUUUUCGAUAGGAAGAAG 3’
           :.:  :::::.::::::.
query   3’ AGACUGCUAUUCUUCUUU 5’

query=JS16_t0467279, target=FGSG_05748, score=4, range=1063-1080, strand=-1

target  5’ AAACACGAUAAGAAGAAA 3’
               ::::::::::::::
query   3’ AGACUGCUAUUCUUCUUU 5’

query=JS16_t0467279, target=FGSG_12646, score=4, range=447-463, strand=-1

target  5’ CCUGGCGAUAA-AAGAAG 3’
            :::.:::::: :::::.
query   3’ AGACUGCUAUUCUUCUUU 5’

query=JS16_t0467279, target=FGSG_04039, score=4, range=325-342, strand=-1

target  5’ ACGGACGAGAAGAAGAAA 3’
            : ::::: :::::::::
query   3’ AGACUGCUAUUCUUCUUU 5’

query=JS16_t0467279, target=FGSG_09694, score=4, range=361-377, strand=-1

target  5’ UAUGA-GAUAAGAAGGAA 3’
           : ::: :::::::::.::
query   3’ AGACUGCUAUUCUUCUUU 5’

query=JS16_t0467279, target=FGSG_02422, score=4, range=11-27, strand=-1

target  5’ GCGGACGAU-AGAAGAAA 3’
            : :::::: ::::::::
query   3’ AGACUGCUAUUCUUCUUU 5’

query=JS16_t0467279, target=FGSG_00012, score=4, range=885-901, strand=-1

target  5’ UCAGAC-AUGAGAAGAAA 3’
           :: ::: ::.::::::::
query   3’ AGACUGCUAUUCUUCUUU 5’

query=JS16_t0467279, target=FGSG_11911, score=4, range=521-537, strand=-1

target  5’ UUUGAUGA-AAGAAGAAG 3’
           :.:::.:: ::::::::.
query   3’ AGACUGCUAUUCUUCUUU 5’

No results for JS16_t4019560
No results for JS16_t3122863
No results for JS16_t4019634
No results for JS16_t1330313
No results for JS16_t1330339

target  5’ AGACUGGAGAAACGCCUG 3’
            :: :::: :::::::::
query   3’ GCUCACCUGUUUGCGGAC 5’

query=JS16_t3185668, target=FGSG_02847, score=4, range=247-265, strand=-1

target  5’ CGAGUUUGACAAACGCCUU 3’
           :::: : :::::::::::
query   3’ GCUC-ACCUGUUUGCGGAC 5’

query=JS16_t3185668, target=FGSG_10577, score=4, range=696-712, strand=-1

target  5’ CGA-UGGACACACGCUUG 3’
           ::: :::::: ::::.::
query   3’ GCUCACCUGUUUGCGGAC 5’

query=JS16_t3185668, target=FGSG_03080, score=4, range=1059-1077, strand=-1

target  5’ CGACUGGACGAAACGCUUG 3’
           ::: ::::: ::::::.::
query   3’ GCUCACCUG-UUUGCGGAC 5’

query=JS16_t2289448, target=FGSG_06098, score=3, range=415-435, strand=-1

target  5’ GCCCUGGAACUUUUCACAGAA 3’
           :  :: :::::::::::::::
query   3’ CAAGAACUUGAAAAGUGUCUU 5’

query=JS16_t2289448, target=FGSG_05425, score=3.5, range=295-315, strand=-1

target  5’ GUUCUUGCACUUUUUGCAGAG 3’
           ::::::: ::::::..::::.
query   3’ CAAGAACUUGAAAAGUGUCUU 5’

query=JS16_t2289448, target=FGSG_06021, score=4, range=295-315, strand=-1

target  5’ CUUCUUGAACUUGUCACGGAA 3’
            ::::::::::: ::::.:::
query   3’ CAAGAACUUGAAAAGUGUCUU 5’

query=JS16_t2289448, target=FGSG_02153, score=4, range=1181-1200, strand=-1

target  5’ GUUCUUGAACUUUUU-UAGAA 3’
           ::::::::::::::. .::::
query   3’ CAAGAACUUGAAAAGUGUCUU 5’

query=JS16_t2289448, target=FGSG_04524, score=4, range=763-783, strand=-1

target  5’ GUUCUUUAACUUUUCGGAGAA 3’
           :::::: ::::::::. ::::
query   3’ CAAGAACUUGAAAAGUGUCUU 5’

query=JS16_t2289448, target=FGSG_06818, score=4, range=313-333, strand=-1

target  5’ GUUAAUGAGCUUUUCACAGAC 3’
           :::  :::.:::::::::::
query   3’ CAAGAACUUGAAAAGUGUCUU 5’

query=JS16_t0307609, target=FGSG_10130, score=3.5, range=1375-1394, strand=-1

target  5’ GCGAUCUUGCUCUCCGCUUC 3’
              :.:::::::::::::::
query   3’ AAAUGGAACGAGAGGCGAAG 5’

query=JS16_t0307609, target=FGSG_04198, score=3.5, range=1131-1149, strand=-1

target  5’ UUCAUC-UGCUCUCUGCUUC 3’
           :: :.: :::::::.:::::
query   3’ AAAUGGAACGAGAGGCGAAG 5’

query=JS16_t0307609, target=FGSG_01959, score=4, range=1006-1025, strand=1

target  5’ UUCACCUUCCUCUUCGCUUC 3’
           :: ::::: ::::.::::::
query   3’ AAAUGGAACGAGAGGCGAAG 5’

query=JS16_t0307609, target=FGSG_06691, score=4, range=1502-1521, strand=1

target  5’ UUUACAUUGCUCUUCUCUUC 3’
           ::::: :::::::.: ::::
query   3’ AAAUGGAACGAGAGGCGAAG 5’

query=JS16_t0307609, target=FGSG_12784, score=4, range=258-276, strand=-1

target  5’ UUUUCCUUGCUCUCC-UUUC 3’
           ::: ::::::::::: .:::
query   3’ AAAUGGAACGAGAGGCGAAG 5’

No results for JS16_t3185869
No results for JS16_t0337404
No results for JS16_t2289769
No results for JS16_t4082621
```

```
No results for JS16_t1330384
query=JS16_t2226926, target=FGSG_00958, score=2, range=1081-1098, strand=-1

target  5’ GAGG-AGAAAAUCUUGAUC 3’
           ::::  :::::::::::::
query   3’ CUCCAGCUUUUAGAACUAG 5’

query=JS16_t2226926, target=FGSG_13153, score=3.5, range=2211-2230, strand=-1

target  5’ GAGGUCGAGAAUCAUUGAUU 3’
           ::::::::.:::: :::::.
query   3’ CUCCAGCUUUUAG-AACUAG 5’

query=JS16_t2226926, target=FGSG_13920, score=3.5, range=1438-1457, strand=-1

target  5’ GGGAUCUGGAAAUCUUGAUC 3’
           :.: :: :.::::::::::
query   3’ CUCCAG-CUUUUAGAACUAG 5’

query=JS16_t2226926, target=FGSG_13787, score=4, range=189-207, strand=1

target  5’ GGGGUUGAAAAUCAUGAUG 3’
           :.:::.::::::: ::::
query   3’ CUCCAGCUUUUAGAACUAG 5’

query=JS16_t2226926, target=FGSG_12776, score=4, range=3688-3706, strand=1

target  5’ GAGGCCGAAUAUCUUGGUC 3’
           :::: :::: ::::::.::
query   3’ CUCCAGCUUUUAGAACUAG 5’

query=JS16_t2226926, target=FGSG_05520, score=4, range=2326-2343, strand=1

target  5’ GAGAUCGAAAGUCUU-AUC 3’
           ::: ::::::.:::: :::
query   3’ CUCCAGCUUUUAGAACUAG 5’

query=JS16_t2226926, target=FGSG_08969, score=4, range=145-163, strand=1

target  5’ GACUUCGAAAAUGUUGAUC 3’
           ::  :::::::: ::::::
query   3’ CUCCAGCUUUUAGAACUAG 5’

query=JS16_t2226926, target=FGSG_07098, score=4, range=321-339, strand=-1

target  5’ CAGGUCGAGAAUUUUGAUG 3’
            :::::::.:::.:::::
query   3’ CUCCAGCUUUUAGAACUAG 5’

query=JS16_t2226926, target=FGSG_01207, score=4, range=1059-1078, strand=-1

target  5’ GAUGGUGGAAGAUCUUGGUC 3’
           :: ::: :::.::::::.::
query   3’ CU-CCAGCUUUUAGAACUAG 5’

query=JS16_t2226926, target=FGSG_09271, score=4, range=105-123, strand=-1

target  5’ AAUCUCGAAGAUCUUGAUC 3’
            :  :::::.:::::::::
query   3’ CUCCAGCUUUUAGAACUAG 5’

query=JS16_t2226926, target=FGSG_00807, score=4, range=2196-2214, strand=-1

target  5’ CACGUCCAAAAUCUUGAUC 3’
            : ::: ::::::::::::
query   3’ CUCCAGCUUUUAGAACUAG 5’

query=JS16_t2226926, target=FGSG_00440, score=4, range=399-417, strand=-1

target  5’ GAGGGCGAUAAUCUUGAUG 3’
           :::: ::: :::::::::
query   3’ CUCCAGCUUUUAGAACUAG 5’

query=JS16_t2226926, target=FGSG_05106, score=4, range=2485-2504, strand=-1

target  5’ GAGGCUCCAAAAUCUUGAUA 3’
           :::: :: :::::::::::
query   3’ CUCC-AGCUUUUAGAACUAG 5’

query=JS16_t2226926, target=FGSG_09974, score=4, range=82-99, strand=-1

target  5’ GACGUCGAAGAUCUUG-UC 3’
           :: ::::::.:::::: ::
query   3’ CUCCAGCUUUUAGAACUAG 5’

query=JS16_t2226926, target=FGSG_09313, score=4, range=375-393, strand=-1

target  5’ GACCUCGAAAAUGUUGAUC 3’
           ::  :::::::: ::::::
query   3’ CUCCAGCUUUUAGAACUAG 5’

query=JS16_t2226926, target=FGSG_12470, score=4, range=463-480, strand=-1
```

```
No results for JS16_t0474905
query=JS16_t4082675, target=FGSG_01312, score=3, range=4123-4141, strand=1

target  5’ AACUCUUCCAGUCUGUCGC 3’
            :::::: :::::::::::
query   3’ CUGAGAAUGUCAGACAGCG 5’

query=JS16_t4082675, target=FGSG_05709, score=3, range=1732-1750, strand=1

target  5’ GUCAGUUACAGUCUGUCGC 3’
           : :  :::::::::::::
query   3’ CUGAGAAUGUCAGACAGCG 5’

query=JS16_t4082675, target=FGSG_04928, score=3.5, range=891-910, strand=1

target  5’ GCCUGUCUACAGUCUGUCGU 3’
           : :: : ::::::::::::.
query   3’ CUGAGA-AUGUCAGACAGCG 5’

query=JS16_t4082675, target=FGSG_02302, score=3.5, range=841-860, strand=1

target  5’ GAUUCAUACAGUGCUGUCGC 3’
           ::.:: :::::: :::::::
query   3’ CUGAGAAUGUCA-GACAGCG 5’

query=JS16_t4082675, target=FGSG_13426, score=3.5, range=531-548, strand=1

target  5’ GGCACUUACAG-CUGUCGC 3’
           :.: ::::::: :::::::
query   3’ CUGAGAAUGUCAGACAGCG 5’

query=JS16_t4082675, target=FGSG_11268, score=3.5, range=2510-2528, strand=-1

target  5’ AGCUCAUACAGUCUGUUGC 3’
            .::: ::::::::::.::
query   3’ CUGAGAAUGUCAGACAGCG 5’

query=JS16_t4082675, target=FGSG_06833, score=3.5, range=935-952, strand=-1

target  5’ GA-UCAUACAGUUUGUCGU 3’
           :: :: ::::::.:::::.
query   3’ CUGAGAAUGUCAGACAGCG 5’

query=JS16_t4082675, target=FGSG_03431, score=4, range=993-1010, strand=-1

target  5’ GACU-UUACAGGCUGUUGC 3’
           :::: :::::: ::::.::
query   3’ CUGAGAAUGUCAGACAGCG 5’

query=JS16_t3186063, target=FGSG_08466, score=2.5, range=784-800, strand=-1

target  5’ GC-UUCCUGCGGGUUGAC 3’
           :: :.:::::::.:::::
query   3’ CGCAGGGACGCCUAACUG 5’

query=JS16_t3186063, target=FGSG_04357, score=3, range=1363-1379, strand=-1

target  5’ ACGU-CCUGCGGGUUGAC 3’
            ::: :::::::.:::::
query   3’ CGCAGGGACGCCUAACUG 5’

query=JS16_t3186063, target=FGSG_00769, score=3.5, range=12-29, strand=1

target  5’ GCGUCUCUUCGGAUUGAU 3’
           :::::.:: ::::::::.
query   3’ CGCAGGGACGCCUAACUG 5’

query=JS16_t3186063, target=FGSG_08167, score=3.5, range=84-102, strand=1

target  5’ GCGUUGUCUGUGGAUUGAC 3’
           ::::. .:::.::::::::
query   3’ CGCAG-GGACGCCUAACUG 5’

query=JS16_t3186063, target=FGSG_05166, score=3.5, range=1330-1347, strand=-1

target  5’ GAGUUCCUGUGGGUUGAC 3’
           : ::.::::.::.:::::
query   3’ CGCAGGGACGCCUAACUG 5’

query=JS16_t3186063, target=FGSG_12516, score=3.5, range=1228-1244, strand=-1

target  5’ AUG-CCCUGCGGAUUGAA 3’
            .: :::::::::::::
query   3’ CGCAGGGACGCCUAACUG 5’

query=JS16_t3186063, target=FGSG_07155, score=4, range=247-263, strand=1

target  5’ CCGUCCCUGCGG-UUGAG 3’
            ::::::::::: ::::
query   3’ CGCAGGGACGCCUAACUG 5’

query=JS16_t3186063, target=FGSG_05372, score=4, range=5725-5742, strand=-1
```

```
target  5’ GAUUUCGAAAAUCUUG-UC 3’
           ::  :::::::::::: ::
query   3’ CUCCAGCUUUUAGAACUAG 5’

No results for JS16_t2226955
query=JS16_t0394117, target=FGSG_03915, score=2, range=504-522, strand=1

target  5’ CUACACACGCAUCAAUGAU 3’
            :: ::::::::::::::
query   3’ AAU-UGUGCGUAGUUACUA 5’

query=JS16_t0394117, target=FGSG_01049, score=2, range=22066-22082, strand=1

target  5’ UUCA-ACGCAUCAAUGAU 3’
           :: : ::::::::::::
query   3’ AAUUGUGCGUAGUUACUA 5’

query=JS16_t0394117, target=FGSG_05709, score=3, range=336-353, strand=1

target  5’ UCAAGAUGCAUCAAUGAU 3’
           : :: :.::::::::::
query   3’ AAUUGUGCGUAGUUACUA 5’

query=JS16_t0394117, target=FGSG_00500, score=3, range=1421-1437, strand=1

target  5’ UUAA-GCGCAUCAAUGAG 3’
           :::: .::::::::::
query   3’ AAUUGUGCGUAGUUACUA 5’

query=JS16_t0394117, target=FGSG_04175, score=3, range=2041-2058, strand=-1

target  5’ UUGAUACGUAUCGAUGAU 3’
           ::.:.:::.:::.:::::
query   3’ AAUUGUGCGUAGUUACUA 5’

query=JS16_t0394117, target=FGSG_08928, score=3, range=328-344, strand=-1

target  5’ GUAA-AUGCAUCAAUGAU 3’
            ::: :.::::::::::
query   3’ AAUUGUGCGUAGUUACUA 5’

query=JS16_t0394117, target=FGSG_04230, score=3.5, range=790-807, strand=1

target  5’ GUAGCACGCAUCAACGAU 3’
            ::.:::::::::: :::
query   3’ AAUUGUGCGUAGUUACUA 5’

query=JS16_t0394117, target=FGSG_10540, score=3.5, range=1637-1653, strand=1

target  5’ UUGACGCGCAU-AAUGAU 3’
           ::.::.::::: ::::::
query   3’ AAUUGUGCGUAGUUACUA 5’

query=JS16_t0394117, target=FGSG_00120, score=3.5, range=740-756, strand=1

target  5’ UUGA-GUGCAUCAAUGAU 3’
           ::.: ..::::::::::
query   3’ AAUUGUGCGUAGUUACUA 5’

query=JS16_t0394117, target=FGSG_03627, score=3.5, range=256-273, strand=1

target  5’ UUAUUACGCAUCAAAGAU 3’
           ::: .::::::::: :::
query   3’ AAUUGUGCGUAGUUACUA 5’

query=JS16_t0394117, target=FGSG_10678, score=3.5, range=1399-1415, strand=-1

target  5’ UUGAC-UGCAUCAAUGAU 3’
           ::.:: .::::::::::
query   3’ AAUUGUGCGUAGUUACUA 5’

query=JS16_t0394117, target=FGSG_02910, score=3.5, range=382-399, strand=-1

target  5’ CGGAAACGCAUCAAUGAU 3’
             .: ::::::::::::
query   3’ AAUUGUGCGUAGUUACUA 5’

query=JS16_t0394117, target=FGSG_08474, score=3.5, range=5953-5969, strand=-1

target  5’ UUGACA-GCAUCGAUGAU 3’
           ::.::: :::::.:::::
query   3’ AAUUGUGCGUAGUUACUA 5’

query=JS16_t0394117, target=FGSG_02441, score=3.5, range=508-524, strand=-1

target  5’ UUGACA-GCAUCAGUGAU 3’
           ::.::: ::::::.::::
query   3’ AAUUGUGCGUAGUUACUA 5’

query=JS16_t0394117, target=FGSG_11015, score=4, range=501-518, strand=1

target  5’ ACGUCCCUGCAGAUUGGC 3’
            :::::::: :::::.:
query   3’ CGCAGGGACGCCUAACUG 5’

query=JS16_t3186063, target=FGSG_12216, score=4, range=300-317, strand=-1

target  5’ GCUUCCUUGUGGAUUGAG 3’
           :: :::.::.:::::::
query   3’ CGCAGGGACGCCUAACUG 5’

query=JS16_t3186063, target=FGSG_08208, score=4, range=2971-2988, strand=-1

target  5’ GGGUUCCUGCGGAUUGUU 3’
           : ::.:::::::::: .
query   3’ CGCAGGGACGCCUAACUG 5’

query=JS16_t3186063, target=FGSG_08672, score=4, range=2000-2018, strand=-1

target  5’ GCCUCUCCUGCGUAUUGAC 3’
           :: :: :::::: ::::::
query   3’ CGCAG-GGACGCCUAACUG 5’

query=JS16_t3186063, target=FGSG_00299, score=4, range=1585-1601, strand=-1

target  5’ GCAUCCUUGCGG-UUGAC 3’
           :: :::.::::: :::::
query   3’ CGCAGGGACGCCUAACUG 5’

query=JS16_t4082751, target=FGSG_03967, score=4, range=849-869, strand=-1

target  5’ GC-AAAGCUAUCACCCCCAGGU 3’
           :: :::.:: :::::::::::.
query   3’ CGAUUUUGAGAGUGGGGGUCCG 5’

query=JS16_t4082767, target=FGSG_10995, score=2, range=617-635, strand=-1

target  5’ CAGA-AGUAAACACCAAGGU 3’
           :::: : ::::::::::::
query   3’ GUCUAUGAUUUGUGGUUCCA 5’

query=JS16_t4082767, target=FGSG_06362, score=3, range=314-333, strand=-1

target  5’ CACAUUCUGAACACCAAGGU 3’
           :: :: ::.:::::::::::
query   3’ GUCUAUGAUUUGUGGUUCCA 5’

query=JS16_t4082767, target=FGSG_11193, score=3.5, range=275-293, strand=1

target  5’ AAGA-AUUAAACACCAAGGA 3’
            ::: :.:::::::::::
query   3’ GUCUAUGAUUUGUGGUUCCA 5’

query=JS16_t4082767, target=FGSG_01803, score=3.5, range=2168-2186, strand=-1

target  5’ CGUA-ACUGAACACCAAGGU 3’
           :.  : :::.::::::::::
query   3’ GUCUAUGAUUUGUGGUUCCA 5’

query=JS16_t4082767, target=FGSG_00384, score=4, range=187-206, strand=1

target  5’ CUGAUACUACACAUCAAGGU 3’
           : ::::::: :::.::::::
query   3’ GUCUAUGAUUUGUGGUUCCA 5’

query=JS16_t4082767, target=FGSG_04872, score=4, range=2138-2156, strand=1

target  5’ UGGAU-CUCAACACCAAGGU 3’
           ..::: :: ::::::::::
query   3’ GUCUAUGAUUUGUGGUUCCA 5’

query=JS16_t4082767, target=FGSG_07722, score=4, range=413-431, strand=1

target  5’ UAGG-ACUAAACACCAAUGU 3’
           .::.  :::::::::: ::
query   3’ GUCUAUGAUUUGUGGUUCCA 5’

query=JS16_t4082767, target=FGSG_11966, score=4, range=2330-2348, strand=1

target  5’ CAGCU-CUAAACACCGAGGA 3’
           ::: : :::::::::.:::
query   3’ GUCUAUGAUUUGUGGUUCCA 5’

query=JS16_t0337413, target=FGSG_03194, score=3.5, range=1060-1080, strand=1

target  5’ UGGACUUGGUCCAACGUUCAA 3’
           ..:::::.::::::::.:::
query   3’ GUCUGAAUCAGGUUGCGAGUG 5’

query=JS16_t0337413, target=FGSG_13203, score=4, range=89-109, strand=-1

target  5’ UAGGCUUGGGCCAACGCUCAU 3’
```

```
target  5’ AUAUCGCGCAUCAAUGAG 3’
            :: :.:::::::::::
query   3’ AAUUGUGCGUAGUUACUA 5’

query=JS16_t0394117, target=FGSG_12071, score=4, range=52-69, strand=1

target  5’ CUAUCACGCAUCAAUAAU 3’
            :: ::::::::::: ::
query   3’ AAUUGUGCGUAGUUACUA 5’

query=JS16_t0394117, target=FGSG_06759, score=4, range=408-426, strand=1

target  5’ GUACACACGCAUCAAAGAU 3’
            :: ::::::::::: :::
query   3’ AAU-UGUGCGUAGUUACUA 5’

query=JS16_t0394117, target=FGSG_04694, score=4, range=4965-4982, strand=1

target  5’ UGAAGACGCGUCAAUGAA 3’
           : :: ::::.:::::::
query   3’ AAUUGUGCGUAGUUACUA 5’

query=JS16_t0394117, target=FGSG_03278, score=4, range=1691-1707, strand=1

target  5’ UUACCACGCAUC-GUGAU 3’
           ::: :::::::: .::::
query   3’ AAUUGUGCGUAGUUACUA 5’

query=JS16_t0394117, target=FGSG_07723, score=4, range=508-525, strand=1

target  5’ CAAAGACGCAUCAAUGAG 3’
             :: ::::::::::::
query   3’ AAUUGUGCGUAGUUACUA 5’

query=JS16_t0394117, target=FGSG_02643, score=4, range=2359-2375, strand=1

target  5’ UUCA-ACGCGUCGAUGAU 3’
           :: : ::::.::.:::::
query   3’ AAUUGUGCGUAGUUACUA 5’

query=JS16_t0394117, target=FGSG_03751, score=4, range=1268-1284, strand=1

target  5’ CUAA-GCGCAUCAAUGAC 3’
            ::: .:::::::::::
query   3’ AAUUGUGCGUAGUUACUA 5’

query=JS16_t0394117, target=FGSG_00002, score=4, range=233-249, strand=1

target  5’ UCGGCAC-CAUCAAUGAU 3’
           : ..::: ::::::::::
query   3’ AAUUGUGCGUAGUUACUA 5’

query=JS16_t0394117, target=FGSG_03234, score=4, range=1024-1041, strand=-1

target  5’ UUUCCAUGCAUCGAUGAU 3’
           ::  ::.:::::.:::::
query   3’ AAUUGUGCGUAGUUACUA 5’

query=JS16_t0394117, target=FGSG_00072, score=4, range=51-68, strand=-1

target  5’ AAAAGACGCAUCGAUGAU 3’
             :: :::::::.:::::
query   3’ AAUUGUGCGUAGUUACUA 5’

query=JS16_t0394117, target=FGSG_01947, score=4, range=940-956, strand=-1

target  5’ AUGGCACGCAUC-AUGAU 3’
            :..:::::::: :::::
query   3’ AAUUGUGCGUAGUUACUA 5’

query=JS16_t0394117, target=FGSG_10123, score=4, range=499-515, strand=-1

target  5’ UUGGC-CGCAUCAAUGAC 3’
           ::..: :::::::::::
query   3’ AAUUGUGCGUAGUUACUA 5’

query=JS16_t0394117, target=FGSG_09549, score=4, range=553-569, strand=-1

target  5’ UUAACA-GCAUCGGUGAU 3’
           :::::: :::::..::::
query   3’ AAUUGUGCGUAGUUACUA 5’

query=JS16_t0394117, target=FGSG_06517, score=4, range=238-254, strand=-1

target  5’ UUCACA-GCAUCAAUGAG 3’
           :: ::: ::::::::::
query   3’ AAUUGUGCGUAGUUACUA 5’

query=JS16_t0394117, target=FGSG_10397, score=4, range=355-371, strand=-1

target  5’ UGAACAU-CAUCAAUGAU 3’
           : ::::. ::::::::::
           .::.:::.: ::::::::::.
query   3’ GUCUGAAUCAGGUUGCGAGUG 5’

No results for JS16_t1393891
No results for JS16_t1393934
query=JS16_t2289995, target=FGSG_04356, score=4, range=671-691, strand=-1

target  5’ AAGUCGAUGUCCGGUCGGGAA 3’
            ::: :::::::.:::::.::
query   3’ GUCAACUACAGGUCAGCCUUU 5’

query=JS16_t1393975, target=FGSG_09785, score=3, range=202-220, strand=1

target  5’ UUGAG-CCAAUUCCAUGGUU 3’
           ::::: ::::::: ::::::
query   3’ AACUCAGGUUAAGCUACCAA 5’

query=JS16_t1393975, target=FGSG_10986, score=4, range=1619-1638, strand=1

target  5’ UUGAUUCCAAUUUGAAGGUU 3’
           :::: :::::::.:: ::::
query   3’ AACUCAGGUUAAGCUACCAA 5’

query=JS16_t1393975, target=FGSG_11826, score=4, range=484-504, strand=-1

target  5’ UGGAGUGCCAAUUCGAAGGUU 3’
           : :::: ::::::::: ::::
query   3’ AACUCA-GGUUAAGCUACCAA 5’

query=JS16_t1393975, target=FGSG_11026, score=4, range=6907-6925, strand=-1

target  5’ UCGAGUCCAAUUCGA-GGUA 3’
           : ::::::::::::: :::
query   3’ AACUCAGGUUAAGCUACCAA 5’

No results for JS16_t0474934
query=JS16_t0692527, target=FGSG_13450, score=3, range=57-75, strand=1

target  5’ CCCA-GGGUGUAAGCUUUCC 3’
           :::: ::::::::::::.:
query   3’ GGGUACCCACAUUCGAAGGU 5’

query=JS16_t0692527, target=FGSG_06771, score=3, range=866-885, strand=-1

target  5’ ACCAUGGGCGUAAGCUUCCA 3’
            ::::::: :::::::::::
query   3’ GGGUACCCACAUUCGAAGGU 5’

query=JS16_t0692527, target=FGSG_02825, score=4, range=319-337, strand=1

target  5’ CUCAUCGGUG-AAGCUUCCG 3’
           :.::: :::: :::::::::.
query   3’ GGGUACCCACAUUCGAAGGU 5’

query=JS16_t0692527, target=FGSG_08823, score=4, range=788-807, strand=-1

target  5’ CCCAUGGGUAUGAGCUUCCU 3’
           ::::::::: :.:::::::
query   3’ GGGUACCCACAUUCGAAGGU 5’

query=JS16_t0692527, target=FGSG_00455, score=4, range=668-686, strand=-1

target  5’ UCCAUGGGUGUAAG-UUCUG 3’
           .::::::::::::: :::..
query   3’ GGGUACCCACAUUCGAAGGU 5’

No results for JS16_t4082885
query=JS16_t3186255, target=FGSG_12643, score=3.5, range=102-121, strand=-1

target  5’ CACCUGU-GCUGGCGGAAUGU 3’
           ::::.:: ::::::..:::::
query   3’ GUGGGCAGCGACCGUUUUACA 5’

query=JS16_t3186255, target=FGSG_02628, score=4, range=329-350, strand=1

target  5’ CACUCGCUGCUGGCAAACAUGU 3’
           :::.:: .::::::::: ::::
query   3’ GUGGGCAGCGACCGUUU-UACA 5’

No results for JS16_t1394032
No results for JS16_t4082951
query=JS16_t4082967, target=FGSG_13844, score=4, range=635-660, strand=1

target  5’ GUGAUA-UGCCGGUCUAUGUUGCGAUC 3’
           :::::: ..:::::: :::::::::::
query   3’ CACUAUGGUGGCCAGUUACAACGCUAG 5’

No results for JS16_t4082974
query=JS16_t2290146, target=FGSG_11655, score=3, range=1028-1046, strand=1

target  5’ UUGCCACUAUUUUGGUCAA 3’
           ..:::::::::::: ::::
```

```
query   3' AAUUGUGCGUAGUUACUA 5'

No results for JS16_t1330513
No results for JS16_t0685019
No results for JS16_t0283082
No results for JS16_t0685025
query=JS16_t0305521, target=FGSG_09976, score=3.5, range=1274-1293, strand=1

target  5' UCGCUCCCAAGCGUGACAUG 3'
           .:::: :::::::.::::.:
query   3' GGCGAAGGUUCGCGCUGUGC 5'

query=JS16_t0305521, target=FGSG_05719, score=4, range=1612-1632, strand=1

target  5' CCGCCUUCUAAGCGCGACAUC 3'
           ::: ::::.::::::::::.
query   3' GGC-GAAGGUUCGCGCUGUGC 5'

query=JS16_t0305521, target=FGSG_02515, score=4, range=45-63, strand=1

target  5' CCG-UUCCAGGCGCGAUACU 3'
           ::: :::::.::::::.::
query   3' GGCGAAGGUUCGCGCUGUGC 5'

query=JS16_t0305521, target=FGSG_01734, score=4, range=431-449, strand=1

target  5' CCCCUUCCAAGCGCGGC-CG 3'
           :: ::::::::::::.: ::
query   3' GGCGAAGGUUCGCGCUGUGC 5'

No results for JS16_t1330605
No results for JS16_t2227104
No results for JS16_t0576343
No results for JS16_t1330686
No results for JS16_t3123292
No results for JS16_t2227214
No results for JS16_t2227236
No results for JS16_t3123355
query=JS16_t1330789, target=FGSG_02553, score=4, range=972-994, strand=1

target  5' CUACUUCAGCGAGGAUGAAAUGC 3'
            :::::::: :::::::::: ::
query   3' CAUGAAGUCCCUCCUACUUU-CG 5'

No results for JS16_t0576370
query=JS16_t2227306, target=FGSG_13640, score=2.5, range=1588-1605, strand=1

target  5' AUAGGAAGCUAUCAAUCU 3'
           :.  ::::::::.::::::
query   3' UGACCUUCGAUGGUUAGA 5'

query=JS16_t2227306, target=FGSG_10081, score=3, range=402-418, strand=1

target  5' CCU-GAAGCUACCAAUCC 3'
            :: :::::::::::::
query   3' UGACCUUCGAUGGUUAGA 5'

query=JS16_t2227306, target=FGSG_08246, score=3.5, range=710-726, strand=-1

target  5' GCUGG-AGUUACCAAUCU 3'
           .:::: ::.:::::::::
query   3' UGACCUUCGAUGGUUAGA 5'

query=JS16_t2227306, target=FGSG_08795, score=3.5, range=414-430, strand=-1

target  5' AUU-GAGGCUGCCAAUCU 3'
           :.: ::.:::.:::::::
query   3' UGACCUUCGAUGGUUAGA 5'

query=JS16_t2227306, target=FGSG_05586, score=3.5, range=901-917, strand=-1

target  5' AUU-GAAGCUAUCAAUUU 3'
           :.: :::::::.::::.:
query   3' UGACCUUCGAUGGUUAGA 5'

query=JS16_t2227306, target=FGSG_07099, score=4, range=536-553, strand=1

target  5' UCUGGAAGCUGCCCAUCU 3'
            :::::::::.:: ::::
query   3' UGACCUUCGAUGGUUAGA 5'

query=JS16_t2227306, target=FGSG_12558, score=4, range=477-494, strand=1

target  5' ACUGCAAGCUACUGAUCC 3'
           :::: :::::::..:::
query   3' UGACCUUCGAUGGUUAGA 5'

query=JS16_t2227306, target=FGSG_11379, score=4, range=169-186, strand=1

target  5' ACUCGAACCUACCAGUCU 3'
           ::: ::: ::::::.:::
query   3' UGACCUUCGAUGGUUAGA 5'

query   3' GGCGGUGAUAAAACAAGUU 5'

query=JS16_t2290146, target=FGSG_09172, score=3.5, range=176-194, strand=-1

target  5' UCAUCGCUGUUUUGUUCAA 3'
           .: .:.::.::::::::::
query   3' GGCGGUGAUAAAACAAGUU 5'

query=JS16_t2290146, target=FGSG_11475, score=4, range=901-920, strand=1

target  5' UUGCUAUAUAUUUUGUUCAA 3'
           ..::.:. ::::::::::::
query   3' GGCGGUG-AUAAAACAAGUU 5'

query=JS16_t2290146, target=FGSG_06431, score=4, range=126-144, strand=1

target  5' AUGCUUCUAUUUUGUUCAU 3'
            .::. ::::::::::::
query   3' GGCGGUGAUAAAACAAGUU 5'

query=JS16_t2290146, target=FGSG_13196, score=4, range=677-695, strand=1

target  5' CCGAAACUAUUUUGUUUAU 3'
           :::  :::::::::::.:
query   3' GGCGGUGAUAAAACAAGUU 5'

query=JS16_t2290146, target=FGSG_02080, score=4, range=3650-3667, strand=1

target  5' CCACUGCUAUUUUG-UCAA 3'
           :: :..:::::::: ::::
query   3' GGCGGUGAUAAAACAAGUU 5'

query=JS16_t2290146, target=FGSG_06328, score=4, range=745-762, strand=1

target  5' CAGCC-CUAUUUUGUUGAA 3'
           : ::: :::::::::: ::
query   3' GGCGGUGAUAAAACAAGUU 5'

query=JS16_t2290146, target=FGSG_04731, score=4, range=923-941, strand=-1

target  5' CUGGCGCUAUUUUGUUCGC 3'
           :.: :.:::::::::::.
query   3' GGCGGUGAUAAAACAAGUU 5'

query=JS16_t2290146, target=FGSG_01062, score=4, range=8-25, strand=-1

target  5' CUGUCACUAUUUUG-UCGA 3'
           :.:.:::::::::: ::.:
query   3' GGCGGUGAUAAAACAAGUU 5'

query=JS16_t2290146, target=FGSG_08239, score=4, range=836-853, strand=-1

target  5' AUG-CGCUAUUUUGUUUAA 3'
            .: :.:::::::::.::
query   3' GGCGGUGAUAAAACAAGUU 5'

query=JS16_t2290175, target=FGSG_11462, score=3.5, range=37-55, strand=1

target  5' GAGUUGAAAC-GAAAAGCGG 3'
           .::::: ::: :::::::::
query   3' UUCAACCUUGCCUUUUCGCC 5'

query=JS16_t2290175, target=FGSG_06962, score=3.5, range=156-176, strand=-1

target  5' AGGUUGGCAAUGGAAAGGCGG 3'
           :.::::: ::.:::::.::::
query   3' UUCAACC-UUGCCUUUUCGCC 5'

query=JS16_t2290175, target=FGSG_04695, score=4, range=357-376, strand=1

target  5' AUAUUGGAACAGAAAAGCGG 3'
           :  ::::::: :::::::::
query   3' UUCAACCUUGCCUUUUCGCC 5'

query=JS16_t2290175, target=FGSG_02774, score=4, range=31-50, strand=-1

target  5' AUGACGGAACGGAAAAGCGA 3'
           : :  ::::::::::::::
query   3' UUCAACCUUGCCUUUUCGCC 5'

No results for JS16_t4083091
No results for JS16_t2290277
query=JS16_t4083221, target=FGSG_09885, score=2.5, range=540-561, strand=-1

target  5' CAUGAUGUUGCGAACGUUCUUG 3'
           ::.::.. ::::::::::::::
query   3' GUGCUGUCACGCUUGCAAGAAC 5'

No results for JS16_t2290402
query=JS16_t1394417, target=FGSG_01724, score=3, range=687-705, strand=1

target  5' AAGGUA-CCUAACCAAUCUC 3'
```

```
query=JS16_t2227306, target=FGSG_13240, score=4, range=882-899, strand=1

target  5’ ACUUGAAGAUGCCAAUCU 3’
           ::: :::: :.:::::::
query   3’ UGACCUUCGAUGGUUAGA 5’

query=JS16_t2227306, target=FGSG_12138, score=4, range=182-200, strand=1

target  5’ ACGGCGAAGCUACCGGUCU 3’
           :: : :::::::::..:::
query   3’ UGAC-CUUCGAUGGUUAGA 5’

query=JS16_t2227306, target=FGSG_04440, score=4, range=516-533, strand=1

target  5’ CCAGGAAGCUAUCAAUCG 3’
            : ::::::::.:::::
query   3’ UGACCUUCGAUGGUUAGA 5’

query=JS16_t2227306, target=FGSG_10435, score=4, range=4809-4827, strand=1

target  5’ CCUCGGAAGCUACCAAGCU 3’
            :: :::::::::::: ::
query   3’ UGA-CCUUCGAUGGUUAGA 5’

query=JS16_t2227306, target=FGSG_06008, score=4, range=70-86, strand=1

target  5’ AC-GGUAGCUACCAAUCC 3’
           :: :: :::::::::::
query   3’ UGACCUUCGAUGGUUAGA 5’

query=JS16_t2227306, target=FGSG_11400, score=4, range=638-655, strand=1

target  5’ ACAUCAAGCUACCAAUCC 3’
           ::   ::::::::::::
query   3’ UGACCUUCGAUGGUUAGA 5’

query=JS16_t2227306, target=FGSG_00640, score=4, range=661-678, strand=-1

target  5’ CCUGGAAGCUACAGAUCU 3’
            ::::::::::: .::::
query   3’ UGACCUUCGAUGGUUAGA 5’

query=JS16_t2227306, target=FGSG_00878, score=4, range=594-612, strand=-1

target  5’ ACUGGUAAGCUCCCAGUCU 3’
           ::::: ::::: :::.:::
query   3’ UGACC-UUCGAUGGUUAGA 5’

query=JS16_t2227306, target=FGSG_13345, score=4, range=76-94, strand=-1

target  5’ ACUGGUAAGGUACCAAUUU 3’
           ::::: ::: :::::::.:
query   3’ UGACC-UUCGAUGGUUAGA 5’

query=JS16_t2227306, target=FGSG_04717, score=4, range=1398-1415, strand=-1

target  5’ UCUGGAAGCUUCCAAUCA 3’
            ::::::::: ::::::
query   3’ UGACCUUCGAUGGUUAGA 5’

query=JS16_t2227306, target=FGSG_12132, score=4, range=1952-1968, strand=-1

target  5’ ACU-GAAGUUAUCAAUCA 3’
           ::: ::::.::.:::::
query   3’ UGACCUUCGAUGGUUAGA 5’

query=JS16_t2227306, target=FGSG_03756, score=4, range=872-889, strand=-1

target  5’ AGAGGAAGCUGCCAAUCC 3’
           :  :::::::.::::::
query   3’ UGACCUUCGAUGGUUAGA 5’

query=JS16_t2227306, target=FGSG_08749, score=4, range=443-460, strand=-1

target  5’ AGAUCAAGCUACCAAUCU 3’
           :    :::::::::::::
query   3’ UGACCUUCGAUGGUUAGA 5’

No results for JS16_t4020149
No results for JS16_t0364818
No results for JS16_t4020211
No results for JS16_t2227388
No results for JS16_t2227413
No results for JS16_t1330951
query=JS16_t0685069, target=FGSG_02680, score=2.5, range=643-665, strand=1

target  5’ AACUUCAGCUUUGUUUUCAAAGA 3’
           ::: .::.:.:::::::::::::
query   3’ UUG-GGUUGGAACAAAAGUUUCU 5’

query=JS16_t0685069, target=FGSG_12768, score=4, range=759-781, strand=-1
```

```
           : :::: :::::::::.:::
query   3’ UCCCAUCGGAUUGGUUGGAG 5’

query=JS16_t1394417, target=FGSG_07344, score=3, range=1316-1334, strand=1

target  5’ AGGGUAGCC-GACCAACCUC 3’
           ::::::::: .:::::::::
query   3’ UCCCAUCGGAUUGGUUGGAG 5’

query=JS16_t1394417, target=FGSG_04212, score=3, range=835-854, strand=-1

target  5’ CGUGUAGCCUGACCAACCUC 3’
            : :::::::.:::::::::
query   3’ UCCCAUCGGAUUGGUUGGAG 5’

query=JS16_t1394417, target=FGSG_10913, score=4, range=3158-3177, strand=1

target  5’ AGGGUGUCCAAACCAACCUU 3’
           :::::. :: :::::::::.
query   3’ UCCCAUCGGAUUGGUUGGAG 5’

query=JS16_t1394417, target=FGSG_07227, score=4, range=1563-1581, strand=1

target  5’ UGGGUAUCCU-ACCAACCUC 3’
            ::::: ::: :::::::::
query   3’ UCCCAUCGGAUUGGUUGGAG 5’

query=JS16_t3186703, target=FGSG_08759, score=4, range=373-393, strand=-1

target  5’ ACUGUCGCCAAGCGCAUCGUC 3’
            :::::::::::::: ::::
query   3’ AGACAGCGGUUCGCG-AGCAA 5’

No results for JS16_t0692589
No results for JS16_t0284067
No results for JS16_t4083349
No results for JS16_t0692596
query=JS16_t0396232, target=FGSG_04496, score=4, range=547-566, strand=1

target  5’ AUGCUAUGGAGUGUGUUGAG 3’
            :::: :::::::.::.:::
query   3’ AACGA-ACCUCACGCAGCUC 5’

query=JS16_t0396232, target=FGSG_10707, score=4, range=675-693, strand=1

target  5’ UUGCCUGGAGUGGGUCGAA 3’
           :::: ::::::: :::::
query   3’ AACGAACCUCACGCAGCUC 5’

query=JS16_t0396232, target=FGSG_12822, score=4, range=400-417, strand=1

target  5’ UUGCUUGGAGUG-GGCGAG 3’
           :::::::::::: : ::::
query   3’ AACGAACCUCACGCAGCUC 5’

query=JS16_t0396232, target=FGSG_08005, score=4, range=824-843, strand=1

target  5’ GUGACCUGGAGUGCGUCGAA 3’
            :: : :::::::::::::
query   3’ AAC-GAACCUCACGCAGCUC 5’

query=JS16_t0396232, target=FGSG_09344, score=4, range=976-993, strand=1

target  5’ UUG-AUGGAGUGCGUGGAG 3’
           :::  :::::::::: :::
query   3’ AACGAACCUCACGCAGCUC 5’

query=JS16_t0396232, target=FGSG_01135, score=4, range=482-501, strand=-1

target  5’ GUGCUUGGGGUUGCGUCGAG 3’
            :::::::.: :::::::::
query   3’ AACGAACCUC-ACGCAGCUC 5’

query=JS16_t0396232, target=FGSG_10657, score=4, range=133-151, strand=-1

target  5’ UUGAUUGGAGUGCUUCGGG 3’
           ::: ::::::::: :::.:
query   3’ AACGAACCUCACGCAGCUC 5’

query=JS16_t0396232, target=FGSG_09796, score=4, range=161-179, strand=-1

target  5’ UUCGCAGGAGUGCGUCGAG 3’
           ::    :::::::::::::
query   3’ AACGAACCUCACGCAGCUC 5’

query=JS16_t0396232, target=FGSG_12820, score=4, range=100-117, strand=-1

target  5’ UUG-AUGGAAUGCGUCGAG 3’
           :::  :::: :::::::::
query   3’ AACGAACCUCACGCAGCUC 5’

query=JS16_t3186844, target=FGSG_09041, score=2, range=991-1007, strand=1
```

```
target  5’ AAUCGAACUUUGUUUUCAGAAGA 3’
           ::.: :::.:::::::::: ::::
query   3’ UUGGGUUGGAACAAAAGU-UUCU 5’

No results for JS16_t0335337
query=JS16_t0335339, target=FGSG_13824, score=3, range=1769-1787, strand=1

target  5’ UUC-UGAUCCAAGCAAUCGA 3’
           ::: ::: ::::::::::::
query   3’ AAGAACUCGGUUCGUUAGCU 5’

query=JS16_t0335339, target=FGSG_05615, score=3, range=929-948, strand=-1

target  5’ UUCUUGAGCCAAGCAAACGU 3’
           :::::::::::::::: ::
query   3’ AAGAACUCGGUUCGUUAGCU 5’

query=JS16_t0335339, target=FGSG_05201, score=3.5, range=512-530, strand=-1

target  5’ AUCUUGAGCCAAGCAA-CGG 3’
            ::::::::::::::: ::.
query   3’ AAGAACUCGGUUCGUUAGCU 5’

query=JS16_t0335339, target=FGSG_04317, score=4, range=1094-1112, strand=-1

target  5’ UUCUUG-GUCAAGUAAUUGA 3’
           :::::: :.::::.:::.::
query   3’ AAGAACUCGGUUCGUUAGCU 5’

query=JS16_t0335339, target=FGSG_00091, score=4, range=90-109, strand=-1

target  5’ UUCUGGAGGCGAGCAAUCGA 3’
           :::: ::: :.:::::::::
query   3’ AAGAACUCGGUUCGUUAGCU 5’

query=JS16_t0335339, target=FGSG_00973, score=4, range=902-920, strand=-1

target  5’ AUCUU-AGUCAAGCAAUUGA 3’
            :::: ::.::::::::.::
query   3’ AAGAACUCGGUUCGUUAGCU 5’

query=JS16_t0335339, target=FGSG_06799, score=4, range=3266-3284, strand=-1

target  5’ UUCUUGAGCCAAGCCA-CGA 3’
           :::::::::::::: : :::
query   3’ AAGAACUCGGUUCGUUAGCU 5’

query=JS16_t0335339, target=FGSG_12616, score=4, range=890-908, strand=-1

target  5’ UUCAUG-GCCAUGCAAUCGA 3’
           ::: :: :::: ::::::::
query   3’ AAGAACUCGGUUCGUUAGCU 5’

No results for JS16_t0117438
No results for JS16_t1331048
query=JS16_t0161884, target=FGSG_07720, score=3.5, range=104-121, strand=1

target  5’ AAUCU-CCAGUUUCAUCAU 3’
             ::: ::::::::::::.
query   3’ ACAGAUGGUCAAAGUAGUG 5’

query=JS16_t0161884, target=FGSG_10475, score=4, range=746-763, strand=1

target  5’ UGU-UACCAGUUUUGUCAG 3’
           ::: :::::::::..:::
query   3’ ACAGAUGGUCAAAGUAGUG 5’

query=JS16_t0161884, target=FGSG_06476, score=4, range=330-348, strand=1

target  5’ UGUCGACCAGCUUCAUCAA 3’
           :::: ::::: :::::::
query   3’ ACAGAUGGUCAAAGUAGUG 5’

query=JS16_t0161884, target=FGSG_06316, score=4, range=1377-1394, strand=1

target  5’ UGU-UGACAGUUUCAUCAU 3’
           ::: :.  :::::::::::.
query   3’ ACAGAUGGUCAAAGUAGUG 5’

query=JS16_t0161884, target=FGSG_03799, score=4, range=720-737, strand=1

target  5’ UG-CUGCCAUUUUCAUCAU 3’
           :: ::.::: ::::::::.
query   3’ ACAGAUGGUCAAAGUAGUG 5’

query=JS16_t0161884, target=FGSG_03367, score=4, range=387-404, strand=1

target  5’ UG-CUACCAUUUUCAUCAG 3’
           :: :::::: ::::::::
query   3’ ACAGAUGGUCAAAGUAGUG 5’

target  5’ AUG-GAGGAUGUCAAGUG 3’
           ::: :::::::.::::::
query   3’ UACGCUCCUACGGUUCAC 5’

query=JS16_t3186844, target=FGSG_05757, score=3, range=552-569, strand=1

target  5’ AUACGAGGAUGCCAAGGG 3’
           :: ::::::::::::: :
query   3’ UACGCUCCUACGGUUCAC 5’

query=JS16_t3186844, target=FGSG_09989, score=3, range=575-593, strand=1

target  5’ CUGCUGAGGAUGCCAAGUU 3’
            ::: :::::::::::::
query   3’ UACG-CUCCUACGGUUCAC 5’

query=JS16_t3186844, target=FGSG_08328, score=3, range=718-734, strand=1

target  5’ AU-CGAGGGUGCCAAGUC 3’
           :: :::::.::::::::
query   3’ UACGCUCCUACGGUUCAC 5’

query=JS16_t3186844, target=FGSG_02010, score=3, range=1211-1229, strand=1

target  5’ AUGAGAAGGAUGCCAAGUU 3’
           ::: : ::::::::::::
query   3’ UACGC-UCCUACGGUUCAC 5’

query=JS16_t3186844, target=FGSG_08122, score=3, range=166-182, strand=1

target  5’ AAGC-AGGGUGCCAAGUG 3’
           : :: :::.:::::::::
query   3’ UACGCUCCUACGGUUCAC 5’

query=JS16_t3186844, target=FGSG_01582, score=3, range=2141-2159, strand=-1

target  5’ UUGCGGAGGAUGUCAAGUG 3’
            ::: :::::::.::::::
query   3’ UACG-CUCCUACGGUUCAC 5’

query=JS16_t3186844, target=FGSG_03442, score=3.5, range=633-650, strand=1

target  5’ AUCUGAGGAUGUCAAGUU 3’
           :: .:::::::.:::::
query   3’ UACGCUCCUACGGUUCAC 5’

query=JS16_t3186844, target=FGSG_07857, score=3.5, range=453-470, strand=1

target  5’ AGAUGAGGAUGCCGAGUG 3’
           :  .:::::::::.::::
query   3’ UACGCUCCUACGGUUCAC 5’

query=JS16_t3186844, target=FGSG_05007, score=3.5, range=345-362, strand=1

target  5’ AGCUGAGGGUGCCAAGUG 3’
           :  .::::.:::::::::
query   3’ UACGCUCCUACGGUUCAC 5’

query=JS16_t3186844, target=FGSG_09894, score=3.5, range=88-105, strand=-1

target  5’ GCUCGAGGAUGCCGAGUG 3’
           .   ::::::::::.::::
query   3’ UACGCUCCUACGGUUCAC 5’

query=JS16_t3186844, target=FGSG_03032, score=3.5, range=362-380, strand=-1

target  5’ GUGCUGAGGUUGCCAAGUG 3’
           .::: :::: :::::::::
query   3’ UACG-CUCCUACGGUUCAC 5’

query=JS16_t3186844, target=FGSG_05373, score=3.5, range=1953-1970, strand=-1

target  5’ AGAUGAGGAUGCCAAGUU 3’
           :  .:::::::::::::
query   3’ UACGCUCCUACGGUUCAC 5’

query=JS16_t3186844, target=FGSG_01854, score=4, range=861-878, strand=1

target  5’ AUGCGAGGCUGCCGAGUU 3’
           :::::::: ::::.:::
query   3’ UACGCUCCUACGGUUCAC 5’

query=JS16_t3186844, target=FGSG_06016, score=4, range=3904-3921, strand=1

target  5’ AUGGGAGGAUACCAGGUG 3’
           ::: :::::: :::.:::
query   3’ UACGCUCCUACGGUUCAC 5’

query=JS16_t3186844, target=FGSG_03907, score=4, range=309-326, strand=1

target  5’ AUUCGAGGAUGCCAAGAA 3’
```

```
query=JS16_t0161884, target=FGSG_07778, score=4, range=818-835, strand=1

target  5’ UGGC-ACCAGUUUUAUCAG 3’
           :: : ::::::::.::::
query   3’ ACAGAUGGUCAAAGUAGUG 5’

query=JS16_t0161884, target=FGSG_07786, score=4, range=4-21, strand=1

target  5’ AGUCGACCAGUUUC-UCAC 3’
            ::: ::::::::: ::::
query   3’ ACAGAUGGUCAAAGUAGUG 5’

query=JS16_t0161884, target=FGSG_09994, score=4, range=1001-1019, strand=-1

target  5’ UGGCUAUCAGCUUCAUCAC 3’
           :: :::.::: ::::::::
query   3’ ACAGAUGGUCAAAGUAGUG 5’

query=JS16_t0161884, target=FGSG_08388, score=4, range=860-877, strand=-1

target  5’ UGUC-GCUAGUUUCAUCGU 3’
           :::: .:.:::::::::..
query   3’ ACAGAUGGUCAAAGUAGUG 5’

query=JS16_t0161884, target=FGSG_04333, score=4, range=503-521, strand=-1

target  5’ UAUUUGCCAGUUUCAUCUC 3’
           : :.:.::::::::::: :
query   3’ ACAGAUGGUCAAAGUAGUG 5’

query=JS16_t0161884, target=FGSG_10256, score=4, range=749-766, strand=-1

target  5’ UGGC-AUCAGUUUCAUCGC 3’
           :: : :.::::::::::.:
query   3’ ACAGAUGGUCAAAGUAGUG 5’

query=JS16_t0161884, target=FGSG_13169, score=4, range=386-403, strand=-1

target  5’ CAUCU-CCAGUUUCAUCAG 3’
             ::: ::::::::::::
query   3’ ACAGAUGGUCAAAGUAGUG 5’

query=JS16_t0161884, target=FGSG_08357, score=4, range=305-322, strand=-1

target  5’ UAUC-ACCAGGUUCAUCAC 3’
           : :: ::::: ::::::::
query   3’ ACAGAUGGUCAAAGUAGUG 5’

query=JS16_t4020332, target=FGSG_08648, score=3.5, range=1526-1544, strand=1

target  5’ GGUUCCCAAGG-UGGCAUUU 3’
           :.::::::::: ::::::.:
query   3’ CUAAGGGUUCCUACCGUAGA 5’

query=JS16_t4020332, target=FGSG_08249, score=3.5, range=532-551, strand=1

target  5’ GAUCUCCAAGGAUGGGAUCU 3’
           ::: .:::::::::: ::::
query   3’ CUAAGGGUUCCUACCGUAGA 5’

query=JS16_t4020332, target=FGSG_00434, score=4, range=1086-1105, strand=1

target  5’ GAUUCUUGAGGAUGGCAGCU 3’
           :::::...::::::::: ::
query   3’ CUAAGGGUUCCUACCGUAGA 5’

query=JS16_t4020332, target=FGSG_03311, score=4, range=1464-1483, strand=1

target  5’ GAUCACCAAGGAUGGUGUCU 3’
           :::  ::::::::::..:::
query   3’ CUAAGGGUUCCUACCGUAGA 5’

query=JS16_t4020332, target=FGSG_03620, score=4, range=2040-2059, strand=1

target  5’ CAUACCCAAGGAUGGCGUCG 3’
            :: ::::::::::::.::
query   3’ CUAAGGGUUCCUACCGUAGA 5’

query=JS16_t4020332, target=FGSG_03484, score=4, range=366-385, strand=1

target  5’ GCUUGCAGAGGAUGGCAUCU 3’
           : :: : .::::::::::::
query   3’ CUAAGGGUUCCUACCGUAGA 5’

query=JS16_t4020332, target=FGSG_10566, score=4, range=1137-1156, strand=-1

target  5’ GAUUCCCAGUGAUGGCAUCC 3’
           ::::::::. :::::::::
query   3’ CUAAGGGUUCCUACCGUAGA 5’

query=JS16_t4020332, target=FGSG_05263, score=4, range=1269-1289, strand=-1

           :: :::::::::::::
query   3’ UACGCUCCUACGGUUCAC 5’

query=JS16_t3186844, target=FGSG_04264, score=4, range=1097-1115, strand=1

target  5’ AUGACGAGGAUGCCAAGGA 3’
           ::: :::::::::::::
query   3’ UAC-GCUCCUACGGUUCAC 5’

query=JS16_t3186844, target=FGSG_06411, score=4, range=197-215, strand=1

target  5’ UUGUCGAGGAUGUCAAGUU 3’
            :: ::::::::.:::::
query   3’ UAC-GCUCCUACGGUUCAC 5’

query=JS16_t3186844, target=FGSG_05040, score=4, range=685-701, strand=1

target  5’ UUGCG-GGAUGUCAAGUG 3’
            :::: :::::.::::::
query   3’ UACGCUCCUACGGUUCAC 5’

query=JS16_t3186844, target=FGSG_05338, score=4, range=558-575, strand=1

target  5’ UCCCGAGGAUGCCAAGUU 3’
              :::::::::::::
query   3’ UACGCUCCUACGGUUCAC 5’

query=JS16_t3186844, target=FGSG_08481, score=4, range=36-53, strand=1

target  5’ AGACGAGGAUGCCGAGUA 3’
           :  ::::::::::.:::
query   3’ UACGCUCCUACGGUUCAC 5’

query=JS16_t3186844, target=FGSG_01414, score=4, range=333-350, strand=1

target  5’ GCUUGAGGAUGCCAAGUC 3’
           .  .:::::::::::::
query   3’ UACGCUCCUACGGUUCAC 5’

query=JS16_t3186844, target=FGSG_00625, score=4, range=573-590, strand=1

target  5’ AUGGGAGGACGCCAAGUC 3’
           ::: ::::: :::::::
query   3’ UACGCUCCUACGGUUCAC 5’

query=JS16_t3186844, target=FGSG_10539, score=4, range=867-884, strand=1

target  5’ AAGCGAGGAUGCCAUGUC 3’
           : ::::::::::: ::
query   3’ UACGCUCCUACGGUUCAC 5’

query=JS16_t3186844, target=FGSG_11150, score=4, range=768-785, strand=1

target  5’ CUACGAGGAUGCCAAGCG 3’
            : ::::::::::::: :
query   3’ UACGCUCCUACGGUUCAC 5’

query=JS16_t3186844, target=FGSG_09981, score=4, range=1553-1571, strand=1

target  5’ AUGCUGAGGAUGCCAACUC 3’
           :::: :::::::::: :
query   3’ UACG-CUCCUACGGUUCAC 5’

query=JS16_t3186844, target=FGSG_09023, score=4, range=731-749, strand=1

target  5’ UUGACGAGGAUGCCAAGCG 3’
            :: ::::::::::::: :
query   3’ UAC-GCUCCUACGGUUCAC 5’

query=JS16_t3186844, target=FGSG_06788, score=4, range=753-770, strand=1

target  5’ AUGGUGGGAUGCCAAGUC 3’
           :::  .:::::::::::
query   3’ UACGCUCCUACGGUUCAC 5’

query=JS16_t3186844, target=FGSG_00906, score=4, range=2529-2546, strand=1

target  5’ CAACAAGGAUGCCAAGUG 3’
              : :::::::::::::
query   3’ UACGCUCCUACGGUUCAC 5’

query=JS16_t3186844, target=FGSG_01059, score=4, range=478-494, strand=1

target  5’ AUG-GAGGAUGCCAAGCC 3’
           ::: :::::::::::
query   3’ UACGCUCCUACGGUUCAC 5’

query=JS16_t3186844, target=FGSG_05596, score=4, range=1024-1042, strand=1

target  5’ AUGACCAGGAUACCAAGUG 3’
           ::: : ::::: :::::::
query   3’ UAC-GCUCCUACGGUUCAC 5’
```

```
target  5' GACUCCGCAAGGAUGGCAUUC 3'
           :: ::: ::::::::::::.
query   3' CUAAGG-GUUCCUACCGUAGA 5'

query=JS16_t4020332, target=FGSG_04305, score=4, range=378-397, strand=-1

target  5' GAGGCCCAAGGAUGGCAACU 3'
           ::  ::::::::::::: ::
query   3' CUAAGGGUUCCUACCGUAGA 5'

No results for JS16_t4020335
query=JS16_t0467391, target=FGSG_06238, score=3, range=3553-3571, strand=-1

target  5' AUGCUAUCGAGGUUGGUGA 3'
           ::::::.:::::::::: :
query   3' UACGAUGGCUCCAACCAAU 5'

query=JS16_t0467391, target=FGSG_00633, score=3.5, range=3454-3472, strand=-1

target  5' AUGCUUCCGAGGAUGGUUG 3'
           ::::: :::::: :::::.
query   3' UACGAUGGCUCCAACCAAU 5'

query=JS16_t0467391, target=FGSG_04332, score=3.5, range=941-959, strand=-1

target  5' GUGACAUCGAGGUUGGUUA 3'
           .::  :.:::::::::::
query   3' UACGAUGGCUCCAACCAAU 5'

query=JS16_t0467391, target=FGSG_08013, score=3.5, range=1477-1494, strand=-1

target  5' AGGC-AUCGAGGUUGGUUG 3'
           : :: :.:::::::::::.
query   3' UACGAUGGCUCCAACCAAU 5'

query=JS16_t0467391, target=FGSG_10569, score=4, range=2540-2558, strand=1

target  5' UUGCUACCGAGGUUGGGUU 3'
            ::::::::::::::: :
query   3' UACGAUGGCUCCAACCAAU 5'

query=JS16_t0467391, target=FGSG_04756, score=4, range=491-509, strand=1

target  5' AAGCUGCCGAGAUUGGUUG 3'
           : :::.::::: ::::::.
query   3' UACGAUGGCUCCAACCAAU 5'

query=JS16_t0467391, target=FGSG_07924, score=4, range=146-163, strand=1

target  5' CUGCUGCCGAGGUU-GUUG 3'
            ::::.:::::::: :::.
query   3' UACGAUGGCUCCAACCAAU 5'

query=JS16_t0467391, target=FGSG_05467, score=4, range=332-349, strand=1

target  5' AUCCUGCCGAGGUU-GUUG 3'
           :: ::.:::::::: :::.
query   3' UACGAUGGCUCCAACCAAU 5'

query=JS16_t0467391, target=FGSG_13343, score=4, range=1567-1584, strand=1

target  5' AUG-AGCCGGGGUUGGUUG 3'
           :::  .:::.::::::::.
query   3' UACGAUGGCUCCAACCAAU 5'

No results for JS16_t3123729
query=JS16_t0394141, target=FGSG_10949, score=3, range=1836-1854, strand=1

target  5' CUACCCGGGCAAGUACACU 3'
           :: :::.::::::.:::::
query   3' GAAGGGUCCGUUCGUGUGA 5'

query=JS16_t0394141, target=FGSG_09921, score=3.5, range=1193-1212, strand=1

target  5' CUCUUCCAGGCGAGUACACU 3'
           :: :.::::::.::.:::::
query   3' GA-AGGGUCCGUUCGUGUGA 5'

query=JS16_t0394141, target=FGSG_08939, score=3.5, range=614-632, strand=1

target  5' UUUCCAAGGCUAGCACACU 3'
           .:::: :::: ::::::::
query   3' GAAGGGUCCGUUCGUGUGA 5'

query=JS16_t0394141, target=FGSG_05213, score=4, range=5348-5367, strand=1

target  5' CUUCACCAGGCAAGCCCAUU 3'
           :::: :::::::::: ::.:
query   3' GAAG-GGUCCGUUCGUGUGA 5'

query=JS16_t0394141, target=FGSG_00184, score=4, range=659-678, strand=1

query=JS16_t3186844, target=FGSG_11169, score=4, range=1457-1475, strand=1

target  5' AUGGAGAGGAUGCCAAGCG 3'
           :: : ::::::::::::: :
query   3' UA-CGCUCCUACGGUUCAC 5'

query=JS16_t3186844, target=FGSG_06751, score=4, range=226-242, strand=1

target  5' AU-CGAGGCUGCCAAGUC 3'
           :: ::::: ::::::::
query   3' UACGCUCCUACGGUUCAC 5'

query=JS16_t3186844, target=FGSG_12701, score=4, range=225-241, strand=1

target  5' UUUCGAGGAUGCCAA-UG 3'
            : :::::::::::: ::
query   3' UACGCUCCUACGGUUCAC 5'

query=JS16_t3186844, target=FGSG_12034, score=4, range=3254-3270, strand=1

target  5' AAGC-AGGAAGCCAAGUG 3'
           : :: :::: ::::::::
query   3' UACGCUCCUACGGUUCAC 5'

query=JS16_t3186844, target=FGSG_02082, score=4, range=1835-1851, strand=1

target  5' CUGAGAGGAU-CCAAGUG 3'
            :: :::::: :::::::
query   3' UACGCUCCUACGGUUCAC 5'

query=JS16_t3186844, target=FGSG_05545, score=4, range=1127-1144, strand=-1

target  5' CAGCGAGGAUGCUGAGUG 3'
             ::::::::::..::::
query   3' UACGCUCCUACGGUUCAC 5'

query=JS16_t3186844, target=FGSG_09379, score=4, range=624-641, strand=-1

target  5' UUGCGAGGAUUCCAAGUC 3'
            ::::::::: ::::::
query   3' UACGCUCCUACGGUUCAC 5'

query=JS16_t3186844, target=FGSG_05149, score=4, range=3690-3707, strand=-1

target  5' AUGAUGGGAUGCCAGGUG 3'
           :::  .::::::::.:::
query   3' UACGCUCCUACGGUUCAC 5'

query=JS16_t3186844, target=FGSG_08015, score=4, range=152-169, strand=-1

target  5' GGGUGAGGACGCCAAGUG 3'
           .  :.::::: ::::::::
query   3' UACGCUCCUACGGUUCAC 5'

query=JS16_t3186844, target=FGSG_04869, score=4, range=366-384, strand=-1

target  5' GUACGGGCGAUGCCAAGUG 3'
           .: ::.: :::::::::::
query   3' UACGCUC-CUACGGUUCAC 5'

query=JS16_t3186844, target=FGSG_13781, score=4, range=182-199, strand=-1

target  5' UUUCGAGGAUGCCAAGGG 3'
            : ::::::::::::: :
query   3' UACGCUCCUACGGUUCAC 5'

query=JS16_t3186844, target=FGSG_01064, score=4, range=1134-1151, strand=-1

target  5' AACCGAGGAUGCCAAGGG 3'
           :  ::::::::::::: :
query   3' UACGCUCCUACGGUUCAC 5'

query=JS16_t3186844, target=FGSG_06454, score=4, range=502-520, strand=-1

target  5' AUCCCAGCGAUGCCAAGUG 3'
           :: : :: :::::::::::
query   3' UACGCUC-CUACGGUUCAC 5'

No results for JS16_t3186849
query=JS16_t3186858, target=FGSG_09265, score=3.5, range=1609-1628, strand=-1

target  5' UUCCACU-CCGCAUGCCGGUG 3'
           ::::::.  ::::::::::::
query   3' AAGGUGGUUGCGUACGGCCAC 5'

No results for JS16_t2290604
query=JS16_t4083504, target=FGSG_11185, score=3.5, range=2874-2894, strand=1

target  5' GUUGUUUGAUAUCAUUUCAUG 3'
           ::.: ..::::::::.:::::
query   3' CAGC-GGCUAUAGUAGAGUAC 5'
```

```
target  5' CUACCUGGGCCAAGCACACU 3'
           :: ::..:: ::::::::::
query   3' GAAGGGUCC-GUUCGUGUGA 5'

query=JS16_t0394141, target=FGSG_00659, score=4, range=1197-1214, strand=-1

target  5' CCUCCCAGG-AAGCACACC 3'
           : ::::::: ::::::::
query   3' GAAGGGUCCGUUCGUGUGA 5'

No results for JS16_t1331115
query=JS16_t2227613, target=FGSG_11659, score=4, range=7708-7729, strand=1

target  5' CCCGA-AGCCAAACACCUUGUCG 3'
           :: :: ::::::::::::.:.::
query   3' GGUCUCUCGGUUUGUGGAGCGGC 5'

query=JS16_t0228394, target=FGSG_04432, score=2.5, range=632-650, strand=1

target  5' AGAAUAUAGACUGGUUUCA 3'
           :: :.::::::::::.::
query   3' UC-UGUAUCUGACCAAGGU 5'

query=JS16_t0228394, target=FGSG_09019, score=3, range=1201-1218, strand=-1

target  5' AGGCAUAGGCUGGUUCUG 3'
           ::.:::::.:::::::..
query   3' UCUGUAUCUGACCAAGGU 5'

query=JS16_t0228394, target=FGSG_07118, score=3, range=2604-2621, strand=-1

target  5' AGACCUAGACCGGUUCCA 3'
           :::: ::::: :::::::
query   3' UCUGUAUCUGACCAAGGU 5'

query=JS16_t0228394, target=FGSG_02588, score=3.5, range=147-164, strand=1

target  5' GGACAUUGACUGGUUCUA 3'
           .::::: :::::::::.:
query   3' UCUGUAUCUGACCAAGGU 5'

query=JS16_t0228394, target=FGSG_07916, score=3.5, range=78-95, strand=1

target  5' AGAUAUAGAUCGGUUCCA 3'
           :::.:::::. :::::::
query   3' UCUGUAUCUGACCAAGGU 5'

query=JS16_t0228394, target=FGSG_12023, score=3.5, range=265-281, strand=1

target  5' AAAC-UAGACUGGUUUCG 3'
           : :: ::::::::::.:.
query   3' UCUGUAUCUGACCAAGGU 5'

query=JS16_t0228394, target=FGSG_09321, score=3.5, range=348-366, strand=-1

target  5' GGACAUGGACUCGGUUCCA 3'
           .:::::.:::: :::::::
query   3' UCUGUAUCUGA-CCAAGGU 5'

query=JS16_t0228394, target=FGSG_12026, score=4, range=1473-1489, strand=1

target  5' AGAUG-AGACUGGUUCUA 3'
           :::.. ::::::::::.:
query   3' UCUGUAUCUGACCAAGGU 5'

query=JS16_t0228394, target=FGSG_04913, score=4, range=1287-1305, strand=1

target  5' UCACAUAGACUGGUUCGCA 3'
             :::::::::::::: ::
query   3' UCUGUAUCUGACCAAG-GU 5'

query=JS16_t0228394, target=FGSG_06684, score=4, range=1239-1256, strand=1

target  5' ACAUGUAGACUUGUUCCA 3'
           : :..:::::: ::::::
query   3' UCUGUAUCUGACCAAGGU 5'

query=JS16_t0228394, target=FGSG_11451, score=4, range=549-565, strand=1

target  5' AGAC-UACAUUGGUUCCA 3'
           :::: :: :.::::::::
query   3' UCUGUAUCUGACCAAGGU 5'

query=JS16_t0228394, target=FGSG_01772, score=4, range=653-670, strand=1

target  5' ACAAAAAGACUGGUUCCA 3'
           : : : :::::::::::
query   3' UCUGUAUCUGACCAAGGU 5'

query=JS16_t0228394, target=FGSG_05297, score=4, range=892-909, strand=-1

target  5' AGGCAUAGACAGGUUCUG 3'

query=JS16_t4083504, target=FGSG_00253, score=3.5, range=331-349, strand=1

target  5' GUCCUCGAUAUCAU-UCAUG 3'
           ::: .::::::::: :::::
query   3' CAGCGGCUAUAGUAGAGUAC 5'

query=JS16_t4083504, target=FGSG_07810, score=4, range=574-593, strand=1

target  5' GUCGCCGCUGUCAUCUCAUU 3'
           ::::::: :.:::::::::
query   3' CAGCGGCUAUAGUAGAGUAC 5'

query=JS16_t4083504, target=FGSG_05976, score=4, range=309-327, strand=-1

target  5' GUCGUUGAUGUCAU-UCAUG 3'
           ::::..:::.:::: :::::
query   3' CAGCGGCUAUAGUAGAGUAC 5'

query=JS16_t4083504, target=FGSG_04095, score=4, range=1846-1866, strand=-1

target  5' GUUCUCGAAUAUCAUUUCAUG 3'
           ::. .:: :::::::.:::::
query   3' CAGCGGC-UAUAGUAGAGUAC 5'

query=JS16_t4083504, target=FGSG_00259, score=4, range=1545-1563, strand=-1

target  5' GGCGCCGAUAUCGUCU-AUG 3'
           : ::::::::::.::: :::
query   3' CAGCGGCUAUAGUAGAGUAC 5'

No results for JS16_t3186922
query=JS16_t0157698, target=FGSG_04975, score=2.5, range=125-142, strand=1

target  5' GUAACGACGAUGGCUCUU 3'
           :: :::::.:::::::::.
query   3' CAGUGCUGUUACCGAGAG 5'

query=JS16_t0157698, target=FGSG_10234, score=2.5, range=1796-1813, strand=1

target  5' GUCUUGACAAUGGCUCUA 3'
           ::: .:::::::::::
query   3' CAGUGCUGUUACCGAGAG 5'

query=JS16_t0157698, target=FGSG_10338, score=3, range=71-87, strand=1

target  5' GUUAUGACAAUGGC-CUC 3'
           ::.:.::::::::: :::
query   3' CAGUGCUGUUACCGAGAG 5'

query=JS16_t0157698, target=FGSG_09116, score=3, range=1739-1756, strand=-1

target  5' UAUACGACAAUGGCUCUU 3'
             .::::::::::::::.
query   3' CAGUGCUGUUACCGAGAG 5'

query=JS16_t0157698, target=FGSG_01501, score=3, range=483-499, strand=-1

target  5' GUU-CGAUAAUGGCUCUU 3'
           ::.  :::.:::::::::.
query   3' CAGUGCUGUUACCGAGAG 5'

query=JS16_t0157698, target=FGSG_06533, score=3.5, range=1759-1776, strand=1

target  5' GUCAUGACAGUGGUUUUC 3'
           ::::.::::.:::.:.::
query   3' CAGUGCUGUUACCGAGAG 5'

query=JS16_t0157698, target=FGSG_01130, score=3.5, range=930-947, strand=1

target  5' GUCGAGACAGUGGCUUUC 3'
           :::.  ::::.:::::.::
query   3' CAGUGCUGUUACCGAGAG 5'

query=JS16_t0157698, target=FGSG_06759, score=3.5, range=788-805, strand=1

target  5' GCAAUGGCAAUGGCUCUC 3'
           :  :.:.:::::::::::
query   3' CAGUGCUGUUACCGAGAG 5'

query=JS16_t0157698, target=FGSG_10759, score=3.5, range=695-712, strand=1

target  5' GUUCCGACAAUGGCUAUC 3'
           ::.  ::::::::::: ::
query   3' CAGUGCUGUUACCGAGAG 5'

query=JS16_t0157698, target=FGSG_08394, score=3.5, range=274-291, strand=1

target  5' GUGACGACAAAGGCUCUU 3'
           :: ::::::: ::::::.
query   3' CAGUGCUGUUACCGAGAG 5'
```

```
           ::.:::::::: :::::..
query   3’ UCUGUAUCUGACCAAGGU 5’

query=JS16_t0228394, target=FGSG_01371, score=4, range=315-333, strand=-1

target  5’ AGGUAUAGAUUGGUUGCCA 3’
           ::..:::::.::::: :::
query   3’ UCUGUAUCUGACCAA-GGU 5’

query=JS16_t0228394, target=FGSG_05622, score=4, range=1414-1432, strand=-1

target  5’ AGACAUUAGGCUGGUCCCA 3’
           ::::: :::.::::: :::
query   3’ UCUGU-AUCUGACCAAGGU 5’

query=JS16_t0228394, target=FGSG_01366, score=4, range=779-796, strand=-1

target  5’ UGAGUUGGACUGGUUCCA 3’
            ::  :.:::::::::::
query   3’ UCUGUAUCUGACCAAGGU 5’

query=JS16_t0228394, target=FGSG_07759, score=4, range=293-309, strand=-1

target  5’ AGAC-UGGACUGAUUCCA 3’
           :::: :.::::: :::::
query   3’ UCUGUAUCUGACCAAGGU 5’

query=JS16_t0228394, target=FGSG_00641, score=4, range=275-291, strand=-1

target  5’ GGGCAU-GAUUGGUUCCA 3’
           .:.::: ::.::::::::
query   3’ UCUGUAUCUGACCAAGGU 5’

No results for JS16_t1331151
query=JS16_t2227655, target=FGSG_04082, score=4, range=939-959, strand=1

target  5’ CUGGAACACGCCGAUUGUUCG 3’
           :::: :: ::::.::.:::::
query   3’ GACC-UGAGCGGUUAGCAAGC 5’

query=JS16_t2227655, target=FGSG_08729, score=4, range=927-945, strand=1

target  5’ CUGUAC-CGUCGAUCGUUCG 3’
           ::: :: ::.:.::::::::
query   3’ GACCUGAGCGGUUAGCAAGC 5’

query=JS16_t2227655, target=FGSG_04802, score=4, range=1754-1772, strand=1

target  5’ CUGGUCUCGCCAAUC-UUCU 3’
           :::: :::::::::: :::
query   3’ GACCUGAGCGGUUAGCAAGC 5’

query=JS16_t2227655, target=FGSG_08614, score=4, range=1732-1751, strand=-1

target  5’ UUGGGCUCGCCAAUCUUUUG 3’
           .:::.:::::::::: ::.:
query   3’ GACCUGAGCGGUUAGCAAGC 5’

No results for JS16_t1331166
No results for JS16_t0794358
No results for JS16_t3123850
No results for JS16_t0394146
query=JS16_t0467419, target=FGSG_06448, score=3.5, range=1229-1247, strand=1

target  5’ AAGACU-GGCUGGAGUCUAU 3’
           : .::: :::.:::::::::
query   3’ UCUUGACCCGGCCUCAGAUA 5’

query=JS16_t0467419, target=FGSG_01316, score=3.5, range=1191-1210, strand=-1

target  5’ AGAGCUGGGCUGGAGUCUUU 3’
           :::.::::::.::::::: :
query   3’ UCUUGACCCGGCCUCAGAUA 5’

No results for JS16_t1331346
No results for JS16_t4020624
No results for JS16_t1331358
No results for JS16_t2227855
No results for JS16_t4020705
query=JS16_t4020710, target=FGSG_01710, score=4, range=517-536, strand=1

target  5’ UGUCUUUACAUGGCCGCUGU 3’
           :::: : :::::.::.::::
query   3’ ACAGCACUGUACUGGUGACA 5’

query=JS16_t4020710, target=FGSG_05415, score=4, range=693-713, strand=1

target  5’ UGCCGAUGGCAUGACUACUGU 3’
           :: :: ::.:::::::.:::::
query   3’ ACAGC-ACUGUACUGGUGACA 5’

query=JS16_t4020710, target=FGSG_08047, score=4, range=858-878, strand=1

query=JS16_t0157698, target=FGSG_05015, score=3.5, range=653-671, strand=1

target  5’ GUGGCGACAAUGGCAUCUC 3’
           :: .:::::::::: ::::
query   3’ CAGUGCUGUUACCG-AGAG 5’

query=JS16_t0157698, target=FGSG_08701, score=3.5, range=1418-1435, strand=1

target  5’ AUCUAGACAAUGGCUCUU 3’
            ::  ::::::::::::.
query   3’ CAGUGCUGUUACCGAGAG 5’

query=JS16_t0157698, target=FGSG_04416, score=3.5, range=718-735, strand=-1

target  5’ GUCGCGAUGAUGGCUCUG 3’
           :::.:::..::::::::
query   3’ CAGUGCUGUUACCGAGAG 5’

query=JS16_t0157698, target=FGSG_07764, score=3.5, range=1144-1161, strand=-1

target  5’ GUCACGACGAUGGAUCUU 3’
           ::::::::.:::: :::.
query   3’ CAGUGCUGUUACCGAGAG 5’

query=JS16_t0157698, target=FGSG_10633, score=3.5, range=1170-1187, strand=-1

target  5’ GUCAUGACAAUAGCUUUC 3’
           ::::.:::::: :::.::
query   3’ CAGUGCUGUUACCGAGAG 5’

query=JS16_t0157698, target=FGSG_09408, score=3.5, range=1971-1988, strand=-1

target  5’ GGCAUGACAAUGGCGCUC 3’
           : ::.:::::::::: :::
query   3’ CAGUGCUGUUACCGAGAG 5’

query=JS16_t0157698, target=FGSG_01948, score=3.5, range=2933-2951, strand=-1

target  5’ GUCACAACAAUGGCCUCUU 3’
           ::::: ::::::: ::::.
query   3’ CAGUGCUGUUACC-GAGAG 5’

query=JS16_t0157698, target=FGSG_05384, score=3.5, range=681-699, strand=-1

target  5’ GACGCCGACAAUGGCUCUA 3’
           : :. :::::::::::::
query   3’ CAGU-GCUGUUACCGAGAG 5’

query=JS16_t0157698, target=FGSG_00262, score=3.5, range=640-656, strand=-1

target  5’ GUCAUGAUAA-GGCUCUC 3’
           ::::.::.:: :::::::
query   3’ CAGUGCUGUUACCGAGAG 5’

query=JS16_t0157698, target=FGSG_04785, score=3.5, range=2134-2152, strand=-1

target  5’ GUCGCGACAUAUGGUUCUC 3’
           :::.::::: ::::.::::
query   3’ CAGUGCUGU-UACCGAGAG 5’

query=JS16_t0157698, target=FGSG_12966, score=4, range=113-130, strand=1

target  5’ GUUUCGAUAAUGGCUUUU 3’
           ::.  :::.:::::::.:.
query   3’ CAGUGCUGUUACCGAGAG 5’

query=JS16_t0157698, target=FGSG_04245, score=4, range=2530-2548, strand=1

target  5’ GUCGAUGACAGUGGCUUUU 3’
           ::: :.::::.:::::.:.
query   3’ CAG-UGCUGUUACCGAGAG 5’

query=JS16_t0157698, target=FGSG_10526, score=4, range=590-607, strand=1

target  5’ CACGUGAUAAUGGCUCUC 3’
             :..::.::::::::::
query   3’ CAGUGCUGUUACCGAGAG 5’

query=JS16_t0157698, target=FGSG_05358, score=4, range=857-874, strand=1

target  5’ CUCUCGGUAAUGGCUCUC 3’
            :: ::..::::::::::
query   3’ CAGUGCUGUUACCGAGAG 5’

query=JS16_t0157698, target=FGSG_07369, score=4, range=848-865, strand=1

target  5’ AUCGCGACAACGGCUCUU 3’
            ::.::::::: ::::::.
query   3’ CAGUGCUGUUACCGAGAG 5’

query=JS16_t0157698, target=FGSG_04279, score=4, range=301-319, strand=1
```

```
target  5' UGUCGGUUACAUGGCCAUUGU 3'
           :::: :: :::::.:::.:::
query   3' ACAG-CACUGUACUGGUGACA 5'

query=JS16_t4020710, target=FGSG_01917, score=4, range=1028-1048, strand=-1

target  5' UGUCGAUGAUGUGACCAUUGU 3'
           ::::: :::..::::::.:::
query   3' ACAGC-ACUGUACUGGUGACA 5'

No results for JS16_t4020727
No results for JS16_t4020733
No results for JS16_t1331467
No results for JS16_t3124120
query=JS16_t2227967, target=FGSG_11048, score=3.5, range=921-939, strand=1

target  5' UGGAG-CGGGAUUGUUUUAU 3'
            .::: ::::::.:::::::
query   3' UUCUCAGCCCUAGCAAAAUA 5'

No results for JS16_t1331531
No results for JS16_t1331579
No results for JS16_t3124204
query=JS16_t3124268, target=FGSG_01107, score=3.5, range=1619-1641, strand=-1

target  5' UUUUGGA-CAUCGUUUCUGACUCA 3'
           :.:::.: ::.::.::::::::::
query   3' AGAACUUCGUGGCGAAGACUGAGU 5'

query=JS16_t3124268, target=FGSG_05220, score=3.5, range=63-85, strand=-1

target  5' GCCUGGAG-ACCGCUUCUGACUCA 3'
            : ::.:: ::::::::::::::
query   3' AGAACUUCGUGGCGAAGACUGAGU 5'

query=JS16_t4020932, target=FGSG_00203, score=2.5, range=942-959, strand=-1

target  5' AUCAU-GUAGACGAGUAGU 3'
           :.: : :::::::::::::
query   3' UGGCAGCAUCUGCUCAUCA 5'

query=JS16_t4020932, target=FGSG_08477, score=3, range=335-354, strand=-1

target  5' AUCGUUGUAGACGUAGUAGU 3'
           :.:::.::::::: ::::::
query   3' UGGCAGCAUCUGC-UCAUCA 5'

query=JS16_t4020932, target=FGSG_04480, score=3.5, range=324-342, strand=-1

target  5' AGCGUUGUAGGCGGGUAGU 3'
           : :::.::::.::.:::::
query   3' UGGCAGCAUCUGCUCAUCA 5'

query=JS16_t4020932, target=FGSG_00220, score=3.5, range=1382-1401, strand=-1

target  5' ACCGUUGUAGACGAAGUAGC 3'
           :::::.::::::: :::::
query   3' UGGCAGCAUCUGC-UCAUCA 5'

query=JS16_t4020932, target=FGSG_10036, score=4, range=708-726, strand=-1

target  5' AUCGUUGUGGACGUGUAGU 3'
           :.:::.::.:::: :::::
query   3' UGGCAGCAUCUGCUCAUCA 5'

query=JS16_t4020932, target=FGSG_03475, score=4, range=515-534, strand=-1

target  5' GCUGUGCGAAGACGAGUAGU 3'
           .:.:: :: :::::::::::
query   3' UGGCA-GCAUCUGCUCAUCA 5'

query=JS16_t4020932, target=FGSG_03033, score=4, range=942-959, strand=-1

target  5' GCC-UUGUAGAUGAGUAGC 3'
           .:: :.:::::.::::::
query   3' UGGCAGCAUCUGCUCAUCA 5'

query=JS16_t4020932, target=FGSG_03685, score=4, range=532-549, strand=-1

target  5' ACCG-CGUAGACGAGUAAA 3'
           :::: ::::::::::::
query   3' UGGCAGCAUCUGCUCAUCA 5'

query=JS16_t3124289, target=FGSG_03223, score=3, range=2450-2470, strand=-1

target  5' CCCAGGCGCGUCGGUCACCAU 3'
           ::: :: :::::::::::::
query   3' GGGACC-CGCAGCCAGUGGUU 5'

query=JS16_t3124289, target=FGSG_02056, score=3.5, range=1762-1780, strand=-1
```

```
target  5' AUCAGCGGCGAUGGCUCUC 3'
            ::: ::.:.:::::::::
query   3' CAGU-GCUGUUACCGAGAG 5'

query=JS16_t0157698, target=FGSG_11418, score=4, range=79-96, strand=1

target  5' GUCACGACAACCGCUCUC 3'
           ::::::::::  ::::::
query   3' CAGUGCUGUUACCGAGAG 5'

query=JS16_t0157698, target=FGSG_04103, score=4, range=581-599, strand=1

target  5' GUCCCGACAAUGGCGUUUC 3'
           ::: :::::::::: :.::
query   3' CAGUGCUGUUACCG-AGAG 5'

query=JS16_t0157698, target=FGSG_10158, score=4, range=388-405, strand=1

target  5' GGCAAGGCAAUGGCUUUC 3'
           : :: :.::::::::.::
query   3' CAGUGCUGUUACCGAGAG 5'

query=JS16_t0157698, target=FGSG_10145, score=4, range=364-382, strand=1

target  5' GUAUACAACAAUGGCUCUU 3'
           :: .:: :::::::::::.
query   3' CA-GUGCUGUUACCGAGAG 5'

query=JS16_t0157698, target=FGSG_01910, score=4, range=230-247, strand=1

target  5' UUAACGACGAUGGCUCUG 3'
            : :::::.::::::::
query   3' CAGUGCUGUUACCGAGAG 5'

query=JS16_t0157698, target=FGSG_04058, score=4, range=476-493, strand=1

target  5' CUGACAACAAUGGCUCUC 3'
            : :: ::::::::::::
query   3' CAGUGCUGUUACCGAGAG 5'

query=JS16_t0157698, target=FGSG_10508, score=4, range=200-218, strand=1

target  5' CUCAAGACAAUGGCGUCUC 3'
            ::: ::::::::: ::::
query   3' CAGUGCUGUUACCG-AGAG 5'

query=JS16_t0157698, target=FGSG_03083, score=4, range=568-586, strand=1

target  5' AUCGACAACAAUGGCUCUC 3'
            :: :: ::::::::::::
query   3' CAG-UGCUGUUACCGAGAG 5'

query=JS16_t0157698, target=FGSG_08606, score=4, range=197-214, strand=1

target  5' GAAACGACCAUGGCUCUC 3'
           :  ::::: :::::::::
query   3' CAGUGCUGUUACCGAGAG 5'

query=JS16_t0157698, target=FGSG_02637, score=4, range=628-645, strand=1

target  5' GGAAAGACAAUGGCUUUC 3'
           :  : ::::::::::.::
query   3' CAGUGCUGUUACCGAGAG 5'

query=JS16_t0157698, target=FGSG_06804, score=4, range=923-940, strand=1

target  5' GUGCCGACAUUGGCUCUC 3'
           ::  ::::: ::::::::
query   3' CAGUGCUGUUACCGAGAG 5'

query=JS16_t0157698, target=FGSG_13899, score=4, range=10-28, strand=1

target  5' GGCACAAUCAAUGGCUCUC 3'
           : ::: : :::::::::::
query   3' CAGUGCU-GUUACCGAGAG 5'

query=JS16_t0157698, target=FGSG_00442, score=4, range=234-250, strand=1

target  5' AUC-CGACAAUGGCCCUC 3'
            :: :::::::::: :::
query   3' CAGUGCUGUUACCGAGAG 5'

query=JS16_t0157698, target=FGSG_08235, score=4, range=1979-1995, strand=1

target  5' CUCGCG-CAAUGGCUCUU 3'
            ::.:: ::::::::::.
query   3' CAGUGCUGUUACCGAGAG 5'

query=JS16_t0157698, target=FGSG_01359, score=4, range=624-642, strand=-1

target  5' GUCACCGACGAUGACUCUC 3'
           :::: ::::.::: :::::
```

```
target  5' CACU-GGCGUUGGUCACCAG 3'
           : :: :::::.::::::::.
query   3' GGGACCCGCAGCCAGUGGUU 5'

query=JS16_t3124289, target=FGSG_00475, score=3.5, range=301-319, strand=-1

target  5' CGCU-GUCGUCGGUCACCAG 3'
           : :: : ::::::::::::.
query   3' GGGACCCGCAGCCAGUGGUU 5'

query=JS16_t3124289, target=FGSG_00850, score=4, range=634-653, strand=1

target  5' UCUUGGUCGUCGGUCAGCAA 3'
           .:.::: ::::::::: :::
query   3' GGGACCCGCAGCCAGUGGUU 5'

query=JS16_t3124289, target=FGSG_04312, score=4, range=927-945, strand=1

target  5' CUCU-GCCGUCGGUUACCAG 3'
           :.:: : :::::::.::::.
query   3' GGGACCCGCAGCCAGUGGUU 5'

No results for JS16_t2228134
No results for JS16_t4021029
No results for JS16_t0305562
No results for JS16_t4021052
No results for JS16_t3124439
No results for JS16_t0685162
No results for JS16_t2228284
No results for JS16_t3124512
No results for JS16_t3124522
No results for JS16_t0254480
No results for JS16_t4021173
query=JS16_t1331882, target=FGSG_03648, score=3, range=157-177, strand=-1

target  5' GACAAUUUCUUCAGCAAUUUG 3'
           : :::::::::::.:::.:::
query   3' CCGUUAAAGAAGUUGUUGAAC 5'

query=JS16_t1331882, target=FGSG_04804, score=3, range=742-762, strand=-1

target  5' GGUAGUAUCUUCAACGACUUG 3'
           ::.:.: ::::::::.:::::
query   3' CCGUUAAAGAAGUUGUUGAAC 5'

query=JS16_t1331882, target=FGSG_07691, score=3, range=418-438, strand=-1

target  5' GCCAAAUUCUUCGACAACUUG 3'
           : ::: ::::::.::::::::
query   3' CCGUUAAAGAAGUUGUUGAAC 5'

query=JS16_t1331882, target=FGSG_06042, score=3.5, range=175-195, strand=-1

target  5' GGCGAUAUUUUCAGCAACUUG 3'
           :::.:: :.::::.:::::::
query   3' CCGUUAAAGAAGUUGUUGAAC 5'

query=JS16_t1331882, target=FGSG_10742, score=4, range=943-963, strand=1

target  5' AACAAUUUCUUCAACAACCUG 3'
             :::::::::::::::: ::
query   3' CCGUUAAAGAAGUUGUUGAAC 5'

query=JS16_t1331882, target=FGSG_10987, score=4, range=367-387, strand=1

target  5' UACAAGUUUUUCAACAACUUG 3'
             ::: ::.::::::::::::
query   3' CCGUUAAAGAAGUUGUUGAAC 5'

query=JS16_t1331882, target=FGSG_09052, score=4, range=510-529, strand=1

target  5' UGUAG-CUCUUCAACAACUUG 3'
            :.:.  ::::::::::::::
query   3' CCGUUAAAGAAGUUGUUGAAC 5'

query=JS16_t1331882, target=FGSG_04935, score=4, range=633-653, strand=-1

target  5' GGCAAGUUCUUGAACGACUUG 3'
           ::::: ::::: :::.:::::
query   3' CCGUUAAAGAAGUUGUUGAAC 5'

No results for JS16_t2228365
No results for JS16_t0228401
No results for JS16_t2228402
No results for JS16_t3124647
query=JS16_t2228451, target=FGSG_09017, score=3, range=2894-2911, strand=-1

target  5' CACGA-GAAGGAAUUCUUG 3'
           :: :: ::::::::::::
query   3' GUACUGCUUCCUUAAGAAA 5'

query=JS16_t2228451, target=FGSG_00672, score=3.5, range=3951-3969, strand=1
query   3' CAGU-GCUGUUACCGAGAG 5'

query=JS16_t0157698, target=FGSG_04294, score=4, range=478-495, strand=-1

target  5' AGCACGACCAUGGCUCUC 3'
             :::::: :::::::::
query   3' CAGUGCUGUUACCGAGAG 5'

query=JS16_t0157698, target=FGSG_02905, score=4, range=294-312, strand=-1

target  5' AAUGCUGACAAUGGCUCUC 3'
             ..: :::::::::::::
query   3' CAGUG-CUGUUACCGAGAG 5'

query=JS16_t0157698, target=FGSG_03094, score=4, range=69-86, strand=-1

target  5' GUCUCGACAAUGACUCUG 3'
           ::: :::::::: ::::
query   3' CAGUGCUGUUACCGAGAG 5'

query=JS16_t0157698, target=FGSG_01632, score=4, range=1792-1808, strand=-1

target  5' GUCCCGAUAAUGGC-CUC 3'
           ::: :::.:::::: :::
query   3' CAGUGCUGUUACCGAGAG 5'

query=JS16_t0157698, target=FGSG_08448, score=4, range=429-445, strand=-1

target  5' GUC-CGACAGUCGCUCUC 3'
           ::: :::::.: ::::::
query   3' CAGUGCUGUUACCGAGAG 5'

query=JS16_t0157698, target=FGSG_02554, score=4, range=1474-1490, strand=-1

target  5' UCCACGACAAUGG-UCUC 3'
             ::::::::::: ::::
query   3' CAGUGCUGUUACCGAGAG 5'

query=JS16_t0157698, target=FGSG_08672, score=4, range=313-329, strand=-1

target  5' GCUG-GACGAUGGCUCUC 3'
           : ..  :::.:::::::::
query   3' CAGUGCUGUUACCGAGAG 5'

query=JS16_t0157698, target=FGSG_09006, score=4, range=855-873, strand=-1

target  5' GCCACGGACAAUGGCUCCC 3'
           : ::: ::::::::::: :
query   3' CAGUG-CUGUUACCGAGAG 5'

No results for JS16_t3186970
query=JS16_t2290737, target=FGSG_13572, score=2.5, range=298-316, strand=1

target  5' CUGA-UAACUGCGACAUUUU 3'
           :::: .::::::.:::::::
query   3' GACUCGUUGACGUUGUAAAA 5'

query=JS16_t2290737, target=FGSG_06694, score=3, range=2767-2787, strand=1

target  5' CUCGAGCAACUGAAACAUUUU 3'
           :: ::::::::: ::::::::
query   3' GA-CUCGUUGACGUUGUAAAA 5'

query=JS16_t2290737, target=FGSG_05644, score=3, range=904-923, strand=-1

target  5' CUAAGCAAUUGCAACAUUUC 3'
           :: :::::.::::::::::
query   3' GACUCGUUGACGUUGUAAAA 5'

query=JS16_t2290737, target=FGSG_08114, score=3.5, range=71-89, strand=1

target  5' UUGA-CAACUGCGACGUUUU 3'
           .:::  :::::::.::.::::
query   3' GACUCGUUGACGUUGUAAAA 5'

query=JS16_t2290737, target=FGSG_07816, score=3.5, range=1714-1732, strand=1

target  5' CUUA-UAGCUGCAACAUUUU 3'
           :: : .:.::::::::::::
query   3' GACUCGUUGACGUUGUAAAA 5'

query=JS16_t2290737, target=FGSG_07816, score=3.5, range=1615-1633, strand=1

target  5' CUUA-UAGCUGCAACAUUUU 3'
           :: : .:.::::::::::::
query   3' GACUCGUUGACGUUGUAAAA 5'

query=JS16_t2290737, target=FGSG_07693, score=3.5, range=307-327, strand=-1

target  5' CUGAGCAGCUGCAAACAUUUC 3'
           :::::::.:::: :::::::
query   3' GACUCGUUGACG-UUGUAAAA 5'
```

```
target  5’ UGCGGAGAAGGAAUUCUUU 3’
           .. :. :::::::::::::
query   3’ GUACUGCUUCCUUAAGAAA 5’

query=JS16_t2228451, target=FGSG_02800, score=3.5, range=883-901, strand=-1

target  5’ CUUCGCGAAGGAAUUCUUC 3’
           : : .:::::::::::::
query   3’ GUACUGCUUCCUUAAGAAA 5’

query=JS16_t2228451, target=FGSG_04981, score=4, range=999-1017, strand=1

target  5’ GAUGACGAGUGAAUUCUUU 3’
            :::::::. :::::::::
query   3’ GUACUGCUUCCUUAAGAAA 5’

query=JS16_t2228451, target=FGSG_05524, score=4, range=376-394, strand=1

target  5’ CUUGACGGAGGAAUUGUUU 3’
           : :::::.::::::: :::
query   3’ GUACUGCUUCCUUAAGAAA 5’

query=JS16_t2228451, target=FGSG_02663, score=4, range=3742-3759, strand=1

target  5’ CAUG-CGGCGGAAUUCUUU 3’
           :::: ::. ::::::::::
query   3’ GUACUGCUUCCUUAAGAAA 5’

query=JS16_t2228451, target=FGSG_03920, score=4, range=393-411, strand=-1

target  5’ CAUGACGAGAGAGUUCUUU 3’
           ::::::::. ::.::::::
query   3’ GUACUGCUUCCUUAAGAAA 5’

query=JS16_t2228451, target=FGSG_04408, score=4, range=817-835, strand=-1

target  5’ CGUGCUGGAGGAGUUCUUU 3’
           :.:: .:.::::.::::::
query   3’ GUACUGCUUCCUUAAGAAA 5’

query=JS16_t2228451, target=FGSG_12943, score=4, range=1083-1102, strand=-1

target  5’ CAUGACCAAGGAAUGUCUUG 3’
           :::::: ::::::: ::::
query   3’ GUACUGCUUCCUUA-AGAAA 5’

query=JS16_t2228451, target=FGSG_02944, score=4, range=742-760, strand=-1

target  5’ CCUCACGAAGGAAGUCUUU 3’
           : : ::::::::: :::::
query   3’ GUACUGCUUCCUUAAGAAA 5’

No results for JS16_t0194441
No results for JS16_t1332020
query=JS16_t3124711, target=FGSG_01908, score=2.5, range=951-973, strand=-1

target  5’ CAGGGCUCAUAGCCAGGUAAAGC 3’
           ::::: :: .:::::::::::::
query   3’ GUCCCCAG-GUCGGUCCAUUUCG 5’

query=JS16_t3124711, target=FGSG_06114, score=4, range=1000-1021, strand=-1

target  5’ CAGGGGCCCAGUCGGGUGAAGC 3’
           :::::: ::::.:.:::.::::
query   3’ GUCCCCAGGUCGGUCCAUUUCG 5’

No results for JS16_t1332022
query=JS16_t3124716, target=FGSG_02315, score=3.5, range=21855-21872, strand=

target  5’ GUA-CGAGGCAACUUACCU 3’
           ::: :::::::: :::::.
query   3’ CAUCGCUCCGUUCAAUGGG 5’

query=JS16_t3124716, target=FGSG_12714, score=3.5, range=720-738, strand=-1

target  5’ GUAGCCAGGCAUGUUACCU 3’
           ::::: ::::: ::::::.
query   3’ CAUCGCUCCGUUCAAUGGG 5’

query=JS16_t3124716, target=FGSG_10669, score=3.5, range=181-198, strand=-1

target  5’ GU-GCGAGGCAAGUUCCCU 3’
           :: :::::::::::: ::.
query   3’ CAUCGCUCCGUUCAAUGGG 5’

query=JS16_t3124716, target=FGSG_00404, score=4, range=1473-1491, strand=1

target  5’ GUGGCGAGGCAUGUUGCCU 3’
           ::.:::::::: :::.::.
query   3’ CAUCGCUCCGUUCAAUGGG 5’

query=JS16_t2290737, target=FGSG_13371, score=4, range=299-318, strand=1

target  5’ UUGAGCGACGGCAACAUUUG 3’
           .:::::.:: :::::::::
query   3’ GACUCGUUGACGUUGUAAAA 5’

query=JS16_t2290737, target=FGSG_07522, score=4, range=1977-1995, strand=-1

target  5’ CUG-GCAACUGUAGCAUUUC 3’
           ::: :::::::.:.:::::
query   3’ GACUCGUUGACGUUGUAAAA 5’

query=JS16_t2290737, target=FGSG_04127, score=4, range=1061-1080, strand=-1

target  5’ CUCAGCAACUGCAACAAUUG 3’
           :: ::::::::::::: ::
query   3’ GACUCGUUGACGUUGUAAAA 5’

No results for JS16_t0171238
query=JS16_t0802220, target=FGSG_05783, score=3.5, range=514-537, strand=-1

target  5’ GUUGUC-CUUGAGGUAGGAACGCCG 3’
           ::.:::  :::::::::::::::
query   3’ CAGCAGCCAACUCCAUCCUUGCGGG 5’

query=JS16_t1394889, target=FGSG_12745, score=4, range=1525-1545, strand=-1

target  5’ CUUGGCGUGCUCAGAAUUGUU 3’
            :::.: ..:::::::::::
query   3’ CAACUG-GUGAGUCUUAACAA 5’

query=JS16_t1394889, target=FGSG_08633, score=4, range=799-818, strand=-1

target  5’ GAUGACAACUUGGAAUUGUU 3’
           : :::: :::..::::::::
query   3’ CAACUGGUGAGUCUUAACAA 5’

query=JS16_t1394889, target=FGSG_08862, score=4, range=129-149, strand=-1

target  5’ GUUGGAACACUUAGGAUUGUU 3’
           ::: :: ::::.::.::::::
query   3’ CAA-CUGGUGAGUCUUAACAA 5’

query=JS16_t1394889, target=FGSG_08874, score=4, range=1828-1847, strand=-1

target  5’ AUCUCCCACUCAGAAUUGUU 3’
            :   :::::::::::::::
query   3’ CAACUGGUGAGUCUUAACAA 5’

query=JS16_t1394889, target=FGSG_03621, score=4, range=159-179, strand=-1

target  5’ CUUGAUCGCUUCAGAAUUGUU 3’
            ::::.:.: ::::::::::
query   3’ CAACUGGUG-AGUCUUAACAA 5’

No results for JS16_t1394945
No results for JS16_t4083834
query=JS16_t2290968, target=FGSG_08179, score=3, range=1045-1062, strand=1

target  5’ UGUCUCAAGUCACAGGUU 3’
           : . :::::::::::::.
query   3’ AAGCAGUUCAGUGUCCAG 5’

query=JS16_t2290968, target=FGSG_13846, score=3, range=105-122, strand=-1

target  5’ UUCCUCAAGUCACACGUC 3’
           ::: :::::::::: :::
query   3’ AAGCAGUUCAGUGUCCAG 5’

query=JS16_t2290968, target=FGSG_02163, score=3.5, range=431-448, strand=1

target  5’ UGUCUCAAGUCACAGGUA 3’
           : . :::::::::::::
query   3’ AAGCAGUUCAGUGUCCAG 5’

query=JS16_t2290968, target=FGSG_02477, score=3.5, range=127-145, strand=1

target  5’ UUCGUUAAGCUCACAGGUG 3’
           :::::.::: ::::::::
query   3’ AAGCAGUUC-AGUGUCCAG 5’

query=JS16_t2290968, target=FGSG_11059, score=4, range=307-324, strand=1

target  5’ UUUGUCAAGUCGCAGGGU 3’
           ::.:::::::::.:::: .
query   3’ AAGCAGUUCAGUGUCCAG 5’

query=JS16_t2290968, target=FGSG_05898, score=4, range=1640-1657, strand=1

target  5’ GUCGUCAAGUCGCUGGUC 3’
            ::::::::::.: ::::
```

```
query=JS16_t3124716, target=FGSG_08771, score=4, range=234-252, strand=1

target  5’ GCAGCUGGGCAAGUUGCCC 3’
           : ::: .::::::::.:::
query   3’ CAUCGCUCCGUUCAAUGGG 5’

query=JS16_t3124716, target=FGSG_10221, score=4, range=2349-2368, strand=-1

target  5’ GAAGCGAGGCAAGUUGACUC 3’
           : ::::::::::::: ::.:
query   3’ CAUCGCUCCGUUCAA-UGGG 5’

query=JS16_t3124716, target=FGSG_13543, score=4, range=1145-1163, strand=-1

target  5’ GCAGCAAGGUGAGUUACCC 3’
           : ::: :::..::::::::
query   3’ CAUCGCUCCGUUCAAUGGG 5’

query=JS16_t3124716, target=FGSG_04309, score=4, range=627-645, strand=-1

target  5’ GAAGCGAGGCAAGAUACCG 3’
           : ::::::::::: ::::
query   3’ CAUCGCUCCGUUCAAUGGG 5’

query=JS16_t3124716, target=FGSG_10156, score=4, range=210-228, strand=-1

target  5’ GUAGAAAGGCAGGUUACCA 3’
           ::::  :::::.::::::
query   3’ CAUCGCUCCGUUCAAUGGG 5’

query=JS16_t4021377, target=FGSG_04879, score=3.5, range=1398-1417, strand=1

target  5’ GCUACUUGCAGUGUACUGGU 3’
           :::: :..::.:::::::::
query   3’ CGAUAAGUGUUACAUGACCA 5’

query=JS16_t4021377, target=FGSG_02130, score=4, range=3198-3217, strand=-1

target  5’ GUUGCUCGCAAUGUACUGGA 3’
           :.:. ::.:::::::::::
query   3’ CGAUAAGUGUUACAUGACCA 5’

query=JS16_t4021377, target=FGSG_11941, score=4, range=1421-1441, strand=-1

target  5’ GCAAUUCACGAUGUACGUGGU 3’
           :: ::::::.:::::: ::::
query   3’ CGAUAAGUGUUACAUG-ACCA 5’

query=JS16_t4021377, target=FGSG_00261, score=4, range=2691-2709, strand=-1

target  5’ GCUCUCCACAAUG-ACUGGU 3’
           ::: : ::::::: ::::::
query   3’ CGAUAAGUGUUACAUGACCA 5’

No results for JS16_t1332145
query=JS16_t1332156, target=FGSG_07454, score=2, range=1388-1405, strand=-1

target  5’ UUCCGCCAUGGCCAUAAC 3’
           :: ::::::.::::::::
query   3’ AACGCGGUAUCGGUAUUG 5’

query=JS16_t1332156, target=FGSG_13130, score=3, range=710-727, strand=-1

target  5’ UUGCGCCAUAGCUAUGAG 3’
           ::::::::::::.::.:
query   3’ AACGCGGUAUCGGUAUUG 5’

query=JS16_t1332156, target=FGSG_07848, score=3, range=1039-1056, strand=-1

target  5’ UUGCAUCAUAGCCAUGAC 3’
           :::: .:::::::::.::
query   3’ AACGCGGUAUCGGUAUUG 5’

query=JS16_t1332156, target=FGSG_09781, score=3, range=142-160, strand=-1

target  5’ AUGACGCCAUAGCCGUAAC 3’
            :: ::::::::::.::::
query   3’ AAC-GCGGUAUCGGUAUUG 5’

query=JS16_t1332156, target=FGSG_10869, score=3, range=1702-1718, strand=-1

target  5’ UUGC-CCAUAGUUAUAAC 3’
           :::: ::::::..:::::
query   3’ AACGCGGUAUCGGUAUUG 5’

query=JS16_t1332156, target=FGSG_09386, score=3, range=172-188, strand=-1

target  5’ UUGC-UCAUAGCCGUAAC 3’
           :::: .:::::::.::::
query   3’ AACGCGGUAUCGGUAUUG 5’

query=JS16_t1332156, target=FGSG_05430, score=3, range=1453-1469, strand=-1

query   3’ AAGCAGUUCAGUGUCCAG 5’

query=JS16_t2290968, target=FGSG_10285, score=4, range=131-148, strand=1

target  5’ UGCGUCAAGUCGCCGGUC 3’
           : :::::::::.: ::::
query   3’ AAGCAGUUCAGUGUCCAG 5’

query=JS16_t2290968, target=FGSG_03638, score=4, range=688-705, strand=1

target  5’ UUGGUCGAGUCAAAGGUC 3’
           :: :::.::::: :::::
query   3’ AAGCAGUUCAGUGUCCAG 5’

query=JS16_t2290968, target=FGSG_11112, score=4, range=646-663, strand=1

target  5’ UUCCCCAAGUCAUGGGUC 3’
           :::  :::::::..::::
query   3’ AAGCAGUUCAGUGUCCAG 5’

query=JS16_t2290968, target=FGSG_12682, score=4, range=16-33, strand=1

target  5’ AUAGUCAAGUUACAGGUG 3’
            : :::::::.::::::
query   3’ AAGCAGUUCAGUGUCCAG 5’

query=JS16_t2290968, target=FGSG_01442, score=4, range=969-987, strand=1

target  5’ GCCGUUCAAGUCACGGGUC 3’
             :: ::::::::::.::::
query   3’ AAGC-AGUUCAGUGUCCAG 5’

query=JS16_t2290968, target=FGSG_13878, score=4, range=14708-14724, strand=1

target  5’ UUCGUCAAGUCA-AGGAC 3’
           ::::::::::::: ::: :
query   3’ AAGCAGUUCAGUGUCCAG 5’

query=JS16_t2290968, target=FGSG_05427, score=4, range=1097-1114, strand=1

target  5’ UUCGCCAAGUCACUGGUG 3’
           :::: :::::::: :::
query   3’ AAGCAGUUCAGUGUCCAG 5’

query=JS16_t2290968, target=FGSG_02054, score=4, range=1191-1209, strand=1

target  5’ CUCGCUCAAGUCACACGUC 3’
            ::: :::::::::: :::
query   3’ AAGC-AGUUCAGUGUCCAG 5’

query=JS16_t2290968, target=FGSG_11731, score=4, range=271-288, strand=1

target  5’ AUCAACGAGUCACAGGUC 3’
            ::  :.:::::::::::
query   3’ AAGCAGUUCAGUGUCCAG 5’

query=JS16_t2290968, target=FGSG_08722, score=4, range=707-723, strand=1

target  5’ AGCGUCAAGUCA-AGGUC 3’
             :::::::::: :::::
query   3’ AAGCAGUUCAGUGUCCAG 5’

query=JS16_t2290968, target=FGSG_00473, score=4, range=183-199, strand=1

target  5’ UGGGUCAAGUCACA-GUC 3’
           :  ::::::::::: :::
query   3’ AAGCAGUUCAGUGUCCAG 5’

query=JS16_t2290968, target=FGSG_05232, score=4, range=1277-1293, strand=1

target  5’ UCCCUCAAGUCACA-GUC 3’
           : : :::::::::: :::
query   3’ AAGCAGUUCAGUGUCCAG 5’

query=JS16_t2290968, target=FGSG_13915, score=4, range=104-120, strand=1

target  5’ CUGGUCAAGUCA-AGGUC 3’
            : ::::::::: :::::
query   3’ AAGCAGUUCAGUGUCCAG 5’

query=JS16_t2290968, target=FGSG_07505, score=4, range=1402-1420, strand=1

target  5’ AUCUUCAACGUCACAGGUC 3’
            :: :::: ::::::::::
query   3’ AAGCAGUU-CAGUGUCCAG 5’

query=JS16_t2290968, target=FGSG_05651, score=4, range=409-426, strand=-1

target  5’ UUCCUCAGGUGACAGGUC 3’
           ::: :::.:: :::::::
query   3’ AAGCAGUUCAGUGUCCAG 5’
```

```
target  5’ UUAC-CCAUAGCCAUGAC 3’
           :: : ::::::::::.::
query   3’ AACGCGGUAUCGGUAUUG 5’

query=JS16_t1332156, target=FGSG_02690, score=3.5, range=960-976, strand=-1

target  5’ UUG-GCCAUGGCCAUGAU 3’
           ::: :::::.:::::.:.
query   3’ AACGCGGUAUCGGUAUUG 5’

query=JS16_t1332156, target=FGSG_04182, score=3.5, range=988-1004, strand=-1

target  5’ AUGU-CCAUAGCCAUGAC 3’
            ::. ::::::::::.::
query   3’ AACGCGGUAUCGGUAUUG 5’

query=JS16_t1332156, target=FGSG_00994, score=3.5, range=209-225, strand=-1

target  5’ UUG-GCCAAAGCCAUAAU 3’
           ::: :::: ::::::::.
query   3’ AACGCGGUAUCGGUAUUG 5’

query=JS16_t1332156, target=FGSG_10096, score=4, range=822-839, strand=1

target  5’ UGGCGGCAUAGCCAUGAC 3’
           : ::: :::::::::.::
query   3’ AACGCGGUAUCGGUAUUG 5’

query=JS16_t1332156, target=FGSG_09236, score=4, range=723-740, strand=1

target  5’ UUCUGCCAUUGCCAUAAU 3’
           :: .::::: :::::::.
query   3’ AACGCGGUAUCGGUAUUG 5’

query=JS16_t1332156, target=FGSG_11848, score=4, range=507-525, strand=1

target  5’ UUCUAGCCAUAGCCGUAAU 3’
           :: . :::::::::.:::.
query   3’ AACG-CGGUAUCGGUAUUG 5’

query=JS16_t1332156, target=FGSG_08719, score=4, range=5389-5406, strand=1

target  5’ UUGGUUCAUAGCCAUAAG 3’
           :::  .:::::::::::
query   3’ AACGCGGUAUCGGUAUUG 5’

query=JS16_t1332156, target=FGSG_13298, score=4, range=484-501, strand=1

target  5’ GUCCUCCAUAGCUAUAAC 3’
            : : :::::::.:::::
query   3’ AACGCGGUAUCGGUAUUG 5’

query=JS16_t1332156, target=FGSG_06260, score=4, range=256-273, strand=-1

target  5’ UUGCGCCAUACCCAUGGC 3’
           :::::::::: ::::..:
query   3’ AACGCGGUAUCGGUAUUG 5’

query=JS16_t1332156, target=FGSG_11141, score=4, range=733-750, strand=-1

target  5’ CUGCGCCAUAGGCGUAAC 3’
            :::::::::: :.::::
query   3’ AACGCGGUAUCGGUAUUG 5’

query=JS16_t1332156, target=FGSG_10871, score=4, range=602-620, strand=-1

target  5’ UUGUCGUUAUAGCCAUAAA 3’
           ::: ::..::::::::::
query   3’ AAC-GCGGUAUCGGUAUUG 5’

query=JS16_t1332156, target=FGSG_03693, score=4, range=76-93, strand=-1

target  5’ UUGCUCCAUAUCCAUAGC 3’
           :::: ::::: :::::.:
query   3’ AACGCGGUAUCGGUAUUG 5’

query=JS16_t1332156, target=FGSG_09042, score=4, range=622-640, strand=-1

target  5’ UUGCGAUCCUAGCCAUAAC 3’
           ::::: .: ::::::::::
query   3’ AACGC-GGUAUCGGUAUUG 5’

query=JS16_t1332156, target=FGSG_08720, score=4, range=14-31, strand=-1

target  5’ GUGCCCCAUGGCCAUAAA 3’
            ::: ::::.:::::::
query   3’ AACGCGGUAUCGGUAUUG 5’

query=JS16_t1332156, target=FGSG_08873, score=4, range=991-1008, strand=-1

target  5’ GUGCUUCAUAGCCAUAAG 3’

query=JS16_t2290968, target=FGSG_06346, score=4, range=513-530, strand=-1

target  5’ UUCAUCAAGUUCCAGGUC 3’
           ::: ::::::. ::::::
query   3’ AAGCAGUUCAGUGUCCAG 5’

query=JS16_t2290968, target=FGSG_07232, score=4, range=29-46, strand=-1

target  5’ GACGUCCAGUCACAGGUC 3’
             :::: :::::::::::
query   3’ AAGCAGUUCAGUGUCCAG 5’

query=JS16_t2290968, target=FGSG_01406, score=4, range=198-215, strand=-1

target  5’ UGAGUCAAGUCACGGGUA 3’
           :  ::::::::::.:::
query   3’ AAGCAGUUCAGUGUCCAG 5’

query=JS16_t2290968, target=FGSG_12012, score=4, range=768-786, strand=-1

target  5’ UUCGAUCAAGUCAAAGGUA 3’
           :::: :::::::: ::::
query   3’ AAGC-AGUUCAGUGUCCAG 5’

query=JS16_t2290968, target=FGSG_03546, score=4, range=689-707, strand=-1

target  5’ UCCAUCAUAGUCACAGGUC 3’
           : : ::: :::::::::::
query   3’ AAGCAGU-UCAGUGUCCAG 5’

query=JS16_t2290968, target=FGSG_05365, score=4, range=338-356, strand=-1

target  5’ UGCUUCAAGUCACAGAGUC 3’
           : : ::::::::::: :::
query   3’ AAGCAGUUCAGUGUC-CAG 5’

query=JS16_t2290968, target=FGSG_06687, score=4, range=86-102, strand=-1

target  5’ AUAGUC-AGUCACAGGUC 3’
            : ::: :::::::::::
query   3’ AAGCAGUUCAGUGUCCAG 5’

No results for JS16_t4083896
No results for JS16_t2291062
query=JS16_t0366955, target=FGSG_13231, score=3.5, range=1723-1741, strand=-1

target  5’ CGUGUGCUUGCUUGGGGGU 3’
           :::  .:::::.:::::::
query   3’ GCAAUUGAACGGACCCCCA 5’

query=JS16_t0366955, target=FGSG_08610, score=4, range=76-95, strand=-1

target  5’ UGUUGAUCUUGGCUGGGGGU 3’
           .:::.: :::: ::::::::
query   3’ GCAAUU-GAACGGACCCCCA 5’

query=JS16_t0366955, target=FGSG_06069, score=4, range=485-502, strand=-1

target  5’ AUUUAA-UUGCCUGGGGGU 3’
             :::: ::::::::::::
query   3’ GCAAUUGAACGGACCCCCA 5’

query=JS16_t1395106, target=FGSG_02498, score=3, range=641-658, strand=1

target  5’ CUCCUUUUGGAC-CUGGUA 3’
           :::::::.:::: ::::::
query   3’ GAGGAAAGCCUGUGACCAU 5’

query=JS16_t1395106, target=FGSG_05698, score=3.5, range=1028-1047, strand=-1

target  5’ CUCCGUCUCGGGCACUGGUG 3’
           :::: : ::::.:::::::.
query   3’ GAGG-AAAGCCUGUGACCAU 5’

query=JS16_t1395106, target=FGSG_02292, score=4, range=21-40, strand=1

target  5’ AUCCUCUUCGGACAAUGGUA 3’
            :::: :::::::: :::::
query   3’ GAGGA-AAGCCUGUGACCAU 5’

query=JS16_t1395106, target=FGSG_13692, score=4, range=975-994, strand=-1

target  5’ CUCCUUUCGGGACAUUGGUC 3’
           :::::::: :::::.::::
query   3’ GAGGAAAG-CCUGUGACCAU 5’

query=JS16_t1395106, target=FGSG_13660, score=4, range=1495-1513, strand=-1

target  5’ UUCUUUUCGGAAACUGGUC 3’
           .::.:::::::: ::::::
query   3’ GAGGAAAGCCUGUGACCAU 5’
```

```
            ::: .:::::::::::
query   3’ AACGCGGUAUCGGUAUUG 5’

query=JS16_t1332156, target=FGSG_01589, score=4, range=5311-5327, strand=-1

target  5’ UUG-GCUAUAGCCAUAGG 3’
           ::: ::.:::::::::.
query   3’ AACGCGGUAUCGGUAUUG 5’

query=JS16_t1332156, target=FGSG_04348, score=4, range=462-478, strand=-1

target  5’ UUGC-CCGUAGCCAUGAG 3’
           :::: ::.:::::::.:
query   3’ AACGCGGUAUCGGUAUUG 5’

query=JS16_t1332156, target=FGSG_13556, score=4, range=1594-1611, strand=-1

target  5’ AUUCGCCAUAGCCAUAUC 3’
            : ::::::::::::: :
query   3’ AACGCGGUAUCGGUAUUG 5’

query=JS16_t1332156, target=FGSG_08662, score=4, range=1255-1272, strand=-1

target  5’ CUGGGCCAUAGCCAUACC 3’
            :: :::::::::::: :
query   3’ AACGCGGUAUCGGUAUUG 5’

query=JS16_t1332156, target=FGSG_03666, score=4, range=1038-1054, strand=-1

target  5’ UUGCUCCAUAGCCA-AGC 3’
           :::: ::::::::: :.:
query   3’ AACGCGGUAUCGGUAUUG 5’

query=JS16_t1332156, target=FGSG_03340, score=4, range=6349-6366, strand=-1

target  5’ CUCAACCAUAGCCAUAAC 3’
            :   :::::::::::::
query   3’ AACGCGGUAUCGGUAUUG 5’

query=JS16_t1332156, target=FGSG_01217, score=4, range=1396-1414, strand=-1

target  5’ UUCGGCCGAUAGCCAUAAC 3’
           ::  ::: :::::::::::
query   3’ AACGCGG-UAUCGGUAUUG 5’

query=JS16_t1332170, target=FGSG_06560, score=4, range=970-992, strand=1

target  5’ UCGAUCCCGUUUAUCAAGAACAG 3’
           .::::: ::.:.::::::::::
query   3’ GGCUAGUGCGAGUAGUUCUUGUA 5’

query=JS16_t1332170, target=FGSG_06605, score=4, range=1631-1652, strand=1

target  5’ GCGAUGA-GCUCAUCAAGAACGU 3’
            :::: : :::::::::::::.:
query   3’ GGCUAGUGCGAGUAGUUCUUGUA 5’

query=JS16_t1332170, target=FGSG_07274, score=4, range=365-387, strand=-1

target  5’ GCGAUCCAGCUCAUCAGGAACAU 3’
            :::::  ::::::::.::::::
query   3’ GGCUAGUGCGAGUAGUUCUUGUA 5’

query=JS16_t0576529, target=FGSG_12506, score=3, range=453-470, strand=1

target  5’ CUUGUUAGGAGUAUGCAU 3’
             : ::::::::::::::
query   3’ UCAGAAUCCUCAUACGUA 5’

query=JS16_t0576529, target=FGSG_02339, score=3, range=587-604, strand=-1

target  5’ AGUCUUGGGGGUAUGCAA 3’
           ::::::.::.:::::::
query   3’ UCAGAAUCCUCAUACGUA 5’

query=JS16_t0576529, target=FGSG_03705, score=3.5, range=648-665, strand=1

target  5’ AGCUGUGGGAGUAUGCAU 3’
           :: .  :.::::::::::
query   3’ UCAGAAUCCUCAUACGUA 5’

query=JS16_t0576529, target=FGSG_09793, score=3.5, range=2610-2626, strand=1

target  5’ AGUUUU-GGAGUAUGCAA 3’
           :::.:: :::::::::
query   3’ UCAGAAUCCUCAUACGUA 5’

query=JS16_t0576529, target=FGSG_12415, score=3.5, range=934-950, strand=1

target  5’ GGUC-UAGGAGUAUGUAC 3’
           .::: ::::::::::.:
query   3’ UCAGAAUCCUCAUACGUA 5’

query=JS16_t1395106, target=FGSG_08650, score=4, range=5900-5918, strand=-1

target  5’ AUCCUCUUGGACACUGGUU 3’
            :::: :.::::::::::
query   3’ GAGGAAAGCCUGUGACCAU 5’

query=JS16_t1395106, target=FGSG_00633, score=4, range=6104-6123, strand=-1

target  5’ GUCCUUCUCGGCCACUGGUA 3’
            ::::: :::: ::::::::
query   3’ GAGGAA-AGCCUGUGACCAU 5’

query=JS16_t1395106, target=FGSG_01959, score=4, range=1093-1110, strand=-1

target  5’ CUCCUCUCGGACAC-GGUC 3’
           ::::: :::::::: :::
query   3’ GAGGAAAGCCUGUGACCAU 5’

query=JS16_t0396254, target=FGSG_04033, score=4, range=2249-2267, strand=1

target  5’ GUUUGAACUUCUUGGCUGG 3’
           ::::::: :.:::.:::::
query   3’ CAAACUUUAGGAAUCGACC 5’

query=JS16_t0396254, target=FGSG_06052, score=4, range=161-180, strand=-1

target  5’ GUAUUGAAAUUCUUGGUUGG 3’
           :: :::::::.:::.:.:::
query   3’ CA-AACUUUAGGAAUCGACC 5’

query=JS16_t0396254, target=FGSG_06953, score=4, range=4132-4151, strand=-1

target  5’ GGUGGAGAAUCCUUGGCUGG 3’
           : : :: :::::::.:::::
query   3’ CAAACU-UUAGGAAUCGACC 5’

query=JS16_t2291161, target=FGSG_01412, score=2, range=805-821, strand=1

target  5’ AGC-CGAAUCUUCAGGGG 3’
           :::  :::::::::::::
query   3’ UCGCUCUUAGAAGUCCCC 5’

query=JS16_t2291161, target=FGSG_06193, score=2, range=1397-1415, strand=-1

target  5’ GGCGUGGAAUCUUCAGGGG 3’
           .::: .:::::::::::::
query   3’ UCGC-UCUUAGAAGUCCCC 5’

query=JS16_t2291161, target=FGSG_10313, score=3, range=1211-1227, strand=1

target  5’ AGC-AGGAUCUUCAGGGA 3’
           ::: ::.::::::::::
query   3’ UCGCUCUUAGAAGUCCCC 5’

query=JS16_t2291161, target=FGSG_12742, score=3, range=943-959, strand=1

target  5’ AG-GAGAAUCUUCUGGGG 3’
           :: :::::::::: ::::
query   3’ UCGCUCUUAGAAGUCCCC 5’

query=JS16_t2291161, target=FGSG_13600, score=3, range=70-88, strand=-1

target  5’ AGUCUAGAAUCUUCAGGGU 3’
           :: : :::::::::::::
query   3’ UC-GCUCUUAGAAGUCCCC 5’

query=JS16_t2291161, target=FGSG_01562, score=3.5, range=1258-1274, strand=1

target  5’ GGCGA-AAUUUUCAGGGG 3’
           .:::: :::.::::::::
query   3’ UCGCUCUUAGAAGUCCCC 5’

query=JS16_t2291161, target=FGSG_03603, score=3.5, range=1556-1573, strand=1

target  5’ AUCGGGAAUCUUCAGUGG 3’
           : ::.:::::::::: ::
query   3’ UCGCUCUUAGAAGUCCCC 5’

query=JS16_t2291161, target=FGSG_01051, score=3.5, range=1075-1092, strand=-1

target  5’ GGCGAGAAUCUUGAGGGU 3’
           .::::::::::: ::::
query   3’ UCGCUCUUAGAAGUCCCC 5’

query=JS16_t2291161, target=FGSG_08906, score=3.5, range=1172-1188, strand=-1

target  5’ GGCGAGAA-CUUCGGGGG 3’
           .::::::: ::::.::::
query   3’ UCGCUCUUAGAAGUCCCC 5’

query=JS16_t2291161, target=FGSG_10616, score=3.5, range=2816-2834, strand=-1
```

```
query=JS16_t0576529, target=FGSG_06639, score=4, range=668-685, strand=1

target  5' AGACUUACGAGUAUGUAU 3'
           :: :::: :::::::.::
query   3' UCAGAAUCCUCAUACGUA 5'

query=JS16_t0576529, target=FGSG_01918, score=4, range=177-193, strand=1

target  5' AGUCUU-GGAGCAUGCAU 3'
           :::::: :::: ::::::
query   3' UCAGAAUCCUCAUACGUA 5'

query=JS16_t0576529, target=FGSG_02730, score=4, range=1630-1647, strand=-1

target  5' CGUCAUAGGAGUAUGAAU 3'
            ::: :::::::::: ::
query   3' UCAGAAUCCUCAUACGUA 5'

query=JS16_t0576529, target=FGSG_10939, score=4, range=494-510, strand=-1

target  5' AGUC-UGGGCGUAUGCAU 3'
           :::: :.:: ::::::::
query   3' UCAGAAUCCUCAUACGUA 5'

query=JS16_t0576529, target=FGSG_04409, score=4, range=1-17, strand=-1

target  5' AGUAUUGGGAGU-UGCAU 3'
           ::: ::.::::: :::::
query   3' UCAGAAUCCUCAUACGUA 5'

No results for JS16_t3124908
No results for JS16_t3124928
No results for JS16_t0685203
query=JS16_t2228714, target=FGSG_13008, score=2.5, range=2496-2513, strand=1

target  5' UUUCAGGGGACCAGCCAG 3'
           .:::::::: ::::::::
query   3' GAAGUCCCCAGGUCGGUC 5'

query=JS16_t2228714, target=FGSG_10011, score=3, range=198-216, strand=1

target  5' CUUCAAGGGGUUCAGCCAA 3'
           :::: ::::::.::::::
query   3' GAAG-UCCCCAGGUCGGUC 5'

query=JS16_t2228714, target=FGSG_13586, score=3, range=352-369, strand=1

target  5' CUCAAGGGGUCCGGCCAG 3'
           ::  ::::::::.:::::
query   3' GAAGUCCCCAGGUCGGUC 5'

query=JS16_t2228714, target=FGSG_03981, score=3, range=271-288, strand=-1

target  5' CUUCAGGGUUUCAGCCAG 3'
           :::::::: :.:::::::
query   3' GAAGUCCCCAGGUCGGUC 5'

query=JS16_t2228714, target=FGSG_08019, score=3, range=1187-1204, strand=-1

target  5' CCUCAGGGGUCCAGCCGU 3'
           : ::::::::::::::.
query   3' GAAGUCCCCAGGUCGGUC 5'

query=JS16_t2228714, target=FGSG_01446, score=3, range=992-1009, strand=-1

target  5' CGUCAGGGGUCCAGCGAG 3'
           : ::::::::::::: ::
query   3' GAAGUCCCCAGGUCGGUC 5'

query=JS16_t2228714, target=FGSG_10205, score=3, range=224-241, strand=-1

target  5' CUUGAGGGGUCCAGACAG 3'
           ::: :::::::::: :::
query   3' GAAGUCCCCAGGUCGGUC 5'

query=JS16_t2228714, target=FGSG_02073, score=3.5, range=1196-1213, strand=-1

target  5' GGUUAGGGGUCCAGUCAG 3'
             :.::::::::::.:::
query   3' GAAGUCCCCAGGUCGGUC 5'

query=JS16_t2228714, target=FGSG_03341, score=3.5, range=1150-1168, strand=-1

target  5' GUUCGGGGGUCCAGCCCAG 3'
            :::.::::::::: ::::
query   3' GAAGUCCCCAGGUC-GGUC 5'

query=JS16_t2228714, target=FGSG_02481, score=3.5, range=687-705, strand=-1

target  5' CUCUCGGGUGUCCAGCCAG 3'
           :: ::.:: ::::::::::

target  5' AGUGUAGAAUCUUCAGAGG 3'
           ::.: ::::::::::: ::
query   3' UCGC-UCUUAGAAGUCCCC 5'

query=JS16_t2291161, target=FGSG_06004, score=3.5, range=1142-1158, strand=-1

target  5' GGGGAGAAUC-UCAGGGG 3'
           .: ::::::: :::::::
query   3' UCGCUCUUAGAAGUCCCC 5'

query=JS16_t2291161, target=FGSG_01506, score=3.5, range=557-573, strand=-1

target  5' GGCAAGAAUC-UCAGGGG 3'
           .:: :::::: :::::::
query   3' UCGCUCUUAGAAGUCCCC 5'

query=JS16_t2291161, target=FGSG_02587, score=4, range=933-950, strand=1

target  5' AGCGAGAAUCUUUGGGCG 3'
           ::::::::::::..:: :
query   3' UCGCUCUUAGAAGUCCCC 5'

query=JS16_t2291161, target=FGSG_01733, score=4, range=1113-1130, strand=1

target  5' AGCAAGAGUCUUUGGGGG 3'
           ::: :::.::::..::::
query   3' UCGCUCUUAGAAGUCCCC 5'

query=JS16_t2291161, target=FGSG_09394, score=4, range=101-119, strand=1

target  5' AGCGAGAGUCGUUCAGGGA 3'
           :::::::.:: :::::::
query   3' UCGCUCUUAG-AAGUCCCC 5'

query=JS16_t2291161, target=FGSG_02393, score=4, range=1084-1101, strand=1

target  5' GAUGAGAAUCUUCAGGAG 3'
           . .:::::::::::: :
query   3' UCGCUCUUAGAAGUCCCC 5'

query=JS16_t2291161, target=FGSG_08521, score=4, range=1202-1218, strand=1

target  5' AGC-AGGGUCUUCAGGGA 3'
           ::: ::..:::::::::
query   3' UCGCUCUUAGAAGUCCCC 5'

query=JS16_t2291161, target=FGSG_01352, score=4, range=1012-1029, strand=1

target  5' AUCGAGAAUCUUCUGGGA 3'
           : ::::::::::: :::
query   3' UCGCUCUUAGAAGUCCCC 5'

query=JS16_t2291161, target=FGSG_06700, score=4, range=203-220, strand=1

target  5' AGCUAGAAUCUUCAGCGA 3'
           ::: :::::::::: :
query   3' UCGCUCUUAGAAGUCCCC 5'

query=JS16_t2291161, target=FGSG_06845, score=4, range=286-302, strand=1

target  5' AGCG-GAAUCUUCGGCGG 3'
           :::: ::::::::.: ::
query   3' UCGCUCUUAGAAGUCCCC 5'

query=JS16_t2291161, target=FGSG_12849, score=4, range=926-942, strand=1

target  5' AGCCAG-GUCUUCAGGGG 3'
           ::: :: .::::::::::
query   3' UCGCUCUUAGAAGUCCCC 5'

query=JS16_t2291161, target=FGSG_08939, score=4, range=406-423, strand=-1

target  5' AGCGAGGAUCUUAGGGGG 3'
           ::::::.::::: .::::
query   3' UCGCUCUUAGAAGUCCCC 5'

query=JS16_t2291161, target=FGSG_02809, score=4, range=8-25, strand=-1

target  5' CCUGGGAAUCUUUAGGGG 3'
             .:.::::::::.:::::
query   3' UCGCUCUUAGAAGUCCCC 5'

query=JS16_t2291161, target=FGSG_03466, score=4, range=710-727, strand=-1

target  5' AGCGAGAAUCGUCGGGGA 3'
           :::::::::: ::.:::
query   3' UCGCUCUUAGAAGUCCCC 5'

query=JS16_t2291161, target=FGSG_02990, score=4, range=400-418, strand=-1

target  5' AGCGGAGAGUUUUCAGGGA 3'
           ::: ::::.:.:::::::
```

```
query   3' GA-AGUCCCCAGGUCGGUC 5'

query=JS16_t2228714, target=FGSG_06895, score=4, range=1423-1440, strand=1

target  5' CUUCAGUGGUCUGGCCAG 3'
           :::::: ::::..:::::
query   3' GAAGUCCCCAGGUCGGUC 5'

query=JS16_t2228714, target=FGSG_08282, score=4, range=1333-1350, strand=1

target  5' CUCUGGAGGUCCAGCCAG 3'
           :: ..: :::::::::::
query   3' GAAGUCCCCAGGUCGGUC 5'

query=JS16_t2228714, target=FGSG_03967, score=4, range=1345-1361, strand=1

target  5' CAACAGGGGUCCAG-CAG 3'
           :  ::::::::::: :::
query   3' GAAGUCCCCAGGUCGGUC 5'

query=JS16_t2228714, target=FGSG_12362, score=4, range=712-729, strand=-1

target  5' CUUUGGGGCUCUAGCCAG 3'
           :::..::: ::.::::::
query   3' GAAGUCCCCAGGUCGGUC 5'

query=JS16_t2228714, target=FGSG_03366, score=4, range=322-339, strand=-1

target  5' CUCCAGGGGUCCAGUCGC 3'
           :: :::::::::::.:.
query   3' GAAGUCCCCAGGUCGGUC 5'

query=JS16_t2228714, target=FGSG_13239, score=4, range=797-814, strand=-1

target  5' CCUCAGGCGUCCAGCCAU 3'
           : ::::: :::::::::
query   3' GAAGUCCCCAGGUCGGUC 5'

query=JS16_t2228714, target=FGSG_06901, score=4, range=556-574, strand=-1

target  5' CAUCAGGGGUCCAUGCCAA 3'
           : ::::::::::: ::::
query   3' GAAGUCCCCAGGU-CGGUC 5'

query=JS16_t2228714, target=FGSG_00771, score=4, range=408-424, strand=-1

target  5' CUUCAGGAGUCCA-CCAG 3'
           ::::::: ::::: ::::
query   3' GAAGUCCCCAGGUCGGUC 5'

query=JS16_t2228714, target=FGSG_00829, score=4, range=1557-1573, strand=-1

target  5' CUUC-GGCGUCUAGCCAG 3'
           :::: :: :::.::::::
query   3' GAAGUCCCCAGGUCGGUC 5'

query=JS16_t2228714, target=FGSG_02326, score=4, range=1017-1033, strand=-1

target  5' CUUC-GGCGUCCGGCCAG 3'
           :::: :: ::::.:::::
query   3' GAAGUCCCCAGGUCGGUC 5'

query=JS16_t2228714, target=FGSG_10779, score=4, range=684-701, strand=-1

target  5' GUGACGGGGUCCAGCCAG 3'
            :   :::::::::::::
query   3' GAAGUCCCCAGGUCGGUC 5'

query=JS16_t2228714, target=FGSG_13235, score=4, range=63-81, strand=-1

target  5' CGUCAAGGGUCCAGUCCAG 3'
           : ::: :::::::: ::::
query   3' GAAGUCCCCAGGUC-GGUC 5'

query=JS16_t2228714, target=FGSG_10596, score=4, range=1450-1468, strand=-1

target  5' CUUCAGGAGGUCCAGCGAG 3'
           ::::::: :::::::: ::
query   3' GAAGUCC-CCAGGUCGGUC 5'

query=JS16_t2228714, target=FGSG_03613, score=4, range=1179-1197, strand=-1

target  5' CUUCAGGCGCUCCAGCCAG 3'
           ::::::: : :::::::::
query   3' GAAGUCC-CCAGGUCGGUC 5'

query=JS16_t2228714, target=FGSG_06143, score=4, range=396-412, strand=-1

target  5' CGCCAGGGGU-CAGCCAG 3'
           :  ::::::: :::::::
query   3' GAAGUCCCCAGGUCGGUC 5'

query   3' UCG-CUCUUAGAAGUCCCC 5'

query=JS16_t2291161, target=FGSG_10532, score=4, range=1529-1546, strand=-1

target  5' GGUGCGAAUCUUCGGGGU 3'
           .:.: ::::::::.:::
query   3' UCGCUCUUAGAAGUCCCC 5'

query=JS16_t2291161, target=FGSG_03613, score=4, range=1189-1206, strand=-1

target  5' AGCGAGAUUCUUCAGGCG 3'
           ::::::: :::::::: :
query   3' UCGCUCUUAGAAGUCCCC 5'

query=JS16_t2291161, target=FGSG_05433, score=4, range=607-624, strand=-1

target  5' AGGGAGAGUCUUCUGGGG 3'
           :: ::::.::::: ::::
query   3' UCGCUCUUAGAAGUCCCC 5'

query=JS16_t2291161, target=FGSG_06208, score=4, range=497-513, strand=-1

target  5' AGCGAGGAUC-UCAGGGC 3'
           ::::::.::: ::::::
query   3' UCGCUCUUAGAAGUCCCC 5'

query=JS16_t2291161, target=FGSG_00074, score=4, range=380-397, strand=-1

target  5' UGCCAGAAUCUUCAGGUG 3'
            :: ::::::::::: :
query   3' UCGCUCUUAGAAGUCCCC 5'

query=JS16_t2291161, target=FGSG_07877, score=4, range=4103-4120, strand=-1

target  5' UGGGUGAGUCUUCAGGGG 3'
            : : ::.::::::::::
query   3' UCGCUCUUAGAAGUCCCC 5'

query=JS16_t2291161, target=FGSG_07016, score=4, range=404-421, strand=-1

target  5' CGGGUGAAUCUUCGGGGG 3'
            : : ::::::::.::::
query   3' UCGCUCUUAGAAGUCCCC 5'

query=JS16_t2291161, target=FGSG_13596, score=4, range=332-348, strand=-1

target  5' AGC-AGGAUCGUCAGGGG 3'
           ::: ::.::: :::::::
query   3' UCGCUCUUAGAAGUCCCC 5'

query=JS16_t2291161, target=FGSG_09806, score=4, range=934-951, strand=-1

target  5' ACGGAGAAUCUUGAGGGG 3'
           :  ::::::::: :::::
query   3' UCGCUCUUAGAAGUCCCC 5'

query=JS16_t2291161, target=FGSG_06925, score=4, range=3314-3330, strand=-1

target  5' GGCAGGAAU-UUCAGGGG 3'
           .:: .:::: ::::::::
query   3' UCGCUCUUAGAAGUCCCC 5'

query=JS16_t2291161, target=FGSG_06970, score=4, range=3344-3360, strand=-1

target  5' AGCGAGAA-CUUCAGGAG 3'
           :::::::: ::::::: :
query   3' UCGCUCUUAGAAGUCCCC 5'

No results for JS16_t0241248
No results for JS16_t1395280
No results for JS16_t4084258
No results for JS16_t0366967
query=JS16_t4084314, target=FGSG_11519, score=3.5, range=1387-1406, strand=-1

target  5' AGUCGCUUAUUCUCACGCAG 3'
           :: :. :::.::::::::::
query   3' UCCGUAAAUGAGAGUGCGUC 5'

query=JS16_t4084314, target=FGSG_05279, score=4, range=174-194, strand=1

target  5' AGGCAUUGACUCUCACGGCAA 3'
           ::::::: :::::::: :::
query   3' UCCGUAAAUGAGAGUG-CGUC 5'

query=JS16_t4084314, target=FGSG_05392, score=4, range=1556-1574, strand=-1

target  5' AGGC-UUGACUCUCGCGCAG 3'
           :::: :: ::::::.:::::
query   3' UCCGUAAAUGAGAGUGCGUC 5'

No results for JS16_t3187675
query=JS16_t4084390, target=FGSG_09499, score=2, range=1601-1617, strand=-1
```

```
No results for JS16_t0283110
query=JS16_t2228736, target=FGSG_08714, score=2, range=546-563, strand=1

target  5’ GGUCA-CUGGUGGUCGCAA 3’
           ::::: :::.:::::::::
query   3’ CCAGUGGACUACCAGCGUU 5’

query=JS16_t2228736, target=FGSG_10498, score=3, range=557-575, strand=1

target  5’ GGUCCCCUGAUGCUCGCAA 3’
           :::: ::::::: ::::::
query   3’ CCAGUGGACUACCAGCGUU 5’

query=JS16_t2228736, target=FGSG_02615, score=3.5, range=506-523, strand=1

target  5’ GGUUACCUGAUGGUC-CAU 3’
           :::.::::::::::: ::
query   3’ CCAGUGGACUACCAGCGUU 5’

query=JS16_t2228736, target=FGSG_00729, score=3.5, range=876-893, strand=1

target  5’ GGCCG-CUGGUGGUCGCAA 3’
           :: :. :::.:::::::::
query   3’ CCAGUGGACUACCAGCGUU 5’

query=JS16_t2228736, target=FGSG_13422, score=3.5, range=1924-1942, strand=-1

target  5’ GGCCGUCUGAUGGUCGUAG 3’
           :: :..::::::::::.:.
query   3’ CCAGUGGACUACCAGCGUU 5’

query=JS16_t2228736, target=FGSG_01290, score=3.5, range=83-101, strand=-1

target  5’ GAACGCCUGAUGGUCGCAU 3’
           :  :.:::::::::::::
query   3’ CCAGUGGACUACCAGCGUU 5’

query=JS16_t2228736, target=FGSG_04451, score=4, range=823-842, strand=1

target  5’ GCUUACUUGAUGGUCGUCAA 3’
           : :.::.::::::::: :::
query   3’ CCAGUGGACUACCAGC-GUU 5’

query=JS16_t2228736, target=FGSG_04643, score=4, range=278-296, strand=-1

target  5’ GGUCUCCUGGUGGUUGCAU 3’
           :::: ::::.::::.:::
query   3’ CCAGUGGACUACCAGCGUU 5’

query=JS16_t2228736, target=FGSG_05043, score=4, range=638-655, strand=-1

target  5’ GGUUG-UUGGUGGUCGCAA 3’
           :::.. .::.:::::::::
query   3’ CCAGUGGACUACCAGCGUU 5’

query=JS16_t2228736, target=FGSG_13026, score=4, range=1658-1677, strand=-1

target  5’ ACUCACGCUGAUGGUCGCAC 3’
             :::: ::::::::::::
query   3’ CCAGUG-GACUACCAGCGUU 5’

query=JS16_t4021598, target=FGSG_12030, score=3, range=348-365, strand=-1

target  5’ CUCUGCGUUAAUGAGUCG 3’
           : :::::: :::::::::
query   3’ GCGACGCACUUACUCAGC 5’

query=JS16_t4021598, target=FGSG_13026, score=3, range=671-687, strand=-1

target  5’ AGCUGCGUGAAU-AGUCG 3’
            ::::::::::: :::::
query   3’ GCGACGCACUUACUCAGC 5’

query=JS16_t4021598, target=FGSG_02041, score=3.5, range=957-974, strand=-1

target  5’ UGCUGCGUGAAUGAGGCA 3’
           .:::::::::::::: :
query   3’ GCGACGCACUUACUCAGC 5’

query=JS16_t4021598, target=FGSG_07267, score=3.5, range=544-560, strand=-1

target  5’ CGUU-CGUGAAUGAGUGG 3’
           ::.: ::::::::::: :
query   3’ GCGACGCACUUACUCAGC 5’

query=JS16_t4021598, target=FGSG_12773, score=4, range=1017-1034, strand=1

target  5’ CGCUGCGUGGAUGGGUGG 3’
           :::::::::.:::.:: :
query   3’ GCGACGCACUUACUCAGC 5’

query=JS16_t4021598, target=FGSG_09360, score=4, range=527-544, strand=1

target  5’ UUGG-CUAGUGCUUCCAC 3’
           .::. :::::::::::::
query   3’ GACUAGAUCACGAAGGUG 5’

query=JS16_t4084390, target=FGSG_00340, score=3, range=1775-1792, strand=1

target  5’ UUGGGCUAGUGCUUCUAC 3’
           .::. ::::::::::.::
query   3’ GACUAGAUCACGAAGGUG 5’

query=JS16_t4084390, target=FGSG_03465, score=3.5, range=660-677, strand=1

target  5’ CUGGGCUGGUGCUUCUAC 3’
           :::. ::.:::::::.::
query   3’ GACUAGAUCACGAAGGUG 5’

query=JS16_t4084390, target=FGSG_09933, score=3.5, range=442-458, strand=1

target  5’ CUGG-CUGGUGCUUCCGC 3’
           :::. ::.::::::::.:
query   3’ GACUAGAUCACGAAGGUG 5’

query=JS16_t4084390, target=FGSG_02035, score=3.5, range=1802-1819, strand=1

target  5’ CCGAUCUAGUGCUUCCUU 3’
           : :::::::::::::: .
query   3’ GACUAGAUCACGAAGGUG 5’

query=JS16_t4084390, target=FGSG_08298, score=3.5, range=882-899, strand=-1

target  5’ UUGAUCUGGUCCUUCCAC 3’
           .::::::.:: :::::::
query   3’ GACUAGAUCACGAAGGUG 5’

query=JS16_t4084390, target=FGSG_05794, score=3.5, range=1201-1218, strand=-1

target  5’ CUGGUCUAGUUCUUCCAA 3’
           :::.:::::: ::::::
query   3’ GACUAGAUCACGAAGGUG 5’

query=JS16_t4084390, target=FGSG_06513, score=4, range=739-757, strand=1

target  5’ CUGAUCUUAGUGUUUCCAA 3’
           :::::: :::::.:::::
query   3’ GACUAG-AUCACGAAGGUG 5’

query=JS16_t4084390, target=FGSG_03774, score=4, range=838-855, strand=1

target  5’ CUGAUCAAGUGCUUCGAC 3’
           :::::: :::::::: ::
query   3’ GACUAGAUCACGAAGGUG 5’

query=JS16_t4084390, target=FGSG_09830, score=4, range=343-360, strand=1

target  5’ CAGAUCUGGUUCUUCCAC 3’
           : :::::.:: :::::::
query   3’ GACUAGAUCACGAAGGUG 5’

query=JS16_t4084390, target=FGSG_11282, score=4, range=406-424, strand=1

target  5’ CUGGAUCUAGUGCUUACGC 3’
           :: :::::::::::: :.:
query   3’ GA-CUAGAUCACGAAGGUG 5’

query=JS16_t4084390, target=FGSG_06888, score=4, range=665-681, strand=1

target  5’ CUG-UCUGGUUCUUCCAC 3’
           ::: :::.:: :::::::
query   3’ GACUAGAUCACGAAGGUG 5’

query=JS16_t4084390, target=FGSG_00158, score=4, range=65-81, strand=-1

target  5’ UUGAUCUGGUG-UUCCAU 3’
           .::::::.::: :::::.
query   3’ GACUAGAUCACGAAGGUG 5’

query=JS16_t4084390, target=FGSG_07232, score=4, range=2341-2358, strand=-1

target  5’ CUGCUCUAGUUCUUUCAC 3’
           ::: :::::: :::.:::
query   3’ GACUAGAUCACGAAGGUG 5’

query=JS16_t4084390, target=FGSG_06840, score=4, range=1348-1365, strand=-1

target  5’ CAGAUCUAGUGCUUUCUC 3’
           : ::::::::::::.: :
query   3’ GACUAGAUCACGAAGGUG 5’

query=JS16_t4084390, target=FGSG_11696, score=4, range=2644-2661, strand=-1

target  5’ CACAUCUGGUGCUUCCAA 3’
```

```
target  5' CGCCGCGUGGAUGGGUUG 3'
           ::: :::::.:::.::.:
query   3' GCGACGCACUUACUCAGC 5'

query=JS16_t4021598, target=FGSG_06925, score=4, range=273-291, strand=1

target  5' UGUUGAGUGGAAUGAGUCG 3'
           .:.:: :: ::::::::::
query   3' GCGACGCA-CUUACUCAGC 5'

query=JS16_t4021598, target=FGSG_13878, score=4, range=4581-4598, strand=-1

target  5' CCCUGCGUGGAAGAGUCG 3'
           : :::::::.: ::::::
query   3' GCGACGCACUUACUCAGC 5'

query=JS16_t4021598, target=FGSG_04150, score=4, range=148-165, strand=-1

target  5' GACUGCGUGAAAGAGUCG 3'
             ::::::::: ::::::
query   3' GCGACGCACUUACUCAGC 5'

query=JS16_t4021598, target=FGSG_05455, score=4, range=429-445, strand=-1

target  5' GGCU-CGUGAAUGGGUCC 3'
            ::: ::::::::.:::
query   3' GCGACGCACUUACUCAGC 5'

query=JS16_t4021598, target=FGSG_09647, score=4, range=917-933, strand=-1

target  5' AGCU-CCUGAAUGAGUCG 3'
            ::: : ::::::::::
query   3' GCGACGCACUUACUCAGC 5'

query=JS16_t4021598, target=FGSG_04110, score=4, range=2057-2073, strand=-1

target  5' CGCUUCGUGAA-GGGUCG 3'
           :::: :::::: :.::::
query   3' GCGACGCACUUACUCAGC 5'

query=JS16_t0467535, target=FGSG_12745, score=4, range=1516-1537, strand=1

target  5' GAUGCAAUGAACAAUUCUGAGC 3'
           ::.:  :.:::::::::::::
query   3' CUGCUAUGCUUGUUAAGACUCA 5'

No results for JS16_t4021645
query=JS16_t1332370, target=FGSG_13700, score=2.5, range=1511-1531, strand=1

target  5' CGCCUGCUGAUGAACCAGAGC 3'
            :: :::.:::::::::::::
query   3' CCGUACGGCUACUUGGUCUCG 5'

query=JS16_t1332370, target=FGSG_09637, score=3, range=355-375, strand=1

target  5' GGCUUUCCGAUGAACCAGGGC 3'
           ::: : ::::::::::::.::
query   3' CCGUACGGCUACUUGGUCUCG 5'

query=JS16_t1332370, target=FGSG_05722, score=3.5, range=370-389, strand=-1

target  5' AGCAUG-AGAUGAACCAGAGU 3'
            :::::  ::::::::::::.
query   3' CCGUACGGCUACUUGGUCUCG 5'

query=JS16_t1332370, target=FGSG_10882, score=4, range=1754-1774, strand=1

target  5' AGUAUGUCGGUGAAUCAGAGC 3'
            :.:::.::.::::.::::::
query   3' CCGUACGGCUACUUGGUCUCG 5'

query=JS16_t1332370, target=FGSG_06858, score=4, range=435-455, strand=-1

target  5' GAUGCGCCGAUGAGCCAGAGC 3'
           : ..  ::::::::.:::::::
query   3' CCGUACGGCUACUUGGUCUCG 5'

query=JS16_t3125092, target=FGSG_09184, score=3.5, range=529-548, strand=-1

target  5' UGUCGAAGGAACGCGCGGUA 3'
            :::.::::::::.:::::.:
query   3' CCAGUUUCCUUGUGCGCCGU 5'

query=JS16_t3125092, target=FGSG_06666, score=4, range=1373-1392, strand=-1

target  5' AGUCAACGGAACAGGCGGCA 3'
            ::::: :::::: ::::::
query   3' CCAGUUUCCUUGUGCGCCGU 5'

query=JS16_t0176347, target=FGSG_02870, score=2.5, range=2071-2089, strand=1
           :  ::::.:::::::::
query   3' GACUAGAUCACGAAGGUG 5'

query=JS16_t4084390, target=FGSG_08716, score=4, range=1107-1125, strand=-1

target  5' AUGAUCCAAGUGCUUCCAC 3'
            ::::  : ::::::::::
query   3' GACUA-GAUCACGAAGGUG 5'

query=JS16_t4084390, target=FGSG_05128, score=4, range=575-591, strand=-1

target  5' CUUA-CCAGUGCUUCCAC 3'
           :: : : :::::::::::
query   3' GACUAGAUCACGAAGGUG 5'

query=JS16_t4084390, target=FGSG_07462, score=4, range=1418-1434, strand=-1

target  5' CUCAUC-AGUGCUUCUAC 3'
           :: ::: ::::::::.::
query   3' GACUAGAUCACGAAGGUG 5'

query=JS16_t4084390, target=FGSG_13748, score=4, range=42-60, strand=-1

target  5' CUGCUCUGGUGGCUUCCAC 3'
           ::: :::.:: ::::::::
query   3' GACUAGAUCA-CGAAGGUG 5'

query=JS16_t4084390, target=FGSG_07452, score=4, range=532-550, strand=-1

target  5' AUGGUUUAGUUGCUUCCAC 3'
            ::.:.::: :::::::::
query   3' GACUAGAUC-ACGAAGGUG 5'

query=JS16_t0307672, target=FGSG_04902, score=4, range=273-293, strand=1

target  5' GGGUGAUCGCGUAGCUUUCCU 3'
           :::.: :::::: :::::::.
query   3' CCCGCAAGCGCAGCGAAAGGG 5'

No results for JS16_t3187779
No results for JS16_t0396266
No results for JS16_t4084545
query=JS16_t4084558, target=FGSG_10187, score=3, range=2385-2403, strand=1

target  5' UAUGGAACGUCGUCCAAGC 3'
           ::::: ::::::.:::::
query   3' AUACCGUGCAGCGGGUUCU 5'

query=JS16_t4084558, target=FGSG_01210, score=3, range=405-422, strand=-1

target  5' UGUGGUACGUCG-CCAAGA 3'
           :.:::.:::::: ::::::
query   3' AUACCGUGCAGCGGGUUCU 5'

query=JS16_t4084558, target=FGSG_11352, score=3, range=174-193, strand=-1

target  5' CAUCGGGACGUCGCCCAAGA 3'
            :: :: ::::::::::::
query   3' AUA-CCGUGCAGCGGGUUCU 5'

query=JS16_t4084558, target=FGSG_05691, score=4, range=2169-2188, strand=1

target  5' UAUAGCCAAGUCGCCCAAGA 3'
           ::: : :: :::::::::::
query   3' AUA-CCGUGCAGCGGGUUCU 5'

query=JS16_t4084558, target=FGSG_03579, score=4, range=332-350, strand=1

target  5' CAUUACACGUCGCCCAAGC 3'
            ::  :::::::::::::
query   3' AUACCGUGCAGCGGGUUCU 5'

query=JS16_t4084558, target=FGSG_09509, score=4, range=2925-2942, strand=1

target  5' CAUGG-ACGUCGCCCAUGA 3'
            :::: :::::::::: ::
query   3' AUACCGUGCAGCGGGUUCU 5'

query=JS16_t4084558, target=FGSG_02151, score=4, range=45-62, strand=1

target  5' CAAGG-AUGUCGCCCAAGA 3'
            : :: :.::::::::::
query   3' AUACCGUGCAGCGGGUUCU 5'

query=JS16_t4084558, target=FGSG_09000, score=4, range=308-325, strand=1

target  5' UCUGG-ACCUCGCCCAAGA 3'
           : ::: :: ::::::::::
query   3' AUACCGUGCAGCGGGUUCU 5'

query=JS16_t4084558, target=FGSG_04736, score=4, range=177-195, strand=-1
```

```
target  5’ GUGCUCAUUUACUUCAUGG 3’
            ::::::.::::::::::.
query   3’ GACGAGUGAAUGAAGUACU 5’

query=JS16_t0176347, target=FGSG_00630, score=3, range=980-998, strand=1

target  5’ CUGCUGAUUUGCUUCAUGA 3’
           ::::: :.::.::::::::
query   3’ GACGAGUGAAUGAAGUACU 5’

query=JS16_t0176347, target=FGSG_11550, score=4, range=1366-1384, strand=1

target  5’ AUGCCCAAUUACUUCAUGA 3’
            ::: :: :::::::::::
query   3’ GACGAGUGAAUGAAGUACU 5’

query=JS16_t0176347, target=FGSG_06069, score=4, range=1661-1678, strand=1

target  5’ UUGCU-ACUUACUUGAUGG 3’
           .:::: :::::::: :::.
query   3’ GACGAGUGAAUGAAGUACU 5’

query=JS16_t0176347, target=FGSG_08861, score=4, range=1463-1481, strand=-1

target  5’ CUGCUCACAAACUUCAUGA 3’
           ::::::::  :::::::::
query   3’ GACGAGUGAAUGAAGUACU 5’

query=JS16_t0335385, target=FGSG_02711, score=2, range=380-397, strand=1

target  5’ CCAACCAGUUGGUGUCCA 3’
            ::::::::::.::::::
query   3’ UGUUGGUCAACUACAGGU 5’

query=JS16_t0335385, target=FGSG_11978, score=3, range=1445-1462, strand=1

target  5’ ACAACCAGUUGAUGCUCA 3’
           :::::::::::::: .::
query   3’ UGUUGGUCAACUACAGGU 5’

query=JS16_t0335385, target=FGSG_02366, score=3, range=194-210, strand=1

target  5’ ACGGCCAGUUGAUG-CCA 3’
           ::..:::::::::: :::
query   3’ UGUUGGUCAACUACAGGU 5’

query=JS16_t0335385, target=FGSG_06489, score=3, range=613-629, strand=1

target  5’ ACAA-CAGUUGAUGCCCA 3’
           :::: ::::::::: :::
query   3’ UGUUGGUCAACUACAGGU 5’

query=JS16_t0335385, target=FGSG_08033, score=3, range=94-111, strand=-1

target  5’ AGAAGUAGUUGAUGUCCA 3’
           : :: .::::::::::::
query   3’ UGUUGGUCAACUACAGGU 5’

query=JS16_t0335385, target=FGSG_02288, score=3, range=3776-3793, strand=-1

target  5’ GAAAGCAGUUGAUGUCCG 3’
           . :: ::::::::::::.
query   3’ UGUUGGUCAACUACAGGU 5’

query=JS16_t0335385, target=FGSG_06272, score=3.5, range=1907-1924, strand=1

target  5’ AUAACCAGUUGAUUUCCU 3’
           :.:::::::::: :::
query   3’ UGUUGGUCAACUACAGGU 5’

query=JS16_t0335385, target=FGSG_01843, score=3.5, range=485-502, strand=1

target  5’ UCAUCCAGUUGAUGUUCG 3’
            :: ::::::::::.:.
query   3’ UGUUGGUCAACUACAGGU 5’

query=JS16_t0335385, target=FGSG_06128, score=3.5, range=1519-1537, strand=1

target  5’ ACAAACCAGUUGAUCUCCG 3’
           :: ::::::::::: :::.
query   3’ UG-UUGGUCAACUACAGGU 5’

query=JS16_t0335385, target=FGSG_13485, score=3.5, range=128-146, strand=1

target  5’ GUAUCCCAGUUGAUGUCCG 3’
           ..:  :::::::::::::.
query   3’ UGU-UGGUCAACUACAGGU 5’

query=JS16_t0335385, target=FGSG_12776, score=3.5, range=3608-3625, strand=1

target  5’ GCUUCCAGUUGAUGUCCC 3’
           .:  ::::::::::::
```

```
target  5’ GAUGGCGCGUCGCUCAAGU 3’
            ::::.::::::.::::
query   3’ AUACCGUGCAGCGGGUUCU 5’

query=JS16_t4084558, target=FGSG_04345, score=4, range=1280-1298, strand=-1

target  5’ AAGAACACGUCGCCCAAGA 3’
            :   :::::::::::::
query   3’ AUACCGUGCAGCGGGUUCU 5’

No results for JS16_t3187895
No results for JS16_t0157702
No results for JS16_t4084593
query=JS16_t3187942, target=FGSG_08795, score=3.5, range=412-430, strand=-1

target  5’ AUU-GAGGCUGCCAAUCUGG 3’
           :.: ::.:::.::::::::.
query   3’ UGACCUUCGAUGGUUAGACU 5’

query=JS16_t3187942, target=FGSG_12138, score=4, range=182-202, strand=1

target  5’ ACGGCGAAGCUACCGGUCUGA 3’
           :: : :::::::::..:::::
query   3’ UGAC-CUUCGAUGGUUAGACU 5’

query=JS16_t3187942, target=FGSG_06720, score=4, range=319-337, strand=1

target  5’ ACUGG-UGCUAUCAAUCUGC 3’
           :::::  ::::.:::::::
query   3’ UGACCUUCGAUGGUUAGACU 5’

query=JS16_t3187942, target=FGSG_09663, score=4, range=1269-1288, strand=-1

target  5’ CCUGGCGGCUACCAGUCUGG 3’
            :::: .:::::::.::::.
query   3’ UGACCUUCGAUGGUUAGACU 5’

query=JS16_t3187942, target=FGSG_07258, score=4, range=568-586, strand=-1

target  5’ ACUCG-AGCUUCCAAUCUGA 3’
           ::: : :::: :::::::::
query   3’ UGACCUUCGAUGGUUAGACU 5’

query=JS16_t0202710, target=FGSG_01364, score=3, range=1641-1661, strand=1

target  5’ UCUCAUCACGUUCUUUUGGCU 3’
           :::::::::::: ::::::
query   3’ AGAGUAGUGCAAG-AAACCGC 5’

query=JS16_t0202710, target=FGSG_08230, score=3.5, range=686-706, strand=1

target  5’ UCGUCCUUAUGUUCUUUGGCG 3’
           :: :: :.:.::::::::::
query   3’ AG-AGUAGUGCAAGAAACCGC 5’

query=JS16_t0202710, target=FGSG_03083, score=3.5, range=832-850, strand=1

target  5’ UUUCA-CAUGUUCUUUGGCA 3’
           :.::: ::.::::::::::
query   3’ AGAGUAGUGCAAGAAACCGC 5’

query=JS16_t0202710, target=FGSG_03412, score=3.5, range=1390-1408, strand=1

target  5’ UUUCAGCACG-UCUUUGGCG 3’
           :.::: :::: :::::::::
query   3’ AGAGUAGUGCAAGAAACCGC 5’

query=JS16_t0202710, target=FGSG_09571, score=3.5, range=28-46, strand=-1

target  5’ UCUUAU-ACGCUCUUUGGCG 3’
           :::.:: ::: :::::::::
query   3’ AGAGUAGUGCAAGAAACCGC 5’

query=JS16_t0202710, target=FGSG_04043, score=3.5, range=275-295, strand=-1

target  5’ UCUCGUCUUCGUUCUUUGGCG 3’
           ::::.::  :::::::::::
query   3’ AGAGUAG-UGCAAGAAACCGC 5’

query=JS16_t0202710, target=FGSG_12619, score=4, range=219-238, strand=1

target  5’ UCUCAUGACCUUUUUUGGCG 3’
           :::::: :: ::.::::::
query   3’ AGAGUAGUGCAAGAAACCGC 5’

query=JS16_t0202710, target=FGSG_02400, score=4, range=3278-3298, strand=1

target  5’ UCAUCAUCAUCUUCUUUGGCG 3’
           :: :::::. ::::::::::
query   3’ AG-AGUAGUGCAAGAAACCGC 5’

query=JS16_t0202710, target=FGSG_07917, score=4, range=654-673, strand=1
```

```
query   3' UGUUGGUCAACUACAGGU 5'

query=JS16_t0335385, target=FGSG_02548, score=3.5, range=288-305, strand=-1

target  5' GCAACUAGUUGCUGUCCA 3'
           .::::.::::: ::::::
query   3' UGUUGGUCAACUACAGGU 5'

query=JS16_t0335385, target=FGSG_02133, score=3.5, range=561-579, strand=-1

target  5' GCAGUCAGUUGAUGUGCCA 3'
           .::..:::::::::: :::
query   3' UGUUGGUCAACUACA-GGU 5'

query=JS16_t0335385, target=FGSG_11395, score=3.5, range=599-617, strand=-1

target  5' GCGAGUCGGUUGAUGUCCA 3'
           .: :..:.:::::::::::
query   3' UG-UUGGUCAACUACAGGU 5'

query=JS16_t0335385, target=FGSG_04397, score=3.5, range=602-619, strand=-1

target  5' ACUGGUAGUUGAUGUCCA 3'
           :: . .::::::::::::
query   3' UGUUGGUCAACUACAGGU 5'

query=JS16_t0335385, target=FGSG_13355, score=3.5, range=356-372, strand=-1

target  5' ACAAUC-GUUGAUGUCCC 3'
           ::::.: ::::::::::
query   3' UGUUGGUCAACUACAGGU 5'

query=JS16_t0335385, target=FGSG_02477, score=4, range=1187-1205, strand=1

target  5' GUGAUCAGGUUGAUGUCCA 3'
           ...:.:: :::::::::::
query   3' UGUUGGU-CAACUACAGGU 5'

query=JS16_t0335385, target=FGSG_05530, score=4, range=122-139, strand=1

target  5' CCAACCAGUUGAUGGUCA 3'
            ::::::::::::: .::
query   3' UGUUGGUCAACUACAGGU 5'

query=JS16_t0335385, target=FGSG_05028, score=4, range=1050-1067, strand=1

target  5' UCUGCUAGUUGAUGUCCG 3'
            : .:.:::::::::::.
query   3' UGUUGGUCAACUACAGGU 5'

query=JS16_t0335385, target=FGSG_05999, score=4, range=227-244, strand=1

target  5' ACAACCACUUGAUGCCCA 3'
           ::::::: :::::: :::
query   3' UGUUGGUCAACUACAGGU 5'

query=JS16_t0335385, target=FGSG_08209, score=4, range=7028-7046, strand=1

target  5' UCAAUCCAGUUGAUGUUCC 3'
            ::: ::::::::::::.:
query   3' UGUU-GGUCAACUACAGGU 5'

query=JS16_t0335385, target=FGSG_01834, score=4, range=449-466, strand=1

target  5' AGAACCAGUGGAUGUCCU 3'
           : ::::::: :::::::
query   3' UGUUGGUCAACUACAGGU 5'

query=JS16_t0335385, target=FGSG_11554, score=4, range=1202-1220, strand=1

target  5' UCAACGCAGUUGAUAUCCA 3'
            :::: :::::::: ::::
query   3' UGUUG-GUCAACUACAGGU 5'

query=JS16_t0335385, target=FGSG_05523, score=4, range=1857-1874, strand=1

target  5' GCUCUCAGUUGAUGUCCC 3'
           .:  .:::::::::::::
query   3' UGUUGGUCAACUACAGGU 5'

query=JS16_t0335385, target=FGSG_05542, score=4, range=3420-3436, strand=1

target  5' GCAG-CAGUGGAUGUCCA 3'
           .::. :::: ::::::::
query   3' UGUUGGUCAACUACAGGU 5'

query=JS16_t0335385, target=FGSG_04524, score=4, range=909-925, strand=1

target  5' ACAG-CAGGUGAUGUCCG 3'
           :::. ::: ::::::::.
query   3' UGUUGGUCAACUACAGGU 5'
```

```
target  5' UGUCCUCAUGUUCUUUGGUG 3'
           : :: :::.:::::::::.:
query   3' AGAGUAGUGCAAGAAACCGC 5'

query=JS16_t0202710, target=FGSG_08856, score=4, range=158-178, strand=-1

target  5' UCUCCUUGCGUUCUUUGCGCG 3'
           :::: :..::::::::: :::
query   3' AGAGUAGUGCAAGAAAC-CGC 5'

query=JS16_t0202710, target=FGSG_06137, score=4, range=489-508, strand=-1

target  5' UCUCUUCACGUUCCUUGGCU 3'
           :::: :::::::: :::::
query   3' AGAGUAGUGCAAGAAACCGC 5'

No results for JS16_t0584129
No results for JS16_t1395715
No results for JS16_t4084723
query=JS16_t3188038, target=FGSG_04346, score=2, range=521-538, strand=-1

target  5' CAG-GGCCGAAAGGAGCAG 3'
           ::: : :::::::::::::
query   3' GUCACAGGCUUUCCUCGUC 5'

query=JS16_t3188038, target=FGSG_09374, score=3, range=218-236, strand=-1

target  5' CAUAGUCCGAGAGGAGCAG 3'
           ::  ::::::.::::::::
query   3' GUCACAGGCUUUCCUCGUC 5'

query=JS16_t3188038, target=FGSG_08791, score=3, range=2128-2145, strand=-1

target  5' CAAU-UCUGAAAGGAGCAG 3'
           :: : ::.:::::::::::
query   3' GUCACAGGCUUUCCUCGUC 5'

query=JS16_t3188038, target=FGSG_02534, score=4, range=492-510, strand=1

target  5' AAGUGUGCGAAGGGAGCAG 3'
            ::::: ::::.:::::::
query   3' GUCACAGGCUUUCCUCGUC 5'

query=JS16_t3188038, target=FGSG_07198, score=4, range=1486-1504, strand=1

target  5' CAAUGUCUGGAAGGAGCAU 3'
           :: ::::.:.::::::::
query   3' GUCACAGGCUUUCCUCGUC 5'

query=JS16_t3188038, target=FGSG_00893, score=4, range=1077-1095, strand=1

target  5' GAGUAUCCGAGAGGAGCAA 3'
            ::: :::::.:::::::
query   3' GUCACAGGCUUUCCUCGUC 5'

query=JS16_t3188038, target=FGSG_00289, score=4, range=96-113, strand=1

target  5' AAG-GUCCGAAAGGGGCAA 3'
            :: ::::::::::.:::
query   3' GUCACAGGCUUUCCUCGUC 5'

query=JS16_t3188038, target=FGSG_04070, score=4, range=660-678, strand=1

target  5' CCGUGACCGCAAGGAGCAG 3'
           : ::: ::: :::::::::
query   3' GUCACAGGCUUUCCUCGUC 5'

query=JS16_t3188038, target=FGSG_00351, score=4, range=287-306, strand=1

target  5' CAGCGUACGAGAAGGAGCAG 3'
           ::: :: ::: :::::::::
query   3' GUCACAGGCU-UUCCUCGUC 5'

query=JS16_t3188038, target=FGSG_02434, score=4, range=1859-1876, strand=-1

target  5' UGGUGUCCG-AAGGAGCGG 3'
           ..::::::: :::::::.:
query   3' GUCACAGGCUUUCCUCGUC 5'

query=JS16_t3188038, target=FGSG_08551, score=4, range=84-101, strand=-1

target  5' UGGUGUUCGAAAGG-GCAG 3'
           ..::::.::::::: ::::
query   3' GUCACAGGCUUUCCUCGUC 5'

query=JS16_t3188038, target=FGSG_03417, score=4, range=1339-1358, strand=-1

target  5' CGGGGUUCGAAAGGAGGCAG 3'
           :.: ::.:::::::: ::::
query   3' GUCACAGGCUUUCCU-CGUC 5'
```

```
query=JS16_t0335385, target=FGSG_04727, score=4, range=269-285, strand=1

target  5’ AGGACCAGUUGAU-UCCG 3’
           : .::::::::: :::.
query   3’ UGUUGGUCAACUACAGGU 5’

query=JS16_t0335385, target=FGSG_08454, score=4, range=826-842, strand=1

target  5’ UCAACUA-UUGAUGUCCA 3’
            ::::.: :::::::::
query   3’ UGUUGGUCAACUACAGGU 5’

query=JS16_t0335385, target=FGSG_10633, score=4, range=1219-1236, strand=-1

target  5’ ACAGCCAGUUGAUCUCUG 3’
           :::.:::::::: ::..
query   3’ UGUUGGUCAACUACAGGU 5’

query=JS16_t0335385, target=FGSG_08716, score=4, range=269-286, strand=-1

target  5’ GCGACUCGUUGAUGUCCA 3’
           .:.::. ::::::::::
query   3’ UGUUGGUCAACUACAGGU 5’

query=JS16_t0335385, target=FGSG_13572, score=4, range=365-383, strand=-1

target  5’ ACAAUUGGUUGGAUGUCCA 3’
           ::::...::: ::::::::
query   3’ UGUUGGUCAA-CUACAGGU 5’

query=JS16_t0335385, target=FGSG_03214, score=4, range=677-694, strand=-1

target  5’ CGGGCCAGUUGAUGUUCA 3’
             ..::::::::::.::
query   3’ UGUUGGUCAACUACAGGU 5’

query=JS16_t0335385, target=FGSG_00142, score=4, range=428-445, strand=-1

target  5’ CCAUCCAGUUGGUGUUCA 3’
            :: ::::::.:::.::
query   3’ UGUUGGUCAACUACAGGU 5’

query=JS16_t0335385, target=FGSG_03111, score=4, range=1844-1861, strand=-1

target  5’ ACAACCAGAUGAUGCCCA 3’
           :::::::: ::::: :::
query   3’ UGUUGGUCAACUACAGGU 5’

query=JS16_t0335385, target=FGSG_08330, score=4, range=268-284, strand=-1

target  5’ AUAGUCAGUUGAU-UCCG 3’
           :.:..:::::::: :::.
query   3’ UGUUGGUCAACUACAGGU 5’

query=JS16_t0335385, target=FGSG_10358, score=4, range=1185-1202, strand=-1

target  5’ AUACCCAGUUGACGUCCG 3’
           :.: :::::::: ::::.
query   3’ UGUUGGUCAACUACAGGU 5’

query=JS16_t0335385, target=FGSG_08472, score=4, range=209-227, strand=-1

target  5’ AUGCACCAGUGGAUGUCCA 3’
           :.. :::::: ::::::::
query   3’ UGU-UGGUCAACUACAGGU 5’

query=JS16_t0335385, target=FGSG_01440, score=4, range=308-326, strand=-1

target  5’ ACAACCCAGUUGAUGUACU 3’
           :::: ::::::::::: :
query   3’ UGUU-GGUCAACUACAGGU 5’

query=JS16_t0335385, target=FGSG_11115, score=4, range=1630-1646, strand=-1

target  5’ GC-ACCAGUUGAUGUCAG 3’
           .: ::::::::::::: .
query   3’ UGUUGGUCAACUACAGGU 5’

query=JS16_t0335385, target=FGSG_07777, score=4, range=713-729, strand=-1

target  5’ GC-GCCAGUUGAUCUCCA 3’
           .: .::::::::: ::::
query   3’ UGUUGGUCAACUACAGGU 5’

query=JS16_t0335385, target=FGSG_04367, score=4, range=567-583, strand=-1

target  5’ ACAUCCA-UUGAUGUCUA 3’
           ::: ::: ::::::::.:
query   3’ UGUUGGUCAACUACAGGU 5’

query=JS16_t0335385, target=FGSG_03265, score=4, range=450-466, strand=-1

query=JS16_t3188038, target=FGSG_03285, score=4, range=500-517, strand=-1

target  5’ UGAUG-CCGAAAGGAGCAA 3’
           .. :: ::::::::::::
query   3’ GUCACAGGCUUUCCUCGUC 5’

No results for JS16_t2291801
No results for JS16_t2291825
No results for JS16_t1395781
No results for JS16_t4084786
No results for JS16_t2291839
query=JS16_t1395821, target=FGSG_02938, score=4, range=1337-1356, strand=1

target  5’ UCGUGAUUUCAGAGGAUGGU 3’
           ::::: ::::: ::::::.:
query   3’ AGCAC-AAAGUGUCCUACUA 5’

query=JS16_t1395821, target=FGSG_01810, score=4, range=909-927, strand=1

target  5’ UGGUGUUUCACAAGAUGAG 3’
           : :::::::::: :::::
query   3’ AGCACAAAGUGUCCUACUA 5’

query=JS16_t1395821, target=FGSG_09632, score=4, range=589-608, strand=1

target  5’ UCGUUAUUCACGAGGAUGAU 3’
           ::::  ::::: ::::::::
query   3’ AGCACAAAGUG-UCCUACUA 5’

query=JS16_t1395821, target=FGSG_02835, score=4, range=176-193, strand=-1

target  5’ UC-UGGUUCACAGGUUGAU 3’
           :: :: :::::::: ::::
query   3’ AGCACAAAGUGUCCUACUA 5’

No results for JS16_t0584147
No results for JS16_t4084876
query=JS16_t3188251, target=FGSG_04479, score=2.5, range=146-163, strand=-1

target  5’ CGCCGGUUUGGAAGGAGA 3’
            .:::::::::::::::
query   3’ UUGGCCAAACCUUCCUCA 5’

query=JS16_t3188251, target=FGSG_04635, score=3, range=1674-1691, strand=1

target  5’ AACGGGUUUGGAGGGAGA 3’
           ::: ::::::::.::::
query   3’ UUGGCCAAACCUUCCUCA 5’

query=JS16_t3188251, target=FGSG_08356, score=3, range=315-331, strand=1

target  5’ UACC-GUUUGGAAGGAGA 3’
            ::: ::::::::::::
query   3’ UUGGCCAAACCUUCCUCA 5’

query=JS16_t3188251, target=FGSG_04321, score=3, range=160-177, strand=-1

target  5’ AAGCGGUUUGGAAGAAGU 3’
           :: ::::::::::: :::
query   3’ UUGGCCAAACCUUCCUCA 5’

query=JS16_t3188251, target=FGSG_11105, score=3.5, range=1695-1712, strand=1

target  5’ UAUGGGUUUGGAGGGAGU 3’
            :.  ::::::::.:::::
query   3’ UUGGCCAAACCUUCCUCA 5’

query=JS16_t3188251, target=FGSG_06660, score=3.5, range=758-775, strand=1

target  5’ GAACGGUCUGGAAGGAGU 3’
           .: :::: ::::::::::
query   3’ UUGGCCAAACCUUCCUCA 5’

query=JS16_t3188251, target=FGSG_05303, score=3.5, range=950-967, strand=-1

target  5’ GACCGGUUCGGAAGGGGU 3’
           .::::::: ::::::.::
query   3’ UUGGCCAAACCUUCCUCA 5’

query=JS16_t3188251, target=FGSG_12595, score=3.5, range=2880-2897, strand=-1

target  5’ AAUCGGUUUCGGAGGAGU 3’
           ::.:::::: :.::::::
query   3’ UUGGCCAAACCUUCCUCA 5’

query=JS16_t3188251, target=FGSG_05080, score=4, range=1639-1656, strand=1

target  5’ GAGUGGUUUGGAAAGAGU 3’
           .: .::::::::: ::::
query   3’ UUGGCCAAACCUUCCUCA 5’

query=JS16_t3188251, target=FGSG_11129, score=4, range=1000-1018, strand=1
```

```
target  5’ AGUACCAGUUGA-GUCCA 3’
           :  ::::::::: :::::
query   3’ UGUUGGUCAACUACAGGU 5’

query=JS16_t1332512, target=FGSG_04507, score=3.5, range=505-525, strand=-1

target  5’ GAGGAUGACCUUGCGGACGGU 3’
           .::.:.: ::::::::::::
query   3’ UUCUUGCGGGAACGCCUGCCU 5’

query=JS16_t1332512, target=FGSG_01015, score=3.5, range=1981-2001, strand=-1

target  5’ CAUGACGGCCUUGCGGACGGA 3’
            : .::: :::::::::::::
query   3’ UUCUUGCGGGAACGCCUGCCU 5’

query=JS16_t1332512, target=FGSG_06502, score=4, range=1070-1089, strand=-1

target  5’ AUCAACGCCCUUGCGG-CGGA 3’
           :  ::::::::::::: ::::
query   3’ UUCUUGCGGGAACGCCUGCCU 5’

query=JS16_t1332512, target=FGSG_00151, score=4, range=94-113, strand=-1

target  5’ AAG-ACGAUCUUGCGGACGGC 3’
           ::: ::: .:::::::::::
query   3’ UUCUUGCGGGAACGCCUGCCU 5’

query=JS16_t0794525, target=FGSG_03790, score=3, range=98-118, strand=-1

target  5’ GCAGAUCUUUAGACAGAUCCU 3’
           :::.: :.:::::::.:::::
query   3’ CGUUUCGGAAUCUGUUUAGGA 5’

No results for JS16_t4021819
query=JS16_t0685232, target=FGSG_08388, score=3, range=404-422, strand=-1

target  5’ CCUCUCUUGACUUUGCGGU 3’
           ::. . :::::::::::::
query   3’ GGGUGCAACUGAAACGCCA 5’

query=JS16_t0685232, target=FGSG_08798, score=3.5, range=399-416, strand=1

target  5’ CCUA-GGUGACUUUGCGGU 3’
           ::.: : ::::::::::::
query   3’ GGGUGCAACUGAAACGCCA 5’

query=JS16_t0685232, target=FGSG_02362, score=3.5, range=176-194, strand=-1

target  5’ AUCACGUUGGCUUUGCGGA 3’
            .:::::::.::::::::
query   3’ GGGUGCAACUGAAACGCCA 5’

query=JS16_t0685232, target=FGSG_04284, score=3.5, range=293-311, strand=-1

target  5’ AGAGCGUUGACUUUGCGGU 3’
              .:::::::::::::::
query   3’ GGGUGCAACUGAAACGCCA 5’

query=JS16_t0685232, target=FGSG_07761, score=3.5, range=553-570, strand=-1

target  5’ CCCGC-UUGACUUUGCAGU 3’
           :::.: :::::::::: ::
query   3’ GGGUGCAACUGAAACGCCA 5’

query=JS16_t0685232, target=FGSG_01463, score=4, range=1634-1652, strand=1

target  5’ CCUAUGUUGACUUUGCCGA 3’
           ::.:.::::::::::: :
query   3’ GGGUGCAACUGAAACGCCA 5’

query=JS16_t0685232, target=FGSG_06580, score=4, range=5486-5505, strand=1

target  5’ CCAACGAUGACUUUGCUGGU 3’
           :: ::: ::::::::: :::
query   3’ GGGUGCAACUGAAACG-CCA 5’

query=JS16_t0685232, target=FGSG_05794, score=4, range=6296-6314, strand=1

target  5’ ACAAGGUUGACUUUGCGGA 3’
            : : :::::::::::::
query   3’ GGGUGCAACUGAAACGCCA 5’

query=JS16_t0685232, target=FGSG_03123, score=4, range=160-178, strand=-1

target  5’ GCAGUGUUGACUUUGUGGU 3’
            : ..::::::::::.:::
query   3’ GGGUGCAACUGAAACGCCA 5’

query=JS16_t0685232, target=FGSG_06361, score=4, range=676-694, strand=-1

target  5’ CAUGCUUUGGCUUUGCGGU 3’
```

```
target  5’ AAGCGUGUUUGGGAGGAGA 3’
           :: :: ::::::.:::::
query   3’ UUGGC-CAAACCUUCCUCA 5’

query=JS16_t3188251, target=FGSG_05471, score=4, range=682-698, strand=1

target  5’ AUCCUGUUUGG-AGGAGU 3’
           : :: :::::: ::::::
query   3’ UUGGCCAAACCUUCCUCA 5’

query=JS16_t3188251, target=FGSG_07352, score=4, range=216-232, strand=1

target  5’ UACCAGUUU-GAAGGAGU 3’
            ::: :::: ::::::::
query   3’ UUGGCCAAACCUUCCUCA 5’

query=JS16_t3188251, target=FGSG_02454, score=4, range=437-454, strand=-1

target  5’ GAUCGGUUUGGAAGAGGU 3’
           .:.:::::::::: .::
query   3’ UUGGCCAAACCUUCCUCA 5’

query=JS16_t3188251, target=FGSG_12121, score=4, range=34-51, strand=-1

target  5’ GAUCGGUUUGGGAGGACU 3’
           .:.:::::::.:::: :
query   3’ UUGGCCAAACCUUCCUCA 5’

query=JS16_t3188251, target=FGSG_07536, score=4, range=497-514, strand=-1

target  5’ CACCGGUCUGGAGGGAGU 3’
            :::::: ::::.:::::
query   3’ UUGGCCAAACCUUCCUCA 5’

query=JS16_t3188251, target=FGSG_06195, score=4, range=1087-1104, strand=-1

target  5’ CAGCGGUUUAGAAGGAGU 3’
            : :::::: ::::::::
query   3’ UUGGCCAAACCUUCCUCA 5’

query=JS16_t3188251, target=FGSG_08679, score=4, range=611-628, strand=-1

target  5’ CAGCGGUUUGGAAGGCGU 3’
            : :::::::::::: ::
query   3’ UUGGCCAAACCUUCCUCA 5’

query=JS16_t3188251, target=FGSG_10260, score=4, range=679-696, strand=-1

target  5’ AACAUCUUUGGAAGGAGU 3’
           :::   ::::::::::::
query   3’ UUGGCCAAACCUUCCUCA 5’

query=JS16_t3188251, target=FGSG_00014, score=4, range=612-628, strand=-1

target  5’ CACCCGUUUGG-AGGAGU 3’
            ::: :::::: ::::::
query   3’ UUGGCCAAACCUUCCUCA 5’

No results for JS16_t2292022
No results for JS16_t4084983
No results for JS16_t1396026
query=JS16_t0229040, target=FGSG_06939, score=2.5, range=1254-1273, strand=1

target  5’ GCGAACUGGUCGUCACCUUU 3’
           .: :: ::::::::::::::
query   3’ UG-UUCACCAGCAGUGGAAA 5’

query=JS16_t0229040, target=FGSG_05862, score=3.5, range=1060-1077, strand=1

target  5’ AUGGGUGGU-GUCACCUUU 3’
           :...::::: :::::::::
query   3’ UGUUCACCAGCAGUGGAAA 5’

query=JS16_t0229040, target=FGSG_07520, score=3.5, range=1941-1958, strand=-1

target  5’ ACGAG-GGUUGUCACUUUU 3’
           ::.:: :::.:::::.:::
query   3’ UGUUCACCAGCAGUGGAAA 5’

query=JS16_t0229040, target=FGSG_02558, score=3.5, range=340-357, strand=-1

target  5’ GC-AGUGGUCGUCACAUUU 3’
           .: :::::::::::: :::
query   3’ UGUUCACCAGCAGUGGAAA 5’

query=JS16_t0229040, target=FGSG_13286, score=4, range=39-57, strand=1

target  5’ ACAAGUUGUCGACACCUUU 3’
           :::::: :::: :::::::
query   3’ UGUUCACCAGCAGUGGAAA 5’
```

```
           : ..: :::.:::::::::
query   3’ GGGUGCAACUGAAACGCCA 5’

query=JS16_t0685232, target=FGSG_08829, score=4, range=862-880, strand=-1

target  5’ CCCAGGUUGAGUUUGCGGC 3’
           :::: ::::: :::::::
query   3’ GGGUGCAACUGAAACGCCA 5’

query=JS16_t0685232, target=FGSG_13685, score=4, range=98-116, strand=-1

target  5’ CCAACGUUGACGUUGCGGG 3’
           :: :::::::: ::::::
query   3’ GGGUGCAACUGAAACGCCA 5’

query=JS16_t4021846, target=FGSG_03667, score=3, range=575-593, strand=1

target  5’ UACGUGGAAAGUUCAUUAC 3’
             : :::::::::::::::
query   3’ UAGGACCUUUCAAGUAAUG 5’

query=JS16_t4021846, target=FGSG_12087, score=3.5, range=146-164, strand=-1

target  5’ GUCUUGGAAAUUUCAUUAU 3’
           .::.:::::: :::::::.
query   3’ UAGGACCUUUCAAGUAAUG 5’

query=JS16_t4021846, target=FGSG_00503, score=3.5, range=67-85, strand=-1

target  5’ AUCCGGGAAGGUUCGUUAU 3’
           :::: ::::.::::.:::.
query   3’ UAGGACCUUUCAAGUAAUG 5’

query=JS16_t4021846, target=FGSG_00121, score=4, range=1613-1631, strand=1

target  5’ ACGGUGGAAAGUUCAUUGC 3’
           :   :::::::::::::.:
query   3’ UAGGACCUUUCAAGUAAUG 5’

query=JS16_t4021846, target=FGSG_06989, score=4, range=794-811, strand=-1

target  5’ AUCCUGGAGAGUUUA-UAC 3’
           ::::::::.::::.: :::
query   3’ UAGGACCUUUCAAGUAAUG 5’

query=JS16_t4021846, target=FGSG_03938, score=4, range=1171-1189, strand=-1

target  5’ AUCCGGGAAGGUUAAUUAC 3’
           :::: ::::.::: :::::
query   3’ UAGGACCUUUCAAGUAAUG 5’

No results for JS16_t4021925
No results for JS16_t3125359
query=JS16_t2229147, target=FGSG_04097, score=2.5, range=387-405, strand=1

target  5’ CCUGAACCCUUACUCAUGG 3’
           : :: :::::::::::::.
query   3’ GAACAUGGGAAUGAGUACU 5’

query=JS16_t2229147, target=FGSG_00801, score=3, range=165-182, strand=1

target  5’ CUUGGACCCUUAC-CAUGA 3’
           :::: :::::::: :::::
query   3’ GAACAUGGGAAUGAGUACU 5’

query=JS16_t2229147, target=FGSG_07399, score=3, range=836-853, strand=-1

target  5’ CUUGU-CCCUUGCUCAUGC 3’
           ::::: :::::.::::::
query   3’ GAACAUGGGAAUGAGUACU 5’

query=JS16_t2229147, target=FGSG_09638, score=3, range=4379-4398, strand=-1

target  5’ CUGGUAUCCCUUACUCAUGU 3’
           :: ::: ::::::::::::
query   3’ GAACAU-GGGAAUGAGUACU 5’

query=JS16_t2229147, target=FGSG_04527, score=3.5, range=390-408, strand=1

target  5’ CCUGAACCCUUACUCGUGG 3’
           : :: ::::::::::.::.
query   3’ GAACAUGGGAAUGAGUACU 5’

query=JS16_t2229147, target=FGSG_10379, score=4, range=718-736, strand=1

target  5’ GGUGAACCUUUACUCAUGA 3’
             :: :::.::::::::::
query   3’ GAACAUGGGAAUGAGUACU 5’

query=JS16_t2229147, target=FGSG_04175, score=4, range=1562-1580, strand=1

target  5’ AUGGUACCCUUUCUCAUGA 3’
```

```
query=JS16_t0229040, target=FGSG_01809, score=4, range=252-270, strand=1

target  5’ ACAAAAGGUCGUCAUCUUG 3’
           ::::  ::::::::.:::
query   3’ UGUUCACCAGCAGUGGAAA 5’

query=JS16_t0229040, target=FGSG_06067, score=4, range=1251-1268, strand=1

target  5’ ACAAG-GGACGUCGCCUUU 3’
           ::::: :: ::::.:::::
query   3’ UGUUCACCAGCAGUGGAAA 5’

query=JS16_t0229040, target=FGSG_11580, score=4, range=222-239, strand=-1

target  5’ UCAA-UGGUCGUCGCUUUU 3’
            ::: ::::::::.:.:::
query   3’ UGUUCACCAGCAGUGGAAA 5’

query=JS16_t0229040, target=FGSG_05253, score=4, range=1525-1542, strand=-1

target  5’ AGAAG-GCUCGUCACCUUU 3’
           : ::: : :::::::::::
query   3’ UGUUCACCAGCAGUGGAAA 5’

No results for JS16_t4085019
query=JS16_t0241264, target=FGSG_07504, score=3, range=2168-2185, strand=-1

target  5’ UUUCAGAAACUGUUU-CAG 3’
           ::::::::::.:::: :::
query   3’ AAAGUCUUUGGCAAACGUC 5’

query=JS16_t0241264, target=FGSG_07748, score=4, range=824-841, strand=1

target  5’ UUUC-GGAAUCGUUUGCAA 3’
           :::: :.::.::::::::
query   3’ AAAGUCUUUGGCAAACGUC 5’

query=JS16_t0241264, target=FGSG_07616, score=4, range=10-27, strand=1

target  5’ CUUC-GAAACCGUUUGCCG 3’
            ::: :::::::::::: :
query   3’ AAAGUCUUUGGCAAACGUC 5’

query=JS16_t0241264, target=FGSG_10464, score=4, range=7190-7209, strand=-1

target  5’ UUUCAAGAAGCCGUUGGCAG 3’
           :::: ::::.::::: ::::
query   3’ AAAG-UCUUUGGCAAACGUC 5’

query=JS16_t0241264, target=FGSG_03942, score=4, range=269-287, strand=-1

target  5’ UUACAAAGACUGUUUGCAG 3’
           :: :: :.::.::::::::
query   3’ AAAGUCUUUGGCAAACGUC 5’

query=JS16_t0241264, target=FGSG_09450, score=4, range=14-31, strand=-1

target  5’ AUUCA-AAGCCGUUUGCAC 3’
            :::: ::.:::::::::
query   3’ AAAGUCUUUGGCAAACGUC 5’

query=JS16_t0337478, target=FGSG_08785, score=3, range=235-253, strand=1

target  5’ ACCAUCUUCGU-CAAGCUUU 3’
            :::::::::: ::::::::
query   3’ GGGUAGAAGCACGUUCGAAA 5’

query=JS16_t0337478, target=FGSG_07053, score=4, range=2214-2234, strand=1

target  5’ CUUCUCUUCGUGCAACGCUUU 3’
           :..  ::::::::::: :::::
query   3’ GGGUAGAAGCACGUU-CGAAA 5’

No results for JS16_t1396085
No results for JS16_t0153581
query=JS16_t0337482, target=FGSG_03778, score=4, range=306-325, strand=-1

target  5’ CGUCGAUACCGUAGACUUCA 3’
           ::::.:.:::.::::::.:
query   3’ GCAGUUGUGGUAUCUGAGGC 5’

query=JS16_t0337482, target=FGSG_02269, score=4, range=134-153, strand=-1

target  5’ CGUCAACGCCGUAGACUCGG 3’
           :::::::.::.::::::: :
query   3’ GCAGUUGUGGUAUCUGAGGC 5’

query=JS16_t0337482, target=FGSG_06456, score=4, range=969-988, strand=-1

target  5’ CUUCAACACCAUAGACCCUG 3’
           : ::::::::::::: :.:
query   3’ GCAGUUGUGGUAUCUGAGGC 5’
```

```
            : :::::::: :::::::
query   3' GAACAUGGGAAUGAGUACU 5'

query=JS16_t2229147, target=FGSG_04725, score=4, range=873-890, strand=1

target  5' CUU-UACCUAUACUCAUGA 3'
           ::: ::::. :::::::::
query   3' GAACAUGGGAAUGAGUACU 5'

query=JS16_t2229147, target=FGSG_09510, score=4, range=2563-2580, strand=1

target  5' CCUGU-UCCUUACUCAUGU 3'
           : ::: .:::::::::::
query   3' GAACAUGGGAAUGAGUACU 5'

query=JS16_t2229147, target=FGSG_11105, score=4, range=965-983, strand=-1

target  5' AGUGUAUCCUUAUUCAUGA 3'
             ::::.:::::.::::::
query   3' GAACAUGGGAAUGAGUACU 5'

No results for JS16_t4021992
query=JS16_t3125393, target=FGSG_09341, score=2, range=378-398, strand=-1

target  5' GCCAUACCGGGGAACUUGGCA 3'
           ::::::::.:.::::::::::
query   3' CGGUAUGGUCUCUUGAACCGU 5'

query=JS16_t3125393, target=FGSG_10080, score=2, range=762-781, strand=-1

target  5' GACA-ACCAGAGAACUUGGCA 3'
           : :: ::::::::::::::::
query   3' CGGUAUGGUCUCUUGAACCGU 5'

query=JS16_t3125393, target=FGSG_08430, score=3.5, range=570-591, strand=1

target  5' GCUGAUUCCAGAGAACUUGGUA 3'
           ::. :: :::::::::::::.:
query   3' CGG-UAUGGUCUCUUGAACCGU 5'

query=JS16_t3125393, target=FGSG_04617, score=4, range=320-340, strand=1

target  5' CUCAUGCCAGAGAGCUUGGCC 3'
            .:::.:::::::.::::::
query   3' CGGUAUGGUCUCUUGAACCGU 5'

query=JS16_t3125393, target=FGSG_00633, score=4, range=8159-8179, strand=1

target  5' GCGAUUCUAGAGAACUUGGUG 3'
           :: :: :.:::::::::::..
query   3' CGGUAUGGUCUCUUGAACCGU 5'

query=JS16_t3125393, target=FGSG_08722, score=4, range=2112-2133, strand=-1

target  5' ACCAUCAGCAGAGAACUUGGCU 3'
            :::: : :::::::::::::
query   3' CGGUA-UGGUCUCUUGAACCGU 5'

query=JS16_t0188455, target=FGSG_07391, score=3.5, range=567-585, strand=1

target  5' AAAUGCACGAAUCAACCGU 3'
           ::::: ::: ::::::::.
query   3' UUUACUUGCGUAGUUGGCG 5'

query=JS16_t0188455, target=FGSG_07255, score=3.5, range=656-674, strand=1

target  5' ACAUGGACGCAUCAACAGC 3'
           : :::.:::::::::: ::
query   3' UUUACUUGCGUAGUUGGCG 5'

query=JS16_t0188455, target=FGSG_06359, score=3.5, range=818-837, strand=1

target  5' CAAUGAACGACAUCAACCGU 3'
            :::::::: :::::::::.
query   3' UUUACUUGC-GUAGUUGGCG 5'

query=JS16_t0188455, target=FGSG_10736, score=4, range=921-940, strand=1

target  5' AGAUGAGCGCAUCAAAUCGC 3'
           :.::::.:::::: ::.:::
query   3' UUUACUUGCGUAG-UUGGCG 5'

query=JS16_t0188455, target=FGSG_07383, score=4, range=1058-1076, strand=1

target  5' GAUUGGACGCGUCGACCGC 3'
           .: ::.::::.::.:::::
query   3' UUUACUUGCGUAGUUGGCG 5'

query=JS16_t0188455, target=FGSG_00219, score=4, range=63-80, strand=1

target  5' AAACG-AUGUAUCAACCGC 3'
           ::: : :.:.:::::::::

query=JS16_t0337482, target=FGSG_06280, score=4, range=746-766, strand=-1

target  5' CUUCAAAAACCAUAGAUUCCG 3'
           : :: :: ::::::::.::::
query   3' GCAG-UUGUGGUAUCUGAGGC 5'

No results for JS16_t0337485
No results for JS16_t3188501
No results for JS16_t0284093
No results for JS16_t1396257
No results for JS16_t2292268
query=JS16_t1396276, target=FGSG_01426, score=3, range=390-412, strand=-1

target  5' UUUCAUCUUCACUGGAGACGUAA 3'
           ::::  :::::::::::::.:::
query   3' AAAGCUGAAGUGACCUCUGUAUU 5'

query=JS16_t1396276, target=FGSG_13245, score=4, range=956-979, strand=1

target  5' AUUCCACUCCGACUGGAGACAUAA 3'
            ::: ::: : :::::::::::::
query   3' AAAGCUGAAG-UGACCUCUGUAUU 5'

No results for JS16_t0475244
No results for JS16_t0255506
No results for JS16_t2292367
No results for JS16_t0367009
No results for JS16_t1396368
query=JS16_t0692828, target=FGSG_01814, score=1, range=264-281, strand=1

target  5' GAA-GAUGACUGGAGAGCA 3'
           ::: :::::::::::::::
query   3' CUUGCUACUGACCUCUCGU 5'

query=JS16_t0692828, target=FGSG_11009, score=2.5, range=1677-1694, strand=1

target  5' GAAUGAUG-CUGGAGAGCA 3'
           :::.:::: ::::::::::
query   3' CUUGCUACUGACCUCUCGU 5'

query=JS16_t0692828, target=FGSG_08019, score=3.5, range=1516-1534, strand=1

target  5' GGACGAUCACUGGAGAGUA 3'
           :.::::: :::::::::.:
query   3' CUUGCUACUGACCUCUCGU 5'

query=JS16_t0692828, target=FGSG_03011, score=3.5, range=597-615, strand=1

target  5' GAUCGGUGACUGGGGAGCU 3'
           :: ::.:::::::.::::
query   3' CUUGCUACUGACCUCUCGU 5'

query=JS16_t0692828, target=FGSG_06230, score=3.5, range=332-350, strand=1

target  5' GAACUAUGCCUGGAGAGCG 3'
           :::: ::: :::::::::.
query   3' CUUGCUACUGACCUCUCGU 5'

query=JS16_t0692828, target=FGSG_01163, score=3.5, range=1422-1439, strand=1

target  5' GAA-GGUCACUGGAGAGCA 3'
           ::: :.: :::::::::::
query   3' CUUGCUACUGACCUCUCGU 5'

query=JS16_t0692828, target=FGSG_00104, score=3.5, range=1428-1445, strand=1

target  5' GGCCGAUGACUGGA-AGCA 3'
           :.  ::::::::::: ::::
query   3' CUUGCUACUGACCUCUCGU 5'

query=JS16_t0692828, target=FGSG_09212, score=3.5, range=75-93, strand=-1

target  5' GAUUGAUGGCUGGGGAGCA 3'
           :: .::::.::::.:::::
query   3' CUUGCUACUGACCUCUCGU 5'

query=JS16_t0692828, target=FGSG_01243, score=3.5, range=1686-1704, strand=-1

target  5' AAACGAUGACUUGAGAGCG 3'
            :::::::::: ::::::.
query   3' CUUGCUACUGACCUCUCGU 5'

query=JS16_t0692828, target=FGSG_03727, score=3.5, range=301-318, strand=-1

target  5' GAGCGAUG-CUGGAGAGCU 3'
           ::.::::: :::::::::
query   3' CUUGCUACUGACCUCUCGU 5'

query=JS16_t0692828, target=FGSG_06003, score=4, range=90-108, strand=1

target  5' GAAUGGUGACUGGGGAGGA 3'
```

```
query   3’ UUUACUUGCGUAGUUGGCG 5’

query=JS16_t0188455, target=FGSG_13356, score=4, range=346-364, strand=-1

target  5’ GACUGGCCGCAUCAACCGC 3’
           .: ::. ::::::::::::
query   3’ UUUACUUGCGUAGUUGGCG 5’

query=JS16_t0188455, target=FGSG_01691, score=4, range=361-378, strand=-1

target  5’ AAUUGAAU-CAUCAACCGC 3’
           :: ::::. ::::::::::
query   3’ UUUACUUGCGUAGUUGGCG 5’

query=JS16_t1332779, target=FGSG_03376, score=2, range=305-322, strand=-1

target  5’ ACACGGCUUUCGUGAUCU 3’
           :: :::::::::.:::::
query   3’ UGGGCCGAAAGCGCUAGA 5’

query=JS16_t1332779, target=FGSG_13479, score=3.5, range=851-869, strand=1

target  5’ ACCUGGCUUUCGCUGGUCU 3’
           :::.::::::::: :.:::
query   3’ UGGGCCGAAAGCG-CUAGA 5’

query=JS16_t1332779, target=FGSG_09891, score=3.5, range=377-394, strand=1

target  5’ ACCUUGCUUUUGCGAUCC 3’
           :::. :::::.::::::
query   3’ UGGGCCGAAAGCGCUAGA 5’

query=JS16_t1332779, target=FGSG_05545, score=3.5, range=167-183, strand=1

target  5’ ACCUGGCUUUUG-GAUCU 3’
           :::.::::::.: :::::
query   3’ UGGGCCGAAAGCGCUAGA 5’

query=JS16_t1332779, target=FGSG_09584, score=3.5, range=197-215, strand=1

target  5’ GCGCCGGCUUUCGCGGUCG 3’
           .: ::::::::::::.::
query   3’ UG-GGCCGAAAGCGCUAGA 5’

query=JS16_t1332779, target=FGSG_13162, score=3.5, range=3051-3068, strand=1

target  5’ ACAUGGCUUUGGCGAUCU 3’
           :: .:::::: :::::::
query   3’ UGGGCCGAAAGCGCUAGA 5’

query=JS16_t1332779, target=FGSG_08312, score=3.5, range=4460-4477, strand=1

target  5’ ACAUGGCUUUCGCGCUCU 3’
           :: .:::::::::: :::
query   3’ UGGGCCGAAAGCGCUAGA 5’

query=JS16_t1332779, target=FGSG_02557, score=3.5, range=2711-2727, strand=1

target  5’ AUUU-GCUUUCGCGAUCA 3’
           :... ::::::::::::
query   3’ UGGGCCGAAAGCGCUAGA 5’

query=JS16_t1332779, target=FGSG_12534, score=3.5, range=748-764, strand=1

target  5’ AUGC-GCUUUCGCGAUCG 3’
           :. : ::::::::::::
query   3’ UGGGCCGAAAGCGCUAGA 5’

query=JS16_t1332779, target=FGSG_09678, score=4, range=861-878, strand=1

target  5’ UCCCGGUCUUCGCGAUCU 3’
            :::::. ::::::::::
query   3’ UGGGCCGAAAGCGCUAGA 5’

query=JS16_t1332779, target=FGSG_09073, score=4, range=1217-1235, strand=1

target  5’ AUCAUGGCUUUCGCUAUCU 3’
           :.: .::::::::: ::::
query   3’ UGG-GCCGAAAGCGCUAGA 5’

query=JS16_t1332779, target=FGSG_02346, score=4, range=1046-1063, strand=1

target  5’ ACCCAGCUUUCGCGAACA 3’
           :::: :::::::::: :
query   3’ UGGGCCGAAAGCGCUAGA 5’

query=JS16_t1332779, target=FGSG_10766, score=4, range=3528-3545, strand=1

target  5’ ACAAGGUUUUCGCGAUCA 3’
           ::  ::.::::::::::
query   3’ UGGGCCGAAAGCGCUAGA 5’

           :::.:.:::::::.::: :
query   3’ CUUGCUACUGACCUCUCGU 5’

query=JS16_t0692828, target=FGSG_10215, score=4, range=1238-1256, strand=1

target  5’ GGAUGAUCACUGGAGAGCC 3’
           :.:.::: ::::::::::
query   3’ CUUGCUACUGACCUCUCGU 5’

query=JS16_t0692828, target=FGSG_09231, score=4, range=1122-1139, strand=1

target  5’ GAGUGA-GACUGGGGAGCA 3’
           ::..:: ::::::.:::::
query   3’ CUUGCUACUGACCUCUCGU 5’

query=JS16_t0692828, target=FGSG_01829, score=4, range=755-773, strand=1

target  5’ GAAAGAUGAUUGGAGACCA 3’
           ::: :::::.:::::: ::
query   3’ CUUGCUACUGACCUCUCGU 5’

query=JS16_t0692828, target=FGSG_01908, score=4, range=410-427, strand=1

target  5’ GAACGAUGACUGGACA-CA 3’
           ::::::::::::: : ::
query   3’ CUUGCUACUGACCUCUCGU 5’

query=JS16_t0692828, target=FGSG_05477, score=4, range=991-1008, strand=1

target  5’ GAACAAUGGCUGG-GAGCA 3’
           :::: :::.:::: :::::
query   3’ CUUGCUACUGACCUCUCGU 5’

query=JS16_t0692828, target=FGSG_00484, score=4, range=108-127, strand=1

target  5’ GUAUGAUGACUCGGAGAGCG 3’
           : :.::::::: :::::::.
query   3’ CUUGCUACUGA-CCUCUCGU 5’

query=JS16_t0692828, target=FGSG_13583, score=4, range=4925-4942, strand=-1

target  5’ GGAUGGUGACUGGAG-GCG 3’
           :.:.:.::::::::: ::.
query   3’ CUUGCUACUGACCUCUCGU 5’

query=JS16_t0692828, target=FGSG_05214, score=4, range=249-267, strand=-1

target  5’ GAGCGCUGACUUGAGAGCG 3’
           ::.:: ::::: ::::::.
query   3’ CUUGCUACUGACCUCUCGU 5’

query=JS16_t0692828, target=FGSG_12166, score=4, range=973-991, strand=-1

target  5’ CAACGUUGACUGGGGAGCU 3’
            :::: :::::::.::::
query   3’ CUUGCUACUGACCUCUCGU 5’

query=JS16_t0692828, target=FGSG_06231, score=4, range=1338-1355, strand=-1

target  5’ GGAUG-UGACAGGAGAGCA 3’
           :.:.: :::: ::::::::
query   3’ CUUGCUACUGACCUCUCGU 5’

No results for JS16_t4085399
No results for JS16_t3188662
query=JS16_t4085413, target=FGSG_13479, score=4, range=622-641, strand=-1

target  5’ UGUACGGGUAUGCGGACAAG 3’
            ..:  ::::::::::::::
query   3’ UUGUCGCCAUACGCCUGUUC 5’

No results for JS16_t0802434
No results for JS16_t0396299
No results for JS16_t3188750
No results for JS16_t0584224
No results for JS16_t0692848
No results for JS16_t2292650
No results for JS16_t0692853
No results for JS16_t0255510
query=JS16_t0307710, target=FGSG_07469, score=3.5, range=2186-2208, strand=1

target  5’ CUGAUGGGUGGACUUCAGAGAUU 3’
           ::: .::.: ::::::::::::.
query   3’ GAC-GCCUAACUGAAGUCUCUAG 5’

query=JS16_t0307710, target=FGSG_07636, score=4, range=1546-1566, strand=-1

target  5’ AUGCGUAUUGACUUCA-AGAUC 3’
            :::: :::::::::: :::::
query   3’ GACGCCUAACUGAAGUCUCUAG 5’

query=JS16_t0307710, target=FGSG_07398, score=4, range=645-665, strand=-1
```

```
query=JS16_t1332779, target=FGSG_12782, score=4, range=93-109, strand=1

target  5’ CCCAGGCUUUCGC-AUCU 3’
            :: ::::::::: ::::
query   3’ UGGGCCGAAAGCGCUAGA 5’

query=JS16_t1332779, target=FGSG_07430, score=4, range=272-289, strand=-1

target  5’ AUCUGGCUUUCUCGAUUU 3’
           :.:.::::::: ::::.:
query   3’ UGGGCCGAAAGCGCUAGA 5’

query=JS16_t1332779, target=FGSG_07598, score=4, range=141-158, strand=-1

target  5’ ACCAGGUUUUCGUGAUUU 3’
           ::: ::.:::::.:::.:
query   3’ UGGGCCGAAAGCGCUAGA 5’

query=JS16_t1332779, target=FGSG_03679, score=4, range=1376-1393, strand=-1

target  5’ UCUUGGCUUUCGCAAUCU 3’
            :..::::::::: ::::
query   3’ UGGGCCGAAAGCGCUAGA 5’

query=JS16_t1332779, target=FGSG_08810, score=4, range=126-143, strand=-1

target  5’ ACUUGGCCUUCGCGAUCG 3’
           ::..::: :::::::::
query   3’ UGGGCCGAAAGCGCUAGA 5’

query=JS16_t1332779, target=FGSG_02387, score=4, range=1003-1020, strand=-1

target  5’ AUGUGGCUUUUGCGAUCC 3’
           :. .::::::.::::::
query   3’ UGGGCCGAAAGCGCUAGA 5’

query=JS16_t1332779, target=FGSG_12917, score=4, range=102-119, strand=-1

target  5’ AGCAGGCUUUUGUGAUCU 3’
           : : ::::::.:.:::::
query   3’ UGGGCCGAAAGCGCUAGA 5’

query=JS16_t1332779, target=FGSG_01442, score=4, range=3342-3360, strand=-1

target  5’ AUUCGGGCGUUCGCGAUCU 3’
           :..: ::: ::::::::::
query   3’ UGGG-CCGAAAGCGCUAGA 5’

query=JS16_t1332779, target=FGSG_10380, score=4, range=1014-1032, strand=-1

target  5’ AUUGCGGCUUUGGCGAUCU 3’
           :.. ::::::: :::::::
query   3’ UGG-GCCGAAAGCGCUAGA 5’

query=JS16_t1332779, target=FGSG_06373, score=4, range=197-213, strand=-1

target  5’ ACCCGGCUGUCGCGA-CU 3’
           :::::::: :::::: ::
query   3’ UGGGCCGAAAGCGCUAGA 5’

query=JS16_t0335397, target=FGSG_03547, score=2.5, range=293-312, strand=1

target  5’ UCACUGGAAAGUCUUCUUCU 3’
           ::::.:: ::::::::::::
query   3’ AGUGGCC-UUCAGAAGAAGA 5’

query=JS16_t0335397, target=FGSG_13878, score=3, range=28376-28394, strand=-1

target  5’ UCAUUAGAAGUCUUCUUCA 3’
           :::.. ::::::::::::
query   3’ AGUGGCCUUCAGAAGAAGA 5’

query=JS16_t0335397, target=FGSG_04474, score=3, range=459-476, strand=-1

target  5’ UCGUCGG-AGUCUUCUUCU 3’
           ::..::: :::::::::::
query   3’ AGUGGCCUUCAGAAGAAGA 5’

query=JS16_t0335397, target=FGSG_02621, score=3, range=1212-1229, strand=-1

target  5’ UCA-CGGAAGUCUUCUUUG 3’
           ::: :::::::::::::.
query   3’ AGUGGCCUUCAGAAGAAGA 5’

query=JS16_t0335397, target=FGSG_00550, score=3, range=2331-2348, strand=-1

target  5’ UCAAC-GAGGUCUUCUUCU 3’
           ::: : ::.::::::::::
query   3’ AGUGGCCUUCAGAAGAAGA 5’

query=JS16_t0335397, target=FGSG_09323, score=3.5, range=408-427, strand=1
```

```
target  5’ UUACGGGUUG-CUUCAGAGAUC 3’
           .: :::.::: :::::::::::
query   3’ GACGCCUAACUGAAGUCUCUAG 5’

No results for JS16_t0396313
No results for JS16_t0475287
No results for JS16_t2292728
No results for JS16_t4085694
No results for JS16_t3188967
query=JS16_t3188985, target=FGSG_10623, score=4, range=92-114, strand=-1

target  5’ GCCCCAUUCACUUUCACUACCAA 3’
           . ::: .:: :::::::::::::
query   3’ UAGGGAGAGGGAAAGUGAUGGUU 5’

query=JS16_t1396763, target=FGSG_07816, score=3.5, range=387-407, strand=1

target  5’ AUA-UUUUCCAGCAGGAGAUUU 3’
           ::: ::.: ::.::::::::::
query   3’ UAUGAAGAAGUUGUCCUCUAAA 5’

query=JS16_t1396769, target=FGSG_08368, score=4, range=871-891, strand=1

target  5’ GAGCAUGUGGCUGUGGAUCCU 3’
            ::: ::: ::::::::::::
query   3’ GUCG-ACAGCGACACCUAGGA 5’

query=JS16_t1396769, target=FGSG_00139, score=4, range=729-748, strand=-1

target  5’ CAGCUGUGGCUGUGGACCCU 3’
           ::::::: :::::::: :::
query   3’ GUCGACAGCGACACCUAGGA 5’

query=JS16_t1396769, target=FGSG_08650, score=4, range=560-580, strand=-1

target  5’ UAGUUCGUCGCUCUGGAUCCU 3’
           .::.: :::::: ::::::::
query   3’ GUCGA-CAGCGACACCUAGGA 5’

No results for JS16_t3189049
No results for JS16_t0220350
query=JS16_t1396805, target=FGSG_01295, score=2.5, range=1874-1891, strand=-1

target  5’ GGU-AAGUUUUCCCUUCAU 3’
           :::  ::::::::::::.
query   3’ CCACGUCAAAAGGGAAGUG 5’

query=JS16_t1396805, target=FGSG_01311, score=3.5, range=1447-1465, strand=1

target  5’ GGUGAGGUUUUCCCUACAC 3’
           :::: .::::::::: :::
query   3’ CCACGUCAAAAGGGAAGUG 5’

query=JS16_t1396805, target=FGSG_04700, score=3.5, range=1339-1357, strand=1

target  5’ GCUGAAGUUUUCCCUUCGU 3’
           : :: ::::::::::::..
query   3’ CCACGUCAAAAGGGAAGUG 5’

query=JS16_t1396805, target=FGSG_06279, score=4, range=203-221, strand=-1

target  5’ GCUGCAGCUUUCUCUUCAC 3’
           : ::::: ::::.::::::
query   3’ CCACGUCAAAAGGGAAGUG 5’

query=JS16_t1396805, target=FGSG_02772, score=4, range=749-767, strand=-1

target  5’ AGUGCAGUUUUCCCAUCAG 3’
            ::::::::::::: :::
query   3’ CCACGUCAAAAGGGAAGUG 5’

query=JS16_t1396805, target=FGSG_08660, score=4, range=658-676, strand=-1

target  5’ GCUGCAGUUUUCCCUGCAA 3’
           : ::::::::::::: ::
query   3’ CCACGUCAAAAGGGAAGUG 5’

No results for JS16_t1396808
query=JS16_t0396319, target=FGSG_11891, score=4, range=2205-2227, strand=1

target  5’ UCUCGUCGUCCUUGUCAUGCUUC 3’
           : :::: :: ::::::::::.::
query   3’ AAAGCA-CAUGAACAGUACGGAG 5’

No results for JS16_t1396859
No results for JS16_t3189215
No results for JS16_t0307716
No results for JS16_t1396962
query=JS16_t0367024, target=FGSG_06279, score=3.5, range=1838-1857, strand=1

target  5’ GCGAAUCACAGAGCAUGUUU 3’
```

```
target  5' CUGUCGCGAAGUCUUCUUCU 3'
            ...:: :::::::::::::
query   3' AGUGGC-CUUCAGAAGAAGA 5'

query=JS16_t0335397, target=FGSG_13839, score=3.5, range=1522-1540, strand=1

target  5' CUACCAGAAGUCUUCUUCC 3'
            .::: :::::::::::
query   3' AGUGGCCUUCAGAAGAAGA 5'

query=JS16_t0335397, target=FGSG_13715, score=3.5, range=485-502, strand=1

target  5' CCAUCGGAAGU-UUCUUCU 3'
            ::.::::::: :::::::
query   3' AGUGGCCUUCAGAAGAAGA 5'

query=JS16_t0335397, target=FGSG_02307, score=3.5, range=1091-1109, strand=1

target  5' CCCUCCGAAGUCUUCUUCU 3'
            : .: :::::::::::::
query   3' AGUGGCCUUCAGAAGAAGA 5'

query=JS16_t0335397, target=FGSG_13162, score=3.5, range=5409-5426, strand=-1

target  5' UCAUCGGAGGUC-UCUUCU 3'
           :::.::::.::: ::::::
query   3' AGUGGCCUUCAGAAGAAGA 5'

query=JS16_t0335397, target=FGSG_02778, score=3.5, range=294-311, strand=-1

target  5' UCACUGG-AGUCUUCUUCA 3'
           ::::.:: ::::::::::
query   3' AGUGGCCUUCAGAAGAAGA 5'

query=JS16_t0335397, target=FGSG_00438, score=4, range=755-773, strand=1

target  5' UUAUCGGUGGUCUUCUUCU 3'
           :.:.::: .::::::::::
query   3' AGUGGCCUUCAGAAGAAGA 5'

query=JS16_t0335397, target=FGSG_11600, score=4, range=246-263, strand=1

target  5' UCGUCGGAAGU-UUUUUCU 3'
           ::..::::::: ::.::::
query   3' AGUGGCCUUCAGAAGAAGA 5'

query=JS16_t0335397, target=FGSG_06088, score=4, range=19-37, strand=1

target  5' GCACCCGAGGUCUUCUUCA 3'
            :::: ::.:::::::::
query   3' AGUGGCCUUCAGAAGAAGA 5'

query=JS16_t0335397, target=FGSG_02521, score=4, range=474-492, strand=1

target  5' GCACCACAAGUCUUCUUCU 3'
            ::::  ::::::::::::
query   3' AGUGGCCUUCAGAAGAAGA 5'

query=JS16_t0335397, target=FGSG_01283, score=4, range=570-587, strand=1

target  5' UCACC-GAGGUAUUCUUCU 3'
           ::::: ::.:: :::::::
query   3' AGUGGCCUUCAGAAGAAGA 5'

query=JS16_t0335397, target=FGSG_00566, score=4, range=589-607, strand=1

target  5' UCUCCUGAAGGCUUCUUCU 3'
           :: :: :::: :::::::
query   3' AGUGGCCUUCAGAAGAAGA 5'

query=JS16_t0335397, target=FGSG_06494, score=4, range=17-34, strand=1

target  5' UC-CCGGAAGUCCUCUUCA 3'
           :: ::::::::: :::::
query   3' AGUGGCCUUCAGAAGAAGA 5'

query=JS16_t0335397, target=FGSG_04669, score=4, range=1096-1115, strand=1

target  5' GCUCCGGAAGUGCUUCUUCU 3'
            : :::::::: ::::::::
query   3' AGUGGCCUUCA-GAAGAAGA 5'

query=JS16_t0335397, target=FGSG_11731, score=4, range=366-384, strand=-1

target  5' GUAUCGGAAGGCUUCUUCU 3'
            .:.:::::: ::::::::
query   3' AGUGGCCUUCAGAAGAAGA 5'

query=JS16_t0335397, target=FGSG_00258, score=4, range=96-113, strand=-1

target  5' UC-UUGGGAGUCUUUUUCU 3'
           :: ..::.::::::.::::
            .:::::::: :::::.::::
query   3' UGCUUAGUG-CUCGUGCAAA 5'

query=JS16_t0367024, target=FGSG_02188, score=3.5, range=640-658, strand=-1

target  5' ACGUGUGACGAGCACGUUU 3'
            ::: .: ::::::::::::
query   3' UGCUUAGUGCUCGUGCAAA 5'

query=JS16_t0367024, target=FGSG_08794, score=3.5, range=318-337, strand=-1

target  5' AUGAUACCACGAGCAUGUUU 3'
            :.:: : ::::::::.::::
query   3' UGCU-UAGUGCUCGUGCAAA 5'

query=JS16_t0367024, target=FGSG_09848, score=4, range=1388-1406, strand=1

target  5' CCGAAUUGCGAGCGCGUUU 3'
             :::::..:::::.:::::
query   3' UGCUUAGUGCUCGUGCAAA 5'

query=JS16_t0367024, target=FGSG_08060, score=4, range=849-867, strand=-1

target  5' ACAAAUCACGGGCGUGUUU 3'
            :: :::::::.::..::::
query   3' UGCUUAGUGCUCGUGCAAA 5'

query=JS16_t0367024, target=FGSG_11624, score=4, range=997-1014, strand=-1

target  5' ACGAAU-ACGGGCAUGUUU 3'
            :::::: :::.:::.::::
query   3' UGCUUAGUGCUCGUGCAAA 5'

No results for JS16_t3189287
No results for JS16_t3189290
query=JS16_t1397031, target=FGSG_01270, score=1, range=81-98, strand=-1

target  5' GGGUGAUCAUGAGCUUGA 3'
            ::: :::::::::::::
query   3' CCCUCUAGUACUCGAACU 5'

query=JS16_t1397031, target=FGSG_09857, score=3, range=347-364, strand=1

target  5' AGGAGAUUAUGGGCUUGA 3'
             ::::::.:::.::::::
query   3' CCCUCUAGUACUCGAACU 5'

query=JS16_t1397031, target=FGSG_07642, score=3, range=1305-1322, strand=1

target  5' GGGUGGUCGUGAGCUUGA 3'
            ::: :.::.:::::::::
query   3' CCCUCUAGUACUCGAACU 5'

query=JS16_t1397031, target=FGSG_07910, score=3, range=241-257, strand=1

target  5' GGGA-AUCUUGAGCUUGA 3'
            :::: ::: :::::::::
query   3' CCCUCUAGUACUCGAACU 5'

query=JS16_t1397031, target=FGSG_10209, score=3, range=284-301, strand=-1

target  5' UGGAAAUCAUGGGCUUGA 3'
             ::: ::::::.::::::
query   3' CCCUCUAGUACUCGAACU 5'

query=JS16_t1397031, target=FGSG_07798, score=3, range=4553-4570, strand=-1

target  5' CAGACAUCAUGAGCUUGA 3'
              :: :::::::::::::
query   3' CCCUCUAGUACUCGAACU 5'

query=JS16_t1397031, target=FGSG_09663, score=3.5, range=575-592, strand=1

target  5' GAAAGGUCAUGAGCUUGG 3'
            :  ::.:::::::::::.
query   3' CCCUCUAGUACUCGAACU 5'

query=JS16_t1397031, target=FGSG_11108, score=3.5, range=57-74, strand=-1

target  5' GGCAGAUCAUGUGCUUGG 3'
            :: :::::::: :::::.
query   3' CCCUCUAGUACUCGAACU 5'

query=JS16_t1397031, target=FGSG_10132, score=3.5, range=554-570, strand=-1

target  5' GGGAGA-CAUGGGCUUGG 3'
            :::::: ::::.:::::.
query   3' CCCUCUAGUACUCGAACU 5'

query=JS16_t1397031, target=FGSG_02584, score=4, range=215-232, strand=1

target  5' UCGAGGUCAUGGGCUUGA 3'
```

```
query   3' AGUGGCCUUCAGAAGAAGA 5'

query=JS16_t0335397, target=FGSG_10800, score=4, range=428-446, strand=-1

target  5' UUCUCGGCAGUCUUCUUCU 3'
           :. .::: :::::::::::
query   3' AGUGGCCUUCAGAAGAAGA 5'

query=JS16_t0335397, target=FGSG_03202, score=4, range=830-848, strand=-1

target  5' GCACCAGGAGUCUUCUUCA 3'
            :::: :.::::::::::
query   3' AGUGGCCUUCAGAAGAAGA 5'

query=JS16_t0335397, target=FGSG_12901, score=4, range=1691-1709, strand=-1

target  5' ACAACGGCAGUCUUCUUCU 3'
            :: ::: :::::::::::
query   3' AGUGGCCUUCAGAAGAAGA 5'

query=JS16_t0335397, target=FGSG_01088, score=4, range=41-59, strand=-1

target  5' GCAGCUGGAGUCUUCUUCU 3'
            :: : :.:::::::::::
query   3' AGUGGCCUUCAGAAGAAGA 5'

query=JS16_t0335397, target=FGSG_07786, score=4, range=633-651, strand=-1

target  5' CCGGUCGAAGUCUUCUUCU 3'
            :. . :::::::::::::
query   3' AGUGGCCUUCAGAAGAAGA 5'

query=JS16_t0335397, target=FGSG_11159, score=4, range=522-541, strand=-1

target  5' UUGCCAUGAAGUCUUCUUCG 3'
           :..::  ::::::::::::
query   3' AGUGG-CCUUCAGAAGAAGA 5'

query=JS16_t0335397, target=FGSG_11273, score=4, range=237-254, strand=-1

target  5' UCA-CGGAAGUCUUCAUCA 3'
           ::: ::::::::::: ::
query   3' AGUGGCCUUCAGAAGAAGA 5'

query=JS16_t0335397, target=FGSG_06849, score=4, range=1643-1660, strand=-1

target  5' UCUCCUGAAGUC-UCUUCU 3'
           :: :: :::::: ::::::
query   3' AGUGGCCUUCAGAAGAAGA 5'

query=JS16_t0335397, target=FGSG_00299, score=4, range=644-661, strand=-1

target  5' UUAUC-GAAGGCUUCUUCU 3'
           :.:.: :::: ::::::::
query   3' AGUGGCCUUCAGAAGAAGA 5'

No results for JS16_t4022133
No results for JS16_t2229317
query=JS16_t0794565, target=FGSG_05213, score=3.5, range=3318-3337, strand=1

target  5' CAUCCUAGAGGGCAAUCCUA 3'
           :: : .:::::::::::::
query   3' GU-GUGUCUCCCGUUAGGAA 5'

query=JS16_t0794565, target=FGSG_10143, score=3.5, range=988-1006, strand=-1

target  5' CGCCCUGAGGGCAGUCCUU 3'
           :.: : :::::::.:::::
query   3' GUGUGUCUCCCGUUAGGAA 5'

query=JS16_t0794565, target=FGSG_00523, score=4, range=82-100, strand=1

target  5' CUCACUGAGGGCAAGCCUU 3'
           : ::: :::::::: ::::
query   3' GUGUGUCUCCCGUUAGGAA 5'

query=JS16_t0794565, target=FGSG_01066, score=4, range=2264-2282, strand=-1

target  5' CGUACAGAGGGCAUUCCUC 3'
           :..:::::::::: ::::
query   3' GUGUGUCUCCCGUUAGGAA 5'

query=JS16_t0219690, target=FGSG_09558, score=3.5, range=1975-1995, strand=-1

target  5' CGCCGCAUUGGUAGGGUGGCC 3'
            :::.:: :::::::::.:::
query   3' ACGGUGU-ACCAUCCCAUCGG 5'

query=JS16_t0219690, target=FGSG_12992, score=3.5, range=201-220, strand=-1

target  5' UGCUCCAUGGUAGGCUAGCC 3'
           :::. ::::::::: :::::
             :::.:::::.::::::
query   3' CCCUCUAGUACUCGAACU 5'

query=JS16_t1397031, target=FGSG_11532, score=4, range=576-593, strand=1

target  5' UGGAGAUCGUGUGCUUGA 3'
            :::::::.:: ::::::
query   3' CCCUCUAGUACUCGAACU 5'

query=JS16_t1397031, target=FGSG_12194, score=4, range=794-812, strand=1

target  5' UGGAGAUCAUGGGCUUUGA 3'
            ::::::::::.:: ::::
query   3' CCCUCUAGUACUCG-AACU 5'

query=JS16_t1397031, target=FGSG_02053, score=4, range=805-821, strand=1

target  5' GGGAGGUCAUGGGC-UGA 3'
           :::::.:::::.:: :::
query   3' CCCUCUAGUACUCGAACU 5'

query=JS16_t1397031, target=FGSG_10065, score=4, range=533-550, strand=1

target  5' CGGGUAUCGUGAGCUUGG 3'
            ::. :::.::::::::.
query   3' CCCUCUAGUACUCGAACU 5'

query=JS16_t1397031, target=FGSG_08358, score=4, range=1076-1093, strand=1

target  5' GUGAGUUCAUGGGCUUGA 3'
           : ::: :::::.::::::
query   3' CCCUCUAGUACUCGAACU 5'

query=JS16_t1397031, target=FGSG_06748, score=4, range=381-398, strand=1

target  5' GGAAGAUGAUGAGUUUGA 3'
           :: :::: :::::.::::
query   3' CCCUCUAGUACUCGAACU 5'

query=JS16_t1397031, target=FGSG_02609, score=4, range=347-364, strand=1

target  5' AAGAGAUCUUGAGCUUGA 3'
             :::::: :::::::::
query   3' CCCUCUAGUACUCGAACU 5'

query=JS16_t1397031, target=FGSG_08764, score=4, range=552-569, strand=1

target  5' GGCCGGUUAUGAGCUUGA 3'
           ::  :.:.::::::::::
query   3' CCCUCUAGUACUCGAACU 5'

query=JS16_t1397031, target=FGSG_06965, score=4, range=1062-1079, strand=1

target  5' CGAAGAUGAUGAGCUUGA 3'
            : :::: ::::::::::
query   3' CCCUCUAGUACUCGAACU 5'

query=JS16_t1397031, target=FGSG_12354, score=4, range=779-796, strand=1

target  5' AGAAGAUCAUGAGAUUGA 3'
            : :::::::::: ::::
query   3' CCCUCUAGUACUCGAACU 5'

query=JS16_t1397031, target=FGSG_02230, score=4, range=567-584, strand=1

target  5' AGAAGAUCAUGAGCUGGA 3'
            : ::::::::::::: ::
query   3' CCCUCUAGUACUCGAACU 5'

query=JS16_t1397031, target=FGSG_01738, score=4, range=1958-1976, strand=1

target  5' CGGAGAAUCAUCAGCUUGA 3'
            :::: ::::: :::::::
query   3' CCCUC-UAGUACUCGAACU 5'

query=JS16_t1397031, target=FGSG_07472, score=4, range=194-212, strand=1

target  5' UGGAGCUCCAUGAGCUUGA 3'
            :::: : :::::::::::
query   3' CCCUCUA-GUACUCGAACU 5'

query=JS16_t1397031, target=FGSG_13489, score=4, range=531-548, strand=1

target  5' GAGCGAUUAUGAGCUUGU 3'
           : : :::.:::::::::
query   3' CCCUCUAGUACUCGAACU 5'

query=JS16_t1397031, target=FGSG_06016, score=4, range=2554-2570, strand=1

target  5' GGUAG-UCAUGAGUUUGA 3'
           :: :: :::::::.::::
query   3' CCCUCUAGUACUCGAACU 5'
```

```
query   3’ ACGGUGUACCAUCCCAUCGG 5’

query=JS16_t0219690, target=FGSG_00430, score=4, range=1738-1757, strand=-1

target  5’ UGGCGCAUGAUAGGGUAGCU 3’
           :: :.:::: :::::::::.
query   3’ ACGGUGUACCAUCCCAUCGG 5’

query=JS16_t0219690, target=FGSG_00634, score=4, range=16-34, strand=-1

target  5’ UGCCAU-UGGCAGGGUAGCU 3’
           :::::. ::: ::::::::.
query   3’ ACGGUGUACCAUCCCAUCGG 5’

query=JS16_t1332962, target=FGSG_04321, score=3.5, range=1282-1302, strand=1

target  5’ GGUCAGAUUACGCAAGUUGAG 3’
           :.:: : :::::::::::.::
query   3’ CUAGGCCAAUGCGUUCAAUUC 5’

query=JS16_t1332962, target=FGSG_12863, score=4, range=5869-5889, strand=-1

target  5’ GAUGGGGUUACGUAAGUUGAG 3’
           :::  :::::::.:::::.::
query   3’ CUAGGCCAAUGCGUUCAAUUC 5’

query=JS16_t1332999, target=FGSG_11304, score=3.5, range=934-952, strand=1

target  5’ GUUGCCAUUACCGAGCUUG 3’
           .::: :::::::::.:::
query   3’ UAACUGUAAUGGCUUGAAA 5’

query=JS16_t1332999, target=FGSG_01497, score=3.5, range=5029-5046, strand=1

target  5’ AGUGGCAUUACCG-ACUUU 3’
           : ::.:::::::: :::::
query   3’ UAACUGUAAUGGCUUGAAA 5’

query=JS16_t1332999, target=FGSG_08289, score=4, range=418-436, strand=1

target  5’ UUUGACAUUGCCGAAUUUC 3’
            ::::::::.:::::.::
query   3’ UAACUGUAAUGGCUUGAAA 5’

query=JS16_t1332999, target=FGSG_05671, score=4, range=853-871, strand=1

target  5’ AUCGACAUUAACGAGCUUU 3’
           :: ::::::: :::.::::
query   3’ UAACUGUAAUGGCUUGAAA 5’

query=JS16_t1332999, target=FGSG_00707, score=4, range=229-246, strand=1

target  5’ AUUGAGGUUACCG-ACUUU 3’
           ::::: .:::::: :::::
query   3’ UAACUGUAAUGGCUUGAAA 5’

query=JS16_t1332999, target=FGSG_07454, score=4, range=82-99, strand=1

target  5’ UUUGACAUUACC-AACUUC 3’
            ::::::::::: :::::
query   3’ UAACUGUAAUGGCUUGAAA 5’

query=JS16_t1332999, target=FGSG_07644, score=4, range=580-598, strand=1

target  5’ AGUCAGAUUGCCGAACUUU 3’
           : : : :::.:::::::::
query   3’ UAACUGUAAUGGCUUGAAA 5’

query=JS16_t1332999, target=FGSG_02793, score=4, range=285-303, strand=-1

target  5’ AUUGGUAUUAUCGGACUUC 3’
           ::::..::::.::.::::
query   3’ UAACUGUAAUGGCUUGAAA 5’

query=JS16_t1332999, target=FGSG_06643, score=4, range=459-477, strand=-1

target  5’ AUUGCCAUUACCGGCCUUU 3’
           :::: ::::::::. ::::
query   3’ UAACUGUAAUGGCUUGAAA 5’

query=JS16_t1333074, target=FGSG_04763, score=2.5, range=182-199, strand=1

target  5’ UUCGAAAAGUCAACGUGG 3’
            .:: :::::::::::::
query   3’ UGGCGUUUCAGUUGCACC 5’

query=JS16_t1333074, target=FGSG_06303, score=3, range=528-544, strand=1

target  5’ ACCGC-AGGUCAACGUGG 3’
           ::::: :.::::::::::
query   3’ UGGCGUUUCAGUUGCACC 5’

query=JS16_t1397031, target=FGSG_01579, score=4, range=201-217, strand=1

target  5’ GGGUGGUCAUGAGC-UGA 3’
           ::: :.:::::::: :::
query   3’ CCCUCUAGUACUCGAACU 5’

query=JS16_t1397031, target=FGSG_11188, score=4, range=38-56, strand=1

target  5’ CACAGUAUCAUGAGCUUGA 3’
              :: :::::::::::::
query   3’ CCCUC-UAGUACUCGAACU 5’

query=JS16_t1397031, target=FGSG_00015, score=4, range=826-842, strand=1

target  5’ AAGAGAUCAUG-GCUUGA 3’
             ::::::::: ::::::
query   3’ CCCUCUAGUACUCGAACU 5’

query=JS16_t1397031, target=FGSG_01820, score=4, range=10-26, strand=1

target  5’ GUGA-ACCAUGAGCUUGA 3’
           : :: : ::::::::::
query   3’ CCCUCUAGUACUCGAACU 5’

query=JS16_t1397031, target=FGSG_10021, score=4, range=1162-1178, strand=1

target  5’ AGGAAAU-AUGAGCUUGA 3’
            ::: :: ::::::::::
query   3’ CCCUCUAGUACUCGAACU 5’

query=JS16_t1397031, target=FGSG_07143, score=4, range=1424-1442, strand=1

target  5’ GUGAGCUCGAUGAGCUUGA 3’
           : ::: :: ::::::::::
query   3’ CCCUCUAG-UACUCGAACU 5’

query=JS16_t1397031, target=FGSG_07631, score=4, range=869-886, strand=-1

target  5’ GGGAGAUCAAGAGCAUGA 3’
           ::::::::: :::: :::
query   3’ CCCUCUAGUACUCGAACU 5’

query=JS16_t1397031, target=FGSG_02288, score=4, range=428-445, strand=-1

target  5’ GGGAAAACAUGGGCUUGA 3’
           :::: : ::::.::::::
query   3’ CCCUCUAGUACUCGAACU 5’

query=JS16_t1397031, target=FGSG_00776, score=4, range=206-223, strand=-1

target  5’ GGUAGAUCAUGGGCAUGA 3’
           :: ::::::::.:: :::
query   3’ CCCUCUAGUACUCGAACU 5’

query=JS16_t1397031, target=FGSG_01336, score=4, range=1224-1241, strand=-1

target  5’ AAGAGAUCAAGAGCUUGA 3’
             ::::::: ::::::::
query   3’ CCCUCUAGUACUCGAACU 5’

query=JS16_t1397031, target=FGSG_01575, score=4, range=1538-1555, strand=-1

target  5’ CGGAGCUCAUGAGCUUGU 3’
            :::: :::::::::::
query   3’ CCCUCUAGUACUCGAACU 5’

query=JS16_t1397031, target=FGSG_08055, score=4, range=1031-1048, strand=-1

target  5’ GGCGGAUCAUGUGCUUGG 3’
           :: .::::::: :::::.
query   3’ CCCUCUAGUACUCGAACU 5’

query=JS16_t1397031, target=FGSG_06575, score=4, range=332-349, strand=-1

target  5’ GAGGGAUGAUGAGCUUGG 3’
           : :.::: :::::::::.
query   3’ CCCUCUAGUACUCGAACU 5’

query=JS16_t1397031, target=FGSG_14036, score=4, range=779-796, strand=-1

target  5’ GGUUGAUCAUGGGCUUGU 3’
           ::  :::::::.:::::
query   3’ CCCUCUAGUACUCGAACU 5’

query=JS16_t1397031, target=FGSG_08806, score=4, range=749-766, strand=-1

target  5’ CAAACAUCAUGAGCUUGA 3’
              : :::::::::::::
query   3’ CCCUCUAGUACUCGAACU 5’

query=JS16_t1397031, target=FGSG_07263, score=4, range=1391-1407, strand=-1
```

```
query=JS16_t1333074, target=FGSG_02403, score=3, range=163-181, strand=1

target  5’ UUUGCCAAAGUCAACGUGG 3’
            ..: ::::::::::::::
query   3’ UGGC-GUUUCAGUUGCACC 5’

query=JS16_t1333074, target=FGSG_06225, score=3, range=810-826, strand=1

target  5’ ACCG-AAAGUCAUCGUGG 3’
           :::: ::::::: :::::
query   3’ UGGCGUUUCAGUUGCACC 5’

query=JS16_t1333074, target=FGSG_09151, score=3, range=959-976, strand=-1

target  5’ ACAACAAAGUCAGCGUGG 3’
           ::  ::::::::.:::::
query   3’ UGGCGUUUCAGUUGCACC 5’

query=JS16_t1333074, target=FGSG_02198, score=3.5, range=478-495, strand=1

target  5’ AUGGCGAAGUCAAUGUGG 3’
           :.  ::.:::::::.::::
query   3’ UGGCGUUUCAGUUGCACC 5’

query=JS16_t1333074, target=FGSG_00222, score=3.5, range=608-625, strand=1

target  5’ UCCGUAAAGUCAAGGUGG 3’
            :::.::::::::: ::::
query   3’ UGGCGUUUCAGUUGCACC 5’

query=JS16_t1333074, target=FGSG_00664, score=3.5, range=515-532, strand=1

target  5’ CUCCCAAAGUCGACGUGG 3’
            .: :::::::.::::::
query   3’ UGGCGUUUCAGUUGCACC 5’

query=JS16_t1333074, target=FGSG_03946, score=3.5, range=163-181, strand=1

target  5’ UUCGUCAAAGUCGACGUGG 3’
            .:: :::::::.::::::
query   3’ UGGC-GUUUCAGUUGCACC 5’

query=JS16_t1333074, target=FGSG_05442, score=3.5, range=1171-1189, strand=1

target  5’ AUGCGCAAAGUCAACUUGG 3’
           :.  :::::::::::: :::
query   3’ UG-GCGUUUCAGUUGCACC 5’

query=JS16_t1333074, target=FGSG_06490, score=3.5, range=110-127, strand=-1

target  5’ ACUGCAAAGUCCAUGUGG 3’
           ::.::::::::: :.::::
query   3’ UGGCGUUUCAGUUGCACC 5’

query=JS16_t1333074, target=FGSG_00273, score=3.5, range=476-494, strand=-1

target  5’ ACUGCAAAGUCAACGGUGC 3’
           ::.:::::::::::: :::
query   3’ UGGCGUUUCAGUUG-CACC 5’

query=JS16_t1333074, target=FGSG_05589, score=3.5, range=1043-1060, strand=-1

target  5’ GCAGCAGAGUCAACGUGA 3’
           .: :::.:::::::::::
query   3’ UGGCGUUUCAGUUGCACC 5’

query=JS16_t1333074, target=FGSG_04363, score=3.5, range=36-54, strand=-1

target  5’ AUCGCCAAAGUCAACGUCG 3’
           :.:: :::::::::::: :
query   3’ UGGC-GUUUCAGUUGCACC 5’

query=JS16_t1333074, target=FGSG_03260, score=3.5, range=1495-1511, strand=-1

target  5’ ACCUUAAAGUCAAC-UGG 3’
           ::: .::::::::: :::
query   3’ UGGCGUUUCAGUUGCACC 5’

query=JS16_t1333074, target=FGSG_02362, score=4, range=176-193, strand=1

target  5’ UCCGCAAAGCCAACGUGA 3’
            :::::::: :::::::
query   3’ UGGCGUUUCAGUUGCACC 5’

query=JS16_t1333074, target=FGSG_05337, score=4, range=5449-5467, strand=1

target  5’ GCUGCCAAGAUCAACGUGG 3’
           .:.:: ::: :::::::::
query   3’ UGGCGUUUC-AGUUGCACC 5’

query=JS16_t1333074, target=FGSG_05781, score=4, range=826-844, strand=1

target  5’ AGGA-AUCAUGAGUUUGU 3’
            ::: ::::::::.:::
query   3’ CCCUCUAGUACUCGAACU 5’

query=JS16_t1397031, target=FGSG_03783, score=4, range=1797-1814, strand=-1

target  5’ AGCAUAUCAUGAGCUUGU 3’
            : : ::::::::::::
query   3’ CCCUCUAGUACUCGAACU 5’

query=JS16_t1397031, target=FGSG_05871, score=4, range=55-71, strand=-1

target  5’ UGGAAAUCAUGAG-UUGA 3’
            ::: :::::::: ::::
query   3’ CCCUCUAGUACUCGAACU 5’

query=JS16_t1397031, target=FGSG_05056, score=4, range=192-208, strand=-1

target  5’ GCGA-AACAUGAGCUUGA 3’
           : :: : :::::::::::
query   3’ CCCUCUAGUACUCGAACU 5’

query=JS16_t0241278, target=FGSG_05488, score=4, range=364-384, strand=1

target  5’ GAUUUGGACCCCGGUUUUGAG 3’
           ::::::::::::.: .:::::
query   3’ CUAAACCUGGGGUC-GAACUC 5’

query=JS16_t0241278, target=FGSG_13147, score=4, range=172-191, strand=-1

target  5’ GAUUUGCGCCCCAUCUUGAG 3’
           :::::: .::::: ::::::
query   3’ CUAAACCUGGGGUCGAACUC 5’

No results for JS16_t0307722
No results for JS16_t1397089
No results for JS16_t0255518
No results for JS16_t0269720
query=JS16_t2293132, target=FGSG_04240, score=4, range=347-367, strand=-1

target  5’ CGAAGUAAGGAGUGACCA-UGA 3’
            :::..:::::::::::: :::
query   3’ ACUUUGUUCCUCACUGGUAACU 5’

query=JS16_t0229051, target=FGSG_13407, score=3, range=629-649, strand=1

target  5’ AAUUCCUAGGCCAACGAACAU 3’
            : ::::::.:::::::::::
query   3’ AUUAGGAUCUGGUUGCUUGUA 5’

No results for JS16_t2293231
query=JS16_t1397239, target=FGSG_11750, score=2.5, range=183-201, strand=-1

target  5’ CA-UAUGCCUGUGCAGAACG 3’
           :: ::::::::::::.:::.
query   3’ GUCAUACGGACACGUUUUGU 5’

query=JS16_t1397239, target=FGSG_10185, score=4, range=965-985, strand=1

target  5’ GAGAAUGGCUUGUGCAAAACA 3’
            :: :: ::.:::::::::::
query   3’ GUCAUA-CGGACACGUUUUGU 5’

query=JS16_t1397239, target=FGSG_05738, score=4, range=256-275, strand=-1

target  5’ UAGUAAGCCUGUGCAGGACG 3’
           .:::: :::::::::..::.
query   3’ GUCAUACGGACACGUUUUGU 5’

query=JS16_t1397239, target=FGSG_06442, score=4, range=1069-1088, strand=-1

target  5’ AAGUAUGCCUGUGCCAGACA 3’
            ::::::::::::: :.:::
query   3’ GUCAUACGGACACGUUUUGU 5’

No results for JS16_t2293254
No results for JS16_t2293257
No results for JS16_t3189538
query=JS16_t3189554, target=FGSG_12668, score=2.5, range=1470-1488, strand=1

target  5’ CGACGAGGAGCUCAAUUCU 3’
            .: ::::::::::::::
query   3’ UUUACUCCUCGAGUUAAGA 5’

query=JS16_t3189554, target=FGSG_06762, score=3.5, range=2872-2890, strand=-1

target  5’ AAGUGAGGAGCUCCAUUCG 3’
           ::.:::::::::: ::::
query   3’ UUUACUCCUCGAGUUAAGA 5’

query=JS16_t3189554, target=FGSG_13397, score=4, range=587-606, strand=1
```

```
target  5’ GCUAGCAAAGUCAACUUGG 3’
           .:. ::::::::::: :::
query   3’ UGG-CGUUUCAGUUGCACC 5’

query=JS16_t1333074, target=FGSG_10171, score=4, range=302-320, strand=1

target  5’ ACGGCAAAGUCAAGCGUGA 3’
           :: :::::::::: ::::
query   3’ UGGCGUUUCAGUU-GCACC 5’

query=JS16_t1333074, target=FGSG_07974, score=4, range=367-385, strand=1

target  5’ ACCAUCAAGGUCAACGUGA 3’
           :::  :::.:::::::::
query   3’ UGG-CGUUUCAGUUGCACC 5’

query=JS16_t1333074, target=FGSG_00450, score=4, range=387-403, strand=1

target  5’ ACC-CAAGGUCAACGAGG 3’
           ::: :::.::::::: ::
query   3’ UGGCGUUUCAGUUGCACC 5’

query=JS16_t1333074, target=FGSG_05152, score=4, range=2523-2540, strand=-1

target  5’ GCUGCAGAGUCAAGGUGG 3’
           .:.:::.:::::: ::::
query   3’ UGGCGUUUCAGUUGCACC 5’

query=JS16_t1333074, target=FGSG_02237, score=4, range=1929-1946, strand=-1

target  5’ GCUGCAAAGGCGACGUGG 3’
           .:.:::::: :.::::::
query   3’ UGGCGUUUCAGUUGCACC 5’

query=JS16_t1333074, target=FGSG_06775, score=4, range=882-899, strand=-1

target  5’ GCCAUGAAGUCAGCGUGG 3’
           .:: ..::::::.:::::
query   3’ UGGCGUUUCAGUUGCACC 5’

query=JS16_t1333074, target=FGSG_05189, score=4, range=373-389, strand=-1

target  5’ ACCG-AAAGUUGGCGUGG 3’
           :::: :::::...:::::
query   3’ UGGCGUUUCAGUUGCACC 5’

query=JS16_t1333074, target=FGSG_11855, score=4, range=51-69, strand=-1

target  5’ CUCGAUAAGGUCAACGUGG 3’
            .:: .::.::::::::::
query   3’ UGGC-GUUUCAGUUGCACC 5’

query=JS16_t1333074, target=FGSG_06511, score=4, range=2787-2804, strand=-1

target  5’ AAGGCAGAGUCAAUGUGG 3’
           :  :::.::::::.::::
query   3’ UGGCGUUUCAGUUGCACC 5’

query=JS16_t1333074, target=FGSG_00192, score=4, range=600-617, strand=-1

target  5’ CCCUCAAAGUCGACGUGC 3’
            :: :::::::.:::::
query   3’ UGGCGUUUCAGUUGCACC 5’

query=JS16_t1333074, target=FGSG_05297, score=4, range=2538-2555, strand=-1

target  5’ AGAGCAAAGUCAAUGUGC 3’
           :  ::::::::::.:::
query   3’ UGGCGUUUCAGUUGCACC 5’

query=JS16_t1333074, target=FGSG_06495, score=4, range=504-521, strand=-1

target  5’ ACGACAAAGUCAACUUGG 3’
           ::  :::::::::: :::
query   3’ UGGCGUUUCAGUUGCACC 5’

No results for JS16_t0685284
query=JS16_t4022326, target=FGSG_01196, score=3.5, range=440-459, strand=1

target  5’ CCUUCUACGAUCGGAAGAAG 3’
            :.::: :::::::::.:::
query   3’ CGGAGAAGCUAGCCUUUUUC 5’

query=JS16_t4022326, target=FGSG_09699, score=4, range=756-775, strand=1

target  5’ AGAUCUUCGAUCGGAAGAAG 3’
              :::::::::::::.:::
query   3’ CGGAGAAGCUAGCCUUUUUC 5’

query=JS16_t4022326, target=FGSG_02681, score=4, range=265-283, strand=1

target  5’ GCCU-UCCGAACGGAAAAAG 3’

target  5’ AAAUCAGGAGUUCAAUCUCU 3’
           :::: :::::.::::: :::
query   3’ UUUACUCCUCGAGUUA-AGA 5’

query=JS16_t3189554, target=FGSG_01215, score=4, range=560-579, strand=1

target  5’ UCAUGAAGGAGCUCGAUUCU 3’
             ::: ::::::::.:::::
query   3’ UUUAC-UCCUCGAGUUAAGA 5’

query=JS16_t3189554, target=FGSG_12743, score=4, range=369-387, strand=-1

target  5’ CGGUGAGGAGCUUAAUUCC 3’
            ..:::::::::.:::::
query   3’ UUUACUCCUCGAGUUAAGA 5’

query=JS16_t3189554, target=FGSG_07005, score=4, range=372-390, strand=-1

target  5’ AAGUGGGGAGGUCAAUUCC 3’
           ::.::.:::: :::::::
query   3’ UUUACUCCUCGAGUUAAGA 5’

query=JS16_t3189554, target=FGSG_05771, score=4, range=898-916, strand=-1

target  5’ GAGUUUGGAGCUCAAUUCG 3’
           .:.:  ::::::::::::
query   3’ UUUACUCCUCGAGUUAAGA 5’

query=JS16_t3189554, target=FGSG_08522, score=4, range=1841-1858, strand=-1

target  5’ AGGUCAGGAGCUC-AUUCU 3’
           :..: :::::::: :::::
query   3’ UUUACUCCUCGAGUUAAGA 5’

No results for JS16_t0475372
No results for JS16_t2293321
No results for JS16_t3189611
query=JS16_t0367036, target=FGSG_13130, score=4, range=711-731, strand=-1

target  5’ CCUCUUGCGCCAUAGCUAUGA 3’
             ::::::::::::::.::.:
query   3’ CAAGAACGCGGUAUCGGUAUU 5’

query=JS16_t0367036, target=FGSG_07454, score=4, range=1389-1409, strand=-1

target  5’ AUACUUCCGCCAUGGCCAUAA 3’
            : ::: ::::::.:::::::
query   3’ CAAGAACGCGGUAUCGGUAUU 5’

No results for JS16_t0475380
No results for JS16_t3189682
No results for JS16_t0802568
query=JS16_t3189827, target=FGSG_10030, score=4, range=591-610, strand=1

target  5’ UGCCUU-UACGGCAACAGGCA 3’
            : ::: :::::::::::::
query   3’ CCAGAACAUGCCGUUGUCCGG 5’

query=JS16_t3189827, target=FGSG_04138, score=4, range=300-319, strand=-1

target  5’ GGUCUGGU-CGGUAACAGGCC 3’
           ::::: :: :::.::::::::
query   3’ CCAGAACAUGCCGUUGUCCGG 5’

No results for JS16_t1397606
query=JS16_t3189908, target=FGSG_05408, score=3.5, range=873-892, strand=1

target  5’ GUACAAGAA-UUCUCGUAUCG 3’
           . ::::::: ::::::::::
query   3’ UUUGUUCUUCAAGAGCAUAGC 5’

query=JS16_t3189908, target=FGSG_07497, score=4, range=2160-2179, strand=1

target  5’ AGGCAACAAGUUCUCGU-UCG 3’
           :..::: :::::::::: :::
query   3’ UUUGUUCUUCAAGAGCAUAGC 5’

No results for JS16_t1397636
No results for JS16_t1397649
query=JS16_t1397674, target=FGSG_11631, score=4, range=1426-1446, strand=1

target  5’ AACAGGCA-ACUUCUUAACACU 3’
           ::::: :: ::..:::::::::
query   3’ UUGUCGGUGUGGGGAAUUGUGA 5’

No results for JS16_t0396359
No results for JS16_t1397693
query=JS16_t0475426, target=FGSG_06589, score=3.5, range=830-850, strand=-1

target  5’ AAC-GUUUGUACGCUUUGACUA 3’
           ::: :.:::::::: :::::::
```

```
          :::: : ::: :::::::::
query   3' CGGAGAAGCUAGCCUUUUUC 5'

No results for JS16_t3125796
query=JS16_t0283121, target=FGSG_00205, score=2, range=3807-3825, strand=1

target  5' UACGACCAGUCGGAACUGU 3'
          :::::::::: ::::::::
query   3' AUGCUGGUCA-CCUUGACA 5'

query=JS16_t0283121, target=FGSG_05407, score=2, range=751-768, strand=1

target  5' UACCCCCAGUGGAACUGU 3'
          :::  :::::::::::::
query   3' AUGCUGGUCACCUUGACA 5'

query=JS16_t0283121, target=FGSG_01909, score=2, range=585-602, strand=-1

target  5' UACCACCAGUGGAACUGC 3'
          ::: :::::::::::::
query   3' AUGCUGGUCACCUUGACA 5'

query=JS16_t0283121, target=FGSG_11507, score=3, range=1718-1735, strand=-1

target  5' GACCACCAGUGGAACUGC 3'
           :: :::::::::::::
query   3' AUGCUGGUCACCUUGACA 5'

query=JS16_t0283121, target=FGSG_01378, score=3.5, range=2857-2873, strand=1

target  5' UG-GAACAGUGGAACUGU 3'
          :. :: ::::::::::::
query   3' AUGCUGGUCACCUUGACA 5'

query=JS16_t0283121, target=FGSG_13249, score=3.5, range=267-283, strand=1

target  5' UAUCA-CAGUGGAACUGU 3'
          ::. : ::::::::::::
query   3' AUGCUGGUCACCUUGACA 5'

query=JS16_t0283121, target=FGSG_10111, score=3.5, range=3665-3682, strand=-1

target  5' UAUGAUCAGUGGUACUGU 3'
          ::.::.:::::: :::::
query   3' AUGCUGGUCACCUUGACA 5'

query=JS16_t0283121, target=FGSG_03697, score=4, range=759-776, strand=1

target  5' UAUGGCCAGUGGAGUUGG 3'
          ::.:.::::::::..::
query   3' AUGCUGGUCACCUUGACA 5'

query=JS16_t0283121, target=FGSG_04276, score=4, range=1587-1604, strand=1

target  5' UAUGGCCAGUGAAGCUGU 3'
          ::.:.:::::: :.::::
query   3' AUGCUGGUCACCUUGACA 5'

query=JS16_t0283121, target=FGSG_03275, score=4, range=1093-1110, strand=1

target  5' AACGAUCAGUGGGACUGG 3'
           ::::.::::::.::::
query   3' AUGCUGGUCACCUUGACA 5'

query=JS16_t0283121, target=FGSG_05299, score=4, range=225-242, strand=1

target  5' UACAACUGGUGGAACUGG 3'
          ::: ::..:::::::::
query   3' AUGCUGGUCACCUUGACA 5'

query=JS16_t0283121, target=FGSG_06089, score=4, range=2546-2563, strand=1

target  5' UACGAACACUGGAACUGU 3'
          ::::: :: :::::::::
query   3' AUGCUGGUCACCUUGACA 5'

query=JS16_t0283121, target=FGSG_04127, score=4, range=2149-2166, strand=1

target  5' UACGCCAAGUGGAACUGG 3'
          :::: : ::::::::::
query   3' AUGCUGGUCACCUUGACA 5'

query=JS16_t0283121, target=FGSG_06717, score=4, range=812-828, strand=1

target  5' UGU-ACCAGUGGAGCUGC 3'
          :.. :::::::::.:::
query   3' AUGCUGGUCACCUUGACA 5'

query=JS16_t0283121, target=FGSG_03219, score=4, range=436-452, strand=1

target  5' UACG-CCGCUGGAACUGU 3'
          :::: ::. :::::::::

query   3' UUGACGAACAUGCGUAACUGAU 5'

query=JS16_t0475426, target=FGSG_04537, score=4, range=1047-1069, strand=1

target  5' GAAUGCAUUUUACGCAUUGACUG 3'
          .: ::: :: ::::::::::::.
query   3' UUGACG-AACAUGCGUAACUGAU 5'

query=JS16_t1397767, target=FGSG_07267, score=4, range=544-563, strand=-1

target  5' GGACGUU-CGUGAAUGAGUGG 3'
          :.:::.: ::::::::::: :
query   3' CUUGCGACGCACUUACUCAGC 5'

query=JS16_t1397767, target=FGSG_05455, score=4, range=429-448, strand=-1

target  5' GAAGGCU-CGUGAAUGGGUCC 3'
          ::: ::: ::::::::.:::
query   3' CUUGCGACGCACUUACUCAGC 5'

query=JS16_t0584365, target=FGSG_04830, score=3, range=962-981, strand=-1

target  5' ACGAUCUCUUUGUCUGUGAA 3'
          : ::: :::::::::::::
query   3' UUCUAUAGAAACAGACACUC 5'

query=JS16_t0584365, target=FGSG_09129, score=4, range=1663-1682, strand=-1

target  5' AUGAUAUCUUUGAUUGUGAG 3'
          : :::::::::: .::::::
query   3' UUCUAUAGAAACAGACACUC 5'

query=JS16_t0584365, target=FGSG_07962, score=4, range=2105-2125, strand=-1

target  5' AAGACAAGCUUUGUUUGUGAG 3'
          ::::  : ::::::.::::::
query   3' UUCU-AUAGAAACAGACACUC 5'

query=JS16_t0693021, target=FGSG_10695, score=2.5, range=622-638, strand=-1

target  5' GCUUUCGC-AUCUUUUGG 3'
          :::::::: ::::::::.
query   3' CGAAAGCGCUAGAAAACU 5'

query=JS16_t0693021, target=FGSG_12490, score=3, range=1026-1043, strand=-1

target  5' GCUUUUGCGAUAUUUUGA 3'
          :::::.::::: ::::::
query   3' CGAAAGCGCUAGAAAACU 5'

query=JS16_t0693021, target=FGSG_10995, score=3, range=1428-1444, strand=-1

target  5' GCUCUCGC-AUCUUUUGA 3'
          ::: :::: :::::::::
query   3' CGAAAGCGCUAGAAAACU 5'

query=JS16_t0693021, target=FGSG_04421, score=3.5, range=469-487, strand=1

target  5' GCUGGUUGCGAUCUUUUGG 3'
          :::  :.:::::::::::.
query   3' CGA-AAGCGCUAGAAAACU 5'

query=JS16_t0693021, target=FGSG_06355, score=4, range=98-116, strand=1

target  5' GCUUUCGCGACUUUUUUGC 3'
          :::::::::: :.:::::
query   3' CGAAAGCGCU-AGAAAACU 5'

query=JS16_t0693021, target=FGSG_12534, score=4, range=752-769, strand=1

target  5' GCUUUCGCGAUCGCUUGA 3'
          ::::::::::::  ::::
query   3' CGAAAGCGCUAGAAAACU 5'

query=JS16_t0693021, target=FGSG_13866, score=4, range=710-727, strand=1

target  5' GCUUCCGCCGUCUUUUGA 3'
          :::: ::: .::::::::
query   3' CGAAAGCGCUAGAAAACU 5'

query=JS16_t0693021, target=FGSG_04764, score=4, range=1627-1644, strand=1

target  5' GCUCUUGCGAUCUUUGGA 3'
          ::: :.:::::::::: ::
query   3' CGAAAGCGCUAGAAAACU 5'

query=JS16_t0693021, target=FGSG_02934, score=4, range=1392-1409, strand=1

target  5' GCCUUUGCGAUCUGUUGA 3'
          :: ::.::::::: ::::
query   3' CGAAAGCGCUAGAAAACU 5'
```

```
query   3’ AUGCUGGUCACCUUGACA 5’

query=JS16_t0283121, target=FGSG_11026, score=4, range=7448-7466, strand=1

target  5’ UAUCUACCAGUGGCACUGU 3’
           :: : :::::::: :::::
query   3’ AU-GCUGGUCACCUUGACA 5’

query=JS16_t0283121, target=FGSG_10511, score=4, range=2085-2101, strand=1

target  5’ CACUACCAGUGGAA-UGU 3’
            :: :::::::::: :::
query   3’ AUGCUGGUCACCUUGACA 5’

query=JS16_t0283121, target=FGSG_00586, score=4, range=685-701, strand=1

target  5’ GACG-CCACUGGAACUGU 3’
            ::: ::: :::::::::
query   3’ AUGCUGGUCACCUUGACA 5’

query=JS16_t0283121, target=FGSG_06289, score=4, range=778-794, strand=1

target  5’ CACGUCCAGUGG-ACUGU 3’
            ::: ::::::: :::::
query   3’ AUGCUGGUCACCUUGACA 5’

query=JS16_t0283121, target=FGSG_01794, score=4, range=258-274, strand=1

target  5’ CAAGACC-GUGGAACUGU 3’
            : :::: ::::::::::
query   3’ AUGCUGGUCACCUUGACA 5’

query=JS16_t0283121, target=FGSG_09293, score=4, range=725-742, strand=-1

target  5’ CACCACCACUGGAACUGU 3’
            :: :::: :::::::::
query   3’ AUGCUGGUCACCUUGACA 5’

query=JS16_t0283121, target=FGSG_12215, score=4, range=459-477, strand=-1

target  5’ UACCACCCAGCGGAACUGU 3’
           ::: : :::: ::::::::
query   3’ AUGCU-GGUCACCUUGACA 5’

No results for JS16_t3125817
query=JS16_t4022368, target=FGSG_09040, score=2, range=94-112, strand=1

target  5’ GGAUACUUGCCUUUUUCUC 3’
           :::: :::::::::.::::
query   3’ CCUAGGAACGGAAAGAGAG 5’

query=JS16_t4022368, target=FGSG_06954, score=2.5, range=1173-1191, strand=-1

target  5’ GGUUCUUUGCCUUUCUCUG 3’
           :: ::.:::::::::::
query   3’ CCUAGGAACGGAAAGAGAG 5’

query=JS16_t4022368, target=FGSG_01105, score=2.5, range=541-558, strand=-1

target  5’ GGA-GCUUGCCUUUCUCUU 3’
           :::  ::::::::::::.
query   3’ CCUAGGAACGGAAAGAGAG 5’

query=JS16_t4022368, target=FGSG_01680, score=3, range=4104-4122, strand=1

target  5’ CGACCCUUGUCUUUCUCUC 3’
            :: :::::.:::::::::
query   3’ CCUAGGAACGGAAAGAGAG 5’

query=JS16_t4022368, target=FGSG_07700, score=3, range=1147-1164, strand=1

target  5’ GGA-CCUUGUCUUUCUCUA 3’
           ::: :::::.::::::::
query   3’ CCUAGGAACGGAAAGAGAG 5’

query=JS16_t4022368, target=FGSG_00152, score=3, range=263-281, strand=1

target  5’ UGCUACUUGCCUUUCUCUC 3’
            : : ::::::::::::::
query   3’ CCUAGGAACGGAAAGAGAG 5’

query=JS16_t4022368, target=FGSG_08852, score=3.5, range=562-580, strand=1

target  5’ GGAUCUUUGCCUUCCUUUC 3’
           :::::.::::::: ::.::
query   3’ CCUAGGAACGGAAAGAGAG 5’

query=JS16_t4022368, target=FGSG_07848, score=3.5, range=655-673, strand=-1

target  5’ UGGUUUUUGCCUUUUUCUC 3’
            :.:..::::::::.::::
query   3’ CCUAGGAACGGAAAGAGAG 5’

query=JS16_t0693021, target=FGSG_08042, score=4, range=905-923, strand=1

target  5’ GCUUGUCGGGGUCUUUUGA 3’
           :::: ::: :.::::::::
query   3’ CGAA-AGCGCUAGAAAACU 5’

query=JS16_t0693021, target=FGSG_11672, score=4, range=1448-1465, strand=1

target  5’ AAUUUCUCGAUCUUUUGA 3’
             :::: :::::::::::
query   3’ CGAAAGCGCUAGAAAACU 5’

query=JS16_t0693021, target=FGSG_09178, score=4, range=1121-1138, strand=1

target  5’ GAUUCCGCGAGCUUUUGA 3’
           : :: ::::: :::::::
query   3’ CGAAAGCGCUAGAAAACU 5’

query=JS16_t0693021, target=FGSG_09334, score=4, range=20-38, strand=1

target  5’ GCCCUCGCGAUCCUUUUGA 3’
           ::  ::::::: :::::::
query   3’ CGAAAGCGCUA-GAAAACU 5’

query=JS16_t0693021, target=FGSG_09244, score=4, range=1914-1930, strand=1

target  5’ GCGUUUGCG-UCUUUUGA 3’
           :: ::.::: ::::::::
query   3’ CGAAAGCGCUAGAAAACU 5’

query=JS16_t0693021, target=FGSG_07326, score=4, range=808-826, strand=-1

target  5’ ACUUUCGCGAUUUCUUUGA 3’
            ::::::::::.: :::::
query   3’ CGAAAGCGCUAGA-AAACU 5’

query=JS16_t0693021, target=FGSG_04963, score=4, range=1597-1614, strand=-1

target  5’ GCUGUUGCGAUUUUUUGU 3’
           ::: :.:::::.:::::
query   3’ CGAAAGCGCUAGAAAACU 5’

query=JS16_t0693021, target=FGSG_07680, score=4, range=282-299, strand=-1

target  5’ GGUUUCGUGAUCUGUUGA 3’
           : :::::.::::: ::::
query   3’ CGAAAGCGCUAGAAAACU 5’

query=JS16_t0693021, target=FGSG_11023, score=4, range=125-142, strand=-1

target  5’ GAUAUUGCGAUCUUUUGU 3’
           : : :.:::::::::::
query   3’ CGAAAGCGCUAGAAAACU 5’

query=JS16_t0693021, target=FGSG_04064, score=4, range=239-257, strand=-1

target  5’ GCUGUGGCGAUCUUGUUGA 3’
           ::: : :::::::: ::::
query   3’ CGAAAGCGCUAGAA-AACU 5’

query=JS16_t4086882, target=FGSG_11705, score=4, range=352-371, strand=1

target  5’ GUC-AAAGAAGCCAAGGAGCA 3’
           ::: :  :::.::::::::::
query   3’ CAGAUACCUUUGGUUCCUCGU 5’

No results for JS16_t2293942
No results for JS16_t4086910
query=JS16_t4086939, target=FGSG_05505, score=2.5, range=313-331, strand=-1

target  5’ UUCGUAGAUUCGACCAGCC 3’
           .: ..::::::::::::::
query   3’ GACUGUCUAAGCUGGUCGG 5’

query=JS16_t4086939, target=FGSG_09148, score=2.5, range=1146-1163, strand=-1

target  5’ UUGA-AGAUUCGACCAGCA 3’
           .::: :::::::::::::
query   3’ GACUGUCUAAGCUGGUCGG 5’

query=JS16_t4086939, target=FGSG_10081, score=3, range=251-270, strand=1

target  5’ GGGAUCAGAUUCGACCAGCC 3’
             :: :::::::::::::::
query   3’ GACU-GUCUAAGCUGGUCGG 5’

query=JS16_t4086939, target=FGSG_02305, score=3, range=831-849, strand=-1

target  5’ CUGACGGAUUCUACCAGCU 3’
           :::::.::::: :::::::.
query   3’ GACUGUCUAAGCUGGUCGG 5’
```

```
query=JS16_t4022368, target=FGSG_05227, score=3.5, range=629-647, strand=-1

target  5' CGGUCCUUGCCCUUCUCUC 3'
            :.:::::::: :::::::
query   3' CCUAGGAACGGAAAGAGAG 5'

query=JS16_t4022368, target=FGSG_01463, score=3.5, range=640-658, strand=-1

target  5' CGUUCUUUGCCUUUUUCUC 3'
            : ::.::::::::.::::
query   3' CCUAGGAACGGAAAGAGAG 5'

query=JS16_t4022368, target=FGSG_05230, score=3.5, range=367-384, strand=-1

target  5' GGGUUCUUG-CUUUCUCUU 3'
           ::.:.:::: ::::::::.
query   3' CCUAGGAACGGAAAGAGAG 5'

query=JS16_t4022368, target=FGSG_11339, score=4, range=661-679, strand=1

target  5' UGGUUCAUGCCUUUCUCUC 3'
            :.:.: ::::::::::::
query   3' CCUAGGAACGGAAAGAGAG 5'

query=JS16_t4022368, target=FGSG_05429, score=4, range=256-274, strand=1

target  5' GGCUCCUUGCUCUUCUCUC 3'
           :: :::::::. :::::::
query   3' CCUAGGAACGGAAAGAGAG 5'

query=JS16_t4022368, target=FGSG_07798, score=4, range=5445-5463, strand=1

target  5' AGUUCCUUUCCUUUCUCUC 3'
            : ::::: ::::::::::
query   3' CCUAGGAACGGAAAGAGAG 5'

query=JS16_t4022368, target=FGSG_13860, score=4, range=688-706, strand=1

target  5' GCAGCCUUGCCUUUCUAUC 3'
           : : :::::::::::: ::
query   3' CCUAGGAACGGAAAGAGAG 5'

query=JS16_t4022368, target=FGSG_07644, score=4, range=394-412, strand=-1

target  5' CGAUCUUUGCCUUUAUCUU 3'
            ::::.:::::::: :::.
query   3' CCUAGGAACGGAAAGAGAG 5'

query=JS16_t4022368, target=FGSG_00497, score=4, range=881-899, strand=-1

target  5' GGCUCCUUGCCCUUUUCUC 3'
           :: :::::::: ::.::::
query   3' CCUAGGAACGGAAAGAGAG 5'

query=JS16_t4022368, target=FGSG_02482, score=4, range=2323-2342, strand=-1

target  5' GCGUCCUUGGCCUUUCUCUU 3'
           : .::::: ::::::::::.
query   3' CCUAGGAA-CGGAAAGAGAG 5'

query=JS16_t4022368, target=FGSG_01357, score=4, range=40-57, strand=-1

target  5' UGAU-UUUGUCUUUCUCUU 3'
            ::: .:::.::::::::.
query   3' CCUAGGAACGGAAAGAGAG 5'

query=JS16_t4022368, target=FGSG_09901, score=4, range=529-546, strand=-1

target  5' CGCUCCUUGCCUUU-UCUC 3'
            : ::::::::::: ::::
query   3' CCUAGGAACGGAAAGAGAG 5'

No results for JS16_t3125842
No results for JS16_t4022412
No results for JS16_t1333241
No results for JS16_t3126008
query=JS16_t2229785, target=FGSG_09683, score=4, range=630-650, strand=-1

target  5' GUGGUA-UGAACUUCGUCCUCG 3'
            :::.: ::::::::.:::::.
query   3' GACCGUAACUUGAAGUAGGAGU 5'

query=JS16_t2229785, target=FGSG_06977, score=4, range=2073-2093, strand=-1

target  5' CUGUCGUUGAACUUCAU-CUCG 3'
           ::: :.:::::::::: :::.
query   3' GACCGUAACUUGAAGUAGGAGU 5'

No results for JS16_t0685330
query=JS16_t0794636, target=FGSG_03369, score=3.5, range=1059-1078, strand=-1

query=JS16_t4086939, target=FGSG_06900, score=4, range=1315-1333, strand=1

target  5' UUGAUAGAUUCCACUAGCC 3'
           .:::.:::::: ::.::::
query   3' GACUGUCUAAGCUGGUCGG 5'

query=JS16_t4086939, target=FGSG_02952, score=4, range=1009-1028, strand=1

target  5' CUCGACAGCUUCGACCAGCG 3'
           :: ::::: ::::::::::
query   3' GA-CUGUCUAAGCUGGUCGG 5'

query=JS16_t4086939, target=FGSG_00684, score=4, range=785-804, strand=1

target  5' CUGUCAAGAUUCGACCAGAC 3'
           ::: : :::::::::::: :
query   3' GACUG-UCUAAGCUGGUCGG 5'

query=JS16_t4086939, target=FGSG_02498, score=4, range=947-966, strand=1

target  5' CUAAUGGAUUCGCACCAGCC 3'
           :: :..:::::: :::::::
query   3' GACUGUCUAAGC-UGGUCGG 5'

query=JS16_t4086939, target=FGSG_08119, score=4, range=704-722, strand=-1

target  5' UUGGCAGAUUCGUUCAGCC 3'
           .::.:::::::: .:::::
query   3' GACUGUCUAAGCUGGUCGG 5'

query=JS16_t4086939, target=FGSG_02689, score=4, range=954-971, strand=-1

target  5' UUGAUGGAUUCG-CCAGCU 3'
           .:::..:::::: :::::.
query   3' GACUGUCUAAGCUGGUCGG 5'

query=JS16_t4086939, target=FGSG_01395, score=4, range=884-902, strand=-1

target  5' UUGAAAGAUUCAACCAGCU 3'
           .::: :::::: ::::::.
query   3' GACUGUCUAAGCUGGUCGG 5'

query=JS16_t4086939, target=FGSG_06892, score=4, range=305-323, strand=-1

target  5' CUGUCGCAUUCGACCAGCU 3'
           ::: :. :::::::::::.
query   3' GACUGUCUAAGCUGGUCGG 5'

query=JS16_t4086939, target=FGSG_06334, score=4, range=770-787, strand=-1

target  5' CUG-CAGAUUCCACCAGUC 3'
           ::: ::::::: :::::.:
query   3' GACUGUCUAAGCUGGUCGG 5'

query=JS16_t2294041, target=FGSG_04551, score=4, range=377-395, strand=1

target  5' CUCAA-GUCACUGAUGUCUU 3'
           ::::: ::::::.: :::::
query   3' GAGUUGCAGUGAUUUCAGAA 5'

query=JS16_t2294041, target=FGSG_07535, score=4, range=32-51, strand=-1

target  5' UUCGACGUCACUUAGGUCUU 3'
           .::.::::::::: :.:::::
query   3' GAGUUGCAGUGAUUUCAGAA 5'

query=JS16_t2294041, target=FGSG_08861, score=4, range=653-672, strand=-1

target  5' CUCGGCGUCUUUAAAGUCUU 3'
           :::..:::: .:::::::::
query   3' GAGUUGCAGUGAUUUCAGAA 5'

query=JS16_t2294041, target=FGSG_10381, score=4, range=1775-1794, strand=-1

target  5' GAAAACGUCGCUAAAGUCUU 3'
               ::::::.::::::::::
query   3' GAGUUGCAGUGAUUUCAGAA 5'

query=JS16_t2294041, target=FGSG_12491, score=4, range=116-134, strand=-1

target  5' CUCAACCUCACU-AGGUCUU 3'
           :::::: ::::: :.:::::
query   3' GAGUUGCAGUGAUUUCAGAA 5'

No results for JS16_t2294079
query=JS16_t3190433, target=FGSG_05889, score=1, range=267-284, strand=1

target  5' CAUAGACAACUUCAUCAC 3'
           :::::::::::::::::
query   3' GUAUCUGUUGAAGUAGUC 5'

query=JS16_t3190433, target=FGSG_08891, score=1.5, range=821-839, strand=1
```

```
target  5’ GAAGGCACG-AGUUGAUCAAG 3’
           .:: ::::: :::::::::::
query   3’ UUUACGUGCGUCAACUAGUUC 5’

query=JS16_t0794636, target=FGSG_07731, score=4, range=175-195, strand=1

target  5’ GAAAGCCUGCAGUUGGUCAAG 3’
           .:: :: .:::::::.:::::
query   3’ UUUACGUGCGUCAACUAGUUC 5’

query=JS16_t0794636, target=FGSG_07634, score=4, range=744-763, strand=1

target  5’ GCGUG-ACGCAGUUGGUCAAG 3’
           . .:: :::::::::.:::::
query   3’ UUUACGUGCGUCAACUAGUUC 5’

query=JS16_t0794636, target=FGSG_10971, score=4, range=1157-1176, strand=1

target  5’ CAAUCCACGCAGUUGA-CAAG 3’
            ::: ::::::::::: ::::
query   3’ UUUACGUGCGUCAACUAGUUC 5’

query=JS16_t0794636, target=FGSG_05191, score=4, range=1873-1892, strand=-1

target  5’ AACUGGACG-AGUUGAUCAAG 3’
           :: :: ::: :::::::::::
query   3’ UUUACGUGCGUCAACUAGUUC 5’

query=JS16_t3126111, target=FGSG_13795, score=3, range=343-360, strand=1

target  5’ UACAUGGCUGGAACCCUG 3’
           ::: :::::::: :::::
query   3’ AUGGACCGACCUGGGGAC 5’

query=JS16_t3126111, target=FGSG_05962, score=3, range=336-352, strand=1

target  5’ UGUC-GGCUGGAUCCCUG 3’
           :..: :::::::.:::::
query   3’ AUGGACCGACCUGGGGAC 5’

query=JS16_t3126111, target=FGSG_07178, score=3, range=2178-2194, strand=1

target  5’ UA-CAGGCUGGGCCCCUG 3’
           :: : ::::::.::::::
query   3’ AUGGACCGACCUGGGGAC 5’

query=JS16_t3126111, target=FGSG_13601, score=3, range=50-67, strand=-1

target  5’ UGCUUGGCUGGACCUCUC 3’
           :.:.::::::::::.::
query   3’ AUGGACCGACCUGGGGAC 5’

query=JS16_t3126111, target=FGSG_09802, score=3.5, range=1007-1024, strand=1

target  5’ GUUCUGGCUGGACUCCUG 3’
             .::::::::::.::::
query   3’ AUGGACCGACCUGGGGAC 5’

query=JS16_t3126111, target=FGSG_03680, score=3.5, range=748-765, strand=1

target  5’ UACUGGGUUGGACCCCUU 3’
           :::. ::.:::::::::
query   3’ AUGGACCGACCUGGGGAC 5’

query=JS16_t3126111, target=FGSG_07770, score=3.5, range=99-115, strand=1

target  5’ UAUCUGGC-GGACCCCUU 3’
           ::.::::: ::::::::
query   3’ AUGGACCGACCUGGGGAC 5’

query=JS16_t3126111, target=FGSG_09954, score=3.5, range=483-500, strand=-1

target  5’ UGCACGGCUGGACCCUUG 3’
           :.:  ::::::::::.::
query   3’ AUGGACCGACCUGGGGAC 5’

query=JS16_t3126111, target=FGSG_12039, score=4, range=771-789, strand=1

target  5’ UACCUUGGCUGGACUUCUC 3’
           :::: :::::::::..::
query   3’ AUGG-ACCGACCUGGGGAC 5’

query=JS16_t3126111, target=FGSG_08002, score=4, range=541-559, strand=1

target  5’ UACAGUGGCUGGGCCUCUG 3’
           :::  :::::::.::.:::
query   3’ AUG-GACCGACCUGGGGAC 5’

query=JS16_t3126111, target=FGSG_13489, score=4, range=266-284, strand=1

target  5’ UUCCUGGGCUGGACUCUUG 3’
           : ::: ::::::::.:.::
```

```
target  5’ CAUGGAACAACUUCAUCAG 3’
           :::.: :::::::::::::
query   3’ GUAUC-UGUUGAAGUAGUC 5’

query=JS16_t3190433, target=FGSG_05092, score=2.5, range=2341-2357, strand=1

target  5’ CG-AGAUAACUUCAUCAG 3’
           :. :::.:::::::::::
query   3’ GUAUCUGUUGAAGUAGUC 5’

query=JS16_t3190433, target=FGSG_10948, score=2.5, range=620-637, strand=-1

target  5’ CAUGGACAACUUCACCAG 3’
           :::.:::::::::: :::
query   3’ GUAUCUGUUGAAGUAGUC 5’

query=JS16_t3190433, target=FGSG_04360, score=3, range=21-38, strand=1

target  5’ CAUAAACGGCUUCAUCAG 3’
           :::: ::..:::::::::
query   3’ GUAUCUGUUGAAGUAGUC 5’

query=JS16_t3190433, target=FGSG_05146, score=3, range=110-127, strand=1

target  5’ CAAAGACAACUUCAACAG 3’
           :: ::::::::::: :::
query   3’ GUAUCUGUUGAAGUAGUC 5’

query=JS16_t3190433, target=FGSG_03958, score=3, range=860-877, strand=1

target  5’ CAGAGACAACUUCAGCAG 3’
           :: ::::::::::: :::
query   3’ GUAUCUGUUGAAGUAGUC 5’

query=JS16_t3190433, target=FGSG_04700, score=3, range=1401-1418, strand=1

target  5’ GAUAAACAACUUCAUCAU 3’
            ::: ::::::::::::
query   3’ GUAUCUGUUGAAGUAGUC 5’

query=JS16_t3190433, target=FGSG_02409, score=3, range=422-439, strand=1

target  5’ CUUUGACAACUUCAUCAC 3’
           : : :::::::::::::
query   3’ GUAUCUGUUGAAGUAGUC 5’

query=JS16_t3190433, target=FGSG_01366, score=3, range=281-298, strand=-1

target  5’ UGUAGCCAACUUCAUCAG 3’
           ..::: ::::::::::::
query   3’ GUAUCUGUUGAAGUAGUC 5’

query=JS16_t3190433, target=FGSG_02967, score=3, range=914-931, strand=-1

target  5’ CAAAGACAACUUCGUCAA 3’
           :: ::::::::::.:::
query   3’ GUAUCUGUUGAAGUAGUC 5’

query=JS16_t3190433, target=FGSG_09931, score=3, range=584-600, strand=-1

target  5’ CA-AGACAGCUUCGUCAG 3’
           :: :::::.::::.::::
query   3’ GUAUCUGUUGAAGUAGUC 5’

query=JS16_t3190433, target=FGSG_04610, score=3.5, range=246-263, strand=1

target  5’ CAUGGGCAACUUCAUCGC 3’
           :::.:.::::::::::.
query   3’ GUAUCUGUUGAAGUAGUC 5’

query=JS16_t3190433, target=FGSG_03918, score=3.5, range=162-179, strand=1

target  5’ CAUGGAGAACUUCAUCAC 3’
           :::.:: ::::::::::
query   3’ GUAUCUGUUGAAGUAGUC 5’

query=JS16_t3190433, target=FGSG_03735, score=3.5, range=3520-3537, strand=1

target  5’ CGAAGACUACUUCAUCAG 3’
           :.  :::: ::::::::::
query   3’ GUAUCUGUUGAAGUAGUC 5’

query=JS16_t3190433, target=FGSG_01042, score=3.5, range=1398-1415, strand=1

target  5’ CGUUGACCACUUCAUCAG 3’
           :.: ::: ::::::::::
query   3’ GUAUCUGUUGAAGUAGUC 5’

query=JS16_t3190433, target=FGSG_00555, score=3.5, range=645-662, strand=1

target  5’ CAGGGACCACUUCAUCAG 3’
```

```
query   3’ AUGGA-CCGACCUGGGGAC 5’

query=JS16_t3126111, target=FGSG_06923, score=4, range=1287-1304, strand=1

target  5’ GACGUGGCUGGAUCCCUA 3’
            :: ::::::::.::::
query   3’ AUGGACCGACCUGGGGAC 5’

query=JS16_t3126111, target=FGSG_11753, score=4, range=313-330, strand=1

target  5’ AACGUGGCUGGACCCUUU 3’
            :: :::::::::::.:
query   3’ AUGGACCGACCUGGGGAC 5’

query=JS16_t3126111, target=FGSG_12298, score=4, range=525-542, strand=1

target  5’ GAGGUGGCUGGGCCCCUG 3’
            :  :::::::.::::::
query   3’ AUGGACCGACCUGGGGAC 5’

query=JS16_t3126111, target=FGSG_02113, score=4, range=159-177, strand=1

target  5’ CAGCUGGCUGGAACCCCUG 3’
            : :::::::: :::::::
query   3’ AUGGACCGACC-UGGGGAC 5’

query=JS16_t3126111, target=FGSG_06901, score=4, range=553-571, strand=1

target  5’ GGCUUGGCAUGGACCCCUG 3’
            .:.:::: ::::::::::
query   3’ AUGGACCG-ACCUGGGGAC 5’

query=JS16_t3126111, target=FGSG_13582, score=4, range=1652-1669, strand=-1

target  5’ UACCUGGCUGGUCUCCUU 3’
           ::::::::::: :.:::
query   3’ AUGGACCGACCUGGGGAC 5’

query=JS16_t3126111, target=FGSG_13484, score=4, range=244-261, strand=-1

target  5’ UAGAAGGCUGGACCUCUG 3’
           ::   :::::::::.:::
query   3’ AUGGACCGACCUGGGGAC 5’

query=JS16_t3126125, target=FGSG_11505, score=2.5, range=3221-3240, strand=1

target  5’ GUUCUUCAACACAAGGUGGU 3’
           ::.::::::::::: :::::
query   3’ CAGGAAGUUGUGUUACACCA 5’

query=JS16_t3126125, target=FGSG_02320, score=3, range=779-798, strand=-1

target  5’ GUUUUUCAGCACGAUGUGGU 3’
           ::..::::.:::.:::::::
query   3’ CAGGAAGUUGUGUUACACCA 5’

query=JS16_t3126125, target=FGSG_12655, score=4, range=589-609, strand=1

target  5’ GACCAUCAACACAAUGAUGGU 3’
           : :: ::::::::::: ::::
query   3’ CAGGAAGUUGUGUUAC-ACCA 5’

query=JS16_t0794648, target=FGSG_08721, score=3.5, range=423-441, strand=1

target  5’ UGGUCCCCGACCUGCUUGU 3’
           :::::::: ::::::::..
query   3’ ACCAGGGGGUGGACGAAUG 5’

query=JS16_t0794648, target=FGSG_02816, score=3.5, range=2949-2967, strand=1

target  5’ UGGUCUCCCGCCUACUUAC 3’
           :::::.:::.::: :::::
query   3’ ACCAGGGGGUGGACGAAUG 5’

query=JS16_t0794648, target=FGSG_11459, score=3.5, range=852-870, strand=1

target  5’ UUCUUCCUCACCUGCUUAC 3’
           :  :.::.:::::::::::
query   3’ ACCAGGGGGUGGACGAAUG 5’

query=JS16_t0794648, target=FGSG_09208, score=4, range=12-30, strand=1

target  5’ UGGGCCCCUGCCUGCUUGC 3’
           ::: ::::..:::::::.:
query   3’ ACCAGGGGGUGGACGAAUG 5’

query=JS16_t0794648, target=FGSG_07592, score=4, range=464-482, strand=-1

target  5’ UGGUCUCCAACCUGCUUGU 3’
           :::::.:: ::::::::..
query   3’ ACCAGGGGGUGGACGAAUG 5’

           :: .::: ::::::::::
query   3’ GUAUCUGUUGAAGUAGUC 5’

query=JS16_t3190433, target=FGSG_04074, score=3.5, range=409-425, strand=1

target  5’ CGU-GACAACUUCAUCGC 3’
           :.: ::::::::::::.
query   3’ GUAUCUGUUGAAGUAGUC 5’

query=JS16_t3190433, target=FGSG_00780, score=3.5, range=1112-1129, strand=1

target  5’ AGAAGAUAACUUCAUCAG 3’
            .  :::.::::::::::
query   3’ GUAUCUGUUGAAGUAGUC 5’

query=JS16_t3190433, target=FGSG_00322, score=3.5, range=794-811, strand=-1

target  5’ CAUGGACAGGUUCAUCAG 3’
           :::.::::. ::::::::
query   3’ GUAUCUGUUGAAGUAGUC 5’

query=JS16_t3190433, target=FGSG_13878, score=3.5, range=26333-26350, strand=-[illegible]

target  5’ UACAGGCAGCUUCAUCAG 3’
           .: ::.::.:::::::::
query   3’ GUAUCUGUUGAAGUAGUC 5’

query=JS16_t3190433, target=FGSG_11028, score=3.5, range=1100-1117, strand=-1

target  5’ UAUAAACAACUUCAUCUG 3’
           .::: ::::::::::: :
query   3’ GUAUCUGUUGAAGUAGUC 5’

query=JS16_t3190433, target=FGSG_00543, score=3.5, range=1348-1366, strand=-1

target  5’ CAUGGUCAACUUCACUCAG 3’
           :::.: :::::::: ::::
query   3’ GUAUCUGUUGAAGU-AGUC 5’

query=JS16_t3190433, target=FGSG_09910, score=3.5, range=74-91, strand=-1

target  5’ CUUGUGCAACUUCAUCAG 3’
           : :. .::::::::::::
query   3’ GUAUCUGUUGAAGUAGUC 5’

query=JS16_t3190433, target=FGSG_03257, score=3.5, range=2465-2481, strand=-1

target  5’ CGUUGACAACUUC-UCAG 3’
           :.: ::::::::: ::::
query   3’ GUAUCUGUUGAAGUAGUC 5’

query=JS16_t3190433, target=FGSG_08064, score=3.5, range=2405-2421, strand=-1

target  5’ CGUAGA-AACUUCAUCAA 3’
           :.:::: ::::::::::
query   3’ GUAUCUGUUGAAGUAGUC 5’

query=JS16_t3190433, target=FGSG_00780, score=4, range=282-299, strand=1

target  5’ UAUGGACAGCUUCAUGAG 3’
           .::.::::.:::::: ::
query   3’ GUAUCUGUUGAAGUAGUC 5’

query=JS16_t3190433, target=FGSG_06901, score=4, range=152-169, strand=1

target  5’ CGUGGGCACCUUCAUCAG 3’
           :.:.:.:: :::::::::
query   3’ GUAUCUGUUGAAGUAGUC 5’

query=JS16_t3190433, target=FGSG_02519, score=4, range=1335-1352, strand=1

target  5’ CAUAGACGACGUCAUCAA 3’
           :::::::.:: ::::::
query   3’ GUAUCUGUUGAAGUAGUC 5’

query=JS16_t3190433, target=FGSG_04862, score=4, range=720-737, strand=1

target  5’ CAUAGAUAUCUUCAUCAU 3’
           ::::::.: ::::::::
query   3’ GUAUCUGUUGAAGUAGUC 5’

query=JS16_t3190433, target=FGSG_03932, score=4, range=489-506, strand=1

target  5’ GGUGGACAACAUCAUCAG 3’
            .:.:::::: :::::::
query   3’ GUAUCUGUUGAAGUAGUC 5’

query=JS16_t3190433, target=FGSG_08785, score=4, range=1742-1760, strand=1

target  5’ CACUAGACGGCUUCAUCAU 3’
           :: :::::..::::::::
query   3’ GU-AUCUGUUGAAGUAGUC 5’
```

query=JS16_t2229904, target=FGSG_10789, score=3, range=602-619, strand=-1

target 5' UCUCCUGGAUAGGUUCCU 3'
           :::: ::::.:::::::
query   3' AGAGUACCUGUCCAAGGU 5'

query=JS16_t2229904, target=FGSG_09617, score=3, range=1000-1016, strand=-1

target 5' GCUU-UGGACAGGUUCCG 3'
            ::. ::::::::::::.
query   3' AGAGUACCUGUCCAAGGU 5'

query=JS16_t2229904, target=FGSG_13687, score=3.5, range=23-40, strand=1

target 5' UUUCAUGGCCAGGUUCCU 3'
           :.:::::: ::::::::
query   3' AGAGUACCUGUCCAAGGU 5'

query=JS16_t2229904, target=FGSG_10944, score=3.5, range=3957-3973, strand=1

target 5' UUUCAUGGA-AGGUUUCA 3'
           :.::::::: :::::.::
query   3' AGAGUACCUGUCCAAGGU 5'

query=JS16_t2229904, target=FGSG_06177, score=3.5, range=939-955, strand=1

target 5' UUUCAUGGACA-GUUUCA 3'
           :.::::::::: :::.::
query   3' AGAGUACCUGUCCAAGGU 5'

query=JS16_t2229904, target=FGSG_05236, score=3.5, range=1925-1943, strand=1

target 5' UCUCGCUGGACAUGUUCCA 3'
           ::::. :::::: ::::::
query   3' AGAGU-ACCUGUCCAAGGU 5'

query=JS16_t2229904, target=FGSG_03668, score=3.5, range=684-700, strand=1

target 5' UCGCA-GGACAGGUUCCG 3'
           :: :: :::::::::::.
query   3' AGAGUACCUGUCCAAGGU 5'

query=JS16_t2229904, target=FGSG_08320, score=3.5, range=1453-1469, strand=1

target 5' UUUC-UGGACUGGUUCCA 3'
           :.:: ::::: :::::::
query   3' AGAGUACCUGUCCAAGGU 5'

query=JS16_t2229904, target=FGSG_06405, score=3.5, range=365-382, strand=-1

target 5' UUUCAGGGACGGGUUCCA 3'
           :.::: ::::.:::::::
query   3' AGAGUACCUGUCCAAGGU 5'

query=JS16_t2229904, target=FGSG_05351, score=3.5, range=877-894, strand=-1

target 5' UUCCUUGGACAGGUUCCU 3'
           :. : ::::::::::::
query   3' AGAGUACCUGUCCAAGGU 5'

query=JS16_t2229904, target=FGSG_07613, score=3.5, range=833-849, strand=-1

target 5' CCUU-UGGACAGGUUCCU 3'
            ::. ::::::::::::
query   3' AGAGUACCUGUCCAAGGU 5'

query=JS16_t2229904, target=FGSG_05188, score=4, range=2751-2768, strand=1

target 5' UCUCACGGAUGGGUUCCA 3'
           ::::: :::..:::::::
query   3' AGAGUACCUGUCCAAGGU 5'

query=JS16_t2229904, target=FGSG_06333, score=4, range=12-29, strand=1

target 5' UCUCAUGGACAAGAUCCA 3'
           ::::::::::: : ::::
query   3' AGAGUACCUGUCCAAGGU 5'

query=JS16_t2229904, target=FGSG_08611, score=4, range=206-224, strand=1

target 5' UCAUUAUGGACAGGUUCGG 3'
           :: :.:::::::::::: .
query   3' AG-AGUACCUGUCCAAGGU 5'

query=JS16_t2229904, target=FGSG_00257, score=4, range=187-204, strand=1

target 5' GCUCUUGGACAGGUUCUU 3'
            ::: :::::::::::.
query   3' AGAGUACCUGUCCAAGGU 5'

query=JS16_t2229904, target=FGSG_04859, score=4, range=3834-3851, strand=1

query=JS16_t3190433, target=FGSG_13033, score=4, range=1017-1034, strand=1

target 5' UGAAGACGACUUCAUCAU 3'
           .. ::::.:::::::::
query   3' GUAUCUGUUGAAGUAGUC 5'

query=JS16_t3190433, target=FGSG_00541, score=4, range=213-230, strand=1

target 5' CAUCGACAACUUCAACGG 3'
           ::: :::::::::: :.:
query   3' GUAUCUGUUGAAGUAGUC 5'

query=JS16_t3190433, target=FGSG_03020, score=4, range=426-443, strand=1

target 5' CAUAUACGACAUCAUCAG 3'
           :::: ::.:: :::::::
query   3' GUAUCUGUUGAAGUAGUC 5'

query=JS16_t3190433, target=FGSG_06374, score=4, range=955-972, strand=1

target 5' CAUACACAACUUAGUCAG 3'
           :::: ::::::: .::::
query   3' GUAUCUGUUGAAGUAGUC 5'

query=JS16_t3190433, target=FGSG_04839, score=4, range=618-635, strand=1

target 5' CACAGACAACUUUAACAG 3'
           :: :::::::::.: :::
query   3' GUAUCUGUUGAAGUAGUC 5'

query=JS16_t3190433, target=FGSG_06205, score=4, range=342-359, strand=1

target 5' CAUCGACAGCCUCAUCAG 3'
           ::: ::::.: :::::::
query   3' GUAUCUGUUGAAGUAGUC 5'

query=JS16_t3190433, target=FGSG_06486, score=4, range=1506-1523, strand=1

target 5' UGGAGACAACUUCAACAG 3'
           .. ::::::::::: :::
query   3' GUAUCUGUUGAAGUAGUC 5'

query=JS16_t3190433, target=FGSG_11716, score=4, range=1216-1234, strand=1

target 5' UAUAAGCAACUUCACUCAG 3'
           .::: .:::::::: ::::
query   3' GUAUCUGUUGAAGU-AGUC 5'

query=JS16_t3190433, target=FGSG_01497, score=4, range=542-559, strand=1

target 5' CAUUGAAAACUUCAUCAC 3'
           ::: :: ::::::::::
query   3' GUAUCUGUUGAAGUAGUC 5'

query=JS16_t3190433, target=FGSG_00775, score=4, range=342-359, strand=1

target 5' CACAGCCAACUUCAUCAA 3'
           :: :: :::::::::::
query   3' GUAUCUGUUGAAGUAGUC 5'

query=JS16_t3190433, target=FGSG_07471, score=4, range=12-29, strand=1

target 5' CAAAGUCAACUUCAUCAC 3'
           :: :: ::::::::::
query   3' GUAUCUGUUGAAGUAGUC 5'

query=JS16_t3190433, target=FGSG_03420, score=4, range=158-175, strand=1

target 5' AAAAGACUACUUCAUCAG 3'
            : :::: ::::::::::
query   3' GUAUCUGUUGAAGUAGUC 5'

query=JS16_t3190433, target=FGSG_11472, score=4, range=1854-1871, strand=1

target 5' CACUGAUAACUUCAUCAA 3'
           :: ::.::::::::::
query   3' GUAUCUGUUGAAGUAGUC 5'

query=JS16_t3190433, target=FGSG_06326, score=4, range=3529-3547, strand=1

target 5' CCUAGUACGACUUCAUCAU 3'
           : ::: ::.:::::::::
query   3' GUAUC-UGUUGAAGUAGUC 5'

query=JS16_t3190433, target=FGSG_13195, score=4, range=322-339, strand=1

target 5' CUUCGACAACUUCAUCGC 3'
           : : :::::::::::.
query   3' GUAUCUGUUGAAGUAGUC 5'

query=JS16_t3190433, target=FGSG_06257, score=4, range=798-815, strand=1

```
target  5’ UAUCAUGAACAGGUUCCC 3’
           : ::::: :::::::::
query   3’ AGAGUACCUGUCCAAGGU 5’

query=JS16_t2229904, target=FGSG_04452, score=4, range=996-1013, strand=1

target  5’ UCAGAUGGACAUGUUCCA 3’
           ::  ::::::: ::::::
query   3’ AGAGUACCUGUCCAAGGU 5’

query=JS16_t2229904, target=FGSG_13634, score=4, range=1487-1503, strand=1

target  5’ GCUC-UGGACAGGUUGCA 3’
            ::: :::::::::: ::
query   3’ AGAGUACCUGUCCAAGGU 5’

query=JS16_t2229904, target=FGSG_09115, score=4, range=215-231, strand=1

target  5’ UGUUGUGG-CAGGUUCCA 3’
           : :..::: :::::::::
query   3’ AGAGUACCUGUCCAAGGU 5’

query=JS16_t2229904, target=FGSG_06866, score=4, range=455-471, strand=1

target  5’ UCUC-UGGACCGGUUCCC 3’
           :::: ::::: ::::::
query   3’ AGAGUACCUGUCCAAGGU 5’

query=JS16_t2229904, target=FGSG_00693, score=4, range=404-420, strand=1

target  5’ UGACAUGGACA-GUUCCA 3’
           :  :::::::: ::::::
query   3’ AGAGUACCUGUCCAAGGU 5’

query=JS16_t2229904, target=FGSG_00259, score=4, range=650-667, strand=-1

target  5’ UCUCAUGGAUAGCUUCCU 3’
           :::::::::.:: ::::
query   3’ AGAGUACCUGUCCAAGGU 5’

query=JS16_t2229904, target=FGSG_13670, score=4, range=920-937, strand=-1

target  5’ UCUCCUGGGCAGGUUCUU 3’
           :::: :::.:::::::.
query   3’ AGAGUACCUGUCCAAGGU 5’

query=JS16_t2229904, target=FGSG_02895, score=4, range=1002-1018, strand=-1

target  5’ UCUUG-GGAUAGGUUCCA 3’
           :::.. :::.::::::::
query   3’ AGAGUACCUGUCCAAGGU 5’

query=JS16_t2229904, target=FGSG_13814, score=4, range=128-144, strand=-1

target  5’ UUUUAUGGGCA-GUUCCA 3’
           :.:.::::.:: ::::::
query   3’ AGAGUACCUGUCCAAGGU 5’

query=JS16_t2229904, target=FGSG_07208, score=4, range=1125-1142, strand=-1

target  5’ UCGCGUGGAAAGGUUCCG 3’
           :: :.:::: :::::::.
query   3’ AGAGUACCUGUCCAAGGU 5’

query=JS16_t2229904, target=FGSG_08053, score=4, range=470-487, strand=-1

target  5’ UCUCCUGUACAGGUUCCC 3’
           :::: :: :::::::::
query   3’ AGAGUACCUGUCCAAGGU 5’

query=JS16_t2229904, target=FGSG_08185, score=4, range=1190-1207, strand=-1

target  5’ CCUCUUGGACAAGUUCCA 3’
            ::: :::::: ::::::
query   3’ AGAGUACCUGUCCAAGGU 5’

query=JS16_t2229904, target=FGSG_00844, score=4, range=852-868, strand=-1

target  5’ UCUCAUGGACAGG-GCCA 3’
           :::::::::::::  :::
query   3’ AGAGUACCUGUCCAAGGU 5’

query=JS16_t2229904, target=FGSG_00531, score=4, range=1024-1040, strand=-1

target  5’ UC-CAUGGGAAGGUUCCA 3’
           :: :::::. ::::::::
query   3’ AGAGUACCUGUCCAAGGU 5’

query=JS16_t2229904, target=FGSG_05221, score=4, range=529-545, strand=-1

target  5’ UC-CUUGGAAAGGUUCCA 3’
           :: : :::: ::::::::

target  5’ CAACGACAACUUCGUCAA 3’
           ::  :::::::::.:::
query   3’ GUAUCUGUUGAAGUAGUC 5’

query=JS16_t3190433, target=FGSG_12999, score=4, range=17-34, strand=1

target  5’ CAUCAGCAACUUCAUCAA 3’
           :::  .:::::::::::
query   3’ GUAUCUGUUGAAGUAGUC 5’

query=JS16_t3190433, target=FGSG_09054, score=4, range=900-916, strand=1

target  5’ CA-AGCCAACUUCGUCAG 3’
           :: :: :::::::.::::
query   3’ GUAUCUGUUGAAGUAGUC 5’

query=JS16_t3190433, target=FGSG_04442, score=4, range=869-887, strand=1

target  5’ CAUACAACGACUUCAUCAU 3’
           ::::  ::.:::::::::
query   3’ GUAU-CUGUUGAAGUAGUC 5’

query=JS16_t3190433, target=FGSG_04892, score=4, range=24-40, strand=1

target  5’ CACAG-CAGCUUCAUCAG 3’
           :: :: ::.:::::::::
query   3’ GUAUCUGUUGAAGUAGUC 5’

query=JS16_t3190433, target=FGSG_05685, score=4, range=904-920, strand=1

target  5’ CA-ACACAACUUUAUCAA 3’
           :: : :::::::.::::
query   3’ GUAUCUGUUGAAGUAGUC 5’

query=JS16_t3190433, target=FGSG_00194, score=4, range=534-551, strand=1

target  5’ CUUCAACAACUUCAUCAC 3’
           : :  :::::::::::
query   3’ GUAUCUGUUGAAGUAGUC 5’

query=JS16_t3190433, target=FGSG_08785, score=4, range=81-98, strand=1

target  5’ CAUCAACAACUUCAUCGC 3’
           :::  :::::::::::.
query   3’ GUAUCUGUUGAAGUAGUC 5’

query=JS16_t3190433, target=FGSG_05859, score=4, range=1520-1537, strand=-1

target  5’ CAUAGAUAAUAUCAUCAG 3’
           ::::::.::. :::::::
query   3’ GUAUCUGUUGAAGUAGUC 5’

query=JS16_t3190433, target=FGSG_02318, score=4, range=416-433, strand=-1

target  5’ CAAAGACGAUUUCAUCGG 3’
           :: ::::.:.::::::.:
query   3’ GUAUCUGUUGAAGUAGUC 5’

query=JS16_t3190433, target=FGSG_01805, score=4, range=1559-1576, strand=-1

target  5’ CAAAGGUGACUUCAUCAG 3’
           :: ::...::::::::::
query   3’ GUAUCUGUUGAAGUAGUC 5’

query=JS16_t3190433, target=FGSG_11021, score=4, range=1517-1534, strand=-1

target  5’ UGUAGACAACUUCAUCUA 3’
           ..::::::::::::::
query   3’ GUAUCUGUUGAAGUAGUC 5’

query=JS16_t3190433, target=FGSG_07936, score=4, range=107-123, strand=-1

target  5’ CA-AGGCAGCUUCAUCGG 3’
           :: ::.::.:::::::.:
query   3’ GUAUCUGUUGAAGUAGUC 5’

query=JS16_t3190433, target=FGSG_01693, score=4, range=1523-1540, strand=-1

target  5’ CAUCGACAGCUUCUUCAG 3’
           ::: ::::.:::: ::::
query   3’ GUAUCUGUUGAAGUAGUC 5’

query=JS16_t3190433, target=FGSG_00890, score=4, range=386-403, strand=-1

target  5’ CAGAGACAGCUUCCUCAG 3’
           :: :::::.:::: ::::
query   3’ GUAUCUGUUGAAGUAGUC 5’

query=JS16_t3190433, target=FGSG_10780, score=4, range=1145-1162, strand=-1

target  5’ CAAAGACGACUUCAACAG 3’
```

```
query   3’ AGAGUACCUGUCCAAGGU 5’

query=JS16_t2229904, target=FGSG_12329, score=4, range=164-180, strand=-1

target  5’ CCACAUGGACA-GUUCCA 3’
            : :::::::: ::::::
query   3’ AGAGUACCUGUCCAAGGU 5’

query=JS16_t0685341, target=FGSG_09114, score=2.5, range=333-353, strand=-1

target  5’ UCCAAGCACUCAGAGAUGUAG 3’
           .:::::: ::::::::.::::
query   3’ GGGUUCGAGAGUCUCUGCAUC 5’

query=JS16_t0685341, target=FGSG_11831, score=4, range=245-264, strand=1

target  5’ UUCAAGCCCUCAGAGACG-AG 3’
           ..::::: :::::::::: ::
query   3’ GGGUUCGAGAGUCUCUGCAUC 5’

query=JS16_t0685341, target=FGSG_00299, score=4, range=2931-2952, strand=-1

target  5’ CUCGAGCUGGUCAGAGACGUAG 3’
           :.:.::::  ::::::::::::
query   3’ GGGUUCGA-GAGUCUCUGCAUC 5’

query=JS16_t0794658, target=FGSG_06120, score=2.5, range=374-390, strand=-1

target  5’ GAG-AUCGUUCUUCGCAC 3’
           .:: :::.::::::::::
query   3’ UUCGUAGUAAGAAGCGUG 5’

query=JS16_t0794658, target=FGSG_10043, score=3, range=400-416, strand=1

target  5’ AAGCG-CAUUCUUCGCAU 3’
           ::::. :::::::::::.
query   3’ UUCGUAGUAAGAAGCGUG 5’

query=JS16_t0794658, target=FGSG_01847, score=3, range=806-824, strand=1

target  5’ AAGACUUCAUUUUUCGCAC 3’
           ::: : :::::.:::::::
query   3’ UUC-GUAGUAAGAAGCGUG 5’

query=JS16_t0794658, target=FGSG_07598, score=3, range=484-500, strand=1

target  5’ AAG-AUCUUUCUUCGCAC 3’
           ::: ::: ::::::::::
query   3’ UUCGUAGUAAGAAGCGUG 5’

query=JS16_t0794658, target=FGSG_00930, score=3, range=447-464, strand=-1

target  5’ CAGCAUCGUUCUUCGCAG 3’
            ::::::.:::::::::
query   3’ UUCGUAGUAAGAAGCGUG 5’

query=JS16_t0794658, target=FGSG_01668, score=3, range=362-379, strand=-1

target  5’ GAUCAUCGUUCUUCGCAU 3’
           .: ::::.:::::::::.
query   3’ UUCGUAGUAAGAAGCGUG 5’

query=JS16_t0794658, target=FGSG_06831, score=3.5, range=2967-2984, strand=1

target  5’ AAGCAUCAUUAUUCGCGU 3’
           :::::::::: :::::..
query   3’ UUCGUAGUAAGAAGCGUG 5’

query=JS16_t0794658, target=FGSG_03606, score=3.5, range=1340-1358, strand=1

target  5’ AGGCGGUUAUUCUUCGCAU 3’
           :.:: .:.::::::::::.
query   3’ UUCG-UAGUAAGAAGCGUG 5’

query=JS16_t0794658, target=FGSG_08229, score=3.5, range=938-955, strand=1

target  5’ CGGGAUCAUUCUUCGCGC 3’
            .: ::::::::::::.:
query   3’ UUCGUAGUAAGAAGCGUG 5’

query=JS16_t0794658, target=FGSG_08701, score=3.5, range=2611-2628, strand=1

target  5’ CUGGAUCAUUCUUCGCAU 3’
             : :::::::::::::.
query   3’ UUCGUAGUAAGAAGCGUG 5’

query=JS16_t0794658, target=FGSG_03702, score=3.5, range=824-840, strand=1

target  5’ GAGC-UCAUUCUUUGCAA 3’
           .::: ::::::::.:::
query   3’ UUCGUAGUAAGAAGCGUG 5’

           :: ::::.:::::: :::
query   3’ GUAUCUGUUGAAGUAGUC 5’

query=JS16_t3190433, target=FGSG_08907, score=4, range=770-787, strand=-1

target  5’ CAUUGGCAACAUCAUCAG 3’
           ::: :.:::: :::::::
query   3’ GUAUCUGUUGAAGUAGUC 5’

query=JS16_t3190433, target=FGSG_11490, score=4, range=293-310, strand=-1

target  5’ CAUCGACGACUUGAUCAG 3’
           ::: :::.:::: :::::
query   3’ GUAUCUGUUGAAGUAGUC 5’

query=JS16_t3190433, target=FGSG_09876, score=4, range=707-724, strand=-1

target  5’ CAUCAACAGCUUCGUCAG 3’
           :::  :::.::::.::::
query   3’ GUAUCUGUUGAAGUAGUC 5’

query=JS16_t3190433, target=FGSG_02299, score=4, range=110-127, strand=-1

target  5’ CAAAUGCAAUUUCAUCAG 3’
           :: : .:::.::::::::
query   3’ GUAUCUGUUGAAGUAGUC 5’

query=JS16_t3190433, target=FGSG_11076, score=4, range=317-334, strand=-1

target  5’ GAUCGACAACUUCAUCGC 3’
            :: ::::::::::::.
query   3’ GUAUCUGUUGAAGUAGUC 5’

query=JS16_t3190433, target=FGSG_09446, score=4, range=233-251, strand=-1

target  5’ AAUACGACAACUUCAUCGA 3’
            ::: ::::::::::::.
query   3’ GUAU-CUGUUGAAGUAGUC 5’

query=JS16_t3190433, target=FGSG_07993, score=4, range=2252-2268, strand=-1

target  5’ CA-AGACAACUUUGUCAC 3’
           :: :::::::::..:::
query   3’ GUAUCUGUUGAAGUAGUC 5’

query=JS16_t3190433, target=FGSG_01049, score=4, range=8264-8281, strand=-1

target  5’ CUUCGACAACUUCAGCAG 3’
           : : :::::::::: :::
query   3’ GUAUCUGUUGAAGUAGUC 5’

query=JS16_t3190433, target=FGSG_00612, score=4, range=92-108, strand=-1

target  5’ CAUAG-CUACUUCAUCAG 3’
           ::::: : ::::::::::
query   3’ GUAUCUGUUGAAGUAGUC 5’

query=JS16_t3190433, target=FGSG_04768, score=4, range=823-841, strand=-1

target  5’ CAUAGCCAACUUCCAUCAU 3’
           ::::: :::::: :::::
query   3’ GUAUCUGUUGAA-GUAGUC 5’

query=JS16_t3190433, target=FGSG_02504, score=4, range=638-655, strand=-1

target  5’ CCUUGACAACUUCGUCAC 3’
           : : :::::::::.:::
query   3’ GUAUCUGUUGAAGUAGUC 5’

query=JS16_t3190433, target=FGSG_05191, score=4, range=686-704, strand=-1

target  5’ CACUUGACAACUUCGUCAC 3’
           :: : :::::::::.:::
query   3’ GU-AUCUGUUGAAGUAGUC 5’

query=JS16_t3190433, target=FGSG_08738, score=4, range=2297-2314, strand=-1

target  5’ CAGAAACACCUUCAUCAG 3’
           :: : ::: :::::::::
query   3’ GUAUCUGUUGAAGUAGUC 5’

query=JS16_t3190433, target=FGSG_01332, score=4, range=2501-2518, strand=-1

target  5’ CAGCAGCAACUUCAUCAG 3’
           ::   .::::::::::::
query   3’ GUAUCUGUUGAAGUAGUC 5’

query=JS16_t3190433, target=FGSG_04793, score=4, range=1-18, strand=-1

target  5’ AAAAUACAACUUCAUCAU 3’
            : : :::::::::::::
query   3’ GUAUCUGUUGAAGUAGUC 5’
```

```
query=JS16_t0794658, target=FGSG_13854, score=3.5, range=911-929, strand=-1

target  5’ AGGCAUCGUUCUUCCGCAC 3’
           :.:::::.::::: :::::
query   3’ UUCGUAGUAAGAA-GCGUG 5’

query=JS16_t0794658, target=FGSG_01483, score=4, range=828-845, strand=1

target  5’ AGGUAUCAUUCUUCCUAC 3’
           :.:.:::::::::: .::
query   3’ UUCGUAGUAAGAAGCGUG 5’

query=JS16_t0794658, target=FGSG_02328, score=4, range=2016-2034, strand=1

target  5’ AAGCAGUCGUUCUUCGUAG 3’
           ::::: ::.:::::::.:
query   3’ UUCGU-AGUAAGAAGCGUG 5’

query=JS16_t0794658, target=FGSG_08026, score=4, range=297-314, strand=1

target  5’ GCGCAUCAUUCUUCUCAU 3’
           .  :::::::::::: ::.
query   3’ UUCGUAGUAAGAAGCGUG 5’

query=JS16_t0794658, target=FGSG_11266, score=4, range=110-126, strand=1

target  5’ AAGCAUCAUUGUU-GCAC 3’
           :::::::::: :: ::::
query   3’ UUCGUAGUAAGAAGCGUG 5’

query=JS16_t0794658, target=FGSG_07328, score=4, range=553-569, strand=1

target  5’ AAG-AUCAUUCUUCUUAC 3’
           ::: :::::::::: .::
query   3’ UUCGUAGUAAGAAGCGUG 5’

query=JS16_t0794658, target=FGSG_05056, score=4, range=194-210, strand=1

target  5’ AAGC-UCAUGUUUCGCAC 3’
           :::: :::: .:::::::
query   3’ UUCGUAGUAAGAAGCGUG 5’

query=JS16_t0794658, target=FGSG_06355, score=4, range=715-731, strand=1

target  5’ AAG-GUCAUUCUACGCAU 3’
           ::: .::::::: ::::.
query   3’ UUCGUAGUAAGAAGCGUG 5’

query=JS16_t0794658, target=FGSG_11135, score=4, range=85-101, strand=1

target  5’ GAG-AUCACUCUUCGCAU 3’
           .:: :::: ::::::::.
query   3’ UUCGUAGUAAGAAGCGUG 5’

query=JS16_t0794658, target=FGSG_10047, score=4, range=1356-1372, strand=1

target  5’ AAGCUUCAUUCUUC-CAG 3’
           :::: ::::::::: ::
query   3’ UUCGUAGUAAGAAGCGUG 5’

query=JS16_t0794658, target=FGSG_01949, score=4, range=1978-1995, strand=-1

target  5’ AAGCUUCAUUCUUCGUGG 3’
           :::: ::::::::::..
query   3’ UUCGUAGUAAGAAGCGUG 5’

query=JS16_t0794658, target=FGSG_12425, score=4, range=123-140, strand=-1

target  5’ CAGCGACAUUCUUCGCAU 3’
            :::. :::::::::::.
query   3’ UUCGUAGUAAGAAGCGUG 5’

query=JS16_t0794658, target=FGSG_13664, score=4, range=488-505, strand=-1

target  5’ CAGCCUUGUUCUUCGCAC 3’
            ::: :..::::::::::
query   3’ UUCGUAGUAAGAAGCGUG 5’

query=JS16_t0794658, target=FGSG_05085, score=4, range=185-202, strand=-1

target  5’ AACCGGCAUUCUUCGCAU 3’
           :: :.  :::::::::::.
query   3’ UUCGUAGUAAGAAGCGUG 5’

query=JS16_t0794658, target=FGSG_11376, score=4, range=1142-1159, strand=-1

target  5’ AACUAGCAUUCUUCGCAU 3’
           :: .: :::::::::::.
query   3’ UUCGUAGUAAGAAGCGUG 5’

query=JS16_t0794658, target=FGSG_11713, score=4, range=364-381, strand=-1

query=JS16_t3190433, target=FGSG_12068, score=4, range=122-138, strand=-1

target  5’ GAU-GACAACGUCAUCAG 3’
            :: :::::: :::::::
query   3’ GUAUCUGUUGAAGUAGUC 5’

query=JS16_t3190433, target=FGSG_08023, score=4, range=1480-1498, strand=-1

target  5’ CGCGGACAACUUCAGUCAG 3’
           :. .:::::::::: ::::
query   3’ GUAUCUGUUGAAGU-AGUC 5’

query=JS16_t3190433, target=FGSG_02791, score=4, range=914-930, strand=-1

target  5’ UAGGGAC-ACUUCAUCAG 3’
           .: .::: ::::::::::
query   3’ GUAUCUGUUGAAGUAGUC 5’

No results for JS16_t2294184
No results for JS16_t2294210
query=JS16_t2294223, target=FGSG_05568, score=3.5, range=1194-1217, strand=-1

target  5’ AGGAAAGGUGUCCCAUGAAGUGAU 3’
           :::::  :.: :::::::::::::
query   3’ UCCUUGGCGC-GGGUACUUCACUA 5’

query=JS16_t0229065, target=FGSG_05427, score=3.5, range=526-545, strand=1

target  5’ CCUCAGCAGCAAACUCAAGG 3’
           ::.:::::::::::::: ::
query   3’ GGGGUCGUCGUUUGAGCUCA 5’

query=JS16_t0229065, target=FGSG_05566, score=3.5, range=636-654, strand=1

target  5’ CUGCAG-AGUAAACUCGAGU 3’
           :.  ::: ::.::::::::::
query   3’ GGGGUCGUCGUUUGAGCUCA 5’

query=JS16_t0229065, target=FGSG_02470, score=3.5, range=2154-2173, strand=-1

target  5’ CCUCAUCAGUAAACUCGGGU 3’
           ::.:: :::.:::::::.::
query   3’ GGGGUCGUCGUUUGAGCUCA 5’

query=JS16_t0229065, target=FGSG_03012, score=3.5, range=1121-1140, strand=-1

target  5’ CCAGGGCAGCAAACUUGAGU 3’
           ::  .::::::::::.::::
query   3’ GGGGUCGUCGUUUGAGCUCA 5’

query=JS16_t0229065, target=FGSG_13104, score=3.5, range=82-100, strand=-1

target  5’ CAUCAG-AGCAAACUCGAGA 3’
           : .::: ::::::::::::
query   3’ GGGGUCGUCGUUUGAGCUCA 5’

query=JS16_t0229065, target=FGSG_10848, score=4, range=1314-1333, strand=1

target  5’ CUUCAGCAGCGGACUCGAGG 3’
           :..:::::::..:::::::
query   3’ GGGGUCGUCGUUUGAGCUCA 5’

query=JS16_t0229065, target=FGSG_12129, score=4, range=1142-1162, strand=1

target  5’ UCACAGCGGCAAACUACGAGU 3’
           .: ::::.:::::::: :::::
query   3’ GGGGUCGUCGUUUGA-GCUCA 5’

No results for JS16_t3190551
query=JS16_t0584414, target=FGSG_06793, score=1.5, range=471-489, strand=-1

target  5’ GAGAUCGCCGUUCUUUGCU 3’
           :::: ::::::::::::::.
query   3’ CUCU-GCGGCAAGAAACGG 5’

query=JS16_t0584414, target=FGSG_04588, score=3, range=3678-3695, strand=1

target  5’ GAGACGCCGUUUUAUGCC 3’
           ::::::::::::.: ::::
query   3’ CUCUGCGGCAAGAAACGG 5’

query=JS16_t0584414, target=FGSG_03455, score=3, range=714-731, strand=-1

target  5’ GGGGCGCCGUUCUUUGUG 3’
           :.:.::::::::::::.
query   3’ CUCUGCGGCAAGAAACGG 5’

query=JS16_t0584414, target=FGSG_04518, score=3, range=1359-1376, strand=-1

target  5’ AGGGCGCCGUUCUUUGCA 3’
            .:.::::::::::::::
```

```
target  5’ CAUGAUCAUUUUUCGCAC 3’
            :  ::::::.:::::::
query   3’ UUCGUAGUAAGAAGCGUG 5’

query=JS16_t0794658, target=FGSG_13353, score=4, range=1543-1560, strand=-1

target  5’ AAGAACCAUUCUUCGCAG 3’
           ::: : :::::::::::
query   3’ UUCGUAGUAAGAAGCGUG 5’

query=JS16_t0794658, target=FGSG_11937, score=4, range=209-226, strand=-1

target  5’ CAUCAUCAUUCUUCUCAC 3’
            : ::::::::::: :::
query   3’ UUCGUAGUAAGAAGCGUG 5’

query=JS16_t0794658, target=FGSG_13822, score=4, range=1681-1699, strand=-1

target  5’ UAUCAUCAUUCUUCGACAC 3’
            : :::::::::::: :::
query   3’ UUCGUAGUAAGAAGC-GUG 5’

query=JS16_t0794658, target=FGSG_08634, score=4, range=908-924, strand=-1

target  5’ AAG-AUCAUUCUGCGCAG 3’
           ::: :::::::: ::::
query   3’ UUCGUAGUAAGAAGCGUG 5’

query=JS16_t0794658, target=FGSG_09640, score=4, range=1224-1240, strand=-1

target  5’ GUGCAUC-UUCUUCGCAU 3’
           . ::::: :::::::::.
query   3’ UUCGUAGUAAGAAGCGUG 5’

query=JS16_t0794658, target=FGSG_10522, score=4, range=2547-2563, strand=-1

target  5’ AAGCGUCG-UCUUCGCAU 3’
           ::::.::. ::::::::.
query   3’ UUCGUAGUAAGAAGCGUG 5’

query=JS16_t2229958, target=FGSG_06930, score=2.5, range=60-77, strand=1

target  5’ UUCUGAUAUCGAGCUAGA 3’
           :: :.:::.:::::::::
query   3’ AAUAUUAUGGCUCGAUCU 5’

query=JS16_t2229958, target=FGSG_07504, score=3, range=873-889, strand=-1

target  5’ UUAU-AUAUCGAGUUAGA 3’
           :::: :::.::::.::::
query   3’ AAUAUUAUGGCUCGAUCU 5’

query=JS16_t2229958, target=FGSG_09121, score=3.5, range=149-166, strand=-1

target  5’ UUCUGAUACCGAGGUAGA 3’
           :: :.:::::::: ::::
query   3’ AAUAUUAUGGCUCGAUCU 5’

query=JS16_t2229958, target=FGSG_04654, score=4, range=972-989, strand=1

target  5’ UUAUCAUGCCGAGUUGGA 3’
           :::: ::.:::::.:.::
query   3’ AAUAUUAUGGCUCGAUCU 5’

query=JS16_t2229958, target=FGSG_00716, score=4, range=486-503, strand=1

target  5’ GAAGGAUACCGAGCUAGG 3’
             : .::::::::::::.
query   3’ AAUAUUAUGGCUCGAUCU 5’

query=JS16_t2229958, target=FGSG_10074, score=4, range=1318-1336, strand=-1

target  5’ UUGCUGAUACCGAGCUGGC 3’
           ::. :.::::::::::.:
query   3’ AAU-AUUAUGGCUCGAUCU 5’

query=JS16_t2229958, target=FGSG_11525, score=4, range=822-838, strand=-1

target  5’ UUA-AAUGCUGAGUUAGA 3’
           ::: :::.:.:::.::::
query   3’ AAUAUUAUGGCUCGAUCU 5’

query=JS16_t2229958, target=FGSG_00046, score=4, range=1576-1592, strand=-1

target  5’ AUAUAA-ACCGAGCUGGA 3’
            ::::: ::::::::.::
query   3’ AAUAUUAUGGCUCGAUCU 5’

query=JS16_t2229958, target=FGSG_05797, score=4, range=1052-1069, strand=-1

target  5’ UGACAAUACCGAGCUGGC 3’
           : : :::::::::::.:
query   3’ CUCUGCGGCAAGAAACGG 5’

query=JS16_t0584414, target=FGSG_07256, score=3, range=747-764, strand=-1

target  5’ UGGUUGCCGUUCUUUGCC 3’
            .: .:::::::::::::
query   3’ CUCUGCGGCAAGAAACGG 5’

query=JS16_t0584414, target=FGSG_03348, score=3.5, range=245-262, strand=1

target  5’ UCGAUGCUGUUCUUUGCC 3’
             ::.::.::::::::::
query   3’ CUCUGCGGCAAGAAACGG 5’

query=JS16_t0584414, target=FGSG_06468, score=3.5, range=460-477, strand=1

target  5’ GAGGCGCCGUUCUUUCCG 3’
           :::.:::::::::::: :
query   3’ CUCUGCGGCAAGAAACGG 5’

query=JS16_t0584414, target=FGSG_00695, score=3.5, range=1172-1189, strand=1

target  5’ GGGACGCCGUUCUUGGCG 3’
           :.:::::::::::: ::
query   3’ CUCUGCGGCAAGAAACGG 5’

query=JS16_t0584414, target=FGSG_09924, score=3.5, range=201-218, strand=1

target  5’ GGGUCGCCGUUCUGUGCC 3’
           :.: ::::::::: ::::
query   3’ CUCUGCGGCAAGAAACGG 5’

query=JS16_t0584414, target=FGSG_06563, score=3.5, range=369-386, strand=-1

target  5’ UACACGUCGUUCUUUGCU 3’
            : :::.::::::::::.
query   3’ CUCUGCGGCAAGAAACGG 5’

query=JS16_t0584414, target=FGSG_08309, score=3.5, range=1618-1634, strand=-1

target  5’ GAGGCGCUGUUC-UUGCC 3’
           :::.:::.:::: :::::
query   3’ CUCUGCGGCAAGAAACGG 5’

query=JS16_t0584414, target=FGSG_09806, score=3.5, range=3-21, strand=-1

target  5’ GAUAUUGUCGUUCUUUGCC 3’
           :: : .:.:::::::::::
query   3’ CUCU-GCGGCAAGAAACGG 5’

query=JS16_t0584414, target=FGSG_02293, score=3.5, range=771-788, strand=-1

target  5’ UUGUUGCCGUUCUUUGCC 3’
             : .:::::::::::::
query   3’ CUCUGCGGCAAGAAACGG 5’

query=JS16_t0584414, target=FGSG_03530, score=3.5, range=168-185, strand=-1

target  5’ GUAGCGCCGUUCUUUGCA 3’
           :  .:::::::::::::
query   3’ CUCUGCGGCAAGAAACGG 5’

query=JS16_t0584414, target=FGSG_11208, score=4, range=1622-1639, strand=1

target  5’ GAGAUGUCGUUCUUUGGU 3’
           ::::.:.::::::::: .
query   3’ CUCUGCGGCAAGAAACGG 5’

query=JS16_t0584414, target=FGSG_02983, score=4, range=701-718, strand=1

target  5’ UCUACGCCGUUUUUUGCC 3’
              ::::::::.::::::
query   3’ CUCUGCGGCAAGAAACGG 5’

query=JS16_t0584414, target=FGSG_07504, score=4, range=1027-1044, strand=1

target  5’ GAGAGGCUGUUCUUCGCC 3’
           :::: ::.:::::: :::
query   3’ CUCUGCGGCAAGAAACGG 5’

query=JS16_t0584414, target=FGSG_00933, score=4, range=89-105, strand=1

target  5’ UAGGCGCCGUUC-UUGCU 3’
            ::.:::::::: ::::.
query   3’ CUCUGCGGCAAGAAACGG 5’

query=JS16_t0584414, target=FGSG_00220, score=4, range=1558-1576, strand=1

target  5’ AAGAACGCCGUUCUUAGCC 3’
            :: ::::::::::: :::
query   3’ CUC-UGCGGCAAGAAACGG 5’
```

```
query   3' AAUAUUAUGGCUCGAUCU 5'

No results for JS16_t2229962
No results for JS16_t0335416
No results for JS16_t4022794
No results for JS16_t2230008
No results for JS16_t2230019
No results for JS16_t0268697
No results for JS16_t4022974
query=JS16_t2230245, target=FGSG_08856, score=3, range=509-527, strand=1

target  5' UCUUUGUUGGCAACUUGCC 3'
           :: :::.:.::::::::::
query   3' AGUAACGAUCGUUGAACGG 5'

query=JS16_t2230245, target=FGSG_09331, score=3, range=317-335, strand=-1

target  5' UAAUUGCAAGCAACUUGCC 3'
           : ::::: :::::::::::
query   3' AGUAACGAUCGUUGAACGG 5'

query=JS16_t2230245, target=FGSG_07167, score=3, range=1354-1373, strand=-1

target  5' CGAUUGCCUAGCAACUUGCC 3'
             :::: :::::::::::::
query   3' AGUAAC-GAUCGUUGAACGG 5'

query=JS16_t2230245, target=FGSG_00667, score=4, range=1499-1517, strand=1

target  5' GUGUUGUUAGCAACUUGCA 3'
            ..:::.::::::::::::
query   3' AGUAACGAUCGUUGAACGG 5'

query=JS16_t2230245, target=FGSG_11395, score=4, range=2724-2742, strand=1

target  5' CUAUUUCUGGCAACUUGCU 3'
            .::: ::.:::::::::.
query   3' AGUAACGAUCGUUGAACGG 5'

query=JS16_t2230245, target=FGSG_11097, score=4, range=467-485, strand=1

target  5' CUGUCGCUAGCGACUUGCC 3'
            ..: ::::::.:::::::
query   3' AGUAACGAUCGUUGAACGG 5'

query=JS16_t2230245, target=FGSG_09115, score=4, range=713-731, strand=1

target  5' UCAUGGGUAGCAACUUGCG 3'
           :::: : :::::::::::
query   3' AGUAACGAUCGUUGAACGG 5'

query=JS16_t2230245, target=FGSG_06545, score=4, range=1419-1436, strand=1

target  5' UC-UUGCUAGCAACUCGCA 3'
           :: ::::::::::::: ::
query   3' AGUAACGAUCGUUGAACGG 5'

query=JS16_t2230245, target=FGSG_12035, score=4, range=3272-3290, strand=-1

target  5' CCGUUGUUGGCAACUUGCU 3'
            :.:::.:.:::::::::.
query   3' AGUAACGAUCGUUGAACGG 5'

query=JS16_t2230245, target=FGSG_08444, score=4, range=871-889, strand=-1

target  5' UCAGAGCUAGCAGCUUGUC 3'
           :::  :::::::.::::.:
query   3' AGUAACGAUCGUUGAACGG 5'

query=JS16_t2230245, target=FGSG_09146, score=4, range=3053-3070, strand=-1

target  5' UCA-UGAUAGCAACUUGCA 3'
           ::: :: :::::::::::
query   3' AGUAACGAUCGUUGAACGG 5'

query=JS16_t2230245, target=FGSG_02785, score=4, range=832-851, strand=-1

target  5' GCAUCGUCUAGCAACUUGCG 3'
            ::: : :::::::::::::
query   3' AGUAAC-GAUCGUUGAACGG 5'

query=JS16_t2230245, target=FGSG_04197, score=4, range=608-627, strand=-1

target  5' GCACUGGCUAGCAACUUGCA 3'
            :: : :::::::::::::
query   3' AGU-AACGAUCGUUGAACGG 5'

query=JS16_t2230245, target=FGSG_06702, score=4, range=514-533, strand=-1

target  5' UCAGUGGUAGCAACCUUGCC 3'
           ::: :: :::::: ::::::
query   3' AGUAACGAUCGUU-GAACGG 5'

query=JS16_t0584414, target=FGSG_03154, score=4, range=1002-1018, strand=1

target  5' GAGG-GCCGCUCUUUGCU 3'
           :::. :::: :::::::.
query   3' CUCUGCGGCAAGAAACGG 5'

query=JS16_t0584414, target=FGSG_05627, score=4, range=1523-1539, strand=1

target  5' GUGGUGCC-UUCUUUGCC 3'
           : :..::: :::::::::
query   3' CUCUGCGGCAAGAAACGG 5'

query=JS16_t0584414, target=FGSG_00566, score=4, range=81-98, strand=-1

target  5' GAGAAGUUGUUCUUUGCA 3'
           :::: :..::::::::::
query   3' CUCUGCGGCAAGAAACGG 5'

query=JS16_t0584414, target=FGSG_01481, score=4, range=256-273, strand=-1

target  5' GAGAAGCUGCUCUUUGCC 3'
           :::: ::.: ::::::::
query   3' CUCUGCGGCAAGAAACGG 5'

query=JS16_t0584414, target=FGSG_04316, score=4, range=1083-1101, strand=-1

target  5' GUGAUGCCGUUCCUUUGCU 3'
           : ::.:::::: ::::::.
query   3' CUCUGCGGCAA-GAAACGG 5'

query=JS16_t0584414, target=FGSG_10451, score=4, range=1292-1308, strand=-1

target  5' GA-ACGCCGUUUUUUGUA 3'
           :: ::::::::.::::.
query   3' CUCUGCGGCAAGAAACGG 5'

query=JS16_t0584414, target=FGSG_06058, score=4, range=618-635, strand=-1

target  5' GAGCCGCCAUUCUUUGCA 3'
           ::: :::: :::::::::
query   3' CUCUGCGGCAAGAAACGG 5'

query=JS16_t0584414, target=FGSG_11691, score=4, range=204-221, strand=-1

target  5' GAGUCGCGGUUCUUUGCA 3'
           ::: ::: ::::::::::
query   3' CUCUGCGGCAAGAAACGG 5'

query=JS16_t0584414, target=FGSG_10006, score=4, range=540-558, strand=-1

target  5' CAGCACGCCCUUCUUUGCC 3'
            :: ::::: :::::::::
query   3' CUC-UGCGGCAAGAAACGG 5'

query=JS16_t0584414, target=FGSG_08177, score=4, range=871-887, strand=-1

target  5' GAGACGCCCUUC-UUGCC 3'
           :::::::: ::: :::::
query   3' CUCUGCGGCAAGAAACGG 5'

query=JS16_t0584414, target=FGSG_12003, score=4, range=1369-1385, strand=-1

target  5' GAGACGCCG-UCUUUGAC 3'
           ::::::::: :::::: :
query   3' CUCUGCGGCAAGAAACGG 5'

query=JS16_t0584414, target=FGSG_02666, score=4, range=511-527, strand=-1

target  5' AAGAGGCCGUUC-UUGCC 3'
            ::: ::::::: :::::
query   3' CUCUGCGGCAAGAAACGG 5'

query=JS16_t0584414, target=FGSG_10006, score=4, range=819-835, strand=-1

target  5' UAGA-GCUGUUCUUUGCA 3'
            ::: ::.::::::::::
query   3' CUCUGCGGCAAGAAACGG 5'

query=JS16_t2294330, target=FGSG_08193, score=4, range=229-250, strand=-1

target  5' UCUU-CAUGUCCUUGAUGACUUU 3'
           ::::   ..::::::::::::::
query   3' AGAAUCCGUAGGAACUACUGAAA 5'

No results for JS16_t4087265
query=JS16_t0693074, target=FGSG_06885, score=3.5, range=2416-2435, strand=1

target  5' GCCAAGGGUAUCCUGCAUUA 3'
           : ::::..:::::::::::
query   3' CAGUUCUUAUAGGACGUAAA 5'

query=JS16_t0693074, target=FGSG_03485, score=3.5, range=591-609, strand=-1
```

```
query=JS16_t2230245, target=FGSG_06111, score=4, range=1292-1309, strand=-1

target  5’ UCUUUGCU-GCAACUUGCG 3’
           :: ::::: :::::::::
query   3’ AGUAACGAUCGUUGAACGG 5’

query=JS16_t3126592, target=FGSG_09960, score=4, range=1549-1569, strand=1

target  5’ GCAUUCCUUGU-GGUUGCAGCG 3’
           ::::::::..: ::::::::.
query   3’ CGUAAGGAGUAGCCAACGUCGU 5’

query=JS16_t0467727, target=FGSG_12867, score=2, range=233-250, strand=-1

target  5’ CUAGUCUUAGCUUUUCGA 3’
            : :::::::::::::::
query   3’ CAGCAGAAUCGAAAAGCU 5’

query=JS16_t0467727, target=FGSG_04425, score=3, range=1268-1285, strand=-1

target  5’ GUCGUCUUAGUUGUUCGA 3’
           ::::::::::.: :::::
query   3’ CAGCAGAAUCGAAAAGCU 5’

query=JS16_t0467727, target=FGSG_07974, score=3, range=1125-1142, strand=-1

target  5’ GUGGCCUUGGCUUUUCGA 3’
           :: : :::.:::::::::
query   3’ CAGCAGAAUCGAAAAGCU 5’

query=JS16_t0467727, target=FGSG_13491, score=3, range=361-377, strand=-1

target  5’ GUCG-CUUGGCUUUUCGC 3’
           :::: :::.::::::::
query   3’ CAGCAGAAUCGAAAAGCU 5’

query=JS16_t0467727, target=FGSG_01558, score=3.5, range=677-694, strand=-1

target  5’ GUGGUUUUGGCUUUUCGG 3’
           :: ::.::.::::::::.
query   3’ CAGCAGAAUCGAAAAGCU 5’

query=JS16_t0467727, target=FGSG_02507, score=3.5, range=955-971, strand=-1

target  5’ GUU-UCUUGGCUUUUUGA 3’
           ::. ::::.::::::.::
query   3’ CAGCAGAAUCGAAAAGCU 5’

query=JS16_t0467727, target=FGSG_13860, score=4, range=54-71, strand=1

target  5’ AUGGUCUUGGCUUUUUGA 3’
            : ::::.::::::.::
query   3’ CAGCAGAAUCGAAAAGCU 5’

query=JS16_t0467727, target=FGSG_06069, score=4, range=781-798, strand=1

target  5’ GGCGUCUUAGCUGUUCGU 3’
           : :::::::::: ::::
query   3’ CAGCAGAAUCGAAAAGCU 5’

query=JS16_t0467727, target=FGSG_07502, score=4, range=1187-1204, strand=1

target  5’ GUAGUCUUACCUUUUCGC 3’
           :: :::::: :::::::
query   3’ CAGCAGAAUCGAAAAGCU 5’

query=JS16_t0467727, target=FGSG_08858, score=4, range=32-48, strand=1

target  5’ GUC-UUUUACCUUUUCGA 3’
           ::: :.::: ::::::::
query   3’ CAGCAGAAUCGAAAAGCU 5’

query=JS16_t0467727, target=FGSG_10475, score=4, range=759-776, strand=1

target  5’ GUCAGCUUCGCUUUUCGA 3’
           :::  ::: :::::::::
query   3’ CAGCAGAAUCGAAAAGCU 5’

query=JS16_t0467727, target=FGSG_04265, score=4, range=213-230, strand=-1

target  5’ CUCUUCUUGGUUUUUCGA 3’
            :: ::::.:.:::::::
query   3’ CAGCAGAAUCGAAAAGCU 5’

query=JS16_t0467727, target=FGSG_00352, score=4, range=864-882, strand=-1

target  5’ GUCCGUCUUAGCUUUUUUA 3’
           :: ::::::::::::. :
query   3’ CA-GCAGAAUCGAAAAGCU 5’

query=JS16_t0467727, target=FGSG_08658, score=4, range=1604-1620, strand=-1

target  5’ GUCAACGAUAUCCU-CAUUU 3’
           ::::: .::::::: :::::
query   3’ CAGUUCUUAUAGGACGUAAA 5’

query=JS16_t0693074, target=FGSG_07246, score=4, range=344-362, strand=1

target  5’ CUCCAG-AUAUUCUGCAUUU 3’
            :: :: ::::.::::::::
query   3’ CAGUUCUUAUAGGACGUAAA 5’

query=JS16_t0693074, target=FGSG_05042, score=4, range=749-768, strand=-1

target  5’ CUCAAGAAUGUCCUUCAUUU 3’
            ::::::::.:::: :::::
query   3’ CAGUUCUUAUAGGACGUAAA 5’

query=JS16_t0693074, target=FGSG_05542, score=4, range=1703-1722, strand=-1

target  5’ GGCGAGGAUAUCCUGCUUUU 3’
           : :.::.::::::::: :::
query   3’ CAGUUCUUAUAGGACGUAAA 5’

query=JS16_t0693074, target=FGSG_01451, score=4, range=615-633, strand=-1

target  5’ GUCAA-AGUAUCCUGGAUUU 3’
           ::::: :.::::::: ::::
query   3’ CAGUUCUUAUAGGACGUAAA 5’

No results for JS16_t0284123
No results for JS16_t3190658
No results for JS16_t1398352
query=JS16_t0802688, target=FGSG_11143, score=4, range=594-617, strand=-1

target  5’ UUCACGUACGGGUCGUUGGUUUUG 3’
           ::::::.::. :::.:::.:::::
query   3’ AAGUGCGUGUGCAGUAACUAAAAC 5’

No results for JS16_t1398355
query=JS16_t0396392, target=FGSG_01490, score=4, range=890-910, strand=1

target  5’ AGUUGGCUCAGAG-AAAUGCAA 3’
           ::.::.::::::: :::::::
query   3’ UCGACUGAGUCUCUUUUACGUG 5’

query=JS16_t3190770, target=FGSG_05424, score=2.5, range=643-660, strand=-1

target  5’ CGUGAUGUUUUCGACAUG 3’
            .:::::.::::::::::
query   3’ UUACUACGAAAGCUGUAC 5’

query=JS16_t3190770, target=FGSG_03194, score=3, range=580-597, strand=1

target  5’ ACUGAUGCUUUCGAUAUC 3’
           : ::::::::::::.::
query   3’ UUACUACGAAAGCUGUAC 5’

query=JS16_t3190770, target=FGSG_09527, score=3, range=2737-2754, strand=1

target  5’ UUUGAUGCUUUCGACAUU 3’
             :::::::::::::::
query   3’ UUACUACGAAAGCUGUAC 5’

query=JS16_t3190770, target=FGSG_02645, score=3, range=2290-2307, strand=-1

target  5’ ACCGAUGCUUUCGACAUC 3’
           :  ::::::::::::::
query   3’ UUACUACGAAAGCUGUAC 5’

query=JS16_t3190770, target=FGSG_04752, score=3.5, range=784-801, strand=1

target  5’ AUUGGUGGUUUCGACAUG 3’
           : ::.:: ::::::::::
query   3’ UUACUACGAAAGCUGUAC 5’

query=JS16_t3190770, target=FGSG_05038, score=3.5, range=121-138, strand=1

target  5’ GAGGAUGCUUUCGACACG 3’
           .: ::::::::::::: :
query   3’ UUACUACGAAAGCUGUAC 5’

query=JS16_t3190770, target=FGSG_13979, score=3.5, range=398-415, strand=1

target  5’ ACUGGGGCUUUCGACAUG 3’
           : ::. ::::::::::::
query   3’ UUACUACGAAAGCUGUAC 5’

query=JS16_t3190770, target=FGSG_06311, score=3.5, range=1621-1639, strand=1

target  5’ AUUGGUGCUUUCGACAAUG 3’
           : ::.:::::::::: :::
query   3’ UUACUACGAAAGCUG-UAC 5’
```

```
target  5’ CUC-UCUUGGUUUUUCGA 3’
             :: ::::.:.:::::::
query   3’ CAGCAGAAUCGAAAAGCU 5’

query=JS16_t0467727, target=FGSG_11655, score=4, range=676-693, strand=-1

target  5’ GACAUCUUGGCUUUUCGC 3’
           : : ::::.::::::::
query   3’ CAGCAGAAUCGAAAAGCU 5’

query=JS16_t0467727, target=FGSG_08009, score=4, range=47-63, strand=-1

target  5’ UUCGUCUU-GCUUUUCGC 3’
            ::::::: ::::::::
query   3’ CAGCAGAAUCGAAAAGCU 5’

query=JS16_t0467727, target=FGSG_11831, score=4, range=221-237, strand=-1

target  5’ AUCGU-UUAGCUUUUCGU 3’
            :::: ::::::::::
query   3’ CAGCAGAAUCGAAAAGCU 5’

query=JS16_t0467727, target=FGSG_04102, score=4, range=1355-1373, strand=-1

target  5’ GUGUUCUUGAGCUUUUCGA 3’
           ::  :::: ::::::::::
query   3’ CAGCAGAA-UCGAAAAGCU 5’

query=JS16_t0467727, target=FGSG_05211, score=4, range=275-291, strand=-1

target  5’ CUC-UCUUUGCUUUUCGA 3’
            :: :::: :::::::::
query   3’ CAGCAGAAUCGAAAAGCU 5’

No results for JS16_t1333978
No results for JS16_t1334008
No results for JS16_t3126720
query=JS16_t1334043, target=FGSG_02669, score=3.5, range=136-155, strand=1

target  5’ CGUUUGACCGAUGCCGGAUA 3’
           :.  : ::::::::::::::
query   3’ GU-AUCUGGCUACGGCCUAA 5’

query=JS16_t1334043, target=FGSG_01166, score=4, range=398-416, strand=1

target  5’ UAUAUGCCGAUGCCGUAUU 3’
           .::: .:::::::::: :::
query   3’ GUAUCUGGCUACGGCCUAA 5’

query=JS16_t1334043, target=FGSG_11468, score=4, range=145-164, strand=1

target  5’ CAUGACAUCGAUGCUGGAUU 3’
           ::: : :.::::::.:::::
query   3’ GUA-UCUGGCUACGGCCUAA 5’

query=JS16_t1334043, target=FGSG_10451, score=4, range=1109-1127, strand=-1

target  5’ GGGAGGCCGAUGCCGGAUG 3’
            .  ::.:::::::::::
query   3’ GUAUCUGGCUACGGCCUAA 5’

query=JS16_t1334043, target=FGSG_01762, score=4, range=1156-1173, strand=-1

target  5’ CG-AGGUCGAUGCCGGAUA 3’
           :.  ::..::::::::::
query   3’ GUAUCUGGCUACGGCCUAA 5’

query=JS16_t1334043, target=FGSG_10046, score=4, range=2264-2283, strand=-1

target  5’ CAGACGACUGAUGCCGGAUG 3’
           :: : :::.::::::::::
query   3’ GUAU-CUGGCUACGGCCUAA 5’

query=JS16_t4023267, target=FGSG_08738, score=3.5, range=2638-2657, strand=1

target  5’ GUUGGGCAAAAGAAAGC-UGA 3’
           :::...:::::::::: :::
query   3’ CAAUUUGUUUUCUUUCGAACU 5’

query=JS16_t4023267, target=FGSG_13168, score=4, range=439-459, strand=-1

target  5’ GAUAGCCCAAAGAAAGCUUGG 3’
           : ::. : ::::::::::::.
query   3’ CAAUUUGUUUUCUUUCGAACU 5’

query=JS16_t4023267, target=FGSG_07280, score=4, range=2106-2125, strand=-1

target  5’ GGUCAAC-AAGGAAAGCUUGA 3’
           : : ::: ::.::::::::::
query   3’ CAAUUUGUUUUCUUUCGAACU 5’

query=JS16_t3190770, target=FGSG_08119, score=3.5, range=1085-1101, strand=1

target  5’ AGUG-UCCUUUCGACAUG 3’
           :.:: : :::::::::::
query   3’ UUACUACGAAAGCUGUAC 5’

query=JS16_t3190770, target=FGSG_03608, score=3.5, range=1185-1202, strand=1

target  5’ GUUCUUGCUUUCGACAUG 3’
           . :  :::::::::::::
query   3’ UUACUACGAAAGCUGUAC 5’

query=JS16_t3190770, target=FGSG_06997, score=3.5, range=2268-2285, strand=-1

target  5’ GAGGAUGCUUUUGGCAUG 3’
           .: ::::::::.:.::::
query   3’ UUACUACGAAAGCUGUAC 5’

query=JS16_t3190770, target=FGSG_00697, score=3.5, range=277-294, strand=-1

target  5’ AAUAGUGCUUUCGGCAUU 3’
           ::: .::::::::.:::
query   3’ UUACUACGAAAGCUGUAC 5’

query=JS16_t3190770, target=FGSG_05929, score=3.5, range=1747-1764, strand=-1

target  5’ GAGGAUGCUUUCCACAUG 3’
           .: ::::::::: :::::
query   3’ UUACUACGAAAGCUGUAC 5’

query=JS16_t3190770, target=FGSG_03204, score=3.5, range=657-674, strand=-1

target  5’ AAUCGUGCUUUCGACACG 3’
           ::: .::::::::::: :
query   3’ UUACUACGAAAGCUGUAC 5’

query=JS16_t3190770, target=FGSG_12273, score=3.5, range=1650-1668, strand=-1

target  5’ GAUGACCGCUUUCGACAUG 3’
           .::::  ::::::::::::
query   3’ UUACU-ACGAAAGCUGUAC 5’

query=JS16_t3190770, target=FGSG_05066, score=4, range=610-627, strand=1

target  5’ GAUGGUGUUUUCGAAAUG 3’
           .:::.::.:::::: :::
query   3’ UUACUACGAAAGCUGUAC 5’

query=JS16_t3190770, target=FGSG_10152, score=4, range=325-342, strand=1

target  5’ AGUGGUGCUUUCGACAAC 3’
           :.::.:::::::::::
query   3’ UUACUACGAAAGCUGUAC 5’

query=JS16_t3190770, target=FGSG_10665, score=4, range=1270-1287, strand=1

target  5’ AAUGAAGCUUUUGACAUA 3’
           ::::: :::::.:::::
query   3’ UUACUACGAAAGCUGUAC 5’

query=JS16_t3190770, target=FGSG_06075, score=4, range=112-130, strand=1

target  5’ AAUGAUGCUUCCGACGAUG 3’
           :::::::::: :::: :::
query   3’ UUACUACGAAAGCUG-UAC 5’

query=JS16_t3190770, target=FGSG_09838, score=4, range=2647-2665, strand=1

target  5’ AAUGAUGCUUUCGAACAGG 3’
           ::::::::::::: ::: :
query   3’ UUACUACGAAAGC-UGUAC 5’

query=JS16_t3190770, target=FGSG_07258, score=4, range=12346-12363, strand=1

target  5’ AAGGAUGCUGUCGAUAUG 3’
           :: :::::: ::::.:::
query   3’ UUACUACGAAAGCUGUAC 5’

query=JS16_t3190770, target=FGSG_03177, score=4, range=604-621, strand=1

target  5’ AAAGAUGAUUUUGACAUG 3’
           :: :::: :::.::::::
query   3’ UUACUACGAAAGCUGUAC 5’

query=JS16_t3190770, target=FGSG_02160, score=4, range=301-319, strand=1

target  5’ AUUGAUGCUUUUGACAAUG 3’
           : :::::::::.::: :::
query   3’ UUACUACGAAAGCUG-UAC 5’

query=JS16_t3190770, target=FGSG_10332, score=4, range=199-216, strand=1
```

```
query=JS16_t3126754, target=FGSG_09707, score=2, range=206-226, strand=-1

target  5’ UAAGCGUCAACUUGACAGAGC 3’
           :::: ::::::::::::::::
query   3’ AUUC-CAGUUGAACUGUCUCC 5’

query=JS16_t3126754, target=FGSG_02547, score=2.5, range=582-601, strand=1

target  5’ CGACGUCAACUUGACAGAGG 3’
            .: ::::::::::::::::
query   3’ AUUCCAGUUGAACUGUCUCC 5’

query=JS16_t3126754, target=FGSG_03166, score=3.5, range=398-417, strand=-1

target  5’ GAGGCUGAACUUGACAGAGG 3’
            :.: : ::::::::::::
query   3’ AUUCCAGUUGAACUGUCUCC 5’

query=JS16_t3126754, target=FGSG_08141, score=4, range=1980-1999, strand=1

target  5’ UCAGGGCAACUUGACACAGG 3’
           : ::: :::::::::: :::
query   3’ AUUCCAGUUGAACUGUCUCC 5’

query=JS16_t3126754, target=FGSG_12228, score=4, range=268-286, strand=-1

target  5’ UCAGGUCAACUUGA-AGAGC 3’
           : :::::::::::: ::::
query   3’ AUUCCAGUUGAACUGUCUCC 5’

No results for JS16_t0268704
query=JS16_t4023297, target=FGSG_01228, score=2.5, range=181-201, strand=-1

target  5’ GUCAGUAGAGGUGUUUGGCUC 3’
           ::: :. :::::::::::::
query   3’ CAGCCG-CUCCACAAACCGAG 5’

query=JS16_t4023297, target=FGSG_05008, score=3, range=733-752, strand=-1

target  5’ AUCGUCGAGGUGUUUGGUUC 3’
            ::: ::::::::::::.::
query   3’ CAGCCGCUCCACAAACCGAG 5’

query=JS16_t4023297, target=FGSG_06621, score=4, range=305-324, strand=1

target  5’ GUCACCGAGGUGUUUGGCCC 3’
           :::  ::::::::::::: :
query   3’ CAGCCGCUCCACAAACCGAG 5’

query=JS16_t4023297, target=FGSG_03875, score=4, range=99-117, strand=1

target  5’ GUCAG-GAGGUGGUUGGCUC 3’
           ::: : :::::: :::::::
query   3’ CAGCCGCUCCACAAACCGAG 5’

query=JS16_t4023297, target=FGSG_00887, score=4, range=1632-1652, strand=-1

target  5’ GUCGGCGAGGUGUUCUUGCUC 3’
           :::::::::::::: : ::::
query   3’ CAGCCGCUCCACAA-ACCGAG 5’

query=JS16_t1334090, target=FGSG_05634, score=4, range=1681-1703, strand=1

target  5’ UGGGCAGUGUUUCUUGCAAAAUG 3’
           :.:::: .:::::::: ::::::
query   3’ AUCCGUAGCAAAGAACCUUUUAC 5’

No results for JS16_t3126807
query=JS16_t2230545, target=FGSG_13358, score=3, range=2007-2023, strand=1

target  5’ UC-CUUUUAGACGCAAGA 3’
           :: :::::::::: ::::
query   3’ AGAGAAAAUCUGCUUUCU 5’

query=JS16_t2230545, target=FGSG_08798, score=3, range=1053-1069, strand=1

target  5’ UC-CUUUUAUACGAAAGA 3’
           :: :::::: ::::::::
query   3’ AGAGAAAAUCUGCUUUCU 5’

query=JS16_t2230545, target=FGSG_08690, score=3, range=1240-1257, strand=-1

target  5’ ACUGUUUUGGACGAAAGA 3’
            :: ::::.:::::::::
query   3’ AGAGAAAAUCUGCUUUCU 5’

query=JS16_t2230545, target=FGSG_08129, score=3, range=1634-1651, strand=-1

target  5’ UGUCUUUUAGACGAACGA 3’
           : ::::::::::::: ::
query   3’ AGAGAAAAUCUGCUUUCU 5’

target  5’ CAUGAUGCUUUCGAGAUU 3’
            ::::::::::::: ::
query   3’ UUACUACGAAAGCUGUAC 5’

query=JS16_t3190770, target=FGSG_08144, score=4, range=2215-2232, strand=1

target  5’ AUUGCUGUUUUCGAUAUG 3’
           : :: ::.::::::.:::
query   3’ UUACUACGAAAGCUGUAC 5’

query=JS16_t3190770, target=FGSG_11788, score=4, range=208-225, strand=1

target  5’ AAUUCUGCUUUUGACGUG 3’
           :::  ::::::.:::.::
query   3’ UUACUACGAAAGCUGUAC 5’

query=JS16_t3190770, target=FGSG_11703, score=4, range=496-514, strand=1

target  5’ CAAGAUGACUUUCGACAUG 3’
            : :::: :::::::::::
query   3’ UUACUAC-GAAAGCUGUAC 5’

query=JS16_t3190770, target=FGSG_10158, score=4, range=756-773, strand=1

target  5’ AAUCAUGCUUUCAACAUC 3’
           ::: :::::::: ::::
query   3’ UUACUACGAAAGCUGUAC 5’

query=JS16_t3190770, target=FGSG_08236, score=4, range=147-164, strand=1

target  5’ CAAGAUUCUUUCGACAUG 3’
            : ::: :::::::::::
query   3’ UUACUACGAAAGCUGUAC 5’

query=JS16_t3190770, target=FGSG_09608, score=4, range=880-897, strand=1

target  5’ AACUAUGCUUUCGAUAUC 3’
           ::  ::::::::::.::
query   3’ UUACUACGAAAGCUGUAC 5’

query=JS16_t3190770, target=FGSG_11680, score=4, range=1102-1118, strand=1

target  5’ AAUCAUGUUUUCG-CAUG 3’
           ::: :::.::::: ::::
query   3’ UUACUACGAAAGCUGUAC 5’

query=JS16_t3190770, target=FGSG_02282, score=4, range=398-414, strand=1

target  5’ UAU-AUGCUCUCGACAUG 3’
            :: ::::: ::::::::
query   3’ UUACUACGAAAGCUGUAC 5’

query=JS16_t3190770, target=FGSG_09223, score=4, range=665-681, strand=1

target  5’ GCUGGUG-UUUCGACAUG 3’
           .  ::.:: ::::::::::
query   3’ UUACUACGAAAGCUGUAC 5’

query=JS16_t3190770, target=FGSG_00343, score=4, range=1417-1434, strand=-1

target  5’ GAUGGUGCUAUCGGCAUG 3’
           .:::.:::: :::.::::
query   3’ UUACUACGAAAGCUGUAC 5’

query=JS16_t3190770, target=FGSG_05701, score=4, range=178-195, strand=-1

target  5’ AAUGAAGUUUUCGACAUC 3’
           ::::: :.:::::::::
query   3’ UUACUACGAAAGCUGUAC 5’

query=JS16_t3190770, target=FGSG_02152, score=4, range=1152-1169, strand=-1

target  5’ CGAGGUGUUUUCGACAUG 3’
            .  :.::.::::::::::
query   3’ UUACUACGAAAGCUGUAC 5’

query=JS16_t3190770, target=FGSG_05245, score=4, range=235-252, strand=-1

target  5’ GACGGUGCUUUCGGCAUU 3’
           .: :.::::::::.:::
query   3’ UUACUACGAAAGCUGUAC 5’

query=JS16_t3190770, target=FGSG_10470, score=4, range=77-93, strand=-1

target  5’ AGUGGUGUUUUCGA-AUG 3’
           :.::.::.:::::: :::
query   3’ UUACUACGAAAGCUGUAC 5’

query=JS16_t3190770, target=FGSG_07518, score=4, range=3523-3540, strand=-1

target  5’ AAGGAUGUUUUCGACAGG 3’
```

```
query=JS16_t2230545, target=FGSG_10000, score=3.5, range=1029-1046, strand=-1

target  5’ UCUUUUGUAGGCGAAAGA 3’
           :::.:: :::.:::::::
query   3’ AGAGAAAAUCUGCUUUCU 5’

query=JS16_t2230545, target=FGSG_10257, score=4, range=100-117, strand=1

target  5’ UCUCUUCUAGACGAAACA 3’
           :::::: ::::::::: :
query   3’ AGAGAAAAUCUGCUUUCU 5’

query=JS16_t2230545, target=FGSG_08263, score=4, range=1326-1344, strand=1

target  5’ GCUGCUUUUAUACGAAAGA 3’
            :: :::::: ::::::::
query   3’ AGA-GAAAAUCUGCUUUCU 5’

query=JS16_t2230545, target=FGSG_08795, score=4, range=1747-1763, strand=1

target  5’ UCUC-UUUAUAUGAAAGA 3’
           :::: :::: :.::::::
query   3’ AGAGAAAAUCUGCUUUCU 5’

query=JS16_t2230545, target=FGSG_08155, score=4, range=3103-3119, strand=1

target  5’ UUGCUUUUAGA-GAAAGG 3’
           :. :::::::: :::::.
query   3’ AGAGAAAAUCUGCUUUCU 5’

query=JS16_t2230545, target=FGSG_07279, score=4, range=55-72, strand=-1

target  5’ UUUCUUUGAGACGAAGGG 3’
           :.::::: :::::::.:.
query   3’ AGAGAAAAUCUGCUUUCU 5’

query=JS16_t2230545, target=FGSG_12380, score=4, range=87-104, strand=-1

target  5’ UCUUGUUUGGACGAAGGG 3’
           :::. :::.::::::.:.
query   3’ AGAGAAAAUCUGCUUUCU 5’

query=JS16_t2230545, target=FGSG_11181, score=4, range=2185-2202, strand=-1

target  5’ ACUGUUUUGGACGAGAGA 3’
            :: ::::.:::::.:::
query   3’ AGAGAAAAUCUGCUUUCU 5’

query=JS16_t2230545, target=FGSG_09404, score=4, range=288-305, strand=-1

target  5’ ACACUUUUAGGCGGAAGA 3’
            : :::::::.::.::::
query   3’ AGAGAAAAUCUGCUUUCU 5’

query=JS16_t2230545, target=FGSG_06089, score=4, range=5206-5223, strand=-1

target  5’ UCUCCUAUAGAUGAAAGA 3’
           :::: : ::::.::::::
query   3’ AGAGAAAAUCUGCUUUCU 5’

query=JS16_t2230545, target=FGSG_08278, score=4, range=593-611, strand=-1

target  5’ UCAUCUUUUGGUCGAAAGA 3’
           :: :::::::.: :::::::
query   3’ AG-AGAAAAUCUGCUUUCU 5’

query=JS16_t2230545, target=FGSG_05362, score=4, range=386-404, strand=-1

target  5’ UCUCUGUGUAGACGGAAGA 3’
           ::::: : :::::::.::::
query   3’ AGAGA-AAAUCUGCUUUCU 5’

query=JS16_t2230545, target=FGSG_11044, score=4, range=32-49, strand=-1

target  5’ CGUCCUUUAGACGGAAGA 3’
             :: ::::::::.::::
query   3’ AGAGAAAAUCUGCUUUCU 5’

query=JS16_t2230545, target=FGSG_12014, score=4, range=561-578, strand=-1

target  5’ CCACUUUUAUACGAAAGA 3’
            : :::::: ::::::::
query   3’ AGAGAAAAUCUGCUUUCU 5’

query=JS16_t0131168, target=FGSG_03016, score=3, range=290-308, strand=1

target  5’ AUCUUGCGCGACAAGCGGA 3’
           ::::::::::::::: ::
query   3’ UAGAACGCGCUGUUCACCA 5’

query=JS16_t0131168, target=FGSG_03962, score=3, range=823-840, strand=1
           :: ::::.:::::::: :
query   3’ UUACUACGAAAGCUGUAC 5’

query=JS16_t3190770, target=FGSG_06680, score=4, range=211-228, strand=-1

target  5’ AAGGAUGUAUUCGACAUG 3’
           :: ::::. :::::::::
query   3’ UUACUACGAAAGCUGUAC 5’

query=JS16_t3190770, target=FGSG_10342, score=4, range=1075-1092, strand=-1

target  5’ AAAGAUGCUUUCGACACU 3’
           :: :::::::::::::
query   3’ UUACUACGAAAGCUGUAC 5’

query=JS16_t3190770, target=FGSG_13441, score=4, range=939-956, strand=-1

target  5’ GAGCGUGCUUUUGACAUG 3’
           .:  .::::::.::::::
query   3’ UUACUACGAAAGCUGUAC 5’

query=JS16_t3190770, target=FGSG_07777, score=4, range=345-361, strand=-1

target  5’ AAU-AUGUUUUUGACAUC 3’
           ::: :::.:::.:::::
query   3’ UUACUACGAAAGCUGUAC 5’

query=JS16_t3190770, target=FGSG_02807, score=4, range=1331-1347, strand=-1

target  5’ AAUGAUGCCUUCGA-AUG 3’
           :::::::: ::::: :::
query   3’ UUACUACGAAAGCUGUAC 5’

query=JS16_t3190770, target=FGSG_13491, score=4, range=140-156, strand=-1

target  5’ ACUGA-GCUUUCGACGUG 3’
           : ::: :::::::::.::
query   3’ UUACUACGAAAGCUGUAC 5’

query=JS16_t3190770, target=FGSG_06507, score=4, range=1287-1303, strand=-1

target  5’ CAAGAUGCUUUCGA-AUG 3’
            : ::::::::::: :::
query   3’ UUACUACGAAAGCUGUAC 5’

query=JS16_t3190770, target=FGSG_05847, score=4, range=412-428, strand=-1

target  5’ AAUGUUGCUUU-GACAUU 3’
           :::: :::::: :::::
query   3’ UUACUACGAAAGCUGUAC 5’

query=JS16_t1398515, target=FGSG_05581, score=4, range=542-564, strand=-1

target  5’ ACAAGUCG-GGUCUGGAUCCGAGU 3’
           :  ::: : :::::::::::::::
query   3’ UUAUCACCGCCAGACCUAGGCUCA 5’

No results for JS16_t0475531
query=JS16_t0337557, target=FGSG_04369, score=2, range=1469-1488, strand=1

target  5’ UUAUAUGUGGUGGUGGAUAU 3’
           :: : :::::::::::::::
query   3’ AAGA-ACACCACCACCUAUA 5’

query=JS16_t0337557, target=FGSG_02630, score=3, range=130-149, strand=-1

target  5’ UUGUUGGUGGUGGUGGAUGU 3’
           :: :: ::::::::::::.:
query   3’ AAGAA-CACCACCACCUAUA 5’

query=JS16_t0337557, target=FGSG_06951, score=3.5, range=1043-1060, strand=-1

target  5’ UUUUGGUGGUGGUGG-UAU 3’
           ::.: :::::::::: :::
query   3’ AAGAACACCACCACCUAUA 5’

query=JS16_t0337557, target=FGSG_06175, score=4, range=672-690, strand=1

target  5’ UCCUUCUGGUGGUGGAGAU 3’
           : ::: :::::::::: ::
query   3’ AAGAACACCACCACCUAUA 5’

query=JS16_t0337557, target=FGSG_11934, score=4, range=478-495, strand=1

target  5’ UUC-GGUGGUGGUGGAUGG 3’
           :::  ::::::::::::.
query   3’ AAGAACACCACCACCUAUA 5’

query=JS16_t0337557, target=FGSG_06992, score=4, range=3713-3732, strand=1

target  5’ UUCUUGUUGGUGGUGGAAAC 3’
           :::::: :::::::::: :
```

```
target  5’ UUCUUGCGCGAC-AGUGGU 3’
            ::::::::::: ::::::
query   3’ UAGAACGCGCUGUUCACCA 5’

query=JS16_t0131168, target=FGSG_03995, score=3.5, range=2026-2045, strand=-1

target  5’ GUCGUUGCGCGACGAGUGGC 3’
           .:: :::::::::.:::::
query   3’ UAG-AACGCGCUGUUCACCA 5’

query=JS16_t0131168, target=FGSG_01964, score=4, range=4933-4951, strand=1

target  5’ AUCUUGCGCCGCAAGUGGG 3’
           ::::::::: .:::::::
query   3’ UAGAACGCGCUGUUCACCA 5’

query=JS16_t0131168, target=FGSG_07667, score=4, range=1016-1034, strand=1

target  5’ AUGUUGCGCGAAAAGUGGA 3’
           :: :::::::: ::::::
query   3’ UAGAACGCGCUGUUCACCA 5’

query=JS16_t0131168, target=FGSG_10659, score=4, range=154-171, strand=1

target  5’ AUCUU-CGCGACUGGUGGU 3’
           ::::: :::::: .:::::
query   3’ UAGAACGCGCUGUUCACCA 5’

query=JS16_t0131168, target=FGSG_06544, score=4, range=1159-1176, strand=1

target  5’ ACCAUGCGCGACAAG-GGU 3’
           : : ::::::::::: :::
query   3’ UAGAACGCGCUGUUCACCA 5’

query=JS16_t0131168, target=FGSG_09774, score=4, range=69-87, strand=-1

target  5’ UUCUGGCGCGACAAGUUGU 3’
            ::: ::::::::::: ::
query   3’ UAGAACGCGCUGUUCACCA 5’

query=JS16_t2230554, target=FGSG_10914, score=3.5, range=539-557, strand=1

target  5’ CGACUCGCCCCGGUAUUCU 3’
           ::: ::::: ::::::::.
query   3’ GCUUAGCGGCGCCAUAAGG 5’

query=JS16_t2230554, target=FGSG_01624, score=4, range=19-36, strand=1

target  5’ CGAGUCGCCGCGG-AUUUU 3’
           :::.::::::::: :::..
query   3’ GCUUAGCGGCGCCAUAAGG 5’

query=JS16_t2230554, target=FGSG_01357, score=4, range=833-851, strand=1

target  5’ CUGGUCGCGGCGGUAUUCC 3’
           : ..:::: ::::::::::
query   3’ GCUUAGCGGCGCCAUAAGG 5’

query=JS16_t2230554, target=FGSG_02965, score=4, range=333-352, strand=1

target  5’ CGUGAUUGCCUCGGUAUUCC 3’
           :: .::.::: :::::::::
query   3’ GC-UUAGCGGCGCCAUAAGG 5’

query=JS16_t2230554, target=FGSG_04875, score=4, range=652-671, strand=1

target  5’ CGAAUCGCAGCGCGUAUUCC 3’
           :::::::: ::: :::::::
query   3’ GCUUAGCGGCGC-CAUAAGG 5’

query=JS16_t2230554, target=FGSG_09746, score=4, range=555-573, strand=-1

target  5’ UGGGUCGCCGCGGUAUCCU 3’
           .:..:::::::::::: :.
query   3’ GCUUAGCGGCGCCAUAAGG 5’

query=JS16_t2230554, target=FGSG_06456, score=4, range=18-37, strand=-1

target  5’ CGGGUGCGCCGUGGUGUUCC 3’
           ::..: :::::.:::.::::
query   3’ GCUUA-GCGGCGCCAUAAGG 5’

query=JS16_t2230554, target=FGSG_06801, score=4, range=327-344, strand=-1

target  5’ UGCA-CGCUGCGGUAUUCU 3’
           .: : :::.:::::::::.
query   3’ GCUUAGCGGCGCCAUAAGG 5’

No results for JS16_t3126823
No results for JS16_t1334150
query=JS16_t2230573, target=FGSG_00771, score=2, range=721-738, strand=1
query   3’ AAGAAC-ACCACCACCUAUA 5’

query=JS16_t0337557, target=FGSG_13700, score=4, range=108-126, strand=-1

target  5’ UUCUUGUGGUGGAGGAGAU 3’
           :::::::::::: ::: ::
query   3’ AAGAACACCACCACCUAUA 5’

query=JS16_t0337557, target=FGSG_03506, score=4, range=458-476, strand=-1

target  5’ AUCUUGUGGUGGUCGAUAG 3’
            :::::::::::: ::::
query   3’ AAGAACACCACCACCUAUA 5’

query=JS16_t0337557, target=FGSG_09474, score=4, range=61-79, strand=-1

target  5’ GACUUUUGGUGGUGGAUGU 3’
             ::: :::::::::::.:
query   3’ AAGAACACCACCACCUAUA 5’

query=JS16_t0337557, target=FGSG_01397, score=4, range=176-194, strand=-1

target  5’ UUCAGGUGGUGGAGGAUAU 3’
           :::  ::::::: ::::::
query   3’ AAGAACACCACCACCUAUA 5’

No results for JS16_t0194920
query=JS16_t0584445, target=FGSG_04401, score=2, range=863-879, strand=1

target  5’ AUUG-GUAUGUGCAGCAG 3’
           :::: :::.:::::::::
query   3’ UAACUCAUGCACGUCGUC 5’

query=JS16_t0584445, target=FGSG_11840, score=3, range=213-231, strand=-1

target  5’ AUUCAGUACGUGCACGCAG 3’
           ::: :::::::::: ::::
query   3’ UAACUCAUGCACGU-CGUC 5’

query=JS16_t0584445, target=FGSG_01378, score=3.5, range=940-958, strand=1

target  5’ AUGGGGUACCGUGCAGCAG 3’
           :: :.::: ::::::::::
query   3’ UAACUCAU-GCACGUCGUC 5’

query=JS16_t0584445, target=FGSG_13135, score=3.5, range=98-114, strand=-1

target  5’ GUUGAGUAUG-GCAGCAG 3’
           .:::::::.: :::::::
query   3’ UAACUCAUGCACGUCGUC 5’

query=JS16_t0584445, target=FGSG_12940, score=4, range=3459-3476, strand=1

target  5’ AUUGAGUAUGAGCGGCAG 3’
           ::::::::.: ::.::::
query   3’ UAACUCAUGCACGUCGUC 5’

query=JS16_t0584445, target=FGSG_11864, score=4, range=117-135, strand=1

target  5’ AUCUGAGUGUGUGCAGCAA 3’
           :: :::::..::::::::
query   3’ UA-ACUCAUGCACGUCGUC 5’

query=JS16_t0584445, target=FGSG_02288, score=4, range=778-795, strand=1

target  5’ AUCGAGUACAUGCAGCAA 3’
           :: :::::: :::::::
query   3’ UAACUCAUGCACGUCGUC 5’

query=JS16_t0584445, target=FGSG_05919, score=4, range=594-610, strand=1

target  5’ AUUG-GUGCUUGCAGCAG 3’
           :::: ::.: ::::::::
query   3’ UAACUCAUGCACGUCGUC 5’

query=JS16_t0584445, target=FGSG_10163, score=4, range=293-311, strand=1

target  5’ AUGUCAGUACAUGCAGCAG 3’
           :: : ::::: ::::::::
query   3’ UA-ACUCAUGCACGUCGUC 5’

query=JS16_t0584445, target=FGSG_09356, score=4, range=49-65, strand=1

target  5’ AUGGAGUACGUGCA-CAA 3’
           :: ::::::::::: ::
query   3’ UAACUCAUGCACGUCGUC 5’

query=JS16_t0584445, target=FGSG_12476, score=4, range=22-38, strand=1

target  5’ AUU-AUUACGUGCAGCAA 3’
           ::: : :::::::::::
query   3’ UAACUCAUGCACGUCGUC 5’
```

```
target  5' GAGACUGCAGUUGAUCGA 3'
           :::: :::::.:::::::
query   3' CUCUUACGUCGACUAGCU 5'

query=JS16_t2230573, target=FGSG_09118, score=2, range=2878-2895, strand=1

target  5' GUGAAUGCAGUUGAUCGA 3'
           : ::::::::.:::::::
query   3' CUCUUACGUCGACUAGCU 5'

query=JS16_t2230573, target=FGSG_02771, score=2.5, range=881-898, strand=-1

target  5' GAGGAUGCUGCUGAUCGA 3'
           :::.:::: :::::::::
query   3' CUCUUACGUCGACUAGCU 5'

query=JS16_t2230573, target=FGSG_06068, score=3, range=1513-1529, strand=1

target  5' GAG-AUGCAGCUGGUUGA 3'
           ::: :::::::::.:.::
query   3' CUCUUACGUCGACUAGCU 5'

query=JS16_t2230573, target=FGSG_10602, score=3, range=788-805, strand=-1

target  5' GGCGAUGUAGCUGAUCGA 3'
           :. .:::.::::::::::
query   3' CUCUUACGUCGACUAGCU 5'

query=JS16_t2230573, target=FGSG_01033, score=3, range=227-243, strand=-1

target  5' GAGAAUGCGGCUGAU-GA 3'
           ::::::::.:::::: ::
query   3' CUCUUACGUCGACUAGCU 5'

query=JS16_t2230573, target=FGSG_08730, score=3, range=826-842, strand=-1

target  5' GGGAAUGCAGCUGA-CGG 3'
           :.:::::::::::: ::.
query   3' CUCUUACGUCGACUAGCU 5'

query=JS16_t2230573, target=FGSG_04385, score=3.5, range=1345-1363, strand=1

target  5' AAGAGUGCAGCUGAUGCGA 3'
            :::.:::::::::: :::
query   3' CUCUUACGUCGACUA-GCU 5'

query=JS16_t2230573, target=FGSG_05512, score=3.5, range=2163-2180, strand=1

target  5' GUGGAUGCAGCCGAUCGA 3'
           : :.::::::: ::::::
query   3' CUCUUACGUCGACUAGCU 5'

query=JS16_t2230573, target=FGSG_08804, score=3.5, range=829-847, strand=1

target  5' GAGAUUGCAGCUGCAUCGG 3'
           :::: :::::::: ::::.
query   3' CUCUUACGUCGAC-UAGCU 5'

query=JS16_t2230573, target=FGSG_02635, score=3.5, range=967-983, strand=1

target  5' GA-AGAGCAGCUGAUCGA 3'
           :: :. ::::::::::::
query   3' CUCUUACGUCGACUAGCU 5'

query=JS16_t2230573, target=FGSG_06595, score=3.5, range=599-616, strand=-1

target  5' GAGGAUUCAGCUGAUUGA 3'
           :::.:: ::::::::.::
query   3' CUCUUACGUCGACUAGCU 5'

query=JS16_t2230573, target=FGSG_09284, score=3.5, range=65-82, strand=-1

target  5' GGGAAUGGAGCUGGUCGA 3'
           :.::::: :::::.::::
query   3' CUCUUACGUCGACUAGCU 5'

query=JS16_t2230573, target=FGSG_05525, score=3.5, range=41-58, strand=-1

target  5' GCUGAUGCAGCUGAUCGU 3'
           :  .:::::::::::::
query   3' CUCUUACGUCGACUAGCU 5'

query=JS16_t2230573, target=FGSG_01355, score=4, range=261-278, strand=1

target  5' GAGGGUGCAGCUGAUGGC 3'
           :::..:::::::::: :
query   3' CUCUUACGUCGACUAGCU 5'

query=JS16_t2230573, target=FGSG_03410, score=4, range=1273-1289, strand=1

target  5' GAGAA-GCAGCUGGUUGA 3'
           ::::: :::::::.:.::
```

```
query=JS16_t0584445, target=FGSG_00704, score=4, range=481-497, strand=1

target  5' AUGGAG-GCGUGCAGCAG 3'
           :: ::: .::::::::::
query   3' UAACUCAUGCACGUCGUC 5'

query=JS16_t0584445, target=FGSG_04120, score=4, range=526-542, strand=1

target  5' AAGGAGUACGUGC-GCAG 3'
           :  :::::::::: ::::
query   3' UAACUCAUGCACGUCGUC 5'

query=JS16_t0584445, target=FGSG_04409, score=4, range=542-558, strand=-1

target  5' GUUGGGUACGUG-GGCAG 3'
           .:::.:::::::: .::::
query   3' UAACUCAUGCACGUCGUC 5'

No results for JS16_t2294577
No results for JS16_t0475536
No results for JS16_t0307763
No results for JS16_t2294634
query=JS16_t1398639, target=FGSG_00794, score=2, range=75-92, strand=-1

target  5' AUUCUGUUGGUAGGGUCG 3'
           ::: :::::.::::::::
query   3' UAAAACAACUAUCCCAGC 5'

query=JS16_t1398639, target=FGSG_04369, score=3, range=1394-1410, strand=1

target  5' AUUUUGUUG-UAGGGUUG 3'
           ::::::::: ::::::.:
query   3' UAAAACAACUAUCCCAGC 5'

query=JS16_t1398639, target=FGSG_02618, score=3, range=318-335, strand=-1

target  5' AUUUUCUUGAUAGGGUCA 3'
           ::::: ::::::::::
query   3' UAAAACAACUAUCCCAGC 5'

query=JS16_t1398639, target=FGSG_07008, score=3, range=859-875, strand=-1

target  5' AUUUUGUUGAUA-GGUUG 3'
           :::::::::::: :::.:
query   3' UAAAACAACUAUCCCAGC 5'

query=JS16_t1398639, target=FGSG_07928, score=3, range=1492-1509, strand=-1

target  5' AUGUGGUUGAUAGGGUCC 3'
           :: : ::::::::::::
query   3' UAAAACAACUAUCCCAGC 5'

query=JS16_t1398639, target=FGSG_06576, score=3, range=675-692, strand=-1

target  5' AGGUGGUUGAUAGGGUCG 3'
           :  : :::::::::::::
query   3' UAAAACAACUAUCCCAGC 5'

query=JS16_t1398639, target=FGSG_04321, score=3.5, range=1773-1790, strand=-1

target  5' GCUUUGCUGAUAGGGUCG 3'
           . :::: :::::::::::
query   3' UAAAACAACUAUCCCAGC 5'

query=JS16_t1398639, target=FGSG_00973, score=4, range=755-772, strand=1

target  5' AUUUAGUUGAUUGGGUUG 3'
           :::: :::::: ::::.:
query   3' UAAAACAACUAUCCCAGC 5'

query=JS16_t1398639, target=FGSG_09694, score=4, range=731-748, strand=1

target  5' CUGCGGUUGAUAGGGUCG 3'
            :   :::::::::::::
query   3' UAAAACAACUAUCCCAGC 5'

query=JS16_t1398639, target=FGSG_00946, score=4, range=233-251, strand=-1

target  5' AUUGUGUUUGAUGGGGUCG 3'
           ::: :: :::::.::::::
query   3' UAAAAC-AACUAUCCCAGC 5'

query=JS16_t1398639, target=FGSG_07395, score=4, range=300-318, strand=-1

target  5' AUCUUUGUUGUUGGGGUCG 3'
           :: ::::::: :.::::::
query   3' UA-AAACAACUAUCCCAGC 5'

query=JS16_t1398639, target=FGSG_05996, score=4, range=6114-6131, strand=-1

target  5' UUCUGGUUGAUAGGGUUG 3'
```

```
query   3’ CUCUUACGUCGACUAGCU 5’

query=JS16_t2230573, target=FGSG_02768, score=4, range=289-306, strand=1

target  5’ CUGAAUGCAGCUGAUUGU 3’
             :::::::::::::.:
query   3’ CUCUUACGUCGACUAGCU 5’

query=JS16_t2230573, target=FGSG_04981, score=4, range=171-188, strand=1

target  5’ AGGCGUGCAGCUGAUUGA 3’
            .: .:::::::::.::
query   3’ CUCUUACGUCGACUAGCU 5’

query=JS16_t2230573, target=FGSG_10189, score=4, range=1615-1632, strand=1

target  5’ GAGAAUGCAGCGGAUGGA 3’
           ::::::::::: ::: ::
query   3’ CUCUUACGUCGACUAGCU 5’

query=JS16_t2230573, target=FGSG_11988, score=4, range=1551-1567, strand=1

target  5’ GGGAAUGCAGUUG-UCGG 3’
           :.::::::::.:: :::.
query   3’ CUCUUACGUCGACUAGCU 5’

query=JS16_t2230573, target=FGSG_05918, score=4, range=73-90, strand=1

target  5’ GAGAAUGCAGCUGCUGGA 3’
           ::::::::::::: : ::
query   3’ CUCUUACGUCGACUAGCU 5’

query=JS16_t2230573, target=FGSG_08407, score=4, range=1690-1708, strand=1

target  5’ GAGAAGGCAGCUGAUAUGA 3’
           ::::: ::::::::: .::
query   3’ CUCUUACGUCGACUA-GCU 5’

query=JS16_t2230573, target=FGSG_08816, score=4, range=991-1008, strand=1

target  5’ CUGAAUGCAGCUGAACGA 3’
             :::::::::::: :::
query   3’ CUCUUACGUCGACUAGCU 5’

query=JS16_t2230573, target=FGSG_13065, score=4, range=463-480, strand=1

target  5’ AAGAAUGCAGCGGAUCGC 3’
            :::::::::: :::::
query   3’ CUCUUACGUCGACUAGCU 5’

query=JS16_t2230573, target=FGSG_11328, score=4, range=549-566, strand=1

target  5’ GGGCGUGCAGCUGAUCCA 3’
           :.: .::::::::::: :
query   3’ CUCUUACGUCGACUAGCU 5’

query=JS16_t2230573, target=FGSG_08996, score=4, range=603-620, strand=1

target  5’ GACGAAGCAGCUGAUCGG 3’
           :: .: :::::::::::.
query   3’ CUCUUACGUCGACUAGCU 5’

query=JS16_t2230573, target=FGSG_13526, score=4, range=284-302, strand=1

target  5’ UCGACAUGCAGUUGAUCGA 3’
             :: ::::::.:::::::
query   3’ CUCU-UACGUCGACUAGCU 5’

query=JS16_t2230573, target=FGSG_13678, score=4, range=235-252, strand=1

target  5’ CUUAAUGCAGCUGAUCGC 3’
              ::::::::::::::
query   3’ CUCUUACGUCGACUAGCU 5’

query=JS16_t2230573, target=FGSG_10665, score=4, range=1123-1140, strand=1

target  5’ AAGUAUGCAGCUGAUAGA 3’
            :: ::::::::::: ::
query   3’ CUCUUACGUCGACUAGCU 5’

query=JS16_t2230573, target=FGSG_11434, score=4, range=936-952, strand=1

target  5’ GAGAAUGCAGCAG-UCGA 3’
           ::::::::::: : ::::
query   3’ CUCUUACGUCGACUAGCU 5’

query=JS16_t2230573, target=FGSG_02416, score=4, range=895-911, strand=1

target  5’ GAGAA-GGAGCUGAUCGA 3’
           ::::: : ::::::::::
query   3’ CUCUUACGUCGACUAGCU 5’

            : : :::::::::::.:
query   3’ UAAAACAACUAUCCCAGC 5’

query=JS16_t1398639, target=FGSG_04781, score=4, range=756-772, strand=-1

target  5’ AGUUUGUUGAU-GGGUUG 3’
           : ::::::::: ::::.:
query   3’ UAAAACAACUAUCCCAGC 5’

query=JS16_t1398639, target=FGSG_06417, score=4, range=4828-4845, strand=-1

target  5’ UGUUGGUUGAUAGGGUCU 3’
             :: :::::::::::
query   3’ UAAAACAACUAUCCCAGC 5’

query=JS16_t1398677, target=FGSG_11413, score=2, range=296-316, strand=1

target  5’ UCACU-GUCAACACAACACAAG 3’
           ::::: ::::::::::::::
query   3’ AGUGAGCAGUUGUGUUGUGUUA 5’

No results for JS16_t3191023
No results for JS16_t0307766
No results for JS16_t0475562
No results for JS16_t0475563
query=JS16_t0584474, target=FGSG_10514, score=4, range=27-47, strand=1

target  5’ GCUGGGCUCCGUAUUUGGCAC 3’
           ::: ..:::::::::.:::
query   3’ CGA-UUGAGGCAUAAAUCGUC 5’

query=JS16_t0584474, target=FGSG_11285, score=4, range=299-317, strand=1

target  5’ GC-AACGCCGUAUUUAGCGC 3’
           :: ::: ::::::::::.
query   3’ CGAUUGAGGCAUAAAUCGUC 5’

query=JS16_t1398825, target=FGSG_05671, score=4, range=235-257, strand=-1

target  5’ GGCAGCACAGUCGGAACAAGCAG 3’
           :.:: ::::.:::::::::: ::
query   3’ CUGUAGUGUUAGCCUUGUUC-UC 5’

query=JS16_t1398825, target=FGSG_07415, score=4, range=393-414, strand=-1

target  5’ GGUAUCAGAAUCGGAAGAAGAG 3’
           :..:::: :::::::: :::::
query   3’ CUGUAGUGUUAGCCUUGUUCUC 5’

query=JS16_t0475577, target=FGSG_03337, score=3, range=2433-2450, strand=1

target  5’ GCUAUCAAAACCGUGGUC 3’
           ::::::::::::::.:.
query   3’ CGAUAGUUUUGGCAUCGC 5’

query=JS16_t0475577, target=FGSG_08803, score=3, range=832-849, strand=-1

target  5’ GCGAUCAAGACUGUAGCG 3’
           :: :::::.::.::::::
query   3’ CGAUAGUUUUGGCAUCGC 5’

query=JS16_t0475577, target=FGSG_04433, score=3, range=310-327, strand=-1

target  5’ GGUAUCAAAACCGUAGAG 3’
           : ::::::::::::: :
query   3’ CGAUAGUUUUGGCAUCGC 5’

query=JS16_t0475577, target=FGSG_04378, score=4, range=695-712, strand=1

target  5’ GCGAACAAGACCGUGGCG 3’
           :: : :::.:::::.:::
query   3’ CGAUAGUUUUGGCAUCGC 5’

query=JS16_t0475577, target=FGSG_12231, score=4, range=1326-1342, strand=1

target  5’ UCUA-CAAAACCGUGGUG 3’
            ::: :::::::::.:.:
query   3’ CGAUAGUUUUGGCAUCGC 5’

query=JS16_t0475577, target=FGSG_04708, score=4, range=2416-2434, strand=1

target  5’ GCUGAUCAAUACCGUAGCC 3’
           ::: ::::: ::::::::
query   3’ CGA-UAGUUUUGGCAUCGC 5’

query=JS16_t0475577, target=FGSG_09527, score=4, range=1212-1228, strand=1

target  5’ GCGUUCAAAA-CGUAGCG 3’
           ::  :::::: :::::::
query   3’ CGAUAGUUUUGGCAUCGC 5’

query=JS16_t0475577, target=FGSG_09786, score=4, range=304-321, strand=-1
```

```
query=JS16_t2230573, target=FGSG_06144, score=4, range=194-212, strand=1

target  5’ AAGAACUGCAACUGAUCGA 3’
            :::: :::: ::::::::
query   3’ CUCUU-ACGUCGACUAGCU 5’

query=JS16_t2230573, target=FGSG_09939, score=4, range=1576-1592, strand=1

target  5’ GAG-AUGCAGCAGGUCGA 3’
           ::: ::::::: :.:::
query   3’ CUCUUACGUCGACUAGCU 5’

query=JS16_t2230573, target=FGSG_02043, score=4, range=1282-1298, strand=1

target  5’ GACAA-GCAGUUGAUCGA 3’
           :: :: ::::.::::::
query   3’ CUCUUACGUCGACUAGCU 5’

query=JS16_t2230573, target=FGSG_08415, score=4, range=1595-1611, strand=1

target  5’ GA-AAUGAAGCUGGUCGA 3’
           :: :::: ::::.::::
query   3’ CUCUUACGUCGACUAGCU 5’

query=JS16_t2230573, target=FGSG_06592, score=4, range=586-603, strand=1

target  5’ GCUAAUGCAGCUGAUCUA 3’
           :  ::::::::::::: :
query   3’ CUCUUACGUCGACUAGCU 5’

query=JS16_t2230573, target=FGSG_01385, score=4, range=4-20, strand=1

target  5’ GUUAAUG-AGCUGAUCGA 3’
           :  :::: :::::::::
query   3’ CUCUUACGUCGACUAGCU 5’

query=JS16_t2230573, target=FGSG_00236, score=4, range=388-404, strand=1

target  5’ GAGAAUG-GGCUGGUCGA 3’
           ::::::: .::::.:::
query   3’ CUCUUACGUCGACUAGCU 5’

query=JS16_t2230573, target=FGSG_04024, score=4, range=667-684, strand=-1

target  5’ GAGGGGGCAGUUGAUCGA 3’
           :::.. ::::.:::::::
query   3’ CUCUUACGUCGACUAGCU 5’

query=JS16_t2230573, target=FGSG_05709, score=4, range=553-570, strand=-1

target  5’ AAGAAUGCAGCCGGUCGA 3’
            :::::::::: :.::::
query   3’ CUCUUACGUCGACUAGCU 5’

query=JS16_t2230573, target=FGSG_08542, score=4, range=986-1003, strand=-1

target  5’ CGGAGUGGAGCUGAUCGA 3’
            .::.:: ::::::::::
query   3’ CUCUUACGUCGACUAGCU 5’

query=JS16_t2230573, target=FGSG_03869, score=4, range=1182-1200, strand=-1

target  5’ GACAAUGUAGCUGAUACGA 3’
           :: ::::.::::::: :::
query   3’ CUCUUACGUCGACUA-GCU 5’

query=JS16_t2230573, target=FGSG_00959, score=4, range=6-23, strand=-1

target  5’ GUGUAUGCGGCUGAUUGA 3’
           : : ::::.::::::.::
query   3’ CUCUUACGUCGACUAGCU 5’

query=JS16_t2230573, target=FGSG_08885, score=4, range=713-729, strand=-1

target  5’ AGGAAUG-AGCUGAUCGG 3’
            .::::: ::::::::.
query   3’ CUCUUACGUCGACUAGCU 5’

No results for JS16_t0335441
query=JS16_t0142825, target=FGSG_09353, score=4, range=289-308, strand=-1

target  5’ CCAGC-CUCAACCUGAGUGAC 3’
           : ::: ::::::::: ::::
query   3’ GCUCGCGAGUUGGACUGACUG 5’

No results for JS16_t2230629
No results for JS16_t4023517
query=JS16_t3127013, target=FGSG_05581, score=2, range=543-561, strand=-1

target  5’ AGUCG-GGUCUGGAUCCGAG 3’
           ::: : :::::::::::::
query   3’ UCACCGCCAGACCUAGGCUC 5’

target  5’ GCUAUCGAAAUCGUAGUC 3’
           ::::::.:::.:::::.
query   3’ CGAUAGUUUUGGCAUCGC 5’

query=JS16_t0475577, target=FGSG_02701, score=4, range=1707-1724, strand=-1

target  5’ GCCAUCAAAGCUGUAGUG 3’
           :: ::::::.:.::::.:
query   3’ CGAUAGUUUUGGCAUCGC 5’

query=JS16_t0475577, target=FGSG_01289, score=4, range=976-993, strand=-1

target  5’ AAUAUCAAAACCGUGGUG 3’
             ::::::::::::.:.:
query   3’ CGAUAGUUUUGGCAUCGC 5’

query=JS16_t0475577, target=FGSG_08623, score=4, range=1378-1395, strand=-1

target  5’ GUUGGCAAGACCGUAGUG 3’
           :.:. :::.:::::::.:
query   3’ CGAUAGUUUUGGCAUCGC 5’

query=JS16_t0475577, target=FGSG_08013, score=4, range=793-809, strand=-1

target  5’ GC-AUCAGAGCCGUGGCG 3’
           :: ::::.:.::::.:::
query   3’ CGAUAGUUUUGGCAUCGC 5’

query=JS16_t0475577, target=FGSG_04578, score=4, range=963-979, strand=-1

target  5’ GC-AUCGAAACCGUGGUG 3’
           :: :::.:::::::.:.:
query   3’ CGAUAGUUUUGGCAUCGC 5’

query=JS16_t0475577, target=FGSG_09118, score=4, range=2119-2135, strand=-1

target  5’ GCU-UCAAAGCCGUAGUU 3’
           ::: :::::.::::::.
query   3’ CGAUAGUUUUGGCAUCGC 5’

query=JS16_t0475577, target=FGSG_03277, score=4, range=970-986, strand=-1

target  5’ GCU-UCAAGAUCGUAGCC 3’
           ::: ::::.:.::::::
query   3’ CGAUAGUUUUGGCAUCGC 5’

query=JS16_t0475577, target=FGSG_10539, score=4, range=1505-1521, strand=-1

target  5’ UUUG-CAAAGCCGUAGCG 3’
            .:.  ::::.::::::::
query   3’ CGAUAGUUUUGGCAUCGC 5’

query=JS16_t0475577, target=FGSG_04473, score=4, range=922-939, strand=-1

target  5’ GGUAGCAAGACCGUAGCU 3’
           : :: :::.::::::::
query   3’ CGAUAGUUUUGGCAUCGC 5’

query=JS16_t0475577, target=FGSG_11597, score=4, range=795-811, strand=-1

target  5’ GCU-UCAAAAAUGUAGCG 3’
           ::: :::::: .::::::
query   3’ CGAUAGUUUUGGCAUCGC 5’

query=JS16_t0475577, target=FGSG_03726, score=4, range=358-374, strand=-1

target  5’ GCU-UCAAGACCGUAGAG 3’
           ::: ::::.::::::: :
query   3’ CGAUAGUUUUGGCAUCGC 5’

query=JS16_t0475577, target=FGSG_06493, score=4, range=1624-1640, strand=-1

target  5’ GC-ACCAAAGUCGUAGCG 3’
           :: : ::::..:::::::
query   3’ CGAUAGUUUUGGCAUCGC 5’

query=JS16_t0475577, target=FGSG_09776, score=4, range=301-317, strand=-1

target  5’ ACUA-CAAAAUCGUAGCC 3’
            ::: :::::.::::::
query   3’ CGAUAGUUUUGGCAUCGC 5’

query=JS16_t0475577, target=FGSG_00161, score=4, range=742-759, strand=-1

target  5’ GCCUUCAAAACCGUUGCG 3’
           ::  :::::::::: :::
query   3’ CGAUAGUUUUGGCAUCGC 5’

query=JS16_t0475577, target=FGSG_12118, score=4, range=6681-6698, strand=-1

target  5’ UCAACCAAAACCGUAGCU 3’
```

```
query=JS16_t3127013, target=FGSG_06555, score=2.5, range=472-491, strand=1

target  5’ AGUGGUGGUUUGGAUCCGAU 3’
           :::::.:::.:::::::::
query   3’ UCACCGCCAGACCUAGGCUC 5’

query=JS16_t3127013, target=FGSG_13648, score=3.5, range=290-309, strand=-1

target  5’ GGUGGCGGUCUGGUUCCGAA 3’
           .:::::::::::: :::::
query   3’ UCACCGCCAGACCUAGGCUC 5’

query=JS16_t3127013, target=FGSG_12029, score=4, range=3009-3028, strand=1

target  5’ UCUGGCGGUUUGGAUCCGAA 3’
             :::::::.:::::::::
query   3’ UCACCGCCAGACCUAGGCUC 5’

query=JS16_t3127013, target=FGSG_03229, score=4, range=250-270, strand=1

target  5’ AAUGAGCGGUCUGGAUUCGAU 3’
           : :: :::::::::::.:::
query   3’ UCAC-CGCCAGACCUAGGCUC 5’

query=JS16_t3127013, target=FGSG_08115, score=4, range=604-622, strand=1

target  5’ ACAGGC-GUCUGGAUCCGAC 3’
           :  ::: ::::::::::::
query   3’ UCACCGCCAGACCUAGGCUC 5’

query=JS16_t3127013, target=FGSG_03827, score=4, range=1466-1484, strand=-1

target  5’ GGUGCUGGUCUGGAUCC-AG 3’
           .::: .:::::::::: ::
query   3’ UCACCGCCAGACCUAGGCUC 5’

No results for JS16_t2230757
No results for JS16_t4023577
No results for JS16_t1334420
No results for JS16_t0210942
No results for JS16_t3127172
query=JS16_t0685471, target=FGSG_11549, score=3, range=306-323, strand=1

target  5’ UAAU-UCUGUUGAUGUUGU 3’
           .::: .::::::::::.::
query   3’ GUUAUGGACAACUACAGCA 5’

query=JS16_t0685471, target=FGSG_04899, score=3, range=9-26, strand=1

target  5’ CAA-ACCUGUUGAUGUCCU 3’
           ::: ::::::::::::: :
query   3’ GUUAUGGACAACUACAGCA 5’

query=JS16_t0685471, target=FGSG_13368, score=3, range=130-148, strand=-1

target  5’ UCGUACCUGUUGAUGUCGG 3’
           . .:::::::::::::::
query   3’ GUUAUGGACAACUACAGCA 5’

query=JS16_t0685471, target=FGSG_08462, score=3, range=137-154, strand=-1

target  5’ UGAU-CUUGUUGAUGUCGU 3’
           ..:: :.::::::::::::
query   3’ GUUAUGGACAACUACAGCA 5’

query=JS16_t0685471, target=FGSG_09761, score=3, range=268-285, strand=-1

target  5’ CAGU-UCUGUUGAUGUUGU 3’
           ::.: .::::::::::.::
query   3’ GUUAUGGACAACUACAGCA 5’

query=JS16_t0685471, target=FGSG_08784, score=3, range=455-472, strand=-1

target  5’ CGGGA-CUGUUGAUGUCGU 3’
           :.. : :::::::::::::
query   3’ GUUAUGGACAACUACAGCA 5’

query=JS16_t0685471, target=FGSG_05252, score=3.5, range=3232-3250, strand=1

target  5’ CAGGACCUGUUGAUGACGU 3’
           ::. ::::::::::: :::
query   3’ GUUAUGGACAACUACAGCA 5’

query=JS16_t0685471, target=FGSG_07577, score=3.5, range=693-710, strand=1

target  5’ CAA-GCCUGUUGGUGUCGA 3’
           ::: .:::::::.:::::
query   3’ GUUAUGGACAACUACAGCA 5’

query=JS16_t0685471, target=FGSG_05422, score=3.5, range=975-993, strand=-1
             : : ::::::::::::
query   3’ CGAUAGUUUUGGCAUCGC 5’

query=JS16_t0475577, target=FGSG_06515, score=4, range=280-296, strand=-1

target  5’ GCUA-CAAAACCGAAGCC 3’
           :::: :::::::: :::
query   3’ CGAUAGUUUUGGCAUCGC 5’

query=JS16_t0307773, target=FGSG_05945, score=2.5, range=1074-1093, strand=1

target  5’ CGCUGUUUUGAAGCUCCACU 3’
           :::::.::::::::.::::
query   3’ GCGACGAAACUUCGGGGUGC 5’

query=JS16_t0307773, target=FGSG_10617, score=3, range=404-422, strand=1

target  5’ CGCU-CUUUGAAGCCACACG 3’
           :::: :::::::::: ::::
query   3’ GCGACGAAACUUCGGGGUGC 5’

query=JS16_t0307773, target=FGSG_07480, score=3.5, range=520-538, strand=-1

target  5’ UGCAGC-UUGAAGCCUCACG 3’
           .:: :: ::::::::.::::
query   3’ GCGACGAAACUUCGGGGUGC 5’

query=JS16_t0307773, target=FGSG_09526, score=4, range=2996-3015, strand=1

target  5’ CGCUGCUUUGGCGCCCCACA 3’
           ::::::::::. :::::::
query   3’ GCGACGAAACUUCGGGGUGC 5’

query=JS16_t0307773, target=FGSG_02130, score=4, range=5243-5262, strand=-1

target  5’ UGUUGCUUUGAAGCCCGACU 3’
           .:.:::::::::::: ::
query   3’ GCGACGAAACUUCGGGGUGC 5’

query=JS16_t0307773, target=FGSG_06740, score=4, range=320-338, strand=-1

target  5’ CG-UGCUUUGAAGGCCCGCG 3’
           :: :::::::::: :::.::
query   3’ GCGACGAAACUUCGGGGUGC 5’

query=JS16_t2294892, target=FGSG_08547, score=3, range=250-267, strand=-1

target  5’ GUCAUAAGGGUCUUUAAG 3’
           .::: ::::.:::::::.
query   3’ UAGUUUUCCUAGAAAUUU 5’

query=JS16_t2294892, target=FGSG_11232, score=3, range=1285-1301, strand=-1

target  5’ UUCAAAA-GAUCUUUAAA 3’
            :::::: ::::::::::
query   3’ UAGUUUUCCUAGAAAUUU 5’

query=JS16_t2294892, target=FGSG_01266, score=3.5, range=1217-1234, strand=1

target  5’ AUCAAAAGGGUCUUGAAG 3’
           :::::::::.:::: ::.
query   3’ UAGUUUUCCUAGAAAUUU 5’

query=JS16_t2294892, target=FGSG_11743, score=3.5, range=1201-1218, strand=1

target  5’ GUCCGAAGGAUCUUUGAG 3’
           .:: .:::::::::.:.
query   3’ UAGUUUUCCUAGAAAUUU 5’

query=JS16_t2294892, target=FGSG_13993, score=3.5, range=571-587, strand=1

target  5’ AUU-AAAGGAUCUUUGGA 3’
           ::. :::::::::::..:
query   3’ UAGUUUUCCUAGAAAUUU 5’

query=JS16_t2294892, target=FGSG_01506, score=3.5, range=450-467, strand=1

target  5’ AUCCGACGGAUCUUUAAA 3’
           ::: .: :::::::::::
query   3’ UAGUUUUCCUAGAAAUUU 5’

query=JS16_t2294892, target=FGSG_08320, score=3.5, range=805-821, strand=1

target  5’ AUC-GAAGAAUCUUUAAA 3’
           ::: .::: :::::::::
query   3’ UAGUUUUCCUAGAAAUUU 5’

query=JS16_t2294892, target=FGSG_05297, score=3.5, range=1699-1716, strand=-1

target  5’ GUCAAAGGGAUCGUUAAA 3’
           .:::::.::::: :::::
query   3’ UAGUUUUCCUAGAAAUUU 5’
```

```
target   5’ CUGUACUUGUUGAUGUUGU 3’
            : .::.:::::::::.::
query    3’ GUUAUGGACAACUACAGCA 5’

query=JS16_t0685471, target=FGSG_02210, score=3.5, range=218-236, strand=-1

target   5’ AAUUGCUUGUUGAUGUCGU 3’
             : :.:.:::::::::::::
query    3’ GUUAUGGACAACUACAGCA 5’

query=JS16_t0685471, target=FGSG_11942, score=3.5, range=212-229, strand=-1

target   5’ CAAUG-CUGUUGAUGUUGC 3’
            ::::. ::::::::::.:
query    3’ GUUAUGGACAACUACAGCA 5’

query=JS16_t0685471, target=FGSG_02394, score=3.5, range=5948-5965, strand=-1

target   5’ UAAU-CCUGUUGAUGGCGU 3’
            .::: :::::::::: :::
query    3’ GUUAUGGACAACUACAGCA 5’

query=JS16_t0685471, target=FGSG_00982, score=3.5, range=217-234, strand=-1

target   5’ CAG-ACCUCUUGAUGUCGU 3’
            ::. :::: ::::::::::
query    3’ GUUAUGGACAACUACAGCA 5’

query=JS16_t0685471, target=FGSG_13539, score=4, range=197-214, strand=1

target   5’ CGGUACCUGUUGAU-UUGU 3’
            :..::::::::::: :.::
query    3’ GUUAUGGACAACUACAGCA 5’

query=JS16_t0685471, target=FGSG_08769, score=4, range=2213-2231, strand=1

target   5’ CAACACCUGUUGGUGUCAU 3’
            ::: ::::::::.:::: :
query    3’ GUUAUGGACAACUACAGCA 5’

query=JS16_t0685471, target=FGSG_03672, score=4, range=776-794, strand=1

target   5’ CCAUACCUGGUGAUGUCGA 3’
            : ::::::: ::::::::
query    3’ GUUAUGGACAACUACAGCA 5’

query=JS16_t0685471, target=FGSG_05398, score=4, range=405-422, strand=1

target   5’ UGGUGCCU-UUGAUGUCGU 3’
            ...:.::: ::::::::::
query    3’ GUUAUGGACAACUACAGCA 5’

query=JS16_t0685471, target=FGSG_13491, score=4, range=534-552, strand=-1

target   5’ UAAUGAUUGUUGAUGUCGA 3’
            .:::. .:::::::::::
query    3’ GUUAUGGACAACUACAGCA 5’

query=JS16_t0685471, target=FGSG_06965, score=4, range=1381-1399, strand=-1

target   5’ CACUGUCUGUUGGUGUCGG 3’
            :: :..::::::.:::::
query    3’ GUUAUGGACAACUACAGCA 5’

query=JS16_t0685471, target=FGSG_01743, score=4, range=1157-1175, strand=-1

target   5’ CGAAGGCUGUUGGUGUCGU 3’
            :.: .  ::::::.::::::
query    3’ GUUAUGGACAACUACAGCA 5’

query=JS16_t0685471, target=FGSG_04888, score=4, range=2558-2577, strand=-1

target   5’ CUGUAUAUUGUUGAUGUCGU 3’
            : .::. .::::::::::::
query    3’ GUUAUG-GACAACUACAGCA 5’

query=JS16_t0685471, target=FGSG_00826, score=4, range=428-445, strand=-1

target   5’ CAA-ACUUCUUGAUGUCGU 3’
            ::: ::.: ::::::::::
query    3’ GUUAUGGACAACUACAGCA 5’

query=JS16_t0685471, target=FGSG_06917, score=4, range=491-508, strand=-1

target   5’ CAA-ACUUGUUGAUGUCCU 3’
            ::: ::.:::::::::: :
query    3’ GUUAUGGACAACUACAGCA 5’

query=JS16_t0685471, target=FGSG_11742, score=4, range=2133-2152, strand=-1

target   5’ UAAAAUGCUGUUGAUGUCGC 3’
            .:: :.  ::::::::::::

query=JS16_t2294892, target=FGSG_13722, score=3.5, range=960-977, strand=-1

target   5’ GUCAAAAAGGUCUUUAAA 3’
            .:::::: :.::::::::
query    3’ UAGUUUUCCUAGAAAUUU 5’

query=JS16_t2294892, target=FGSG_07798, score=3.5, range=2506-2523, strand=-1

target   5’ AUUGAAAGGACCUUUAAG 3’
            ::..:::::: ::::::.
query    3’ UAGUUUUCCUAGAAAUUU 5’

query=JS16_t2294892, target=FGSG_11966, score=3.5, range=2947-2964, strand=-1

target   5’ AGUGAAAGGAUCUUUAGG 3’
            : ..::::::::::::..
query    3’ UAGUUUUCCUAGAAAUUU 5’

query=JS16_t2294892, target=FGSG_12375, score=4, range=709-726, strand=1

target   5’ AUCAAAAGGUUUUUUGAA 3’
            ::::::::: :.:::.::
query    3’ UAGUUUUCCUAGAAAUUU 5’

query=JS16_t2294892, target=FGSG_11669, score=4, range=930-948, strand=1

target   5’ AUCAAAGGGGAUUUUUAAA 3’
            :::::: .::::.::::::
query    3’ UAGUUU-UCCUAGAAAUUU 5’

query=JS16_t2294892, target=FGSG_09229, score=4, range=780-797, strand=1

target   5’ UUUAGAAGGAUCUGUAAA 3’
             :.:.:::::::: ::::
query    3’ UAGUUUUCCUAGAAAUUU 5’

query=JS16_t2294892, target=FGSG_01227, score=4, range=207-225, strand=1

target   5’ AUUAGCAAGGAUCUUUGAC 3’
            ::.:. ::::::::::.:
query    3’ UAGUU-UUCCUAGAAAUUU 5’

query=JS16_t2294892, target=FGSG_07044, score=4, range=1781-1798, strand=1

target   5’ ACCAAAAGGAUCUUGAGA 3’
            : ::::::::::: :.:
query    3’ UAGUUUUCCUAGAAAUUU 5’

query=JS16_t2294892, target=FGSG_00796, score=4, range=1564-1582, strand=1

target   5’ AUCAAAAAGGAUCUUGAGA 3’
            ::: ::::::::::: :.:
query    3’ UAG-UUUUCCUAGAAAUUU 5’

query=JS16_t2294892, target=FGSG_10117, score=4, range=106-124, strand=1

target   5’ GUGCAAAAGGAUCUUUCAG 3’
            .: ::::::::::::: :.
query    3’ UA-GUUUUCCUAGAAAUUU 5’

query=JS16_t2294892, target=FGSG_05468, score=4, range=1194-1210, strand=1

target   5’ AUC-AAAGGGUCUUCAAA 3’
            ::: :::::.:::: :::
query    3’ UAGUUUUCCUAGAAAUUU 5’

query=JS16_t2294892, target=FGSG_00528, score=4, range=263-279, strand=1

target   5’ AACAAGAGGA-CUUUAAA 3’
            : :::.:::: :::::::
query    3’ UAGUUUUCCUAGAAAUUU 5’

query=JS16_t2294892, target=FGSG_07653, score=4, range=268-285, strand=-1

target   5’ AUCAAAAGGGUCUUUGGC 3’
            :::::::::.:::::..
query    3’ UAGUUUUCCUAGAAAUUU 5’

query=JS16_t2294892, target=FGSG_01057, score=4, range=358-375, strand=-1

target   5’ AUUGAGAGGAUAUUUAAA 3’
            ::..:.:::::: ::::::
query    3’ UAGUUUUCCUAGAAAUUU 5’

query=JS16_t2294892, target=FGSG_04345, score=4, range=1216-1233, strand=-1

target   5’ AUUGGAUGGAUCUUUAAG 3’
            ::...: ::::::::::.
query    3’ UAGUUUUCCUAGAAAUUU 5’

query=JS16_t2294892, target=FGSG_04866, score=4, range=823-840, strand=-1
```

```
query   3’ GUUAUG-GACAACUACAGCA 5’

query=JS16_t0685471, target=FGSG_12210, score=4, range=548-567, strand=-1

target  5’ CGAUCUUCUGUUGAUGUCGC 3’
           :.::  .:::::::::::
query   3’ GUUA-UGGACAACUACAGCA 5’

query=JS16_t0685471, target=FGSG_12927, score=4, range=583-601, strand=-1

target  5’ CUUGAACUGUUGAUGUCGU 3’
           :   : :::::::::::::
query   3’ GUUAUGGACAACUACAGCA 5’

query=JS16_t0685471, target=FGSG_06057, score=4, range=1626-1643, strand=-1

target  5’ CAA-AACUGUUGAAGUCGU 3’
           ::: : ::::::: :::::
query   3’ GUUAUGGACAACUACAGCA 5’

query=JS16_t0685471, target=FGSG_09515, score=4, range=2873-2890, strand=-1

target  5’ CAAUA-CUGUUGAAGUUGU 3’
           ::::: ::::::: ::.::
query   3’ GUUAUGGACAACUACAGCA 5’

query=JS16_t0685471, target=FGSG_07508, score=4, range=1237-1254, strand=-1

target  5’ CCAUA-CUGUUGAUGGCGU 3’
           : ::: ::::::::: :::
query   3’ GUUAUGGACAACUACAGCA 5’

query=JS16_t0685471, target=FGSG_12694, score=4, range=980-997, strand=-1

target  5’ UAGUA-CUGUUGAUGUCCU 3’
           .:.:: ::::::::::: :
query   3’ GUUAUGGACAACUACAGCA 5’

No results for JS16_t1334508
query=JS16_t4023761, target=FGSG_01560, score=3.5, range=1243-1261, strand=1

target  5’ GAGCGACUAAGG-GGGCUGA 3’
           .:.:.:::::::: :::::::
query   3’ UUUGUUGAUUCCUCCCGACU 5’

query=JS16_t4023761, target=FGSG_06977, score=3.5, range=519-538, strand=-1

target  5’ AAACAGCGAAGGGGGGCUGA 3’
           :::::.: ::::.:::::::
query   3’ UUUGUUGAUUCCUCCCGACU 5’

query=JS16_t4023761, target=FGSG_10524, score=3.5, range=1529-1548, strand=-1

target  5’ AAGAGGCUGAGGAGGGCUGA 3’
           ::. ..::.:::::::::::
query   3’ UUUGUUGAUUCCUCCCGACU 5’

query=JS16_t4023761, target=FGSG_10694, score=4, range=158-178, strand=1

target  5’ ACACAACGAAGGAGGGACUGA 3’
           : ::::: :::::::: ::::
query   3’ UUUGUUGAUUCCUCCC-GACU 5’

query=JS16_t4023761, target=FGSG_12155, score=4, range=36-55, strand=-1

target  5’ AAGGGAAUAAGGAGGGCUGC 3’
           ::. .: ::::::::::::
query   3’ UUUGUUGAUUCCUCCCGACU 5’

No results for JS16_t2230958
No results for JS16_t1334555
No results for JS16_t0576828
query=JS16_t3127260, target=FGSG_02364, score=4, range=244-265, strand=1

target  5’ UCAAAUCCUGGAAC-GAUCAAGU 3’
           ::::::::. :::: :::::::.
query   3’ AGUUUAGGGACUUGACUAGUUCG 5’

query=JS16_t0102176, target=FGSG_09015, score=2.5, range=874-892, strand=1

target  5’ GUCAAUGCGUAUGGUAAAA 3’
           .  :::::::::.:::::::
query   3’ UUGUUACGCAUGCCAUUUU 5’

query=JS16_t0102176, target=FGSG_05968, score=4, range=821-838, strand=1

target  5’ GACGAUGUGU-CGGUAAAA 3’
           .::.:::.:: ::::::::
query   3’ UUGUUACGCAUGCCAUUUU 5’

query=JS16_t0102176, target=FGSG_04766, score=4, range=50-67, strand=1

target  5’ AACAAAGGGAUCUUUGAC 3’
           : ::::.:::::::::.:
query   3’ UAGUUUUCCUAGAAAUUU 5’

query=JS16_t2294892, target=FGSG_02401, score=4, range=744-761, strand=-1

target  5’ AUAGGAAGGAUUUUUAAU 3’
           :: ..::::::.:::::
query   3’ UAGUUUUCCUAGAAAUUU 5’

query=JS16_t2294892, target=FGSG_01815, score=4, range=9-26, strand=-1

target  5’ AGGAAGAGGAUCUUUGAA 3’
           :  ::.::::::::::.::
query   3’ UAGUUUUCCUAGAAAUUU 5’

query=JS16_t2294892, target=FGSG_11296, score=4, range=1051-1068, strand=-1

target  5’ AAGAGAAGGAUCUUUGAG 3’
           :  :.:::::::::::.:.
query   3’ UAGUUUUCCUAGAAAUUU 5’

query=JS16_t2294892, target=FGSG_09799, score=4, range=1305-1323, strand=-1

target  5’ AUUGACAAGGAUCUUGAAA 3’
           ::..: ::::::::: :::
query   3’ UAGUU-UUCCUAGAAAUUU 5’

No results for JS16_t4087929
query=JS16_t2295092, target=FGSG_01196, score=2, range=2907-2926, strand=1

target  5’ UGUGCUGAACUUUGGUGGUA 3’
           ::.:::.:::::.:::::::
query   3’ ACGCGAUUUGAAGCCACCAU 5’

query=JS16_t2295092, target=FGSG_05924, score=4, range=1797-1816, strand=1

target  5’ AGCGACCAACUUCGGUGGUA 3’
            :::   :::::::::::::
query   3’ ACGCGAUUUGAAGCCACCAU 5’

query=JS16_t2295092, target=FGSG_04680, score=4, range=1232-1251, strand=-1

target  5’ GGUGCUGAACUUUGGUGGUU 3’
            :.:::.:::::.::::::
query   3’ ACGCGAUUUGAAGCCACCAU 5’

query=JS16_t2295092, target=FGSG_00871, score=4, range=141-160, strand=-1

target  5’ AGUUCUCAACUUCGGUGGUG 3’
            :.  :: ::::::::::::.
query   3’ ACGCGAUUUGAAGCCACCAU 5’

No results for JS16_t1399108
query=JS16_t1399153, target=FGSG_08208, score=4, range=6844-6864, strand=-1

target  5’ CUUGGGUGCGGUGACCUUGCA 3’
           :::::: : :::::::.::::
query   3’ GAACCCUCUCCACUGGGACGU 5’

query=JS16_t2295226, target=FGSG_02529, score=4, range=862-881, strand=1

target  5’ GAUGGCGAGUACGCAGAUGA 3’
            :: .:.:::::::::::.:
query   3’ GUAGUGUUCAUGCGUCUAUU 5’

query=JS16_t2295226, target=FGSG_01659, score=4, range=811-830, strand=1

target  5’ CUUUAUCAGUACGCAGAUGA 3’
           : :.:.  ::::::::::.:
query   3’ GUAGUGUUCAUGCGUCUAUU 5’

query=JS16_t2295226, target=FGSG_06097, score=4, range=757-775, strand=1

target  5’ CG-CACAAGUACGCAGAUUG 3’
           :.  ::::::::::::::: .
query   3’ GUAGUGUUCAUGCGUCUAUU 5’

No results for JS16_t4088172
No results for JS16_t0367103
No results for JS16_t4088265
No results for JS16_t1399304
query=JS16_t2295353, target=FGSG_02672, score=3, range=1543-1563, strand=-1

target  5’ GAUCUCGGCUGCCCGCCGUUU 3’
           ::::::::::::: :::.:::
query   3’ CUAGAGCCGACGGUCGGUAAA 5’

query=JS16_t1399343, target=FGSG_08826, score=3.5, range=762-782, strand=1

target  5’ AGAGGAAGAUGUUGCAGUUGG 3’
```

```
target  5’ AACAA-GCCUACGGUAAAC 3’
           ::::: :: :::::::::
query   3’ UUGUUACGCAUGCCAUUUU 5’

No results for JS16_t1334586
No results for JS16_t1334609
query=JS16_t4023875, target=FGSG_04135, score=3.5, range=557-577, strand=1

target  5’ CUGAAGC-ACGCAUCCAAGCCA 3’
           ::.: :: ::.:::::::::::
query   3’ GAUUCCGUUGUGUAGGUUCGGU 5’

No results for JS16_t2231103
query=JS16_t0794805, target=FGSG_04512, score=4, range=1168-1187, strand=1

target  5’ CUUGUC-AACGUUAUGGCUUG 3’
             .:.: ::::::::::::::
query   3’ ACGCGGCUUGCAAUACCGAAC 5’

query=JS16_t0794805, target=FGSG_08740, score=4, range=569-589, strand=-1

target  5’ UGGGCAGAACGUGAUGGCUUG 3’
           :: :: :::::: ::::::::
query   3’ ACGCGGCUUGCAAUACCGAAC 5’

No results for JS16_t1334773
No results for JS16_t0111691
No results for JS16_t0202114
query=JS16_t0194465, target=FGSG_05066, score=4, range=1555-1575, strand=-1

target  5’ UCGCUCGAUGUAUGAGAUGUA 3’
           :::::  ::::::.:::::.:
query   3’ AGCGAUGUACAUAUUCUACGU 5’

No results for JS16_t2231326
query=JS16_t0364941, target=FGSG_01432, score=3.5, range=398-420, strand=-1

target  5’ CUUAA-UGAUCUUGAGGAAGCCAG 3’
           :..:: :::::.:::::::::::.
query   3’ GGGUUCACUAGGACUCCUUCGGUU 5’

query=JS16_t3127600, target=FGSG_09495, score=4, range=872-892, strand=1

target  5’ AGUUCCAGAAGCUUCGCCAGC 3’
           : ::::  :::::::::::.:
query   3’ UAAAGGCGUUCGAAGCGGUUG 5’

query=JS16_t3127600, target=FGSG_07174, score=4, range=503-522, strand=1

target  5’ CUUACCGCAAGCUUCG-CAAC 3’
            :: :::::::::::: ::::
query   3’ UAAAGGCGUUCGAAGCGGUUG 5’

query=JS16_t3127600, target=FGSG_10049, score=4, range=1045-1065, strand=-1

target  5’ GAUUCUACAAGCUUCGCCAAA 3’
           . :::. :::::::::::::
query   3’ UAAAGGCGUUCGAAGCGGUUG 5’

query=JS16_t3127600, target=FGSG_13552, score=4, range=261-281, strand=-1

target  5’ UUCUCGGCAAGCUUCGCCAAA 3’
            : :: ::::::::::::::
query   3’ UAAAGGCGUUCGAAGCGGUUG 5’

query=JS16_t0685518, target=FGSG_13878, score=4, range=23147-23167, strand=1

target  5’ ACGGAUCUGAGGAUUCGAAGG 3’
           : :: :::::::::::::: :
query   3’ UACCCAGACUCCUAAGCUUGC 5’

No results for JS16_t1334946
query=JS16_t0394275, target=FGSG_06845, score=3, range=1669-1690, strand=1

target  5’ AUCGAGCGACAGCUUGCUGCCG 3’
             : ::::::::::::::::::
query   3’ ACGGUCGCUGUCGAACGACGGC 5’

query=JS16_t0394275, target=FGSG_07364, score=4, range=2668-2688, strand=-1

target  5’ UUCCAUCGACAGCUUGCU-CCG 3’
           : ::: :::::::::::: :::
query   3’ ACGGUCGCUGUCGAACGACGGC 5’

query=JS16_t0467864, target=FGSG_01964, score=3.5, range=1806-1823, strand=1

target  5’ UAGU-GUCAUGUCACGAAA 3’
           .::: ::::::::::: :
query   3’ GUCACCAGUACAGUGCUGU 5’

query=JS16_t0467864, target=FGSG_01049, score=4, range=5750-5768, strand=1

            : ::::.:::::::::::
query   3’ GCCCCUUUUACAACGUCAACG 5’

query=JS16_t1399343, target=FGSG_06276, score=4, range=195-215, strand=-1

target  5’ CGGAGGAGAUGGUGCAGUUGC 3’
           ::: :.:.::: :::::::::
query   3’ GCCCCUUUUACAACGUCAACG 5’

query=JS16_t1399343, target=FGSG_07430, score=4, range=12-33, strand=-1

target  5’ CGGCGAGCGAUGUUGUAGUUGC 3’
           ::: ::. .::::::.::::::
query   3’ GCCCCUU-UUACAACGUCAACG 5’

query=JS16_t1399353, target=FGSG_05896, score=4, range=1584-1608, strand=1

target  5’ UCCGACCAGAACGCCCAGCAAUC-UC 3’
           ::::: :::: :::::::::::: ::
query   3’ AGGCUUGUCUAGCGGGUCGUUAGUAG 5’

query=JS16_t4088408, target=FGSG_04113, score=3, range=1021-1043, strand=1

target  5’ AAGGAACUGGAAAAUAUCGUCAU 3’
           : :::: :::::::::::.::::
query   3’ UGCCUU-ACCUUUUAUAGUAGUA 5’

query=JS16_t4088408, target=FGSG_07035, score=4, range=839-859, strand=1

target  5’ ACGGAA-GCGAAAUAUCGUCAU 3’
           :::::: : .:::::::.::::
query   3’ UGCCUUACCUUUUAUAGUAGUA 5’

query=JS16_t2295524, target=FGSG_12950, score=2, range=197-216, strand=-1

target  5’ UUGGUCG-ACUGCAAGAUCUU 3’
           .:::::: ::::::::::::.
query   3’ GACCAGCAUGACGUUCUAGAG 5’

query=JS16_t2295524, target=FGSG_09329, score=4, range=4245-4265, strand=1

target  5’ GUGCCAGUACUGCAAGAUCUC 3’
            ::   :::::::::::::::
query   3’ GACCAGCAUGACGUUCUAGAG 5’

No results for JS16_t2295548
No results for JS16_t4088552
query=JS16_t1399571, target=FGSG_06212, score=3, range=1602-1622, strand=1

target  5’ CCAACAUACAUUACGUGUCAA 3’
           :::::  :::::::::::::
query   3’ GGUUGCUUGUAAUGCACAGUA 5’

query=JS16_t1399571, target=FGSG_02556, score=4, range=349-370, strand=-1

target  5’ CCACACCAACAUUACCUGUCAU 3’
           ::: :: :::::::: ::::::
query   3’ GGU-UGCUUGUAAUGCACAGUA 5’

No results for JS16_t0211567
query=JS16_t3191860, target=FGSG_04291, score=2, range=5035-5052, strand=1

target  5’ UUCGAUGUUUUGGCGCAG 3’
           ..:: :::::::::::::
query   3’ GGGCAACAAAACCGCGUC 5’

query=JS16_t3191860, target=FGSG_05432, score=2, range=1633-1650, strand=-1

target  5’ AUUGUUGUUUUGGCGCAG 3’
            ..::::::::::::::::
query   3’ GGGCAACAAAACCGCGUC 5’

query=JS16_t3191860, target=FGSG_06740, score=2.5, range=116-133, strand=-1

target  5’ CUGGUUGUUUUGGCGCGG 3’
           :.  ::::::::::::.:
query   3’ GGGCAACAAAACCGCGUC 5’

query=JS16_t3191860, target=FGSG_11547, score=3, range=217-234, strand=1

target  5’ GUUGUUGUUUUGGCGCAC 3’
            ..::::::::::::::
query   3’ GGGCAACAAAACCGCGUC 5’

query=JS16_t3191860, target=FGSG_04935, score=3, range=825-842, strand=-1

target  5’ CCCGUUGUUUUGUCGUAG 3’
           :::::::::::: ::.::
query   3’ GGGCAACAAAACCGCGUC 5’

query=JS16_t3191860, target=FGSG_08308, score=3.5, range=145-162, strand=1
```

```
target  5’ CGGUCGUCAUGUCAAGACG 3’
           :.:: ::::::::: :::.
query   3’ GUCACCAGUACAGUGCUGU 5’

query=JS16_t0467864, target=FGSG_12481, score=4, range=490-507, strand=-1

target  5’ CA-UGGUCAUCUCGCGACA 3’
           :: ::::::: ::.:::::
query   3’ GUCACCAGUACAGUGCUGU 5’

query=JS16_t0467864, target=FGSG_06605, score=4, range=922-939, strand=-1

target  5’ CA-UGGUCAUGUCAAGACC 3’
           :: ::::::::::: :::
query   3’ GUCACCAGUACAGUGCUGU 5’

query=JS16_t0467864, target=FGSG_09365, score=4, range=475-493, strand=-1

target  5’ CAAAGAUCAUGUCACGACC 3’
           ::  : ::::::::::::
query   3’ GUCACCAGUACAGUGCUGU 5’

No results for JS16_t4024285
query=JS16_t3127736, target=FGSG_03511, score=3.5, range=272-289, strand=1

target  5’ GU-GAUGUCUUCCUAGGCA 3’
           .: ::::.:::::.:::::
query   3’ UACCUACGGAAGGGUCCGU 5’

query=JS16_t3127736, target=FGSG_06202, score=3.5, range=505-523, strand=1

target  5’ AACGCUGCCUUCCCAGGCG 3’
           :  : :::::::::::::.
query   3’ UACCUACGGAAGGGUCCGU 5’

query=JS16_t3127736, target=FGSG_06291, score=4, range=2404-2422, strand=1

target  5’ AUGGAUGCCUUCACGGGUA 3’
           :::::::::::: :.::.:
query   3’ UACCUACGGAAGGGUCCGU 5’

query=JS16_t3127736, target=FGSG_09877, score=4, range=259-277, strand=1

target  5’ GUGCCUGCUUUCCCAGGCG 3’
           .::  :::.:::::::::.
query   3’ UACCUACGGAAGGGUCCGU 5’

query=JS16_t3127736, target=FGSG_01503, score=4, range=694-712, strand=1

target  5’ CGAGAUGCCUUCCCAGGCU 3’
              :::::::::::::::
query   3’ UACCUACGGAAGGGUCCGU 5’

query=JS16_t3127736, target=FGSG_11185, score=4, range=1154-1172, strand=-1

target  5’ AUGGAUGCCUUGCCGGGCU 3’
           ::::::::::: ::.:::
query   3’ UACCUACGGAAGGGUCCGU 5’

query=JS16_t3127736, target=FGSG_02561, score=4, range=140-158, strand=-1

target  5’ ACGGGAGCCUUCUCAGGCG 3’
           : ::. ::::::.:::::.
query   3’ UACCUACGGAAGGGUCCGU 5’

query=JS16_t3127736, target=FGSG_03765, score=4, range=1257-1275, strand=-1

target  5’ AUUGACGUCUUCCCAGGCC 3’
           :: :: :.::::::::::
query   3’ UACCUACGGAAGGGUCCGU 5’

query=JS16_t3127736, target=FGSG_07602, score=4, range=274-292, strand=-1

target  5’ GUCUGUGCCUUCCCAGGCU 3’
           .:  .:::::::::::::
query   3’ UACCUACGGAAGGGUCCGU 5’

query=JS16_t3127736, target=FGSG_10148, score=4, range=769-786, strand=-1

target  5’ AU-GAAGCCUUUCCAGGCU 3’
           :: :: :::::.::::::
query   3’ UACCUACGGAAGGGUCCGU 5’

query=JS16_t3127736, target=FGSG_11886, score=4, range=366-383, strand=-1

target  5’ ACGGA-GCCUUCCCUGGCA 3’
           : ::: :::::::: ::::
query   3’ UACCUACGGAAGGGUCCGU 5’

No results for JS16_t3127747
No results for JS16_t4024308
No results for JS16_t4024330

target  5’ GUAGCUGUUUUGGCGCAG 3’
            . : :::::::::::::
query   3’ GGGCAACAAAACCGCGUC 5’

query=JS16_t3191860, target=FGSG_05263, score=3.5, range=1126-1143, strand=-1

target  5’ CUCGUUGUUUUUGCGCAU 3’
           :.::::::::: :::::
query   3’ GGGCAACAAAACCGCGUC 5’

query=JS16_t3191860, target=FGSG_00516, score=4, range=401-418, strand=1

target  5’ GCCGUUGUUUCGGUGCAG 3’
            ::::::::: ::.::::
query   3’ GGGCAACAAAACCGCGUC 5’

query=JS16_t3191860, target=FGSG_11396, score=4, range=1963-1980, strand=1

target  5’ UUCGUUGUUUUGGGGCAA 3’
           ..::::::::::: :::
query   3’ GGGCAACAAAACCGCGUC 5’

query=JS16_t3191860, target=FGSG_04208, score=4, range=85-103, strand=1

target  5’ GCAGUUGUUAUUGGCGCAG 3’
            : :::::: :::::::::
query   3’ GGGCAACAA-AACCGCGUC 5’

query=JS16_t3191860, target=FGSG_00036, score=4, range=1434-1451, strand=-1

target  5’ CUUGAUGUUUGGGCGCAG 3’
           :..: ::::: :::::::
query   3’ GGGCAACAAAACCGCGUC 5’

query=JS16_t3191860, target=FGSG_02752, score=4, range=95-112, strand=-1

target  5’ CUUCUUGUUUUUGCGCAG 3’
           :.. ::::::: ::::::
query   3’ GGGCAACAAAACCGCGUC 5’

query=JS16_t3191860, target=FGSG_00799, score=4, range=401-419, strand=-1

target  5’ UUAGUUCGUUUUGGCGCAG 3’
           .. ::: ::::::::::::
query   3’ GGGCAA-CAAAACCGCGUC 5’

query=JS16_t3191860, target=FGSG_07515, score=4, range=59-75, strand=-1

target  5’ UUCGUUGUUUUGG-GCAU 3’
           ..::::::::::: :::
query   3’ GGGCAACAAAACCGCGUC 5’

query=JS16_t3191860, target=FGSG_07382, score=4, range=1633-1650, strand=-1

target  5’ GGCGCUGUUUUGGCGCAU 3’
             :: ::::::::::::
query   3’ GGGCAACAAAACCGCGUC 5’

query=JS16_t3191860, target=FGSG_07559, score=4, range=1188-1205, strand=-1

target  5’ CAAGUGGUUUUGGCGCAG 3’
           :  :: ::::::::::::
query   3’ GGGCAACAAAACCGCGUC 5’

No results for JS16_t1399609
No results for JS16_t0396428
query=JS16_t3191897, target=FGSG_05370, score=2, range=589-605, strand=-1

target  5’ AACU-GUCGAUGAACGCU 3’
            ::: :::::::::::::
query   3’ GUGACCAGCUACUUGCGA 5’

query=JS16_t3191897, target=FGSG_10617, score=3, range=1497-1514, strand=-1

target  5’ ACCUGGUCGAUGAAUGCU 3’
             ::::::::::::.:::
query   3’ GUGACCAGCUACUUGCGA 5’

query=JS16_t3191897, target=FGSG_03137, score=3.5, range=1288-1305, strand=1

target  5’ CAUUGGUUGAUGAACCCU 3’
           ::.::::.::::::: ::
query   3’ GUGACCAGCUACUUGCGA 5’

query=JS16_t3191897, target=FGSG_06196, score=3.5, range=1263-1279, strand=1

target  5’ CGCU-GUUGGUGAACGCU 3’
           :.:: ::.:.::::::::
query   3’ GUGACCAGCUACUUGCGA 5’

query=JS16_t3191897, target=FGSG_11012, score=3.5, range=147-163, strand=1
```

```
query=JS16_t0219713, target=FGSG_00024, score=4, range=315-335, strand=1

target  5’ GGAAGAUGUUCUUGUACGACA 3’
           :::::::: ::::::::: ::
query   3’ CCUUCUACCAGAACAUGCCGU 5’

query=JS16_t0219713, target=FGSG_06676, score=4, range=1152-1171, strand=-1

target  5’ UGACGAUGGUCUUGUA-GGCA 3’
            :: ::::::::::::: ::::
query   3’ CCUUCUACCAGAACAUGCCGU 5’

query=JS16_t0219713, target=FGSG_06717, score=4, range=992-1011, strand=-1

target  5’ UGGAGGU-GUCUUGUACGGCU 3’
            :.::.: ::::::::::::
query   3’ CCUUCUACCAGAACAUGCCGU 5’

query=JS16_t0219713, target=FGSG_04450, score=4, range=486-505, strand=-1

target  5’ UGACGAUGGUCUUG-ACGGCA 3’
            :: :::::::::: ::::::
query   3’ CCUUCUACCAGAACAUGCCGU 5’

No results for JS16_t4024423
No results for JS16_t1335175
query=JS16_t2231664, target=FGSG_11208, score=4, range=94-113, strand=1

target  5’ GCGCCUGGCAUUGAAUUCCA 3’
           :: ::::::::::::.: ::
query   3’ CGAGGACCGUAACUUGAAGU 5’

query=JS16_t2231664, target=FGSG_03673, score=4, range=1144-1163, strand=1

target  5’ GCUUGGGGCAUUGAGCUUCG 3’
           :::.  ::::::::.::::.
query   3’ CGAGGACCGUAACUUGAAGU 5’

query=JS16_t2231664, target=FGSG_06078, score=4, range=93-111, strand=1

target  5’ GCACCUUGCAUUG-ACUUCA 3’
           :: ::: :::::: ::::::
query   3’ CGAGGACCGUAACUUGAAGU 5’

query=JS16_t2231664, target=FGSG_12056, score=4, range=43-63, strand=1

target  5’ GAUCCUGACAUUGACACUUCA 3’
           : ::::: :::::: ::::::
query   3’ CGAGGACCGUAACU-UGAAGU 5’

query=JS16_t2231664, target=FGSG_00561, score=4, range=655-673, strand=1

target  5’ GCCCCCGGCA-UGAACUUCA 3’
           :: :: :::: :::::::::
query   3’ CGAGGACCGUAACUUGAAGU 5’

query=JS16_t2231664, target=FGSG_03294, score=4, range=1768-1787, strand=-1

target  5’ GCUGCUGGCGUUGGACUUCC 3’
           ::: :::::.:::.:::::
query   3’ CGAGGACCGUAACUUGAAGU 5’

query=JS16_t2231664, target=FGSG_03431, score=4, range=925-945, strand=-1

target  5’ CUUCUUGGCAUUGAACUUGCA 3’
            .::.::::::::::::: ::
query   3’ CGAGGACCGUAACUUGAA-GU 5’

query=JS16_t2231666, target=FGSG_13723, score=3, range=746-763, strand=1

target  5’ CAUU-CGUUAGGAGGCGUG 3’
           :::: ::: ::::::::::
query   3’ GUAAUGCACUCCUCCGCAC 5’

query=JS16_t2231666, target=FGSG_08072, score=4, range=310-329, strand=-1

target  5’ UAUUGUCGUGAGGAGUCGUG 3’
           .:::. ::::::::: ::::
query   3’ GUAAU-GCACUCCUCCGCAC 5’

query=JS16_t2231666, target=FGSG_00140, score=4, range=252-270, strand=-1

target  5’ CUUGGUGUGAGGAGGUGUG 3’
           : : ..:::::::::.:::
query   3’ GUAAUGCACUCCUCCGCAC 5’

query=JS16_t2231666, target=FGSG_10402, score=4, range=865-882, strand=-1

target  5’ CAUU-CGUGGUGAGGCGUG 3’
           :::: ::::. ::::::::
query   3’ GUAAUGCACUCCUCCGCAC 5’

target  5’ UA-UGGUCGAUGAAUGCC 3’
           .: ::::::::::.::
query   3’ GUGACCAGCUACUUGCGA 5’

query=JS16_t3191897, target=FGSG_05073, score=3.5, range=693-709, strand=1

target  5’ UACU-GUCGAUGAACCCU 3’
           .::: :::::::::: ::
query   3’ GUGACCAGCUACUUGCGA 5’

query=JS16_t3191897, target=FGSG_12927, score=3.5, range=627-643, strand=-1

target  5’ CAUU-GUCGAUGGACGCC 3’
           ::.: :::::::.::::
query   3’ GUGACCAGCUACUUGCGA 5’

query=JS16_t3191897, target=FGSG_02315, score=4, range=16235-16252, strand=1

target  5’ CACUGGUUGUUGAAUGCU 3’
           ::::::.: ::::.:::
query   3’ GUGACCAGCUACUUGCGA 5’

query=JS16_t3191897, target=FGSG_02576, score=4, range=566-583, strand=1

target  5’ CACUGAUCGAUGAACGUA 3’
           ::::: ::::::::::.
query   3’ GUGACCAGCUACUUGCGA 5’

query=JS16_t3191897, target=FGSG_04131, score=4, range=1250-1267, strand=1

target  5’ UCUUGGUUGAUGAACGCA 3’
           . .::::.:::::::::
query   3’ GUGACCAGCUACUUGCGA 5’

query=JS16_t3191897, target=FGSG_11659, score=4, range=9461-9478, strand=1

target  5’ CCCUGGUCGUUGAAUGCU 3’
           : ::::::: ::::.:::
query   3’ GUGACCAGCUACUUGCGA 5’

query=JS16_t3191897, target=FGSG_04635, score=4, range=1129-1146, strand=1

target  5’ CUCUCGUUGAUGAAUGCU 3’
           : :: ::.::::::.:::
query   3’ GUGACCAGCUACUUGCGA 5’

query=JS16_t3191897, target=FGSG_00467, score=4, range=299-316, strand=1

target  5’ CACCGGCCGAUGAACGCA 3’
           ::: :: ::::::::::
query   3’ GUGACCAGCUACUUGCGA 5’

query=JS16_t3191897, target=FGSG_00913, score=4, range=157-174, strand=1

target  5’ CAAUGGUGGAUGAACGCC 3’
           :: :::: :::::::::
query   3’ GUGACCAGCUACUUGCGA 5’

query=JS16_t3191897, target=FGSG_04135, score=4, range=959-975, strand=1

target  5’ CACUGGUCGAUGCAC-CU 3’
           :::::::::::: :: ::
query   3’ GUGACCAGCUACUUGCGA 5’

query=JS16_t3191897, target=FGSG_11854, score=4, range=461-479, strand=1

target  5’ GACUGUUCGAUGAGACGCU 3’
            :::: ::::::: :::::
query   3’ GUGACCAGCUACU-UGCGA 5’

query=JS16_t3191897, target=FGSG_06075, score=4, range=2960-2976, strand=1

target  5’ UGCUCGUCGAUGAA-GCU 3’
           ..:: ::::::::: :::
query   3’ GUGACCAGCUACUUGCGA 5’

query=JS16_t3191897, target=FGSG_01443, score=4, range=182-199, strand=-1

target  5’ CACUGGUUGAUGGAUGCA 3’
           :::::::.::::.:.::
query   3’ GUGACCAGCUACUUGCGA 5’

query=JS16_t3191897, target=FGSG_02034, score=4, range=31-48, strand=-1

target  5’ UGCUGGUCGAAGAAUGCU 3’
           ..:::::::: :::.:::
query   3’ GUGACCAGCUACUUGCGA 5’

query=JS16_t3191897, target=FGSG_04477, score=4, range=894-911, strand=-1

target  5’ CACUGGUCGAGGAAUGCC 3’
           :::::::::: :::.::
```

```
query=JS16_t2231666, target=FGSG_05853, score=4, range=105-122, strand=-1

target  5’ CGUU-UGUGAGGAGGCGCG 3’
           :.:: .:::::::::: :
query   3’ GUAAUGCACUCCUCCGCAC 5’

query=JS16_t4024545, target=FGSG_01484, score=1.5, range=504-521, strand=-1

target  5’ UAUGAAAUAGUAAUGCCA 3’
           :::.::::::.:::::::
query   3’ AUAUUUUAUCGUUACGGU 5’

query=JS16_t4024545, target=FGSG_02394, score=3, range=4688-4705, strand=1

target  5’ GAUGGAAUGGCAAUGCCA 3’
            ::..:::.:::::::::
query   3’ AUAUUUUAUCGUUACGGU 5’

query=JS16_t4024545, target=FGSG_12083, score=3, range=376-393, strand=-1

target  5’ UGUAGAAUAGCAAUGUCU 3’
           :.::.::::::::::.:
query   3’ AUAUUUUAUCGUUACGGU 5’

query=JS16_t4024545, target=FGSG_11021, score=3, range=1410-1427, strand=-1

target  5’ UAUGCAGUAGCAAUGCCG 3’
           :::. :.:::::::::.
query   3’ AUAUUUUAUCGUUACGGU 5’

query=JS16_t4024545, target=FGSG_00620, score=3, range=74-92, strand=-1

target  5’ UAUAGAAAUAGAAAUGCCA 3’
           :::: :::::: :::::::
query   3’ AUAU-UUUAUCGUUACGGU 5’

query=JS16_t4024545, target=FGSG_11557, score=3, range=48-64, strand=-1

target  5’ UACAAAAUAG-AAUGCCA 3’
           :: ::::::: :::::::
query   3’ AUAUUUUAUCGUUACGGU 5’

query=JS16_t4024545, target=FGSG_13137, score=3.5, range=546-562, strand=1

target  5’ UGU-AGAUAGCAAUGCUA 3’
           :.: :.::::::::::.:
query   3’ AUAUUUUAUCGUUACGGU 5’

query=JS16_t4024545, target=FGSG_09490, score=3.5, range=330-347, strand=-1

target  5’ UGAAGAAUAGCAAUGCUG 3’
           :.  :.::::::::::..
query   3’ AUAUUUUAUCGUUACGGU 5’

query=JS16_t4024545, target=FGSG_06670, score=3.5, range=1881-1899, strand=-1

target  5’ UGUGAGAAUAGCAAUGUCG 3’
           :.: :.::::::::::.:.
query   3’ AUA-UUUUAUCGUUACGGU 5’

query=JS16_t4024545, target=FGSG_04281, score=3.5, range=327-344, strand=-1

target  5’ GAUGCAAUAGCGAUGCCA 3’
            ::. ::::::.::::::
query   3’ AUAUUUUAUCGUUACGGU 5’

query=JS16_t4024545, target=FGSG_03164, score=3.5, range=507-524, strand=-1

target  5’ CAGAGAAUAGCAAUGUCA 3’
            : :.::::::::::.::
query   3’ AUAUUUUAUCGUUACGGU 5’

query=JS16_t4024545, target=FGSG_03544, score=3.5, range=90-106, strand=-1

target  5’ UGU-AGAUGGCAAUGCCA 3’
           :.: :.::.:::::::::
query   3’ AUAUUUUAUCGUUACGGU 5’

query=JS16_t4024545, target=FGSG_13369, score=3.5, range=606-622, strand=-1

target  5’ UGUAAAAUA-CAAUGUCA 3’
           :.::::::: :::::.::
query   3’ AUAUUUUAUCGUUACGGU 5’

query=JS16_t4024545, target=FGSG_12018, score=4, range=1033-1050, strand=1

target  5’ AGUGAAAUAGCAACGCCA 3’
            .:.::::::::: ::::
query   3’ AUAUUUUAUCGUUACGGU 5’

query=JS16_t4024545, target=FGSG_04168, score=4, range=4651-4669, strand=1

query   3’ GUGACCAGCUACUUGCGA 5’

query=JS16_t3191897, target=FGSG_06278, score=4, range=280-296, strand=-1

target  5’ UAUUGGUUGAUGAAC-CU 3’
           .:.::::.::::::: ::
query   3’ GUGACCAGCUACUUGCGA 5’

query=JS16_t3191897, target=FGSG_05891, score=4, range=3361-3378, strand=-1

target  5’ CACGGGUCGGUGAUCGCU 3’
           ::: :::::.::: ::::
query   3’ GUGACCAGCUACUUGCGA 5’

query=JS16_t3191897, target=FGSG_00697, score=4, range=1596-1613, strand=-1

target  5’ UGCUCGUCGAGGAACGCU 3’
           ..:: ::::: :::::::
query   3’ GUGACCAGCUACUUGCGA 5’

query=JS16_t3191897, target=FGSG_12771, score=4, range=77-94, strand=-1

target  5’ AUCUCGUCGAUGAAUGCU 3’
             :: :::::::::.:::
query   3’ GUGACCAGCUACUUGCGA 5’

query=JS16_t3191897, target=FGSG_01954, score=4, range=1314-1330, strand=-1

target  5’ CGUU-GUCGAUGAGCGCG 3’
           :..: ::::::::.:::
query   3’ GUGACCAGCUACUUGCGA 5’

query=JS16_t3191897, target=FGSG_01884, score=4, range=429-445, strand=-1

target  5’ UGCU-CUCGAUGAACGCU 3’
           ..::  ::::::::::::
query   3’ GUGACCAGCUACUUGCGA 5’

No results for JS16_t2295698
No results for JS16_t1399652
No results for JS16_t4088629
No results for JS16_t3191943
No results for JS16_t0584565
query=JS16_t3191987, target=FGSG_05548, score=3.5, range=675-692, strand=1

target  5’ UCACUUAAUGCU-GAAAAG 3’
           ::: .:::::::: ::::::
query   3’ AGUAGAUUACGAGCUUUUC 5’

query=JS16_t3191987, target=FGSG_11594, score=4, range=2121-2139, strand=1

target  5’ UGAUGUAAUGCUUGAAAGG 3’
           : :: :::::::.::::.:
query   3’ AGUAGAUUACGAGCUUUUC 5’

query=JS16_t3191987, target=FGSG_07692, score=4, range=578-595, strand=1

target  5’ UCAU-GGAUGCUCGAGAAG 3’
           ::::  .:::::::::.:::
query   3’ AGUAGAUUACGAGCUUUUC 5’

query=JS16_t3191987, target=FGSG_11594, score=4, range=58-76, strand=1

target  5’ UUGGAUAAUGCUCGGAAAG 3’
           :..   :::::::::.::::
query   3’ AGUAGAUUACGAGCUUUUC 5’

query=JS16_t3191987, target=FGSG_00862, score=4, range=293-311, strand=-1

target  5’ UCAUCAAAUGCUUGUAAAG 3’
           ::::: ::::::.: ::::
query   3’ AGUAGAUUACGAGCUUUUC 5’

query=JS16_t3191987, target=FGSG_07693, score=4, range=540-558, strand=-1

target  5’ UCCUCUAGUGCUCGAACAG 3’
           :: ::::.:::::::: ::
query   3’ AGUAGAUUACGAGCUUUUC 5’

query=JS16_t3191987, target=FGSG_05794, score=4, range=7582-7600, strand=-1

target  5’ UCAUGGAGUGCUCGAAAAU 3’
           ::::  :.::::::::::
query   3’ AGUAGAUUACGAGCUUUUC 5’

query=JS16_t3191987, target=FGSG_01564, score=4, range=884-901, strand=-1

target  5’ UCA-CGGAUGCUCGAAAGG 3’
           ::: : .::::::::::.:
query   3’ AGUAGAUUACGAGCUUUUC 5’

No results for JS16_t3192028
```

```
target  5’ GAUGAAGAUAGCAGUGCCA 3’
            :: ::.::::::.:::::
query   3’ AUA-UUUUAUCGUUACGGU 5’

query=JS16_t4024545, target=FGSG_06373, score=4, range=2437-2455, strand=1

target  5’ CAUACCAAUGGCAAUGCCA 3’
            :::  :::.:::::::::
query   3’ AUAU-UUUAUCGUUACGGU 5’

query=JS16_t4024545, target=FGSG_11417, score=4, range=651-667, strand=1

target  5’ UG-AGAAUACCAAUGCCA 3’
           :. :.:::: ::::::::
query   3’ AUAUUUUAUCGUUACGGU 5’

query=JS16_t4024545, target=FGSG_05213, score=4, range=5677-5695, strand=1

target  5’ UCUAACACUAGCAAUGCCA 3’
           : ::: : :::::::::::
query   3’ AUAUU-UUAUCGUUACGGU 5’

query=JS16_t4024545, target=FGSG_08487, score=4, range=480-497, strand=-1

target  5’ GGUGUAAUAGCAGUGCCA 3’
            .:. :::::::.:::::
query   3’ AUAUUUUAUCGUUACGGU 5’

query=JS16_t4024545, target=FGSG_02147, score=4, range=900-916, strand=-1

target  5’ UGUAGAG-AGCAAUGCCA 3’
           :.::.:. ::::::::::
query   3’ AUAUUUUAUCGUUACGGU 5’

query=JS16_t4024545, target=FGSG_02442, score=4, range=1467-1484, strand=-1

target  5’ ACGAGAAUAGCAAUGCCG 3’
              :.::::::::::::.
query   3’ AUAUUUUAUCGUUACGGU 5’

query=JS16_t4024545, target=FGSG_06840, score=4, range=1179-1196, strand=-1

target  5’ UGUGUAAGAGCAAUGCCA 3’
           :.:. :: ::::::::::
query   3’ AUAUUUUAUCGUUACGGU 5’

query=JS16_t4024545, target=FGSG_07954, score=4, range=567-584, strand=-1

target  5’ UAUCCAAUGGCGAUGCCA 3’
           :::  :::.::.::::::
query   3’ AUAUUUUAUCGUUACGGU 5’

query=JS16_t4024545, target=FGSG_10057, score=4, range=2493-2509, strand=-1

target  5’ CAUAAGAUAGCAAU-CCA 3’
            ::::.:::::::: :::
query   3’ AUAUUUUAUCGUUACGGU 5’

query=JS16_t4024545, target=FGSG_06375, score=4, range=1338-1356, strand=-1

target  5’ UAUGGAAUGAGCAAUGCCC 3’
           :::..::: :::::::::
query   3’ AUAUUUUA-UCGUUACGGU 5’

query=JS16_t2231743, target=FGSG_09870, score=2.5, range=197-217, strand=-1

target  5’ AAGAGAUAACACAGUCGGAGA 3’
           ::. :: ::::::::::::::
query   3’ UUUGCU-UUGUGUCAGCCUCU 5’

query=JS16_t2231743, target=FGSG_04368, score=3.5, range=268-287, strand=1

target  5’ GAACGAAACGCAGUCGGAAA 3’
           .::::::::.:::::::: :
query   3’ UUUGCUUUGUGUCAGCCUCU 5’

query=JS16_t2231743, target=FGSG_03311, score=3.5, range=1239-1258, strand=-1

target  5’ CGACGAAACACAGUCGGCGA 3’
            .::::::::::::::: ::
query   3’ UUUGCUUUGUGUCAGCCUCU 5’

query=JS16_t2231743, target=FGSG_05335, score=3.5, range=548-566, strand=-1

target  5’ GAACG-AACAUAGUUGGAGA 3’
           .:::: ::::.:::.:::::
query   3’ UUUGCUUUGUGUCAGCCUCU 5’

query=JS16_t0364956, target=FGSG_01451, score=3, range=1072-1089, strand=1

target  5’ UUUUUGUUGAGUUACAGU 3’
           ::: ::::::..::::::

No results for JS16_t3192054
query=JS16_t1399777, target=FGSG_09065, score=4, range=1117-1137, strand=-1

target  5’ GAGAAUCAAAGGAGAGGGAU 3’
           :  : : :::::::::::::
query   3’ CCAUCACUUUCCCUCUCCCUA 5’

No results for JS16_t4088747
query=JS16_t0188912, target=FGSG_01924, score=2, range=602-619, strand=1

target  5’ UUCGCAAGAACCAGUUCU 3’
             ::::::::::::::::
query   3’ CCGCGUUCUUGGUCAAGA 5’

query=JS16_t0188912, target=FGSG_10136, score=3, range=1612-1629, strand=1

target  5’ GGCUCAAGAGCUAGUUCU 3’
           ::: :::::.:.::::::
query   3’ CCGCGUUCUUGGUCAAGA 5’

query=JS16_t0188912, target=FGSG_10087, score=3, range=444-461, strand=-1

target  5’ GGCGCAAGAGCCAGUGCU 3’
           :::::::::.::::: ::
query   3’ CCGCGUUCUUGGUCAAGA 5’

query=JS16_t0188912, target=FGSG_02523, score=3, range=299-315, strand=-1

target  5’ GGCGGAAGAACC-GUUCU 3’
           :::: ::::::: :::::
query   3’ CCGCGUUCUUGGUCAAGA 5’

query=JS16_t0188912, target=FGSG_08806, score=3.5, range=341-358, strand=1

target  5’ UGUCCAAGAGCCAGUUCU 3’
            :. :::::.::::::::
query   3’ CCGCGUUCUUGGUCAAGA 5’

query=JS16_t0188912, target=FGSG_09581, score=3.5, range=1857-1873, strand=1

target  5’ GGC-UAAGAACCAGGUCU 3’
           ::: .::::::::: :::
query   3’ CCGCGUUCUUGGUCAAGA 5’

query=JS16_t0188912, target=FGSG_09830, score=3.5, range=344-361, strand=-1

target  5’ GGUGGAAGAACCAGAUCU 3’
           ::.: ::::::::: :::
query   3’ CCGCGUUCUUGGUCAAGA 5’

query=JS16_t0188912, target=FGSG_01280, score=3.5, range=837-854, strand=-1

target  5’ UGAGUAAGAACCAGUUCG 3’
            : :.::::::::::::
query   3’ CCGCGUUCUUGGUCAAGA 5’

query=JS16_t0188912, target=FGSG_13353, score=3.5, range=1549-1565, strand=-1

target  5’ GAUGCAAGAACCA-UUCU 3’
           : .:::::::::: ::::
query   3’ CCGCGUUCUUGGUCAAGA 5’

query=JS16_t0188912, target=FGSG_05640, score=4, range=236-253, strand=1

target  5’ AGCGCAAGGAUCAGUUCG 3’
            :::::::.:.::::::
query   3’ CCGCGUUCUUGGUCAAGA 5’

query=JS16_t0188912, target=FGSG_05276, score=4, range=482-499, strand=1

target  5’ UGAGCGAGGACCAGUUCU 3’
            : ::.::.:::::::::
query   3’ CCGCGUUCUUGGUCAAGA 5’

query=JS16_t0188912, target=FGSG_02786, score=4, range=3240-3257, strand=1

target  5’ UCAGCAAGAGCCAGUUCU 3’
              ::::::.::::::::
query   3’ CCGCGUUCUUGGUCAAGA 5’

query=JS16_t0188912, target=FGSG_06711, score=4, range=507-523, strand=1

target  5’ GGC-CAAGGGUCAGUUCU 3’
           ::: ::::...:::::::
query   3’ CCGCGUUCUUGGUCAAGA 5’

query=JS16_t0188912, target=FGSG_01299, score=4, range=276-293, strand=1

target  5’ GACGCUGGAACCAGUUCU 3’
           : ::: .:::::::::::
query   3’ CCGCGUUCUUGGUCAAGA 5’
```

```
query   3’ AAAUACAACUUGAUGUCA 5’

query=JS16_t0364956, target=FGSG_13055, score=3.5, range=533-550, strand=1

target  5’ AUUGGGUUGAACUACAGA 3’
            ::. ::::::::::::
query   3’ AAAUACAACUUGAUGUCA 5’

query=JS16_t0364956, target=FGSG_07684, score=4, range=574-592, strand=1

target  5’ UGUAUGUUGAACUCGCAGU 3’
           : ::::::::::: .::::
query   3’ AAAUACAACUUGA-UGUCA 5’

query=JS16_t0364956, target=FGSG_08221, score=4, range=1516-1533, strand=1

target  5’ GAUAUGAUGAACUACAGU 3’
             :::: :::::::::::
query   3’ AAAUACAACUUGAUGUCA 5’

query=JS16_t0364956, target=FGSG_04861, score=4, range=217-233, strand=1

target  5’ UUCACGUUGAAC-ACAGU 3’
           :: : ::::::: :::::
query   3’ AAAUACAACUUGAUGUCA 5’

query=JS16_t0364956, target=FGSG_12242, score=4, range=2218-2235, strand=-1

target  5’ UUUUUGUAGAACUGCAGU 3’
           ::: ::: :::::.::::
query   3’ AAAUACAACUUGAUGUCA 5’

query=JS16_t0364956, target=FGSG_00349, score=4, range=2353-2370, strand=-1

target  5’ UUGAUGUUGAACCACAGG 3’
           :: ::::::::: ::::
query   3’ AAAUACAACUUGAUGUCA 5’

query=JS16_t0364956, target=FGSG_12442, score=4, range=169-186, strand=-1

target  5’ UAGAAGUUGAACUGCAGU 3’
           :  : ::::::::.::::
query   3’ AAAUACAACUUGAUGUCA 5’

No results for JS16_t0576922
No results for JS16_t4024648
query=JS16_t0335482, target=FGSG_01495, score=4, range=480-502, strand=-1

target  5’ CAGUGCGCACGUUCUCUGUUCCG 3’
           ::::::: ::::::: ::::::
query   3’ GUCACGCUUGCAAGA-ACAAGGG 5’

No results for JS16_t1335422
query=JS16_t4024674, target=FGSG_02151, score=3.5, range=765-785, strand=-1

target  5’ AUGAGGAGGAAGAACUGCUUG 3’
           ::::.:: :::::::::::.
query   3’ UACUUCUGCUUCUUGACGAGA 5’

query=JS16_t4024674, target=FGSG_09171, score=4, range=293-313, strand=1

target  5’ CUGAAUACGAGGAGCUGCUCU 3’
            :::: ::::.::.:::::::
query   3’ UACUUCUGCUUCUUGACGAGA 5’

query=JS16_t4024674, target=FGSG_07940, score=4, range=3606-3625, strand=-1

target  5’ AUGGGGA-GAGGAACUGCUCG 3’
           :::..:: ::.:::::::::
query   3’ UACUUCUGCUUCUUGACGAGA 5’

No results for JS16_t2231885
No results for JS16_t4024742
No results for JS16_t1335488
query=JS16_t0128574, target=FGSG_06812, score=4, range=547-568, strand=1

target  5’ GCACAUCGCGAGCGAUUGUCGA 3’
           .::::::.:::.:::::: :::
query   3’ UGUGUAGUGCUUGCUAACCGCU 5’

query=JS16_t0128574, target=FGSG_10578, score=4, range=664-686, strand=-1

target  5’ AUAGCGUGACGAAUGAUUGGCGA 3’
           :.: :.: :::::.:::::::::
query   3’ UGU-GUAGUGCUUGCUAACCGCU 5’

No results for JS16_t2231974
No results for JS16_t0210956
No results for JS16_t3128301
No results for JS16_t1335611
No results for JS16_t4024934
query=JS16_t3128386, target=FGSG_11240, score=3, range=199-218, strand=1

query=JS16_t0188912, target=FGSG_10473, score=4, range=60-77, strand=1

target  5’ GGCCCGUGAACCAGUUCU 3’
           ::: :.  :::::::::::
query   3’ CCGCGUUCUUGGUCAAGA 5’

query=JS16_t0188912, target=FGSG_03139, score=4, range=1422-1439, strand=1

target  5’ UCCACAAGAGCCAGUUCU 3’
             : :::::.::::::::
query   3’ CCGCGUUCUUGGUCAAGA 5’

query=JS16_t0188912, target=FGSG_05330, score=4, range=713-730, strand=1

target  5’ AGCUCGAGAACCAGUUCC 3’
            :: :.::::::::::
query   3’ CCGCGUUCUUGGUCAAGA 5’

query=JS16_t0188912, target=FGSG_04972, score=4, range=125-142, strand=1

target  5’ AUCUCGAGAACCAGUUCU 3’
             : :.::::::::::::
query   3’ CCGCGUUCUUGGUCAAGA 5’

query=JS16_t0188912, target=FGSG_06486, score=4, range=1099-1115, strand=1

target  5’ AGC-CAAGAAUCGGUUCU 3’
            :: ::::::.:.:::::
query   3’ CCGCGUUCUUGGUCAAGA 5’

query=JS16_t0188912, target=FGSG_11755, score=4, range=1742-1759, strand=1

target  5’ CGACCAAGAGCCAGUUCU 3’
            :  :::::.::::::::
query   3’ CCGCGUUCUUGGUCAAGA 5’

query=JS16_t0188912, target=FGSG_06037, score=4, range=974-991, strand=1

target  5’ GUCUCAAGAACCAGUUUG 3’
           : : ::::::::::::.
query   3’ CCGCGUUCUUGGUCAAGA 5’

query=JS16_t0188912, target=FGSG_06326, score=4, range=1425-1441, strand=1

target  5’ GGCUC-AGAAUCAGUUCU 3’
           ::: : ::::.:::::::
query   3’ CCGCGUUCUUGGUCAAGA 5’

query=JS16_t0188912, target=FGSG_10524, score=4, range=255-271, strand=1

target  5’ GGC-CAAGAACCGCUUCU 3’
           ::: ::::::::. ::::
query   3’ CCGCGUUCUUGGUCAAGA 5’

query=JS16_t0188912, target=FGSG_06911, score=4, range=1562-1579, strand=1

target  5’ CCCACAAGAACCAGUUCA 3’
             : ::::::::::::
query   3’ CCGCGUUCUUGGUCAAGA 5’

query=JS16_t0188912, target=FGSG_02433, score=4, range=1464-1480, strand=1

target  5’ AGC-CGAGAACCAGUUCA 3’
            :: :.:::::::::::
query   3’ CCGCGUUCUUGGUCAAGA 5’

query=JS16_t0188912, target=FGSG_08638, score=4, range=1279-1296, strand=1

target  5’ GGAUCAAGAACCAGUACU 3’
           ::  ::::::::::: ::
query   3’ CCGCGUUCUUGGUCAAGA 5’

query=JS16_t0188912, target=FGSG_01004, score=4, range=1458-1475, strand=1

target  5’ GGAAGAAGAGCCAGUUCU 3’
           ::   ::::.::::::::
query   3’ CCGCGUUCUUGGUCAAGA 5’

query=JS16_t0188912, target=FGSG_05715, score=4, range=198-214, strand=1

target  5’ GACG-AAGAACCACUUCU 3’
           : :: :::::::: ::::
query   3’ CCGCGUUCUUGGUCAAGA 5’

query=JS16_t0188912, target=FGSG_03163, score=4, range=1583-1600, strand=-1

target  5’ GGCAGAGGAACCGGUUCU 3’
           :::  :.:::::.:::::
query   3’ CCGCGUUCUUGGUCAAGA 5’

query=JS16_t0188912, target=FGSG_07466, score=4, range=3746-3764, strand=-1
```

```
target  5’ UUCGACUUUCAUGAGUGGUC 3’
           :::: : ::::::::::::
query   3’ AAGCAG-AAGUACUCACCAC 5’

query=JS16_t3128386, target=FGSG_02010, score=4, range=302-320, strand=1

target  5’ GUCUUCUUCGUGAGUGGUU 3’
            :: :::::.::::::::
query   3’ AAGCAGAAGUACUCACCAC 5’

query=JS16_t3128386, target=FGSG_09160, score=4, range=1216-1234, strand=1

target  5’ CCUGCUUUCAUGAGUGGUG 3’
             .: .:::::::::::::
query   3’ AAGCAGAAGUACUCACCAC 5’

query=JS16_t3128386, target=FGSG_10483, score=4, range=355-373, strand=1

target  5’ GUCAUCUUCAUGAUUGGUG 3’
            :: ::::::::: :::::
query   3’ AAGCAGAAGUACUCACCAC 5’

query=JS16_t3128386, target=FGSG_04386, score=4, range=574-592, strand=1

target  5’ GUCGCCUUGAUGAGUGGUG 3’
            ::: ::: ::::::::::
query   3’ AAGCAGAAGUACUCACCAC 5’

query=JS16_t3128386, target=FGSG_10871, score=4, range=259-277, strand=1

target  5’ CUAGUGUUUAUGAGUGGUG 3’
            : :: ::.::::::::::
query   3’ AAGCAGAAGUACUCACCAC 5’

query=JS16_t3128386, target=FGSG_06039, score=4, range=1175-1192, strand=1

target  5’ UUC-UCUUCAUUGGUGGUG 3’
           ::: ::::::: .::::::
query   3’ AAGCAGAAGUACUCACCAC 5’

query=JS16_t3128386, target=FGSG_00017, score=4, range=1355-1372, strand=1

target  5’ UUCGU-UUCAUGGGUAGUG 3’
           ::::: ::::::.:: :::
query   3’ AAGCAGAAGUACUCACCAC 5’

query=JS16_t3128386, target=FGSG_12917, score=4, range=20-38, strand=-1

target  5’ UUCAUAUUCUUGAGUGGUG 3’
           ::: : ::: :::::::::
query   3’ AAGCAGAAGUACUCACCAC 5’

query=JS16_t3128386, target=FGSG_01386, score=4, range=1035-1052, strand=-1

target  5’ UUCUUCUUCAUGAG-GGUA 3’
           ::: :::::::::: :::
query   3’ AAGCAGAAGUACUCACCAC 5’

No results for JS16_t0268726
query=JS16_t4024981, target=FGSG_01672, score=3.5, range=845-868, strand=1

target  5’ ACGGAAAGCGUCUUGGUGUGCUUG 3’
           : :::: .::::::.:::::::::
query   3’ UACCUUGUGCAGAAUCACACGAAC 5’

No results for JS16_t4025036
No results for JS16_t2232292
No results for JS16_t3128553
query=JS16_t3128569, target=FGSG_07781, score=4, range=1147-1165, strand=1

target  5’ UCAUCU-UUGUCUUACCACU 3’
           : :: : ::::::::::::
query   3’ ACUAAAUAACAGAAUGGUGC 5’

query=JS16_t0268730, target=FGSG_07836, score=4, range=481-500, strand=1

target  5’ AAGCAG-CUGAAGGAAGCUGG 3’
           : :::: :::::.:::::::
query   3’ UCCGUCAGACUUUCUUCGACA 5’

query=JS16_t0268730, target=FGSG_08042, score=4, range=1444-1463, strand=1

target  5’ AGGC-GCCUGAAAGAAGUUGG 3’
           :::: : ::::::::::.::
query   3’ UCCGUCAGACUUUCUUCGACA 5’

query=JS16_t0268730, target=FGSG_03049, score=4, range=785-805, strand=-1

target  5’ CGGGACUCUGGAAGAAGCUGU 3’
            :: : ::::.:::::::::
query   3’ UCCGUCAGACUUUCUUCGACA 5’

target  5’ GCCGCGAAGGGCCAGUUCU 3’
           : ::: :::..::::::::
query   3’ CCGCG-UUCUUGGUCAAGA 5’

query=JS16_t0188912, target=FGSG_10615, score=4, range=2288-2305, strand=-1

target  5’ UGCCCAAGACCCAGUUCU 3’
            :: ::::: ::::::::
query   3’ CCGCGUUCUUGGUCAAGA 5’

query=JS16_t0188912, target=FGSG_09495, score=4, range=1818-1835, strand=-1

target  5’ GACACAAGGACCAGUUCG 3’
           : : ::::.::::::::
query   3’ CCGCGUUCUUGGUCAAGA 5’

query=JS16_t0188912, target=FGSG_07013, score=4, range=1050-1066, strand=-1

target  5’ GUCG-AGGAACUAGUUCU 3’
           : :: :.::::.::::::
query   3’ CCGCGUUCUUGGUCAAGA 5’

query=JS16_t0188912, target=FGSG_00034, score=4, range=215-231, strand=-1

target  5’ GGCG-AAGAACGAGUUCA 3’
           :::: :::::: :::::
query   3’ CCGCGUUCUUGGUCAAGA 5’

query=JS16_t0188912, target=FGSG_06387, score=4, range=118-134, strand=-1

target  5’ GGCGCAA-AGCCAGUUCC 3’
           ::::::: :.:::::::
query   3’ CCGCGUUCUUGGUCAAGA 5’

No results for JS16_t2295875
No results for JS16_t2295922
No results for JS16_t2295928
No results for JS16_t3192139
No results for JS16_t2295961
No results for JS16_t4088952
No results for JS16_t0367125
No results for JS16_t2296058
No results for JS16_t2296071
No results for JS16_t4089070
No results for JS16_t0367129
No results for JS16_t0307809
query=JS16_t0584620, target=FGSG_09793, score=4, range=2069-2088, strand=1

target  5’ GUUUUGCUGUGCGAGACUUG 3’
           :: .:. :.:::::::::::
query   3’ CAUGAUAAUACGCUCUGAAC 5’

query=JS16_t0584620, target=FGSG_11186, score=4, range=1300-1319, strand=1

target  5’ GAUCUAUUAUGCGAGUCUUG 3’
           :  :::::::::::: ::::
query   3’ CAUGAUAAUACGCUCUGAAC 5’

query=JS16_t0584620, target=FGSG_06480, score=4, range=880-898, strand=1

target  5’ GGAC-AUUAUGCGAGACCUG 3’
           : :: ::::::::::: ::
query   3’ CAUGAUAAUACGCUCUGAAC 5’

query=JS16_t0584620, target=FGSG_01735, score=4, range=1435-1454, strand=-1

target  5’ GUGUUCUUAUGCGAGAAUUG 3’
           ::..: :::::::::: :::
query   3’ CAUGAUAAUACGCUCUGAAC 5’

query=JS16_t3192361, target=FGSG_07466, score=4, range=1500-1520, strand=-1

target  5’ AG-AGUUUCAUCCGUUGCUGGC 3’
           :: :: ::::.::::.::::::
query   3’ UCGUCCAAGUGGGCAGCGACCG 5’

query=JS16_t0584622, target=FGSG_10904, score=2.5, range=4418-4436, strand=-1

target  5’ GGAUGAGCAGGAAGCUUAA 3’
           :::.:::::::::::.::
query   3’ CCUGCUCGUCCUUCGGAUC 5’

query=JS16_t0584622, target=FGSG_00996, score=3, range=497-516, strand=-1

target  5’ AGACGCAGCAGGAGGCCUAG 3’
            :::: :::::::.::::::
query   3’ CCUGC-UCGUCCUUCGGAUC 5’

query=JS16_t0584622, target=FGSG_10532, score=4, range=2204-2222, strand=1

target  5’ GGACGAGCAGGGAGUUUAA 3’
           :::::::::::.::..::
```

```
query=JS16_t2232382, target=FGSG_04821, score=3, range=122-140, strand=1

target  5' AGGUCACUGCGUCUCUCAC 3'
           :  ::::::::::::::::
query   3' UAAAGUGACGCAGAGAGUC 5'

query=JS16_t2232382, target=FGSG_10434, score=3.5, range=619-637, strand=-1

target  5' CCUUUACUGCGUCUCUCAC 3'
             ::.::::::::::::::
query   3' UAAAGUGACGCAGAGAGUC 5'

query=JS16_t2232382, target=FGSG_07518, score=4, range=2882-2900, strand=1

target  5' AUAUCUUUGCGUCUCUCGG 3'
           :: :: .:::::::::::.:
query   3' UAAAGUGACGCAGAGAGUC 5'

query=JS16_t2232382, target=FGSG_09863, score=4, range=628-646, strand=1

target  5' GUUGUACUGCUUCUCUCAG 3'
           .:: .::::: ::::::::
query   3' UAAAGUGACGCAGAGAGUC 5'

query=JS16_t2232382, target=FGSG_04700, score=4, range=1346-1364, strand=1

target  5' UUUUCCCUUCGUCUCUCAG 3'
            :::: :: ::::::::::
query   3' UAAAGUGACGCAGAGAGUC 5'

query=JS16_t2232382, target=FGSG_04112, score=4, range=265-282, strand=1

target  5' AUUUCACUGCGCCUC-CAG 3'
           ::::::::::: ::: :::
query   3' UAAAGUGACGCAGAGAGUC 5'

query=JS16_t2232382, target=FGSG_00108, score=4, range=1073-1092, strand=-1

target  5' AUUUCUCUGCGUCUCUACAU 3'
           ::::: :::::::::: ::
query   3' UAAAGUGACGCAGAGA-GUC 5'

query=JS16_t2232382, target=FGSG_04165, score=4, range=1249-1266, strand=-1

target  5' ACUUC-CUGUGUCUCUUAG 3'
           : ::: :::.::::::.::
query   3' UAAAGUGACGCAGAGAGUC 5'

No results for JS16_t1335972
No results for JS16_t4025247
No results for JS16_t3128696
No results for JS16_t3128707
query=JS16_t0219720, target=FGSG_05568, score=4, range=1193-1215, strand=-1

target  5' GAAAGGUGUCCCAUGAAGUGAUG 3'
           :::  :.: :::::::::::::.
query   3' CUUGGCGC-GGGUACUUCACUAU 5'

query=JS16_t0305703, target=FGSG_02315, score=4, range=20734-20754, strand=1

target  5' CAAGAAGACUACGCCAACAGU 3'
            ::::::::::::: :::::
query   3' UUUCUUCUGAUGCGAUUGUCU 5'

query=JS16_t0305703, target=FGSG_05403, score=4, range=1611-1630, strand=1

target  5' AAAGACAACUACGC-AACAGA 3'
           :::::  ::::::: ::::::
query   3' UUUCUUCUGAUGCGAUUGUCU 5'

No results for JS16_t2232598
No results for JS16_t2232628
query=JS16_t3128876, target=FGSG_02255, score=3.5, range=173-195, strand=-1

target  5' GCCAGUGUUCUUUGCGACGAAGU 3'
           ::::::::.:::.::: ::::::
query   3' CGGUCACAGGAAGCGC-GCUUCA 5'

query=JS16_t1336181, target=FGSG_09900, score=2, range=1165-1182, strand=1

target  5' CCCAGGAGCACUGUGCUU 3'
            ::::::::::.::::::
query   3' CGGUCCUCGUGGCACGAA 5'

query=JS16_t1336181, target=FGSG_08312, score=2.5, range=49-66, strand=-1

target  5' GCCGGGAGCACCUUGCUU 3'
           :::.::::::::: :::::
query   3' CGGUCCUCGUGGCACGAA 5'

query=JS16_t1336181, target=FGSG_01531, score=2.5, range=781-798, strand=-1

query   3' CCUGCUCGUCCUUCGGAUC 5'

query=JS16_t0584622, target=FGSG_10796, score=4, range=147-164, strand=1

target  5' GGACGAGCGGGAAG-CUGG 3'
           ::::::::.::::: ::.:
query   3' CCUGCUCGUCCUUCGGAUC 5'

query=JS16_t0584622, target=FGSG_05656, score=4, range=426-444, strand=1

target  5' AAACGAGCAGGAGGCCUAU 3'
             :::::::::::.:::::
query   3' CCUGCUCGUCCUUCGGAUC 5'

query=JS16_t0584622, target=FGSG_09227, score=4, range=296-314, strand=-1

target  5' GGACGAGUAGGAGGCUUGG 3'
           :::::::.::::.::.:.:
query   3' CCUGCUCGUCCUUCGGAUC 5'

query=JS16_t0584622, target=FGSG_13500, score=4, range=323-341, strand=-1

target  5' GGACGAGGAGGGGGCCUAG 3'
           ::::::: :::..::::::
query   3' CCUGCUCGUCCUUCGGAUC 5'

query=JS16_t0584622, target=FGSG_06820, score=4, range=916-934, strand=-1

target  5' UGAAGAGCGGGAGGCCUAG 3'
            :: ::::.:::.::::::
query   3' CCUGCUCGUCCUUCGGAUC 5'

query=JS16_t0584622, target=FGSG_10970, score=4, range=461-478, strand=-1

target  5' AGGCGGGCAGGAAG-CUAG 3'
            :.::.:::::::: ::::
query   3' CCUGCUCGUCCUUCGGAUC 5'

query=JS16_t0584622, target=FGSG_03932, score=4, range=354-373, strand=-1

target  5' UGACGAUCAGGAAGCCGUAG 3'
            ::::: ::::::::: :::
query   3' CCUGCUCGUCCUUCGG-AUC 5'

No results for JS16_t4089138
No results for JS16_t3192424
query=JS16_t0693297, target=FGSG_10930, score=2, range=234-251, strand=1

target  5' CACGCUUGAGGUCUUGCA 3'
            :::::::::.:::::::
query   3' UUGCGAACUCUAGAACGU 5'

query=JS16_t0693297, target=FGSG_10719, score=2, range=1442-1458, strand=-1

target  5' AGU-CUUGAGAUCUUGCA 3'
           :..  ::::::::::::::
query   3' UUGCGAACUCUAGAACGU 5'

query=JS16_t0693297, target=FGSG_04955, score=2.5, range=57-74, strand=1

target  5' GAUGUUUGAGAUUUUGCA 3'
           .:.:.:::::::.:::::
query   3' UUGCGAACUCUAGAACGU 5'

query=JS16_t0693297, target=FGSG_11497, score=2.5, range=1162-1179, strand=1

target  5' GACGCUUGCGAUCUUGCA 3'
           .::::::: :::::::::
query   3' UUGCGAACUCUAGAACGU 5'

query=JS16_t0693297, target=FGSG_08360, score=2.5, range=1140-1157, strand=1

target  5' CAUGAUUGAGAUCUUGCA 3'
            :.: :::::::::::::
query   3' UUGCGAACUCUAGAACGU 5'

query=JS16_t0693297, target=FGSG_02270, score=3, range=743-760, strand=1

target  5' GGCCAUUGAGAUCUUGCA 3'
           ..:  :::::::::::::
query   3' UUGCGAACUCUAGAACGU 5'

query=JS16_t0693297, target=FGSG_09965, score=3, range=2912-2929, strand=-1

target  5' GCCGCUUGAGAUUUUGCG 3'
           .  ::::::::::.::::.
query   3' UUGCGAACUCUAGAACGU 5'

query=JS16_t0693297, target=FGSG_06301, score=3, range=425-441, strand=-1

target  5' AGC-CUUGGGAUCUUGCG 3'
           :.: ::::.::::::::.
```

```
target  5' GGCGGGAGCACCGUGCUG 3'
           : :.:::::::::::::
query   3' CGGUCCUCGUGGCACGAA 5'

query=JS16_t1336181, target=FGSG_08664, score=3, range=143-160, strand=1

target  5' GCCAGGGGCACAGUGCUU 3'
           ::::::.:::: ::::::
query   3' CGGUCCUCGUGGCACGAA 5'

query=JS16_t1336181, target=FGSG_00340, score=3, range=1873-1890, strand=1

target  5' GGCAGGAGUAUCGUGCUU 3'
           : ::::::.:.:::::::
query   3' CGGUCCUCGUGGCACGAA 5'

query=JS16_t1336181, target=FGSG_03198, score=3, range=1076-1093, strand=1

target  5' GACAGGAGUAUCGUGCUU 3'
           : ::::::.:.:::::::
query   3' CGGUCCUCGUGGCACGAA 5'

query=JS16_t1336181, target=FGSG_04207, score=3.5, range=313-330, strand=-1

target  5' AGCGGGAGCACCGUGCUG 3'
             :.:::::::::::::
query   3' CGGUCCUCGUGGCACGAA 5'

query=JS16_t1336181, target=FGSG_10796, score=4, range=791-808, strand=1

target  5' UUUAUGAGCACCGUGCUG 3'
            ..: ::::::::::::
query   3' CGGUCCUCGUGGCACGAA 5'

query=JS16_t1336181, target=FGSG_05691, score=4, range=2314-2330, strand=1

target  5' GCCA-GAGCACUGUGUUC 3'
           :::: ::::::.:::.:
query   3' CGGUCCUCGUGGCACGAA 5'

query=JS16_t1336181, target=FGSG_10464, score=4, range=1546-1563, strand=1

target  5' ACCAAGCGCACCGUGCUU 3'
            ::: : :::::::::::
query   3' CGGUCCUCGUGGCACGAA 5'

query=JS16_t1336181, target=FGSG_13575, score=4, range=426-442, strand=1

target  5' GACAGGGGCACCG-GCUU 3'
           : ::::.:::::: ::::
query   3' CGGUCCUCGUGGCACGAA 5'

query=JS16_t1336181, target=FGSG_10166, score=4, range=300-316, strand=1

target  5' GCCACGAGCGCCGU-CUU 3'
           :::: ::::.:::: :::
query   3' CGGUCCUCGUGGCACGAA 5'

query=JS16_t1336181, target=FGSG_04727, score=4, range=372-388, strand=1

target  5' ACCAGGAGCACC-UGCUC 3'
            ::::::::::: ::::
query   3' CGGUCCUCGUGGCACGAA 5'

query=JS16_t1336181, target=FGSG_12904, score=4, range=2751-2767, strand=1

target  5' GGCA-GAGUACCGUGCUC 3'
           : :: :::.::::::::
query   3' CGGUCCUCGUGGCACGAA 5'

query=JS16_t1336181, target=FGSG_06984, score=4, range=82-98, strand=1

target  5' GUCGUGAGCACCGU-CUU 3'
           :.:. ::::::::: :::
query   3' CGGUCCUCGUGGCACGAA 5'

query=JS16_t1336181, target=FGSG_03968, score=4, range=688-705, strand=-1

target  5' ACCAGGAGCAUCGUACUU 3'
            :::::::::.::: :::
query   3' CGGUCCUCGUGGCACGAA 5'

query=JS16_t1336181, target=FGSG_00294, score=4, range=1426-1442, strand=-1

target  5' GCCAGG-GCAUUGUGCUU 3'
           :::::: :::..::::::
query   3' CGGUCCUCGUGGCACGAA 5'

query=JS16_t1336181, target=FGSG_12421, score=4, range=1666-1683, strand=-1

target  5' GCCAGGAGCUCCAUGCUU 3'
query   3' UUGCGAACUCUAGAACGU 5'

query=JS16_t0693297, target=FGSG_04899, score=3, range=383-400, strand=-1

target  5' AACCCUUGAAAUCUUGCA 3'
           ::: ::::: ::::::::
query   3' UUGCGAACUCUAGAACGU 5'

query=JS16_t0693297, target=FGSG_07139, score=3, range=2275-2292, strand=-1

target  5' CAGGCUUGAGAUCUUGCU 3'
            : :::::::::::::
query   3' UUGCGAACUCUAGAACGU 5'

query=JS16_t0693297, target=FGSG_07615, score=3, range=830-846, strand=-1

target  5' AAC-CUUGAGAGCUUGCA 3'
           ::: ::::::: ::::::
query   3' UUGCGAACUCUAGAACGU 5'

query=JS16_t0693297, target=FGSG_13635, score=3, range=215-231, strand=-1

target  5' AUCGCUU-AGAUCUUGCA 3'
           : ::::: ::::::::::
query   3' UUGCGAACUCUAGAACGU 5'

query=JS16_t0693297, target=FGSG_08308, score=3.5, range=3155-3172, strand=1

target  5' GCCGCUUGAGAUCUUUCA 3'
           . :::::::::::: ::
query   3' UUGCGAACUCUAGAACGU 5'

query=JS16_t0693297, target=FGSG_02732, score=3.5, range=1389-1406, strand=1

target  5' AGCACUAGAGAUCUUGCA 3'
           :.: :: ::::::::::
query   3' UUGCGAACUCUAGAACGU 5'

query=JS16_t0693297, target=FGSG_12084, score=3.5, range=268-285, strand=1

target  5' AGCGAUAGAGAUCUUGCA 3'
           :.:: : ::::::::::
query   3' UUGCGAACUCUAGAACGU 5'

query=JS16_t0693297, target=FGSG_06025, score=3.5, range=900-917, strand=1

target  5' AAUGAUUGAGUUCUUGCA 3'
           ::.: ::::: :::::::
query   3' UUGCGAACUCUAGAACGU 5'

query=JS16_t0693297, target=FGSG_01337, score=3.5, range=896-912, strand=1

target  5' GAC-UUUGGGAUCUUGCG 3'
           .:: .:::.::::::::.
query   3' UUGCGAACUCUAGAACGU 5'

query=JS16_t0693297, target=FGSG_05777, score=3.5, range=1317-1334, strand=1

target  5' AACUCUUGAGAUCUUCCG 3'
           ::: ::::::::::: :.
query   3' UUGCGAACUCUAGAACGU 5'

query=JS16_t0693297, target=FGSG_02237, score=3.5, range=2617-2634, strand=1

target  5' AAGAUUUGGGAUCUUGCA 3'
           ::  .:::.:::::::::
query   3' UUGCGAACUCUAGAACGU 5'

query=JS16_t0693297, target=FGSG_04534, score=3.5, range=276-292, strand=1

target  5' GAC-CUUGAGGUCUUGCU 3'
           .:: ::::::.::::::
query   3' UUGCGAACUCUAGAACGU 5'

query=JS16_t0693297, target=FGSG_01446, score=3.5, range=630-646, strand=1

target  5' AAU-CUUGGGAUCUUGCU 3'
           ::. ::::.:::::::
query   3' UUGCGAACUCUAGAACGU 5'

query=JS16_t0693297, target=FGSG_09146, score=3.5, range=2560-2576, strand=1

target  5' AAUG-UUGAGAUCAUGCA 3'
           ::.: :::::::: ::::
query   3' UUGCGAACUCUAGAACGU 5'

query=JS16_t0693297, target=FGSG_07916, score=3.5, range=132-150, strand=1

target  5' GAAGCUUGAGGAUCUUGCA 3'
           .: :::::: :::::::::
query   3' UUGCGAACU-CUAGAACGU 5'
```

```
           ::::::::: :: :::::
query   3' CGGUCCUCGUGGCACGAA 5'

query=JS16_t1336181, target=FGSG_09790, score=4, range=1849-1866, strand=-1

target  5' GCCACGCGCGCCGUGCUU 3'
           :::: : ::.::::::::
query   3' CGGUCCUCGUGGCACGAA 5'

query=JS16_t1336181, target=FGSG_11576, score=4, range=912-929, strand=-1

target  5' GCCCGGAGUACCUUGCUU 3'
           ::: ::::.::: :::::
query   3' CGGUCCUCGUGGCACGAA 5'

query=JS16_t1336181, target=FGSG_05508, score=4, range=199-216, strand=-1

target  5' GGCAGGGGCACCGUUCUU 3'
           : ::::.::::::: :::
query   3' CGGUCCUCGUGGCACGAA 5'

query=JS16_t1336181, target=FGSG_00849, score=4, range=952-969, strand=-1

target  5' GCGAGGAGCACCGUACUG 3'
           :: ::::::::::: ::
query   3' CGGUCCUCGUGGCACGAA 5'

query=JS16_t1336181, target=FGSG_02138, score=4, range=667-683, strand=-1

target  5' GCC-UGAGCACCGAGCUU 3'
           :::  :::::::: ::::
query   3' CGGUCCUCGUGGCACGAA 5'

query=JS16_t1336181, target=FGSG_10254, score=4, range=244-260, strand=-1

target  5' UCCAGGAGUACC-UGCUU 3'
            :::::::.::: :::::
query   3' CGGUCCUCGUGGCACGAA 5'

query=JS16_t0305708, target=FGSG_08511, score=4, range=688-707, strand=1

target  5' GUCAUGAUUGGUAGCAUGUC 3'
            ::::::. :::::::::::
query   3' GAGUACUGUCCAUCGUACAG 5'

query=JS16_t0305708, target=FGSG_03157, score=4, range=267-285, strand=-1

target  5' CUCUUGGCAGGUAG-AUGUU 3'
           ::: ::.::::::: ::::.
query   3' GAGUACUGUCCAUCGUACAG 5'

query=JS16_t3128886, target=FGSG_01302, score=3.5, range=1350-1369, strand=1

target  5' UGGUGUUGGCGGGGGAGGUU 3'
            .:.::.::::::::::.::
query   3' CUCGCAGCCGCCCCCUCUAA 5'

query=JS16_t3128886, target=FGSG_05744, score=3.5, range=141-161, strand=-1

target  5' GAGACGUUGGCGGGGGUGAUU 3'
           ::: :::.:::::::: ::::
query   3' CUC-GCAGCCGCCCCCUCUAA 5'

query=JS16_t3128886, target=FGSG_08986, score=3.5, range=17-35, strand=-1

target  5' GAGUG-CGCCGGGGGAGAUU 3'
           :::.: :: ::::::::::
query   3' CUCGCAGCCGCCCCCUCUAA 5'

query=JS16_t3128886, target=FGSG_09177, score=4, range=1274-1293, strand=1

target  5' GAGAGACGGUGGGGGAGAUC 3'
           ::: : :::.:::::::::
query   3' CUCGCAGCCGCCCCCUCUAA 5'

query=JS16_t3128886, target=FGSG_07069, score=4, range=333-352, strand=-1

target  5' GGGUGCCGGUGGGGGAGAUA 3'
           :.:.: :::.:::::::::
query   3' CUCGCAGCCGCCCCCUCUAA 5'

query=JS16_t3128886, target=FGSG_10597, score=4, range=115-133, strand=-1

target  5' GAGAGUCGGU-GGGGAGAUU 3'
           ::: :::::. :::::::::
query   3' CUCGCAGCCGCCCCCUCUAA 5'

No results for JS16_t0268738
query=JS16_t0795009, target=FGSG_04834, score=3.5, range=62-80, strand=-1

target  5' UA-GUUGCAGGUGGCGUUGA 3'
           .: ::: :::::.:::::::

query=JS16_t0693297, target=FGSG_11627, score=3.5, range=379-396, strand=-1

target  5' CAUGCUUGAGGUCUUGCC 3'
            :.:::::::.::::::
query   3' UUGCGAACUCUAGAACGU 5'

query=JS16_t0693297, target=FGSG_11053, score=3.5, range=143-160, strand=-1

target  5' GACGCUUGAAAUCUUGCC 3'
           .::::::::: :::::::
query   3' UUGCGAACUCUAGAACGU 5'

query=JS16_t0693297, target=FGSG_12922, score=3.5, range=1318-1334, strand=-1

target  5' AGCGC-UGAGAUCUUGUA 3'
           :.::: :::::::::::.:
query   3' UUGCGAACUCUAGAACGU 5'

query=JS16_t0693297, target=FGSG_09782, score=3.5, range=3071-3088, strand=-1

target  5' ACCACUUGAGAUUUUGCG 3'
           : : ::::::::.::::.
query   3' UUGCGAACUCUAGAACGU 5'

query=JS16_t0693297, target=FGSG_00307, score=3.5, range=53-71, strand=-1

target  5' GUCGCGUUGGGAUCUUGCA 3'
           .  ::: :::.:::::::::
query   3' UUGCG-AACUCUAGAACGU 5'

query=JS16_t0693297, target=FGSG_08365, score=3.5, range=92-108, strand=-1

target  5' GAC-CUUGAGAUCUUGGA 3'
           .:: :::::::::::: :
query   3' UUGCGAACUCUAGAACGU 5'

query=JS16_t0693297, target=FGSG_02937, score=3.5, range=1226-1242, strand=-1

target  5' GGAG-UUGAGAUCUUGCG 3'
           ..  : ::::::::::::.
query   3' UUGCGAACUCUAGAACGU 5'

query=JS16_t0693297, target=FGSG_08680, score=3.5, range=35-53, strand=-1

target  5' GGCGCUUGAGGAUCUUGCG 3'
           ..::::::: ::::::::.
query   3' UUGCGAACU-CUAGAACGU 5'

query=JS16_t0693297, target=FGSG_11113, score=4, range=1185-1201, strand=1

target  5' GAUGCUUGAGAUCUU-UA 3'
           .:.::::::::::::: .:
query   3' UUGCGAACUCUAGAACGU 5'

query=JS16_t0693297, target=FGSG_03409, score=4, range=498-516, strand=1

target  5' GGCGUCUUGGGAUCUUGCU 3'
           ..:: ::::.::::::::
query   3' UUGC-GAACUCUAGAACGU 5'

query=JS16_t0693297, target=FGSG_09341, score=4, range=1794-1811, strand=1

target  5' AGAGUUUGAGAUGUUGCA 3'
           :.  :.::::::: :::::
query   3' UUGCGAACUCUAGAACGU 5'

query=JS16_t0693297, target=FGSG_04131, score=4, range=715-732, strand=1

target  5' GUCAUUUGGGAUCUUGCA 3'
           .  : .:::.:::::::::
query   3' UUGCGAACUCUAGAACGU 5'

query=JS16_t0693297, target=FGSG_04591, score=4, range=399-415, strand=1

target  5' CGCGUUUGAGAUCUU-CA 3'
            .::.:::::::::: ::
query   3' UUGCGAACUCUAGAACGU 5'

query=JS16_t0693297, target=FGSG_01632, score=4, range=2435-2451, strand=1

target  5' AGC-UUUGGGAUCUUGCU 3'
           :.: .:::.::::::::
query   3' UUGCGAACUCUAGAACGU 5'

query=JS16_t0693297, target=FGSG_05555, score=4, range=2211-2228, strand=1

target  5' CAAGCUUGAGCUCUUGCA 3'
             : ::::::: :::::::
query   3' UUGCGAACUCUAGAACGU 5'

query=JS16_t0693297, target=FGSG_09401, score=4, range=961-977, strand=1
```

```
query   3' GUCCAAAGUCCAUCGCAACU 5'

No results for JS16_t0467995
query=JS16_t0188473, target=FGSG_00600, score=3, range=221-241, strand=1

target  5' GCGAUGACACCAGAGCAUGGG 3'
             :::::: ::::::::::::
query   3' GGCUACU-UGGUCUCGUACCA 5'

query=JS16_t0188473, target=FGSG_06858, score=3, range=430-449, strand=-1

target  5' CCGAUGAGCCAGAGCAGGGU 3'
           :::::::.:::::::: :::
query   3' GGCUACUUGGUCUCGUACCA 5'

query=JS16_t0188473, target=FGSG_03964, score=3, range=4337-4355, strand=-1

target  5' CUGGU-CACCAGAGCAUGGU 3'
           :.:.:  :::::::::::::
query   3' GGCUACUUGGUCUCGUACCA 5'

query=JS16_t0188473, target=FGSG_00031, score=3.5, range=3262-3281, strand=1

target  5' UCCAUGAACCAGGGCGUGGU 3'
           .: :::::::::.::.::::
query   3' GGCUACUUGGUCUCGUACCA 5'

query=JS16_t0188473, target=FGSG_10516, score=3.5, range=1093-1112, strand=1

target  5' CCGACGGGCCAGAGCAUGGG 3'
           :::: :..:::::::::::
query   3' GGCUACUUGGUCUCGUACCA 5'

query=JS16_t0188473, target=FGSG_06174, score=3.5, range=1940-1958, strand=1

target  5' CCGGU-AACCAGAUCAUGGU 3'
           :::.: ::::::: ::::::
query   3' GGCUACUUGGUCUCGUACCA 5'

query=JS16_t0188473, target=FGSG_08445, score=4, range=320-339, strand=1

target  5' ACAAUGUACCAGAGCGUGGU 3'
            : ::: ::::::::.::::
query   3' GGCUACUUGGUCUCGUACCA 5'

query=JS16_t0188473, target=FGSG_09799, score=4, range=1687-1706, strand=1

target  5' CUGGCAAACCAGAGCAUGGA 3'
           :.:.   ::::::::::::
query   3' GGCUACUUGGUCUCGUACCA 5'

query=JS16_t0188473, target=FGSG_02315, score=4, range=10531-10550, strand=1

target  5' CACUUCAACCAGAGCAUGGU 3'
           :   : ::::::::::::::
query   3' GGCUACUUGGUCUCGUACCA 5'

query=JS16_t0188473, target=FGSG_12255, score=4, range=1677-1695, strand=1

target  5' CCCAAGAA-CAGAGCAUGGU 3'
           :: : ::: ::::::::::
query   3' GGCUACUUGGUCUCGUACCA 5'

query=JS16_t0188473, target=FGSG_02700, score=4, range=437-455, strand=1

target  5' CCGA-CAACCAGAUCAUGGU 3'
           ::::  ::::::: ::::::
query   3' GGCUACUUGGUCUCGUACCA 5'

query=JS16_t0188473, target=FGSG_12548, score=4, range=124-143, strand=-1

target  5' UCGGUGAACCAGACCGUGGU 3'
           .::.::::::::: :.::::
query   3' GGCUACUUGGUCUCGUACCA 5'

No results for JS16_t3129067
No results for JS16_t0188474
No results for JS16_t3129127
query=JS16_t1336546, target=FGSG_11170, score=2, range=2008-2027, strand=-1

target  5' GUUUUGAUGUCGUCAACCAU 3'
            : :::::::::::::::::
query   3' GACAACUACAGCAGUUGGUA 5'

query=JS16_t1336546, target=FGSG_04274, score=3, range=111-130, strand=-1

target  5' CCGUUGAUGUCGUCAAUCGU 3'
           : ::::::::::::::.:.:
query   3' GACAACUACAGCAGUUGGUA 5'

query=JS16_t1336546, target=FGSG_05685, score=3, range=589-609, strand=-1

target  5' GGCG-UUGACAUCUUGCA 3'
           ..:: :::: ::::::::
query   3' UUGCGAACUCUAGAACGU 5'

query=JS16_t0693297, target=FGSG_01204, score=4, range=952-968, strand=1

target  5' CACG-UUGAGAUCUUGUU 3'
            ::: :::::::::::.
query   3' UUGCGAACUCUAGAACGU 5'

query=JS16_t0693297, target=FGSG_11407, score=4, range=709-725, strand=1

target  5' AAC-AUUGAGGUCUUGCU 3'
           :::  :::::.::::::
query   3' UUGCGAACUCUAGAACGU 5'

query=JS16_t0693297, target=FGSG_10116, score=4, range=1258-1275, strand=-1

target  5' AAUGUUGGAGAUCUUGUA 3'
           ::.:.: :::::::::.:
query   3' UUGCGAACUCUAGAACGU 5'

query=JS16_t0693297, target=FGSG_11609, score=4, range=1049-1066, strand=-1

target  5' AGCGUUUGAGAUUUUGAA 3'
           :.::.:::::::.::: :
query   3' UUGCGAACUCUAGAACGU 5'

query=JS16_t0693297, target=FGSG_03626, score=4, range=1477-1494, strand=-1

target  5' AGUGUUUGCGAUCUUGCG 3'
           :..:.::: ::::::::.
query   3' UUGCGAACUCUAGAACGU 5'

query=JS16_t0693297, target=FGSG_10142, score=4, range=657-674, strand=-1

target  5' UGUGCUUGAGAUCUUGGA 3'
            ..::::::::::::: :
query   3' UUGCGAACUCUAGAACGU 5'

query=JS16_t0693297, target=FGSG_07227, score=4, range=114-130, strand=-1

target  5' GAUGCUUGAGAUUUU-CA 3'
           .:.:::::::::.:: ::
query   3' UUGCGAACUCUAGAACGU 5'

query=JS16_t0693297, target=FGSG_01805, score=4, range=219-235, strand=-1

target  5' AGUGCUUGAGGUCUU-CA 3'
           :..:::::::.:::: ::
query   3' UUGCGAACUCUAGAACGU 5'

query=JS16_t0693297, target=FGSG_03497, score=4, range=1103-1119, strand=-1

target  5' GGCG-UUGAGGUUUUGCA 3'
           ..:: :::::.:.:::::
query   3' UUGCGAACUCUAGAACGU 5'

query=JS16_t0693297, target=FGSG_05179, score=4, range=1694-1710, strand=-1

target  5' GAC-CUUGAGGUCUUGUG 3'
           .:: ::::::.:::::..
query   3' UUGCGAACUCUAGAACGU 5'

query=JS16_t0693297, target=FGSG_11293, score=4, range=1628-1645, strand=-1

target  5' AAUUCUUGAGAUGUUGCG 3'
           ::.  :::::::: ::::.
query   3' UUGCGAACUCUAGAACGU 5'

query=JS16_t0693297, target=FGSG_00714, score=4, range=1247-1264, strand=-1

target  5' GGGGCUUGACAUCUUGCA 3'
           ..  :::::: ::::::::
query   3' UUGCGAACUCUAGAACGU 5'

query=JS16_t0693297, target=FGSG_03507, score=4, range=479-497, strand=-1

target  5' AUUGCCUUGAGGUCUUGCG 3'
           : .: ::::::.::::::.
query   3' UUGC-GAACUCUAGAACGU 5'

query=JS16_t0693297, target=FGSG_05492, score=4, range=1844-1861, strand=-1

target  5' AACACUUGAGAGCUUGCU 3'
           ::: ::::::: :::::
query   3' UUGCGAACUCUAGAACGU 5'

query=JS16_t0693297, target=FGSG_06334, score=4, range=1324-1340, strand=-1

target  5' GGU-UUUGAGAUCUUGCU 3'
           ... .:::::::::::::
```

```
target  5’ AUCUUCGAUGUCGUCAACCAU 3’
            : :: :::::::::::::::
query   3’ GACAA-CUACAGCAGUUGGUA 5’

query=JS16_t1336546, target=FGSG_08314, score=3.5, range=668-687, strand=1

target  5’ UUGUCGAUGUUGUCAACCAA 3’
           .::: :::::.::::::::
query   3’ GACAACUACAGCAGUUGGUA 5’

query=JS16_t1336546, target=FGSG_12024, score=3.5, range=732-750, strand=1

target  5’ CUG-UGGUGUCGUCAACCCU 3’
           ::: ::.:::::::::: :
query   3’ GACAACUACAGCAGUUGGUA 5’

query=JS16_t1336546, target=FGSG_03407, score=3.5, range=727-746, strand=-1

target  5’ UUGUUUCUGUCGUCAACCAA 3’
           .::::  ::::::::::::
query   3’ GACAACUACAGCAGUUGGUA 5’

query=JS16_t1336546, target=FGSG_07051, score=4, range=84-102, strand=1

target  5’ CU-UUGAUGUCGCCAACCAG 3’
           :: ::::::::: ::::::
query   3’ GACAACUACAGCAGUUGGUA 5’

query=JS16_t1336546, target=FGSG_02069, score=4, range=802-821, strand=-1

target  5’ CCGUUGAUGUUGUCAACGAU 3’
           : ::::::::.:::::: ::
query   3’ GACAACUACAGCAGUUGGUA 5’

query=JS16_t1336546, target=FGSG_03199, score=4, range=1-19, strand=-1

target  5’ CU-UUGCUGUCGUUAGCCAU 3’
           :: ::: :::::.:.::::
query   3’ GACAACUACAGCAGUUGGUA 5’

query=JS16_t1336546, target=FGSG_10523, score=4, range=1126-1146, strand=-1

target  5’ CGCUUGCAUGUCGUCAACCAA 3’
           :  ::: :::::::::::::
query   3’ GACAAC-UACAGCAGUUGGUA 5’

No results for JS16_t0795043
query=JS16_t0157413, target=FGSG_08233, score=3, range=569-586, strand=-1

target  5’ CAGGACU-AAGCAAGUGUA 3’
           ::::::: :::::::::.:
query   3’ GUCCUGAUUUCGUUCACGU 5’

query=JS16_t0157413, target=FGSG_05325, score=4, range=2139-2157, strand=1

target  5’ CAGGAAUAGAGCAAGUGUC 3’
           ::::: ::.::::::::.
query   3’ GUCCUGAUUUCGUUCACGU 5’

query=JS16_t0157413, target=FGSG_07260, score=4, range=50-68, strand=1

target  5’ UGGAAGUGAAGCAAGUGCA 3’
           ..: : :.:::::::::::
query   3’ GUCCUGAUUUCGUUCACGU 5’

query=JS16_t0157413, target=FGSG_02184, score=4, range=807-824, strand=1

target  5’ CAUGACUGAAGCAAGU-CA 3’
           :: ::::.:::::::: ::
query   3’ GUCCUGAUUUCGUUCACGU 5’

query=JS16_t0157413, target=FGSG_05442, score=4, range=562-579, strand=1

target  5’ CAGGA-UAAAUCGAGUGCA 3’
           ::::: :::: :.::::::
query   3’ GUCCUGAUUUCGUUCACGU 5’

query=JS16_t0157413, target=FGSG_07803, score=4, range=486-504, strand=-1

target  5’ CGUGGCUAGAGCAGGUGCA 3’
           :.  :.:::.::::.:::::
query   3’ GUCCUGAUUUCGUUCACGU 5’

query=JS16_t0157413, target=FGSG_10339, score=4, range=583-601, strand=-1

target  5’ CAGGACUAAAGCAGAUGCC 3’
           :::::::::::::. :::
query   3’ GUCCUGAUUUCGUUCACGU 5’

query=JS16_t0157413, target=FGSG_11240, score=4, range=1893-1911, strand=-1

target  5’ UGUGACUAAAGCAAGUACA 3’

query   3’ UUGCGAACUCUAGAACGU 5’

query=JS16_t0693297, target=FGSG_00540, score=4, range=380-396, strand=-1

target  5’ AACG-UUGAGGUCUUCCA 3’
           :::: :::::.:::: ::
query   3’ UUGCGAACUCUAGAACGU 5’

query=JS16_t0693297, target=FGSG_05135, score=4, range=974-990, strand=-1

target  5’ AGU-CUUGAGAUCUUGGA 3’
           :.. ::::::::::: :
query   3’ UUGCGAACUCUAGAACGU 5’

query=JS16_t0693297, target=FGSG_00840, score=4, range=2441-2457, strand=-1

target  5’ GGC-CUUGAGAGCUUGCA 3’
           ..: ::::::: ::::::
query   3’ UUGCGAACUCUAGAACGU 5’

query=JS16_t0693297, target=FGSG_04825, score=4, range=555-571, strand=-1

target  5’ AGU-CUUGUGAUCUUGCA 3’
           :.. :::: :::::::::
query   3’ UUGCGAACUCUAGAACGU 5’

query=JS16_t0693297, target=FGSG_00504, score=4, range=1187-1204, strand=-1

target  5’ AAGGGUUGAGAUCUCGCA 3’
           :: : ::::::::: :::
query   3’ UUGCGAACUCUAGAACGU 5’

query=JS16_t0693297, target=FGSG_09383, score=4, range=613-630, strand=-1

target  5’ AACAGUUGAGAUCUUGAA 3’
           :::  ::::::::::: :
query   3’ UUGCGAACUCUAGAACGU 5’

query=JS16_t0693297, target=FGSG_00773, score=4, range=67-85, strand=-1

target  5’ AAAGCAUAGAGAUCUUGCA 3’
           :: :: : :::::::::::
query   3’ UUGCG-AACUCUAGAACGU 5’

query=JS16_t0693297, target=FGSG_05736, score=4, range=3018-3034, strand=-1

target  5’ CACUCUUGAGAUC-UGCA 3’
            :: ::::::::: ::::
query   3’ UUGCGAACUCUAGAACGU 5’

query=JS16_t4089227, target=FGSG_10469, score=4, range=424-442, strand=-1

target  5’ GAAGGACGGUGUCUCUUGA 3’
           .:::.::::.:::::.::
query   3’ UUUCUUGCCGCAGAGGACC 5’

query=JS16_t4089227, target=FGSG_13028, score=4, range=1460-1477, strand=-1

target  5’ GGAGAAUGGCGUC-CCUGG 3’
           ..::::.:::::: :::::
query   3’ UUUCUUGCCGCAGAGGACC 5’

query=JS16_t4089227, target=FGSG_02475, score=4, range=60-77, strand=-1

target  5’ AUAGAA-GGUGUCUCCUGG 3’
           : :::: ::.:::::::::
query   3’ UUUCUUGCCGCAGAGGACC 5’

query=JS16_t4089227, target=FGSG_01886, score=4, range=877-896, strand=-1

target  5’ AAAGACUCUGCGUCUCCUGG 3’
           :::::  : :::::::::::
query   3’ UUUCU-UGCCGCAGAGGACC 5’

No results for JS16_t3192528
No results for JS16_t2296389
query=JS16_t4089351, target=FGSG_04707, score=3, range=503-520, strand=-1

target  5’ CGCUAUUCUCUGCUCUUU 3’
           :: :::: ::::::::::
query   3’ GCUAUAAAAGACGAGAAA 5’

query=JS16_t4089351, target=FGSG_06626, score=3, range=907-925, strand=-1

target  5’ CGAUCUUUUCUGCUUCUUU 3’
           :::: :::::::: :::::
query   3’ GCUAUAAAAGACG-AGAAA 5’

query=JS16_t4089351, target=FGSG_01503, score=3.5, range=785-802, strand=1

target  5’ CCAUGUUUGCUGCUCUUU 3’
           : ::.::: :::::::::
```

```
           .. ::::::::::::: ::
query   3’ GUCCUGAUUUCGUUCACGU 5’

query=JS16_t0157413, target=FGSG_05395, score=4, range=435-453, strand=-1

target  5’ UGGCACUAAAGCAAGGGCA 3’
           ..: ::::::::::: :::
query   3’ GUCCUGAUUUCGUUCACGU 5’

query=JS16_t0157413, target=FGSG_03728, score=4, range=814-832, strand=-1

target  5’ CAGGCAUGAAGCAAGUGUA 3’
           ::::  :.::::::::.:
query   3’ GUCCUGAUUUCGUUCACGU 5’

query=JS16_t0157413, target=FGSG_07760, score=4, range=333-350, strand=-1

target  5’ AAGGAC-GAAGCAAGUGCA 3’
            ::::: .::::::::::
query   3’ GUCCUGAUUUCGUUCACGU 5’

query=JS16_t0157413, target=FGSG_11376, score=4, range=750-767, strand=-1

target  5’ AAGGAC-AAGGCAAGUGCA 3’
            ::::: ::.::::::::
query   3’ GUCCUGAUUUCGUUCACGU 5’

No results for JS16_t0254556
query=JS16_t3129341, target=FGSG_08561, score=4, range=366-387, strand=1

target  5’ UUUCCAGGUUCUC-CGACUCAAG 3’
           ::.:::: ::::: ::::::::.
query   3’ AAGGGUCAAAGAGCGCUGAGUUU 5’

query=JS16_t2233137, target=FGSG_06968, score=3, range=928-948, strand=1

target  5’ UGGGACGCGAGAAGCUGGGAG 3’
           ::.: ::::::::.::::::.
query   3’ ACUCAGCGCUCUUUGACCCUU 5’

query=JS16_t2233137, target=FGSG_04734, score=3, range=1497-1517, strand=1

target  5’ CGAGUCGGGAGAAACUGGGAC 3’
            :::::: ::::::::::::
query   3’ ACUCAGCGCUCUUUGACCCUU 5’

query=JS16_t2233137, target=FGSG_11175, score=3.5, range=433-452, strand=-1

target  5’ UGAGGUG-GAGAAACUGGGGA 3’
           :::: .: :::::::::::.:
query   3’ ACUCAGCGCUCUUUGACCCUU 5’

query=JS16_t2233137, target=FGSG_01563, score=4, range=394-413, strand=1

target  5’ UUAG-CGCGAGAAACUCGGAA 3’
           : :: ::::::::::: ::::
query   3’ ACUCAGCGCUCUUUGACCCUU 5’

No results for JS16_t0335536
No results for JS16_t1336736
query=JS16_t1336739, target=FGSG_00303, score=3, range=268-289, strand=1

target  5’ UUGGCCGCGGCGGAUUACCUGG 3’
            ::::::  :::::::::::::
query   3’ UACCGGCAGCGCCUAAUGGACC 5’

No results for JS16_t4026023
No results for JS16_t2233290
query=JS16_t3129514, target=FGSG_09073, score=2, range=2439-2456, strand=1

target  5’ UGAUUUCACUUGGGCAUC 3’
           :::  :::::::::::::
query   3’ ACUUUAGUGAACCCGUAG 5’

query=JS16_t3129514, target=FGSG_02982, score=2.5, range=931-948, strand=-1

target  5’ UGAGAUUAUUUGGGCAUC 3’
           :::.::.:.:::::::::
query   3’ ACUUUAGUGAACCCGUAG 5’

query=JS16_t3129514, target=FGSG_07230, score=3.5, range=738-756, strand=1

target  5’ AGAACGUCGCUUGGGCAUC 3’
            ::: .::.::::::::::
query   3’ ACUU-UAGUGAACCCGUAG 5’

query=JS16_t3129514, target=FGSG_00285, score=3.5, range=1104-1121, strand=1

target  5’ UGCAUUUACUUGGGCAUU 3’
           :: : :.::::::::::.
query   3’ ACUUUAGUGAACCCGUAG 5’

query   3’ GCUAUAAAAGACGAGAAA 5’

query=JS16_t4089351, target=FGSG_12550, score=3.5, range=1284-1300, strand=-1

target  5’ CGGUAUUUCU-UUCUUU 3’
           ::.::::::: .:::::
query   3’ GCUAUAAAAGACGAGAAA 5’

query=JS16_t4089351, target=FGSG_01837, score=3.5, range=275-293, strand=-1

target  5’ UGGUGCUUUUCUGCUCUUG 3’
           .:.:.  ::::::::::::
query   3’ GCUAU-AAAAGACGAGAAA 5’

query=JS16_t4089351, target=FGSG_06139, score=4, range=2202-2219, strand=1

target  5’ CAGUGUUUACUGCUCUUU 3’
           : .:.::: :::::::::
query   3’ GCUAUAAAAGACGAGAAA 5’

query=JS16_t4089351, target=FGSG_04033, score=4, range=2265-2283, strand=1

target  5’ UGGUCUUUUGCUGCUCUUU 3’
           .:.: :::: :::::::::
query   3’ GCUAUAAAA-GACGAGAAA 5’

query=JS16_t4089351, target=FGSG_01484, score=4, range=67-84, strand=1

target  5’ ACUGAUUUUCUGCUCUUU 3’
               ::::::::::::::
query   3’ GCUAUAAAAGACGAGAAA 5’

query=JS16_t4089351, target=FGSG_05972, score=4, range=98-115, strand=1

target  5’ CGCCAUUUUUUGCUCUUC 3’
           ::  :::::.:::::::
query   3’ GCUAUAAAAGACGAGAAA 5’

query=JS16_t4089351, target=FGSG_01473, score=4, range=2754-2771, strand=1

target  5’ CAUUAUUUUCAGCUCUUU 3’
           :  ::::::: :::::::
query   3’ GCUAUAAAAGACGAGAAA 5’

query=JS16_t4089351, target=FGSG_11648, score=4, range=768-784, strand=1

target  5’ CGAGA-UUUCUGCUCUUG 3’
           ::: : :::::::::::
query   3’ GCUAUAAAAGACGAGAAA 5’

query=JS16_t4089351, target=FGSG_07276, score=4, range=422-439, strand=-1

target  5’ CGAUAUUUCUUGCUCUUG 3’
           :::::::: .::::::::
query   3’ GCUAUAAAAGACGAGAAA 5’

query=JS16_t4089351, target=FGSG_13488, score=4, range=71-88, strand=-1

target  5’ UGGCAAUUUCUGCUCUUU 3’
           .:.  : :::::::::::
query   3’ GCUAUAAAAGACGAGAAA 5’

query=JS16_t4089351, target=FGSG_08107, score=4, range=181-198, strand=-1

target  5’ CGAUCUUAUCUGCUCUUC 3’
           :::: :: :::::::::
query   3’ GCUAUAAAAGACGAGAAA 5’

query=JS16_t4089351, target=FGSG_09749, score=4, range=208-224, strand=-1

target  5’ CGAAAUUUU-UGCUUUUU 3’
           ::: ::::: ::::.:::
query   3’ GCUAUAAAAGACGAGAAA 5’

query=JS16_t4089351, target=FGSG_02238, score=4, range=646-663, strand=-1

target  5’ CAACAUCUUCUGCUCUUU 3’
           : : :: :::::::::::
query   3’ GCUAUAAAAGACGAGAAA 5’

query=JS16_t4089351, target=FGSG_00506, score=4, range=945-961, strand=-1

target  5’ CAAU-UUUUCUUCUCUUU 3’
           : :: :::::: ::::::
query   3’ GCUAUAAAAGACGAGAAA 5’

No results for JS16_t4089370
No results for JS16_t2296533
query=JS16_t0584670, target=FGSG_03340, score=2.5, range=7630-7647, strand=1

target  5’ AUCAGUGACUUGACGAAC 3’
           :.: :::::::::::::
```

```
query=JS16_t3129514, target=FGSG_03407, score=3.5, range=2063-2080, strand=-1

target  5’ ACAGAUCACUUGGGCAUA 3’
             :.:::::::::::::
query   3’ ACUUUAGUGAACCCGUAG 5’

query=JS16_t3129514, target=FGSG_05815, score=3.5, range=758-775, strand=-1

target  5’ UCAAAUCACUUGUGCAUU 3’
           : :::::::::: ::::.
query   3’ ACUUUAGUGAACCCGUAG 5’

query=JS16_t3129514, target=FGSG_00125, score=4, range=579-595, strand=1

target  5’ UGAAG-CGCUUGGGCAUU 3’
           ::::. :.:::::::::.
query   3’ ACUUUAGUGAACCCGUAG 5’

query=JS16_t3129514, target=FGSG_13651, score=4, range=842-858, strand=1

target  5’ CGGAA-CACUUGGGCAUU 3’
            :.:: :::::::::::.
query   3’ ACUUUAGUGAACCCGUAG 5’

query=JS16_t3129514, target=FGSG_02590, score=4, range=900-918, strand=1

target  5’ CGAAAGCACUUGGGCUAUC 3’
            :::: ::::::::: :::
query   3’ ACUUUAGUGAACCCG-UAG 5’

query=JS16_t3129514, target=FGSG_13369, score=4, range=276-294, strand=1

target  5’ UGAGACCAUCUUGGGCAUU 3’
           :::.: :: :::::::::.
query   3’ ACUUUAGU-GAACCCGUAG 5’

query=JS16_t3129514, target=FGSG_04532, score=4, range=229-245, strand=1

target  5’ UGGAAUU-CUUGGGCAUU 3’
           ::.:::. :::::::::.
query   3’ ACUUUAGUGAACCCGUAG 5’

query=JS16_t3129514, target=FGSG_03050, score=4, range=1188-1204, strand=1

target  5’ CGAGGUCA-UUGGGCAUC 3’
            ::..::: :::::::::
query   3’ ACUUUAGUGAACCCGUAG 5’

query=JS16_t3129514, target=FGSG_08902, score=4, range=465-482, strand=-1

target  5’ UGAUAUUGCUUGGGUAUC 3’
           ::: ::..::::::.:::
query   3’ ACUUUAGUGAACCCGUAG 5’

query=JS16_t3129514, target=FGSG_11054, score=4, range=523-541, strand=-1

target  5’ UGCAAAUGGCUUGGGCAUC 3’
           :: :::: .::::::::::
query   3’ AC-UUUAGUGAACCCGUAG 5’

query=JS16_t3129514, target=FGSG_11623, score=4, range=853-871, strand=-1

target  5’ UGGGAACGACUUGGGCAUC 3’
           ::..: : ::::::::::
query   3’ ACUUUAG-UGAACCCGUAG 5’

query=JS16_t3129514, target=FGSG_02047, score=4, range=861-877, strand=-1

target  5’ UGAAAACA-UUGGGCAUC 3’
           ::::: :: :::::::::
query   3’ ACUUUAGUGAACCCGUAG 5’

No results for JS16_t2233308
No results for JS16_t4026060
query=JS16_t2233351, target=FGSG_09219, score=3.5, range=638-656, strand=1

target  5’ GCGCCGAAGCCAU-ACAGCG 3’
           :::::. :::::: ::::::
query   3’ CGCGGUAUCGGUAUUGUCGC 5’

No results for JS16_t3129583
No results for JS16_t1336916
query=JS16_t0468058, target=FGSG_07374, score=3, range=1239-1258, strand=-1

target  5’ UGCUCUUGAAGUGGUAACCC 3’
           .::.: :::::::.::::::
query   3’ GCGGGUACUUCACUAUUGGG 5’

query=JS16_t0468058, target=FGSG_13958, score=3.5, range=965-983, strand=1

target  5’ CUCCCG-GAAGUGGUAACCC 3’
           : :::. ::::::.::::::
query   3’ UGGACACUGAACUGCUUU 5’

query=JS16_t0584670, target=FGSG_02305, score=3, range=175-192, strand=1

target  5’ AUUUGUGACUUGAUGGAA 3’
           :..::::::::::.:.::
query   3’ UGGACACUGAACUGCUUU 5’

query=JS16_t0584670, target=FGSG_11185, score=3, range=1555-1572, strand=1

target  5’ CCCAGUGACUUGACGGAA 3’
            :: :::::::::::.::
query   3’ UGGACACUGAACUGCUUU 5’

query=JS16_t0584670, target=FGSG_05227, score=3, range=860-876, strand=1

target  5’ ACCU-UGGCUUGACGGAA 3’
           :::: ::.:::::::.::
query   3’ UGGACACUGAACUGCUUU 5’

query=JS16_t0584670, target=FGSG_02774, score=3, range=1136-1152, strand=1

target  5’ GUC-GUGACUUGGCGAAA 3’
           ..: ::::::::.:::::
query   3’ UGGACACUGAACUGCUUU 5’

query=JS16_t0584670, target=FGSG_05427, score=3, range=1096-1113, strand=-1

target  5’ ACCAGUGACUUGGCGAAU 3’
           ::: ::::::::.::::
query   3’ UGGACACUGAACUGCUUU 5’

query=JS16_t0584670, target=FGSG_13878, score=3, range=14707-14723, strand=-1

target  5’ UCCU-UGACUUGACGAAC 3’
            ::: ::::::::::::
query   3’ UGGACACUGAACUGCUUU 5’

query=JS16_t0584670, target=FGSG_13846, score=3.5, range=106-123, strand=1

target  5’ ACGUGUGACUUGAGGAAG 3’
           :: :::::::::: :::.
query   3’ UGGACACUGAACUGCUUU 5’

query=JS16_t0584670, target=FGSG_12012, score=3.5, range=769-787, strand=1

target  5’ ACCUUUGACUUGAUCGAAG 3’
           :::: :::::::: ::::.
query   3’ UGGACACUGAACU-GCUUU 5’

query=JS16_t0584670, target=FGSG_07633, score=3.5, range=574-591, strand=1

target  5’ GCCAAUGGCUUGACGAAA 3’
           .::  ::.::::::::::
query   3’ UGGACACUGAACUGCUUU 5’

query=JS16_t0584670, target=FGSG_00205, score=3.5, range=1177-1194, strand=-1

target  5’ AUCUGUGGCUUGACAAAA 3’
           :.:::::.:::::: :::
query   3’ UGGACACUGAACUGCUUU 5’

query=JS16_t0584670, target=FGSG_06183, score=3.5, range=744-762, strand=-1

target  5’ ACUUGUGAUUUGAGCGAAA 3’
           ::.:::::.:::: :::::
query   3’ UGGACACUGAACU-GCUUU 5’

query=JS16_t0584670, target=FGSG_12409, score=3.5, range=855-872, strand=-1

target  5’ GCGUGUGGCUUGACGAAC 3’
           .: ::::.:::::::::
query   3’ UGGACACUGAACUGCUUU 5’

query=JS16_t0584670, target=FGSG_10502, score=3.5, range=990-1006, strand=-1

target  5’ AUC-GUGACUUGACGGGA 3’
           :.: ::::::::::..:
query   3’ UGGACACUGAACUGCUUU 5’

query=JS16_t0584670, target=FGSG_05388, score=3.5, range=768-785, strand=-1

target  5’ GCCGGUGACUUGACGACA 3’
           .:: :::::::::::: :
query   3’ UGGACACUGAACUGCUUU 5’

query=JS16_t0584670, target=FGSG_02111, score=3.5, range=1225-1241, strand=-1

target  5’ ACUUUUGACUUGA-GAAA 3’
           ::.: :::::::: ::::
query   3’ UGGACACUGAACUGCUUU 5’
```

```
query   3’ GCGGGUACUUCACUAUUGGG 5’

query=JS16_t0468058, target=FGSG_01448, score=3.5, range=368-386, strand=1

target  5’ CGCCCAUGGAG-GAUAACCU 3’
           ::::::::.:: :::::::.
query   3’ GCGGGUACUUCACUAUUGGG 5’

query=JS16_t0468058, target=FGSG_10884, score=3.5, range=279-297, strand=-1

target  5’ CG-CCAUGAAGUGAUGGCCU 3’
           :: ::::::::::::..::.
query   3’ GCGGGUACUUCACUAUUGGG 5’

query=JS16_t0468058, target=FGSG_01978, score=4, range=201-220, strand=1

target  5’ UGGCCAUGAAGGGAUAACCU 3’
           .: :::::::: :::::::.
query   3’ GCGGGUACUUCACUAUUGGG 5’

query=JS16_t0468058, target=FGSG_04361, score=4, range=3135-3154, strand=-1

target  5’ CAUCGGCGAAGUGAUAACCC 3’
           : .: . :::::::::::::
query   3’ GCGGGUACUUCACUAUUGGG 5’

query=JS16_t1336957, target=FGSG_07353, score=4, range=900-922, strand=-1

target  5’ UUCGUGCUCGAUACCAGUUGUAG 3’
           ::: :.:.::::::::::: :::
query   3’ AAGAAUGGGCUAUGGUCAA-AUC 5’

No results for JS16_t2233454
No results for JS16_t4026226
query=JS16_t2233506, target=FGSG_07753, score=3.5, range=214-234, strand=1

target  5’ UCGCACGCAGGAUCACGAAU 3’
           . : :::::::.::::::::
query   3’ GUCCUGCGUCCUUAGUGCUUA 5’

query=JS16_t2233506, target=FGSG_13401, score=4, range=793-813, strand=1

target  5’ GAAGCCGCAGGAAUCAUGAAU 3’
            : : :::::::::::.::::
query   3’ GUCCUGCGUCCUUAGUGCUUA 5’

query=JS16_t2233518, target=FGSG_01874, score=2.5, range=905-923, strand=1

target  5’ AAAGUGAUGAUUUUCUUGG 3’
           ::::::::::.::::.::.
query   3’ UUUCACUACUGAAAGGACU 5’

query=JS16_t2233518, target=FGSG_03415, score=2.5, range=376-393, strand=-1

target  5’ AGAGU-AUGACUUUUCUGA 3’
           :.::: ::::::::.::::
query   3’ UUUCACUACUGAAAGGACU 5’

query=JS16_t2233518, target=FGSG_00039, score=3, range=699-716, strand=1

target  5’ AAACU-AUGACUUUCCUGC 3’
           ::: : :::::::::::::
query   3’ UUUCACUACUGAAAGGACU 5’

query=JS16_t2233518, target=FGSG_11696, score=3, range=1897-1914, strand=-1

target  5’ GAGGU-AUGACUUUCCUGU 3’
           .:.:: :::::::::::::
query   3’ UUUCACUACUGAAAGGACU 5’

query=JS16_t2233518, target=FGSG_14021, score=3.5, range=1238-1256, strand=1

target  5’ AGAGUGAUGACUUUCUUCA 3’
           :.:::::::::::::.: :
query   3’ UUUCACUACUGAAAGGACU 5’

query=JS16_t2233518, target=FGSG_01186, score=3.5, range=460-479, strand=1

target  5’ AGAGUGGUGACUUUCUCUGG 3’
           :.::::.::::::::: :::.
query   3’ UUUCACUACUGAAAG-GACU 5’

query=JS16_t2233518, target=FGSG_00528, score=3.5, range=406-424, strand=1

target  5’ CAUAUGAUGACUUUCCUGG 3’
            :  ::::::::::::::.
query   3’ UUUCACUACUGAAAGGACU 5’

query=JS16_t2233518, target=FGSG_05514, score=3.5, range=286-304, strand=-1

target  5’ GAACUGAUGACUUUUUUGA 3’
           .:: ::::::::::..:::
```

```
query=JS16_t0584670, target=FGSG_09440, score=3.5, range=3048-3064, strand=-1

target  5’ ACUUGU-ACUUGACGAGA 3’
           ::.::: :::::::::.:
query   3’ UGGACACUGAACUGCUUU 5’

query=JS16_t0584670, target=FGSG_10040, score=3.5, range=348-366, strand=-1

target  5’ AUCGGUGAGCUUGACGAAA 3’
           :.: :::: ::::::::::
query   3’ UGGACACU-GAACUGCUUU 5’

query=JS16_t0584670, target=FGSG_04817, score=4, range=1288-1305, strand=1

target  5’ ACUUUUGGCUUGGCGAAG 3’
           ::.: ::.::::.::::.
query   3’ UGGACACUGAACUGCUUU 5’

query=JS16_t0584670, target=FGSG_03421, score=4, range=1092-1110, strand=1

target  5’ CUUUGUUGACUUGACGGAA 3’
            ..:: ::::::::::.::
query   3’ UGGAC-ACUGAACUGCUUU 5’

query=JS16_t0584670, target=FGSG_12100, score=4, range=67-84, strand=1

target  5’ GAUUGUGACUUGACUAAA 3’
           . .:::::::::: :::
query   3’ UGGACACUGAACUGCUUU 5’

query=JS16_t0584670, target=FGSG_03411, score=4, range=177-195, strand=1

target  5’ ACUUCGUGACUUGAGGAAG 3’
           ::.: ::::::::: :::.
query   3’ UGGA-CACUGAACUGCUUU 5’

query=JS16_t0584670, target=FGSG_00931, score=4, range=231-247, strand=1

target  5’ ACAUGUGACUUGG-GAAA 3’
           :: :::::::::. ::::
query   3’ UGGACACUGAACUGCUUU 5’

query=JS16_t0584670, target=FGSG_12136, score=4, range=989-1005, strand=1

target  5’ ACAUG-GACUUGGCGAAA 3’
           :: :: ::::::.:::::
query   3’ UGGACACUGAACUGCUUU 5’

query=JS16_t0584670, target=FGSG_05079, score=4, range=929-945, strand=1

target  5’ CCCU-UGACUUUACGAAA 3’
            ::: :::::: ::::::
query   3’ UGGACACUGAACUGCUUU 5’

query=JS16_t0584670, target=FGSG_07978, score=4, range=487-505, strand=1

target  5’ ACCUGGGACUUUGACGAGA 3’
           ::::: ::: :::::::.:
query   3’ UGGACACUG-AACUGCUUU 5’

query=JS16_t0584670, target=FGSG_04948, score=4, range=3676-3693, strand=-1

target  5’ AUCAGGGACUUGACGAAG 3’
           :.: : :::::::::::.
query   3’ UGGACACUGAACUGCUUU 5’

query=JS16_t0584670, target=FGSG_12626, score=4, range=676-692, strand=-1

target  5’ UCUUGUGACUUGA-GAAG 3’
            :.:::::::::: :::.
query   3’ UGGACACUGAACUGCUUU 5’

query=JS16_t0584670, target=FGSG_00222, score=4, range=607-623, strand=-1

target  5’ ACCU-UGACUUUACGGAA 3’
           :::: :::::: :::.::
query   3’ UGGACACUGAACUGCUUU 5’

query=JS16_t0584670, target=FGSG_13883, score=4, range=1843-1860, strand=-1

target  5’ UUCACUGACUUGACGAAG 3’
            .:  ::::::::::::.
query   3’ UGGACACUGAACUGCUUU 5’

query=JS16_t0584670, target=FGSG_08148, score=4, range=1221-1238, strand=-1

target  5’ AGAGGUGACUUGACGAAU 3’
           :    :::::::::::::
query   3’ UGGACACUGAACUGCUUU 5’

query=JS16_t0584670, target=FGSG_07312, score=4, range=3078-3096, strand=-1
```

```
query   3’ UUUCACUACUGAAAGGACU 5’

query=JS16_t2233518, target=FGSG_10130, score=3.5, range=1417-1435, strand=-1

target  5’ CAGGUGAUGACUUUUCUGU 3’
            :.:::::::::::.:::
query   3’ UUUCACUACUGAAAGGACU 5’

query=JS16_t2233518, target=FGSG_01837, score=3.5, range=109-128, strand=-1

target  5’ AGAGUGAUGACUUUGCCUGU 3’
           :.::::::::::: ::::
query   3’ UUUCACUACUGAAA-GGACU 5’

query=JS16_t2233518, target=FGSG_05106, score=4, range=3083-3101, strand=1

target  5’ CAAGCGAUGACUUUGCUGA 3’
            ::: ::::::::: ::::
query   3’ UUUCACUACUGAAAGGACU 5’

query=JS16_t2233518, target=FGSG_06891, score=4, range=344-361, strand=1

target  5’ AAGAUGAUGAC-UUCCUGG 3’
           ::. ::::::: ::::::.
query   3’ UUUCACUACUGAAAGGACU 5’

query=JS16_t2233518, target=FGSG_05772, score=4, range=813-830, strand=1

target  5’ AAACU-UUGACUUUCCUGA 3’
           ::: :  :::::::::::
query   3’ UUUCACUACUGAAAGGACU 5’

query=JS16_t2233518, target=FGSG_13165, score=4, range=53-72, strand=-1

target  5’ CAAGUAGGUGACUUUCUUGA 3’
            :::: :.::::::::.:::
query   3’ UUUCA-CUACUGAAAGGACU 5’

query=JS16_t2233518, target=FGSG_12094, score=4, range=1020-1038, strand=-1

target  5’ AAAGUCACGACUUUCUUGA 3’
           ::::: : :::::::.:::
query   3’ UUUCACUACUGAAAGGACU 5’

No results for JS16_t1337140
query=JS16_t0795122, target=FGSG_10995, score=3, range=617-638, strand=-1

target  5’ AGCCAGA-AGUAAACACCAAGGU 3’
           : ::::: : :::::::::::::
query   3’ UAGGUCUAUGAUUUGUGGUUCCA 5’

query=JS16_t0795122, target=FGSG_07722, score=4, range=410-431, strand=1

target  5’ AUCUAGG-ACUAAACACCAAUGU 3’
           :::.::. ::::::::::: ::
query   3’ UAGGUCUAUGAUUUGUGGUUCCA 5’

query=JS16_t0254565, target=FGSG_11401, score=3, range=683-700, strand=-1

target  5’ GGA-UAGGUCUGGGAUCCU 3’
           ::: ::.::::::.:::::
query   3’ CCUGAUUCAGACCUUAGGA 5’

query=JS16_t0254565, target=FGSG_07726, score=3.5, range=109-128, strand=1

target  5’ GGAUCUGAGUCUGAAAUCCU 3’
           ::: ::.:::::: ::::::
query   3’ CCU-GAUUCAGACCUUAGGA 5’

query=JS16_t0254565, target=FGSG_01283, score=3.5, range=1061-1079, strand=-1

target  5’ GGAUCAAGUCUGGAAUCGU 3’
           :::. ::::::::::: :
query   3’ CCUGAUUCAGACCUUAGGA 5’

query=JS16_t0254565, target=FGSG_10945, score=4, range=859-878, strand=1

target  5’ GGACAAUGGUCUGGAAUCUU 3’
           :::: : .::::::::::.:
query   3’ CCUGAU-UCAGACCUUAGGA 5’

query=JS16_t0254565, target=FGSG_11114, score=4, range=299-316, strand=1

target  5’ GGAAAAAGUCUGGAA-CCU 3’
           :::  ::::::::: :::
query   3’ CCUGAUUCAGACCUUAGGA 5’

query=JS16_t0254565, target=FGSG_10221, score=4, range=357-374, strand=1

target  5’ GGACU-ACUCUGGAAUCCA 3’
           ::::: : ::::::::::
query   3’ CCUGAUUCAGACCUUAGGA 5’

target  5’ ACCCAUGACCUUGACGAAA 3’
           :::  ::: ::::::::::
query   3’ UGGACACU-GAACUGCUUU 5’

query=JS16_t0584670, target=FGSG_08038, score=4, range=1897-1913, strand=-1

target  5’ ACCU-UGACUUGACCAAC 3’
           :::: ::::::::: ::
query   3’ UGGACACUGAACUGCUUU 5’

No results for JS16_t0284152
query=JS16_t3192739, target=FGSG_06582, score=4, range=3948-3967, strand=-1

target  5’ UCGUUCG-GAUGUGUUGCGGA 3’
           ::::::: :::::::.:::.
query   3’ AGCAAGCACUACACAGCGCUC 5’

query=JS16_t3192739, target=FGSG_10144, score=4, range=7-26, strand=-1

target  5’ UCGAUCGUGAUG-GUCGCGGG 3’
           ::: :::::::: ::::::.:
query   3’ AGCAAGCACUACACAGCGCUC 5’

query=JS16_t0396453, target=FGSG_02547, score=3, range=584-604, strand=-1

target  5’ GGUCCUCUGUCAAGUUGACGU 3’
           ..:::::::::::::::: :
query   3’ UUAGGAGACAGUUCAACUGGA 5’

query=JS16_t0396453, target=FGSG_05530, score=4, range=38-58, strand=-1

target  5’ AAGCGUCGGUCAGGUUGACCU 3’
           :: : :: ::::.:::::::::
query   3’ UUAGGAGACAGUUCAACUGGA 5’

No results for JS16_t0475786
No results for JS16_t4089475
No results for JS16_t3192797
query=JS16_t0241340, target=FGSG_11242, score=4, range=1058-1077, strand=1

target  5’ GU-GUUGCAUUCGAGUUCCAU 3’
           :: ::::: .::::::::::
query   3’ CACCAACGCGAGCUCAAGGUA 5’

No results for JS16_t1400596
No results for JS16_t1400657
query=JS16_t0166714, target=FGSG_08230, score=4, range=593-614, strand=1

target  5’ UAGGUGCUGUCAUCGGAAGUCU 3’
            ::::: : :::.:::::::::
query   3’ UUCCAC-AAAGUGGCCUUCAGA 5’

No results for JS16_t0475809
query=JS16_t0182862, target=FgHV1gp2, score=2, range=10029-10048, strand=-1

target  5’ CAACAGGUUGGAAUCUGCGU 3’
           ::: ::::::.:::::::::
query   3’ GUUCUCCAACUUUAGACGCA 5’

query=JS16_t0182862, target=FGSG_08139, score=2.5, range=2427-2446, strand=1

target  5’ GAAGGGGUUGAAGUCUGCGU 3’
            :::.:::::::.:::::::
query   3’ GUUCUCCAACUUUAGACGCA 5’

query=JS16_t0182862, target=FGSG_12402, score=3, range=143-162, strand=-1

target  5’ AGGGAGGUUGAAAUCUGCGA 3’
            ..::::::::::::::::
query   3’ GUUCUCCAACUUUAGACGCA 5’

query=JS16_t0182862, target=FGSG_09241, score=4, range=4431-4450, strand=1

target  5’ AAAGAUCUUGGAAUCUGCGU 3’
            ::::  :::.:::::::::
query   3’ GUUCUCCAACUUUAGACGCA 5’

query=JS16_t0182862, target=FGSG_06052, score=4, range=908-927, strand=-1

target  5’ UACGGGGUUGAAGUCUGCGA 3’
           .: :.:::::::.::::::
query   3’ GUUCUCCAACUUUAGACGCA 5’

query=JS16_t0182862, target=FGSG_10861, score=4, range=647-665, strand=-1

target  5’ UGA-AGGUUGGGAUCUGCGU 3’
           ..: ::::::..::::::::
query   3’ GUUCUCCAACUUUAGACGCA 5’

query=JS16_t0182862, target=FGSG_00939, score=4, range=297-315, strand=-1

target  5’ CCAGAGGUUGAAAUCU-UGU 3’
```

```
query=JS16_t3129904, target=FGSG_05279, score=2, range=550-567, strand=1

target  5' GAAAGCGUUUGCAAGGAU 3'
           : :::::::.::::::::
query   3' CAUUCGCAAGCGUUCCUA 5'

query=JS16_t3129904, target=FGSG_00647, score=2.5, range=4113-4131, strand=1

target  5' GCAGGCCGUUCGCAAGGAU 3'
           : :.: :::::::::::::
query   3' CAUUC-GCAAGCGUUCCUA 5'

query=JS16_t3129904, target=FGSG_02460, score=3, range=1291-1308, strand=1

target  5' GAGGGUGUUCGCAAGGAU 3'
           : ..:.::::::::::::
query   3' CAUUCGCAAGCGUUCCUA 5'

query=JS16_t3129904, target=FGSG_03140, score=3.5, range=439-456, strand=-1

target  5' GUAGGCGUUCGCAAAGAC 3'
           :::.:::::::::: ::
query   3' CAUUCGCAAGCGUUCCUA 5'

query=JS16_t3129904, target=FGSG_11716, score=4, range=749-766, strand=1

target  5' AUAAGUUUUCGCAAGGAU 3'
            ::::. :::::::::::
query   3' CAUUCGCAAGCGUUCCUA 5'

query=JS16_t3129904, target=FGSG_01144, score=4, range=2458-2475, strand=1

target  5' CUGGCCGUUUGCAAGGAU 3'
            :.. ::::.::::::::
query   3' CAUUCGCAAGCGUUCCUA 5'

query=JS16_t3129904, target=FGSG_07579, score=4, range=400-418, strand=1

target  5' GUCAAGCGAUUGCAAGGAU 3'
           :: ::::: :.::::::::
query   3' CA-UUCGCAAGCGUUCCUA 5'

query=JS16_t3129904, target=FGSG_11792, score=4, range=958-975, strand=1

target  5' GUUCGUGUUCGCAAGGAC 3'
           ::  :.::::::::::::
query   3' CAUUCGCAAGCGUUCCUA 5'

query=JS16_t3129904, target=FGSG_05321, score=4, range=2720-2736, strand=1

target  5' CCAAGC-UUCGCAAGGAU 3'
             :::: :::::::::::
query   3' CAUUCGCAAGCGUUCCUA 5'

query=JS16_t3129904, target=FGSG_02419, score=4, range=803-819, strand=1

target  5' AAAAG-GUUCGCAAGGAU 3'
             ::: ::::::::::::
query   3' CAUUCGCAAGCGUUCCUA 5'

query=JS16_t3129904, target=FGSG_05599, score=4, range=2626-2643, strand=-1

target  5' GUACUUGUUCGCGAGGAU 3'
           :::  .::::::.:::::
query   3' CAUUCGCAAGCGUUCCUA 5'

query=JS16_t3129904, target=FGSG_09931, score=4, range=92-108, strand=-1

target  5' GUAA-CGUUCGUAGGGAC 3'
           :::: ::::::.:.:::
query   3' CAUUCGCAAGCGUUCCUA 5'

query=JS16_t3129904, target=FGSG_04849, score=4, range=394-410, strand=-1

target  5' CUGGG-GUUCGCAAGGAU 3'
            :..: ::::::::::::
query   3' CAUUCGCAAGCGUUCCUA 5'

query=JS16_t3129904, target=FGSG_03799, score=4, range=1257-1274, strand=-1

target  5' GCUAGCGUUCGCGAGGAA 3'
           :  :::::::::.::::
query   3' CAUUCGCAAGCGUUCCUA 5'

query=JS16_t3129904, target=FGSG_00975, score=4, range=2033-2050, strand=-1

target  5' GAAAGCGAUCGCAAGGAA 3'
           : ::::: :::::::::
query   3' CAUUCGCAAGCGUUCCUA 5'

query=JS16_t3129904, target=FGSG_02933, score=4, range=2094-2110, strand=-1

           : ::::::::::::: .::
query   3' GUUCUCCAACUUUAGACGCA 5'

No results for JS16_t1400688
query=JS16_t3192982, target=FGSG_13904, score=4, range=324-344, strand=-1

target  5' UCUGCACCUUGGCUCGCUGGU 3'
           ::: :::::::::: .:::::
query   3' AGAAGUGGAACCGAAUGACCA 5'

No results for JS16_t3192993
No results for JS16_t2296847
query=JS16_t3193042, target=FGSG_08720, score=3.5, range=2673-2692, strand=-1

target  5' UACUGUCAUCCGAGACUUGC 3'
           ::::.: :: ::::::::::
query   3' AUGAUAAUACGCUCUGAACG 5'

query=JS16_t3193042, target=FGSG_11186, score=4, range=1301-1320, strand=1

target  5' AUCUAUUAUGCGAGUCUUGC 3'
             :::::::::::: :::::
query   3' AUGAUAAUACGCUCUGAACG 5'

No results for JS16_t4089771
No results for JS16_t4089781
No results for JS16_t0475825
No results for JS16_t4089821
No results for JS16_t1400825
query=JS16_t2296930, target=FGSG_07545, score=4, range=1074-1097, strand=-1

target  5' UUUGUUUCAUUGGCCUCGCUUUCU 3'
           .:::.::: ::::::::.:::::
query   3' GAACGAAGAAACCGGAGUGAAAGU 5'

No results for JS16_t3193141
No results for JS16_t3193186
query=JS16_t0220394, target=FGSG_05690, score=2, range=738-757, strand=1

target  5' UGGCGCCGUGGAUCCUGUGA 3'
           :: ::: :::::::::::::
query   3' ACAGCGACACCUAGGACACU 5'

query=JS16_t0220394, target=FGSG_00139, score=3, range=725-744, strand=-1

target  5' UGUGGCUGUGGACCCUGUGA 3'
           ::: :::::::: :::::::
query   3' ACAGCGACACCUAGGACACU 5'

query=JS16_t0220394, target=FGSG_06940, score=4, range=1852-1872, strand=-1

target  5' CGACGUCUGUGGAUCUUGUGA 3'
            : :: :::::::::.:::::
query   3' ACAGC-GACACCUAGGACACU 5'

query=JS16_t3193201, target=FGSG_07684, score=3, range=525-542, strand=1

target  5' CUGCACCUGUGUGAGAGG 3'
           : :::::::.:::::::
query   3' GUCGUGGACGCACUCUCA 5'

query=JS16_t3193201, target=FGSG_01172, score=3, range=69-86, strand=1

target  5' CAAUGCCUGCGUGAGAGG 3'
           :: ..::::::::::::
query   3' GUCGUGGACGCACUCUCA 5'

query=JS16_t3193201, target=FGSG_07169, score=3, range=370-387, strand=-1

target  5' GAGCUCCUGCGUGAGGGU 3'
            ::: ::::::::::.::
query   3' GUCGUGGACGCACUCUCA 5'

query=JS16_t3193201, target=FGSG_05487, score=3, range=1610-1627, strand=-1

target  5' CAGCCCCUGCGUCAGAGU 3'
           :::: ::::::: :::::
query   3' GUCGUGGACGCACUCUCA 5'

query=JS16_t3193201, target=FGSG_06274, score=3.5, range=850-866, strand=1

target  5' CCGUACCUGCGUGA-AGU 3'
           : :.:::::::::: :::
query   3' GUCGUGGACGCACUCUCA 5'

query=JS16_t3193201, target=FGSG_02916, score=3.5, range=833-849, strand=1

target  5' CA-UACAUGCGUGAGAGU 3'
           :: .:: :::::::::::
query   3' GUCGUGGACGCACUCUCA 5'

query=JS16_t3193201, target=FGSG_10861, score=3.5, range=764-780, strand=1
```

```
target  5’ GUAA-CGUUCCCAAGGGU 3’
           :::: ::::: :::::.:
query   3’ CAUUCGCAAGCGUUCCUA 5’

query=JS16_t3129904, target=FGSG_03912, score=4, range=516-532, strand=-1

target  5’ GU-AGCGUCUGCAAGGAU 3’
           :: ::::: .::::::::
query   3’ CAUUCGCAAGCGUUCCUA 5’

query=JS16_t3129954, target=FGSG_01799, score=3, range=5228-5247, strand=1

target  5’ UCGACUCGGGCAAUGCUUUC 3’
           ::::: :..::::::::::
query   3’ AGCUG-GUUCGUUACGAAAG 5’

query=JS16_t3129954, target=FGSG_06299, score=3, range=1189-1207, strand=1

target  5’ UUUAUCAAGCAAUGCUUUG 3’
           :.  :.:::::::::::::
query   3’ AGCUGGUUCGUUACGAAAG 5’

query=JS16_t3129954, target=FGSG_11266, score=3.5, range=1764-1782, strand=1

target  5’ UUGGCCACGCAAUGCUUUU 3’
           :.:.::: ::::::::::.
query   3’ AGCUGGUUCGUUACGAAAG 5’

query=JS16_t3129954, target=FGSG_09233, score=3.5, range=1601-1620, strand=1

target  5’ CUAACCAAAGCAAUGCUUUC 3’
            . ::: :::::::::::::
query   3’ AGCUGG-UUCGUUACGAAAG 5’

query=JS16_t3129954, target=FGSG_11536, score=3.5, range=1045-1062, strand=1

target  5’ UUUA-CAAGCAAUGCUUUA 3’
           :.  : :::::::::::::
query   3’ AGCUGGUUCGUUACGAAAG 5’

query=JS16_t3129954, target=FGSG_04681, score=3.5, range=151-169, strand=-1

target  5’ UUGACCAGGUGAUGCUUUC 3’
           :.:::::.:..::::::::
query   3’ AGCUGGUUCGUUACGAAAG 5’

query=JS16_t3129954, target=FGSG_11733, score=3.5, range=579-596, strand=-1

target  5’ UCGAUCAAGC-AUGCUUUG 3’
           ::::.::::: :::::::
query   3’ AGCUGGUUCGUUACGAAAG 5’

query=JS16_t3129954, target=FGSG_03387, score=3.5, range=599-618, strand=-1

target  5’ UUGAGCAAGCCAAUGCUUUC 3’
           :.:: :::: ::::::::::
query   3’ AGCUGGUUC-GUUACGAAAG 5’

query=JS16_t3129954, target=FGSG_09277, score=4, range=69-87, strand=1

target  5’ GGGAACAAGCAGUGCUUUC 3’
             :: ::::::.:::::::
query   3’ AGCUGGUUCGUUACGAAAG 5’

query=JS16_t3129954, target=FGSG_04828, score=4, range=70-87, strand=1

target  5’ GUGA-CGAGCAAUGCUUUU 3’
            .:: :.:::::::::::.
query   3’ AGCUGGUUCGUUACGAAAG 5’

query=JS16_t3129954, target=FGSG_13628, score=4, range=60-78, strand=1

target  5’ UCGUCAAAGCAAGGCUUUC 3’
           ::: : :::::: ::::::
query   3’ AGCUGGUUCGUUACGAAAG 5’

query=JS16_t3129954, target=FGSG_00560, score=4, range=442-461, strand=1

target  5’ UCGGAGCAAGAAAUGCUUUC 3’
           :: :: :::: :::::::::
query   3’ AG-CUGGUUCGUUACGAAAG 5’

query=JS16_t3129954, target=FGSG_04292, score=4, range=401-420, strand=1

target  5’ UCGCCCAAGCCAAUGCUUUA 3’
           ::: ::::: :::::::::
query   3’ AGCUGGUUC-GUUACGAAAG 5’

query=JS16_t3129954, target=FGSG_00464, score=4, range=232-250, strand=-1

target  5’ UCGACCAAGCACUGGUUUC 3’
```

```
target  5’ CGGCACCUG-GUGAGAGC 3’
           :.:::::: :::::::
query   3’ GUCGUGGACGCACUCUCA 5’

query=JS16_t3193201, target=FGSG_04963, score=3.5, range=1537-1554, strand=-1

target  5’ AAGCGCUUGUGUGAGAGU 3’
            :::.:.::.::::::::
query   3’ GUCGUGGACGCACUCUCA 5’

query=JS16_t3193201, target=FGSG_01156, score=3.5, range=1370-1386, strand=-1

target  5’ CAG-GCCUGUGUGAGGGU 3’
           ::: .::::.:::::.::
query   3’ GUCGUGGACGCACUCUCA 5’

query=JS16_t3193201, target=FGSG_00153, score=3.5, range=1704-1722, strand=-1

target  5’ CUGUACCUGCGUGACGAGU 3’
           : :.:::::::::: ::::
query   3’ GUCGUGGACGCACU-CUCA 5’

query=JS16_t3193201, target=FGSG_12276, score=4, range=6500-6517, strand=1

target  5’ CUGCACCUGUGUGGGAGC 3’
           : :::::::.:::.:::
query   3’ GUCGUGGACGCACUCUCA 5’

query=JS16_t3193201, target=FGSG_00308, score=4, range=873-891, strand=1

target  5’ UCGCAUCGUGCGUGAGAGU 3’
           . :::.: :::::::::::
query   3’ GUCGUGG-ACGCACUCUCA 5’

query=JS16_t3193201, target=FGSG_11519, score=4, range=1382-1398, strand=1

target  5’ UAGU-CCUGCGUGAGAAU 3’
           .::. ::::::::::: :
query   3’ GUCGUGGACGCACUCUCA 5’

query=JS16_t3193201, target=FGSG_04769, score=4, range=337-355, strand=1

target  5’ CAGAAGCUGCGUGAGAAGU 3’
           ::: : ::::::::: :::
query   3’ GUCGUGGACGCACUC-UCA 5’

query=JS16_t3193201, target=FGSG_02130, score=4, range=3391-3407, strand=1

target  5’ CAG-ACCUGCGCGAGAGG 3’
           ::: ::::::: :::::
query   3’ GUCGUGGACGCACUCUCA 5’

query=JS16_t3193201, target=FGSG_00952, score=4, range=816-833, strand=-1

target  5’ CAGCCCUUGCGUGGGAGC 3’
           :::: :.::::::.:::
query   3’ GUCGUGGACGCACUCUCA 5’

query=JS16_t3193201, target=FGSG_06990, score=4, range=56-73, strand=-1

target  5’ GAGGAUCUGUGUGAGAGU 3’
            :: :.:::.::::::::
query   3’ GUCGUGGACGCACUCUCA 5’

query=JS16_t3193201, target=FGSG_03675, score=4, range=901-917, strand=-1

target  5’ CAG-AUUUGCGUGAGAGC 3’
           ::: :..::::::::::
query   3’ GUCGUGGACGCACUCUCA 5’

query=JS16_t3193201, target=FGSG_06122, score=4, range=1075-1093, strand=-1

target  5’ CAGCAGGCUGCGUGAGAGG 3’
           :::::  :::::::::::
query   3’ GUCGU-GGACGCACUCUCA 5’

query=JS16_t3193201, target=FGSG_09163, score=4, range=108-126, strand=-1

target  5’ CAGGACCUGCGUGAUGAGG 3’
           ::: :::::::::: :::
query   3’ GUCGUGGACGCACU-CUCA 5’

query=JS16_t3193201, target=FGSG_04794, score=4, range=31-47, strand=-1

target  5’ CGGU-CCUGCGUGAUAGU 3’
           :.:. ::::::::: :::
query   3’ GUCGUGGACGCACUCUCA 5’

query=JS16_t3193201, target=FGSG_04501, score=4, range=377-394, strand=-1

target  5’ CACCCCCUGCGAGAGAGU 3’
```

```
           ::::::::::: :: ::::
query   3' AGCUGGUUCGUUACGAAAG 5'

query=JS16_t3129954, target=FGSG_13189, score=4, range=1000-1019, strand=-1

target  5' UUGGUCCAAGCAAUGGUUUC 3'
           :.:. :::::::::: ::::
query   3' AGCU-GGUUCGUUACGAAAG 5'

No results for JS16_t4026524
query=JS16_t0577136, target=FGSG_01986, score=4, range=1230-1250, strand=-1

target  5' UUGUUGGCCAACCAUGAGAUA 3'
           : ::::  ::::::::::::
query   3' ACCAACGCGUUGGUACUCUAC 5'

query=JS16_t0577136, target=FGSG_05865, score=4, range=934-954, strand=-1

target  5' UGGAAGCACAACCAUGAGAUA 3'
           :::  :: ::::::::::::
query   3' ACCAACGCGUUGGUACUCUAC 5'

query=JS16_t0577136, target=FGSG_03708, score=4, range=1279-1300, strand=-1

target  5' UGGUUCCGUUAACCAUGAGAUC 3'
           ::::: :: .:::::::::::
query   3' ACCAACGC-GUUGGUACUCUAC 5'

No results for JS16_t3130006
No results for JS16_t0305746
No results for JS16_t1337394
No results for JS16_t4026648
No results for JS16_t1337597
query=JS16_t3130265, target=FGSG_05194, score=2, range=348-365, strand=-1

target  5' AAGAACAUGCCGAAGCUG 3'
           ::::::::.:::::::.:
query   3' UUCUUGUAUGGCUUCGGC 5'

query=JS16_t3130265, target=FGSG_07169, score=2.5, range=2676-2693, strand=-1

target  5' AAUGACAUGCCGAAGCCG 3'
           :: .::::.:::::::::
query   3' UUCUUGUAUGGCUUCGGC 5'

query=JS16_t3130265, target=FGSG_03725, score=2.5, range=1248-1266, strand=-1

target  5' GAGAACCAUGCCGAAGCCG 3'
           .:::: :::.:::::::::
query   3' UUCUU-GUAUGGCUUCGGC 5'

query=JS16_t3130265, target=FGSG_06106, score=3, range=611-627, strand=-1

target  5' AAGAACAU-CCGAAGUCG 3'
           :::::::: ::::::.::
query   3' UUCUUGUAUGGCUUCGGC 5'

query=JS16_t3130265, target=FGSG_12904, score=3.5, range=2156-2173, strand=1

target  5' AGGAAGAUACCGAAGCUG 3'
           :.::: ::::::::::.:
query   3' UUCUUGUAUGGCUUCGGC 5'

query=JS16_t3130265, target=FGSG_04320, score=3.5, range=80-97, strand=1

target  5' AGGAACCUACCGAGGCCG 3'
           :.:::: ::::::.::::
query   3' UUCUUGUAUGGCUUCGGC 5'

query=JS16_t3130265, target=FGSG_10927, score=3.5, range=314-331, strand=1

target  5' GUGGGCAUACCGAAGUCG 3'
           .  :..::::::::::.::
query   3' UUCUUGUAUGGCUUCGGC 5'

query=JS16_t3130265, target=FGSG_03815, score=3.5, range=42-59, strand=1

target  5' AUGGACAUACCCAAGCCG 3'
           : :.::::::: ::::::
query   3' UUCUUGUAUGGCUUCGGC 5'

query=JS16_t3130265, target=FGSG_03646, score=3.5, range=67-85, strand=1

target  5' GAGAACAGUACCGAAGCCA 3'
           .:::::: ::::::::::
query   3' UUCUUGU-AUGGCUUCGGC 5'

query=JS16_t3130265, target=FGSG_08177, score=3.5, range=681-698, strand=-1

target  5' AACAGCAUACCGAGGCCA 3'
           :: :.:::::::::.:::
query   3' UUCUUGUAUGGCUUCGGC 5'
```

```
           :: : :::::: ::::::
query   3' GUCGUGGACGCACUCUCA 5'

query=JS16_t3193201, target=FGSG_06680, score=4, range=2117-2133, strand=-1

target  5' GAGC-CCUGCGUGAGAUU 3'
            ::: ::::::::::: :
query   3' GUCGUGGACGCACUCUCA 5'

No results for JS16_t3193208
No results for JS16_t3193209
query=JS16_t2297048, target=FGSG_06363, score=2.5, range=56-75, strand=-1

target  5' GGAAAGGUUAUCUUCGGGAG 3'
           :.:::.::::::.::::::.
query   3' CUUUUUCAAUAGGAGCCCUU 5'

query=JS16_t2297048, target=FGSG_00262, score=4, range=43-62, strand=-1

target  5' GAGAGGGUUAUGCUCGGGAG 3'
           ::.:..::::: :::::::.
query   3' CUUUUUCAAUAGGAGCCCUU 5'

query=JS16_t2297048, target=FGSG_04442, score=4, range=1067-1085, strand=-1

target  5' GAACGGGUU-UCCUCGGGAA 3'
           ::: ..::: ::::::::::
query   3' CUUUUUCAAUAGGAGCCCUU 5'

query=JS16_t1400966, target=FGSG_00797, score=3.5, range=1806-1824, strand=-1

target  5' AGGGUAUCCCUU-CUUCUUG 3'
           ::::.:::::::: ::::::
query   3' UCCCGUAGGGAACGAAGAAA 5'

query=JS16_t1400966, target=FGSG_02753, score=3.5, range=783-801, strand=-1

target  5' AGUGUAUCCCUUGC-UCUUU 3'
           :: :.::::::::: :::::
query   3' UCCCGUAGGGAACGAAGAAA 5'

query=JS16_t1400966, target=FGSG_13562, score=4, range=1412-1431, strand=1

target  5' UGUGCAUGCCUUGCUUCUUU 3'
            : :::: ::::::::::::
query   3' UCCCGUAGGGAACGAAGAAA 5'

query=JS16_t1400966, target=FGSG_11803, score=4, range=97-116, strand=1

target  5' CAAGCAGCCCUUGCUUCUUU 3'
              ::: :::::::::::::
query   3' UCCCGUAGGGAACGAAGAAA 5'

query=JS16_t1400966, target=FGSG_04204, score=4, range=760-779, strand=-1

target  5' AGGGCAACCUUUCCUUCUUU 3'
           :::::: ::.:: :::::::
query   3' UCCCGUAGGGAACGAAGAAA 5'

query=JS16_t1400966, target=FGSG_00990, score=4, range=937-956, strand=-1

target  5' ACUUCAUCUCUUGCUUCUUU 3'
           :   ::::.:::::::::::
query   3' UCCCGUAGGGAACGAAGAAA 5'

query=JS16_t1400966, target=FGSG_09439, score=4, range=257-275, strand=-1

target  5' AGGUCAU-UCUUGCUUCUUU 3'
           ::: ::: .:::::::::::
query   3' UCCCGUAGGGAACGAAGAAA 5'

query=JS16_t1400966, target=FGSG_12952, score=4, range=267-285, strand=-1

target  5' GGUGUAUCCCUUG-UUCUUU 3'
           .: :.:::::::: ::::::
query   3' UCCCGUAGGGAACGAAGAAA 5'

No results for JS16_t2297066
No results for JS16_t1400996
No results for JS16_t1401036
No results for JS16_t2297115
No results for JS16_t2297162
query=JS16_t0255581, target=FGSG_10394, score=2.5, range=939-958, strand=1

target  5' CAAA-GCUGCUGGAAGUGUUA 3'
           :::: .::::::::::::::
query   3' GUUUAUGACGACCUUCACAAG 5'

query=JS16_t0255581, target=FGSG_13543, score=4, range=3527-3547, strand=1

target  5' CAGAUCUUGCUGGGAGUGUUG 3'
           ::.:: .::::::.::::::
```

```
query=JS16_t3130265, target=FGSG_11546, score=3.5, range=489-505, strand=-1

target  5' GAG-ACAUGCCGGAGCCG 3'
           .:: ::::.:::.:::::
query   3' UUCUUGUAUGGCUUCGGC 5'

query=JS16_t3130265, target=FGSG_13150, score=4, range=281-298, strand=1

target  5' AAGAACAUGUUGAAGCUG 3'
           ::::::::...:::::.:
query   3' UUCUUGUAUGGCUUCGGC 5'

query=JS16_t3130265, target=FGSG_09772, score=4, range=845-862, strand=1

target  5' AAGAAUAUAUCGAAGUCA 3'
           :::::.:::.:::::.:
query   3' UUCUUGUAUGGCUUCGGC 5'

query=JS16_t3130265, target=FGSG_09753, score=4, range=98-115, strand=1

target  5' CAGAACGUGCCGAAGCUG 3'
            :::::.:.:::::::.:
query   3' UUCUUGUAUGGCUUCGGC 5'

query=JS16_t3130265, target=FGSG_11412, score=4, range=95-112, strand=1

target  5' AAGAACAUAGCGAAACCG 3'
           ::::::::: :::: :::
query   3' UUCUUGUAUGGCUUCGGC 5'

query=JS16_t3130265, target=FGSG_00890, score=4, range=746-763, strand=1

target  5' AUGAACAAACCGAGGCCG 3'
           : ::::: :::::.::::
query   3' UUCUUGUAUGGCUUCGGC 5'

query=JS16_t3130265, target=FGSG_04270, score=4, range=67-85, strand=1

target  5' AAGAACCAUGCCAAAGCCG 3'
           ::::: :::.:: ::::::
query   3' UUCUU-GUAUGGCUUCGGC 5'

query=JS16_t3130265, target=FGSG_12573, score=4, range=112-130, strand=1

target  5' AAGAAACUUGCCGAAGCCG 3'
           ::: ::: :.:::::::::
query   3' UUC-UUGUAUGGCUUCGGC 5'

query=JS16_t3130265, target=FGSG_05736, score=4, range=1613-1630, strand=1

target  5' AUGCACAUGCCGAGGCCG 3'
           : : ::::.::::.::::
query   3' UUCUUGUAUGGCUUCGGC 5'

query=JS16_t3130265, target=FGSG_00843, score=4, range=296-313, strand=1

target  5' AAUCACAUACCGAAGUCA 3'
           ::  :::::::::::.:
query   3' UUCUUGUAUGGCUUCGGC 5'

query=JS16_t3130265, target=FGSG_10701, score=4, range=449-466, strand=1

target  5' ACGUCUAUACCGAAGCCG 3'
           : :  .::::::::::::
query   3' UUCUUGUAUGGCUUCGGC 5'

query=JS16_t3130265, target=FGSG_00118, score=4, range=181-197, strand=1

target  5' AAG-ACAUACCGAACCCU 3'
           ::: :::::::::: ::
query   3' UUCUUGUAUGGCUUCGGC 5'

query=JS16_t3130265, target=FGSG_09481, score=4, range=490-506, strand=1

target  5' AUGAAUAUACC-AAGCCG 3'
           : :::.::::: ::::::
query   3' UUCUUGUAUGGCUUCGGC 5'

query=JS16_t3130265, target=FGSG_11333, score=4, range=153-169, strand=1

target  5' AACAAC-UACUGAAGCCG 3'
           :: ::: :::.:::::::
query   3' UUCUUGUAUGGCUUCGGC 5'

query=JS16_t3130265, target=FGSG_09812, score=4, range=421-439, strand=1

target  5' ACGACCAUGACCGAAGCCG 3'
           : :: ::: ::::::::::
query   3' UUCUUGUA-UGGCUUCGGC 5'

query=JS16_t3130265, target=FGSG_01964, score=4, range=2145-2162, strand=-1
query   3' GUUUAUGACGACCUUCACAAG 5'

No results for JS16_t4090144
No results for JS16_t2297218
No results for JS16_t1401197
No results for JS16_t2297355
No results for JS16_t2297380
No results for JS16_t4090316
No results for JS16_t3193563
query=JS16_t4090342, target=FGSG_08786, score=3, range=6110-6127, strand=-1

target  5' UGGCGAUGGCUUGUUCCG 3'
           :::: :::::::::: ::
query   3' ACCGAUACCGAACAAAGC 5'

query=JS16_t4090342, target=FGSG_08664, score=3, range=269-286, strand=-1

target  5' UGCAUAUGGCUUGUUUCA 3'
           ::  :::::::::::::
query   3' ACCGAUACCGAACAAAGC 5'

query=JS16_t4090342, target=FGSG_02697, score=3.5, range=437-454, strand=1

target  5' UGGUCAUGGCUGGUUUCG 3'
           :::. :::::: ::::::
query   3' ACCGAUACCGAACAAAGC 5'

query=JS16_t4090342, target=FGSG_01231, score=3.5, range=443-460, strand=-1

target  5' UGAUCAUGGCUUGUUUCU 3'
           :: . ::::::::::::
query   3' ACCGAUACCGAACAAAGC 5'

query=JS16_t4090342, target=FGSG_09994, score=4, range=1416-1433, strand=1

target  5' UGGCAAUGGUUUGUUUGG 3'
           :::: ::::.:::::: :
query   3' ACCGAUACCGAACAAAGC 5'

query=JS16_t4090342, target=FGSG_01995, score=4, range=1054-1071, strand=1

target  5' GGGCUAUGGCUUAUUUCC 3'
            ::::::::::: ::::
query   3' ACCGAUACCGAACAAAGC 5'

query=JS16_t4090342, target=FGSG_09198, score=4, range=1197-1214, strand=1

target  5' GGGCUAUGGGUUGUUUCC 3'
            :::::::: :::::::
query   3' ACCGAUACCGAACAAAGC 5'

query=JS16_t4090342, target=FGSG_09025, score=4, range=1071-1087, strand=1

target  5' UCGC-AUGGCUUUUUUCG 3'
           : :: ::::::: :::::
query   3' ACCGAUACCGAACAAAGC 5'

query=JS16_t4090342, target=FGSG_06770, score=4, range=834-850, strand=1

target  5' UGGCUAUGGC-AGUUUCG 3'
           ::::::::::  ::::::
query   3' ACCGAUACCGAACAAAGC 5'

query=JS16_t4090342, target=FGSG_02308, score=4, range=644-661, strand=-1

target  5' UGGCCAUGGCUUGUUCUG 3'
           :::: :::::::::: .:
query   3' ACCGAUACCGAACAAAGC 5'

query=JS16_t4090342, target=FGSG_07136, score=4, range=611-628, strand=-1

target  5' UUGCUUUGGCUUGUUUUG 3'
           : ::: ::::::::::.:
query   3' ACCGAUACCGAACAAAGC 5'

query=JS16_t4090342, target=FGSG_11083, score=4, range=104-121, strand=-1

target  5' UGUCUUUGGCUUGUUUUG 3'
           :: :: ::::::::::.:
query   3' ACCGAUACCGAACAAAGC 5'

query=JS16_t4090342, target=FGSG_03152, score=4, range=614-631, strand=-1

target  5' UGGCCAUGGUUUGUUGCG 3'
           :::: ::::.::::: ::
query   3' ACCGAUACCGAACAAAGC 5'

query=JS16_t4090342, target=FGSG_10904, score=4, range=117-135, strand=-1

target  5' UGGCUGAUGGGUUGUUUUG 3'
           ::::: :::: ::::::.:
query   3' ACCGA-UACCGAACAAAGC 5'
```

```
target  5’ AAGAACAGACUGAAGUCG 3’
           ::::::: ::.::::.::
query   3’ UUCUUGUAUGGCUUCGGC 5’

query=JS16_t3130265, target=FGSG_01429, score=4, range=2298-2315, strand=-1

target  5’ AAGAACAUGCCAAAGCUG 3’
           ::::::::.:: ::::.:
query   3’ UUCUUGUAUGGCUUCGGC 5’

query=JS16_t3130265, target=FGSG_10194, score=4, range=969-986, strand=-1

target  5’ AAGAACAUGCCGCAGUCG 3’
           ::::::::.::: ::.::
query   3’ UUCUUGUAUGGCUUCGGC 5’

query=JS16_t3130265, target=FGSG_11507, score=4, range=1365-1383, strand=-1

target  5’ GGGAACAUACUGGAAGCCG 3’
           ..::::::::. :::::::
query   3’ UUCUUGUAUGG-CUUCGGC 5’

query=JS16_t3130265, target=FGSG_10611, score=4, range=80-97, strand=-1

target  5’ GACGACAUGCCGAGGCCG 3’
           .: .::::.::::.::::
query   3’ UUCUUGUAUGGCUUCGGC 5’

query=JS16_t3130265, target=FGSG_09492, score=4, range=1158-1175, strand=-1

target  5’ UGGGACAUACCGAUGCCG 3’
            .:.::::::::: ::::
query   3’ UUCUUGUAUGGCUUCGGC 5’

query=JS16_t3130265, target=FGSG_04364, score=4, range=114-130, strand=-1

target  5’ GAGGA-AUACCGAAGCUG 3’
           .::.: ::::::::::.:
query   3’ UUCUUGUAUGGCUUCGGC 5’

query=JS16_t3130265, target=FGSG_09329, score=4, range=66-82, strand=-1

target  5’ UAGAACAUGCCG-AGCCG 3’
            :::::::.::: :::::
query   3’ UUCUUGUAUGGCUUCGGC 5’

query=JS16_t3130265, target=FGSG_01210, score=4, range=2478-2494, strand=-1

target  5’ GAUGA-AUACCGAAGCCG 3’
           .: .: ::::::::::::
query   3’ UUCUUGUAUGGCUUCGGC 5’

query=JS16_t3130265, target=FGSG_00900, score=4, range=108-126, strand=-1

target  5’ AAGGGCAUGACCGAAGCUG 3’
           :::..::: ::::::::.:
query   3’ UUCUUGUA-UGGCUUCGGC 5’

query=JS16_t4026810, target=FGSG_02963, score=3, range=621-639, strand=1

target  5’ GGACAAGGGCUGGAAGCAA 3’
            ::::::::::::: ::::
query   3’ ACUGUUCCCGACCUACGUU 5’

query=JS16_t4026810, target=FGSG_03124, score=3, range=1402-1419, strand=1

target  5’ UGG-AAGGGUUGGAUGCAG 3’
           ::.  :::::.::::::::.
query   3’ ACUGUUCCCGACCUACGUU 5’

query=JS16_t4026810, target=FGSG_01713, score=3.5, range=338-357, strand=1

target  5’ UGGCUAAGGGUUGGAUGCAC 3’
           ::.: :::::.::::::::
query   3’ ACUG-UUCCCGACCUACGUU 5’

query=JS16_t4026810, target=FGSG_10575, score=3.5, range=1516-1533, strand=1

target  5’ UGG-AAGGGGUGGAUGCAA 3’
           ::.  ::::: :::::::::
query   3’ ACUGUUCCCGACCUACGUU 5’

query=JS16_t4026810, target=FGSG_01589, score=3.5, range=2632-2650, strand=-1

target  5’ UGGCAAGGGCCGGAUGCGA 3’
           ::.:::::::: ::::::.:
query   3’ ACUGUUCCCGACCUACGUU 5’

query=JS16_t4026810, target=FGSG_09638, score=3.5, range=2847-2866, strand=-1

target  5’ UGACGAAGGGCUGGAUGCCG 3’

query=JS16_t4090342, target=FGSG_08771, score=4, range=14-30, strand=-1

target  5’ UGGCUGUGGC-UGUUUCU 3’
           :::::.:::: ::::::
query   3’ ACCGAUACCGAACAAAGC 5’

query=JS16_t4090342, target=FGSG_08273, score=4, range=230-247, strand=-1

target  5’ UUUGUGUGGCUUGUUUCG 3’
           :   :.::::::::::::
query   3’ ACCGAUACCGAACAAAGC 5’

query=JS16_t4090342, target=FGSG_05889, score=4, range=1573-1591, strand=-1

target  5’ UGGGCAUGGGCUUGUUUCG 3’
           :::  :: :::::::::::
query   3’ ACCGAUA-CCGAACAAAGC 5’

query=JS16_t4090342, target=FGSG_08713, score=4, range=714-731, strand=-1

target  5’ AGGAGAUGGCUUGUUUCC 3’
            ::  ::::::::::::
query   3’ ACCGAUACCGAACAAAGC 5’

query=JS16_t4090342, target=FGSG_00850, score=4, range=1006-1023, strand=-1

target  5’ GGUCCAUGGCUUGUUUCC 3’
            : : ::::::::::::
query   3’ ACCGAUACCGAACAAAGC 5’

query=JS16_t4090342, target=FGSG_12067, score=4, range=1428-1444, strand=-1

target  5’ UGGC-CUGGCUUGUUUCU 3’
           ::::  :::::::::::
query   3’ ACCGAUACCGAACAAAGC 5’

query=JS16_t4090342, target=FGSG_02278, score=4, range=29-46, strand=-1

target  5’ UAUCCAUGGCUUGUUUCU 3’
           :  : ::::::::::::
query   3’ ACCGAUACCGAACAAAGC 5’

query=JS16_t4090421, target=FGSG_04229, score=2, range=394-411, strand=1

target  5’ UUUGAGCGAUCGGAAGCG 3’
           ::::::::::::: ::::
query   3’ AAACUCGCUAGCCGUCGC 5’

query=JS16_t4090421, target=FGSG_00559, score=2, range=252-268, strand=-1

target  5’ GUUG-GCGAUCGGCAGCG 3’
            ::: :::::::::::::
query   3’ AAACUCGCUAGCCGUCGC 5’

query=JS16_t4090421, target=FGSG_02766, score=3.5, range=1924-1942, strand=1

target  5’ AUUGGGACGAUCGGCAGCG 3’
            :::.: ::::::::::::
query   3’ AAACUC-GCUAGCCGUCGC 5’

query=JS16_t4090421, target=FGSG_00150, score=3.5, range=924-940, strand=1

target  5’ GUUGGGCGAU-GGCAGCG 3’
            :::.::::: :::::::
query   3’ AAACUCGCUAGCCGUCGC 5’

query=JS16_t4090421, target=FGSG_06885, score=3.5, range=54-70, strand=-1

target  5’ UUUGGG-GAUCGGCGGCG 3’
           ::::.: :::::::.:::
query   3’ AAACUCGCUAGCCGUCGC 5’

query=JS16_t4090421, target=FGSG_13779, score=4, range=260-277, strand=1

target  5’ CUUUAGCGAGCGGCAGCG 3’
            :: ::::: ::::::::
query   3’ AAACUCGCUAGCCGUCGC 5’

query=JS16_t4090421, target=FGSG_10589, score=4, range=12-29, strand=1

target  5’ UGAGAGCGAUCGACAGCG 3’
           :  ::::::::: :::::
query   3’ AAACUCGCUAGCCGUCGC 5’

query=JS16_t4090421, target=FGSG_05680, score=4, range=837-853, strand=1

target  5’ CUGGAGC-AUCGGCAGCG 3’
            : :::: ::::::::::
query   3’ AAACUCGCUAGCCGUCGC 5’

query=JS16_t4090421, target=FGSG_00267, score=4, range=1154-1171, strand=-1
```

```
           :::: ::::::::::::: .
query   3' ACUG-UUCCCGACCUACGUU 5'

query=JS16_t4026810, target=FGSG_06749, score=4, range=294-312, strand=1

target  5' GGCCAAGGGCUGGAUGCGU 3'
            : ::::::::::::::.
query   3' ACUGUUCCCGACCUACGUU 5'

query=JS16_t4026810, target=FGSG_02432, score=4, range=909-927, strand=1

target  5' CCCCAAGGGCUGGAUGCAC 3'
              :::::::::::::::
query   3' ACUGUUCCCGACCUACGUU 5'

query=JS16_t4026810, target=FGSG_10561, score=4, range=1177-1196, strand=1

target  5' UGGGGUUGGGCUGGAUGCAA 3'
           ::. .   :::::::::::::
query   3' ACUGU-UCCCGACCUACGUU 5'

query=JS16_t4026810, target=FGSG_10017, score=4, range=173-190, strand=1

target  5' AGACAGGGGCU-GAUGCAG 3'
            ::::.::::: ::::::.
query   3' ACUGUUCCCGACCUACGUU 5'

query=JS16_t4026810, target=FGSG_08613, score=4, range=253-271, strand=-1

target  5' UGACAAGGGCGGGAUGCGU 3'
           :::::::::: ::::::.
query   3' ACUGUUCCCGACCUACGUU 5'

query=JS16_t4026810, target=FGSG_11482, score=4, range=735-754, strand=-1

target  5' AGACAGUGGGCUGGAUGUAG 3'
            ::::. ::::::::::.:.
query   3' ACUGUU-CCCGACCUACGUU 5'

query=JS16_t4026810, target=FGSG_05179, score=4, range=1160-1177, strand=-1

target  5' UGG-GAGGGCUGGAUGUGA 3'
           ::. .::::::::::..:
query   3' ACUGUUCCCGACCUACGUU 5'

query=JS16_t4026810, target=FGSG_06744, score=4, range=715-733, strand=-1

target  5' UUUCGGGGGCUGGGUGCAA 3'
           :  :..:::::::.:::::
query   3' ACUGUUCCCGACCUACGUU 5'

query=JS16_t4026810, target=FGSG_00813, score=4, range=444-463, strand=-1

target  5' UGAACAAGGGUUGGAUCCAA 3'
           :: :::::::.::::: :::
query   3' AC-UGUUCCCGACCUACGUU 5'

query=JS16_t4026810, target=FGSG_07890, score=4, range=129-148, strand=-1

target  5' UGAGCGAGGGCUGGAAGCAG 3'
           ::: :.::::::::: :::.
query   3' ACU-GUUCCCGACCUACGUU 5'

query=JS16_t4026810, target=FGSG_11504, score=4, range=1082-1099, strand=-1

target  5' CGGCAAGGGCUGGA-GCAG 3'
            :.:::::::::: :::.
query   3' ACUGUUCCCGACCUACGUU 5'

query=JS16_t4026810, target=FGSG_13683, score=4, range=251-268, strand=-1

target  5' CGACAAGGGCUGGA-GCAU 3'
            ::::::::::::: :::
query   3' ACUGUUCCCGACCUACGUU 5'

query=JS16_t4026810, target=FGSG_08757, score=4, range=732-751, strand=-1

target  5' UGACCAUGGGCUGGAUGCUA 3'
           ::: :: ::::::::::: :
query   3' ACU-GUUCCCGACCUACGUU 5'

No results for JS16_t0394364
No results for JS16_t2234151
No results for JS16_t1337710
No results for JS16_t3130384
No results for JS16_t2234239
query=JS16_t0577179, target=FGSG_03591, score=3, range=839-858, strand=1

target  5' UGGUUC-UCGGCAUGCCCAUC 3'
           :::::: :::::::::: :::
query   3' ACCAAGUAGCCGUACGGUUAG 5'

target  5' UUUCUGCGAUCGGUGGCG 3'
           :::  ::::::::..:::
query   3' AAACUCGCUAGCCGUCGC 5'

query=JS16_t4090421, target=FGSG_04113, score=4, range=1233-1251, strand=-1

target  5' UGUGAAGUGAUUGGCAGCG 3'
           : :: ::.:::.:::::::
query   3' AAAC-UCGCUAGCCGUCGC 5'

query=JS16_t4090421, target=FGSG_07254, score=4, range=1314-1331, strand=-1

target  5' UCUGAGCGAUCGACAGCA 3'
           : :::::::::: ::::
query   3' AAACUCGCUAGCCGUCGC 5'

query=JS16_t4090421, target=FGSG_04023, score=4, range=974-990, strand=-1

target  5' AUUGAGUGAUC-GCAGCG 3'
            :::::.:::: ::::::
query   3' AAACUCGCUAGCCGUCGC 5'

query=JS16_t4090421, target=FGSG_06424, score=4, range=117-135, strand=-1

target  5' CUUGAACGCAUCGGCAGCG 3'
            :::: :: ::::::::::
query   3' AAACUCGC-UAGCCGUCGC 5'

query=JS16_t4090421, target=FGSG_12868, score=4, range=384-400, strand=-1

target  5' GAUGAGC-AUCGGCAGCG 3'
             ::::: ::::::::::
query   3' AAACUCGCUAGCCGUCGC 5'

query=JS16_t4090421, target=FGSG_04165, score=4, range=909-925, strand=-1

target  5' GUCGAGCG-UCGGCAGCG 3'
            : ::::: :::::::::
query   3' AAACUCGCUAGCCGUCGC 5'

No results for JS16_t3193669
No results for JS16_t0803034
No results for JS16_t4090517
No results for JS16_t4090526
No results for JS16_t1401564
No results for JS16_t1401572
query=JS16_t4090574, target=FGSG_03954, score=4, range=370-390, strand=1

target  5' GUUCUCUUCCGUUGCGAUGAC 3'
           : :: : :::::::::.:::::
query   3' CUAGUGUAGGCAACGUUACUG 5'

No results for JS16_t1401599
No results for JS16_t4090581
query=JS16_t3193847, target=FGSG_01173, score=4, range=1239-1259, strand=1

target  5' CAGCCAGCUUCGGGGAUCUCC 3'
            :::   ::::::::::::::
query   3' UUCGCGAGAAGCCCCUAGAGG 5'

query=JS16_t3193847, target=FGSG_02395, score=4, range=747-767, strand=-1

target  5' AAGCGAUCUUCUGGGAUUUCC 3'
           ::::: ::::: :::::.:::
query   3' UUCGCGAGAAGCCCCUAGAGG 5'

query=JS16_t3193847, target=FGSG_01733, score=4, range=1443-1463, strand=-1

target  5' GACCGUCCUUCGGGGAUUUCC 3'
           .: ::. ::::::::::.:::
query   3' UUCGCGAGAAGCCCCUAGAGG 5'

query=JS16_t3193847, target=FGSG_09474, score=4, range=140-161, strand=-1

target  5' AGCUGCUUCUUCGGGGGUCUCC 3'
           :. .:: ::::::::::.:::::
query   3' UUCGCG-AGAAGCCCCUAGAGG 5'

query=JS16_t3193847, target=FGSG_07294, score=4, range=1212-1231, strand=-1

target  5' AAG-GCUCUUCAGGGAUCUCG 3'
           ::: ::::::: ::::::::
query   3' UUCGCGAGAAGCCCCUAGAGG 5'

No results for JS16_t1401630
No results for JS16_t2297722
No results for JS16_t2297804
No results for JS16_t4090669
query=JS16_t3193965, target=FGSG_02769, score=4, range=713-732, strand=1

target  5' UCAACGAUGUGCAAGACUUU 3'
```

```
query=JS16_t1337840, target=FGSG_03452, score=3.5, range=141-162, strand=-1

target  5’ UAUGUAGAUGAUGAAGAGAUCG 3’
           .: :: :::::::::::::::
query   3’ GUUCAGCUACUACUUCUCUAGA 5’

query=JS16_t1337840, target=FGSG_12985, score=4, range=519-541, strand=1

target  5’ GAAGUACGAAGAUGAAGAGAUCG 3’
            :::: ::: ::::::::::::
query   3’ GUUCA-GCUACUACUUCUCUAGA 5’

query=JS16_t1337840, target=FGSG_01194, score=4, range=1908-1929, strand=-1

target  5’ CAGGUUGAUGAUGGAGUGAUCU 3’
           ::.::.:::::::.:: :::::
query   3’ GUUCAGCUACUACUUCUCUAGA 5’

query=JS16_t1337840, target=FGSG_11590, score=4, range=150-170, strand=-1

target  5’ GAAGGCGAUGAUGAAGA-AUCU 3’
            ::: :::::::::::: ::::
query   3’ GUUCAGCUACUACUUCUCUAGA 5’

No results for JS16_t0365041
No results for JS16_t0394371
No results for JS16_t2234348
query=JS16_t1337984, target=FGSG_08690, score=2.5, range=1239-1256, strand=-1

target  5’ CUGUUUUGGACGAAAGAG 3’
           :: ::::.:::::::::.
query   3’ GAGAAAAUCUGCUUUCUU 5’

query=JS16_t1337984, target=FGSG_07046, score=3, range=428-444, strand=1

target  5’ CUGUUUUAGACG-AAGAA 3’
           :: ::::::::: :::::
query   3’ GAGAAAAUCUGCUUUCUU 5’

query=JS16_t1337984, target=FGSG_09404, score=3.5, range=287-304, strand=-1

target  5’ CACUUUUAGGCGGAAGAG 3’
           : :::::::.::.::::.
query   3’ GAGAAAAUCUGCUUUCUU 5’

query=JS16_t1337984, target=FGSG_11044, score=3.5, range=31-48, strand=-1

target  5’ GUCCUUUAGACGGAAGAG 3’
            :: ::::::::.::::.
query   3’ GAGAAAAUCUGCUUUCUU 5’

query=JS16_t1337984, target=FGSG_05516, score=4, range=2984-3000, strand=1

target  5’ CUCUUUUAGA-GAAGGGA 3’
           :::::::::: :::.:.:
query   3’ GAGAAAAUCUGCUUUCUU 5’

query=JS16_t1337984, target=FGSG_11032, score=4, range=1132-1148, strand=1

target  5’ CUC-UUUGGAUGGAAGAA 3’
           ::: :::.::.:.:::::
query   3’ GAGAAAAUCUGCUUUCUU 5’

query=JS16_t1337984, target=FGSG_09589, score=4, range=1496-1513, strand=1

target  5’ CUCAUGUAGACGAGAGAA 3’
           ::: : :::::::.::::
query   3’ GAGAAAAUCUGCUUUCUU 5’

query=JS16_t1337984, target=FGSG_12630, score=4, range=270-287, strand=1

target  5’ GACUUUUAGUCGAAAGAA 3’
             ::::::: ::::::::
query   3’ GAGAAAAUCUGCUUUCUU 5’

query=JS16_t1337984, target=FGSG_13358, score=4, range=2007-2024, strand=1

target  5’ UCCUUUUAGACGCAAGAG 3’
           . :::::::::: ::::.
query   3’ GAGAAAAUCUGCUUUCUU 5’

query=JS16_t1337984, target=FGSG_08798, score=4, range=1053-1070, strand=1

target  5’ UCCUUUUAUACGAAAGAG 3’
           . :::::: ::::::::.
query   3’ GAGAAAAUCUGCUUUCUU 5’

query=JS16_t1337984, target=FGSG_08263, score=4, range=1327-1345, strand=1

target  5’ CUGCUUUUAUACGAAAGAU 3’
           :: :::::: ::::::::
query   3’ GA-GAAAAUCUGCUUUCUU 5’
           ::: :.::: :::::::::.
query   3’ AGUGGUUACCCGUUCUGAAG 5’

query=JS16_t3193965, target=FGSG_06952, score=4, range=234-253, strand=-1

target  5’ UCUUCAGCGGGCAAGACUUC 3’
           :: .::. ::::::::::::
query   3’ AGUGGUUACCCGUUCUGAAG 5’

query=JS16_t3193965, target=FGSG_11028, score=4, range=1836-1856, strand=-1

target  5’ UCACCAUCGGGCAAGACCUUC 3’
           ::::::  :::::::: ::::
query   3’ AGUGGUUACCCGUUCU-GAAG 5’

No results for JS16_t0284172
query=JS16_t2297870, target=FGSG_08135, score=2.5, range=1146-1164, strand=1

target  5’ ACCUUUUAGUUUCGAAACA 3’
           : :::..:::::::::::
query   3’ UCGAAGGUCAAAGCUUUGU 5’

query=JS16_t2297870, target=FGSG_11671, score=3, range=1573-1592, strand=-1

target  5’ AGCUUGCUAGUUUUGAAACA 3’
           ::::: :.:::::.::::::
query   3’ UCGAA-GGUCAAAGCUUUGU 5’

query=JS16_t2297870, target=FGSG_01364, score=3.5, range=6114-6132, strand=1

target  5’ AGCUUUCAGUUUCGAGAUU 3’
           :::::.::::::::::.:.
query   3’ UCGAAGGUCAAAGCUUUGU 5’

query=JS16_t2297870, target=FGSG_08209, score=3.5, range=1095-1113, strand=1

target  5’ GGCUUCCACUUUCGGAACA 3’
           .::::::: :::::.::::
query   3’ UCGAAGGUCAAAGCUUUGU 5’

query=JS16_t2297870, target=FGSG_06133, score=3.5, range=1468-1486, strand=1

target  5’ ACCUUCCAGAUUCGAAACG 3’
           : ::::::: ::::::::.
query   3’ UCGAAGGUCAAAGCUUUGU 5’

query=JS16_t2297870, target=FGSG_11268, score=3.5, range=1730-1748, strand=-1

target  5’ CGCUUUCGGUUUCGAAACU 3’
            ::::.:.::::::::::
query   3’ UCGAAGGUCAAAGCUUUGU 5’

query=JS16_t2297870, target=FGSG_02198, score=3.5, range=505-523, strand=-1

target  5’ CGUCUCCAGUUUUGAAACA 3’
            :.  ::::::::.::::::
query   3’ UCGAAGGUCAAAGCUUUGU 5’

query=JS16_t2297870, target=FGSG_03556, score=3.5, range=829-846, strand=-1

target  5’ AG-UUUCAGUCUCGAAACA 3’
           :: ::.:::: ::::::::
query   3’ UCGAAGGUCAAAGCUUUGU 5’

query=JS16_t2297870, target=FGSG_04278, score=4, range=1032-1050, strand=1

target  5’ AGCUUUCAGUUUCGAGAAG 3’
           :::::.::::::::::.: .
query   3’ UCGAAGGUCAAAGCUUUGU 5’

query=JS16_t2297870, target=FGSG_03455, score=4, range=791-809, strand=1

target  5’ AAUUGCCAGUUUCGAAAUG 3’
           : .: :::::::::::::..
query   3’ UCGAAGGUCAAAGCUUUGU 5’

query=JS16_t2297870, target=FGSG_08651, score=4, range=172-189, strand=1

target  5’ AGCUCCCAGUUUC-AAGCA 3’
           :::: :::::::: ::.::
query   3’ UCGAAGGUCAAAGCUUUGU 5’

query=JS16_t2297870, target=FGSG_04769, score=4, range=133-150, strand=1

target  5’ UACUUCC-GUUUCGAAACA 3’
             ::::: :::::::::::
query   3’ UCGAAGGUCAAAGCUUUGU 5’

query=JS16_t2297870, target=FGSG_10736, score=4, range=201-219, strand=1

target  5’ AGAUCCCAGUUUCGACACA 3’
           :: : :::::::::: :::
```

```
query=JS16_t1337984, target=FGSG_10043, score=4, range=631-649, strand=1

target  5’ CUCAUCUUUGACGAAAGAA 3’
           ::: : :: ::::::::::
query   3’ GAG-AAAAUCUGCUUUCUU 5’

query=JS16_t1337984, target=FGSG_08278, score=4, range=592-609, strand=-1

target  5’ AUCUUUUGGUCGAAAGAA 3’
            ::::::.: ::::::::
query   3’ GAGAAAAUCUGCUUUCUU 5’

query=JS16_t1337984, target=FGSG_11181, score=4, range=2184-2201, strand=-1

target  5’ CUGUUUUGGACGAGAGAC 3’
           :: ::::.:::::.:::
query   3’ GAGAAAAUCUGCUUUCUU 5’

query=JS16_t1337984, target=FGSG_10061, score=4, range=2150-2167, strand=-1

target  5’ CGCUUUUAGACGAGACAA 3’
           : ::::::::::.: ::
query   3’ GAGAAAAUCUGCUUUCUU 5’

query=JS16_t1337984, target=FGSG_08129, score=4, range=1633-1650, strand=-1

target  5’ GUCUUUUAGACGAACGAU 3’
            ::::::::::::: ::
query   3’ GAGAAAAUCUGCUUUCUU 5’

query=JS16_t1337984, target=FGSG_12014, score=4, range=560-577, strand=-1

target  5’ CACUUUUAUACGAAAGAC 3’
           : :::::: ::::::::
query   3’ GAGAAAAUCUGCUUUCUU 5’

query=JS16_t1337984, target=FGSG_05050, score=4, range=188-204, strand=-1

target  5’ CUC-UUUGGACGAAAAAA 3’
           ::: :::.::::::: ::
query   3’ GAGAAAAUCUGCUUUCUU 5’

query=JS16_t0577197, target=FGSG_01442, score=3, range=4033-4052, strand=-1

target  5’ AAGGUGAAGAGCCACGGGAA 3’
           ::::::::::.:::::: ::
query   3’ UUCCACUUCUUGGUGCCGUU 5’

query=JS16_t0577197, target=FGSG_01272, score=3.5, range=1994-2013, strand=-1

target  5’ AGGCUGAGGAACCACGGCAU 3’
           :.: :::.:::::::::::
query   3’ UUCCACUUCUUGGUGCCGUU 5’

query=JS16_t0577197, target=FGSG_07891, score=3.5, range=2144-2162, strand=-1

target  5’ GAUGUG-AGAACCACGGCGA 3’
           .: ::: :::::::::::.:
query   3’ UUCCACUUCUUGGUGCCGUU 5’

query=JS16_t0577197, target=FGSG_09520, score=4, range=594-613, strand=1

target  5’ AAGGUCAAGAACUACGGGAA 3’
           ::::: ::::::.:::: ::
query   3’ UUCCACUUCUUGGUGCCGUU 5’

query=JS16_t0577197, target=FGSG_01150, score=4, range=1240-1259, strand=1

target  5’ AAGAAGAAGGGCCACGGCAA 3’
           :::  ::::..:::::::::
query   3’ UUCCACUUCUUGGUGCCGUU 5’

query=JS16_t0577197, target=FGSG_09816, score=4, range=1465-1483, strand=1

target  5’ GGGUUGAAGAACCAC-GCAA 3’
           ..: ::::::::::: ::::
query   3’ UUCCACUUCUUGGUGCCGUU 5’

query=JS16_t0577197, target=FGSG_03049, score=4, range=23-42, strand=-1

target  5’ AAGGCCAAGAGCCACGGUAA 3’
           ::::  ::::.::::::.::
query   3’ UUCCACUUCUUGGUGCCGUU 5’

No results for JS16_t2234452
query=JS16_t2234455, target=FGSG_08832, score=4, range=805-824, strand=1

target  5’ GAGCGC-GUUCUUGAGGUCUA 3’
           : :::: ::::.::::::::
query   3’ CCCGCGCCAAGGACUCCAGAA 5’

query   3’ UCGAAGGUCAAAGCUUUGU 5’

query=JS16_t2297870, target=FGSG_10651, score=4, range=732-749, strand=1

target  5’ AUCUCCCAG-UUCGAAACA 3’
           : :: :::: :::::::::
query   3’ UCGAAGGUCAAAGCUUUGU 5’

query=JS16_t2297870, target=FGSG_08831, score=4, range=520-538, strand=-1

target  5’ GUCGUCCAGUUUCGAAGCG 3’
           .  : ::::::::::::.:.
query   3’ UCGAAGGUCAAAGCUUUGU 5’

query=JS16_t2297870, target=FGSG_03407, score=4, range=2539-2557, strand=-1

target  5’ UGCUGCCAGUUUCGAAAGA 3’
            ::: :::::::::::: :
query   3’ UCGAAGGUCAAAGCUUUGU 5’

query=JS16_t2297870, target=FGSG_10302, score=4, range=184-203, strand=-1

target  5’ GGCUUCUCAGUUUCGAAUCG 3’
           .::::: :::::::::: :.
query   3’ UCGAAG-GUCAAAGCUUUGU 5’

No results for JS16_t0269804
query=JS16_t0139842, target=FGSG_02581, score=4, range=691-714, strand=1

target  5’ GAGGUUCAGCCCAAAUCACAGUUU 3’
           : :::::::  ::::::::::::
query   3’ CCCCAAGUC-AGUUUAGUGUCAAC 5’

query=JS16_t0269808, target=FGSG_13678, score=3.5, range=239-260, strand=-1

target  5’ ACGUCGUGGCGAUCAGCUGCAU 3’
            :.::::  :::::::::::::
query   3’ GGUAGCAAAGCUAGUCGACGUA 5’

query=JS16_t0188920, target=FGSG_06206, score=3.5, range=352-373, strand=1

target  5’ CGAGUUCCAUC-ACACGAAGAUG 3’
           :.  :::::::: :::::::::::
query   3’ GUCCAAGGUAGUUGUGCUUCUAC 5’

No results for JS16_t2298052
No results for JS16_t0803077
query=JS16_t4090969, target=FGSG_00067, score=4, range=449-466, strand=1

target  5’ UUGGUCUAGGAAUCGUUU 3’
           ::::::::::: : ::::
query   3’ AACCAGAUCCUAAUCAAA 5’

query=JS16_t4090969, target=FGSG_12940, score=4, range=1792-1808, strand=-1

target  5’ UUGGUCUCGGAUU-GUUU 3’
           ::::::: ::::: ::::
query   3’ AACCAGAUCCUAAUCAAA 5’

query=JS16_t0194949, target=FGSG_10591, score=3.5, range=584-602, strand=-1

target  5’ ACAAUGUC-ACAGUCGUGUA 3’
           ::: :.:: :::::::::::
query   3’ UGUAAUAGUUGUCAGCACAU 5’

query=JS16_t0194949, target=FGSG_02937, score=4, range=992-1010, strand=1

target  5’ AUGUUAACAACAG-CGUGUA 3’
           :..::: :::::: ::::::
query   3’ UGUAAUAGUUGUCAGCACAU 5’

query=JS16_t0194949, target=FGSG_06881, score=4, range=228-248, strand=-1

target  5’ ACAUUCAUCAACGGUAGUGUA 3’
           ::::: ::::::.:: :::::
query   3’ UGUAA-UAGUUGUCAGCACAU 5’

query=JS16_t0194949, target=FGSG_11899, score=4, range=76-95, strand=-1

target  5’ ACUUCAUCAACAGUAGUGUA 3’
           :: : ::::::::: :::::
query   3’ UGUAAUAGUUGUCAGCACAU 5’

No results for JS16_t3194182
query=JS16_t0284177, target=FGSG_09838, score=2.5, range=2729-2746, strand=1

target  5’ UGGCACAGUUCAUUGAGC 3’
           ::.  :::::.::::::::
query   3’ ACUCUGUCAGGUAACUCG 5’

query=JS16_t0284177, target=FGSG_09111, score=3, range=1896-1912, strand=1
```

```
query=JS16_t2234455, target=FGSG_03512, score=4, range=1121-1140, strand=-1

target  5’ GGACGCGUUUCC-GAGGUCUU 3’
           :: :::: :::: ::::::::
query   3’ CCCGCGCCAAGGACUCCAGAA 5’

No results for JS16_t4027196
No results for JS16_t2234587
query=JS16_t0795222, target=FGSG_13201, score=4, range=457-476, strand=-1

target  5’ GCCC-ACGGCAACAUGUCCAA 3’
           :::: : :::::::..:::::
query   3’ CGGGAUUCCGUUGUGUAGGUU 5’

query=JS16_t0335580, target=FGSG_01797, score=3, range=123-142, strand=-1

target  5’ AUCCA-GGGACCGUGGAGUUC 3’
           ::::: :::::::::.:::.:
query   3’ UAGGUACCCUGGCACUUCAGG 5’

query=JS16_t0335580, target=FGSG_05154, score=4, range=765-785, strand=-1

target  5’ AUCCAUGGUACUGUGGAGUCC 3’
           :::::::: ::.:::.:::::
query   3’ UAGGUACCCUGGCACUUCAGG 5’

query=JS16_t0335580, target=FGSG_06837, score=4, range=624-644, strand=-1

target  5’ UUUCCUUGGACCGUGAAGUCU 3’
            :.: : ::::::::::::.
query   3’ UAGGUACCCUGGCACUUCAGG 5’

No results for JS16_t1338135
No results for JS16_t0188487
query=JS16_t3130792, target=FGSG_06487, score=2.5, range=100-121, strand=-1

target  5’ AUUGCGUGACUGGCCUGUUGCC 3’
           :.: :..::::::::::::::
query   3’ UGA-GUGCUGACCGGACAACGG 5’

query=JS16_t3130792, target=FGSG_02615, score=3.5, range=554-573, strand=1

target  5’ UCUU-CGACUGGCUUGUUGCC 3’
            ::.  ::::::::.:::::::
query   3’ UGAGUGCUGACCGGACAACGG 5’

query=JS16_t3130792, target=FGSG_01688, score=4, range=314-333, strand=1

target  5’ GCUCAC-CCUGGCUUGUUGCU 3’
           .:::::  :::::.::::::.
query   3’ UGAGUGCUGACCGGACAACGG 5’

No results for JS16_t0219742
query=JS16_t0335584, target=FGSG_10284, score=3.5, range=1081-1100, strand=1

target  5’ GGCGAGCAGUCACAA-UUCAU 3’
           .:::: ::::::::: :::::
query   3’ UCGCUAGUCAGUGUUCAAGUA 5’

query=JS16_t0335584, target=FGSG_13252, score=4, range=2245-2264, strand=1

target  5’ GGC-AUCGGUCGCAAGUUCAA 3’
           .:: :::.:::.::::::::
query   3’ UCGCUAGUCAGUGUUCAAGUA 5’

query=JS16_t0335584, target=FGSG_10059, score=4, range=2689-2708, strand=1

target  5’ AGC-AUCAGUCACAACUUCAG 3’
           ::: ::::::::::: ::::
query   3’ UCGCUAGUCAGUGUUCAAGUA 5’

No results for JS16_t0577232
query=JS16_t0394388, target=FGSG_09592, score=3, range=643-659, strand=1

target  5’ GAG-AUCAGCAAUCACGU 3’
           ::: :::::::::: :::
query   3’ CUCGUAGUCGUUAGCGCA 5’

query=JS16_t0394388, target=FGSG_10966, score=3.5, range=87-104, strand=1

target  5’ GAGUUUUAGCAAUCGCGC 3’
           :::. :.::::::::::
query   3’ CUCGUAGUCGUUAGCGCA 5’

query=JS16_t0394388, target=FGSG_03343, score=3.5, range=2766-2783, strand=1

target  5’ GGGCUACAGCAAUCGCGU 3’
           :.::  ::::::::::::
query   3’ CUCGUAGUCGUUAGCGCA 5’

query=JS16_t0394388, target=FGSG_00939, score=3.5, range=2533-2549, strand=1

target  5’ CGAG-CAGUCUAUUGAGC 3’
            ::: :::::.:::::::
query   3’ ACUCUGUCAGGUAACUCG 5’

query=JS16_t0284177, target=FGSG_05190, score=3, range=1218-1234, strand=1

target  5’ UGAGACAG-CCGUUGAGC 3’
           :::::::: ::.::::::
query   3’ ACUCUGUCAGGUAACUCG 5’

query=JS16_t0284177, target=FGSG_12299, score=3.5, range=159-175, strand=1

target  5’ UGA-ACAGUCCCUUGAGU 3’
           ::: ::::::: :::::.
query   3’ ACUCUGUCAGGUAACUCG 5’

query=JS16_t0284177, target=FGSG_13256, score=3.5, range=332-348, strand=1

target  5’ GGAGGCAGU-CAUUGAGC 3’
            :::.:::: ::::::::
query   3’ ACUCUGUCAGGUAACUCG 5’

query=JS16_t0284177, target=FGSG_03409, score=3.5, range=303-321, strand=-1

target  5’ UGGGAUAGUCCACUUGAGU 3’
           ::.::.:::::: :::::.
query   3’ ACUCUGUCAGGU-AACUCG 5’

query=JS16_t0284177, target=FGSG_09539, score=3.5, range=6-23, strand=-1

target  5’ UGAGGCAGUCCAUUAAGG 3’
           ::::.::::::::: ::
query   3’ ACUCUGUCAGGUAACUCG 5’

query=JS16_t0284177, target=FGSG_08955, score=3.5, range=1935-1952, strand=-1

target  5’ AGGUACAGUUCAUUGAGC 3’
            :.  :::::.::::::::
query   3’ ACUCUGUCAGGUAACUCG 5’

query=JS16_t0284177, target=FGSG_07496, score=4, range=1791-1809, strand=1

target  5’ CGAGACAGUCUAUUUGAGC 3’
            ::::::::.: ::::::
query   3’ ACUCUGUCAGGU-AACUCG 5’

query=JS16_t0284177, target=FGSG_04392, score=4, range=482-499, strand=1

target  5’ UGAGACAGUACUUUGAGC 3’
           ::::::::: : ::::::
query   3’ ACUCUGUCAGGUAACUCG 5’

query=JS16_t0284177, target=FGSG_07758, score=4, range=143-159, strand=1

target  5’ UGAAACAGUCCA-UGGGC 3’
           ::: :::::::: ::.::
query   3’ ACUCUGUCAGGUAACUCG 5’

query=JS16_t0284177, target=FGSG_13085, score=4, range=2-18, strand=1

target  5’ UGAG-CGGUGCAUUGAGC 3’
           :::: :.:: ::::::::
query   3’ ACUCUGUCAGGUAACUCG 5’

query=JS16_t0284177, target=FGSG_00979, score=4, range=599-616, strand=1

target  5’ UCACUCAGUUCAUUGAGC 3’
           : :  ::::.::::::::
query   3’ ACUCUGUCAGGUAACUCG 5’

query=JS16_t0284177, target=FGSG_03593, score=4, range=1508-1524, strand=1

target  5’ CGA-ACAGUCCAUUCAGC 3’
            :: :::::::::: :::
query   3’ ACUCUGUCAGGUAACUCG 5’

query=JS16_t0284177, target=FGSG_06301, score=4, range=300-316, strand=1

target  5’ UGA-UCAGGCCAUUGAGC 3’
           :::  ::: :::::::::
query   3’ ACUCUGUCAGGUAACUCG 5’

query=JS16_t0284177, target=FGSG_08704, score=4, range=2831-2848, strand=1

target  5’ UGGAAUGGUCCAUUGAGU 3’
           ::.  :..::::::::::.
query   3’ ACUCUGUCAGGUAACUCG 5’

query=JS16_t0284177, target=FGSG_01724, score=4, range=275-291, strand=1

target  5’ UAAGCCAGUCCA-UGAGC 3’
           : :: ::::::: :::::
```

```
target  5’ GAG-GUCAGCAAUCGUGA 3’
           ::: .::::::::::.:
query   3’ CUCGUAGUCGUUAGCGCA 5’

query=JS16_t0394388, target=FGSG_13783, score=3.5, range=18764-18781, strand=

target  5’ GGACAUCGGCAAUCGUGU 3’
           :. ::::.:::::::.::
query   3’ CUCGUAGUCGUUAGCGCA 5’

query=JS16_t0394388, target=FGSG_01973, score=3.5, range=1775-1792, strand=-1

target  5’ CAGUAUCAGCCAUCGCGU 3’
            ::.:::::: :::::::
query   3’ CUCGUAGUCGUUAGCGCA 5’

query=JS16_t0394388, target=FGSG_11155, score=3.5, range=1163-1180, strand=-1

target  5’ GACUCUCAGCAAUCGCGC 3’
           :: . ::::::::::::
query   3’ CUCGUAGUCGUUAGCGCA 5’

query=JS16_t0394388, target=FGSG_05536, score=4, range=979-995, strand=1

target  5’ GAGC-UUGGCAGUCGCGU 3’
           :::: :..:::.::::::
query   3’ CUCGUAGUCGUUAGCGCA 5’

query=JS16_t0394388, target=FGSG_04633, score=4, range=230-247, strand=1

target  5’ GAUCAUCACCAGUCGCGU 3’
           :: ::::: ::.::::::
query   3’ CUCGUAGUCGUUAGCGCA 5’

query=JS16_t0394388, target=FGSG_10282, score=4, range=417-434, strand=1

target  5’ GACCAUCAGCAAGCGUGU 3’
           :: ::::::::: ::.::
query   3’ CUCGUAGUCGUUAGCGCA 5’

query=JS16_t0394388, target=FGSG_06769, score=4, range=2031-2048, strand=1

target  5’ UCGCAUCAGCAAUCGCAU 3’
             :::::::::::::: :
query   3’ CUCGUAGUCGUUAGCGCA 5’

query=JS16_t0394388, target=FGSG_06101, score=4, range=3014-3030, strand=1

target  5’ AAGC-UCAGCAGUUGCGU 3’
            ::: ::::::.:.::::
query   3’ CUCGUAGUCGUUAGCGCA 5’

query=JS16_t0394388, target=FGSG_11395, score=4, range=1264-1280, strand=1

target  5’ GA-UGUCAGCAAUCGUGA 3’
           :: ..::::::::::.:
query   3’ CUCGUAGUCGUUAGCGCA 5’

query=JS16_t0394388, target=FGSG_10722, score=4, range=2980-2996, strand=1

target  5’ GAG-AUCAUCAAUCGUGU 3’
           ::: :::: ::::::.::
query   3’ CUCGUAGUCGUUAGCGCA 5’

query=JS16_t0394388, target=FGSG_10533, score=4, range=3-19, strand=1

target  5’ GAG-AUCAACAAUUGCGU 3’
           ::: :::: ::::.::::
query   3’ CUCGUAGUCGUUAGCGCA 5’

query=JS16_t0394388, target=FGSG_08233, score=4, range=275-293, strand=1

target  5’ GUGCAGCAGCAAUCGGCGU 3’
           : ::: :::::::: ::::
query   3’ CUCGUAGUCGUUAG-CGCA 5’

query=JS16_t0394388, target=FGSG_03480, score=4, range=1319-1335, strand=1

target  5’ UAG-AUCAGCAAUCGAGU 3’
            :: ::::::::::: ::
query   3’ CUCGUAGUCGUUAGCGCA 5’

query=JS16_t0394388, target=FGSG_02897, score=4, range=222-238, strand=1

target  5’ UAUCAUCAGCAAUCG-GU 3’
            : :::::::::::: ::
query   3’ CUCGUAGUCGUUAGCGCA 5’

query=JS16_t0394388, target=FGSG_02825, score=4, range=850-866, strand=1

target  5’ GAGCA-CAGGAAUCGCGU 3’
           ::::: ::: ::::::::

query   3’ ACUCUGUCAGGUAACUCG 5’

query=JS16_t0284177, target=FGSG_00678, score=4, range=576-593, strand=-1

target  5’ UGAGGCAGUCCAUGGGGU 3’
           ::::.:::::::: :.:.
query   3’ ACUCUGUCAGGUAACUCG 5’

query=JS16_t0284177, target=FGSG_05882, score=4, range=1182-1199, strand=-1

target  5’ GGAGACAGUUCGUUGAGA 3’
            ::::::::.:.:::::
query   3’ ACUCUGUCAGGUAACUCG 5’

query=JS16_t0284177, target=FGSG_04660, score=4, range=1039-1056, strand=-1

target  5’ GGUGAUAGUCCGUUGAGC 3’
            : ::.:::::.::::::
query   3’ ACUCUGUCAGGUAACUCG 5’

query=JS16_t0284177, target=FGSG_05050, score=4, range=1008-1024, strand=-1

target  5’ UGAG-UAGUUCGUUGAGC 3’
           :::: .:::.:.::::::
query   3’ ACUCUGUCAGGUAACUCG 5’

query=JS16_t0284177, target=FGSG_11565, score=4, range=259-276, strand=-1

target  5’ AGUGACAGGCCAUUGAGC 3’
            : ::::: :::::::::
query   3’ ACUCUGUCAGGUAACUCG 5’

query=JS16_t0284177, target=FGSG_06423, score=4, range=1143-1161, strand=-1

target  5’ CCAGUACAGUCCAUUGAGA 3’
             :: :::::::::::::
query   3’ ACUC-UGUCAGGUAACUCG 5’

query=JS16_t0284177, target=FGSG_02734, score=4, range=1240-1258, strand=-1

target  5’ UGAUUCGAGUCCAUUGAGC 3’
           :::  : ::::::::::::
query   3’ ACUCUG-UCAGGUAACUCG 5’

No results for JS16_t0693540
No results for JS16_t3194304
query=JS16_t4091155, target=FGSG_01159, score=4, range=1645-1663, strand=1

target  5’ CGAGGG-UUGCGCAACCCGU 3’
            ::::: :::::::::::.
query   3’ ACUCCCGAACGCGUUGGGUU 5’

query=JS16_t4091155, target=FGSG_01865, score=4, range=225-245, strand=1

target  5’ UGAGGCCAUGCGCAACUCCAA 3’
           ::::: : :::::::: ::::
query   3’ ACUCCCGAACGCGUUG-GGUU 5’

query=JS16_t4091155, target=FGSG_10548, score=4, range=4896-4914, strand=-1

target  5’ UGA-GGCUUGUGUGACCCAA 3’
           ::: ::::::.:..::::::
query   3’ ACUCCCGAACGCGUUGGGUU 5’

query=JS16_t1402160, target=FGSG_07325, score=4, range=898-918, strand=1

target  5’ GAUGCAGAAGAUGAUGAUCAA 3’
              ::::::::::::::.:::
query   3’ AACCGUCUUCUACUACUGGUU 5’

query=JS16_t1402160, target=FGSG_08701, score=4, range=3372-3392, strand=1

target  5’ CAAGCAGAAGAUGAUGAUCAA 3’
              ::::::::::::::.:::
query   3’ AACCGUCUUCUACUACUGGUU 5’

query=JS16_t1402160, target=FGSG_06071, score=4, range=1026-1046, strand=1

target  5’ ACAGCAGAGGAUGAUGACCAA 3’
              :::::.::::::::::::
query   3’ AACCGUCUUCUACUACUGGUU 5’

query=JS16_t1402160, target=FGSG_00630, score=4, range=414-434, strand=1

target  5’ AUUUCAGAAGAUGAUGGCCAA 3’
            :  ::::::::::::.::::
query   3’ AACCGUCUUCUACUACUGGUU 5’

query=JS16_t1402160, target=FGSG_05581, score=4, range=2389-2408, strand=1

target  5’ UUGGCAGCGGAUGAUG-CCAA 3’
           ::::::: .::::::: ::::
```

```
query   3' CUCGUAGUCGUUAGCGCA 5'

query=JS16_t0394388, target=FGSG_06181, score=4, range=224-241, strand=-1

target  5' CAGCAUCAGUGGUCGCGU 3'
            ::::::::...::::::
query   3' CUCGUAGUCGUUAGCGCA 5'

query=JS16_t0394388, target=FGSG_07946, score=4, range=4954-4972, strand=-1

target  5' GACGCAUUAGCGAUUGCGU 3'
           :: ::::.:::.::.::::
query   3' CU-CGUAGUCGUUAGCGCA 5'

query=JS16_t0394388, target=FGSG_04165, score=4, range=1287-1303, strand=-1

target  5' GGGUAUCAGCAAUC-UGU 3'
           :.:.:::::::::: .::
query   3' CUCGUAGUCGUUAGCGCA 5'

query=JS16_t0394388, target=FGSG_12633, score=4, range=410-427, strand=-1

target  5' CAGCAUCAGCAAGCGCGC 3'
            ::::::::::: ::::
query   3' CUCGUAGUCGUUAGCGCA 5'

query=JS16_t0394388, target=FGSG_09227, score=4, range=1316-1332, strand=-1

target  5' GAGC-UCAGCAGUUGCGG 3'
           :::: ::::::.:.:::
query   3' CUCGUAGUCGUUAGCGCA 5'

query=JS16_t0394388, target=FGSG_07511, score=4, range=749-766, strand=-1

target  5' GUUCAUUAGCAAUCGCGC 3'
           :  :::.::::::::::
query   3' CUCGUAGUCGUUAGCGCA 5'

query=JS16_t0394388, target=FGSG_03955, score=4, range=40-57, strand=-1

target  5' GACCAUCAGCCAUCGCGG 3'
           :: ::::::: ::::::
query   3' CUCGUAGUCGUUAGCGCA 5'

query=JS16_t0394388, target=FGSG_00541, score=4, range=839-856, strand=-1

target  5' AAGAUUUAGCAAUCGCGU 3'
            ::  :.:::::::::::
query   3' CUCGUAGUCGUUAGCGCA 5'

query=JS16_t0394388, target=FGSG_09308, score=4, range=1223-1239, strand=-1

target  5' GAGC-UCAGCAAUCUUGU 3'
           :::: ::::::::: .::
query   3' CUCGUAGUCGUUAGCGCA 5'

query=JS16_t0394388, target=FGSG_05556, score=4, range=1100-1116, strand=-1

target  5' GAG-AUCAGCAACCGCGG 3'
           ::: :::::::: ::::
query   3' CUCGUAGUCGUUAGCGCA 5'

query=JS16_t0394388, target=FGSG_08604, score=4, range=97-113, strand=-1

target  5' GA-CUUCAGCAGUCGCGA 3'
           :: : ::::::.:::::
query   3' CUCGUAGUCGUUAGCGCA 5'

No results for JS16_t3130907
query=JS16_t0335587, target=FGSG_10469, score=3.5, range=424-441, strand=-1

target  5' AAGGACGGUGUCUCUUGA 3'
           :::.::::.:::::.::
query   3' UUCUUGCCGCAGAGGACC 5'

query=JS16_t0335587, target=FGSG_10564, score=3.5, range=5685-5702, strand=-1

target  5' AAGAGCGGUGUCUCCUCG 3'
           ::::.:::.::::::: :
query   3' UUCUUGCCGCAGAGGACC 5'

query=JS16_t0335587, target=FGSG_13028, score=3.5, range=1460-1476, strand=-1

target  5' GAGAAUGGCGUC-CCUGG 3'
           .::::.:::::: :::::
query   3' UUCUUGCCGCAGAGGACC 5'

query=JS16_t0335587, target=FGSG_10392, score=4, range=437-454, strand=1

target  5' GGGAACGGUGUCUCCAGG 3'
           ..::::::.:::::: ::
query   3' UUCUUGCCGCAGAGGACC 5'
```

```
query   3' AACCGUCUUCUACUACUGGUU 5'

query=JS16_t1402160, target=FGSG_01909, score=4, range=1552-1571, strand=1

target  5' UUGUCAAAAGAUGAUG-CCAA 3'
           ::: :: ::::::::: ::::
query   3' AACCGUCUUCUACUACUGGUU 5'

query=JS16_t1402160, target=FGSG_04829, score=4, range=236-255, strand=1

target  5' ACGGC-CAAGAUGAUGACCAA 3'
             :::  ::::::::::::::
query   3' AACCGUCUUCUACUACUGGUU 5'

query=JS16_t1402160, target=FGSG_07634, score=4, range=1666-1686, strand=-1

target  5' CAGGCUGAAGAUGAUGGCCAA 3'
             ::: ::::::::::.::::
query   3' AACCGUCUUCUACUACUGGUU 5'

query=JS16_t1402162, target=FGSG_09310, score=4, range=55-75, strand=-1

target  5' CA-GUGCAGAAACCCCACGGAG 3'
           :: :::: :::::: :::::::
query   3' GUACACGGCUUUGGAGUGCCUC 5'

query=JS16_t0584869, target=FGSG_01733, score=2, range=1524-1541, strand=1

target  5' UGCUGCGAAGAAUGAUGU 3'
           .: ::::::::::::::.
query   3' GCCACGCUUCUUACUACG 5'

query=JS16_t0584869, target=FGSG_12607, score=2.5, range=244-260, strand=1

target  5' UGG-GUGAAGAAUGAUGC 3'
           .:: :.::::::::::::
query   3' GCCACGCUUCUUACUACG 5'

query=JS16_t0584869, target=FGSG_09077, score=2.5, range=548-565, strand=-1

target  5' UGGUUCGAGGAAUGAUGC 3'
           .::: :::.:::::::::
query   3' GCCACGCUUCUUACUACG 5'

query=JS16_t0584869, target=FGSG_13983, score=3, range=1850-1868, strand=1

target  5' GGGUGUCGAAGAAUGAUGG 3'
            :::: ::::::::::::
query   3' GCCAC-GCUUCUUACUACG 5'

query=JS16_t0584869, target=FGSG_13822, score=3, range=1679-1697, strand=1

target  5' CCGUGUCGAAGAAUGAUGA 3'
           : ::: ::::::::::::
query   3' GCCAC-GCUUCUUACUACG 5'

query=JS16_t0584869, target=FGSG_00923, score=3, range=1567-1584, strand=-1

target  5' CGAGGCGAAGAGUGAUGC 3'
           ::  :::::::.::::::
query   3' GCCACGCUUCUUACUACG 5'

query=JS16_t0584869, target=FGSG_10750, score=3.5, range=3252-3269, strand=1

target  5' UGGUGUCAAGAAUGAUGC 3'
           .::::. :::::::::::
query   3' GCCACGCUUCUUACUACG 5'

query=JS16_t0584869, target=FGSG_02063, score=3.5, range=64-80, strand=1

target  5' UGGUUC-AAGAAUGAUGC 3'
           .::: : :::::::::::
query   3' GCCACGCUUCUUACUACG 5'

query=JS16_t0584869, target=FGSG_08548, score=3.5, range=1292-1309, strand=-1

target  5' UGGUGCGGAGAAUGUUGC 3'
           .::::::.:::::: :::
query   3' GCCACGCUUCUUACUACG 5'

query=JS16_t0584869, target=FGSG_04361, score=3.5, range=3415-3432, strand=-1

target  5' UCGUGUGAAGAGUGAUGC 3'
           . :::.:::::.::::::
query   3' GCCACGCUUCUUACUACG 5'

query=JS16_t0584869, target=FGSG_13414, score=3.5, range=554-572, strand=-1

target  5' AGGUCGCGAAGAAUGGUGU 3'
            ::: ::::::::::.::.
query   3' GCCA-CGCUUCUUACUACG 5'
```

```
query=JS16_t0335587, target=FGSG_04565, score=4, range=440-456, strand=1

target  5’ UAGAAC-GCGUUUCCUGG 3’
            ::::: ::::.::::::
query   3’ UUCUUGCCGCAGAGGACC 5’

query=JS16_t0335587, target=FGSG_03483, score=4, range=778-795, strand=-1

target  5’ GAGGACGGUGUUUCCUGC 3’
           .::.::::.::.:::::
query   3’ UUCUUGCCGCAGAGGACC 5’

query=JS16_t0335587, target=FGSG_03603, score=4, range=2119-2136, strand=-1

target  5’ AAGAAUGGCGUCUCUAGG 3’
           :::::.::::::::. ::
query   3’ UUCUUGCCGCAGAGGACC 5’

query=JS16_t0335587, target=FGSG_02743, score=4, range=1293-1310, strand=-1

target  5’ GAGGAUGGCGUCUCCGGG 3’
           .::.:.::::::::: ::
query   3’ UUCUUGCCGCAGAGGACC 5’

query=JS16_t0335587, target=FGSG_10103, score=4, range=831-848, strand=-1

target  5’ GGGGGCGGCGACUCCUGG 3’
           ..:..::::: :::::::
query   3’ UUCUUGCCGCAGAGGACC 5’

query=JS16_t0335587, target=FGSG_04071, score=4, range=135-153, strand=-1

target  5’ GAUAAUGCGCGUCUCCUGG 3’
           .: ::.: ::::::::::
query   3’ UUCUUGC-CGCAGAGGACC 5’

query=JS16_t0335587, target=FGSG_00220, score=4, range=1556-1572, strand=-1

target  5’ AAGAACGGCGU-UCUUGC 3’
           ::::::::::: ::.::
query   3’ UUCUUGCCGCAGAGGACC 5’

query=JS16_t0335587, target=FGSG_02475, score=4, range=60-76, strand=-1

target  5’ UAGAA-GGUGUCUCCUGG 3’
            :::: ::.:::::::::
query   3’ UUCUUGCCGCAGAGGACC 5’

query=JS16_t0335587, target=FGSG_07672, score=4, range=452-468, strand=-1

target  5’ GAGGA-GGCGUCUCCUGA 3’
           .::.: ::::::::::
query   3’ UUCUUGCCGCAGAGGACC 5’

query=JS16_t0335587, target=FGSG_01629, score=4, range=976-993, strand=-1

target  5’ CAGAUCGACGUCUCCUGG 3’
            ::: :: ::::::::::
query   3’ UUCUUGCCGCAGAGGACC 5’

query=JS16_t0335587, target=FGSG_03145, score=4, range=658-675, strand=-1

target  5’ AGCGUCGGCGUCUCCUGU 3’
           :. . ::::::::::::
query   3’ UUCUUGCCGCAGAGGACC 5’

query=JS16_t0335587, target=FGSG_01886, score=4, range=877-895, strand=-1

target  5’ AAGACUCUGCGUCUCCUGG 3’
           ::::  : :::::::::::
query   3’ UUCU-UGCCGCAGAGGACC 5’

No results for JS16_t0685942
query=JS16_t0335591, target=FGSG_02774, score=3, range=1138-1156, strand=1

target  5’ CGUGACUUGGCGAAAGCCG 3’
            ::::::::.::::::::
query   3’ ACACUGAACUGCUUUCGGA 5’

query=JS16_t0335591, target=FGSG_04931, score=3, range=1362-1380, strand=-1

target  5’ UGAGGUUUGACGAAAGCCA 3’
           :: :..::::::::::::
query   3’ ACACUGAACUGCUUUCGGA 5’

query=JS16_t0335591, target=FGSG_06885, score=3, range=2108-2126, strand=-1

target  5’ UCUGACUUGCCGAAAGCCU 3’
           : ::::::: :::::::::
query   3’ ACACUGAACUGCUUUCGGA 5’

query=JS16_t0584869, target=FGSG_11088, score=3.5, range=137-153, strand=-1

target  5’ UGG-GCCAAGAAUGAUGC 3’
           .:: :: ::::::::::
query   3’ GCCACGCUUCUUACUACG 5’

query=JS16_t0584869, target=FGSG_04336, score=3.5, range=628-645, strand=-1

target  5’ CGAGACGAAGAAUGAUGU 3’
           ::   ::::::::::::.
query   3’ GCCACGCUUCUUACUACG 5’

query=JS16_t0584869, target=FGSG_10269, score=4, range=384-401, strand=1

target  5’ CCGUGCGAAGAAGGGUGC 3’
           : :::::::::: :.:::
query   3’ GCCACGCUUCUUACUACG 5’

query=JS16_t0584869, target=FGSG_11560, score=4, range=2223-2240, strand=1

target  5’ AGGUGCGAAGAAGGAUGG 3’
            ::::::::::: ::::
query   3’ GCCACGCUUCUUACUACG 5’

query=JS16_t0584869, target=FGSG_02905, score=4, range=1604-1620, strand=1

target  5’ CGGU-CGAGGAAUGGUGG 3’
           :::: :::.:::::.::
query   3’ GCCACGCUUCUUACUACG 5’

query=JS16_t0584869, target=FGSG_07008, score=4, range=972-989, strand=1

target  5’ CUCUGCGAAUAAUGAUGC 3’
           :  :::::: ::::::::
query   3’ GCCACGCUUCUUACUACG 5’

query=JS16_t0584869, target=FGSG_08431, score=4, range=753-770, strand=1

target  5’ CUGUUCGAAGAAUGAUUC 3’
           : :: :::::::::: :
query   3’ GCCACGCUUCUUACUACG 5’

query=JS16_t0584869, target=FGSG_07465, score=4, range=124-140, strand=1

target  5’ CGG-GCGAAGAAGGAUGG 3’
           ::: :::::::: ::::
query   3’ GCCACGCUUCUUACUACG 5’

query=JS16_t0584869, target=FGSG_07773, score=4, range=2539-2555, strand=1

target  5’ UGGGGC-AAGAAUGAUGU 3’
           .:: :: ::::::::::.
query   3’ GCCACGCUUCUUACUACG 5’

query=JS16_t0584869, target=FGSG_06149, score=4, range=1061-1079, strand=1

target  5’ CGGUGCAGAAGUAUGAUGC 3’
           :::::: :::: :::::::
query   3’ GCCACG-CUUCUUACUACG 5’

query=JS16_t0584869, target=FGSG_07112, score=4, range=46-63, strand=-1

target  5’ UGGUGUGAAGAAGGAUGU 3’
           .::::.:::::: ::::.
query   3’ GCCACGCUUCUUACUACG 5’

query=JS16_t0584869, target=FGSG_06364, score=4, range=167-184, strand=-1

target  5’ GGGUGCGAAGAGUAAUGC 3’
            ::::::::::.: ::::
query   3’ GCCACGCUUCUUACUACG 5’

query=JS16_t0584869, target=FGSG_09423, score=4, range=512-529, strand=-1

target  5’ CGGUGAAAAGAAUGAUGC 3’
           :::::  :::::::::::
query   3’ GCCACGCUUCUUACUACG 5’

query=JS16_t0584869, target=FGSG_09409, score=4, range=471-488, strand=-1

target  5’ UGGGACGAGGAAUGAUGU 3’
           .::  :::.::::::::.
query   3’ GCCACGCUUCUUACUACG 5’

query=JS16_t0584869, target=FGSG_08909, score=4, range=577-593, strand=-1

target  5’ CGGUUC-AAGAAUGGUGC 3’
           :::: : :::::::.:::
query   3’ GCCACGCUUCUUACUACG 5’

query=JS16_t0584869, target=FGSG_12344, score=4, range=191-207, strand=-1
```

```
query=JS16_t0335591, target=FGSG_01895, score=3, range=212-230, strand=-1

target  5’ UGAGACUUGACGUAAGCCU 3’
           :: ::::::::: ::::::
query   3’ ACACUGAACUGCUUUCGGA 5’

query=JS16_t0335591, target=FGSG_00166, score=3, range=113-131, strand=-1

target  5’ UGAGAAUUGACGGAAGCCU 3’
           :: :: ::::::.::::::
query   3’ ACACUGAACUGCUUUCGGA 5’

query=JS16_t0335591, target=FGSG_07214, score=3, range=1370-1387, strand=-1

target  5’ AGU-ACUUGGCGAAAGCCU 3’
            :: :::::.:::::::::
query   3’ ACACUGAACUGCUUUCGGA 5’

query=JS16_t0335591, target=FGSG_01672, score=4, range=861-878, strand=1

target  5’ UGUG-CUUGAUGAGAGCUU 3’
           :::: :::::.::.:::.:
query   3’ ACACUGAACUGCUUUCGGA 5’

query=JS16_t0335591, target=FGSG_07193, score=4, range=1119-1136, strand=1

target  5’ UGU-ACUUGGUGAAAGUCU 3’
           ::: :::::..:::::.::
query   3’ ACACUGAACUGCUUUCGGA 5’

query=JS16_t0335591, target=FGSG_04008, score=4, range=289-308, strand=1

target  5’ UGUGAGCUUGCCGAAGGCCU 3’
           ::::: :::: ::::.::::
query   3’ ACACU-GAACUGCUUUCGGA 5’

query=JS16_t0335591, target=FGSG_03245, score=4, range=2527-2545, strand=1

target  5’ GGUGACUGGACGAAAGCCA 3’
            :::::: ::::::::::
query   3’ ACACUGAACUGCUUUCGGA 5’

query=JS16_t0335591, target=FGSG_06502, score=4, range=199-216, strand=1

target  5’ GGUGACUUGAC-AAAGUCU 3’
            :::::::::: ::::.::
query   3’ ACACUGAACUGCUUUCGGA 5’

query=JS16_t0335591, target=FGSG_07821, score=4, range=4306-4323, strand=1

target  5’ GGUGACUUGAC-AAAGCCC 3’
            :::::::::: ::::::
query   3’ ACACUGAACUGCUUUCGGA 5’

query=JS16_t0335591, target=FGSG_03282, score=4, range=60-79, strand=1

target  5’ UUUGAUCUUGACCAAAGCCU 3’
           : ::: :::::: :::::::
query   3’ ACACU-GAACUGCUUUCGGA 5’

query=JS16_t0335591, target=FGSG_04948, score=4, range=4429-4448, strand=1

target  5’ UAUGAAGUUGACGAAAGCCA 3’
           : :: : ::::::::::::
query   3’ ACAC-UGAACUGCUUUCGGA 5’

query=JS16_t0335591, target=FGSG_11192, score=4, range=752-770, strand=-1

target  5’ AGUGACUCGAUGAAAGCCU 3’
            :::::: ::.::::::::
query   3’ ACACUGAACUGCUUUCGGA 5’

query=JS16_t0335591, target=FGSG_09543, score=4, range=1043-1061, strand=-1

target  5’ GGUGACUUGGCAAAAGCCU 3’
            ::::::::.: :::::::
query   3’ ACACUGAACUGCUUUCGGA 5’

query=JS16_t0335591, target=FGSG_07300, score=4, range=111-129, strand=-1

target  5’ UGGGACUUGAAGAGAGCCU 3’
           :: ::::::: ::.:::::
query   3’ ACACUGAACUGCUUUCGGA 5’

query=JS16_t0335591, target=FGSG_03398, score=4, range=872-890, strand=-1

target  5’ UGCGACUUGGCCAAAGCCU 3’
           :: ::::::.: :::::::
query   3’ ACACUGAACUGCUUUCGGA 5’

query=JS16_t0335591, target=FGSG_08140, score=4, range=602-621, strand=-1

target  5’ GGGUACGAAGAA-GAUGC 3’
            ::: ::::::: :::::
query   3’ GCCACGCUUCUUACUACG 5’

query=JS16_t1402187, target=FGSG_01312, score=3.5, range=4117-4141, strand=1

target  5’ UCGAGUA-ACUCUUCCAGUCUGUCGC 3’
           .:::::: :::::: :::::::::::
query   3’ GGCUCAUCUGAGAAUGUCAGACAGCG 5’

No results for JS16_t2298310
No results for JS16_t2298317
query=JS16_t3194436, target=FGSG_11597, score=2.5, range=380-402, strand=1

target  5’ AGGGCGCCAACAUUGAUUUGGAC 3’
           :::::. ::::::.:::::::::
query   3’ UCCCGUAGUUGUAGCUAAACCUG 5’

query=JS16_t3194436, target=FGSG_09120, score=4, range=805-827, strand=-1

target  5’ AGUGCAUUGACCUCGAUUUGGAC 3’
           :: ::::..:: :::::::::::
query   3’ UCCCGUAGUUGUAGCUAAACCUG 5’

query=JS16_t3194436, target=FGSG_06557, score=4, range=916-938, strand=-1

target  5’ AGGGGAUCAACAUCGAUGUGGAA 3’
           :::: ::::::::::: ::::
query   3’ UCCCGUAGUUGUAGCUAAACCUG 5’

No results for JS16_t1402246
No results for JS16_t2298394
No results for JS16_t0220406
No results for JS16_t4091373
query=JS16_t0229098, target=FGSG_06089, score=4, range=2749-2773, strand=-1

target  5’ CAUCGGCGUACUCCUUGUAAGCAAG 3’
           :  :::::::: ::.::::::::::
query   3’ GCUGCCGCAUGUGGGACAUUCGUUC 5’

query=JS16_t0396515, target=FGSG_03202, score=4, range=2926-2946, strand=1

target  5’ GGUCUCGAUGGUGUUCAGUGG 3’
           .::: :: :::::::::.::.
query   3’ UCAGCGC-ACCACAAGUUACU 5’

query=JS16_t0396515, target=FGSG_04703, score=4, range=5-23, strand=1

target  5’ AGUC-UGUGGUGUUCAAAGG 3’
           :::: .::::::::::: :.
query   3’ UCAGCGCACCACAAGUUACU 5’

query=JS16_t4091416, target=FGSG_02846, score=3, range=85-103, strand=-1

target  5’ UUUGCUGCAGGGUUGCUUC 3’
           :::::::::::::..:.::
query   3’ AAACGACGUCCCAGUGGAG 5’

query=JS16_t4091416, target=FGSG_09948, score=4, range=30-48, strand=1

target  5’ GUUUCUGCAGCGUCACCUC 3’
            :: :::::: ::::::::
query   3’ AAACGACGUCCCAGUGGAG 5’

query=JS16_t4091416, target=FGSG_05973, score=4, range=1660-1677, strand=-1

target  5’ UUUG-GGCAUGGUCACCUC 3’
           ::::  ::: :::::::::
query   3’ AAACGACGUCCCAGUGGAG 5’

query=JS16_t4091416, target=FGSG_01112, score=4, range=653-670, strand=-1

target  5’ UUUGCUG-AGGGACACCUC 3’
           ::::::: :::: ::::::
query   3’ AAACGACGUCCCAGUGGAG 5’

query=JS16_t0307879, target=FGSG_07127, score=3, range=1793-1811, strand=1

target  5’ UAUUCCCAGGCAAAGAGAU 3’
           :    ::::::::::::::
query   3’ ACUUGGGUCCGUUUCUCUA 5’

query=JS16_t0307879, target=FGSG_11098, score=3, range=1055-1073, strand=-1

target  5’ UCAAGCCAGGUAAAGAGAU 3’
           : :: :::::.::::::::
query   3’ ACUUGGGUCCGUUUCUCUA 5’

query=JS16_t0307879, target=FGSG_05432, score=3.5, range=2207-2225, strand=1

target  5’ UCAAUCUAGGCAAAGGGAU 3’
           : ::.:.::::::::.:::
```

```
target  5’ UGUUGUCUUGAUGAAAGUCU 3’
           :: :: :::::.:::::.::
query   3’ AC-ACUGAACUGCUUUCGGA 5’

query=JS16_t0335591, target=FGSG_06289, score=4, range=383-400, strand=-1

target  5’ CGU-ACUUGGUGAAAGCCU 3’
            :: :::::..::::::::
query   3’ ACACUGAACUGCUUUCGGA 5’

query=JS16_t0335591, target=FGSG_10616, score=4, range=1685-1702, strand=-1

target  5’ UGU-ACUUGAUGAAUGCCU 3’
           ::: ::::::.::: ::::
query   3’ ACACUGAACUGCUUUCGGA 5’

query=JS16_t0335591, target=FGSG_09454, score=4, range=1907-1924, strand=-1

target  5’ UGU-ACUUGACGAAGUCCU 3’
           ::: ::::::::::. :::
query   3’ ACACUGAACUGCUUUCGGA 5’

query=JS16_t0335591, target=FGSG_04320, score=4, range=443-462, strand=-1

target  5’ AGGGAUCUUGACGAGAGCCU 3’
            : :: ::::::::.:::::
query   3’ ACACU-GAACUGCUUUCGGA 5’

query=JS16_t0335591, target=FGSG_01970, score=4, range=777-794, strand=-1

target  5’ UGGGACUUGAC-AAAGUCU 3’
           :: :::::::: ::::.::
query   3’ ACACUGAACUGCUUUCGGA 5’

query=JS16_t0335591, target=FGSG_00014, score=4, range=773-790, strand=-1

target  5’ GCUGA-UUGAUGAAAGCCU 3’
             ::: ::::.::::::::
query   3’ ACACUGAACUGCUUUCGGA 5’

query=JS16_t0335591, target=FGSG_05694, score=4, range=362-381, strand=-1

target  5’ UAUGAGCUUGACGACAGCCU 3’
           : ::: :::::::: :::::
query   3’ ACACU-GAACUGCUUUCGGA 5’

query=JS16_t0335591, target=FGSG_01589, score=4, range=1803-1820, strand=-1

target  5’ UG-GACUGGACGAAAGCCC 3’
           :: :::: ::::::::::
query   3’ ACACUGAACUGCUUUCGGA 5’

query=JS16_t0335591, target=FGSG_03259, score=4, range=899-916, strand=-1

target  5’ UGU-UCUUGACGAAAGCAU 3’
           :::  :::::::::::: :
query   3’ ACACUGAACUGCUUUCGGA 5’

No results for JS16_t1338459
No results for JS16_t4027712
No results for JS16_t4027745
No results for JS16_t3131207
query=JS16_t0685985, target=FGSG_09117, score=3.5, range=227-247, strand=-1

target  5’ UGAAAAGAGAGGUGACGAAUU 3’
           :.  : :::::::::::::::
query   3’ AUGAUGCUCUCCACUGCUUAA 5’

query=JS16_t0685985, target=FGSG_12814, score=4, range=531-550, strand=-1

target  5’ GACCAC-CGAGGUGACGAAUU 3’
            :: ::  :::::::::::::
query   3’ AUGAUGCUCUCCACUGCUUAA 5’

No results for JS16_t3131253
No results for JS16_t0795297
No results for JS16_t0685992
query=JS16_t0468259, target=FGSG_06542, score=2.5, range=2056-2076, strand=1

target  5’ GGUCGACGAGAUCGCGGAGGU 3’
           ::. : :::::::::::::::
query   3’ CCGUCGGCUCUAGCGCCUCCA 5’

query=JS16_t0468259, target=FGSG_00554, score=3, range=451-471, strand=1

target  5’ GGUGGCCGAGGUCGUGGAGGU 3’
           ::..::::::.:::.::::::
query   3’ CCGUCGGCUCUAGCGCCUCCA 5’

query=JS16_t0468259, target=FGSG_01291, score=3.5, range=641-661, strand=-1

target  5’ UGCACUCGAGGUCGCGGAGGU 3’
query   3’ ACUUGGGUCCGUUUCUCUA 5’

query=JS16_t0307879, target=FGSG_04279, score=3.5, range=4619-4637, strand=1

target  5’ AGGACGCAGGCGAAGAGAU 3’
             :.:: :::::.::::::::
query   3’ ACUUGGGUCCGUUUCUCUA 5’

query=JS16_t0307879, target=FGSG_10461, score=3.5, range=1403-1421, strand=1

target  5’ UGGAACCGGGCAAAGAGAG 3’
           ::.: ::.::::::::::
query   3’ ACUUGGGUCCGUUUCUCUA 5’

query=JS16_t0307879, target=FGSG_03162, score=3.5, range=577-594, strand=1

target  5’ GGAAUCCAGGCAAA-AGAU 3’
            :::.::::::::: ::::
query   3’ ACUUGGGUCCGUUUCUCUA 5’

query=JS16_t0307879, target=FGSG_03388, score=3.5, range=4526-4544, strand=-1

target  5’ CCAAUCCAGGUAAAGAGAU 3’
             ::.:::::.::::::::
query   3’ ACUUGGGUCCGUUUCUCUA 5’

query=JS16_t0307879, target=FGSG_06701, score=3.5, range=481-498, strand=-1

target  5’ UGAA-UCAGGCAGAGAGAA 3’
           :::: .::::::.:::::
query   3’ ACUUGGGUCCGUUUCUCUA 5’

query=JS16_t0307879, target=FGSG_05283, score=4, range=959-977, strand=1

target  5’ UGAACGCAGCCAAGGAGAU 3’
           ::::: ::: :::.:::::
query   3’ ACUUGGGUCCGUUUCUCUA 5’

query=JS16_t0307879, target=FGSG_13829, score=4, range=323-340, strand=1

target  5’ GGAACCCAGGC-AAGGGAU 3’
            :::::::::: :::.:::
query   3’ ACUUGGGUCCGUUUCUCUA 5’

query=JS16_t0307879, target=FGSG_09391, score=4, range=1964-1982, strand=1

target  5’ GCGAUGCAGGCAAAGAGAU 3’
             .:.  ::::::::::::
query   3’ ACUUGGGUCCGUUUCUCUA 5’

query=JS16_t0307879, target=FGSG_06231, score=4, range=1258-1277, strand=1

target  5’ CGACAGCUAGGCAAAGAGAU 3’
            :: : :.:::::::::::
query   3’ ACU-UGGGUCCGUUUCUCUA 5’

query=JS16_t0307879, target=FGSG_00899, score=4, range=626-643, strand=-1

target  5’ UGAGUCC-GGCGAAGAGAU 3’
           :::..:: :::.:::::::
query   3’ ACUUGGGUCCGUUUCUCUA 5’

query=JS16_t0307879, target=FGSG_00775, score=4, range=395-413, strand=-1

target  5’ UCAACCCAGGCAAAGACGU 3’
           : ::::::::::::: .:
query   3’ ACUUGGGUCCGUUUCUCUA 5’

query=JS16_t0307879, target=FGSG_03708, score=4, range=743-760, strand=-1

target  5’ CGAA-CCAGGCAAAGGGGU 3’
            ::: ::::::::::.:.:
query   3’ ACUUGGGUCCGUUUCUCUA 5’

query=JS16_t0307879, target=FGSG_04351, score=4, range=1673-1690, strand=-1

target  5’ UGAGAUC-GGCAAAGAGAU 3’
           :::. .: :::::::::::
query   3’ ACUUGGGUCCGUUUCUCUA 5’

No results for JS16_t2298553
query=JS16_t0693596, target=FGSG_00138, score=2, range=708-726, strand=-1

target  5’ GCCCCAGAUCUUUUCAUCA 3’
           :: ::::::::.:::::::
query   3’ CGAGGUCUAGAGAAGUAGU 5’

query=JS16_t0693596, target=FGSG_03452, score=2.5, range=136-154, strand=1

target  5’ AUUCCCGAUCUCUUCAUCA 3’
            .::: :::::::::::::
query   3’ CGAGGUCUAGAGAAGUAGU 5’
```

```
           ::: .::::.::::::::::
query   3' CCGUCGGCUCUAGCGCCUCCA 5'

query=JS16_t0468259, target=FGSG_13122, score=4, range=252-273, strand=1

target  5' UGUGGCCGAGAUCGCGGAGAGU 3'
           :..:::::::::::::::: ::
query   3' CCGUCGGCUCUAGCGCCUC-CA 5'

query=JS16_t0468259, target=FGSG_07365, score=4, range=308-328, strand=-1

target  5' AGUGGACGAGAUCGCGGAGGA 3'
           :..: ::::::::::::::
query   3' CCGUCGGCUCUAGCGCCUCCA 5'

query=JS16_t0468259, target=FGSG_07366, score=4, range=32-53, strand=-1

target  5' CGCGGUCGAAGAUCGCGGAGGA 3'
           ::.:.:: ::::::::::::
query   3' CCGUCGGC-UCUAGCGCCUCCA 5'

query=JS16_t3131294, target=FGSG_07303, score=4, range=713-735, strand=-1

target  5' CGUUG-CAUCGACGAAUUGGCCCG 3'
           : ::: ::.:.::::::::::::
query   3' GGAACAGUGGUUGCUUAACCGGGA 5'

No results for JS16_t4027893
No results for JS16_t0268782
No results for JS16_t2235233
No results for JS16_t1338724
No results for JS16_t0795301
No results for JS16_t3131390
No results for JS16_t0176388
No results for JS16_t2235347
No results for JS16_t1338851
No results for JS16_t3131501
No results for JS16_t1338857
No results for JS16_t3131530
query=JS16_t2235429, target=FGSG_06199, score=3.5, range=864-882, strand=1

target  5' CGAGCUGGAUGAGA-AGUGC 3'
           ::::::.:::::.: :::::
query   3' GCUCGAUCUACUUUAUCACG 5'

query=JS16_t2235429, target=FGSG_09600, score=4, range=736-754, strand=1

target  5' CGGGAU-GAUGAAAUGGUGU 3'
           ::.: : ::::::::.:::.
query   3' GCUCGAUCUACUUUAUCACG 5'

query=JS16_t2235429, target=FGSG_10830, score=4, range=255-274, strand=-1

target  5' CGGACUGGAUGAAAUAGUCC 3'
           ::. ::.:::::::::: :
query   3' GCUCGAUCUACUUUAUCACG 5'

query=JS16_t2235429, target=FGSG_05226, score=4, range=177-195, strand=-1

target  5' CGAG-UCGAUGAGGUAGUGC 3'
           :::: : :::::..::::::
query   3' GCUCGAUCUACUUUAUCACG 5'

No results for JS16_t4028134
query=JS16_t2235457, target=FGSG_00108, score=4, range=818-837, strand=1

target  5' AUUGCAUCUGGCUCGGUGCA 3'
           ::: :::::.:::::::: :
query   3' UAAAGUAGAUCGAGCCACUU 5'

query=JS16_t2235457, target=FGSG_07926, score=4, range=1391-1409, strand=-1

target  5' AUGUCA-CUAGCUCGGAGAA 3'
           :: ::: ::::::::: :::
query   3' UAAAGUAGAUCGAGCCACUU 5'

query=JS16_t0577303, target=FGSG_01584, score=3, range=384-401, strand=-1

target  5' CCAGCGAUGAGCAUCGCG 3'
           : :::.::::.:::::::
query   3' GAUCGUUACUUGUAGCGC 5'

query=JS16_t0577303, target=FGSG_02324, score=3, range=1204-1221, strand=-1

target  5' UUGUCGAUGAACAUCGCG 3'
           .:. :.::::::::::::
query   3' GAUCGUUACUUGUAGCGC 5'

query=JS16_t0577303, target=FGSG_09352, score=3.5, range=1013-1030, strand=1

target  5' UUCUCAAUGAACAUCGUG 3'
           .:  ::::::::::::.:

query=JS16_t0693596, target=FGSG_04251, score=2.5, range=798-815, strand=1

target  5' GCUUCAG-UCUCUUCAUCA 3'
           :::.::: :::::::::::
query   3' CGAGGUCUAGAGAAGUAGU 5'

query=JS16_t0693596, target=FGSG_04505, score=2.5, range=354-373, strand=-1

target  5' GUUCCAUGAUCUCUUCGUCA 3'
           :.:::: ::::::::::.:::
query   3' CGAGGU-CUAGAGAAGUAGU 5'

query=JS16_t0693596, target=FGSG_03443, score=3, range=850-868, strand=1

target  5' GUGCUAGAUUUCUUCAUCA 3'
           :.  :.::::.:::::::::
query   3' CGAGGUCUAGAGAAGUAGU 5'

query=JS16_t0693596, target=FGSG_03726, score=3, range=757-775, strand=1

target  5' GGUACAGGUCUCUUCAUCA 3'
           : :  :::.:::::::::::
query   3' CGAGGUCUAGAGAAGUAGU 5'

query=JS16_t0693596, target=FGSG_07243, score=3, range=675-694, strand=-1

target  5' GUUCCAUGAUCUCUUCGUCG 3'
           :.:::: ::::::::::.::.
query   3' CGAGGU-CUAGAGAAGUAGU 5'

query=JS16_t0693596, target=FGSG_14016, score=3, range=1776-1795, strand=-1

target  5' GCUCCAAGGUCUCUUCAUCC 3'
           ::::: ::.::::::::::
query   3' CGAGG-UCUAGAGAAGUAGU 5'

query=JS16_t0693596, target=FGSG_10523, score=3.5, range=6556-6574, strand=1

target  5' UUUCCAGAUCUUUUCGUCA 3'
            .:::::::::.:::.:::
query   3' CGAGGUCUAGAGAAGUAGU 5'

query=JS16_t0693596, target=FGSG_03180, score=3.5, range=375-394, strand=1

target  5' UCUCCGCGGUCUCUUCAUCA 3'
            ::::.  :.:::::::::::
query   3' CGAGGU-CUAGAGAAGUAGU 5'

query=JS16_t0693596, target=FGSG_05504, score=3.5, range=2437-2455, strand=1

target  5' GUGCUCGAUCUCUUCAUCG 3'
           :.  :.  ::::::::::::.
query   3' CGAGGUCUAGAGAAGUAGU 5'

query=JS16_t0693596, target=FGSG_01638, score=3.5, range=1659-1678, strand=1

target  5' GUUCCACGCUCUCUUCAUCA 3'
           :.:::: : :::::::::::
query   3' CGAGGU-CUAGAGAAGUAGU 5'

query=JS16_t0693596, target=FGSG_04351, score=3.5, range=1692-1710, strand=-1

target  5' UCUCCUGAUUUCUUCAUCG 3'
            :::: :::.::::::::.
query   3' CGAGGUCUAGAGAAGUAGU 5'

query=JS16_t0693596, target=FGSG_01258, score=3.5, range=912-929, strand=-1

target  5' UCUUC-GAUCUUUUCAUCA 3'
            ::.: :::::.:::::::
query   3' CGAGGUCUAGAGAAGUAGU 5'

query=JS16_t0693596, target=FGSG_04532, score=3.5, range=3-20, strand=-1

target  5' GCUUC-GAUUUCUUCAUCC 3'
           :::.: :::.::::::::
query   3' CGAGGUCUAGAGAAGUAGU 5'

query=JS16_t0693596, target=FGSG_06641, score=3.5, range=980-999, strand=-1

target  5' ACUGUCUGAUCUCUUCAUCA 3'
            :: .: :::::::::::::
query   3' CGA-GGUCUAGAGAAGUAGU 5'

query=JS16_t0693596, target=FGSG_10587, score=4, range=1502-1520, strand=1

target  5' UUUCCGGAUCUCUUCAUGA 3'
            .:::.:::::::::::: :
query   3' CGAGGUCUAGAGAAGUAGU 5'

query=JS16_t0693596, target=FGSG_09081, score=4, range=331-349, strand=1
```

```
query   3’ GAUCGUUACUUGUAGCGC 5’

query=JS16_t0577303, target=FGSG_02776, score=4, range=476-493, strand=1

target  5’ UCGGCAAUGGGCAUCGCG 3’
           . .::::::..:::::::
query   3’ GAUCGUUACUUGUAGCGC 5’

query=JS16_t0577303, target=FGSG_10798, score=4, range=688-706, strand=1

target  5’ CUAGGCAAUGGAUAUCGCA 3’
           ::: ::::::.:.:::::
query   3’ GAU-CGUUACUUGUAGCGC 5’

query=JS16_t0577303, target=FGSG_11676, score=4, range=733-751, strand=1

target  5’ CUAGCAAUCAACAUCGACG 3’
           :::::::: ::::::: ::
query   3’ GAUCGUUACUUGUAGC-GC 5’

query=JS16_t0577303, target=FGSG_05579, score=4, range=380-397, strand=1

target  5’ CUCACGAUGAACGUCGCG 3’
           ::  :.::::::.:::::
query   3’ GAUCGUUACUUGUAGCGC 5’

query=JS16_t0577303, target=FGSG_09076, score=4, range=4931-4948, strand=1

target  5’ GUUGCAAUGAGCAUCGCA 3’
            : :::::::.::::::
query   3’ GAUCGUUACUUGUAGCGC 5’

query=JS16_t0577303, target=FGSG_05899, score=4, range=554-571, strand=1

target  5’ CUAAGAAUGAACGUCGCA 3’
           :::  :::::::.::::
query   3’ GAUCGUUACUUGUAGCGC 5’

query=JS16_t0577303, target=FGSG_11399, score=4, range=588-606, strand=1

target  5’ CGAGUCAAUGAACAUCGUU 3’
           : :: ::::::::::::.
query   3’ GAUC-GUUACUUGUAGCGC 5’

query=JS16_t0577303, target=FGSG_10617, score=4, range=2237-2254, strand=1

target  5’ CGAUCAACGAACAUCGCG 3’
           : : ::: ::::::::::
query   3’ GAUCGUUACUUGUAGCGC 5’

query=JS16_t0577303, target=FGSG_03522, score=4, range=173-190, strand=1

target  5’ CGAGGAAGGAACAUCGCG 3’
           : :: :: ::::::::::
query   3’ GAUCGUUACUUGUAGCGC 5’

query=JS16_t0577303, target=FGSG_02094, score=4, range=742-760, strand=1

target  5’ CUUGCUAUGAACAUCCGCG 3’
           :: :: :::::::: ::::
query   3’ GAUCGUUACUUGUA-GCGC 5’

query=JS16_t0577303, target=FGSG_12774, score=4, range=120-138, strand=1

target  5’ CGAGACAAGGAACAUCGCG 3’
           : :: ::: ::::::::::
query   3’ GAUC-GUUACUUGUAGCGC 5’

query=JS16_t0577303, target=FGSG_04678, score=4, range=555-571, strand=1

target  5’ CU-GCAAUGAACCUCGCU 3’
           :: ::::::::: ::::
query   3’ GAUCGUUACUUGUAGCGC 5’

query=JS16_t0577303, target=FGSG_07676, score=4, range=86-102, strand=1

target  5’ CUA-CAAAGAACAUCGCC 3’
           ::: ::: :::::::::
query   3’ GAUCGUUACUUGUAGCGC 5’

query=JS16_t0577303, target=FGSG_03170, score=4, range=1785-1802, strand=-1

target  5’ CUAGCAAUGAAUAGUGCG 3’
           :::::::::::.: .:::
query   3’ GAUCGUUACUUGUAGCGC 5’

query=JS16_t0577303, target=FGSG_09781, score=4, range=887-904, strand=-1

target  5’ CUUGCAAUGGAUAUCGUG 3’
           :: ::::::.:.::::.:
query   3’ GAUCGUUACUUGUAGCGC 5’

target  5’ AUUCUCGGUCUCUUCAUCA 3’
            .::. :.:::::::::::
query   3’ CGAGGUCUAGAGAAGUAGU 5’

query=JS16_t0693596, target=FGSG_01342, score=4, range=147-165, strand=1

target  5’ ACACCGGAUCUUUUCAUCG 3’
            : ::.:::::.::::::.
query   3’ CGAGGUCUAGAGAAGUAGU 5’

query=JS16_t0693596, target=FGSG_10506, score=4, range=297-316, strand=1

target  5’ GCUGCAGAUCUUUUUCAUCA 3’
           ::: :::::: :.:::::::
query   3’ CGAGGUCUAG-AGAAGUAGU 5’

query=JS16_t0693596, target=FGSG_09329, score=4, range=2064-2083, strand=1

target  5’ CCUCGAGAGUCUCUUCAUCA 3’
            ::: ::: :::::::::::
query   3’ CGAGGUCU-AGAGAAGUAGU 5’

query=JS16_t0693596, target=FGSG_02229, score=4, range=143-160, strand=1

target  5’ GUACC-GGUCUCUUCAUCG 3’
           :. :: :.::::::::::.
query   3’ CGAGGUCUAGAGAAGUAGU 5’

query=JS16_t0693596, target=FGSG_06375, score=4, range=628-646, strand=1

target  5’ GCGACCGAUUUCUUCAUCA 3’
           ::  : :::.:::::::::
query   3’ CGAGGUCUAGAGAAGUAGU 5’

query=JS16_t0693596, target=FGSG_11794, score=4, range=909-928, strand=1

target  5’ CCUGCGAGAUCUCUUCAUCC 3’
            :: : :::::::::::::
query   3’ CGA-GGUCUAGAGAAGUAGU 5’

query=JS16_t0693596, target=FGSG_01642, score=4, range=594-611, strand=1

target  5’ UCUCC-GAUCUCUCCAUCA 3’
            :::: ::::::: :::::
query   3’ CGAGGUCUAGAGAAGUAGU 5’

query=JS16_t0693596, target=FGSG_11590, score=4, range=145-162, strand=1

target  5’ AAUCCAGAU-UCUUCAUCA 3’
             ::::::: :::::::::
query   3’ CGAGGUCUAGAGAAGUAGU 5’

query=JS16_t0693596, target=FGSG_05557, score=4, range=927-945, strand=-1

target  5’ AUACUGGAUCUCUUCAUCG 3’
            . :..::::::::::::.
query   3’ CGAGGUCUAGAGAAGUAGU 5’

query=JS16_t0693596, target=FGSG_07916, score=4, range=1521-1539, strand=-1

target  5’ GCUCAAGCUCUCUUCGUCA 3’
           :::: :: :::::::.:::
query   3’ CGAGGUCUAGAGAAGUAGU 5’

query=JS16_t0693596, target=FGSG_00413, score=4, range=456-475, strand=-1

target  5’ GCUCCAGGAACUCUUCGUCA 3’
           :::::: :: ::::::.:::
query   3’ CGAGGU-CUAGAGAAGUAGU 5’

query=JS16_t0693596, target=FGSG_01666, score=4, range=297-315, strand=-1

target  5’ GCGCAGGGUCUCUUCAUCG 3’
           :: : .:.::::::::::.
query   3’ CGAGGUCUAGAGAAGUAGU 5’

query=JS16_t0693596, target=FGSG_03502, score=4, range=156-174, strand=-1

target  5’ CUUCUUGAUCUCUUCAUCU 3’
            .::. ::::::::::::
query   3’ CGAGGUCUAGAGAAGUAGU 5’

query=JS16_t0693596, target=FGSG_11897, score=4, range=2022-2040, strand=-1

target  5’ CCACCCGAUCUCUUCGUCA 3’
            : :: :::::::::.:::
query   3’ CGAGGUCUAGAGAAGUAGU 5’

query=JS16_t0693596, target=FGSG_12684, score=4, range=136-154, strand=-1

target  5’ GCUCACGAUCUCUUCGUCU 3’
```

```
query=JS16_t0577303, target=FGSG_11098, score=4, range=126-144, strand=-1

target  5’ CUCAGCAAUGGGCGUCGCG 3’
           :: :::::::..:.:::::
query   3’ GA-UCGUUACUUGUAGCGC 5’

query=JS16_t0577303, target=FGSG_13324, score=4, range=46-63, strand=-1

target  5’ AAAGCGAUGAAUAUCGCG 3’
             :::.:::::.::::::
query   3’ GAUCGUUACUUGUAGCGC 5’

query=JS16_t0577303, target=FGSG_04019, score=4, range=59-75, strand=-1

target  5’ UUGGCAAUGGACAU-GCG 3’
           .:.:::::::.:::: :::
query   3’ GAUCGUUACUUGUAGCGC 5’

query=JS16_t0577303, target=FGSG_03360, score=4, range=1605-1623, strand=-1

target  5’ CUCAGCAAUGAACAUCGAA 3’
           :: ::::::::::::::
query   3’ GA-UCGUUACUUGUAGCGC 5’

query=JS16_t0577303, target=FGSG_04247, score=4, range=130-147, strand=-1

target  5’ CCAGAAAUGGACAUCGCU 3’
           : :: ::::.:::::::
query   3’ GAUCGUUACUUGUAGCGC 5’

query=JS16_t0577303, target=FGSG_06426, score=4, range=1558-1576, strand=-1

target  5’ CUCGGAAUUGAACAUCGCG 3’
           :: : :: :::::::::::
query   3’ GAUCGUU-ACUUGUAGCGC 5’

query=JS16_t0577303, target=FGSG_13705, score=4, range=438-454, strand=-1

target  5’ GUAGAAAUGAACAU-GCG 3’
            ::: ::::::::: :::
query   3’ GAUCGUUACUUGUAGCGC 5’

query=JS16_t0577303, target=FGSG_06582, score=4, range=3115-3131, strand=-1

target  5’ CUCUCAA-GAACAUCGCG 3’
           ::  ::: ::::::::::
query   3’ GAUCGUUACUUGUAGCGC 5’

No results for JS16_t1338944
query=JS16_t0268787, target=FGSG_02988, score=2, range=1071-1088, strand=1

target  5’ GGCUGAGGUCUUGGAUGA 3’
             ::::::::::::::::
query   3’ AGGACUCCAGAACCUACU 5’

query=JS16_t0268787, target=FGSG_03584, score=3, range=380-398, strand=1

target  5’ UCUUGAGGUCUUGGGAUGG 3’
           ::.::::::::: :::::.
query   3’ AGGACUCCAGAA-CCUACU 5’

query=JS16_t0268787, target=FGSG_03435, score=3, range=494-512, strand=1

target  5’ ACCGGCAGGUCUUGGAUGA 3’
            :: : :::::::::::::
query   3’ AGGAC-UCCAGAACCUACU 5’

query=JS16_t0268787, target=FGSG_02724, score=3, range=2026-2043, strand=-1

target  5’ UUUUGAUGUCUUGGAUGA 3’
           :..::: ::::::::::
query   3’ AGGACUCCAGAACCUACU 5’

query=JS16_t0268787, target=FGSG_01137, score=3, range=150-167, strand=-1

target  5’ UGCUGAGGUUUUGGGUGA 3’
           : :::::::.::::.:::
query   3’ AGGACUCCAGAACCUACU 5’

query=JS16_t0268787, target=FGSG_12618, score=3, range=42-58, strand=-1

target  5’ UCCUCAGGUCUUGGA-GA 3’
           :::: :::::::::: ::
query   3’ AGGACUCCAGAACCUACU 5’

query=JS16_t0268787, target=FGSG_05606, score=3, range=1033-1049, strand=-1

target  5’ UCCUGAGG-CUUGGGUGA 3’
           :::::::: :::::.:::
query   3’ AGGACUCCAGAACCUACU 5’

query=JS16_t0268787, target=FGSG_03155, score=3.5, range=815-832, strand=1

           ::::  ::::::::::.::
query   3’ CGAGGUCUAGAGAAGUAGU 5’

query=JS16_t0693596, target=FGSG_08481, score=4, range=567-584, strand=-1

target  5’ GCUGCAG-UUUCUUCAUCA 3’
           ::: ::: :.::::::::::
query   3’ CGAGGUCUAGAGAAGUAGU 5’

query=JS16_t0693596, target=FGSG_08089, score=4, range=849-868, strand=-1

target  5’ GCUCAGAGAUCUCUUCAUUU 3’
           ::::  ::::::::::::.
query   3’ CGAG-GUCUAGAGAAGUAGU 5’

query=JS16_t0693596, target=FGSG_08985, score=4, range=2073-2090, strand=-1

target  5’ GCAUCA-AUCUCUUCAUCG 3’
           :: .:: :::::::::::.
query   3’ CGAGGUCUAGAGAAGUAGU 5’

query=JS16_t0693596, target=FGSG_07066, score=4, range=579-597, strand=-1

target  5’ GCUCGCUAUCUCUUCAUCA 3’
           ::::   ::::::::::::
query   3’ CGAGGUCUAGAGAAGUAGU 5’

query=JS16_t0693596, target=FGSG_12783, score=4, range=198-216, strand=-1

target  5’ GACCUUGAUCUCUUCAUCG 3’
           :  :. ::::::::::::.
query   3’ CGAGGUCUAGAGAAGUAGU 5’

query=JS16_t0693596, target=FGSG_00017, score=4, range=669-687, strand=-1

target  5’ GCCAGAGAUCUCUUCGUCA 3’
           ::   ::::::::::.:::
query   3’ CGAGGUCUAGAGAAGUAGU 5’

query=JS16_t0693596, target=FGSG_00396, score=4, range=771-788, strand=-1

target  5’ GC-CCUGAUCUCUUCGUCC 3’
           :: :: ::::::::::.::
query   3’ CGAGGUCUAGAGAAGUAGU 5’

query=JS16_t0693596, target=FGSG_10783, score=4, range=1908-1926, strand=-1

target  5’ GAUAGCGAUCUCUUCAUCA 3’
           : :   :::::::::::::
query   3’ CGAGGUCUAGAGAAGUAGU 5’

query=JS16_t0693596, target=FGSG_01341, score=4, range=1770-1789, strand=-1

target  5’ GCUCCAUCAUCUCUUCAUCC 3’
           ::::::  :::::::::::
query   3’ CGAGGU-CUAGAGAAGUAGU 5’

No results for JS16_t2298564
No results for JS16_t3194669
query=JS16_t3194685, target=FGSG_02544, score=3, range=269-288, strand=-1

target  5’ CUUGAUGGAGGAUUCCA-ACU 3’
           :::::..:::::::::: :::
query   3’ GAACUGUCUCCUAAGGUCUGA 5’

query=JS16_t3194685, target=FGSG_00971, score=3, range=2-22, strand=-1

target  5’ CUUGUGAGAGGAUUCCAGACA 3’
           ::::  ::::::::::::::
query   3’ GAACUGUCUCCUAAGGUCUGA 5’

query=JS16_t3194685, target=FGSG_03393, score=3.5, range=1083-1104, strand=-1

target  5’ UUUGCCAGAAGGAUUCCAGACA 3’
           .::: ::: ::::::::::::
query   3’ GAACUGUC-UCCUAAGGUCUGA 5’

query=JS16_t3194685, target=FGSG_02368, score=4, range=1857-1877, strand=1

target  5’ CUUGACAGAGGGUUCCACAUU 3’
           :::::::::::.::::: :.:
query   3’ GAACUGUCUCCUAAGGUCUGA 5’

query=JS16_t3194685, target=FGSG_09632, score=4, range=652-671, strand=1

target  5’ AUUGA-AAAGGAUUCCAGACG 3’
            :::: : ::::::::::::
query   3’ GAACUGUCUCCUAAGGUCUGA 5’

query=JS16_t3194685, target=FGSG_10912, score=4, range=155-174, strand=-1

target  5’ CUUGUC-GAGGAUUCCAGUCU 3’
```

```
target  5’ UUCUGGGGUCUUUGAUGA 3’
           :.:::.:::::: :::::
query   3’ AGGACUCCAGAACCUACU 5’

query=JS16_t0268787, target=FGSG_03538, score=3.5, range=1155-1172, strand=1

target  5’ UCUUCAGGUCUUGGAAGA 3’
           ::.: :::::::::: ::
query   3’ AGGACUCCAGAACCUACU 5’

query=JS16_t0268787, target=FGSG_11293, score=3.5, range=458-474, strand=1

target  5’ UCCU-ACGUCUUGGAUGG 3’
           :::: : ::::::::::.
query   3’ AGGACUCCAGAACCUACU 5’

query=JS16_t0268787, target=FGSG_09337, score=3.5, range=1545-1562, strand=1

target  5’ UGGUAAGGUCUUGGAUGG 3’
           :  : ::::::::::::.
query   3’ AGGACUCCAGAACCUACU 5’

query=JS16_t0268787, target=FGSG_04898, score=3.5, range=714-730, strand=1

target  5’ UCUUCAGGUC-UGGAUGA 3’
           ::.: ::::: :::::::
query   3’ AGGACUCCAGAACCUACU 5’

query=JS16_t0268787, target=FGSG_13208, score=3.5, range=370-387, strand=-1

target  5’ UGCUGAGGUUUUGGGUGG 3’
           : :::::::.::::.::.
query   3’ AGGACUCCAGAACCUACU 5’

query=JS16_t0268787, target=FGSG_03825, score=3.5, range=829-846, strand=-1

target  5’ CUCUGAGGUCUUGGGUGU 3’
            .::::::::::::.::
query   3’ AGGACUCCAGAACCUACU 5’

query=JS16_t0268787, target=FGSG_05079, score=3.5, range=1751-1768, strand=-1

target  5’ CGUUGAGGUCUUGGAUGU 3’
             .::::::::::::::
query   3’ AGGACUCCAGAACCUACU 5’

query=JS16_t0268787, target=FGSG_06948, score=3.5, range=553-570, strand=-1

target  5’ UCCCGCGGUCUUGGAUGG 3’
           ::: : :::::::::::.
query   3’ AGGACUCCAGAACCUACU 5’

query=JS16_t0268787, target=FGSG_04782, score=3.5, range=137-154, strand=-1

target  5’ UCUCGAGGUCAUGGAUGA 3’
           ::. :::::: :::::::
query   3’ AGGACUCCAGAACCUACU 5’

query=JS16_t0268787, target=FGSG_09753, score=3.5, range=134-151, strand=-1

target  5’ UUGUGAGGGCUUGGAUGA 3’
           :. ::::: :::::::::
query   3’ AGGACUCCAGAACCUACU 5’

query=JS16_t0268787, target=FGSG_11214, score=3.5, range=572-589, strand=-1

target  5’ UGCUGAGGUCUUGGAGGG 3’
           : ::::::::::::: :.
query   3’ AGGACUCCAGAACCUACU 5’

query=JS16_t0268787, target=FGSG_03512, score=3.5, range=1114-1132, strand=-1

target  5’ UUCCGAGGUCUUGCGAUGA 3’
           :.: ::::::::: :::::
query   3’ AGGACUCCAGAAC-CUACU 5’

query=JS16_t0268787, target=FGSG_12470, score=3.5, range=763-780, strand=-1

target  5’ UUCAAAGGUCUUGGGUGA 3’
           :.:  :::::::::.:::
query   3’ AGGACUCCAGAACCUACU 5’

query=JS16_t0268787, target=FGSG_10256, score=3.5, range=452-469, strand=-1

target  5’ CCUUUAGGUCUUGGAUGU 3’
            :.: ::::::::::::
query   3’ AGGACUCCAGAACCUACU 5’

query=JS16_t0268787, target=FGSG_10765, score=3.5, range=913-929, strand=-1

target  5’ UCCUUAGGU-UUGGAUGG 3’
           :::: : ::::::::::: ::
query   3’ GAACUGUCUCCUAAGGUCUGA 5’

No results for JS16_t4091572
No results for JS16_t3194765
query=JS16_t2298684, target=FGSG_00390, score=4, range=72-94, strand=1

target  5’ AGACCAAGAAGCGUCAUGGGAAC 3’
           ::::::::: .::.::::.::::
query   3’ UCUGGUUCUCUGCGGUACUCUUG 5’

query=JS16_t2298684, target=FGSG_02584, score=4, range=366-387, strand=1

target  5’ AGCCCAAG-GACGCCAGGAGAAC 3’
           :: ::::: ::::::: ::::::
query   3’ UCUGGUUCUCUGCGGUACUCUUG 5’

query=JS16_t2298684, target=FGSG_02315, score=4, range=3301-3322, strand=-1

target  5’ AGACC-AUAGCCGCCAUGAGAAC 3’
           ::::: : :: :::::::::::
query   3’ UCUGGUUCUCUGCGGUACUCUUG 5’

No results for JS16_t4091648
No results for JS16_t3194820
No results for JS16_t3194900
query=JS16_t2298788, target=FGSG_07070, score=3.5, range=1045-1062, strand=-1

target  5’ GUUUU-UGGUGUUGGGUUG 3’
           :::.. :.::::::::::.
query   3’ CAAGGUAUCACAACCCAAU 5’

query=JS16_t2298788, target=FGSG_08097, score=3.5, range=2086-2105, strand=-1

target  5’ GAUACAUUAGUGUUGGGUUG 3’
           : : :: ::::::::::::.
query   3’ CAAGGU-AUCACAACCCAAU 5’

query=JS16_t2298788, target=FGSG_07802, score=4, range=36-55, strand=-1

target  5’ GUUGCCCUGGUGUUGGGUUC 3’
           ::: :: :.::::::::::
query   3’ CAA-GGUAUCACAACCCAAU 5’

query=JS16_t0367196, target=FGSG_04919, score=3, range=984-1002, strand=1

target  5’ CUAUGCCGUCGCCACUGGU 3’
            : ::::::::::::.:::
query   3’ CACACGGCAGCGGUGGCCA 5’

query=JS16_t0367196, target=FGSG_03510, score=4, range=758-777, strand=1

target  5’ GUGAUGCCGUCGCCAUUGGC 3’
           ::: :::::::::::..::
query   3’ CAC-ACGGCAGCGGUGGCCA 5’

query=JS16_t0367196, target=FGSG_06780, score=4, range=515-534, strand=1

target  5’ CUGUGCCCGUCGCCGCUGGU 3’
            :::: ::::::::.:.:::
query   3’ CACAC-GGCAGCGGUGGCCA 5’

query=JS16_t0367196, target=FGSG_06048, score=4, range=249-267, strand=1

target  5’ GGGUGCCGCCGCCAUCGGU 3’
           : :::::: :::::.::::
query   3’ CACACGGCAGCGGUGGCCA 5’

query=JS16_t0367196, target=FGSG_10885, score=4, range=1097-1116, strand=1

target  5’ GUGAUGCCAUCGCCACUGGU 3’
           ::: :::: :::::::.:::
query   3’ CAC-ACGGCAGCGGUGGCCA 5’

query=JS16_t0367196, target=FGSG_00209, score=4, range=1382-1400, strand=1

target  5’ AUGUGACGUCGCCAUCGGA 3’
            :::: :::::::::.:::
query   3’ CACACGGCAGCGGUGGCCA 5’

query=JS16_t0367196, target=FGSG_02700, score=4, range=1249-1266, strand=1

target  5’ GU-UGCCGUUGCCGCCGGC 3’
           :: ::::::.:::.::::
query   3’ CACACGGCAGCGGUGGCCA 5’

query=JS16_t0367196, target=FGSG_08173, score=4, range=1519-1536, strand=1

target  5’ GUCU-CUGUCGCCGCCGGU 3’
           :: : :.::::::.:::::
query   3’ CACACGGCAGCGGUGGCCA 5’
```

```
           :::: :::: :::::::.
query   3' AGGACUCCAGAACCUACU 5'

query=JS16_t0268787, target=FGSG_09509, score=4, range=1164-1181, strand=1

target  5' AUUUGAGGACUUGGAUGA 3'
            ..::::: :::::::::
query   3' AGGACUCCAGAACCUACU 5'

query=JS16_t0268787, target=FGSG_00735, score=4, range=75-92, strand=1

target  5' UUUUGAUGUCUUGGAUGC 3'
           :..::: :::::::::
query   3' AGGACUCCAGAACCUACU 5'

query=JS16_t0268787, target=FGSG_11331, score=4, range=739-756, strand=1

target  5' ACUAGGGGUCUUGGAUGG 3'
            :.  :.:::::::::::.
query   3' AGGACUCCAGAACCUACU 5'

query=JS16_t0268787, target=FGSG_07456, score=4, range=1644-1661, strand=1

target  5' UCCUGAGGUCUACGAUGA 3'
           :::::::::::  :::::
query   3' AGGACUCCAGAACCUACU 5'

query=JS16_t0268787, target=FGSG_05478, score=4, range=45-62, strand=1

target  5' UCCUGAGGCCUUGGAUAA 3'
           :::::::: ::::::: :
query   3' AGGACUCCAGAACCUACU 5'

query=JS16_t0268787, target=FGSG_04738, score=4, range=1027-1043, strand=1

target  5' UUUUGGGGU-UUGGAUGA 3'
           :..::.::: ::::::::
query   3' AGGACUCCAGAACCUACU 5'

query=JS16_t0268787, target=FGSG_08058, score=4, range=525-542, strand=1

target  5' GGACGAGGUCUUGGAUGA 3'
               ::::::::::::::
query   3' AGGACUCCAGAACCUACU 5'

query=JS16_t0268787, target=FGSG_07050, score=4, range=576-594, strand=1

target  5' UCUUAUGGGUCUUGGAUGG 3'
           ::.:  .:::::::::::.
query   3' AGGA-CUCCAGAACCUACU 5'

query=JS16_t0268787, target=FGSG_08626, score=4, range=27-44, strand=1

target  5' UGCUGAGGUCUCGGAUGU 3'
           : ::::::::: :::::
query   3' AGGACUCCAGAACCUACU 5'

query=JS16_t0268787, target=FGSG_08601, score=4, range=321-337, strand=1

target  5' UCCUGAGGUC-UGGAAGA 3'
           :::::::::: :::: ::
query   3' AGGACUCCAGAACCUACU 5'

query=JS16_t0268787, target=FGSG_07650, score=4, range=1567-1584, strand=1

target  5' GUCGAAGGUCUUGGAUGG 3'
            .:  ::::::::::::.
query   3' AGGACUCCAGAACCUACU 5'

query=JS16_t0268787, target=FGSG_10051, score=4, range=703-719, strand=1

target  5' UCUAGAGGUCUUGG-UGG 3'
           ::. :::::::::: ::.
query   3' AGGACUCCAGAACCUACU 5'

query=JS16_t0268787, target=FGSG_08786, score=4, range=4686-4703, strand=1

target  5' ACAAGAGGUCUUGGAUGC 3'
            :  :::::::::::::
query   3' AGGACUCCAGAACCUACU 5'

query=JS16_t0268787, target=FGSG_07413, score=4, range=1926-1943, strand=1

target  5' UGAAGAGGUCUUGGAUGC 3'
           :    :::::::::::::
query   3' AGGACUCCAGAACCUACU 5'

query=JS16_t0268787, target=FGSG_10338, score=4, range=1404-1421, strand=1

target  5' UGCUAAGGUCUUGGAUAA 3'
           : :: ::::::::::: :
query   3' AGGACUCCAGAACCUACU 5'

query=JS16_t0367196, target=FGSG_09187, score=4, range=459-477, strand=1

target  5' AUUCACCGUCGCCACCGGU 3'
            :   ::::::::::::::
query   3' CACACGGCAGCGGUGGCCA 5'

query=JS16_t0367196, target=FGSG_04442, score=4, range=349-367, strand=-1

target  5' GAGUGCUGCCGCCACCGGU 3'
           : ::::.: ::::::::::
query   3' CACACGGCAGCGGUGGCCA 5'

query=JS16_t0367196, target=FGSG_10424, score=4, range=178-197, strand=-1

target  5' GUGAGGCUGUCGCCAUCGGU 3'
           :::  ::.::::::::.::::
query   3' CAC-ACGGCAGCGGUGGCCA 5'

query=JS16_t0367196, target=FGSG_11597, score=4, range=91-110, strand=-1

target  5' AUGUCGGCGUCGCCAUCGGU 3'
            ::: : ::::::::.::::
query   3' CACA-CGGCAGCGGUGGCCA 5'

query=JS16_t0367196, target=FGSG_12548, score=4, range=1327-1345, strand=-1

target  5' UGGCGCCGUCGCCACCGGA 3'
             : ::::::::::::::
query   3' CACACGGCAGCGGUGGCCA 5'

query=JS16_t0367196, target=FGSG_06089, score=4, range=10215-10232, strand=-1

target  5' GU-UUCCGUCGCCGCCGGA 3'
           :: : ::::::::.::::
query   3' CACACGGCAGCGGUGGCCA 5'

No results for JS16_t1402735
No results for JS16_t0176764
query=JS16_t0693632, target=FGSG_00398, score=2.5, range=1090-1110, strand=-1

target  5' UCGCCAAAGACCUUCCUCAAU 3'
           : .::: ::::::::::::::
query   3' ACUGGUGUCUGGAAGGAGUUA 5'

query=JS16_t0693632, target=FGSG_08474, score=3, range=4500-4520, strand=-1

target  5' GGACCAGAGAUCUUCCUCAAU 3'
            ::::: :::.::::::::::
query   3' ACUGGUGUCUGGAAGGAGUUA 5'

query=JS16_t0693632, target=FGSG_09560, score=4, range=80-99, strand=1

target  5' GUACCA-AGACCUUCCUCAAG 3'
             :::: :::::::::::::
query   3' ACUGGUGUCUGGAAGGAGUUA 5'

query=JS16_t0693632, target=FGSG_09312, score=4, range=496-515, strand=-1

target  5' UGAACAC-GACCUUCCUUGAU 3'
           ::: ::: ::::::::::..::
query   3' ACUGGUGUCUGGAAGGAGUUA 5'

No results for JS16_t4091826
No results for JS16_t3195020
No results for JS16_t4091896
No results for JS16_t0367202
No results for JS16_t3195147
query=JS16_t3195206, target=FGSG_05364, score=3, range=2507-2525, strand=1

target  5' AAGUUGUUAGAAGAGUUCU 3'
            :: ::.:::::::::::
query   3' AUC-ACGAUCUUCUCAAGA 5'

query=JS16_t3195206, target=FGSG_07769, score=3.5, range=392-409, strand=1

target  5' UGGUGCUGGACGAGUUCU 3'
           :.:::::.:: :::::::
query   3' AUCACGAUCUUCUCAAGA 5'

query=JS16_t3195206, target=FGSG_04682, score=3.5, range=604-620, strand=1

target  5' UGG-CCUAGAAGGGUUCU 3'
           :.:  :::::::.:::::
query   3' AUCACGAUCUUCUCAAGA 5'

query=JS16_t3195206, target=FGSG_00853, score=3.5, range=663-679, strand=-1

target  5' UGG-GCUGGAAGGGUUCU 3'
           :.: :::.::::.:::::
query   3' AUCACGAUCUUCUCAAGA 5'

query=JS16_t3195206, target=FGSG_07809, score=3.5, range=261-277, strand=-1
```

```
query=JS16_t0268787, target=FGSG_02583, score=4, range=2322-2338, strand=1

target  5’ UCCUGAGGUC-AGGAUGA 3’
           ::::::::::  ::::::
query   3’ AGGACUCCAGAACCUACU 5’

query=JS16_t0268787, target=FGSG_02013, score=4, range=31-48, strand=-1

target  5’ UUUUGAGGUCGUGGAUGU 3’
           :..::::::: ::::::
query   3’ AGGACUCCAGAACCUACU 5’

query=JS16_t0268787, target=FGSG_00875, score=4, range=1445-1463, strand=-1

target  5’ UCCUGUGGGUCCUGGAUGA 3’
           ::::: .:::: :::::::
query   3’ AGGAC-UCCAGAACCUACU 5’

query=JS16_t0268787, target=FGSG_01719, score=4, range=71-89, strand=-1

target  5’ UCUGUGAGGUCUAGGAUGG 3’
           ::. :::::::: :::::.
query   3’ AGG-ACUCCAGAACCUACU 5’

query=JS16_t0268787, target=FGSG_00984, score=4, range=476-493, strand=-1

target  5’ GGUAGAGGUCUUGGAUGG 3’
             . :::::::::::::.
query   3’ AGGACUCCAGAACCUACU 5’

query=JS16_t0268787, target=FGSG_08980, score=4, range=79-96, strand=-1

target  5’ UCCCCAGGUCUUGGGUGU 3’
           :::  :::::::::.::
query   3’ AGGACUCCAGAACCUACU 5’

query=JS16_t0268787, target=FGSG_02963, score=4, range=413-431, strand=-1

target  5’ UCGUGUAGGUUUUGGAUGU 3’
           :: :: ::::.:::::::
query   3’ AGGAC-UCCAGAACCUACU 5’

query=JS16_t0268787, target=FGSG_03656, score=4, range=659-676, strand=-1

target  5’ GGCUCAGGUCUUGGAUGU 3’
             :: ::::::::::::
query   3’ AGGACUCCAGAACCUACU 5’

query=JS16_t0268787, target=FGSG_06015, score=4, range=536-554, strand=-1

target  5’ CGCUGAAGGUCUUGGAUGC 3’
             ::: ::::::::::::
query   3’ AGGAC-UCCAGAACCUACU 5’

query=JS16_t0268787, target=FGSG_13510, score=4, range=1280-1297, strand=-1

target  5’ UGCCGAGCUCUUGGAUGA 3’
           : : ::: ::::::::::
query   3’ AGGACUCCAGAACCUACU 5’

query=JS16_t0268787, target=FGSG_10558, score=4, range=944-960, strand=-1

target  5’ GCGUGA-GUCUUGGAUGA 3’
            : ::: :::::::::::
query   3’ AGGACUCCAGAACCUACU 5’

query=JS16_t0268787, target=FGSG_11118, score=4, range=1106-1122, strand=-1

target  5’ UACUGAGGUCUU-GAUGC 3’
           : :::::::::: ::::
query   3’ AGGACUCCAGAACCUACU 5’

query=JS16_t0268787, target=FGSG_00972, score=4, range=883-899, strand=-1

target  5’ UCC-GAGGUCAUGGAUGU 3’
           ::: :::::: ::::::
query   3’ AGGACUCCAGAACCUACU 5’

query=JS16_t0268787, target=FGSG_08136, score=4, range=1331-1347, strand=-1

target  5’ ACCUGA-GUCUUGGGUGA 3’
            ::::: :::::::.:::
query   3’ AGGACUCCAGAACCUACU 5’

query=JS16_t0268787, target=FGSG_05973, score=4, range=1127-1143, strand=-1

target  5’ UCCGUAGGUCUUGG-UGA 3’
           :::  ::::::::: :::
query   3’ AGGACUCCAGAACCUACU 5’

query=JS16_t0268787, target=FGSG_02964, score=4, range=171-187, strand=-1

target  5’ UGGU-CCAGAAGAGUUCU 3’
           :.:: : :::::::::::
query   3’ AUCACGAUCUUCUCAAGA 5’

query=JS16_t3195206, target=FGSG_06417, score=4, range=657-673, strand=1

target  5’ UA-UGUUGGAAGAGUUUU 3’
           :: ::.:.::::::::.:
query   3’ AUCACGAUCUUCUCAAGA 5’

query=JS16_t3195206, target=FGSG_13318, score=4, range=1931-1949, strand=1

target  5’ UAGCUGCGGGAAGAGUUCU 3’
           ::: ::: .::::::::::
query   3’ AUC-ACGAUCUUCUCAAGA 5’

query=JS16_t3195206, target=FGSG_10007, score=4, range=2546-2563, strand=1

target  5’ UACCACUAGAAGAGUUCA 3’
           ::    ::::::::::::
query   3’ AUCACGAUCUUCUCAAGA 5’

query=JS16_t3195206, target=FGSG_03606, score=4, range=471-487, strand=1

target  5’ UAGCACU-GAAGAGUUCU 3’
           :::  :: ::::::::::
query   3’ AUCACGAUCUUCUCAAGA 5’

query=JS16_t3195206, target=FGSG_02878, score=4, range=240-257, strand=-1

target  5’ GAGUGCUGGGAGGGUUCU 3’
            ::::::.:.::.:::::
query   3’ AUCACGAUCUUCUCAAGA 5’

query=JS16_t3195206, target=FGSG_04408, score=4, range=819-836, strand=-1

target  5’ ACGUGCUGGAGGAGUUCU 3’
             :::::.::.:::::::
query   3’ AUCACGAUCUUCUCAAGA 5’

query=JS16_t3195206, target=FGSG_10530, score=4, range=8645-8663, strand=-1

target  5’ UAGUGACUAGAGAAGUUCU 3’
           ::::: :::::. ::::::
query   3’ AUCAC-GAUCUUCUCAAGA 5’

query=JS16_t3195206, target=FGSG_04481, score=4, range=196-213, strand=-1

target  5’ AAGUUCUAGGAGAGUUCG 3’
            ::: ::::.:::::::
query   3’ AUCACGAUCUUCUCAAGA 5’

query=JS16_t3195206, target=FGSG_07262, score=4, range=2948-2965, strand=-1

target  5’ CAGGGCUAGAAGACUUCU 3’
            :: ::::::::: ::::
query   3’ AUCACGAUCUUCUCAAGA 5’

query=JS16_t3195206, target=FGSG_10738, score=4, range=348-365, strand=-1

target  5’ UAGCGCUUGAAGAGUUCA 3’
           ::: ::: :::::::::
query   3’ AUCACGAUCUUCUCAAGA 5’

query=JS16_t3195206, target=FGSG_07757, score=4, range=863-880, strand=-1

target  5’ GAGUCCUUGAAGAGUUCU 3’
            ::: :: ::::::::::
query   3’ AUCACGAUCUUCUCAAGA 5’

query=JS16_t3195206, target=FGSG_00763, score=4, range=795-812, strand=-1

target  5’ AACUCCUGGAAGAGUUCU 3’
            : : ::.::::::::::
query   3’ AUCACGAUCUUCUCAAGA 5’

query=JS16_t3195206, target=FGSG_12651, score=4, range=236-252, strand=-1

target  5’ UACUGCUAGAGGAG-UCU 3’
           :: :::::::.::: :::
query   3’ AUCACGAUCUUCUCAAGA 5’

query=JS16_t3195206, target=FGSG_00132, score=4, range=310-326, strand=-1

target  5’ GAGU-CUAGCAGAGUUCU 3’
            ::: :::: ::::::::
query   3’ AUCACGAUCUUCUCAAGA 5’

query=JS16_t3195206, target=FGSG_10086, score=4, range=440-458, strand=-1

target  5’ UCGUGGUAGAAGACGUUCU 3’
```

```
target  5' UGCUGAGGUC-UGGGUGA 3'
           : :::::::: :::.:::
query   3' AGGACUCCAGAACCUACU 5'

query=JS16_t0268787, target=FGSG_08510, score=4, range=100-118, strand=-1

target  5' CCUUGGGGUGCUUGGAUGA 3'
            :.::.::: :::::::::
query   3' AGGACUCCA-GAACCUACU 5'

query=JS16_t2235497, target=FGSG_06228, score=3, range=736-756, strand=1

target  5' UUCGAGUUGAUGGAGCCUGUA 3'
           :::: :::::::::.:::::
query   3' AAGCACAACUACCUUGGACAG 5'

query=JS16_t2235497, target=FGSG_10997, score=4, range=693-714, strand=-1

target  5' AUCGAUAGUGAUGGAACCUGUC 3'
            ::: :  ::::::::::::::
query   3' AAGC-ACAACUACCUUGGACAG 5'

No results for JS16_t2235537
No results for JS16_t2235555
query=JS16_t1339105, target=FGSG_04465, score=3.5, range=335-352, strand=1

target  5' GAUUUGCUUUCGCGAAUG 3'
           .:..::::::::::: ::
query   3' UUGGACGAAAGCGCUGAC 5'

query=JS16_t1339105, target=FGSG_03360, score=3.5, range=1126-1143, strand=1

target  5' ACCUUGCUUUUGCGACUU 3'
           : :.::::::.::::::
query   3' UUGGACGAAAGCGCUGAC 5'

query=JS16_t1339105, target=FGSG_00719, score=3.5, range=1516-1532, strand=1

target  5' AAUCUGCUUUUGC-ACUG 3'
           ::.:::::::.:: ::::
query   3' UUGGACGAAAGCGCUGAC 5'

query=JS16_t1339105, target=FGSG_06999, score=3.5, range=496-513, strand=-1

target  5' CACUCGCUUUUGCGACUG 3'
            ::.  :::::.:::::::
query   3' UUGGACGAAAGCGCUGAC 5'

query=JS16_t1339105, target=FGSG_05571, score=4, range=1163-1180, strand=1

target  5' AGCUCGCUUUUGUGACUG 3'
           :.:.  :::::.:.:::::
query   3' UUGGACGAAAGCGCUGAC 5'

query=JS16_t1339105, target=FGSG_09048, score=4, range=827-845, strand=1

target  5' AACCUGCUUUCGACGGCUC 3'
           :::::::::::: ::.::
query   3' UUGGACGAAAGC-GCUGAC 5'

query=JS16_t1339105, target=FGSG_03413, score=4, range=368-385, strand=1

target  5' CGCUUGCUUUCGCAACUG 3'
            .:.::::::::: ::::
query   3' UUGGACGAAAGCGCUGAC 5'

query=JS16_t1339105, target=FGSG_02522, score=4, range=572-590, strand=1

target  5' AACCUGCUUUCGCGUCGUG 3'
           :::::::::::::: : ::
query   3' UUGGACGAAAGCGCUG-AC 5'

query=JS16_t1339105, target=FGSG_12672, score=4, range=1216-1234, strand=1

target  5' GGCGCUGCUUUCGCAACUG 3'
           ..: :::::::::: ::::
query   3' UUG-GACGAAAGCGCUGAC 5'

query=JS16_t1339105, target=FGSG_00814, score=4, range=714-730, strand=1

target  5' UGUC-GUUUUCGCGACUG 3'
            ..: :.::::::::::
query   3' UUGGACGAAAGCGCUGAC 5'

query=JS16_t1339105, target=FGSG_05869, score=4, range=412-428, strand=1

target  5' AACC-GUUUUCUCGACUG 3'
           :::: :.:::: ::::::
query   3' UUGGACGAAAGCGCUGAC 5'

query=JS16_t1339105, target=FGSG_13680, score=4, range=209-225, strand=1

           : ::: ::::::: :::::
query   3' AUCACGAUCUUCU-CAAGA 5'

query=JS16_t0211595, target=FGSG_11485, score=4, range=597-619, strand=1

target  5' CUACGAAGCAAUUGGCGCAACCA 3'
           :::  :: ::::::::.::::::
query   3' GAUUGUUAGUUAACCGUGUUGGU 5'

query=JS16_t0693666, target=FGSG_00899, score=2.5, range=1590-1608, strand=1

target  5' CGCUUUAGGUCAGAGUCAA 3'
           :: :.:::::::::::::
query   3' GCAAGAUCCAGUCUCAGUC 5'

query=JS16_t0693666, target=FGSG_05468, score=3, range=212-229, strand=-1

target  5' CG-UCAAGGUCAGAGUCAU 3'
           :: :: ::::::::::::
query   3' GCAAGAUCCAGUCUCAGUC 5'

query=JS16_t0693666, target=FGSG_10147, score=4, range=936-955, strand=1

target  5' CUUUCUAGGUCAUGGGUCAG 3'
           : :::::::::: :.:::::
query   3' GCAAGAUCCAGU-CUCAGUC 5'

query=JS16_t0693666, target=FGSG_02628, score=4, range=183-202, strand=1

target  5' CGUUGCAGGGUCAGGGUCAG 3'
           :::: : .::::::.:::::
query   3' GCAA-GAUCCAGUCUCAGUC 5'

query=JS16_t0693666, target=FGSG_02521, score=4, range=979-997, strand=1

target  5' CGCCCUAGGUUAGAGUCAC 3'
           ::  ::::::.:::::::
query   3' GCAAGAUCCAGUCUCAGUC 5'

query=JS16_t0693666, target=FGSG_05436, score=4, range=1409-1426, strand=1

target  5' AGUUCGAGG-CAGAGUCAG 3'
            :::: ::: :::::::::
query   3' GCAAGAUCCAGUCUCAGUC 5'

query=JS16_t0693666, target=FGSG_08607, score=4, range=227-245, strand=-1

target  5' CGUUCUGGGUCGGAGUCGU 3'
           :::::.::::.:::::.
query   3' GCAAGAUCCAGUCUCAGUC 5'

query=JS16_t0693666, target=FGSG_02228, score=4, range=245-262, strand=-1

target  5' CG-UCUAGGUCAGAAUCGG 3'
           :: ::::::::::: ::.:
query   3' GCAAGAUCCAGUCUCAGUC 5'

query=JS16_t0693666, target=FGSG_13484, score=4, range=308-325, strand=-1

target  5' CGCUC-AGGUCAGGGUCAA 3'
           :: :: :::::::.::::
query   3' GCAAGAUCCAGUCUCAGUC 5'

query=JS16_t0693666, target=FGSG_09572, score=4, range=1424-1441, strand=-1

target  5' CGUAGUAGGUCAG-GUCAG 3'
           :::  :::::::: :::::
query   3' GCAAGAUCCAGUCUCAGUC 5'

No results for JS16_t0367212
query=JS16_t1403041, target=FGSG_09093, score=2, range=1095-1113, strand=1

target  5' CAACAAGUGGACCAUGCUC 3'
             :::::::::::::::::
query   3' CAUGUUCACCUGGUACGAG 5'

query=JS16_t1403041, target=FGSG_08506, score=2.5, range=700-718, strand=-1

target  5' GCAUGAGUGGACCAUGCUU 3'
           : :..:::::::::::::.
query   3' CAUGUUCACCUGGUACGAG 5'

query=JS16_t1403041, target=FGSG_05249, score=3, range=1629-1647, strand=1

target  5' CGACAAGUGGACCAUGUUC 3'
             :::::::::::::.::
query   3' CAUGUUCACCUGGUACGAG 5'

query=JS16_t1403041, target=FGSG_00659, score=3.5, range=1581-1599, strand=1

target  5' GCACGCGUGGACCAUGUUC 3'
           : ::. ::::::::::.::
```

```
target  5’ GGCAUGCUUUCGC-ACUG 3’
           ..: ::::::::: ::::
query   3’ UUGGACGAAAGCGCUGAC 5’

query=JS16_t1339105, target=FGSG_07855, score=4, range=684-702, strand=1

target  5’ AACAUACUUUCGCGAUCUG 3’
           ::: : ::::::::: :::
query   3’ UUGGACGAAAGCGCU-GAC 5’

query=JS16_t1339105, target=FGSG_05723, score=4, range=824-840, strand=1

target  5’ AGUCUG-UUUCGCGAUUG 3’
           :..::: ::::::::.::
query   3’ UUGGACGAAAGCGCUGAC 5’

query=JS16_t1339105, target=FGSG_08784, score=4, range=394-411, strand=-1

target  5’ GGCCUGCUUUCGCGACCA 3’
           ..::::::::::::::
query   3’ UUGGACGAAAGCGCUGAC 5’

query=JS16_t1339105, target=FGSG_04822, score=4, range=1776-1793, strand=-1

target  5’ GCUCUGCUGUCGCGACUG 3’
           . .::::: :::::::::
query   3’ UUGGACGAAAGCGCUGAC 5’

query=JS16_t1339105, target=FGSG_00741, score=4, range=86-103, strand=-1

target  5’ GACUAGCUGUCGCGACUG 3’
           .::. ::: :::::::::
query   3’ UUGGACGAAAGCGCUGAC 5’

query=JS16_t1339105, target=FGSG_06373, score=4, range=196-213, strand=-1

target  5’ ACCCGGCUGUCGCGACUG 3’
           : :: ::: :::::::::
query   3’ UUGGACGAAAGCGCUGAC 5’

query=JS16_t1339105, target=FGSG_08896, score=4, range=398-414, strand=-1

target  5’ GAUCUGCUUUC-CGACUC 3’
           .:.:::::::: :::::
query   3’ UUGGACGAAAGCGCUGAC 5’

query=JS16_t1339105, target=FGSG_08000, score=4, range=139-155, strand=-1

target  5’ AGGUUGCUUU-GCGACUG 3’
           :. .:::::: :::::::
query   3’ UUGGACGAAAGCGCUGAC 5’

No results for JS16_t0577327
No results for JS16_t3131846
No results for JS16_t2235726
query=JS16_t0170960, target=FGSG_02689, score=3, range=593-612, strand=1

target  5’ ACGCCGUCGUCCUAGCUUUC 3’
           ::::::::::: :::::::
query   3’ UGCGGCAGCAGAAUCGAAAA 5’

query=JS16_t0170960, target=FGSG_04292, score=3.5, range=728-747, strand=-1

target  5’ GCGUUGUCGUCGUAGCUUUU 3’
           .::..:::::: ::::::::
query   3’ UGCGGCAGCAGAAUCGAAAA 5’

query=JS16_t0170960, target=FGSG_05849, score=3.5, range=697-716, strand=-1

target  5’ AUGCCCUCGUCUUGGCUUUG 3’
           :.::: :::::::.:::::
query   3’ UGCGGCAGCAGAAUCGAAAA 5’

No results for JS16_t3131902
No results for JS16_t4028496
No results for JS16_t2235876
query=JS16_t1339355, target=FGSG_00567, score=4, range=1689-1710, strand=-1

target  5’ CAGCGCGAGCUUUAGCUUUCGA 3’
           :: : :::::::::.:.:::::
query   3’ GUGGAGCUCGAAAUUGGAAGCU 5’

No results for JS16_t1339375
query=JS16_t1339391, target=FGSG_10042, score=2.5, range=1183-1202, strand=-1

target  5’ CCAAAGACAGUACCAAGAAG 3’
           :::::. :::::::::.:::
query   3’ GGUUUUGGUCAUGGUUUUUC 5’

query=JS16_t1339391, target=FGSG_13420, score=3, range=992-1011, strand=1

query   3’ CAUGUUCACCUGGUACGAG 5’

query=JS16_t1403041, target=FGSG_03100, score=3.5, range=905-924, strand=1

target  5’ GGACGGAGUGGACUAUGCUC 3’
           : :: .:::::::.::::::
query   3’ CAUG-UUCACCUGGUACGAG 5’

query=JS16_t1403041, target=FGSG_13459, score=3.5, range=295-313, strand=-1

target  5’ UUACGAGUGGACCAUGUUG 3’
            :::.:::::::::::.:
query   3’ CAUGUUCACCUGGUACGAG 5’

query=JS16_t1403041, target=FGSG_07521, score=3.5, range=292-309, strand=-1

target  5’ GUAUAGGUGGACCA-GCUU 3’
           :::.:.:::::::: :::.
query   3’ CAUGUUCACCUGGUACGAG 5’

query=JS16_t1403041, target=FGSG_09231, score=4, range=519-537, strand=1

target  5’ CUACAACUGGACCAUGUUC 3’
            ::::: :::::::::.::
query   3’ CAUGUUCACCUGGUACGAG 5’

query=JS16_t1403041, target=FGSG_02839, score=4, range=1719-1737, strand=1

target  5’ AGACAAGUGGAACAUGCUC 3’
             ::::::::: :::::::
query   3’ CAUGUUCACCUGGUACGAG 5’

query=JS16_t1403041, target=FGSG_00399, score=4, range=1845-1863, strand=1

target  5’ GUUGAAGCGGACCAUGCUC 3’
           ::  ::: :::::::::::
query   3’ CAUGUUCACCUGGUACGAG 5’

query=JS16_t1403041, target=FGSG_12407, score=4, range=472-489, strand=1

target  5’ GCAGAAGUGG-CCAUGCUC 3’
           : : :::::: ::::::::
query   3’ CAUGUUCACCUGGUACGAG 5’

query=JS16_t1403041, target=FGSG_07075, score=4, range=747-765, strand=-1

target  5’ GUACAAGUGGACGAUGUUG 3’
           :::::::::::: :::.:
query   3’ CAUGUUCACCUGGUACGAG 5’

query=JS16_t1403041, target=FGSG_03529, score=4, range=437-456, strand=-1

target  5’ GUAUUGGUGGACCAUGCCUC 3’
           :::. .:::::::::: :::
query   3’ CAUGUUCACCUGGUAC-GAG 5’

query=JS16_t1403041, target=FGSG_09785, score=4, range=577-595, strand=-1

target  5’ GGACACGGGGACCAUGCUC 3’
           : ::: : :::::::::::
query   3’ CAUGUUCACCUGGUACGAG 5’

No results for JS16_t1403071
No results for JS16_t4092184
No results for JS16_t4092188
No results for JS16_t2299270
No results for JS16_t0202770
No results for JS16_t0241381
No results for JS16_t0476127
No results for JS16_t0229112
query=JS16_t1403215, target=FGSG_02543, score=3.5, range=369-391, strand=-1

target  5’ UCAAAGCAAGGUUUGGGUUACCA 3’
           : :::.::::: :::::::::::
query   3’ ACUUUUGUUCC-AACCCAAUGGU 5’

query=JS16_t2299401, target=FGSG_12263, score=3.5, range=506-525, strand=-1

target  5’ UGGUGGAAAUCUCAU-UCCAG 3’
           :::.::: ::::::: :::::
query   3’ ACCGCCUGUAGAGUAUAGGUC 5’

query=JS16_t2299401, target=FGSG_02184, score=4, range=1086-1106, strand=1

target  5’ UGAAGGAGAUCUCAUAUCCGG 3’
           ::  ::: :::::::::::.:
query   3’ ACCGCCUGUAGAGUAUAGGUC 5’

query=JS16_t2299401, target=FGSG_09914, score=4, range=1135-1156, strand=-1

target  5’ UGCCGAGGUAUCUCAUAUCCAU 3’
           :: :: :..:::::::::::
```

```
target  5’ CCAAUACCGGUACCAAGAAG 3’
           :::: :::.:::::::.:::
query   3’ GGUUUUGGUCAUGGUUUUUC 5’

query=JS16_t1339391, target=FGSG_07797, score=3, range=524-543, strand=1

target  5’ CCAACAACAGUACCAAGAAG 3’
           :::: : :::::::::.:::
query   3’ GGUUUUGGUCAUGGUUUUUC 5’

query=JS16_t1339391, target=FGSG_05108, score=3.5, range=362-381, strand=1

target  5’ CCAAGACCGGUACCGGAAAG 3’
           ::::.:::.:::::..::::
query   3’ GGUUUUGGUCAUGGUUUUUC 5’

query=JS16_t1339391, target=FGSG_11985, score=3.5, range=161-180, strand=1

target  5’ CCAGCACCAGUACCAAAAGC 3’
           :::. :::::::::::::.
query   3’ GGUUUUGGUCAUGGUUUUUC 5’

query=JS16_t1339391, target=FGSG_13121, score=3.5, range=165-185, strand=1

target  5’ CCAGAACGCGGUACCAAAAAA 3’
           :::.::: :.::::::::::
query   3’ GGUUUUG-GUCAUGGUUUUUC 5’

query=JS16_t1339391, target=FGSG_09642, score=3.5, range=335-353, strand=1

target  5’ CCGAAACC-GUACCAAAAAC 3’
           ::.::::: ::::::::::
query   3’ GGUUUUGGUCAUGGUUUUUC 5’

query=JS16_t1339391, target=FGSG_02010, score=3.5, range=277-295, strand=1

target  5’ CCAAAG-AAGUACCAAAAAG 3’
           :::::.  ::::::::::::
query   3’ GGUUUUGGUCAUGGUUUUUC 5’

query=JS16_t1339391, target=FGSG_05784, score=3.5, range=430-450, strand=-1

target  5’ CCAGAGACCGGUACCGAAAAG 3’
           ::: :.:::.:::::.:::::
query   3’ GGU-UUUGGUCAUGGUUUUUC 5’

query=JS16_t1339391, target=FGSG_07371, score=3.5, range=1534-1553, strand=-1

target  5’ UCAAACGCAGUACCAAAGAG 3’
           .::::  ::::::::::.::
query   3’ GGUUUUGGUCAUGGUUUUUC 5’

query=JS16_t1339391, target=FGSG_07003, score=3.5, range=1549-1568, strand=-1

target  5’ CCUCGACCAGUAUCAAAAAG 3’
           ::  .:::::::.:::::::
query   3’ GGUUUUGGUCAUGGUUUUUC 5’

query=JS16_t1339391, target=FGSG_09111, score=3.5, range=1822-1841, strand=-1

target  5’ UCAUGGACAGUACCAAAAAG 3’
           .:: .. :::::::::::::
query   3’ GGUUUUGGUCAUGGUUUUUC 5’

query=JS16_t1339391, target=FGSG_10221, score=4, range=85-104, strand=1

target  5’ UCAAAAUCGGUACAAAAAAG 3’
           .:::::.:.:::: ::::::
query   3’ GGUUUUGGUCAUGGUUUUUC 5’

query=JS16_t1339391, target=FGSG_09338, score=4, range=2360-2379, strand=1

target  5’ CCAAGGGCGGUGCCAAAAAG 3’
           ::::.. :.::.::::::::
query   3’ GGUUUUGGUCAUGGUUUUUC 5’

query=JS16_t1339391, target=FGSG_05427, score=4, range=1164-1183, strand=1

target  5’ CCAGAGCCAGAACCAAAAAC 3’
           :::.:.:::: ::::::::
query   3’ GGUUUUGGUCAUGGUUUUUC 5’

query=JS16_t1339391, target=FGSG_08579, score=4, range=1550-1569, strand=1

target  5’ GCAAGGCCAUUACCAAAAAG 3’
            :::..::: ::::::::::
query   3’ GGUUUUGGUCAUGGUUUUUC 5’

query=JS16_t1339391, target=FGSG_05173, score=4, range=1056-1074, strand=1

target  5’ CCAAAAACAGU-CCAAGAAG 3’
           :::::: :::: ::::.:::
```

```
query   3’ ACCGC-CUGUAGAGUAUAGGUC 5’

No results for JS16_t0396549
No results for JS16_t4092373
No results for JS16_t4092382
No results for JS16_t0693699
query=JS16_t4092498, target=FGSG_12977, score=4, range=109-128, strand=1

target  5’ GCU-GCUGAAGUUCGAGUUGG 3’
           ::: :::.:::::::::..::.
query   3’ CGAGCGAUUUCAAGCUUGACU 5’

query=JS16_t4092498, target=FGSG_01896, score=4, range=147-166, strand=1

target  5’ GCGCGCU-GAGUUUGAACUGA 3’
           :: :::: .::::.:::::::
query   3’ CGAGCGAUUUCAAGCUUGACU 5’

No results for JS16_t4092524
No results for JS16_t0803261
No results for JS16_t4092548
query=JS16_t0585040, target=FGSG_00509, score=3.5, range=1009-1028, strand=-1

target  5’ AUCUCUUCGUCAGGAGUAAG 3’
           :: .:::::::: :::::::
query   3’ UAAGGAAGCAGUACUCAUUC 5’

query=JS16_t0585040, target=FGSG_01847, score=4, range=1132-1151, strand=-1

target  5’ AUUCCUUCGUCGUCAGUGAG 3’
           :::::::::::.: :::.::
query   3’ UAAGGAAGCAGUACUCAUUC 5’

No results for JS16_t0269832
No results for JS16_t0476167
No results for JS16_t2299724
No results for JS16_t0269835
No results for JS16_t0693743
No results for JS16_t3195854
No results for JS16_t4092718
No results for JS16_t0188934
query=JS16_t0220427, target=FGSG_13878, score=2, range=23147-23164, strand=1

target  5’ ACGGAUCUGAGGAUUCGA 3’
           : :: :::::::::::::
query   3’ UACCCAGACUCCUAAGCU 5’

query=JS16_t0220427, target=FGSG_13789, score=3, range=281-298, strand=1

target  5’ AUGAGUUUGAGGAUUUGA 3’
           ::: ::.::::::::.::
query   3’ UACCCAGACUCCUAAGCU 5’

query=JS16_t0220427, target=FGSG_05329, score=3, range=2439-2457, strand=-1

target  5’ AUUGGUCUGAGGAUAUCGA 3’
           :: ::::::::::: ::::
query   3’ UACCCAGACUCCUA-AGCU 5’

query=JS16_t0220427, target=FGSG_03142, score=3.5, range=1035-1052, strand=-1

target  5’ AUGGUUCUGAGGAUACGG 3’
           :::: ::::::::: ::.
query   3’ UACCCAGACUCCUAAGCU 5’

query=JS16_t0220427, target=FGSG_09128, score=4, range=1-18, strand=1

target  5’ AUGGGUCUGGGGAUUCUU 3’
           :::::::::.::::::
query   3’ UACCCAGACUCCUAAGCU 5’

query=JS16_t0220427, target=FGSG_06748, score=4, range=1568-1585, strand=1

target  5’ AUGGGGCUGAGGAGUCGA 3’
           ::::: ::::::: ::::
query   3’ UACCCAGACUCCUAAGCU 5’

query=JS16_t0220427, target=FGSG_03840, score=4, range=617-634, strand=1

target  5’ AUGUGUCUGUGGGUUCGA 3’
           ::: ::::: ::.:::::
query   3’ UACCCAGACUCCUAAGCU 5’

query=JS16_t0220427, target=FGSG_06935, score=4, range=44-62, strand=1

target  5’ AUGGCUUUGAGGAUAUCGA 3’
           :::: :.::::::: ::::
query   3’ UACCCAGACUCCUA-AGCU 5’

query=JS16_t0220427, target=FGSG_12112, score=4, range=3398-3415, strand=1

target  5’ ACGGGUCUGCGGAUUCGU 3’
```

```
query   3’ GGUUUUGGUCAUGGUUUUUC 5’

query=JS16_t1339391, target=FGSG_00739, score=4, range=1126-1145, strand=-1

target  5’ CCAAUACCAGUACCGAUAAG 3’
           :::: :::::::::.: :::
query   3’ GGUUUUGGUCAUGGUUUUUC 5’

query=JS16_t1339391, target=FGSG_10519, score=4, range=331-349, strand=-1

target  5’ CCAAAACCAG-AGCAAAAAG 3’
           :::::::::: : :::::::
query   3’ GGUUUUGGUCAUGGUUUUUC 5’

query=JS16_t2235955, target=FGSG_00489, score=4, range=143-163, strand=1

target  5’ AAUGCCAGGUUUGCGUCGUUG 3’
           :::  ::::::::: ::::::
query   3’ UUAGCGUCCAAACGAAGCAAC 5’

query=JS16_t2235955, target=FGSG_10526, score=4, range=753-773, strand=-1

target  5’ GGUCGCAAGUUUGCUUCGAUG 3’
           ..::::: :::::::::: ::
query   3’ UUAGCGUCCAAACGAAGCAAC 5’

query=JS16_t2235955, target=FGSG_05079, score=4, range=4034-4053, strand=-1

target  5’ AGAUGCA-GUUUGCUUCGUUC 3’
           :. .::: ::::::::::::
query   3’ UUAGCGUCCAAACGAAGCAAC 5’

No results for JS16_t2235988
query=JS16_t4028631, target=FGSG_06663, score=4, range=802-822, strand=1

target  5’ GACGGUGGUUCGUGGAGCGGU 3’
            :: ::: ::::::.::::::
query   3’ GUGACACGAAGCACUUCGCCA 5’

query=JS16_t4028631, target=FGSG_10523, score=4, range=4405-4426, strand=-1

target  5’ UGCUGUGCUUUGUGACAGCGGU 3’
           ..::::::::.:::: ::::::
query   3’ GUGACACGAAGCACU-UCGCCA 5’

query=JS16_t4028631, target=FGSG_12481, score=4, range=849-869, strand=-1

target  5’ CACUGUGCUUCGUGAAGCUCU 3’
           ::::::::::::::::::  :
query   3’ GUGACACGAAGCACUUCGCCA 5’

query=JS16_t4028631, target=FGSG_06764, score=4, range=644-663, strand=-1

target  5’ CAC-GAGCUUCAUGAAGCGGU 3’
           ::: : ::::: :::::::::
query   3’ GUGACACGAAGCACUUCGCCA 5’

query=JS16_t0194492, target=FGSG_01092, score=3, range=380-400, strand=1

target  5’ UCGACGAGACAGAGCAGCUGG 3’
           .::::::.::::::::: :::
query   3’ GGCUGCUUUGUCUCGUCUACC 5’

query=JS16_t0194492, target=FGSG_07294, score=3, range=1886-1906, strand=1

target  5’ CCGACGAUACGGAGCAGAUGC 3’
           ::::::: ::.:::::::::
query   3’ GGCUGCUUUGUCUCGUCUACC 5’

query=JS16_t0194492, target=FGSG_00052, score=3, range=71-90, strand=-1

target  5’ CCGAC-AAAGAGAGCAGAUGG 3’
           ::::: ::: :::::::::::
query   3’ GGCUGCUUUGUCUCGUCUACC 5’

query=JS16_t0194492, target=FGSG_11228, score=3.5, range=1586-1605, strand=-1

target  5’ ACGACGGAACAGAGC-GAUGG 3’
            :::::.:::::::: :::::
query   3’ GGCUGCUUUGUCUCGUCUACC 5’

query=JS16_t0194492, target=FGSG_11890, score=4, range=883-904, strand=-1

target  5’ UUGAAGACAACGGAGCAGAUGG 3’
           ..:: :: :::.:::::::::
query   3’ GGCUGCU-UUGUCUCGUCUACC 5’

query=JS16_t0577373, target=FGSG_01049, score=3, range=12618-12634, strand=1

target  5’ UA-CAACUAACCUAGCAG 3’
           :: :::::::::: :::
query   3’ AUAGUUGAUUGGAUGGUC 5’

           : ::::::: :::::::
query   3’ UACCCAGACUCCUAAGCU 5’

query=JS16_t0220427, target=FGSG_00508, score=4, range=302-319, strand=-1

target  5’ AUGGCUCUGGGGGUUCGU 3’
           :::: ::::.::.::::
query   3’ UACCCAGACUCCUAAGCU 5’

query=JS16_t0220427, target=FGSG_13878, score=4, range=32255-32272, strand=-1

target  5’ AUGGGCCUGAGGAUCCGA 3’
           ::::: :::::::: :::
query   3’ UACCCAGACUCCUAAGCU 5’

query=JS16_t0220427, target=FGSG_06168, score=4, range=856-873, strand=-1

target  5’ AUGGGCCUGAGGAUGCGA 3’
           ::::: :::::::: :::
query   3’ UACCCAGACUCCUAAGCU 5’

query=JS16_t0220427, target=FGSG_12752, score=4, range=273-290, strand=-1

target  5’ AUGGGUCUAAGAAUUCGA 3’
           :::::::: :: ::::::
query   3’ UACCCAGACUCCUAAGCU 5’

query=JS16_t0220427, target=FGSG_13491, score=4, range=108-126, strand=-1

target  5’ AUGGGGCUGAGGAUUGUGA 3’
           ::::: ::::::::: .::
query   3’ UACCCAGACUCCUAA-GCU 5’

query=JS16_t0220427, target=FGSG_09018, score=4, range=110-128, strand=-1

target  5’ GUAGGGUCUGAUGAUUCGG 3’
           .: :::::::: ::::::.
query   3’ UA-CCCAGACUCCUAAGCU 5’

query=JS16_t0220427, target=FGSG_06593, score=4, range=488-504, strand=-1

target  5’ UUGGGUCUGGGGA-UCGA 3’
            ::::::::.::: ::::
query   3’ UACCCAGACUCCUAAGCU 5’

query=JS16_t0220427, target=FGSG_01619, score=4, range=88-104, strand=-1

target  5’ AU-GGUCUGAGGGUUUGU 3’
           :: :::::::::.::.:
query   3’ UACCCAGACUCCUAAGCU 5’

query=JS16_t0220427, target=FGSG_05990, score=4, range=2056-2072, strand=-1

target  5’ AUGGGGCUGAGGA-UCGA 3’
           ::::: ::::::: ::::
query   3’ UACCCAGACUCCUAAGCU 5’

query=JS16_t0220427, target=FGSG_13924, score=4, range=131-148, strand=-1

target  5’ CUGCAUCUGAGGAUUUGA 3’
            ::  ::::::::::.::
query   3’ UACCCAGACUCCUAAGCU 5’

query=JS16_t0220427, target=FGSG_08510, score=4, range=12-28, strand=-1

target  5’ AU-GAUCUGAGGGUUCGU 3’
           :: : :::::::.::::
query   3’ UACCCAGACUCCUAAGCU 5’

query=JS16_t0220427, target=FGSG_10927, score=4, range=117-135, strand=-1

target  5’ GUGUGUCUUGAGGAUUCGG 3’
           .:: ::: ::::::::::.
query   3’ UACCCAG-ACUCCUAAGCU 5’

query=JS16_t0220427, target=FGSG_00374, score=4, range=722-738, strand=-1

target  5’ AGGGGUCUGAGG-UUCGU 3’
           : :::::::::: ::::
query   3’ UACCCAGACUCCUAAGCU 5’

query=JS16_t2299844, target=FGSG_02063, score=3, range=189-209, strand=1

target  5’ GAAGUCACGACUAGUCCGAAC 3’
           .:::. : :::::::::::::
query   3’ UUUCGCUUCUGAUCAGGCUUG 5’

query=JS16_t3195959, target=FGSG_01605, score=3.5, range=720-739, strand=1

target  5’ GCGAGUGGAGG-GCUCUUCGG 3’
           .::::::::.: :::::::::
query   3’ UGCUCACCUUCGCGAGAAGCC 5’
```

```
query=JS16_t0577373, target=FGSG_04946, score=3, range=801-817, strand=1

target  5’ UAGC-ACUAAUCUACCAG 3’
           :: : :::::.:::::::
query   3’ AUAGUUGAUUGGAUGGUC 5’

query=JS16_t0577373, target=FGSG_04030, score=4, range=2649-2666, strand=1

target  5’ UAUCAACUACUCUACCAA 3’
           ::::::::: .::::::
query   3’ AUAGUUGAUUGGAUGGUC 5’

query=JS16_t0577373, target=FGSG_02876, score=4, range=916-933, strand=1

target  5’ GAUCAACUAAACUGCCAG 3’
            ::::::::: ::.::::
query   3’ AUAGUUGAUUGGAUGGUC 5’

query=JS16_t0577373, target=FGSG_01542, score=4, range=456-473, strand=1

target  5’ UAGCAACCGACCUACCAG 3’
           :: :::: .:::::::::
query   3’ AUAGUUGAUUGGAUGGUC 5’

query=JS16_t0577373, target=FGSG_03904, score=4, range=940-957, strand=1

target  5’ UAUGAACUAACCAACCAU 3’
           ::: :::::::: ::::
query   3’ AUAGUUGAUUGGAUGGUC 5’

query=JS16_t0577373, target=FGSG_10660, score=4, range=1332-1348, strand=1

target  5’ CAUC-ACUAAGCUACCAG 3’
            ::: ::::: :::::::
query   3’ AUAGUUGAUUGGAUGGUC 5’

query=JS16_t0577373, target=FGSG_12575, score=4, range=47-64, strand=-1

target  5’ GAUCAACUAACCAACCAA 3’
            ::::::::::: ::::
query   3’ AUAGUUGAUUGGAUGGUC 5’

query=JS16_t0577373, target=FGSG_01899, score=4, range=3500-3516, strand=-1

target  5’ UAU-AGUUAACCUACCAC 3’
           ::: :..:::::::::
query   3’ AUAGUUGAUUGGAUGGUC 5’

No results for JS16_t0468368
No results for JS16_t0188497
query=JS16_t0686122, target=FGSG_01577, score=3.5, range=1741-1760, strand=1

target  5’ UUGCCAAAAGA-UUCUUCCUC 3’
           :.:::::::::: ::::::::
query   3’ AGCGGUUUUCUAAAGAAGGAA 5’

query=JS16_t0686122, target=FGSG_10027, score=3.5, range=568-587, strand=-1

target  5’ AUACC-AAAGAUUUCUUCCUU 3’
            . :: :::::::::::::::
query   3’ AGCGGUUUUCUAAAGAAGGAA 5’

query=JS16_t0686122, target=FGSG_02123, score=4, range=213-234, strand=1

target  5’ UCGCGUAGCAGGUUUCUUCCUU 3’
           :::: .:. ::.::::::::::
query   3’ AGCG-GUUUUCUAAAGAAGGAA 5’

query=JS16_t0686122, target=FGSG_07786, score=4, range=2386-2406, strand=-1

target  5’ UCGCCAAAAGACUUCCUCCUU 3’
           ::::::::::: ::: :::::
query   3’ AGCGGUUUUCUAAAGAAGGAA 5’

query=JS16_t0686122, target=FGSG_08955, score=4, range=1993-2013, strand=-1

target  5’ CCGUUUCAAGAUUUCUUCCUU 3’
            ::..  ::::::::::::::
query   3’ AGCGGUUUUCUAAAGAAGGAA 5’

query=JS16_t0686122, target=FGSG_13865, score=4, range=1144-1164, strand=-1

target  5’ UGGCCCAAAUAUUUCUUCCUU 3’
           : ::: ::: ::::::::::
query   3’ AGCGGUUUUCUAAAGAAGGAA 5’

query=JS16_t3132369, target=FGSG_06878, score=4, range=719-739, strand=-1

target  5’ CAUCCUUCCAGU-AGCGUUCGU 3’
           ::::::. :::: ::::::::.
query   3’ GUAGGAGCGUCAUUCGCAAGCG 5’

query=JS16_t3195959, target=FGSG_02621, score=4, range=1383-1404, strand=1

target  5’ GCGUGGUGAAAGCGCUCUUCGC 3’
           .:: .::: ::::::::::::
query   3’ UGC-UCACCUUCGCGAGAAGCC 5’

No results for JS16_t3195993
No results for JS16_t1403798
No results for JS16_t0396566
No results for JS16_t3196068
No results for JS16_t0269844
query=JS16_t0396570, target=FGSG_11632, score=2, range=1163-1180, strand=-1

target  5’ UCCUUGACCACUUGUUGA 3’
           : ::::::::::::::
query   3’ AAGAACUGGUGAACAACG 5’

query=JS16_t0396570, target=FGSG_01272, score=3, range=3452-3469, strand=-1

target  5’ UUCUUGACCGCUUGUCGC 3’
           :::::::::.::::: ::
query   3’ AAGAACUGGUGAACAACG 5’

query=JS16_t0396570, target=FGSG_11124, score=3, range=928-945, strand=-1

target  5’ UUCUGGGCCACUUGUUGA 3’
           :::: :.:::::::::
query   3’ AAGAACUGGUGAACAACG 5’

query=JS16_t0396570, target=FGSG_01350, score=3, range=624-641, strand=-1

target  5’ UGCUCGACUACUUGUUGC 3’
           : :: :::.:::::::::
query   3’ AAGAACUGGUGAACAACG 5’

query=JS16_t0396570, target=FGSG_07927, score=3.5, range=679-697, strand=1

target  5’ CUCUUGAACCACUUGUUGU 3’
            ::::: :::::::::::.
query   3’ AAGAAC-UGGUGAACAACG 5’

query=JS16_t0396570, target=FGSG_07119, score=3.5, range=759-776, strand=-1

target  5’ AUUUUGACUACUUGUUGA 3’
            :.:::::.::::::::
query   3’ AAGAACUGGUGAACAACG 5’

query=JS16_t0396570, target=FGSG_06904, score=3.5, range=134-152, strand=-1

target  5’ GUCUUGGACCAUUUGUUGU 3’
            :::: :::::.::::::.
query   3’ AAGAA-CUGGUGAACAACG 5’

query=JS16_t0396570, target=FGSG_11814, score=3.5, range=1878-1895, strand=-1

target  5’ GCAUUGACCACUUGUUGU 3’
              ::::::::::::::.
query   3’ AAGAACUGGUGAACAACG 5’

query=JS16_t0396570, target=FGSG_13833, score=4, range=359-376, strand=1

target  5’ GGCUUGACCGUUUGUUGC 3’
             :::::::..:::::::
query   3’ AAGAACUGGUGAACAACG 5’

query=JS16_t0396570, target=FGSG_00067, score=4, range=543-560, strand=1

target  5’ AUCUUGGCCACUUGGUGC 3’
            :::::.::::::: :::
query   3’ AAGAACUGGUGAACAACG 5’

query=JS16_t0396570, target=FGSG_03622, score=4, range=1061-1079, strand=1

target  5’ UGCUUGCACCGCUUGUUGC 3’
           : :::: :::.:::::::::
query   3’ AAGAAC-UGGUGAACAACG 5’

query=JS16_t0396570, target=FGSG_08229, score=4, range=897-914, strand=1

target  5’ UGCGCGACUACUUGUUGC 3’
           : :  :::.:::::::::
query   3’ AAGAACUGGUGAACAACG 5’

query=JS16_t0396570, target=FGSG_10511, score=4, range=1163-1179, strand=1

target  5’ AUC-UGACCACUGGUUGC 3’
            :: :::::::: :::::
query   3’ AAGAACUGGUGAACAACG 5’

query=JS16_t0396570, target=FGSG_05705, score=4, range=309-326, strand=1
```

```
query=JS16_t0394440, target=FGSG_05511, score=3, range=1747-1764, strand=1

target  5’ AGUGCAUACGCGACUCCU 3’
            ::: ::::.::::::::
query   3’ CCACUUAUGUGCUGAGGA 5’

query=JS16_t0394440, target=FGSG_05968, score=3, range=340-356, strand=1

target  5’ GGUGUAUACACGA-UCCU 3’
           :::: :::::::: ::::
query   3’ CCACUUAUGUGCUGAGGA 5’

query=JS16_t0394440, target=FGSG_10682, score=3.5, range=193-210, strand=1

target  5’ GGUGGAUAUGCGAUUCCU 3’
           ::::.:::..:::.::::
query   3’ CCACUUAUGUGCUGAGGA 5’

query=JS16_t0394440, target=FGSG_09567, score=3.5, range=1683-1700, strand=-1

target  5’ GCUUGAUACACGACUCCA 3’
           : : .::::::::::::
query   3’ CCACUUAUGUGCUGAGGA 5’

query=JS16_t0394440, target=FGSG_10228, score=4, range=2584-2601, strand=1

target  5’ CGUGAGUACAUGGCUCCU 3’
            ::::.::::.:.:::::
query   3’ CCACUUAUGUGCUGAGGA 5’

query=JS16_t0394440, target=FGSG_07810, score=4, range=946-963, strand=1

target  5’ GGCGAGUACACGACUUCC 3’
           :: ::.:::::::::.:
query   3’ CCACUUAUGUGCUGAGGA 5’

query=JS16_t0394440, target=FGSG_06449, score=4, range=388-406, strand=1

target  5’ GGUGAUUACACUGACUUCU 3’
           ::::: ::::: ::::.::
query   3’ CCACUUAUGUG-CUGAGGA 5’

query=JS16_t0394440, target=FGSG_03692, score=4, range=43-60, strand=-1

target  5’ AGUGAAUGCACGGCUCCC 3’
            ::::::.::::.::::
query   3’ CCACUUAUGUGCUGAGGA 5’

query=JS16_t0394440, target=FGSG_07328, score=4, range=1233-1249, strand=-1

target  5’ GGU-AGUACACGACUUUU 3’
           ::: :.:::::::::..:
query   3’ CCACUUAUGUGCUGAGGA 5’

query=JS16_t0394440, target=FGSG_02238, score=4, range=860-878, strand=-1

target  5’ GGUGGAAUAGGCGACUCCU 3’
           ::: ::::: .::::::::
query   3’ CCA-CUUAUGUGCUGAGGA 5’

query=JS16_t0394440, target=FGSG_00697, score=4, range=2081-2098, strand=-1

target  5’ CCUGCAUACGCGACUCCU 3’
             :: ::::.::::::::
query   3’ CCACUUAUGUGCUGAGGA 5’

query=JS16_t0394440, target=FGSG_04351, score=4, range=1375-1392, strand=-1

target  5’ CGUCAAUACACGCCUCCU 3’
            :: :::::::: :::::
query   3’ CCACUUAUGUGCUGAGGA 5’

query=JS16_t0394440, target=FGSG_09491, score=4, range=2081-2097, strand=-1

target  5’ GGUGAAUCCACG-CUCCU 3’
           ::::::: :::: :::::
query   3’ CCACUUAUGUGCUGAGGA 5’

query=JS16_t0394440, target=FGSG_11463, score=4, range=454-471, strand=-1

target  5’ GCUCAAUACACGACUUCG 3’
           : : ::::::::::.:
query   3’ CCACUUAUGUGCUGAGGA 5’

query=JS16_t0394440, target=FGSG_05715, score=4, range=583-599, strand=-1

target  5’ GGUGUAUACAUGAC-CCU 3’
           :::: :::::.::: :::
query   3’ CCACUUAUGUGCUGAGGA 5’

query=JS16_t0394440, target=FGSG_04524, score=4, range=135-152, strand=-1

target  5’ UUGCGGACCACUUGUUGG 3’
           ::   ::::::::::::
query   3’ AAGAACUGGUGAACAACG 5’

query=JS16_t0396570, target=FGSG_10074, score=4, range=557-575, strand=1

target  5’ UUCGUGGCUCACUUGUUGC 3’
           ::: ::.: ::::::::::
query   3’ AAGAACUG-GUGAACAACG 5’

query=JS16_t0396570, target=FGSG_01144, score=4, range=1321-1337, strand=1

target  5’ UUCUUG-CCGCUUGUUGA 3’
           :::::: ::.::::::::
query   3’ AAGAACUGGUGAACAACG 5’

query=JS16_t0396570, target=FGSG_07053, score=4, range=1690-1706, strand=1

target  5’ CCCUUGACCA-UUGUUGC 3’
             :::::::: :::::::
query   3’ AAGAACUGGUGAACAACG 5’

query=JS16_t0396570, target=FGSG_02173, score=4, range=19-35, strand=1

target  5’ UUCUUGGCU-CUUGUUGC 3’
           ::::::.:. ::::::::
query   3’ AAGAACUGGUGAACAACG 5’

query=JS16_t0396570, target=FGSG_12424, score=4, range=640-656, strand=1

target  5’ AUCUUGA-CAUUUGUUGC 3’
            :::::: ::.:::::::
query   3’ AAGAACUGGUGAACAACG 5’

query=JS16_t0396570, target=FGSG_03034, score=4, range=1733-1751, strand=-1

target  5’ UUCUUGGUCACAUUGUUGC 3’
           ::::::..::: :::::::
query   3’ AAGAACUGGUG-AACAACG 5’

query=JS16_t0396570, target=FGSG_09949, score=4, range=1889-1907, strand=-1

target  5’ UUCUUGAUCAUUUUGUUGC 3’
           :::::::.:: .:::::::
query   3’ AAGAACUGGU-GAACAACG 5’

query=JS16_t0396570, target=FGSG_06874, score=4, range=2115-2132, strand=-1

target  5’ AGCUUGGCCAUUUGUUGC 3’
             ::::.:::.:::::::
query   3’ AAGAACUGGUGAACAACG 5’

query=JS16_t0396570, target=FGSG_09801, score=4, range=1529-1546, strand=-1

target  5’ CUCGUGAUCGCUUGUUGC 3’
            :: :::.:.::::::::
query   3’ AAGAACUGGUGAACAACG 5’

query=JS16_t0396570, target=FGSG_08728, score=4, range=2371-2388, strand=-1

target  5’ UUCAUGGCCACUUGCUGC 3’
           ::: ::.::::::: :::
query   3’ AAGAACUGGUGAACAACG 5’

query=JS16_t0396570, target=FGSG_05392, score=4, range=1071-1088, strand=-1

target  5’ UACUUGGCAACUUGUUGC 3’
           : ::::.: :::::::::
query   3’ AAGAACUGGUGAACAACG 5’

query=JS16_t0396570, target=FGSG_04152, score=4, range=1099-1116, strand=-1

target  5’ UUCGUGAUCACUGGUUGC 3’
           ::: :::.:::: :::::
query   3’ AAGAACUGGUGAACAACG 5’

query=JS16_t0396570, target=FGSG_08793, score=4, range=839-856, strand=-1

target  5’ CAUUCGACCACUUGUUGU 3’
             .: ::::::::::::.
query   3’ AAGAACUGGUGAACAACG 5’

query=JS16_t0396570, target=FGSG_07236, score=4, range=223-240, strand=-1

target  5’ GUCUCGACCACUUGGUGC 3’
            ::: ::::::::: :::
query   3’ AAGAACUGGUGAACAACG 5’

query=JS16_t0396570, target=FGSG_12369, score=4, range=2093-2111, strand=-1

target  5’ UUCUUGGACCACUUGUAGG 3’
           ::::: :::::::::: :
```

```
target  5’ CCUGUAUACACGACUCCA 3’
             :: ::::::::::::
query   3’ CCACUUAUGUGCUGAGGA 5’

query=JS16_t0394440, target=FGSG_02589, score=4, range=573-589, strand=-1

target  5’ UGUGAA-ACACGACUCCG 3’
            ::::: ::::::::::
query   3’ CCACUUAUGUGCUGAGGA 5’

query=JS16_t0394440, target=FGSG_04753, score=4, range=243-259, strand=-1

target  5’ GGU-CAUACGCGACUCCC 3’
           :::  ::::.:::::::
query   3’ CCACUUAUGUGCUGAGGA 5’

query=JS16_t0686128, target=FGSG_07383, score=3, range=2647-2663, strand=-1

target  5’ ACCG-UGUACUCGAUGUC 3’
           :::: :::::.::::::
query   3’ UGGCAACAUGGGCUACAC 5’

query=JS16_t0686128, target=FGSG_04875, score=3.5, range=910-927, strand=1

target  5’ AUCGUUGUACGCGAUGUC 3’
           :.:::::::: ::::::
query   3’ UGGCAACAUGGGCUACAC 5’

query=JS16_t0686128, target=FGSG_12080, score=3.5, range=871-888, strand=1

target  5’ ACUGUUGUACCGGAUGUA 3’
           ::.:::::::: :::::
query   3’ UGGCAACAUGGGCUACAC 5’

query=JS16_t0686128, target=FGSG_02224, score=3.5, range=924-942, strand=1

target  5’ GCCGAUGUACCCGAUCGUG 3’
           .::: :::::::::: :::
query   3’ UGGCAACAUGGGCUA-CAC 5’

query=JS16_t0686128, target=FGSG_03151, score=3.5, range=1056-1074, strand=-1

target  5’ AUCGUCUGUACUUGAUGUG 3’
           :.::: :::::..::::::
query   3’ UGGCA-ACAUGGGCUACAC 5’

query=JS16_t0686128, target=FGSG_01359, score=3.5, range=1021-1038, strand=-1

target  5’ AUCUUUGUACCCGAUGAG 3’
           :.: :::::::::::: :
query   3’ UGGCAACAUGGGCUACAC 5’

query=JS16_t0686128, target=FGSG_13318, score=4, range=637-654, strand=1

target  5’ CUUGUCGUACCCGAUGUG 3’
            ..:: ::::::::::::
query   3’ UGGCAACAUGGGCUACAC 5’

query=JS16_t0686128, target=FGSG_08248, score=4, range=1931-1948, strand=1

target  5’ ACCUUAGUACUCGAUGUG 3’
           ::: : ::::.:::::::
query   3’ UGGCAACAUGGGCUACAC 5’

query=JS16_t0686128, target=FGSG_12731, score=4, range=280-297, strand=1

target  5’ GCUGCUGUACCGGAUGUG 3’
           .:.: :::::: ::::::
query   3’ UGGCAACAUGGGCUACAC 5’

query=JS16_t0686128, target=FGSG_09182, score=4, range=6230-6246, strand=1

target  5’ GUC-UUGUACUCGAUGUA 3’
           ..: ::::::.::::::
query   3’ UGGCAACAUGGGCUACAC 5’

query=JS16_t0686128, target=FGSG_11656, score=4, range=1460-1476, strand=1

target  5’ ACC-UUGUACCUGAUUUG 3’
           ::: :::::::.::: ::
query   3’ UGGCAACAUGGGCUACAC 5’

query=JS16_t0686128, target=FGSG_09064, score=4, range=96-112, strand=1

target  5’ ACC-UUGUACUCAAUGUG 3’
           ::: ::::::.: :::::
query   3’ UGGCAACAUGGGCUACAC 5’

query=JS16_t0686128, target=FGSG_02664, score=4, range=574-591, strand=1

target  5’ AAAGGUGUACCCGAUGUU 3’
query   3’ AAGAA-CUGGUGAACAACG 5’

query=JS16_t0396570, target=FGSG_12589, score=4, range=1628-1646, strand=-1

target  5’ UUCUCGUACCACUUGUUGG 3’
           :::: : :::::::::::
query   3’ AAGAAC-UGGUGAACAACG 5’

query=JS16_t0396570, target=FGSG_08278, score=4, range=2005-2022, strand=-1

target  5’ AUCCCGACCAUUUGUUGC 3’
            ::  :::::.:::::::
query   3’ AAGAACUGGUGAACAACG 5’

query=JS16_t0396570, target=FGSG_10115, score=4, range=961-977, strand=-1

target  5’ ACCUUGACCACUUG-UGC 3’
             :::::::::::: :::
query   3’ AAGAACUGGUGAACAACG 5’

query=JS16_t0396570, target=FGSG_05076, score=4, range=164-180, strand=-1

target  5’ UGCUUGACCA-UUGUUGA 3’
           : :::::::: ::::::
query   3’ AAGAACUGGUGAACAACG 5’

query=JS16_t0396570, target=FGSG_00065, score=4, range=812-828, strand=-1

target  5’ UUCCUGACCA-UUGUUGA 3’
           ::: :::::: ::::::
query   3’ AAGAACUGGUGAACAACG 5’

query=JS16_t0396570, target=FGSG_10420, score=4, range=380-396, strand=-1

target  5’ AUCUUGACC-CUUGUUGG 3’
            :::::::: :::::::
query   3’ AAGAACUGGUGAACAACG 5’

query=JS16_t2300086, target=FGSG_09994, score=3, range=2348-2365, strand=1

target  5’ UGUUCAGCUUCGGCGGGG 3’
           :::::::::::.:::.:
query   3’ ACAAGUCGAAGUCGCUCA 5’

query=JS16_t2300086, target=FGSG_13878, score=3, range=2457-2474, strand=1

target  5’ AGUUCAGCUUCACCGAGU 3’
            ::::::::::: :::::
query   3’ ACAAGUCGAAGUCGCUCA 5’

query=JS16_t2300086, target=FGSG_11663, score=3, range=489-505, strand=1

target  5’ UG-UCAGCUUUGGCGAGU 3’
           :: :::::::..::::::
query   3’ ACAAGUCGAAGUCGCUCA 5’

query=JS16_t2300086, target=FGSG_01204, score=3, range=2222-2239, strand=1

target  5’ UGCUCAGCUUCAUCGAGU 3’
           :: ::::::::: :::::
query   3’ ACAAGUCGAAGUCGCUCA 5’

query=JS16_t2300086, target=FGSG_08290, score=3, range=198-215, strand=1

target  5’ UCCUCAGCUUCAGCGAGA 3’
           :  :::::::::::::
query   3’ ACAAGUCGAAGUCGCUCA 5’

query=JS16_t2300086, target=FGSG_06265, score=3, range=2067-2084, strand=-1

target  5’ UGUUCAGCUUCAUCGAGC 3’
           :::::::::::: ::::
query   3’ ACAAGUCGAAGUCGCUCA 5’

query=JS16_t2300086, target=FGSG_03534, score=3, range=695-712, strand=-1

target  5’ UGUCCAACUUCAGCGAGU 3’
           ::: :: :::::::::::
query   3’ ACAAGUCGAAGUCGCUCA 5’

query=JS16_t2300086, target=FGSG_10661, score=3, range=94-110, strand=-1

target  5’ UGUUCAGCUUCAGC-AGC 3’
           :::::::::::::: ::
query   3’ ACAAGUCGAAGUCGCUCA 5’

query=JS16_t2300086, target=FGSG_08400, score=3, range=1774-1790, strand=-1

target  5’ UCUUCAGCUUCAG-GAGU 3’
           : ::::::::::: ::::
query   3’ ACAAGUCGAAGUCGCUCA 5’
```

```
           :  : ::::::::::::
query   3’ UGGCAACAUGGGCUACAC 5’

query=JS16_t0686128, target=FGSG_06673, score=4, range=1681-1697, strand=1

target  5’ ACCAUUG-ACCCGAUGUC 3’
           ::: ::: :::::::::
query   3’ UGGCAACAUGGGCUACAC 5’

query=JS16_t0686128, target=FGSG_06945, score=4, range=1105-1122, strand=-1

target  5’ ACCGUUGUAUCCGGUGGG 3’
           :::::::::.:::.:: :
query   3’ UGGCAACAUGGGCUACAC 5’

query=JS16_t0686128, target=FGSG_11395, score=4, range=1128-1145, strand=-1

target  5’ CCCGUUGUGCCAGAUGUG 3’
            :::::::.:: ::::::
query   3’ UGGCAACAUGGGCUACAC 5’

query=JS16_t0686128, target=FGSG_04689, score=4, range=766-783, strand=-1

target  5’ AUUCUUGUACUCGAUGUC 3’
           :.. ::::::.::::::
query   3’ UGGCAACAUGGGCUACAC 5’

query=JS16_t0686128, target=FGSG_05111, score=4, range=480-498, strand=-1

target  5’ ACCGUUGGACCCGAGUGUG 3’
           ::::::: :::::: ::::
query   3’ UGGCAACAUGGGCU-ACAC 5’

query=JS16_t0686128, target=FGSG_00437, score=4, range=1860-1877, strand=-1

target  5’ GCUGCUGCACCCGAUGUG 3’
           .:.: :: ::::::::::
query   3’ UGGCAACAUGGGCUACAC 5’

query=JS16_t0686128, target=FGSG_03736, score=4, range=564-582, strand=-1

target  5’ ACCAGUUGUCCCCGAUGUA 3’
           ::: ::::: ::::::::
query   3’ UGG-CAACAUGGGCUACAC 5’

query=JS16_t0577397, target=FGSG_04042, score=3.5, range=2478-2498, strand=-1

target  5’ UCGGCUCGAUUCUUGUAGUAC 3’
           .::::: :::::: :::::::
query   3’ GGCCGA-CUAAGACCAUCAUG 5’

No results for JS16_t1339814
No results for JS16_t3132475
query=JS16_t0202165, target=FGSG_13904, score=4, range=391-415, strand=-1

target  5’ UCCAGC-CCAAGGCAACGGGUCCACU 3’
           : : :: ::::..:::::::::::::
query   3’ AUGACGCGGUUUUGUUGCCCAGGUGA 5’

No results for JS16_t3132488
query=JS16_t2236440, target=FGSG_11400, score=2.5, range=260-278, strand=1

target  5’ GAUCAGUCAAUUGGCACAA 3’
            : ::.:::::::::::::
query   3’ AUUGUUAGUUAACCGUGUU 5’

query=JS16_t2236440, target=FGSG_09193, score=3, range=1037-1055, strand=1

target  5’ UAACAAUUAAUUGGUACAC 3’
           :::::::.::::::.:::
query   3’ AUUGUUAGUUAACCGUGUU 5’

query=JS16_t2236440, target=FGSG_00034, score=3, range=797-814, strand=-1

target  5’ UGAC-AUCAAUUGGCGCAG 3’
           :.:: ::::::::::.::.
query   3’ AUUGUUAGUUAACCGUGUU 5’

query=JS16_t2236440, target=FGSG_11603, score=3.5, range=1670-1688, strand=1

target  5’ AGGUUAUCAAUUGGCACAA 3’
            ...  ::::::::::::::
query   3’ AUUGUUAGUUAACCGUGUU 5’

query=JS16_t2236440, target=FGSG_02690, score=3.5, range=361-379, strand=-1

target  5’ UCUCAAUCAAUUGGCGCAG 3’
           :   ::::::::::::.::.
query   3’ AUUGUUAGUUAACCGUGUU 5’

query=JS16_t2236440, target=FGSG_11578, score=4, range=826-844, strand=1

query=JS16_t2300086, target=FGSG_09414, score=3.5, range=900-917, strand=-1

target  5’ UGUGUAGCUUCGGCGAGC 3’
           ::: .::::::.:::::
query   3’ ACAAGUCGAAGUCGCUCA 5’

query=JS16_t2300086, target=FGSG_05345, score=4, range=969-986, strand=1

target  5’ CUUUCAGUUUCGGCGAGU 3’
             :::::.:::.::::::
query   3’ ACAAGUCGAAGUCGCUCA 5’

query=JS16_t2300086, target=FGSG_06493, score=4, range=1440-1456, strand=1

target  5’ UGUU-GGCUUCGGUGAGU 3’
           :::: .:::::.:.::::
query   3’ ACAAGUCGAAGUCGCUCA 5’

query=JS16_t2300086, target=FGSG_03562, score=4, range=816-833, strand=1

target  5’ UGUUCAGCUUCAACGACU 3’
           :::::::::::: ::: :
query   3’ ACAAGUCGAAGUCGCUCA 5’

query=JS16_t2300086, target=FGSG_00629, score=4, range=614-631, strand=1

target  5’ UGUACUGCUUCGGCGAGU 3’
           ::: : :::::.::::::
query   3’ ACAAGUCGAAGUCGCUCA 5’

query=JS16_t2300086, target=FGSG_11268, score=4, range=8945-8962, strand=1

target  5’ ACUUCAGCUACAGCGAGU 3’
             ::::::: ::::::::
query   3’ ACAAGUCGAAGUCGCUCA 5’

query=JS16_t2300086, target=FGSG_07000, score=4, range=697-714, strand=1

target  5’ CUUUCAGCUUCAGCAAGU 3’
             :::::::::::: :::
query   3’ ACAAGUCGAAGUCGCUCA 5’

query=JS16_t2300086, target=FGSG_07829, score=4, range=230-247, strand=1

target  5’ AGUCCAGCUUCGGCGAGC 3’
            :: :::::::.:::::
query   3’ ACAAGUCGAAGUCGCUCA 5’

query=JS16_t2300086, target=FGSG_10885, score=4, range=620-637, strand=1

target  5’ AGCUCGGCUUCAGCGAGC 3’
            : ::.:::::::::::
query   3’ ACAAGUCGAAGUCGCUCA 5’

query=JS16_t2300086, target=FGSG_02315, score=4, range=15897-15913, strand=1

target  5’ UGUU-GGCUUCGGCGAGA 3’
           :::: .:::::.:::::
query   3’ ACAAGUCGAAGUCGCUCA 5’

query=JS16_t2300086, target=FGSG_00690, score=4, range=550-567, strand=1

target  5’ UGGUCAGCUUCAUCGAGG 3’
           :: ::::::::: ::::
query   3’ ACAAGUCGAAGUCGCUCA 5’

query=JS16_t2300086, target=FGSG_02727, score=4, range=405-422, strand=1

target  5’ UAUUCAGCUUCAGCCAGG 3’
           : :::::::::::: ::
query   3’ ACAAGUCGAAGUCGCUCA 5’

query=JS16_t2300086, target=FGSG_09408, score=4, range=1932-1949, strand=1

target  5’ UGUUGAGCUUCAGCAAGC 3’
           :::: ::::::::: ::
query   3’ ACAAGUCGAAGUCGCUCA 5’

query=JS16_t2300086, target=FGSG_08262, score=4, range=128-145, strand=1

target  5’ UAUUCAGCUUCAGCCAGA 3’
           : :::::::::::: ::
query   3’ ACAAGUCGAAGUCGCUCA 5’

query=JS16_t2300086, target=FGSG_12134, score=4, range=366-382, strand=1

target  5’ UGAUCAGUUUCAGC-AGU 3’
           :: ::::.:::::: :::
query   3’ ACAAGUCGAAGUCGCUCA 5’

query=JS16_t2300086, target=FGSG_04561, score=4, range=1916-1933, strand=1
```

```
target  5' UGGCAAUCAGUUGGCACCA 3'
           :..:::::.::::::: :
query   3' AUUGUUAGUUAACCGUGUU 5'

query=JS16_t2236440, target=FGSG_07554, score=4, range=67-86, strand=1

target  5' UACCAAACAAUUGGCCACAA 3'
           :: ::: ::::::: :::::
query   3' AUUGUUAGUUAACC-GUGUU 5'

query=JS16_t2236440, target=FGSG_01548, score=4, range=698-715, strand=-1

target  5' UGGCAG-CAAUUGGCACAG 3'
           :..::. :::::::::::.
query   3' AUUGUUAGUUAACCGUGUU 5'

query=JS16_t2236440, target=FGSG_02990, score=4, range=716-733, strand=-1

target  5' CAGU-AUCGAUUGGCACAA 3'
            :.. :::.::::::::::
query   3' AUUGUUAGUUAACCGUGUU 5'

query=JS16_t2236440, target=FGSG_07053, score=4, range=2150-2167, strand=-1

target  5' UGACCAUCAA-UGGCACAG 3'
           :.:: ::::: :::::::.
query   3' AUUGUUAGUUAACCGUGUU 5'

No results for JS16_t3132542
query=JS16_t4029051, target=FGSG_10081, score=4, range=253-278, strand=-1

target  5' GAGAGCUUGGCUGGUCGAAUCUGAUC 3'
           .:.:: :::::::::::::::: ::
query   3' UUUUCAAACCGACCAGCUUAGAC-AG 5'

No results for JS16_t0202166
No results for JS16_t2236443
No results for JS16_t2236463
query=JS16_t3132569, target=FGSG_13118, score=3.5, range=1058-1077, strand=-1

target  5' CUCGGCACGUCGUGGGACAG 3'
             :: ::::::::::::::.
query   3' UUGCGGUGCAGCACCCUGUU 5'

query=JS16_t3132569, target=FGSG_01962, score=4, range=1060-1080, strand=1

target  5' AACGCCUACGUCGUCGGACGA 3'
           :::::: ::::::: ::::.:
query   3' UUGCGG-UGCAGCACCCUGUU 5'

query=JS16_t3132569, target=FGSG_00196, score=4, range=399-418, strand=-1

target  5' GAUGCAACGUCGUGGGACUA 3'
           .:.:: :::::::::::: :
query   3' UUGCGGUGCAGCACCCUGUU 5'

No results for JS16_t0686152
No results for JS16_t0686154
No results for JS16_t4029120
No results for JS16_t0577425
query=JS16_t0795466, target=FGSG_12512, score=3, range=229-249, strand=1

target  5' GAAAAAGAUGUCGUGUUCAAU 3'
           ::  ::: :::::::::::::
query   3' CUACUUCAACAGCACAAGUUA 5'

query=JS16_t0795466, target=FGSG_13783, score=3.5, range=19372-19391, strand=

target  5' GAUGAGG-UGUCGUGGUCAAU 3'
           :::::.: ::::::: :::::
query   3' CUACUUCAACAGCACAAGUUA 5'

query=JS16_t4029242, target=FGSG_10081, score=1.5, range=255-273, strand=1

target  5' UCAGAUUCGACCAGCCAAG 3'
            :::::::::::::::::.
query   3' UGUCUAAGCUGGUCGGUUU 5'

query=JS16_t4029242, target=FGSG_12945, score=3, range=26-45, strand=1

target  5' GUAGUUAUCGACCAGCCAAA 3'
           ..:: : :::::::::::::
query   3' UGUCUA-AGCUGGUCGGUUU 5'

query=JS16_t4029242, target=FGSG_04920, score=3, range=4-21, strand=1

target  5' ACAGACUCGA-CAGCCAAA 3'
           ::::: :::: ::::::::
query   3' UGUCUAAGCUGGUCGGUUU 5'

query=JS16_t4029242, target=FGSG_11614, score=3.5, range=633-651, strand=1
```

```
target  5' UCCACAGCUUCAGCGAGG 3'
           :   :::::::::::::
query   3' ACAAGUCGAAGUCGCUCA 5'

query=JS16_t2300086, target=FGSG_08673, score=4, range=1206-1223, strand=1

target  5' UGAACAGCCUCAGCGAGU 3'
           ::  :::: :::::::::
query   3' ACAAGUCGAAGUCGCUCA 5'

query=JS16_t2300086, target=FGSG_01386, score=4, range=1557-1574, strand=1

target  5' GGCGCAGCUUCAGCGAGA 3'
            :  :::::::::::::
query   3' ACAAGUCGAAGUCGCUCA 5'

query=JS16_t2300086, target=FGSG_09615, score=4, range=2002-2020, strand=1

target  5' GGUGCAAGCUUCAGCGAGA 3'
            :: : ::::::::::::
query   3' ACAAG-UCGAAGUCGCUCA 5'

query=JS16_t2300086, target=FGSG_12470, score=4, range=522-538, strand=1

target  5' UG-UCAGCGUCAGCGAGC 3'
           :: ::::: ::::::::
query   3' ACAAGUCGAAGUCGCUCA 5'

query=JS16_t2300086, target=FGSG_01119, score=4, range=825-841, strand=1

target  5' UGCUCAGCUUCAG-GAGG 3'
           :: ::::::::::: :::
query   3' ACAAGUCGAAGUCGCUCA 5'

query=JS16_t2300086, target=FGSG_08133, score=4, range=96-112, strand=1

target  5' CGUUCAGCUUC-GCGAGC 3'
            :::::::::: :::::
query   3' ACAAGUCGAAGUCGCUCA 5'

query=JS16_t2300086, target=FGSG_02232, score=4, range=539-556, strand=-1

target  5' UGUUCGGCUGCAGCGAGG 3'
           :::::.::: :::::::
query   3' ACAAGUCGAAGUCGCUCA 5'

query=JS16_t2300086, target=FGSG_04680, score=4, range=465-482, strand=-1

target  5' UCAUCAGCUUCGGCGGGU 3'
           :  ::::::::.:::.::
query   3' ACAAGUCGAAGUCGCUCA 5'

query=JS16_t2300086, target=FGSG_04948, score=4, range=3485-3502, strand=-1

target  5' UGUACAGCUUCAGCGAAG 3'
           ::: ::::::::::::
query   3' ACAAGUCGAAGUCGCUCA 5'

query=JS16_t2300086, target=FGSG_05416, score=4, range=866-883, strand=-1

target  5' UGUACAGCUUCAGCGACC 3'
           ::: ::::::::::::
query   3' ACAAGUCGAAGUCGCUCA 5'

query=JS16_t2300086, target=FGSG_11994, score=4, range=11-28, strand=-1

target  5' GGUCCAGCUUCAGCGAUU 3'
            :: ::::::::::::: :
query   3' ACAAGUCGAAGUCGCUCA 5'

query=JS16_t2300086, target=FGSG_08170, score=4, range=911-928, strand=-1

target  5' UGAUCACCUUCAGCGAGC 3'
           :: ::: ::::::::::
query   3' ACAAGUCGAAGUCGCUCA 5'

query=JS16_t2300086, target=FGSG_02090, score=4, range=69-86, strand=-1

target  5' UGAACAGCUUUAGCGAGA 3'
           ::  ::::::.::::::
query   3' ACAAGUCGAAGUCGCUCA 5'

query=JS16_t2300086, target=FGSG_09815, score=4, range=33-50, strand=-1

target  5' GGUGAAGCUUCAGCGGGU 3'
            ::  ::::::::::.::
query   3' ACAAGUCGAAGUCGCUCA 5'

query=JS16_t2300086, target=FGSG_04212, score=4, range=113-130, strand=-1

target  5' CGUAAAGCUUCAGCGGGU 3'
            ::  ::::::::::.::
```

```
target  5’ CUGGGAUCGACCAGCCAAA 3’
            ..:. :::::::::::::
query   3’ UGUCUAAGCUGGUCGGUUU 5’

query=JS16_t4029242, target=FGSG_06793, score=3.5, range=2213-2231, strand=1

target  5’ AAGGAAUCGACCAGCCAGA 3’
           : .:: :::::::::::.:
query   3’ UGUCUAAGCUGGUCGGUUU 5’

query=JS16_t4029242, target=FGSG_01790, score=3.5, range=4814-4832, strand=1

target  5’ CUAUAUUCGACCAGCCAAU 3’
            .: ::::::::::::::
query   3’ UGUCUAAGCUGGUCGGUUU 5’

query=JS16_t4029242, target=FGSG_13795, score=3.5, range=70-87, strand=1

target  5’ ACA-AUUCUACCAGCCAAG 3’
           ::: :::: :::::::::.
query   3’ UGUCUAAGCUGGUCGGUUU 5’

query=JS16_t4029242, target=FGSG_05505, score=3.5, range=310-328, strand=-1

target  5’ GUAGAUUCGACCAGCCUAG 3’
           ..:::::::::::::: :.
query   3’ UGUCUAAGCUGGUCGGUUU 5’

query=JS16_t4029242, target=FGSG_02641, score=3.5, range=381-399, strand=-1

target  5’ AAAGAAUCGGCCAGCCAAG 3’
           : ::: :::.::::::::.
query   3’ UGUCUAAGCUGGUCGGUUU 5’

query=JS16_t4029242, target=FGSG_04291, score=3.5, range=3684-3701, strand=-1

target  5’ GCAGAGU-GACCAGCCAAA 3’
           .:::: : :::::::::::
query   3’ UGUCUAAGCUGGUCGGUUU 5’

query=JS16_t4029242, target=FGSG_05706, score=3.5, range=534-551, strand=-1

target  5’ ACA-AUUCGACCAGCCCAG 3’
           ::: ::::::::::::: :.
query   3’ UGUCUAAGCUGGUCGGUUU 5’

query=JS16_t4029242, target=FGSG_12187, score=4, range=311-329, strand=1

target  5’ CCAGAUUCGGCCCGCCAAA 3’
            ::::::::.:: ::::::
query   3’ UGUCUAAGCUGGUCGGUUU 5’

query=JS16_t4029242, target=FGSG_07212, score=4, range=1793-1810, strand=1

target  5’ AUGGA-UCGACCGGCCAGA 3’
           :..:: ::::::.::::.:
query   3’ UGUCUAAGCUGGUCGGUUU 5’

query=JS16_t4029242, target=FGSG_08223, score=4, range=112-131, strand=1

target  5’ AUGGACUUCGACCAGCCAUA 3’
           :..:: :::::::::::: :
query   3’ UGUCU-AAGCUGGUCGGUUU 5’

query=JS16_t4029242, target=FGSG_10294, score=4, range=336-354, strand=1

target  5’ AGGAAUUCGGCCAGCCAAG 3’
           : .  :::::.::::::::.
query   3’ UGUCUAAGCUGGUCGGUUU 5’

query=JS16_t4029242, target=FGSG_07222, score=4, range=1782-1799, strand=1

target  5’ ACAUA-UCGACCAGCUGAA 3’
           ::: : ::::::::::..::
query   3’ UGUCUAAGCUGGUCGGUUU 5’

query=JS16_t4029242, target=FGSG_01558, score=4, range=956-975, strand=1

target  5’ GCAUCUCUCGACCAGCCAAG 3’
           .::  : ::::::::::::.
query   3’ UGUCUA-AGCUGGUCGGUUU 5’

query=JS16_t4029242, target=FGSG_10564, score=4, range=2004-2022, strand=-1

target  5’ ACAGAGUUGAUCAGUCAAA 3’
           ::::: :.::.:::.::::
query   3’ UGUCUAAGCUGGUCGGUUU 5’

query=JS16_t4029242, target=FGSG_05236, score=4, range=1476-1494, strand=-1

target  5’ GCGGGUGCGACCAGCCAAG 3’
           .:.:.: ::::::::::.
```

```
query   3’ ACAAGUCGAAGUCGCUCA 5’

query=JS16_t2300086, target=FGSG_02056, score=4, range=201-217, strand=-1

target  5’ UG-UCAGCUUCAGAGAGG 3’
           :: :::::::::: :::
query   3’ ACAAGUCGAAGUCGCUCA 5’

query=JS16_t2300086, target=FGSG_00288, score=4, range=340-356, strand=-1

target  5’ UGGUCAGCUUCAGC-AGC 3’
           :: ::::::::::: ::
query   3’ ACAAGUCGAAGUCGCUCA 5’

query=JS16_t2300086, target=FGSG_05899, score=4, range=1555-1571, strand=-1

target  5’ UCUUGAGCUUCAGC-AGU 3’
           : :: ::::::::: :::
query   3’ ACAAGUCGAAGUCGCUCA 5’

query=JS16_t0146657, target=FGSG_00912, score=3.5, range=2110-2130, strand=-1

target  5’ GUCCUGAGCAACAAGUCGUCG 3’
           :: ::::::::::::: :::.
query   3’ CACGACUCGUUGUUCACCAGU 5’

query=JS16_t0146657, target=FGSG_01819, score=4, range=554-573, strand=1

target  5’ UUCCUGA-CAACGAGUGGUCA 3’
            : :::: ::::.::::::::
query   3’ CACGACUCGUUGUUCACCAGU 5’

query=JS16_t3196201, target=FGSG_00047, score=4, range=1171-1191, strand=-1

target  5’ AUGGAUCGUAGUAUGAGUAAA 3’
           :..::  ::::::.:::::::
query   3’ UGUCUUCCAUCAUGCUCAUUU 5’

query=JS16_t3196225, target=FGSG_05900, score=2, range=529-548, strand=1

target  5’ GAGAACGUUGGUUUCCGGUA 3’
           :::::  :::::::::::::
query   3’ CUCUUUAAACCAAAGGCCAU 5’

query=JS16_t3196225, target=FGSG_00226, score=4, range=1366-1385, strand=1

target  5’ GAGAAGCUUGGUUUCCAGUG 3’
           :::::. ::::::::: ::.
query   3’ CUCUUUAAACCAAAGGCCAU 5’

query=JS16_t3196225, target=FGSG_05451, score=4, range=2326-2345, strand=1

target  5’ CAGAAACUUGGUUUGCGGUA 3’
            ::::: ::::::: :::::
query   3’ CUCUUUAAACCAAAGGCCAU 5’

query=JS16_t3196225, target=FGSG_00074, score=4, range=655-673, strand=1

target  5’ GAGGACUUU-GUUUCCGGUG 3’
           :::.: ::: :::::::::.
query   3’ CUCUUUAAACCAAAGGCCAU 5’

query=JS16_t3196225, target=FGSG_01095, score=4, range=504-523, strand=-1

target  5’ GCGAGUUUUGGUUUUCGGUG 3’
           : ::. ::::::::.::::.
query   3’ CUCUUUAAACCAAAGGCCAU 5’

No results for JS16_t0367247
query=JS16_t2300254, target=FGSG_07175, score=3, range=545-563, strand=-1

target  5’ UCUCGCAUGGCGAAAGCAA 3’
           ::::::::: ::::::::
query   3’ AGAGCGUACAGCUUUCGUA 5’

query=JS16_t2300254, target=FGSG_10801, score=3, range=659-677, strand=-1

target  5’ UCUGGGAUGUCGGAAGCAU 3’
           ::: : ::::::.::::::
query   3’ AGAGCGUACAGCUUUCGUA 5’

query=JS16_t2300254, target=FGSG_10158, score=3.5, range=760-777, strand=-1

target  5’ UAUUG-AUGUUGAAAGCAU 3’
           : :.: ::::.::::::::
query   3’ AGAGCGUACAGCUUUCGUA 5’

query=JS16_t2300254, target=FGSG_01797, score=3.5, range=434-451, strand=-1

target  5’ CGUUG-AUGUCGAAAGCAU 3’
             :.: :::::::::::::
query   3’ AGAGCGUACAGCUUUCGUA 5’
```

```
query   3’ UGUCUAAGCUGGUCGGUUU 5’

query=JS16_t4029242, target=FGSG_10795, score=4, range=945-964, strand=-1

target  5’ ACAUAUUUCGACCAGCUAGA 3’
           ::: : ::::::::::.:.:
query   3’ UGUCU-AAGCUGGUCGGUUU 5’

query=JS16_t4029242, target=FGSG_01108, score=4, range=1772-1790, strand=-1

target  5’ AUGGCCUCGACCAGCCAGA 3’
           :..:  :::::::::::.:
query   3’ UGUCUAAGCUGGUCGGUUU 5’

query=JS16_t4029242, target=FGSG_09843, score=4, range=1449-1468, strand=-1

target  5’ UCAGUAUUCGACCAGCCAUA 3’
            ::: ::::::::::::: :
query   3’ UGUC-UAAGCUGGUCGGUUU 5’

query=JS16_t4029242, target=FGSG_13720, score=4, range=106-124, strand=-1

target  5’ ACAGCGUCGACCAGUCAAU 3’
           ::::  ::::::::.:::
query   3’ UGUCUAAGCUGGUCGGUUU 5’

query=JS16_t4029242, target=FGSG_13166, score=4, range=627-644, strand=-1

target  5’ GCA-CUUCGACCAGCCAGG 3’
           .::  ::::::::::::..
query   3’ UGUCUAAGCUGGUCGGUUU 5’

query=JS16_t4029242, target=FGSG_00324, score=4, range=1161-1179, strand=-1

target  5’ ACUCAGUCGACCAGCCGAA 3’
           ::  : ::::::::::.::
query   3’ UGUCUAAGCUGGUCGGUUU 5’

query=JS16_t4029242, target=FGSG_10889, score=4, range=2547-2566, strand=-1

target  5’ AGAGACGUCGACCAGCCGAA 3’
           : :::  ::::::::::.::
query   3’ UGUCU-AAGCUGGUCGGUUU 5’

No results for JS16_t2236679
query=JS16_t4029310, target=FGSG_04097, score=1.5, range=515-534, strand=-1

target  5’ GUCCAAGAAAGCUUGCAUGA 3’
           :.:::::::::::::::.::
query   3’ CGGGUUCUUUCGAACGUGCU 5’

query=JS16_t4029310, target=FGSG_10536, score=3.5, range=930-948, strand=1

target  5’ GCCC-GGAAAGCUUGCACAA 3’
           :::: .:::::::::::: :
query   3’ CGGGUUCUUUCGAACGUGCU 5’

query=JS16_t4029310, target=FGSG_10799, score=4, range=2289-2308, strand=1

target  5’ GGCCAAGGAAGCUAGCACGA 3’
           : :::::.::::: ::::::
query   3’ CGGGUUCUUUCGAACGUGCU 5’

query=JS16_t4029310, target=FGSG_07356, score=4, range=659-678, strand=1

target  5’ UCACAAGAAUGCUUGCACGA 3’
            : :::::: ::::::::::
query   3’ CGGGUUCUUUCGAACGUGCU 5’

query=JS16_t4029310, target=FGSG_00675, score=4, range=1172-1190, strand=1

target  5’ GCCCAAUGAAGCUUGCA-GA 3’
           :::::: .::::::::: ::
query   3’ CGGGUUCUUUCGAACGUGCU 5’

query=JS16_t4029310, target=FGSG_01724, score=4, range=546-565, strand=-1

target  5’ GCCACAGAAAGCUUGCUCGA 3’
           :::  ::::::::::: :::
query   3’ CGGGUUCUUUCGAACGUGCU 5’

No results for JS16_t3132838
No results for JS16_t4029370
query=JS16_t0254626, target=FGSG_11322, score=4, range=548-569, strand=1

target  5’ GUAGUAACACACUUCCUCUAAU 3’
           ::: :: :::::::::: ::::
query   3’ CAU-AUGGUGUGAAGGACAUUA 5’

query=JS16_t0254626, target=FGSG_04722, score=4, range=1080-1099, strand=-1

target  5’ GUCUGCU-CGCUUCCUGUAAU 3’

query=JS16_t2300254, target=FGSG_10342, score=4, range=1070-1088, strand=1

target  5’ CGUCGAGUGUCGAAAGCAU 3’
              ::: .::::::::::::
query   3’ AGAGCGUACAGCUUUCGUA 5’

query=JS16_t2300254, target=FGSG_02645, score=4, range=2286-2303, strand=1

target  5’ GACCG-AUGUCGAAAGCAU 3’
               :: :::::::::::::
query   3’ AGAGCGUACAGCUUUCGUA 5’

query=JS16_t2300254, target=FGSG_07553, score=4, range=2230-2248, strand=-1

target  5’ UCUUGUGUGUGGAAAGCAU 3’
           :::.:..::: ::::::::
query   3’ AGAGCGUACAGCUUUCGUA 5’

query=JS16_t2300254, target=FGSG_02997, score=4, range=125-142, strand=-1

target  5’ UUUUG-AUGUCGGAAGCAA 3’
           :.:.: ::::::.:::::
query   3’ AGAGCGUACAGCUUUCGUA 5’

query=JS16_t2300254, target=FGSG_01280, score=4, range=1112-1129, strand=-1

target  5’ UGUCG-GUGUCGAAAGCAA 3’
           : ::: .:::::::::::
query   3’ AGAGCGUACAGCUUUCGUA 5’

query=JS16_t2300254, target=FGSG_03608, score=4, range=1189-1207, strand=-1

target  5’ UGCCACAUGUCGAAAGCAA 3’
           :  : :::::::::::::
query   3’ AGAGCGUACAGCUUUCGUA 5’

No results for JS16_t1404120
No results for JS16_t4093191
query=JS16_t3196398, target=FGSG_05447, score=2, range=175-192, strand=1

target  5’ UUUGAUACCGCCGGUCAA 3’
           : :::::::.::::::::
query   3’ ACACUAUGGUGGCCAGUU 5’

query=JS16_t3196398, target=FGSG_01099, score=2.5, range=184-201, strand=1

target  5’ UGGGAUACCGCCGGUCAG 3’
           :: ::::::.:::::::.
query   3’ ACACUAUGGUGGCCAGUU 5’

query=JS16_t3196398, target=FGSG_03711, score=2.5, range=1063-1080, strand=-1

target  5’ UGUGAUACCACCGAUCAG 3’
           ::::::::::::: :::.
query   3’ ACACUAUGGUGGCCAGUU 5’

query=JS16_t3196398, target=FGSG_05141, score=3, range=184-201, strand=1

target  5’ UGGGAUACUGCCGGUCAA 3’
           :: :::::..::::::::
query   3’ ACACUAUGGUGGCCAGUU 5’

query=JS16_t3196398, target=FGSG_07820, score=3.5, range=802-819, strand=-1

target  5’ CGUGGUAUCACCGGUCGA 3’
            :::.::.::::::::.:
query   3’ ACACUAUGGUGGCCAGUU 5’

query=JS16_t3196398, target=FGSG_10175, score=3.5, range=743-760, strand=-1

target  5’ UGUCAGACCACCGGUCAG 3’
           ::: : :::::::::::.
query   3’ ACACUAUGGUGGCCAGUU 5’

query=JS16_t3196398, target=FGSG_10241, score=4, range=196-213, strand=1

target  5’ GGUGCUGCCACUGGUCAA 3’
            ::: :.::::.::::::
query   3’ ACACUAUGGUGGCCAGUU 5’

query=JS16_t3196398, target=FGSG_10677, score=4, range=783-801, strand=1

target  5’ AGUCGAUAUCACCGGUCGA 3’
            :: ::::.::::::::.:
query   3’ ACA-CUAUGGUGGCCAGUU 5’

query=JS16_t3196398, target=FGSG_10873, score=4, range=184-201, strand=1

target  5’ UGGGAUACCGCCGGCCAA 3’
           :: ::::::.:::: :::
query   3’ ACACUAUGGUGGCCAGUU 5’
```

```
           :: :.:. :.:::::::::::
query   3’ CAUAUGGUGUGAAGGACAUUA 5’

No results for JS16_t4029472
query=JS16_t4029477, target=FGSG_03353, score=4, range=796-815, strand=-1

target  5’ UUGAGAUCUCGCGCAAUUUU 3’
           ::.:.:::.::::: :::::
query   3’ AAUUUUAGGGCGCGAUAAAA 5’

No results for JS16_t1340317
query=JS16_t2236994, target=FGSG_13828, score=4, range=904-926, strand=1

target  5’ GAACUGCCAGGCGUUUGUAACGG 3’
           : ::::: : ::::.::::::::
query   3’ CGUGACGAUACGCAGACAUUGCC 5’

query=JS16_t0254628, target=FGSG_01803, score=2.5, range=968-987, strand=-1

target  5’ AAGGCG-AUGGAAUUGUCUCG 3’
           ::.: : ::::::::::::::
query   3’ UUUCACAUACCUUAACAGAGC 5’

query=JS16_t0254628, target=FGSG_05783, score=4, range=1092-1111, strand=-1

target  5’ GAGGUGU-UGGAAUUGUCUGG 3’
           .:.:::: ::::::::::: :
query   3’ UUUCACAUACCUUAACAGAGC 5’

No results for JS16_t4029648
query=JS16_t0157432, target=FGSG_06563, score=3, range=826-844, strand=1

target  5’ GUUUUUGAUCGUGCCAUGA 3’
           .:.: :::::::::::::
query   3’ UAGACACUAGCACGGUACA 5’

query=JS16_t0157432, target=FGSG_06856, score=3, range=992-1009, strand=1

target  5’ AUC-GUGAUGGUGCCAUGU 3’
           ::: ::::: :::::::::
query   3’ UAGACACUAGCACGGUACA 5’

query=JS16_t0157432, target=FGSG_03223, score=3, range=1483-1500, strand=-1

target  5’ AGCU-UGGUCGUGCCAUGU 3’
           : :: ::.:::::::::::
query   3’ UAGACACUAGCACGGUACA 5’

query=JS16_t0157432, target=FGSG_01992, score=3, range=913-930, strand=-1

target  5’ AUCCG-GAUCGUGCCAUGA 3’
           ::: : ::::::::::::
query   3’ UAGACACUAGCACGGUACA 5’

query=JS16_t0157432, target=FGSG_08395, score=3.5, range=34-51, strand=-1

target  5’ GUC-GAGAUCGUGCCAUGA 3’
           .:: : ::::::::::::
query   3’ UAGACACUAGCACGGUACA 5’

query=JS16_t0157432, target=FGSG_04086, score=4, range=241-259, strand=1

target  5’ UUCUGCGAGCGUGCCAUGU 3’
            :::: :: ::::::::::
query   3’ UAGACACUAGCACGGUACA 5’

query=JS16_t0157432, target=FGSG_01853, score=4, range=675-694, strand=1

target  5’ AUCGGUGAUCGAUGCCAUGG 3’
           ::: ::::::: :::::::
query   3’ UAGACACUAGC-ACGGUACA 5’

query=JS16_t0157432, target=FGSG_09199, score=4, range=549-567, strand=-1

target  5’ AUCUGUGAAUGUGUCAUGU 3’
           :::::::: .:::.:::::
query   3’ UAGACACUAGCACGGUACA 5’

query=JS16_t0157432, target=FGSG_12230, score=4, range=3403-3420, strand=-1

target  5’ AUCUGUGAUCGUUCCA-GU 3’
           :::::::::::: ::: ::
query   3’ UAGACACUAGCACGGUACA 5’

query=JS16_t0157432, target=FGSG_11780, score=4, range=50-67, strand=-1

target  5’ AUC-GUGAUCGUGUCUUGU 3’
           ::: :::::::::.: :::
query   3’ UAGACACUAGCACGGUACA 5’

query=JS16_t0686216, target=FGSG_00162, score=3, range=209-226, strand=1

query=JS16_t3196398, target=FGSG_00304, score=4, range=328-345, strand=1

target  5’ UGGGAUACCGCCGGCCAA 3’
           :: ::::::.:::: :::
query   3’ ACACUAUGGUGGCCAGUU 5’

query=JS16_t3196398, target=FGSG_02479, score=4, range=202-219, strand=1

target  5’ UGGGAUACCGCCGGACAA 3’
           :: ::::::.:::: :::
query   3’ ACACUAUGGUGGCCAGUU 5’

query=JS16_t3196398, target=FGSG_11660, score=4, range=931-948, strand=1

target  5’ UCUGGCACCACCGGUCAG 3’
           : ::. :::::::::::.
query   3’ ACACUAUGGUGGCCAGUU 5’

query=JS16_t3196398, target=FGSG_01630, score=4, range=26-42, strand=1

target  5’ AGUGG-ACCACCGGUCAG 3’
            :::. :::::::::::.
query   3’ ACACUAUGGUGGCCAGUU 5’

query=JS16_t3196398, target=FGSG_01605, score=4, range=961-978, strand=-1

target  5’ GUUGAUACCACGGGUCAA 3’
             ::::::::: ::::::
query   3’ ACACUAUGGUGGCCAGUU 5’

query=JS16_t3196398, target=FGSG_00011, score=4, range=1898-1915, strand=-1

target  5’ CGUCAUACCACCGGUCUA 3’
            :: :::::::::::: :
query   3’ ACACUAUGGUGGCCAGUU 5’

query=JS16_t3196398, target=FGSG_04825, score=4, range=77-94, strand=-1

target  5’ AGAGAUACCACAGGUCAA 3’
            : :::::::: ::::::
query   3’ ACACUAUGGUGGCCAGUU 5’

query=JS16_t3196398, target=FGSG_00567, score=4, range=916-932, strand=-1

target  5’ UGUG-UAACACUGGUCAA 3’
           :::: :: :::.::::::
query   3’ ACACUAUGGUGGCCAGUU 5’

query=JS16_t3196398, target=FGSG_07379, score=4, range=1774-1792, strand=-1

target  5’ UGCUGUUACCACCGGCCAA 3’
           :: :: ::::::::: :::
query   3’ AC-ACUAUGGUGGCCAGUU 5’

query=JS16_t3196398, target=FGSG_07574, score=4, range=839-855, strand=-1

target  5’ UUUGUUACCACC-GUCAA 3’
           : :: ::::::: :::::
query   3’ ACACUAUGGUGGCCAGUU 5’

query=JS16_t2300340, target=FGSG_01270, score=2.5, range=75-93, strand=-1

target  5’ AUCAUGAGCUUGAGCCUUU 3’
           ::::::::::::::: ::.
query   3’ UAGUACUCGAACUCGCAAG 5’

query=JS16_t2300340, target=FGSG_11327, score=2.5, range=308-326, strand=-1

target  5’ UUCAUUAGCUUGAGCGUUU 3’
            :::: ::::::::::::.
query   3’ UAGUACUCGAACUCGCAAG 5’

query=JS16_t2300340, target=FGSG_02288, score=3, range=33-51, strand=-1

target  5’ CACACGAGCUUGAGCGUUC 3’
             :: ::::::::::::::
query   3’ UAGUACUCGAACUCGCAAG 5’

query=JS16_t2300340, target=FGSG_12570, score=3, range=235-252, strand=-1

target  5’ AUCUUGAGCUUGAGC-UUC 3’
           ::: ::::::::::: :::
query   3’ UAGUACUCGAACUCGCAAG 5’

query=JS16_t2300340, target=FGSG_12705, score=3, range=344-361, strand=-1

target  5’ GUU-UUAGCUUGAGCGUUC 3’
           .:.  : :::::::::::::
query   3’ UAGUACUCGAACUCGCAAG 5’

query=JS16_t2300340, target=FGSG_05184, score=3.5, range=106-124, strand=1
```

```
target  5’ ACUUAGUCUGGGAGGGAG 3’
           : .::::::.:::::::.
query   3’ UAGAUCAGAUCCUCCCUU 5’

query=JS16_t0686216, target=FGSG_04561, score=3, range=3699-3717, strand=1

target  5’ AUCUAGUGUUAGGAGGGAA 3’
           ::::::: .::::::::::
query   3’ UAGAUCA-GAUCCUCCCUU 5’

query=JS16_t0686216, target=FGSG_06631, score=3.5, range=4956-4973, strand=1

target  5’ GUCUCGUCUAGGAGAGAA 3’
           .::: ::::::::: :::
query   3’ UAGAUCAGAUCCUCCCUU 5’

query=JS16_t0686216, target=FGSG_07868, score=3.5, range=363-381, strand=-1

target  5’ AUCUUGGUCUGGGAGGGGA 3’
           ::: :.::::.::::::.:
query   3’ UAG-AUCAGAUCCUCCCUU 5’

query=JS16_t0686216, target=FGSG_10820, score=3.5, range=216-233, strand=-1

target  5’ UUCUGGGCUAGGAGGGAA 3’
            :::.: :::::::::::
query   3’ UAGAUCAGAUCCUCCCUU 5’

query=JS16_t0686216, target=FGSG_13556, score=3.5, range=1729-1746, strand=-1

target  5’ UUCAAGUUUAGGAGGGAG 3’
            :: :::.:::::::::.
query   3’ UAGAUCAGAUCCUCCCUU 5’

query=JS16_t0686216, target=FGSG_01316, score=3.5, range=1777-1793, strand=-1

target  5’ AUUUGG-CUAGGAGGGAG 3’
           ::.:.: ::::::::::.
query   3’ UAGAUCAGAUCCUCCCUU 5’

query=JS16_t0686216, target=FGSG_07689, score=4, range=1232-1249, strand=1

target  5’ AUCUAGACUAUGAGGGAA 3’
           :::::: ::: :::::::
query   3’ UAGAUCAGAUCCUCCCUU 5’

query=JS16_t0686216, target=FGSG_05252, score=4, range=3250-3267, strand=1

target  5’ UUCCAGUCUAGGAUGGAA 3’
            :: ::::::::: ::::
query   3’ UAGAUCAGAUCCUCCCUU 5’

query=JS16_t0686216, target=FGSG_01285, score=4, range=1155-1172, strand=1

target  5’ AUCUCGUCUAGGAUGGAU 3’
           :::: :::::::: :::
query   3’ UAGAUCAGAUCCUCCCUU 5’

query=JS16_t0686216, target=FGSG_00212, score=4, range=406-423, strand=1

target  5’ AACGAGACUAGGAGGGAA 3’
           : : :: :::::::::::
query   3’ UAGAUCAGAUCCUCCCUU 5’

query=JS16_t0686216, target=FGSG_06345, score=4, range=458-474, strand=1

target  5’ AGCU-GUCAAGGAGGGAA 3’
           : :: ::: :::::::::
query   3’ UAGAUCAGAUCCUCCCUU 5’

query=JS16_t0686216, target=FGSG_11115, score=4, range=168-185, strand=-1

target  5’ AUUGAGUUUGGGAGGGAG 3’
           ::. :::.:.:::::::.
query   3’ UAGAUCAGAUCCUCCCUU 5’

query=JS16_t0686216, target=FGSG_01428, score=4, range=1499-1517, strand=-1

target  5’ AUGCUAGUCUGGCAGGGAA 3’
           :: :::::::.: ::::::
query   3’ UA-GAUCAGAUCCUCCCUU 5’

query=JS16_t0686216, target=FGSG_05930, score=4, range=316-333, strand=-1

target  5’ CUUGGGUCUAGGAGGGAU 3’
            :.  .::::::::::::
query   3’ UAGAUCAGAUCCUCCCUU 5’

query=JS16_t0686216, target=FGSG_09203, score=4, range=2463-2480, strand=-1

target  5’ AUCGAGUCUCGGAGGGAC 3’
           ::: ::::: :::::::

target  5’ AUCGUGAGUUUGGGCGUUG 3’
           :::.::::.:::.:::::
query   3’ UAGUACUCGAACUCGCAAG 5’

query=JS16_t2300340, target=FGSG_04475, score=3.5, range=214-231, strand=1

target  5’ AUCGUGGGCUUGAGC-UUC 3’
           :::.::.:::::::: :::
query   3’ UAGUACUCGAACUCGCAAG 5’

query=JS16_t2300340, target=FGSG_10753, score=3.5, range=1130-1148, strand=1

target  5’ AGAAUCAGCUUGAGCGUUU 3’
           :  :: ::::::::::::.
query   3’ UAGUACUCGAACUCGCAAG 5’

query=JS16_t2300340, target=FGSG_02239, score=3.5, range=165-183, strand=-1

target  5’ AUCGAGAGCUUGGGCGUUG 3’
           :::.  :::::::.:::::
query   3’ UAGUACUCGAACUCGCAAG 5’

query=JS16_t2300340, target=FGSG_06809, score=3.5, range=471-489, strand=-1

target  5’ AGCGUGGGCUUGAGCGUUG 3’
           : :.::.:::::::::::
query   3’ UAGUACUCGAACUCGCAAG 5’

query=JS16_t2300340, target=FGSG_12853, score=3.5, range=58-75, strand=-1

target  5’ AUU-UGAGCUUGAGCUUUC 3’
           ::.  ::::::::::: :::
query   3’ UAGUACUCGAACUCGCAAG 5’

query=JS16_t2300340, target=FGSG_09321, score=4, range=854-872, strand=1

target  5’ AGCGUGAGCCUGAGCGUUU 3’
           : :.::::: ::::::::.
query   3’ UAGUACUCGAACUCGCAAG 5’

query=JS16_t2300340, target=FGSG_07642, score=4, range=1310-1327, strand=1

target  5’ GUCGUGAGCUUGA-CGUUG 3’
           .::.::::::::: ::::
query   3’ UAGUACUCGAACUCGCAAG 5’

query=JS16_t2300340, target=FGSG_04305, score=4, range=136-155, strand=1

target  5’ AUCAAUGAGCUUGAGCGAUG 3’
           ::: ::::::::::::: :
query   3’ UAG-UACUCGAACUCGCAAG 5’

query=JS16_t2300340, target=FGSG_08042, score=4, range=1721-1740, strand=1

target  5’ AUCCAGGAGCUUGGGCGUUA 3’
           :: :: :::::::.:::::
query   3’ UA-GUACUCGAACUCGCAAG 5’

query=JS16_t2300340, target=FGSG_08735, score=4, range=794-812, strand=1

target  5’ AGCCUGAGCUCGAGCGUUC 3’
           : : :::::: ::::::::
query   3’ UAGUACUCGAACUCGCAAG 5’

query=JS16_t2300340, target=FGSG_10292, score=4, range=1652-1670, strand=1

target  5’ ACGAGGAGCUUGAGCGUUA 3’
           :  : :::::::::::::
query   3’ UAGUACUCGAACUCGCAAG 5’

query=JS16_t2300340, target=FGSG_05208, score=4, range=2675-2692, strand=1

target  5’ AGGAUGAGCUUGAGC-UUC 3’
           :  :::::::::::: :::
query   3’ UAGUACUCGAACUCGCAAG 5’

query=JS16_t2300340, target=FGSG_13294, score=4, range=239-256, strand=1

target  5’ UUAAUGAGCUUGA-CGUUC 3’
             : :::::::::: :::::
query   3’ UAGUACUCGAACUCGCAAG 5’

query=JS16_t2300340, target=FGSG_05855, score=4, range=2526-2544, strand=-1

target  5’ AUCAGGAGUUUGAGCCUUC 3’
           :::: :::.:::::: :::
query   3’ UAGUACUCGAACUCGCAAG 5’

query=JS16_t2300340, target=FGSG_03327, score=4, range=624-642, strand=-1

target  5’ AAUGAGGGCUUGAGCGUUC 3’
```

```
query   3' UAGAUCAGAUCCUCCCUU 5'

query=JS16_t0686216, target=FGSG_04666, score=4, range=277-293, strand=-1

target  5' GUUU-GUCUAGGAGGGGC 3'
           .:.: :::::::::::.
query   3' UAGAUCAGAUCCUCCCUU 5'

No results for JS16_t3133212
No results for JS16_t3133213
No results for JS16_t1340512
query=JS16_t2237101, target=FGSG_06089, score=3.5, range=4573-4591, strand=-1

target  5' AG-AUAUUGAGAAUAGCGGU 3'
           :: :.:::: ::::::::::
query   3' UCAUGUAACACUUAUCGCCA 5'

query=JS16_t3133253, target=FGSG_05891, score=2.5, range=1832-1850, strand=-1

target  5' AUAACCAUGGAAUGUGUAU 3'
           : ::::::::::.:::::.
query   3' UUUUGGUACCUUGCACAUG 5'

query=JS16_t3133253, target=FGSG_02138, score=3.5, range=637-654, strand=1

target  5' GAGGCC-UGGAACGUGUAC 3'
           .:..:: ::::::::::::
query   3' UUUUGGUACCUUGCACAUG 5'

query=JS16_t3133253, target=FGSG_12474, score=3.5, range=4-21, strand=-1

target  5' AAGACAAUGGAACGUG-AC 3'
           ::.:: :::::::::: ::
query   3' UUUUGGUACCUUGCACAUG 5'

query=JS16_t3133253, target=FGSG_13254, score=4, range=1678-1697, strand=1

target  5' AAGGCCAUGGAAUGUGCUAC 3'
           ::..::::::::.::: :::
query   3' UUUUGGUACCUUGCAC-AUG 5'

query=JS16_t3133253, target=FGSG_06103, score=4, range=22-40, strand=1

target  5' AGCACCAUGGAGCGUGUGU 3'
           :. ::::::::.:::::..
query   3' UUUUGGUACCUUGCACAUG 5'

query=JS16_t3133253, target=FGSG_03825, score=4, range=1405-1422, strand=1

target  5' GAAACUAUGGAACGU-UGC 3'
           .::::.::::::::: :.:
query   3' UUUUGGUACCUUGCACAUG 5'

query=JS16_t3133253, target=FGSG_11208, score=4, range=1994-2013, strand=1

target  5' GAAGCAAUUGGAACGUGUAC 3'
           .::.: : ::::::::::::
query   3' UUUUGGU-ACCUUGCACAUG 5'

query=JS16_t3133253, target=FGSG_08513, score=4, range=805-822, strand=1

target  5' ACAACCGUGGAACGUG-AC 3'
           : ::::.::::::::: ::
query   3' UUUUGGUACCUUGCACAUG 5'

query=JS16_t3133253, target=FGSG_04113, score=4, range=237-254, strand=-1

target  5' GAAGCCAUGGAACGU-UGC 3'
           .::.::::::::::: :.:
query   3' UUUUGGUACCUUGCACAUG 5'

query=JS16_t3133253, target=FGSG_09665, score=4, range=1149-1167, strand=-1

target  5' AAAACCAAGGAAAGUGUAC 3'
           ::::::: :::: ::::::
query   3' UUUUGGUACCUUGCACAUG 5'

query=JS16_t3133253, target=FGSG_10935, score=4, range=1764-1782, strand=-1

target  5' AAAAACAUAGAACGUGUAA 3'
           :::: ::: :::::::::
query   3' UUUUGGUACCUUGCACAUG 5'

query=JS16_t3133253, target=FGSG_13613, score=4, range=841-858, strand=-1

target  5' AAAUUCAUGGAACG-GUAU 3'
           ::: .::::::::: :::.
query   3' UUUUGGUACCUUGCACAUG 5'

query=JS16_t0365122, target=FGSG_10046, score=4, range=2980-3000, strand=-1

target  5' AGAUGAGUGAAUAGAAGGCAU 3'
           : .. :.:::::::::::::
query   3' UAGUACUCGAACUCGCAAG 5'

query=JS16_t2300340, target=FGSG_03771, score=4, range=33-51, strand=-1

target  5' GGCUUGGGCUUGAGCGUUU 3'
           . : ::.:::::::::::.
query   3' UAGUACUCGAACUCGCAAG 5'

query=JS16_t2300340, target=FGSG_01541, score=4, range=747-764, strand=-1

target  5' AUCAUGACCUUGAG-GUUC 3'
           ::::::: :::::: ::::
query   3' UAGUACUCGAACUCGCAAG 5'

query=JS16_t2300340, target=FGSG_08840, score=4, range=1647-1664, strand=-1

target  5' UUCAUGAGCUUGAG-GUUA 3'
            ::::::::::::: :::
query   3' UAGUACUCGAACUCGCAAG 5'

query=JS16_t2300340, target=FGSG_05372, score=4, range=13549-13568, strand=-1

target  5' AUACAGCAGUUUGAGCGUUC 3'
           :: ::  ::.::::::::::
query   3' UA-GUACUCGAACUCGCAAG 5'

query=JS16_t2300340, target=FGSG_13709, score=4, range=234-251, strand=-1

target  5' UUCAGGAGCUUGAGC-UUC 3'
            ::: :::::::::: :::
query   3' UAGUACUCGAACUCGCAAG 5'

query=JS16_t2300340, target=FGSG_07457, score=4, range=60-77, strand=-1

target  5' UUCAAGAGCUUGAGC-UUC 3'
            ::: :::::::::: :::
query   3' UAGUACUCGAACUCGCAAG 5'

query=JS16_t2300340, target=FGSG_08577, score=4, range=838-855, strand=-1

target  5' AGCA-GAGCUAGAGCGUUC 3'
           : :: ::::: ::::::::
query   3' UAGUACUCGAACUCGCAAG 5'

No results for JS16_t2300365
No results for JS16_t1404242
query=JS16_t2300451, target=FGSG_03230, score=4, range=877-900, strand=1

target  5' UGUGGCAAUACUGAGCGUCUAGCU 3'
           ::.:.  :: ::::::::::::::
query   3' ACGCUCCUACGACUCGCAGAUCGA 5'

query=JS16_t3196531, target=FGSG_10546, score=3, range=779-798, strand=-1

target  5' GUGAUUCUCUUGGUGGUUGG 3'
           .:: :::::::::.:::::.
query   3' UACAAAGAGAACCGCCAACU 5'

query=JS16_t3196531, target=FGSG_11933, score=4, range=323-342, strand=1

target  5' UUGUUUUUCUUGGCGGUUAG 3'
            :::::.:::::::::: .
query   3' UACAAAGAGAACCGCCAACU 5'

query=JS16_t3196531, target=FGSG_02528, score=4, range=1261-1280, strand=1

target  5' GUGUUCCUCUUGGCGUUUGG 3'
           .:::: ::::::::: :::.
query   3' UACAAAGAGAACCGCCAACU 5'

query=JS16_t3196531, target=FGSG_08398, score=4, range=1128-1148, strand=-1

target  5' GUCUUUUUCAUUGGCGGUUGA 3'
           .: :::.:: :::::::::::
query   3' UACAAAGAG-AACCGCCAACU 5'

query=JS16_t3196531, target=FGSG_13427, score=4, range=337-356, strand=-1

target  5' AUGACUGUCUUGGUGGUUGA 3'
           :::  : ::::::.::::::
query   3' UACAAAGAGAACCGCCAACU 5'

query=JS16_t3196531, target=FGSG_06499, score=4, range=95-113, strand=-1

target  5' AUGGGU-UCUUGGUGGUUGA 3'
           :::  : ::::::.::::::
query   3' UACAAAGAGAACCGCCAACU 5'

No results for JS16_t4093356
No results for JS16_t3196560
No results for JS16_t1404325
```

```
           ::: ::: ::::::: :::::
query   3’ UCUUCUCUCUUAUCUCCCGUA 5’

query=JS16_t1340583, target=FGSG_10088, score=4, range=814-834, strand=-1

target  5’ UGUUGCUGGGUUAGAAGUUUG 3’
           :::.. ::.::::.:::::::
query   3’ ACAGUUACUCAAUUUUCAAAC 5’

query=JS16_t1340583, target=FGSG_01210, score=4, range=4141-4162, strand=-1

target  5’ UGUAAAGCGAGUUGAAAGUUUG 3’
           ::: ::  :::::.::::::::
query   3’ ACAGUU-ACUCAAUUUUCAAAC 5’

No results for JS16_t3133308
query=JS16_t4029892, target=FGSG_00443, score=3, range=22-40, strand=1

target  5’ GAGCCCUCUGAGUUCUGGC 3’
           : ::: :::::::::.:::
query   3’ CACGGCAGACUCAAGGCCG 5’

query=JS16_t4029892, target=FGSG_08651, score=3.5, range=1461-1479, strand=-1

target  5’ GUCCUGUCUGGGUUCCGGA 3’
           :: :.:::::.:::::::
query   3’ CACGGCAGACUCAAGGCCG 5’

query=JS16_t4029892, target=FGSG_10923, score=4, range=38-56, strand=-1

target  5’ GUGCAGUUUGAGUUCGGGC 3’
           :::: ::.::::::: :::
query   3’ CACGGCAGACUCAAGGCCG 5’

query=JS16_t4029892, target=FGSG_11213, score=4, range=233-252, strand=-1

target  5’ GUGUUCAUCUGAGUUCUGGU 3’
           ::: .: :::::::::.::.
query   3’ CAC-GGCAGACUCAAGGCCG 5’

query=JS16_t4029892, target=FGSG_13662, score=4, range=1955-1973, strand=-1

target  5’ AUGACUUCUGAGUUCCGGA 3’
            :: : ::::::::::::
query   3’ CACGGCAGACUCAAGGCCG 5’

No results for JS16_t2237261
No results for JS16_t0365131
No results for JS16_t0121561
No results for JS16_t1340742
query=JS16_t2237342, target=FGSG_07055, score=2.5, range=1160-1178, strand=-1

target  5’ UACU-CGAGCAAGUUGUUUG 3’
           .::: :.:::::::::::::.
query   3’ GUGAGGUUCGUUCAACAAAU 5’

query=JS16_t2237342, target=FGSG_04260, score=3, range=257-276, strand=-1

target  5’ CUCUUCGGGCAAGUUGUUUA 3’
           : ::.:..::::::::::::
query   3’ GUGAGGUUCGUUCAACAAAU 5’

query=JS16_t2237342, target=FGSG_01862, score=3.5, range=1416-1435, strand=1

target  5’ CAACCCGAGCAAGUUGUUUC 3’
           ::  ::.:::::::::::
query   3’ GUGAGGUUCGUUCAACAAAU 5’

query=JS16_t2237342, target=FGSG_06845, score=4, range=171-190, strand=1

target  5’ CAGUUCAAGCAACUUGUUUG 3’
           :: :.::::::: ::::::.
query   3’ GUGAGGUUCGUUCAACAAAU 5’

query=JS16_t2237342, target=FGSG_07973, score=4, range=1506-1525, strand=1

target  5’ CUCUGCAAGGAAGUUGUUUA 3’
           : :: :::: ::::::::::
query   3’ GUGAGGUUCGUUCAACAAAU 5’

query=JS16_t2237342, target=FGSG_06928, score=4, range=981-1001, strand=-1

target  5’ UGUAUUGAAGCAAGUUGUUUA 3’
           ... :.  ::::::::::::::
query   3’ GUG-AGGUUCGUUCAACAAAU 5’

query=JS16_t2237362, target=FGSG_06405, score=4, range=1524-1544, strand=1

target  5’ UGUUCCUGUAACCGAUGAGGG 3’
            .:: :::.:::::::::::.
query   3’ CUAACGACGUUGGCUACUCCU 5’

query=JS16_t0126362, target=FGSG_00620, score=2, range=816-833, strand=1

target  5’ CUCUGCCCUCUCAGCUAA 3’
            ::: :::::::::::::
query   3’ UAGAAGGGAGAGUCGAUU 5’

query=JS16_t0126362, target=FGSG_13307, score=3.5, range=226-243, strand=1

target  5’ GUCUUCCCUCGCAGCUAC 3’
           .::::::::: ::::::
query   3’ UAGAAGGGAGAGUCGAUU 5’

query=JS16_t0126362, target=FGSG_00261, score=3.5, range=2575-2592, strand=-1

target  5’ AGCUUCCUUCUCAGCUGG 3’
           : :::::.:::::::::..
query   3’ UAGAAGGGAGAGUCGAUU 5’

query=JS16_t0126362, target=FGSG_00470, score=4, range=546-563, strand=1

target  5’ CCCUUUCCUUUCAGCUAA 3’
             :::.:::.::::::::
query   3’ UAGAAGGGAGAGUCGAUU 5’

query=JS16_t0126362, target=FGSG_03512, score=4, range=1228-1246, strand=1

target  5’ AUCUUCCCUCUCAAGUUAC 3’
           :::::::::::: ::.::
query   3’ UAGAAGGGAGAG-UCGAUU 5’

query=JS16_t0126362, target=FGSG_03607, score=4, range=943-960, strand=1

target  5’ AUCCUUCCUCUCAGCAAA 3’
           ::: :.:::::::::: ::
query   3’ UAGAAGGGAGAGUCGAUU 5’

query=JS16_t0126362, target=FGSG_08749, score=4, range=1054-1071, strand=1

target  5’ UUCUUCCCUCUCAUCUAC 3’
            :::::::::::: :::
query   3’ UAGAAGGGAGAGUCGAUU 5’

query=JS16_t0126362, target=FGSG_06970, score=4, range=1148-1165, strand=1

target  5’ AUUUCCCCACUCAGCUAG 3’
           ::.: ::: ::::::::.
query   3’ UAGAAGGGAGAGUCGAUU 5’

query=JS16_t0126362, target=FGSG_02233, score=4, range=373-390, strand=1

target  5’ AUCCUCCCUCUCAGCAAC 3’
           ::: ::::::::::: :
query   3’ UAGAAGGGAGAGUCGAUU 5’

query=JS16_t0126362, target=FGSG_07099, score=4, range=1274-1291, strand=1

target  5’ AUCGUCCAUCUCAGCUAU 3’
           ::: ::: :::::::::
query   3’ UAGAAGGGAGAGUCGAUU 5’

query=JS16_t0126362, target=FGSG_04578, score=4, range=111-128, strand=1

target  5’ CUCUGCCCUCCCAGCUAA 3’
            ::: ::::: :::::::
query   3’ UAGAAGGGAGAGUCGAUU 5’

query=JS16_t0126362, target=FGSG_13358, score=4, range=1067-1084, strand=1

target  5’ AGCAUCCUUCUCAGCUAC 3’
           : : :::.:::::::::
query   3’ UAGAAGGGAGAGUCGAUU 5’

query=JS16_t0126362, target=FGSG_07044, score=4, range=291-308, strand=1

target  5’ AGCUGCCCUCUCAGAUAA 3’
           : :: ::::::::: :::
query   3’ UAGAAGGGAGAGUCGAUU 5’

query=JS16_t0126362, target=FGSG_03767, score=4, range=411-428, strand=1

target  5’ AGCUCCCGUCUCAGCUAA 3’
           : :: :: ::::::::::
query   3’ UAGAAGGGAGAGUCGAUU 5’

query=JS16_t0126362, target=FGSG_13392, score=4, range=88-105, strand=1

target  5’ AACUGCCCUCUCUGCUAA 3’
           : :: ::::::: :::::
query   3’ UAGAAGGGAGAGUCGAUU 5’

query=JS16_t0126362, target=FGSG_02812, score=4, range=67-83, strand=1
```

```
query=JS16_t2237362, target=FGSG_06212, score=4, range=2200-2219, strand=1

target  5’ GAUUAC-GCAUCCGAUGAGGA 3’
           :::: : ::: ::::::::::
query   3’ CUAACGACGUUGGCUACUCCU 5’

No results for JS16_t0365134
query=JS16_t0072616, target=FGSG_06456, score=3, range=968-985, strand=-1

target  5’ CAACACCAUAGACCCUGG 3’
           ::::::::::::: :.::
query   3’ GUUGUGGUAUCUGAGGCC 5’

query=JS16_t0072616, target=FGSG_00573, score=3, range=1692-1709, strand=-1

target  5’ AAACACCUUAGACUCCGG 3’
            :::::: ::::::::::
query   3’ GUUGUGGUAUCUGAGGCC 5’

query=JS16_t0072616, target=FGSG_08548, score=3.5, range=1693-1709, strand=1

target  5’ CGAC-CCAUGGACUCCGU 3’
           :.:: ::::.:::::::
query   3’ GUUGUGGUAUCUGAGGCC 5’

query=JS16_t0072616, target=FGSG_02269, score=3.5, range=133-150, strand=-1

target  5’ CAACGCCGUAGACUCGGG 3’
           ::::.::.::::::: ::
query   3’ GUUGUGGUAUCUGAGGCC 5’

query=JS16_t0072616, target=FGSG_10464, score=3.5, range=3248-3264, strand=-1

target  5’ CAAAGCCAUAGAC-CCGG 3’
           ::: .:::::::: ::::
query   3’ GUUGUGGUAUCUGAGGCC 5’

query=JS16_t0072616, target=FGSG_09968, score=4, range=99-116, strand=1

target  5’ CGACGCUAUAGGCUCCGC 3’
           :.::.:.::::.:::::
query   3’ GUUGUGGUAUCUGAGGCC 5’

query=JS16_t0072616, target=FGSG_11532, score=4, range=231-248, strand=1

target  5’ CAACACUGAAGACUCCGG 3’
           ::::::.. :::::::::
query   3’ GUUGUGGUAUCUGAGGCC 5’

query=JS16_t0072616, target=FGSG_09272, score=4, range=201-218, strand=1

target  5’ CAUUGUCAUAGAUUCCGG 3’
           :: ...::::::.:::::
query   3’ GUUGUGGUAUCUGAGGCC 5’

query=JS16_t0072616, target=FGSG_08084, score=4, range=62-79, strand=1

target  5’ CAAAAUCAGAGACUCCGG 3’
           ::: :.:: :::::::::
query   3’ GUUGUGGUAUCUGAGGCC 5’

query=JS16_t0072616, target=FGSG_09696, score=4, range=660-676, strand=1

target  5’ CAACACCAUAG-CUCUGA 3’
           ::::::::::: :::.:
query   3’ GUUGUGGUAUCUGAGGCC 5’

query=JS16_t0072616, target=FGSG_04908, score=4, range=153-170, strand=1

target  5’ CAACUCCAAAGACUCCGA 3’
           :::: ::: ::::::::
query   3’ GUUGUGGUAUCUGAGGCC 5’

query=JS16_t0072616, target=FGSG_11731, score=4, range=526-543, strand=-1

target  5’ AAAUGUCAUAGACUCUGG 3’
            ::...::::::::::.::
query   3’ GUUGUGGUAUCUGAGGCC 5’

query=JS16_t0072616, target=FGSG_02643, score=4, range=1469-1486, strand=-1

target  5’ CAGCGCCGUAGACUCCAG 3’
           ::.:.::.::::::::: :
query   3’ GUUGUGGUAUCUGAGGCC 5’

query=JS16_t0072616, target=FGSG_00109, score=4, range=800-817, strand=-1

target  5’ CGGCACCAUAAACUUCGG 3’
           :..:::::::: :::.:::
query   3’ GUUGUGGUAUCUGAGGCC 5’

query=JS16_t0072616, target=FGSG_02968, score=4, range=202-219, strand=-1

target  5’ AUC-GCCUUCUCAGCUAU 3’
           :::  ::.:::::::::
query   3’ UAGAAGGGAGAGUCGAUU 5’

query=JS16_t0126362, target=FGSG_09418, score=4, range=779-796, strand=1

target  5’ AUGCGCCCUCUCAGCUAC 3’
           ::   ::::::::::::
query   3’ UAGAAGGGAGAGUCGAUU 5’

query=JS16_t0126362, target=FGSG_02645, score=4, range=1936-1953, strand=-1

target  5’ GGCUUUCUUCUCAGCUAG 3’
           . :::.:.:::::::::.
query   3’ UAGAAGGGAGAGUCGAUU 5’

query=JS16_t0126362, target=FGSG_05967, score=4, range=177-195, strand=-1

target  5’ CUCUUCCCUCUCAGCCUGA 3’
            :::::::::::::: ::.:
query   3’ UAGAAGGGAGAGUC-GAUU 5’

query=JS16_t0126362, target=FGSG_10126, score=4, range=648-665, strand=-1

target  5’ AUAUUCCAUCUCAGCUGA 3’
           :: :::: ::::::::.:
query   3’ UAGAAGGGAGAGUCGAUU 5’

query=JS16_t0126362, target=FGSG_09140, score=4, range=1651-1667, strand=-1

target  5’ AUC-UCUUUCUCAGCUAC 3’
           ::: ::..:::::::::
query   3’ UAGAAGGGAGAGUCGAUU 5’

query=JS16_t0126362, target=FGSG_00957, score=4, range=2216-2234, strand=-1

target  5’ AUCUUCCACUCUAAGCUAA 3’
           ::::::: :::: ::::::
query   3’ UAGAAGG-GAGAGUCGAUU 5’

query=JS16_t0126362, target=FGSG_04555, score=4, range=592-610, strand=-1

target  5’ AUCUUCCCUGCUCAUCUAA 3’
           ::::::::: :::: ::::
query   3’ UAGAAGGGA-GAGUCGAUU 5’

No results for JS16_t2300536
query=JS16_t4093425, target=FGSG_13549, score=4, range=3167-3190, strand=1

target  5’ AGUGGACAGAGACAGCUGUUGUUG 3’
           :.::: ::::.:::::::::.:.::
query   3’ UUACCCGUCUUUGUCGACAGCGAC 5’

query=JS16_t4093425, target=FGSG_08170, score=4, range=272-295, strand=1

target  5’ AAUGGGUGAAAACAACUGUCGCUG 3’
           :::::::.. ::::: :::::::::
query   3’ UUACCCGUCUUUGUCGACAGCGAC 5’

No results for JS16_t4093427
query=JS16_t1404390, target=FGSG_04876, score=3.5, range=890-910, strand=-1

target  5’ AGGCGGCGUUGAGACCGACGA 3’
           ::: .:::::::::: :::::
query   3’ UCCAUCGCAACUCUGUCUGCU 5’

No results for JS16_t1404412
No results for JS16_t3196675
No results for JS16_t3196684
No results for JS16_t3196710
No results for JS16_t4093529
No results for JS16_t3196730
No results for JS16_t3196746
No results for JS16_t1404500
No results for JS16_t1404510
No results for JS16_t3196797
query=JS16_t0157742, target=FGSG_04456, score=3, range=1796-1816, strand=-1

target  5’ GAUCCAUGAGGUGUUUGGCGA 3’
           ::::   ::::::::::::::
query   3’ CUAGCGCCUCCACAAACCGCU 5’

query=JS16_t0157742, target=FGSG_05744, score=3.5, range=843-863, strand=1

target  5’ CUUUGCCGAGGUGUUUGGCGA 3’
               :.:: ::::::::::::::
query   3’ CUAGCGCCUCCACAAACCGCU 5’

query=JS16_t0157742, target=FGSG_00664, score=3.5, range=1432-1451, strand=1

target  5’ GGAAGC-GAGGUGUUUGGCGA 3’
           :.   :: ::::::::::::::
```

```
target  5’ CAAUGCCAUAGACGCUGG 3’
           :::..:::::::: :.::
query   3’ GUUGUGGUAUCUGAGGCC 5’

query=JS16_t0072616, target=FGSG_07216, score=4, range=200-217, strand=-1

target  5’ CAACACCAUAACCUCCGG 3’
           ::::::::::  ::::::
query   3’ GUUGUGGUAUCUGAGGCC 5’

query=JS16_t0072616, target=FGSG_12689, score=4, range=1579-1595, strand=-1

target  5’ CAG-GUCAUAGGCUCCGG 3’
           ::. ..:::::.::::::
query   3’ GUUGUGGUAUCUGAGGCC 5’

query=JS16_t0072616, target=FGSG_06280, score=4, range=745-762, strand=-1

target  5’ AAAAACCAUAGAUUCCGC 3’
            :: ::::::::.::::
query   3’ GUUGUGGUAUCUGAGGCC 5’

query=JS16_t0072616, target=FGSG_06012, score=4, range=1950-1967, strand=-1

target  5’ UAUUUCCAUAGACUUCGG 3’
           .: .  :::::::::.:::
query   3’ GUUGUGGUAUCUGAGGCC 5’

query=JS16_t0072616, target=FGSG_00776, score=4, range=650-667, strand=-1

target  5’ CAUCACCAUAGACACCGC 3’
           :: :::::::::: :::
query   3’ GUUGUGGUAUCUGAGGCC 5’

query=JS16_t0072616, target=FGSG_08281, score=4, range=122-139, strand=-1

target  5’ CAAGUCCAUAGAUUCCGU 3’
           :::  :::::::.::::
query   3’ GUUGUGGUAUCUGAGGCC 5’

query=JS16_t0072616, target=FGSG_06298, score=4, range=371-387, strand=-1

target  5’ CCAUG-CAUAGACUCCGG 3’
           : :.. :::::::::::
query   3’ GUUGUGGUAUCUGAGGCC 5’

query=JS16_t0166412, target=FGSG_06379, score=3, range=1162-1180, strand=-1

target  5’ GAAGU-CGUGUCCAGGUGAC 3’
           ::::: :..::::::::::
query   3’ CUUCAUGUGCAGGUCCACUG 5’

query=JS16_t0166412, target=FGSG_04636, score=3, range=1231-1250, strand=-1

target  5’ GAUGCAUACGUCCAGGUGAU 3’
           :: : :.:::::::::::.
query   3’ CUUCAUGUGCAGGUCCACUG 5’

query=JS16_t0166412, target=FGSG_04468, score=3.5, range=1051-1070, strand=1

target  5’ UACGUGUACGUCCAGGUGAU 3’
            : ::..::::::::::::.
query   3’ CUUCAUGUGCAGGUCCACUG 5’

query=JS16_t0166412, target=FGSG_09526, score=4, range=1299-1318, strand=-1

target  5’ GAGGUAGACGUCGAGGUGAU 3’
           ::.::: ::::: ::::::.
query   3’ CUUCAUGUGCAGGUCCACUG 5’

query=JS16_t0166412, target=FGSG_07086, score=4, range=1421-1440, strand=-1

target  5’ GCGGUAAAUGUCCAGGUGAU 3’
           : .::: :.::::::::::.
query   3’ CUUCAUGUGCAGGUCCACUG 5’

query=JS16_t0166412, target=FGSG_11092, score=4, range=786-804, strand=-1

target  5’ GAUG-ACACGUCCAGUUGAC 3’
           :: : :::::::::: ::::
query   3’ CUUCAUGUGCAGGUCCACUG 5’

query=JS16_t4030058, target=FGSG_04731, score=2.5, range=279-300, strand=-1

target  5’ CCAGGC-AUGGACACCAAGAAGC 3’
           ::::.: ::::::::::::::
query   3’ GGUCUGCUACCUGUGGUUCUUCA 5’

No results for JS16_t1340846
query=JS16_t4030131, target=FGSG_11505, score=2.5, range=3221-3239, strand=1
query   3’ CUAGCGCCUCCACAAACCGCU 5’

query=JS16_t0157742, target=FGSG_07847, score=4, range=890-909, strand=-1

target  5’ GAUU-UGGAGGUGUUUGGAGA 3’
           :::. .:::::::::::: ::
query   3’ CUAGCGCCUCCACAAACCGCU 5’

No results for JS16_t1404554
No results for JS16_t2300844
query=JS16_t4093699, target=FGSG_10868, score=3.5, range=1074-1091, strand=1

target  5’ CGUC-GAGAAUGCUCCGGU 3’
           :::: .:::::: ::::::
query   3’ GCAGUUUCUUACUAGGCCA 5’

query=JS16_t4093699, target=FGSG_00009, score=3.5, range=555-572, strand=1

target  5’ UGUCAAAG-GUGAUCCGGU 3’
           .::::::: .:::::::::
query   3’ GCAGUUUCUUACUAGGCCA 5’

query=JS16_t4093699, target=FGSG_11087, score=3.5, range=744-762, strand=-1

target  5’ CGUUAAAGGAUGAUCUGGC 3’
           :::.::::.::::::.::
query   3’ GCAGUUUCUUACUAGGCCA 5’

query=JS16_t4093699, target=FGSG_06688, score=3.5, range=1088-1105, strand=-1

target  5’ CGUCGUAGAAUGAU-CGGU 3’
           ::::. :::::::: ::::
query   3’ GCAGUUUCUUACUAGGCCA 5’

query=JS16_t4093699, target=FGSG_08229, score=3.5, range=937-954, strand=-1

target  5’ CG-CGAAGAAUGAUCCCGU 3’
           :: :.:::::::::: ::
query   3’ GCAGUUUCUUACUAGGCCA 5’

query=JS16_t4093699, target=FGSG_12282, score=4, range=244-262, strand=1

target  5’ CGUCAAAGAAUGAUCCCAU 3’
           :::::::::::::::  :
query   3’ GCAGUUUCUUACUAGGCCA 5’

query=JS16_t4093699, target=FGSG_01223, score=4, range=795-812, strand=1

target  5’ CGUU-GGGGAUGAUCCGGU 3’
           :::. ..:.::::::::::
query   3’ GCAGUUUCUUACUAGGCCA 5’

query=JS16_t4093699, target=FGSG_09199, score=4, range=488-506, strand=1

target  5’ CGUUCGAGAAUGAUCCUGU 3’
           :::. .:::::::::: ::
query   3’ GCAGUUUCUUACUAGGCCA 5’

query=JS16_t4093699, target=FGSG_11096, score=4, range=338-355, strand=1

target  5’ CUUCAAAGAA-GGUCCGGU 3’
           : :::::::: :.::::::
query   3’ GCAGUUUCUUACUAGGCCA 5’

query=JS16_t4093699, target=FGSG_11800, score=4, range=628-646, strand=-1

target  5’ GGUCAAGGGAUGGUCCGGU 3’
            :::::.:.:::.::::::
query   3’ GCAGUUUCUUACUAGGCCA 5’

query=JS16_t4093699, target=FGSG_09582, score=4, range=1165-1183, strand=-1

target  5’ UGUUAAAGAAUGCUUCGGU 3’
           .::.:::::::: :.::::
query   3’ GCAGUUUCUUACUAGGCCA 5’

query=JS16_t4093699, target=FGSG_06181, score=4, range=1553-1570, strand=-1

target  5’ CGUCAAAG-AUGAUCUGGA 3’
           :::::::: ::::::.::
query   3’ GCAGUUUCUUACUAGGCCA 5’

No results for JS16_t2300962
No results for JS16_t3197009
No results for JS16_t2300990
query=JS16_t4093830, target=FGSG_05308, score=2.5, range=220-237, strand=-1

target  5’ GAUGGUCAGAAGGUCGCU 3’
           .:.:::::::::.:::::.
query   3’ UUGCCAGUCUUUCAGCGG 5’

query=JS16_t4093830, target=FGSG_10858, score=3, range=98-115, strand=1
```

```
target  5' GUUCUUCAACACAAGGUGG 3'
           ::.:::::::::: ::::
query   3' CAGGAAGUUGUGUUACACC 5'

query=JS16_t4030131, target=FGSG_02320, score=3, range=780-798, strand=-1

target  5' GUUUUUCAGCACGAUGUGG 3'
           ::..::::.:::.::::::
query   3' CAGGAAGUUGUGUUACACC 5'

query=JS16_t4030131, target=FGSG_07100, score=3.5, range=2260-2278, strand=-1

target  5' GUUCUUCAACACGAGGUGG 3'
           ::.:::::::::.: ::::
query   3' CAGGAAGUUGUGUUACACC 5'

query=JS16_t4030131, target=FGSG_00633, score=4, range=5154-5173, strand=1

target  5' CUUCUUUAACAACAAUGUGG 3'
            :.:::.::: :::::::::
query   3' CAGGAAGUUG-UGUUACACC 5'

query=JS16_t4030131, target=FGSG_12655, score=4, range=589-608, strand=1

target  5' GACCAUCAACACAAUGAUGG 3'
           : :: ::::::::::: :::
query   3' CAGGAAGUUGUGUUAC-ACC 5'

query=JS16_t4030131, target=FGSG_13531, score=4, range=314-333, strand=1

target  5' CUGCUUCAACAGCAAUGUGG 3'
            : :::::::: ::::::::
query   3' CAGGAAGUUGU-GUUACACC 5'

query=JS16_t4030131, target=FGSG_08220, score=4, range=757-775, strand=-1

target  5' AUGCUUCAAUACAAUGUGU 3'
            : ::::::.::::::::
query   3' CAGGAAGUUGUGUUACACC 5'

No results for JS16_t4030135
query=JS16_t0202186, target=FGSG_00434, score=3, range=1875-1893, strand=1

target  5' AAAUCAGGAUCCCUCUUCC 3'
           ::::: .:::::::::.::
query   3' UUUAGGUCUAGGGAGAGGG 5'

query=JS16_t0202186, target=FGSG_05996, score=3, range=6537-6555, strand=1

target  5' AAAGCCAGAGCCCUCUCCC 3'
           ::: ::::: :::::::::
query   3' UUUAGGUCUAGGGAGAGGG 5'

query=JS16_t0202186, target=FGSG_10247, score=3, range=1325-1343, strand=-1

target  5' AUAUCCAGAUCUUUCUCCC 3'
           : :::::::::..::::::
query   3' UUUAGGUCUAGGGAGAGGG 5'

query=JS16_t0202186, target=FGSG_12314, score=3.5, range=943-962, strand=1

target  5' ACGCCCGAGAUCCCUCUCCC 3'
           : .  :: :::::::::::::
query   3' UUUAGG-UCUAGGGAGAGGG 5'

query=JS16_t0202186, target=FGSG_12058, score=3.5, range=205-223, strand=-1

target  5' AACUCUGGAUCCCUCUUCC 3'
           :: ::..:::::::::.::
query   3' UUUAGGUCUAGGGAGAGGG 5'

query=JS16_t0202186, target=FGSG_03089, score=4, range=210-228, strand=1

target  5' AAAUGCAGAUUCCUUUUCC 3'
           :::: :::::.:::.:.::
query   3' UUUAGGUCUAGGGAGAGGG 5'

query=JS16_t0202186, target=FGSG_12581, score=4, range=27-45, strand=1

target  5' AGGUCCAGAUCCCAUUCCC 3'
           :..:::::::::: .::::
query   3' UUUAGGUCUAGGGAGAGGG 5'

query=JS16_t0202186, target=FGSG_10986, score=4, range=133-152, strand=1

target  5' AAGUCCAAGAUCCUUCUUCU 3'
           ::.::: ::::::.:::.:.
query   3' UUUAGG-UCUAGGGAGAGGG 5'

query=JS16_t0202186, target=FGSG_08161, score=4, range=13-30, strand=1

target  5' AGAU-UAGAUCCCUUUUCC 3'

target  5' AACGGACAGAAGGUCGCC 3'
           ::::: :::::.::::::
query   3' UUGCCAGUCUUUCAGCGG 5'

query=JS16_t4093830, target=FGSG_07449, score=3, range=694-711, strand=1

target  5' GGCAGUCGGAAAGUCGCC 3'
           ..: :::.::::::::::
query   3' UUGCCAGUCUUUCAGCGG 5'

query=JS16_t4093830, target=FGSG_02921, score=3, range=251-267, strand=-1

target  5' AAC-UUCAGAAAGUCGCA 3'
           :::  ::::::::::::
query   3' UUGCCAGUCUUUCAGCGG 5'

query=JS16_t4093830, target=FGSG_13826, score=3.5, range=601-619, strand=1

target  5' AAUGGUCAGAAAGUCUGCG 3'
           ::.:::::::::::: ::
query   3' UUGCCAGUCUUUCAG-CGG 5'

query=JS16_t4093830, target=FGSG_02609, score=3.5, range=1602-1619, strand=1

target  5' AACGCUCAGAAAGUCCCU 3'
           :::: :::::::::: :.
query   3' UUGCCAGUCUUUCAGCGG 5'

query=JS16_t4093830, target=FGSG_03141, score=3.5, range=4-21, strand=-1

target  5' GACGGUCGAAAAGUCGCC 3'
           .::::::. :::::::::
query   3' UUGCCAGUCUUUCAGCGG 5'

query=JS16_t4093830, target=FGSG_01990, score=3.5, range=946-963, strand=-1

target  5' AAUGUUCAGAAAGACGCC 3'
           ::.: :::::::: ::::
query   3' UUGCCAGUCUUUCAGCGG 5'

query=JS16_t4093830, target=FGSG_09356, score=3.5, range=569-585, strand=-1

target  5' AGC-CUCAGAAAGUUGCC 3'
           :.:  :::::::::.:::
query   3' UUGCCAGUCUUUCAGCGG 5'

query=JS16_t4093830, target=FGSG_02509, score=4, range=1408-1425, strand=1

target  5' ACCGGUCAGAAAUUCGCA 3'
           : :::::::::: ::::
query   3' UUGCCAGUCUUUCAGCGG 5'

query=JS16_t4093830, target=FGSG_10111, score=4, range=637-655, strand=1

target  5' CACGUUGCAGAAAGUCGCC 3'
            ::: : ::::::::::::
query   3' UUGCCA-GUCUUUCAGCGG 5'

query=JS16_t4093830, target=FGSG_07196, score=4, range=3157-3174, strand=1

target  5' AAGGCUCAGAAAGACGCC 3'
           :: : :::::::: ::::
query   3' UUGCCAGUCUUUCAGCGG 5'

query=JS16_t4093830, target=FGSG_01355, score=4, range=989-1005, strand=1

target  5' CAC-GUCAGAAAGGCGCC 3'
            :: ::::::::: ::::
query   3' UUGCCAGUCUUUCAGCGG 5'

query=JS16_t4093830, target=FGSG_11162, score=4, range=326-342, strand=1

target  5' AAC-CUCAUAAAGUCGCC 3'
           :::  ::: :::::::::
query   3' UUGCCAGUCUUUCAGCGG 5'

query=JS16_t4093830, target=FGSG_09838, score=4, range=724-741, strand=-1

target  5' AACGGUUAGAGGGUUGCC 3'
           ::::::.:::..::.:::
query   3' UUGCCAGUCUUUCAGCGG 5'

query=JS16_t4093830, target=FGSG_01144, score=4, range=2941-2958, strand=-1

target  5' AACGGUUUGAAAGUCGUC 3'
           ::::::. ::::::::.:
query   3' UUGCCAGUCUUUCAGCGG 5'

query=JS16_t4093830, target=FGSG_04115, score=4, range=379-396, strand=-1

target  5' AGCGGUCAGAAGGUCACU 3'
```

```
          :.:: .::::::::.:.::
query   3' UUUAGGUCUAGGGAGAGGG 5'

query=JS16_t0202186, target=FGSG_02248, score=4, range=1045-1063, strand=1

target  5' GACUCUAUAUCCCUCUCCC 3'
          .: ::.: :::::::::::
query   3' UUUAGGUCUAGGGAGAGGG 5'

query=JS16_t0202186, target=FGSG_01176, score=4, range=291-309, strand=1

target  5' AAGACCAAAUCCCUCUCCU 3'
          ::. ::: ::::::::::.
query   3' UUUAGGUCUAGGGAGAGGG 5'

query=JS16_t0202186, target=FGSG_10170, score=4, range=347-365, strand=1

target  5' GAGCCCAGAUCGCUCUCCC 3'
          .:. ::::::: :::::::
query   3' UUUAGGUCUAGGGAGAGGG 5'

query=JS16_t0202186, target=FGSG_11008, score=4, range=749-767, strand=1

target  5' ACUACCAGAUCCCUCUCUC 3'
          :   :::::::::::::.:
query   3' UUUAGGUCUAGGGAGAGGG 5'

query=JS16_t0202186, target=FGSG_04433, score=4, range=283-300, strand=1

target  5' AAAUACGGAUCCCU-UCCC 3'
          :::: :.::::::: ::::
query   3' UUUAGGUCUAGGGAGAGGG 5'

query=JS16_t0202186, target=FGSG_12595, score=4, range=4825-4842, strand=1

target  5' GAAUACA-AUCCCUCUCCU 3'
          .::: :: ::::::::::.
query   3' UUUAGGUCUAGGGAGAGGG 5'

query=JS16_t0202186, target=FGSG_11670, score=4, range=39-56, strand=1

target  5' GAGUCUAG-UCCCUCUCCU 3'
          .:.::.:: :::::::::.
query   3' UUUAGGUCUAGGGAGAGGG 5'

query=JS16_t0202186, target=FGSG_02540, score=4, range=3454-3472, strand=-1

target  5' GCGUCCAGAUUCCUCUUCC 3'
          . .:::::::.:::::.::
query   3' UUUAGGUCUAGGGAGAGGG 5'

query=JS16_t0202186, target=FGSG_01795, score=4, range=30-48, strand=-1

target  5' CGAGCUGGAUCCCUCUCCC 3'
           .: :..::::::::::::
query   3' UUUAGGUCUAGGGAGAGGG 5'

query=JS16_t0202186, target=FGSG_10188, score=4, range=1796-1814, strand=-1

target  5' AGACCUAGAUCCUUCUCCA 3'
          :.: :.::::::.:::::
query   3' UUUAGGUCUAGGGAGAGGG 5'

query=JS16_t0202186, target=FGSG_00999, score=4, range=362-380, strand=-1

target  5' GAAUCAAGAUCCAUCUCCU 3'
          .:::: :::::: :::::.
query   3' UUUAGGUCUAGGGAGAGGG 5'

query=JS16_t0202186, target=FGSG_00855, score=4, range=702-721, strand=-1

target  5' AUGUCGUAGAUCCCUCUUCC 3'
          : .:: .:::::::::.::
query   3' UUUAG-GUCUAGGGAGAGGG 5'

query=JS16_t0202186, target=FGSG_12097, score=4, range=608-626, strand=-1

target  5' AUAUCAAUAUCCCUCUCCC 3'
          : ::: : :::::::::::
query   3' UUUAGGUCUAGGGAGAGGG 5'

query=JS16_t3133661, target=FGSG_08477, score=3, range=334-353, strand=-1

target  5' UCGUUGUAGACGUAGUAGUU 3'
          .:::.::::::: :::::::
query   3' GGCAGCAUCUGC-UCAUCAA 5'

query=JS16_t3133661, target=FGSG_02730, score=3.5, range=1033-1051, strand=-1

target  5' UCGACGUAGGUGAGUAGUU 3'
          .:: :::::..::::::::
query   3' GGCAGCAUCUGCUCAUCAA 5'
```

```
          :.:::::::::.::: :.
query   3' UUGCCAGUCUUUCAGCGG 5'

query=JS16_t4093830, target=FGSG_10524, score=4, range=898-915, strand=-1

target  5' AACAGUCAGAGGGUUGCC 3'
          ::: ::::::..::.:::
query   3' UUGCCAGUCUUUCAGCGG 5'

query=JS16_t4093830, target=FGSG_00843, score=4, range=1918-1936, strand=-1

target  5' CACGGUUCAGGAAGUCGUC 3'
           :::: ::::.:::::::.:
query   3' UUGCC-AGUCUUUCAGCGG 5'

query=JS16_t4093830, target=FGSG_12375, score=4, range=1129-1147, strand=-1

target  5' AACUGGUCAGAGAGUCGUA 3'
          ::: :::::::.:::::.
query   3' UUG-CCAGUCUUUCAGCGG 5'

query=JS16_t4093830, target=FGSG_03497, score=4, range=2393-2409, strand=-1

target  5' CAC-GUCAGAAAGUUGCG 3'
           :: :::::::::::.::
query   3' UUGCCAGUCUUUCAGCGG 5'

query=JS16_t0337758, target=FGSG_07353, score=2.5, range=904-921, strand=-1

target  5' UCGUGCUCGAUACCAGUU 3'
          :: :.:.:::::::::::
query   3' AGAAUGGGCUAUGGUCAA 5'

query=JS16_t0337758, target=FGSG_07258, score=3, range=1929-1945, strand=-1

target  5' UCUUA-CCGAUACCGGUU 3'
          ::::: ::::::::.:::
query   3' AGAAUGGGCUAUGGUCAA 5'

query=JS16_t0337758, target=FGSG_10076, score=3, range=1426-1442, strand=-1

target  5' UCUU-CCAGAUACCAGUU 3'
          :::: :: ::::::::::
query   3' AGAAUGGGCUAUGGUCAA 5'

query=JS16_t0337758, target=FGSG_01610, score=3, range=907-923, strand=-1

target  5' UCUU-CCCGACACCAGUU 3'
          :::: ::::: :::::::
query   3' AGAAUGGGCUAUGGUCAA 5'

query=JS16_t0337758, target=FGSG_10266, score=3.5, range=1154-1171, strand=1

target  5' UUUCACCCGAUGCCAGUC 3'
          :.: :::::::.:::::
query   3' AGAAUGGGCUAUGGUCAA 5'

query=JS16_t0337758, target=FGSG_12894, score=3.5, range=329-345, strand=1

target  5' UUUUACCCGAUACU-GUU 3'
          :.::::::::::::. :::
query   3' AGAAUGGGCUAUGGUCAA 5'

query=JS16_t0337758, target=FGSG_03556, score=3.5, range=275-292, strand=-1

target  5' UUUCACCUGAUACCAGUA 3'
          :.: :::.:::::::::
query   3' AGAAUGGGCUAUGGUCAA 5'

query=JS16_t0337758, target=FGSG_08684, score=3.5, range=1679-1696, strand=-1

target  5' CCCAGCCCGAUACCAGUU 3'
           :  .:::::::::::::
query   3' AGAAUGGGCUAUGGUCAA 5'

query=JS16_t0337758, target=FGSG_00669, score=3.5, range=1947-1963, strand=-1

target  5' UUUUACCCG-UACCAGUC 3'
          :.::::::: :::::::
query   3' AGAAUGGGCUAUGGUCAA 5'

query=JS16_t0337758, target=FGSG_09140, score=4, range=1302-1318, strand=1

target  5' GCUU-CCUGAUACCAGUG 3'
           ::: ::.:::::::::
query   3' AGAAUGGGCUAUGGUCAA 5'

query=JS16_t0337758, target=FGSG_12479, score=4, range=212-229, strand=1

target  5' UCGUUCCCAAUACCAGUU 3'
          :: : ::: :::::::::
query   3' AGAAUGGGCUAUGGUCAA 5'
```

```
query=JS16_t3133661, target=FGSG_07075, score=4, range=585-603, strand=1

target  5' CCGUGGUAUGCGAGUAGUU 3'
           :::: ::: .:::::::::
query   3' GGCAGCAUCUGCUCAUCAA 5'

query=JS16_t3133661, target=FGSG_10036, score=4, range=707-725, strand=-1

target  5' UCGUUGUGGACGUGUAGUU 3'
           .:::.::.:::: ::::::
query   3' GGCAGCAUCUGCUCAUCAA 5'

query=JS16_t3133661, target=FGSG_00203, score=4, range=941-959, strand=-1

target  5' AUCAUGUAGACGAGUAGUU 3'
            .  .::::::::::::::
query   3' GGCAGCAUCUGCUCAUCAA 5'

query=JS16_t3133661, target=FGSG_07058, score=4, range=73-92, strand=-1

target  5' UCAUUGUAGACGCAGUAGUU 3'
           .: :.:::::::: :::::::
query   3' GGCAGCAUCUGC-UCAUCAA 5'

No results for JS16_t2237533
query=JS16_t0795586, target=FGSG_10262, score=3.5, range=885-904, strand=-1

target  5' CCGACUUCUUCUUGGUUUCC 3'
            :.::: ::.::::::::::
query   3' CGUUGACGAGGAACCAAAGG 5'

query=JS16_t0795586, target=FGSG_04305, score=4, range=1078-1096, strand=1

target  5' GUCA-UGCUUCUUGGUUUCU 3'
           :. : ::::.:::::::::.
query   3' CGUUGACGAGGAACCAAAGG 5'

query=JS16_t0795586, target=FGSG_03587, score=4, range=364-382, strand=1

target  5' GCGA-UCCUUCUUGGUUUCU 3'
           ::.: : ::.:::::::::.
query   3' CGUUGACGAGGAACCAAAGG 5'

query=JS16_t0795586, target=FGSG_02026, score=4, range=2241-2261, strand=-1

target  5' GCAACAUCCUUCUUGGUUUUC 3'
           ::::: : ::.::::::::.:
query   3' CGUUG-ACGAGGAACCAAAGG 5'

query=JS16_t0795586, target=FGSG_04366, score=4, range=2508-2527, strand=-1

target  5' UCAUCUGCUCCUUGGUUGCC 3'
            :: ::::::::::::: ::
query   3' CGUUGACGAGGAACCAAAGG 5'

query=JS16_t0795586, target=FGSG_10296, score=4, range=165-185, strand=-1

target  5' UUAGCUUCCUCCUUGGUUUCC 3'
            .:.: : :::::::::::::
query   3' CGUUG-ACGAGGAACCAAAGG 5'

query=JS16_t0795586, target=FGSG_05116, score=4, range=613-631, strand=-1

target  5' GCCAC-GCUCCUGGGUUUCC 3'
           :: :: :::::: :::::::
query   3' CGUUGACGAGGAACCAAAGG 5'

query=JS16_t2237597, target=FGSG_06141, score=1.5, range=3967-3984, strand=-1

target  5' AGACAAGAAGUUCUCGUC 3'
           :.::::::::::::::::
query   3' UUUGUUCUUCAAGAGCAU 5'

query=JS16_t2237597, target=FGSG_11427, score=2.5, range=295-312, strand=-1

target  5' GGGCCAGAAGUUCUCGUA 3'
           ...: :::::::::::::
query   3' UUUGUUCUUCAAGAGCAU 5'

query=JS16_t2237597, target=FGSG_07258, score=3, range=1193-1210, strand=-1

target  5' GAAUAAGAAGUUCUUGUC 3'
           .::.:::::::::::.::
query   3' UUUGUUCUUCAAGAGCAU 5'

query=JS16_t2237597, target=FGSG_02506, score=3, range=376-394, strand=-1

target  5' AGUCGGAGAAGUUCUCGUA 3'
           :.  : .:::::::::::::
query   3' UUUG-UUCUUCAAGAGCAU 5'

query=JS16_t0337758, target=FGSG_01481, score=4, range=478-494, strand=1

target  5' ACUUUCCCGAUACC-GUU 3'
            ::: ::::::::: :::
query   3' AGAAUGGGCUAUGGUCAA 5'

query=JS16_t0337758, target=FGSG_05597, score=4, range=631-648, strand=-1

target  5' UGUUACCCGGUACCAGAU 3'
           : :::::::.:::::: :
query   3' AGAAUGGGCUAUGGUCAA 5'

query=JS16_t0337758, target=FGSG_12577, score=4, range=549-566, strand=-1

target  5' UCGUCCUCGAUACCAGUC 3'
           :: : :.::::::::::
query   3' AGAAUGGGCUAUGGUCAA 5'

query=JS16_t0337758, target=FGSG_09503, score=4, range=1218-1234, strand=-1

target  5' CCAUACCC-AUACCAGUU 3'
            : ::::: :::::::::
query   3' AGAAUGGGCUAUGGUCAA 5'

No results for JS16_t2301003
No results for JS16_t1404814
query=JS16_t0803437, target=FGSG_06635, score=3, range=1179-1197, strand=1

target  5' UGUUGCCGUGUACUGGAUC 3'
           :::: ::.:::::.:::::
query   3' ACAA-GGUACAUGGCCUAG 5'

query=JS16_t0803437, target=FGSG_04815, score=3, range=315-331, strand=-1

target  5' UGUGCCAUGUACC-GAUC 3'
           ::: ::::::::: ::::
query   3' ACAAGGUACAUGGCCUAG 5'

query=JS16_t0803437, target=FGSG_12032, score=4, range=414-432, strand=1

target  5' GGUUAGCAUGUACUGGAUC 3'
            :::  :::::::.:::::
query   3' ACAA-GGUACAUGGCCUAG 5'

query=JS16_t0803437, target=FGSG_03831, score=4, range=1344-1362, strand=1

target  5' UGUUGGCAUGUACCGUAUC 3'
           ::::  ::::::::: :::
query   3' ACAA-GGUACAUGGCCUAG 5'

query=JS16_t0803437, target=FGSG_04036, score=4, range=59-76, strand=-1

target  5' UGUUCCAUGUACCGAAGC 3'
           :::::::::::::: : :
query   3' ACAAGGUACAUGGCCUAG 5'

query=JS16_t0803437, target=FGSG_10546, score=4, range=815-832, strand=-1

target  5' UGCUCCAUGUCCUGGAUC 3'
           :: ::::::: :.:::::
query   3' ACAAGGUACAUGGCCUAG 5'

query=JS16_t0803437, target=FGSG_01454, score=4, range=556-574, strand=-1

target  5' UGAUUCCGUGUACCGCAUC 3'
           :: ::::.::::::: :::
query   3' AC-AAGGUACAUGGCCUAG 5'

query=JS16_t0803437, target=FGSG_00342, score=4, range=483-501, strand=-1

target  5' UGUGCGCAUGUGCCGGGUC 3'
           ::: : :::::.::::.::
query   3' ACAAG-GUACAUGGCCUAG 5'

query=JS16_t0803437, target=FGSG_12863, score=4, range=539-556, strand=-1

target  5' AGUACCAUGUACCGGCUC 3'
            :: ::::::::::: ::
query   3' ACAAGGUACAUGGCCUAG 5'

query=JS16_t0803437, target=FGSG_10628, score=4, range=1450-1468, strand=-1

target  5' CGUUGCCAUCUACCGGAUC 3'
            ::: :::: :::::::::
query   3' ACAA-GGUACAUGGCCUAG 5'

query=JS16_t0803437, target=FGSG_07080, score=4, range=1347-1364, strand=-1

target  5' UUUUGCAUGUAUCGGAUG 3'
           : :: ::::::.:::::
query   3' ACAAGGUACAUGGCCUAG 5'
```

```
query=JS16_t2237597, target=FGSG_08272, score=3.5, range=1867-1883, strand=1

target  5’ GAGCAAGAAGUUCUC-UG 3’
           .:.:::::::::::: :.
query   3’ UUUGUUCUUCAAGAGCAU 5’

query=JS16_t2237597, target=FGSG_11083, score=3.5, range=170-187, strand=1

target  5’ GAUCAAGAAGAUCUCGUA 3’
           .: ::::::: :::::::
query   3’ UUUGUUCUUCAAGAGCAU 5’

query=JS16_t2237597, target=FGSG_10771, score=3.5, range=88-104, strand=1

target  5’ GAACAAGAAGUUCU-GUU 3’
           .::::::::::::: ::
query   3’ UUUGUUCUUCAAGAGCAU 5’

query=JS16_t2237597, target=FGSG_03230, score=3.5, range=1046-1063, strand=-1

target  5’ AGAUGAGACGUUCUCGUA 3’
           :.:..::: :::::::::
query   3’ UUUGUUCUUCAAGAGCAU 5’

query=JS16_t2237597, target=FGSG_10218, score=3.5, range=442-459, strand=-1

target  5’ GAGAGAGAAGUUCUUGUA 3’
           .:. .:::::::::.:::
query   3’ UUUGUUCUUCAAGAGCAU 5’

query=JS16_t2237597, target=FGSG_02315, score=3.5, range=16213-16231, strand=-1

target  5’ AAGUGAAGAAGUUCUUGUG 3’
           ::.. ::::::::::.::.
query   3’ UUUG-UUCUUCAAGAGCAU 5’

query=JS16_t2237597, target=FGSG_10897, score=3.5, range=1495-1512, strand=-1

target  5’ GAACAUGAAGUUCUCGUC 3’
           .:::: :::::::::::
query   3’ UUUGUUCUUCAAGAGCAU 5’

query=JS16_t2237597, target=FGSG_09343, score=3.5, range=862-878, strand=-1

target  5’ AAAU-GGAAGUUCUUGUA 3’
           :::. .::::::::.:::
query   3’ UUUGUUCUUCAAGAGCAU 5’

query=JS16_t2237597, target=FGSG_06411, score=4, range=550-567, strand=1

target  5’ AAACGAGAGGUUCUCGAG 3’
           ::::.:::.::::::: .
query   3’ UUUGUUCUUCAAGAGCAU 5’

query=JS16_t2237597, target=FGSG_11349, score=4, range=446-463, strand=1

target  5’ AGAUGAGAAGUGCUCGUG 3’
           :.:..:::::: :::::.
query   3’ UUUGUUCUUCAAGAGCAU 5’

query=JS16_t2237597, target=FGSG_07497, score=4, range=2160-2177, strand=1

target  5’ AGGCAACAAGUUCUCGUU 3’
           :..::: ::::::::::
query   3’ UUUGUUCUUCAAGAGCAU 5’

query=JS16_t2237597, target=FGSG_11550, score=4, range=604-621, strand=1

target  5’ GCGCAAGGAGUUCUCGUU 3’
           . .::::.:::::::::
query   3’ UUUGUUCUUCAAGAGCAU 5’

query=JS16_t2237597, target=FGSG_05260, score=4, range=603-619, strand=1

target  5’ GGACGAGAAGUUCUC-UG 3’
           ..::.:::::::::: :.
query   3’ UUUGUUCUUCAAGAGCAU 5’

query=JS16_t2237597, target=FGSG_10687, score=4, range=277-294, strand=1

target  5’ ACACAAGAGGAUCUCGUA 3’
           : ::::::.: :::::::
query   3’ UUUGUUCUUCAAGAGCAU 5’

query=JS16_t2237597, target=FGSG_08742, score=4, range=232-249, strand=1

target  5’ CAACUAGAACUUCUCGUA 3’
            ::: :::: ::::::::
query   3’ UUUGUUCUUCAAGAGCAU 5’

query=JS16_t2237597, target=FGSG_09090, score=4, range=199-215, strand=1

query=JS16_t0803437, target=FGSG_03223, score=4, range=2070-2086, strand=-1

target  5’ UGUU-CAUGUACCGGUUG 3’
           :::: :::::::::: :
query   3’ ACAAGGUACAUGGCCUAG 5’

query=JS16_t0803437, target=FGSG_08476, score=4, range=1661-1677, strand=-1

target  5’ UGUUCCAGGU-CCGGAUC 3’
           ::::::: :: :::::::
query   3’ ACAAGGUACAUGGCCUAG 5’

query=JS16_t0307969, target=FGSG_10714, score=2, range=467-485, strand=1

target  5’ CUGCGCAACUCAACCUGGG 3’
            ::::::::.:::::::::
query   3’ AACGCGUUGGGUUGGACCC 5’

query=JS16_t0307969, target=FGSG_09304, score=3, range=977-995, strand=1

target  5’ UUACUCAGCCCAACCUGGG 3’
           :: : ::.:::::::::::
query   3’ AACGCGUUGGGUUGGACCC 5’

query=JS16_t0307969, target=FGSG_10548, score=3, range=3230-3248, strand=-1

target  5’ UUCCGCAACUCAACCUGGU 3’
           :: ::::::.::::::::
query   3’ AACGCGUUGGGUUGGACCC 5’

query=JS16_t0307969, target=FGSG_02417, score=3.5, range=342-359, strand=-1

target  5’ UUAUGCAACCC-ACCUGGG 3’
           :: .::::::: :::::::
query   3’ AACGCGUUGGGUUGGACCC 5’

query=JS16_t0307969, target=FGSG_07092, score=4, range=94-113, strand=1

target  5’ UUGCGCAACACCAACUUGGA 3’
           ::::::::: :::::.:::
query   3’ AACGCGUUG-GGUUGGACCC 5’

query=JS16_t0307969, target=FGSG_13934, score=4, range=2342-2359, strand=1

target  5’ UUG-GCGACUCAACCUGGC 3’
           ::: ::.::.::::::::
query   3’ AACGCGUUGGGUUGGACCC 5’

query=JS16_t0307969, target=FGSG_11469, score=4, range=845-863, strand=-1

target  5’ UUGAGGAACUCAGCCUGGG 3’
           ::: : :::.::.::::::
query   3’ AACGCGUUGGGUUGGACCC 5’

query=JS16_t0307969, target=FGSG_07715, score=4, range=441-460, strand=-1

target  5’ UUGCGGGAACCCAACUUGGC 3’
           :::: : ::::::::.:::
query   3’ AACG-CGUUGGGUUGGACCC 5’

No results for JS16_t3197108
No results for JS16_t3197133
No results for JS16_t1404921
query=JS16_t0803446, target=FGSG_10189, score=3, range=1610-1632, strand=-1

target  5’ UCCAUCCGCUGCAUUCUCAAAAG 3’
           :: ::: ::::::::::::::
query   3’ AGCUAGUCGACGUAAGAGUUUUA 5’

query=JS16_t0803446, target=FGSG_11434, score=3, range=931-952, strand=-1

target  5’ UCGA-CUGCUGCAUUCUCAGAAU 3’
           :::: : ::::::::::::.:::
query   3’ AGCUAGUCGACGUAAGAGUUUUA 5’

query=JS16_t0803446, target=FGSG_00604, score=3.5, range=1445-1467, strand=-1

target  5’ UUCACCACCUGCAUUCUCAAAAU 3’
           :.  : :: ::::::::::::::
query   3’ AGCUAGUCGACGUAAGAGUUUUA 5’

No results for JS16_t1404945
No results for JS16_t2301197
No results for JS16_t0693889
query=JS16_t3197268, target=FGSG_06771, score=2, range=865-885, strand=1

target  5’ CUGGAAGCUUACGCCCAUGGU 3’
           ::::::::::::.:::::::
query   3’ GACCUUCGAAUGUGGGUACCC 5’

query=JS16_t3197268, target=FGSG_11268, score=4, range=7232-7252, strand=-1
```

```
target  5' GAGC-AGAAGUUCUCGGA 3'
           .:.: ::::::::::: :
query   3' UUUGUUCUUCAAGAGCAU 5'

query=JS16_t2237597, target=FGSG_06134, score=4, range=186-204, strand=1

target  5' AAACACAGAUGUUCUCGUU 3'
           ::::: ::: ::::::::
query   3' UUUGU-UCUUCAAGAGCAU 5'

query=JS16_t2237597, target=FGSG_00005, score=4, range=961-978, strand=-1

target  5' GAACUGGAAGUUCUUGUG 3'
           .::: .::::::::.::.
query   3' UUUGUUCUUCAAGAGCAU 5'

query=JS16_t2237597, target=FGSG_03226, score=4, range=418-436, strand=-1

target  5' AGACGUGGAAGUUCUUGUA 3'
           :.::. .::::::::.:::
query   3' UUUGU-UCUUCAAGAGCAU 5'

query=JS16_t2237597, target=FGSG_09732, score=4, range=1510-1527, strand=-1

target  5' CCACGAGAGGUUCUCGUG 3'
             ::.:::.::::::::.
query   3' UUUGUUCUUCAAGAGCAU 5'

query=JS16_t2237597, target=FGSG_13663, score=4, range=538-555, strand=-1

target  5' AAACAGGAAGUCCUCGUU 3'
           :::::.::::: :::::
query   3' UUUGUUCUUCAAGAGCAU 5'

query=JS16_t2237597, target=FGSG_01544, score=4, range=1882-1900, strand=-1

target  5' AAACAAGAGGUUGCUCGUC 3'
           ::::::::.::: :::::
query   3' UUUGUUCUUCAA-GAGCAU 5'

query=JS16_t2237597, target=FGSG_10141, score=4, range=546-563, strand=-1

target  5' ACGCGAGAAGUUUUCGUU 3'
           : .:.::::::::.::::
query   3' UUUGUUCUUCAAGAGCAU 5'

query=JS16_t2237597, target=FGSG_07553, score=4, range=1831-1848, strand=-1

target  5' GAACCAGAAGUUGUCGUG 3'
           .::: ::::::: ::::.
query   3' UUUGUUCUUCAAGAGCAU 5'

query=JS16_t2237597, target=FGSG_00418, score=4, range=1876-1893, strand=-1

target  5' GAGGAAGAAGUGCUCGUA 3'
           .:. ::::::: ::::::
query   3' UUUGUUCUUCAAGAGCAU 5'

query=JS16_t2237597, target=FGSG_02044, score=4, range=162-179, strand=-1

target  5' CCACUAGAGGUUCUCGUA 3'
             :: :::.:::::::::
query   3' UUUGUUCUUCAAGAGCAU 5'

query=JS16_t2237597, target=FGSG_10079, score=4, range=166-182, strand=-1

target  5' GAAGAA-AAGUUCUCGUG 3'
           .:: :: ::::::::::.
query   3' UUUGUUCUUCAAGAGCAU 5'

query=JS16_t2237597, target=FGSG_13962, score=4, range=840-858, strand=-1

target  5' AAACAAGACAGUUUUCGUC 3'
           :::::::: ::::.::::
query   3' UUUGUUCU-UCAAGAGCAU 5'

query=JS16_t2237597, target=FGSG_07258, score=4, range=9610-9627, strand=-1

target  5' AAGGAAGAGGUUCUUGUG 3'
           ::. ::::.:::::.::.
query   3' UUUGUUCUUCAAGAGCAU 5'

No results for JS16_t0686278
No results for JS16_t3133738
No results for JS16_t0468549
No results for JS16_t3133772
No results for JS16_t0219779
No results for JS16_t3133815
No results for JS16_t4030333
query=JS16_t4030353, target=FGSG_06141, score=3, range=3968-3988, strand=-1

target  5' CGUGAGACAAGAAGUUCUCGU 3'

target  5' AUGGCAGCUUGCACCCAUGGA 3'
            ::: :::::.:::::::::
query   3' GACCUUCGAAUGUGGGUACCC 5'

query=JS16_t3197268, target=FGSG_04863, score=4, range=153-172, strand=-1

target  5' CUGGGACUUUAC-CCCAUGGG 3'
           ::::.: .:::: ::::::::
query   3' GACCUUCGAAUGUGGGUACCC 5'

query=JS16_t2301256, target=FGSG_11703, score=2.5, range=4201-4220, strand=-1

target  5' GGCGUUGACCUAGGCGACGG 3'
           ::::: .:::::::::::.:
query   3' CCGCACUUGGAUCCGCUGUC 5'

query=JS16_t2301256, target=FGSG_07724, score=3, range=142-160, strand=1

target  5' GGCGUCAACC-AGGCGACAG 3'
           ::::: :::: :::::::::
query   3' CCGCACUUGGAUCCGCUGUC 5'

query=JS16_t2301256, target=FGSG_00270, score=3.5, range=664-683, strand=1

target  5' GGCGCGGACCUAGGCGGCGG 3'
           :::: :.:::::::::.:.:
query   3' CCGCACUUGGAUCCGCUGUC 5'

query=JS16_t2301256, target=FGSG_04286, score=3.5, range=679-698, strand=1

target  5' GGUCUGAACCUGGGCGACAA 3'
           ::.  :::::::.:::::::
query   3' CCGCACUUGGAUCCGCUGUC 5'

query=JS16_t2301256, target=FGSG_07056, score=4, range=328-347, strand=1

target  5' GGCAUGAACCCAGGCGACGG 3'
           ::: :::::: :::::::.:
query   3' CCGCACUUGGAUCCGCUGUC 5'

query=JS16_t2301256, target=FGSG_05177, score=4, range=226-245, strand=1

target  5' GUCGACAACCUAGGCGACAA 3'
           : ::  :::::::::::::
query   3' CCGCACUUGGAUCCGCUGUC 5'

query=JS16_t2301256, target=FGSG_10499, score=4, range=1-20, strand=-1

target  5' GGCUUGAGCCUGGGCGACAU 3'
           ::: :::.:::.:::::::
query   3' CCGCACUUGGAUCCGCUGUC 5'

query=JS16_t2301256, target=FGSG_06486, score=4, range=1901-1920, strand=-1

target  5' AGCAAGAACCUGGGCGACAG 3'
            ::  ::::::.::::::::
query   3' CCGCACUUGGAUCCGCUGUC 5'

query=JS16_t1405038, target=FGSG_04794, score=3.5, range=25-43, strand=1

target  5' ACGUUUACUAUCACGCAGG 3'
            :.:::::: :::::::::
query   3' CGUAAAUGAGAGUGCGUCC 5'

query=JS16_t1405038, target=FGSG_11519, score=3.5, range=1386-1404, strand=-1

target  5' UCGCUUAUUCUCACGCAGG 3'
            :.  :::.:::::::::::
query   3' CGUAAAUGAGAGUGCGUCC 5'

query=JS16_t1405038, target=FGSG_04100, score=4, range=1012-1030, strand=1

target  5' GCAUAUUCUCUCAUGCAGG 3'
           :::: : ::::::.:::::
query   3' CGUAAAUGAGAGUGCGUCC 5'

query=JS16_t1405038, target=FGSG_02236, score=4, range=67-85, strand=1

target  5' GCAGCUACUUUCAUGCAGG 3'
           :::  ::::.:::.:::::
query   3' CGUAAAUGAGAGUGCGUCC 5'

query=JS16_t1405038, target=FGSG_09767, score=4, range=330-348, strand=-1

target  5' CCAUUUAUUCUCACGCAUG 3'
            ::::::.::::::::: :
query   3' CGUAAAUGAGAGUGCGUCC 5'

query=JS16_t1405038, target=FGSG_05392, score=4, range=1555-1572, strand=-1

target  5' GC-UUGACUCUCGCGCAGC 3'
```

```
            . ::.:::::::::::::::
query   3' CUUCUUUGUUCUUCAAGAGCA 5'

No results for JS16_t3133877
No results for JS16_t4030428
No results for JS16_t4030458
query=JS16_t0577579, target=FGSG_11755, score=3, range=1150-1171, strand=1

target  5' AACUUGGUGUUUUUGUCUCUCA 3'
           : :::::::::::::: :::::
query   3' UGGAACCACAAAAACA-AGAGU 5'

query=JS16_t0577579, target=FGSG_01718, score=4, range=675-696, strand=1

target  5' ACCAUGGUCGUUUUUGUUUUCC 3'
           ::: :::: :::::::::.::
query   3' UGGAACCA-CAAAAACAAGAGU 5'

query=JS16_t0577579, target=FGSG_07696, score=4, range=381-401, strand=-1

target  5' ACCGAGGUGUUUUUGUUGUCA 3'
           :::  :::::::::::: :::
query   3' UGGAACCACAAAAACAAGAGU 5'

query=JS16_t0577587, target=FGSG_12400, score=4, range=2-22, strand=-1

target  5' CCA-AGCUGCUUUCAGACUGCA 3'
            :: :::: ::::::::::::
query   3' UGUGUCGAAGAAAGUCUGACGG 5'

No results for JS16_t0194503
query=JS16_t0577600, target=FGSG_11440, score=2.5, range=945-963, strand=1

target  5' CUUGGGCUUUAACCCAGAU 3'
           ::: :.:::::::::.::
query   3' GAA-CUGAAAUUGGGUUUA 5'

query=JS16_t0577600, target=FGSG_06687, score=3, range=550-567, strand=1

target  5' CUUGCCUUUAGCCCAAGU 3'
           :::: :::::.:::::.:
query   3' GAACUGAAAUUGGGUUUA 5'

query=JS16_t0577600, target=FGSG_11576, score=3, range=484-501, strand=1

target  5' CAUGCCUUUAACCCAAAG 3'
           : :: ::::::::::::
query   3' GAACUGAAAUUGGGUUUA 5'

query=JS16_t0577600, target=FGSG_02263, score=3.5, range=64-82, strand=1

target  5' CUUGGCUUUAACACCAAAG 3'
           ::::.:::::::: :::::
query   3' GAACUGAAAUUG-GGUUUA 5'

query=JS16_t0577600, target=FGSG_07363, score=3.5, range=163-180, strand=-1

target  5' CUUGGCUUUGACCUGAAU 3'
           ::::.::::.:::..:::
query   3' GAACUGAAAUUGGGUUUA 5'

query=JS16_t0577600, target=FGSG_01777, score=3.5, range=435-453, strand=-1

target  5' UUUGAACUUUGACUCAAAU 3'
           .::: :::::.::.:::::
query   3' GAAC-UGAAAUUGGGUUUA 5'

query=JS16_t0577600, target=FGSG_03269, score=4, range=2374-2391, strand=1

target  5' CUUGACUUUGACCCCAAG 3'
           :::::::::.:::: ::
query   3' GAACUGAAAUUGGGUUUA 5'

query=JS16_t0577600, target=FGSG_00509, score=4, range=784-801, strand=1

target  5' CUUGACUUCAACCCAGAC 3'
           :::::::: ::::::.:
query   3' GAACUGAAAUUGGGUUUA 5'

query=JS16_t0577600, target=FGSG_00594, score=4, range=1080-1097, strand=1

target  5' UGUGGCUUUAACCCAAGC 3'
           .  ::.:::::::::::.
query   3' GAACUGAAAUUGGGUUUA 5'

query=JS16_t0577600, target=FGSG_02183, score=4, range=607-624, strand=1

target  5' CUUGCCUCUGACCCAAAU 3'
           :::: :: :.::::::::
query   3' GAACUGAAAUUGGGUUUA 5'

query=JS16_t0577600, target=FGSG_03616, score=4, range=121-139, strand=1

           :: :: ::::::.:::::
query   3' CGUAAAUGAGAGUGCGUCC 5'

query=JS16_t1405038, target=FGSG_04580, score=4, range=4214-4231, strand=-1

target  5' CCAUAUACUCUC-CGCAGG 3'
            ::: ::::::: ::::::
query   3' CGUAAAUGAGAGUGCGUCC 5'

query=JS16_t2301292, target=FGSG_09636, score=3.5, range=1335-1355, strand=-1

target  5' AGAUUUGU-UUGGAACCAGUCU 3'
            :::. :: :::::::::::::
query   3' CCUAGCCAUAACCUUGGUCAGA 5'

query=JS16_t2301292, target=FGSG_01366, score=4, range=769-790, strand=1

target  5' GCAUGGGUCUUGGAACCAGUCC 3'
           : :: ::: ::::::::::::
query   3' CCUAGCCAUAACCUUGGUCAGA 5'

No results for JS16_t3197340
query=JS16_t0367284, target=FGSG_10378, score=1.5, range=471-487, strand=-1

target  5' UUG-GCCAUGUCUUGAAG 3'
           :.: ::::::::::::::
query   3' AGCACGGUACAGAACUUC 5'

query=JS16_t0367284, target=FGSG_00923, score=1.5, range=328-344, strand=-1

target  5' UUG-GCCAUGUCUUGAAG 3'
           :.: ::::::::::::::
query   3' AGCACGGUACAGAACUUC 5'

query=JS16_t0367284, target=FGSG_10122, score=2.5, range=402-419, strand=-1

target  5' UUGUGCCAUGUCUUGAUG 3'
           :.:::::::::::::: :
query   3' AGCACGGUACAGAACUUC 5'

query=JS16_t0367284, target=FGSG_00567, score=3, range=7602-7619, strand=-1

target  5' UCGUGCUUUGUCUUGAAG 3'
           ::::::. ::::::::::
query   3' AGCACGGUACAGAACUUC 5'

query=JS16_t0367284, target=FGSG_04200, score=3, range=334-351, strand=-1

target  5' CGGUGCCAUGUCUUGAGG 3'
             ::::::::::::::.:
query   3' AGCACGGUACAGAACUUC 5'

query=JS16_t0367284, target=FGSG_08209, score=3, range=4792-4809, strand=-1

target  5' ACGUGCCAGGUCUUGAAG 3'
            ::::::: :::::::::
query   3' AGCACGGUACAGAACUUC 5'

query=JS16_t0367284, target=FGSG_10850, score=3, range=1685-1702, strand=-1

target  5' UCGUGCCAUGUCCUGAAA 3'
           :::::::::::: ::::
query   3' AGCACGGUACAGAACUUC 5'

query=JS16_t0367284, target=FGSG_00377, score=3, range=345-362, strand=-1

target  5' UCGGGCCACGUCUUGAAG 3'
           ::: :::: :::::::::
query   3' AGCACGGUACAGAACUUC 5'

query=JS16_t0367284, target=FGSG_09082, score=3, range=247-263, strand=-1

target  5' UC-UUCCAUGUCUUGGAG 3'
           :: : ::::::::::.::
query   3' AGCACGGUACAGAACUUC 5'

query=JS16_t0367284, target=FGSG_10059, score=3.5, range=1789-1806, strand=1

target  5' UUAUGCCAUGUCUUGAUG 3'
           :.  ::::::::::::: :
query   3' AGCACGGUACAGAACUUC 5'

query=JS16_t0367284, target=FGSG_12395, score=3.5, range=115-131, strand=1

target  5' UUGU-CCAUGUCUUGGAA 3'
           :.:: ::::::::::.:
query   3' AGCACGGUACAGAACUUC 5'

query=JS16_t0367284, target=FGSG_04722, score=3.5, range=396-413, strand=-1

target  5' UUGUGCCAUGUUUUGAGC 3'
           :.:::::::::.::::.
```

```
target  5’ CUUAACUUUCAACCCGAAU 3’
           ::: ::::: :::::.:::
query   3’ GAACUGAAA-UUGGGUUUA 5’

query=JS16_t0577600, target=FGSG_05731, score=4, range=1295-1312, strand=1

target  5’ CUUACCUUUGGCCCAAAU 3’
           :::  ::::..:::::::
query   3’ GAACUGAAAUUGGGUUUA 5’

query=JS16_t0577600, target=FGSG_09038, score=4, range=196-213, strand=1

target  5’ CGUCACUUUAACUCAAAG 3’
           : : ::::::::.::::
query   3’ GAACUGAAAUUGGGUUUA 5’

query=JS16_t0577600, target=FGSG_09288, score=4, range=338-354, strand=1

target  5’ CUUG-CUUCAACCCAGAU 3’
           :::: ::: ::::::.::
query   3’ GAACUGAAAUUGGGUUUA 5’

query=JS16_t0577600, target=FGSG_12836, score=4, range=793-810, strand=-1

target  5’ CUUGACUUUGGCCCAGAG 3’
           :::::::::..::::.:
query   3’ GAACUGAAAUUGGGUUUA 5’

query=JS16_t0577600, target=FGSG_04900, score=4, range=3-20, strand=-1

target  5’ AUUGACUUUAAUCCAAGC 3’
            ::::::::::.::::.
query   3’ GAACUGAAAUUGGGUUUA 5’

query=JS16_t0577600, target=FGSG_02366, score=4, range=611-628, strand=-1

target  5’ CUAGACUUUGACCCAAGA 3’
           :: ::::::.::::::.
query   3’ GAACUGAAAUUGGGUUUA 5’

query=JS16_t0577600, target=FGSG_05961, score=4, range=857-873, strand=-1

target  5’ CUUGACUUUAG-CCAAGU 3’
           ::::::::::. ::::.:
query   3’ GAACUGAAAUUGGGUUUA 5’

query=JS16_t0577600, target=FGSG_05187, score=4, range=1189-1206, strand=-1

target  5’ CUUGACCUUAACCCCAAU 3’
           :::::: ::::::: :::
query   3’ GAACUGAAAUUGGGUUUA 5’

query=JS16_t0577600, target=FGSG_00848, score=4, range=825-842, strand=-1

target  5’ CCUGCCUUUGACCCAAAA 3’
           : :: ::::.:::::::
query   3’ GAACUGAAAUUGGGUUUA 5’

query=JS16_t0577600, target=FGSG_10579, score=4, range=934-951, strand=-1

target  5’ CGUGUCUUUAGCCCAAAG 3’
           : :: :::::.::::::
query   3’ GAACUGAAAUUGGGUUUA 5’

No results for JS16_t3134060
No results for JS16_t0283254
No results for JS16_t0305877
No results for JS16_t4030637
No results for JS16_t3134153
query=JS16_t4030739, target=FGSG_00070, score=4, range=440-463, strand=1

target  5’ CCUUCAUCUUCAUCACCUUCUUUG 3’
           :::: ::: ::::::: :::::::
query   3’ GGAAUUAG-AGUAGUGCAAGAAAC 5’

No results for JS16_t4030766
query=JS16_t0254645, target=FGSG_05819, score=3.5, range=1171-1189, strand=1

target  5’ GAAUUAGAGGCGUACAUCA 3’
           :::. ::: ::::::::::
query   3’ CUUGCUCUGCGCAUGUAGU 5’

query=JS16_t0254645, target=FGSG_09223, score=3.5, range=683-700, strand=-1

target  5’ GGGUG-GACGCGUACGUCA 3’
           :...: :::::::::.:::
query   3’ CUUGCUCUGCGCAUGUAGU 5’

query=JS16_t0254645, target=FGSG_02999, score=4, range=189-207, strand=1

target  5’ GAUCGAGACGUUUACAUCA 3’
query   3’ AGCACGGUACAGAACUUC 5’

query=JS16_t0367284, target=FGSG_04608, score=3.5, range=820-837, strand=-1

target  5’ UUGUGCCAUGUCCUGGAG 3’
           :.:::::::::: ::.::
query   3’ AGCACGGUACAGAACUUC 5’

query=JS16_t0367284, target=FGSG_06364, score=3.5, range=2292-2310, strand=-1

target  5’ UUGUCGCCAUGUCUUGAGU 3’
           :.:: ::::::::::::.
query   3’ AGCA-CGGUACAGAACUUC 5’

query=JS16_t0367284, target=FGSG_14013, score=3.5, range=627-644, strand=-1

target  5’ UUGAGCCAUAUCUUGAAG 3’
           :.: ::::: ::::::::
query   3’ AGCACGGUACAGAACUUC 5’

query=JS16_t0367284, target=FGSG_05518, score=3.5, range=3882-3899, strand=-1

target  5’ UUGGUCCAUGUCUUGAGG 3’
           :.:  :::::::::::.:
query   3’ AGCACGGUACAGAACUUC 5’

query=JS16_t0367284, target=FGSG_14016, score=3.5, range=730-747, strand=-1

target  5’ UUGAACCAUGUUUUGAAG 3’
           :.:  ::::::.::::::
query   3’ AGCACGGUACAGAACUUC 5’

query=JS16_t0367284, target=FGSG_03027, score=3.5, range=237-255, strand=-1

target  5’ CUGCUUCCAUGUCUUGAAG 3’
            .: : :::::::::::::
query   3’ AGC-ACGGUACAGAACUUC 5’

query=JS16_t0367284, target=FGSG_11001, score=3.5, range=19-35, strand=-1

target  5’ UUGU-CCAUGUCUUGAUG 3’
           :.:: ::::::::::: :
query   3’ AGCACGGUACAGAACUUC 5’

query=JS16_t0367284, target=FGSG_07881, score=4, range=435-452, strand=1

target  5’ UGGUGCCAUGUUUUGGGG 3’
           : :::::::::.:::..:
query   3’ AGCACGGUACAGAACUUC 5’

query=JS16_t0367284, target=FGSG_08812, score=4, range=1020-1037, strand=1

target  5’ UCGAGCCAUGUCGUGGAG 3’
           ::: :::::::: ::.::
query   3’ AGCACGGUACAGAACUUC 5’

query=JS16_t0367284, target=FGSG_09590, score=4, range=814-831, strand=1

target  5’ UCGUUCCAUGUCUGGAGG 3’
           :::: :::::::: ::.:
query   3’ AGCACGGUACAGAACUUC 5’

query=JS16_t0367284, target=FGSG_01283, score=4, range=635-653, strand=1

target  5’ UCGAUGCCAUGUCGUGGAG 3’
           ::: ::::::::: ::.::
query   3’ AGC-ACGGUACAGAACUUC 5’

query=JS16_t0367284, target=FGSG_01268, score=4, range=188-205, strand=1

target  5’ CCAUGCUAUGUCUUGAAU 3’
            : :::.::::::::::
query   3’ AGCACGGUACAGAACUUC 5’

query=JS16_t0367284, target=FGSG_11026, score=4, range=4409-4426, strand=1

target  5’ UCGAGCCUUGUCUUGAAC 3’
           ::: ::: :::::::::
query   3’ AGCACGGUACAGAACUUC 5’

query=JS16_t0367284, target=FGSG_12444, score=4, range=194-210, strand=1

target  5’ UCAU-CCGUGUCUUGGAG 3’
           :: : ::.:::::::.::
query   3’ AGCACGGUACAGAACUUC 5’

query=JS16_t0367284, target=FGSG_13878, score=4, range=8581-8598, strand=1

target  5’ UCGCUCCAAGUCUUGAAG 3’
           :::  ::: :::::::::
query   3’ AGCACGGUACAGAACUUC 5’
```

```
            :: :::::::. :::::::
query   3’ CUUGCUCUGCGCAUGUAGU 5’

query=JS16_t0254645, target=FGSG_06885, score=4, range=1368-1387, strand=1

target  5’ GAACGAGACGCACUACAUCA 3’
           :::::::::::  :::::::
query   3’ CUUGCUCUGCG-CAUGUAGU 5’

No results for JS16_t0254647
No results for JS16_t0468616
No results for JS16_t2238181
query=JS16_t0335689, target=FGSG_05723, score=4, range=383-403, strand=-1

target  5’ CGUCAUCCGCGAUGGCCAUCA 3’
            :: ::::::::.:::::::
query   3’ CCAUUAGGCGCUGCCGGUAGC 5’

No results for JS16_t4030828
query=JS16_t2238211, target=FGSG_11224, score=2.5, range=94-110, strand=-1

target  5’ GUA-UGUGCGUCAUAUAA 3’
           :.: :.:::::::::::::
query   3’ CGUCAUACGCAGUAUAUU 5’

query=JS16_t2238211, target=FGSG_01659, score=3, range=645-662, strand=1

target  5’ AAAGGAUGCGUCAUAUAA 3’
             :: :::::::::::::
query   3’ CGUCAUACGCAGUAUAUU 5’

query=JS16_t2238211, target=FGSG_01609, score=3.5, range=86-102, strand=-1

target  5’ GCGGAAUGCGUCA-AUAA 3’
           ::.: :::::::: ::::
query   3’ CGUCAUACGCAGUAUAUU 5’

query=JS16_t2238211, target=FGSG_02730, score=4, range=1638-1655, strand=-1

target  5’ GGAGUAUGCGUCAUAGGA 3’
           : ::::::::::::: .:
query   3’ CGUCAUACGCAGUAUAUU 5’

query=JS16_t2238211, target=FGSG_02394, score=4, range=3467-3485, strand=-1

target  5’ GCACUUAUGCGUCGUAUAU 3’
           :::  ::::::::.::::
query   3’ CGU-CAUACGCAGUAUAUU 5’

query=JS16_t2238211, target=FGSG_06516, score=4, range=505-522, strand=-1

target  5’ GAAAUAUGCGUCACAUAA 3’
           : : ::::::::: ::::
query   3’ CGUCAUACGCAGUAUAUU 5’

query=JS16_t2238211, target=FGSG_12917, score=4, range=882-899, strand=-1

target  5’ GAUUCAUGCGUCAUAUAA 3’
           :    :::::::::::::
query   3’ CGUCAUACGCAGUAUAUU 5’

query=JS16_t2238211, target=FGSG_11285, score=4, range=596-613, strand=-1

target  5’ GCUUUAUGCGCCAUAUAA 3’
           ::  :::::: :::::::
query   3’ CGUCAUACGCAGUAUAUU 5’

No results for JS16_t2238237
No results for JS16_t4030910
No results for JS16_t2238263
query=JS16_t3134365, target=FGSG_07616, score=4, range=5-24, strand=1

target  5’ ACUUCCUUC-GAAACCGUUUG 3’
           : ::: ::: :::::::::::
query   3’ UAAAGAAAGUCUUUGGCAAAC 5’

query=JS16_t0394505, target=FGSG_00737, score=2.5, range=3034-3054, strand=1

target  5’ GUUGGUGU-UGGAACUCGAGCG 3’
           .:..:::: :::::::::::::
query   3’ UAGUCACAUACCUUGAGCUCGC 5’

query=JS16_t0394505, target=FGSG_07990, score=4, range=998-1020, strand=-1

target  5’ ACCAGAUAUUUGGAACUCGAGCG 3’
           : ::: : : :::::::::::::
query   3’ UAGUC-ACAUACCUUGAGCUCGC 5’

No results for JS16_t0577647
query=JS16_t0365168, target=FGSG_03629, score=3.5, range=668-687, strand=1

target  5’ UCAACAAUGUCAACGCUGAU 3’

query=JS16_t0367284, target=FGSG_11542, score=4, range=1166-1182, strand=1

target  5’ UCUUUCC-UGUCUUGAAG 3’
           :: : :: ::::::::::
query   3’ AGCACGGUACAGAACUUC 5’

query=JS16_t0367284, target=FGSG_04314, score=4, range=1237-1253, strand=1

target  5’ UCGU-CCAUGUCUGGAAA 3’
           :::: :::::::: :::
query   3’ AGCACGGUACAGAACUUC 5’

query=JS16_t0367284, target=FGSG_00437, score=4, range=3425-3443, strand=-1

target  5’ UCGUGCAAUGUCAUUGAAG 3’
           :::::: ::::: ::::::
query   3’ AGCACGGUACAG-AACUUC 5’

query=JS16_t0367284, target=FGSG_05788, score=4, range=1453-1470, strand=-1

target  5’ UCCUGCCAUCUCUUGAGG 3’
           :: :::::: ::::::.:
query   3’ AGCACGGUACAGAACUUC 5’

query=JS16_t0367284, target=FGSG_01196, score=4, range=2119-2137, strand=-1

target  5’ UCAGUGUCAUGUCUUGAUG 3’
           :: :::.:::::::::: :
query   3’ AG-CACGGUACAGAACUUC 5’

query=JS16_t0367284, target=FGSG_11428, score=4, range=648-665, strand=-1

target  5’ CCGUGCCAGGUCUUGAAU 3’
            ::::::: ::::::::
query   3’ AGCACGGUACAGAACUUC 5’

query=JS16_t0367284, target=FGSG_07742, score=4, range=217-234, strand=-1

target  5’ UCGACUCAUGUCUUGAGG 3’
           :::  .::::::::::.:
query   3’ AGCACGGUACAGAACUUC 5’

query=JS16_t0367284, target=FGSG_10938, score=4, range=924-941, strand=-1

target  5’ CAGUACCAUGUCUUGAGG 3’
             :: ::::::::::.:
query   3’ AGCACGGUACAGAACUUC 5’

query=JS16_t0367284, target=FGSG_10970, score=4, range=2415-2431, strand=-1

target  5’ GCGU-CUGUGUCUUGAAG 3’
            ::: :..::::::::::
query   3’ AGCACGGUACAGAACUUC 5’

query=JS16_t0367284, target=FGSG_11438, score=4, range=199-215, strand=-1

target  5’ UCGU-CCAUGUUUUGGAA 3’
           :::: ::::::.:::.:
query   3’ AGCACGGUACAGAACUUC 5’

query=JS16_t0367284, target=FGSG_04328, score=4, range=2698-2715, strand=-1

target  5’ ACUUGACAUGUCUUGAAG 3’
            : :: ::::::::::::
query   3’ AGCACGGUACAGAACUUC 5’

query=JS16_t0367284, target=FGSG_08608, score=4, range=669-686, strand=-1

target  5’ UCAUGCCACGUCUUGAAA 3’
           :: ::::: ::::::::
query   3’ AGCACGGUACAGAACUUC 5’

query=JS16_t0367284, target=FGSG_07105, score=4, range=75-92, strand=-1

target  5’ UCGGGCGAUGUCUUGAAU 3’
           ::: :: ::::::::::
query   3’ AGCACGGUACAGAACUUC 5’

query=JS16_t0367284, target=FGSG_09577, score=4, range=1031-1049, strand=-1

target  5’ UCAGUGCCAUGUCUGGAAA 3’
           :: ::::::::::: :::
query   3’ AG-CACGGUACAGAACUUC 5’

query=JS16_t0367284, target=FGSG_05322, score=4, range=567-583, strand=-1

target  5’ UCGAG-UAUGUCUUGAAG 3’
           ::: : .::::::::::
query   3’ AGCACGGUACAGAACUUC 5’

query=JS16_t0367284, target=FGSG_12866, score=4, range=389-405, strand=-1
```

```
           .:: :: ::::::::::.::
query   3' GGUGGUCACAGUUGCGAUUA 5'

query=JS16_t0365168, target=FGSG_04813, score=3.5, range=1342-1361, strand=-1

target  5' CCAUCAUUGUCAACGCUGAC 3'
           :::.:: ::::::::::.:
query   3' GGUGGUCACAGUUGCGAUUA 5'

query=JS16_t0365168, target=FGSG_04849, score=4, range=47-66, strand=1

target  5' GUGCCAGUGUCAACGCUGGU 3'
            ..::::::::::::::..:
query   3' GGUGGUCACAGUUGCGAUUA 5'

query=JS16_t0365168, target=FGSG_09515, score=4, range=62-81, strand=1

target  5' CCACUGCUGUCAACCCUAAU 3'
           ::::.. ::::::: :::::
query   3' GGUGGUCACAGUUGCGAUUA 5'

query=JS16_t0365168, target=FGSG_13562, score=4, range=1003-1022, strand=-1

target  5' CCACCAGUGACAAUGCUGAU 3'
           ::::::::: :::.:::.::
query   3' GGUGGUCACAGUUGCGAUUA 5'

query=JS16_t0365168, target=FGSG_06628, score=4, range=1192-1211, strand=-1

target  5' UCGACAGUGUCAACGCUGAA 3'
           .:. :::::::::::::.:
query   3' GGUGGUCACAGUUGCGAUUA 5'

query=JS16_t0365168, target=FGSG_07950, score=4, range=1105-1123, strand=-1

target  5' UCAUCA-UGUCAGCGCUAAC 3'
           .::.:: :::::.::::::
query   3' GGUGGUCACAGUUGCGAUUA 5'

No results for JS16_t2238370
query=JS16_t2238392, target=FGSG_00958, score=2, range=1081-1099, strand=-1

target  5' CGAGG-AGAAAAUCUUGAUC 3'
           :::::  :::::::::::::
query   3' GCUCCAGCUUUUAGAACUAG 5'

query=JS16_t2238392, target=FGSG_04505, score=3.5, range=3442-3460, strand=-1

target  5' CGGUGU-GGAAAUCUUGAUC 3'
           ::. :: :.:::::::::::
query   3' GCUCCAGCUUUUAGAACUAG 5'

query=JS16_t2238392, target=FGSG_08969, score=4, range=144-163, strand=1

target  5' CGACUUCGAAAAUGUUGAUC 3'
           :::  :::::::: ::::::
query   3' GCUCCAGCUUUUAGAACUAG 5'

query=JS16_t2238392, target=FGSG_13153, score=4, range=2211-2231, strand=-1

target  5' UGAGGUCGAGAAUCAUUGAUU 3'
           .::::::::.:::: :::::.
query   3' GCUCCAGCUUUUAG-AACUAG 5'

query=JS16_t2238392, target=FGSG_07098, score=4, range=321-340, strand=-1

target  5' CCAGGUCGAGAAUUUUGAUG 3'
           : :::::::.:::.:::::
query   3' GCUCCAGCUUUUAGAACUAG 5'

query=JS16_t2238392, target=FGSG_13920, score=4, range=1438-1458, strand=-1

target  5' UGGGAUCUGGAAAUCUUGAUC 3'
           .:.: :: :.:::::::::::
query   3' GCUCCAG-CUUUUAGAACUAG 5'

query=JS16_t2238392, target=FGSG_09974, score=4, range=82-100, strand=-1

target  5' CGACGUCGAAGAUCUUG-UC 3'
           ::: ::::::.:::::: ::
query   3' GCUCCAGCUUUUAGAACUAG 5'

query=JS16_t2238392, target=FGSG_09271, score=4, range=105-124, strand=-1

target  5' CAAUCUCGAAGAUCUUGAUC 3'
           : :  :::::.:::::::::
query   3' GCUCCAGCUUUUAGAACUAG 5'

query=JS16_t4031055, target=FGSG_12608, score=2.5, range=1816-1833, strand=-1

target  5' UAGCGCUGUAUGACCAGA 3'
           : : .:::::::::::::
```

```
target  5' UC-UGCCAUCUCUUGGAG 3'
           :: :::::: :::::.::
query   3' AGCACGGUACAGAACUUC 5'

query=JS16_t0367284, target=FGSG_11261, score=4, range=697-713, strand=-1

target  5' UC-UGCCAUGUCUUCGAG 3'
           :: ::::::::::: .::
query   3' AGCACGGUACAGAACUUC 5'

query=JS16_t0367284, target=FGSG_12602, score=4, range=125-141, strand=-1

target  5' UCUUGCCAUGUC-UGAGG 3'
           :: ::::::::: :::.:
query   3' AGCACGGUACAGAACUUC 5'

query=JS16_t0367284, target=FGSG_01700, score=4, range=496-512, strand=-1

target  5' UCUUG-CAUGUCUUGAGG 3'
           :: :: ::::::::::.:
query   3' AGCACGGUACAGAACUUC 5'

query=JS16_t0367284, target=FGSG_10936, score=4, range=841-857, strand=-1

target  5' UCGU-CCAUGUGUUGGAG 3'
           :::: :::::: :::.::
query   3' AGCACGGUACAGAACUUC 5'

query=JS16_t0367284, target=FGSG_12339, score=4, range=219-235, strand=-1

target  5' UCGU-CCAUGCCUUGAGG 3'
           :::: ::::: :::::.:
query   3' AGCACGGUACAGAACUUC 5'

query=JS16_t0367284, target=FGSG_10956, score=4, range=205-221, strand=-1

target  5' UCGU-CCAUAUUUUGAAG 3'
           :::: :::: :.::::::
query   3' AGCACGGUACAGAACUUC 5'

query=JS16_t0367284, target=FGSG_13801, score=4, range=1614-1631, strand=-1

target  5' CCGAACCAUGUCUUGAAC 3'
            ::  :::::::::::
query   3' AGCACGGUACAGAACUUC 5'

query=JS16_t0367284, target=FGSG_13232, score=4, range=1312-1328, strand=-1

target  5' GCGU-CCAUGUCUUCAAG 3'
            ::: ::::::::: :::
query   3' AGCACGGUACAGAACUUC 5'

query=JS16_t0367284, target=FGSG_11899, score=4, range=58-74, strand=-1

target  5' ACG-GCCUUGUCUUGAAG 3'
            :: ::: ::::::::::
query   3' AGCACGGUACAGAACUUC 5'

query=JS16_t0367284, target=FGSG_05831, score=4, range=201-217, strand=-1

target  5' UC-UUCCAUGUCUUGAGA 3'
           :: : :::::::::::.
query   3' AGCACGGUACAGAACUUC 5'

query=JS16_t0367284, target=FGSG_10864, score=4, range=238-254, strand=-1

target  5' UCCUCCCAUGU-UUGAAG 3'
           :: : :::::: ::::::
query   3' AGCACGGUACAGAACUUC 5'

No results for JS16_t4094175
No results for JS16_t1405141
No results for JS16_t2301367
No results for JS16_t4094229
query=JS16_t1405200, target=FGSG_01235, score=4, range=989-1010, strand=1

target  5' ACGCGCUGUACCUGGAGAACCA 3'
            ::::   ::::::::::::::
query   3' AGCGCCUAAUGGACCUCUUGGU 5'

query=JS16_t1405200, target=FGSG_00827, score=4, range=233-254, strand=-1

target  5' UCGCCGAUGACCUGGAGAAUCU 3'
           :::: ::: ::::::::::.:
query   3' AGCGCCUAAUGGACCUCUUGGU 5'

No results for JS16_t0284233
No results for JS16_t1405253
query=JS16_t0220442, target=FGSG_09203, score=3, range=636-654, strand=1

target  5' GCGCUUUGUUCUGUUUACU 3'
           :: : .:::::::::::.
```

```
query   3' ACCUUGACAUACUGGUCU 5'

query=JS16_t4031055, target=FGSG_11381, score=3.5, range=128-145, strand=-1

target  5' UGGAAAUGUAUGGCCAGG 3'
           ::::: ::::::.::::.
query   3' ACCUUGACAUACUGGUCU 5'

query=JS16_t4031055, target=FGSG_13170, score=3.5, range=1554-1571, strand=-1

target  5' UCGAACUGUGUGAUCAGG 3'
           : :::::::.:::.:::.
query   3' ACCUUGACAUACUGGUCU 5'

query=JS16_t4031055, target=FGSG_10676, score=3.5, range=820-837, strand=-1

target  5' UGUGCCUGUAUGAUCAGA 3'
           :: . ::::::::.::::
query   3' ACCUUGACAUACUGGUCU 5'

query=JS16_t4031055, target=FGSG_04650, score=4, range=1461-1478, strand=1

target  5' UGGAAAUGUAUCACCAGA 3'
           ::::: ::::: ::::::
query   3' ACCUUGACAUACUGGUCU 5'

query=JS16_t4031055, target=FGSG_11875, score=4, range=256-273, strand=1

target  5' UGGCACUGUAUGACCAAU 3'
           ::: :::::::::::
query   3' ACCUUGACAUACUGGUCU 5'

query=JS16_t4031055, target=FGSG_12370, score=4, range=389-406, strand=1

target  5' CCGACCUGUGUGACCAGA 3'
             :: ::::.::::::::
query   3' ACCUUGACAUACUGGUCU 5'

query=JS16_t4031055, target=FGSG_05192, score=4, range=657-673, strand=1

target  5' CGGGA-UGUAUGACCAGG 3'
            ::.: :::::::::::.
query   3' ACCUUGACAUACUGGUCU 5'

query=JS16_t4031055, target=FGSG_06306, score=4, range=480-498, strand=1

target  5' UGGUACUGCUAUGACCAGC 3'
           ::: :::: :::::::::
query   3' ACCUUGAC-AUACUGGUCU 5'

query=JS16_t4031055, target=FGSG_10601, score=4, range=2704-2722, strand=-1

target  5' UGGAGUUUGUAUGACUAGG 3'
           ::::. .::::::::.::.
query   3' ACCUU-GACAUACUGGUCU 5'

query=JS16_t4031055, target=FGSG_06242, score=4, range=244-261, strand=-1

target  5' UUGAAUUGAAUGACCAGA 3'
           : :::.:: :::::::::
query   3' ACCUUGACAUACUGGUCU 5'

query=JS16_t4031055, target=FGSG_13562, score=4, range=1155-1172, strand=-1

target  5' ACGAACUGGAUGACCAGA 3'
             :::::: :::::::::
query   3' ACCUUGACAUACUGGUCU 5'

query=JS16_t4031055, target=FGSG_11462, score=4, range=137-155, strand=-1

target  5' UGGCGACUGUAUGCCCAGG 3'
           ::: .:::::::: ::::.
query   3' ACC-UUGACAUACUGGUCU 5'

query=JS16_t4031055, target=FGSG_07240, score=4, range=1749-1766, strand=-1

target  5' AGCAACUGAAUGACCAGA 3'
            : ::::: :::::::::
query   3' ACCUUGACAUACUGGUCU 5'

query=JS16_t4031055, target=FGSG_09010, score=4, range=474-492, strand=-1

target  5' UGGAACUGUUAUGACAAGA 3'
           :::::::: :::::: :::
query   3' ACCUUGAC-AUACUGGUCU 5'

No results for JS16_t2238441
No results for JS16_t1341796
No results for JS16_t1341802
No results for JS16_t2238504
No results for JS16_t0335697
No results for JS16_t2238516
```

```
query   3' CGAGCGACAAGACAAAUGG 5'

query=JS16_t0220442, target=FGSG_04143, score=3.5, range=300-318, strand=-1

target  5' UCUUGCUGUUUUGUUUGCC 3'
             ::.::::::.:::::.::
query   3' CGAGCGACAAGACAAAUGG 5'

query=JS16_t0220442, target=FGSG_13787, score=3.5, range=253-272, strand=-1

target  5' GCUUUCUGUCUCUGUUUACC 3'
           :::. :::: ::::::::::
query   3' CGAGCGACA-AGACAAAUGG 5'

query=JS16_t0220442, target=FGSG_01840, score=4, range=544-562, strand=1

target  5' GCUCAAUGUUCUGUUUGCA 3'
           ::::  ::::::::::.:
query   3' CGAGCGACAAGACAAAUGG 5'

query=JS16_t0220442, target=FGSG_13558, score=4, range=131-149, strand=1

target  5' GAUCCAUGUUCUGUUUAUC 3'
           : ::  :::::::::::.:
query   3' CGAGCGACAAGACAAAUGG 5'

query=JS16_t0220442, target=FGSG_06669, score=4, range=343-360, strand=1

target  5' GCUCG-UGUGCUGUUUGCC 3'
           ::::: ::: ::::::.::
query   3' CGAGCGACAAGACAAAUGG 5'

query=JS16_t0220442, target=FGSG_08038, score=4, range=1644-1663, strand=1

target  5' CAUCGCUGUUUCUGUUUACC 3'
               :::::: ::::::::::
query   3' CGAGCGAC-AAGACAAAUGG 5'

query=JS16_t0220442, target=FGSG_03887, score=4, range=179-196, strand=1

target  5' GCUCUCUGUU-UGUUUAUC 3'
           :::: ::::: ::::::.:
query   3' CGAGCGACAAGACAAAUGG 5'

query=JS16_t0220442, target=FGSG_05068, score=4, range=285-303, strand=-1

target  5' AUUUGCUGUUGUGUUUACC 3'
             .:.:::::: :::::::
query   3' CGAGCGACAAGACAAAUGG 5'

query=JS16_t0220442, target=FGSG_01024, score=4, range=978-995, strand=-1

target  5' GCUU-UUGUUCUGUUUGCA 3'
           :::. .::::::::::.:
query   3' CGAGCGACAAGACAAAUGG 5'

query=JS16_t1405340, target=FGSG_10604, score=2.5, range=166-183, strand=-1

target  5' GGUGACACCAGUGUCAAC 3'
           :.:  :::::::::::::
query   3' CUAGGGUGGUCACAGUUG 5'

query=JS16_t1405340, target=FGSG_03589, score=3, range=949-966, strand=1

target  5' GAUCUCAGCAGUGUCAAU 3'
           ::::.:: :::::::::.
query   3' CUAGGGUGGUCACAGUUG 5'

query=JS16_t1405340, target=FGSG_09577, score=3, range=984-1002, strand=1

target  5' GAUCCCCACUAGUGUCAAG 3'
           ::: :::::.::::::::
query   3' CUA-GGGUGGUCACAGUUG 5'

query=JS16_t1405340, target=FGSG_01790, score=3, range=1348-1365, strand=1

target  5' GAGCGCAUCAGUGUCAAC 3'
           :: : ::.::::::::::
query   3' CUAGGGUGGUCACAGUUG 5'

query=JS16_t1405340, target=FGSG_00340, score=3, range=136-153, strand=1

target  5' GUGACCACCAGUGUCAAC 3'
           :    :::::::::::::
query   3' CUAGGGUGGUCACAGUUG 5'

query=JS16_t1405340, target=FGSG_09274, score=3, range=1856-1872, strand=1

target  5' GAU-CGACCAGUGUCAAC 3'
           ::: : ::::::::::::
query   3' CUAGGGUGGUCACAGUUG 5'
```

```
No results for JS16_t0686376
query=JS16_t2238533, target=FGSG_05520, score=3, range=595-616, strand=-1

target  5’ UUCGAUCAUUUUCGAUCAAUUC 3’
           : ::: : ::::::::::::::
query   3’ ACGCUUG-AAAAGCUAGUUAAG 5’

query=JS16_t2238533, target=FGSG_02709, score=4, range=1224-1243, strand=1

target  5’ UGC-AGCUUUUCGAUCAAUCU 3’
           ::: :.:::::::::::: .
query   3’ ACGCUUGAAAAGCUAGUUAAG 5’

No results for JS16_t3134655
query=JS16_t2238558, target=FGSG_02844, score=2.5, range=703-720, strand=1

target  5’ UUUGGGGAGAUCUUUGUG 3’
           :. :::::::::::.:::
query   3’ AGCCCCCUCUAGAAGCAC 5’

query=JS16_t2238558, target=FGSG_05216, score=2.5, range=2724-2741, strand=-1

target  5’ UUGCGGGGGAUCUUCGUG 3’
           :.: :::.::::::::::
query   3’ AGCCCCCUCUAGAAGCAC 5’

query=JS16_t2238558, target=FGSG_06451, score=3, range=1456-1473, strand=1

target  5’ UCGGUGGAGAUAUUCGUG 3’
           :::: :::::: ::::::
query   3’ AGCCCCCUCUAGAAGCAC 5’

query=JS16_t2238558, target=FGSG_13543, score=3, range=2305-2321, strand=-1

target  5’ UC-GGGGAGAUCUUUGUU 3’
           :: :::::::::::.::
query   3’ AGCCCCCUCUAGAAGCAC 5’

query=JS16_t2238558, target=FGSG_08934, score=3, range=1192-1208, strand=-1

target  5’ UC-GCGGAGAUCUUCGUC 3’
           :: : ::::::::::::
query   3’ AGCCCCCUCUAGAAGCAC 5’

query=JS16_t2238558, target=FGSG_12863, score=3.5, range=579-596, strand=-1

target  5’ UUGGGGUAGAUCUUUGUG 3’
           :.:::: :::::::.:::
query   3’ AGCCCCCUCUAGAAGCAC 5’

query=JS16_t2238558, target=FGSG_07237, score=3.5, range=1284-1301, strand=-1

target  5’ AUGGGGGAGAUCUUCUUG 3’
            .::::::::::::: ::
query   3’ AGCCCCCUCUAGAAGCAC 5’

query=JS16_t2238558, target=FGSG_07445, score=3.5, range=1254-1270, strand=-1

target  5’ UUGGGGGAGGUC-UCGUG 3’
           :.:::::::.:: :::::
query   3’ AGCCCCCUCUAGAAGCAC 5’

query=JS16_t2238558, target=FGSG_05198, score=3.5, range=2105-2122, strand=-1

target  5’ UUGUGGGAGAGCUUCGUG 3’
           :.: :::::: :::::::
query   3’ AGCCCCCUCUAGAAGCAC 5’

query=JS16_t2238558, target=FGSG_11482, score=4, range=786-804, strand=1

target  5’ UCGGGAGGAGAUCUUCGGA 3’
           ::::: :::::::::::
query   3’ AGCCC-CCUCUAGAAGCAC 5’

query=JS16_t2238558, target=FGSG_13153, score=4, range=694-711, strand=1

target  5’ GCGUGGGAGAUCUUUGUU 3’
            :: ::::::::::.::
query   3’ AGCCCCCUCUAGAAGCAC 5’

query=JS16_t2238558, target=FGSG_01767, score=4, range=518-535, strand=1

target  5’ CCAGUGGAGAUUUUCGUG 3’
            : : ::::::.::::::
query   3’ AGCCCCCUCUAGAAGCAC 5’

query=JS16_t2238558, target=FGSG_10285, score=4, range=1478-1495, strand=1

target  5’ UCUUGGGAGAUCUUGGUG 3’
           ::  :::::::::: :::
query   3’ AGCCCCCUCUAGAAGCAC 5’

query=JS16_t1405340, target=FGSG_11281, score=3, range=343-360, strand=-1

target  5’ AAUCUCAUCAGUGUCAAU 3’
            :::.::.:::::::::.
query   3’ CUAGGGUGGUCACAGUUG 5’

query=JS16_t1405340, target=FGSG_06160, score=3, range=2470-2487, strand=-1

target  5’ GAUUUCACCAGUCUCAAC 3’
           :::..:::::::: :::::
query   3’ CUAGGGUGGUCACAGUUG 5’

query=JS16_t1405340, target=FGSG_06074, score=3.5, range=2332-2348, strand=1

target  5’ GGUUCCACCAGU-UCAAU 3’
           :.:.:::::::: ::::.
query   3’ CUAGGGUGGUCACAGUUG 5’

query=JS16_t1405340, target=FGSG_13586, score=3.5, range=1837-1855, strand=1

target  5’ GAUUUCCACCAGGGUCAAC 3’
           :: :.::::::: ::::::
query   3’ CU-AGGGUGGUCACAGUUG 5’

query=JS16_t1405340, target=FGSG_07953, score=3.5, range=1669-1686, strand=1

target  5’ GACCGCGCCAGUGUCAAU 3’
           :: : :.:::::::::::.
query   3’ CUAGGGUGGUCACAGUUG 5’

query=JS16_t1405340, target=FGSG_09338, score=3.5, range=3149-3165, strand=1

target  5’ GAUCAC-CCAGUGUCAAU 3’
           :::: : ::::::::::.
query   3’ CUAGGGUGGUCACAGUUG 5’

query=JS16_t1405340, target=FGSG_11566, score=3.5, range=1000-1017, strand=-1

target  5’ GAUCUCACUAGUGUCAGG 3’
           ::::.:::.:::::::.
query   3’ CUAGGGUGGUCACAGUUG 5’

query=JS16_t1405340, target=FGSG_00259, score=3.5, range=1216-1233, strand=-1

target  5’ GAUUCCACCAGUGUUGAA 3’
           :::.::::::::::..:
query   3’ CUAGGGUGGUCACAGUUG 5’

query=JS16_t1405340, target=FGSG_04438, score=3.5, range=1343-1360, strand=-1

target  5’ CAUCCCACCAGUGUAAAU 3’
            ::::::::::::: ::.
query   3’ CUAGGGUGGUCACAGUUG 5’

query=JS16_t1405340, target=FGSG_05435, score=3.5, range=2248-2265, strand=-1

target  5’ GUUCUCUCCAGUGUCAAC 3’
           : ::.: :::::::::::
query   3’ CUAGGGUGGUCACAGUUG 5’

query=JS16_t1405340, target=FGSG_12448, score=3.5, range=480-496, strand=-1

target  5’ GAU-UCACCAGUGUCGAG 3’
           ::: .::::::::::.:
query   3’ CUAGGGUGGUCACAGUUG 5’

query=JS16_t1405340, target=FGSG_03132, score=4, range=652-669, strand=1

target  5’ UAUUCCACCGGUGUCGAU 3’
            ::.:::::.:::::.:.
query   3’ CUAGGGUGGUCACAGUUG 5’

query=JS16_t1405340, target=FGSG_05979, score=4, range=1060-1077, strand=1

target  5’ GAUCCUACCGGUCUCAAC 3’
           :::::.:::.:: :::::
query   3’ CUAGGGUGGUCACAGUUG 5’

query=JS16_t1405340, target=FGSG_07114, score=4, range=442-459, strand=1

target  5’ GAUUUCUUCAGUGUCAAC 3’
           :::..: .::::::::::
query   3’ CUAGGGUGGUCACAGUUG 5’

query=JS16_t1405340, target=FGSG_00431, score=4, range=667-684, strand=1

target  5’ GAUUCCAUCACUGUCAAU 3’
           :::.:::.:: ::::::.
query   3’ CUAGGGUGGUCACAGUUG 5’

query=JS16_t1405340, target=FGSG_08550, score=4, range=478-495, strand=1
```

```
query=JS16_t2238558, target=FGSG_12162, score=4, range=154-171, strand=1

target  5' GCCAGGGAGAUCUUCGUA 3'
             :  :::::::::::::
query   3' AGCCCCCUCUAGAAGCAC 5'

query=JS16_t2238558, target=FGSG_09829, score=4, range=1556-1572, strand=1

target  5' UCCGUGGAGAUCUU-GUG 3'
           :: : ::::::::: :::
query   3' AGCCCCCUCUAGAAGCAC 5'

query=JS16_t2238558, target=FGSG_04582, score=4, range=405-422, strand=-1

target  5' UCGGGGGAGGUCUUUGCG 3'
           :::::::::.::::.: :
query   3' AGCCCCCUCUAGAAGCAC 5'

query=JS16_t2238558, target=FGSG_08428, score=4, range=1006-1023, strand=-1

target  5' ACGGCGGAGAUCUUUGUC 3'
            ::: :::::::::.::
query   3' AGCCCCCUCUAGAAGCAC 5'

query=JS16_t2238558, target=FGSG_05068, score=4, range=820-837, strand=-1

target  5' ACCUGGGGGAUCUUCGUG 3'
             :  :::.::::::::::
query   3' AGCCCCCUCUAGAAGCAC 5'

query=JS16_t2238558, target=FGSG_11659, score=4, range=297-314, strand=-1

target  5' UCGUGGGCGAUCUUCGUC 3'
           ::: ::: :::::::::
query   3' AGCCCCCUCUAGAAGCAC 5'

query=JS16_t2238558, target=FGSG_04958, score=4, range=1176-1193, strand=-1

target  5' CCGUGGGAGAUCUUGGUG 3'
            :: :::::::::: :::
query   3' AGCCCCCUCUAGAAGCAC 5'

query=JS16_t2238558, target=FGSG_08924, score=4, range=1040-1058, strand=-1

target  5' UCGGCUGGGGAUCUUCGUC 3'
           ::::  ::.:::::::::
query   3' AGCC-CCCUCUAGAAGCAC 5'

query=JS16_t2238558, target=FGSG_07888, score=4, range=619-635, strand=-1

target  5' UC-GGGGAGGACUUCGUG 3'
           :: ::::::. :::::::
query   3' AGCCCCCUCUAGAAGCAC 5'

query=JS16_t2238558, target=FGSG_09368, score=4, range=2776-2793, strand=-1

target  5' UGUUCGGAGAUCUUCGUG 3'
           :     :::::::::::::
query   3' AGCCCCCUCUAGAAGCAC 5'

query=JS16_t2238558, target=FGSG_03419, score=4, range=553-569, strand=-1

target  5' UC-GCGGAGAUCUUUGUU 3'
           :: : :::::::::.::
query   3' AGCCCCCUCUAGAAGCAC 5'

No results for JS16_t2238562
No results for JS16_t3134709
No results for JS16_t3134715
No results for JS16_t2238681
No results for JS16_t2238682
query=JS16_t2238706, target=FGSG_04655, score=2, range=172-191, strand=-1

target  5' UGCGAGAGCAAAAGGGGACU 3'
           : :.:.::::::::::::::
query   3' AAGUUUUCGUUUUCCCCUGA 5'

query=JS16_t2238706, target=FGSG_02152, score=3, range=3185-3204, strand=1

target  5' UUGGGAAGCAGAAGGGGACU 3'
           :: ..:::::.:::::::::
query   3' AAGUUUUCGUUUUCCCCUGA 5'

query=JS16_t2238706, target=FGSG_10213, score=3.5, range=298-317, strand=1

target  5' UUCAAAGGCGAAACGGGACU 3'
           ::::::.::.::: ::::::
query   3' AAGUUUUCGUUUUCCCCUGA 5'

query=JS16_t2238706, target=FGSG_04049, score=3.5, range=720-739, strand=1

target  5' UACAAAGGCAAAGGGGGGCU 3'
```

```
target  5' GAUUUCAUCAAUGUCAAC 3'
           :::..::.:: :::::::
query   3' CUAGGGUGGUCACAGUUG 5'

query=JS16_t1405340, target=FGSG_08278, score=4, range=1803-1820, strand=1

target  5' UAUCCUACCAGUCUCAAC 3'
            ::::.:::::: :::::
query   3' CUAGGGUGGUCACAGUUG 5'

query=JS16_t1405340, target=FGSG_00792, score=4, range=418-435, strand=1

target  5' GAUCCCGCCAGUGACAAG 3'
           ::::::.:::::: :::
query   3' CUAGGGUGGUCACAGUUG 5'

query=JS16_t1405340, target=FGSG_06479, score=4, range=2206-2223, strand=1

target  5' GAGCCCACCAUUGUCAGC 3'
           :: ::::::: :::::.:
query   3' CUAGGGUGGUCACAGUUG 5'

query=JS16_t1405340, target=FGSG_10607, score=4, range=319-336, strand=1

target  5' ACUACCAUCAGUGUCAAC 3'
              : :::.::::::::::
query   3' CUAGGGUGGUCACAGUUG 5'

query=JS16_t1405340, target=FGSG_11665, score=4, range=1245-1263, strand=1

target  5' CUUCGCCGCCAGUGUCAAC 3'
              :: ::.::::::::::
query   3' CUAG-GGUGGUCACAGUUG 5'

query=JS16_t1405340, target=FGSG_09912, score=4, range=294-311, strand=1

target  5' AGUUACACCAGUGUCAAG 3'
            .:.  :::::::::::
query   3' CUAGGGUGGUCACAGUUG 5'

query=JS16_t1405340, target=FGSG_05372, score=4, range=7215-7232, strand=1

target  5' GCUGCCGCCAGUGUCAAG 3'
           : : ::.::::::::::
query   3' CUAGGGUGGUCACAGUUG 5'

query=JS16_t1405340, target=FGSG_06172, score=4, range=1821-1839, strand=1

target  5' GACUCCCACCAGUAUCAAG 3'
           :: :::::::::: ::::
query   3' CU-AGGGUGGUCACAGUUG 5'

query=JS16_t1405340, target=FGSG_00836, score=4, range=1422-1440, strand=1

target  5' GAGCACCACCGGUGUCAAG 3'
           :: : :::::.:::::::
query   3' CUAG-GGUGGUCACAGUUG 5'

query=JS16_t1405340, target=FGSG_07348, score=4, range=770-786, strand=1

target  5' GGU-CCACCCGUGUCAAU 3'
           :.: ::::: :::::::.
query   3' CUAGGGUGGUCACAGUUG 5'

query=JS16_t1405340, target=FGSG_01385, score=4, range=361-379, strand=1

target  5' GAUCCCACCAUGUGUCGGC 3'
           :::::::::: :::::..:
query   3' CUAGGGUGGU-CACAGUUG 5'

query=JS16_t1405340, target=FGSG_04738, score=4, range=1408-1425, strand=-1

target  5' GAUCUCGCCAGCGUCAAU 3'
           ::::.:.:::: :::::.
query   3' CUAGGGUGGUCACAGUUG 5'

query=JS16_t1405340, target=FGSG_04813, score=4, range=1348-1366, strand=-1

target  5' GAUCCCCAUCAUUGUCAAC 3'
           ::: ::::.:: :::::::
query   3' CUA-GGGUGGUCACAGUUG 5'

query=JS16_t1405340, target=FGSG_12275, score=4, range=1287-1304, strand=-1

target  5' CCUCCCACCCGUGUCAAC 3'
              ::::::: ::::::::
query   3' CUAGGGUGGUCACAGUUG 5'

query=JS16_t1405340, target=FGSG_00806, score=4, range=502-519, strand=-1

target  5' GAUGAUACCAGUGUCGAC 3'
           :::  .:::::::::.::
```

```
           : ::::.:::::.::::.::
query   3’ AAGUUUUCGUUUUCCCCUGA 5’

query=JS16_t2238706, target=FGSG_02172, score=4, range=376-395, strand=1

target  5’ GGCAAAAGCACAAGGGGACU 3’
             :::::::: :::::::::
query   3’ AAGUUUUCGUUUUCCCCUGA 5’

query=JS16_t2238706, target=FGSG_13783, score=4, range=12687-12706, strand=-1

target  5’ UUUCGAAGCAAAAAGGGACU 3’
           ::. .:::::::: ::::::
query   3’ AAGUUUUCGUUUUCCCCUGA 5’

query=JS16_t2238711, target=FGSG_07469, score=4, range=1609-1630, strand=1

target  5’ UCCG-AUGAGCAGUUUGUUACCA 3’
           : :: :..::::::::::::::
query   3’ ACGCAUGUUCGUCAAACAAUGGA 5’

query=JS16_t4031355, target=FGSG_06580, score=3.5, range=5205-5223, strand=1

target  5’ UCUUGGUGUUGAGUGUCUG 3’
           .::::::::: :::.::::
query   3’ GGAACCACAAAUCAUAGAC 5’

query=JS16_t4031355, target=FGSG_10995, score=4, range=618-635, strand=1

target  5’ CCUUGGUGUUUACU-UCUG 3’
           :::::::::::: : ::::
query   3’ GGAACCACAAAUCAUAGAC 5’

query=JS16_t4031355, target=FGSG_05204, score=4, range=511-529, strand=-1

target  5’ CGAUGGUGUUUGGUAUUUG 3’
           :  ::::::::.::::.::
query   3’ GGAACCACAAAUCAUAGAC 5’

query=JS16_t4031355, target=FGSG_00995, score=4, range=442-460, strand=-1

target  5’ CCAUGGUGUUUGGUCUCUG 3’
           :: ::::::::.:: ::::
query   3’ GGAACCACAAAUCAUAGAC 5’

query=JS16_t4031355, target=FGSG_10454, score=4, range=589-607, strand=-1

target  5’ CCAUGAUGUUUAGUGUCUC 3’
           :: :: ::::::::.:::
query   3’ GGAACCACAAAUCAUAGAC 5’

query=JS16_t4031355, target=FGSG_04912, score=4, range=373-392, strand=-1

target  5’ ACUGGAGUGUUUGGUAUCUG 3’
            :: : ::::::.:::::::
query   3’ GGAAC-CACAAAUCAUAGAC 5’

query=JS16_t4031357, target=FGSG_06183, score=3.5, range=894-911, strand=-1

target  5’ GGCACAGGUUCG-GCCUUG 3’
           :.:::::::::: .:::::
query   3’ CUGUGUCCAAGCAUGGAAC 5’

query=JS16_t4031357, target=FGSG_13709, score=3.5, range=1245-1262, strand=-1

target  5’ GGAACAGGUUCG-ACCUUG 3’
           :.  ::::::::: ::::::
query   3’ CUGUGUCCAAGCAUGGAAC 5’

query=JS16_t4031357, target=FGSG_12765, score=4, range=277-294, strand=1

target  5’ GAC-CAGGUUCCUAUCUUG 3’
           ::: ::::::: ::.::::
query   3’ CUGUGUCCAAGCAUGGAAC 5’

query=JS16_t4031357, target=FGSG_02561, score=4, range=5154-5172, strand=-1

target  5’ GACGCGUGUUCGUGCCUUG 3’
           :::.:.  ::::::.:::::
query   3’ CUGUGUCCAAGCAUGGAAC 5’

query=JS16_t4031357, target=FGSG_07267, score=4, range=1746-1765, strand=-1

target  5’ AACACAUGUUCGUAUCCUUG 3’
            ::::: ::::::: :::::
query   3’ CUGUGUCCAAGCAU-GGAAC 5’

query=JS16_t4031357, target=FGSG_11965, score=4, range=75-92, strand=-1

target  5’ GACAC-GGUACGUACCUUC 3’
           ::::: ::: ::::::::
query   3’ CUGUGUCCAAGCAUGGAAC 5’

query   3’ CUAGGGUGGUCACAGUUG 5’

query=JS16_t1405340, target=FGSG_00366, score=4, range=3909-3927, strand=-1

target  5’ GGUCUUCAGCAGUGUCAAC 3’
           :.:: .:: ::::::::::
query   3’ CUAG-GGUGGUCACAGUUG 5’

query=JS16_t1405340, target=FGSG_11989, score=4, range=2443-2460, strand=-1

target  5’ GGAGCCGCCAGUGUCAAU 3’
           :.   ::.::::::::::.
query   3’ CUAGGGUGGUCACAGUUG 5’

query=JS16_t1405340, target=FGSG_08904, score=4, range=2206-2223, strand=-1

target  5’ CAUCACACCAGUAUCAAC 3’
            ::: ::::::: :::::
query   3’ CUAGGGUGGUCACAGUUG 5’

query=JS16_t1405340, target=FGSG_00428, score=4, range=1900-1917, strand=-1

target  5’ GAUGCCACCAGUGGCAAG 3’
           ::: ::::::::: :::
query   3’ CUAGGGUGGUCACAGUUG 5’

query=JS16_t1405340, target=FGSG_01303, score=4, range=160-176, strand=-1

target  5’ GAU-CUACCAGUGUCUAC 3’
           ::: :.::::::::: ::
query   3’ CUAGGGUGGUCACAGUUG 5’

query=JS16_t1405340, target=FGSG_07325, score=4, range=196-212, strand=-1

target  5’ GAU-GCACCAGUGUUGAC 3’
           :::  :::::::::..::
query   3’ CUAGGGUGGUCACAGUUG 5’

query=JS16_t1405340, target=FGSG_04291, score=4, range=5020-5037, strand=-1

target  5’ GAAACCACCAGUGCCAAC 3’
           ::  ::::::::: ::::
query   3’ CUAGGGUGGUCACAGUUG 5’

No results for JS16_t0255653
query=JS16_t0803482, target=FGSG_11175, score=3, range=226-243, strand=-1

target  5’ UUUC-AGAUGUUCAUUGCA 3’
           ::::  ::::::::::::
query   3’ AAAGCGCUACAAGUAACGA 5’

query=JS16_t0803482, target=FGSG_11560, score=3.5, range=693-712, strand=1

target  5’ UUUUGCGCAUGUUCAUUGCG 3’
           :::.::: :::::::::::
query   3’ AAAGCGC-UACAAGUAACGA 5’

query=JS16_t0803482, target=FGSG_09042, score=4, range=399-418, strand=1

target  5’ UUUCGACGGUGUUUAUUGUU 3’
           ::::: ::.::::.::::.:
query   3’ AAAGC-GCUACAAGUAACGA 5’

query=JS16_t0803482, target=FGSG_12925, score=4, range=8-26, strand=1

target  5’ UUUCAGGGUGUUUAUUGCU 3’
           ::::  :.::::.::::::
query   3’ AAAGCGCUACAAGUAACGA 5’

query=JS16_t0803482, target=FGSG_01378, score=4, range=2571-2589, strand=-1

target  5’ GUUUGUGGUGUUUAUUGCU 3’
            ::.:.:.::::.::::::
query   3’ AAAGCGCUACAAGUAACGA 5’

query=JS16_t0803482, target=FGSG_03522, score=4, range=175-193, strand=-1

target  5’ UUUCGCGAUGUUCCUUCCU 3’
           ::::::::::::: :: ::
query   3’ AAAGCGCUACAAGUAACGA 5’

query=JS16_t0803482, target=FGSG_07359, score=4, range=64-82, strand=-1

target  5’ UUUCGCGAUCUUCUUUGCU 3’
           ::::::::: ::: :::::
query   3’ AAAGCGCUACAAGUAACGA 5’

query=JS16_t0803482, target=FGSG_07127, score=4, range=606-625, strand=-1

target  5’ UUUCGAAAUGUUCAUAUGCU 3’
           :::::  :::::::: ::::
query   3’ AAAGCGCUACAAGUA-ACGA 5’
```

```
No results for JS16_t2238718
query=JS16_t1342050, target=FGSG_13959, score=2, range=694-711, strand=1

target  5’ UGGCCAGAACAGCGAGCA 3’
           :.. ::::::::::::::
query   3’ AUUUGUCUUGUCGCUCGU 5’

query=JS16_t1342050, target=FGSG_07701, score=2, range=1415-1431, strand=-1

target  5’ UAAA-GGAACAGCGAGCA 3’
           :::: .:::::::::::::
query   3’ AUUUGUCUUGUCGCUCGU 5’

query=JS16_t1342050, target=FGSG_11124, score=3, range=1592-1609, strand=1

target  5’ UGAAUGGAACAGCGGGCA 3’
           :.::..::::::::.:::
query   3’ AUUUGUCUUGUCGCUCGU 5’

query=JS16_t1342050, target=FGSG_13389, score=3, range=2214-2231, strand=1

target  5’ CAAGCAGAGCAGCGAGCG 3’
            ::.::::.:::::::::.
query   3’ AUUUGUCUUGUCGCUCGU 5’

query=JS16_t1342050, target=FGSG_10308, score=3, range=165-181, strand=1

target  5’ GAAACAGAACAGCG-GCA 3’
            ::::::::::::: :::
query   3’ AUUUGUCUUGUCGCUCGU 5’

query=JS16_t1342050, target=FGSG_04592, score=3.5, range=894-911, strand=1

target  5’ UAAAGAGAAUAGUGAGCG 3’
           :::: ::::.::.::::.
query   3’ AUUUGUCUUGUCGCUCGU 5’

query=JS16_t1342050, target=FGSG_07383, score=3.5, range=239-256, strand=1

target  5’ CAAGCAGAAGAGCGAGCA 3’
            ::.::::: ::::::::
query   3’ AUUUGUCUUGUCGCUCGU 5’

query=JS16_t1342050, target=FGSG_09518, score=3.5, range=602-619, strand=1

target  5’ CAAGCAGAACAUCGAGCA 3’
            ::.::::::: ::::::
query   3’ AUUUGUCUUGUCGCUCGU 5’

query=JS16_t1342050, target=FGSG_08389, score=3.5, range=652-669, strand=1

target  5’ GAGCCAGGACAGCGAGCA 3’
            :.  :::.:::::::::
query   3’ AUUUGUCUUGUCGCUCGU 5’

query=JS16_t1342050, target=FGSG_09716, score=3.5, range=1164-1181, strand=1

target  5’ AGAAGAGAAUAGCGAGCA 3’
            .:: ::::.::::::::
query   3’ AUUUGUCUUGUCGCUCGU 5’

query=JS16_t1342050, target=FGSG_05367, score=3.5, range=1079-1095, strand=1

target  5’ UGAA-GGAGCAGCGAGCA 3’
           :.:: .::.:::::::::
query   3’ AUUUGUCUUGUCGCUCGU 5’

query=JS16_t1342050, target=FGSG_00786, score=3.5, range=79-95, strand=1

target  5’ AAGACAGAACAGC-AGCA 3’
            :.:::::::::: ::::
query   3’ AUUUGUCUUGUCGCUCGU 5’

query=JS16_t1342050, target=FGSG_07084, score=3.5, range=4322-4339, strand=-1

target  5’ UGAACAGAGCAGCAAGCA 3’
           :.::::::.:::: ::::
query   3’ AUUUGUCUUGUCGCUCGU 5’

query=JS16_t1342050, target=FGSG_04854, score=3.5, range=471-487, strand=-1

target  5’ UAAGCAGAAUAGCGA-CA 3’
           :::.:::::.::::: ::
query   3’ AUUUGUCUUGUCGCUCGU 5’

query=JS16_t1342050, target=FGSG_00481, score=3.5, range=594-610, strand=-1

target  5’ UGACCAGAACAGCG-GCA 3’
           :.: :::::::::: :::
query   3’ AUUUGUCUUGUCGCUCGU 5’

No results for JS16_t3197648
No results for JS16_t3197720
No results for JS16_t0396620
query=JS16_t3197804, target=FGSG_09004, score=3.5, range=960-977, strand=1

target  5’ GC-UCCUCCUCGGUACCGC 3’
           .: :: :::::.:::::::
query   3’ UGAAGUAGGAGUCAUGGCG 5’

query=JS16_t3197804, target=FGSG_01133, score=3.5, range=1508-1527, strand=-1

target  5’ GCAUGCAUUCUCAGUACCGC 3’
           .: : :::.:::::::::::
query   3’ UG-AAGUAGGAGUCAUGGCG 5’

query=JS16_t3197804, target=FGSG_07012, score=3.5, range=737-755, strand=-1

target  5’ GGGUCAUCCUCAGUACCGG 3’
           .  :::::::::::::::
query   3’ UGAAGUAGGAGUCAUGGCG 5’

query=JS16_t3197804, target=FGSG_08601, score=3.5, range=1208-1226, strand=-1

target  5’ ACGCCGUCCUCAGUACCGG 3’
           ::  :.::::::::::::
query   3’ UGAAGUAGGAGUCAUGGCG 5’

query=JS16_t3197804, target=FGSG_03418, score=4, range=587-605, strand=1

target  5’ GCUCCGUCCUCAGUAUCGA 3’
           .:: :.:::::::::.::
query   3’ UGAAGUAGGAGUCAUGGCG 5’

query=JS16_t3197804, target=FGSG_01499, score=4, range=143-161, strand=1

target  5’ ACUCUAUCCCCAGUACCGU 3’
           ::: .:::: ::::::::.
query   3’ UGAAGUAGGAGUCAUGGCG 5’

query=JS16_t3197804, target=FGSG_07926, score=4, range=1758-1776, strand=1

target  5’ CCUUCUUACUCAGUACCGC 3’
            :::: : :::::::::::
query   3’ UGAAGUAGGAGUCAUGGCG 5’

query=JS16_t3197804, target=FGSG_06313, score=4, range=438-455, strand=1

target  5’ AC-UCCUCCUCAGUGUCGC 3’
           :: :: ::::::::..:::
query   3’ UGAAGUAGGAGUCAUGGCG 5’

query=JS16_t3197804, target=FGSG_08373, score=4, range=4734-4751, strand=1

target  5’ AGUUU-UCCUCAGUAUCGU 3’
           : ::. :::::::::.::.
query   3’ UGAAGUAGGAGUCAUGGCG 5’

query=JS16_t3197804, target=FGSG_09660, score=4, range=2322-2341, strand=1

target  5’ ACCUCCUCCUCAGUCACCGC 3’
           :: :: :::::::: :::::
query   3’ UGAAGUAGGAGUCA-UGGCG 5’

query=JS16_t3197804, target=FGSG_05405, score=4, range=988-1005, strand=1

target  5’ ACUUC-UACUCGGUACCGC 3’
           ::::: : :::.:::::::
query   3’ UGAAGUAGGAGUCAUGGCG 5’

query=JS16_t3197804, target=FGSG_04428, score=4, range=453-470, strand=-1

target  5’ UCUUCAUCCUCAGU-CUGC 3’
            ::::::::::::: :.::
query   3’ UGAAGUAGGAGUCAUGGCG 5’

query=JS16_t3197804, target=FGSG_01643, score=4, range=200-218, strand=-1

target  5’ ACGCCAUCCUCAGUGCCGG 3’
           ::  ::::::::::.:::
query   3’ UGAAGUAGGAGUCAUGGCG 5’

query=JS16_t3197804, target=FGSG_05556, score=4, range=1478-1495, strand=-1

target  5’ AC-UCAUCCUCAUUAUCGC 3’
           :: ::::::::: ::.:::
query   3’ UGAAGUAGGAGUCAUGGCG 5’

query=JS16_t3197804, target=FGSG_08032, score=4, range=57-74, strand=-1

target  5’ ACUUCUUCUUCAGU-CCGC 3’
           ::::: ::.::::: ::::
```

```
query=JS16_t1342050, target=FGSG_09998, score=3.5, range=117-133, strand=-1

target  5’ UGAACAGAAC-GUGAGCA 3’
           :.:::::::: :.:::::
query   3’ AUUUGUCUUGUCGCUCGU 5’

query=JS16_t1342050, target=FGSG_00487, score=4, range=347-364, strand=1

target  5’ CAAAUGGAACGGCGAGCG 3’
            :::..::::.::::::.
query   3’ AUUUGUCUUGUCGCUCGU 5’

query=JS16_t1342050, target=FGSG_00712, score=4, range=1361-1378, strand=1

target  5’ CAAGCAGAGCAGCGGGCG 3’
            ::.::::.:::::.::.
query   3’ AUUUGUCUUGUCGCUCGU 5’

query=JS16_t1342050, target=FGSG_07513, score=4, range=156-173, strand=1

target  5’ CAGGCACAACAGCGAGCA 3’
            :..:: :::::::::::
query   3’ AUUUGUCUUGUCGCUCGU 5’

query=JS16_t1342050, target=FGSG_09114, score=4, range=267-284, strand=1

target  5’ AAAAGAGAGCAGUGAGCA 3’
            ::: :::.:::.:::::
query   3’ AUUUGUCUUGUCGCUCGU 5’

query=JS16_t1342050, target=FGSG_12027, score=4, range=280-298, strand=1

target  5’ AGAACAGAACAGCGCAGCG 3’
            .:::::::::::: :::.
query   3’ AUUUGUCUUGUCGC-UCGU 5’

query=JS16_t1342050, target=FGSG_09843, score=4, range=267-284, strand=1

target  5’ UAAACAGAUCAGCGAGGA 3’
           :::::::: ::::::: :
query   3’ AUUUGUCUUGUCGCUCGU 5’

query=JS16_t1342050, target=FGSG_02871, score=4, range=207-224, strand=1

target  5’ UCAACAGAACAUUGAGCA 3’
           : ::::::::: .:::::
query   3’ AUUUGUCUUGUCGCUCGU 5’

query=JS16_t1342050, target=FGSG_12041, score=4, range=1806-1823, strand=1

target  5’ UAACAAGGGCAGCGAGCA 3’
           :::  ::..:::::::::
query   3’ AUUUGUCUUGUCGCUCGU 5’

query=JS16_t1342050, target=FGSG_02394, score=4, range=381-397, strand=1

target  5’ CAAACAGAACAGUGA-CA 3’
            :::::::::::.:: ::
query   3’ AUUUGUCUUGUCGCUCGU 5’

query=JS16_t1342050, target=FGSG_05220, score=4, range=526-542, strand=1

target  5’ GAGACAGAACAGC-AGCG 3’
            :.:::::::::: :::.
query   3’ AUUUGUCUUGUCGCUCGU 5’

query=JS16_t1342050, target=FGSG_07698, score=4, range=2040-2056, strand=1

target  5’ UAACGAGAACAGCG-GCA 3’
           :::  ::::::::: :::
query   3’ AUUUGUCUUGUCGCUCGU 5’

query=JS16_t1342050, target=FGSG_12678, score=4, range=1239-1256, strand=-1

target  5’ UGAAUAGAACUGUGAGCA 3’
           :.::.::::: :.:::::
query   3’ AUUUGUCUUGUCGCUCGU 5’

query=JS16_t1342050, target=FGSG_09363, score=4, range=410-427, strand=-1

target  5’ UGAUUAGAGCAGCGAGUA 3’
           :.: .:::.:::::::.:
query   3’ AUUUGUCUUGUCGCUCGU 5’

query=JS16_t1342050, target=FGSG_05770, score=4, range=400-418, strand=-1

target  5’ CAAACAAGAACGGCGAGUA 3’
            :::: :::::.::::::.:
query   3’ AUUUG-UCUUGUCGCUCGU 5’

query=JS16_t1342050, target=FGSG_10021, score=4, range=681-697, strand=-1

query   3’ UGAAGUAGGAGUCAUGGCG 5’

query=JS16_t0337790, target=FGSG_12689, score=3, range=1507-1525, strand=1

target  5’ GAAAUGGAACAGAUGGUAA 3’
           ::::..:: ::::::::::
query   3’ CUUUGUCUCGUCUACCAUU 5’

query=JS16_t0337790, target=FGSG_06410, score=3, range=655-672, strand=-1

target  5’ GGGA-AGAGCAGAUGGUAU 3’
           :..: :::::::::::::
query   3’ CUUUGUCUCGUCUACCAUU 5’

query=JS16_t0337790, target=FGSG_01092, score=3.5, range=385-403, strand=1

target  5’ GAGACAGAGCAGCUGGUGA 3’
           ::.::::::::: ::::.:
query   3’ CUUUGUCUCGUCUACCAUU 5’

query=JS16_t0337790, target=FGSG_02315, score=3.5, range=8068-8086, strand=1

target  5’ GGAAUAGAGGAGAUGGUAG 3’
           :.::.:::: ::::::::.
query   3’ CUUUGUCUCGUCUACCAUU 5’

query=JS16_t0337790, target=FGSG_02011, score=3.5, range=256-274, strand=1

target  5’ CAAAAGGAGCAGAUGGUGA 3’
            ::: .::::::::::.:
query   3’ CUUUGUCUCGUCUACCAUU 5’

query=JS16_t0337790, target=FGSG_01618, score=3.5, range=615-632, strand=1

target  5’ CAAGCAGAGCA-AUGGUAA 3’
            ::.::::::: :::::::
query   3’ CUUUGUCUCGUCUACCAUU 5’

query=JS16_t0337790, target=FGSG_06596, score=4, range=167-185, strand=1

target  5’ GGAAGAGAGAAGAUGGUAG 3’
           :.:: :::: ::::::::.
query   3’ CUUUGUCUCGUCUACCAUU 5’

query=JS16_t0337790, target=FGSG_01309, score=4, range=712-730, strand=1

target  5’ GAAUUGGAGGAGAUGGUAA 3’
           ::: ..::: :::::::::
query   3’ CUUUGUCUCGUCUACCAUU 5’

query=JS16_t0337790, target=FGSG_05326, score=4, range=905-923, strand=1

target  5’ GCAACCGAACAGAUGGUAA 3’
           : ::: :: ::::::::::
query   3’ CUUUGUCUCGUCUACCAUU 5’

query=JS16_t0337790, target=FGSG_07150, score=4, range=887-904, strand=1

target  5’ CAAAC-GAGCAGACGGUAA 3’
            :::: ::::::: :::::
query   3’ CUUUGUCUCGUCUACCAUU 5’

query=JS16_t0337790, target=FGSG_08116, score=4, range=1011-1028, strand=1

target  5’ GAAACAGAGCA-AUGGCAA 3’
           :::::::::: :::: ::
query   3’ CUUUGUCUCGUCUACCAUU 5’

query=JS16_t0337790, target=FGSG_10374, score=4, range=687-705, strand=-1

target  5’ GGAAGAGAGCAGAAGGUAG 3’
           :.:: :::::::: ::::.
query   3’ CUUUGUCUCGUCUACCAUU 5’

query=JS16_t0337790, target=FGSG_11890, score=4, range=880-899, strand=-1

target  5’ GACAACGGAGCAGAUGGAAG 3’
           :: :::.::::::::::: :.
query   3’ CU-UUGUCUCGUCUACCAUU 5’

query=JS16_t0337790, target=FGSG_09013, score=4, range=242-260, strand=-1

target  5’ GCGUCGGAGUAGAUGGUAA 3’
           : .  :.:::.::::::::
query   3’ CUUUGUCUCGUCUACCAUU 5’

query=JS16_t0337790, target=FGSG_00052, score=4, range=68-86, strand=-1

target  5’ CAAAGAGAGCAGAUGGGAA 3’
            ::: ::::::::::: ::
query   3’ CUUUGUCUCGUCUACCAUU 5’
```

```
target  5’ UAAA-AGGACGGCGGGCA 3’
           :::: ::.::.:::.:::
query   3’ AUUUGUCUUGUCGCUCGU 5’

query=JS16_t1342050, target=FGSG_05682, score=4, range=880-897, strand=-1

target  5’ UACACAGAAUAGCGAGAA 3’
           :: :::::.:::::: :
query   3’ AUUUGUCUUGUCGCUCGU 5’

query=JS16_t1342050, target=FGSG_02047, score=4, range=449-466, strand=-1

target  5’ UGAUGAGAAUAGCGAGCG 3’
           :.:  ::::.::::::::.
query   3’ AUUUGUCUUGUCGCUCGU 5’

query=JS16_t1342050, target=FGSG_01320, score=4, range=282-299, strand=-1

target  5’ CACUCAGAACGGCGAGCA 3’
            :  ::::::.:::::::
query   3’ AUUUGUCUUGUCGCUCGU 5’

query=JS16_t1342050, target=FGSG_01388, score=4, range=967-985, strand=-1

target  5’ CAACCAGGAACAGCGAGCA 3’
            :: :: ::::::::::::
query   3’ AUUUGU-CUUGUCGCUCGU 5’

query=JS16_t1342050, target=FGSG_01889, score=4, range=1349-1367, strand=-1

target  5’ UUGGCAGAAGCAGCGAGCA 3’
           : ..::::: :::::::::
query   3’ AUUUGUCUU-GUCGCUCGU 5’

query=JS16_t1342050, target=FGSG_03887, score=4, range=178-194, strand=-1

target  5’ UAAACA-AACAGAGAGCA 3’
           :::::: ::::: :::::
query   3’ AUUUGUCUUGUCGCUCGU 5’

query=JS16_t1342050, target=FGSG_11419, score=4, range=137-153, strand=-1

target  5’ CAACCAGAACAG-GAGCA 3’
            :: :::::::: :::::
query   3’ AUUUGUCUUGUCGCUCGU 5’

No results for JS16_t0202198
query=JS16_t0283269, target=FGSG_01580, score=3.5, range=413-430, strand=1

target  5’ GUUUC-UGGUUUUCGUAUG 3’
           :::.: :::::::::::.
query   3’ CAAGGUACCAAAAGCAUGA 5’

query=JS16_t0283269, target=FGSG_03966, score=3.5, range=1025-1043, strand=-1

target  5’ GUGCGGUGGUUUUCGUAUU 3’
           :: : .:::::::::::.:
query   3’ CAAGGUACCAAAAGCAUGA 5’

query=JS16_t0283269, target=FGSG_05463, score=4, range=413-432, strand=1

target  5’ AUUGCAUGGUUUUCGUAGCU 3’
            :: ::::::::::::: ::
query   3’ CAAGGUACCAAAAGCAU-GA 5’

query=JS16_t0119371, target=FGSG_01354, score=3, range=1182-1199, strand=1

target  5’ CG-AGUGGUUCCACUGGUC 3’
           :: :...::::::::::::
query   3’ GCAUUGUCAAGGUGACCAG 5’

query=JS16_t0119371, target=FGSG_07985, score=3, range=1681-1699, strand=-1

target  5’ CGUAGCCGUUCCACUGGUU 3’
           ::::.: :::::::::::.
query   3’ GCAUUGUCAAGGUGACCAG 5’

query=JS16_t0119371, target=FGSG_01909, score=3.5, range=581-599, strand=1

target  5’ CAGAGCAGUUCCACUGGUG 3’
           :  :.:::::::::::::
query   3’ GCAUUGUCAAGGUGACCAG 5’

query=JS16_t0119371, target=FGSG_05643, score=4, range=1116-1135, strand=1

target  5’ UGUAAUUAUUUCCACUGGUC 3’
           .:::: .: :::::::::::
query   3’ GCAUU-GUCAAGGUGACCAG 5’

query=JS16_t0119371, target=FGSG_08022, score=4, range=681-698, strand=1

target  5’ UGUCG-AGUUCCACUGGUA 3’

query=JS16_t0337790, target=FGSG_00045, score=4, range=213-230, strand=-1

target  5’ GGAA-GGAGGAGAUGGUAA 3’
           :.:: .::: :::::::::
query   3’ CUUUGUCUCGUCUACCAUU 5’

No results for JS16_t1405555
No results for JS16_t4094671
No results for JS16_t1405724
No results for JS16_t0803550
query=JS16_t1405812, target=FGSG_10196, score=3, range=1077-1095, strand=-1

target  5’ GAAUAG-ACGGAUGAUAAAG 3’
           :  ::: :::::::::::::
query   3’ CACAUCUUGCCUACUAUUUC 5’

query=JS16_t1405812, target=FGSG_10866, score=4, range=204-224, strand=-1

target  5’ AUGUAGAGACGGGUGAUGAAG 3’
            :::::: ::::.::::.:::
query   3’ CACAUCU-UGCCUACUAUUUC 5’

query=JS16_t1405812, target=FGSG_13625, score=4, range=737-756, strand=-1

target  5’ UCUUAGAGCGGAUGAUAAAG 3’
              ::::.::::::::::::
query   3’ CACAUCUUGCCUACUAUUUC 5’

query=JS16_t1405812, target=FGSG_02700, score=4, range=753-771, strand=-1

target  5’ GUGU-GCACGGAUGGUAGAG 3’
           :::: : :::::::.::.::
query   3’ CACAUCUUGCCUACUAUUUC 5’

No results for JS16_t0396631
No results for JS16_t0396632
query=JS16_t4094876, target=FGSG_08487, score=2, range=4511-4528, strand=1

target  5’ AUCGUCUGGAUUCUUUUG 3’
           ::::::::::::::::.:
query   3’ UAGCAGACCUAAGAAGAA 5’

query=JS16_t4094876, target=FGSG_03980, score=2, range=1828-1845, strand=1

target  5’ AUCCUCUGGAUUCUUCUC 3’
           ::: :::::::::::::
query   3’ UAGCAGACCUAAGAAGAA 5’

query=JS16_t4094876, target=FGSG_00156, score=2, range=196-213, strand=-1

target  5’ ACCGUCUGGAUUCUUCUC 3’
           : :::::::::::::::
query   3’ UAGCAGACCUAAGAAGAA 5’

query=JS16_t4094876, target=FGSG_07962, score=3, range=1955-1971, strand=1

target  5’ AUCGUCUGG-UUCUUUUU 3’
           ::::::::: :::::.::
query   3’ UAGCAGACCUAAGAAGAA 5’

query=JS16_t4094876, target=FGSG_02094, score=3, range=808-825, strand=1

target  5’ AUCGCCUGGAUUCUACUU 3’
           :::: ::::::::: :::
query   3’ UAGCAGACCUAAGAAGAA 5’

query=JS16_t4094876, target=FGSG_09877, score=3, range=685-701, strand=1

target  5’ AUCG-CUGGUUUCUUCUU 3’
           :::: :::: ::::::::
query   3’ UAGCAGACCUAAGAAGAA 5’

query=JS16_t4094876, target=FGSG_10300, score=3, range=1101-1117, strand=1

target  5’ CUCGUCU-GAUUCUUCUU 3’
            :::::: ::::::::::
query   3’ UAGCAGACCUAAGAAGAA 5’

query=JS16_t4094876, target=FGSG_01065, score=3, range=1426-1443, strand=-1

target  5’ AUCAUCUGGAAUCUUCUU 3’
           ::: :::::: :::::::
query   3’ UAGCAGACCUAAGAAGAA 5’

query=JS16_t4094876, target=FGSG_05622, score=3, range=305-321, strand=-1

target  5’ AUC-UCUGGAUACUUCUU 3’
           ::: ::::::: ::::::
query   3’ UAGCAGACCUAAGAAGAA 5’

query=JS16_t4094876, target=FGSG_06016, score=3.5, range=4076-4094, strand=1
```

```
           .:: . ::::::::::::
query   3’ GCAUUGUCAAGGUGACCAG 5’

query=JS16_t0119371, target=FGSG_07493, score=4, range=2988-3005, strand=1

target  5’ CG-ACCAGUUCCACAGGUC 3’
           :: : ::::::::: ::::
query   3’ GCAUUGUCAAGGUGACCAG 5’

query=JS16_t0119371, target=FGSG_11026, score=4, range=7452-7470, strand=-1

target  5’ GGUAACAGUGCCACUGGUA 3’
            :::::::: ::::::::
query   3’ GCAUUGUCAAGGUGACCAG 5’

query=JS16_t0119371, target=FGSG_05298, score=4, range=335-354, strand=-1

target  5’ UGUAUAUAGUUCCACUUGUC 3’
           .::: :.::::::::: :::
query   3’ GCAU-UGUCAAGGUGACCAG 5’

query=JS16_t0119371, target=FGSG_03894, score=4, range=210-228, strand=-1

target  5’ GGUAAGAGUUCUACUGGUG 3’
            :::: :::::.::::::
query   3’ GCAUUGUCAAGGUGACCAG 5’

query=JS16_t0119371, target=FGSG_01564, score=4, range=581-600, strand=-1

target  5’ AGUAUCAAGUUCCAUUGGUC 3’
            ::: : :::::::.:::::
query   3’ GCAUUG-UCAAGGUGACCAG 5’

query=JS16_t0119371, target=FGSG_10982, score=4, range=1711-1728, strand=-1

target  5’ CAUAGCAGUUCCAC-GGUU 3’
           : ::.::::::::: :::.
query   3’ GCAUUGUCAAGGUGACCAG 5’

query=JS16_t0119371, target=FGSG_13933, score=4, range=301-318, strand=-1

target  5’ UCUGA-GGUUCCACUGGUC 3’
           . :.: .::::::::::::
query   3’ GCAUUGUCAAGGUGACCAG 5’

No results for JS16_t3134848
No results for JS16_t0686408
No results for JS16_t0335711
query=JS16_t4031509, target=FGSG_09661, score=4, range=989-1011, strand=1

target  5’ GCAAGCAGUUUGGCUCUCUGGUC 3’
           :: ::  ::::::::::::::
query   3’ CGCUCCACAAACCGAGAGACCAA 5’

No results for JS16_t0142861
No results for JS16_t0468720
query=JS16_t2239020, target=FGSG_07937, score=3, range=2079-2097, strand=1

target  5’ AGGUCCUGGAGGUCGUCCU 3’
           :::: ::.::.::::::::
query   3’ UCCA-GAUCUUCAGCAGGA 5’

query=JS16_t2239020, target=FGSG_13160, score=3, range=271-288, strand=1

target  5’ CUGUCUAGGAGUCGUCCU 3’
             ::::::.:::::::::
query   3’ UCCAGAUCUUCAGCAGGA 5’

query=JS16_t2239020, target=FGSG_11001, score=3.5, range=1-19, strand=-1

target  5’ GGGUCGUAGAAGUCGUCAU 3’
           .:::: ::::::::::: :
query   3’ UCCAG-AUCUUCAGCAGGA 5’

query=JS16_t2239020, target=FGSG_09515, score=4, range=3769-3786, strand=1

target  5’ AGGUCUAGAAGUCCUUCC 3’
           ::::::::::::: :.:
query   3’ UCCAGAUCUUCAGCAGGA 5’

query=JS16_t2239020, target=FGSG_03862, score=4, range=1212-1229, strand=1

target  5’ AGGUCUAGCAGUCGGCCU 3’
           :::::::: ::::: :::
query   3’ UCCAGAUCUUCAGCAGGA 5’

query=JS16_t2239020, target=FGSG_01990, score=4, range=1447-1464, strand=1

target  5’ ACUUCUGGAAGUCGUCUU 3’
           :  :::.:::::::::.:
query   3’ UCCAGAUCUUCAGCAGGA 5’

target  5’ AUCGUUUGGAUUUGUUCUU 3’
           :::::.::::::. :::::
query   3’ UAGCAGACCUAAG-AAGAA 5’

query=JS16_t4094876, target=FGSG_07873, score=3.5, range=853-870, strand=1

target  5’ GUCGGCUGGUUUCUUCUU 3’
           .::: :::: ::::::::
query   3’ UAGCAGACCUAAGAAGAA 5’

query=JS16_t4094876, target=FGSG_11561, score=3.5, range=1085-1101, strand=1

target  5’ AUU-UCUGCAUUCUUCUU 3’
           ::. :::: :::::::::
query   3’ UAGCAGACCUAAGAAGAA 5’

query=JS16_t4094876, target=FGSG_08703, score=3.5, range=22-39, strand=-1

target  5’ CUUGUCUGGAUGCUUCUU 3’
            :.:::::::: ::::::
query   3’ UAGCAGACCUAAGAAGAA 5’

query=JS16_t4094876, target=FGSG_07830, score=3.5, range=85-101, strand=-1

target  5’ GUCGUCUGGA-UCUUUUU 3’
           .:::::::::: ::::.::
query   3’ UAGCAGACCUAAGAAGAA 5’

query=JS16_t4094876, target=FGSG_04425, score=3.5, range=2332-2349, strand=-1

target  5’ GUCAUCAGGAUUCUUCUU 3’
           .:: :: :::::::::::
query   3’ UAGCAGACCUAAGAAGAA 5’

query=JS16_t4094876, target=FGSG_13153, score=3.5, range=121-138, strand=-1

target  5’ GGCGUCUGGAUACUUCUU 3’
           .  ::::::::: ::::::
query   3’ UAGCAGACCUAAGAAGAA 5’

query=JS16_t4094876, target=FGSG_09575, score=3.5, range=2572-2589, strand=-1

target  5’ GGCAUCUGGGUUCUUCUU 3’
           .  : :::::.::::::::
query   3’ UAGCAGACCUAAGAAGAA 5’

query=JS16_t4094876, target=FGSG_07544, score=3.5, range=1603-1620, strand=-1

target  5’ GGCCUCUGGAUUCUUUUU 3’
           .  : :::::::::::.::
query   3’ UAGCAGACCUAAGAAGAA 5’

query=JS16_t4094876, target=FGSG_09654, score=3.5, range=359-375, strand=-1

target  5’ GACGU-UGGAUUCUUCUU 3’
           .  ::: ::::::::::::
query   3’ UAGCAGACCUAAGAAGAA 5’

query=JS16_t4094876, target=FGSG_00338, score=4, range=322-339, strand=1

target  5’ AGCGUUUGGGUUCUUCUC 3’
           :  :::.:::.:::::::
query   3’ UAGCAGACCUAAGAAGAA 5’

query=JS16_t4094876, target=FGSG_05532, score=4, range=214-231, strand=1

target  5’ AUCGGCUGGAUUGUUCUC 3’
           :::: ::::::: ::::
query   3’ UAGCAGACCUAAGAAGAA 5’

query=JS16_t4094876, target=FGSG_11417, score=4, range=781-798, strand=1

target  5’ AUGUUCUGGAUUCUUUUC 3’
           ::  ::::::::::.:
query   3’ UAGCAGACCUAAGAAGAA 5’

query=JS16_t4094876, target=FGSG_05236, score=4, range=1114-1130, strand=1

target  5’ AUC-UUUGGAUUCGUCUU 3’
           ::: :.::::::: ::::
query   3’ UAGCAGACCUAAGAAGAA 5’

query=JS16_t4094876, target=FGSG_05404, score=4, range=1129-1145, strand=1

target  5’ AUC-UUUGGUUUCUUCUU 3’
           ::: :.::: ::::::::
query   3’ UAGCAGACCUAAGAAGAA 5’

query=JS16_t4094876, target=FGSG_09337, score=4, range=1324-1341, strand=1

target  5’ AAAGUCUGGAAUCUUCUU 3’
           :  ::::::: :::::::
```

```
query=JS16_t2239020, target=FGSG_03148, score=4, range=381-398, strand=1

target  5’ UGGUCAAGAAGUCGUCCG 3’
            :::: :::::::::::
query   3’ UCCAGAUCUUCAGCAGGA 5’

query=JS16_t2239020, target=FGSG_07833, score=4, range=297-315, strand=1

target  5’ AGGUCCAGAAGUCGCUCCA 3’
           ::::: :::::::: :::
query   3’ UCCAGAUCUUCAGC-AGGA 5’

query=JS16_t2239020, target=FGSG_03682, score=4, range=29-47, strand=1

target  5’ AAGUCGAGAAAGUCGUCCU 3’
           : ::: :: ::::::::::
query   3’ UCCAGAUC-UUCAGCAGGA 5’

query=JS16_t2239020, target=FGSG_00551, score=4, range=3053-3070, strand=-1

target  5’ AGGUCUGGCAGUCGUCCA 3’
           ::::::.: ::::::::
query   3’ UCCAGAUCUUCAGCAGGA 5’

query=JS16_t2239020, target=FGSG_05085, score=4, range=1132-1149, strand=-1

target  5’ AGGUGUAGAAGUUGUCUC 3’
           :::: :::::::.:::.
query   3’ UCCAGAUCUUCAGCAGGA 5’

query=JS16_t2239020, target=FGSG_11306, score=4, range=540-557, strand=-1

target  5’ AGGUCCAGAAGUGGUCCU 3’
           ::::: :::::: :::::
query   3’ UCCAGAUCUUCAGCAGGA 5’

query=JS16_t2239020, target=FGSG_02577, score=4, range=6962-6979, strand=-1

target  5’ AGAUCUGGAAGUGGUCCU 3’
           :: :::.::::: :::::
query   3’ UCCAGAUCUUCAGCAGGA 5’

query=JS16_t2239020, target=FGSG_04040, score=4, range=465-482, strand=-1

target  5’ AGUUCUGGAAUUCGUCCU 3’
           :: :::.::: :::::::
query   3’ UCCAGAUCUUCAGCAGGA 5’

query=JS16_t2239020, target=FGSG_02611, score=4, range=1762-1779, strand=-1

target  5’ AGGUGUUGAAGUUGUCCU 3’
           :::: : :::::.:::::
query   3’ UCCAGAUCUUCAGCAGGA 5’

query=JS16_t2239020, target=FGSG_00680, score=4, range=91-108, strand=-1

target  5’ AUGUCUGGAAGUCGGCCU 3’
           : ::::.::::::: :::
query   3’ UCCAGAUCUUCAGCAGGA 5’

query=JS16_t2239020, target=FGSG_07267, score=4, range=1673-1691, strand=-1

target  5’ AGGUCGUCGAGGUCGUCCU 3’
           ::::: : ::.::::::::
query   3’ UCCAG-AUCUUCAGCAGGA 5’

query=JS16_t2239020, target=FGSG_06052, score=4, range=1718-1736, strand=-1

target  5’ AGGGCUGAGAAGUCGUUCU 3’
           ::: :: :::::::::.::
query   3’ UCCAGA-UCUUCAGCAGGA 5’

query=JS16_t2239020, target=FGSG_04187, score=4, range=815-832, strand=-1

target  5’ UGGUGUAGAAGCCGUCCU 3’
            ::: :::::: ::::::
query   3’ UCCAGAUCUUCAGCAGGA 5’

query=JS16_t2239020, target=FGSG_12611, score=4, range=187-204, strand=-1

target  5’ AGGAGUAGAAGUGGUCCU 3’
           :::  ::::::: :::::
query   3’ UCCAGAUCUUCAGCAGGA 5’

query=JS16_t2239020, target=FGSG_09239, score=4, range=545-563, strand=-1

target  5’ AGGUCGAGAGAGUUGUCCU 3’
           ::::: ::: :::.:::::
query   3’ UCCAGAUCU-UCAGCAGGA 5’

query=JS16_t2239020, target=FGSG_11274, score=4, range=83-99, strand=-1

query   3’ UAGCAGACCUAAGAAGAA 5’

query=JS16_t4094876, target=FGSG_00714, score=4, range=919-936, strand=1

target  5’ ACCAUCUUGAUUCUUCUU 3’
           : : ::: ::::::::::
query   3’ UAGCAGACCUAAGAAGAA 5’

query=JS16_t4094876, target=FGSG_07917, score=4, range=1345-1362, strand=1

target  5’ AGCAACUGGGUUCUUCUU 3’
           : :  ::::.::::::::
query   3’ UAGCAGACCUAAGAAGAA 5’

query=JS16_t4094876, target=FGSG_05143, score=4, range=270-286, strand=1

target  5’ ACCCUC-GGAUUCUUCUU 3’
           : : :: ::::::::::
query   3’ UAGCAGACCUAAGAAGAA 5’

query=JS16_t4094876, target=FGSG_00355, score=4, range=982-998, strand=1

target  5’ AUCGGC-GGAUUUUUCUU 3’
           :::: : :::::.:::::
query   3’ UAGCAGACCUAAGAAGAA 5’

query=JS16_t4094876, target=FGSG_10816, score=4, range=994-1010, strand=1

target  5’ AACGUCUGGG-UCUUCUU 3’
           : :::::::. :::::::
query   3’ UAGCAGACCUAAGAAGAA 5’

query=JS16_t4094876, target=FGSG_03266, score=4, range=382-398, strand=1

target  5’ AGCUUCUGG-UUCUUCUU 3’
           : : ::::: ::::::::
query   3’ UAGCAGACCUAAGAAGAA 5’

query=JS16_t4094876, target=FGSG_04388, score=4, range=127-143, strand=1

target  5’ ACCGGCUGG-UUCUUCUU 3’
           : :: :::: ::::::::
query   3’ UAGCAGACCUAAGAAGAA 5’

query=JS16_t4094876, target=FGSG_05769, score=4, range=248-264, strand=1

target  5’ AUGCUCUGGA-UCUUCUU 3’
           ::  :::::: :::::::
query   3’ UAGCAGACCUAAGAAGAA 5’

query=JS16_t4094876, target=FGSG_01469, score=4, range=344-362, strand=1

target  5’ GUCGAUUGGAUGUCUUCUU 3’
           .::: .::::: :::::::
query   3’ UAGCAGACCUA-AGAAGAA 5’

query=JS16_t4094876, target=FGSG_13533, score=4, range=220-237, strand=-1

target  5’ AUCAUUUGGAUUUUUUUU 3’
           ::: :.::::::.::.::
query   3’ UAGCAGACCUAAGAAGAA 5’

query=JS16_t4094876, target=FGSG_04511, score=4, range=552-569, strand=-1

target  5’ AUCGUUCGGAUUCUUCUC 3’
           :::::. ::::::::::
query   3’ UAGCAGACCUAAGAAGAA 5’

query=JS16_t4094876, target=FGSG_07344, score=4, range=1639-1656, strand=-1

target  5’ AUCGUCUGGAAUCUUUUG 3’
           :::::::::: ::::.:
query   3’ UAGCAGACCUAAGAAGAA 5’

query=JS16_t4094876, target=FGSG_00002, score=4, range=1256-1273, strand=-1

target  5’ CAAGUCUGGAUUCUUCUG 3’
              ::::::::::::::
query   3’ UAGCAGACCUAAGAAGAA 5’

query=JS16_t4094876, target=FGSG_05216, score=4, range=5111-5128, strand=-1

target  5’ CUGGUCUGGACUCUUCUU 3’
            : ::::::: :::::::
query   3’ UAGCAGACCUAAGAAGAA 5’

query=JS16_t4094876, target=FGSG_05276, score=4, range=2689-2706, strand=-1

target  5’ CUGGUCGGGAUUCUUCUU 3’
            : ::: :::::::::::
query   3’ UAGCAGACCUAAGAAGAA 5’
```

```
target  5’ AUGGCUAG-AGUCGUCCU 3’
           : : :::: :::::::::
query   3’ UCCAGAUCUUCAGCAGGA 5’

query=JS16_t2239020, target=FGSG_12981, score=4, range=275-291, strand=-1

target  5’ AAGACUAGAAGUC-UCCU 3’
           : : ::::::::: ::::
query   3’ UCCAGAUCUUCAGCAGGA 5’

No results for JS16_t1342493
query=JS16_t0134028, target=FGSG_11279, score=2.5, range=676-694, strand=-1

target  5’ CGCUUCCACGAUCUCGUGG 3’
           ::::. :::::::::.:::
query   3’ GCGAGUGUGCUAGAGUACC 5’

query=JS16_t0134028, target=FGSG_05625, score=3, range=329-346, strand=1

target  5’ AGCUC-CACGAUCUCAUGA 3’
            :::: ::::::::::::
query   3’ GCGAGUGUGCUAGAGUACC 5’

query=JS16_t0134028, target=FGSG_07594, score=3.5, range=1059-1077, strand=1

target  5’ CGCUGGCAUGAUUUCAUGG 3’
           :::: .::.:::.::::::
query   3’ GCGAGUGUGCUAGAGUACC 5’

query=JS16_t0134028, target=FGSG_09642, score=3.5, range=517-534, strand=1

target  5’ CGCUU-CACGAUCUCAUCG 3’
           ::::. ::::::::::: :
query   3’ GCGAGUGUGCUAGAGUACC 5’

query=JS16_t0134028, target=FGSG_07537, score=3.5, range=929-947, strand=-1

target  5’ CGCUCGCGCGAUCCCAUGG 3’
           :::::.:.::::: :::::
query   3’ GCGAGUGUGCUAGAGUACC 5’

query=JS16_t0134028, target=FGSG_13239, score=4, range=782-800, strand=1

target  5’ CGCUGACGCGAGCUCAUGG 3’
           :::: ::.::: :::::::
query   3’ GCGAGUGUGCUAGAGUACC 5’

query=JS16_t0134028, target=FGSG_08521, score=4, range=926-944, strand=1

target  5’ CCCUUGCACGAUCUCUUGG 3’
           : ::..::::::::: :::
query   3’ GCGAGUGUGCUAGAGUACC 5’

query=JS16_t0134028, target=FGSG_05736, score=4, range=5182-5200, strand=1

target  5’ UGCGUUCGCGAUCUCAUGG 3’
           .:: . :.:::::::::::
query   3’ GCGAGUGUGCUAGAGUACC 5’

query=JS16_t0134028, target=FGSG_11101, score=4, range=1104-1123, strand=1

target  5’ CACCCACAUCGAUCUCAUGG 3’
           : : :::: :::::::::::
query   3’ GCGAGUGU-GCUAGAGUACC 5’

query=JS16_t0134028, target=FGSG_02880, score=4, range=963-981, strand=-1

target  5’ CGCUAACAUGAUCUCAUUG 3’
           :::: :::.:::::::: :
query   3’ GCGAGUGUGCUAGAGUACC 5’

query=JS16_t0134028, target=FGSG_13732, score=4, range=222-240, strand=-1

target  5’ AGCCCACGCGAUCUCAUGA 3’
            :: :::.::::::::::
query   3’ GCGAGUGUGCUAGAGUACC 5’

query=JS16_t0134028, target=FGSG_07240, score=4, range=1373-1390, strand=-1

target  5’ CGCUCACACGAUC-GAUGG 3’
           :::::::::::::  ::::
query   3’ GCGAGUGUGCUAGAGUACC 5’

query=JS16_t0134028, target=FGSG_09904, score=4, range=587-604, strand=-1

target  5’ CGAUGACACGAUCUC-UGG 3’
           :: : :::::::::: :::
query   3’ GCGAGUGUGCUAGAGUACC 5’

query=JS16_t0134028, target=FGSG_06379, score=4, range=3102-3119, strand=-1

target  5’ GGCUC-CACAAUCUCAUGG 3’
```

```
query=JS16_t4094876, target=FGSG_02150, score=4, range=124-141, strand=-1

target  5’ AUCUUCGGGAUUCUUCUG 3’
           ::: :: ::::::::::
query   3’ UAGCAGACCUAAGAAGAA 5’

query=JS16_t4094876, target=FGSG_11043, score=4, range=709-725, strand=-1

target  5’ GUUG-CUGGGUUCUUCUG 3’
           .:.: ::::.:::::::
query   3’ UAGCAGACCUAAGAAGAA 5’

query=JS16_t4094876, target=FGSG_03893, score=4, range=1373-1391, strand=-1

target  5’ AUCUUCUGGAUUCUGUCUG 3’
           ::: :::::::::: :::
query   3’ UAGCAGACCUAAGA-AGAA 5’

query=JS16_t4094876, target=FGSG_13883, score=4, range=1126-1142, strand=-1

target  5’ AUGGUCUGG-UUUUUCUU 3’
           :: :::::: ::.:::::
query   3’ UAGCAGACCUAAGAAGAA 5’

query=JS16_t4094876, target=FGSG_09878, score=4, range=709-725, strand=-1

target  5’ AUAGUCUGGA-UCUUUUU 3’
           :: ::::::: ::::.::
query   3’ UAGCAGACCUAAGAAGAA 5’

query=JS16_t4094876, target=FGSG_13065, score=4, range=460-477, strand=-1

target  5’ AUCCGCUGCAUUCUUCUU 3’
           :::  ::: :::::::::
query   3’ UAGCAGACCUAAGAAGAA 5’

query=JS16_t4094876, target=FGSG_04030, score=4, range=5840-5857, strand=-1

target  5’ CUCCGCUGGAUUCUUCUG 3’
            ::  ::::::::::::
query   3’ UAGCAGACCUAAGAAGAA 5’

query=JS16_t4094876, target=FGSG_05627, score=4, range=524-540, strand=-1

target  5’ CUCCUC-GGAUUCUUCUU 3’
            :: :: :::::::::::
query   3’ UAGCAGACCUAAGAAGAA 5’

query=JS16_t4094876, target=FGSG_01135, score=4, range=415-431, strand=-1

target  5’ UUCUUCUGGA-UCUUCUU 3’
            :: :::::: :::::::
query   3’ UAGCAGACCUAAGAAGAA 5’

query=JS16_t4094876, target=FGSG_12474, score=4, range=683-699, strand=-1

target  5’ AUAGU-UGGAUUCUUCUA 3’
           :: :: :::::::::::
query   3’ UAGCAGACCUAAGAAGAA 5’

query=JS16_t4094876, target=FGSG_00846, score=4, range=1814-1830, strand=-1

target  5’ AGCG-CUGGAUCCUUCUU 3’
           : :: :::::: ::::::
query   3’ UAGCAGACCUAAGAAGAA 5’

query=JS16_t4094876, target=FGSG_08068, score=4, range=164-180, strand=-1

target  5’ AUC-ACUGGCUUCUUCUU 3’
           :::  :::: ::::::::
query   3’ UAGCAGACCUAAGAAGAA 5’

No results for JS16_t3198129
query=JS16_t0803562, target=FGSG_05403, score=3, range=888-906, strand=1

target  5’ GACACCCGAGACGAGUGGC 3’
           ::::  ::::::::::::
query   3’ CUGUACGCUCUGCUCACCA 5’

query=JS16_t0803562, target=FGSG_12275, score=3.5, range=1189-1207, strand=1

target  5’ GCCGUGCGAGGCGAGUGGG 3’
           : :.::::::.:::::::
query   3’ CUGUACGCUCUGCUCACCA 5’

query=JS16_t0803562, target=FGSG_00294, score=3.5, range=991-1010, strand=1

target  5’ GACGUUCGAGACGAGAUGGU 3’
           :::.: ::::::::: ::::
query   3’ CUGUACGCUCUGCUC-ACCA 5’

query=JS16_t0803562, target=FGSG_05370, score=3.5, range=811-828, strand=1
```

```
             :::: ::: :::::::::
query   3' GCGAGUGUGCUAGAGUACC 5'

No results for JS16_t3135337
query=JS16_t0188527, target=FGSG_11425, score=3, range=2282-2301, strand=-1

target  5' CAAUCGCUCUUCAAACUUAC 3'
            :::: ::::::::::::: :
query   3' GUUACCGAGAAGUUUGAAAG 5'

query=JS16_t0188527, target=FGSG_09014, score=3.5, range=881-900, strand=1

target  5' UGGUGGCUCUUCAAGCUUUG 3'
            ...:::::::::::.::::
query   3' GUUACCGAGAAGUUUGAAAG 5'

query=JS16_t0188527, target=FGSG_05660, score=3.5, range=1965-1983, strand=1

target  5' CAAU-GUUCUUCAAACCUUC 3'
            :::: :.::::::::: :::
query   3' GUUACCGAGAAGUUUGAAAG 5'

query=JS16_t0188527, target=FGSG_09725, score=3.5, range=2174-2194, strand=-1

target  5' CAAUCUCUUCUUCAAACUUUU 3'
            ::::  : ::::::::::::.
query   3' GUUACCG-AGAAGUUUGAAAG 5'

query=JS16_t0188527, target=FGSG_07889, score=4, range=60-79, strand=1

target  5' UCUUGGUUCUUCAGACUUUC 3'
            .  :::.::::::.::::::
query   3' GUUACCGAGAAGUUUGAAAG 5'

query=JS16_t0188527, target=FGSG_12381, score=4, range=548-567, strand=-1

target  5' CAAUGUCUCUUCACACUUUG 3'
            ::::: ::::::: :::::
query   3' GUUACCGAGAAGUUUGAAAG 5'

query=JS16_t0188527, target=FGSG_08710, score=4, range=737-757, strand=-1

target  5' CAAAGGUCUCUUCAAACUUGC 3'
            ::: :: :::::::::::: :
query   3' GUUACC-GAGAAGUUUGAAAG 5'

query=JS16_t0188527, target=FGSG_01790, score=4, range=4423-4442, strand=-1

target  5' CGAACAUUCUUCAAACUUUC 3'
            :.:   .:::::::::::::
query   3' GUUACCGAGAAGUUUGAAAG 5'

query=JS16_t2239297, target=FGSG_10613, score=3.5, range=535-552, strand=1

target  5' CGACAUACAUAUUUUGCG 3'
            :: ::::::: ::::::.
query   3' GCCGUAUGUACAAAACGU 5'

query=JS16_t2239297, target=FGSG_08350, score=3.5, range=242-259, strand=-1

target  5' CGGAGUACUUGUUUUGCA 3'
            ::: .::: :::::::::
query   3' GCCGUAUGUACAAAACGU 5'

query=JS16_t2239297, target=FGSG_06911, score=4, range=858-876, strand=1

target  5' CGGAAUGCAAUGUUUUGCA 3'
            ::: ::.: ::::::::::
query   3' GCCGUAUG-UACAAAACGU 5'

query=JS16_t2239297, target=FGSG_05092, score=4, range=2474-2491, strand=-1

target  5' CGGCUUGCAUGUUCUGCA 3'
            :::: :.:::::: ::::
query   3' GCCGUAUGUACAAAACGU 5'

query=JS16_t2239297, target=FGSG_06312, score=4, range=1901-1918, strand=-1

target  5' CGCCAAACAUGUUUUGCC 3'
            :: :: :::::::::::
query   3' GCCGUAUGUACAAAACGU 5'

query=JS16_t2239297, target=FGSG_03373, score=4, range=131-148, strand=-1

target  5' AGACCUGCAUGUUUUGCA 3'
             : : :.:::::::::::
query   3' GCCGUAUGUACAAAACGU 5'

query=JS16_t2239297, target=FGSG_09180, score=4, range=1188-1204, strand=-1

target  5' CGG-AGAUAUGUUUUGCA 3'
            ::: : :.::::::::::
```

```
target  5' CUCG-GCGAGACGAGUGGU 3'
              :. ::::::::::::::
query   3' CUGUACGCUCUGCUCACCA 5'

query=JS16_t0803562, target=FGSG_00180, score=3.5, range=765-782, strand=-1

target  5' GAUAU-CGAGGCGGGUGGU 3'
            ::.:: ::::.::.:::::
query   3' CUGUACGCUCUGCUCACCA 5'

query=JS16_t0803562, target=FGSG_09178, score=3.5, range=323-341, strand=-1

target  5' GAGGCGCGAGACGGGUGGU 3'
            :: . ::::::::.:::::
query   3' CUGUACGCUCUGCUCACCA 5'

query=JS16_t0803562, target=FGSG_12287, score=3.5, range=68-85, strand=-1

target  5' CAUAUG-GAGACGAGUGGU 3'
             :.::: ::::::::::::
query   3' CUGUACGCUCUGCUCACCA 5'

query=JS16_t0803562, target=FGSG_08309, score=3.5, range=2653-2670, strand=-1

target  5' GACGU-CGAGAAGAGUGGU 3'
            :::.: ::::: :::::::
query   3' CUGUACGCUCUGCUCACCA 5'

query=JS16_t0803562, target=FGSG_13596, score=4, range=814-832, strand=1

target  5' GACAUGCGACAUGAGUGGG 3'
            ::::::::: :.::::::
query   3' CUGUACGCUCUGCUCACCA 5'

query=JS16_t0803562, target=FGSG_07198, score=4, range=533-550, strand=1

target  5' GAAAU-CGAGACGAGUUGU 3'
            :: :: :::::::::: ::
query   3' CUGUACGCUCUGCUCACCA 5'

query=JS16_t0803562, target=FGSG_13887, score=4, range=801-818, strand=1

target  5' GAUGCGCGAGACGA-UGGU 3'
            ::.. ::::::::: ::::
query   3' CUGUACGCUCUGCUCACCA 5'

query=JS16_t0803562, target=FGSG_10192, score=4, range=156-174, strand=-1

target  5' AUUGUGUGAGACGAGUGGU 3'
              ..::.:::::::::::
query   3' CUGUACGCUCUGCUCACCA 5'

query=JS16_t0803562, target=FGSG_05523, score=4, range=1187-1205, strand=-1

target  5' GACAAGCGAGACAAGUGGC 3'
            :::: ::::::: :::::
query   3' CUGUACGCUCUGCUCACCA 5'

query=JS16_t0803562, target=FGSG_07389, score=4, range=841-858, strand=-1

target  5' AACCUG-GAGACGAGUGGU 3'
             :: :: ::::::::::::
query   3' CUGUACGCUCUGCUCACCA 5'

query=JS16_t0803562, target=FGSG_00551, score=4, range=4305-4322, strand=-1

target  5' GGGGUGCGAGACGA-UGGU 3'
            :. .:::::::::: ::::
query   3' CUGUACGCUCUGCUCACCA 5'

query=JS16_t0803562, target=FGSG_01655, score=4, range=61-80, strand=-1

target  5' GACAUGCGAGCGCGGGUGGU 3'
            :::::::::: .::.:::::
query   3' CUGUACGCUC-UGCUCACCA 5'

query=JS16_t0188944, target=FGSG_03601, score=2.5, range=1332-1348, strand=-1

target  5' UAU-UACCGGAUCCAGGU 3'
            .:: :::::::::::::
query   3' GUACAUGGCCUAGGUCCU 5'

query=JS16_t0188944, target=FGSG_08042, score=3, range=1711-1728, strand=1

target  5' CAGGUACCUGAUCCAGGA 3'
            :: ::::: :::::::::
query   3' GUACAUGGCCUAGGUCCU 5'

query=JS16_t0188944, target=FGSG_07069, score=3, range=157-174, strand=-1

target  5' CAUGUACUGGGUCCAGGC 3'
```

```
query   3' GCCGUAUGUACAAAACGU 5'

query=JS16_t2239297, target=FGSG_12148, score=4, range=175-191, strand=-1

target  5' CAGCA-ACGUGUUUUGCA 3'
           : ::: ::.:::::::::
query   3' GCCGUAUGUACAAAACGU 5'

query=JS16_t1342608, target=FGSG_05819, score=3, range=922-940, strand=-1

target  5' UGGUAGAGUACACGUCUAG 3'
           :.:: .::::::::::.::
query   3' AUCACUUCAUGUGCAGGUC 5'

query=JS16_t1342608, target=FGSG_12236, score=3.5, range=512-529, strand=-1

target  5' UGGU-AAGUACACCUCCAG 3'
           :.:: :::::::: :::::
query   3' AUCACUUCAUGUGCAGGUC 5'

query=JS16_t1342608, target=FGSG_00942, score=4, range=260-278, strand=1

target  5' UAGUUAACUACACGUCCAA 3'
           :::: :: ::::::::::
query   3' AUCACUUCAUGUGCAGGUC 5'

query=JS16_t1342608, target=FGSG_10133, score=4, range=59-77, strand=1

target  5' UUGAGAAGUACAUGUCCAA 3'
           : : ::::::::.:::::
query   3' AUCACUUCAUGUGCAGGUC 5'

query=JS16_t1342608, target=FGSG_11162, score=4, range=722-741, strand=-1

target  5' UAGUUGAGGUGCAUGUCCAG 3'
           ::: :::.::.::.::::::
query   3' AUC-ACUUCAUGUGCAGGUC 5'

query=JS16_t1342608, target=FGSG_07295, score=4, range=832-850, strand=-1

target  5' UACUCCAGUACACGUCCAU 3'
           :: :  ::::::::::::
query   3' AUCACUUCAUGUGCAGGUC 5'

query=JS16_t1342608, target=FGSG_13824, score=4, range=1977-1994, strand=-1

target  5' UAUUG-UGUACACGUCCAG 3'
           :: ::  ::::::::::::
query   3' AUCACUUCAUGUGCAGGUC 5'

query=JS16_t0305906, target=FGSG_03340, score=4, range=7398-7416, strand=1

target  5' UGUCAA-GGCCUUCCGAAAC 3'
            :::::  ::::::::::::
query   3' CCAGUUAGCGGAAGGCUUUG 5'

No results for JS16_t0686475
query=JS16_t3135476, target=FGSG_09589, score=4, range=1324-1345, strand=1

target  5' ACAUCGAACCGAAACUUUGAGG 3'
           :: : ::: :::::::::.:::
query   3' UGGAUCUU-GCUUUGAAAUUCC 5'

query=JS16_t1342764, target=FGSG_03788, score=4, range=628-648, strand=1

target  5' GUCGACGGUGUCAAGAUGUGG 3'
           .::: :.  :.:::::::::::
query   3' UAGCGGUGAUAGUUCUACACC 5'

query=JS16_t1342764, target=FGSG_05821, score=4, range=952-972, strand=1

target  5' AGCGCCACUAUCAAGAUGCGC 3'
           : :::::::::::::::: :
query   3' UAGCGGUGAUAGUUCUACACC 5'

query=JS16_t0240444, target=FGSG_09921, score=2, range=759-776, strand=1

target  5' ACUCUACCUUGUUUUCAG 3'
           ::.: ::::::::::::.
query   3' UGGGUUGGAACAAAAGUU 5'

query=JS16_t0240444, target=FGSG_00034, score=2.5, range=1526-1544, strand=-1

target  5' GCCGACACCUUGUUUUCAA 3'
           .:: : :::::::::::::
query   3' UGGGU-UGGAACAAAAGUU 5'

query=JS16_t0240444, target=FGSG_05328, score=3, range=1139-1156, strand=-1

target  5' UCUCGAUCUUGUUUUCAA 3'
            :.:.:.:::::::::::
query   3' UGGGUUGGAACAAAAGUU 5'

           :::::::.::.::::::
query   3' GUACAUGGCCUAGGUCCU 5'

query=JS16_t0188944, target=FGSG_09378, score=3, range=171-189, strand=-1

target  5' CAUGAUACUGGGUCCAGGA 3'
           :::: :::.::.:::::::
query   3' GUAC-AUGGCCUAGGUCCU 5'

query=JS16_t0188944, target=FGSG_02036, score=3, range=75-91, strand=-1

target  5' CA-GUACUGGAUCUAGGA 3'
           :: ::::.:::::.::::
query   3' GUACAUGGCCUAGGUCCU 5'

query=JS16_t0188944, target=FGSG_00375, score=3.5, range=340-356, strand=1

target  5' CGUG-AUCGGGUCCAGGA 3'
           :.:: :.:::.:::::::
query   3' GUACAUGGCCUAGGUCCU 5'

query=JS16_t0188944, target=FGSG_13878, score=3.5, range=26641-26658, strand=-[illegible]

target  5' GAUGUACCGGAUGCAGGG 3'
            ::::::::::: ::::.
query   3' GUACAUGGCCUAGGUCCU 5'

query=JS16_t0188944, target=FGSG_07167, score=3.5, range=196-213, strand=-1

target  5' AACGUGCCGGAUCCAGGG 3'
            : ::.:::::::::::.
query   3' GUACAUGGCCUAGGUCCU 5'

query=JS16_t0188944, target=FGSG_02668, score=3.5, range=1761-1779, strand=-1

target  5' CAUGAUACCGGAUCCUGGG 3'
           :::: :::::::::: ::.
query   3' GUAC-AUGGCCUAGGUCCU 5'

query=JS16_t0188944, target=FGSG_01468, score=3.5, range=489-505, strand=-1

target  5' CGU-UACCGGAACCAGGA 3'
           :.: ::::::: ::::::
query   3' GUACAUGGCCUAGGUCCU 5'

query=JS16_t0188944, target=FGSG_02521, score=3.5, range=696-712, strand=-1

target  5' UAAG-ACUGGAUCCAGGA 3'
           .: : ::.::::::::::
query   3' GUACAUGGCCUAGGUCCU 5'

query=JS16_t0188944, target=FGSG_09795, score=4, range=856-873, strand=1

target  5' CAUGUUCCGGGUCUAGGA 3'
           ::::: ::::.::.::::
query   3' GUACAUGGCCUAGGUCCU 5'

query=JS16_t0188944, target=FGSG_06635, score=4, range=1185-1202, strand=1

target  5' CGUGUACUGGAUCCUGGG 3'
           :.:::::.:::::: ::.
query   3' GUACAUGGCCUAGGUCCU 5'

query=JS16_t0188944, target=FGSG_01196, score=4, range=1697-1714, strand=1

target  5' CAUUUAUCGGAUCCAAGA 3'
           ::: ::.::::::::: ::
query   3' GUACAUGGCCUAGGUCCU 5'

query=JS16_t0188944, target=FGSG_03136, score=4, range=480-497, strand=1

target  5' CAUUCACUGGAUUCAGGA 3'
           :::  ::.::::.:::::
query   3' GUACAUGGCCUAGGUCCU 5'

query=JS16_t0188944, target=FGSG_11762, score=4, range=38-54, strand=1

target  5' CAUGUACC-GAUCCCGGA 3'
           :::::::: ::::: :::
query   3' GUACAUGGCCUAGGUCCU 5'

query=JS16_t0188944, target=FGSG_00408, score=4, range=3370-3386, strand=1

target  5' CAUG-GCCGGAUCGAGGA 3'
           :::: .::::::: ::::
query   3' GUACAUGGCCUAGGUCCU 5'

query=JS16_t0188944, target=FGSG_01758, score=4, range=1240-1256, strand=1

target  5' CAUG-ACUGGAACCAGGA 3'
           :::: ::.::: ::::::
query   3' GUACAUGGCCUAGGUCCU 5'
```

```
query=JS16_t0240444, target=FGSG_12768, score=3, range=763-780, strand=-1

target  5’ AUCGAACUUUGUUUUCAG 3’
           :.: :::.:::::::::.
query   3’ UGGGUUGGAACAAAAGUU 5’

query=JS16_t0240444, target=FGSG_01308, score=3.5, range=606-623, strand=1

target  5’ GUUCAACCUUGUUUUUAU 3’
           ...:::::::::::::.:
query   3’ UGGGUUGGAACAAAAGUU 5’

query=JS16_t0240444, target=FGSG_05023, score=3.5, range=327-344, strand=1

target  5’ CUCCAUCCUUGUUUUCAA 3’
            .::: ::::::::::::
query   3’ UGGGUUGGAACAAAAGUU 5’

query=JS16_t0240444, target=FGSG_05114, score=3.5, range=577-593, strand=1

target  5’ GCCC-ACCUUGUUGUCAA 3’
           .::: :::::::: ::::
query   3’ UGGGUUGGAACAAAAGUU 5’

query=JS16_t0240444, target=FGSG_08581, score=3.5, range=392-409, strand=-1

target  5’ CCUCAACCUUGUUUUUAC 3’
            :.:::::::::::::.:
query   3’ UGGGUUGGAACAAAAGUU 5’

query=JS16_t0240444, target=FGSG_10853, score=3.5, range=1349-1366, strand=-1

target  5’ CCUCAACCUUGUUCUCAA 3’
            :.::::::::::: ::::
query   3’ UGGGUUGGAACAAAAGUU 5’

query=JS16_t0240444, target=FGSG_05855, score=3.5, range=2278-2296, strand=-1

target  5’ UCUCAACCUUGAUUUUCAA 3’
            :.:::::::: :::::::
query   3’ UGGGUUGGAAC-AAAAGUU 5’

query=JS16_t0240444, target=FGSG_07469, score=3.5, range=2819-2836, strand=-1

target  5’ CAACGACCUUGUUUUCAA 3’
              :.:::::::::::::
query   3’ UGGGUUGGAACAAAAGUU 5’

query=JS16_t0240444, target=FGSG_01865, score=3.5, range=1517-1533, strand=-1

target  5’ ACCC-ACCUUGUUUGCAG 3’
           :::: ::::::::: ::.
query   3’ UGGGUUGGAACAAAAGUU 5’

query=JS16_t0240444, target=FGSG_02680, score=4, range=645-662, strand=1

target  5’ CUUCAGCUUUGUUUUCAA 3’
            ..::.:.::::::::::
query   3’ UGGGUUGGAACAAAAGUU 5’

query=JS16_t0240444, target=FGSG_05093, score=4, range=303-320, strand=1

target  5’ GCUUGACCUUGUCUUCAA 3’
           .:...::::::: :::::
query   3’ UGGGUUGGAACAAAAGUU 5’

query=JS16_t0240444, target=FGSG_07687, score=4, range=1968-1986, strand=1

target  5’ ACUCGGAUCUUGUUUUCAU 3’
           ::.: .:.::::::::::
query   3’ UGGG-UUGGAACAAAAGUU 5’

query=JS16_t0240444, target=FGSG_07093, score=4, range=500-517, strand=1

target  5’ AGACAGUCUUGUUUUCAA 3’
           :  ::..:::::::::::
query   3’ UGGGUUGGAACAAAAGUU 5’

query=JS16_t0240444, target=FGSG_05168, score=4, range=821-839, strand=1

target  5’ ACCACAACAUUGUUUUCGA 3’
           ::: :::: ::::::::.:
query   3’ UGG-GUUGGAACAAAAGUU 5’

query=JS16_t0240444, target=FGSG_12918, score=4, range=1173-1190, strand=1

target  5’ AUUCCACCUUGUUUCCAA 3’
           :..: ::::::::: :::
query   3’ UGGGUUGGAACAAAAGUU 5’

query=JS16_t0240444, target=FGSG_06410, score=4, range=558-575, strand=1
```

```
query=JS16_t0188944, target=FGSG_03616, score=4, range=3320-3336, strand=1

target  5’ CAU-CACCGGAUCCAGCA 3’
           :::  ::::::::::: :
query   3’ GUACAUGGCCUAGGUCCU 5’

query=JS16_t0188944, target=FGSG_06304, score=4, range=765-783, strand=1

target  5’ CAUGGCCCGGAGUCCAGGA 3’
           ::::  ::::: :::::::
query   3’ GUACAUGGCCU-AGGUCCU 5’

query=JS16_t0188944, target=FGSG_12768, score=4, range=97-113, strand=1

target  5’ CACGAACCGG-UCCAGGA 3’
           :: : ::::: :::::::
query   3’ GUACAUGGCCUAGGUCCU 5’

query=JS16_t0188944, target=FgHV1gp2, score=4, range=3657-3674, strand=-1

target  5’ UAUGCACCGGAUUUAGGG 3’
           .::: :::::::..:::.
query   3’ GUACAUGGCCUAGGUCCU 5’

query=JS16_t0188944, target=FGSG_06429, score=4, range=684-701, strand=-1

target  5’ CCUGUACCGGAUUCGGGU 3’
           : ::::::::::.:.::
query   3’ GUACAUGGCCUAGGUCCU 5’

query=JS16_t0188944, target=FGSG_03323, score=4, range=619-636, strand=-1

target  5’ CAUGUACAGGAUCAAGGA 3’
           ::::::: ::::: ::::
query   3’ GUACAUGGCCUAGGUCCU 5’

query=JS16_t0188944, target=FGSG_02902, score=4, range=1447-1465, strand=-1

target  5’ CAUUGUCCCGGAUCCAGGU 3’
           :: ::: :::::::::::
query   3’ GU-ACAUGGCCUAGGUCCU 5’

query=JS16_t0188944, target=FGSG_02276, score=4, range=17-33, strand=-1

target  5’ AAUG-ACCGGUUCCAGGA 3’
            ::: ::::: :::::::
query   3’ GUACAUGGCCUAGGUCCU 5’

query=JS16_t0188944, target=FGSG_08398, score=4, range=444-460, strand=-1

target  5’ CAAG-ACUGGAUCCAGGU 3’
           :: : ::.:::::::::
query   3’ GUACAUGGCCUAGGUCCU 5’

query=JS16_t0188944, target=FGSG_10811, score=4, range=1035-1051, strand=-1

target  5’ CAAG-ACCGGAUGCAGGA 3’
           :: : ::::::: :::::
query   3’ GUACAUGGCCUAGGUCCU 5’

query=JS16_t1405910, target=FGSG_09368, score=4, range=75-94, strand=1

target  5’ UGG-GAGGACAUAUUCGCGUG 3’
           ::. :::.:::::.:::::.:
query   3’ ACUGCUCUUGUAUGAGCGCGC 5’

query=JS16_t1405910, target=FGSG_09756, score=4, range=295-315, strand=-1

target  5’ AGGCGAUGACAUGCUCGCGCG 3’
            :.::: .::::.::::::::
query   3’ ACUGCUCUUGUAUGAGCGCGC 5’

No results for JS16_t1405951
No results for JS16_t0803573
query=JS16_t3198227, target=FGSG_11716, score=3, range=194-213, strand=-1

target  5’ CCUUUCAAUCUCCUGUUGAA 3’
           ::  : ::::::::::::::
query   3’ GGCCAUUUAGAGGACAACUU 5’

query=JS16_t3198227, target=FGSG_09294, score=3.5, range=1081-1101, strand=1

target  5’ AUGGCUAAAUCUCUUGUUGAA 3’
            .:: ::::::::.:::::::
query   3’ GGCC-AUUUAGAGGACAACUU 5’

query=JS16_t3198227, target=FGSG_04322, score=4, range=279-298, strand=-1

target  5’ UUGGUAAAUUUCUGUUGAC 3’
           ..:::::::.:.:::::::
query   3’ GGCCAUUUAGAGGACAACUU 5’
```

target 5’ ACAUGACCUUGUGUUCAA 3’
:: ..::::::: :::::
query 3’ UGGGUUGGAACAAAAGUU 5’

query=JS16_t0240444, target=FGSG_02252, score=4, range=1503-1521, strand=1

target 5’ GCACAACCUUGCUUUUCAG 3’
.: :::::::: ::::::.
query 3’ UGGGUUGGAAC-AAAAGUU 5’

query=JS16_t0240444, target=FGSG_04275, score=4, range=47-65, strand=1

target 5’ GUCGAUACCUUGUUUUCGA 3’
..: : :::::::::::.:
query 3’ UGGGU-UGGAACAAAAGUU 5’

query=JS16_t0240444, target=FGSG_13776, score=4, range=749-766, strand=1

target 5’ ACCGAACCUUGUCUUCAU 3’
::: :::::::: ::::
query 3’ UGGGUUGGAACAAAAGUU 5’

query=JS16_t0240444, target=FGSG_06980, score=4, range=100-116, strand=1

target 5’ GUACAA-CUUGUUUUCAA 3’
.. ::: ::::::::::
query 3’ UGGGUUGGAACAAAAGUU 5’

query=JS16_t0240444, target=FGSG_04350, score=4, range=761-779, strand=1

target 5’ ACACACCCUUCGUUUUCAA 3’
:: :: :::: ::::::::
query 3’ UGGGUUGGAA-CAAAAGUU 5’

query=JS16_t0240444, target=FGSG_11003, score=4, range=662-679, strand=-1

target 5’ CCCCAACUUUGUUUUCGU 3’
::::::.::::::::.
query 3’ UGGGUUGGAACAAAAGUU 5’

query=JS16_t0240444, target=FGSG_07466, score=4, range=2660-2677, strand=-1

target 5’ GCUUGAGCUUGUUUUCAA 3’
.:...: ::::::::::
query 3’ UGGGUUGGAACAAAAGUU 5’

query=JS16_t0240444, target=FGSG_02039, score=4, range=1757-1774, strand=-1

target 5’ GUCCAAACUUGUUUUCAC 3’
..:::: :::::::::
query 3’ UGGGUUGGAACAAAAGUU 5’

query=JS16_t0240444, target=FGSG_01328, score=4, range=415-432, strand=-1

target 5’ AUUGAACUUUGUUUUCAC 3’
:.. :::.:::::::::
query 3’ UGGGUUGGAACAAAAGUU 5’

query=JS16_t0240444, target=FGSG_03742, score=4, range=1319-1336, strand=-1

target 5’ GCAUAACCUUGCUUUCAA 3’
.: .::::::: ::::::
query 3’ UGGGUUGGAACAAAAGUU 5’

query=JS16_t0240444, target=FGSG_08936, score=4, range=524-541, strand=-1

target 5’ GCCAAACCUUCUUUUCAG 3’
.:: :::::: ::::::.
query 3’ UGGGUUGGAACAAAAGUU 5’

query=JS16_t0240444, target=FGSG_03352, score=4, range=682-699, strand=-1

target 5’ ACGCAUCCUUGUUUUCAC 3’
:: :: ::::::::::
query 3’ UGGGUUGGAACAAAAGUU 5’

query=JS16_t0240444, target=FGSG_01410, score=4, range=1379-1396, strand=-1

target 5’ AAACAACCUUGUUAUCAA 3’
: :::::::::: ::::
query 3’ UGGGUUGGAACAAAAGUU 5’

query=JS16_t0240444, target=FGSG_09075, score=4, range=1115-1131, strand=-1

target 5’ AUCU-ACCUUGUCUUCAA 3’
:.:. ::::::: :::::
query 3’ UGGGUUGGAACAAAAGUU 5’

query=JS16_t0240444, target=FGSG_05497, score=4, range=172-188, strand=-1

target 5’ UCCGAACCUUG-UUUCAA 3’

query=JS16_t3198227, target=FGSG_00348, score=4, range=313-333, strand=-1

target 5’ CUGGUAGAUCUUCUUGUUGAA 3’
:.::::.::: ::.:::::::
query 3’ GGCCAUUUAG-AGGACAACUU 5’

query=JS16_t3198227, target=FGSG_09358, score=4, range=875-894, strand=-1

target 5’ CUGGCGAAUCUCCUGUUCAA 3’
:.:: .:::::::::: ::
query 3’ GGCCAUUUAGAGGACAACUU 5’

query=JS16_t3198227, target=FGSG_04519, score=4, range=1008-1027, strand=-1

target 5’ CUGUGAAGUCUCCUGUUGAG 3’
:.: ::.:::::::::::.
query 3’ GGCCAUUUAGAGGACAACUU 5’

query=JS16_t0396639, target=FGSG_02883, score=3.5, range=400-419, strand=1

target 5’ AACGGGUACCAGUAUGUUGU 3’
: ::.:::::::::..::::
query 3’ UCGCUCAUGGUCAUGUAACA 5’

query=JS16_t0396639, target=FGSG_06549, score=3.5, range=837-857, strand=1

target 5’ GGCCAGCAACCAGUACAUUGU 3’
.:: :: :::::::::::::
query 3’ UCGCUC-AUGGUCAUGUAACA 5’

query=JS16_t0396639, target=FGSG_07076, score=4, range=2807-2826, strand=-1

target 5’ GGUGAGUACCAGUGCGUUGA 3’
.:.::::::::::.:.:::
query 3’ UCGCUCAUGGUCAUGUAACA 5’

query=JS16_t0396639, target=FGSG_09595, score=4, range=1362-1380, strand=-1

target 5’ AGCGAG-ACCAGUGCUUUGU 3’
:::::: ::::::.: ::::
query 3’ UCGCUCAUGGUCAUGUAACA 5’

query=JS16_t0476450, target=FGSG_04351, score=2, range=2272-2289, strand=1

target 5’ ACCACGGAGAAGCCUGCU 3’
::: ::.::::::::::
query 3’ UGGAGCUUCUUCGGACGA 5’

query=JS16_t0476450, target=FGSG_02180, score=2.5, range=1397-1414, strand=1

target 5’ ACCUUGAAGAAGCUUGCC 3’
::::.::::::::.:::
query 3’ UGGAGCUUCUUCGGACGA 5’

query=JS16_t0476450, target=FGSG_00339, score=2.5, range=385-402, strand=1

target 5’ UCUUCGAAGAAGCCUGCC 3’
:.::::::::::::::
query 3’ UGGAGCUUCUUCGGACGA 5’

query=JS16_t0476450, target=FGSG_10862, score=3, range=677-694, strand=1

target 5’ UCCUCGAACAAGCCUGCU 3’
::::::: :::::::::
query 3’ UGGAGCUUCUUCGGACGA 5’

query=JS16_t0476450, target=FGSG_06113, score=3, range=235-251, strand=1

target 5’ GCCUUGAAGAAGCCU-CU 3’
.:::.:::::::::: ::
query 3’ UGGAGCUUCUUCGGACGA 5’

query=JS16_t0476450, target=FGSG_04990, score=3, range=2845-2862, strand=1

target 5’ ACGUCGAAGAAGACUGCU 3’
:: ::::::::: :::::
query 3’ UGGAGCUUCUUCGGACGA 5’

query=JS16_t0476450, target=FGSG_04113, score=3, range=1937-1955, strand=1

target 5’ ACCUCAAAGAAGCCAUGCU 3’
::::: :::::::: ::::
query 3’ UGGAGCUUCUUCGG-ACGA 5’

query=JS16_t0476450, target=FGSG_00857, score=3, range=145-162, strand=1

target 5’ ACCGAGGAGAAGCCUGCU 3’
::: :.:::::::::::
query 3’ UGGAGCUUCUUCGGACGA 5’

query=JS16_t0476450, target=FGSG_02611, score=3, range=1890-1907, strand=-1

```
            :: ::::::: ::::::
query   3’ UGGGUUGGAACAAAAGUU 5’

query=JS16_t0105091, target=FGSG_07348, score=2.5, range=710-728, strand=-1

target  5’ UCGGCAAGGACGACUUCUU 3’
           ::::.: ::::::::::::
query   3’ AGCUG-UCCUGCUGAAGAU 5’

query=JS16_t0105091, target=FGSG_11026, score=3, range=7432-7449, strand=1

target  5’ AUGGCAGGACGGCUUCUA 3’
            .:.:::::::.::::::
query   3’ AGCUGUCCUGCUGAAGAU 5’

query=JS16_t0105091, target=FgHV1gp2, score=3, range=7193-7211, strand=1

target  5’ UCGCACAGGAUGACUUCUC 3’
           ::: ::::::.:::::::
query   3’ AGC-UGUCCUGCUGAAGAU 5’

query=JS16_t0105091, target=FGSG_10756, score=3, range=447-463, strand=1

target  5’ UCGACAGGAUG-CUUCUA 3’
           :::::::::.: ::::::
query   3’ AGCUGUCCUGCUGAAGAU 5’

query=JS16_t0105091, target=FGSG_04187, score=3, range=811-827, strand=1

target  5’ ACGA-AGGACGGCUUCUA 3’
            ::: ::::::.::::::
query   3’ AGCUGUCCUGCUGAAGAU 5’

query=JS16_t0105091, target=FGSG_10672, score=3.5, range=1-18, strand=1

target  5’ AUGACAGGACGACUUCAA 3’
            .:::::::::::::: :
query   3’ AGCUGUCCUGCUGAAGAU 5’

query=JS16_t0105091, target=FGSG_07262, score=3.5, range=4940-4958, strand=1

target  5’ ACGACCAGGAUGACUUCUG 3’
            ::: :::::.:::::::.
query   3’ AGCU-GUCCUGCUGAAGAU 5’

query=JS16_t0105091, target=FGSG_13722, score=3.5, range=12-28, strand=-1

target  5’ UCGGCAGGACGA-UUCUC 3’
           :::.::::::::: ::::
query   3’ AGCUGUCCUGCUGAAGAU 5’

query=JS16_t0105091, target=FGSG_10316, score=3.5, range=412-428, strand=-1

target  5’ UUGA-AGGAUGACUUCUU 3’
           :.:: ::::.:::::::
query   3’ AGCUGUCCUGCUGAAGAU 5’

query=JS16_t0105091, target=FGSG_01430, score=4, range=636-653, strand=1

target  5’ UUGGCAGGAGGACUUUUA 3’
           :.:.::::: :::::.::
query   3’ AGCUGUCCUGCUGAAGAU 5’

query=JS16_t0105091, target=FGSG_05772, score=4, range=944-962, strand=1

target  5’ UCGAACAGGCCGACUUUUA 3’
           ::: ::::: ::::::.::
query   3’ AGC-UGUCCUGCUGAAGAU 5’

query=JS16_t0105091, target=FGSG_05168, score=4, range=47-64, strand=1

target  5’ AGGACACGACGACUUCUA 3’
             :::: :::::::::::
query   3’ AGCUGUCCUGCUGAAGAU 5’

query=JS16_t0105091, target=FGSG_09793, score=4, range=164-182, strand=1

target  5’ UCGAAAAGGACGGCUUCUU 3’
           ::: : ::::::.:::::
query   3’ AGC-UGUCCUGCUGAAGAU 5’

query=JS16_t0105091, target=FGSG_12275, score=4, range=266-282, strand=1

target  5’ UC-ACAGGACGGCAUCUA 3’
           :: ::::::::.: ::::
query   3’ AGCUGUCCUGCUGAAGAU 5’

query=JS16_t0105091, target=FGSG_04433, score=4, range=875-892, strand=1

target  5’ UCAAAAGCACGACUUCUA 3’
           :: : :: ::::::::::
query   3’ AGCUGUCCUGCUGAAGAU 5’
```

```
target  5’ GCCUUGAAGAAGCCUUCU 3’
           .:::.:::::::::: ::
query   3’ UGGAGCUUCUUCGGACGA 5’

query=JS16_t0476450, target=FGSG_12021, score=3, range=2158-2174, strand=-1

target  5’ ACCU-GAGGAAGUCUGCU 3’
           :::: ::.::::.:::::
query   3’ UGGAGCUUCUUCGGACGA 5’

query=JS16_t0476450, target=FGSG_04639, score=3, range=1206-1223, strand=-1

target  5’ AGCUCGAAGAACCCUGCU 3’
           : ::::::::: ::::::
query   3’ UGGAGCUUCUUCGGACGA 5’

query=JS16_t0476450, target=FGSG_00434, score=3, range=3323-3339, strand=-1

target  5’ ACCU-GAGGAAGCCUGCC 3’
           :::: ::.:::::::::
query   3’ UGGAGCUUCUUCGGACGA 5’

query=JS16_t0476450, target=FGSG_03497, score=3.5, range=2111-2128, strand=1

target  5’ AUCUCGAUGAAGCUUGCU 3’
           :.::::: :::::.::::
query   3’ UGGAGCUUCUUCGGACGA 5’

query=JS16_t0476450, target=FGSG_04358, score=3.5, range=1198-1215, strand=1

target  5’ GCUUUGCAGAAGCCUGCU 3’
           .:.:.: :::::::::::
query   3’ UGGAGCUUCUUCGGACGA 5’

query=JS16_t0476450, target=FGSG_05861, score=3.5, range=547-564, strand=1

target  5’ ACCGUGGAGAAGCUUGCU 3’
           ::: .:.::::::.::::
query   3’ UGGAGCUUCUUCGGACGA 5’

query=JS16_t0476450, target=FGSG_09512, score=3.5, range=997-1014, strand=1

target  5’ ACCAUGAAGGAGCUUGCU 3’
           ::: .::::.:::.::::
query   3’ UGGAGCUUCUUCGGACGA 5’

query=JS16_t0476450, target=FGSG_12330, score=3.5, range=242-259, strand=1

target  5’ CCUUAGAAGAAGCUUGCU 3’
            :.: ::::::::.::::
query   3’ UGGAGCUUCUUCGGACGA 5’

query=JS16_t0476450, target=FGSG_00348, score=3.5, range=1880-1896, strand=1

target  5’ GCCUC-AAGAAGUCUGCU 3’
           .:::: ::::::.:::::
query   3’ UGGAGCUUCUUCGGACGA 5’

query=JS16_t0476450, target=FGSG_06247, score=3.5, range=113-129, strand=1

target  5’ GCCUCG-AGAAGCUUGCU 3’
           .::::: ::::::.::::
query   3’ UGGAGCUUCUUCGGACGA 5’

query=JS16_t0476450, target=FGSG_01690, score=3.5, range=4873-4890, strand=1

target  5’ GCCUGGAAGAAGCGUGCU 3’
           .::: :::::::: ::::
query   3’ UGGAGCUUCUUCGGACGA 5’

query=JS16_t0476450, target=FGSG_02881, score=3.5, range=953-970, strand=1

target  5’ ACAUUGAACAAGCCUGCU 3’
           :: :.::: :::::::::
query   3’ UGGAGCUUCUUCGGACGA 5’

query=JS16_t0476450, target=FGSG_07651, score=3.5, range=439-456, strand=1

target  5’ ACUCCCAAGAAGCCUGCU 3’
           ::.  : ::::::::::::
query   3’ UGGAGCUUCUUCGGACGA 5’

query=JS16_t0476450, target=FGSG_01681, score=3.5, range=1010-1026, strand=1

target  5’ GCCCCGAAGAAGCC-GCU 3’
           .:: :::::::::: :::
query   3’ UGGAGCUUCUUCGGACGA 5’

query=JS16_t0476450, target=FGSG_13157, score=3.5, range=771-787, strand=1

target  5’ AUC-CGAAGAUGCCUGCU 3’
```

```
query=JS16_t0105091, target=FGSG_06630, score=4, range=1469-1486, strand=-1

target  5’ UUGACAGGGCGAGUUCUG 3’
           :.::::::.::: ::::.
query   3’ AGCUGUCCUGCUGAAGAU 5’

query=JS16_t0105091, target=FGSG_11267, score=4, range=206-223, strand=-1

target  5’ UCGACAGGAAGGCUUCUC 3’
           ::::::::: :.:::::
query   3’ AGCUGUCCUGCUGAAGAU 5’

query=JS16_t0105091, target=FGSG_01222, score=4, range=811-828, strand=-1

target  5’ UCUGUAGGAUGACUUCUC 3’
           :: ..::::.:::::::
query   3’ AGCUGUCCUGCUGAAGAU 5’

query=JS16_t0105091, target=FGSG_01729, score=4, range=608-625, strand=-1

target  5’ UUGCUAGGACGGCUUCUU 3’
           :.: .::::::.:::::
query   3’ AGCUGUCCUGCUGAAGAU 5’

query=JS16_t0105091, target=FGSG_05850, score=4, range=596-614, strand=-1

target  5’ UUGGCAAGGAUGACUUCUU 3’
           :.:.: ::::.:::::::
query   3’ AGCUG-UCCUGCUGAAGAU 5’

query=JS16_t0105091, target=FGSG_04432, score=4, range=43-59, strand=-1

target  5’ UUG-CAGGGCGGCUUCUG 3’
           :.: ::::.::.:::::.
query   3’ AGCUGUCCUGCUGAAGAU 5’

query=JS16_t0105091, target=FGSG_05424, score=4, range=1301-1318, strand=-1

target  5’ UAGCCAGGACGGUUUCUA 3’
           : : :::::::..:::::
query   3’ AGCUGUCCUGCUGAAGAU 5’

query=JS16_t0105091, target=FGSG_09754, score=4, range=363-380, strand=-1

target  5’ UCUUCAGGACGACUUUUC 3’
           ::  :::::::::::.:
query   3’ AGCUGUCCUGCUGAAGAU 5’

query=JS16_t0105091, target=FGSG_08598, score=4, range=686-702, strand=-1

target  5’ UCGA-AGGCCGGCUUCUA 3’
           :::: ::: ::.::::::
query   3’ AGCUGUCCUGCUGAAGAU 5’

query=JS16_t0105091, target=FGSG_06659, score=4, range=158-174, strand=-1

target  5’ CAGAC-GGACGACUUCUA 3’
             ::: ::::::::::::
query   3’ AGCUGUCCUGCUGAAGAU 5’

query=JS16_t0105091, target=FGSG_01877, score=4, range=1132-1148, strand=-1

target  5’ UUGGCA-GACGACUUCUU 3’
           :.:.:: ::::::::::
query   3’ AGCUGUCCUGCUGAAGAU 5’

query=JS16_t3135635, target=FGSG_07383, score=1, range=100-118, strand=1

target  5’ CAAGAAGGAAAAUGGGGAG 3’
           ::::::::::::::.::::
query   3’ GUUCUUCCUUUUACUCCUC 5’

query=JS16_t3135635, target=FGSG_05286, score=2.5, range=2172-2190, strand=1

target  5’ AGAGAAGGAAAGUGAGGAG 3’
            .:::::::::.:::::::
query   3’ GUUCUUCCUUUUACUCCUC 5’

query=JS16_t3135635, target=FGSG_03031, score=2.5, range=491-509, strand=-1

target  5’ UAAGAAGGGAAAUGAGGAA 3’
           .:::::::.:::::::::
query   3’ GUUCUUCCUUUUACUCCUC 5’

query=JS16_t3135635, target=FGSG_09227, score=3, range=2145-2163, strand=1

target  5’ CAAGAAGGAGUAUGAGGAG 3’
           :::::::::. ::::::::
query   3’ GUUCUUCCUUUUACUCCUC 5’

query=JS16_t3135635, target=FGSG_01393, score=3, range=150-168, strand=1
```

```
           :.: :::::: :::::::
query   3’ UGGAGCUUCUUCGGACGA 5’

query=JS16_t0476450, target=FGSG_10520, score=3.5, range=414-431, strand=-1

target  5’ AUCUCGAAGGAGCUUGCG 3’
           :.:::::::.:::.:::
query   3’ UGGAGCUUCUUCGGACGA 5’

query=JS16_t0476450, target=FGSG_01662, score=3.5, range=880-897, strand=-1

target  5’ AUCGCGAAGAAGUCUGUU 3’
           :.: ::::::::.:::.:
query   3’ UGGAGCUUCUUCGGACGA 5’

query=JS16_t0476450, target=FGSG_09031, score=3.5, range=681-698, strand=-1

target  5’ CCCCUGAAGAAGCCUGUU 3’
            :: .::::::::::::.:
query   3’ UGGAGCUUCUUCGGACGA 5’

query=JS16_t0476450, target=FGSG_11262, score=3.5, range=266-282, strand=-1

target  5’ AUCUCGAAGAA-UCUGCU 3’
           :.::::::::: .:::::
query   3’ UGGAGCUUCUUCGGACGA 5’

query=JS16_t0476450, target=FGSG_06146, score=3.5, range=463-479, strand=-1

target  5’ GUCUUGAAGAAG-CUGCU 3’
           ..::.::::::: :::::
query   3’ UGGAGCUUCUUCGGACGA 5’

query=JS16_t0476450, target=FGSG_03567, score=3.5, range=680-697, strand=-1

target  5’ ACUUGGAAGAACCCUGCU 3’
           ::.: :::::: ::::::
query   3’ UGGAGCUUCUUCGGACGA 5’

query=JS16_t0476450, target=FGSG_03964, score=3.5, range=2738-2756, strand=-1

target  5’ ACUGUCGAAGAAGCCUUCU 3’
           ::.  :::::::::::: ::
query   3’ UGG-AGCUUCUUCGGACGA 5’

query=JS16_t0476450, target=FGSG_04684, score=3.5, range=278-296, strand=-1

target  5’ GCCCCGUAAGAAGCCUGCU 3’
           .:: :: ::::::::::::
query   3’ UGGAGC-UUCUUCGGACGA 5’

query=JS16_t0476450, target=FGSG_08764, score=3.5, range=199-216, strand=-1

target  5’ GGCGCGAAGAAGCUUGCU 3’
           .  : :::::::::.::::
query   3’ UGGAGCUUCUUCGGACGA 5’

query=JS16_t0476450, target=FGSG_03699, score=3.5, range=1461-1477, strand=-1

target  5’ CCUUCGAA-AAGCCUGCU 3’
            :.::::: :::::::::
query   3’ UGGAGCUUCUUCGGACGA 5’

query=JS16_t0476450, target=FGSG_06112, score=3.5, range=98-114, strand=-1

target  5’ ACUUCG-AGAAGCCUGCG 3’
           ::.::: ::::::::::
query   3’ UGGAGCUUCUUCGGACGA 5’

query=JS16_t0476450, target=FGSG_00248, score=4, range=1544-1561, strand=1

target  5’ ACUUUGAAGAGGCUUGCG 3’
           ::.:.:::::.::.:::
query   3’ UGGAGCUUCUUCGGACGA 5’

query=JS16_t0476450, target=FGSG_03159, score=4, range=463-480, strand=1

target  5’ GCUUGGAAGAGGCUUGCU 3’
           .:.: :::::.::.::::
query   3’ UGGAGCUUCUUCGGACGA 5’

query=JS16_t0476450, target=FGSG_13242, score=4, range=662-679, strand=1

target  5’ ACCUCGCUGAAGCCUGCU 3’
           ::::::  ::::::::::
query   3’ UGGAGCUUCUUCGGACGA 5’

query=JS16_t0476450, target=FGSG_04129, score=4, range=640-657, strand=1

target  5’ ACCUCAAAGAAGCCUCCU 3’
           ::::: ::::::::: ::
query   3’ UGGAGCUUCUUCGGACGA 5’
```

```
target  5' AAAGAAGGAAAAUAAGGAG 3'
            :::::::::::: :::::
query   3' GUUCUUCCUUUUACUCCUC 5'

query=JS16_t3135635, target=FGSG_05280, score=3, range=323-341, strand=-1

target  5' UAAGGAGGAUAAUGAGGAG 3'
           .:::.:::: :::::::::
query   3' GUUCUUCCUUUUACUCCUC 5'

query=JS16_t3135635, target=FGSG_06881, score=3.5, range=1398-1416, strand=1

target  5' AAAGGAGGAGAGUGAGGAG 3'
            :::.::::.:.:::::::
query   3' GUUCUUCCUUUUACUCCUC 5'

query=JS16_t3135635, target=FGSG_01123, score=3.5, range=780-798, strand=1

target  5' CAAGGAGGAAGAUGAGGGC 3'
           ::::.:::::.::::::.
query   3' GUUCUUCCUUUUACUCCUC 5'

query=JS16_t3135635, target=FGSG_08463, score=3.5, range=1920-1938, strand=1

target  5' CAAGGAGGAGAUUGAGGAG 3'
           ::::.::::.: :::::::
query   3' GUUCUUCCUUUUACUCCUC 5'

query=JS16_t3135635, target=FGSG_07588, score=3.5, range=621-639, strand=1

target  5' CAAGGAGGAGAAGGAGGAG 3'
           ::::.::::.:: ::::::
query   3' GUUCUUCCUUUUACUCCUC 5'

query=JS16_t3135635, target=FGSG_10967, score=3.5, range=510-528, strand=1

target  5' CAAGGAGGAGAAGGAGGAG 3'
           ::::.::::.:: ::::::
query   3' GUUCUUCCUUUUACUCCUC 5'

query=JS16_t3135635, target=FGSG_04277, score=3.5, range=471-489, strand=1

target  5' CGAGCAGGGAAGUGAGGAG 3'
           :.:: :::.::.:::::::
query   3' GUUCUUCCUUUUACUCCUC 5'

query=JS16_t3135635, target=FGSG_08649, score=3.5, range=1334-1353, strand=1

target  5' CAAGUGAGGGAAGUGAGGAG 3'
           :::: .:::.::.:::::::
query   3' GUUC-UUCCUUUUACUCCUC 5'

query=JS16_t3135635, target=FGSG_03987, score=3.5, range=871-889, strand=-1

target  5' CAAUGAGGAAAACGAGGAG 3'
           ::: .::::::: ::::::
query   3' GUUCUUCCUUUUACUCCUC 5'

query=JS16_t3135635, target=FGSG_05924, score=4, range=1104-1122, strand=1

target  5' CAAGAAGGAGAACGGGGAG 3'
           :::::::::.:: :.::::
query   3' GUUCUUCCUUUUACUCCUC 5'

query=JS16_t3135635, target=FGSG_06885, score=4, range=2505-2523, strand=1

target  5' UGAGAAGGAGAAUGAGAAG 3'
           ..:::::::.:::::: ::
query   3' GUUCUUCCUUUUACUCCUC 5'

query=JS16_t3135635, target=FGSG_02544, score=4, range=72-90, strand=1

target  5' CGAGGAGGAAGAGGAGGAG 3'
           :.::.:::::.: ::::::
query   3' GUUCUUCCUUUUACUCCUC 5'

query=JS16_t3135635, target=FGSG_02511, score=4, range=786-804, strand=1

target  5' CGAGGACGAAGAUGAGGAG 3'
           :.::.: :::.::::::::
query   3' GUUCUUCCUUUUACUCCUC 5'

query=JS16_t3135635, target=FGSG_05686, score=4, range=1503-1521, strand=1

target  5' CGAGGAGGAAGAGGAGGAG 3'
           :.::.:::::.: ::::::
query   3' GUUCUUCCUUUUACUCCUC 5'

query=JS16_t3135635, target=FGSG_08927, score=4, range=552-570, strand=1

target  5' CGAGGAGGAAUAUGGGGAG 3'

query=JS16_t0476450, target=FGSG_03090, score=4, range=641-657, strand=1

target  5' GCCUUGAAGGAG-CUGCU 3'
           .:::.::::.:: :::::
query   3' UGGAGCUUCUUCGGACGA 5'

query=JS16_t0476450, target=FGSG_00716, score=4, range=1308-1326, strand=1

target  5' ACCUCGAAGAAGUCCGGCU 3'
           ::::::::::: :: :::
query   3' UGGAGCUUCUUC-GGACGA 5'

query=JS16_t0476450, target=FGSG_00672, score=4, range=2730-2747, strand=1

target  5' AUUCCGAAAAAGCCUGCU 3'
           :.. :::: :::::::::
query   3' UGGAGCUUCUUCGGACGA 5'

query=JS16_t0476450, target=FGSG_06265, score=4, range=2368-2385, strand=1

target  5' GCUACCAAGAAGCCUGCU 3'
           .:. : ::::::::::::
query   3' UGGAGCUUCUUCGGACGA 5'

query=JS16_t0476450, target=FGSG_12263, score=4, range=331-348, strand=1

target  5' ACAUGGAAGGAGCCUGUU 3'
           :: : ::::.::::::.:
query   3' UGGAGCUUCUUCGGACGA 5'

query=JS16_t0476450, target=FGSG_09276, score=4, range=583-600, strand=1

target  5' GACUUGAAGAAGCCCGCU 3'
           . ::.:::::::: :::
query   3' UGGAGCUUCUUCGGACGA 5'

query=JS16_t0476450, target=FGSG_09548, score=4, range=220-237, strand=1

target  5' GUCGAGGAGAAGCCUGCU 3'
           ..:  :.:::::::::::
query   3' UGGAGCUUCUUCGGACGA 5'

query=JS16_t0476450, target=FGSG_00273, score=4, range=179-195, strand=1

target  5' CCUUUGAAGAAG-CUGCU 3'
            :.:.:::::::: :::::
query   3' UGGAGCUUCUUCGGACGA 5'

query=JS16_t0476450, target=FGSG_09212, score=4, range=1521-1537, strand=1

target  5' ACC-CGAAGAAGUCUGGU 3'
           ::: ::::::::.::: :
query   3' UGGAGCUUCUUCGGACGA 5'

query=JS16_t0476450, target=FGSG_10986, score=4, range=1205-1221, strand=1

target  5' ACCU-GAAGGAGCCUUCU 3'
           :::: ::::.::::: ::
query   3' UGGAGCUUCUUCGGACGA 5'

query=JS16_t0476450, target=FGSG_05385, score=4, range=1405-1421, strand=1

target  5' ACC-CGAAGAAUCUUGCU 3'
           ::: ::::::: :.::::
query   3' UGGAGCUUCUUCGGACGA 5'

query=JS16_t0476450, target=FGSG_05601, score=4, range=200-216, strand=1

target  5' ACCU-GAAGGAGCCUGAU 3'
           :::: ::::.:::::: :
query   3' UGGAGCUUCUUCGGACGA 5'

query=JS16_t0476450, target=FGSG_13605, score=4, range=418-435, strand=1

target  5' GGUGCGAAGAAGCCUGCC 3'
           . .  :::::::::::::
query   3' UGGAGCUUCUUCGGACGA 5'

query=JS16_t0476450, target=FGSG_13319, score=4, range=721-738, strand=1

target  5' GAUGAGAAGAAGCCUGCU 3'
           . .   ::::::::::::
query   3' UGGAGCUUCUUCGGACGA 5'

query=JS16_t0476450, target=FGSG_10235, score=4, range=268-285, strand=1

target  5' AAGGAGAAGAAGCCUGCU 3'
           :     ::::::::::::
query   3' UGGAGCUUCUUCGGACGA 5'

query=JS16_t0476450, target=FGSG_05916, score=4, range=217-234, strand=1
```

```
           :.::.::::: :::.::::
query   3' GUUCUUCCUUUUACUCCUC 5'

query=JS16_t3135635, target=FGSG_13489, score=4, range=609-627, strand=1

target  5' CGAGGAGGAAGAAGAGGAG 3'
           :.::.:::::.: ::::::
query   3' GUUCUUCCUUUUACUCCUC 5'

query=JS16_t3135635, target=FGSG_07467, score=4, range=441-459, strand=1

target  5' CGAGGAGGACGAUGAGGAG 3'
           :.::.:::: .::::::::
query   3' GUUCUUCCUUUUACUCCUC 5'

query=JS16_t3135635, target=FGSG_10766, score=4, range=201-219, strand=1

target  5' UGAGAUGGAGGAUGAGGAG 3'
           ..::: :::..::::::::
query   3' GUUCUUCCUUUUACUCCUC 5'

query=JS16_t3135635, target=FGSG_08644, score=4, range=1500-1518, strand=1

target  5' CGACGAGGAGGAUGAGGAG 3'
           :.: .::::..::::::::
query   3' GUUCUUCCUUUUACUCCUC 5'

query=JS16_t3135635, target=FGSG_03106, score=4, range=826-844, strand=1

target  5' UCAGGAGGAGAAUGGGGAG 3'
           . ::.::::.::::.::::
query   3' GUUCUUCCUUUUACUCCUC 5'

query=JS16_t3135635, target=FGSG_11195, score=4, range=357-375, strand=1

target  5' AAAGAAGGAAGAGGAGGAG 3'
            :::::::::.: ::::::
query   3' GUUCUUCCUUUUACUCCUC 5'

query=JS16_t3135635, target=FGSG_08778, score=4, range=1587-1605, strand=1

target  5' CAAGAAGGAGAAGGAGGAA 3'
           :::::::::.:: :::::
query   3' GUUCUUCCUUUUACUCCUC 5'

query=JS16_t3135635, target=FGSG_07639, score=4, range=1248-1266, strand=1

target  5' CAAGAAGGAAGAUCAGGAA 3'
           ::::::::::.:: ::::
query   3' GUUCUUCCUUUUACUCCUC 5'

query=JS16_t3135635, target=FGSG_08865, score=4, range=132-150, strand=1

target  5' GAAGAAGGACGAUGAGGAG 3'
            :::::::: .::::::::
query   3' GUUCUUCCUUUUACUCCUC 5'

query=JS16_t3135635, target=FGSG_05334, score=4, range=1410-1428, strand=1

target  5' GAAGAAAGAGAAUGAGGAG 3'
            ::::: ::.:::::::::
query   3' GUUCUUCCUUUUACUCCUC 5'

query=JS16_t3135635, target=FGSG_11407, score=4, range=1182-1200, strand=1

target  5' CAAGAAAGAAGAUGAGGAU 3'
           :::::: :::.:::::::
query   3' GUUCUUCCUUUUACUCCUC 5'

query=JS16_t3135635, target=FGSG_13925, score=4, range=639-657, strand=1

target  5' AAAGAAGGAAGAUGACGAG 3'
            :::::::::.:::: :::
query   3' GUUCUUCCUUUUACUCCUC 5'

query=JS16_t3135635, target=FGSG_12057, score=4, range=1692-1710, strand=1

target  5' GGAGAGGGAAAAUCAGGAG 3'
            .:::.::::::: :::::
query   3' GUUCUUCCUUUUACUCCUC 5'

query=JS16_t3135635, target=FGSG_00083, score=4, range=1491-1509, strand=1

target  5' UGAGAAGGAAAAUAAGGAU 3'
           ..::::::::::: ::::
query   3' GUUCUUCCUUUUACUCCUC 5'

query=JS16_t3135635, target=FGSG_05163, score=4, range=618-636, strand=1

target  5' UAGCAAGGGAAAUGAGGAU 3'
           .:.  ::::.:::::::::
query   3' GUUCUUCCUUUUACUCCUC 5'

target  5' AGCAAGAAGAAGUCUGCU 3'
           : :  :::::::.:::::
query   3' UGGAGCUUCUUCGGACGA 5'

query=JS16_t0476450, target=FGSG_04307, score=4, range=316-333, strand=1

target  5' ACGAAGAAGAAGCCUGCA 3'
           ::   ::::::::::::
query   3' UGGAGCUUCUUCGGACGA 5'

query=JS16_t0476450, target=FGSG_09845, score=4, range=295-311, strand=1

target  5' ACCUGGGAGAA-CCUGCU 3'
           :::: :.:::: ::::::
query   3' UGGAGCUUCUUCGGACGA 5'

query=JS16_t0476450, target=FGSG_02701, score=4, range=775-792, strand=-1

target  5' ACCUCGGAGAAUCUUGCU 3'
           ::::::.:::: :.::::
query   3' UGGAGCUUCUUCGGACGA 5'

query=JS16_t0476450, target=FGSG_00293, score=4, range=1774-1791, strand=-1

target  5' ACGUCGAAGGAGCUUGUU 3'
           :: ::::::.:::.::.:
query   3' UGGAGCUUCUUCGGACGA 5'

query=JS16_t0476450, target=FGSG_00759, score=4, range=900-917, strand=-1

target  5' GCCUUGAAGAAGUCUCCU 3'
           .:::.:::::::.:: ::
query   3' UGGAGCUUCUUCGGACGA 5'

query=JS16_t0476450, target=FGSG_05887, score=4, range=51-68, strand=-1

target  5' UCCUCGAAGAGGUCUGCG 3'
            :::::::::.:.::::
query   3' UGGAGCUUCUUCGGACGA 5'

query=JS16_t0476450, target=FGSG_05735, score=4, range=498-515, strand=-1

target  5' UCCUCGAGGAAGGCUGCU 3'
            ::::::.:::: :::::
query   3' UGGAGCUUCUUCGGACGA 5'

query=JS16_t0476450, target=FGSG_10097, score=4, range=467-484, strand=-1

target  5' UCUUUGCAGAAGCCUGCU 3'
            :.:.: :::::::::::
query   3' UGGAGCUUCUUCGGACGA 5'

query=JS16_t0476450, target=FGSG_00633, score=4, range=10372-10389, strand=-1

target  5' AACGCGAAGAAGCUUGUU 3'
           : : :::::::::.::.:
query   3' UGGAGCUUCUUCGGACGA 5'

query=JS16_t0476450, target=FGSG_01629, score=4, range=1451-1468, strand=-1

target  5' AGCUGGAAGAGGCCUGCG 3'
           : :: :::::.::::::
query   3' UGGAGCUUCUUCGGACGA 5'

query=JS16_t0476450, target=FGSG_06054, score=4, range=1805-1821, strand=-1

target  5' ACCU-GAAGAAUCCUGUU 3'
           :::: :::::: ::::.:
query   3' UGGAGCUUCUUCGGACGA 5'

query=JS16_t0476450, target=FGSG_05650, score=4, range=350-366, strand=-1

target  5' AGCUCG-AGAAGCCUGUU 3'
           : :::: :::::::::.:
query   3' UGGAGCUUCUUCGGACGA 5'

query=JS16_t0476450, target=FGSG_08905, score=4, range=240-257, strand=-1

target  5' ACGGCGAAGAAGCCGGCU 3'
           ::  :::::::::: :::
query   3' UGGAGCUUCUUCGGACGA 5'

query=JS16_t0476450, target=FGSG_07953, score=4, range=2260-2276, strand=-1

target  5' ACCUCGAAGGA-CUUGCU 3'
           :::::::::.: :.::::
query   3' UGGAGCUUCUUCGGACGA 5'

No results for JS16_t3198252
query=JS16_t1406055, target=FGSG_07360, score=4, range=304-326, strand=-1
```

```
query=JS16_t3135635, target=FGSG_06647, score=4, range=1008-1026, strand=1

target  5’ CGACGAGGAAGAUGAGGAU 3’
           :.: .:::::.:::::::
query   3’ GUUCUUCCUUUUACUCCUC 5’

query=JS16_t3135635, target=FGSG_04027, score=4, range=1230-1248, strand=1

target  5’ AGAGUGGGAAGAUGAGGAG 3’
            .:: .::::.::::::::
query   3’ GUUCUUCCUUUUACUCCUC 5’

query=JS16_t3135635, target=FGSG_06836, score=4, range=222-240, strand=1

target  5’ CAAGUACGAAAAUGGGGAG 3’
           :::: : :::::::.::::
query   3’ GUUCUUCCUUUUACUCCUC 5’

query=JS16_t3135635, target=FGSG_04107, score=4, range=669-687, strand=1

target  5’ CCAGAAGGAGAACGAGGAG 3’
           : :::::::.:: ::::::
query   3’ GUUCUUCCUUUUACUCCUC 5’

query=JS16_t3135635, target=FGSG_02485, score=4, range=642-660, strand=1

target  5’ GAAGAAGGAAAAAGAGGAU 3’
            ::::::::::: :::::
query   3’ GUUCUUCCUUUUACUCCUC 5’

query=JS16_t3135635, target=FGSG_10760, score=4, range=2100-2118, strand=1

target  5’ GAAAAAGGAAGAUGAGGAC 3’
            :: ::::::.:::::::
query   3’ GUUCUUCCUUUUACUCCUC 5’

query=JS16_t3135635, target=FGSG_02021, score=4, range=505-522, strand=1

target  5’ CAAG-AGGAGAAUGGGGAU 3’
           :::: ::::.::::.:::
query   3’ GUUCUUCCUUUUACUCCUC 5’

query=JS16_t3135635, target=FGSG_00389, score=4, range=1024-1041, strand=1

target  5’ CAAGAA-GAAAAAGAGGAG 3’
           :::::: ::::: ::::::
query   3’ GUUCUUCCUUUUACUCCUC 5’

query=JS16_t3135635, target=FGSG_08582, score=4, range=1896-1914, strand=1

target  5’ UGCGACGGAAAAUGAGGAU 3’
           ..  :: ::::::::::::
query   3’ GUUCUUCCUUUUACUCCUC 5’

query=JS16_t3135635, target=FGSG_07729, score=4, range=1368-1386, strand=1

target  5’ CAACCAAGAAAAUGAGGAG 3’
           :::  : ::::::::::::
query   3’ GUUCUUCCUUUUACUCCUC 5’

query=JS16_t3135635, target=FGSG_02351, score=4, range=364-382, strand=1

target  5’ CCAGAUGGAAAAGGAGGAG 3’
           : ::: :::::: ::::::
query   3’ GUUCUUCCUUUUACUCCUC 5’

query=JS16_t3135635, target=FGSG_10104, score=4, range=690-708, strand=1

target  5’ CACCAAGGAAAAUAAGGAG 3’
           ::  ::::::::: :::::
query   3’ GUUCUUCCUUUUACUCCUC 5’

query=JS16_t3135635, target=FGSG_09981, score=4, range=1621-1638, strand=1

target  5’ CAAGCA-GAAGAUGAGGAG 3’
           :::: : :::.::::::::
query   3’ GUUCUUCCUUUUACUCCUC 5’

query=JS16_t3135635, target=FGSG_00292, score=4, range=1513-1531, strand=-1

target  5’ CGAGAGGGUAGAUGAGGAG 3’
           :.:::.:: :.::::::::
query   3’ GUUCUUCCUUUUACUCCUC 5’

query=JS16_t3135635, target=FGSG_05310, score=4, range=1025-1043, strand=-1

target  5’ CAGGGAGGACAGUGAGGAG 3’
           ::.:.:::: :.:::::::
query   3’ GUUCUUCCUUUUACUCCUC 5’

query=JS16_t3135635, target=FGSG_00849, score=4, range=54-73, strand=-1

target  5’ GCGGCUAUCUUGUUGAGAAUUGG 3’
           : :::: .::::::::::.::.
query   3’ CCCCGACGGAACAACUCUUGACU 5’

query=JS16_t1406055, target=FGSG_05452, score=4, range=416-437, strand=-1

target  5’ UGCGCU-CCUUGUUGAGAGCUGA 3’
            : ::: ::::::::::.::::
query   3’ CCCCGACGGAACAACUCUUGACU 5’

No results for JS16_t3198313
No results for JS16_t0396641
query=JS16_t4095177, target=FGSG_02767, score=4, range=259-278, strand=1

target  5’ GUUG-UUGGCAUCAUCGCAUU 3’
           ::.. :::::::: :::::::
query   3’ CAGUGAACCGUAGGAGCGUAA 5’

No results for JS16_t2302369
query=JS16_t2302370, target=FGSG_08812, score=2.5, range=370-387, strand=1

target  5’ GUCCCUUGGGCGUCGGUC 3’
            .:::.::::::::::::
query   3’ AGGGGGACCCGCAGCCAG 5’

query=JS16_t2302370, target=FGSG_08173, score=2.5, range=1118-1135, strand=1

target  5’ UCCCUCAGGGCGUCGGUC 3’
           ::::.: :::::::::::
query   3’ AGGGGGACCCGCAGCCAG 5’

query=JS16_t2302370, target=FGSG_06513, score=3, range=638-654, strand=1

target  5’ ACCCCCUGGGCGU-GGUC 3’
            :::::::::::: ::::
query   3’ AGGGGGACCCGCAGCCAG 5’

query=JS16_t2302370, target=FGSG_09782, score=3, range=1626-1642, strand=1

target  5’ UUCCUCUGGGC-UCGGUC 3’
           :.::.:::::: ::::::
query   3’ AGGGGGACCCGCAGCCAG 5’

query=JS16_t2302370, target=FGSG_13586, score=3, range=2168-2186, strand=-1

target  5’ UCCGCCUGGGCGUCGUGUC 3’
           ::: ::::::::::: :::
query   3’ AGGGGGACCCGCAGC-CAG 5’

query=JS16_t2302370, target=FGSG_02008, score=3.5, range=623-640, strand=1

target  5’ UUCUUCUGGUCGUCGGUC 3’
           :.:..:::: ::::::::
query   3’ AGGGGGACCCGCAGCCAG 5’

query=JS16_t2302370, target=FGSG_02714, score=3.5, range=161-177, strand=1

target  5’ UACCUCUGGGCGU-GGUC 3’
           : ::.::::::::: ::::
query   3’ AGGGGGACCCGCAGCCAG 5’

query=JS16_t2302370, target=FGSG_06070, score=3.5, range=304-321, strand=-1

target  5’ GCUUCCUGGGUGUCGGUU 3’
            :..::::::.::::::.
query   3’ AGGGGGACCCGCAGCCAG 5’

query=JS16_t2302370, target=FGSG_04117, score=3.5, range=633-649, strand=-1

target  5’ UCUCCUUGGG-GUCGGUC 3’
           ::.::.:::: :::::::
query   3’ AGGGGGACCCGCAGCCAG 5’

query=JS16_t2302370, target=FGSG_12385, score=3.5, range=1098-1115, strand=-1

target  5’ UCAUCCUGUGCGUCGGUC 3’
           :: .:::: :::::::::
query   3’ AGGGGGACCCGCAGCCAG 5’

query=JS16_t2302370, target=FGSG_07026, score=3.5, range=90-107, strand=-1

target  5’ UUGCCCUGGGCGUCUGUC 3’
           :.  ::::::::::: :::
query   3’ AGGGGGACCCGCAGCCAG 5’

query=JS16_t2302370, target=FGSG_09099, score=3.5, range=1125-1142, strand=-1

target  5’ UUGACCUGGGCGUCGGUA 3’
           :.   :::::::::::::
query   3’ AGGGGGACCCGCAGCCAG 5’

query=JS16_t2302370, target=FGSG_08532, score=3.5, range=2485-2501, strand=-1
```

```
target  5’ CGAGGAGGAAGAUGAUGGAG 3’
           :.::.:::::.:::: ::::
query   3’ GUUCUUCCUUUUACU-CCUC 5’

query=JS16_t3135635, target=FGSG_00800, score=4, range=1270-1288, strand=-1

target  5’ CAAGAAGGAAGAUGAGCAA 3’
           ::::::::::.::::: :
query   3’ GUUCUUCCUUUUACUCCUC 5’

query=JS16_t3135635, target=FGSG_13821, score=4, range=927-945, strand=-1

target  5’ CCAGAAGGGAAAUGAGGGC 3’
           : ::::::.::::::::.
query   3’ GUUCUUCCUUUUACUCCUC 5’

query=JS16_t3135635, target=FGSG_13866, score=4, range=195-214, strand=-1

target  5’ CGAGGAGGAAAAUGAUGGAA 3’
           :.::.:::::::::: :::
query   3’ GUUCUUCCUUUUACU-CCUC 5’

query=JS16_t3135635, target=FGSG_07596, score=4, range=799-817, strand=-1

target  5’ CAAUAAGGAAAGUGAUGAG 3’
           ::: :::::::.::: :::
query   3’ GUUCUUCCUUUUACUCCUC 5’

query=JS16_t3135635, target=FGSG_00831, score=4, range=658-675, strand=-1

target  5’ GAAGAAGGAGAAUG-GGAG 3’
            ::::::::.:::: ::::
query   3’ GUUCUUCCUUUUACUCCUC 5’

query=JS16_t3135635, target=FGSG_08879, score=4, range=1002-1020, strand=-1

target  5’ UGAGCCGGAGAAUGAGGAG 3’
           ..::  :::.:::::::::
query   3’ GUUCUUCCUUUUACUCCUC 5’

query=JS16_t3135635, target=FGSG_09016, score=4, range=118-136, strand=-1

target  5’ UGACCAGGAAGAUGAGGAG 3’
           ..:  :::::.::::::::
query   3’ GUUCUUCCUUUUACUCCUC 5’

query=JS16_t3135635, target=FGSG_11364, score=4, range=1508-1527, strand=-1

target  5’ GAAGAACGGAAAAUGAAGAG 3’
            ::::: ::::::::: :::
query   3’ GUUCUU-CCUUUUACUCCUC 5’

query=JS16_t3135635, target=FGSG_11187, score=4, range=76-93, strand=-1

target  5’ CAAG-AUGAAGAUGAGGAG 3’
           :::: : :::.::::::::
query   3’ GUUCUUCCUUUUACUCCUC 5’

No results for JS16_t2239617
No results for JS16_t3135658
query=JS16_t0365220, target=FGSG_13662, score=3.5, range=2601-2623, strand=1

target  5’ UGAAGGUCUAGUUGAUACUGCUG 3’
            : :::: ::::::::::::::.
query   3’ CCAUCCA-AUCAACUAUGACGAU 5’

query=JS16_t0365221, target=FGSG_11812, score=3, range=3133-3153, strand=-1

target  5’ GCUACUUCCAACAGGGCGGCC 3’
           ::: :::::::::::::.:
query   3’ CGA-GAAGGUUGUCCCGCUGA 5’

query=JS16_t0365221, target=FGSG_03856, score=4, range=445-464, strand=1

target  5’ GCUCAUCCAACGGGACGACU 3’
           :::: ::::::.:: :::::
query   3’ CGAGAAGGUUGUCCCGCUGA 5’

query=JS16_t0365221, target=FGSG_08948, score=4, range=689-707, strand=1

target  5’ GCUC-UCCAACAGCGCGACC 3’
           :::: :::::::: :::::
query   3’ CGAGAAGGUUGUCCCGCUGA 5’

query=JS16_t0365221, target=FGSG_09034, score=4, range=705-724, strand=-1

target  5’ CCUCCUCCAACAGGGCGAUA 3’
            ::: :::::::::::::.
query   3’ CGAGAAGGUUGUCCCGCUGA 5’

query=JS16_t0365221, target=FGSG_07869, score=4, range=222-240, strand=-1
```

```
target  5’ UCCAUCUGGGCG-CGGUC 3’
           ::: .::::::: :::::
query   3’ AGGGGGACCCGCAGCCAG 5’

query=JS16_t2302370, target=FGSG_03920, score=4, range=704-721, strand=1

target  5’ UCCUUUUGGCCGUCGGUC 3’
           :::...::: ::::::::
query   3’ AGGGGGACCCGCAGCCAG 5’

query=JS16_t2302370, target=FGSG_07338, score=4, range=1835-1852, strand=1

target  5’ UUCUCCUGAGCGUUGGUC 3’
           :.:.:::: ::::.::::
query   3’ AGGGGGACCCGCAGCCAG 5’

query=JS16_t2302370, target=FGSG_03798, score=4, range=269-286, strand=1

target  5’ UUUUCCUGGGCAUCGGUU 3’
           :...::::::: :::::.
query   3’ AGGGGGACCCGCAGCCAG 5’

query=JS16_t2302370, target=FGSG_10620, score=4, range=852-869, strand=1

target  5’ UGCCCCUGGGCGUCGGAA 3’
           : :::::::::::::
query   3’ AGGGGGACCCGCAGCCAG 5’

query=JS16_t2302370, target=FGSG_06605, score=4, range=1146-1163, strand=-1

target  5’ UUCUCUUGGGCGUAGGUC 3’
           :.:.:.::::::: ::::
query   3’ AGGGGGACCCGCAGCCAG 5’

query=JS16_t2302370, target=FGSG_04042, score=4, range=1733-1750, strand=-1

target  5’ GCUCUCUGGGCGUCGGGC 3’
            :.:.::::::::::: :
query   3’ AGGGGGACCCGCAGCCAG 5’

query=JS16_t2302370, target=FGSG_03176, score=4, range=1144-1161, strand=-1

target  5’ UUGCUCUGGGCGACGGUC 3’
           :.  :.::::::: :::::
query   3’ AGGGGGACCCGCAGCCAG 5’

query=JS16_t2302370, target=FGSG_10905, score=4, range=390-407, strand=-1

target  5’ AUGGCCUGGGCGUCGGUU 3’
            .  :::::::::::::.
query   3’ AGGGGGACCCGCAGCCAG 5’

query=JS16_t2302370, target=FGSG_03867, score=4, range=798-815, strand=-1

target  5’ UAGCCCUGGCCGUCGGUC 3’
           :  :::::: ::::::::
query   3’ AGGGGGACCCGCAGCCAG 5’

query=JS16_t2302370, target=FGSG_07834, score=4, range=885-902, strand=-1
target  5’ UACCGCUGGGCCUCGGUC 3’
           : :: :::::: ::::::
query   3’ AGGGGGACCCGCAGCCAG 5’

No results for JS16_t1406195
No results for JS16_t0269888
No results for JS16_t2302413
No results for JS16_t2302444
query=JS16_t0585399, target=FgHV1gp2, score=4, range=4117-4137, strand=1

target  5’ AUUCGACUGUGGUUCAAUAUG 3’
           ::: .:::::.::::::.::.
query   3’ UAAUUUGACAUCAAGUUGUAU 5’

No results for JS16_t2302543
No results for JS16_t1406396
No results for JS16_t3198651
No results for JS16_t3198716
No results for JS16_t2302678
No results for JS16_t3198724
query=JS16_t1406565, target=FGSG_10000, score=3, range=700-718, strand=-1

target  5’ AUAGUUCAGGUUCACUGGG 3’
           :::.. ::::::::::.::
query   3’ UAUUGCGUCCAAGUGAUCC 5’

query=JS16_t1406565, target=FGSG_08709, score=4, range=649-667, strand=1

target  5’ GUUGCCCAGGUUCACUGGG 3’
           .: .: ::::::::::.::
query   3’ UAUUGCGUCCAAGUGAUCC 5’
```

target 5' GCU-UUCCAGCUGGGCGACU 3'
           ::: :::::.: ::::::::
query   3' CGAGAAGGUUGUCCCGCUGA 5'

query=JS16_t0365221, target=FGSG_07435, score=4, range=340-358, strand=-1

target 5' ACUC-UCCAGCAGGGCGACC 3'
            ::: ::::.:::::::::
query   3' CGAGAAGGUUGUCCCGCUGA 5'

No results for JS16_t4032411
query=JS16_t3135835, target=FGSG_04706, score=3.5, range=531-551, strand=1

target 5' GUUUGGUUUUUUUGUUCUUAC 3'
            .::::: ::::::::::.::
query   3' GGAACCACAAAAACAAGAGUG 5'

query=JS16_t3135835, target=FGSG_11755, score=4, range=1151-1172, strand=1

target 5' ACUUGGUGUUUUUGUCUCUCAG 3'
            :::::::::::::: :::::
query   3' GGAACCACAAAAACA-AGAGUG 5'

query=JS16_t3135835, target=FGSG_02395, score=4, range=2205-2225, strand=1

target 5' CCCAGUUGUUUUUGUUUUCAC 3'
           :: : ::::::::::.::::
query   3' GGAACCACAAAAACAAGAGUG 5'

query=JS16_t3135835, target=FGSG_07696, score=4, range=380-400, strand=-1

target 5' CCGAGGUGUUUUUGUUGUCAC 3'
           :: :::::::::::: ::::
query   3' GGAACCACAAAAACAAGAGUG 5'

query=JS16_t0268855, target=FGSG_08719, score=3, range=6843-6860, strand=1

target 5' GGAGCGUGAACUGGCAGG 3'
            : :::::::::::::.:
query   3' ACACGCACUUGACCGUUC 5'

query=JS16_t0268855, target=FGSG_09184, score=3, range=1050-1066, strand=1

target 5' UGUG-GUGAACUGGCAGC 3'
           :::: :::::::::::.
query   3' ACACGCACUUGACCGUUC 5'

query=JS16_t0268855, target=FGSG_05413, score=3.5, range=313-331, strand=-1

target 5' UAUGUGUGAACUUGGCAAG 3'
           : ::.:::::: :::::::
query   3' ACACGCACUUG-ACCGUUC 5'

query=JS16_t0268855, target=FGSG_04201, score=4, range=2253-2270, strand=1

target 5' UGGGCGUGAAUUGGCAGA 3'
           :: :::::::.:::::.
query   3' ACACGCACUUGACCGUUC 5'

query=JS16_t0268855, target=FGSG_03651, score=4, range=738-755, strand=1

target 5' UGUGCGUGAUGUGGCAAG 3'
           ::::::::: :::::::
query   3' ACACGCACUUGACCGUUC 5'

query=JS16_t0268855, target=FGSG_01589, score=4, range=6059-6076, strand=1

target 5' UGGGAGUGGACUGGCAGG 3'
           :: : :::.:::::::.:
query   3' ACACGCACUUGACCGUUC 5'

query=JS16_t0268855, target=FGSG_11663, score=4, range=182-198, strand=1

target 5' UGUG-GCGAAUUGGCAAG 3'
           :::: : :::.:::::::
query   3' ACACGCACUUGACCGUUC 5'

query=JS16_t0268855, target=FGSG_01826, score=4, range=369-385, strand=1

target 5' UCUGCGUGGACU-GCAAG 3'
           : ::::::.::: :::::
query   3' ACACGCACUUGACCGUUC 5'

query=JS16_t0268855, target=FGSG_02968, score=4, range=216-232, strand=1

target 5' AUUGC-UGAACUGGCAAG 3'
             ::: ::::::::::::
query   3' ACACGCACUUGACCGUUC 5'

query=JS16_t0268855, target=FGSG_02020, score=4, range=1436-1452, strand=1

query=JS16_t1406565, target=FGSG_00277, score=4, range=51-68, strand=-1

target 5' AUCAC-CAGGUUCACCAGG 3'
           :: :: ::::::::: :::
query   3' UAUUGCGUCCAAGUGAUCC 5'

No results for JS16_t1406591
query=JS16_t3198840, target=FGSG_01371, score=2.5, range=1109-1129, strand=1

target 5' ACAUGGAUCUAGACGACAUCC 3'
            ::::. ::::::::::::::
query   3' CGUACUAAGAUCUGCUGUAGG 5'

query=JS16_t3198840, target=FGSG_01713, score=3, range=266-286, strand=1

target 5' GCAAGAAUCUGGACGACAUCC 3'
           ::: :: :::.::::::::::
query   3' CGUACUAAGAUCUGCUGUAGG 5'

query=JS16_t3198840, target=FGSG_06097, score=4, range=3614-3634, strand=1

target 5' UUGUGAUACUAGACGACAUCA 3'
            ..:::: :::::::::::
query   3' CGUACUAAGAUCUGCUGUAGG 5'

query=JS16_t3198840, target=FGSG_09961, score=4, range=961-980, strand=-1

target 5' GCA-GUUGCUAGACGACGUCC 3'
           ::: : : :::::::::.:::
query   3' CGUACUAAGAUCUGCUGUAGG 5'

No results for JS16_t0220455
No results for JS16_t1406609
query=JS16_t4095751, target=FGSG_11250, score=4, range=1016-1034, strand=1

target 5' UCUUGAUUUCCGCU-UGGUC 3'
           :::::::::::::: .:::
query   3' AGAACUAAAGGCGAUGCCAA 5'

query=JS16_t4095751, target=FGSG_10539, score=4, range=639-657, strand=1

target 5' UCUUG-UUGCCGCUACGGUC 3'
           ::::: :: :::::::::
query   3' AGAACUAAAGGCGAUGCCAA 5'

query=JS16_t4095751, target=FGSG_10095, score=4, range=4134-4154, strand=-1

target 5' UCUUGCUUUCCGCUCACGGUC 3'
           ::::: :::::::: :::::
query   3' AGAACUAAAGGCGA-UGCCAA 5'

query=JS16_t0337831, target=FGSG_11829, score=4, range=2316-2337, strand=1

target 5' UACAUCGAUCACAGAUGCUGGG 3'
             :: :::::::::::::::.:
query   3' CCGU-GCUAGUGUCUACGACUC 5'

No results for JS16_t2302897
query=JS16_t0396665, target=FGSG_13220, score=4, range=484-507, strand=1

target 5' GAGGUCACUUCGGGAGUGCCGCAU 3'
           ::.:: :::.::::::: ::::::
query   3' CUUCA-UGAGGCCCUCAAGGCGUA 5'

No results for JS16_t2302969
No results for JS16_t0162252
No results for JS16_t4095893
No results for JS16_t1406838
No results for JS16_t0476562
query=JS16_t1406883, target=FGSG_13705, score=4, range=164-185, strand=-1

target 5' UGCAUA-UUGUGAUGACCACGGU 3'
           ::: :: :::::::::::::.:
query   3' ACGCAUGAACACUACUGGUGUCU 5'

No results for JS16_t3199176
No results for JS16_t4096059
No results for JS16_t1406959
query=JS16_t4096063, target=FGSG_04853, score=2.5, range=638-655, strand=-1

target 5' UCCUCCCAGCGAACGUGU 3'
            ::: ::::::::::::.
query   3' UGGAUGGUCGCUUGCACG 5'

query=JS16_t4096063, target=FGSG_07657, score=3, range=614-631, strand=1

target 5' ACCUACCAGCGGACGGGC 3'
           :::::::::::.::: ::
query   3' UGGAUGGUCGCUUGCACG 5'

query=JS16_t4096063, target=FGSG_00375, score=3, range=1104-1121, strand=1

```
target  5' UGGGCGUGGA-UGGCAAG 3'
           :: :::::.: :::::::
query   3' ACACGCACUUGACCGUUC 5'

query=JS16_t0268855, target=FGSG_00614, score=4, range=1314-1331, strand=-1

target  5' UGUGGGUGGGCUGGCGAG 3'
           :::: :::..:::::.::
query   3' ACACGCACUUGACCGUUC 5'

query=JS16_t0268855, target=FGSG_06501, score=4, range=361-379, strand=-1

target  5' UGUAGCGUGAGUUGGCGAG 3'
           ::: ::::::..::::.::
query   3' ACA-CGCACUUGACCGUUC 5'

query=JS16_t0268855, target=FGSG_12276, score=4, range=2701-2718, strand=-1

target  5' UGGGGGUGGACUGGCAGG 3'
           :: : :::.:::::::.:
query   3' ACACGCACUUGACCGUUC 5'

query=JS16_t0268855, target=FGSG_11755, score=4, range=1877-1894, strand=-1

target  5' CGCGCGUGAACUGGCGAC 3'
            : ::::::::::::.:
query   3' ACACGCACUUGACCGUUC 5'

query=JS16_t0268855, target=FGSG_06804, score=4, range=554-570, strand=-1

target  5' UGUG-GUGAACUGGUGAA 3'
           :::: :::::::::..:
query   3' ACACGCACUUGACCGUUC 5'

query=JS16_t0268855, target=FGSG_10996, score=4, range=1579-1595, strand=-1

target  5' UGUGCGUCAA-UGGCAAG 3'
           ::::::: :: :::::::
query   3' ACACGCACUUGACCGUUC 5'

query=JS16_t0268855, target=FGSG_10842, score=4, range=1087-1104, strand=-1

target  5' GGCGAGUGAAUUGGCAAG 3'
            : : :::::.:::::::
query   3' ACACGCACUUGACCGUUC 5'

query=JS16_t0268855, target=FGSG_02428, score=4, range=47-64, strand=-1

target  5' GGAGAGUGAAUUGGCAAG 3'
            : : :::::.:::::::
query   3' ACACGCACUUGACCGUUC 5'

query=JS16_t0268855, target=FGSG_08795, score=4, range=2517-2533, strand=-1

target  5' UGU-CGAGAACUGGCGAG 3'
           ::: :: ::::::::.::
query   3' ACACGCACUUGACCGUUC 5'

query=JS16_t0268855, target=FGSG_05284, score=4, range=46-62, strand=-1

target  5' CGUGC-UGAACUGGCAAA 3'
            :::: :::::::::::
query   3' ACACGCACUUGACCGUUC 5'

query=JS16_t0268855, target=FGSG_06933, score=4, range=905-922, strand=-1

target  5' UGAGGGUGAAAUGGCAAG 3'
           :: : ::::: :::::::
query   3' ACACGCACUUGACCGUUC 5'

query=JS16_t0268855, target=FGSG_02898, score=4, range=659-675, strand=-1

target  5' UGAGAG-GAACUGGCAAG 3'
           :: : : :::::::::::
query   3' ACACGCACUUGACCGUUC 5'

No results for JS16_t3135887
No results for JS16_t0283288
No results for JS16_t3135958
No results for JS16_t2239950
No results for JS16_t3135982
No results for JS16_t4032749
No results for JS16_t2240139
query=JS16_t1343417, target=FGSG_08586, score=4, range=1362-1386, strand=-1

target  5' CUUGAGGGUAUACUUGACGGAGGAU 3'
           :::.::: .: :::::::.::::::
query   3' GAAUUCCAGU-UGAACUGUCUCCUA 5'

query=JS16_t2240200, target=FGSG_06975, score=4, range=170-190, strand=-1

target  5' UGUGCAAAUCUGCGUGGGCGA 3'

target  5' AUAUACCGGCGAACGUGU 3'
           :.  ::::.:::::::::.
query   3' UGGAUGGUCGCUUGCACG 5'

query=JS16_t4096063, target=FGSG_06016, score=3.5, range=4782-4799, strand=1

target  5' AUCUCCUAGUGAACGUGC 3'
           :.:: :.::.::::::::
query   3' UGGAUGGUCGCUUGCACG 5'

query=JS16_t4096063, target=FGSG_09346, score=3.5, range=1830-1847, strand=-1

target  5' AAAUACCAGCGAAUGUGU 3'
           :  :::::::::::.:::.
query   3' UGGAUGGUCGCUUGCACG 5'

query=JS16_t4096063, target=FGSG_13952, score=4, range=295-312, strand=1

target  5' ACUUGCCAGCAGACGUGC 3'
           ::.:.::::: .::::::
query   3' UGGAUGGUCGCUUGCACG 5'

query=JS16_t4096063, target=FGSG_00870, score=4, range=1281-1298, strand=1

target  5' AUCUACCAGCGAGCGUCU 3'
           :.::::::::::.::: .
query   3' UGGAUGGUCGCUUGCACG 5'

query=JS16_t4096063, target=FGSG_13670, score=4, range=797-813, strand=1

target  5' AUCU-UCAGCGAAUGUGU 3'
           :.:: .::::::::.:::.
query   3' UGGAUGGUCGCUUGCACG 5'

query=JS16_t4096063, target=FGSG_01146, score=4, range=387-404, strand=1

target  5' GGCUACCAGCGAACAUGU 3'
           . ::::::::::: ::.
query   3' UGGAUGGUCGCUUGCACG 5'

query=JS16_t4096063, target=FGSG_07493, score=4, range=1058-1075, strand=-1

target  5' CUCUGCCAGCGAAAGUGC 3'
            .::.::::::::: ::::
query   3' UGGAUGGUCGCUUGCACG 5'

query=JS16_t4096063, target=FGSG_01899, score=4, range=3488-3504, strand=-1

target  5' ACC-ACCAGCGGACGUAC 3'
           ::: :::::::.:::: :
query   3' UGGAUGGUCGCUUGCACG 5'

query=JS16_t4096063, target=FGSG_06324, score=4, range=992-1008, strand=-1

target  5' ACC-AGCAGCGAAUGUGC 3'
           ::: : :::::::.::::
query   3' UGGAUGGUCGCUUGCACG 5'

query=JS16_t4096070, target=FGSG_01015, score=3, range=468-486, strand=1

target  5' UCUUGGCUCUCUGGUUGCC 3'
             ::::::::::::::::
query   3' CAAACCGAGAGACCAACGC 5'

query=JS16_t4096070, target=FGSG_06238, score=4, range=2815-2833, strand=1

target  5' CUCUGUCUCUUUGGUUGCG 3'
            : :: ::::.:::::::::
query   3' CAAACCGAGAGACCAACGC 5'

query=JS16_t4096070, target=FGSG_02204, score=4, range=1478-1495, strand=1

target  5' GUUUGGCUC-CUGGUAGCG 3'
           ::::::::: ::::: :::
query   3' CAAACCGAGAGACCAACGC 5'

query=JS16_t4096070, target=FGSG_05855, score=4, range=831-849, strand=-1

target  5' UUUAGGCUCGCUGGUUGCG 3'
            :: ::::: :::::::::
query   3' CAAACCGAGAGACCAACGC 5'

query=JS16_t4096070, target=FGSG_03826, score=4, range=432-450, strand=-1

target  5' GUCGGGCUUUCUGGUUGCU 3'
           ::  ::::.:::::::::
query   3' CAAACCGAGAGACCAACGC 5'

query=JS16_t4096070, target=FGSG_04200, score=4, range=15-33, strand=-1

target  5' GUAUAUCUCUCUGGUUGUG 3'
```

```
            ::  :::::::::::::::
query   3’ CCAACUUUAGACGCACCCGCA 5’

query=JS16_t2240232, target=FGSG_06771, score=2, range=866-885, strand=1

target  5’ UGGAAGCUUACGCCCAUGGU 3’
           :::::::::::.:::::::
query   3’ ACCUUCGAAUGUGGGUACCC 5’

query=JS16_t2240232, target=FGSG_11268, score=3, range=7232-7251, strand=-1

target  5’ UGGCAGCUUGCACCCAUGGA 3’
           ::: :::::.:::::::::
query   3’ ACCUUCGAAUGUGGGUACCC 5’

query=JS16_t2240232, target=FGSG_08823, score=4, range=788-807, strand=1

target  5’ AGGAAGCUCAUACCCAUGGG 3’
            ::::::: :.:::::::::
query   3’ ACCUUCGAAUGUGGGUACCC 5’

query=JS16_t2240232, target=FGSG_13878, score=4, range=13522-13541, strand=1

target  5’ AGGAAACUUACGCCCAUGGA 3’
            :::: :::::.:::::::
query   3’ ACCUUCGAAUGUGGGUACCC 5’

query=JS16_t2240232, target=FgHV1gp1, score=4, range=472-491, strand=1

target  5’ GGGAAGAUUGCACCCAUGGA 3’
            ::::: ::.:::::::::
query   3’ ACCUUCGAAUGUGGGUACCC 5’

query=JS16_t2240232, target=FGSG_07640, score=4, range=554-572, strand=1

target  5’ UGGAA-CUUGCACCCAAGGG 3’
           ::::: :::.:::::: :::
query   3’ ACCUUCGAAUGUGGGUACCC 5’

query=JS16_t2240232, target=FGSG_00455, score=4, range=668-686, strand=1

target  5’ CAGAA-CUUACACCCAUGGA 3’
             ::: :::::::::::::
query   3’ ACCUUCGAAUGUGGGUACCC 5’

query=JS16_t2240232, target=FGSG_13450, score=4, range=57-75, strand=-1

target  5’ GGAAAGCUUACACCC-UGGG 3’
            : ::::::::::: ::::
query   3’ ACCUUCGAAUGUGGGUACCC 5’

No results for JS16_t4032856
No results for JS16_t4032858
query=JS16_t0228505, target=FGSG_13783, score=2.5, range=16022-16040, strand=1

target  5’ UUCUUCGUCAGGUUUCGGC 3’
           ::::.::: ::::::::::
query   3’ AAGAGGCACUCCAAAGCCG 5’

query=JS16_t0228505, target=FGSG_09310, score=4, range=53-71, strand=1

target  5’ CUCUCCGUGGGGUUUCUGC 3’
            ::::::::.:::::: ::
query   3’ AAGAGGCACUCCAAAGCCG 5’

query=JS16_t0228505, target=FGSG_10893, score=4, range=1298-1316, strand=-1

target  5’ UUCUCCAUGUGGUUUCGGC 3’
           :::::: :: :::::::::
query   3’ AAGAGGCACUCCAAAGCCG 5’

query=JS16_t1343571, target=FGSG_08919, score=3.5, range=154-173, strand=1

target  5’ ACUAUUGCGCGAAACGAGAA 3’
           ::: : .::::::::::.::
query   3’ UGA-ACUGCGCUUUGCUUUU 5’

query=JS16_t1343571, target=FGSG_10996, score=4, range=654-672, strand=1

target  5’ UGUUAACGCGAAACGAAGA 3’
             :: ::::::::::::.:
query   3’ UGAACUGCGCUUUGCUUUU 5’

query=JS16_t1343571, target=FGSG_02130, score=4, range=1083-1100, strand=1

target  5’ ACUUG-UGCGAAACGAUAA 3’
           ::::: .::::::::: ::
query   3’ UGAACUGCGCUUUGCUUUU 5’

query=JS16_t1343571, target=FGSG_02016, score=4, range=743-760, strand=1

target  5’ ACUUGUCGC-AAACGGAAA 3’
```

```
           :: :  :::::::::::.:
query   3’ CAAACCGAGAGACCAACGC 5’

query=JS16_t4096070, target=FGSG_06491, score=4, range=130-147, strand=-1

target  5’ AGUUGGCU-UCUGGUUGCG 3’
             :::::: ::::::::::
query   3’ CAAACCGAGAGACCAACGC 5’

No results for JS16_t1407049
query=JS16_t3199280, target=FGSG_04474, score=4, range=4235-4254, strand=-1

target  5’ AUAGUGUUCGGUGCACUGCA 3’
           :::..:::::::.::::: ::
query   3’ UAUUGCAAGCCGCGUGAAGU 5’

query=JS16_t3199280, target=FGSG_04661, score=4, range=327-347, strand=-1

target  5’ AUAGUAGUACGGCGCACUUCA 3’
           :::.. :: ::::::::::::
query   3’ UAUUG-CAAGCCGCGUGAAGU 5’

query=JS16_t3199280, target=FGSG_07758, score=4, range=740-758, strand=-1

target  5’ AUAAUG-UCGACGCACUUCG 3’
           ::::.: ::: ::::::::.
query   3’ UAUUGCAAGCCGCGUGAAGU 5’

query=JS16_t3199291, target=FGSG_06091, score=3, range=153-171, strand=-1

target  5’ AGCAUCAGGUGAUUUUGGG 3’
           : ::::::::::..:::::
query   3’ UGGUAGUCCACUGGAACCC 5’

query=JS16_t3199291, target=FGSG_02969, score=3, range=172-189, strand=-1

target  5’ ACC-UGAGGUGACCUUGGA 3’
           ::: : ::::::::::::
query   3’ UGGUAGUCCACUGGAACCC 5’

query=JS16_t3199291, target=FGSG_08173, score=3.5, range=480-498, strand=1

target  5’ AUCAUCAGGUGAGCUUGGU 3’
           :.:::::::::: :::::
query   3’ UGGUAGUCCACUGGAACCC 5’

query=JS16_t3199291, target=FGSG_09029, score=3.5, range=883-901, strand=-1

target  5’ ACCAGUAGUUGACCUUGGG 3’
           :::: .:: ::::::::::
query   3’ UGGUAGUCCACUGGAACCC 5’

query=JS16_t3199291, target=FGSG_06793, score=3.5, range=452-470, strand=-1

target  5’ AACGUGAGGUGACCUUGGC 3’
           : :.: ::::::::::::
query   3’ UGGUAGUCCACUGGAACCC 5’

query=JS16_t3199291, target=FGSG_04427, score=3.5, range=1817-1836, strand=-1

target  5’ CUCAACAAGGUGACCUUGGG 3’
            .:: : :::::::::::::
query   3’ UGGUAG-UCCACUGGAACCC 5’

query=JS16_t3199291, target=FGSG_01634, score=4, range=1456-1474, strand=1

target  5’ CCUCUUAGGUGGCCUUGGG 3’
            :.  :.:::::.:::::::
query   3’ UGGUAGUCCACUGGAACCC 5’

query=JS16_t3199291, target=FGSG_11287, score=4, range=1542-1561, strand=1

target  5’ ACCAUCAGGUGAAGCUUGGG 3’
           ::::::::::: : ::::::
query   3’ UGGUAGUCCAC-UGGAACCC 5’

query=JS16_t3199291, target=FGSG_06326, score=4, range=4497-4515, strand=1

target  5’ ACGCUCAGGUGGCCUUGGU 3’
           ::  :::::::.::::::
query   3’ UGGUAGUCCACUGGAACCC 5’

query=JS16_t3199291, target=FGSG_12809, score=4, range=2131-2149, strand=-1

target  5’ ACUUUUGGGUGACCUUGGC 3’
           ::.  :..::::::::::
query   3’ UGGUAGUCCACUGGAACCC 5’

query=JS16_t3199291, target=FGSG_09125, score=4, range=110-128, strand=-1

target  5’ GCUACCAUGUGACCUUGGG 3’
           .:.: :: :::::::::::
```

```
          ::::: ::: :::::.:::
query   3' UGAACUGCGCUUUGCUUUU 5'

query=JS16_t1343571, target=FGSG_07116, score=4, range=297-315, strand=1

target  5' UGGUGUCGCGAAACGAAAA 3'
             :: :::::::::::::
query   3' UGAACUGCGCUUUGCUUUU 5'

query=JS16_t1343571, target=FGSG_01564, score=4, range=376-393, strand=1

target  5' GCUUGUCGCGAAA-GAAAG 3'
           .:::: ::::::: ::::.
query   3' UGAACUGCGCUUUGCUUUU 5'

query=JS16_t1343571, target=FGSG_10917, score=4, range=938-956, strand=1

target  5' ACUACACGCAAAACGAAAA 3'
           :::  :::: :::::::::
query   3' UGAACUGCGCUUUGCUUUU 5'

query=JS16_t1343571, target=FGSG_12623, score=4, range=440-457, strand=1

target  5' ACUUUAU-CGAAACGAAAA 3'
           :::: :. :::::::::::
query   3' UGAACUGCGCUUUGCUUUU 5'

No results for JS16_t4032904
query=JS16_t4032954, target=FGSG_10893, score=4, range=139-157, strand=-1

target  5' GGGAAGCUGCGUUAUAAGA 3'
           : ::: ::::::::: ::
query   3' CACUUGGACGCAAUAUACU 5'

No results for JS16_t3136385
No results for JS16_t4032977
No results for JS16_t3136407
No results for JS16_t0240450
No results for JS16_t0305937
No results for JS16_t1343733
No results for JS16_t3136471
query=JS16_t0228510, target=FGSG_05644, score=3, range=550-569, strand=1

target  5' GCAUGGAUCUUGUACGGCAG 3'
           . :::: :::::::::::.
query   3' UCUACC-AGAACAUGCCGUU 5'

query=JS16_t0228510, target=FGSG_11555, score=3.5, range=867-884, strand=1

target  5' AGG-GCUCUUGUAUGGCAA 3'
           ::. : :::::::.:::::
query   3' UCUACCAGAACAUGCCGUU 5'

query=JS16_t0228510, target=FGSG_06676, score=3.5, range=1151-1168, strand=-1

target  5' CGAUGGUCUUGUA-GGCAG 3'
            :::::::::::: ::::.
query   3' UCUACCAGAACAUGCCGUU 5'

query=JS16_t0228510, target=FGSG_04450, score=3.5, range=485-502, strand=-1

target  5' CGAUGGUCUUG-ACGGCAG 3'
            :::::::::: ::::::.
query   3' UCUACCAGAACAUGCCGUU 5'

query=JS16_t0228510, target=FGSG_05911, score=4, range=551-569, strand=1

target  5' AGAGGGACUUGUACGGCGA 3'
           ::: :: ::::::::::.:
query   3' UCUACCAGAACAUGCCGUU 5'

query=JS16_t0228510, target=FGSG_00024, score=4, range=318-336, strand=1

target  5' AGAUGUUCUUGUACGACAC 3'
           ::::: ::::::::: ::
query   3' UCUACCAGAACAUGCCGUU 5'

query=JS16_t0228510, target=FGSG_05764, score=4, range=1101-1119, strand=1

target  5' ACAUGCUCUUGGACGGCAA 3'
           : ::: ::::: :::::::
query   3' UCUACCAGAACAUGCCGUU 5'

query=JS16_t0228510, target=FGSG_00565, score=4, range=1107-1124, strand=1

target  5' CGAU-GUCUUGGACGGCAA 3'
            ::: :::::: :::::::
query   3' UCUACCAGAACAUGCCGUU 5'

query=JS16_t0228510, target=FGSG_05066, score=4, range=1414-1432, strand=-1

target  5' AGAUGGACUUGUACGUCAA 3'
query   3' UGGUAGUCCACUGGAACCC 5'

query=JS16_t3199291, target=FGSG_05320, score=4, range=29-47, strand=-1

target  5' AGCAUGGGGUGGCCUUGGG 3'
           : ::: .::::.:::::::
query   3' UGGUAGUCCACUGGAACCC 5'

query=JS16_t3199291, target=FGSG_07553, score=4, range=1581-1599, strand=-1

target  5' GUCAUGAGGUAACCUUGGG 3'
           ..::: :::: ::::::::
query   3' UGGUAGUCCACUGGAACCC 5'

query=JS16_t3199291, target=FGSG_04749, score=4, range=890-909, strand=-1

target  5' UCCAGCAAGGUGACCUUGGU 3'
            ::: : :::::::::::
query   3' UGGUAG-UCCACUGGAACCC 5'

query=JS16_t3199291, target=FGSG_00866, score=4, range=727-744, strand=-1

target  5' ACUGUCA-GUGACCUUGGU 3'
           ::..::: ::::::::::
query   3' UGGUAGUCCACUGGAACCC 5'

No results for JS16_t2303312
query=JS16_t1407104, target=FGSG_04051, score=4, range=53-72, strand=1

target  5' CAGAGAACCUUAUUGAUGCC 3'
           :: ::::::.::: ::::::
query   3' GUGUCUUGGGAUAUCUACGG 5'

query=JS16_t0182896, target=FGSG_06565, score=4, range=3357-3377, strand=1

target  5' AGUUGAAG-AACGUUUCCAGGA 3'
           :::..::: ::::::.:::.::
query   3' UCAGUUUCGUUGCAAGGGUUCU 5'

No results for JS16_t2303372
No results for JS16_t0476596
No results for JS16_t0269901
No results for JS16_t3199442
No results for JS16_t1407278
No results for JS16_t0803756
No results for JS16_t4096391
No results for JS16_t4096409
query=JS16_t0803761, target=FGSG_06555, score=2.5, range=470-490, strand=1

target  5' AGAGUGGUGGUUUGGAUCCGA 3'
           : :::::.:::.:::::::::
query   3' UAUCACCGCCAGACCUAGGCU 5'

query=JS16_t0803761, target=FGSG_12029, score=3, range=3007-3027, strand=1

target  5' AUUCUGGCGGUUUGGAUCCGA 3'
           ::  :::::::.:::::::::
query   3' UAUCACCGCCAGACCUAGGCU 5'

query=JS16_t0803761, target=FGSG_13648, score=3.5, range=291-311, strand=-1

target  5' UUGGUGGCGGUCUGGUUCCGA 3'
            :.:::::::::::: :::::
query   3' UAUCACCGCCAGACCUAGGCU 5'

query=JS16_t0803761, target=FGSG_05581, score=4, range=544-563, strand=-1

target  5' CAAGUCG-GGUCUGGAUCCGA 3'
             ::: : :::::::::::::
query   3' UAUCACCGCCAGACCUAGGCU 5'

No results for JS16_t0284266
No results for JS16_t3199547
No results for JS16_t3199558
No results for JS16_t2303590
No results for JS16_t3199568
No results for JS16_t1407395
query=JS16_t0337849, target=FGSG_08201, score=2.5, range=1113-1131, strand=-1

target  5' UCUUGUGUUCUGCUGAGCC 3'
           :::. :::::.::::::::
query   3' AGAG-ACAAGGCGACUCGG 5'

query=JS16_t0337849, target=FGSG_00974, score=3, range=229-245, strand=1

target  5' CCUC-GUUCCGCUGAGCA 3'
            ::: :::::::::::::
query   3' AGAGACAAGGCGACUCGG 5'

query=JS16_t0337849, target=FGSG_01887, score=3, range=450-467, strand=-1

target  5' UCGAUGUUCUGCUGAGCC 3'
```

```
           :::::: :::::::: :::
query   3’ UCUACCAGAACAUGCCGUU 5’

query=JS16_t0228510, target=FGSG_04435, score=4, range=1363-1380, strand=-1

target  5’ AGGUGGUCUUGUA-GGUAG 3’
           ::.:::::::::: ::.:.
query   3’ UCUACCAGAACAUGCCGUU 5’

query=JS16_t0228510, target=FGSG_12224, score=4, range=1421-1439, strand=-1

target  5’ AGCUGGUCUUGUGCUGCAA 3’
           :: :::::::::.: ::::
query   3’ UCUACCAGAACAUGCCGUU 5’

query=JS16_t0228510, target=FGSG_10248, score=4, range=2265-2283, strand=-1

target  5’ GGGAGAUCUUGUACGGCGA 3’
           .:. : :::::::::::.:
query   3’ UCUACCAGAACAUGCCGUU 5’

query=JS16_t0228510, target=FGSG_09200, score=4, range=929-947, strand=-1

target  5’ AGAACGUCUUGUGCGGCAU 3’
           :::  :::::::.:::::
query   3’ UCUACCAGAACAUGCCGUU 5’

query=JS16_t0228510, target=FGSG_06717, score=4, range=991-1008, strand=-1

target  5’ AGGU-GUCUUGUACGGCUG 3’
           ::.: :::::::::::: .
query   3’ UCUACCAGAACAUGCCGUU 5’

query=JS16_t0228510, target=FGSG_04419, score=4, range=803-820, strand=-1

target  5’ AGAUAAUCUUGUAC-GCAA 3’
           ::::  :::::::: ::::
query   3’ UCUACCAGAACAUGCCGUU 5’

query=JS16_t1343769, target=FGSG_07264, score=4, range=402-420, strand=1

target  5’ CGGCAUUGGAACU-GUCUAU 3’
           ::: ::::::::. ::::::
query   3’ GCCAUAACCUUGGUCAGAUA 5’

query=JS16_t1343769, target=FGSG_08115, score=4, range=1227-1247, strand=-1

target  5’ CGGUAGUGGAACCAGUCGUAC 3’
           ::::: ::::::::::: ::
query   3’ GCCAUAACCUUGGUCAG-AUA 5’

query=JS16_t1343769, target=FGSG_01587, score=4, range=864-883, strand=-1

target  5’ CAGCAUUGGAACCAGUCUGG 3’
           : : ::::::::::::::.
query   3’ GCCAUAACCUUGGUCAGAUA 5’

query=JS16_t1343769, target=FGSG_07097, score=4, range=146-164, strand=-1

target  5’ CGGUUUUGGAACCAGU-UGU 3’
           :::: ::::::::::: :.:
query   3’ GCCAUAACCUUGGUCAGAUA 5’

query=JS16_t1343769, target=FGSG_02011, score=4, range=914-932, strand=-1

target  5’ CGGUCAUGGAACCAGUC-AU 3’
           ::::  ::::::::::: ::
query   3’ GCCAUAACCUUGGUCAGAUA 5’

No results for JS16_t0305940
query=JS16_t0283296, target=FGSG_04949, score=2.5, range=1735-1751, strand=1

target  5’ AAUA-UGUCAAUCCCUCU 3’
           ::.: ::::::::::::
query   3’ UUGUAACAGUUAGGGAGC 5’

query=JS16_t0283296, target=FGSG_03103, score=3, range=70-87, strand=1

target  5’ AACAUUGUCGAUCCCGCG 3’
           :::::::::.::::: ::
query   3’ UUGUAACAGUUAGGGAGC 5’

query=JS16_t0283296, target=FGSG_11306, score=3, range=375-393, strand=-1

target  5’ GGCAUCUGUCAAUCCCUUG 3’
           ..::: :::::::::::.:
query   3’ UUGUA-ACAGUUAGGGAGC 5’

query=JS16_t0283296, target=FGSG_11489, score=3.5, range=736-754, strand=1

target  5’ GAUGCUUGUCAAUCCCUUG 3’
           .:..  ::::::::::::.:
```

```
           ::  :::::.::::::::
query   3’ AGAGACAAGGCGACUCGG 5’

query=JS16_t0337849, target=FGSG_04020, score=3.5, range=149-167, strand=1

target  5’ UUUCUGUUACCGUUGAGCC 3’
           :.::::::: :::.::::::
query   3’ AGAGACAA-GGCGACUCGG 5’

query=JS16_t0337849, target=FGSG_10060, score=3.5, range=1555-1572, strand=-1

target  5’ UUGCAGUUCUGCUGAGCC 3’
           :.  : ::::.::::::::
query   3’ AGAGACAAGGCGACUCGG 5’

query=JS16_t0337849, target=FGSG_08920, score=4, range=1068-1085, strand=1

target  5’ GUUCUGUUCCGAUGAGCU 3’
            .::::::::: :::::.
query   3’ AGAGACAAGGCGACUCGG 5’

query=JS16_t0337849, target=FGSG_07258, score=4, range=3844-3861, strand=1

target  5’ GACCUGUUCCGCUGGGCC 3’
               :::::::::::.:::
query   3’ AGAGACAAGGCGACUCGG 5’

query=JS16_t0337849, target=FGSG_12137, score=4, range=694-710, strand=1

target  5’ UUUCUGUUCUGCUG-GCU 3’
           :.:::::::.:::: ::.
query   3’ AGAGACAAGGCGACUCGG 5’

query=JS16_t0337849, target=FGSG_07020, score=4, range=1949-1967, strand=1

target  5’ UCUCUAGUUCUUCUGAGCC 3’
           ::::: ::::. :::::::
query   3’ AGAGA-CAAGGCGACUCGG 5’

query=JS16_t0337849, target=FGSG_05252, score=4, range=1675-1693, strand=1

target  5’ UCUCUCGUUUCGCUGAACC 3’
           ::::: :::.::::::: ::
query   3’ AGAGA-CAAGGCGACUCGG 5’

query=JS16_t0337849, target=FGSG_10819, score=4, range=1323-1340, strand=1

target  5’ UUUCCGUGCCGCUGAGCU 3’
           :.:: :: :::::::::.
query   3’ AGAGACAAGGCGACUCGG 5’

query=JS16_t0337849, target=FGSG_12298, score=4, range=434-451, strand=1

target  5’ UUUGUGUUCCUCUGAGCU 3’
           :.: :::::: ::::::.
query   3’ AGAGACAAGGCGACUCGG 5’

query=JS16_t0337849, target=FGSG_02004, score=4, range=1537-1553, strand=1

target  5’ UCGC-GCUCCGCUGAGCC 3’
           :: : : ::::::::::
query   3’ AGAGACAAGGCGACUCGG 5’

query=JS16_t0337849, target=FGSG_13982, score=4, range=793-809, strand=1

target  5’ UCCCUGUU-CGCUGGGCC 3’
           :: ::::: :::::.:::
query   3’ AGAGACAAGGCGACUCGG 5’

query=JS16_t0337849, target=FGSG_11072, score=4, range=104-121, strand=-1

target  5’ UCUCUGUUUCGCUGGUCC 3’
           ::::::::.:::::.  ::
query   3’ AGAGACAAGGCGACUCGG 5’

query=JS16_t0337849, target=FGSG_02482, score=4, range=2313-2330, strand=-1

target  5’ UUUCUCUUCUGCUGAGCU 3’
           :.::: :::.:::::::.
query   3’ AGAGACAAGGCGACUCGG 5’

query=JS16_t0337849, target=FGSG_06914, score=4, range=2230-2247, strand=-1

target  5’ UCGCUGUUUUGCUGAGUC 3’
           :: :::::..::::::.:
query   3’ AGAGACAAGGCGACUCGG 5’

query=JS16_t0337849, target=FGSG_13919, score=4, range=720-738, strand=-1

target  5’ UUUUUGUUUCCGCUGAGUC 3’
           :.:.:: ::::::::::.:
query   3’ AGAGAC-AAGGCGACUCGG 5’
```

```
query   3’ UUGU-AACAGUUAGGGAGC 5’

query=JS16_t0283296, target=FGSG_09615, score=3.5, range=1462-1478, strand=1

target  5’ GACAUUGUCGA-CCCUCG 3’
           .::::::::.: ::::::
query   3’ UUGUAACAGUUAGGGAGC 5’

query=JS16_t0283296, target=FGSG_09915, score=3.5, range=739-756, strand=1

target  5’ AGCCUUGUCAUUCCCUCG 3’
           :.: :::::: :::::::
query   3’ UUGUAACAGUUAGGGAGC 5’

query=JS16_t0283296, target=FGSG_01513, score=3.5, range=3-20, strand=1

target  5’ GCCAGUGUCAAUCCCUCU 3’
           . :: ::::::::::::
query   3’ UUGUAACAGUUAGGGAGC 5’

query=JS16_t0283296, target=FGSG_05135, score=3.5, range=2505-2523, strand=-1

target  5’ GACAUUGUCAAUUCCAUCG 3’
           .:::::::::::.:: :::
query   3’ UUGUAACAGUUAGGG-AGC 5’

query=JS16_t0283296, target=FGSG_08795, score=3.5, range=1006-1023, strand=-1

target  5’ GGGGUUGUCAAUUCCUCG 3’
           .. .::::::::.:::::
query   3’ UUGUAACAGUUAGGGAGC 5’

query=JS16_t0283296, target=FGSG_08534, score=3.5, range=865-882, strand=-1

target  5’ AAUAAUGUCAAUCUCUCC 3’
           ::.: ::::::::.:::
query   3’ UUGUAACAGUUAGGGAGC 5’

query=JS16_t0283296, target=FGSG_04414, score=3.5, range=745-763, strand=-1

target  5’ AAUAGUUGGCAAUCCCUCG 3’
           ::.: ::: ::::::::::
query   3’ UUGU-AACAGUUAGGGAGC 5’

query=JS16_t0283296, target=FGSG_04082, score=3.5, range=36-54, strand=-1

target  5’ GCCAUCUGUCGAUCCCUCG 3’
           . ::: ::::.::::::::
query   3’ UUGUA-ACAGUUAGGGAGC 5’

query=JS16_t0283296, target=FGSG_04842, score=4, range=580-597, strand=1

target  5’ AACAUUGUCAGUAUCUCG 3’
           ::::::::::.: .::::
query   3’ UUGUAACAGUUAGGGAGC 5’

query=JS16_t0283296, target=FGSG_10874, score=4, range=577-594, strand=1

target  5’ GGCACUGUCAGUCCUUCG 3’
           ..:: :::::.:::.:::
query   3’ UUGUAACAGUUAGGGAGC 5’

query=JS16_t0283296, target=FGSG_02523, score=4, range=196-214, strand=1

target  5’ AACAUUGUCAACUCCUUCC 3’
           ::::::::::: :::.::
query   3’ UUGUAACAGUU-AGGGAGC 5’

query=JS16_t0283296, target=FGSG_09307, score=4, range=740-757, strand=1

target  5’ AGCGUUUUCAAUCCCUCA 3’
           :.:.:: ::::::::::
query   3’ UUGUAACAGUUAGGGAGC 5’

query=JS16_t0283296, target=FGSG_12742, score=4, range=421-438, strand=1

target  5’ AAUGAUGUCAAUCCUUCU 3’
           ::.. :::::::::.::
query   3’ UUGUAACAGUUAGGGAGC 5’

query=JS16_t0283296, target=FGSG_01280, score=4, range=1206-1223, strand=1

target  5’ AUUGUUGUCAUUCCCUCG 3’
           : ..:::::: :::::::
query   3’ UUGUAACAGUUAGGGAGC 5’

query=JS16_t0283296, target=FGSG_06036, score=4, range=202-219, strand=1

target  5’ AUUGUUUUCAAUCCCUCG 3’
           : ..:: :::::::::::
query   3’ UUGUAACAGUUAGGGAGC 5’

query=JS16_t0337849, target=FGSG_06369, score=4, range=837-854, strand=-1

target  5’ UUUUCGUUUUGCUGAGCC 3’
           :.:. :::..::::::::
query   3’ AGAGACAAGGCGACUCGG 5’

query=JS16_t0337849, target=FGSG_06308, score=4, range=264-281, strand=-1

target  5’ UCUCUGUACUGCUGAGCA 3’
           ::::::: :.:::::::
query   3’ AGAGACAAGGCGACUCGG 5’

query=JS16_t0337849, target=FGSG_12530, score=4, range=66-83, strand=-1

target  5’ UUGCUGUUCAGCUGAGCU 3’
           :. :::::: :::::::.
query   3’ AGAGACAAGGCGACUCGG 5’

query=JS16_t0337849, target=FGSG_06925, score=4, range=1665-1681, strand=-1

target  5’ UCU-UGUCCUGCUGAGCC 3’
           ::: ::: :.::::::::
query   3’ AGAGACAAGGCGACUCGG 5’

query=JS16_t0337849, target=FGSG_06771, score=4, range=79-95, strand=-1

target  5’ UCU-UGUUCGGUUGAGCC 3’
           ::: ::::: :.::::::
query   3’ AGAGACAAGGCGACUCGG 5’

query=JS16_t0337849, target=FGSG_09134, score=4, range=225-241, strand=-1

target  5’ UCUC-GGUCCGCUGAGUC 3’
           :::: : :::::::::.:
query   3’ AGAGACAAGGCGACUCGG 5’

query=JS16_t0337849, target=FGSG_13483, score=4, range=1047-1063, strand=-1

target  5’ CGUCUGUUCCGCUG-GCC 3’
             :::::::::::: :::
query   3’ AGAGACAAGGCGACUCGG 5’

query=JS16_t0337849, target=FGSG_07369, score=4, range=262-278, strand=-1

target  5’ GCUC-GGUCCGCUGAGCC 3’
            ::: : :::::::::::
query   3’ AGAGACAAGGCGACUCGG 5’

query=JS16_t1407408, target=FGSG_06225, score=3.5, range=249-266, strand=1

target  5’ AACCGUCAGUAGUGCAUU 3’
           ::: ..:::::::::::
query   3’ UUGCUGGUCAUCACGUAC 5’

query=JS16_t1407408, target=FGSG_12550, score=4, range=796-814, strand=1

target  5’ AGUGGACCAGUAGUGCGUU 3’
           :.. ::::::::::::.:
query   3’ UUG-CUGGUCAUCACGUAC 5’

query=JS16_t1407408, target=FGSG_06438, score=4, range=71-88, strand=-1

target  5’ AAUCGUCAGUAGUGCAUC 3’
           ::. ..:::::::::::
query   3’ UUGCUGGUCAUCACGUAC 5’

query=JS16_t1407408, target=FGSG_10418, score=4, range=699-716, strand=-1

target  5’ AGCUGCCAGUAGUUCAUG 3’
           :.: .:::::::: ::::
query   3’ UUGCUGGUCAUCACGUAC 5’

query=JS16_t1407408, target=FGSG_12837, score=4, range=445-463, strand=-1

target  5’ GAUGACCCAGGAGUGCAUG 3’
           .:.:: :::: ::::::::
query   3’ UUGCU-GGUCAUCACGUAC 5’

query=JS16_t1407408, target=FGSG_10548, score=4, range=4404-4420, strand=-1

target  5’ GAUGACCAGUAGUG-AUA 3’
           .:.:::::::::: ::
query   3’ UUGCUGGUCAUCACGUAC 5’

query=JS16_t1407408, target=FGSG_03947, score=4, range=283-300, strand=-1

target  5’ CAAGACCAGUUGUGCAUG 3’
            : ::::::: :::::::
query   3’ UUGCUGGUCAUCACGUAC 5’

query=JS16_t1407408, target=FGSG_03811, score=4, range=10-26, strand=-1
```

```
query=JS16_t0283296, target=FGSG_04347, score=4, range=401-419, strand=1

target  5’ GAUAUGAGUCAAUCCCUCG 3’
           .:.::  ::::::::::::
query   3’ UUGUA-ACAGUUAGGGAGC 5’

query=JS16_t0283296, target=FGSG_11852, score=4, range=1953-1969, strand=1

target  5’ AAC-UUUUCAGUCCCUCG 3’
           ::: :: :::.:::::::
query   3’ UUGUAACAGUUAGGGAGC 5’

query=JS16_t0283296, target=FGSG_05798, score=4, range=1217-1233, strand=1

target  5’ AAC-UUGUCAAACCUUCG 3’
           ::: ::::::: ::.:::
query   3’ UUGUAACAGUUAGGGAGC 5’

query=JS16_t0283296, target=FGSG_05383, score=4, range=282-298, strand=1

target  5’ AACA-GGUCAAUCCUUCG 3’
           ::::  ::::::::.:::
query   3’ UUGUAACAGUUAGGGAGC 5’

query=JS16_t0283296, target=FGSG_10611, score=4, range=1143-1159, strand=1

target  5’ GGC-UGGUCAAUCCCUCG 3’
           ..: : ::::::::::::
query   3’ UUGUAACAGUUAGGGAGC 5’

query=JS16_t0283296, target=FGSG_09862, score=4, range=1366-1382, strand=1

target  5’ AACUCUGUCAAU-CCUCG 3’
           :::  ::::::: :::::
query   3’ UUGUAACAGUUAGGGAGC 5’

query=JS16_t0283296, target=FGSG_03025, score=4, range=295-313, strand=1

target  5’ AAAAUAGUCAAUGCCCUCG 3’
           :: :: :::::: ::::::
query   3’ UUGUAACAGUUA-GGGAGC 5’

query=JS16_t0283296, target=FGSG_01314, score=4, range=847-863, strand=1

target  5’ GAUAUUGUC-AUCUCUCG 3’
           .:.:::::: :::.::::
query   3’ UUGUAACAGUUAGGGAGC 5’

query=JS16_t0283296, target=FGSG_02060, score=4, range=97-114, strand=-1

target  5’ AACAUCGUCAAUUCCUUG 3’
           ::::: ::::::.:::.:
query   3’ UUGUAACAGUUAGGGAGC 5’

query=JS16_t0283296, target=FGSG_00938, score=4, range=678-695, strand=-1

target  5’ AGCGUUGUCGAACCCUCG 3’
           :.:.:::::.: ::::::
query   3’ UUGUAACAGUUAGGGAGC 5’

query=JS16_t0283296, target=FGSG_07614, score=4, range=504-521, strand=-1

target  5’ AACAUUGUCAAUCUAUCU 3’
           :::::::::::::. ::
query   3’ UUGUAACAGUUAGGGAGC 5’

query=JS16_t0283296, target=FGSG_03927, score=4, range=822-840, strand=-1

target  5’ AACAUUGUCAAUCGUCUCC 3’
           ::::::::::::: .:::
query   3’ UUGUAACAGUUAG-GGAGC 5’

query=JS16_t0283296, target=FGSG_11085, score=4, range=304-322, strand=-1

target  5’ AGUACUUGUCAAUCUCUCU 3’
           :..: :::::::::.:::
query   3’ UUGU-AACAGUUAGGGAGC 5’

query=JS16_t0283296, target=FGSG_04930, score=4, range=1698-1715, strand=-1

target  5’ GGCCUUGUCAACCCCUCG 3’
           ..: ::::::: ::::::
query   3’ UUGUAACAGUUAGGGAGC 5’

query=JS16_t0283296, target=FGSG_03906, score=4, range=1240-1257, strand=-1

target  5’ GCCGUUGUCAAUCCAUCG 3’
           .  :.::::::::: :::
query   3’ UUGUAACAGUUAGGGAGC 5’

query=JS16_t0283296, target=FGSG_08134, score=4, range=537-555, strand=-1

target  5’ AACG-CCAGUAGUGUUUG 3’
           :::: :::::::::. ::
query   3’ UUGCUGGUCAUCACGUAC 5’

query=JS16_t1407408, target=FGSG_11067, score=4, range=2221-2237, strand=-1

target  5’ AGAGG-CAGUAGUGCAUG 3’
           :. :.  ::::::::::::
query   3’ UUGCUGGUCAUCACGUAC 5’

No results for JS16_t3199629
query=JS16_t3199673, target=FGSG_03091, score=2.5, range=788-807, strand=1

target  5’ AAGUUCGAACUGAGAUUGGG 3’
           :::::::::::::: ::::.
query   3’ UUCAAGCUUGACUC-AACCU 5’

query=JS16_t3199673, target=FGSG_00677, score=4, range=757-776, strand=1

target  5’ AAGUACGAGAUUGAGUUGGA 3’
           :::: ::: :.:::::::::
query   3’ UUCAAGCU-UGACUCAACCU 5’

query=JS16_t3199673, target=FGSG_07574, score=4, range=13-30, strand=1

target  5’ AAGUUCGGACUGA-UUGGC 3’
           :::::::.::::: ::::
query   3’ UUCAAGCUUGACUCAACCU 5’

query=JS16_t3199673, target=FGSG_11459, score=4, range=409-426, strand=1

target  5’ AUUUUCGAACUG-GUUGGA 3’
           :  ::::::::: ::::::
query   3’ UUCAAGCUUGACUCAACCU 5’

query=JS16_t3199673, target=FGSG_13711, score=4, range=160-179, strand=-1

target  5’ AGGUUUGGACUGAUGUUGGA 3’
           :.:::.:.::::: ::::::
query   3’ UUCAAGCUUGACU-CAACCU 5’

query=JS16_t3199673, target=FGSG_09435, score=4, range=526-544, strand=-1

target  5’ GAGGCUGGACUGAGUUGGA 3’
           .::  .:.:::::::::::
query   3’ UUCAAGCUUGACUCAACCU 5’

query=JS16_t3199673, target=FGSG_00324, score=4, range=4389-4407, strand=-1

target  5’ CUGCUCGAACUGAGUUGGU 3’
             : ::::::::::::::
query   3’ UUCAAGCUUGACUCAACCU 5’

query=JS16_t3199673, target=FGSG_13429, score=4, range=852-869, strand=-1

target  5’ ACGUUCGAACU-AGUUGGC 3’
           : ::::::::: ::::::
query   3’ UUCAAGCUUGACUCAACCU 5’

query=JS16_t0241448, target=FGSG_04731, score=3.5, range=280-302, strand=-1

target  5’ UCCCAGGC-AUGGACACCAAGAAG 3’
             ::::.: :::::::::::::::
query   3’ UAGGUCUGCUACCUGUGGUUCUUC 5’

No results for JS16_t1407527
No results for JS16_t1407530
query=JS16_t2303751, target=FGSG_00958, score=3, range=853-870, strand=1

target  5’ GAC-UCUAUCGCUGCUAUC 3’
           ::: ::::::::..:::::
query   3’ CUGAAGAUAGCGGUGAUAG 5’

query=JS16_t2303751, target=FGSG_05238, score=3.5, range=942-960, strand=1

target  5’ GGCAGCUGUCGCCACUAUC 3’
           :.:  ::.:::::::::::
query   3’ CUGAAGAUAGCGGUGAUAG 5’

query=JS16_t2303751, target=FGSG_06787, score=3.5, range=1470-1488, strand=1

target  5’ UACAGCUAUCGCCACUAUU 3’
            ::  :::::::::::::.
query   3’ CUGAAGAUAGCGGUGAUAG 5’

query=JS16_t2303751, target=FGSG_06896, score=4, range=762-780, strand=1

target  5’ GGUUCCUGUCGCCAUUAUC 3’
           :..: ::.::::::.::::
query   3’ CUGAAGAUAGCGGUGAUAG 5’
```

```
target  5’ AGUAUGUGUCAAUCCCUGG 3’
           :..:: ::::::::::: :
query   3’ UUGUA-ACAGUUAGGGAGC 5’

query=JS16_t0283296, target=FGSG_00418, score=4, range=2542-2559, strand=-1

target  5’ UUCCUUGUCAAUCCCUUG 3’
             : ::::::::::::.:
query   3’ UUGUAACAGUUAGGGAGC 5’

query=JS16_t0283296, target=FGSG_03978, score=4, range=940-956, strand=-1

target  5’ AAC-UUGUCGAUUCCUCC 3’
           ::: :::::.::.::::
query   3’ UUGUAACAGUUAGGGAGC 5’

query=JS16_t0283296, target=FGSG_01619, score=4, range=25-42, strand=-1

target  5’ ACCAUUGUCAUUCCCUCC 3’
           : :::::::: ::::::
query   3’ UUGUAACAGUUAGGGAGC 5’

query=JS16_t0283296, target=FGSG_07350, score=4, range=1425-1441, strand=-1

target  5’ GAUA-UGUCAAUUCCUCA 3’
           .:.: :::::::.::::
query   3’ UUGUAACAGUUAGGGAGC 5’

query=JS16_t0283296, target=FGSG_10398, score=4, range=367-384, strand=-1

target  5’ AUCAUAGUCAAUCCCUCC 3’
           : ::: ::::::::::
query   3’ UUGUAACAGUUAGGGAGC 5’

query=JS16_t0283296, target=FGSG_07016, score=4, range=114-130, strand=-1

target  5’ AAC-UUGUCAAGCCCUUG 3’
           ::: ::::::: ::::.:
query   3’ UUGUAACAGUUAGGGAGC 5’

query=JS16_t0283296, target=FGSG_05740, score=4, range=427-443, strand=-1

target  5’ AAC-UUGACAAUCUCUCG 3’
           ::: ::: :::::.::::
query   3’ UUGUAACAGUUAGGGAGC 5’

query=JS16_t0283296, target=FGSG_09475, score=4, range=198-216, strand=-1

target  5’ AAAAAUGUACAAUCCCUCG 3’
           :: : ::: ::::::::::
query   3’ UUGUAACA-GUUAGGGAGC 5’

query=JS16_t0283296, target=FGSG_08188, score=4, range=531-547, strand=-1

target  5’ CACA-UGUCAUUCCCUCG 3’
            ::: ::::: :::::::
query   3’ UUGUAACAGUUAGGGAGC 5’

No results for JS16_t0577888
query=JS16_t2240696, target=FGSG_05900, score=1.5, range=1049-1066, strand=-1

target  5’ CGACAAACUCGAGUUUCU 3’
           :.  ::::::::::::::
query   3’ GUCGUUUGAGCUCAAAGA 5’

query=JS16_t2240696, target=FGSG_02868, score=2, range=215-231, strand=-1

target  5’ CAG-AGACUCGAGUUUCU 3’
           ::: :.::::::::::::
query   3’ GUCGUUUGAGCUCAAAGA 5’

query=JS16_t2240696, target=FGSG_01640, score=2.5, range=518-535, strand=1

target  5’ CAGUAAACUCGGGUUUCA 3’
           :::.:::::::.:::::
query   3’ GUCGUUUGAGCUCAAAGA 5’

query=JS16_t2240696, target=FGSG_08543, score=2.5, range=1706-1722, strand=-1

target  5’ CA-CGGACUCGAGUUUCU 3’
           :: :..::::::::::::
query   3’ GUCGUUUGAGCUCAAAGA 5’

query=JS16_t2240696, target=FGSG_06049, score=3, range=844-860, strand=1

target  5’ CA-CAAACUCGAGGUUCU 3’
           :: :::::::::: ::::
query   3’ GUCGUUUGAGCUCAAAGA 5’

query=JS16_t2240696, target=FGSG_09022, score=3, range=1933-1949, strand=1

target  5’ CA-CAAACUCGAGUCUCU 3’

query=JS16_t2303751, target=FGSG_11656, score=4, range=3829-3846, strand=1

target  5’ GAUUUCUAUCGCCGC-AUU 3’
           ::.:::::::::.: ::.
query   3’ CUGAAGAUAGCGGUGAUAG 5’

query=JS16_t2303751, target=FGSG_03399, score=4, range=618-636, strand=1

target  5’ GACUUCUAGCGACACUAUC 3’
           :::::::: :: :::::::
query   3’ CUGAAGAUAGCGGUGAUAG 5’

query=JS16_t2303751, target=FGSG_11659, score=4, range=5945-5964, strand=1

target  5’ GACCUACUAUUGCCACUGUC 3’
           :: :: ::::.:::::::.::
query   3’ CU-GAAGAUAGCGGUGAUAG 5’

query=JS16_t2303751, target=FGSG_07233, score=4, range=473-492, strand=1

target  5’ GCCUUCCUAUCGCUGCUAUC 3’
           : ::: :::::::..:::::
query   3’ CUGAA-GAUAGCGGUGAUAG 5’

query=JS16_t2303751, target=FGSG_13715, score=4, range=811-828, strand=1

target  5’ UACUU-UAUUGUCACUAUC 3’
             :::: :::.:.:::::::
query   3’ CUGAAGAUAGCGGUGAUAG 5’

query=JS16_t2303751, target=FGSG_02821, score=4, range=177-194, strand=1

target  5’ CAUUUUUAUCG-CACUAUC 3’
             :.::.::::: :::::::
query   3’ CUGAAGAUAGCGGUGAUAG 5’

query=JS16_t2303751, target=FGSG_07873, score=4, range=187-204, strand=-1

target  5’ GACUUC-AUCGUCGCUAUC 3’
           :::::: ::::.:.:::::
query   3’ CUGAAGAUAGCGGUGAUAG 5’

query=JS16_t2303751, target=FGSG_00234, score=4, range=886-903, strand=-1

target  5’ GAAUUCUAUCGUCACU-UC 3’
           :: ::::::::.:::: ::
query   3’ CUGAAGAUAGCGGUGAUAG 5’

No results for JS16_t0337863
query=JS16_t2303869, target=FGSG_07803, score=3.5, range=1383-1403, strand=-1

target  5’ UACGUGGACUUUGUCUGGCAG 3’
           :  ::::::::::.::::::.
query   3’ AGCCACCUGAAACGGACCGUU 5’

query=JS16_t2303869, target=FGSG_01304, score=4, range=612-632, strand=-1

target  5’ UUGGUGGACUUUGCCUGACGG 3’
           :.:::::::::::::::: :..
query   3’ AGCCACCUGAAACGGACCGUU 5’

No results for JS16_t3199874
query=JS16_t4096898, target=FGSG_12282, score=3, range=240-260, strand=-1

target  5’ GGGAUCAUUCUUUGACGGUGU 3’
            ::::::::::::::::: :
query   3’ GCCUAGUAAGAAACUGCCAAA 5’

query=JS16_t4096898, target=FGSG_06181, score=4, range=1555-1574, strand=1

target  5’ CAGAUCA-UCUUUGACGGGUU 3’
           : ::::: :::::::::: ::
query   3’ GCCUAGUAAGAAACUGCCAAA 5’

No results for JS16_t4096962
No results for JS16_t3200081
No results for JS16_t4097027
No results for JS16_t4097056
No results for JS16_t1407957
query=JS16_t0476711, target=FGSG_09422, score=2.5, range=530-549, strand=-1

target  5’ CUUUGUCUGGCAAGCGAAAA 3’
           :::::.::::::::: ::::
query   3’ GAAACGGACCGUUCGAUUUU 5’

query=JS16_t0476711, target=FGSG_01564, score=4, range=621-640, strand=-1

target  5’ CAAUGGCUGGCAAGUUAAAA 3’
           :  :: ::::::::.:::::
query   3’ GAAACGGACCGUUCGAUUUU 5’

query=JS16_t0476711, target=FGSG_08101, score=4, range=25-43, strand=-1
```

```
           :: ::::::::::: :::
query   3’ GUCGUUUGAGCUCAAAGA 5’

query=JS16_t2240696, target=FGSG_01334, score=3, range=293-310, strand=-1

target  5’ CGGCGGACUUGAGUUUCU 3’
           :.::..:::.::::::::
query   3’ GUCGUUUGAGCUCAAAGA 5’

query=JS16_t2240696, target=FGSG_04262, score=3, range=674-691, strand=-1

target  5’ AAGCAAGCUUGAGUUUCU 3’
            :::::.::.::::::::
query   3’ GUCGUUUGAGCUCAAAGA 5’

query=JS16_t2240696, target=FGSG_10530, score=3, range=2198-2215, strand=-1

target  5’ UGACAGACUCGAGUUUCU 3’
           ..  ::.:::::::::::
query   3’ GUCGUUUGAGCUCAAAGA 5’

query=JS16_t2240696, target=FGSG_12373, score=3, range=836-854, strand=-1

target  5’ UGGCAAAACUUGAGUUUCU 3’
           ..:: :::::.::::::::
query   3’ GUCG-UUUGAGCUCAAAGA 5’

query=JS16_t2240696, target=FGSG_08159, score=3, range=206-223, strand=-1

target  5’ GAGCAUACUCGAGUUUCU 3’
            :::: ::::::::::::
query   3’ GUCGUUUGAGCUCAAAGA 5’

query=JS16_t2240696, target=FGSG_08378, score=3, range=1000-1018, strand=-1

target  5’ UGGCAGAACUCGAGUUUCC 3’
           ..::: ::::::::::::
query   3’ GUCGU-UUGAGCUCAAAGA 5’

query=JS16_t2240696, target=FGSG_04087, score=3, range=284-301, strand=-1

target  5’ CAACAAACUCGAGUGUCU 3’
           :: ::::::::::: :::
query   3’ GUCGUUUGAGCUCAAAGA 5’

query=JS16_t2240696, target=FGSG_08690, score=3.5, range=442-459, strand=-1

target  5’ CAGCGAGUUCGAGUUUCC 3’
           ::::.:..:::::::::
query   3’ GUCGUUUGAGCUCAAAGA 5’

query=JS16_t2240696, target=FGSG_08471, score=3.5, range=881-898, strand=-1

target  5’ CGGCAAACUUGCGUUUCU 3’
           :.:::::::.: ::::::
query   3’ GUCGUUUGAGCUCAAAGA 5’

query=JS16_t2240696, target=FGSG_13384, score=3.5, range=306-324, strand=-1

target  5’ CAGCAGACUCUGGGUUUCU 3’
           :::::.:::: :.::::::
query   3’ GUCGUUUGAG-CUCAAAGA 5’

query=JS16_t2240696, target=FGSG_09124, score=3.5, range=224-242, strand=-1

target  5’ UCGCAAACGUCGAGUUUCU 3’
           . :::::: ::::::::::
query   3’ GUCGUUUG-AGCUCAAAGA 5’

query=JS16_t2240696, target=FGSG_02938, score=4, range=990-1007, strand=1

target  5’ UAGUAAAUACGAGUUUCU 3’
           .::.:::. :::::::::
query   3’ GUCGUUUGAGCUCAAAGA 5’

query=JS16_t2240696, target=FGSG_10095, score=4, range=833-850, strand=1

target  5’ AAGCAAACUUGGGUUUCG 3’
            ::::::::.:.:::::
query   3’ GUCGUUUGAGCUCAAAGA 5’

query=JS16_t2240696, target=FGSG_00027, score=4, range=894-911, strand=1

target  5’ CAGCAAUCUUGAGUUUCA 3’
           :::::: ::.:::::::
query   3’ GUCGUUUGAGCUCAAAGA 5’

query=JS16_t2240696, target=FGSG_10971, score=4, range=1190-1208, strand=1

target  5’ AGGCAGACUCGAGUGUUCU 3’
            .:::.:::::::: ::::
query   3’ GUCGUUUGAGCUCA-AAGA 5’

target  5’ CUUCGCCUGGCAAG-UAAAC 3’
           ::: :::::::::: ::::
query   3’ GAAACGGACCGUUCGAUUUU 5’

No results for JS16_t4097183
No results for JS16_t3200249
query=JS16_t2304296, target=FGSG_04224, score=3, range=1517-1536, strand=-1

target  5’ UGAGGCCUUGAACGUCAUUC 3’
           :::::  :.:::::::::::
query   3’ ACUCCUAAGCUUGCAGUAAG 5’

query=JS16_t2304296, target=FGSG_12210, score=3.5, range=1320-1338, strand=-1

target  5’ UGAGG-UUUGAAUGUCAUUU 3’
           ::::: ::.:::.::::::.
query   3’ ACUCCUAAGCUUGCAGUAAG 5’

query=JS16_t2304296, target=FGSG_12062, score=4, range=198-217, strand=1

target  5’ UGAUGAUUUGGACGUCAUUG 3’
           ::: ::::.:.::::::::
query   3’ ACUCCUAAGCUUGCAGUAAG 5’

query=JS16_t2304296, target=FGSG_12529, score=4, range=920-939, strand=1

target  5’ AGAGACUUCGAAUGUCAUUC 3’
            :::  ::::::.:::::::
query   3’ ACUCCUAAGCUUGCAGUAAG 5’

query=JS16_t2304296, target=FGSG_10994, score=4, range=446-466, strand=1

target  5’ UGCAAGAUUCGAACGUGAUUC 3’
           :: : ::::::::::: ::::
query   3’ AC-UCCUAAGCUUGCAGUAAG 5’

query=JS16_t2304296, target=FGSG_06379, score=4, range=1667-1686, strand=-1

target  5’ UGGGUCUUCGAAUGUCAUUU 3’
           ::.:  ::::::.::::::.
query   3’ ACUCCUAAGCUUGCAGUAAG 5’

query=JS16_t2304296, target=FGSG_12942, score=4, range=134-152, strand=-1

target  5’ CCAGGA-UCGAGCGUCAUUC 3’
             :::: ::::.::::::::
query   3’ ACUCCUAAGCUUGCAGUAAG 5’

query=JS16_t2304302, target=FGSG_04755, score=4, range=545-568, strand=1

target  5’ AUGGCAUAGAGCUUCACGGAGCAC 3’
           :.::::.:  ::::::::.:::::
query   3’ UGCCGUGUGGCGAAGUGCUUCGUG 5’

query=JS16_t3200321, target=FGSG_03915, score=1.5, range=507-524, strand=-1

target  5’ AGAUCAUUGAUGCGUGUG 3’
           :.:::::::::::::::
query   3’ UUUAGUAACUACGCACAA 5’

query=JS16_t3200321, target=FGSG_07383, score=2.5, range=585-602, strand=-1

target  5’ AAGUCAUUGAUGAGUGUU 3’
           ::.:::::::::: :::::
query   3’ UUUAGUAACUACGCACAA 5’

query=JS16_t3200321, target=FGSG_02420, score=3.5, range=815-832, strand=1

target  5’ AGAUGAUUGAUGCGGGUU 3’
           :.:: ::::::::: :::
query   3’ UUUAGUAACUACGCACAA 5’

query=JS16_t3200321, target=FGSG_07640, score=3.5, range=278-294, strand=1

target  5’ GAACCAUUGAUG-GUGUU 3’
           .:: :::::::: :::::
query   3’ UUUAGUAACUACGCACAA 5’

query=JS16_t3200321, target=FGSG_01119, score=3.5, range=243-260, strand=-1

target  5’ GGAAUAUUGAUGUGUGUU 3’
           ..: .:::::::.:::::
query   3’ UUUAGUAACUACGCACAA 5’

query=JS16_t3200321, target=FGSG_12963, score=3.5, range=142-158, strand=-1

target  5’ AGAUCAUUGAUG-GUGUC 3’
           :.:::::::::: ::::
query   3’ UUUAGUAACUACGCACAA 5’

query=JS16_t3200321, target=FGSG_13068, score=3.5, range=382-398, strand=-1
```

```
query=JS16_t2240696, target=FGSG_10356, score=4, range=186-203, strand=1

target  5’ CAGGAAGCUCGAGUUGCU 3’
           ::: ::.:::::::: ::
query   3’ GUCGUUUGAGCUCAAAGA 5’

query=JS16_t2240696, target=FGSG_04901, score=4, range=1780-1797, strand=1

target  5’ AUCCAAACUCGAGUUUCG 3’
              ::::::::::::::
query   3’ GUCGUUUGAGCUCAAAGA 5’

query=JS16_t2240696, target=FGSG_13641, score=4, range=1593-1609, strand=1

target  5’ CA-UGAACUUGAGUUUCG 3’
           :: ..::::.:::::::
query   3’ GUCGUUUGAGCUCAAAGA 5’

query=JS16_t2240696, target=FGSG_01374, score=4, range=903-919, strand=1

target  5’ UA-UAAACUCGAGCUUCU 3’
           .: .::::::::: ::::
query   3’ GUCGUUUGAGCUCAAAGA 5’

query=JS16_t2240696, target=FGSG_04408, score=4, range=3567-3584, strand=1

target  5’ UGAACAACUCGAGUUUCU 3’
           ..   :::::::::::::
query   3’ GUCGUUUGAGCUCAAAGA 5’

query=JS16_t2240696, target=FGSG_00716, score=4, range=671-689, strand=1

target  5’ CAACACAACUCAAGUUUCU 3’
           :: :: ::::: :::::::
query   3’ GUCGU-UUGAGCUCAAAGA 5’

query=JS16_t2240696, target=FGSG_05900, score=4, range=1048-1064, strand=1

target  5’ GAG-AAACUCGAGUUUGU 3’
            :: :::::::::::: :
query   3’ GUCGUUUGAGCUCAAAGA 5’

query=JS16_t2240696, target=FGSG_02868, score=4, range=214-230, strand=1

target  5’ GAG-AAACUCGAGUCUCU 3’
            :: :::::::::: :::
query   3’ GUCGUUUGAGCUCAAAGA 5’

query=JS16_t2240696, target=FGSG_08242, score=4, range=584-600, strand=1

target  5’ CUGC-GACUCGAGUUUCG 3’
           : :: .:::::::::::
query   3’ GUCGUUUGAGCUCAAAGA 5’

query=JS16_t2240696, target=FGSG_11240, score=4, range=2768-2785, strand=-1

target  5’ CAGCAAAUUGGAGUUUUU 3’
           :::::::.: ::::::.:
query   3’ GUCGUUUGAGCUCAAAGA 5’

query=JS16_t2240696, target=FGSG_05692, score=4, range=716-733, strand=-1

target  5’ UAGUAAACUCGAGUUUGA 3’
           .::.::::::::::::
query   3’ GUCGUUUGAGCUCAAAGA 5’

query=JS16_t2240696, target=FGSG_05691, score=4, range=941-958, strand=-1

target  5’ CAGCAAAGUCGAGUUUUG 3’
           ::::::: ::::::::.
query   3’ GUCGUUUGAGCUCAAAGA 5’

query=JS16_t2240696, target=FGSG_07495, score=4, range=1106-1123, strand=-1

target  5’ CAGUGACCUCGAGUUUCG 3’
           :::..: ::::::::::
query   3’ GUCGUUUGAGCUCAAAGA 5’

query=JS16_t2240696, target=FGSG_11063, score=4, range=238-256, strand=-1

target  5’ CAGUGAACUCGAUGUUUCC 3’
           :::..::::::: :::::
query   3’ GUCGUUUGAGCU-CAAAGA 5’

query=JS16_t2240696, target=FGSG_02793, score=4, range=1028-1045, strand=-1

target  5’ CUUCAGACUUGAGUUUCU 3’
           :  ::.:::.::::::::
query   3’ GUCGUUUGAGCUCAAAGA 5’

query=JS16_t2240696, target=FGSG_12106, score=4, range=1124-1140, strand=-1

target  5’ ACGUC-UUGAUGCGUGUU 3’
           : .:: ::::::::::::
query   3’ UUUAGUAACUACGCACAA 5’

query=JS16_t3200321, target=FGSG_02223, score=4, range=587-605, strand=1

target  5’ GAAUUAUUGGUGCGAUGUU 3’
           .:::.::::.:::: ::::
query   3’ UUUAGUAACUACGC-ACAA 5’

query=JS16_t3200321, target=FGSG_11377, score=4, range=833-850, strand=1

target  5’ GGAUGAUUGAUGCCUGUU 3’
           ..:: :::::::: ::::
query   3’ UUUAGUAACUACGCACAA 5’

query=JS16_t3200321, target=FGSG_01708, score=4, range=240-258, strand=1

target  5’ GGAUCUCUUGAUGCGUGUU 3’
           ..:::  ::::::::::::
query   3’ UUUAG-UAACUACGCACAA 5’

query=JS16_t3200321, target=FGSG_06759, score=4, range=411-428, strand=-1

target  5’ GGAUCUUUGAUGCGUGUG 3’
           ..::: :::::::::::
query   3’ UUUAGUAACUACGCACAA 5’

query=JS16_t3200321, target=FGSG_09608, score=4, range=57-74, strand=-1

target  5’ UCAUCGUUGAUGCGUGUC 3’
             :::.:::::::::::
query   3’ UUUAGUAACUACGCACAA 5’

query=JS16_t3200321, target=FGSG_12071, score=4, range=54-71, strand=-1

target  5’ GUAUUAUUGAUGCGUGAU 3’
           . ::.::::::::::: :
query   3’ UUUAGUAACUACGCACAA 5’

query=JS16_t3200321, target=FGSG_12373, score=4, range=13-31, strand=-1

target  5’ GGAUGGAUUGAUGUGUGUU 3’
           ..::  :::::::.:::::
query   3’ UUUA-GUAACUACGCACAA 5’

query=JS16_t3200321, target=FGSG_10960, score=4, range=2031-2048, strand=-1

target  5’ ACGAUAUUGAUGCGUGUG 3’
           : . .::::::::::::
query   3’ UUUAGUAACUACGCACAA 5’

query=JS16_t3200321, target=FGSG_08378, score=4, range=583-599, strand=-1

target  5’ AACUCAUUGAUGC-UGUC 3’
           :: :::::::::: :::
query   3’ UUUAGUAACUACGCACAA 5’

query=JS16_t3200321, target=FGSG_04273, score=4, range=334-350, strand=-1

target  5’ UAAGCAUUGAUG-GUGUU 3’
            :: :::::::: :::::
query   3’ UUUAGUAACUACGCACAA 5’

No results for JS16_t0803852
No results for JS16_t2304415
query=JS16_t3200433, target=FGSG_08209, score=3, range=3838-3855, strand=1

target  5’ AUUUACAAUGCGUACGGC 3’
            :: :::::::::::::
query   3’ AAAUUGUUACGCAUGCCA 5’

query=JS16_t3200433, target=FGSG_04651, score=3.5, range=394-411, strand=-1

target  5’ CUUGACAAUGCAUACGGU 3’
            ::.::::::: ::::::
query   3’ AAAUUGUUACGCAUGCCA 5’

query=JS16_t3200433, target=FGSG_10767, score=3.5, range=1757-1773, strand=-1

target  5’ UUUAGCAAUGCG-ACGGG 3’
           ::::.::::::: ::::
query   3’ AAAUUGUUACGCAUGCCA 5’

query=JS16_t3200433, target=FGSG_09790, score=4, range=1608-1625, strand=-1

target  5’ UUUGGCAUUGCGUAUGGU 3’
           :::..:: ::::::.:::
query   3’ AAAUUGUUACGCAUGCCA 5’

query=JS16_t3200433, target=FGSG_06957, score=4, range=1829-1845, strand=-1
```

```
target  5' CA-UGAAUUUGAGUUUCU 3'
           :: ..::.:.::::::::
query   3' GUCGUUUGAGCUCAAAGA 5'

query=JS16_t2240696, target=FGSG_01597, score=4, range=1204-1221, strand=-1

target  5' CACCACGCUCGAGUUUCU 3'
           :: :: .:::::::::::
query   3' GUCGUUUGAGCUCAAAGA 5'

query=JS16_t2240696, target=FGSG_07978, score=4, range=327-345, strand=-1

target  5' CAGCACUGCUCGAGUUUCU 3'
           :::::  .:::::::::::
query   3' GUCGU-UUGAGCUCAAAGA 5'

query=JS16_t2240696, target=FGSG_01973, score=4, range=35-52, strand=-1

target  5' CAUGAAGCUUGAGUUUCU 3'
           ::  ::.::.::::::::
query   3' GUCGUUUGAGCUCAAAGA 5'

query=JS16_t2240696, target=FGSG_08748, score=4, range=2048-2065, strand=-1

target  5' CAUCAAACUCGACUUUCA 3'
           :: ::::::::: ::::
query   3' GUCGUUUGAGCUCAAAGA 5'

query=JS16_t2240696, target=FGSG_01288, score=4, range=503-520, strand=-1

target  5' CCUCAAACUCGAGUUCCU 3'
           :  :::::::::::: ::
query   3' GUCGUUUGAGCUCAAAGA 5'

query=JS16_t2240696, target=FGSG_11026, score=4, range=8801-8818, strand=-1

target  5' CAUCCAACUCGAGUCUCU 3'
           :: : ::::::::: :::
query   3' GUCGUUUGAGCUCAAAGA 5'

query=JS16_t2240696, target=FGSG_11599, score=4, range=1631-1647, strand=-1

target  5' UAGU-AACUCUAGUUUCU 3'
           .::. ::::: :::::::
query   3' GUCGUUUGAGCUCAAAGA 5'

query=JS16_t2240696, target=FGSG_08215, score=4, range=2019-2035, strand=-1

target  5' CUGC-GAUUCGAGUUUCU 3'
           : :: .:.::::::::::
query   3' GUCGUUUGAGCUCAAAGA 5'

query=JS16_t2240696, target=FGSG_01411, score=4, range=725-741, strand=-1

target  5' UGG-AAACUCGAGUCUCU 3'
           ..: ::::::::: :::
query   3' GUCGUUUGAGCUCAAAGA 5'

query=JS16_t2240696, target=FGSG_12033, score=4, range=911-927, strand=-1

target  5' UUGU-AACUUGAGUUUCU 3'
           . :. ::::.::::::::
query   3' GUCGUUUGAGCUCAAAGA 5'

query=JS16_t2240696, target=FGSG_10356, score=4, range=188-204, strand=-1

target  5' CAGC-AACUCGAGCUUCC 3'
           :::: :::::::: :::
query   3' GUCGUUUGAGCUCAAAGA 5'

query=JS16_t3136749, target=FGSG_07358, score=3.5, range=1111-1130, strand=1

target  5' GUCAGAGUGAUCGGUAUCGG 3'
           :: ::::..::::::::::.
query   3' CACUCUCGUUAGCCAUAGCU 5'

query=JS16_t3136749, target=FGSG_11207, score=4, range=2257-2276, strand=1

target  5' GUUCAAGCAGUCGGUAUCGA 3'
           ::   ::::.::::::::::
query   3' CACUCUCGUUAGCCAUAGCU 5'

query=JS16_t3136749, target=FGSG_09215, score=4, range=26-44, strand=1

target  5' GUG-GAAUAAUCGGUAUCGC 3'
           ::: :: .::::::::::::
query   3' CACUCUCGUUAGCCAUAGCU 5'

query=JS16_t3136749, target=FGSG_02874, score=4, range=1927-1947, strand=1

target  5' GUGAGAGUAUAUCGGUAUUGG 3'
```

```
target  5' GUUAACAAUGU-UACGGU 3'
            :::::::::. ::::::
query   3' AAAUUGUUACGCAUGCCA 5'

query=JS16_t4097353, target=FGSG_00171, score=3, range=19-38, strand=-1

target  5' GGCUCGGGAGAGGGUUCACG 3'
           :. ::.:::::::::::::
query   3' CUCAGUCCUCUCCCAAGUGA 5'

query=JS16_t4097353, target=FGSG_08887, score=4, range=1887-1905, strand=1

target  5' GCCUCA-GAGAGGGUUCACA 3'
           :  ::: ::::::::::::
query   3' CUCAGUCCUCUCCCAAGUGA 5'

query=JS16_t4097353, target=FGSG_04938, score=4, range=993-1013, strand=-1

target  5' GAAACAGCGAGAGGGUUCGCU 3'
           ::  ::: ::::::::::.::
query   3' CUCAGUC-CUCUCCCAAGUGA 5'

query=JS16_t3200460, target=FGSG_00971, score=4, range=3-25, strand=-1

target  5' GCACUUGUGAGAGGAUUCCAGAC 3'
             :::::  ::::::::::::::
query   3' GUUGAACUGUCUCCUAAGGUCUG 5'

No results for JS16_t3200472
No results for JS16_t4097398
query=JS16_t1408281, target=FGSG_07214, score=2.5, range=2465-2482, strand=-1

target  5' UUCCUCCACGACGUGGCG 3'
           ::  .:::::::::::::
query   3' AACAGGGUGCUGCACCGC 5'

query=JS16_t1408281, target=FGSG_13118, score=3, range=1058-1075, strand=1

target  5' CUGUCCCACGACGUGCCG 3'
            :::::::::::::: ::
query   3' AACAGGGUGCUGCACCGC 5'

query=JS16_t1408281, target=FGSG_00283, score=3, range=2001-2018, strand=1

target  5' UUGGACCACGGCGUGGCG 3'
           :::  :::::.:::::::
query   3' AACAGGGUGCUGCACCGC 5'

query=JS16_t1408281, target=FGSG_09611, score=3, range=1572-1588, strand=1

target  5' UUGGCCCACGAC-UGGCG 3'
           ::: :::::::: :::::
query   3' AACAGGGUGCUGCACCGC 5'

query=JS16_t1408281, target=FGSG_09065, score=3.5, range=1216-1233, strand=-1

target  5' AAGUUCCACGAUGUGGCG 3'
             ::.::::::.::::::
query   3' AACAGGGUGCUGCACCGC 5'

query=JS16_t1408281, target=FGSG_06904, score=3.5, range=403-420, strand=-1

target  5' UUUCUUCACGACGUGGCG 3'
           ::  ..::::::::::::
query   3' AACAGGGUGCUGCACCGC 5'

query=JS16_t1408281, target=FGSG_04208, score=4, range=925-942, strand=1

target  5' AUGUCCAAUGACGUGGCG 3'
            ::::: :.:::::::::
query   3' AACAGGGUGCUGCACCGC 5'

query=JS16_t1408281, target=FGSG_07798, score=4, range=2582-2598, strand=1

target  5' AUGU-CCAUGAUGUGGCG 3'
            ::: :::.::.::::::
query   3' AACAGGGUGCUGCACCGC 5'

query=JS16_t1408281, target=FGSG_00196, score=4, range=399-416, strand=1

target  5' UAGUCCCACGACGUUGCA 3'
           : :::::::::::: ::
query   3' AACAGGGUGCUGCACCGC 5'

query=JS16_t1408281, target=FGSG_06171, score=4, range=413-429, strand=1

target  5' UUG-CCCGCGACGUGGAG 3'
           ::: :::.:::::::: :
query   3' AACAGGGUGCUGCACCGC 5'

query=JS16_t1408281, target=FGSG_11328, score=4, range=583-599, strand=1
```

```
           :::::::.: ::::::::.:.
query   3' CACUCUCGU-UAGCCAUAGCU 5'

query=JS16_t3136749, target=FGSG_08179, score=4, range=617-636, strand=-1

target  5' AUGAGAGUAAACGGUAUCGA 3'
            ::::::.:: :::::::::
query   3' CACUCUCGUUAGCCAUAGCU 5'

query=JS16_t3136749, target=FGSG_03589, score=4, range=150-169, strand=-1

target  5' UGGACAGCAAUCGGUAUUGA 3'
             :: ::::::::::::.::
query   3' CACUCUCGUUAGCCAUAGCU 5'

query=JS16_t3136749, target=FGSG_10404, score=4, range=805-824, strand=-1

target  5' GUGAAGCCAAUCGGUGUCGG 3'
           :::: . ::::::::.:::.
query   3' CACUCUCGUUAGCCAUAGCU 5'

query=JS16_t3136749, target=FGSG_07648, score=4, range=163-182, strand=-1

target  5' GGCAGAGUAAUCGGUAUCGC 3'
           :  ::::.:::::::::::
query   3' CACUCUCGUUAGCCAUAGCU 5'

query=JS16_t3136749, target=FGSG_01606, score=4, range=1184-1203, strand=-1

target  5' AUGAGACAAAUCGGUAUCGA 3'
            :::::  ::::::::::::
query   3' CACUCUCGUUAGCCAUAGCU 5'

query=JS16_t3136749, target=FGSG_00906, score=4, range=782-800, strand=-1

target  5' GUGA-AGCAAUCGGUAGCGU 3'
           :::: ::::::::::: ::
query   3' CACUCUCGUUAGCCAUAGCU 5'

query=JS16_t3136749, target=FGSG_10043, score=4, range=2038-2056, strand=-1

target  5' CUGA-ACCAAUCGGUGUCGA 3'
            ::: : ::::::::.::::
query   3' CACUCUCGUUAGCCAUAGCU 5'

query=JS16_t3136749, target=FGSG_06592, score=4, range=1115-1133, strand=-1

target  5' GCGAGAGC-GUCGGUAUCGA 3'
           : :::::: .::::::::::
query   3' CACUCUCGUUAGCCAUAGCU 5'

query=JS16_t1343996, target=FGSG_04218, score=2.5, range=377-395, strand=-1

target  5' AUGG-CGACAACUUGCUUGG 3'
           ::::  .::::::::::::::
query   3' UACCAUUUGUUGAACGAACC 5'

query=JS16_t1343996, target=FGSG_03401, score=3.5, range=392-410, strand=1

target  5' GUGGUAAACAAC-UGCUUGC 3'
           .::::::::::: ::::::
query   3' UACCAUUUGUUGAACGAACC 5'

query=JS16_t1343996, target=FGSG_09801, score=3.5, range=3218-3236, strand=-1

target  5' GAGGU-AGCAACUUGCUUGG 3'
           . ::: :.::::::::::::
query   3' UACCAUUUGUUGAACGAACC 5'

query=JS16_t1343996, target=FGSG_00526, score=3.5, range=575-593, strand=-1

target  5' GUGAU-AACAACUUGUUUGG 3'
           .:: : :::::::::.::::
query   3' UACCAUUUGUUGAACGAACC 5'

query=JS16_t1343996, target=FGSG_00748, score=4, range=673-692, strand=1

target  5' AUGUUCAACAGCUUGCUUGC 3'
           ::: : ::::.::::::::
query   3' UACCAUUUGUUGAACGAACC 5'

query=JS16_t1343996, target=FGSG_03914, score=4, range=53-72, strand=-1

target  5' CUUGUAAGCAGCUUGCUUGG 3'
            : ::::.::.:::::::::
query   3' UACCAUUUGUUGAACGAACC 5'

query=JS16_t1343996, target=FGSG_07198, score=4, range=955-974, strand=-1

target  5' AUGUUGGCCAACUUGCUUGG 3'
           ::: :.. ::::::::::::
query   3' UACCAUUUGUUGAACGAACC 5'

target  5' AUG-CCCGCGACGUGGCA 3'
            :: :::.:::::::::
query   3' AACAGGGUGCUGCACCGC 5'

query=JS16_t1408281, target=FGSG_01390, score=4, range=844-861, strand=1

target  5' UUACCCCACGACUUGGCG 3'
           ::  :::::::: :::::
query   3' AACAGGGUGCUGCACCGC 5'

query=JS16_t1408281, target=FGSG_04807, score=4, range=506-523, strand=-1

target  5' UUGUCUCACGACGUGCUG 3'
           :::::.::::::::: .:
query   3' AACAGGGUGCUGCACCGC 5'

query=JS16_t1408281, target=FGSG_00675, score=4, range=1328-1345, strand=-1

target  5' UUGUCCCUCGACGUGGAG 3'
           ::::::: :::::::: :
query   3' AACAGGGUGCUGCACCGC 5'

query=JS16_t1408281, target=FGSG_05995, score=4, range=1130-1147, strand=-1

target  5' UUGGCCCACGAUAUGGCG 3'
           ::: :::::::. :::::
query   3' AACAGGGUGCUGCACCGC 5'

query=JS16_t1408281, target=FGSG_04323, score=4, range=227-244, strand=-1

target  5' UCGACCUACGACGUGGUG 3'
           : : ::.:::::::::.:
query   3' AACAGGGUGCUGCACCGC 5'

query=JS16_t1408281, target=FGSG_04821, score=4, range=1495-1512, strand=-1

target  5' GUGGCCCACGACGUGGUU 3'
            :: ::::::::::::.
query   3' AACAGGGUGCUGCACCGC 5'

query=JS16_t1408281, target=FGSG_10730, score=4, range=625-642, strand=-1

target  5' CUGCGCUACGACGUGGCG 3'
            ::  :.:::::::::::
query   3' AACAGGGUGCUGCACCGC 5'

query=JS16_t1408281, target=FGSG_06868, score=4, range=810-826, strand=-1

target  5' UUGUGCCACGAC-UGGCU 3'
           :::: ::::::: ::::
query   3' AACAGGGUGCUGCACCGC 5'

query=JS16_t1408281, target=FGSG_06993, score=4, range=249-265, strand=-1

target  5' AUGUGCCA-GACGUGGCG 3'
            ::: ::: :::::::::
query   3' AACAGGGUGCUGCACCGC 5'

No results for JS16_t2304553
query=JS16_t0476756, target=FGSG_03269, score=4, range=838-858, strand=-1

target  5' CUUGG-GAUCCUCGACUUCAAG 3'
           ::::: :::::.:: :::::::
query   3' GAACCACUAGGGGCCGAAGUUC 5'

No results for JS16_t0585638
No results for JS16_t1408368
No results for JS16_t2304623
query=JS16_t3200706, target=FGSG_12504, score=2, range=121-138, strand=-1

target  5' CAGGCACUUCAUUAUGUG 3'
           :::: :::::::::.:::
query   3' GUCCUUGAAGUAAUGCAC 5'

query=JS16_t3200706, target=FGSG_02816, score=3, range=193-210, strand=1

target  5' GAGGAACUUCGUUACGUA 3'
            :::::::::.::::::
query   3' GUCCUUGAAGUAAUGCAC 5'

query=JS16_t3200706, target=FGSG_13878, score=3.5, range=28232-28249, strand=1

target  5' CGGGAACUUCGUUACCUG 3'
           :.::::::::.:::: ::
query   3' GUCCUUGAAGUAAUGCAC 5'

query=JS16_t3200706, target=FGSG_11664, score=3.5, range=434-451, strand=1

target  5' AAGGGACUUCAUUACUUG 3'
            :::.:::::::::: ::
query   3' GUCCUUGAAGUAAUGCAC 5'
```

```
query=JS16_t1343996, target=FGSG_10009, score=4, range=2447-2466, strand=-1

target  5’ AUAGGCAACGACUUGCUUGG 3’
           :: :  :::.::::::::::
query   3’ UACCAUUUGUUGAACGAACC 5’

query=JS16_t1343996, target=FGSG_00990, score=4, range=613-632, strand=-1

target  5’ ACGGCACACAACUUGCUUGC 3’
           : :: : ::::::::::::
query   3’ UACCAUUUGUUGAACGAACC 5’

No results for JS16_t2240739
No results for JS16_t2240756
No results for JS16_t0686671
No results for JS16_t1344053
No results for JS16_t2240783
query=JS16_t3136862, target=FGSG_11314, score=2.5, range=1502-1520, strand=1

target  5’ CUCCCGUUUUUGAUGACGA 3’
            .::::::.::::::::::
query   3’ CGGGGCAAGAACUACUGCU 5’

query=JS16_t3136862, target=FGSG_00680, score=2.5, range=1691-1709, strand=1

target  5’ AUCUUGUUCUUGAUGACGA 3’
            .:..::::::::::::::
query   3’ CGGGGCAAGAACUACUGCU 5’

query=JS16_t3136862, target=FGSG_02337, score=2.5, range=710-728, strand=-1

target  5’ GCCUCGUUCUUGCUGACGA 3’
           :::.:::::::: ::::::
query   3’ CGGGGCAAGAACUACUGCU 5’

query=JS16_t3136862, target=FGSG_01433, score=3, range=6073-6092, strand=1

target  5’ GCUCGUGUUCUUGGUGACGA 3’
           ::.: .:::::::.::::::
query   3’ CGGG-GCAAGAACUACUGCU 5’

query=JS16_t3136862, target=FGSG_11715, score=3, range=437-455, strand=1

target  5’ GCACGGUUUUUGAUGACGA 3’
           :: : :::.::::::::::
query   3’ CGGGGCAAGAACUACUGCU 5’

query=JS16_t3136862, target=FGSG_02645, score=3, range=1631-1649, strand=-1

target  5’ GCGUUGUUCUUGAUGACGU 3’
           :: ..:::::::::::::
query   3’ CGGGGCAAGAACUACUGCU 5’

query=JS16_t3136862, target=FGSG_01955, score=3, range=110-128, strand=-1

target  5’ GGGCGGUUCUUGAUGACGA 3’
           :  : ::::::::::::::
query   3’ CGGGGCAAGAACUACUGCU 5’

query=JS16_t3136862, target=FGSG_07897, score=3.5, range=155-173, strand=1

target  5’ GCUUCGUUCUCGAUGACGG 3’
           ::..:::::: :::::::.
query   3’ CGGGGCAAGAACUACUGCU 5’

query=JS16_t3136862, target=FGSG_06705, score=3.5, range=533-551, strand=1

target  5’ GAGUCGUUCUUGGUGACGA 3’
           :  .::::::::.::::::
query   3’ CGGGGCAAGAACUACUGCU 5’

query=JS16_t3136862, target=FGSG_01920, score=3.5, range=662-680, strand=1

target  5’ ACGCUAUUCUUGAUGACGA 3’
            : :. :::::::::::::
query   3’ CGGGGCAAGAACUACUGCU 5’

query=JS16_t3136862, target=FGSG_02974, score=3.5, range=1520-1538, strand=-1

target  5’ AUCUCGUUCUUGAUGGCGG 3’
            .:.:::::::::::.::.
query   3’ CGGGGCAAGAACUACUGCU 5’

query=JS16_t3136862, target=FGSG_04787, score=3.5, range=20-38, strand=-1

target  5’ GACCUGUUCUUGAUGGUGA 3’
           : ::.::::::::::..::
query   3’ CGGGGCAAGAACUACUGCU 5’

query=JS16_t3136862, target=FGSG_08362, score=3.5, range=44-62, strand=-1

query=JS16_t3200706, target=FGSG_06671, score=4, range=1057-1074, strand=1

target  5’ AAGGAACCUUAUUACGUG 3’
            :::::: :.::::::::
query   3’ GUCCUUGAAGUAAUGCAC 5’

query=JS16_t3200706, target=FGSG_00840, score=4, range=1455-1472, strand=1

target  5’ CAAGAACUUCAUUACCUC 3’
           :: :::::::::::: :
query   3’ GUCCUUGAAGUAAUGCAC 5’

query=JS16_t3200706, target=FGSG_11278, score=4, range=139-156, strand=-1

target  5’ CAAGAGUUUCAUUGCGUG 3’
           :: ::..::::::.::::
query   3’ GUCCUUGAAGUAAUGCAC 5’

query=JS16_t3200706, target=FGSG_10835, score=4, range=1553-1570, strand=-1

target  5’ GGGGGACUUCAUUGCGUA 3’
            .::.::::::::.:::
query   3’ GUCCUUGAAGUAAUGCAC 5’

query=JS16_t3200706, target=FGSG_12604, score=4, range=767-784, strand=-1

target  5’ CUCCAACUUCAUUAUGUG 3’
           :   ::::::::::.:::
query   3’ GUCCUUGAAGUAAUGCAC 5’

query=JS16_t3200706, target=FGSG_01739, score=4, range=751-768, strand=-1

target  5’ GAUGAACUUCAGUACGUG 3’
            : :::::::: ::::::
query   3’ GUCCUUGAAGUAAUGCAC 5’

query=JS16_t3200706, target=FGSG_04904, score=4, range=452-469, strand=-1

target  5’ CUGGUACUUUAUUACGUU 3’
           : :: ::::.:::::::
query   3’ GUCCUUGAAGUAAUGCAC 5’

query=JS16_t3200706, target=FGSG_05370, score=4, range=1319-1336, strand=-1

target  5’ CUGCAACUUCAUUAGGUG 3’
           : : :::::::::: :::
query   3’ GUCCUUGAAGUAAUGCAC 5’

No results for JS16_t3200716
query=JS16_t0476767, target=FGSG_04804, score=3, range=863-881, strand=-1

target  5’ UUGA-UACUGUGCUUCGUGG 3’
            ::: .:::::::::::::.
query   3’ UACUGGUGACACGAAGCACU 5’

query=JS16_t0476767, target=FGSG_12481, score=3, range=855-874, strand=-1

target  5’ AGAAACACUGUGCUUCGUGA 3’
           :  : :::::::::::::::
query   3’ UACUGGUGACACGAAGCACU 5’

query=JS16_t0476767, target=FGSG_12126, score=3.5, range=5836-5855, strand=1

target  5’ AUGGCCAUGGUGCUUCGUGA 3’
           :::.:::.  ::::::::::
query   3’ UACUGGUGACACGAAGCACU 5’

query=JS16_t0476767, target=FGSG_10523, score=3.5, range=4412-4431, strand=-1

target  5’ AAGAUUGCUGUGCUUUGUGA 3’
           : ::...::::::::.::::
query   3’ UACUGGUGACACGAAGCACU 5’

query=JS16_t0476767, target=FGSG_07119, score=4, range=1568-1586, strand=1

target  5’ AUGA-GACUCUGCUUCGUGA 3’
           ::::  ::: ::::::::::
query   3’ UACUGGUGACACGAAGCACU 5’

query=JS16_t0476767, target=FGSG_12095, score=4, range=2025-2043, strand=-1

target  5’ CUGACC-UUGUGUUUCGUGA 3’
            ::::: .::::.:::::::
query   3’ UACUGGUGACACGAAGCACU 5’

query=JS16_t0476767, target=FGSG_12040, score=4, range=441-459, strand=-1

target  5’ GUGA-CGCUGUUCUUCGUGA 3’
           .::: :.:::: ::::::::
query   3’ UACUGGUGACACGAAGCACU 5’
```

```
target  5’ AGGUCGUUCUUGAUGACGA 3’
              .:::::::::::::::
query   3’ CGGGGCAAGAACUACUGCU 5’

query=JS16_t3136862, target=FGSG_07524, score=3.5, range=2237-2255, strand=-1

target  5’ GCUCCCUUCUUGAUAACGA 3’
           ::.:: :::::::: ::::
query   3’ CGGGGCAAGAACUACUGCU 5’

query=JS16_t3136862, target=FGSG_06844, score=3.5, range=1231-1248, strand=-1

target  5’ GCGCU-UUCUUGAUGACGC 3’
           :: :. ::::::::::::
query   3’ CGGGGCAAGAACUACUGCU 5’

query=JS16_t3136862, target=FGSG_07908, score=3.5, range=416-433, strand=-1

target  5’ GAUCC-UUCUUGAUGACGU 3’
           : .:: ::::::::::::
query   3’ CGGGGCAAGAACUACUGCU 5’

query=JS16_t3136862, target=FGSG_03261, score=4, range=956-974, strand=1

target  5’ GUUUUGUUCUUGAUGAAGA 3’
           :....::::::::::: ::
query   3’ CGGGGCAAGAACUACUGCU 5’

query=JS16_t3136862, target=FGSG_02315, score=4, range=4601-4619, strand=1

target  5’ UCCCUGUCCUUGAUGACGG 3’
            :::.:: ::::::::::.
query   3’ CGGGGCAAGAACUACUGCU 5’

query=JS16_t3136862, target=FGSG_11310, score=4, range=70-89, strand=-1

target  5’ GCUUCGUUCUUGAUGGAUGA 3’
           ::..:::::::::: ::.::
query   3’ CGGGGCAAGAACUA-CUGCU 5’

query=JS16_t3136862, target=FGSG_12551, score=4, range=236-255, strand=-1

target  5’ GCCGCCGUUCUUGGUGGCGC 3’
           ::: :::::::::.::.::
query   3’ CGG-GGCAAGAACUACUGCU 5’

query=JS16_t3136862, target=FGSG_03388, score=4, range=245-263, strand=-1

target  5’ AUAUUGUUCUUGAUGACGG 3’
            . ..:::::::::::::.
query   3’ CGGGGCAAGAACUACUGCU 5’

query=JS16_t3136862, target=FGSG_02386, score=4, range=251-269, strand=-1

target  5’ GCACCGUUCUUGAGGAUGA 3’
           :: :::::::::: ::.::
query   3’ CGGGGCAAGAACUACUGCU 5’

query=JS16_t3136862, target=FGSG_13341, score=4, range=179-197, strand=-1

target  5’ GCCUGGUUCUUGAGGACGG 3’
           :::. :::::::: ::::.
query   3’ CGGGGCAAGAACUACUGCU 5’

query=JS16_t3136862, target=FGSG_00490, score=4, range=1403-1422, strand=-1

target  5’ GUACUCGUUCUUGUUGACGA 3’
           :. :.:::::::: ::::::
query   3’ CG-GGGCAAGAACUACUGCU 5’

query=JS16_t3136862, target=FGSG_00076, score=4, range=935-953, strand=-1

target  5’ GCUACCUUCUUGAUGAUGG 3’
           ::. : :::::::::.:.
query   3’ CGGGGCAAGAACUACUGCU 5’

query=JS16_t3136862, target=FGSG_13878, score=4, range=27470-27488, strand=-1

target  5’ GCCAGGUUCUUGAUGAUGC 3’
           :::  :::::::::::.:
query   3’ CGGGGCAAGAACUACUGCU 5’

query=JS16_t3136862, target=FGSG_04922, score=4, range=779-798, strand=-1

target  5’ GCCACCGUUCUUGAGGACGU 3’
           ::: :::::::::: ::::
query   3’ CGG-GGCAAGAACUACUGCU 5’

query=JS16_t3136862, target=FGSG_07384, score=4, range=244-262, strand=-1

target  5’ GCUUGAUUCUUGAUGACGU 3’
           ::..  ::::::::::::

query=JS16_t3200741, target=FGSG_09748, score=3, range=64-85, strand=1

target  5’ UGGAGGACUGUCCAUGAGAUGG 3’
           :::: . ::::::::::::::.
query   3’ ACCU-UGGACAGGUACUCUACU 5’

query=JS16_t3200741, target=FGSG_06333, score=3.5, range=10-29, strand=-1

target  5’ UGGAUCUUGUCCAUGAGA-GA 3’
           :::: :.::::::::::: ::
query   3’ ACCUUGGACAGGUACUCUACU 5’

query=JS16_t3200741, target=FGSG_04333, score=4, range=158-178, strand=-1

target  5’ GGGCACCGGUCCAUGGGAUGA 3’
            :: ::: :::::::.:::::
query   3’ ACCUUGGACAGGUACUCUACU 5’

query=JS16_t0220470, target=FGSG_11973, score=4, range=601-620, strand=1

target  5’ UGGCUCACUGCCUCGCUCAU 3’
           :::::: :::::. ::::::
query   3’ ACCGAGGGACGGGCCGAGUA 5’

No results for JS16_t2304782
query=JS16_t4097705, target=FGSG_07139, score=4, range=3106-3124, strand=-1

target  5’ UGGCA-GUGAGUUCGCAGAG 3’
           ::::: :::::.::.::::
query   3’ ACCGUCCACUCGAGUGUCUG 5’

query=JS16_t4097705, target=FGSG_09448, score=4, range=1339-1357, strand=-1

target  5’ GGGUA-GUGAGCUCACGGAU 3’
            ::.: :::::::::.::.
query   3’ ACCGUCCACUCGAGUGUCUG 5’

query=JS16_t4097705, target=FGSG_02809, score=4, range=226-244, strand=-1

target  5’ UCGC-GAUGAGCUCACGGAC 3’
           : :: : :::::::::.:::
query   3’ ACCGUCCACUCGAGUGUCUG 5’

No results for JS16_t3200843
query=JS16_t0803904, target=FGSG_13396, score=4, range=181-201, strand=1

target  5’ CUCAGCAAAGACGGUCAUCUA 3’
            :: ::: ::::::::::::
query   3’ UAGCCGUGUCUGCCAGUAGAA 5’

query=JS16_t0803904, target=FGSG_10087, score=4, range=943-962, strand=1

target  5’ AUCGGC-CAGUCGGUCAUUUU 3’
           :::::: ::: :::::::.::
query   3’ UAGCCGUGUCUGCCAGUAGAA 5’

query=JS16_t0803904, target=FGSG_00437, score=4, range=2665-2684, strand=-1

target  5’ AUCAGCA-AGACGGGCAUCUU 3’
           ::: ::: :::::: ::::::
query   3’ UAGCCGUGUCUGCCAGUAGAA 5’

query=JS16_t3200873, target=FGSG_11282, score=1.5, range=609-627, strand=-1

target  5’ GAUACCCAGCUUGAGCAUC 3’
           ::.  :::::::::::::::
query   3’ CUG-GGGUCGAACUCGUAG 5’

query=JS16_t3200873, target=FGSG_00152, score=2, range=1192-1209, strand=1

target  5’ GGCGUCAGCUUGAGCAUC 3’
           :.: .:::::::::::::
query   3’ CUGGGGUCGAACUCGUAG 5’

query=JS16_t3200873, target=FGSG_10769, score=2.5, range=826-843, strand=-1

target  5’ GGCACCAGUUUGAGCAUC 3’
           :.: ::::.:::::::::
query   3’ CUGGGGUCGAACUCGUAG 5’

query=JS16_t3200873, target=FGSG_04229, score=3, range=386-404, strand=1

target  5’ GACCCCAGUUUGAGCGAUC 3’
           ::::::::.:::::: :::
query   3’ CUGGGGUCGAACUCG-UAG 5’

query=JS16_t3200873, target=FGSG_07413, score=3, range=1156-1173, strand=1

target  5’ GCCCCUAGCUUGAGCGUC 3’
           : :::.:::::::::.::
query   3’ CUGGGGUCGAACUCGUAG 5’
```

```
query   3’ CGGGGCAAGAACUACUGCU 5’

query=JS16_t3136862, target=FGSG_13319, score=4, range=815-832, strand=-1

target  5’ GCCCU-UUCUUGCUGACGG 3’
           ::::. :::::: :::::.
query   3’ CGGGGCAAGAACUACUGCU 5’

query=JS16_t3136862, target=FGSG_07169, score=4, range=1727-1745, strand=-1

target  5’ GCGCCAUCCUUGAUGACGA 3’
           :: :: : :::::::::::
query   3’ CGGGGCAAGAACUACUGCU 5’

query=JS16_t3136862, target=FGSG_10164, score=4, range=1169-1186, strand=-1

target  5’ GCGUC-UUCUUGAUGAUGG 3’
           :: .: ::::::::::.:.
query   3’ CGGGGCAAGAACUACUGCU 5’

query=JS16_t3136862, target=FGSG_12068, score=4, range=131-149, strand=-1

target  5’ GCGCCCUUCUUGAUGACAA 3’
           :: :: ::::::::::: :
query   3’ CGGGGCAAGAACUACUGCU 5’

query=JS16_t3136862, target=FGSG_11852, score=4, range=2219-2237, strand=-1

target  5’ GGCAAGUUCUUGAUGAUGA 3’
           : :  :::::::::::.::
query   3’ CGGGGCAAGAACUACUGCU 5’

query=JS16_t3136862, target=FGSG_03589, score=4, range=186-203, strand=-1

target  5’ ACGCCGUUCUUGAUGA-GA 3’
            : ::::::::::::: ::
query   3’ CGGGGCAAGAACUACUGCU 5’

query=JS16_t3136862, target=FGSG_05128, score=4, range=128-146, strand=-1

target  5’ GCAUGCUUCUUGAUGACGG 3’
           :: .  ::::::::::::.
query   3’ CGGGGCAAGAACUACUGCU 5’

query=JS16_t3136862, target=FGSG_11962, score=4, range=248-265, strand=-1

target  5’ GACCC-UUCUUGAUCACGA 3’
           : ::: :::::::: ::::
query   3’ CGGGGCAAGAACUACUGCU 5’

query=JS16_t0268868, target=FGSG_08634, score=3, range=491-510, strand=1

target  5’ UGGAGGCGAGCAAUCGACUU 3’
           : ::: :.::::::::::::
query   3’ AACUCGGUUCGUUAGCUGAA 5’

query=JS16_t0268868, target=FGSG_01862, score=4, range=596-615, strand=1

target  5’ UUGUGCCGAGCAUUCGACUU 3’
           ::: :::.:::: :::::::
query   3’ AACUCGGUUCGUUAGCUGAA 5’

query=JS16_t0268868, target=FGSG_02695, score=4, range=872-892, strand=1

target  5’ UUGAUCCCAAGCAAUUGACUG 3’
           ::::  :::::::::.::::
query   3’ AACU-CGGUUCGUUAGCUGAA 5’

query=JS16_t0268868, target=FGSG_03930, score=4, range=40-58, strand=1

target  5’ UUG-CCCAAGCAAUUGACUG 3’
           :::  :::::::::.::::
query   3’ AACUCGGUUCGUUAGCUGAA 5’

query=JS16_t0268868, target=FGSG_05547, score=4, range=2374-2392, strand=-1

target  5’ CUGAG-GAAGCAAUCGACUC 3’
            ::::  ::::::::::::
query   3’ AACUCGGUUCGUUAGCUGAA 5’

No results for JS16_t3136909
No results for JS16_t3136925
No results for JS16_t4033485
No results for JS16_t1344144
query=JS16_t0577933, target=FGSG_09834, score=3.5, range=584-603, strand=-1

target  5’ GUUGCCAGGAUCGUUCUUUG 3’
           :: .:: :::::.:::::::
query   3’ CAUUGG-CCUAGUAAGAAAC 5’

query=JS16_t0577933, target=FGSG_07816, score=3.5, range=233-250, strand=-1

query=JS16_t3200873, target=FGSG_07218, score=3, range=1657-1674, strand=1

target  5’ GAUACCAGCUUGGGCAUU 3’
           ::. ::::::::.::::.
query   3’ CUGGGGUCGAACUCGUAG 5’

query=JS16_t3200873, target=FGSG_02972, score=3, range=1741-1758, strand=1

target  5’ UACCCCAGCUUGCGCAUC 3’
            ::::::::::: :::::
query   3’ CUGGGGUCGAACUCGUAG 5’

query=JS16_t3200873, target=FGSG_02604, score=3, range=2119-2137, strand=-1

target  5’ GAUCCACAGCUUGAGUAUU 3’
           ::.:: :::::::::.::.
query   3’ CUGGG-GUCGAACUCGUAG 5’

query=JS16_t3200873, target=FGSG_03340, score=3, range=901-918, strand=-1

target  5’ AAUUCCAGCUUGAGCAUA 3’
            :..:::::::::::::
query   3’ CUGGGGUCGAACUCGUAG 5’

query=JS16_t3200873, target=FGSG_05919, score=3, range=1005-1022, strand=-1

target  5’ CCGCCCAGCUUGAGCAUC 3’
              :::::::::::::::
query   3’ CUGGGGUCGAACUCGUAG 5’

query=JS16_t3200873, target=FGSG_04232, score=3, range=1761-1779, strand=-1

target  5’ GACGCCCAGCUUGACCAUC 3’
           ::: :::::::::: ::::
query   3’ CUG-GGGUCGAACUCGUAG 5’

query=JS16_t3200873, target=FGSG_03415, score=3, range=146-163, strand=-1

target  5’ GGACACAGCUUGAGCAUU 3’
           :. : ::::::::::::.
query   3’ CUGGGGUCGAACUCGUAG 5’

query=JS16_t3200873, target=FGSG_09982, score=3.5, range=108-125, strand=1

target  5’ UAAUCCAGCUUGGGCAUC 3’
            : .::::::::.:::::
query   3’ CUGGGGUCGAACUCGUAG 5’

query=JS16_t3200873, target=FGSG_08703, score=3.5, range=3670-3687, strand=-1

target  5’ UGCUCCAGCUUGAGCGUU 3’
            .:.:::::::::::.:.
query   3’ CUGGGGUCGAACUCGUAG 5’

query=JS16_t3200873, target=FGSG_01370, score=3.5, range=610-627, strand=-1

target  5’ GCCCUUGGCUUGAGCAUC 3’
           : ::...:::::::::::
query   3’ CUGGGGUCGAACUCGUAG 5’

query=JS16_t3200873, target=FGSG_09064, score=3.5, range=1741-1758, strand=-1

target  5’ CACUCCAGCUUGAACAUC 3’
            ::.:::::::::: ::::
query   3’ CUGGGGUCGAACUCGUAG 5’

query=JS16_t3200873, target=FGSG_01211, score=3.5, range=1362-1379, strand=-1

target  5’ GACUCCAGCUUGAGGAUA 3’
           :::.:::::::::: ::
query   3’ CUGGGGUCGAACUCGUAG 5’

query=JS16_t3200873, target=FGSG_11472, score=3.5, range=2005-2022, strand=-1

target  5’ GAGCUCAGCAUGAGCAUC 3’
           :: :.:::: ::::::::
query   3’ CUGGGGUCGAACUCGUAG 5’

query=JS16_t3200873, target=FGSG_12353, score=3.5, range=931-948, strand=-1

target  5’ GACCGCACCUUGAGCAUU 3’
           :::: :: :::::::::.
query   3’ CUGGGGUCGAACUCGUAG 5’

query=JS16_t3200873, target=FGSG_06016, score=3.5, range=6030-6047, strand=-1

target  5’ CGGCCCAGCUUGAGCAUG 3’
            .  ::::::::::::::
query   3’ CUGGGGUCGAACUCGUAG 5’

query=JS16_t3200873, target=FGSG_03009, score=3.5, range=389-405, strand=-1
```

```
target  5' GUAAUCGGAUC-UUCUUUA 3'
           ::::.:::::: ::::::
query   3' CAUUGGCCUAGUAAGAAAC 5'

query=JS16_t0577933, target=FGSG_09095, score=3.5, range=693-710, strand=-1

target  5' GUGA-CGGACCAUUCUUUG 3'
           ::.: :::: :::::::::
query   3' CAUUGGCCUAGUAAGAAAC 5'

query=JS16_t0577933, target=FGSG_11123, score=3.5, range=1298-1315, strand=-1

target  5' GU-GCAGGAUCAUUCUUUA 3'
           :: .: ::::::::::::
query   3' CAUUGGCCUAGUAAGAAAC 5'

query=JS16_t0577933, target=FGSG_09212, score=4, range=1703-1721, strand=-1

target  5' CUGGCAGGGUCAUUCUUUG 3'
            :..: ::.::::::::::
query   3' CAUUGGCCUAGUAAGAAAC 5'

query=JS16_t0577933, target=FGSG_06233, score=4, range=908-926, strand=-1

target  5' GUAACCGGCUCAUUCGUUG 3'
           :::::::: :::::: :::
query   3' CAUUGGCCUAGUAAGAAAC 5'

query=JS16_t0577933, target=FGSG_08067, score=4, range=159-176, strand=-1

target  5' GU-GUCGGAUCGUUUUUUG 3'
           :: ..::::::.::.::::
query   3' CAUUGGCCUAGUAAGAAAC 5'

query=JS16_t0577933, target=FGSG_12282, score=4, range=247-265, strand=-1

target  5' UGGAUGGGAUCAUUCUUUG 3'
             .:. :::::::::::::
query   3' CAUUGGCCUAGUAAGAAAC 5'

query=JS16_t0577933, target=FGSG_01811, score=4, range=818-836, strand=-1

target  5' AUGGCGGGAUCAUUCUUUU 3'
            :..: ::::::::::::
query   3' CAUUGGCCUAGUAAGAAAC 5'

query=JS16_t0577933, target=FGSG_13999, score=4, range=1874-1892, strand=-1

target  5' GAAACCGGAUCAGUCUUUC 3'
           : :::::::::: :::::
query   3' CAUUGGCCUAGUAAGAAAC 5'

query=JS16_t2240946, target=FGSG_11912, score=4, range=657-676, strand=1

target  5' GAUCUCA-ACUGUCGCCUAUG 3'
           ::::: : ::::::::::: :
query   3' CUAGAAUGUGACAGCGGAUCC 5'

No results for JS16_t1344249
query=JS16_t0577943, target=FGSG_03415, score=3.5, range=64-82, strand=-1

target  5' UUCUUCGACCGCGGGUGCU 3'
           :.::: :::: ::::::::
query   3' AGGAAACUGGGGCCCACGA 5'

query=JS16_t0577943, target=FGSG_01832, score=4, range=104-123, strand=1

target  5' UCCUUCUGUCCCUGGGUGCU 3'
           ::::: :: :::.:::::::
query   3' AGGAA-ACUGGGGCCCACGA 5'

No results for JS16_t3137078
query=JS16_t2241017, target=FGSG_08721, score=4, range=416-438, strand=-1

target  5' AGCAGGUCGGGGACCAGCGUUAC 3'
           ::::::: ::::::::: ::::
query   3' UCGUCCACCCCCUGGUCCCAAUC 5'

No results for JS16_t0131192
No results for JS16_t4033646
No results for JS16_t3137160
No results for JS16_t1344375
No results for JS16_t0577967
No results for JS16_t1344530
query=JS16_t4033913, target=FGSG_00647, score=4, range=5605-5624, strand=1

target  5' AUUGGGUCCAAGUAUGAUGA 3'
           :::..: :::::::.::::
query   3' UAAUUCCGGUUCAUGCUACC 5'

query=JS16_t4033913, target=FGSG_03357, score=4, range=405-425, strand=1
```

```
target  5' GCCUCCAGCUUGA-CAUC 3'
           : :.:::::::::: ::::
query   3' CUGGGGUCGAACUCGUAG 5'

query=JS16_t3200873, target=FGSG_00038, score=3.5, range=346-364, strand=-1

target  5' CAUCAGCAGCUUGAGCAUC 3'
            :.:  :::::::::::::
query   3' CUGG-GGUCGAACUCGUAG 5'

query=JS16_t3200873, target=FGSG_13828, score=3.5, range=80-96, strand=-1

target  5' GA-CACAGUUUGAGCAUU 3'
           :: : :::.::::::::.
query   3' CUGGGGUCGAACUCGUAG 5'

query=JS16_t3200873, target=FGSG_04473, score=3.5, range=913-931, strand=-1

target  5' GACCGUAGCUUCGAGCAUC 3'
           :::: .::::: :::::::
query   3' CUGGGGUCGAA-CUCGUAG 5'

query=JS16_t3200873, target=FGSG_06133, score=4, range=1606-1623, strand=1

target  5' GACCCCGGCUUGGGUGUC 3'
           ::::::.:::::.:..::
query   3' CUGGGGUCGAACUCGUAG 5'

query=JS16_t3200873, target=FGSG_03227, score=4, range=705-722, strand=1

target  5' CGCCCCAGCUUGGGCGUU 3'
            .::::::::::.::.:.
query   3' CUGGGGUCGAACUCGUAG 5'

query=JS16_t3200873, target=FGSG_12689, score=4, range=1871-1889, strand=1

target  5' CACCUCAGCUUGAGCGAUU 3'
            :::.:::::::::: ::.
query   3' CUGGGGUCGAACUCG-UAG 5'

query=JS16_t3200873, target=FGSG_06158, score=4, range=242-259, strand=1

target  5' AAGCUCAGCUUGAGCGUU 3'
            : :.::::::::::.:.
query   3' CUGGGGUCGAACUCGUAG 5'

query=JS16_t3200873, target=FGSG_07422, score=4, range=1587-1605, strand=1

target  5' GACCCCCAGCUUGGGAAUC 3'
           :: ::::::::::.: :::
query   3' CU-GGGGUCGAACUCGUAG 5'

query=JS16_t3200873, target=FGSG_05143, score=4, range=413-430, strand=1

target  5' CACCCGAGCUUGAGCAUG 3'
            :::: :::::::::::
query   3' CUGGGGUCGAACUCGUAG 5'

query=JS16_t3200873, target=FGSG_06489, score=4, range=1517-1534, strand=1

target  5' AACCGCAGCUUGAGCUUC 3'
            ::: :::::::::: ::
query   3' CUGGGGUCGAACUCGUAG 5'

query=JS16_t3200873, target=FGSG_08157, score=4, range=153-169, strand=1

target  5' GA-CCCAGCUUGAGCAAA 3'
           :: :::::::::::::
query   3' CUGGGGUCGAACUCGUAG 5'

query=JS16_t3200873, target=FGSG_06528, score=4, range=1269-1285, strand=1

target  5' GA-UUCAGCUUGCGCAUC 3'
           :: ..::::::: :::::
query   3' CUGGGGUCGAACUCGUAG 5'

query=JS16_t3200873, target=FGSG_00928, score=4, range=566-582, strand=1

target  5' GAGUCCAGCUUG-GCAUU 3'
           :: .:::::::: ::::.
query   3' CUGGGGUCGAACUCGUAG 5'

query=JS16_t3200873, target=FGSG_11824, score=4, range=1620-1637, strand=-1

target  5' GAUUCCAGCUUGUGCGUC 3'
           ::..:::::::: ::.::
query   3' CUGGGGUCGAACUCGUAG 5'

query=JS16_t3200873, target=FGSG_04569, score=4, range=289-306, strand=-1

target  5' GAUCUCAGCUUGGACAUC 3'
           ::.:.::::::. ::::
```

```
target  5’ AUUAAGCCCAAGUAGUGAUGG 3’
           :::::: ::::::: .:::::
query   3’ UAAUUCCGGUUCAU-GCUACC 5’

query=JS16_t4033913, target=FGSG_09116, score=4, range=373-392, strand=1

target  5’ AUGAAGGCCAAGUACGAAGC 3’
           :: ::::::::::::: :
query   3’ UAAUUCCGGUUCAUGCUACC 5’

query=JS16_t4033913, target=FGSG_05891, score=4, range=1408-1427, strand=1

target  5’ AUUUCGACCAAGUACGAUGC 3’
           :::  : ::::::::::::
query   3’ UAAUUCCGGUUCAUGCUACC 5’

query=JS16_t4033913, target=FGSG_07053, score=4, range=3596-3615, strand=-1

target  5’ CAUCAGGCCAAGUGCGAUGG 3’
             : :::::::::.::::::
query   3’ UAAUUCCGGUUCAUGCUACC 5’

query=JS16_t4033913, target=FGSG_07212, score=4, range=1933-1952, strand=-1

target  5’ AAUAAAGCCAAGGACGAUGG 3’
           : ::: :::::: :::::::
query   3’ UAAUUCCGGUUCAUGCUACC 5’

query=JS16_t4033913, target=FGSG_04083, score=4, range=327-345, strand=-1

target  5’ AUCAA-GCCAAGUACGAUCG 3’
           :: :: :::::::::::: :
query   3’ UAAUUCCGGUUCAUGCUACC 5’

query=JS16_t4033913, target=FGSG_04588, score=4, range=1341-1359, strand=-1

target  5’ AUGAAGGCCAAGU-CGAUGC 3’
           :: :::::::::: :::::
query   3’ UAAUUCCGGUUCAUGCUACC 5’

query=JS16_t4033913, target=FGSG_00365, score=4, range=351-369, strand=-1

target  5’ AAUAAGGCCAAGU-CGGUGG 3’
           : ::::::::::: ::.:::
query   3’ UAAUUCCGGUUCAUGCUACC 5’

No results for JS16_t2241315
query=JS16_t0211036, target=FGSG_11224, score=4, range=1873-1892, strand=1

target  5’ UUG-GCUCUUCUUUUGGUACU 3’
           ::: :: :::::::.:::::
query   3’ AACACGCGAAGAAAGCCAUGU 5’

No results for JS16_t4033968
No results for JS16_t1344626
query=JS16_t3137445, target=FGSG_04139, score=2.5, range=1148-1166, strand=1

target  5’ UUGAGGAGGCCAAGGUGGU 3’
           .::::::.:::::.:::::
query   3’ GACUCCUUCGGUUUCACCA 5’

query=JS16_t3137445, target=FGSG_03006, score=2.5, range=158-177, strand=-1

target  5’ CUGGGGAAGCUCAAAGUGGU 3’
           :::.:::::: :::::::::
query   3’ GACUCCUUCG-GUUUCACCA 5’

query=JS16_t3137445, target=FGSG_06087, score=3, range=25-43, strand=-1

target  5’ CGUAGGAAGCCAAAGUGGC 3’
           :  :::::::::::::::
query   3’ GACUCCUUCGGUUUCACCA 5’

query=JS16_t3137445, target=FGSG_07908, score=4, range=278-295, strand=1

target  5’ CUGAGGAGGUC-AAGUGGU 3’
           :::::::.:.: :::::::
query   3’ GACUCCUUCGGUUUCACCA 5’

query=JS16_t3137445, target=FGSG_12678, score=4, range=331-348, strand=1

target  5’ CU-AGGAAGCCAUGGUGGU 3’
           :: ::::::::: .:::::
query   3’ GACUCCUUCGGUUUCACCA 5’

query=JS16_t3137445, target=FGSG_10095, score=4, range=1561-1578, strand=1

target  5’ CAUAGGAAGCC-AAGUGGU 3’
           :  :::::::: :::::::
query   3’ GACUCCUUCGGUUUCACCA 5’

query=JS16_t3137445, target=FGSG_09076, score=4, range=956-974, strand=-1

query   3’ CUGGGGUCGAACUCGUAG 5’

query=JS16_t3200873, target=FGSG_07229, score=4, range=564-582, strand=-1

target  5’ GACCUUGGGCUUGAGCAUC 3’
           ::::.. .:::::::::::
query   3’ CUGGGG-UCGAACUCGUAG 5’

query=JS16_t3200873, target=FGSG_09495, score=4, range=1896-1913, strand=-1

target  5’ UCUUCCAGUUUGAGCAUC 3’
             ..::::.:::::::::
query   3’ CUGGGGUCGAACUCGUAG 5’

query=JS16_t3200873, target=FGSG_06097, score=4, range=5464-5481, strand=-1

target  5’ AGCCUCAGCCUGAGCAUC 3’
            .::.:::: ::::::::
query   3’ CUGGGGUCGAACUCGUAG 5’

query=JS16_t3200873, target=FGSG_07820, score=4, range=4714-4731, strand=-1

target  5’ GACACCAGCUUGGGUAUA 3’
           ::: ::::::::.:.::
query   3’ CUGGGGUCGAACUCGUAG 5’

query=JS16_t3200873, target=FGSG_12595, score=4, range=289-306, strand=-1

target  5’ GAUCUCAGCAUGAGCAUG 3’
           ::.:.:::: :::::::
query   3’ CUGGGGUCGAACUCGUAG 5’

query=JS16_t3200873, target=FGSG_06689, score=4, range=543-561, strand=-1

target  5’ AGCCCCAGCUUGAAGCAUU 3’
            .:::::::::: :::::.
query   3’ CUGGGGUCGAAC-UCGUAG 5’

query=JS16_t3200873, target=FGSG_00977, score=4, range=829-845, strand=-1

target  5’ GACUCCAGCUUGA-UAUU 3’
           :::.:::::::::: .::.
query   3’ CUGGGGUCGAACUCGUAG 5’

query=JS16_t3200873, target=FGSG_00945, score=4, range=1285-1302, strand=-1

target  5’ GUCCCCAGCUUGCGCGUC 3’
           : :::::::::: ::.::
query   3’ CUGGGGUCGAACUCGUAG 5’

query=JS16_t3200873, target=FGSG_10627, score=4, range=1786-1804, strand=-1

target  5’ GACCCAUAGCUUGAGAAUC 3’
           ::::: .:::::::: :::
query   3’ CUGGG-GUCGAACUCGUAG 5’

query=JS16_t3200873, target=FGSG_06816, score=4, range=964-981, strand=-1

target  5’ GAGCUCAUCUUGAGCAUU 3’
           :: :.:: :::::::::.
query   3’ CUGGGGUCGAACUCGUAG 5’

query=JS16_t3200873, target=FGSG_06255, score=4, range=399-416, strand=-1

target  5’ GGCUGCAGCUUGACCAUC 3’
           :.:.  :::::::: ::::
query   3’ CUGGGGUCGAACUCGUAG 5’

query=JS16_t3200873, target=FGSG_02681, score=4, range=571-588, strand=-1

target  5’ GACACCACCUUGAGCAUA 3’
           ::: ::: :::::::::
query   3’ CUGGGGUCGAACUCGUAG 5’

query=JS16_t3200873, target=FGSG_01584, score=4, range=208-224, strand=-1

target  5’ GACCCAAGCUUGAG-AUC 3’
           ::::: :::::::: :::
query   3’ CUGGGGUCGAACUCGUAG 5’

No results for JS16_t2304913
query=JS16_t4097825, target=FGSG_08079, score=3, range=1001-1017, strand=1

target  5’ UGC-GAACUCCCGGUGUA 3’
           ::: :::::::::: :::
query   3’ ACGCCUUGAGGGCCUCAU 5’

query=JS16_t4097825, target=FGSG_09270, score=3.5, range=191-209, strand=-1

target  5’ UGGGGAACUCCCGGAAGUG 3’
           :: ::::::::::: :::.
query   3’ ACGCCUUGAGGGCC-UCAU 5’
```

```
target  5' CUGAGUAGGUCAAAGUGGC 3'
           ::::: :.:.::::::::
query   3' GACUCCUUCGGUUUCACCA 5'

query=JS16_t3137445, target=FGSG_09272, score=4, range=28-46, strand=-1

target  5' GCGUUGAAGCCAAAGUGGU 3'
             :  :::::::::::::
query   3' GACUCCUUCGGUUUCACCA 5'

query=JS16_t3137445, target=FGSG_03247, score=4, range=1001-1020, strand=-1

target  5' CUCGAUGAAGCCAAAGUUGU 3'
           :: :: ::::::::::: ::
query   3' GA-CUCCUUCGGUUUCACCA 5'

No results for JS16_t0796041
No results for JS16_t4034071
No results for JS16_t0283309
No results for JS16_t2241529
query=JS16_t3137617, target=FGSG_07815, score=4, range=665-684, strand=-1

target  5' CGACCCAAACUUGAAAUUUC 3'
           : :::  :::::.:::::::
query   3' GGUGGCAUUGAAUUUUAAAG 5'

query=JS16_t0796059, target=FGSG_02324, score=3, range=4965-4984, strand=-1

target  5' UGGAUGUAUCGGCUGCGAAC 3'
           : ::: ::::.:::::::::
query   3' AACUAAAUAGUCGACGCUUG 5'

query=JS16_t0305981, target=FGSG_01382, score=4, range=1750-1769, strand=1

target  5' AUGGUGUACACUACCCUGGU 3'
           :::::::::::::  :::::
query   3' UACCACAUGUGAUUUGACCA 5'

query=JS16_t0305981, target=FGSG_10478, score=4, range=767-785, strand=1

target  5' AUGUUG-ACACUAAGCUGGG 3'
           ::: :: :::::::.::::
query   3' UACCACAUGUGAUUUGACCA 5'

No results for JS16_t4034262
No results for JS16_t0182477
query=JS16_t0796071, target=FGSG_13878, score=3.5, range=21853-21872, strand=

target  5' AUUGAUACGUCGAAUGUGUA 3'
           :::::.:::.:::: :::::
query   3' UAACUGUGCGGCUUUCACAU 5'

query=JS16_t0796071, target=FGSG_06326, score=4, range=4609-4627, strand=1

target  5' AUU-AUGCGCCGAAAGAGUA 3'
           ::: :..::::::::: :::
query   3' UAACUGUGCGGCUUUCACAU 5'

query=JS16_t2241706, target=FGSG_03142, score=3.5, range=1035-1054, strand=-1

target  5' CCAUGGUUCUGAGGAUACGG 3'
           :::::: ::::::::: ::.
query   3' GGUACCCAGACUCCUAAGCU 5'

query=JS16_t2241706, target=FGSG_13789, score=4, range=279-298, strand=1

target  5' ACAUGAGUUUGAGGAUUUGA 3'
            :::: ::.::::::::.::
query   3' GGUACCCAGACUCCUAAGCU 5'

query=JS16_t2241706, target=FGSG_13878, score=4, range=23145-23164, strand=1

target  5' AAACGGAUCUGAGGAUUCGA 3'
             : :: :::::::::::::
query   3' GGUACCCAGACUCCUAAGCU 5'

query=JS16_t0335808, target=FGSG_00020, score=3.5, range=604-623, strand=-1

target  5' GUAUCAUCA-ACAGAGGGCAG 3'
           :: :::::: ::::::::::.
query   3' CAAAGUAGUGUGUCUCCCGUU 5'

query=JS16_t0578035, target=FGSG_04752, score=2.5, range=1686-1703, strand=-1

target  5' AUGAGCUUAUCGAUCACU 3'
           ::::.:: :::::::::
query   3' UACUUGACUAGCUAGUGA 5'

query=JS16_t0578035, target=FGSG_09785, score=2.5, range=598-615, strand=-1

target  5' AUAAGCUGAUCGGUCACU 3'
```

```
query=JS16_t4097825, target=FGSG_05411, score=4, range=261-278, strand=1

target  5' UGAGGAAUUCUUGGAGUA 3'
           :: ::::.::..::::::
query   3' ACGCCUUGAGGGCCUCAU 5'

query=JS16_t4097825, target=FGSG_07397, score=4, range=618-635, strand=1

target  5' UGCGGAACUCUCGGAAUC 3'
           ::::::::::.:::: :
query   3' ACGCCUUGAGGGCCUCAU 5'

query=JS16_t4097825, target=FGSG_10028, score=4, range=1663-1679, strand=1

target  5' UGCGGACCUCCC-GAGUA 3'
           :::::: ::::: :::::
query   3' ACGCCUUGAGGGCCUCAU 5'

query=JS16_t4097825, target=FGSG_11544, score=4, range=1377-1394, strand=1

target  5' UGGAGAGCUCCCGGAGUC 3'
           ::  ::.:::::::::
query   3' ACGCCUUGAGGGCCUCAU 5'

query=JS16_t4097825, target=FGSG_02079, score=4, range=898-914, strand=-1

target  5' UG-GGGACUCUUGGAGUA 3'
           :: ::.::::..::::::
query   3' ACGCCUUGAGGGCCUCAU 5'

query=JS16_t4097825, target=FGSG_00590, score=4, range=361-379, strand=-1

target  5' UGCGGAAAUCCUCGGAGUA 3'
           ::::::: ::: :::::::
query   3' ACGCCUUGAGG-GCCUCAU 5'

query=JS16_t4097825, target=FGSG_04995, score=4, range=838-856, strand=-1

target  5' UGACAGAGCUUCCGGAGUA 3'
           :: : ::.::.::::::::
query   3' AC-GCCUUGAGGGCCUCAU 5'

query=JS16_t4097825, target=FGSG_07379, score=4, range=1872-1888, strand=-1

target  5' UGAGGAACUCCU-GAGUA 3'
           :: ::::::::. :::::
query   3' ACGCCUUGAGGGCCUCAU 5'

query=JS16_t4097825, target=FGSG_02996, score=4, range=789-805, strand=-1

target  5' UGCCGAACUCUCGGA-UA 3'
           ::: ::::::.:::: ::
query   3' ACGCCUUGAGGGCCUCAU 5'

query=JS16_t1408708, target=FGSG_04263, score=2.5, range=3114-3131, strand=1

target  5' GCUUUUGGACGACAUCCA 3'
           : ::.:.:::::::::::
query   3' CUAAGAUCUGCUGUAGGU 5'

query=JS16_t1408708, target=FGSG_01371, score=2.5, range=1113-1130, strand=1

target  5' GGAUCUAGACGACAUCCU 3'
           :. ::::::::::::::
query   3' CUAAGAUCUGCUGUAGGU 5'

query=JS16_t1408708, target=FGSG_01320, score=2.5, range=2282-2299, strand=-1

target  5' GGUUCUGGACGACAUCCU 3'
           :.::::.::::::::::
query   3' CUAAGAUCUGCUGUAGGU 5'

query=JS16_t1408708, target=FGSG_01713, score=3, range=270-287, strand=1

target  5' GAAUCUGGACGACAUCCU 3'
           :: :::.::::::::::
query   3' CUAAGAUCUGCUGUAGGU 5'

query=JS16_t1408708, target=FGSG_05255, score=3, range=961-977, strand=1

target  5' GAUU-UGGAUGACAUCCA 3'
           :::: :.::.::::::::
query   3' CUAAGAUCUGCUGUAGGU 5'

query=JS16_t1408708, target=FGSG_11690, score=3, range=3870-3887, strand=1

target  5' UAUCCUAGACGACAUCCU 3'
            :: :::::::::::::
query   3' CUAAGAUCUGCUGUAGGU 5'

query=JS16_t1408708, target=FGSG_11364, score=3.5, range=358-376, strand=1
```

```
           :: :.:::::::.:::::
query   3' UACUUGACUAGCUAGUGA 5'

query=JS16_t0578035, target=FGSG_08209, score=3, range=8499-8515, strand=-1

target  5' GUG-GCUGGUCGAUCACU 3'
           .:: .:::.:::::::::
query   3' UACUUGACUAGCUAGUGA 5'

query=JS16_t0578035, target=FGSG_11480, score=3.5, range=1459-1475, strand=1

target  5' AUG-GCUGGUCGAUCGCU 3'
           ::: .:::.::::::.::
query   3' UACUUGACUAGCUAGUGA 5'

query=JS16_t0578035, target=FGSG_01575, score=3.5, range=466-483, strand=-1

target  5' GUGAACUGAUCGAUGACC 3'
           .::::::::::::: ::
query   3' UACUUGACUAGCUAGUGA 5'

query=JS16_t0578035, target=FGSG_01453, score=3.5, range=219-236, strand=-1

target  5' AUGCGCUGAUUGAUCACA 3'
           ::: .:::::.::::::
query   3' UACUUGACUAGCUAGUGA 5'

query=JS16_t0578035, target=FGSG_06077, score=3.5, range=1201-1218, strand=-1

target  5' AGGCGCUGAUUGAUCACU 3'
           : : .:::::.:::::::
query   3' UACUUGACUAGCUAGUGA 5'

query=JS16_t0578035, target=FGSG_01883, score=3.5, range=541-557, strand=-1

target  5' UUGAGCUGAUC-AUCACU 3'
            :::.:::::: ::::::
query   3' UACUUGACUAGCUAGUGA 5'

query=JS16_t0578035, target=FGSG_03947, score=4, range=365-382, strand=1

target  5' AUCAAUUGAUCGAUGACU 3'
           :: ::.:::::::: :::
query   3' UACUUGACUAGCUAGUGA 5'

query=JS16_t0578035, target=FGSG_05800, score=4, range=26-43, strand=1

target  5' AUCAACUGAUUGAUAACU 3'
           :: :::::::.::: :::
query   3' UACUUGACUAGCUAGUGA 5'

query=JS16_t0578035, target=FGSG_07284, score=4, range=2468-2486, strand=1

target  5' AUGAAGUGAUUGAUCAACU 3'
           ::::: ::::.:::: :::
query   3' UACUUGACUAGCUAG-UGA 5'

query=JS16_t0578035, target=FGSG_03952, score=4, range=749-767, strand=1

target  5' AUGACCUGAUUGAUCAACU 3'
           :::: :::::.:::: :::
query   3' UACUUGACUAGCUAG-UGA 5'

query=JS16_t0578035, target=FGSG_02836, score=4, range=290-306, strand=1

target  5' AGGAACUGGUCGA-CACU 3'
           : ::::::.:::: ::::
query   3' UACUUGACUAGCUAGUGA 5'

query=JS16_t0578035, target=FGSG_09754, score=4, range=52-70, strand=1

target  5' AUGACUCUCAUCGAUCACU 3'
           ::::  :: ::::::::::
query   3' UACU-UGACUAGCUAGUGA 5'

query=JS16_t0578035, target=FGSG_07168, score=4, range=404-420, strand=1

target  5' AAGACCUGAUCGAUC-CU 3'
           : :: :::::::::: ::
query   3' UACUUGACUAGCUAGUGA 5'

query=JS16_t0578035, target=FGSG_06479, score=4, range=1383-1400, strand=-1

target  5' GUGGACUGGUCGGUCACG 3'
           .::.::::.:::.::::
query   3' UACUUGACUAGCUAGUGA 5'

query=JS16_t0578035, target=FGSG_04033, score=4, range=1156-1174, strand=-1

target  5' AUGAUACUGGUCGAUCGUU 3'
           :::: ::::.:::::::..:
query   3' UACU-UGACUAGCUAGUGA 5'

target  5' AAUUUUGAGACGACAUCCA 3'
            :::.: ::::::::::::
query   3' CUAAGA-UCUGCUGUAGGU 5'

query=JS16_t1408708, target=FGSG_06555, score=3.5, range=163-179, strand=1

target  5' GAUUC-AGACGAUAUCCG 3'
           ::::: ::::::.::::.
query   3' CUAAGAUCUGCUGUAGGU 5'

query=JS16_t1408708, target=FGSG_10620, score=3.5, range=606-623, strand=1

target  5' CGGUCUAGACGACAUCCU 3'
            . ::::::::::::::
query   3' CUAAGAUCUGCUGUAGGU 5'

query=JS16_t1408708, target=FGSG_02970, score=3.5, range=119-137, strand=-1

target  5' GGAUCUAGACGAGCAUCCA 3'
           :. ::::::::: ::::::
query   3' CUAAGAUCUGCU-GUAGGU 5'

query=JS16_t1408708, target=FGSG_02906, score=4, range=411-428, strand=1

target  5' GAUUCUGGACCACAUUCA 3'
           ::::::.::: ::::.::
query   3' CUAAGAUCUGCUGUAGGU 5'

query=JS16_t1408708, target=FGSG_05322, score=4, range=3258-3275, strand=1

target  5' AAUUCUUGAUGACAUCCA 3'
            ::::: ::.::::::::
query   3' CUAAGAUCUGCUGUAGGU 5'

query=JS16_t1408708, target=FGSG_03137, score=4, range=696-713, strand=1

target  5' GAUUCUAGACGAGGUCCU 3'
           :::::::::::: .:::
query   3' CUAAGAUCUGCUGUAGGU 5'

query=JS16_t1408708, target=FGSG_08715, score=4, range=1029-1046, strand=1

target  5' AAUUAUGGACGACGUCCA 3'
            ::: :.::::::.::::
query   3' CUAAGAUCUGCUGUAGGU 5'

query=JS16_t1408708, target=FGSG_01310, score=4, range=2212-2229, strand=1

target  5' GAAUCUAGACGACCUUCA 3'
           :: :::::::::: :.::
query   3' CUAAGAUCUGCUGUAGGU 5'

query=JS16_t1408708, target=FGSG_10639, score=4, range=918-935, strand=1

target  5' GCUUCUGGACGACAUACA 3'
           : ::::.:::::::: ::
query   3' CUAAGAUCUGCUGUAGGU 5'

query=JS16_t1408708, target=FGSG_06097, score=4, range=3618-3635, strand=1

target  5' GAUACUAGACGACAUCAU 3'
           ::: ::::::::::::
query   3' CUAAGAUCUGCUGUAGGU 5'

query=JS16_t1408708, target=FGSG_08476, score=4, range=702-718, strand=1

target  5' UGUUCUAGACGAC-UCCG 3'
            .:::::::::::: :::.
query   3' CUAAGAUCUGCUGUAGGU 5'

query=JS16_t1408708, target=FGSG_04887, score=4, range=507-524, strand=1

target  5' GUGGCUAGACGAUAUCCA 3'
           :   ::::::::.:::::
query   3' CUAAGAUCUGCUGUAGGU 5'

query=JS16_t1408708, target=FGSG_10526, score=4, range=1567-1583, strand=1

target  5' GAUAC-AGACGACAUCUA 3'
           ::: : ::::::::::.:
query   3' CUAAGAUCUGCUGUAGGU 5'

query=JS16_t1408708, target=FGSG_10566, score=4, range=2548-2564, strand=1

target  5' GA-UGUAGACGACAUUCU 3'
           :: : ::::::::::.:
query   3' CUAAGAUCUGCUGUAGGU 5'

query=JS16_t1408708, target=FGSG_11429, score=4, range=150-166, strand=1

target  5' GACUGUAGACGACA-CCA 3'
```

query=JS16_t0578035, target=FGSG_04544, score=4, range=594-611, strand=-1

target 5' AUGAAUUGACCGAUCACA 3'
           :::::.::: :::::::
query   3' UACUUGACUAGCUAGUGA 5'

query=JS16_t0578035, target=FGSG_04228, score=4, range=193-209, strand=-1

target 5' AUGAACUGGUCGAU-GCU 3'
           ::::::::.::::: .::
query   3' UACUUGACUAGCUAGUGA 5'

query=JS16_t0578035, target=FGSG_00647, score=4, range=3049-3066, strand=-1

target 5' GGGAGCCGAUCGAUCACU 3'
           . ::.: :::::::::::
query   3' UACUUGACUAGCUAGUGA 5'

query=JS16_t0578035, target=FGSG_05135, score=4, range=420-437, strand=-1

target 5' ACGAUCUGAUCGAUCAUC 3'
           : :: :::::::::::.
query   3' UACUUGACUAGCUAGUGA 5'

query=JS16_t0578035, target=FGSG_03744, score=4, range=805-821, strand=-1

target 5' AUG-ACUGAUCGGCCACU 3'
           ::: ::::::::. ::::
query   3' UACUUGACUAGCUAGUGA 5'

query=JS16_t0578035, target=FGSG_08009, score=4, range=1339-1355, strand=-1

target 5' AUGAUCUGGUCGAU-ACU 3'
           :::: :::.::::: :::
query   3' UACUUGACUAGCUAGUGA 5'

query=JS16_t0578035, target=FGSG_00626, score=4, range=183-200, strand=-1

target 5' AUGCCCUGAUCGAUAACU 3'
           ::: ::::::::: :::
query   3' UACUUGACUAGCUAGUGA 5'

query=JS16_t0578035, target=FGSG_09526, score=4, range=3406-3423, strand=-1

target 5' ACUAUCUGAUCGAUCACC 3'
           : : :::::::::::
query   3' UACUUGACUAGCUAGUGA 5'

No results for JS16_t1345046
No results for JS16_t3137932
query=JS16_t0254720, target=FGSG_00475, score=3, range=1685-1704, strand=-1

target 5' UUGUACCACUGUUUCAUCUG 3'
           ::: ::::::.:::::.:::
query   3' AACUUGGUGAUAAAGUGGAC 5'

No results for JS16_t0796102
query=JS16_t0254723, target=FGSG_13433, score=3, range=690-707, strand=1

target 5' CGAGUUUUUACCGACUUU 3'
            :::::.:::::.:::::
query   3' UCUCAAGAAUGGUUGAAA 5'

query=JS16_t0254723, target=FGSG_12737, score=3, range=598-615, strand=1

target 5' AAAGUUCUUGCCAGCUUU 3'
           : :::::::.:::.::::
query   3' UCUCAAGAAUGGUUGAAA 5'

query=JS16_t0254723, target=FGSG_02272, score=3, range=72-90, strand=1

target 5' AGAAGAUCUUGCCAACUUU 3'
           :: :: ::::.::::::::
query   3' UC-UCAAGAAUGGUUGAAA 5'

query=JS16_t0254723, target=FGSG_00819, score=3, range=2371-2388, strand=-1

target 5' AGACAUCUUAUCAACUUU 3'
           ::: :::::.:::::::
query   3' UCUCAAGAAUGGUUGAAA 5'

query=JS16_t0254723, target=FGSG_09291, score=3, range=67-83, strand=-1

target 5' AGAG-UCUUGCCAACUUG 3'
           :::: ::::.:::::::
query   3' UCUCAAGAAUGGUUGAAA 5'

query=JS16_t0254723, target=FGSG_11703, score=3.5, range=5398-5415, strand=1

target 5' AGGGUUCUUCCCGACUUU 3'
           ::.:::::: ::.:::::

           :: : ::::::::: :::
query   3' CUAAGAUCUGCUGUAGGU 5'

query=JS16_t1408708, target=FGSG_06563, score=4, range=1531-1547, strand=1

target 5' GACUCU-GAUGACAUCCA 3'
           :: ::: ::.::::::::
query   3' CUAAGAUCUGCUGUAGGU 5'

query=JS16_t1408708, target=FGSG_13783, score=4, range=12364-12381, strand=-1

target 5' CAUUUUAGGCGACAUUCG 3'
            :::.:::.::::::.:.
query   3' CUAAGAUCUGCUGUAGGU 5'

query=JS16_t1408708, target=FGSG_11629, score=4, range=755-772, strand=-1

target 5' CAUUCUCGGCGACAUCCA 3'
            ::::: :.:::::::::
query   3' CUAAGAUCUGCUGUAGGU 5'

query=JS16_t1408708, target=FGSG_08371, score=4, range=425-442, strand=-1

target 5' GUUUCUUGACGGCAUCCA 3'
           : :::: ::::.::::::
query   3' CUAAGAUCUGCUGUAGGU 5'

query=JS16_t1408708, target=FGSG_01000, score=4, range=398-416, strand=-1

target 5' CAGUCAUAGACGACAUCCU 3'
            : :: :::::::::::
query   3' CUAAG-AUCUGCUGUAGGU 5'

No results for JS16_t3200960
No results for JS16_t1408758
query=JS16_t2305014, target=FGSG_10291, score=3, range=186-205, strand=-1

target 5' GCUGCCGCAAUUGUUCCUUU 3'
           ::::::::::.::.:::::
query   3' CGACGGCGUUGACGAGGAAC 5'

query=JS16_t2305014, target=FGSG_03711, score=3, range=1509-1528, strand=-1

target 5' CCUGUUUCAACUGCUCCUUG 3'
            :::.. ::::::::::::
query   3' CGACGGCGUUGACGAGGAAC 5'

query=JS16_t2305014, target=FGSG_08948, score=3.5, range=2286-2306, strand=-1

target 5' GCUGCUGCAACUGCUGCCUUC 3'
           :::::.::::::::: ::::
query   3' CGACGGCGUUGACGA-GGAAC 5'

query=JS16_t2305014, target=FGSG_06385, score=3.5, range=1023-1042, strand=-1

target 5' ACUGCUUCAGCUGCUCCUUG 3'
            ::::. ::.:::::::::
query   3' CGACGGCGUUGACGAGGAAC 5'

query=JS16_t2305014, target=FGSG_06832, score=3.5, range=1045-1063, strand=-1

target 5' GCUGCUGCGACUG-UCCUUG 3'
           :::::.::.:::: ::::::
query   3' CGACGGCGUUGACGAGGAAC 5'

query=JS16_t2305014, target=FGSG_08285, score=4, range=1171-1190, strand=1

target 5' UCGGCCUCAACUGCUUCUUG 3'
            : ::: ::::::::.::::
query   3' CGACGGCGUUGACGAGGAAC 5'

query=JS16_t2305014, target=FGSG_08785, score=4, range=1237-1256, strand=1

target 5' GAUGCCAUAACUGCUCCUUC 3'
           : :::: .:::::::::::
query   3' CGACGGCGUUGACGAGGAAC 5'

query=JS16_t2305014, target=FGSG_12322, score=4, range=1066-1084, strand=1

target 5' CCUG-CGCAGCUGCUCCUUU 3'
            ::: ::::.:::::::::
query   3' CGACGGCGUUGACGAGGAAC 5'

query=JS16_t2305014, target=FGSG_04644, score=4, range=1406-1424, strand=1

target 5' GCU-ACGCAACUGUUCCUUC 3'
           ::: ::::::::.:::::
query   3' CGACGGCGUUGACGAGGAAC 5'

query=JS16_t2305014, target=FGSG_13198, score=4, range=370-388, strand=1

target 5' GCU-CCCCAACUACUCCUUG 3'

```
query   3’ UCUCAAGAAUGGUUGAAA 5’

query=JS16_t0254723, target=FGSG_03683, score=3.5, range=609-627, strand=1

target  5’ GAAGUGUCUUGCCAACUUU 3’
           . ::: ::::.::::::::
query   3’ UCUCA-AGAAUGGUUGAAA 5’

query=JS16_t0254723, target=FGSG_12118, score=4, range=2275-2292, strand=1

target  5’ ACAGUUUUUAUCAACUUG 3’
           : ::::.:::.::::::
query   3’ UCUCAAGAAUGGUUGAAA 5’

query=JS16_t0254723, target=FGSG_13187, score=4, range=375-392, strand=1

target  5’ AGACUUUUUGCCAACUUC 3’
           ::: ::.::.:::::::
query   3’ UCUCAAGAAUGGUUGAAA 5’

query=JS16_t0254723, target=FGSG_10217, score=4, range=720-738, strand=1

target  5’ AGAGGAUCUUGUCAACUUU 3’
           ::: : ::::..:::::::
query   3’ UCU-CAAGAAUGGUUGAAA 5’

query=JS16_t0254723, target=FGSG_00729, score=4, range=334-351, strand=1

target  5’ AAUCUUCUUGCCAACUUU 3’
           :   :::::.::::::::
query   3’ UCUCAAGAAUGGUUGAAA 5’

query=JS16_t0254723, target=FGSG_05434, score=4, range=694-711, strand=1

target  5’ AAAGCUCUUGCCAACUUC 3’
           : :: ::::.:::::::
query   3’ UCUCAAGAAUGGUUGAAA 5’

query=JS16_t0254723, target=FGSG_07419, score=4, range=168-186, strand=1

target  5’ UCAGCUUCUUACCAACUUA 3’
             :: :::::::::::::
query   3’ UCUC-AAGAAUGGUUGAAA 5’

query=JS16_t0254723, target=FGSG_11742, score=4, range=5740-5757, strand=1

target  5’ AAAUAUCUUACCAACUUC 3’
           : :  ::::::::::::
query   3’ UCUCAAGAAUGGUUGAAA 5’

query=JS16_t0254723, target=FGSG_00697, score=4, range=23-39, strand=1

target  5’ AUCGUUCUUA-CAACUUU 3’
           :  ::::::: :::::::
query   3’ UCUCAAGAAUGGUUGAAA 5’

query=JS16_t0254723, target=FGSG_05126, score=4, range=500-516, strand=1

target  5’ CCAGUUCUU-CCAACUUU 3’
             ::::::: ::::::::
query   3’ UCUCAAGAAUGGUUGAAA 5’

query=JS16_t0254723, target=FGSG_05713, score=4, range=155-172, strand=-1

target  5’ AGAGGUCUUUCCAGCUUU 3’
           :::: :::: :::.::::
query   3’ UCUCAAGAAUGGUUGAAA 5’

query=JS16_t0254723, target=FGSG_13973, score=4, range=60-77, strand=-1

target  5’ AGAGAUCUGACCAGCUUU 3’
           :::: ::: ::::.::::
query   3’ UCUCAAGAAUGGUUGAAA 5’

query=JS16_t0254723, target=FGSG_10394, score=4, range=787-805, strand=-1

target  5’ AGACGUUCUUGACAACUUU 3’
           ::: :::::::. :::::::
query   3’ UCU-CAAGAAUGGUUGAAA 5’

query=JS16_t0254723, target=FGSG_10059, score=4, range=1192-1209, strand=-1

target  5’ CAAGUUCUUUCCAACUUU 3’
             ::::::: ::::::::
query   3’ UCUCAAGAAUGGUUGAAA 5’

query=JS16_t0254723, target=FGSG_08165, score=4, range=765-782, strand=-1

target  5’ GGGCUUCUUCCCAACUUU 3’
           .:. ::::: ::::::::
query   3’ UCUCAAGAAUGGUUGAAA 5’
```

```
           ::: :: ::::: :::::::
query   3’ CGACGGCGUUGACGAGGAAC 5’

query=JS16_t2305014, target=FGSG_13646, score=4, range=1065-1084, strand=-1

target  5’ UCUCCCGCAAUUGCUCCUUC 3’
            :: ::::::.::::::::
query   3’ CGACGGCGUUGACGAGGAAC 5’

query=JS16_t2305014, target=FGSG_06007, score=4, range=469-487, strand=-1

target  5’ UCU-UUGCAACUGUUCCUUG 3’
            :: ..:::::::.::::::
query   3’ CGACGGCGUUGACGAGGAAC 5’

query=JS16_t2305014, target=FGSG_04635, score=4, range=1089-1107, strand=-1

target  5’ GCUG-GGCAAUUGCUUCUUG 3’
           ::::  ::::.::::.::::
query   3’ CGACGGCGUUGACGAGGAAC 5’

query=JS16_t2305014, target=FGSG_02014, score=4, range=1206-1224, strand=-1

target  5’ CUUGUCG-AACUGCUCCUUG 3’
            .::.:: ::::::::::::
query   3’ CGACGGCGUUGACGAGGAAC 5’

query=JS16_t4097942, target=FGSG_02316, score=3, range=212-230, strand=1

target  5’ ACUGGCUGUAUUGCAAGAU 3’
           ::::: .:::.::::::::
query   3’ UGACCAGCAUGACGUUCUA 5’

query=JS16_t4097942, target=FGSG_06160, score=3, range=1514-1532, strand=1

target  5’ UCUGGUGGUACUGCAAGAU 3’
            ::::: ::::::::::::
query   3’ UGACCAGCAUGACGUUCUA 5’

query=JS16_t4097942, target=FGSG_12950, score=3, range=200-217, strand=-1

target  5’ GUUGGUCG-ACUGCAAGAU 3’
           ..:::::: ::::::::::
query   3’ UGACCAGCAUGACGUUCUA 5’

query=JS16_t4097942, target=FGSG_02324, score=3.5, range=2407-2424, strand=1

target  5’ GCU-GUCCUACUGCAAGAU 3’
           .:: ::: :::::::::::
query   3’ UGACCAGCAUGACGUUCUA 5’

query=JS16_t4097942, target=FGSG_02644, score=4, range=1159-1178, strand=1

target  5’ ACUGGUCGUAUUGUCAAGAA 3’
           ::::::::::.:: :::::
query   3’ UGACCAGCAUGAC-GUUCUA 5’

query=JS16_t4097942, target=FGSG_08930, score=4, range=894-913, strand=-1

target  5’ ACUGGGUCGUGCUGUGAGAU 3’
           ::: ::::::.:::..::::
query   3’ UGA-CCAGCAUGACGUUCUA 5’

query=JS16_t4097942, target=FGSG_00780, score=4, range=157-175, strand=-1

target  5’ ACAGGUUGUACUGCAUGAU 3’
           :: :::.:::::::: :::
query   3’ UGACCAGCAUGACGUUCUA 5’

query=JS16_t4097942, target=FGSG_05220, score=4, range=1105-1123, strand=-1

target  5’ CCUCAUCGUACUGCAAGAA 3’
            ::  :::::::::::::
query   3’ UGACCAGCAUGACGUUCUA 5’

No results for JS16_t3201074
No results for JS16_t2305051
query=JS16_t1408849, target=FGSG_10534, score=3, range=1150-1169, strand=-1

target  5’ AUCUUGCUAUCAAACGGCAU 3’
           :  .:.::::::::::::::
query   3’ UCCGAUGAUAGUUUGCCGUA 5’

query=JS16_t1408849, target=FGSG_02467, score=3.5, range=938-957, strand=1

target  5’ AGGAUGCUAUCAAAUGGCGU 3’
           ::: :.::::::::.:::.:
query   3’ UCCGAUGAUAGUUUGCCGUA 5’

query=JS16_t4097977, target=FGSG_08849, score=3, range=214-231, strand=1

target  5’ AUUUCAGAUAUGACUUUA 3’
```

```
query=JS16_t0254723, target=FGSG_07128, score=4, range=1777-1793, strand=-1

target  5' AGAG-UCUUGCCAGCUUC 3'
           :::: ::::.:::.:::
query   3' UCUCAAGAAUGGUUGAAA 5'

query=JS16_t0254723, target=FGSG_12492, score=4, range=1552-1570, strand=-1

target  5' AGUAGUUCUCACCAACUUC 3'
           :: :::::: ::::::::
query   3' UC-UCAAGAAUGGUUGAAA 5'

query=JS16_t0254723, target=FGSG_00077, score=4, range=154-171, strand=-1

target  5' AUAUUUCUUGCCAACUUG 3'
           : : :::::.::::::::
query   3' UCUCAAGAAUGGUUGAAA 5'

No results for JS16_t2241949
No results for JS16_t4034563
No results for JS16_t0305999
query=JS16_t3138093, target=FGSG_05721, score=1.5, range=1675-1692, strand=-1

target  5' GUACUCGGGGUCAAAGGG 3'
           :.::.:::::::::::::.
query   3' CGUGGGCCCCAGUUUCCU 5'

query=JS16_t3138093, target=FGSG_05572, score=3, range=37-54, strand=-1

target  5' GCACUCGGAGUCAAAGGG 3'
           ::::.::: ::::::::.
query   3' CGUGGGCCCCAGUUUCCU 5'

query=JS16_t3138093, target=FGSG_01216, score=3.5, range=499-516, strand=-1

target  5' GUAUCCGGUGUCAAAGGG 3'
           :.:.:::: ::::::::.
query   3' CGUGGGCCCCAGUUUCCU 5'

query=JS16_t3138093, target=FGSG_01425, score=3.5, range=1354-1371, strand=-1

target  5' GGAGACGGGGUCAAAGGG 3'
           : :  ::::::::::::.
query   3' CGUGGGCCCCAGUUUCCU 5'

query=JS16_t3138093, target=FGSG_09687, score=4, range=1184-1202, strand=1

target  5' CCGUCGCGGGGUCAAAGGC 3'
            :..: ::::::::::::
query   3' CGUGG-GCCCCAGUUUCCU 5'

query=JS16_t3138093, target=FGSG_13921, score=4, range=158-174, strand=1

target  5' UCACCCGGGG-CAAAGGC 3'
            ::::::::: ::::::
query   3' CGUGGGCCCCAGUUUCCU 5'

query=JS16_t3138093, target=FGSG_06539, score=4, range=223-239, strand=1

target  5' GAAGCC-GGGUCAAAGGA 3'
           : : :: ::::::::::
query   3' CGUGGGCCCCAGUUUCCU 5'

query=JS16_t3138093, target=FGSG_07070, score=4, range=1124-1142, strand=1

target  5' GUGGCCGGGGUCCAAAGGA 3'
           :.. ::::::: :::::::
query   3' CGUGGGCCCCA-GUUUCCU 5'

query=JS16_t3138093, target=FGSG_01621, score=4, range=407-425, strand=1

target  5' GCGCCUGGUGGUCAAAGGU 3'
           ::.::.:: :::::::::
query   3' CGUGGGCC-CCAGUUUCCU 5'

query=JS16_t3138093, target=FGSG_10817, score=4, range=405-422, strand=-1

target  5' GCACCCGGGGUCGGGGGC 3'
           ::::::::::::...::
query   3' CGUGGGCCCCAGUUUCCU 5'

query=JS16_t3138093, target=FGSG_10799, score=4, range=804-821, strand=-1

target  5' GCAUCCUGGGUCGAAGGG 3'
           :::.:: :::::.::::.
query   3' CGUGGGCCCCAGUUUCCU 5'

query=JS16_t3138093, target=FGSG_03686, score=4, range=1354-1371, strand=-1

target  5' UCGCUCGGGGUCGAAGGU 3'
            :.:.:::::::.::::
query   3' CGUGGGCCCCAGUUUCCU 5'
```

```
           ::::::::.: :::::::
query   3' UAAAGUCUGUUCUGAAAU 5'

query=JS16_t4097977, target=FGSG_08539, score=3, range=1188-1206, strand=1

target  5' GUUUGAAGACAAGACUUUG 3'
           .:::  ::::::::::::.
query   3' UAAA-GUCUGUUCUGAAAU 5'

query=JS16_t4097977, target=FGSG_11303, score=3.5, range=967-984, strand=1

target  5' UUUUCAGACGAGGCUUUG 3'
            ::::::::.::.::::.
query   3' UAAAGUCUGUUCUGAAAU 5'

query=JS16_t4097977, target=FGSG_04970, score=3.5, range=1201-1218, strand=1

target  5' AUUUCAUACAAGGCUUUG 3'
           :::::: :::::.::::.
query   3' UAAAGUCUGUUCUGAAAU 5'

query=JS16_t4097977, target=FGSG_05554, score=3.5, range=109-126, strand=1

target  5' GUUUCAGAAAAGACUUUC 3'
           .::::::: ::::::::
query   3' UAAAGUCUGUUCUGAAAU 5'

query=JS16_t4097977, target=FGSG_11401, score=3.5, range=707-724, strand=1

target  5' AUUUGAGACACGACUUUG 3'
           :::: ::::: ::::::.
query   3' UAAAGUCUGUUCUGAAAU 5'

query=JS16_t4097977, target=FGSG_07468, score=3.5, range=598-615, strand=1

target  5' AUUCGAGGCAAGACUUUG 3'
           :::  ::.:::::::::.
query   3' UAAAGUCUGUUCUGAAAU 5'

query=JS16_t4097977, target=FGSG_13526, score=3.5, range=439-456, strand=1

target  5' GAGUCAGACAAGACUUUC 3'
           .   ::::::::::::::
query   3' UAAAGUCUGUUCUGAAAU 5'

query=JS16_t4097977, target=FGSG_07549, score=3.5, range=3114-3131, strand=-1

target  5' GCUUCAGACAAGAUUUUC 3'
           .  :::::::::::.:::
query   3' UAAAGUCUGUUCUGAAAU 5'

query=JS16_t4097977, target=FGSG_02390, score=3.5, range=166-183, strand=-1

target  5' AUUUAAGACAAGCCUUUG 3'
           :::: ::::::: ::::.
query   3' UAAAGUCUGUUCUGAAAU 5'

query=JS16_t4097977, target=FGSG_11108, score=3.5, range=681-697, strand=-1

target  5' GUUUUA-ACAAGACUUUG 3'
           .:::.: ::::::::::.
query   3' UAAAGUCUGUUCUGAAAU 5'

query=JS16_t4097977, target=FGSG_08752, score=4, range=2425-2442, strand=1

target  5' AUGUUGGACGAGACUUUG 3'
           :: :..:::.:::::::.
query   3' UAAAGUCUGUUCUGAAAU 5'

query=JS16_t4097977, target=FGSG_10009, score=4, range=2943-2961, strand=1

target  5' AUUCUUGGACGAGACUUUG 3'
           ::: :..:::.:::::::.
query   3' UAA-AGUCUGUUCUGAAAU 5'

query=JS16_t4097977, target=FGSG_03575, score=4, range=359-376, strand=1

target  5' AUCUCGGACAGGACUUUC 3'
           :: ::.::::.::::::
query   3' UAAAGUCUGUUCUGAAAU 5'

query=JS16_t4097977, target=FGSG_00291, score=4, range=559-577, strand=1

target  5' GUUACAGACAAGGACUUUG 3'
           .:: ::::::: ::::::.
query   3' UAAAGUCUGUU-CUGAAAU 5'

query=JS16_t4097977, target=FGSG_07847, score=4, range=1166-1183, strand=1

target  5' CUAUCAGACAGGACUUUU 3'
            : :::::::.::::::
query   3' UAAAGUCUGUUCUGAAAU 5'
```

```
query=JS16_t3138093, target=FGSG_02872, score=4, range=1327-1344, strand=-1

target  5’ GCGCUCUGGGUCAAAGGU 3’
           ::.:.: ::::::::::
query   3’ CGUGGGCCCCAGUUUCCU 5’

query=JS16_t3138093, target=FGSG_00633, score=4, range=11296-11313, strand=-1

target  5’ AUAGUCGGGGUCGAAGGA 3’
            .: .:::::::.:::::
query   3’ CGUGGGCCCCAGUUUCCU 5’

query=JS16_t3138093, target=FGSG_08760, score=4, range=10-27, strand=-1

target  5’ GAACCCGGGGGCGAAGGA 3’
           : :::::::: :.:::::
query   3’ CGUGGGCCCCAGUUUCCU 5’

query=JS16_t3138093, target=FGSG_08710, score=4, range=193-211, strand=-1

target  5’ GCAAUUCGGGGCCAAAGGA 3’
           :: :..::::: :::::::
query   3’ CG-UGGGCCCCAGUUUCCU 5’

query=JS16_t3138093, target=FGSG_07066, score=4, range=793-809, strand=-1

target  5’ GCA-GCGGCGUCAAAGGA 3’
           :::  ::: :::::::::
query   3’ CGUGGGCCCCAGUUUCCU 5’

No results for JS16_t3138146
No results for JS16_t3138152
query=JS16_t0306004, target=FGSG_07344, score=3, range=1316-1332, strand=1

target  5’ AGGGUAGCC-GACCAACC 3’
           ::::::::: .:::::::
query   3’ UCCCAUCGGAUUGGUUGG 5’

query=JS16_t0306004, target=FGSG_04212, score=3, range=837-854, strand=-1

target  5’ CGUGUAGCCUGACCAACC 3’
            : :::::::.:::::::
query   3’ UCCCAUCGGAUUGGUUGG 5’

query=JS16_t0306004, target=FGSG_04954, score=4, range=249-266, strand=1

target  5’ AUGGAAGCCUAGCCAGCC 3’
           : :: ::::::.:::.::
query   3’ UCCCAUCGGAUUGGUUGG 5’

query=JS16_t0306004, target=FGSG_03514, score=4, range=545-563, strand=1

target  5’ AGGGAGGCCUAACCAUACU 3’
           :::: .::::::::: ::.
query   3’ UCCCAUCGGAUUGGU-UGG 5’

query=JS16_t0306004, target=FGSG_00827, score=4, range=671-688, strand=1

target  5’ AGUUCAGCCUGACCAACC 3’
           ::   :::::.:::::::
query   3’ UCCCAUCGGAUUGGUUGG 5’

query=JS16_t0306004, target=FGSG_01724, score=4, range=687-703, strand=1

target  5’ AAGGUA-CCUAACCAAUC 3’
           : :::: :::::::::.:
query   3’ UCCCAUCGGAUUGGUUGG 5’

query=JS16_t0306004, target=FGSG_07405, score=4, range=1337-1353, strand=1

target  5’ AGGGUAGCC-AGCCAGCC 3’
           ::::::::: :.:::.::
query   3’ UCCCAUCGGAUUGGUUGG 5’

query=JS16_t0306004, target=FGSG_01181, score=4, range=518-534, strand=-1

target  5’ AGGG-AGCUUGGCCAACC 3’
           :::: :::.:..::::::
query   3’ UCCCAUCGGAUUGGUUGG 5’

query=JS16_t0306004, target=FGSG_08635, score=4, range=962-979, strand=-1

target  5’ AGAGGAGUCUAACCAGCC 3’
           :: : ::.:::::::.::
query   3’ UCCCAUCGGAUUGGUUGG 5’

query=JS16_t0306004, target=FGSG_00863, score=4, range=933-950, strand=-1

target  5’ UGGGGAGCCUGACCAACA 3’
            ::: :::::.::::::
query   3’ UCCCAUCGGAUUGGUUGG 5’

query=JS16_t4097977, target=FGSG_04310, score=4, range=2251-2268, strand=1

target  5’ GGUACAGGCAAGACUUUG 3’
           .  : :::.::::::::::.
query   3’ UAAAGUCUGUUCUGAAAU 5’

query=JS16_t4097977, target=FGSG_02910, score=4, range=270-287, strand=-1

target  5’ UUUUCAGACAAGGCCUUA 3’
            ::::::::::.: :::
query   3’ UAAAGUCUGUUCUGAAAU 5’

query=JS16_t4097977, target=FGSG_13036, score=4, range=174-191, strand=-1

target  5’ ACGUCGGGCAAGACUUUA 3’
           :  ::.:.::::::::::
query   3’ UAAAGUCUGUUCUGAAAU 5’

query=JS16_t2305080, target=FGSG_11418, score=3, range=79-99, strand=-1

target  5’ AGCGAGAGCGGUUGUCGUGAC 3’
           ::::::::: .::::::::::
query   3’ UCGCUCUCGGUAACAGCACUG 5’

query=JS16_t2305080, target=FGSG_02894, score=4, range=81-101, strand=1

target  5’ AGCAGGAGCCAGUGUCGUGAU 3’
           ::: .:::::: ::::::::.
query   3’ UCGCUCUCGGUAACAGCACUG 5’

query=JS16_t2305080, target=FGSG_11995, score=4, range=94-114, strand=-1

target  5’ AGCGAGCGUGAUUGUCGUGAC 3’
           :::::: :. ::::::::::
query   3’ UCGCUCUCGGUAACAGCACUG 5’

No results for JS16_t1408896
query=JS16_t3201285, target=FGSG_07632, score=3, range=622-640, strand=-1

target  5’ CUAGCGCAUAACCCAGAGG 3’
           :: ::::::: ::::::::
query   3’ GAACGCGUAUGGGGUCUCC 5’

query=JS16_t3201285, target=FGSG_04256, score=3.5, range=1297-1315, strand=1

target  5’ UUCGCGUAUAUCCCAGAGG 3’
           .: :::.:::.::::::::
query   3’ GAACGCGUAUGGGGUCUCC 5’

query=JS16_t3201285, target=FGSG_00781, score=3.5, range=226-243, strand=1

target  5’ UUUGC-CAUGCCCCAGAGU 3’
           .:::: :::.::::::::
query   3’ GAACGCGUAUGGGGUCUCC 5’

query=JS16_t3201285, target=FGSG_13474, score=3.5, range=276-294, strand=-1

target  5’ UUUGCCCAUACCCCCGAGG 3’
           .:::: :::::::: ::::
query   3’ GAACGCGUAUGGGGUCUCC 5’

query=JS16_t3201285, target=FGSG_00070, score=4, range=538-556, strand=1

target  5’ CCUGCGUAUGCCUCAGAGG 3’
           : ::::.::.::.::::::
query   3’ GAACGCGUAUGGGGUCUCC 5’

query=JS16_t3201285, target=FGSG_01376, score=4, range=697-715, strand=1

target  5’ CUUGCUCAUACUCGAGAGG 3’
           ::::: :::::.: :::::
query   3’ GAACGCGUAUGGGGUCUCC 5’

query=JS16_t3201285, target=FGSG_01366, score=4, range=352-370, strand=1

target  5’ CUUCCGUAUACCCCUGAGG 3’
           ::: ::.::::::: ::::
query   3’ GAACGCGUAUGGGGUCUCC 5’

query=JS16_t3201285, target=FGSG_02165, score=4, range=18-37, strand=1

target  5’ CUUCCGCGGUACCCCAGAGG 3’
           ::: ::: .:::::::::::
query   3’ GAACGCG-UAUGGGGUCUCC 5’

query=JS16_t3201285, target=FGSG_10565, score=4, range=368-385, strand=1

target  5’ CUGGAG-AUACCCCAGAGG 3’
           :: : : :::::::::::
query   3’ GAACGCGUAUGGGGUCUCC 5’
```

```
query=JS16_t0306004, target=FGSG_03152, score=4, range=1512-1528, strand=-1

target  5’ AGGG-AGCUUGACCAACA 3’
           :::: :::.:.::::::
query   3’ UCCCAUCGGAUUGGUUGG 5’

No results for JS16_t0578081
query=JS16_t0578084, target=FGSG_10030, score=3, range=1731-1749, strand=-1

target  5’ GCUU-CGCAGCGUUCCCUCG 3’
           . :. :::::::::::::::
query   3’ UAAGUGCGUCGCAAGGGAGC 5’

query=JS16_t0578084, target=FGSG_10046, score=3.5, range=3572-3590, strand=1

target  5’ AUCUA-GCAGCGUUCCUUCG 3’
           :: .: ::::::::::.:::
query   3’ UAAGUGCGUCGCAAGGGAGC 5’

query=JS16_t0578084, target=FGSG_13084, score=4, range=1552-1570, strand=1

target  5’ GUUCGAGCAGCGUU-CCUCG 3’
           .:::. :::::::: :::::
query   3’ UAAGUGCGUCGCAAGGGAGC 5’

query=JS16_t0578084, target=FGSG_10787, score=4, range=679-699, strand=-1

target  5’ AUUCACAGCAGCGUUUCGUCG 3’
           :::::: ::::::::.: :::
query   3’ UAAGUG-CGUCGCAAGGGAGC 5’

query=JS16_t0578084, target=FGSG_00695, score=4, range=318-338, strand=-1

target  5’ GGUCGCGCAGCGUUGCCCUCG 3’
           . ::.::::::::: ::::::
query   3’ UAAGUGCGUCGCAA-GGGAGC 5’

No results for JS16_t0469102
No results for JS16_t4034790
query=JS16_t0268895, target=FGSG_00156, score=2, range=194-211, strand=-1

target  5’ CGUCUGGAUUCUUCUCGA 3’
           ::::::::::::::: ::
query   3’ GCAGACCUAAGAAGAACU 5’

query=JS16_t0268895, target=FGSG_04426, score=2.5, range=532-550, strand=1

target  5’ CCUCUCGGAUUCUUCUUGG 3’
           : ::: ::::::::::::.
query   3’ GCAGA-CCUAAGAAGAACU 5’

query=JS16_t0268895, target=FGSG_04425, score=2.5, range=2330-2347, strand=-1

target  5’ CAUCAGGAUUCUUCUUGG 3’
           : :: ::::::::::::.
query   3’ GCAGACCUAAGAAGAACU 5’

query=JS16_t0268895, target=FGSG_10196, score=2.5, range=605-622, strand=-1

target  5’ UGUGAGGAUUCUUCUUGA 3’
           .::  :::::::::::::
query   3’ GCAGACCUAAGAAGAACU 5’

query=JS16_t0268895, target=FGSG_11946, score=2.5, range=233-250, strand=-1

target  5’ CGUAGGGAUUCUUCUUGG 3’
           :::  ::::::::::::.
query   3’ GCAGACCUAAGAAGAACU 5’

query=JS16_t0268895, target=FGSG_13687, score=3, range=153-170, strand=1

target  5’ AGUCUGGAUCCUUCUUGA 3’
            :::::::: ::::::::
query   3’ GCAGACCUAAGAAGAACU 5’

query=JS16_t0268895, target=FGSG_09337, score=3, range=1326-1343, strand=1

target  5’ AGUCUGGAAUCUUCUUGA 3’
            ::::::: :::::::::
query   3’ GCAGACCUAAGAAGAACU 5’

query=JS16_t0268895, target=FGSG_07962, score=3, range=1957-1973, strand=1

target  5’ CGUCUGG-UUCUUUUUGA 3’
           ::::::: :::::.::::
query   3’ GCAGACCUAAGAAGAACU 5’

query=JS16_t0268895, target=FGSG_02094, score=3, range=810-827, strand=1

target  5’ CGCCUGGAUUCUACUUGA 3’
           :: ::::::::: :::::
```

```
query=JS16_t3201285, target=FGSG_05184, score=4, range=774-793, strand=-1

target  5’ CUUUCGACAUGCCCCAGAGA 3’
           ::: :: :::.::::::::
query   3’ GAACGC-GUAUGGGGUCUCC 5’

query=JS16_t3201285, target=FGSG_07767, score=4, range=77-95, strand=-1

target  5’ CUGGGACAUACCCCAGGGG 3’
           :: :  ::::::::::.::
query   3’ GAACGCGUAUGGGGUCUCC 5’

No results for JS16_t1409067
No results for JS16_t0124027
No results for JS16_t4098185
query=JS16_t0337903, target=FGSG_08265, score=2, range=282-299, strand=-1

target  5’ CCUG-UUUCGCGAUUGCUA 3’
           :::: ::::::::.:::::
query   3’ GGACGAAAGCGCUGACGAU 5’

query=JS16_t0337903, target=FGSG_03413, score=2.5, range=370-388, strand=1

target  5’ CUUGCUUUCGCAACUGCUA 3’
           :.::::::::: :::::::
query   3’ GGACGAAAGCGCUGACGAU 5’

query=JS16_t0337903, target=FGSG_06999, score=3, range=493-511, strand=-1

target  5’ CUCGCUUUUGCGACUGCUG 3’
           :.  :::::.::::::::.
query   3’ GGACGAAAGCGCUGACGAU 5’

query=JS16_t0337903, target=FGSG_04465, score=3.5, range=337-355, strand=1

target  5’ UUUGCUUUCGCGAAUGCUG 3’
           ..::::::::::: ::::.
query   3’ GGACGAAAGCGCUGACGAU 5’

query=JS16_t0337903, target=FGSG_00436, score=3.5, range=655-673, strand=-1

target  5’ CCUGCUUUCGUGGUUGCUG 3’
           ::::::::::.:..::::.
query   3’ GGACGAAAGCGCUGACGAU 5’

query=JS16_t0337903, target=FGSG_00885, score=4, range=727-745, strand=1

target  5’ CCUGCUCUCCCGACUGCUA 3’
           :::::: :: :::::::::
query   3’ GGACGAAAGCGCUGACGAU 5’

query=JS16_t0337903, target=FGSG_06849, score=4, range=98-116, strand=-1

target  5’ UUUGCUUUCUCGGCUGCUA 3’
           ..::::::: ::.::::::
query   3’ GGACGAAAGCGCUGACGAU 5’

query=JS16_t0337903, target=FGSG_00741, score=4, range=83-101, strand=-1

target  5’ CUAGCUGUCGCGACUGCUG 3’
           :.  ::: ::::::::::.
query   3’ GGACGAAAGCGCUGACGAU 5’

query=JS16_t0337903, target=FGSG_06992, score=4, range=2014-2031, strand=-1

target  5’ CCUAUUUC-CGACUGCUG 3’
           ::: .:::: ::::::::.
query   3’ GGACGAAAGCGCUGACGAU 5’

query=JS16_t4098252, target=FGSG_04958, score=2, range=584-600, strand=1

target  5’ AUA-CCAUAUUCAGGAUG 3’
           ::: :::::::::::::
query   3’ UAUCGGUAUAAGUCCUAG 5’

query=JS16_t4098252, target=FGSG_09371, score=3, range=1724-1741, strand=1

target  5’ GAAGGCAUAUUCAGGAUU 3’
           .  :: :::::::::::.
query   3’ UAUCGGUAUAAGUCCUAG 5’

query=JS16_t4098252, target=FGSG_09226, score=3.5, range=2211-2227, strand=1

target  5’ GAAG-CGUAUUCAGGAUC 3’
           .  :: :.:::::::::
query   3’ UAUCGGUAUAAGUCCUAG 5’

query=JS16_t4098252, target=FGSG_07346, score=3.5, range=775-792, strand=-1

target  5’ GUAGCCACGUUCAGGAUC 3’
           .:::::: .:::::::::
query   3’ UAUCGGUAUAAGUCCUAG 5’
```

```
query   3' GCAGACCUAAGAAGAACU 5'

query=JS16_t0268895, target=FGSG_00002, score=3, range=1254-1271, strand=-1

target  5' AGUCUGGAUUCUUCUGGA 3'
            ::::::::::::: ::
query   3' GCAGACCUAAGAAGAACU 5'

query=JS16_t0268895, target=FGSG_01065, score=3, range=1424-1441, strand=-1

target  5' CAUCUGGAAUCUUCUUGA 3'
           : :::::: :::::::::
query   3' GCAGACCUAAGAAGAACU 5'

query=JS16_t0268895, target=FGSG_06577, score=3, range=162-178, strand=-1

target  5' GGUC-GGGUUCUUCUUGA 3'
            ::: ::.::::::::::
query   3' GCAGACCUAAGAAGAACU 5'

query=JS16_t0268895, target=FGSG_13743, score=3, range=336-352, strand=-1

target  5' CGU-UGGAUCCUUCUUGA 3'
           ::: ::::: ::::::::
query   3' GCAGACCUAAGAAGAACU 5'

query=JS16_t0268895, target=FGSG_13065, score=3, range=458-474, strand=-1

target  5' CG-CUGCAUUCUUCUUGA 3'
           :: ::: ::::::::::
query   3' GCAGACCUAAGAAGAACU 5'

query=JS16_t0268895, target=FGSG_04105, score=3, range=15-31, strand=-1

target  5' CGU-UGAAUUCUUCUUGA 3'
           ::: :: ::::::::::
query   3' GCAGACCUAAGAAGAACU 5'

query=JS16_t0268895, target=FGSG_07777, score=3.5, range=815-832, strand=1

target  5' CAAUUGGAUUCUUUUUGA 3'
           :  .:::::::::.::::
query   3' GCAGACCUAAGAAGAACU 5'

query=JS16_t0268895, target=FGSG_03677, score=3.5, range=3627-3644, strand=1

target  5' UGUCAGGAUGCUUCUUGA 3'
           .::: :::: ::::::::
query   3' GCAGACCUAAGAAGAACU 5'

query=JS16_t0268895, target=FGSG_02945, score=3.5, range=515-532, strand=1

target  5' CGUGUCGAUUCUUCUUGG 3'
           ::: : :::::::::::.
query   3' GCAGACCUAAGAAGAACU 5'

query=JS16_t0268895, target=FGSG_05751, score=3.5, range=457-475, strand=-1

target  5' CGUUUGGCAUUUUUCUUGA 3'
           :::.::: :::.:::::::
query   3' GCAGACC-UAAGAAGAACU 5'

query=JS16_t0268895, target=FGSG_13497, score=3.5, range=1634-1651, strand=-1

target  5' CCUUUGGCUUCUUCUUGA 3'
           : :.::: ::::::::::
query   3' GCAGACCUAAGAAGAACU 5'

query=JS16_t0268895, target=FGSG_01846, score=3.5, range=1184-1201, strand=-1

target  5' CGUUGUGAUUCUUCUUGA 3'
           :::.  ::::::::::::
query   3' GCAGACCUAAGAAGAACU 5'

query=JS16_t0268895, target=FGSG_11988, score=3.5, range=3671-3687, strand=-1

target  5' UGUCCGG-UUCUUCUUGA 3'
           .::: :: ::::::::::
query   3' GCAGACCUAAGAAGAACU 5'

query=JS16_t0268895, target=FGSG_11302, score=3.5, range=231-247, strand=-1

target  5' UGUCAGGAUUCUU-UUGA 3'
           .::: :::::::: ::::
query   3' GCAGACCUAAGAAGAACU 5'

query=JS16_t0268895, target=FGSG_09654, score=3.5, range=357-373, strand=-1

target  5' CGU-UGGAUUCUUCUUCG 3'
           ::: :::::::::::: .
query   3' GCAGACCUAAGAAGAACU 5'

query=JS16_t4098252, target=FGSG_08830, score=3.5, range=1127-1143, strand=-1

target  5' AUAGUCGUAUUCA-GAUC 3'
           ::::.:.:::::: ::::
query   3' UAUCGGUAUAAGUCCUAG 5'

query=JS16_t4098252, target=FGSG_02888, score=3.5, range=2236-2253, strand=-1

target  5' AAAGUCACAUUCAGGAUC 3'
           : ::.:: ::::::::::
query   3' UAUCGGUAUAAGUCCUAG 5'

query=JS16_t4098252, target=FGSG_09573, score=3.5, range=361-378, strand=-1

target  5' GUACCCAUAUUGAGGAUC 3'
           .:: ::::::: ::::::
query   3' UAUCGGUAUAAGUCCUAG 5'

query=JS16_t4098252, target=FGSG_07100, score=3.5, range=448-464, strand=-1

target  5' GUCG-CGUAUUCAGGAUC 3'
           .: : :.:::::::::::
query   3' UAUCGGUAUAAGUCCUAG 5'

query=JS16_t4098252, target=FGSG_08097, score=4, range=55-72, strand=1

target  5' AUGGCCAUAUUUAGGAGU 3'
           ::.::::::::.:::: .
query   3' UAUCGGUAUAAGUCCUAG 5'

query=JS16_t4098252, target=FGSG_08056, score=4, range=1889-1906, strand=1

target  5' GUAGUCAUAGUCAGGAUA 3'
           .:::.:::: :::::::
query   3' UAUCGGUAUAAGUCCUAG 5'

query=JS16_t4098252, target=FGSG_09758, score=4, range=800-818, strand=1

target  5' AUAGUCCAUUUUCGGGAUC 3'
           :::: :::: :::.:::::
query   3' UAUC-GGUAUAAGUCCUAG 5'

query=JS16_t4098252, target=FGSG_08295, score=4, range=820-837, strand=1

target  5' GAAGUCAUUUUCAGGAUC 3'
           . ::.::: :::::::::
query   3' UAUCGGUAUAAGUCCUAG 5'

query=JS16_t4098252, target=FGSG_00443, score=4, range=602-619, strand=1

target  5' AUGUUCAUAUUCAUGAUC 3'
           ::. .:::::::: ::::
query   3' UAUCGGUAUAAGUCCUAG 5'

query=JS16_t4098252, target=FGSG_11284, score=4, range=110-126, strand=1

target  5' AU-GCCGUAUUCCGGAUC 3'
           :: :::.::::: :::::
query   3' UAUCGGUAUAAGUCCUAG 5'

query=JS16_t4098252, target=FGSG_05068, score=4, range=797-813, strand=1

target  5' GUAG-CAUAUCCAGGAUU 3'
           .::: ::::: ::::::.
query   3' UAUCGGUAUAAGUCCUAG 5'

query=JS16_t4098252, target=FGSG_12344, score=4, range=610-627, strand=1

target  5' ACAGACAUAUUCUGGAUC 3'
           : :: ::::::: :::::
query   3' UAUCGGUAUAAGUCCUAG 5'

query=JS16_t4098252, target=FGSG_09766, score=4, range=43-59, strand=1

target  5' AUUGACAUAUUC-GGAUC 3'
           :: : ::::::: :::::
query   3' UAUCGGUAUAAGUCCUAG 5'

query=JS16_t4098252, target=FGSG_03832, score=4, range=660-677, strand=-1

target  5' AUAGCUAUGUUCAGGACC 3'
           :::::.::.::::::: :
query   3' UAUCGGUAUAAGUCCUAG 5'

query=JS16_t4098252, target=FGSG_07119, score=4, range=1258-1275, strand=-1

target  5' GUAGUCAUGAUCAGGAUC 3'
           .:::.:::. ::::::::
query   3' UAUCGGUAUAAGUCCUAG 5'

query=JS16_t4098252, target=FGSG_03323, score=4, range=624-641, strand=-1
```

```
query=JS16_t0268895, target=FGSG_00618, score=3.5, range=201-217, strand=-1

target  5' CGU-GGGAUUCUUUUUGG 3'
           :::  :::::::.:::.
query   3' GCAGACCUAAGAAGAACU 5'

query=JS16_t0268895, target=FGSG_04413, score=4, range=973-991, strand=1

target  5' UGUCUGGGUUCUUUCUUGG 3'
           .::::::.::: ::::::.
query   3' GCAGACCUAAG-AAGAACU 5'

query=JS16_t0268895, target=FGSG_08019, score=4, range=572-589, strand=1

target  5' UCUUUGGAUUCUUCUUCA 3'
           . :.:::::::::::: :
query   3' GCAGACCUAAGAAGAACU 5'

query=JS16_t0268895, target=FGSG_07153, score=4, range=833-851, strand=1

target  5' UGUCGGGAUUCUUGCUUGG 3'
           .::: :::::::: ::::.
query   3' GCAGACCUAAGAA-GAACU 5'

query=JS16_t0268895, target=FGSG_00714, score=4, range=921-938, strand=1

target  5' CAUCUUGAUUCUUCUUGC 3'
           : ::: :::::::::::
query   3' GCAGACCUAAGAAGAACU 5'

query=JS16_t0268895, target=FGSG_00504, score=4, range=511-527, strand=1

target  5' CGUC-GGGUUAUUCUUGA 3'
           :::: ::.:: :::::::
query   3' GCAGACCUAAGAAGAACU 5'

query=JS16_t0268895, target=FGSG_12905, score=4, range=170-188, strand=1

target  5' CGGCAUGUAUUCUUCUUGA 3'
           :: : :: :::::::::::
query   3' GCAG-ACCUAAGAAGAACU 5'

query=JS16_t0268895, target=FGSG_05220, score=4, range=1094-1111, strand=1

target  5' CGAGAGGAUUCUUCUUGC 3'
           ::   ::::::::::::
query   3' GCAGACCUAAGAAGAACU 5'

query=JS16_t0268895, target=FGSG_10300, score=4, range=1103-1119, strand=1

target  5' CGUCU-GAUUCUUCUUCA 3'
           ::::: :::::::::: :
query   3' GCAGACCUAAGAAGAACU 5'

query=JS16_t0268895, target=FGSG_06937, score=4, range=230-247, strand=-1

target  5' CGUCAGGGUUCUUCAUGA 3'
           :::: ::.:::::: :::
query   3' GCAGACCUAAGAAGAACU 5'

query=JS16_t0268895, target=FGSG_08583, score=4, range=791-808, strand=-1

target  5' CGACUGUGUUCUUCUUGA 3'
           :: ::: .::::::::::
query   3' GCAGACCUAAGAAGAACU 5'

query=JS16_t0268895, target=FGSG_09231, score=4, range=773-790, strand=-1

target  5' CCUCUGGGUUGUUCUUGA 3'
           : :::::.:: :::::::
query   3' GCAGACCUAAGAAGAACU 5'

query=JS16_t0268895, target=FGSG_03694, score=4, range=839-856, strand=-1

target  5' CGUCGGGGUUCUUCUUAA 3'
           :::: ::.::::::::: :
query   3' GCAGACCUAAGAAGAACU 5'

query=JS16_t0268895, target=FGSG_02157, score=4, range=71-88, strand=-1

target  5' ACUCUGGAUUCUCCUUGA 3'
             :::::::::: :::::
query   3' GCAGACCUAAGAAGAACU 5'

query=JS16_t0268895, target=FGSG_02040, score=4, range=38-55, strand=-1

target  5' UGUCAGGCUUCUUCUUGG 3'
           .::: :: :::::::::.
query   3' GCAGACCUAAGAAGAACU 5'

query=JS16_t0268895, target=FGSG_10824, score=4, range=41-58, strand=-1

target  5' GUGGCCAUGUACAGGAUC 3'
           .:.:::::.: :::::::
query   3' UAUCGGUAUAAGUCCUAG 5'

query=JS16_t4098252, target=FGSG_01075, score=4, range=229-245, strand=-1

target  5' AUAGCCAUGUUUAGG-UC 3'
           ::::::::.::.::: ::
query   3' UAUCGGUAUAAGUCCUAG 5'

query=JS16_t4098252, target=FGSG_06013, score=4, range=268-285, strand=-1

target  5' UUGCUCAUGUUCAGGAUC 3'
            :. .:::.:::::::::
query   3' UAUCGGUAUAAGUCCUAG 5'

query=JS16_t4098252, target=FGSG_08141, score=4, range=493-510, strand=-1

target  5' GUAGACAUAUUCAGCAUU 3'
           .::: ::::::::: ::.
query   3' UAUCGGUAUAAGUCCUAG 5'

query=JS16_t4098252, target=FGSG_10716, score=4, range=1360-1378, strand=-1

target  5' GUAGACCAUUUUCAGGAUU 3'
           .::: :::: ::::::::.
query   3' UAUC-GGUAUAAGUCCUAG 5'

query=JS16_t4098252, target=FGSG_03208, score=4, range=205-221, strand=-1

target  5' AUAG-CGUUUUCAGGAUC 3'
           :::: :.: :::::::::
query   3' UAUCGGUAUAAGUCCUAG 5'

query=JS16_t4098252, target=FGSG_00895, score=4, range=1375-1392, strand=-1

target  5' AAAGACAUUUUCAGGAUC 3'
           : :: ::: :::::::::
query   3' UAUCGGUAUAAGUCCUAG 5'

query=JS16_t4098252, target=FGSG_11653, score=4, range=1321-1338, strand=-1

target  5' AUCAACAUAUUCAGGGUC 3'
           ::   ::::::::::.::
query   3' UAUCGGUAUAAGUCCUAG 5'

query=JS16_t4098252, target=FGSG_00930, score=4, range=300-316, strand=-1

target  5' UCAG-CAUAUUCAGGAUA 3'
             :: ::::::::::::
query   3' UAUCGGUAUAAGUCCUAG 5'

query=JS16_t4098252, target=FGSG_02409, score=4, range=1708-1724, strand=-1

target  5' AAAG-CAUAAUCAGGAUC 3'
           : :: :::: ::::::::
query   3' UAUCGGUAUAAGUCCUAG 5'

query=JS16_t4098252, target=FGSG_08405, score=4, range=2620-2636, strand=-1

target  5' UUCGCCA-AUUCAGGAUC 3'
            : :::: ::::::::::
query   3' UAUCGGUAUAAGUCCUAG 5'

query=JS16_t4098252, target=FGSG_11993, score=4, range=511-527, strand=-1

target  5' AUAGCCAUA-UCAUGAUC 3'
           ::::::::: ::: ::::
query   3' UAUCGGUAUAAGUCCUAG 5'

No results for JS16_t2305404
query=JS16_t4098264, target=FGSG_01045, score=4, range=462-483, strand=1

target  5' CAGUAUGAGUACUGUUGCUAUU 3'
           : : : : ::::::::::::::
query   3' GCCCUUCGCAUGACAACGAUAA 5'

No results for JS16_t2305419
No results for JS16_t4098320
No results for JS16_t3201479
query=JS16_t3201510, target=FGSG_07413, score=3, range=3260-3279, strand=-1

target  5' GU-UGGAAUUGUCUCCUGCUU 3'
           :: ::::::::::::: :::::
query   3' CAUACCUUAACAGAGCACGAA 5'

query=JS16_t3201510, target=FGSG_07014, score=4, range=506-526, strand=1

target  5' GGGAGGAGUUGUCUCGUGCUG 3'
           : . :::.::::::::::::
query   3' CAUACCUUAACAGAGCACGAA 5'
```

```
target  5’ GGUCCGGGUUCUUCUUGU 3’
            ::: ::.:::::::::
query   3’ GCAGACCUAAGAAGAACU 5’

query=JS16_t0268895, target=FGSG_03858, score=4, range=1142-1159, strand=-1

target  5’ UCUUGGGGUUCUUCUUGA 3’
           . :. ::.::::::::::
query   3’ GCAGACCUAAGAAGAACU 5’

query=JS16_t0268895, target=FGSG_02561, score=4, range=1301-1319, strand=-1

target  5’ UGUGUGGGGUUCUUCUUGG 3’
           .:: : ::.:::::::::.
query   3’ GCAGA-CCUAAGAAGAACU 5’

query=JS16_t0268895, target=FGSG_04016, score=4, range=167-184, strand=-1

target  5’ AAUUAGGAUUCUUCUUGG 3’
             :. ::::::::::::.
query   3’ GCAGACCUAAGAAGAACU 5’

query=JS16_t0268895, target=FGSG_13700, score=4, range=6819-6836, strand=-1

target  5’ UCCUUGGAUUCUUCUUGU 3’
           .  .:::::::::::::
query   3’ GCAGACCUAAGAAGAACU 5’

query=JS16_t0268895, target=FGSG_11260, score=4, range=634-652, strand=-1

target  5’ CGUCCGGAUUCUUCGUUGU 3’
           :::: ::::::::: :::
query   3’ GCAGACCUAAGAAG-AACU 5’

query=JS16_t0268895, target=FGSG_03207, score=4, range=1460-1476, strand=-1

target  5’ CGUC-GGAAUUUUCUUGA 3’
           :::: ::: :.:::::::
query   3’ GCAGACCUAAGAAGAACU 5’

query=JS16_t0268895, target=FGSG_04394, score=4, range=89-106, strand=-1

target  5’ CGUGGGGAUUAUUCUUGA 3’
           :::  ::::: :::::::
query   3’ GCAGACCUAAGAAGAACU 5’

query=JS16_t0268895, target=FGSG_10821, score=4, range=74-91, strand=-1

target  5’ CAUCAGCAUUCUUCUUGA 3’
           : :: : :::::::::::
query   3’ GCAGACCUAAGAAGAACU 5’

query=JS16_t0268895, target=FGSG_01910, score=4, range=2154-2171, strand=-1

target  5’ UGCAAGGAUUCUUCUUGG 3’
           .:   ::::::::::::.
query   3’ GCAGACCUAAGAAGAACU 5’

query=JS16_t0268895, target=FGSG_00846, score=4, range=1812-1828, strand=-1

target  5’ CG-CUGGAUCCUUCUUGC 3’
           :: :::::: :::::::
query   3’ GCAGACCUAAGAAGAACU 5’

query=JS16_t0268895, target=FGSG_02150, score=4, range=123-139, strand=-1

target  5’ CUUCGGGAUUCUUC-UGA 3’
           : :: ::::::::: :::
query   3’ GCAGACCUAAGAAGAACU 5’

No results for JS16_t3138334
query=JS16_t0306011, target=FGSG_08926, score=3.5, range=818-837, strand=-1

target  5’ GCCC-UGAAUACUACGAGAGG 3’
           :::: .:: ::::::::::::
query   3’ CGGGCGCUCAUGAUGCUCUCC 5’

query=JS16_t0306011, target=FGSG_11716, score=4, range=1822-1841, strand=1

target  5’ GUUCUCGAGUACUACGA-AGG 3’
           :..: :::::::::::: :::
query   3’ CGGGCGCUCAUGAUGCUCUCC 5’

query=JS16_t0254730, target=FGSG_12026, score=4, range=466-484, strand=1

target  5’ GAUUUCGAACCUGGCUCUG 3’
           ::: ::::::::..:.:::
query   3’ CUAUAGCUUGGAUUGGGAC 5’

query=JS16_t0254730, target=FGSG_07495, score=4, range=1617-1635, strand=1

target  5’ GGUAUUGAACCUCACCCUU 3’

No results for JS16_t0694427
No results for JS16_t0241474
query=JS16_t4098553, target=FGSG_11815, score=3.5, range=423-443, strand=1

target  5’ CGCUGC-GGCGGUCAUCUUCUG 3’
           :: ..: :.:::::::::::::
query   3’ GCCGUGUCUGCCAGUAGAAGAC 5’

query=JS16_t4098553, target=FGSG_12034, score=3.5, range=2468-2488, strand=-1

target  5’ UGGCAAAG-UGGUCAUCUUCUG 3’
           .:::: :: .::::::::::::
query   3’ GCCGUGUCUGCCAGUAGAAGAC 5’

query=JS16_t4098553, target=FGSG_09298, score=4, range=1263-1284, strand=1

target  5’ UAGCGGAAACGGUCAUCUUCUG 3’
           . ::. : ::::::::::::::
query   3’ GCCGUGUCUGCCAGUAGAAGAC 5’

No results for JS16_t3201685
query=JS16_t0585780, target=FGSG_10710, score=3.5, range=196-216, strand=1

target  5’ GCGCAUAACCAAGAUACCCAA 3’
           :::::.:::: :::::::::
query   3’ CGCGUGUUGG-UCUAUGGGUG 5’

query=JS16_t0585780, target=FGSG_00142, score=4, range=278-297, strand=-1

target  5’ GCGCUCGACCAGAUAUCCGU 3’
           :::: :.::::::::.::..
query   3’ CGCGUGUUGGUCUAUGGGUG 5’

query=JS16_t0804004, target=FGSG_06423, score=4, range=1376-1396, strand=1

target  5’ ACCAGUGGAAGAACUGGCUCA 3’
           :: :::..:::::: ::::::
query   3’ UGCUCAUUUUCUUGCCCGAGU 5’

query=JS16_t0229187, target=FGSG_13274, score=2.5, range=658-675, strand=-1

target  5’ CUCCUUGUAAGCAAGUUC 3’
           : ::.:::::::::::.:
query   3’ GUGGGACAUUCGUUCAGG 5’

query=JS16_t0229187, target=FGSG_05119, score=3, range=1400-1417, strand=1

target  5’ CACCCUCUAAGCAGGUCC 3’
           :::::: ::::::.::::
query   3’ GUGGGACAUUCGUUCAGG 5’

query=JS16_t0229187, target=FGSG_04243, score=3.5, range=177-193, strand=1

target  5’ UAUUCUG-AAGCAAGUCC 3’
           .:..::: ::::::::::
query   3’ GUGGGACAUUCGUUCAGG 5’

query=JS16_t0229187, target=FGSG_10530, score=3.5, range=5979-5996, strand=-1

target  5’ GACUCUGUGAGCAAGUUC 3’
            ::.::::.:::::::.:
query   3’ GUGGGACAUUCGUUCAGG 5’

query=JS16_t0229187, target=FGSG_03656, score=3.5, range=349-366, strand=-1

target  5’ CACCUUGUCAGCAAGUUC 3’
           ::::.::: :::::::.:
query   3’ GUGGGACAUUCGUUCAGG 5’

query=JS16_t0229187, target=FGSG_00831, score=3.5, range=1282-1300, strand=-1

target  5’ CAUCCUUGUAAGCGGGUCC 3’
           :: ::.:::::::..::::
query   3’ GU-GGGACAUUCGUUCAGG 5’

query=JS16_t0229187, target=FGSG_09747, score=4, range=307-325, strand=1

target  5’ CGCCUUGAUGAGCAAGUCC 3’
           :.::.:: :.:::::::::
query   3’ GUGGGAC-AUUCGUUCAGG 5’

query=JS16_t0229187, target=FGSG_02113, score=4, range=1758-1774, strand=1

target  5’ CACCCU-UAAGGAAGUCC 3’
           :::::: :::: ::::::
query   3’ GUGGGACAUUCGUUCAGG 5’

query=JS16_t0229187, target=FGSG_00441, score=4, range=73-90, strand=-1

target  5’ CACUUUGUAAGCACGUUC 3’
           :::..:::::::: ::.:
```

```
           :.:::.:::::: :::::
query   3’ CUAUAGCUUGGAUUGGGAC 5’

query=JS16_t0254730, target=FGSG_03568, score=4, range=565-583, strand=1

target  5’ GAUAUAGACCCUGACCCUG 3’
           ::::: :: :::.::::::
query   3’ CUAUAGCUUGGAUUGGGAC 5’

query=JS16_t0254730, target=FGSG_11599, score=4, range=643-661, strand=1

target  5’ GAUCUGGAACUUGACCCUG 3’
           ::: : ::::.:.::::::
query   3’ CUAUAGCUUGGAUUGGGAC 5’

query=JS16_t0254730, target=FGSG_10502, score=4, range=2623-2641, strand=1

target  5’ CCUACCGAACCUAGCCCUG 3’
             :: ::::::::.:::::
query   3’ CUAUAGCUUGGAUUGGGAC 5’

query=JS16_t0254730, target=FGSG_02274, score=4, range=422-439, strand=1

target  5’ GGUGU-GGACCUAACCCUU 3’
           :.:.: :.::::::::::
query   3’ CUAUAGCUUGGAUUGGGAC 5’

query=JS16_t0254730, target=FGSG_09949, score=4, range=430-447, strand=1

target  5’ GAGAUCGAACCU-ACCCUC 3’
           :: ::::::::: :::::
query   3’ CUAUAGCUUGGAUUGGGAC 5’

query=JS16_t0254730, target=FGSG_10502, score=4, range=2749-2767, strand=1

target  5’ CACACCGAACCUAGCCCUG 3’
            : : ::::::::.:::::
query   3’ CUAUAGCUUGGAUUGGGAC 5’

query=JS16_t0254730, target=FGSG_03380, score=4, range=666-684, strand=-1

target  5’ CAUAUCGAACUUGACUCUG 3’
            :::::::::.:.::.:::
query   3’ CUAUAGCUUGGAUUGGGAC 5’

query=JS16_t3138439, target=FGSG_07857, score=4, range=1236-1255, strand=1

target  5’ GUUGAAAGGAGUCAAUCGAG 3’
            ::: ::::::::::.:::
query   3’ AAACCUUCCUCAGUUGGCUA 5’

query=JS16_t3138439, target=FGSG_07104, score=4, range=900-918, strand=1

target  5’ UUUGAAAGGAGUCAACU-AU 3’
           :::: :::::::::::. ::
query   3’ AAACCUUCCUCAGUUGGCUA 5’

query=JS16_t3138439, target=FGSG_09560, score=4, range=267-287, strand=1

target  5’ GUUGACAAGGAGUCAACUGAU 3’
            :::  :::::::::::.:::
query   3’ AAAC-CUUCCUCAGUUGGCUA 5’

query=JS16_t0100726, target=FGSG_00048, score=4, range=284-303, strand=-1

target  5’ AAUGG-ACUCCAGGUAAUCCG 3’
             :::  ::::::::::::::
query   3’ ACACCAAGAGGUCCAUUAGGC 5’

No results for JS16_t1345604
No results for JS16_t2242420
No results for JS16_t3138626
query=JS16_t3138631, target=FGSG_04154, score=3.5, range=1814-1834, strand=-1

target  5’ UG-AACCGUGGAAAUCGCAAAG 3’
           .: :::: ::::::::.:::::
query   3’ GCAUUGGAACCUUUAGUGUUUC 5’

query=JS16_t0796171, target=FGSG_12381, score=3, range=549-569, strand=1

target  5’ AAAGUGUGAAGAGACAUUGGA 3’
           ::::: ::::::: :::::::
query   3’ UUUCAAACUUCUCGGUAACCU 5’

query=JS16_t0796171, target=FGSG_09606, score=3.5, range=229-249, strand=-1

target  5’ AGAGCGUGAGGAGCCAUUGGA 3’
           :.::  :::.::::::::::
query   3’ UUUCAAACUUCUCGGUAACCU 5’

query=JS16_t0796171, target=FGSG_06301, score=3.5, range=2128-2148, strand=-1
```

```
query   3’ GUGGGACAUUCGUUCAGG 5’

query=JS16_t0229187, target=FGSG_05542, score=4, range=5001-5018, strand=-1

target  5’ AAAGCUGUAAGCAAGUUC 3’
            :  ::::::::::::.:
query   3’ GUGGGACAUUCGUUCAGG 5’

query=JS16_t0229187, target=FGSG_02575, score=4, range=297-314, strand=-1

target  5’ CAGCCUGUAAGGAAGUCG 3’
           :: :::::::: :::::
query   3’ GUGGGACAUUCGUUCAGG 5’

query=JS16_t0229187, target=FGSG_08184, score=4, range=117-133, strand=-1

target  5’ AACCCUGUGA-CAAGUCC 3’
            :::::::.: :::::::
query   3’ GUGGGACAUUCGUUCAGG 5’

No results for JS16_t0396757
No results for JS16_t1409588
No results for JS16_t3201759
No results for JS16_t3201782
No results for JS16_t2305882
query=JS16_t2305886, target=FGSG_09263, score=4, range=107-128, strand=-1

target  5’ UCGAUUU-CUUCUUGAGUCAAGU 3’
           ::. ::: :::::::::.::::.
query   3’ AGUAAAAGGAAGAACUCGGUUCG 5’

No results for JS16_t3201894
No results for JS16_t2305925
query=JS16_t0269936, target=FGSG_09900, score=4, range=472-490, strand=1

target  5’ GU-UGCUGUGUUCUAUGCUG 3’
           :: ::::: ::::::.::::
query   3’ CAUACGACCCAAGAUGCGAC 5’

query=JS16_t0269936, target=FGSG_01465, score=4, range=638-656, strand=1

target  5’ GGAUG-AGGGUUCUAUGCUG 3’
           : :::  ::::::::.::::
query   3’ CAUACGACCCAAGAUGCGAC 5’

query=JS16_t0269936, target=FGSG_01054, score=4, range=226-245, strand=-1

target  5’ GUUUGGUGGGUUCUACGCAG 3’
           :: :: ::::::::::: :
query   3’ CAUACGACCCAAGAUGCGAC 5’

query=JS16_t0284306, target=FGSG_02761, score=2.5, range=1103-1121, strand=1

target  5’ ACAUUCUCCCUGAAGUGCU 3’
            :::.:::::::::::.::
query   3’ AGUAGGAGGGACUUCAUGA 5’

query=JS16_t0284306, target=FGSG_01254, score=3, range=397-416, strand=1

target  5’ UCGAUCCUCCCUGAAGAACU 3’
           :: ::::::::::::: :::
query   3’ AG-UAGGAGGGACUUCAUGA 5’

query=JS16_t0284306, target=FGSG_02176, score=3, range=23-41, strand=-1

target  5’ UCGUUCUCCCUGAAGAACU 3’
           ::.:.:::::::::: :::
query   3’ AGUAGGAGGGACUUCAUGA 5’

query=JS16_t0284306, target=FGSG_00382, score=3, range=3245-3263, strand=-1

target  5’ UCUCCCUCCCUGAAGUGCU 3’
           ::  :::::::::::.::
query   3’ AGUAGGAGGGACUUCAUGA 5’

query=JS16_t0284306, target=FGSG_04312, score=3, range=821-839, strand=-1

target  5’ UCAGCAUCUCUGAAGUACU 3’
           ::: : ::.::::::::::
query   3’ AGUAGGAGGGACUUCAUGA 5’

query=JS16_t0284306, target=FGSG_05350, score=3.5, range=1459-1478, strand=-1

target  5’ UCAUCUUCCCUGAUAGUAUU 3’
           :::::.::::::: ::::.:
query   3’ AGUAGGAGGGACU-UCAUGA 5’

query=JS16_t0284306, target=FGSG_05020, score=3.5, range=770-788, strand=-1

target  5’ UCGCCCUCCUUGAGGUACU 3’
           ::. :::::.:::.:::::
query   3’ AGUAGGAGGGACUUCAUGA 5’
```

```
target  5' GAGGAUAGAAGAGCCAUUGGG 3'
           .:.: : :::::::::::::.
query   3' UUUCAAACUUCUCGGUAACCU 5'

query=JS16_t0796171, target=FGSG_12568, score=4, range=966-985, strand=-1

target  5' CAGGUUUGAA-AGCCAUUGGG 3'
            :.::::::: :::::::::.
query   3' UUUCAAACUUCUCGGUAACCU 5'

query=JS16_t0796171, target=FGSG_08325, score=4, range=810-830, strand=-1

target  5' ACCGUUGGAGGAGCCAUUGGA 3'
           :  ::: ::.:::::::::::
query   3' UUUCAAACUUCUCGGUAACCU 5'

No results for JS16_t2242532
No results for JS16_t3138741
query=JS16_t1345827, target=FGSG_02442, score=3, range=144-163, strand=-1

target  5' GUGAUAA-UGACCCGUCGACG 3'
            ::: :: :::::::::::::
query   3' AACUCUUGACUGGGCAGCUGC 5'

No results for JS16_t2242645
No results for JS16_t4035266
No results for JS16_t3138860
No results for JS16_t2242729
query=JS16_t0686895, target=FGSG_04830, score=3.5, range=963-984, strand=-1

target  5' UA-GACGAUCUCUUUGUCUGUGA 3'
           .: :: ::: ::::::::::::
query   3' GUCCUUCUAUAGAAACAGACACU 5'

query=JS16_t4035445, target=FGSG_03323, score=4, range=619-638, strand=1

target  5' UCCUUGAUCCUGUACAUGGC 3'
           :::: ::::: ::::::::
query   3' AGGACCUAGGCCAUGUACCU 5'

No results for JS16_t3138984
No results for JS16_t3139023
query=JS16_t0240489, target=FGSG_00615, score=3, range=3413-3432, strand=1

target  5' CGGCUGACGGUGGCAUCACC 3'
            ::::::::::.:::::::
query   3' ACCGACUGCCAUCGUAGUGC 5'

query=JS16_t0240489, target=FGSG_09729, score=3, range=541-560, strand=-1

target  5' CGGGUGACGGUAGCAUUACG 3'
            :: ::::::::::::.:::
query   3' ACCGACUGCCAUCGUAGUGC 5'

query=JS16_t0240489, target=FGSG_04872, score=4, range=1817-1836, strand=1

target  5' UGGCUGACGGUAUUAUCACU 3'
           :::::::::::: .:::::
query   3' ACCGACUGCCAUCGUAGUGC 5'

query=JS16_t0240489, target=FGSG_00470, score=4, range=641-660, strand=1

target  5' UGGCUCACGGUAUCAUCAUG 3'
           ::::: :::::: :::::.:
query   3' ACCGACUGCCAUCGUAGUGC 5'

query=JS16_t0240489, target=FGSG_04886, score=4, range=633-651, strand=1

target  5' UGG-UGCCGGUAGCAUCGCC 3'
           ::: :: ::::::::::.:
query   3' ACCGACUGCCAUCGUAGUGC 5'

query=JS16_t0240489, target=FGSG_09366, score=4, range=679-697, strand=-1

target  5' UGGC-GACGGUGGCAUCAUA 3'
           :::: ::::::.::::::.
query   3' ACCGACUGCCAUCGUAGUGC 5'

query=JS16_t0240489, target=FGSG_09556, score=4, range=994-1013, strand=-1

target  5' UGGCCGUUGGUAGCAUCACC 3'
           :::: : .:::::::::::
query   3' ACCGACUGCCAUCGUAGUGC 5'

No results for JS16_t1346166
No results for JS16_t1346189
No results for JS16_t1346190
No results for JS16_t2242971
query=JS16_t1346231, target=FGSG_10626, score=2, range=81-99, strand=-1

target  5' AGGACACUGGCUUGGUGGU 3'
           ::::::::::::: :::::

query=JS16_t0284306, target=FGSG_08748, score=3.5, range=173-191, strand=-1

target  5' UCGACAUCCUUGAAGUACU 3'
           ::. : :::.:::::::::
query   3' AGUAGGAGGGACUUCAUGA 5'

query=JS16_t0284306, target=FGSG_00281, score=4, range=565-584, strand=1

target  5' UCAUCAGUCCUUGAAGUGCU 3'
           :::::  :::.::::::.::
query   3' AGUAG-GAGGGACUUCAUGA 5'

query=JS16_t0284306, target=FGSG_04296, score=4, range=36-54, strand=1

target  5' UCAUCGUCCCUCAAGUACC 3'
           ::::: ::::: ::::::
query   3' AGUAGGAGGGACUUCAUGA 5'

query=JS16_t0284306, target=FGSG_08187, score=4, range=275-292, strand=1

target  5' UGAUCCUCCCUGAAG-ACC 3'
           : ::::::::::::: ::
query   3' AGUAGGAGGGACUUCAUGA 5'

query=JS16_t0284306, target=FGSG_10222, score=4, range=155-172, strand=1

target  5' ACAUCAU-CCUGAAGUACU 3'
            :::: : ::::::::::
query   3' AGUAGGAGGGACUUCAUGA 5'

query=JS16_t0284306, target=FGSG_12021, score=4, range=686-704, strand=-1

target  5' GUAUCUUCCCGGAAGUACU 3'
            .:::.:::: ::::::::
query   3' AGUAGGAGGGACUUCAUGA 5'

query=JS16_t0284306, target=FGSG_10156, score=4, range=782-800, strand=-1

target  5' UCAUCGUCCUUGAAGUCCU 3'
           ::::: :::.:::::: ::
query   3' AGUAGGAGGGACUUCAUGA 5'

query=JS16_t0284306, target=FGSG_07192, score=4, range=980-998, strand=-1

target  5' CCACCCUCCCUGAGGUACC 3'
            :: :::::::::.::::
query   3' AGUAGGAGGGACUUCAUGA 5'

query=JS16_t0284306, target=FGSG_01910, score=4, range=1742-1759, strand=-1

target  5' UC-UGCUCCUUGAGGUACU 3'
           :: : ::::.:::.:::::
query   3' AGUAGGAGGGACUUCAUGA 5'

query=JS16_t0284306, target=FGSG_11375, score=4, range=1466-1483, strand=-1

target  5' UC-UUUCCCUGAUGUACU 3'
           :: :..::::::: :::::
query   3' AGUAGGAGGGACUUCAUGA 5'

query=JS16_t0195007, target=FGSG_12361, score=4, range=1753-1772, strand=1

target  5' UGGACUAUGCUGGGUGGUUU 3'
           :..:: :::::.:::::.::
query   3' AUUUGUUACGAUCCACCGAA 5'

No results for JS16_t3201977
No results for JS16_t1409887
No results for JS16_t1409906
query=JS16_t4098959, target=FgHV1gp1, score=3.5, range=347-369, strand=-1

target  5' UUUGUUACCAGUGAAGUCGAAAG 3'
           : :::. ::::::::::::::
query   3' AUACAGAGGUCACUUCAGCUUUA 5'

query=JS16_t4098959, target=FGSG_09765, score=4, range=1918-1940, strand=-1

target  5' UAUGCCCCAAGUGAAGUCGAAGU 3'
           :::: : : ::::::::::::.:
query   3' AUACAGAGGUCACUUCAGCUUUA 5'

No results for JS16_t1409974
No results for JS16_t4099010
No results for JS16_t3202161
No results for JS16_t1410020
No results for JS16_t2306196
No results for JS16_t4099067
query=JS16_t1410038, target=FGSG_07353, score=3.5, range=560-580, strand=-1

target  5' GAAGAGCGGUAGCAUACUGGA 3'
           :::.:::::::.: :::::::
```

```
query   3' UCCUGUGACCGAA-CACCA 5'

query=JS16_t1346231, target=FGSG_00843, score=3, range=2081-2097, strand=-1

target  5' AGGAGACUGG-UUGUGGU 3'
           :::: ::::: :::::::
query   3' UCCUGUGACCGAACACCA 5'

query=JS16_t1346231, target=FGSG_06448, score=3.5, range=1417-1433, strand=1

target  5' AGUGCA-UGGCUUGUGGU 3'
           :: .:: :::::::::::
query   3' UCCUGUGACCGAACACCA 5'

query=JS16_t1346231, target=FGSG_01636, score=3.5, range=458-474, strand=1

target  5' ACGGC-CUGGCUUGUGGU 3'
           : :.: ::::::::::::
query   3' UCCUGUGACCGAACACCA 5'

query=JS16_t1346231, target=FGSG_02667, score=3.5, range=869-885, strand=-1

target  5' AGCG-GCUGGCUUGUGGU 3'
           :: . .::::::::::::
query   3' UCCUGUGACCGAACACCA 5'

query=JS16_t1346231, target=FGSG_02049, score=3.5, range=486-502, strand=-1

target  5' CGGAUAC-GGCUUGUGGU 3'
            :::.:: ::::::::::
query   3' UCCUGUGACCGAACACCA 5'

query=JS16_t1346231, target=FGSG_12516, score=4, range=629-646, strand=1

target  5' AGGAAGCUGGCUCGUGGU 3'
           :::: .:::::: :::::
query   3' UCCUGUGACCGAACACCA 5'

query=JS16_t1346231, target=FGSG_11115, score=4, range=1852-1868, strand=1

target  5' AGGA-ACUGGUCUGUGGU 3'
           :::: :::::. ::::::
query   3' UCCUGUGACCGAACACCA 5'

query=JS16_t1346231, target=FGSG_02144, score=4, range=662-678, strand=1

target  5' AGGA-GCUGGCUAGUGGU 3'
           :::: .:::::: :::::
query   3' UCCUGUGACCGAACACCA 5'

query=JS16_t1346231, target=FGSG_02327, score=4, range=832-849, strand=-1

target  5' AGGACGCUGGUUUGUGAU 3'
           :::::.::::.::::: :
query   3' UCCUGUGACCGAACACCA 5'

query=JS16_t1346231, target=FGSG_02192, score=4, range=303-320, strand=-1

target  5' AGGACACUGGUUUGUCGA 3'
           ::::::::::.:::: :
query   3' UCCUGUGACCGAACACCA 5'

query=JS16_t1346231, target=FGSG_08487, score=4, range=3660-3676, strand=-1

target  5' GGGAUGCUGGCUUG-GGU 3'
           .:::..:::::::: :::
query   3' UCCUGUGACCGAACACCA 5'

query=JS16_t1346231, target=FGSG_03865, score=4, range=78-95, strand=-1

target  5' GCGAUACUGGCUUUUGGU 3'
           . ::.:::::::: ::::
query   3' UCCUGUGACCGAACACCA 5'

query=JS16_t1346231, target=FGSG_05346, score=4, range=766-782, strand=-1

target  5' CAGACACUGGCUUG-GGU 3'
             ::::::::::: :::
query   3' UCCUGUGACCGAACACCA 5'

query=JS16_t1346231, target=FGSG_06780, score=4, range=381-397, strand=-1

target  5' GAGGCACUGGCUUG-GGU 3'
           . :.:::::::::: :::
query   3' UCCUGUGACCGAACACCA 5'

query=JS16_t1346231, target=FGSG_09429, score=4, range=989-1005, strand=-1

target  5' AGGA-ACUGUCUUGUGGA 3'
           :::: :::: :::::::
query   3' UCCUGUGACCGAACACCA 5'

query   3' CUUUUCGCCAUUGGAUGACCU 5'

No results for JS16_t0220484
query=JS16_t0367441, target=FGSG_06754, score=3, range=3020-3038, strand=1

target  5' UCGAGGAACUCGUUGAGCA 3'
           ::: :::::::.:.:::::
query   3' AGCACCUUGAGUAGCUCGU 5'

query=JS16_t0367441, target=FGSG_00846, score=3, range=89-107, strand=-1

target  5' GCGUGCAACUUAUCGAGCA 3'
            :::: ::::.::::::::
query   3' AGCACCUUGAGUAGCUCGU 5'

query=JS16_t0367441, target=FGSG_12002, score=3, range=713-731, strand=-1

target  5' UCGGGGAACUCAUCAAGCA 3'
           ::: :::::::::: ::::
query   3' AGCACCUUGAGUAGCUCGU 5'

query=JS16_t0367441, target=FGSG_04580, score=3.5, range=1313-1331, strand=-1

target  5' GCGUGGAACUCAUCGGGUG 3'
            ::::::::::::::.:..
query   3' AGCACCUUGAGUAGCUCGU 5'

query=JS16_t0367441, target=FGSG_13699, score=3.5, range=64-82, strand=-1

target  5' UUGUGAAACUCAUCGAGUU 3'
           :.::: ::::::::::::.
query   3' AGCACCUUGAGUAGCUCGU 5'

query=JS16_t0367441, target=FGSG_08948, score=3.5, range=26-43, strand=-1

target  5' UUGU-GAACUCGUCGGGCA 3'
           :.:: ::::::.:::.:::
query   3' AGCACCUUGAGUAGCUCGU 5'

query=JS16_t0367441, target=FGSG_13820, score=4, range=98-115, strand=1

target  5' UCGUGGGACUUAUC-AGCA 3'
           ::::::.:::.::: ::::
query   3' AGCACCUUGAGUAGCUCGU 5'

query=JS16_t0367441, target=FGSG_01422, score=4, range=470-488, strand=1

target  5' UCGAGGAGCUCAUCGACCA 3'
           ::: :::.:::::::: ::
query   3' AGCACCUUGAGUAGCUCGU 5'

query=JS16_t0367441, target=FGSG_05081, score=4, range=2783-2801, strand=1

target  5' ACGAUGAAUUCAUCGAGCA 3'
            ::  :::.::::::::::
query   3' AGCACCUUGAGUAGCUCGU 5'

query=JS16_t0367441, target=FGSG_04197, score=4, range=383-400, strand=1

target  5' UC-UGGAACUCAUUCAGCA 3'
           :: ::::::::::. ::::
query   3' AGCACCUUGAGUAGCUCGU 5'

query=JS16_t0367441, target=FGSG_01645, score=4, range=611-629, strand=-1

target  5' UCGUCGAAUUCAUCGAGUU 3'
           :::: :::.::::::::.
query   3' AGCACCUUGAGUAGCUCGU 5'

query=JS16_t0367441, target=FGSG_00633, score=4, range=8756-8774, strand=-1

target  5' UCGCGGAACUCCUCGGGCA 3'
           ::: ::::::: :::.:::
query   3' AGCACCUUGAGUAGCUCGU 5'

query=JS16_t0367441, target=FGSG_07769, score=4, range=395-413, strand=-1

target  5' UCGUAGAACUCGUCCAGCA 3'
           :::: ::::::.:: ::::
query   3' AGCACCUUGAGUAGCUCGU 5'

query=JS16_t0367441, target=FGSG_05750, score=4, range=54-73, strand=-1

target  5' UCGUAGGAAGUCGUCGAGCA 3'
           :::: :::: ::.:::::::
query   3' AGCA-CCUUGAGUAGCUCGU 5'

query=JS16_t0367441, target=FGSG_01119, score=4, range=546-564, strand=-1

target  5' UCGGGCAGCUCGUCGAGCA 3'
           ::: : :.:::.:::::::
query   3' AGCACCUUGAGUAGCUCGU 5'
```

```
query=JS16_t1346231, target=FGSG_13141, score=4, range=451-467, strand=-1

target  5’ AUGCCACUGGCUU-UGGU 3’
           : : ::::::::: ::::
query   3’ UCCUGUGACCGAACACCA 5’

query=JS16_t0469206, target=FGSG_02804, score=3.5, range=437-454, strand=-1

target  5’ UGGU-AAUGCGUAGAAGCA 3’
           :.:: :::::::: :::::
query   3’ AUCAGUUACGCAUGUUCGU 5’

query=JS16_t0469206, target=FGSG_07623, score=4, range=725-744, strand=1

target  5’ UAGUACAAUGCGCACGAGCA 3’
           :::: ::::::: ::.::::
query   3’ AUCA-GUUACGCAUGUUCGU 5’

query=JS16_t0469206, target=FGSG_10897, score=4, range=772-790, strand=1

target  5’ UUGACAAAGCGUACAAGCA 3’
           : : ::: :::::::::::
query   3’ AUCAGUUACGCAUGUUCGU 5’

query=JS16_t0469206, target=FGSG_01862, score=4, range=857-875, strand=-1

target  5’ UCCUUGAUGCGUGCAAGCA 3’
           :  :..::::::.::::::
query   3’ AUCAGUUACGCAUGUUCGU 5’

query=JS16_t0469206, target=FGSG_10993, score=4, range=501-520, strand=-1

target  5’ UACUGCAAUGUGUACAGGCA 3’
           :: : :::::.:::::.:::
query   3’ AUCA-GUUACGCAUGUUCGU 5’

query=JS16_t0469206, target=FGSG_01006, score=4, range=653-670, strand=-1

target  5’ AAGUC-AUGCGUGCGAGCA 3’
            :::: ::::::.:.::::
query   3’ AUCAGUUACGCAUGUUCGU 5’

query=JS16_t0469206, target=FGSG_07265, score=4, range=992-1010, strand=-1

target  5’ UGUUGGAUGCGUACAAGCU 3’
           :. : .::::::::::::
query   3’ AUCAGUUACGCAUGUUCGU 5’

query=JS16_t0469206, target=FGSG_05707, score=4, range=93-110, strand=-1

target  5’ UAACCAAU-CGUACAAGCA 3’
           ::  :::: ::::::::::
query   3’ AUCAGUUACGCAUGUUCGU 5’

No results for JS16_t1346283
query=JS16_t1346289, target=FGSG_07758, score=3, range=917-935, strand=1

target  5’ GCAAGUUUGUUUUUGGCAC 3’
           ::::: :::::.::::::
query   3’ CGUUCUAACAAGAACCGUU 5’

query=JS16_t1346289, target=FGSG_11742, score=3, range=6637-6655, strand=1

target  5’ GAUACAUUGUUCUUGGCAA 3’
           :  : ::::::::::::::
query   3’ CGUUCUAACAAGAACCGUU 5’

query=JS16_t1346289, target=FGSG_05019, score=3, range=90-108, strand=-1

target  5’ GCGAGGUUGUUUUUGGCAU 3’
           ::.::.:::::.::::::
query   3’ CGUUCUAACAAGAACCGUU 5’

query=JS16_t1346289, target=FGSG_00921, score=3, range=170-188, strand=-1

target  5’ GCAGCAUUGUUCUUGGCGG 3’
           :::. ::::::::::::..
query   3’ CGUUCUAACAAGAACCGUU 5’

query=JS16_t1346289, target=FGSG_10009, score=3, range=2528-2546, strand=-1

target  5’ UCAAGAUUGUUCUCGGCAA 3’
            :::::::::::: :::::
query   3’ CGUUCUAACAAGAACCGUU 5’

query=JS16_t1346289, target=FGSG_05321, score=3, range=5000-5018, strand=-1

target  5’ GGAGAGUUGUUCUUGGCAA 3’
           : :. .:::::::::::::
query   3’ CGUUCUAACAAGAACCGUU 5’

query=JS16_t1346289, target=FGSG_00950, score=3.5, range=410-428, strand=-1

query=JS16_t0367441, target=FGSG_01799, score=4, range=3539-3557, strand=-1

target  5’ UUUUUGAACUCGUCGAGCG 3’
           :. : ::::::.::::::.
query   3’ AGCACCUUGAGUAGCUCGU 5’

query=JS16_t0367441, target=FGSG_09579, score=4, range=908-925, strand=-1

target  5’ UCGU-GAACUCGUCGACCA 3’
           :::: ::::::.:::: ::
query   3’ AGCACCUUGAGUAGCUCGU 5’

query=JS16_t0367441, target=FGSG_12470, score=4, range=29-46, strand=-1

target  5’ UC-UGGAACUCCUCGAGCU 3’
           :: :::::::: ::::::
query   3’ AGCACCUUGAGUAGCUCGU 5’

query=JS16_t0476932, target=FGSG_09521, score=2, range=85-103, strand=-1

target  5’ UCAAGUUGCCAUCUUCAAU 3’
           ::::::::::::: :::::
query   3’ AGUUCAACGGUAG-AGUUA 5’

query=JS16_t0476932, target=FGSG_00855, score=3, range=332-350, strand=1

target  5’ UCAACGUUGCCAUCUCGGU 3’
           :::: ::::::::::::..:
query   3’ AGUU-CAACGGUAGAGUUA 5’

query=JS16_t0476932, target=FGSG_02916, score=3, range=496-514, strand=-1

target  5’ UGAAGCUUGCCAUCUCAAC 3’
           : ::: ::::::::::::
query   3’ AGUUC-AACGGUAGAGUUA 5’

query=JS16_t0476932, target=FGSG_13982, score=3.5, range=319-336, strand=1

target  5’ UUCAGUUGCCAUUUUAAU 3’
           :. :::::::::.:.:::
query   3’ AGUUCAACGGUAGAGUUA 5’

query=JS16_t0476932, target=FGSG_06141, score=3.5, range=3063-3080, strand=1

target  5’ UUGGUUUGCCAUCUCGAU 3’
           :... ::::::::::.::
query   3’ AGUUCAACGGUAGAGUUA 5’

query=JS16_t0476932, target=FGSG_13343, score=3.5, range=442-458, strand=-1

target  5’ UC-GGUUGCCUUCUCAAU 3’
           :: .:::::: :::::::
query   3’ AGUUCAACGGUAGAGUUA 5’

query=JS16_t0476932, target=FGSG_09646, score=3.5, range=1342-1358, strand=-1

target  5’ UCGAGUU-CCGUCUCAAU 3’
           ::.:::: ::.:::::::
query   3’ AGUUCAACGGUAGAGUUA 5’

query=JS16_t0476932, target=FGSG_04598, score=4, range=1842-1859, strand=1

target  5’ UCAAGUUGCCACUUCAGU 3’
           ::::::::::: .:::.:
query   3’ AGUUCAACGGUAGAGUUA 5’

query=JS16_t0476932, target=FGSG_00966, score=4, range=962-980, strand=1

target  5’ UUGAGAUUGCUAUCUUAAU 3’
           :..:: ::::.::::.:::
query   3’ AGUUC-AACGGUAGAGUUA 5’

query=JS16_t0476932, target=FGSG_02614, score=4, range=65-83, strand=1

target  5’ CCAACGUUGCCAUCUUGAU 3’
            ::: ::::::::::..::
query   3’ AGUU-CAACGGUAGAGUUA 5’

query=JS16_t0476932, target=FGSG_04475, score=4, range=173-190, strand=1

target  5’ UCAUGUUGCCAUUUCACU 3’
           ::: ::::::::.::: :
query   3’ AGUUCAACGGUAGAGUUA 5’

query=JS16_t0476932, target=FGSG_09464, score=4, range=572-589, strand=1

target  5’ UCAACUUGACAUUUCAAU 3’
           :::: ::: :::.:::::
query   3’ AGUUCAACGGUAGAGUUA 5’

query=JS16_t0476932, target=FGSG_12170, score=4, range=355-372, strand=1
```

```
target  5' UGAGGGUUGUUCUUGGCAG 3'
             :.:.:::::::::::::.
query   3' CGUUCUAACAAGAACCGUU 5'

query=JS16_t1346289, target=FGSG_08597, score=3.5, range=1046-1064, strand=-1

target  5' GCCAGGUAGUUCUUGGCAA 3'
           :: ::.: :::::::::::
query   3' CGUUCUAACAAGAACCGUU 5'

query=JS16_t1346289, target=FGSG_13058, score=3.5, range=743-761, strand=-1

target  5' AGAGGUUUGUUCUUGGCAA 3'
             :.: :::::::::::::
query   3' CGUUCUAACAAGAACCGUU 5'

query=JS16_t1346289, target=FGSG_07350, score=4, range=395-413, strand=1

target  5' GAAAGAUUGUUCUUGGAAU 3'
           : ::::::::::::: :
query   3' CGUUCUAACAAGAACCGUU 5'

query=JS16_t1346289, target=FGSG_01188, score=4, range=1093-1112, strand=1

target  5' GCAGAGAUUGUUCUUGCCAU 3'
           ::: :::::::::::: ::
query   3' CGU-UCUAACAAGAACCGUU 5'

query=JS16_t1346289, target=FGSG_03542, score=4, range=320-337, strand=1

target  5' GCGUGGUUGUU-UUGGCAA 3'
           ::. :.::::: :::::::
query   3' CGUUCUAACAAGAACCGUU 5'

query=JS16_t1346289, target=FGSG_00555, score=4, range=836-855, strand=1

target  5' GUAGCAUUGUUCUUGAGCAA 3'
           :.:. :::::::::: ::::
query   3' CGUUCUAACAAGAAC-CGUU 5'

query=JS16_t1346289, target=FGSG_07350, score=4, range=506-524, strand=1

target  5' CCAAUAUUGUUCUUGGCUA 3'
            ::: :::::::::::: :
query   3' CGUUCUAACAAGAACCGUU 5'

query=JS16_t1346289, target=FGSG_03424, score=4, range=737-755, strand=-1

target  5' GUAAGGUUCUUCUUGGCGA 3'
           :.:::.:: ::::::::.:
query   3' CGUUCUAACAAGAACCGUU 5'

query=JS16_t1346289, target=FGSG_01910, score=4, range=2304-2323, strand=-1

target  5' GCGAGGCUUGUUCUUGGUAC 3'
           ::.::. ::::::::::.:
query   3' CGUUCU-AACAAGAACCGUU 5'

query=JS16_t1346289, target=FGSG_08627, score=4, range=524-542, strand=-1

target  5' GCAAGACUGUUCUUUGCAA 3'
           :::::: ::::::: ::::
query   3' CGUUCUAACAAGAACCGUU 5'

query=JS16_t1346289, target=FGSG_04676, score=4, range=107-125, strand=-1

target  5' GAGAGGUUGUCCUUGGCAA 3'
           : .::.:::: ::::::::
query   3' CGUUCUAACAAGAACCGUU 5'

query=JS16_t1346289, target=FGSG_10304, score=4, range=305-324, strand=-1

target  5' GAGGGUGUUGUUCUUGGCAG 3'
           : ..: .::::::::::::.
query   3' CGUUC-UAACAAGAACCGUU 5'

query=JS16_t1346289, target=FGSG_06469, score=4, range=251-269, strand=-1

target  5' GCAAAAGUGUUCUUGGCAU 3'
           :::: : ::::::::::
query   3' CGUUCUAACAAGAACCGUU 5'

query=JS16_t1346289, target=FGSG_06501, score=4, range=482-500, strand=-1

target  5' CCAAGCUUGUUCUUGACAA 3'
            :::: ::::::::: :::
query   3' CGUUCUAACAAGAACCGUU 5'

query=JS16_t1346289, target=FGSG_10933, score=4, range=19-37, strand=-1

target  5' CCAAGUUUGUUCUUGUCAA 3'

target  5' UCCAGUUGGCGUCUCAAU 3'
           :: ::::: :.:::::::
query   3' AGUUCAACGGUAGAGUUA 5'

query=JS16_t0476932, target=FGSG_01442, score=4, range=729-747, strand=1

target  5' UCCAGUUGCCAUGUUCAAU 3'
           :: ::::::::: .:::::
query   3' AGUUCAACGGUA-GAGUUA 5'

query=JS16_t0476932, target=FGSG_11566, score=4, range=370-386, strand=1

target  5' GUGA-UUGCCAUCUCAAC 3'
            ..: ::::::::::::
query   3' AGUUCAACGGUAGAGUUA 5'

query=JS16_t0476932, target=FGSG_02615, score=4, range=1181-1197, strand=1

target  5' UCAUGUUGCCAUC-CAAA 3'
           ::: ::::::::: :::
query   3' AGUUCAACGGUAGAGUUA 5'

query=JS16_t0476932, target=FGSG_09367, score=4, range=190-206, strand=1

target  5' UCCA-UUGCCAUCUCGAC 3'
           :: : ::::::::::.:
query   3' AGUUCAACGGUAGAGUUA 5'

query=JS16_t0476932, target=FGSG_07192, score=4, range=2546-2563, strand=-1

target  5' UCAAGUUGUCGUCUUGAU 3'
           ::::::::.:.:::..::
query   3' AGUUCAACGGUAGAGUUA 5'

query=JS16_t0476932, target=FGSG_01342, score=4, range=787-805, strand=-1

target  5' GCAAGUUGCCGUCCUCAAU 3'
            :::::::::.: ::::::
query   3' AGUUCAACGGUA-GAGUUA 5'

query=JS16_t0476932, target=FGSG_06788, score=4, range=826-844, strand=-1

target  5' UCAAGUUUCCAUCGUCAAU 3'
           ::::::: ::::: :::::
query   3' AGUUCAACGGUAG-AGUUA 5'

query=JS16_t0476932, target=FGSG_06256, score=4, range=662-679, strand=-1

target  5' UGUAGUUGCCAUCUUGAU 3'
           :  :::::::::::..::
query   3' AGUUCAACGGUAGAGUUA 5'

query=JS16_t0476932, target=FGSG_07575, score=4, range=313-330, strand=-1

target  5' UCGGAUUGCCUUCUCAAU 3'
           ::.. ::::: :::::::
query   3' AGUUCAACGGUAGAGUUA 5'

query=JS16_t0476932, target=FGSG_12345, score=4, range=204-220, strand=-1

target  5' UCAA-UUGCUGUCUCAAA 3'
           :::: ::::..::::::
query   3' AGUUCAACGGUAGAGUUA 5'

query=JS16_t0476932, target=FGSG_10493, score=4, range=886-902, strand=-1

target  5' UUGAGUUGCCAU-UCAAA 3'
           :..::::::::: ::::
query   3' AGUUCAACGGUAGAGUUA 5'

query=JS16_t0476932, target=FGSG_13956, score=4, range=856-873, strand=-1

target  5' ACUAGCUGCCAUCUCAAU 3'
            : :: ::::::::::::
query   3' AGUUCAACGGUAGAGUUA 5'

query=JS16_t0476932, target=FGSG_13369, score=4, range=378-395, strand=-1

target  5' UCACGAUGCCAUCUCAAC 3'
           ::: : ::::::::::
query   3' AGUUCAACGGUAGAGUUA 5'

query=JS16_t0476932, target=FGSG_04139, score=4, range=997-1013, strand=-1

target  5' UCAA-UUGCAGUCUCAAU 3'
           :::: :::: .:::::::
query   3' AGUUCAACGGUAGAGUUA 5'

query=JS16_t0476932, target=FGSG_00404, score=4, range=1497-1514, strand=-1

target  5' UUGUCUUGCCAUCUCAAA 3'
```

```
            :::: ::::::::: :::
query   3’ CGUUCUAACAAGAACCGUU 5’

query=JS16_t1346289, target=FGSG_11721, score=4, range=227-244, strand=-1

target  5’ ACGAG-UUGUUCUUGGCGG 3’
            :.:: ::::::::::::..
query   3’ CGUUCUAACAAGAACCGUU 5’

query=JS16_t1346289, target=FGSG_08383, score=4, range=39-56, strand=-1

target  5’ UCGA-AUUGUUCUUGGUAG 3’
            :.: :::::::::::.:.
query   3’ CGUUCUAACAAGAACCGUU 5’

query=JS16_t1346289, target=FGSG_08703, score=4, range=1672-1689, strand=-1

target  5’ GU-GCAUUGUUCUUGGCGA 3’
           :. . ::::::::::::.:
query   3’ CGUUCUAACAAGAACCGUU 5’

query=JS16_t1346289, target=FGSG_02583, score=4, range=1454-1472, strand=-1

target  5’ GCAAACUGGUUCUUGGCAA 3’
           ::::  : :::::::::::
query   3’ CGUUCUAACAAGAACCGUU 5’

query=JS16_t1346289, target=FGSG_11177, score=4, range=981-998, strand=-1

target  5’ GCCA-AUUGAUCUUGGCAA 3’
           :: : :::: :::::::::
query   3’ CGUUCUAACAAGAACCGUU 5’

query=JS16_t1346289, target=FGSG_05402, score=4, range=1478-1497, strand=-1

target  5’ GCAGGCUUGCUUCUUGGCAG 3’
           :::.: ::: :::::::::.
query   3’ CGUUCUAAC-AAGAACCGUU 5’

query=JS16_t1346289, target=FGSG_02589, score=4, range=410-429, strand=-1

target  5’ GCUGGGUUGCUUCUUGGCAA 3’
           :: .:.::: ::::::::::
query   3’ CGUUCUAAC-AAGAACCGUU 5’

query=JS16_t0176425, target=FGSG_05406, score=3.5, range=1045-1066, strand=-1

target  5’ GGAAGCGAGCACGAACGACAGA 3’
           :: :: .: :::::::::::::
query   3’ CCAUC-UUGGUGCUUGCUGUCU 5’

No results for JS16_t1346299
No results for JS16_t1346375
No results for JS16_t3139291
No results for JS16_t2243167
No results for JS16_t1346423
No results for JS16_t1346460
No results for JS16_t4035920
No results for JS16_t2243238
query=JS16_t3139415, target=FGSG_01099, score=3.5, range=182-201, strand=1

target  5’ UC-UGGGAUACCGCCGGUCAG 3’
           :: :: ::::::.:::::::.
query   3’ AGCACACUAUGGUGGCCAGUU 5’

query=JS16_t1346515, target=FGSG_00071, score=3, range=1392-1410, strand=-1

target  5’ AGGAAU-CUUCCACUCGUAG 3’
           :.:: . :::::::::::::
query   3’ UUCUCGCGAAGGUGAGCAUC 5’

query=JS16_t1346515, target=FGSG_03046, score=3.5, range=204-222, strand=-1

target  5’ AGGAG-GCUUCCACUUGUAC 3’
           :.::: ::::::::::.:::
query   3’ UUCUCGCGAAGGUGAGCAUC 5’

query=JS16_t1346515, target=FGSG_12755, score=4, range=26-44, strand=1

target  5’ GGGAAC-CUUCCACUCGUGG 3’
           ..:: : :::::::::::.:
query   3’ UUCUCGCGAAGGUGAGCAUC 5’

query=JS16_t1346515, target=FGSG_01316, score=4, range=731-750, strand=-1

target  5’ AGGGGCACUUCCACUGGUAG 3’
           :.:.:: :::::::: ::::
query   3’ UUCUCGCGAAGGUGAGCAUC 5’

query=JS16_t1346515, target=FGSG_03046, score=4, range=306-324, strand=-1

target  5’ GGGAG-GCUUCCACUUGUAC 3’
```

```
           :..  ::::::::::::
query   3’ AGUUCAACGGUAGAGUUA 5’

query=JS16_t0476932, target=FGSG_10933, score=4, range=1297-1313, strand=-1

target  5’ UUGUG-UGCCAUCUCAAU 3’
           :.. : ::::::::::::
query   3’ AGUUCAACGGUAGAGUUA 5’

query=JS16_t0476932, target=FGSG_03065, score=4, range=313-329, strand=-1

target  5’ UC-AGUUGACAUCUCAAA 3’
           :: ::::: ::::::::
query   3’ AGUUCAACGGUAGAGUUA 5’

query=JS16_t0476932, target=FGSG_00600, score=4, range=1132-1148, strand=-1

target  5’ UCAUGUU-CCAUCUCGAU 3’
           ::: ::: :::::::.::
query   3’ AGUUCAACGGUAGAGUUA 5’

query=JS16_t0476932, target=FGSG_05366, score=4, range=844-862, strand=-1

target  5’ UCAAGUUGUCCAUCUCGAA 3’
           :::::::: :::::::.:
query   3’ AGUUCAAC-GGUAGAGUUA 5’

query=JS16_t0476932, target=FGSG_09451, score=4, range=330-346, strand=-1

target  5’ UCAUGUUGCCA-CUCAGU 3’
           ::: ::::::: ::::.:
query   3’ AGUUCAACGGUAGAGUUA 5’

No results for JS16_t4099117
query=JS16_t0308144, target=FGSG_03237, score=3, range=290-308, strand=1

target  5’ UUCCAUUCAUCCUCAAGUU 3’
           ::: :::::::::: ::::
query   3’ AAGUUAAGUAGGAGCUCAA 5’

query=JS16_t0308144, target=FGSG_05306, score=3, range=2471-2489, strand=-1

target  5’ UUCAACUCAUCCUCGUGUU 3’
           ::::: ::::::::: :::
query   3’ AAGUUAAGUAGGAGCUCAA 5’

query=JS16_t0308144, target=FGSG_01922, score=3, range=752-770, strand=-1

target  5’ CUCAUCUCAUCCUCGAGUU 3’
            :::  :::::::::::::
query   3’ AAGUUAAGUAGGAGCUCAA 5’

query=JS16_t0308144, target=FGSG_00383, score=3, range=529-546, strand=-1

target  5’ UUCCA-UCAUCUUCGAGUU 3’
           ::: : :::::.:::::::
query   3’ AAGUUAAGUAGGAGCUCAA 5’

query=JS16_t0308144, target=FGSG_11811, score=3.5, range=889-907, strand=1

target  5’ GACAGCUCAUCCUCGAGUU 3’
             ::. :::::::::::::
query   3’ AAGUUAAGUAGGAGCUCAA 5’

query=JS16_t0308144, target=FGSG_07615, score=3.5, range=987-1004, strand=1

target  5’ UUCAGUUUAU-CUCGAGUU 3’
           ::::.::.:: ::::::::
query   3’ AAGUUAAGUAGGAGCUCAA 5’

query=JS16_t0308144, target=FGSG_00633, score=3.5, range=9863-9881, strand=-1

target  5’ UUCAGUUCAUCCUCGGCUU 3’
           ::::.::::::::::. ::
query   3’ AAGUUAAGUAGGAGCUCAA 5’

query=JS16_t0308144, target=FGSG_03328, score=4, range=182-200, strand=1

target  5’ UCCAAUUCAUCCUCGAGGA 3’
           : :::::::::::::::
query   3’ AAGUUAAGUAGGAGCUCAA 5’

query=JS16_t0308144, target=FGSG_12498, score=4, range=350-368, strand=-1

target  5’ UUAAAUUCAUCCUUGCGUU 3’
           :: ::::::::::.: :::
query   3’ AAGUUAAGUAGGAGCUCAA 5’

query=JS16_t0308144, target=FGSG_08887, score=4, range=1805-1823, strand=-1

target  5’ UUCUUUUCGUCUUCGAGUU 3’
           :::  :::.::.:::::::
```

```
           ..::: :::::::::.:::
query   3' UUCUCGCGAAGGUGAGCAUC 5'

No results for JS16_t3139438
query=JS16_t4035976, target=FGSG_05568, score=4, range=1193-1217, strand=-1

target  5' AGGAAAGGUGUCCCAUGAAGUGAUG 3'
           :::::  :.: :::::::::::::.
query   3' UCCUUGGCGC-GGGUACUUCACUAU 5'

No results for JS16_t0469247
query=JS16_t4035999, target=FGSG_06527, score=3, range=357-373, strand=1

target  5' CGU-CUUUGAGUCGCGAC 3'
            :: :::::::::::::
query   3' CCAUGAAACUCAGCGCUC 5'

query=JS16_t4035999, target=FGSG_11485, score=3, range=471-488, strand=-1

target  5' GGGCCUUUGAGUCGCGAU 3'
           ::  ::::::::::::
query   3' CCAUGAAACUCAGCGCUC 5'

query=JS16_t4035999, target=FGSG_09327, score=3, range=1050-1066, strand=-1

target  5' GGUACUUUGA-UUGCGAG 3'
           :::::::::: :.:::::
query   3' CCAUGAAACUCAGCGCUC 5'

query=JS16_t4035999, target=FGSG_02250, score=4, range=1152-1169, strand=1

target  5' GGUGUUUUGGGUGGCGAG 3'
           :::..::::.:: :::::
query   3' CCAUGAAACUCAGCGCUC 5'

query=JS16_t4035999, target=FGSG_04127, score=4, range=2527-2544, strand=1

target  5' GGUGUUUUGAGUGGCGAC 3'
           :::..::::::: ::::
query   3' CCAUGAAACUCAGCGCUC 5'

query=JS16_t4035999, target=FGSG_03030, score=4, range=311-329, strand=1

target  5' CGUCCUUUGAGUCGCGUAG 3'
            :: :::::::::::: ::
query   3' CCAUGAAACUCAGCGC-UC 5'

query=JS16_t4035999, target=FGSG_11743, score=4, range=1208-1224, strand=1

target  5' GGAUCUUUGAGUCG-GAG 3'
           ::  :::::::::: :::
query   3' CCAUGAAACUCAGCGCUC 5'

query=JS16_t4035999, target=FGSG_02691, score=4, range=737-753, strand=1

target  5' GGUACUUUGA-UCGCAAG 3'
           :::::::::: :::: ::
query   3' CCAUGAAACUCAGCGCUC 5'

query=JS16_t4035999, target=FGSG_03936, score=4, range=739-755, strand=1

target  5' GGAACUUUG-GUCGCGAA 3'
           :: :::::: :::::::
query   3' CCAUGAAACUCAGCGCUC 5'

query=JS16_t4035999, target=FGSG_00587, score=4, range=257-273, strand=1

target  5' CUUACUUUGAG-CGCGAG 3'
             ::::::::: ::::::
query   3' CCAUGAAACUCAGCGCUC 5'

query=JS16_t4035999, target=FGSG_04481, score=4, range=359-376, strand=-1

target  5' AGUUCUUUGGGUCGCGGG 3'
            :: :::::.::::::.:
query   3' CCAUGAAACUCAGCGCUC 5'

query=JS16_t4035999, target=FGSG_07668, score=4, range=1387-1405, strand=-1

target  5' CGUACUUUUGGGUUGCGAG 3'
            :::: ::::.::.:::::
query   3' CCAUG-AAACUCAGCGCUC 5'

query=JS16_t4035999, target=FGSG_09281, score=4, range=255-271, strand=-1

target  5' UGGACUUUGAGUCG-GAG 3'
            : ::::::::::: :::
query   3' CCAUGAAACUCAGCGCUC 5'

query=JS16_t3139490, target=FGSG_08297, score=4, range=173-193, strand=1

target  5' ACUACG-ACUCGGGAUGCUGCG 3'
query   3' AAGUUAAGUAGGAGCUCAA 5'

query=JS16_t0308144, target=FGSG_00307, score=4, range=1334-1353, strand=-1

target  5' UUCUGGCUCAUCUUCGAGUU 3'
           ::: .. :::::.:::::::
query   3' AAG-UUAAGUAGGAGCUCAA 5'

query=JS16_t0308144, target=FGSG_06710, score=4, range=413-432, strand=-1

target  5' UUCACUCUCAUUCUCGAGUG 3'
           :::: : ::::.:::::::
query   3' AAGUUA-AGUAGGAGCUCAA 5'

query=JS16_t0308144, target=FGSG_02923, score=4, range=86-105, strand=-1

target  5' GUCAAGCUUAUCCUCGAGUU 3'
            ::::  :.:::::::::::
query   3' AAGUU-AAGUAGGAGCUCAA 5'

query=JS16_t0308144, target=FGSG_11189, score=4, range=116-134, strand=-1

target  5' UGCAACUCAUCCUCGAUUU 3'
           : ::: :::::::::: ::
query   3' AAGUUAAGUAGGAGCUCAA 5'
```
[truncated: 598,876 more chars]
